# Activation of The Phosphatidylcholine to Lysophosphatidylcholine Pathway Is Associated with Osteoarthritis Knee Cartilage Volume Loss Over Time

**DOI:** 10.1038/s41598-019-46185-w

**Published:** 2019-07-04

**Authors:** Guangju Zhai, Jean-Pierre Pelletier, Ming Liu, Dawn Aitken, Edward Randell, Proton Rahman, Graeme Jones, Johanne Martel-Pelletier

**Affiliations:** 1 Discipline of Genetics, Faculty of Medicine, Memorial University of Newfoundland, St. John’s, NL Canada; 2 Osteoarthritis Research Unit, University of Montreal Hospital Research Centre (CRCHUM), Montreal, QC Canada; 3 Menzies Research Institute, University of Tasmania, Hobart, Australia; 4 Department of Laboratory Medicine, Faculty of Medicine, Memorial University of Newfoundland, St. John’s, NL Canada; 5 Discipline of Medicine, Faculty of Medicine, Memorial University of Newfoundland, St. John’s, NL Canada

**Keywords:** Predictive markers, Metabolomics

## Abstract

To identify serum biomarker(s) for predicting knee cartilage volume loss over time, we studied 139 knee osteoarthritis (OA) patients from a previous 24-month clinical trial cohort. Targeted metabolomic profiling was performed on serum collected at baseline. The pairwise metabolite ratios as proxies for enzymatic reaction were calculated and used in the analysis. Cartilage volume loss between baseline and 24 months was assessed quantitatively by magnetic resonance imaging (MRI). Data revealed an association between the serum ratio of lysophosphatidylcholine 18:2 (lysoPC 18:2) to phosphatidylcholine 44:3 (PC44:3) and the cartilage volume loss in the lateral compartment (β = −0.21 ± 0.04, p = 8.53*10^−7^) and with joint degradation markers, COMP (r = 0.32, p = 0.0002) and MMP1 (r = 0.26, p = 0.002). The significance remained after adjustment for age, sex, BMI, diabetes, hypertension, dyslipidemia, and the treatment taken in the original study. As the ratio indicated the over activation of the conversion pathway of PC to lysoPC catalyzed by phospholipase A_2_ (PLA_2_), we assessed and found that a specific PLA_2_, PLA_2_G5, was significantly increased in human OA cartilage and synovial membrane (85% and 19% respectively, both p < 0.04) compared to controls, and its overexpression correlated with IL-6 (r = 0.63, p = 0.0008). Our data suggest that the serum lysoPC 18:2 to PC44:3 ratio is highly associated with a greater risk of cartilage volume loss of the knee and warrants further investigation in an independent cohort.

## Introduction

Osteoarthritis (OA) is the most prevalent joint disease in the world. It affects about 10% of the population globally and about half of the population aged 60 years or older^1^. The disease results in joint pain and disability^2^, and imposes a significant socioeconomic burden on society with an aggregate cost of ~2.5% of gross domestic product^3^. The disease can be difficult to define as it has an insidious onset, and clinically represents a heterogeneous group of diseases. Hence, OA evolution can be slow and last many years, but could progress rapidly^4–6^, leading to the need for total joint replacement therapy. At present, OA diagnosis occurs mainly at a moderate-to-severe stage of the disease and often only where the destruction of the joint tissue has reached an advanced stage. Clinical parameters do not provide sufficient information to make a good prediction or prognosis. To achieve prognosis, one should be able to quickly have accurate results. One means is the use of serum biomarkers which will help clinical practice by selecting patients who will best benefit from specific therapeutic interventions. However, to date, no biomarker has been reported to be useful as a prognostic test in individual patients.

Recent development in metabolomics and its application to the study of OA are promising^7^. Several small studies have identified certain metabolic pathways that play a role in OA^8,9^. Among them, the arginine and proline metabolic pathway appears to be one of the main pathways identified to be associated with knee OA^10,11^. Consistent with these reports, we and others have found that a lower plasma free arginine level was associated with end-stage knee OA in studies with a relatively large sample size^12,13^. Further, our group found that total lysophosphatidylcholines (lysoPCs) to phosphatidylcholines (PCs) ratio was associated with knee OA risk; people with higher lysoPCs to PCs ratio at baseline had a 2.3 times greater risk of undergoing total joint replacement surgery in 10 year follow-up^14^. Such ratio was also found to be associated with early OA^15^, but it is unclear whether these metabolic markers are associated with OA progression. We therefore undertook to identify novel metabolic markers using a previous clinical trial cohort^16^ and a targeted metabolomics approach on knee OA disease progression. The disease progression was measured by the percentage of cartilage volume loss as assessed by magnetic resonance imaging (MRI), which has been demonstrated as being one of the best markers of OA disease progression and outcome as well as the need for total knee replacement (the hard outcome in knee OA) as shown in several studies (refer to the review^17^).

## Results

Table 1 presents the descriptive characteristics of the study population. As originally reported^16^, there was no difference in age, sex, BMI, and baseline lateral and medial cartilage volumes between the two treatment arms. However, the cartilage loss at 24 months was significantly reduced with licofelone treatment compared to naproxen treatment, at the lateral compartment (p = 0.02). The same trend was seen for the medial compartment though it did not reach statistical significance.

**Table 1:** Descriptive characteristics of the study population.

Variables	Licofelone group (n = 69)	Naproxen group (n = 70)
Age (yrs)	61 ± 9	61 ± 8
Sex (% female)	74%	61%
BMI (kg/m^2^)	32.3 ± 5.8	31.2 ± 5.7
Lateral cartilage volume at baseline (mm^3^)	6095.5 ± 1594.7	6364.1 ± 1822.0
Lateral cartilage volume changes at 24 months (%)	−4.70 ± 4.16%	−6.40 ± 4.14%
Medial cartilage volume at baseline (mm^3^)	5523.0 ± 1503.3	5690.0 ± 1640.0
Medial cartilage volume changes at 24 months (%)	−7.58 ± 5.15%	−8.25 ± 6.01%

Results are shown as mean ± SD except for sex which was shown as percentage for females.

### Association of lysoPC 18:2 to PC44:3 ratio with cartilage volume loss

One hundred and fifty-two (152) metabolites standardized by Z-score were used for pair-wise ratios. Unadjusted analyses showed that, based on the adjusted pre-defined significance level (p = 2.3*10^−6^), the lysophosphatidylcholine with 18 carbons and 2 double bonds (lysoPC 18:2) to phosphatidylcholine with 44 carbons and 3 double bonds (PC 44:3) was significantly associated with the percentage of the lateral cartilage loss (p = 8.5*10^−7^) (Table 2). The lateral cartilage volume was reduced by 0.21% (95% CI −0.30%; −0.13%) per unit increase for the ratio. The significance remained (p = 4.5*10^−6^) after adjusting for covariates including age, sex, BMI, diabetes, hypertension, dyslipidemia, and treatments taken in the original study (Table 2). Although not reaching the pre-defined significance level, the second and third most potentially associated metabolite ratios (Table 2) lysoPC 18:1 to PC 44:3 and glutamine to PC 44:3 were also found to be highly correlated with lysoPC 18:2 to PC 44:3 (r = 0.79, p = 2.2*10^−16^; r = 0.49, p = 8.9*10^−11^).

**Table 2:** The top four metabolite ratios that associated with the percentage of the lateral cartilage volume loss at 24 months.

Metabolite ratios	Unadjusted*		Adjusted**	
	β (95% CI)	P value	β (95% CI)	P value
LysoPC 18:2/PC44:3	−0.21 (−0.30; −0.13)	8.5*10^−7^	−0.18 (−0.27; −0.10)	4.5*10^−6^
LysoPC18:1/PC44:3	−0.28 (−0.40; −0.16)	1.3*10^−5^	−0.25 (−0.37; −0.13)	1.5*10^−5^
Glutamine/PC44:3	−0.28 (−0.40; −0.16)	1.9*10^−5^	−0.28 (−0.40; −0.16)	3.6*10^−6^
Methionine/proline	0.09 (0.05; 0.12)	3.0*10^−5^	0.07 (0.04; 0.11)	6.9*10^−5^

^*^Associations were tested by using linear regression model. **Covariates including age, sex, BMI, diabetes, hypertension, dyslipidemia, and treatment were included in the multivariable linear regression model. β stands for percentage change in cartilage volume at 24 months per unit increase for a given ratio. For example, lateral cartilage volume was reduced by 0.21% at 24 months for one unit increase of the ratio of the Z-scores of lysoPC 18:2 and PC 44:3.

LysoPC 18:2: lysophosphatidylcholine with 18 carbons and 2 double bonds; PC44:3: phosphatidylcholine with 44 carbons and 3 double bonds; lysoPC 18:1: lysophosphatidylcholine with 18 carbons and 1 double bond.

For the medial compartment, four metabolite ratios related to phosphatidylcholine metabolism demonstrated high significance associated with this compartment’s cartilage volume loss, although their significance did not reach the adjusted pre-defined level (Table 3).

**Table 3:** The top metabolite ratios that associated with the percentage of the medial cartilage volume loss at 24 months.

Metabolite ratios	Unadjusted*		Adjusted**	
	β (95% CI)	P value	β (95% CI)	P value
PC32:1/PC40:5	0.30 (0.16; 0.44)	2.5*10^−5^	0.30 (0.16; 0.43)	5.7*10^−6^
PC32:2/PC40:5	0.21(0.11; 0.30)	5.1*10^−5^	0.20 (0.11; 0.30)	1.1*10^−5^
PC42:0/PC40:5	0.26 (0.14; 0.38)	5.4*10^−5^	0.26 (0.14; 0.38)	7.5*10^−6^
PC34:1/PC40:5	0.18 (0.09; 0.27)	5.7*10^−5^	0.19 (0.10; 0.27)	4.5*10^−6^

^*^Associations were tested by using linear regression model. **Covariates including age, sex, BMI, diabetes, hypertension, dyslipidemia, and treatment were included in the multivariable linear regression model. β stands for percentage change in cartilage volume at 24 months per unit increase for a given ratio.

PC: phosphatidylcholine; first number represents the total number of carbons in the PC and the second number stands for the number of the double bonds in the compound.

Arginine, which was previously reported to be associated with knee OA risk^12^, was not associated with the percentage of the cartilage volume loss in the current analysis (data not shown). For the complete ratio data of lateral and medial comparing all the metabolite ratios to the percentage of the cartilage volume loss, please refer to the Supplementary Tables 1 and 2.

We next evaluated the correlation between the lysoPC 18:2 to PC 44:3 ratio and two serum biomarkers of cartilage degradation, COMP and MMP-1, that were previously assessed in this cohort^18^. Data showed a weak but significant correlation between the lysoPC 18:2 to PC 44:3 ratio and serum COMP levels at both baseline and follow-up (both r = 0.32, both p = 0.0002). The ratio was also significantly correlated with serum MMP-1 levels at follow-up (r = 0.26, p = 0.002). The significances remained virtually the same after adjustment for age, sex, BMI, diabetes, hypertension, dyslipidemia, and treatments taken in the original study.

### Metabolic pathway involved in the over-expression of lysoPC 18:2 to PC44:3

**Figure 1:**
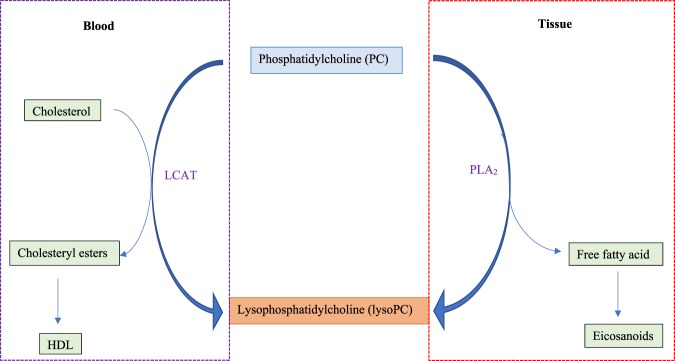
Biochemical conversion pathways of phosphatidylcholine (PC) to lysophosphatidylcholine (lysoPC). *HDL: high density lipoprotein; LCAT: lecithin-cholesterol acyltransferase; PLA_2_: Phospholipase A_2_.

The association of the higher lysoPC 18:2 to PC44:3 ratio with the percentage of the cartilage volume loss implies that the conversion pathway of PC to lysoPC is overactivated in these patients with high cartilage loss. The biochemical conversion from PC to lysoPC is mainly catalyzed by lecithin-cholesterol acyltransferase (LCAT) in blood or phospholipase A_2_ (PLA_2_) in tissues (Fig. 1). In blood, after binding to a lipoprotein, LCAT cleaves the fatty acid in sn-2 position of PC which then is trans-esterified to the 3-β-hydroxyl group on the A-ring of cholesterol to form cholesteryl ester.

We first examined whether the association between lysoPC 18:2 to PC44:3 ratio and cartilage volume loss over time was related to an increased LCAT activity. To this end, the serum LCAT levels were determined in the top and bottom 10% of subjects based on the serum lysoPC 18:2 to PC44:3 ratio levels (n = 12 for the bottom 10% and n = 15 for the top 10%). Although the median value from the patients having the highest ratio was lower than those from the lowest one, this did not reach statistical significance (Supplementary Fig. 1), suggesting the observed association was not due to differences in LCAT activity.

**Figure 2:**
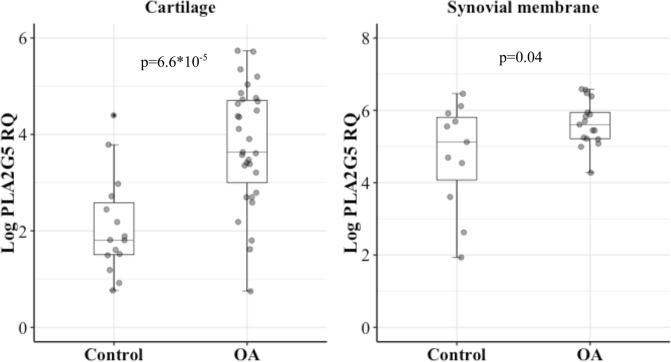
PLA_2_G5 expression levels in human cartilage and synovial membrane. PLA_2_G5: Phospholipase A_2_ group 5. Log PLA_2_G5 RQ: Natural logarithm transformed relative quantification of *PLA*_2_*G5* expression. p values were obtained by Student’s t test.

Next, we evaluated the PLA_2_ pathway in joint tissues. PLA_2_ includes several unrelated protein families with common enzymatic activity. Previous work examining multiple PLA_2_ isoforms in chondrocytes suggested that four forms of the PLA_2_, e.g. PLA_2_G2a, G2d, G4a, and G5, were important in chondrocytes^19^. We therefore examined these four PLA_2_ isoforms. We failed to design a primer to capture PLA_2_G2d but were successful for the three other different isoforms of PLA_2_. Determination was done in three joint tissues, namely cartilage, synovial membrane, and subchondral bone. Data showed that in OA the gene expression of PLA_2_G5, but not the other two isoforms, had 85% increased expression compared to controls (p = 6.6*10^−5^; Fig. 2) in cartilage. Of note, there was no difference between knee (n = 9) and hip (n = 23) OA (p = 0.51). PLA_2_G5 expression was also significantly over-expressed by 19% in OA synovial membrane compared to the controls (p = 0.04; Fig. 2). A similar trend was also seen in OA subchondral bone, but the difference did not reach statistical significance (Supplementary Fig. 2).

**Figure 3:**
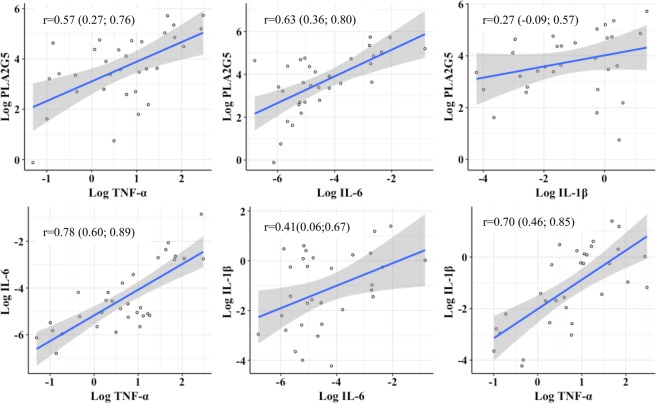
Correlation of three pre-inflammatory cytokines and PLA_2_G5 in osteoarthritic cartilage tissue. X and Y-axes are natural logarithm transformed relative quantification of gene expression. r is Pearson’s correlation coefficient. p values were obtained by the exact test. *PLA*_*2*_*G5*: Phospholipase A_2_ group 5; *TNF-α:* Tumor necrosis factor alpha; *IL-6:* Interleukin 6; *IL-1β:* Interleukin 1 beta.

It has been previously reported^19^ that multiple PLA_2_ isoform expression in chondrocytes can be stimulated by pro-inflammatory cytokines including IL-1β, IL-6, and TNF-α. Data revealed that IL-6 and TNF-α were strongly correlated with PLA_2_G5 expression (r = 0.63, and r = 0.57, respectively, p ≤ 0.0008; Fig. 3). Although a positive correlation was also found for IL-1β (r = 0.27), this did not reach statistical significance. We further looked at the correlation between these cytokines in OA cartilage. As expected, they were highly correlated with each other (all p ≤ 0.02), the higher correlations of which were found between IL-6 and TNF-α (r = 0.78, p = 1.2*10^−7^) (Fig. 3). Multivariable regression modeling with the three cytokines showed that the IL-6 was the only one remaining to be associated with PLA_2_G5 (β = 0.55; p = 0.018).

## Discussion

To the best of our knowledge, this is the first study using a comprehensive metabolic approach to document the association between knee OA progression and the activation of the conversion pathway of PC to lysoPC. In this study we used metabolite ratios rather than individual metabolite concentrations in the analysis because they are not derived from two independent variables but related to each other representing metabolic enzymatic reaction in the body. Previous studies have also reported that metabolite ratios serve as good biomarkers with predictive ability beyond that of the single constituents because of the reduced noise and increased statistical power^20^. It has been shown that metabolite ratios have stronger and more meaningful association with phenotypes^21,22^. Further, we used the percentage of cartilage volume loss assessed by MRI as the OA disease progression measure, which is an objective, quantitative and continuous measure as opposed to pain or symptoms, which used scoring scales and are patient-dependent. Moreover, the pain outcome did not reflect the pathological process and is generally poorly related to joint tissue structural alterations (refer to the review^17^).

Emerging data showed that the serum level ratio of lysoPC 18:2 to PC44:3 has a great potential as a new biomarker in monitoring knee OA disease progression, in that it was highly associated with cartilage volume loss over time in the lateral compartment in symptomatic knee OA patients. Of note, while we can describe the PC 44:3 as a group of glycerophosphocholine compounds containing 1 ester linked fatty acid and 1 ether linked fatty alcohol in their structure, the combined chain length of the fatty alcohol and the fatty acid would be 44 carbon atoms, the total number of double bonds including both chains would be 3, owing to the limitation of the method we used, the details of the actual structure of this compound remains unknown and an item for further characterization using higher resolution methods in future studies.

Interestingly, the lysoPC 18:2 to PC44:3 ratio was also suggested for monitoring anti-inflammatory treatment in another arthritic disease, rheumatoid arthritis^23^. This may suggest that the ratio is a marker to detect active cartilage damage regardless what disease causes the damage. However, the sample size was too small in that study to draw a firm conclusion. In line with such ratio, previous data from the Dutch CHeCK cohort using a relatively small sample size reported that a lysoPC to PC ratio in plasma, the lysoPC with 16 and 18 carbons to PC with 36 and 38 carbons and four double bonds, has a slightly higher level in early and moderate OA as classified by radiography than the control^15^. While this ratio involved lysoPC and PC with different number of carbons and double bonds compared to what we found in the current study, it represents the same conversion pathway of PC to lysoPC. Furthermore, our group demonstrated that total lysoPC to PC ratio in plasma was associated not only with knee OA risk but is predictive of total joint replacement surgery over 10-years^14^. The current study not only extended these findings on OA progression, but more specifically identified the conversion of PC with 44 carbons and 3 double bonds to lysoPC with 18 carbons and 2 double bonds.

The reason for the association with lateral compartment cartilage volume loss is not clear, but this site was also favoured in the association of other serum biomarkers with cartilage volume loss^24,25^ and assessment of disease-modifying OA drugs using MRI^16,18,26,27^, and likely reflects an association with less advanced OA cartilage lesions. Indeed, it is well known that, in symptomatic OA patients, the cartilage alterations are generally less severe in the lateral than in the medial compartment. Moreover, and related to our data, the COMP level was previously found predominately associated with the loss of cartilage in the lateral compartment^18^. Interestingly, here we showed a strong correlation between the lysoPC 18:2 to PC44:3 ratio and the COMP levels at both baseline and follow-up.

With the use of a very conservative method to control for multiple comparison, we did not find any associations between the metabolite ratios and medial compartment cartilage volume loss. However, in this compartment, the top associated metabolite ratios also suggest the involvement of PC metabolic pathway. Since these ratios are all related to PCs with different number of carbons but not with lysoPCs, we speculate that this is related to PC synthesis rather than its catabolic process. Nevertheless, the findings on the medial compartment warrant further investigation in a larger sample size.

It is well known that the conversion of PC to lysoPC is mainly catalyzed by LCAT in blood and PLA_2_ in tissues^28,29^. Another emerging finding of the current study is that an over-activation of LCAT activity in blood is not the factor responsible, but that a PLA_2_ isoform, the PLA_2_G5, in OA tissues appeared to be involved. Hence, both OA cartilage and synovial membrane demonstrated a significant increase in this enzyme (PLA_2_G5), with a higher level in cartilage. This is consistent with a previous report suggesting that although in OA articulations both cartilage and synovial membrane contain large quantities of PLA_2_, the cartilage appeared to be a primary source of this factor^30^. Moreover, the finding that the proinflammatory cytokine IL-6 is involved in this enzyme’s over-expression in OA cartilage also agrees with studies reporting that proinflammatory cytokines, including IL-6, stimulate multiple PLA_2_ isoenzyme expression in OA chondrocytes^19^, and that this cytokine level correlated with knee OA progression and risk of cartilage loss^18,31,32^. These also concur with the findings that the lysoPC 18:2 to PC 44:3 ratio strongly correlated with two well known cartilage degradation biomarkers, COMP and MMP1.

An explanation for the lysoPC 18:2 to PC44:3 ratio being associated with the articular structure degeneration could be as follows. One of the consequences of the augmented conversion of PC to lysoPC is the increased lysoPC levels. LysoPC, also known as lysolecithin, is a potent membrane-dissolving agent that was reported as one of the first truly caspase-derived “find me” signals. It specifically results in the recruitment of phagocytes to the site of apoptosis and facilitates opsonization of apoptotic cells^33^. Chondrocyte apoptosis was significantly associated with cartilage damage and pathogenesis of OA^34^. In addition, lysoPCs, particularly acyl-alkyl phosphatidylcholines, are potential precursors for synthesis of platelet activating factor (PAF), a factor well known to play an important role in the inflammatory response and mediation. Although the role of PAF has been demonstrated in the arthritic disease rheumatoid arthritis and osteoporosis^35,36^, there is no study on OA pathophysiology. It is however possible that PAF, due to its effect on the inflammatory cascade, could play a role in OA. Further studies with PAF on OA are warranted.

Another consequence of the conversion of PC to lysoPC is a release of polyunsaturated fatty acids including arachidonic acid, linoleic acid, and linolenic acid from the sn-2 position of the PC phospholipid glyceryl backbone. These fatty acids are precursors of eicosanoids, prostanoids and endocannabinoids, which are bioactive molecules affecting a variety of physiological processes including inflammatory processes^37^. Data showed that using molecules targeting cyclooxygenase or lipoxygenase alone has no effect on cartilage volume loss^38^, but dual inhibitors did reduce cartilage volume loss^16^, suggesting multiple bioactive molecules produced by the eicosanoid pathway are involved in OA disease progression. In the current study, about half of the patients were treated with licofelone, a dual inhibitor of cyclooxygenase and lipoxygenase. Additional experiments revealed that although a lower level of the lysoPC18:2 to PC44:3 ratio was found in the licofelone group (−0.31 ± 0.57) than in the naproxen group (cyclooxygenase inhibitor; 1.74 ± 9.92), the difference did not reach statistical significance. Moreover, as the lysoPC18:2 to PC44:3 ratio is associated with cartilage volume loss and the licofelone^16^ reduced the cartilage volume loss in the lateral compartment, these both suggest that patients with a lower level of the lysoPC18:2 to PC44:3 ratio who would have less cartilage loss over time could benefit from the licofelone treatment. This important finding, if confirmed in an independent study, could provide new information about this ratio being used as a biomarker to predict drug efficacy on articular structure in clinical trials, at least for evaluating therapy looking at such a dual inhibitor treatment. Furthermore, and in line with the use of this ratio as a biomarker in clinical trials, this specific PC conversion is likely to produce the bioactive molecule oleoylethanolamide (OEA). A recent study reported that the OEA level was increased twice in the synovial fluid of the OA dog knee when compared to the contralateral joints^39^. However, we did not have data on the OEA in the current study, further human studies on OEA in OA are warranted.

This study has some limitations. First, although further studies are required to confirm the finding of the association between the lysoPC18:2 to PC 44:3 ratio and cartilage volume loss over time, we are confident of its validity. In support of this finding is a post-hoc power calculation that showed that we had 100% study power to detect the difference we observed with the sample size and at significance level of 2.3 × 10^−6^ which we used in the study, as well as the data on the expression levels of relevant enzymes involved in this metabolic pathway. Caution should be taken in the interpretation of the gene expression results as these were done on the end-stage OA patients. Secondly, as mentioned above, the actual structure of PC 44:3 remains unknown. As the metabolic profiling was done using Biocrates p180 kits with a triple quad MS, and this assay does not have enough resolution to distinguish compounds with same nominal parent mass and producing same m/z fragment, we could not ascertain the details of its structure. Further studies with a higher resolution method are needed for more information on this compound. Lastly, while we used very conservative statistical method to reduce the chance of false positives, the current study lacks an independent replication which is considered as a gold standard to confirm the findings.

In conclusion, our data suggest that the ratio of lysoPC 18:2 to PC44:3 is associated with OA progression and warrants further investigation in an independent cohort.

## Patients and Methods

### Individual selection

#### OA patients

This post hoc study used a cohort from the previously published Phase III clinical trial of patients with symptomatic knee OA comparing the effect on cartilage volume loss of oral treatment with licofelone, a lipoxygenase/cyclo-oxygenase inhibitor, with naproxen as previously described^16^. The patients (n = 139) selected for the present work were those who completed the study according to protocol (ATP)^16,18,24^, had serum collected at baseline, and MRI performed at baseline and 24 months. Briefly, patients with primary symptomatic knee OA of the medial tibiofemoral compartment, diagnosed according to the American College of Rheumatology (ACR)^40^, were recruited from outpatient rheumatology clinics. Patients had a pain level of no less than 40 mm on the visual analogue scale, radiographic OA grade 2-3 on the Kellgren-Lawrence scale and at least one of the following three risk factors for increased risk of radiographic progression: body mass index (BMI) > 30 kg/m^2^, presence of Heberden’s nodes, or female gender. The original study protocol was approved by a central review board (IRB Institutional Review Board Services, Toronto, ON, Canada) and the institutional review board of the Centre hospitalier de l’Université de Sherbrooke (Sherbrooke, QC, Canada), and all patients gave their oral and written informed consent to participate, including permission for the use of serum to be collected throughout the study for biomarker studies.

#### Human joint tissue samples

Articular cartilage, subchondral bone, and synovial membrane tissue samples were from the biobank of the Newfoundland Osteoarthritis Study (NFOAS) as previously described^12^. OA joint tissues were obtained from total hip or knee joint replacement patients due to primary OA whereas non-OA (control) joint tissues were obtained from hemi-arthroplasty of the hip due to fractures of the femoral neck. All the joint tissue samples were collected within half an hour of the surgery, flash frozen, and stored in liquid nitrogen for later DNA/RNA extraction. Both OA and controls were from the same source population of Newfoundland & Labrador (Canada). OA diagnosis was made based on ACR criteria^40^. Controls were confirmed by their hip X-ray data and the pathological examination of the removed femoral head cartilage. A total of 32 OA and 16 control cartilage samples, 17 OA and 11 control synovium samples, and 40 OA and 9 control subchondral bone samples were included in the study. Non-OA controls were on average older than OA patients (79 ± 10 years vs 64 ± 9 years).

### Serum metabolic profiling

Serum samples collected after overnight fasting at baseline was stored in −80 °C freezer until analysis. Metabolic profiling on serum was performed using Biocrates *AbsoluteIDQ*^*®*^ *p180 kit*, which assesses 186 metabolites including acylcarnitines (n = 40), amino acids (n = 22), biogenic amines (n = 18), hexoses (sum of hexoses) (n = 1), and phopho-and sphingolipids (n = 105). The details of the 186 metabolites are listed in the Supplementary Table 3. The profiling was done using an API4000 Qtrap® tandem mass spectrometry instrument (Applied Biosystems/MDS Analytical Technologies, Foster City, CA) equipped with Agilent 1100 HPLC system at The Metabolomics Innovation Centre (https://www.metabolomicscentre.ca). The complete analytical process (e.g., the targeted metabolite concentration) was performed using the MetIQ software package, which is an integral part of the *AbsoluteIDQ*^*®*^ kit. Concentrations of all metabolites analysed are reported in μM. The complete metabolic profiling method using this kit was as described^41^. The experimental metabolomics measurement technique is describe in detail by US patent US 2007/0004044 and in the manufacturer’s manuals (https://www.biocrates.com/images/p180_Folder_HP_v01-2018.pdf). The kit has been used in over 800 studies (https://www.biocrates.com/resources1/publications/publications-chronological). Our in-house reproducibility of the assay was performed in 23 samples as previously described^41^. The mean of the coefficient of variation (CV) for all the metabolites was 0.07 ± 0.05 and 90% of the metabolites had a CV of less than 0.10.

### Lecithin-cholesterol acyltransferase (LCAT) measurement

Serum LCAT was measured by ELISA (sensitivity 30 ng/ml; Cell Biolabs Inc., San Diego, California, USA). Measurement was carried out in duplicate for each sample in accordance with the manufacturer’s instruction with modification to sample dilution factor (45×) and measurement means were used in the analysis.

### RNA extraction and real-time PCR

Total RNA for human cartilage, subchondral bone and synovial membrane samples was extracted, quantified and treated with DNase as previously described^42^. mRNA levels of PLA_2_G2a, PLA_2_G4a, PLA_2_G5, IL-1β, IL-6, and TNF-α were measured by quantitative PCR method (qPCR) using ABI 7900HT Fast Real-Time PCR System on 96-well plate. Complementary DNA (cDNA) synthesis was done as described previously^42^. *GAPDH* was used as an internal reference gene for data normalization. Primers were designed using NCBI primer-blast tool and validated using a 4-point dilution series of pooled cDNA samples. The details of the primers are listed in Table 4. qPCR was performed in triplicate using 5 µl of cDNA, 10 µl SYBR Green (Power SYBR® Green PCR Master Mix, Applied Biosystems, 4367659), and 0.4 µl of forward and reverse primers in a final volume of 20 µl. Cycling conditions were: 95 °C for 10 min, 95 °C for 15 sec, and 60 °C for 1 min, repeated in 45 cycles, followed by melt-curve analysis. 7900 SDSv2.4 and RQ Manager were used for data collection and analysis. One of the control samples was selected as calibrator, and the relative quantification (RQ) of target genes’ mRNA levels was calculated as fold changes in relation to the calibrator using Livak method^43^.

**Table 4:** Primers used in quantitative PCR (qPCR).

	Forward primer Sequence (5′ > 3′)	Reverse primer Sequence (5′ > 3′)	Amplicon Size (bp)	R2	Efficiency
*GAPDH*	GCAAATTCCATGGCACCGT	TCGCCCCACTTGATTTTGG	106	0.999	94.3~100.8%
*PLA*_2_*G*2*a*	CAACGGATCGCTGCTGTGTC	GCCACATCCACGTTTCTCCA	65	0.998	98.6%
*PLA*_2_ *G4a*	ACAAAGCAGAGGCTCCACAA	TTCAGTGCCTTTGGGTTCGT	115	0.992	98.0%
*PLA*_2_ *G5*	TCCTGGCTTGTAGTGTGCCT	CCGTAGTTTGTCAGGGCGTT	97	0.994	97.5%
*IL-1β*	TGCTCTGGGATTCTCTTCAGC	ATTGCCACTGTAATAAGCCATCA	107	0.996	98.3%
*IL-6*	TGAGGAGACTTGCCTGGTGA	CACAGCTCTGGCTTGTTCCT	109	0.997	96.3%
*TNF-α*	ATGTTGTAGCAAACCCTCAAGC	TCTCTCAGCTCCACGCCATT	91	0.99	94.3%

*GAPDH:* Glyceraldehyde 3-phosphate dehydrogenase; *PLA*_2_*G*2*a*: Phospholipase A_2_ group 2a; *PLA*_*2*_*G4a*: Phospholipase A_2_ group 4a; *PLA*_*2*_*G5*: Phospholipase A_2_ group 5; *IL-1β:* Interleukin 1 beta; *IL-6:* Interleukin 6; *TNF-α:* Tumor necrosis factor alpha.

### Magnetic resonance imaging (MRI) and determination of cartilage volume and loss over time

High-resolution three-dimensional (3-D) MR images were obtained using 1.5 T with an integrated knee coil. These examinations are optimized 3-D FISP acquisitions with water excitation (Siemens, Erlangen, Germany) or 3-D SPGR acquisitions with fat suppression (General Electric, Milwaukee, Wisconsin, USA), as previously described^16^. Knee joint cartilage volume at baseline and 24 months was evaluated at the time of the original study^16^. Briefly, cartilage volume of the knee joint was measured using a specially developed computer program (ArthroLab, Montreal, Quebec, Canada) as previously described^44,45^. Cartilage thickness was defined as the Euclidian distance between the bone-cartilage interface defined by the baseline image and the cartilage-surrounding tissue interface. It was computed in 3D space for each point of the bone surface along the direction perpendicular to the bone surface. Each thickness value measured was expressed as a standardized map representation and designated as a thickness map. Global or subregion volumes were evaluated directly from offset-maps as the sum of elementary volumes. Elementary volume was defined as the volume between the bone-cartilage interface offset-map and its corresponding cartilage-synovium offset-map. Because a patient’s knee cannot reliably be placed in the MRI machine in precisely the same position (orientation and flexion) at each visit, a registration procedure was done to provide a similar standardized view of the tibia and femur for follow-up evaluation over time. Baseline bone-cartilage interfaces were used as a reference. The change in knee cartilage volume was obtained by subtracting the follow-up volume from the baseline volume and expressed as a percentage of cartilage volume loss. Percentage change was calculated by: (baseline knee cartilage volume – follow-up knee cartilage volume/baseline knee cartilage volume) times 100. The change in cartilage volume over time was calculated for the medial and lateral compartments (compartment comprising femur and plateau) of the knee. The reproducibility of the method has previously been shown to be excellent; between-reader agreement of measurement had ICCs ranging from 0.958 to 0.997 for global knee cartilage. Test-retest reliability of within-reader measurements had Pearson correlation coefficients ranging from 0.978 to 0.999^45^. The percentage change in cartilage volume at 24 months was used as disease progression measures.

### Statistical analysis

The following quality control (QC) procedures were applied to the metabolomics data. Metabolites were removed for subsequent analysis if more than 10% of the samples had values below the limit of detection. Then, missing values were imputed by the mean of the given metabolites. Principal component analysis (PCA) was utilized to examine batch effects, which demonstrated that we did not have any batch effect in our experiment (Supplementary Fig. 3); therefore, no correction for batch effects was performed. Among the 186 metabolites, 152 passed the QC procedure and were included in the analysis. The 152 metabolite concentrations were then standardized using the Z-score, 21,952 pair-wise metabolite ratios were calculated as proxies for enzymatic reactions and used in the analysis. Also, we have previously found that metabolite ratios rather than metabolite concentrations themselves were highly correlated between plasma and synovial fluids^46^.

Unadjusted analyses were performed to identify metabolite ratio(s) that associated with cartilage volume changes and linear regression modeling was utilized to adjust for potential confounding factors including age, sex, BMI, and treatment. A metabolome-wide significance level of α = 0.05/21952 = 2.3*10^−6^ was defined after correcting multiple testing with the Bonferroni method. LCAT levels in serum as well as gene expressions were compared between OA and controls by Student’s t test, and Pearson correlation (r) was used to examine the correlations between gene expressions. Multivariable regression modeling was also used to examine the independent correlation between these gene expression and PLA_2_. Unadjusted or adjusted 95% confidence intervals (95% CIs) were provided where appropriate. All the analyses were done in R version 3.4.3 with built in functions for linear regression, Student’s t test, and Pearson correlation coefficient. Visualizations of the results were done with ggplot2 R package^47^.

### Ethics approval and consent for patients to participate

The original study protocol was approved by a central review board (IRB Institutional Review Board Services, Toronto, ON, Canada) and the institutional review board of the Centre hospitalier de l’Université Sherbrooke (Sherbrooke, QC, Canada). The original trial was conducted in compliance with the ethical principles that have their origin in the Declaration of Helsinki (2000) and are consistent with the “Good Clinical Practice” ICH Tripartite Guideline (January 1997) and the applicable laws and regulations of Canada, whichever afforded the greater protection to the individual. Ethical approval for this post hoc study was obtained with the original study, thus further approval was not required. All patients provided informed consent.

## Supplementary information

Supplementary Information

## Data Availability

Data described in the manuscript is available to readers upon the request.
