# Supplementary material for: Activation of The Phosphatidylcholine to Lysophosphatidylcholine Pathway Is Associated with Osteoarthritis Knee Cartilage Volume Loss Over Time
Source: Sci Rep. 2019 Jul 4;9:9648. doi: 10.1038/s41598-019-46185-w (PMC6609700; doi:10.1038/s41598-019-46185-w)
Supplement: Supplementary file 1 — Supplementary Information [file 41598_2019_46185_MOESM1_ESM.pdf]

Supplementary information

Activation of The Phosphatidylcholine to Lysophosphatidylcholine Pathway Is Associated with Osteoarthritis Knee Cartilage Volume Loss Over Time

Running title: Phosphatidylcholine metabolic pathway and knee cartilage volume loss

Guangju Zhai[1*], Jean-Pierre Pelletier[2], Ming Liu[1], Dawn Aitken[3], Edward Randell[4], Proton Rahman[5], Graeme Jones[3], Johanne Martel-Pelletier[2]

[1]Discipline of Genetics, Faculty of Medicine, Memorial University of Newfoundland, St. John's, NL, Canada
[2]Osteoarthritis Research Unit, University of Montreal Hospital Research Centre (CRCHUM), Montreal, QC, Canada
[3]Menzies Research Institute, University of Tasmania, Hobart, Australia
[4]Department of Laboratory Medicine, Faculty of Medicine, Memorial University of Newfoundland, St. John's, NL, Canada
[5]Discipline of Medicine, Faculty of Medicine, Memorial University of Newfoundland, St. John's, NL, Canada

***Corresponding authors:**

Professor Guangju Zhai

Discipline of Genetics, Faculty of Medicine.
Memorial University of Newfoundland.
Craig L Dobbin Genetics Research Centre
St. John’s, NL, Canada, A1B 3V6
Tel:709-864-6683; Fax:709-864-6531
Email: guangju.zhai@med.mun.ca

Supplementary Table 1. Associations between the percentage of the lateral cartilage volume loss and each of the metabolite ratios

Metabolite ratios	Beta	Std. Error	Pr(>|t|)
lysopcac182.pcaec443	-0.214017045	0.041484112	8.53E-07
lysopcac181.pcaec443	-0.280482184	0.061877626	1.26E-05
glutamine.pcaec443	-0.282437292	0.063692508	1.88E-05
methionine.transhydroxyproline	0.085405776	0.019776385	2.99E-05
h1.pcaec443	-0.201635033	0.047630834	4.20E-05
histidine.transhydroxyproline	0.133896264	0.031694163	4.34E-05
c3.pcaec443	0.176094103	0.041738787	4.44E-05
asparticacid.pcaec443	0.20563871	0.048879684	4.65E-05
c4.pcaec443	0.13432126	0.032122452	5.14E-05
lysopcac204.pcaec443	-0.261645701	0.063112247	5.90E-05
c0.pcaec443	0.233216518	0.057406014	8.13E-05
arginine.transhydroxyproline	0.141392435	0.035252103	9.90E-05
pcaec405.pcaec381	-0.141200837	0.035228891	0.000100046
h1.c181	-0.053484959	0.013617778	0.00013542
lysopcac170.pcaec443	-0.182116198	0.046709755	0.000150692
glycine.pcaec443	-0.173924527	0.044628815	0.000151677
creatinine.c181	0.092115277	0.02385926	0.000173525
c51dc.c181	0.033444761	0.008670357	0.00017572
pcaec383.pcaec381	-0.087968051	0.022815492	0.000176851
lysopcac161.pcaec381	-0.05067899	0.01316	0.000179901
pcaac424.pcaec443	0.385132728	0.100197026	0.000184754
alanine.transhydroxyproline	0.064435353	0.016775567	0.000186618
pcaec404.pcaec381	-0.071646452	0.018755024	0.000201492
pcaec425.pcaec381	-0.03866764	0.010133065	0.000204564
isoleucine.c181	-0.042101792	0.011096343	0.0002215
lysopcac170.c181	-0.073061108	0.019300439	0.000228663
alphaaminoadipicacid.pcaec443	0.212495981	0.056657174	0.00025952
methioninesulfoxide.pcaec443	-0.33514345	0.089389991	0.00026077
pcaac322.c181	0.021407459	0.005715791	0.000264479
asparagine.pcaec443	-0.133351243	0.03561279	0.000265284
lysopcac140.pcaec381	-0.039455412	0.010570112	0.00027676
pcaec422.c5	-0.06092616	0.016329287	0.000278396
leucine.c181	-0.043049351	0.01156423	0.000287009
pcaec384.pcaec381	-0.01711204	0.004601398	0.000290905
pcaac321.pcaec443	0.058067464	0.015614658	0.000291009
smohc222.pcaec381	-0.040021658	0.010786619	0.000299997
alanine.pcaec381	0.014037822	0.003789177	0.000306071
lysopcac281.pcaec381	-0.018179274	0.004922086	0.000318712
pcaec402.pcaec381	-0.035402882	0.009590025	0.000320738
citruline.c181	0.033715588	0.009136587	0.000322419
pcaac344.c181	0.022290739	0.006042488	0.000323773
c2.pcaec443	0.231104392	0.06264985	0.000323975
methioninesulfoxide.pcaec381	0.047439374	0.012863757	0.000325127
smc261.smc202	-0.181645538	0.049364174	0.000334696

prolinr.pcaec381	0.024469371	0.006650144	0.00033491
lysopcac160.pcaec381	-0.052822964	0.014363389	0.000337203
pcaac362.pcaec364	-0.072014646	0.019583847	0.000337641
pcaec340.pcaec381	-0.010783589	0.002933506	0.000339136
pcaac403.c5	-0.092949696	0.025286561	0.000339322
c141.c181	0.221027825	0.06014641	0.000340562
asparticacid.pcaec381	-0.024576731	0.006688614	0.000341068
lysopcac203.pcaec381	-0.050717106	0.013807039	0.000342449
pcaac425.pcaec381	-0.014301671	0.003895102	0.000344371
smohc141.pcaec381	-0.044531697	0.012131807	0.000345661
pcaec420.pcaec381	-0.010784925	0.002938819	0.000346696
pcaec361.pcaec381	-0.018857109	0.005139952	0.000348036
asparagine.lysopcac181	0.107946119	0.029425584	0.000348393
pcaec406.pcaec381	-0.023795323	0.006487062	0.000348796
pcaec300.pcaec381	-0.019973546	0.005451891	0.000354448
lysine.c181	-0.053975195	0.014737241	0.000355833
c141oh.pcaec381	0.051844486	0.014155742	0.000355919
pcaec425.pcaec360	-0.0132427	0.003618094	0.000358845
pcaac403.pcaec381	-0.028048741	0.007663925	0.000359215
pcaac321.c181	0.015724052	0.004297101	0.000360004
pcaec341.pcaec381	-0.007433636	0.002032879	0.00036324
pcaac424.pcaec381	-0.048152369	0.013175567	0.000365869
pcaac401.pcaec381	-0.020159492	0.005519383	0.000368712
pcaac402.pcaec381	-0.08373029	0.022937945	0.000371592
pcaec322.pcaec381	-0.018046202	0.004944285	0.000372099
pcaac420.pcaec381	-0.020753475	0.005687922	0.000373711
leucine.pcaec381	0.019057628	0.005223478	0.000374024
pcaac426.pcaec381	-0.009500725	0.002607403	0.000380312
pcaec445.pcaec381	-0.021106769	0.0057936	0.000381164
pcaec382.pcaec381	-0.036247337	0.009951651	0.00038221
pcaec423.pcaec381	-0.016829588	0.004620689	0.000382371
isoleucine.pcaec381	0.018035761	0.004953101	0.000383616
pcaac363.c181	0.016877397	0.004635387	0.000384047
pcaec421.pcaec381	-0.024937519	0.006849392	0.000384255
pcaac404.pcaec443	0.08079884	0.022193329	0.000384466
pcaac364.pcaec381	-0.008375009	0.002300859	0.000385465
histidine.c181	-0.043917298	0.012073188	0.000388696
pcaec424.pcaec381	-0.018261527	0.005020454	0.000388921
pcaac320.pcaec405	-0.194807861	0.053565532	0.000389763
pcaec301.pcaec381	0.024404743	0.006711228	0.000390327
pcaac321.pcaec381	-0.006795222	0.001868775	0.000390623
pcaac323.pcaec381	-0.026350659	0.007253156	0.000395065
pcaac320.pcaec381	-0.0058372	0.001606896	0.000395621
pcaac341.pcaec381	-0.006134374	0.001689165	0.00039701
pcaec362.pcaec381	-0.021064604	0.00580208	0.000398521

pcaec443.pcaec360	-0.010822551	0.00298198	0.000400226
pcaec321.pcaec381	-0.008618666	0.00237499	0.000400779
pcaac421.pcaec381	-0.018255791	0.005032485	0.000402676
pcaec444.pcaec381	-0.010713351	0.002953846	0.000403633
pcaac281.pcaec381	-0.018991735	0.005238006	0.000405289
pcaec360.pcaec381	-0.016523823	0.004559064	0.000407249
asymmetricdimethylarginine.pcaec38	-0.038887438	0.010733097	0.000409057
smohc161.pcaec381	-0.061792564	0.017063507	0.000411669
c4.pcaec381	-0.014574785	0.004025395	0.000412568
h1.pcaec381	0.023255315	0.006423387	0.000412999
pcaec405.pcaec360	-0.011533767	0.003187476	0.000415847
pcaac342.c181	0.017179371	0.004748495	0.000416739
pcaec364.pcaec381	-0.021272444	0.005880789	0.000417589
pcaac386.pcaec381	-0.008762358	0.00242274	0.000418418
pcaac300.pcaec381	-0.011365014	0.003142949	0.00041942
pcaec446.pcaec381	-0.012644712	0.003496908	0.000419517
pcaec385.pcaec381	-0.019171306	0.005303319	0.000421005
methioninesulfoxide.pcaac341	-0.028313425	0.007834212	0.000422326
pcaac383.pcaec364	-0.054731422	0.015144574	0.000422541
methionine.pcaec381	0.016745884	0.004635715	0.000424878
smc261.pcaac341	-0.037258563	0.01031424	0.000424909
pcaac404.pcaec381	-0.009851729	0.002728286	0.000426983
lysine.pcaec381	0.023504727	0.006510036	0.000427616
pcaac386.c181	0.019967276	0.005532845	0.000430143
pcaec422.pcaec381	-0.012149818	0.003366959	0.000430625
pcaac363.pcaec381	-0.007258699	0.002012258	0.000432602
c162.pcaec381	-0.034956638	0.009691441	0.00043303
pcaac383.pcaec381	-0.008362048	0.00231922	0.000435193
pcaec380.pcaec381	-0.036903793	0.010239184	0.000437293
pcaac362.c181	0.023818352	0.006611029	0.000439375
pcaac344.pcaec381	-0.009515313	0.002641695	0.000440686
pcaac361.pcaec381	-0.007220268	0.002005278	0.000442766
pcaec401.pcaec381	-0.017662557	0.00490593	0.000443365
pcaac384.pcaec381	-0.014926183	0.004150143	0.00044916
pcaec424.c5	-0.06389298	0.017765546	0.000449306
lysopcac182.pcaec381	0.023210651	0.006453982	0.000449502
pcaac424.pcaec360	-0.011232884	0.003123643	0.000449889
pcaec401.c181	0.039599003	0.011013212	0.000450669
pcaac385.pcaec381	-0.016181721	0.004500782	0.000451108
pcaac342.pcaec381	-0.00743928	0.002069261	0.000451385
pcaac406.pcaec381	-0.011379308	0.003165977	0.000452797
alanine.pcaec443	-0.103230776	0.028741352	0.000456834
pcaac366.pcaec381	-0.023755656	0.006617668	0.000460021
pcaec401.pcaac341	-0.017504659	0.004877937	0.000461951
pcaec360.c181	0.036527282	0.010183365	0.000464517

c51dc.pcaec381	-0.013680085	0.003814806	0.000465994
pcaac360.pcaac341	-0.025460972	0.00710085	0.00046669
arginine.pcaec381	0.025644707	0.007155286	0.000469312
pcaac383.c181	0.01908422	0.005324886	0.000469402
smc161.pcaec381	-0.024962694	0.006969287	0.000472952
pcaac322.pcaec381	-0.009004595	0.00251399	0.000472985
smohc241.pcaac341	-0.015922081	0.004445377	0.000473114
pcaac343.pcaec381	-0.008773399	0.002450563	0.000475702
c102.pcaec381	-0.007948312	0.002221219	0.000478707
pcaec423.c5	-0.067965716	0.019005848	0.000482594
pcaac406.c181	0.025390372	0.007100562	0.000482959
smc241.pcaec381	-0.024863716	0.006956769	0.000485995
c102.pcaec443	0.066878103	0.018712258	0.000486004
phenylalanine.pcaec381	0.063966564	0.017904392	0.000488304
smc180.pcaec381	-0.030760756	0.008612128	0.000489806
smc181.pcaec381	-0.093071452	0.026065132	0.000491637
pcaac422.pcaec360	-0.011668841	0.003267978	0.000491748
acetylornithoine.pcaec381	-0.015835125	0.004436433	0.000494026
c18.pcaac341	-0.021637955	0.006062199	0.000494049
smc261.pcaec360	-0.013875942	0.003887567	0.000494068
pcaec423.pcaac341	-0.010563021	0.002961768	0.000499013
pcaec365.pcaec381	-0.019261624	0.005401158	0.000499458
pcaec342.pcaec381	-0.014309808	0.004014816	0.00050287
pcaac365.pcaec381	-0.020878152	0.005863993	0.000509664
pcaac341.c181	0.013542828	0.003804526	0.000510974
pcaac361.c181	0.015883532	0.00446339	0.000512822
pcaac240.pcaec381	-0.088772797	0.024958476	0.000516056
smc241.pcaac341	-0.025595778	0.007200388	0.000519736
pcaec386.pcaec381	-0.025529553	0.007181833	0.000519803
c14.pcaec381	-0.036234818	0.010193494	0.000519876
pcaec363.pcaec381	-0.011647629	0.003279315	0.000525058
pcaac403.pcaac341	-0.016565997	0.004665298	0.000526797
prolinr.c181	-0.052424852	0.014765042	0.000527331
c102.c181	0.017397224	0.004902997	0.000531606
smc160.pcaec381	-0.014908494	0.004204717	0.000536474
pcaac380.pcaec381	-0.017464652	0.004927059	0.000538376
pcaac362.pcaec381	-0.010376002	0.002928117	0.000540371
pcaac281.pcaac341	-0.052568307	0.014843712	0.000544366
pcaec383.pcaac341	-0.039203856	0.011072522	0.000545896
pcaac405.pcaec381	-0.017802381	0.0050299	0.000548426
pcaec380.pcaac341	-0.020094759	0.005679987	0.000551273
histidine.pcaec321	0.034679467	0.009811119	0.000557242
lysine.transhydroxyproline	0.106920291	0.030255481	0.000558782
smohc241.pcaec381	-0.0215499	0.006098552	0.000559373
pcaec383.pcaac386	-0.112854094	0.031952326	0.000562593

pcaec343.pcaec381	-0.011941783	0.003381214	0.000562884
glutamicacid.pcaec381	0.048415974	0.013710749	0.000563979
methionine.c5	0.065936482	0.018698987	0.000573895
pcaec363.c181	0.026102534	0.007403215	0.000574627
smohc221.pcaec381	-0.025200858	0.007149234	0.000576348
c51dc.pcaec443	0.110382085	0.031328344	0.000579505
pcaac421.pcaec360	-0.010058423	0.002856589	0.000584056
alphaaminoadipicacid.pcaec381	-0.039182432	0.011129309	0.000585021
pcaec445.pcaac341	-0.012748639	0.003621134	0.000585085
histidine.pcaec381	0.018696556	0.005313551	0.000589071
lysopcac203.pcaec321	0.039825349	0.01132946	0.000596136
pcaac425.pcaec360	-0.013361693	0.003801508	0.000596883
pcaac360.pcaec381	-0.033057847	0.00941007	0.000600626
pcaac426.c181	0.020447176	0.005821538	0.00060207
c3.pcaec381	-0.020568097	0.005861982	0.000609601
pcaec402.pcaac341	-0.018219047	0.005192709	0.000609907
serine.c181	-0.053916528	0.015372966	0.000612755
pcaac380.pcaac341	-0.01615761	0.004608606	0.000615433
pcaac383.pcaec443	0.067511931	0.019273459	0.000622082
pcaec381.pcaac341	-0.031207134	0.008913446	0.000625764
arginine.pcaec321	0.037199924	0.010640006	0.000636398
pcaec365.pcaec364	0.066195023	0.018947117	0.000642021
pcaac341.pcaec300	-0.275338149	0.078825988	0.000643535
pcaec443.pcaac341	-0.017219275	0.004931067	0.000645726
lysopcac182.c181	-0.0486626	0.013935476	0.000645734
glutamine.pcaec360	0.016169839	0.004639822	0.000661397
pcaec446.pcaac341	-0.011528989	0.003310232	0.000666357
methionine.pcaec321	0.017151889	0.004926675	0.000669565
lysopcac140.pcaec321	0.035599665	0.01023423	0.000676353
pcaac426.pcaac341	-0.021940029	0.006309914	0.000679644
pcaec363.pcaec321	0.020746359	0.005966723	0.000679788
kynurenine.pcaec381	-0.017914782	0.005152482	0.000679992
pcaec380.pcaac386	-0.040258006	0.011581004	0.000681654
pcaac404.pcaec321	0.040557696	0.011676203	0.000687926
pcaac422.pcaac341	-0.015231549	0.004385977	0.000689697
pcaec343.pcaac341	-0.013876256	0.003995818	0.000689907
pcaac422.pcaec381	-0.046203402	0.013308008	0.000691903
creatinine.pcaec321	0.025492127	0.007345433	0.00069517
pcaec301.pcaec321	-0.045276271	0.013047308	0.000695909
methionine.lysopcac170	-0.048620261	0.014012817	0.000697011
pcaac425.pcaac341	-0.017061516	0.004917744	0.000697776
pcaac421.pcaac341	-0.010901872	0.003142327	0.000697813
pcaac424.pcaac341	-0.023666874	0.006825002	0.000701857
pcaac403.pcaac386	-0.040943301	0.01180772	0.000702265
pcaac322.pcaac341	0.016776907	0.004838471	0.000702515

pcaac381.pcaac386	-0.041268003	0.011902184	0.000702841
serine.pcaec381	0.024858528	0.007169536	0.000702888
citruline.pcaec381	-0.014098712	0.004073868	0.000718606
pcaec422.c181	0.026018547	0.007519348	0.000719969
pcaac420.pcaac341	-0.01274887	0.003685222	0.000721828
pcaac403.pcaec443	0.213197266	0.061630729	0.000722293
pcaec382.pcaac341	-0.031282869	0.009048934	0.000727711
c0.transhydroxyproline	-0.084163365	0.024372339	0.000737313
asparagine.pcaec381	0.022778495	0.006600412	0.000742762
pcaec420.c181	0.022255386	0.006451754	0.000746725
glycine.pcaec321	0.030626193	0.008879177	0.000747474
pcaec340.c181	0.02232535	0.006473488	0.000748695
smc241.pcaec360	-0.01465544	0.004250118	0.000749953
pcaac402.pcaac341	-0.017564445	0.005097926	0.000757218
lysopcac182.pcaec321	0.033012994	0.009585416	0.000760637
pcaac320.c181	0.012254201	0.003558777	0.000762479
c2.pcaec381	-0.03427095	0.009959564	0.000768636
pcaec403.pcaec360	-0.016998783	0.004940588	0.000769602
c18.pcaec381	-0.024476543	0.007118269	0.00077508
smc261.pcaec381	0.116044234	0.033788164	0.000785917
c51.pcaac341	-0.008017306	0.002334369	0.000785918
pcaac424.pcaac386	-0.034051673	0.009915221	0.000786401
pcaec342.c181	0.03072505	0.008948756	0.000788643
ornithine.pcaec381	0.070625051	0.020576527	0.000791679
pcaac383.pcaec321	0.019768155	0.005761539	0.000795062
asymmetricdimethylarginine.pcaec32	0.015613742	0.004551754	0.000797182
pcaac403.pcaec360	-0.012405205	0.003616681	0.000797919
c3.pcaec321	-0.054892182	0.016006282	0.000799496
pcaec321.pcaac341	-0.042745617	0.012474707	0.000807209
pcaec445.pcaec321	-0.073798864	0.021537589	0.000807395
pcaec341.c181	0.015367089	0.004486061	0.000810122
pcaac361.pcaec443	0.0578963	0.016924847	0.00082321
pcaac406.pcaec443	0.089524418	0.026190844	0.000830569
pcaec343.pcaec321	0.036869582	0.010794376	0.000837702
pcaac360.pcaac386	-0.029756269	0.008712566	0.000838555
pcaec364.pcaac341	-0.023680416	0.006937068	0.00084345
pcaac362.pcaec321	0.016690112	0.004891201	0.000847258
pcaec404.pcaac386	-0.061246073	0.017949604	0.000847716
pcaec403.pcaac386	-0.06039557	0.017705239	0.000850422
c14.c181	0.07103609	0.020828998	0.000852514
pcaac402.pcaec360	-0.014011746	0.004108521	0.000852604
pcaac366.pcaac341	-0.02744723	0.008049313	0.00085412
smohc141.pcaac341	-0.029474072	0.008651864	0.000863431
pcaec404.pcaec360	-0.017094294	0.005018352	0.000864355
smc240.pcaec381	-0.017698659	0.005197986	0.000868593

asparagine.pcaec321	0.019467794	0.005719519	0.000871998
pcaec405.pcaac386	-0.034641275	0.010177406	0.000872009
pcaec362.pcaec321	0.022418389	0.0065879	0.000874302
pcaac401.c5	-0.071580812	0.021037294	0.000875473
pcaac342.pcaec321	0.015720568	0.004620275	0.000875619
pcaac343.pcaec321	0.0135337	0.003978647	0.000878382
pcaec342.pcaec321	0.034302657	0.010084388	0.00087845
pcaac363.pcaec321	0.013722663	0.004035605	0.0008819
serotonin.pcaec321	-0.023104087	0.006795735	0.000883701
pcaac322.pcaec322	-0.137407051	0.040422136	0.000885162
pcaac384.lysopcac180	0.211547195	0.062267211	0.000890825
c142.pcaec321	0.032026268	0.009434127	0.000898913
pcaec382.pcaec443	0.268275498	0.079055784	0.000902638
pcaac322.pcaec321	0.013922472	0.004105067	0.00090862
pcaec424.pcaec360	-0.017749867	0.005234259	0.000909961
lysopcac140.pcaac386	0.050104787	0.014780669	0.000913667
pcaac364.lysopcac180	0.151097063	0.044576992	0.000914623
pcaec423.pcaac386	-0.039205177	0.011584575	0.000931117
c51dc.pcaec321	-0.041211329	0.012191511	0.000943475
citruline.pcaec321	0.020485173	0.006061089	0.000945204
c16.pcaec443	0.205793398	0.060914808	0.000949666
methioninesulfoxide.pcaac386	-0.039497878	0.01169319	0.000951348
lysopcac161.pcaac341	-0.017622912	0.005218405	0.000953853
pcaac361.pcaec321	0.046032973	0.013632351	0.00095489
pcaac420.pcaec360	-0.014055251	0.004162755	0.000955898
pcaec401.pcaac386	-0.103473859	0.030659044	0.000960537
pcaec300.pcaac341	0.016973965	0.005029588	0.000961065
pcaec403.pcaac341	-0.022783013	0.006754161	0.000966364
pcaec443.pcaac386	-0.030223782	0.008965139	0.000972618
pcaac380.pcaac386	-0.054248637	0.016099086	0.000977793
pcaec406.pcaac341	-0.017770536	0.005274273	0.000979054
pcaac341.pcaec443	0.048236761	0.014324847	0.000985425
pcaec424.pcaac341	-0.012174916	0.003615662	0.000985682
c16.pcaec381	-0.035310941	0.010486685	0.000985862
pcaac300.c181	0.022419421	0.006658988	0.000987268
pcaac402.pcaac386	-0.045212871	0.013454218	0.001008264
pcaec425.pcaac341	-0.011067596	0.003295503	0.001015378
pcaec422.pcaac341	-0.013462555	0.004013009	0.00102788
serotonin.pcaec422	-0.098286675	0.029299126	0.001028341
pcaec423.c181	0.033958655	0.01012808	0.001034097
c181.pcaec321	-0.066708376	0.019896575	0.001034665
pcaec444.pcaec321	0.051610178	0.015402357	0.001041449
smc240.pcaec321	0.025827504	0.007708255	0.001042033
glutamine.pcaec381	0.085189664	0.025440867	0.00104933
alanine.c181	-0.027916256	0.008342892	0.00105784

c16.pcaec360	0.033741798	0.010090952	0.001066134
c0.pcaec381	-0.032415171	0.009706844	0.001081693
c141.pcaec321	0.038786127	0.011616577	0.001083688
pcaec341.pcaec321	0.068234126	0.020444334	0.001088396
glycine.lysopcac181	0.134313779	0.040255274	0.001092041
alanine.pcaec321	0.016896192	0.005067797	0.001101228
pcaec402.pcaac386	-0.078782258	0.023636249	0.001104596
pcaac344.pcaec321	0.021491825	0.006449958	0.00110836
pcaac421.pcaac386	-0.030330958	0.009107056	0.001114256
glutamine.pcaec321	0.02471364	0.007424765	0.001121484
pcaac366.pcaac386	-0.052595062	0.015806699	0.001125777
pcaec421.pcaac386	-0.057202642	0.017193134	0.001127001
pcaac425.c5	-0.047127521	0.01417156	0.001132861
lysopcac140.c181	0.071106348	0.021386003	0.001135112
pcaec444.c5	-0.046814138	0.014088769	0.001143054
pcaec321.c181	0.017070372	0.005142475	0.00115567
c101.pcaec381	-0.04030432	0.012145108	0.001159202
pcaac401.pcaac341	-0.013455608	0.004057059	0.001166798
smc240.pcaac341	-0.025621557	0.00772561	0.001167366
pcaec422.pcaac386	-0.037705248	0.011370356	0.001168694
c0.pcaec321	-0.024995371	0.007542711	0.001177453
citruline.pcaac341	0.016429449	0.004960593	0.001184686
pcaec340.pcaac341	0.046665512	0.014094704	0.001189154
lysine.pcaec321	0.050441294	0.015236284	0.001190142
c14.pcaac386	0.043950839	0.01327988	0.001194171
lysopcac204.pcaec381	0.042594454	0.012876123	0.001200352
lysopcac204.pcaec321	-0.051090661	0.015472861	0.001224635
pcaec421.pcaac341	-0.014099596	0.004273394	0.00123503
pcaac405.pcaec443	0.11903204	0.036082882	0.001237231
pcaec382.pcaec321	0.082968666	0.025152104	0.00123794
pcaac343.c181	0.016726656	0.005076846	0.001254297
pcaac364.c181	0.016382922	0.004973775	0.001257744
pcaac426.pcaec321	0.036210511	0.011001099	0.001267411
pcaac401.pcaec360	-0.014336027	0.004359319	0.001279746
smohc221.pcaac341	-0.024707167	0.007516357	0.001285962
c12.pcaec321	0.102127422	0.031070505	0.00128665
putrescine.pcaec381	-0.058168887	0.017709944	0.001296949
lysopcac170.pcaac386	-0.077906719	0.023727793	0.001301987
lysopcac170.pcaec381	0.028911169	0.00880559	0.001302323
alphaaminoadipicacid.pcaec321	-0.039565917	0.01205384	0.00130593
pcaac322.pcaac386	0.041454177	0.012633342	0.001310666
pcaac425.pcaac386	-0.038933963	0.011867471	0.001313257
pcaac341.pcaec321	0.05197772	0.015848116	0.001317526
pcaac343.pcaec322	-0.147801793	0.04506699	0.001318128
lysopcac161.smohc222	0.040595558	0.012379403	0.001319513

lysopcac203.lysopcac181	0.183136853	0.055935786	0.001342341
pcaac383.pcaac341	0.021155109	0.006472003	0.001366058
phenylalanine.pcaac341	-0.013482583	0.004125527	0.001368852
pcaec421.pcaec360	-0.016013205	0.004907137	0.001390711
creatinine.pcaec381	-0.035265335	0.010807382	0.001391482
citruline.pcaac402	0.087909017	0.026958381	0.001401358
pcaec425.pcaac386	-0.039839487	0.012224957	0.001410785
c0.c181	0.063210504	0.019403191	0.001415994
smc241.pcaec364	0.052580275	0.016144542	0.001420128
lysopcac280.pcaec360	-0.014567194	0.004473152	0.001421342
pcaac366.c181	0.045514908	0.01399616	0.001442979
pcaac365.c181	0.04009627	0.012344299	0.001460957
pcaac401.c181	0.038019585	0.011716132	0.001475785
pcaac323.pcaec321	0.034316897	0.010583444	0.00148812
lysopcac160.pcaac341	-0.012948137	0.003999877	0.001514375
lysopcac181.pcaec381	0.039945178	0.012345658	0.001522123
pcaec381.pcaec360	-0.015359638	0.004747579	0.001523648
prolinr.pcaac341	-0.021970799	0.006799778	0.001544344
lysopcac280.pcaac341	0.02123467	0.006572508	0.001545715
histidine.lysopcac181	0.093313532	0.02889317	0.001551887
pcaec403.pcaec381	0.135546063	0.041981504	0.001556408
pcaec362.lysopcac181	0.115477201	0.035782702	0.001564156
pcaec380.pcaec360	-0.011189942	0.003471122	0.001581787
pcaac344.lysopcac181	0.096474741	0.029927231	0.001582199
citruline.pcaec422	0.078683511	0.024441584	0.001604907
acetylornithoine.smohc222	0.008791897	0.002731321	0.001606623
serotonin.pcaec381	0.079108482	0.024576448	0.001606852
pcaec404.pcaac341	-0.015124938	0.004701622	0.001616834
smohc221.pcaec321	0.05685561	0.017674502	0.001617599
asparticacid.c181	0.049253226	0.015314313	0.001621054
leucine.pcaac341	-0.014147165	0.004399883	0.001625292
pcaac425.pcaec321	0.044564488	0.013865384	0.001631972
pcaac385.lysopcac180	0.160241329	0.049857596	0.001632512
pcaac323.c181	0.046072651	0.01434643	0.001646001
pcaac281.c181	0.035927733	0.011189035	0.001648448
h1.pcaec360	0.01800331	0.005614499	0.001672071
asymmetricdimethylarginine.lysopca	-0.04927506	0.015369274	0.001674768
lysopcac280.pcaec381	-0.062289283	0.019431387	0.001677336
pcaac363.pcaec364	-0.035160953	0.010974128	0.001686108
pcaac404.c181	0.01831918	0.005726389	0.001713008
acetylornithoine.pcaac341	-0.032318788	0.010110433	0.001726887
pcaac381.pcaec381	-0.115284946	0.036072022	0.001730298
alanine.pcaac341	0.010719073	0.003354353	0.001732499
pcaec424.pcaac386	-0.061742349	0.019328973	0.001739664
citruline.c141	-0.12707449	0.039782479	0.001739987

pcaac420.pcaac386	-0.042485812	0.013309893	0.001752274
pcaec382.c181	0.067822691	0.021252267	0.001756409
pcaec322.pcaac341	-0.019096075	0.005985257	0.001760921
pcaac341.pcaac386	0.053348294	0.016727586	0.00176818
methioninesulfoxide.c181	-0.075306264	0.023627242	0.001779456
smohc241.pcaec321	-0.073684151	0.023127043	0.00178636
methioninesulfoxide.pcaec360	-0.011480431	0.003605164	0.001795684
pcaec341.pcaac341	0.067562496	0.021219229	0.001798102
pcaec405.pcaac341	-0.014008902	0.004401404	0.001805027
pcaac320.c141	-0.099143794	0.031183686	0.001825303
lysopcac182.pcaac341	-0.01054393	0.003318899	0.001839488
c16.c181	0.073510057	0.023146408	0.001845772
c3.smohc222	-0.021500898	0.006770767	0.001847698
pcaac344.pcaec322	-0.179512571	0.056560816	0.001858102
c3.transhydroxyproline	-0.117787288	0.037126876	0.00186548
c5.pcaec321	-0.089566185	0.028256164	0.001882103
pcaec444.pcaac341	-0.010801745	0.003408129	0.001884434
kynurenine.pcaec321	0.066634351	0.021033863	0.001893209
acetylornithoine.pcaec321	-0.076957792	0.024295855	0.001895807
pcaac343.pcaec422	0.055108779	0.01740711	0.001905848
c51.pcaec381	-0.027627555	0.008726871	0.001906301
c8.pcaec321	0.060279571	0.01904456	0.001910072
pcaec362.c181	0.039852209	0.012592762	0.001913094
pcaac405.pcaac386	0.056298075	0.017816048	0.001942237
pcaac422.pcaac386	-0.030302372	0.009597878	0.001959499
pcaac380.c181	0.032764164	0.010396209	0.001995136
pcaec420.pcaec321	0.03154017	0.010008121	0.001995718
pcaec365.pcaac341	-0.016956215	0.00538189	0.002001145
citruline.smohc222	0.005796177	0.001840184	0.002006413
pcaac342.pcaec322	-0.142343286	0.04519263	0.002006929
glycine.pcaec381	0.038359588	0.012186055	0.002018959
pcaac321.pcaac386	0.063491115	0.020169899	0.002019033
pcaec401.pcaec321	0.032901329	0.010461586	0.002037448
pcaec421.pcaec443	0.180257418	0.057351852	0.00205018
pcaac363.pcaac386	0.040334409	0.012837692	0.002057606
alphaaminoadipicacid.smohc222	-0.014459898	0.004603312	0.002062058
pcaac320.lysopcac180	0.120040377	0.038218386	0.00206395
pcaec361.pcaec321	0.086883585	0.027707307	0.002097982
c5.smohc222	-0.043850486	0.013990674	0.002108
glutamicacid.smohc222	-0.020225195	0.006453616	0.002110234
pcaec386.pcaac341	-0.02105262	0.006722781	0.002126366
pcaec381.pcaac386	-0.047071524	0.015045053	0.002145554
lysopcac281.pcaec321	-0.057007169	0.01822634	0.002152161
smc261.pcaec443	-0.153952573	0.049243395	0.002161573
pcaac343.lysopcac181	0.075051072	0.024016008	0.002170614

pcaec361.pcaac341	-0.024778824	0.007944903	0.002213752
pcaac422.pcaec321	0.053609911	0.017199392	0.002226875
pcaac365.smohc222	0.015394781	0.004943355	0.002246175
alanine.lysopcac170	-0.044475378	0.014284553	0.002251211
pcaac342.pcaac380	-0.179552251	0.057680412	0.002255833
c181.smohc222	0.026994426	0.008675242	0.002264537
c16.pcaec321	-0.084726188	0.027273214	0.002301252
c10.pcaec321	0.068978559	0.022204627	0.002301808
c51dc.pcaec360	0.009002175	0.002900015	0.002318672
pcaec444.c181	0.019550225	0.006304918	0.002343614
pcaec361.c181	0.033376799	0.010776151	0.002369651
pcaec341.pcaec443	0.053387129	0.017240215	0.002374292
asparticacid.pcaec360	-0.014136349	0.004565658	0.002377491
smohc222.pcaec321	0.093060301	0.030090949	0.002404595
pcaec340.pcaec443	0.075097767	0.02428902	0.002410611
pcaac281.pcaec321	0.088173824	0.028540165	0.002428692
pcaec423.pcaec321	0.049469504	0.016016647	0.002435068
pcaac343.pcaac386	0.062067803	0.020117323	0.002460754
pcaac363.pcaec443	0.052776043	0.017110311	0.002467201
lysopcac170.pcaac341	-0.012378209	0.004027628	0.002554878
pcaec301.pcaac341	0.027858822	0.009068444	0.002565005
lysopcac180.pcaac341	-0.010863486	0.003540244	0.002593199
pcaac240.pcaec360	-0.022005057	0.007177404	0.002615111
pcaac320.pcaec443	0.041617908	0.013583412	0.002631534
pcaac300.pcaec321	0.049732375	0.016238915	0.002642529
pcaec361.pcaec443	0.125698071	0.04108041	0.002665219
glutamine.smohc222	0.012074788	0.003954553	0.002719117
c2.pcaec321	-0.075618535	0.024773433	0.002727474
pcaec446.c181	0.022914394	0.007508464	0.002732544
smc160.pcaec360	-0.020825822	0.006824153	0.002732776
leucine.pcaec321	-0.046098083	0.015107389	0.002736366
pcaac344.pcaac386	0.043120273	0.014134641	0.002742166
creatinine.c2	0.123597707	0.04052338	0.002747691
lysopcac140.lysopcac181	0.095289684	0.031304326	0.002799984
c51dc.transhydroxyproline	-0.048955761	0.016092497	0.002815964
pcaac360.pcaec360	-0.008300258	0.002728939	0.002821035
lysopcac160.pcaec321	0.081204556	0.026707937	0.002830735
pcaac402.pcaec443	0.41917049	0.137907092	0.00283912
pcaac385.pcaac386	0.062972215	0.020730594	0.002855637
pcaac405.c181	0.029803836	0.009815828	0.002867569
pcaac426.pcaec443	0.066888196	0.022033839	0.002872921
c10.pcaec364	-0.053517817	0.01763135	0.00287582
pcaec300.c181	0.033141452	0.010921553	0.002883672
c2.pcaac386	0.053197478	0.017543342	0.002903011
smc241.smohc222	0.012784322	0.004221095	0.002936249

pcaec384.pcaac341	-0.017656437	0.005836816	0.002969755
c162.pcaec321	-0.071760222	0.023730368	0.00297923
serine.pcaac341	-0.00768955	0.002544201	0.002994014
pcaac366.pcaec321	0.040918883	0.013543276	0.003003668
pcaec363.spermidine	0.155442971	0.051475541	0.00301856
c101.pcaec321	0.051093606	0.016922107	0.00302233
c16.lysopcac180	0.274957994	0.091100329	0.003033113
smc261.pcaac386	-0.040477935	0.013419209	0.003049766
pcaac342.pcaec443	0.052211045	0.017313512	0.00305725
isoleucine.pcaac341	-0.017294657	0.00574323	0.003098233
pcaac322.spermidine	0.159252859	0.052907649	0.00311065
pcaec342.smc261	0.149234783	0.049597159	0.003121009
smohc222.pcaac341	-0.012694514	0.004219434	0.00312444
pcaac405.c0	0.005996289	0.001993747	0.003134425
smc202.pcaac341	-0.01467838	0.004883009	0.003149305
pcaac386.pcaec321	0.046739113	0.01555098	0.003153869
lysopcac140.c0	0.009148888	0.003047518	0.003187698
pcaac385.smohc222	0.012400508	0.004131234	0.003191928
asparagine.c0	-0.013377238	0.004456885	0.003193608
prolinr.c0	0.002629784	0.000876238	0.003196125
pcaac386.pcaec443	0.061287836	0.020439761	0.003223385
pcaac343.pcaec403	0.188319211	0.062837016	0.003238437
pcaec363.smc261	0.127981668	0.042725787	0.003253682
c2.pcaec360	0.019342951	0.006458997	0.003260591
lysopcac182.c0	-0.001670571	0.00055806	0.003272597
methionine.c181	-0.026929714	0.009002378	0.003294124
pcaac344.pcaac341	0.021762497	0.00727604	0.003298365
c182.smohc222	0.027866921	0.009325935	0.003327564
c181.c0	-0.002882167	0.000964621	0.003329974
pcaac401.pcaac386	-0.039783603	0.013315157	0.003330292
lysopcac280.pcaec321	-0.054031285	0.01809951	0.003357051
smc241.smc202	-0.125455071	0.042040783	0.003368465
pcaec362.smc261	0.081862571	0.027436291	0.003372544
pcaec445.c0	-0.00079512	0.000266502	0.003374544
pcaec385.c0	-0.000908346	0.000304668	0.003396403
pcaec422.pcaec443	0.084097982	0.028209078	0.003398431
pcaac406.pcaac341	-0.047094459	0.015798188	0.003400873
pcaec301.c0	-0.001960409	0.000658693	0.003451166
c141oh.pcaec321	0.04407323	0.014812945	0.003460588
citruline.c0	-0.000745647	0.000250696	0.003471333
c4.pcaec321	-0.073490931	0.024708963	0.00347179
pcaac240.pcaac386	-0.069617965	0.023411972	0.003478771
isoleucine.pcaec321	-0.049449747	0.016637905	0.003494673
histidine.pcaec443	-0.100458445	0.033805733	0.003499745
lysopcac181.pcaac341	-0.009184493	0.00309195	0.00351246

lysopcac204.c0	-0.00154309	0.000519677	0.003524613
pcaec365.smohc161	-0.07879357	0.0265366	0.003525485
arginine.pcaec342	0.206151009	0.069487713	0.003552672
pcaac343.pcaac341	0.029480919	0.009939556	0.003560339
alanine.c5	0.042806858	0.014436009	0.003568352
pcaec446.c0	-0.000805889	0.000271808	0.003572303
smohc241.smohc161	-0.041048884	0.0138451	0.003572885
smc241.pcaac386	-0.045422483	0.015326828	0.003586802
pcaec360.pcaec321	-0.087906782	0.029740136	0.003672684
c182.pcaec381	-0.055402541	0.01874741	0.003679618
phenylalanine.c181	-0.08311315	0.028143085	0.003701806
pcaac341.lysopcac180	0.098042796	0.033208406	0.003711804
c14.pcaec443	0.158887511	0.053880289	0.003750953
pcaec343.c181	0.019598102	0.00664603	0.003751582
pcaec445.smohc222	0.007119516	0.002415738	0.003771068
c12.c0	0.003083167	0.001046253	0.003774215
pcaec445.c2	0.078120771	0.026533913	0.003805043
c18.transhydroxyproline	-0.085835668	0.029158235	0.00380964
c162.pcaec443	0.145677725	0.049498016	0.00381758
glutamine.lysopcac181	0.102119832	0.034717893	0.003837144
smc202.pcaec381	0.045503202	0.015470465	0.003838576
pcaec446.smohc222	0.007304277	0.002483418	0.003839457
smc161.pcaec321	0.056992925	0.01938666	0.003856011
pcaac383.pcaec360	0.012659948	0.00430762	0.003865788
smohc221.smohc161	-0.084140882	0.028649147	0.003889582
pcaec380.c181	0.060087395	0.020463814	0.003897445
alphaaminoadipicacid.pcaec360	-0.01508195	0.005141097	0.003929099
serotonin.c0	-0.001929343	0.000657725	0.003932059
pcaac384.pcaec364	-0.05440785	0.018548775	0.003933551
c4.transhydroxyproline	-0.091647057	0.031252077	0.00394214
alphaaminoadipicacid.pcaec364	0.056641315	0.019319574	0.003950517
pcaec362.pcaac341	-0.031724212	0.010827151	0.00397148
c51dc.c5	-0.039625078	0.013524061	0.003972585
smc261.c0	-0.000750867	0.000256374	0.003986692
smc160.pcaec321	0.095943233	0.032774858	0.004004269
pcaec385.smohc222	0.00848436	0.002898456	0.004005991
pcaec386.smohc222	0.010320336	0.003525739	0.004006704
pcaec364.smohc161	-0.070430511	0.024068205	0.004017049
methionine.lysopcac181	0.076651405	0.02619785	0.004022178
c18.pcaec360	0.030666403	0.010485874	0.00403828
serine.pcaec321	0.068890172	0.023569643	0.004059187
pcaec446.smohc161	-0.034574661	0.011830295	0.004062645
pcaac322.lysopcac181	0.060962299	0.020864788	0.00407215
pcaac405.pcaec321	0.075555116	0.025861827	0.004075683
c141.pcaec364	-0.049175207	0.016832623	0.004076532

pcaac322.pcaec443	0.059442413	0.020348416	0.004078898
pcaec421.pcaec444	0.412373561	0.141360312	0.00412905
lysopcac203.pcaac386	0.046075859	0.015795943	0.004132016
c4.c0	0.002442058	0.000837771	0.004156948
pcaec301.pcaec443	-0.122939265	0.042290518	0.004257408
smc261.smohc222	0.006899787	0.002374439	0.004272248
pcaac364.pcaec321	0.055547204	0.01911686	0.004274752
lysopcac161.pcaac386	0.056795952	0.019551068	0.004283252
c51.pcaac240	-0.041111776	0.014158886	0.004301351
smc260.c0	-0.001495887	0.000515815	0.004347576
pcaac363.lysopcac181	0.061161887	0.021114553	0.004391841
pcaac381.pcaec360	-0.009864124	0.003405436	0.004393004
smc180.pcaec321	-0.091596259	0.031652953	0.004430252
smohc141.pcaec321	0.125202998	0.043270672	0.004434007
creatinine.pcaec364	-0.066926043	0.023188007	0.004531333
pcaec424.pcaec321	0.061587354	0.021342011	0.004538143
pcaac420.c181	0.034182239	0.011847249	0.004544754
lysopcac281.pcaec443	0.109345746	0.037957632	0.004606533
pcaac343.pcaec443	0.057149915	0.019847831	0.004624847
pcaec401.pcaec422	0.147469671	0.05127462	0.004671042
prolinr.pcaac386	0.057640882	0.020061913	0.004711997
lysopcac182.transhydroxyproline	0.076612069	0.026669633	0.004719265
pcaec402.pcaec360	-0.015352741	0.005348055	0.004746335
c10.pcaac341	-0.01779775	0.006209902	0.004813075
pcaac381.pcaac341	-0.016602098	0.005793648	0.004819654
asymmetricdimethylarginine.smohc1	0.019094151	0.006664505	0.004827078
h1.pcaac380	-0.088427563	0.030865579	0.004828903
pcaec422.smohc222	0.018601294	0.006497163	0.004856827
pcaac425.c181	0.02195192	0.007674703	0.004895862
pcaec363.smohc222	0.010798867	0.003776389	0.004906365
c5.pcaac341	-0.019285159	0.006750485	0.004946169
lysopcac204.pcaac341	-0.01074472	0.003762332	0.004960713
pcaec420.smohc161	-0.034447635	0.012070371	0.004989764
pcaac364.pcaac386	0.046355539	0.016254112	0.005019069
pcaac380.c0	-0.001013969	0.000355649	0.00503237
isoleucine.smohc222	-0.010784632	0.003783026	0.005035944
asparagine.c181	-0.035198288	0.012356066	0.005067859
serotonin.smohc222	0.014427369	0.005065535	0.005075702
pcaac401.pcaec443	0.12855985	0.04515213	0.005089003
pcaac422.c5	-0.067230106	0.023644488	0.005147921
pcaac380.smohc222	0.009186383	0.003231425	0.005156243
leucine.smohc222	-0.010901607	0.003835191	0.005160944
pcaac404.lysopcac181	0.126700435	0.044575109	0.005162722
smohc161.pcaec321	-0.10351289	0.036427133	0.005174358
pcaec383.pcaec321	0.079242507	0.027888676	0.00517828

pcaec363.pcaac341	-0.047065382	0.016576505	0.005210607
smohc141.smohc161	-0.096299901	0.033936666	0.005236035
pcaac342.pcaac386	0.055274826	0.01948587	0.005251128
pcaec386.smohc161	-0.101251807	0.035698333	0.005256477
pcaec421.c181	0.037806147	0.013339035	0.005288694
pcaac420.c0	-0.000971453	0.000342987	0.005318701
pcaac240.pcaec321	-0.068471981	0.024180039	0.005327759
asymmetricdimethylarginine.c0	0.005784135	0.00204284	0.005333067
pcaec341.lysopcac180	0.115006509	0.04062192	0.005337396
pcaec321.pcaec364	0.069561256	0.024593734	0.005380558
pcaac381.smohc222	0.021196861	0.007495396	0.005387381
isoleucine.pcaec360	0.020658896	0.007305742	0.005390918
ornithine.c0	0.003151733	0.001115188	0.005415954
ornithine.c181	-0.09549545	0.033797183	0.005426258
methioninesulfoxide.c0	-0.009822044	0.003477924	0.005449218
asparticacid.pcaec404	-0.073082554	0.025890372	0.005470797
smc240.smohc161	-0.085958964	0.030459902	0.005482614
c142.pcaec381	-0.069104158	0.024493082	0.005493312
pcaac360.c181	0.051961763	0.018419257	0.005498459
lysopcac280.pcaac386	-0.037586799	0.013339786	0.0055539
pcaac363.pcaec322	-0.109370832	0.038856276	0.005601335
pcaec363.c0	-0.001020025	0.000362471	0.005612308
glutamicacid.pcaac386	-0.074081286	0.026327691	0.005616778
pcaac361.pcaac386	0.052368588	0.018613746	0.00562305
pcaec382.pcaac386	-0.081244075	0.028880539	0.005628476
alphaaminoadipicacid.pcaac341	-0.010690751	0.00380066	0.005632462
pcaec406.pcaac386	-0.06088272	0.021649125	0.005642702
pcaac321.lysopcac180	0.110582471	0.039325062	0.005646654
pcaec343.pcaec342	-0.348063895	0.123858641	0.005677233
creatinine.smohc222	0.008732618	0.00310818	0.005687409
pcaac383.pcaac386	0.040963902	0.014583526	0.00569817
creatinine.pcaac341	-0.046259348	0.016475714	0.00571804
pcaac322.smc241	0.190994622	0.068031503	0.005722833
pcaac421.smohc161	-0.032730002	0.011666233	0.005754988
c162.c0	0.001282954	0.000457315	0.005757174
pcaec342.smohc222	0.013927233	0.004965861	0.005770821
pcaac362.pcaac386	0.063257401	0.022559499	0.005780481
c101.c0	0.002241352	0.00079936	0.005782045
pcaec383.pcaec360	-0.017729811	0.006329581	0.005830168
asparticacid.pcaec444	0.265196701	0.094703373	0.005844104
pcaac401.smohc161	-0.049559696	0.017706141	0.005865974
smc240.pcaec422	0.091808809	0.032835025	0.005916789
pcaac365.c0	-0.001353574	0.000484171	0.005923844
pcaac425.pcaec443	0.089907745	0.032167982	0.005936166
pcaac320.c51dc	-0.260205935	0.093327461	0.006056116

lysine.c5	0.045786769	0.016443309	0.006119572
pcaec404.pcaec443	0.323095924	0.116221342	0.006200661
pcaac364.pcaec443	0.053019233	0.019075368	0.006210502
pcaec321.pcaec443	0.054760863	0.019714253	0.006241876
methioninesulfoxide.transhydroxypr	0.130373821	0.046947609	0.006255021
pcaec445.smohc161	-0.028132901	0.010139932	0.006301413
c4.smohc222	-0.023274002	0.008391361	0.00631791
acetylornithoine.c0	-0.000854298	0.000308267	0.006359842
lysopcac140.pcaec322	-0.101309436	0.036570478	0.006379071
pcaac362.pcaec443	0.066223399	0.023910031	0.00638947
lysopcac181.c181	-0.061178194	0.022116091	0.006453991
glutamicacid.pcaec321	-0.049073206	0.017745904	0.006470945
smohc241.smc260	-0.172652724	0.06245332	0.006486307
pcaac385.c181	0.022953835	0.0083057	0.006502952
lysopcac203.pcaac341	-0.022299778	0.008075329	0.0065437
creatinine.lysopcac170	-0.038651708	0.014002043	0.006563377
arginine.c181	-0.037763494	0.013683353	0.006575189
pcaac321.pcaac341	0.024044219	0.00871958	0.006619426
pcaac322.pcaec422	0.043366773	0.015731589	0.006635333
pcaec380.pcaac343	0.105596903	0.038309839	0.006640656
pcaec343.spermidine	0.146090661	0.053005006	0.006644961
serine.smohc222	-0.016442202	0.005970145	0.006685392
pcaec425.pcaec364	0.026748477	0.009713632	0.006692463
pcaec401.smohc222	0.016147076	0.005864487	0.006699037
pcaac380.smohc161	-0.045117281	0.016400803	0.006746587
pcaac363.pcaec403	0.162762881	0.059177725	0.006756559
spermidine.c181	-0.164093135	0.059674264	0.00676816
pcaec381.smohc161	-0.080210918	0.02918258	0.006792155
pcaec446.pcaec321	-0.125889287	0.045801993	0.006792826
kynurenine.smohc222	0.013889663	0.005055295	0.006812616
pcaec443.pcaec444	0.210782208	0.076743625	0.006831746
asymmetricdimethylarginine.pcaac4(	0.128580012	0.046840794	0.006862034
h1.pcaac341	0.040221718	0.014653959	0.006867488
pcaac363.pcaec422	0.043355932	0.015811295	0.006920817
lysopcac280.c181	0.095265061	0.034757202	0.00694507
pcaac344.pcaec443	0.057092894	0.020836789	0.006962468
pcaac361.lysopcac180	0.161282767	0.05886712	0.006967038
pcaac300.pcaec443	0.067863192	0.024770029	0.006967977
pcaac321.pcaec360	0.014371178	0.005245843	0.006971868
pcaec342.spermidine	0.143327581	0.052372559	0.007029209
glutamicacid.pcaec360	-0.015426025	0.005641751	0.007078556
pcaac300.pcaac341	0.0235425	0.00862167	0.00715309
pcaac364.c51dc	-0.227758059	0.083439565	0.007173729
pcaac360.smohc222	0.016528986	0.006055757	0.007176833
pcaac342.spermidine	0.139911343	0.051317162	0.007240234

smc260.pcaec381	-0.043835005	0.016086482	0.007270341
acetylornithoine.c181	0.022035906	0.008095955	0.007335659
lysopcac203.glycine	-0.164346807	0.060414565	0.00736778
pcaac402.smohc161	-0.053862784	0.019824924	0.007439802
smohc241.c141oh	0.227244441	0.083674073	0.007463146
phenylalanine.pcaec404	-0.05633397	0.020746894	0.007474523
lysopcac140.c162	-0.181234632	0.066764668	0.007490961
serine.pcaec443	-0.124852565	0.046003584	0.007502855
pcaac384.c0	-0.002347835	0.000866756	0.007615558
pcaec341.smc261	0.095339512	0.035217517	0.007650439
pcaec406.c181	0.033066012	0.012224281	0.007699011
lysopcac182.smohc222	0.008531524	0.003157795	0.007769883
pcaac403.pcaec444	0.298123582	0.110399405	0.0077993
c14.pcaec360	0.012086975	0.004476231	0.007802676
lysopcac161.pcaac425	-0.167973515	0.062254372	0.007849014
c141.pcaec381	-0.091011913	0.033732156	0.007851352
pcaac343.c0	-0.002765566	0.001025111	0.007857033
pcaec444.smohc161	-0.022579165	0.008370488	0.007864848
citruline.pcaac425	-0.147932393	0.054850024	0.007874685
glutamine.pcaac386	0.045131404	0.016743174	0.00790893
pcaec401.c0	-0.002236645	0.000829956	0.007922769
pcaac420.smohc222	0.008739888	0.003247306	0.008001382
pcaec365.smc180	-0.074149596	0.02756911	0.008043301
pcaec321.lysopcac180	0.13613488	0.050649411	0.008084599
c3.c181	0.02726251	0.010148413	0.008116967
citruline.pcaec364	-0.071619995	0.026683258	0.008170196
pcaac381.pcaec321	-0.056739496	0.02114021	0.008172957
pcaec386.c0	-0.000897472	0.000334464	0.008187953
glutamine.pcaac341	0.00697113	0.002599379	0.00822219
pcaec380.pcaec321	0.043435551	0.016206078	0.008260604
glycine.smohc222	0.011667036	0.004353136	0.008261924
serine.transhydroxyproline	0.104824679	0.039123654	0.008281339
pcaec365.smohc222	0.011312384	0.004223524	0.008302349
pcaac322.pcaec403	0.201202057	0.075120292	0.008302941
smohc241.c181	0.03030262	0.011330218	0.008395245
pcaac364.c0	-0.001318812	0.000493243	0.008412807
pcaac361.c141	-0.064600853	0.02418265	0.008469798
pcaac341.c141	-0.08112697	0.030389288	0.00851257
pcaec385.pcaac341	-0.013827431	0.005180988	0.008529718
pcaac344.c0	-0.001747083	0.000654899	0.008557907
pcaac360.pcaac365	0.183979028	0.069055017	0.008642298
c51dc.pcaec364	-0.033892602	0.01275049	0.008792248
pcaac322.c141	-0.08638226	0.03249747	0.008792707
pcaec420.pcaec360	-0.010257461	0.003860305	0.008816508
pcaec363.pcaec422	0.068785677	0.025899385	0.008848435

pcaac281.pcaec443	0.104697324	0.039444344	0.008887789
pcaac426.c5	-0.026904332	0.010141475	0.008922985
pcaac421.c181	0.025414706	0.009583517	0.008947705
citruline.c2	0.063108705	0.023797648	0.008948462
leucine.c5	0.042308795	0.015980084	0.009057159
pcaac361.pcaec364	-0.052044974	0.019659695	0.009064859
pcaec421.pcaec321	0.082792748	0.031298663	0.009117077
methionine.pcaec443	-0.088863815	0.033603268	0.009136292
pcaac421.pcaac365	0.167622603	0.063388886	0.009140038
lysopcac160.c181	0.074111655	0.028033358	0.009156941
c101.pcaec364	-0.043693985	0.016545956	0.009232556
pcaec446.c2	0.073672975	0.027922119	0.009290965
c102.c5	-0.021516701	0.008155121	0.009293271
pcaec364.smohc222	0.012643153	0.004792684	0.009304147
c14.pcaec364	-0.026911757	0.010202363	0.009309742
pcaac300.pcaac386	0.043590638	0.016529257	0.009325822
histidine.c0	0.00227133	0.000861819	0.009369668
lysopcac140.pcaec360	0.010662937	0.004047843	0.009403324
c0.lysopcac170	0.049125269	0.018653267	0.009419807
smc240.c5	-0.044714958	0.016985195	0.009446665
alanine.lysopcac181	0.058900459	0.022381859	0.009472304
lysopcac204.smohc222	0.007098605	0.002699672	0.009530353
kynurenine.pcaac386	0.065132139	0.024790076	0.009586072
glutamine.transhydroxyproline	0.07257211	0.027631744	0.009611391
lysopcac203.pcaec360	0.012601767	0.004802049	0.009669447
pcaac366.pcaec443	0.128974242	0.049198245	0.009743334
pcaec425.c0	-0.001476589	0.000564004	0.009838206
lysopcac203.c162	-0.181834142	0.069502896	0.00988873
pcaec402.smohc161	-0.035519732	0.013594305	0.009982003
kynurenine.c181	0.02343322	0.008968681	0.009983554
pcaec321.c141	-0.056787265	0.021734565	0.009984122
pcaac421.c0	-0.001031777	0.00039501	0.010004541
alphaaminoadipicacid.c181	0.057678493	0.022089355	0.010029202
isoleucine.pcaec322	0.261591454	0.100189546	0.01003419
c3.pcaac386	0.060089079	0.023024263	0.010066347
putrescine.c0	-0.002415659	0.000925791	0.010081013
kynurenine.smohc161	-0.047633833	0.018272745	0.010150426
pcaac384.smohc222	0.01341778	0.005151129	0.010207021
c3.pcaac341	-0.009713426	0.003729416	0.01021495
c16.pcaac386	0.058170674	0.022348786	0.010262928
pcaec384.c141oh	0.227267211	0.087321015	0.01026842
pcaec422.pcaec444	0.248163617	0.095352348	0.010270349
asparticacid.pcaac386	-0.046347627	0.017816983	0.010306858
lysopcac182.pcaac402	0.058032892	0.022326876	0.010366451
c18.pcaec321	-0.056214861	0.021657322	0.010470066

pcaec420.pcaac341	-0.01215532	0.004683006	0.010470888
pcaec386.c181	0.033348366	0.012849713	0.010481387
asparagine.lysopcac170	-0.036709402	0.01414541	0.010484748
c51dc.pcaac341	0.01647601	0.006349352	0.010491543
c8.pcaec364	-0.044595699	0.017189814	0.010509066
creatinine.pcaac402	0.048497309	0.018695365	0.010515689
c12.c8	0.118163564	0.045565517	0.01053944
glutamine.smohc161	0.017161096	0.006617563	0.010539498
smohc221.pcaec443	0.13358411	0.051579715	0.010639374
isoleucine.c0	0.001246017	0.000481785	0.010745984
pcaac342.lysopcac181	0.05504068	0.021332212	0.010928024
pcaac402.pcaec444	0.309886846	0.12013405	0.010947898
h1.pcaec364	-0.039744927	0.015427051	0.011044758
pcaac421.pcaec364	0.020976025	0.008143896	0.011064241
pcaac422.pcaec444	0.24623581	0.095624695	0.011084109
pcaac386.spermidine	0.133126578	0.051739243	0.01114515
pcaec424.c181	0.023874079	0.009279032	0.011148918
pcaac343.spermidine	0.132937127	0.051688787	0.011180597
pcaac384.pcaac386	0.037887196	0.014736935	0.011210635
acetylornithoine.c141	-0.076285428	0.029673005	0.011211591
c12.pcaec364	-0.040123973	0.015612064	0.011236533
pcaac344.spermidine	0.136922139	0.053322843	0.011306798
pcaac342.pcaec422	0.045903247	0.017882903	0.011335314
smc261.c2	0.071422467	0.027825121	0.011336712
pcaac422.c181	0.056708498	0.022120319	0.011436679
pcaac422.pcaac402	0.186502797	0.072781966	0.011472908
pcaec404.c5	-0.0706655	0.027613192	0.01157934
pcaec406.c0	-0.001553131	0.000607005	0.011593415
c2.c181	0.040249502	0.015730986	0.011595489
c51.c141	-0.036372995	0.01421641	0.011598382
pcaec322.c181	0.022071689	0.008631484	0.01164323
pcaec363.lysopcac181	0.077249584	0.030211265	0.011647527
pcaac360.lysopcac160	0.184596634	0.07220427	0.011660002
pcaec446.pcaec342	-0.182961526	0.071612939	0.011715146
arginine.pcaec443	-0.134724338	0.05275164	0.011745036
pcaec384.smc160	0.253658687	0.099422323	0.011829292
pcaac426.c0	-0.004603082	0.001804638	0.011849676
serine.pcaac386	-0.051041923	0.020039745	0.01196892
pcaac405.pcaec360	0.005831749	0.002289947	0.011980694
pcaec424.pcaec443	0.105152108	0.04129007	0.011980909
pcaec384.c0	-0.001402433	0.000550878	0.012008894
pcaac341.pcaec360	0.010667945	0.004193037	0.012061812
pcaac383.pcaec404	-0.076567115	0.030096303	0.012066237
pcaec361.c5	-0.039983781	0.015727576	0.01212562
pcaec444.pcaec443	0.060126818	0.023655438	0.012142063

pcaec364.c51dc	-0.209793133	0.082585783	0.012190864
pcaec343.c141	-0.067505324	0.026587285	0.012234095
c2.pcaec300	0.05963171	0.023490275	0.012248704
pcaac364.lysopcac181	0.095783682	0.037736245	0.012259703
c142.pcaec364	-0.053941542	0.021263174	0.012306178
pcaec343.smohc222	0.009877581	0.003895007	0.012336155
c101.pcaec443	0.133447434	0.052654923	0.012389458
smc160.smohc222	0.011641512	0.004596002	0.012437197
lysopcac203.pcaac402	0.10807072	0.042675295	0.012456485
lysopcac161.smohc161	-0.048936208	0.019332116	0.012492226
c141oh.c181	-0.077644504	0.03068203	0.012516871
pcaec321.spermidine	0.12841034	0.050755225	0.01253819
alphaaminoadipicacid.pcaec322	0.116313796	0.046000585	0.012588203
pcaac344.pcaec360	0.010298877	0.004078797	0.012710218
serotonin.c2	0.150603462	0.059698967	0.012788598
glutamicacid.c181	-0.050749614	0.020139511	0.012886531
creatinine.pcaac425	-0.1533169	0.060863601	0.012917269
pcaec385.smohc161	-0.08397045	0.033337934	0.012926329
lysopcac281.c181	0.020251467	0.008044694	0.012975348
pcaec363.pcaac402	0.081032098	0.032195558	0.012992854
c141.pcaec360	0.017192275	0.006835341	0.013051714
pcaec360.pcaec443	0.087548074	0.034811709	0.013062341
smc240.lysopcac181	0.074549121	0.029645047	0.013068666
pcaac405.c2	-0.151852038	0.060407047	0.013100944
c18.smohc222	0.020809798	0.008278396	0.013103278
pcaec301.pcaec364	0.033758578	0.013444741	0.013204172
pcaac362.smc261	0.080910298	0.03222871	0.013218802
c18.lysopcac170	0.126413035	0.050394416	0.013291587
pcaac341.glycine	0.103216548	0.041157374	0.013313968
c141oh.c0	0.003239683	0.001291851	0.013316412
methionine.pcaac386	0.040080766	0.015984967	0.013330057
pcaec342.c141	-0.058544094	0.023349584	0.013334287
pcaac421.c5	-0.029910346	0.011938674	0.013404863
pcaec363.c141	-0.046693247	0.018639698	0.013415339
pcaac403.c181	0.03372539	0.013464869	0.013427956
c141oh.pcaac386	0.053393993	0.021318907	0.013433681
smc202.pcaac402	0.10110723	0.040378816	0.013454369
c51.smohc161	-0.014746923	0.005890429	0.01346991
serotonin.pcaac341	-0.007942391	0.003175144	0.013546909
c51.c0	-0.001161573	0.000464529	0.013579377
c10.pcaec381	-0.074999758	0.030000462	0.01360087
pcaac420.pcaec321	-0.06830473	0.027327787	0.01361895
glycine.transhydroxyproline	0.04969127	0.019933907	0.013865455
pcaec385.c181	0.022699114	0.009107994	0.013887197
glycine.c181	-0.053117932	0.021315001	0.013893716

pcaac406.pcaec321	0.056822813	0.022804707	0.01390607
pcaac363.pcaac343	-0.079122118	0.031872016	0.014254807
pcaac383.c0	0.002192789	0.000883612	0.014288346
smc202.pcaec321	0.082397972	0.033205187	0.014293709
histidine.lysopcac170	-0.036164272	0.014574073	0.014296298
pcaac384.c181	0.01745194	0.007042479	0.014423937
c102.smohc222	0.025715207	0.010383061	0.014479926
pcaec423.c0	-0.001588481	0.000641675	0.014523728
phenylalanine.pcaac386	0.06565599	0.026533402	0.014564864
glutamine.c0	-0.001057159	0.000427525	0.014632134
pcaac421.c2	0.097937691	0.039648264	0.014733455
c12.pcaac341	-0.022708409	0.009193152	0.014734226
pcaec341.c141	-0.051472581	0.020846482	0.014774386
arginine.smohc221	0.141440148	0.057331683	0.014856532
pcaac403.smohc161	-0.032019662	0.012984523	0.014898789
smc160.smohc161	-0.162014125	0.065758851	0.01498755
lysopcac203.pcaec322	-0.146077972	0.059291347	0.014988743
pcaec445.c181	0.027606481	0.011213468	0.015062011
pcaec340.smc261	0.142512042	0.057898032	0.015080993
pcaac385.pcaec321	0.076245175	0.031010967	0.015193146
pcaac421.smohc222	0.00885459	0.003604702	0.015284557
pcaac381.arginine	-0.138171586	0.056259757	0.01530256
pcaec401.pcaec300	-0.044313383	0.018051623	0.015349208
c2.transhydroxyproline	-0.077876466	0.031750444	0.015433013
lysopcac203.pcaec403	0.127015886	0.051856907	0.015573746
pcaec424.smohc222	0.022811043	0.00931764	0.015623335
pcaac401.smohc222	0.051859761	0.021203489	0.015720882
pcaac366.pcaec360	-0.01123328	0.004594324	0.015753389
pcaec343.c5	-0.036755266	0.01503531	0.01577171
smc260.smohc222	0.011495747	0.004704268	0.015809911
pcaec380.pcaec443	0.190402982	0.0779995	0.015919506
pcaec385.c141	-0.0737015	0.03020464	0.015962067
smc260.pcaec360	-0.018571335	0.00761624	0.016033521
pcaac322.pcaec364	-0.026346348	0.010825729	0.016234545
c14.pcaec380	-0.088548832	0.036388748	0.016245985
pcaec423.smohc161	-0.019503561	0.008017839	0.016284335
pcaec365.c181	0.022507494	0.009255741	0.016318274
pcaec401.lysopcac181	0.111361702	0.045864801	0.01647816
c12.pcaec381	-0.076539376	0.031543427	0.016546454
pcaec423.pcaec360	-0.007957495	0.003280168	0.016569827
citruline.pcaec361	-0.020393363	0.008413742	0.016662984
c16.pcaec364	-0.054266296	0.022391411	0.01667549
smohc241.c2	0.098903894	0.040850607	0.0167822
pcaac381.pcaec322	0.143476793	0.059265358	0.016790614
kynurenine.pcaac341	-0.017783189	0.007348477	0.016832197

pcaac360.smohc161	-0.047950822	0.019852588	0.017039024
c142.pcaac402	0.038339652	0.015875084	0.017050689
c141oh.pcaec364	-0.032113301	0.013297278	0.017053172
pcaac320.smohc161	-0.071028925	0.029422043	0.017093064
pcaac422.pcaec300	-0.055764901	0.023115902	0.017171228
c142.c0	0.001636569	0.000678418	0.01717453
pcaac342.c5	-0.023046448	0.009560278	0.017250743
pcaec424.smohc161	-0.02675639	0.01110422	0.017299592
h1.pcaac386	0.03936721	0.016339756	0.017312301
c51.pcaec321	-0.045147428	0.018771939	0.017506064
smohc161.pcaac425	0.125245526	0.052078448	0.017511071
c3.c0	0.001726445	0.000717906	0.017515889
pcaac343.c141	-0.078944929	0.03283931	0.017555356
asparagine.transhydroxyproline	0.046177465	0.019215044	0.017591433
lysopcac161.pcaac406	0.109173985	0.045430256	0.017595192
c102.smc181	-0.049242204	0.020497374	0.017629595
pcaec403.smohc222	-0.027712944	0.011537396	0.01764606
pcaac420.pcaac365	0.123966731	0.051639585	0.017710706
pcaec420.pcaec443	0.058428603	0.024342589	0.017727038
methionine.c18	0.099320113	0.041384474	0.017742171
pcaec403.smohc161	-0.051402296	0.021422852	0.017766452
pcaec384.c181	0.019185325	0.007997194	0.01778541
pcaec423.pcaec443	0.091261553	0.038048024	0.017804901
kynurenine.pcaac323	-0.270153219	0.112666345	0.017840913
smc160.pcaac341	-0.017037105	0.007114308	0.017983823
pcaac281.pcaec364	-0.044399494	0.01854404	0.018007035
pcaac381.pcaac365	0.097252539	0.040653941	0.018104642
pcaac342.c141	-0.062831439	0.026286252	0.018195988
c181.pcaac341	-0.010701678	0.004482361	0.018327937
pcaec364.c0	-0.000934628	0.000391573	0.018359259
pcaac344.c51dc	-0.214180675	0.089758164	0.018390768
pcaac420.c2	0.078294209	0.032826491	0.018443828
pcaac320.pcaac240	-0.052470285	0.02200776	0.018488123
pcaac383.lysopcac161	0.133461481	0.055995571	0.018523945
c102.pcaec360	-0.013732601	0.005764014	0.018570211
c162.c8	0.128185769	0.053859686	0.018690378
pcaec446.spermidine	0.121223815	0.050952186	0.018730634
pcaac384.pcaec443	0.075794845	0.031868121	0.018768557
pcaec362.c141oh	0.177835145	0.07480452	0.018820345
lysopcac182.pcaec404	0.031624427	0.013321842	0.018990077
kynurenine.pcaec443	0.087972394	0.037064	0.01900741
pcaec402.c181	0.040469183	0.017051826	0.019018361
lysopcac182.c14	0.091407838	0.038521486	0.019038118
leucine.c0	0.001063329	0.000448399	0.019113119
pcaec385.pcaec361	-0.019278204	0.008130364	0.019125752

pcaec404.smohc161	-0.038383417	0.016188613	0.019131881
smc260.pcaac341	-0.095305574	0.040219956	0.019201735
pcaac362.pcaec403	0.117572767	0.049697146	0.019393107
pcaec322.pcaec443	0.088893521	0.037600078	0.019473708
pcaac361.pcaac365	-0.065725473	0.027808428	0.019507805
pcaac420.pcaec364	0.027124547	0.011479922	0.019544601
kynurenine.smc160	-0.137107865	0.058029449	0.019547257
lysopcac280.c141oh	0.173797807	0.073567262	0.019562193
pcaec382.pcaec360	-0.015095761	0.006390852	0.019579684
pcaac384.pcaec360	0.008772388	0.00371476	0.019609812
pcaac404.c0	0.002947687	0.001248712	0.019656169
lysopcac182.c2	0.102049714	0.043268931	0.019762429
pcaec425.smohc161	-0.024304612	0.010314265	0.019869554
pcaec405.pcaec364	0.016845057	0.007154035	0.01996138
pcaec423.smohc222	0.011544384	0.004903661	0.019981263
lysopcac182.pcaec322	-0.137279043	0.058316572	0.019991909
pcaac323.pcaac402	0.074281762	0.031561564	0.020016711
smc240.pcaec443	0.089631073	0.038093368	0.02004879
lysopcac170.pcaec360	-0.01125836	0.004788044	0.020130865
c51.transhydroxyproline	-0.114172666	0.048576942	0.020182951
asparagine.c8	-0.105537648	0.0449349	0.02026997
pcaec340.pcaec321	0.133470432	0.056845951	0.020309032
phenylalanine.smohc161	-0.022169059	0.009454269	0.020469756
smohc161.pcaec363	-0.087537031	0.037334302	0.020479866
pcaec363.pcaac384	-0.114381952	0.048815682	0.020561315
pcaac365.pcaec321	0.079905924	0.034125176	0.020645411
smc180.pcaec443	0.127034863	0.054265771	0.02067611
c141oh.pcaac341	-0.012385862	0.005295678	0.020788804
alphaaminoadipicacid.pcaac425	0.116504237	0.049829814	0.020832636
smohc161.c141oh	0.163986889	0.070162476	0.020875395
smc160.c141oh	0.164129539	0.070234487	0.020894999
pcaac361.pcaec360	0.006132515	0.002624722	0.020918429
pcaac386.lysopcac180	0.08768999	0.037615053	0.021199296
pcaac406.spermidine	0.121399481	0.052135047	0.021345962
c16.pcaec444	-0.184207875	0.079187165	0.021473425
glutamicacid.c0	0.001473459	0.000633567	0.021505128
pcaec321.c0	-0.000639191	0.00027485	0.021508246
pcaec365.c0	-0.00077589	0.000333868	0.021599907
serine.spermidine	-0.121490847	0.052299384	0.021652573
pcaac404.c2	-0.127615652	0.054982492	0.021761644
lysopcac140.pcaac365	-0.097291909	0.041933327	0.021809889
pcaec385.c2	0.068993431	0.029752754	0.021880503
c162.c181	0.028503945	0.012311442	0.022085519
pcaec342.c141oh	0.205975099	0.088990422	0.022122982
pcaec406.pcaac365	0.10112361	0.043692478	0.022130677

pcaac383.lysopcac181	0.052426875	0.022652397	0.022132502
c51dc.pcaac386	0.028963815	0.012514583	0.022132638
pcaec383.pcaec300	-0.066364466	0.02867684	0.022143193
smohc241.c0	-0.000790142	0.000341784	0.02227931
c141oh.pcaec444	-0.150342385	0.065046533	0.02230831
alphaaminoadipicacid.phenylalanine	0.128167916	0.05545849	0.022322233
pcaec342.c0	-0.000884783	0.000383263	0.022465346
pcaac380.c2	0.077681677	0.03372703	0.02277071
pcaac364.smohc161	-0.093039469	0.040395036	0.022771102
glutamine.pcaac402	0.177723886	0.07717315	0.022789424
serine.smohc161	-0.026335872	0.011436878	0.022801525
pcaac344.pcaec422	0.039745632	0.017262253	0.022816363
pcaec343.c0	-0.000699394	0.000303875	0.022867192
pcaec420.c5	-0.030529717	0.013267251	0.022893366
pcaac385.pcaac323	-0.223669505	0.097253951	0.02296814
pcaec386.lysopcac180	0.131914915	0.057408935	0.023087339
pcaec422.c0	-0.001201839	0.000523084	0.023099512
pcaac406.pcaac386	0.20154927	0.087864391	0.023319511
pcaac383.pcaac365	-0.063610057	0.027731296	0.023323481
smc160.pcaec364	0.050135677	0.021859941	0.023341514
pcaec383.smohc222	-0.018485708	0.008060716	0.023352574
pcaec360.c141	-0.077733071	0.033921097	0.023454817
pcaec365.pcaec361	-0.022361259	0.009763009	0.023524939
pcaec446.pcaec361	-0.019472425	0.008502282	0.023533737
methioninesulfoxide.pcaac402	0.27244734	0.118987964	0.023566757
pcaac320.c0	-0.000662793	0.000289468	0.023567356
lysopcac204.c2	0.088805923	0.038819928	0.023690419
pcaec444.c141oh	0.149244456	0.065264574	0.023743039
lysopcac140.transhydroxyproline	0.096444626	0.042176164	0.023746123
leucine.pcaec360	0.016967253	0.007423154	0.02380561
pcaac362.pcaac341	-0.037112515	0.016237865	0.023815622
c18.c0	-0.001547442	0.000677174	0.023840119
methionine.pcaec445	0.121355634	0.053111584	0.023853919
smc161.smc261	0.129337818	0.056608508	0.023862447
c141.pcaac402	0.033585146	0.014704207	0.023906336
pcaec321.c51dc	-0.211652724	0.092697479	0.023953942
pcaec341.spermidine	0.118669638	0.051980207	0.023971236
smc260.c2	0.115612498	0.05064207	0.023973871
pcaac366.pcaac343	0.093451845	0.040943334	0.024002114
pcaac420.pcaec361	-0.041641414	0.018244567	0.02400618
pcaec443.smohc161	-0.032546186	0.014263872	0.024047399
c14.pcaec444	-0.201022197	0.0881166	0.024071787
pcaec365.pcaec321	-0.088120453	0.038704406	0.024350738
pcaec382.pcaec300	-0.032778589	0.014397279	0.024352825
pcaec423.pcaec444	0.245388127	0.107787923	0.024361324

lysopcac280.transhydroxyproline	-0.133227525	0.058522967	0.024366417
pcaec403.pcaec444	0.261656571	0.11499039	0.024430097
pcaac300.c141	-0.06721594	0.029543048	0.024447242
pcaac404.pcaec404	-0.062434021	0.027443566	0.024458934
pcaec443.pcaec364	0.014791906	0.006503786	0.024498644
pcaac364.smohc222	0.010333765	0.004544542	0.024527555
pcaec361.smc261	0.115706434	0.050894968	0.024555339
pcaec382.smohc161	-0.04678333	0.020581406	0.024576901
pcaec385.c51dc	-0.211153114	0.092932068	0.024636644
pcaac361.pcaec403	0.118474304	0.052148823	0.024653602
c12.glycine	-0.051025662	0.022461103	0.024660776
c141.pcaec444	-0.244196178	0.107508727	0.024681058
smc240.smc202	-0.084640868	0.037274104	0.024720811
pcaac344.lysopcac180	0.102141177	0.044981041	0.024721313
pcaac363.asparagine	0.088134311	0.038826662	0.024772236
pcaec384.lysopcac180	0.11471004	0.050645808	0.025085453
pcaac320.pcaec321	0.066015178	0.02915617	0.025133022
smc202.pcaec443	-0.135079892	0.059664664	0.025146127
pcaec322.c141	-0.060950244	0.026921959	0.02514768
ornithine.smohc161	0.020722671	0.009155897	0.02518852
pcaec381.pcaec444	0.225562211	0.099766484	0.025341533
pcaec365.c51dc	-0.208878475	0.092399423	0.025360489
pcaac362.spermidine	0.121075437	0.053587185	0.02543653
pcaec321.transhydroxyproline	-0.056527425	0.025035183	0.025531812
smohc141.c141oh	0.165140612	0.073155703	0.025566013
c4.c5	-0.031161401	0.013810134	0.025628228
pcaec364.c181	0.021074713	0.009350826	0.02579793
lysopcac203.lysopcac170	-0.062012133	0.027519033	0.025820791
pcaac421.pcaec321	-0.071163129	0.031613734	0.025976836
c0.pcaec322	0.133441125	0.059285502	0.025989655
smc241.c0	-0.000803666	0.000357107	0.026011043
pcaec445.transhydroxyproline	-0.070952306	0.031530141	0.026023566
pcaec342.pcaac341	-0.046729506	0.020770665	0.026057244
pcaac341.c51dc	-0.200246297	0.089012386	0.02606627
pcaec422.pcaec360	-0.007063542	0.003144608	0.02628909
c8.pcaac341	-0.016256554	0.007246078	0.026469713
pcaac383.smc261	0.097543089	0.043479215	0.026473371
pcaec402.pcaec443	0.143766511	0.064096653	0.026504929
lysopcac180.smohc161	-0.038336315	0.017094998	0.026532631
putrescine.pcaac386	0.04484095	0.020002684	0.026585574
pcaac320.spermidine	0.115033594	0.051323168	0.026611359
pcaac363.c141	-0.05430852	0.024234217	0.026636114
histidine.methionine	-0.158747364	0.070845672	0.026651811
pcaec384.c141	-0.090146707	0.040233634	0.026662987
pcaec386.pcaec361	-0.0212607	0.009494526	0.026751014

c10.pcaac402	0.036694621	0.016392244	0.026799266
pcaec300.pcaac343	-0.079849417	0.035710511	0.026967711
lysopcac161.c181	0.046866105	0.020999694	0.027255636
pcaac362.pcaac380	-0.119335864	0.053480882	0.027281228
lysopcac281.transhydroxyproline	-0.059027368	0.026460293	0.027320851
pcaec406.lysopcac160	0.138240787	0.061974671	0.027333694
pcaec365.pcaac386	-0.027580884	0.012364823	0.027334284
c102.c0	-0.003719135	0.001667859	0.027382395
asparticacid.pcaac402	-0.122246541	0.054830143	0.027405212
pcaac426.lysopcac180	0.090966121	0.04080944	0.027439543
pcaec386.spermidine	0.115142988	0.051658557	0.027447874
asparagine.c102	0.095512066	0.042854351	0.027459044
pcaec300.c141	-0.04816883	0.021618007	0.027498577
c51.c2	0.096334845	0.043282231	0.027665956
h1.smohc222	0.029358587	0.013218292	0.027988849
c8.pcaac402	0.033772516	0.015212298	0.028056725
lysopcac140.pcaec364	-0.030063682	0.0135462	0.028107888
pcaac360.pcaec444	0.164284453	0.074205809	0.028488926
asymmetricdimethylarginine.lysopca	0.135836033	0.061375709	0.028539258
pcaec321.pcaec360	-0.015496335	0.00700244	0.028553236
pcaac323.smohc161	0.038616279	0.017458364	0.0286298
pcaac300.pcaec405	-0.094404246	0.042691711	0.028672668
lysopcac140.pcaac406	0.089266179	0.040381271	0.028723551
pcaac380.pcaac402	0.085444714	0.038656033	0.028737654
pcaec404.arginine	-0.179723726	0.081394068	0.028902421
pcaec444.smohc222	0.017405962	0.007885454	0.028953708
lysopcac181.pcaec404	0.052538894	0.02380505	0.028975316
pcaac426.pcaec322	-0.13316431	0.060336179	0.028975961
citruline.spermidine	0.114593385	0.05194253	0.02903907
pcaac320.c5	-0.014570455	0.006606358	0.029084723
lysopcac281.c51dc	-0.212904288	0.096539811	0.029096768
pcaec421.c5	-0.047757766	0.021656371	0.029103925
pcaec342.c5	-0.032922811	0.014936024	0.029175549
pcaec300.smohc161	0.038835967	0.01763607	0.029332643
c18.pcaec340	0.101794811	0.046226869	0.029333057
acetylornithoine.c2	0.060613203	0.027559729	0.029530921
pcaec364.pcaec361	-0.025928081	0.011789567	0.029538198
pcaec385.lysopcac180	0.092511124	0.042082781	0.029605606
pcaec446.pcaac386	-0.070272517	0.031968201	0.029613794
lysopcac182.glycine	-0.039953449	0.018185535	0.029702056
smohc221.pcaac425	0.138949381	0.063247373	0.029707255
pcaac381.c0	-0.001246297	0.00056762	0.029799957
pcaec446.c141oh	0.169891861	0.077431282	0.029913942
creatinine.pcaec422	0.057190674	0.026090346	0.030066709
pcaec422.pcaec405	-0.181671986	0.082901697	0.030111754

c51dc.pcaec444	-0.16520789	0.075395134	0.030125583
kynurenine.pcaec364	-0.027217957	0.012429222	0.03022863
pcaac406.lysopcac180	0.10671943	0.048783627	0.030394286
c141.lysopcac181	0.069737212	0.031895234	0.030480934
pcaec360.smohc222	0.015990329	0.007315522	0.030528624
glycine.pcaac341	-0.010072865	0.004608864	0.030548568
pcaec446.pcaac362	-0.069484687	0.031793674	0.030552596
pcaac420.smohc161	-0.022977214	0.010515869	0.03058885
pcaac341.pcaec405	-0.121130915	0.055450062	0.030626269
c14.lysopcac180	0.07962778	0.036453078	0.030634781
pcaec382.lysopcac170	-0.084068521	0.038486552	0.030637046
pcaec385.spermidine	0.111424305	0.051017372	0.030660658
smc240.c181	0.016988214	0.007781827	0.030734619
pcaec363.lysopcac161	0.113459424	0.051978056	0.030751864
pcaec364.pcaec443	0.093926585	0.043043102	0.030803186
pcaec386.pcaac402	0.084189449	0.038595874	0.030866934
lysine.pcaec404	-0.068494287	0.031413404	0.030934289
isoleucine.pcaac386	0.042364109	0.019434781	0.030980253
lysopcac281.c141	-0.043540796	0.019974961	0.030983288
pcaac384.alanine	-0.122222097	0.056096077	0.031056814
c142.glycine	-0.041462824	0.019039003	0.031134036
serotonin.pcaec360	0.019514664	0.008961001	0.03113789
pcaac383.lysopcac160	-0.055376331	0.025430273	0.031149882
pcaec401.lysopcac160	0.05772084	0.02652079	0.031236586
pcaec363.alanine	-0.06857242	0.031512661	0.031267876
prolinr.pcaec321	0.042706004	0.019640086	0.031390045
pcaac320.glycine	0.148451853	0.068331362	0.031536072
c101.spermidine	0.113201118	0.052113785	0.031562036
pcaec343.pcaec361	-0.016261636	0.00748697	0.031577416
smohc241.smohc222	0.008287329	0.003816669	0.031626796
pcaec420.lysopcac181	0.098375794	0.045311018	0.031644391
lysopcac204.pcaac402	0.042474741	0.019564233	0.031650767
pcaac360.pcaec364	0.015155577	0.006982029	0.03168051
smc160.pcaec342	-0.247934002	0.114270348	0.031753265
pcaec422.smohc161	-0.026846033	0.012377059	0.03180738
glutamicacid.pcaec383	0.207989266	0.095910034	0.031840604
pcaec386.c2	0.07615276	0.035118025	0.031848861
pcaac363.c5	-0.019968518	0.009216762	0.031999468
smc180.c141	-0.072915859	0.033665902	0.032052264
pcaac386.c0	-0.001343786	0.000620592	0.03209427
smc160.c181	0.013836182	0.006393336	0.032185934
methionine.pcaec360	0.007656262	0.003538444	0.032218857
pcaec425.c2	0.090878416	0.042018625	0.032291336
pcaac380.pcaac365	0.105668096	0.048876596	0.032360215
pcaec341.c141oh	0.168083195	0.07777312	0.032418277

pcaec341.c5	-0.015151825	0.007013575	0.032484702
lysopcac204.pcaac365	0.064430178	0.029833526	0.032540004
pcaec365.pcaec443	0.084272936	0.039033288	0.032591827
pcaec360.c0	-0.001278923	0.000592418	0.032606282
isoleucine.c5	0.024850689	0.011513338	0.032637422
pcaec343.pcaec360	-0.009018449	0.004178781	0.032659086
pcaac362.pcaec322	-0.100663369	0.046646641	0.032671353
pcaac405.pcaec404	-0.019932378	0.009236606	0.032673091
pcaec444.pcaec300	-0.037535413	0.017406875	0.03280189
pcaac421.pcaec444	0.184190253	0.085440844	0.032849057
lysine.c0	0.001053592	0.00048889	0.032904371
alphaaminoadipicacid.c2	-0.100978888	0.046859118	0.032914242
alanine.smohc161	0.015950702	0.00740557	0.032999553
pcaac362.c141	-0.043579076	0.020248221	0.033130768
pcaac363.smc261	0.049285088	0.022905706	0.033178255
h1.tyrosine	0.135541491	0.063041042	0.033307072
pcaac363.lysopcac180	0.102511413	0.04768477	0.033329582
pcaec340.pcaec360	0.015403643	0.007167223	0.033377549
asparticacid.smohc222	-0.009404284	0.004376972	0.033425791
lysopcac180.c0	0.003508292	0.001633456	0.033491382
c102.pcaac322	-0.129097075	0.060129855	0.033556348
pcaac281.c162	-0.133296583	0.062133494	0.033690182
pcaec343.pcaac384	-0.149065754	0.069497787	0.03372494
pcaec382.smohc222	-0.043269225	0.020175083	0.033742306
arginine.lysopcac181	0.080195234	0.037406722	0.033808775
c141.glycine	-0.055313439	0.025823372	0.033962776
pcaec421.smohc161	-0.028158455	0.013148768	0.034000807
pcaac405.prolinr	0.043220181	0.020183551	0.034014886
acetylornithoine.c5	-0.024716849	0.011546738	0.034077796
pcaec445.pcaec342	-0.143642239	0.067106775	0.034085085
asparticacid.pcaec321	-0.036299943	0.016963026	0.034131014
pcaac425.smohc161	-0.027923736	0.013049951	0.034146434
pcaac321.pcaac425	-0.132283803	0.061829121	0.034167165
c12.pcaec404	0.036785545	0.017198827	0.034222133
pcaac321.pcaec383	-0.16535823	0.077340427	0.034286883
pcaec363.c5	-0.028607436	0.01339591	0.034496014
pcaec380.pcaec444	0.163774476	0.076709397	0.03454063
smc180.smc181	0.109888811	0.051491461	0.034614092
lysopcac203.c14	0.067708545	0.031743998	0.034711063
smohc222.smohc161	-0.038013685	0.017825352	0.034743914
pcaec340.c141	-0.057398428	0.02692293	0.034794959
pcaec343.pcaec443	0.057653166	0.027053684	0.034869416
pcaac424.pcaec300	-0.048557094	0.022788313	0.034892582
pcaac341.pcaec383	-0.147376596	0.069168197	0.03490018
pcaac300.pcaec360	0.010336256	0.004851536	0.034915863

pcaec362.pcaac385	-0.075317424	0.03535542	0.034934233
pcaec405.pcaec444	0.169050721	0.07942234	0.035084998
c182.pcaac341	-0.006836991	0.003212595	0.03511203
pcaac240.pcaec444	0.278374467	0.130804993	0.03511365
c12.pcaac402	0.032400356	0.015230499	0.035183799
pcaec384.smohc161	-0.039996061	0.018809214	0.035262188
lysopcac181.smohc161	-0.031532076	0.014836963	0.035361459
lysopcac182.lysopcac170	-0.047048554	0.022142494	0.03539797
pcaac383.c2	-0.097453545	0.04586844	0.035412977
methioninesulfoxide.pcaec444	0.189371034	0.089217745	0.035588594
asparagine.pcaec300	-0.028574634	0.01346473	0.035621706
alphaaminoadipicacid.pcaac386	-0.037894504	0.017867523	0.03573509
pcaac380.spermidine	0.110323703	0.052019441	0.035738743
arginine.pcaec360	0.014635079	0.006903808	0.0358212
pcaec420.pcaec322	-0.171745964	0.081058193	0.035912142
smohc141.pcaac425	0.115059796	0.054310555	0.035933256
h1.c5	0.033987072	0.016043509	0.035943751
pcaac364.smc261	0.103031105	0.048637004	0.035949148
pcaec386.pcaac386	-0.033868904	0.015999297	0.036075924
pcaac321.pcaec321	0.054266347	0.025635972	0.03608412
pcaac300.pcaec322	-0.086083567	0.040687856	0.036179146
c16.pcaac406	0.082231725	0.038871224	0.036197833
pcaec401.pcaec443	0.084994601	0.040177786	0.036200302
lysopcac280.pcaec444	0.19382841	0.09163877	0.036228664
pcaec421.pcaec300	-0.048340516	0.022893647	0.036542972
pcaec406.lysopcac180	0.122289219	0.057917734	0.03655134
c101.pcaac402	0.019097584	0.009047252	0.036600184
pcaec340.lysopcac180	0.097993438	0.046434033	0.036643214
pcaac300.lysopcac181	0.063110838	0.029911342	0.03668262
pcaac321.glycine	0.06217935	0.029475922	0.036720655
asymmetricdimethylarginine.c141	-0.041119642	0.019492769	0.036721929
c141oh.pcaac402	0.027032738	0.012821106	0.036812041
smc161.pcaac341	-0.00585048	0.002775005	0.036827752
pcaac322.pcaec360	0.007809199	0.003707084	0.036979043
prolinr.lysopcac180	-0.161862363	0.076839974	0.036985609
glutamine.spermidine	0.105759624	0.0502316	0.037078102
methioninesulfoxide.pcaec321	0.04910073	0.023339105	0.037223773
acetylornithoine.smohc221	-0.100124511	0.047605878	0.037277098
pcaec322.c0	-0.001076171	0.000511964	0.037379443
pcaec365.arginine	-0.093509974	0.044509922	0.037483578
lysopcac204.pcaec404	0.022767176	0.010840332	0.037541737
lysopcac204.smohc161	-0.047998857	0.022858974	0.037581717
c4.c181	0.010843326	0.00516455	0.037600817
pcaac385.pcaac240	-0.050810462	0.024204083	0.037629197
pcaac363.spermidine	0.110064808	0.052463109	0.037746304

smohc241.transhydroxyproline	-0.046233895	0.022048641	0.037840058
pcaec340.spermidine	0.112905339	0.05387222	0.037939736
pcaac363.pcaec383	-0.096760445	0.046205887	0.038091968
pcaec384.pcaec443	0.07756039	0.037038631	0.038098735
c181.pcaec444	-0.132032866	0.063057778	0.038116937
pcaec383.pcaec443	0.235501902	0.112499099	0.038159772
smc160.c0	-0.000696106	0.000332557	0.038175353
pcaac425.pcaec300	-0.030221261	0.014440114	0.038205003
smohc161.pcaec360	-0.026829128	0.012821347	0.038235228
pcaac366.c0	-0.001941377	0.000927964	0.038276477
alphaaminoadipicacid.c0	0.00094016	0.000449431	0.038293952
pcaec343.alanine	-0.064900833	0.031029919	0.038324289
pcaac385.pcaec360	0.007645888	0.003657676	0.038432681
pcaac383.smohc222	-0.010337872	0.004947363	0.038505348
pcaec322.spermidine	0.107116229	0.051318083	0.038713514
pcaac383.pcaac343	-0.049663993	0.023821754	0.038942127
lysopcac181.smohc222	0.006649771	0.003190371	0.038987497
pcaac366.transhydroxyproline	0.068057232	0.03265675	0.039015979
pcaec322.pcaec360	-0.013588367	0.006520954	0.039036022
pcaec406.pcaec342	-0.245668703	0.117903513	0.039050699
pcaec444.c2	0.097429132	0.046762037	0.039062984
glycine.pcaec300	-0.035647468	0.017109515	0.039064941
leucine.pcaec322	0.183567895	0.088135235	0.039128844
lysopcac170.pcaac365	0.101556988	0.048774518	0.039186801
prolinr.pcaec443	-0.097227875	0.046700387	0.0392075
c181.prolinr	0.04946916	0.023771372	0.039292134
pcaec420.pcaec363	0.043569839	0.020936645	0.03929277
pcaac424.smohc161	-0.036149034	0.017377429	0.039367558
c102.c8	0.061711151	0.029667149	0.039377969
citruline.c16	-0.068260873	0.032818968	0.039396237
prolinr.pcaec340	0.118448627	0.056976987	0.039492914
pcaec380.pcaec364	0.017234952	0.008291343	0.0395132
pcaec425.pcaec321	-0.045594914	0.02195611	0.039703258
pcaec422.pcaec364	0.014161758	0.006819866	0.039712094
pcaac426.pcaec300	-0.036946659	0.017814092	0.039950351
citruline.pcaac380	0.080242631	0.038697269	0.039989402
pcaac385.c51dc	-0.193763198	0.093461159	0.040027606
pcaec321.c141oh	0.176516128	0.085173391	0.040099683
ornithine.c2	-0.09693551	0.046794177	0.040185275
lysopcac203.pcaec363	0.047769222	0.02305995	0.040185855
glutamicacid.smc261	-0.062361255	0.030109322	0.040220211
lysopcac160.pcaec403	0.138386402	0.066846537	0.0403105
pcaec340.c141oh	0.179334291	0.086651266	0.040367667
c4.pcaac281	0.010026018	0.004844496	0.040371533
smc241.c141oh	0.148968205	0.071989665	0.040397158

c10.pcaac386	0.037737995	0.018237152	0.040397894
pcaec360.spermidine	0.13222983	0.063914835	0.040440589
pcaac380.lysopcac180	0.110973154	0.053666656	0.040538027
pcaac366.glycine	0.091277556	0.044144476	0.040549745
pcaac360.pcaac402	0.135368976	0.065471156	0.040558165
c4.pcaac406	0.08840934	0.04276475	0.04058407
pcaac343.pcaac425	-0.15110497	0.073097806	0.040601284
pcaac300.lysopcac180	0.060732052	0.029411168	0.040815081
pcaac323.smc202	0.098259052	0.047587451	0.040826758
pcaac362.pcaec422	0.032683615	0.015830925	0.040852504
pcaac300.c162	-0.122074528	0.059151875	0.040929019
pcaec301.smohc222	0.008961282	0.004342722	0.040951179
lysopcac181.pcaac402	0.088923905	0.043097965	0.040972416
smc260.pcaec404	0.04212541	0.020429032	0.041094109
pcaac401.pcaac402	0.338170443	0.164040532	0.041145469
pcaec386.arginine	-0.078838078	0.0382646	0.041258389
pcaac424.acetylornithoine	-0.17088366	0.08295722	0.041300965
smohc222.smc261	0.081255443	0.039449444	0.041316978
lysine.lysopcac181	0.063833452	0.030999338	0.041370269
smc181.c141	-0.050549793	0.024556265	0.0414343
c182.pcaec361	-0.013992931	0.006797581	0.041435576
pcaac426.pcaec383	-0.132772583	0.064506536	0.041458476
pcaac341.spermidine	0.11168757	0.05429024	0.041560845
lysopcac182.pcaec364	-0.057478595	0.027941752	0.041574982
lysopcac161.tyrosine	-0.092494619	0.04496654	0.041586777
c162.transhydroxyproline	-0.040389956	0.019639438	0.041625031
pcaac426.pcaac386	-0.0467721	0.022744008	0.041636342
pcaac420.lysopcac170	0.09051146	0.044019142	0.041663137
glutamine.pcaec364	-0.020336817	0.00989078	0.041667559
pcaec425.c5	-0.029755772	0.014475343	0.041718556
h1.pcaac425	0.07024024	0.034172952	0.041736575
asparticacid.glutamicacid	0.205696061	0.100099677	0.041787678
pcaac425.pcaec444	0.171944097	0.083682179	0.041805778
smc160.pcaec363	-0.089758318	0.04371101	0.0419314
pcaec365.pcaec342	-0.253120621	0.123290498	0.041971375
pcaac360.pcaec300	-0.085803176	0.041826829	0.042134191
pcaac341.pcaac365	-0.053989202	0.026321477	0.042158345
pcaec420.pcaac386	-0.028808185	0.014055949	0.042317626
c101.pcaac281	0.008983088	0.004385708	0.04244391
pcaac364.spermidine	0.105398359	0.051469491	0.042491738
pcaec342.pcaec443	0.06536808	0.031926999	0.042527551
pcaac401.pcaec300	-0.061656204	0.030136193	0.042677525
pcaec343.pcaac402	0.045903807	0.022442135	0.042726389
pcaac342.smc261	0.049770587	0.024342202	0.042807219
pcaac300.c0	-0.000723048	0.000353704	0.042847624

pcaac384.pcaec321	0.07774073	0.038128973	0.043384344
putrescine.pcaac402	0.032481789	0.015931498	0.043389187
c141.pcaac386	0.026376006	0.012940373	0.04344678
glutamicacid.c14	-0.139064213	0.068254203	0.043530676
pcaac380.pcaec360	-0.00817266	0.004014436	0.043696236
ornithine.pcaec360	-0.017179568	0.008438797	0.043699655
pcaec405.c5	-0.054857979	0.026950657	0.043728915
pcaac321.pcaac365	-0.042306321	0.020786903	0.043755186
pcaac424.pcaec444	0.158483859	0.077916377	0.043879168
pcaac300.c2	0.096934062	0.047707532	0.044103057
pcaec443.pcaec300	-0.034362687	0.016921764	0.044222285
smohc241.smohc141	0.196655835	0.096861465	0.044263525
c2.pcaec405	-0.098704018	0.048623995	0.044298015
pcaac300.pcaac365	-0.076799339	0.03783806	0.044324793
phenylalanine.pcaec321	-0.063297056	0.031186119	0.044327895
pcaac383.c162	-0.118809922	0.05855393	0.044388414
lysine.methionine	-0.079596786	0.039240187	0.04445221
pcaec422.pcaec300	-0.055328657	0.027302647	0.044654846
pcaac240.pcaec443	0.206081001	0.101737405	0.044745978
lysopcac203.pcaec444	-0.183386805	0.090558605	0.044803671
pcaac385.pcaec443	0.068010243	0.033584613	0.044805796
putrescine.pcaec364	-0.051827697	0.025604614	0.044898209
pcaac364.pcaec405	-0.108277555	0.053512823	0.044977502
pcaac361.pcaec404	-0.052474324	0.025936141	0.044996515
pcaac383.c141	-0.042806894	0.02116529	0.045070522
c10.pcaac365	0.052659284	0.026048083	0.045163283
pcaac361.spermidine	0.11198799	0.055419616	0.045256622
pcaac406.pcaec364	-0.039098065	0.019364286	0.045429794
c102.pcaac323	-0.143188293	0.070921439	0.045441455
smc180.pcaac425	0.129716027	0.064254641	0.045461451
pcaec405.smohc161	-0.028473237	0.01410672	0.045499825
pcaac362.pcaec361	-0.017263083	0.0085569	0.045602278
pcaac385.pcaac402	0.053533989	0.026539224	0.045631981
pcaac422.smohc161	-0.025584168	0.012686382	0.045684961
c141.pcaec404	0.033864116	0.016793026	0.04569614
pcaec363.pcaec361	-0.015636773	0.007754616	0.045707898
ornithine.pcaac402	-0.049050626	0.024326775	0.04572109
pcaec383.pcaec444	0.294165389	0.145905393	0.045740647
pcaec342.lysopcac181	0.058026404	0.028795594	0.04584921
pcaec342.c2	0.086223659	0.042809169	0.045952679
pcaec301.c181	-0.021435848	0.010646806	0.046035746
pcaac384.c51dc	-0.172620158	0.085756611	0.04608386
methioninesulfoxide.c141	-0.087469348	0.043457907	0.046102054
pcaac362.c5	-0.02097293	0.010422384	0.046149027
putrescine.glutamicacid	-0.119424928	0.059379743	0.046265517

c162.pcaac402	0.043617406	0.021696237	0.046355351
methionine.pcaec300	-0.01121465	0.005579368	0.046392422
pcaac341.pcaac240	-0.028291001	0.014078938	0.046453104
c162.c102	0.09222467	0.045915762	0.046549065
c182.prolinr	0.029506094	0.0146904	0.046552643
pcaac406.prolinr	0.044786103	0.022305573	0.046626388
pcaac365.pcaac240	-0.042200051	0.0210298	0.046751957
pcaac300.c51dc	-0.161648263	0.080555325	0.046752476
pcaec365.spermidine	0.101763243	0.050733893	0.046844294
lysopcac140.pcaec403	0.131851012	0.06575631	0.046917577
pcaec445.c141oh	0.130689844	0.065190944	0.0469633
pcaec384.lysopcac181	0.094176509	0.04698688	0.047007611
asparticacid.c2	-0.114975598	0.057392224	0.047114434
pcaac425.pcaec364	0.016167133	0.0080842	0.047494541
pcaec361.c141oh	0.138960105	0.069509409	0.04756982
c181.c18	-0.100631202	0.050349056	0.047622875
pcaec424.c0	-0.001276283	0.000638736	0.04768124
ornithine.pcaac386	-0.056677208	0.028376054	0.047766726
pcaac381.pcaac402	0.048849496	0.024470024	0.047883445
pcaec420.lysopcac160	0.042849809	0.021466954	0.047907359
pcaec386.pcaec364	0.041869147	0.020979043	0.047942818
pcaac363.c51dc	-0.185015683	0.09272038	0.047980877
pcaac380.pcaec361	-0.0171066	0.008575306	0.048041369
c51.c12	-0.109412498	0.054851225	0.048058336
pcaec445.lysopcac180	0.057042821	0.028613839	0.048188201
pcaec443.arginine	-0.138929474	0.069709208	0.048249532
lysopcac204.pcaec422	-0.066470058	0.033387056	0.048481983
pcaec420.pcaec383	-0.155258658	0.077986162	0.048486912
pcaec401.c5	-0.035823494	0.017994994	0.048498166
pcaec420.smc261	0.065914957	0.033126143	0.04860189
pcaac343.pcaec360	0.010463057	0.005259004	0.048631544
pcaec403.pcaec300	-0.032080515	0.016130285	0.048711232
lysopcac160.spermidine	-0.113840685	0.057247597	0.048741448
c102.spermidine	0.111584937	0.05612232	0.048777432
pcaac386.pcaac385	-0.057674134	0.029012787	0.048817559
pcaec443.transhydroxyproline	0.074395156	0.037426269	0.048829602
pcaac322.lysopcac180	0.085259749	0.04289421	0.048841264
pcaec445.c141	-0.044266528	0.022272092	0.048857119
pcaac363.smohc161	-0.074422025	0.037453831	0.048913128
pcaec383.pcaec380	0.122445838	0.061639752	0.048975792
pcaec341.c0	-0.000899202	0.000452693	0.048991332
smohc161.pcaec300	0.0392426	0.019757084	0.049000556
smc181.pcaec363	-0.033130892	0.016683154	0.049041637
arginine.pcaec404	-0.058714265	0.029592445	0.049243437
pcaac322.c162	-0.115068565	0.057996296	0.049246704

serine.pcaec360	-0.007948689	0.004006697	0.049271163
pcaec362.c5	-0.023415546	0.011807591	0.049356761
pcaec300.pcaec443	0.075786266	0.0382212	0.049386073
pcaac320.pcaac341	-0.031575103	0.015936236	0.049555131
kynurenine.c14	-0.050326688	0.025405364	0.049599666
isoleucine.pcaec443	-0.075148399	0.037959608	0.049742083
c5.c0	0.002751992	0.001390282	0.049770061
acetylornithoine.pcaec322	0.156738572	0.079189054	0.049787353
h1.c14	0.132544158	0.066973873	0.049816178
citruline.transhydroxyproline	0.04349036	0.021978839	0.049850915
pcaec340.pcaec405	-0.111576735	0.056450195	0.050100551
pcaac365.pcaec443	0.076649443	0.038790632	0.050166233
c51dc.smc202	0.070574345	0.035720554	0.050194108
glutamine.smc261	0.041865334	0.021189902	0.050195712
pcaac360.c2	0.104027943	0.052657217	0.050213181
h1.pcaec405	0.078647854	0.03982851	0.050317187
smc241.pcaec342	-0.178549151	0.09042556	0.05033089
pcaec423.pcaec343	0.167648061	0.084908083	0.050339813
pcaec445.pcaac240	-0.031838527	0.016125669	0.050347271
pcaec446.lysopcac161	-0.103562617	0.052470006	0.050422183
arginine.smohc161	0.022239596	0.011268903	0.050446576
pcaac403.pcaec300	-0.026366646	0.013361528	0.050470898
smc180.pcaec363	-0.026404169	0.013401353	0.050824805
pcaec444.c0	-0.001278015	0.000648766	0.050864697
pcaec445.pcaec422	-0.069032758	0.035045646	0.050878798
c3.lysopcac161	-0.172727234	0.087720113	0.050962725
c182.pcaec321	-0.075044068	0.03811693	0.050995994
pcaec362.pcaac425	0.097444879	0.049500973	0.051023998
pcaec402.pcaec321	-0.054182483	0.027527239	0.051049837
methionine.c14	0.052830354	0.02684439	0.05108476
pcaec363.c2	0.066857205	0.033972548	0.051089976
pcaec445.spermidine	0.101035136	0.051344517	0.051111984
pcaac405.pcaec364	-0.034527577	0.017554776	0.05122151
serine.tyrosine	-0.086248446	0.043856425	0.051249374
pcaac422.pcaec382	-0.025517846	0.012979862	0.051325534
c181.pcaec364	-0.029406365	0.014959696	0.051354819
pcaec425.transhydroxyproline	-0.111454819	0.05674558	0.051541087
lysopcac280.c102	-0.08459666	0.043076784	0.051571239
smc180.pcaec300	0.040514008	0.020638449	0.051667703
smohc221.c181	0.02089925	0.010650147	0.051749009
pcaec363.pcaec443	0.051154639	0.026073887	0.051799966
pcaac384.histidine	0.080062957	0.040817529	0.051850141
alanine.pcaec422	0.03589783	0.0183023	0.051862053
c181.c2	0.085450186	0.043568591	0.051874308
smc241.smohc161	-0.029805914	0.015202353	0.051953045

pcaec380.smohc161	-0.024278928	0.012386506	0.052012171
lysine.c8	-0.075596561	0.038607953	0.052255328
c4.pcaec404	-0.025825333	0.013192417	0.052310795
c16.transhydroxyproline	-0.036600413	0.018703775	0.052399034
histidine.pcaec300	-0.023652757	0.012087764	0.052410242
pcaac425.lysopcac170	-0.039913316	0.020415155	0.052609023
creatinine.c0	-0.0007732	0.000395576	0.05266447
putrescine.smohc222	0.004311026	0.002206668	0.052781827
c14.glycine	-0.076832205	0.039333429	0.052815258
c141oh.glycine	-0.056392282	0.028879026	0.052892992
pcaec381.pcaec300	-0.016120746	0.008261078	0.053048678
glutamicacid.pcaec364	0.031427071	0.016107017	0.053080854
smc202.glycine	-0.055408819	0.028405681	0.053143023
c162.smc181	-0.03946952	0.020238765	0.053194819
pcaec423.pcaec422	0.138367749	0.070989179	0.053322023
asymmetricdimethylarginine.lysopca	-0.08443405	0.043327134	0.053368084
c2.pcaac406	0.082556953	0.042364173	0.053369572
pcaac380.prolinr	0.046964845	0.024103999	0.053408107
smc240.pcaec300	-0.01487229	0.007633937	0.053437658
citruline.smc160	-0.091545288	0.046993804	0.05345603
methioninesulfoxide.prolinr	-0.094005516	0.048257123	0.053457921
pcaac361.smc160	-0.141527351	0.072656418	0.053471757
pcaac343.pcaac365	-0.081034918	0.04161021	0.053522809
smc240.c141oh	0.154172006	0.079238859	0.053742856
pcaac386.c5	-0.014394865	0.007398761	0.053753043
c12.pcaec444	-0.199234361	0.102427219	0.053807323
kynurenine.c0	-0.001280476	0.000658531	0.053891049
pcaec380.transhydroxyproline	0.064868092	0.033370228	0.053958214
pcaac281.lysopcac180	0.087726108	0.045151008	0.054073117
pcaec386.pcaac365	0.091213194	0.046951299	0.054101168
c14.lysopcac181	0.078505277	0.040425074	0.054189718
pcaec446.c5	-0.015783222	0.008127563	0.054196669
pcaac300.pcaac281	-0.006834508	0.003519786	0.054220932
c181.pcaec361	-0.021500899	0.011073258	0.054226259
pcaec362.c141	-0.034716038	0.017885369	0.054307794
prolinr.lysopcac170	-0.032729743	0.016866752	0.05437413
methioninesulfoxide.smohc161	-0.035646437	0.018375805	0.054451632
kynurenine.pcaac402	0.106533554	0.054941514	0.054552591
pcaac323.pcaec443	0.077212763	0.039823632	0.054573215
c5.pcaac406	0.105124624	0.054234114	0.054637126
lysopcac182.pcaec300	-0.013672672	0.007055021	0.054679352
pcaac402.pcaec300	-0.032481868	0.016763136	0.054717498
pcaec446.lysopcac180	0.072951972	0.037649425	0.054721371
serine.alanine	0.065774915	0.033951921	0.0547669
lysopcac180.pcaec404	0.038998469	0.020137075	0.054846621

methionine.smohc222	-0.008874861	0.004582727	0.054854147
pcaac404.c141	-0.08807102	0.045494497	0.054944189
lysopcac204.valine	-0.11241231	0.058093527	0.055047947
pcaec360.pcaac404	0.017845697	0.009222537	0.055049599
c102.pcaac402	0.023124736	0.011962898	0.05529439
pcaec363.c141oh	0.176722821	0.091428838	0.055311526
lysopcac204.lysopcac180	0.146004286	0.07555339	0.055365692
pcaac281.pcaec342	-0.236836608	0.122590247	0.055431505
smohc141.smohc222	-0.013816664	0.007151838	0.055435602
smc160.smc202	-0.126352497	0.065406101	0.055446668
c14.c8	0.06920022	0.035861708	0.055718296
c8.pcaec381	0.080850213	0.041935922	0.055930768
pcaec445.lysopcac161	-0.103909254	0.053903508	0.05596298
asymmetricdimethylarginine.pcaac28	0.005730737	0.002973147	0.05598704
lysopcac140.pcaec300	-0.016569751	0.008598519	0.056043804
histidine.pcaec360	0.012142119	0.006303097	0.056128617
c102.pcaec321	-0.12907431	0.067030872	0.056226749
pcaac405.lysopcac180	0.090890662	0.047205003	0.056245606
lysopcac182.pcaac281	0.002723892	0.001415119	0.056320711
methionine.pcaec422	0.040429995	0.021005952	0.056340708
pcaac420.pcaac402	0.064696368	0.033613933	0.056341131
pcaac386.pcaac341	-0.016536491	0.008596166	0.056465535
lysopcac161.c141	-0.051179251	0.026609322	0.056509544
pcaac422.pcaec364	0.013109647	0.006816798	0.056537343
pcaec406.smohc222	0.009625774	0.005005632	0.056556335
pcaac380.pcaac323	-0.15205232	0.079149655	0.056799551
pcaec406.prolinr	0.040862737	0.021270961	0.056801618
pcaac363.pcaec384	-0.097048002	0.050519892	0.056810714
pcaec321.pcaec361	-0.01273319	0.00663149	0.056922286
lysopcac170.pcaac281	-0.00687824	0.003584534	0.057081039
c8.pcaec404	0.03463581	0.018052469	0.057112584
pcaec364.c2	0.083753248	0.043679342	0.057261314
pcaec381.pcaac281	-0.006858806	0.003578349	0.057351686
glutamine.alanine	-0.05983575	0.031225951	0.05742009
lysopcac161.c51	-0.121799639	0.063574721	0.057467425
pcaec321.smc261	0.094775212	0.049472075	0.05748267
arginine.pcaac281	-0.006520505	0.003404332	0.057531091
pcaac323.pcaac386	0.034416913	0.017969157	0.057533948
glutamicacid.alanine	0.068974239	0.036018802	0.057583063
pcaac342.lysopcac180	0.074665967	0.039022296	0.057780626
methioninesulfoxide.pcaec300	-0.02021315	0.01056581	0.057825367
creatinine.spermidine	0.099691889	0.052116845	0.057853544
acetylornithoine.lysopcac160	0.016085932	0.008411879	0.057926692
prolinr.pcaac403	0.014841863	0.007765196	0.058050504
c182.c18	-0.055347002	0.028964518	0.058112224

histidine.pcaac386	0.028753387	0.015050315	0.05816039
lysopcac181.pcaac406	0.051579888	0.027007708	0.058246497
pcaac360.c0	-0.000874303	0.000457806	0.058253442
pcaac385.c18	-0.049564801	0.025953634	0.058256482
pcaac240.pcaac341	-0.014700431	0.007698168	0.058275081
c101.c181	0.024142249	0.012649298	0.058407424
c3.pcaec360	0.00565967	0.002965975	0.058457605
c3.c5	-0.050432568	0.026429524	0.058458693
c51dc.smohc161	0.024368894	0.012772837	0.058500669
pcaec322.pcaec361	-0.010962286	0.005749986	0.058680824
spermidine.c0	0.006468365	0.003393015	0.058695504
asymmetricdimethylarginine.spermic	0.097755113	0.051279924	0.058705068
glutamicacid.c2	-0.125194985	0.06567454	0.058706339
c101.tyrosine	0.113397964	0.059492933	0.058735061
pcaec360.smohc161	-0.118072079	0.06195235	0.058764042
c5.c141	-0.06814745	0.035758547	0.058775346
smohc161.pcaac341	-0.02324928	0.012203391	0.05885626
putrescine.pcaec360	0.006560265	0.003444851	0.058958695
pcaec422.pcaac323	-0.143416136	0.075334789	0.059044227
kynurenine.spermidine	0.116764725	0.061373519	0.059200959
lysine.pcaac281	-0.004165175	0.002190654	0.059357604
pcaec300.pcaac281	-0.007220244	0.00379858	0.059432052
h1.pcaac281	0.003994423	0.002101503	0.059435833
pcaec421.pcaec383	-0.274643112	0.14451441	0.059474351
kynurenine.c2	0.10065912	0.05296764	0.059483065
pcaac363.pcaac380	-0.084493269	0.044516793	0.059798554
pcaac344.c141	-0.063239747	0.033322585	0.059825735
histidine.c5	0.034775517	0.018324121	0.059826316
lysopcac280.pcaec343	0.143995344	0.075891449	0.059881404
pcaac401.pcaec444	0.237071311	0.124953618	0.059896189
pcaac365.pcaac323	-0.130629198	0.068860535	0.059931241
pcaac344.smohc221	0.076872179	0.040529861	0.059975313
pcaec386.pcaac323	-0.163955496	0.086444874	0.059979583
c5.pcaec300	0.023987126	0.012648753	0.060012296
pcaec401.pcaac343	0.047003646	0.024801988	0.060178523
pcaac341.c5	-0.013523233	0.007141016	0.060367836
methioninesulfoxide.acetylornithoine	-0.134150298	0.070855084	0.060426185
pcaac321.pcaec300	-0.02748619	0.014518516	0.060442735
pcaec364.lysopcac180	0.109069927	0.057611989	0.060442822
lysine.pcaac380	0.133730295	0.07067087	0.060561387
smc161.pcaec361	-0.013720443	0.007253381	0.060656059
pcaec444.methionine	0.125796259	0.066507826	0.060675179
pcaac363.pcaac365	-0.063407757	0.033530123	0.060726576
pcaec406.pcaac385	-0.087343592	0.046190586	0.060743958
pcaac360.pcaec321	0.057090967	0.030211991	0.060913787

pcaec423.pcaec364	0.014228697	0.007532096	0.060995286
pcaec300.spermidine	0.105972397	0.056113532	0.061068247
c14.pcaec405	-0.062715361	0.033212492	0.061099229
pcaac404.pcaec403	0.102246335	0.054192813	0.061314954
pcaac422.pcaac281	0.005224109	0.002769082	0.061332471
leucine.pcaac386	0.039350819	0.020858688	0.061338103
c4.pcaac323	0.130250893	0.069046224	0.061353436
pcaac281.pcaec445	0.104079347	0.055174001	0.061359742
pcaec404.pcaac281	-0.005151949	0.002731393	0.06138489
asparagine.c14	0.090567758	0.048026135	0.061438844
acetylornithoine.pcaec444	-0.126540201	0.067104896	0.061451631
pcaac383.pcaec422	0.028718948	0.015232098	0.061490297
lysopcac281.pcaac281	0.005015702	0.002661298	0.061591064
smohc221.c141oh	0.144409017	0.07665031	0.061684368
serine.pcaec300	-0.020358266	0.01080726	0.061717025
pcaec386.prolinr	0.041471366	0.02201533	0.061718234
pcaec424.pcaac281	-0.004587237	0.002436626	0.061872656
lysopcac180.pcaac381	-0.049009871	0.026033691	0.061881271
pcaec405.pcaac343	0.037744323	0.020049712	0.061883826
citruline.lysopcac181	0.060403324	0.032097228	0.061972874
pcaec385.pcaac402	0.035392234	0.018808039	0.061989986
isoleucine.pcaac323	0.140749392	0.074815333	0.062054406
c162.alanine	0.057706476	0.030675977	0.062072073
smohc241.pcaac404	0.016633581	0.00884341	0.062107789
pcaac406.pcaec404	-0.014365718	0.007640291	0.06219607
lysopcac160.smohc161	-0.02413623	0.012837367	0.062210296
lysine.pcaac344	0.113558295	0.060420001	0.062302696
c8.pcaac365	0.047225758	0.025129312	0.062326381
pcaec340.transhydroxyproline	-0.042627374	0.02268416	0.062345699
lysine.smohc221	0.105698625	0.056281977	0.062504092
pcaec385.pcaac384	-0.164089911	0.087391762	0.062556933
h1.pcaac365	0.079715228	0.042456186	0.062563536
pcaec403.pcaac281	-0.005741859	0.003058756	0.062618813
pcaac343.transhydroxyproline	0.029832341	0.015896224	0.062687066
pcaac380.c5	-0.028232394	0.015044724	0.06270491
pcaac361.smc261	0.115250686	0.06141755	0.062712341
pcaac360.arginine	-0.083380475	0.044457957	0.062853507
smohc141.pcaec341	0.180063905	0.096011439	0.062860193
pcaac426.pcaac381	-0.074007338	0.039468935	0.062910637
c18.c181	0.017955238	0.009575782	0.062912105
pcaec383.pcaac281	-0.004752907	0.002535474	0.062982005
pcaec362.pcaec322	-0.140728253	0.075073456	0.062985166
pcaac343.pcaec383	-0.112385035	0.059960824	0.063017506
pcaac364.pcaec342	-0.181592578	0.096896234	0.063047285
pcaec321.pcaac240	-0.0395959	0.021132361	0.063100624

pcaec363.prolinr	0.028923972	0.015445384	0.0632464
pcaec422.c141	-0.073507676	0.039258783	0.063284447
pcaac343.pcaec361	-0.021510555	0.011489257	0.063306171
lysopcac170.smohc221	-0.067225831	0.035910226	0.063331195
lysopcac181.pcaac281	0.003237988	0.001729669	0.063334755
pcaec322.pcaac404	0.010497951	0.005607913	0.063340047
smc161.pcaec342	-0.200751066	0.10724183	0.063345856
pcaec444.lysopcac181	0.089866775	0.048013703	0.063381731
pcaac426.smohc222	0.014272647	0.007627621	0.063453023
serotonin.transhydroxyproline	-0.050411668	0.026945072	0.063491337
pcaec342.pcaac402	0.042156257	0.022546526	0.063653507
lysopcac280.c141	-0.03716741	0.019885687	0.0637504
pcaec382.pcaec444	0.329476034	0.176299934	0.063780578
pcaec300.c141oh	0.16117777	0.086260126	0.063826822
putrescine.pcaec404	0.026028339	0.013936864	0.06395587
lysopcac203.pcaec404	0.017723492	0.00949076	0.063975875
pcaec385.c141oh	0.183434331	0.098229149	0.063980743
pcaac364.histidine	0.076860586	0.041162018	0.064000818
lysopcac280.pcaac281	-0.004103976	0.002198123	0.064033544
pcaac383.pcaec322	-0.094061191	0.050420227	0.064243743
pcaac381.lysopcac160	0.058326922	0.031270683	0.064288585
pcaec380.c5	-0.035698309	0.019140565	0.064312112
histidine.pcaac322	0.086218913	0.046238968	0.064371831
pcaac341.c0	-0.001003454	0.000538183	0.064388354
c51.c181	0.019426053	0.010419174	0.064397992
c14.phenylalanine	-0.050773999	0.027234163	0.06441253
lysopcac180.pcaec300	-0.018699601	0.010030896	0.064433597
pcaac322.c5	-0.020555582	0.01102661	0.064436562
asymmetricdimethylarginine.pcaac42	0.097808271	0.052471056	0.064455969
phenylalanine.tyrosine	-0.119653288	0.064266574	0.064769871
serotonin.pcaac365	0.046531308	0.024994689	0.064795422
pcaac366.pcaec444	0.144043378	0.077402966	0.064894025
pcaac321.c51dc	-0.15971437	0.085886642	0.065087622
pcaac421.pcaac402	0.10148593	0.054586121	0.065145506
c0.pcaec300	0.010803952	0.005812229	0.065196637
pcaec343.prolinr	0.028639737	0.015408176	0.065210171
pcaac383.pcaec403	0.077374631	0.04163241	0.065241193
smohc241.smohc221	-0.033446841	0.01799939	0.065283836
creatinine.asparticacid	0.074770887	0.040252749	0.065381954
pcaac300.glycine	0.060364272	0.032505577	0.065452326
serine.pcaac402	-0.044498293	0.023975852	0.065607303
c16.lysopcac170	0.076517469	0.041250193	0.065751373
pcaac321.c51	-0.194276467	0.104795931	0.065910476
pcaec385.pcaac406	0.0770285	0.041554549	0.065936877
pcaac422.lysopcac170	-0.041143729	0.022196427	0.065944436

putrescine.pcaac341	0.01605497	0.008661665	0.065952086
pcaac342.pcaac240	-0.023390891	0.012619911	0.065962967
pcaec403.spermidine	-0.092359015	0.049853836	0.066092131
pcaac386.smohc222	0.010930824	0.005900681	0.06611059
pcaac362.lysopcac181	0.035323554	0.019071614	0.066156089
c141oh.c102	0.083111961	0.044877107	0.066179679
smc202.pcaec424	0.073409097	0.039652794	0.066279981
pcaac342.histidine	0.076925593	0.041557502	0.066313725
pcaec383.smohc161	-0.035541804	0.019201471	0.066323885
c0.smc261	-0.061426743	0.033197468	0.066418034
c3.pcaec404	-0.016526592	0.008932271	0.066437268
c51dc.pcaec361	-0.019353583	0.010464928	0.066558637
alanine.pcaec405	0.056997122	0.030828075	0.066632042
pcaec365.pcaac362	-0.050042057	0.027066983	0.066638984
pcaac420.pcaec444	0.190562451	0.103082766	0.066666254
pcaec363.pcaec424	-0.067415977	0.036471638	0.066693318
pcaac402.pcaec382	-0.06042807	0.032694924	0.066723781
creatinine.pcaac380	0.080092456	0.043341333	0.066766572
smohc141.smc261	0.055686578	0.03014449	0.066857568
c12.pcaac386	0.026368535	0.014274729	0.06687275
pcaec446.pcaec360	-0.015761196	0.008535886	0.066982954
pcaac403.pcaac281	-0.011278783	0.006110447	0.067076962
c0.lysopcac182	0.116744482	0.063256795	0.067114294
pcaac341.pcaac425	-0.110124677	0.059686484	0.067189252
pcaac381.pcaac403	-0.014262054	0.007729905	0.0671897
creatinine.lysopcac160	0.017064287	0.009250572	0.067244702
citruline.pcaac281	-0.004335545	0.002351652	0.067399781
smohc222.pcaec382	-0.033366445	0.018108552	0.067552162
c4.pcaac404	0.011830906	0.006422788	0.067634453
lysopcac160.lysopcac181	0.104480036	0.05672763	0.067669248
c51.pcaac402	-0.043562758	0.023657978	0.067732319
pcaac322.smohc161	0.019289868	0.010477248	0.067767154
pcaac342.pcaec364	-0.022187692	0.012058146	0.06792384
creatinine.smohc241	0.119729197	0.065107423	0.068087988
pcaac343.phenylalanine	-0.093774719	0.051026024	0.068260846
pcaac281.smc261	0.083596313	0.045497256	0.068318702
c0.pcaac406	0.045811811	0.024935304	0.068343275
pcaec406.spermidine	0.096977348	0.052790624	0.068374252
smohc221.lysopcac181	0.05182429	0.028224872	0.068507646
c162.c141	-0.05297384	0.028858001	0.068574455
lysopcac160.pcaec300	-0.013956992	0.007606841	0.068705303
ornithine.pcaec340	0.055194226	0.030098791	0.068858468
pcaec446.pcaac365	0.07729405	0.042150397	0.068858603
methionine.pcaec361	0.015779307	0.008605337	0.068873959
smc202.tyrosine	-0.117257071	0.063967136	0.068961039

pcaec445.pcaec300	0.009691396	0.005288389	0.069036354
pcaac386.pcaac381	-0.039389257	0.021499742	0.069111198
pcaec445.pcaec444	-0.2119112	0.115674459	0.069128969
smc241.smohc221	-0.127093101	0.069395296	0.069207732
pcaac343.c5	-0.01946295	0.010634133	0.069388462
pcaac384.smohc221	0.047943067	0.026195586	0.069394108
lysopcac182.c16	-0.078639469	0.042975431	0.069443153
asparticacid.pcaec405	0.180313206	0.098539459	0.069445036
pcaec425.arginine	-0.045531578	0.024883669	0.069457135
c4.pcaec300	0.010797598	0.005901839	0.069494236
putrescine.spermidine	0.095312176	0.052102159	0.069524139
pcaac420.lysopcac203	-0.042392139	0.023182823	0.069634389
serotonin.tyrosine	-0.103365224	0.056542938	0.069712489
pcaec385.prolinr	0.030536857	0.016718703	0.069950627
leucine.pcaec443	-0.071543316	0.039183452	0.070049615
alanine.pcaec404	-0.040472451	0.022170107	0.070097209
leucine.smohc161	-0.019354962	0.010603565	0.0701299
pcaac405.pcaec340	0.123902285	0.067886567	0.070158587
creatinine.pcaec425	0.063730767	0.034925745	0.070217293
c182.pcaac281	0.007555989	0.004142007	0.070295811
pcaec380.smohc222	0.020957781	0.011492437	0.070389927
pcaec402.smohc221	-0.083246047	0.04564987	0.070395727
c181.c102	0.065257503	0.035806534	0.070559395
pcaec363.pcaac404	0.006539044	0.003588641	0.07061312
c4.c14	-0.068058286	0.037356746	0.070659401
pcaec362.pcaac380	-0.092243768	0.050634048	0.07067067
c18.pcaec443	0.082689008	0.045394573	0.070703048
lysopcac182.pcaec444	-0.115574646	0.0634734	0.070813971
pcaec421.pcaec422	0.192980052	0.10600277	0.070862465
c101.pcaec444	-0.138598263	0.076149286	0.070928727
putrescine.pcaec444	-0.133193049	0.073236179	0.071144643
lysine.alanine	0.05159283	0.02837068	0.071167971
pcaac421.c141oh	0.138347269	0.076090538	0.071219225
pcaec446.pcaac240	-0.025884997	0.014241253	0.071309344
pcaec321.pcaec405	-0.099949485	0.054989984	0.07131125
methionine.pcaac365	-0.050966022	0.028052065	0.071427894
pcaec342.prolinr	0.028787062	0.015846584	0.071462843
lysopcac182.smohc221	-0.051338932	0.02826147	0.071469033
pcaac321.pcaac402	-0.023437299	0.012903025	0.071492268
lysopcac170.pcaac402	0.082701948	0.045531495	0.071500148
pcaac424.pcaec364	0.009967349	0.005488309	0.071540601
c101.c141	0.047723443	0.026288124	0.071649631
c2.pcaac402	0.050774192	0.027970867	0.07167228
pcaec300.c51dc	-0.153023497	0.084305919	0.071696198
pcaac362.smc161	-0.153199213	0.084404912	0.071703457

pcaec405.arginine	-0.060340112	0.033247237	0.071728172
pcaec360.lysopcac180	0.062557727	0.034480637	0.071821805
pcaec382.pcaac281	-0.006099963	0.003362197	0.071822888
asymmetricdimethylarginine.transhy	0.029731698	0.016387767	0.071825521
pcaec406.smohc161	-0.023998598	0.013228877	0.071849462
glutamine.pcaec363	0.036135746	0.019919719	0.071855335
pcaac323.spermidine	0.092424316	0.050949278	0.071858968
serine.c0	0.001045057	0.000576127	0.071875998
c181.pcaac406	0.058101872	0.032031171	0.071878958
c2.pcaec444	-0.097452433	0.053725773	0.071883671
pcaec446.prolinr	0.030984853	0.017082218	0.071886794
serine.lysopcac181	0.046773033	0.025788511	0.071910033
c181.pcaac402	0.030565845	0.016853229	0.071920375
lysopcac203.pcaec300	-0.007683411	0.004237134	0.071966603
c8.c0	0.00133824	0.000738123	0.072016059
c10.pcaec443	0.093316455	0.051482451	0.072085007
c141oh.pcaac365	0.04167702	0.022998251	0.072147978
lysopcac204.c141oh	0.199902655	0.110312626	0.072153491
pcaec382.pcaec403	0.326429626	0.18021075	0.072273129
lysine.lysopcac170	-0.041605197	0.022980715	0.07241909
lysopcac182.pcaec340	0.070999681	0.03922988	0.072513079
ornithine.pcaec404	-0.059965754	0.033133434	0.072514747
pcaec362.spermidine	0.097037195	0.053622406	0.072543953
pcaac421.pcaec443	0.068836898	0.038047055	0.072603589
pcaec406.pcaec361	-0.017451895	0.009649319	0.072704018
pcaec343.arginine	-0.071511324	0.039539932	0.072708597
pcaec383.pcaec405	0.225195288	0.12456763	0.072828976
lysopcac280.pcaec322	0.077658235	0.04295699	0.072829249
pcaec364.spermidine	0.09239357	0.05112527	0.072925438
pcaac385.pcaec405	-0.080245328	0.044405936	0.072943312
histidine.pcaac281	-0.005150447	0.002850514	0.072980476
pcaec361.pcaac281	-0.004228743	0.002343893	0.073404129
smc161.pcaac404	0.004985133	0.002763193	0.073409316
pcaac383.pcaac404	0.005629853	0.00312056	0.073410069
c51dc.pcaec422	-0.051808571	0.02872238	0.073464594
c102.pcaac403	-0.006617386	0.003669377	0.073521639
pcaec342.pcaac384	-0.071977327	0.039925172	0.073616804
serine.pcaac344	0.084580552	0.046941279	0.073769891
lysopcac182.pcaec363	0.026339672	0.01461873	0.073779658
c10.pcaec444	-0.156206844	0.086705199	0.073809728
asymmetricdimethylarginine.pcaec4(	-0.008549467	0.004745565	0.07381223
pcaac360.pcaec384	-0.160444275	0.089066178	0.073838304
c18.pcaec444	-0.096614489	0.053633148	0.073839906
arginine.pcaec343	0.108530584	0.060262624	0.073908805
pcaec402.pcaec300	-0.020375395	0.011315767	0.073962754

pcaac363.pcaec360	0.006155706	0.003419898	0.074066237
smohc141.pcaec342	-0.204218338	0.113468677	0.074096471
pcaec322.pcaec404	-0.009270453	0.005151826	0.074148471
pcaec443.pcaac403	0.02856975	0.015877017	0.074149925
c16.pcaac405	0.071442141	0.039708469	0.074193534
lysopcac203.lysopcac161	0.148924304	0.082856446	0.074478394
pcaac364.pcaec361	-0.033573231	0.018683917	0.074553707
lysopcac204.c181	-0.025076472	0.0139573	0.074593456
methionine.c0	0.000759046	0.000422488	0.074601054
pcaac385.pcaec364	-0.037861963	0.021074686	0.074608656
pcaec401.smohc161	-0.018665558	0.010406148	0.075066028
citruline.smc261	0.045205315	0.025220098	0.075270549
leucine.pcaac380	0.11396754	0.063627287	0.075473194
pcaac385.prolinr	0.02814242	0.01571328	0.075501917
pcaac385.pcaec361	-0.014511091	0.008103183	0.075535282
c101.pcaac365	0.030470798	0.017015388	0.075536919
lysopcac170.pcaac405	-0.106622462	0.059542736	0.075551586
smc161.c141	-0.029364351	0.016403295	0.075638516
pcaac343.lysopcac161	0.116128339	0.064889933	0.075724058
pcaec420.smohc222	0.004803949	0.002685085	0.075804368
c0.pcaac380	0.116772586	0.065310753	0.075993951
asparticacid.lysopcac160	-0.033355563	0.018656111	0.075999994
pcaac320.smc261	0.095849136	0.0536493	0.076215819
pcaac383.pcaec383	-0.104131882	0.058289589	0.076236834
lysopcac161.pcaac281	0.004125766	0.002310765	0.076399998
lysopcac280.pcaec342	0.109571985	0.061382885	0.07646474
lysopcac161.lysine	0.146008567	0.081795863	0.076468247
pcaec385.transhydroxyproline	-0.071490204	0.040051669	0.076482375
pcaec423.c2	0.081038683	0.045403121	0.076495269
pcaac404.c51dc	-0.131556339	0.073707913	0.076501305
pcaac426.lysopcac181	0.046149074	0.025858841	0.076530603
lysopcac180.pcaac281	0.004287688	0.002402767	0.076559053
pcaec343.c2	0.063525287	0.035601935	0.076584845
c3.pcaec300	0.007047093	0.003951181	0.076712253
phenylalanine.smohc221	0.072349187	0.040569733	0.07674689
smc241.arginine	-0.064668367	0.036273522	0.076833736
smohc161.smc181	0.0520953	0.029227927	0.076902028
lysopcac204.pcaec340	0.106467291	0.059756052	0.077013786
lysopcac180.spermidine	-0.096537054	0.054191528	0.077061997
c181.pcaec300	0.010591807	0.005945806	0.077064233
lysopcac170.pcaec382	-0.012529076	0.00703371	0.077080861
pcaec363.pcaec364	-0.029838886	0.016752808	0.077107532
c3.lysopcac182	0.081842139	0.045952501	0.077125817
lysine.glycine	0.065731889	0.036915881	0.077196476
pcaec321.pcaac404	0.008743049	0.004913752	0.077407569

pcaac281.pcaac404	0.006554956	0.003684529	0.077449346
lysopcac181.pcaec300	-0.023341273	0.013142642	0.077953546
c4.c2	-0.090382314	0.050891188	0.077954224
pcaec404.pcaec444	0.216149622	0.121718197	0.077982601
lysopcac180.pcaac386	-0.032030841	0.01803981	0.078025003
kynurenine.pcaac385	0.041238656	0.023230941	0.078091877
pcaac322.pcaac365	-0.062915188	0.035462304	0.078260869
smc261.pcaec444	0.156024538	0.087975503	0.078367735
smc160.c141	-0.03609598	0.020358107	0.078442255
pcaac341.pcaec322	-0.081702586	0.046081768	0.078452303
pcaec365.pcaac365	0.058137295	0.03280328	0.078567282
alphaaminoadipicacid.pcaec300	0.009366298	0.005286723	0.078673493
c16.pcaac281	-0.003887894	0.002195265	0.078778389
leucine.pcaac402	-0.076295923	0.043087175	0.07882887
c3.pcaac344	0.071114501	0.04016304	0.078843671
c10.pcaec300	-0.027070642	0.015288753	0.078847154
histidine.pcaec364	-0.030344785	0.017143728	0.078947843
pcaec420.pcaec422	0.06309246	0.035652865	0.079013172
c51.pcaac404	0.003893274	0.002200555	0.079081564
lysopcac182.smc181	-0.032306927	0.018264021	0.079138613
c181.smohc241	0.115244992	0.065151828	0.079141201
leucine.spermidine	-0.094627338	0.053499538	0.079160916
smc160.pcaec361	-0.018105457	0.010237795	0.079204321
c4.smohc221	0.042885391	0.024266737	0.079412898
smohc141.pcaec404	-0.021473285	0.012154116	0.079496997
c14.pcaac402	0.026492979	0.015000519	0.079600136
lysopcac161.lysopcac180	0.077255118	0.043743067	0.079604582
smc161.pcaec363	-0.063800592	0.036141733	0.079743244
pcaac361.pcaac403	0.009405834	0.005328226	0.079743973
c102.glycine	-0.036302321	0.020567356	0.079784263
smc260.pcaac281	0.004375625	0.00248011	0.079912466
pcaec342.pcaec361	-0.012824402	0.007270504	0.07997876
pcaac300.pcaec300	-0.01497016	0.008489004	0.080049452
pcaac384.pcaec300	0.012579568	0.00713424	0.080084997
asparagine.pcaec446	0.121321106	0.068806433	0.080092336
pcaec380.c0	-0.001910304	0.001083707	0.080172609
alphaaminoadipicacid.pcaac343	0.04412188	0.025032854	0.08020489
smc241.c2	0.067009035	0.03802656	0.080271868
pcaec445.putrescine	-0.110782015	0.062881771	0.080341974
c10.pcaec404	0.033815457	0.019197788	0.080397367
pcaec340.glycine	0.076711588	0.04356074	0.080465132
pcaac383.c5	-0.013297912	0.007551738	0.080485236
pcaec402.c141	-0.097163537	0.055179557	0.080493129
kynurenine.pcaac240	-0.025015904	0.014209037	0.080543845
pcaec365.prolinr	0.03673062	0.020869272	0.08063392

pcaec385.pcaec321	-0.085422803	0.048541573	0.080676014
alphaaminoadipicacid.lysopcac181	-0.076500021	0.043476413	0.080711997
creatinine.pcaac403	-0.010992419	0.006248063	0.080753405
pcaac361.leucine	0.043627969	0.024809484	0.080892008
pcaec383.lysopcac161	0.090602012	0.051539049	0.080992847
arginine.lysopcac160	-0.019074048	0.010853431	0.081079755
pcaec365.c2	0.069547435	0.039575455	0.081094037
c141.pcaec300	-0.017250866	0.009816563	0.081096642
c162.pcaac403	-0.012612501	0.007178948	0.081173525
pcaec342.pcaac404	0.006969887	0.003967304	0.081180529
pcaec300.pcaac404	0.006120959	0.003484563	0.081221473
lysopcac160.pcaec404	0.027660934	0.015747216	0.081227095
pcaec384.pcaec300	0.010954092	0.006239127	0.081373022
pcaec362.prolinr	0.033968929	0.019349397	0.081399453
pcaec365.pcaec300	0.00881011	0.005019347	0.081455149
pcaac421.arginine	-0.043349545	0.024714421	0.081663486
pcaec406.pcaac402	0.044228539	0.025220598	0.081723874
pcaec402.pcaec343	0.076086059	0.043392738	0.081765018
pcaec424.pcaac403	-0.029130607	0.016614351	0.081780223
c51.pcaac406	0.062401234	0.035621106	0.082044977
pcaac383.spermidine	0.093297526	0.053260398	0.082058954
c51dc.smc181	-0.028222995	0.016111896	0.08206542
smc160.pcaac404	0.006360178	0.003631345	0.082103521
citruline.pcaac343	-0.036096916	0.020615983	0.082198136
pcaec300.lysopcac180	0.062663759	0.035790004	0.082206044
pcaec425.smohc222	0.008915957	0.005093089	0.082253487
methioninesulfoxide.smohc221	-0.077191901	0.044098484	0.082280419
smc240.smohc221	-0.054939154	0.031388969	0.082310535
prolinr.pcaec445	0.049050805	0.028042361	0.082501429
pcaac405.smohc221	0.033657831	0.019243174	0.082516901
lysopcac170.c0	0.005903419	0.003375484	0.082546193
lysopcac281.pcaec343	0.11644843	0.066598449	0.082614691
c102.pcaec361	-0.021482216	0.012287824	0.082660466
c18.c141oh	0.123211533	0.070483138	0.08268701
c3.smohc161	-0.019217538	0.010999073	0.082844137
pcaac341.pcaac402	-0.031907043	0.018264469	0.082887667
pcaac363.pcaec404	-0.01445689	0.008275837	0.082899266
lysine.pcaac406	0.053897811	0.030858025	0.082941277
pcaac362.pcaec404	-0.017938443	0.010272364	0.083003421
pcaec322.prolinr	0.02005273	0.011487957	0.083132217
pcaec401.transhydroxyproline	0.037435069	0.021447628	0.083154203
pcaec321.pcaec444	0.217138053	0.124409574	0.083166303
pcaec385.smc161	-0.081076311	0.04646659	0.083256834
smohc141.pcaec382	-0.02505395	0.0143591	0.083259787
pcaac281.pcaac386	0.02097072	0.012020883	0.083310359

pcaac323.lysopcac181	0.051089676	0.029292632	0.083382189
pcaac343.smohc221	0.032429182	0.018596528	0.083431829
c182.glycine	-0.045963887	0.0263691	0.083560329
arginine.pcaac380	0.087764902	0.050376714	0.083722538
pcaec365.pcaac402	0.048050182	0.027582245	0.083740576
c51.pcaec300	0.023032525	0.013222714	0.083771929
smc181.pcaec361	-0.012691668	0.007286155	0.083772379
pcaec383.lysopcac170	-0.047442302	0.027241349	0.083830977
pcaac344.pcaac384	-0.240935426	0.138387786	0.08392563
pcaac361.c5	-0.01355035	0.007784188	0.08397198
c5.smohc221	0.088829733	0.051055357	0.084127383
pcaac401.pcaec382	-0.084450424	0.048540097	0.084138578
pcaec385.pcaec405	-0.083566156	0.048038171	0.084179003
c51.pcaec364	0.029239092	0.016812052	0.084250234
lysine.smohc161	0.015053391	0.008656223	0.08427658
lysopcac180.pcaec381	0.072449544	0.041662301	0.084286109
c51dc.lysopcac170	0.013118733	0.007544703	0.084316286
c16.pcaac402	0.043331087	0.024928574	0.084420681
pcaac362.histidine	0.073514744	0.042302254	0.084485224
pcaec362.pcaec404	-0.016434402	0.009461899	0.084651899
methionine.pcaec322	-0.053627085	0.030879079	0.084691411
pcaec386.c14	-0.063465492	0.036549346	0.084735238
pcaac404.lysopcac160	-0.032259963	0.018580912	0.084778633
pcaec444.lysopcac203	-0.066431939	0.038294463	0.085031243
creatinine.pcaec382	-0.009949585	0.005738593	0.085202837
pcaac365.pcaec364	0.039136543	0.022578492	0.085282492
pcaac380.arginine	-0.055044929	0.031757271	0.085292019
kynurenine.pcaec382	-0.012523762	0.007225559	0.085299692
smc261.pcaac384	-0.177936917	0.102663044	0.085307827
pcaac385.smohc221	0.023568367	0.013601584	0.085387488
pcaac362.leucine	0.032422967	0.018712893	0.085407564
pcaac343.c51dc	-0.151817813	0.08762572	0.085422287
pcaec301.pcaec404	0.022161676	0.012794816	0.08550959
pcaac420.c141oh	0.158011213	0.091281101	0.085696047
c102.smohc221	0.041362043	0.023897294	0.085734411
pcaec384.c51dc	-0.155772488	0.090008503	0.085767268
smc240.lysopcac160	0.047111197	0.027226541	0.085821191
ornithine.spermidine	-0.093595511	0.054129398	0.086042553
pcaec424.pcaac402	0.091404677	0.05287873	0.086138678
arginine.pcaec382	0.020366914	0.011785514	0.086217904
pcaac323.c141	-0.037274278	0.021587398	0.086481299
c51dc.prolinr	0.03701968	0.021441246	0.086500167
pcaac362.pcaac404	0.004597333	0.002662905	0.086523363
pcaec322.smohc222	0.01027353	0.005950827	0.086529022
pcaec383.pcaac402	-0.078006427	0.045185538	0.086537243

c12.pcaec300	-0.016007352	0.009274628	0.086614676
pcaac361.lysopcac280	0.014442002	0.00836875	0.086654992
pcaac323.pcaec422	0.038207372	0.022147316	0.086756017
pcaac381.pcaec300	-0.019178022	0.011118388	0.086802045
pcaac300.spermidine	0.092113592	0.053403783	0.086809432
pcaec445.pcaec341	0.10562647	0.06124841	0.086862415
putrescine.c14	0.075807931	0.04395994	0.08687707
pcaec362.lysopcac161	0.097384374	0.056473884	0.08688852
pcaec385.pcaec300	0.006007859	0.003484427	0.086926768
pcaec425.pcaec405	-0.147543925	0.085573069	0.086929776
pcaac364.smc181	0.037355794	0.021667619	0.086956696
pcaac364.c2	0.089764769	0.052077422	0.087021662
c8.pcaec300	-0.023625509	0.013706647	0.087026333
smc261.pcaac281	0.002137626	0.001240713	0.087162665
pcaec360.pcaac240	-0.03110009	0.018051177	0.087165817
smc241.c181	0.015220949	0.008834871	0.087176306
arginine.c5	0.024029541	0.013950578	0.087239876
c142.smc181	-0.028383794	0.016479005	0.08724981
pcaec381.asparticacid	0.123030751	0.071435084	0.087276722
pcaec403.pcaac402	-0.068608902	0.039847822	0.087367685
phenylalanine.pcaec445	0.102551804	0.059564025	0.087379665
smc240.pcaac380	-0.114340299	0.06644984	0.08756278
pcaec444.smohc221	-0.097526162	0.056679305	0.087569108
pcaac383.pcaec445	0.075455438	0.043881487	0.087776662
kynurenine.pcaec361	-0.009732819	0.005661218	0.087834793
smohc222.pcaec342	-0.178826442	0.104020294	0.087845725
lysopcac160.smohc221	-0.052490532	0.030539361	0.087912699
pcaec406.c14	-0.065036446	0.037843355	0.087951665
c18.c2	0.095864286	0.055790794	0.088004384
pcaac380.pcaac406	0.088955048	0.051776472	0.088045019
smohc222.pcaec300	0.020459362	0.011908701	0.088052595
pcaec385.arginine	-0.071478307	0.041606668	0.088064439
alphaaminoadipicacid.pcaec404	-0.019645496	0.011436192	0.088085997
c182.pcaac403	-0.008030917	0.004676589	0.088191453
pcaec362.pcaec342	-0.19286499	0.112319464	0.08821866
alanine.pcaac365	-0.095480085	0.055610528	0.08824953
pcaec364.pcaec300	0.012269634	0.007146447	0.088259906
c16.pcaec404	0.050676722	0.029523747	0.0883356
lysopcac180.pcaec403	0.08899481	0.051849308	0.088346708
pcaac320.kynurenine	-0.020785098	0.012109856	0.088352956
pcaac320.c2	0.065086498	0.037933331	0.088456818
asparagine.pcaec322	-0.058411509	0.034054228	0.088560272
pcaac360.pcaec443	0.098799102	0.057601576	0.088566631
kynurenine.prolinr	0.024972698	0.014565278	0.088691536
pcaac421.lysopcac203	-0.037401325	0.021823273	0.088822075

pcaac366.pcaec364	0.015494996	0.009042442	0.088866703
pcaec386.pcaec300	0.008860058	0.005170642	0.088876615
c18.pcaec405	-0.073264403	0.042756664	0.088878735
asymmetricdimethylarginine.pcaac34	0.003669103	0.002141315	0.088885947
pcaac426.c141	-0.056343441	0.03288261	0.088887548
alanine.pcaac322	0.085554071	0.049931288	0.088894217
kynurenine.pcaec360	0.006087937	0.003553513	0.088934785
pcaac424.smohc222	-0.03792215	0.022143032	0.089048549
pcaac384.spermidine	0.089359739	0.052178374	0.089051848
pcaac404.lysopcac180	0.090691707	0.05299083	0.089259145
pcaec386.pcaac404	0.021372947	0.012492136	0.089360628
pcaec361.c141	-0.048393623	0.028289946	0.089412939
lysine.c2	-0.07640632	0.04467257	0.089462337
serotonin.pcaac386	0.037349286	0.021838824	0.089488099
pcaac424.pcaac281	-0.007070074	0.004134242	0.089506318
lysopcac180.pcaac402	0.094154064	0.055058315	0.089515045
pcaec341.pcaac384	-0.0629068	0.036793295	0.089578763
c101.prolinr	0.052177724	0.030530799	0.089711728
lysopcac161.smohc221	0.129322523	0.07567359	0.089724251
c16.c18	0.14954117	0.087506529	0.08973121
h1.pcaec401	0.099315258	0.058136098	0.089841081
pcaac380.pcaec364	0.024926293	0.014593185	0.0898868
prolinr.phenylalanine	-0.086356519	0.050578864	0.090019725
lysopcac203.pcaec383	-0.159955341	0.093687722	0.09002704
pcaac361.smohc222	0.014993958	0.00878317	0.090064422
pcaac361.pcaac281	0.004712791	0.002760841	0.090085119
pcaec404.spermidine	-0.085979095	0.050371804	0.090108143
c162.pcaec360	-0.013597621	0.007966389	0.090111015
asymmetricdimethylarginine.c181	0.020220899	0.011853422	0.090290447
pcaac403.lysopcac170	-0.106932126	0.062685508	0.090301939
pcaec362.pcaec384	-0.100651532	0.05900376	0.090302199
pcaac426.pcaac281	0.004857802	0.002847899	0.090320906
pcaac386.pcaec405	-0.101710393	0.059630992	0.090336973
c181.pcaac281	0.013585197	0.007965239	0.090356175
pcaac320.smohc222	0.005356116	0.00314044	0.090361777
pcaac363.prolinr	0.029324877	0.017198115	0.09043823
pcaac403.smohc221	-0.090581396	0.053138004	0.090527514
c0.pcaac405	0.046718986	0.027413039	0.090599185
c5.pcaec404	-0.042332208	0.024841523	0.090631173
pcaac383.lysopcac180	0.083729014	0.04914739	0.090717337
pcaec406.pcaec404	-0.013826885	0.00811848	0.090809946
pcaac405.c141	-0.056091758	0.032936437	0.090829943
pcaac281.pcaec361	-0.015763368	0.009260854	0.0909952
smc202.pcaac281	0.00201578	0.001184436	0.091044273
glutamicacid.pcaac380	0.107485031	0.063164882	0.091087808

isoleucine.pcaec445	0.050807024	0.029860104	0.091117179
pcaec446.transhydroxyproline	-0.059082219	0.034724704	0.091127652
pcaac281.pcaec404	-0.020860739	0.012266522	0.091282516
pcaac323.smohc221	0.010228447	0.006015326	0.09132513
pcaec446.pcaec300	0.006471987	0.003806215	0.091329715
c181.glycine	-0.047795695	0.028109549	0.091336648
asparagine.pcaac405	-0.167570697	0.09856628	0.091384769
alanine.pcaac425	-0.092584072	0.054477583	0.091496477
c12.pcaec380	-0.057401088	0.033785119	0.091587937
alanine.pcaec360	0.007209549	0.004243857	0.091622881
asymmetricdimethylarginine.valine	0.078231894	0.046052647	0.091636302
lysopcac140.c51dc	-0.166175601	0.097874415	0.091807299
pcaac386.smohc161	-0.021238428	0.012512466	0.091895107
c3.smohc221	0.027790623	0.016374364	0.091928888
pcaec342.pcaec422	0.049682631	0.029282269	0.092028139
methioninesulfoxide.pcaac281	-0.005806716	0.003424695	0.092244054
c162.smohc222	-0.006833771	0.004033917	0.092522656
pcaec445.pcaac365	0.06921233	0.040865575	0.092602617
smc161.c0	-0.001652467	0.000975842	0.092656758
pcaac406.c18	-0.050488387	0.029818869	0.092695958
c0.smohc221	0.039057316	0.023070537	0.092737349
ornithine.methionine	-0.048385511	0.028584574	0.092782799
phenylalanine.pcaac402	-0.049588553	0.029296094	0.092791652
pcaec424.c2	0.090713491	0.05359962	0.092837461
lysopcac280.pcaec300	-0.008693578	0.005137994	0.092915797
acetylornithoine.transhydroxyproline	-0.01397843	0.008261929	0.092936658
smc202.smohc221	0.017025616	0.010068772	0.093123442
smc261.pcaac322	0.0839801	0.049685417	0.093256857
smc261.smc160	-0.119292926	0.07058014	0.093268252
pcaec364.prolinr	0.041279552	0.024423402	0.09327075
smohc222.lysopcac181	0.061721995	0.036520404	0.093288993
kynurenine.pcaec404	-0.012039746	0.007124996	0.093342503
pcaac364.pcaec364	-0.026393187	0.015619471	0.093347855
c16.citruline	0.054724318	0.032387131	0.093361035
pcaac420.arginine	-0.043341142	0.025655548	0.093427363
smc202.c51	-0.131588653	0.077905304	0.093477886
c51dc.pcaec300	0.007820269	0.004629893	0.093478377
c5.pcaec361	-0.035283438	0.020901892	0.093676811
pcaec360.pcaac403	-0.024856282	0.014725757	0.09369682
pcaac383.arginine	0.064329811	0.038115375	0.093731583
pcaec361.pcaac402	-0.094259306	0.05585875	0.093790734
c182.pcaec444	-0.095001455	0.056327362	0.093957096
smohc161.smc261	0.067939551	0.040283801	0.093971042
c101.pcaac403	-0.004722048	0.002800534	0.094047989
c18.pcaac402	0.030415762	0.018039631	0.094061833

pcaec446.arginine	-0.047443495	0.028139735	0.09407263
c162.smc202	-0.056784623	0.033701876	0.094282631
pcaac386.pcaec342	-0.132465528	0.078619677	0.094286418
pcaac425.lysopcac180	0.094137377	0.055875055	0.09430705
pcaec406.pcaec300	0.008381888	0.004976792	0.094420972
pcaac364.pcaec300	0.024601766	0.014616616	0.094626081
pcaec381.spermidine	-0.085095603	0.050577554	0.094754052
lysopcac170.c141oh	0.119084605	0.070779396	0.094754453
glycine.lysopcac203	-0.048392935	0.028769747	0.094831854
pcaac381.pcaec444	0.176767799	0.105101785	0.094871644
smc181.pcaec300	0.005554828	0.003303782	0.094972462
phenylalanine.smohc222	-0.007481728	0.004450314	0.095008558
acetylornithoine.arginine	0.026296437	0.015644758	0.095071273
glutamicacid.pcaec342	0.150675031	0.089668072	0.095165209
pcaac320.pcaac365	-0.082547978	0.049130067	0.095198671
putrescine.lysopcac160	0.025602891	0.015239501	0.095229453
pcaec381.pcaec382	-0.043278208	0.025764509	0.095282926
asparticacid.pcaac403	0.04226337	0.025164304	0.095334401
pcaac342.pcaac365	-0.066909222	0.039840622	0.095348993
pcaec405.pcaec404	-0.045308354	0.026982002	0.095391075
pcaac363.pcaac404	0.005237262	0.003119319	0.095436061
pcaec362.pcaac384	-0.049400104	0.029442474	0.095655947
methionine.glycine	0.104975115	0.062589913	0.095785706
pcaec384.smohc222	0.009055228	0.005399322	0.09580222
c51dc.spermidine	0.092805991	0.055348549	0.095870648
pcaac364.smohc221	0.075111021	0.044796648	0.095879449
serotonin.lysopcac181	-0.059432102	0.035456147	0.095976824
smohc141.pcaec300	0.012591654	0.00751237	0.095994806
pcaac385.histidine	0.073362699	0.043789301	0.096145252
pcaac422.transhydroxyproline	0.049680551	0.029665001	0.096270569
histidine.c162	-0.139353034	0.083232926	0.096362214
methionine.pcaac360	-0.105643757	0.063120567	0.096474822
pcaec420.pcaac323	-0.148836343	0.088972076	0.096640209
isoleucine.c162	-0.119734164	0.071579156	0.096658268
pcaac380.c141	-0.040477143	0.024200704	0.09669603
smc202.pcaec445	0.041606271	0.024889787	0.09688183
pcaec403.pcaec382	0.081910941	0.049001526	0.096885856
pcaec384.pcaac344	0.103009633	0.061623972	0.096889022
pcaac342.pcaec404	-0.020006311	0.01197078	0.096952667
pcaec364.pcaac402	0.035758081	0.021399955	0.09701603
smc202.lysopcac181	0.090045156	0.053926621	0.097247861
pcaec381.pcaec380	0.119942989	0.071832143	0.097248703
pcaac386.prolinr	0.037383548	0.022391636	0.097295499
pcaec384.kynurenine	-0.028648389	0.017160685	0.097317692
c12.smc202	-0.063800229	0.038227019	0.097404519

pcaac364.lysopcac160	0.112433737	0.067381772	0.097479015
c51.pcaac403	0.010703831	0.006415826	0.097530603
pcaec424.pcaec380	0.097761995	0.058598146	0.097530968
pcaac363.pcaec363	0.058080821	0.034820638	0.097599835
pcaac386.pcaac323	-0.102747042	0.061609742	0.097657977
pcaec343.lysopcac181	0.08617886	0.051684647	0.097719697
pcaec321.c5	-0.008526151	0.00511491	0.097814644
pcaac381.pcaac323	-0.119443859	0.071672709	0.09789498
pcaec321.smohc222	0.004860348	0.002916627	0.097912979
pcaac406.pcaac381	-0.022604003	0.01356645	0.097964526
pcaac363.pcaec361	-0.016141222	0.009690784	0.098073002
pcaec384.pcaac425	0.098444277	0.059110272	0.098111266
pcaec362.pcaec382	-0.017904801	0.010751465	0.098130918
smc180.c141oh	0.118610594	0.071253785	0.098273714
pcaec444.c3	-0.040264887	0.02418892	0.098277966
pcaac322.pcaac300	-0.137510768	0.082612183	0.098291299
pcaac366.pcaac281	-0.005092914	0.003060094	0.098338067
pcaec365.lysopcac180	0.099059921	0.059538878	0.098441087
c182.pcaec300	0.005327886	0.003202292	0.098443663
pcaec381.smohc221	-0.056941703	0.034226652	0.098465114
smc202.pcaec300	0.004029165	0.00242201	0.098485759
pcaec420.pcaec405	-0.112749671	0.067782657	0.09851833
pcaac420.pcaec300	0.016515408	0.009929951	0.09856017
smc161.c51dc	-0.138037218	0.083003803	0.098593955
pcaac363.smohc221	0.024004604	0.014436404	0.098642096
pcaac322.smc261	0.067514726	0.040603742	0.098644458
pcaac281.histidine	0.079944506	0.048087461	0.098702602
pcaac385.pcaec300	0.005713728	0.003437956	0.098808302
pcaec301.pcaac300	-0.119676859	0.072028037	0.098893393
pcaac363.histidine	0.078913974	0.047495073	0.098895893
leucine.c162	-0.132580405	0.079811925	0.098968055
c141.pcaac365	0.041603073	0.025045804	0.098984302
alanine.pcaac386	0.04728976	0.028474242	0.099042412
pcaec360.pcaec405	-0.121661611	0.073263856	0.099081789
pcaec321.pcaec300	0.006610871	0.003981567	0.09912734
pcaac420.pcaac323	-0.125325491	0.075482287	0.099135249
pcaac425.kynurenine	-0.022821526	0.013745642	0.099146583
acetylornithoine.lysopcac204	0.072393451	0.043606493	0.099170871
pcaac322.pcaac281	-0.005773087	0.003477928	0.099217578
lysopcac181.tyrosine	-0.065887343	0.039695091	0.099234754
methioninesulfoxide.pcaec404	0.040053114	0.024132754	0.099262334
c3.pcaac281	-0.007392764	0.004454464	0.099276211
c141.pcaec322	-0.070586774	0.042531752	0.099277238
pcaec402.c0	-0.000887397	0.000534949	0.099434817
smc241.smc161	0.149100666	0.089933955	0.099627579

pcaec385.pcaac323	-0.122331903	0.073810014	0.099729093
pcaac342.pcaec383	-0.078891871	0.047608049	0.099785165
c51dc.c0	-0.001781123	0.001075069	0.099857909
pcaac405.pcaac341	0.008162719	0.004927186	0.099874783
smohc141.pcaac281	0.025479583	0.015380269	0.099880608
c142.pcaac365	0.04591141	0.027718344	0.099938618
pcaac342.pcaec403	0.088578671	0.053487086	0.099995176
serotonin.lysopcac170	0.019629492	0.011858217	0.100142629
asymmetricdimethylarginine.pcaec38	-0.01144608	0.006914764	0.100150585
pcaec360.c2	0.084312046	0.05093659	0.100165791
pcaac384.lysopcac181	0.061419498	0.037107198	0.100174814
isoleucine.smohc161	-0.022406175	0.013539199	0.100231601
c5.pcaac323	0.183867758	0.111197248	0.100513103
pcaac342.pcaec361	-0.014715808	0.008899682	0.100514595
smc202.c141	-0.0578594	0.03503062	0.10088995
pcaac240.pcaac426	0.109593329	0.066364151	0.100948641
pcaec340.c5	-0.011610901	0.007031792	0.100988022
pcaac420.transhydroxyproline	-0.078699202	0.047670449	0.101049318
c51.pcaac343	0.029858042	0.018086197	0.101054774
c141oh.lysopcac204	0.03433863	0.020801311	0.101071738
c51dc.smohc221	0.031571363	0.019130176	0.101163443
c51.pcaac386	-0.017591349	0.010660817	0.101214579
pcaac323.pcaec364	-0.025314357	0.015343619	0.10126873
pcaac364.pcaec383	-0.090293823	0.054740037	0.101335799
pcaac281.smohc161	-0.045180337	0.027392269	0.101360325
c51dc.pcaec340	0.055933599	0.033916831	0.101410159
glycine.smohc221	-0.086650754	0.052545302	0.101424992
pcaec341.transhydroxyproline	-0.030224807	0.018332153	0.101493862
pcaac363.pcaec445	0.08384442	0.050861218	0.10154271
c51dc.pcaac426	0.083710612	0.05078594	0.101581992
pcaac363.c162	-0.090350663	0.054815538	0.101589292
smc260.c141	-0.024787098	0.015040856	0.10164742
c142.lysopcac204	0.031577465	0.019162389	0.101667324
pcaac365.lysopcac180	0.082171275	0.049869641	0.101701511
pcaac323.pcaec361	-0.006643666	0.004032704	0.101757953
pcaac381.c141oh	0.118369648	0.071856548	0.101787178
asparagine.pcaec422	0.037716938	0.022898465	0.10182149
pcaac343.lysopcac180	0.090962666	0.055224857	0.101822635
pcaec401.pcaac381	-0.088643254	0.053818628	0.101834746
serotonin.pcaec322	0.07632894	0.046354741	0.101926888
smc161.pcaec360	-0.007754291	0.004710322	0.102007761
c181.pcaec424	-0.052000743	0.031589967	0.102032123
pcaec340.smc241	0.167040884	0.101496931	0.10210286
c18.pcaec364	-0.028028797	0.017034017	0.102167364
pcaec321.pcaec404	-0.013092538	0.007960702	0.102335483

c51dc.lysopcac280	-0.012943881	0.007870803	0.102356627
lysopcac203.c181	0.028101442	0.017089549	0.102393774
prolinr.c162	-0.133050021	0.08091554	0.102405508
c14.smohc222	-0.017766104	0.010806981	0.102480062
pcaec341.lysopcac181	0.045256698	0.027531326	0.102505187
pcaac342.smc161	-0.104677685	0.06368384	0.102529361
c141.pcaec380	-0.066773146	0.040624454	0.10253764
pcaec340.pcaac402	-0.074391571	0.045278509	0.102680809
c182.pcaac402	0.025754157	0.015675542	0.102686168
pcaec385.smohc221	0.023608547	0.014370882	0.102716698
pcaac360.pcaac323	-0.13893672	0.084612575	0.102876484
c182.pcaec380	-0.059744412	0.036384513	0.102877594
pcaac362.prolinr	0.035438431	0.021589864	0.10300016
pcaec365.c14	-0.053167175	0.032400056	0.1030997
pcaac406.c5	-0.012822761	0.007817718	0.103253949
c141.phenylalanine	-0.074578137	0.045470513	0.103269576
asymmetricdimethylarginine.c18	-0.035195931	0.021460541	0.103293063
lysopcac182.lysopcac180	-0.128963506	0.078654821	0.103379823
pcaac425.pcaac404	-0.014497478	0.008842155	0.10338545
pcaac323.pcaac343	0.03022648	0.018440398	0.103477545
pcaec321.pcaec342	-0.182432366	0.111308957	0.103513367
smc161.pcaec382	-0.020150249	0.012296885	0.103581424
pcaec423.lysopcac181	0.101803779	0.062138606	0.10364674
c8.c51dc	0.147532974	0.090087288	0.103786052
pcaac322.transhydroxyproline	0.02791549	0.017046974	0.103807814
lysopcac170.pcaec300	-0.015502625	0.009467253	0.103820995
pcaec322.pcaec300	0.003870205	0.002363823	0.103870045
leucine.pcaec445	0.052212529	0.031893202	0.10390351
pcaec321.kynurenine	-0.013145984	0.008033138	0.104036601
c8.c141	0.067121673	0.041018358	0.104055062
pcaec341.smc241	0.22918252	0.140062383	0.104074415
pcaec365.c18	-0.053606991	0.032766114	0.104124524
pcaec341.histidine	0.069230152	0.042327793	0.104224688
c51dc.lysopcac181	-0.10319923	0.063115748	0.104328129
lysine.smohc222	-0.007368171	0.004507688	0.104433162
smc161.smohc161	-0.076029324	0.046531565	0.10456949
pcaac401.pcaec321	0.067239929	0.041158011	0.104617446
pcaac405.pcaec403	0.112956439	0.069168282	0.10475111
pcaec383.c181	0.052599225	0.032209299	0.104755816
lysopcac170.smc261	0.048075294	0.029442244	0.104793049
pcaec443.pcaec381	0.233067913	0.142743864	0.104813647
alanine.pcaec322	-0.057242483	0.035060187	0.104830132
c12.lysopcac204	0.029640739	0.018156745	0.104872282
pcaec401.smc261	0.052940921	0.032452497	0.105116171
acetylornithoine.lysopcac203	-0.031979312	0.019605775	0.105162404

pcaac404.methionine	-0.060533085	0.037112265	0.105170131
c101.smohc221	0.059245847	0.036332645	0.105260962
smc241.smc240	-0.098867262	0.060647081	0.105354877
pcaac362.pcaac281	0.002530931	0.001552607	0.105373703
pcaec340.pcaec300	-0.044118188	0.027069804	0.105442283
ornithine.pcaac406	0.039640651	0.024322923	0.105448272
smc260.pcaac404	0.004108608	0.002521344	0.105497826
pcaac323.pcaac384	-0.100777703	0.061849623	0.105525839
pcaec444.pcaac281	-0.003003487	0.001843352	0.105533746
pcaec363.pcaec322	-0.101413411	0.062241414	0.105534947
glycine.c14	0.056831421	0.034880624	0.105544223
pcaac406.smc160	-0.076227614	0.04679549	0.105620491
pcaec443.pcaec380	0.131620529	0.080848994	0.10582677
c51dc.lysopcac182	0.057249839	0.035168636	0.105850863
ornithine.pcaec321	-0.082599546	0.050741797	0.105856377
pcaac343.smohc161	0.03076077	0.018903588	0.105982855
phenylalanine.c162	-0.112430885	0.069098306	0.106010521
acetylornithoine.c141oh	0.119494832	0.073442095	0.106021803
ornithine.pcaac281	0.005372413	0.003302277	0.106060254
alphaaminoadipicacid.lysopcac280	-0.011197527	0.006883262	0.10608258
c4.lysopcac161	-0.1031545	0.063412828	0.106095908
smc160.spermidine	0.083422545	0.051291844	0.106156422
smc161.lysopcac181	0.054794869	0.033691178	0.106165532
pcaac401.pcaac403	-0.11380926	0.069983754	0.106200076
pcaec406.pcaac323	-0.131296242	0.080748749	0.106251006
pcaac380.pcaec300	0.009877249	0.006075359	0.106292792
pcaec403.pcaac404	-0.022562771	0.013880824	0.106362133
pcaac425.pcaac403	-0.026942016	0.016586768	0.106608702
asymmetricdimethylarginine.lysopca	0.030019192	0.018481657	0.106616857
pcaac421.pcaec361	-0.025784997	0.015875385	0.106629066
lysopcac203.smohc221	-0.028353091	0.017459029	0.106678999
pcaac281.pcaac402	-0.061823106	0.038071236	0.106700147
pcaec381.pcaac402	-0.064926841	0.039993695	0.106796951
phenylalanine.lysopcac160	-0.090928491	0.056016383	0.10683518
pcaac300.kynurenine	-0.014363797	0.008850906	0.106917892
smc161.c181	0.012789069	0.0078841	0.107073683
c142.pcaec443	0.102139802	0.062976821	0.107131753
methionine.c162	-0.106289618	0.065555609	0.107238504
pcaac421.pcaac323	-0.149007277	0.091913949	0.107282517
kynurenine.phenylalanine	0.089350663	0.055143926	0.107463639
spermidine.lysopcac181	-0.251094049	0.154995787	0.107530667
methionine.pcaac323	0.148274325	0.09153109	0.107546071
citruline.alanine	-0.026260146	0.016213802	0.107614297
asparagine.pcaec444	-0.083634281	0.051645156	0.107660488
lysopcac170.pcaac425	0.096595584	0.059652567	0.107681837

methioninesulfoxide.pcaac405	-0.194303553	0.120008618	0.10772987
pcaec383.pcaec343	0.105888994	0.065422637	0.107846747
pcaac422.c0	-0.001045923	0.000646243	0.107862028
pcaac360.pcaac343	0.060519863	0.03739618	0.107888586
asparagine.smc181	0.036765906	0.022721771	0.10794292
lysopcac140.pcaac364	0.081885461	0.050610847	0.107974979
pcaec385.pcaac404	0.018588354	0.01148922	0.107985258
c16.pcaec382	-0.019882483	0.012289367	0.107992731
pcaac344.c162	-0.082646446	0.051084947	0.108000671
c12.pcaec443	0.099597188	0.061567147	0.108027351
smc161.lysopcac180	0.076848266	0.047508917	0.108058792
pcaec444.pcaec382	-0.015028194	0.009293011	0.108146127
pcaac344.pcaac405	0.037946954	0.02346697	0.108170634
c182.pcaec383	0.106795629	0.066058116	0.108245138
prolinr.pcaac323	0.11268254	0.069701865	0.108257263
c141oh.lysopcac181	0.068139538	0.042152059	0.108282891
pcaec341.pcaec360	0.008765875	0.005422975	0.10830117
pcaac385.c141	-0.040232577	0.024895267	0.108378969
c2.pcaac281	0.019391539	0.011999731	0.10839531
c16.glutamicacid	-0.090865096	0.056239983	0.108466876
serine.c18	0.070851894	0.043854496	0.108478612
pcaac362.pcaec383	-0.076682731	0.047468241	0.108513179
pcaac420.lysopcac180	0.065071885	0.040281225	0.108516177
asymmetricdimethylarginine.prolinr	0.016608454	0.01028289	0.108577973
smc241.pcaac404	0.00576857	0.003572884	0.108710806
pcaac383.c51dc	-0.144338154	0.089412417	0.108764013
pcaac383.pcaac384	-0.109179402	0.067675057	0.10898291
pcaec362.pcaac402	0.052971793	0.032837733	0.109015679
lysopcac140.smohc222	-0.007969156	0.004940501	0.109040041
lysopcac160.lysopcac180	0.103775691	0.064347455	0.109102303
pcaec384.transhydroxyproline	-0.038685857	0.024005874	0.109368716
methioninesulfoxide.kynurenine	-0.026727692	0.016585819	0.109376935
pcaec321.pcaec445	0.102466105	0.063588598	0.109395991
smohc221.smc261	0.049731069	0.030868952	0.109472905
asparticacid.c14	-0.063278489	0.039280128	0.109491283
phenylalanine.pcaac281	0.003085635	0.001915423	0.109494083
pcaac344.tyrosine	0.07924915	0.049201584	0.109546062
smc202.smc181	-0.035195497	0.02185131	0.109550832
c182.pcaec364	-0.023663555	0.014695674	0.109647301
c12.pcaac403	-0.007315232	0.004543254	0.109671103
asymmetricdimethylarginine.c14	-0.029118204	0.018084571	0.109674932
pcaac363.pcaac240	-0.023364284	0.0145118	0.109695342
pcaec364.c141	-0.069888169	0.04341669	0.109763576
pcaec446.pcaec364	0.037171878	0.023093348	0.109779258
asymmetricdimethylarginine.pcaac4(	0.012017932	0.007467224	0.109825453

pcaac281.prolinr	0.036760851	0.022843964	0.109871249
pcaac320.pcaec342	-0.160744077	0.099969344	0.110151881
pcaac405.pcaec300	0.008549575	0.005317638	0.110186249
pcaec360.pcaac385	-0.065938875	0.041019988	0.110251035
pcaec343.c141oh	0.139458059	0.0867663	0.110294455
isoleucine.pcaac380	0.087796702	0.054631471	0.110340944
spermidine.c18	0.083905472	0.052212558	0.110357187
lysopcac160.prolinr	-0.051788271	0.032239347	0.11049545
acetylornithoine.alanine	-0.022969418	0.014299068	0.110497817
lysopcac203.arginine	0.079153728	0.049285704	0.110572493
leucine.pcaac323	0.129177399	0.080435977	0.110584055
pcaac366.methionine	-0.053440635	0.033297109	0.110804633
c142.c8	0.082440735	0.051366441	0.110806832
pcaac343.pcaac281	0.005400406	0.003365043	0.110828497
pcaac425.pcaac365	0.08259249	0.051465765	0.110839603
pcaac380.pcaec444	0.169076692	0.105359758	0.110850309
smc161.pcaec300	0.00493842	0.003077739	0.110893233
pcaac383.pcaac402	-0.038666261	0.024107779	0.111040906
pcaac363.lysopcac161	0.086193296	0.053745947	0.111079376
pcaac404.pcaec383	-0.082608645	0.051516369	0.111118097
smc160.smc261	0.053985041	0.033666239	0.111119259
smc261.pcaac403	0.011431182	0.00712882	0.111123597
c16.c102	0.043387075	0.027058377	0.111135722
c102.pcaec445	-0.077147252	0.048113405	0.111139205
pcaac421.pcaec300	0.01459171	0.009102989	0.111246972
pcaec381.pcaec343	0.086244814	0.053814935	0.11132204
c142.pcaec444	-0.130000729	0.081118169	0.111324097
pcaec342.pcaec404	-0.012497013	0.007801103	0.111468877
smc160.pcaec341	0.117499602	0.073380538	0.111627908
pcaec321.c2	0.05307565	0.033154791	0.111714907
lysopcac180.pcaac405	-0.108935878	0.06805064	0.111723329
c2.smc202	-0.040726242	0.025443243	0.111753453
smc181.smohc221	0.021819918	0.013634756	0.111832017
lysopcac203.pcaac406	0.088559047	0.055349755	0.111904421
pcaac385.pcaec404	-0.011655282	0.007284834	0.11191595
pcaac323.pcaec445	0.069687548	0.043562445	0.111965191
glycine.pcaac321	-0.042318833	0.026455608	0.111987278
lysopcac140.pcaec361	0.013852016	0.008660233	0.112013913
pcaac300.smc241	0.111551026	0.069742745	0.11202116
creatinine.c4	-0.04588386	0.028692922	0.112094295
c102.pcaec364	0.029264069	0.018302066	0.112135698
lysopcac161.pcaac364	0.073856761	0.046192681	0.112150032
pcaac322.c102	0.058286393	0.036467102	0.112273833
pcaec406.c141	-0.045629321	0.028552689	0.11233031
pcaac360.c5	-0.034230612	0.02142038	0.112338092

smohc221.c162	-0.116745442	0.073079356	0.112454705
smc261.c141	-0.041984875	0.026287618	0.112539647
asparticacid.smohc221	0.079793828	0.04996358	0.112560872
c16.smohc221	-0.10793935	0.067590909	0.112580754
pcaec406.kynurenine	-0.014738891	0.00922956	0.112587088
pcaac424.pcaac404	-0.016142777	0.010109036	0.11259963
putrescine.pcaac404	-0.009204871	0.005765167	0.112651056
pcaac281.c141oh	0.130316705	0.081625218	0.112675742
smohc222.pcaac384	-0.063972376	0.040071628	0.112692352
smc241.smc261	0.132546322	0.083035127	0.112733117
pcaac365.c141	-0.057880743	0.036264696	0.112778905
pcaac422.pcaec443	0.105444373	0.066089509	0.112909611
pcaec384.histidine	0.068013347	0.042633974	0.112952789
pcaac386.pcaec404	-0.01433397	0.008987316	0.113036486
alphaaminoadipicacid.smohc221	0.034646527	0.021723378	0.113039882
ornithine.tyrosine	-0.059140259	0.037098335	0.11320722
c51.pcaec363	-0.020573342	0.012908068	0.113277296
c4.pcaec343	-0.099334511	0.062332675	0.11332603
citruline.smohc161	0.011718856	0.007353821	0.113336069
pcaec402.pcaac402	-0.125739199	0.07891848	0.113402086
pcaac385.c0	-0.000623285	0.000391252	0.113452749
pcaac364.pcaec322	-0.086529558	0.054327421	0.113522769
pcaec322.smohc221	0.015626495	0.00981337	0.113606797
smc160.pcaec443	0.050658687	0.031814223	0.113615674
smohc221.pcaac402	0.055667724	0.034963818	0.113655223
asymmetricdimethylarginine.pcaec34	0.121084864	0.076064349	0.113717787
asparticacid.transhydroxyproline	-0.06259541	0.039322266	0.113721705
c3.pcaac406	0.045483148	0.028574482	0.113747926
c51dc.c8	0.05569076	0.034992657	0.113802268
smohc161.pcaac384	-0.051580167	0.032443757	0.114177414
kynurenine.pcaec445	-0.068358455	0.043005226	0.114243986
creatinine.pcaac404	-0.005757562	0.003622283	0.114256137
c51.lysopcac181	-0.072072186	0.04535055	0.114314927
pcaac323.pcaec300	0.002303277	0.001449386	0.114334072
pcaec406.arginine	-0.038666117	0.024331504	0.114334377
c141oh.pcaec443	-0.091647983	0.057675415	0.114358687
pcaac341.lysopcac181	0.039009393	0.024550592	0.114379313
c102.pcaac240	-0.015226402	0.009589419	0.114629236
arginine.lysopcac170	-0.023808379	0.014994315	0.11463073
c5.pcaec322	0.184223342	0.116059549	0.114746117
pcaac281.pcaac344	-0.102612386	0.064646306	0.114752493
pcaac361.pcaec383	-0.088735278	0.055914107	0.114819682
c182.pcaac406	0.033275178	0.020969659	0.114857698
lysopcac180.smohc221	-0.06059613	0.038190497	0.114890357
alphaaminoadipicacid.pcaec361	-0.015793843	0.009956119	0.114966457

asparagine.smc261	0.033784388	0.021297385	0.114973039
pcaac380.lysopcac203	-0.025800686	0.016265961	0.115004731
c51dc.lysopcac204	0.027154542	0.017122045	0.115058334
pcaac383.pcaec363	-0.048664381	0.030696191	0.115191094
pcaac320.pcaec322	-0.092607754	0.058415896	0.11519973
c51.lysopcac204	0.024045262	0.015171385	0.115292662
serotonin.pcaec443	-0.085482754	0.053938915	0.115315877
pcaac281.pcaec300	0.010562926	0.006665238	0.11532228
leucine.pcaec340	0.046267862	0.029195203	0.115322821
pcaec384.smohc221	0.040493024	0.025557582	0.11541203
serine.smc202	-0.053239789	0.033610115	0.115490163
smc161.smohc221	0.020100108	0.01268945	0.115499085
pcaec341.pcaec404	-0.025561927	0.016139285	0.115537532
pcaec322.pcaac384	-0.119075616	0.075222322	0.115731268
methionine.pcaac322	0.074089935	0.046819631	0.115851706
pcaec403.pcaec380	0.101435934	0.064100565	0.115852754
smohc141.prolinr	0.04357367	0.027542732	0.115946611
pcaec404.pcaec380	0.090808673	0.057402433	0.115963332
pcaec322.lysopcac180	0.086875149	0.054922121	0.116003906
pcaec343.smc261	0.048381642	0.0305878	0.116017132
smohc221.c141	-0.058842713	0.037205056	0.116051785
pcaec446.pcaac402	0.046305319	0.029278118	0.116054283
pcaec420.pcaac402	0.052628805	0.033276573	0.116056625
smc261.c141oh	0.151097978	0.095546181	0.116089576
lysopcac203.pcaec422	0.068163858	0.043108963	0.116138469
c0.lysopcac181	-0.055045512	0.034816721	0.116182219
pcaac342.pcaac404	0.00392418	0.002483099	0.116331467
pcaac361.c162	-0.094492606	0.05980792	0.116427917
c3.citruline	0.060197109	0.038112359	0.116535735
c51.pcaec360	-0.006420264	0.004065686	0.116611405
pcaac402.c5	-0.038231643	0.024227877	0.116871364
lysopcac281.glycine	-0.06852951	0.043439747	0.11696929
arginine.pcaac362	0.098624031	0.062521724	0.117001484
pcaec341.pcaac402	-0.061518547	0.039003304	0.117040865
histidine.pcaac344	0.043651062	0.027680323	0.117108565
smohc241.lysopcac160	0.022828232	0.014478358	0.117167662
pcaec342.smohc221	0.023191762	0.014714083	0.11729512
pcaac362.pcaac365	-0.065917117	0.041826412	0.117339411
pcaac425.smohc222	0.012797977	0.008121107	0.117357423
pcaac323.pcaec404	-0.005271552	0.003345771	0.117427481
smohc241.pcaac384	-0.07967886	0.050574987	0.117456787
pcaec380.pcaec404	-0.034052882	0.021622516	0.117590484
pcaec365.smc160	0.204712442	0.130020955	0.117688275
smohc141.smc181	0.065682641	0.041717682	0.117688653
h1.c162	-0.140467426	0.08921704	0.117690852

pcaec365.lysine	-0.104903386	0.066641775	0.117761956
kynurenine.lysopcac170	-0.025261603	0.016056497	0.117957165
creatinine.pcaec445	-0.079192506	0.050341827	0.118003283
smc260.pcaac386	-0.036611979	0.023281603	0.118124757
lysopcac161.pcaec322	-0.099062533	0.063015548	0.118249516
pcaac424.spermidine	-0.078887263	0.050185304	0.118275866
alphaaminoadipicacid.c141	-0.028689886	0.018253803	0.118321953
smc160.pcaec382	-0.015184345	0.009667094	0.118553291
lysopcac281.pcaac364	0.078111095	0.049730586	0.118562523
pcaec420.arginine	-0.059617177	0.037958009	0.118580321
pcaec364.smohc221	0.043356806	0.027611194	0.118660946
lysopcac203.pcaac404	0.007826166	0.004985555	0.118775827
pcaac281.c141	-0.041480155	0.02642545	0.118790595
pcaec443.smohc222	0.047316191	0.03014625	0.118825519
glutamine.c182	0.076087838	0.048494303	0.118953428
pcaac381.methioninesulfoxide	0.084444984	0.05382714	0.118997191
pcaac322.smohc221	0.047288573	0.030147646	0.119055928
pcaec403.pcaac403	0.016667025	0.010629954	0.119204303
pcaec365.pcaac404	0.015007188	0.009572477	0.119247899
smohc161.pcaec382	-0.021925273	0.013985282	0.119248903
pcaec424.c141oh	0.101451259	0.064741984	0.119419536
pcaec385.pcaec445	0.081508158	0.052025328	0.1194914
pcaec340.pcaac384	-0.057921512	0.036975667	0.119544033
c51.smc202	-0.04091263	0.02611767	0.1195448
methionine.pcaec405	0.064571698	0.04122614	0.119589902
pcaec386.c141	-0.044403424	0.028358032	0.119698937
c4.smc181	-0.035256793	0.022519366	0.119744309
c51.pcaec341	0.067820041	0.043321191	0.1197688
pcaac406.pcaec300	0.012625562	0.008067294	0.119882687
c12.pcaac365	0.041738373	0.026684545	0.120091614
pcaac362.smc160	-0.148370086	0.094862276	0.120110989
pcaac281.pcaec360	0.007505697	0.004799418	0.120153586
putrescine.smohc221	-0.035656512	0.022802719	0.120196083
pcaac321.pcaec322	-0.083887775	0.053675661	0.120391544
pcaac343.pcaec404	-0.016406244	0.010500696	0.120501779
c10.c0	0.000899057	0.000575518	0.120555373
pcaac343.smc261	0.04041672	0.025874146	0.120583166
kynurenine.pcaec300	0.00490132	0.003138145	0.120629811
c51dc.pcaac403	-0.006786169	0.004345145	0.120646432
c141.pcaac406	0.133038485	0.085202759	0.120728444
lysopcac180.arginine	0.065980405	0.042262575	0.120783226
lysopcac203.pcaec364	-0.014733742	0.009438004	0.120805389
alphaaminoadipicacid.pcaac321	0.03074943	0.019698294	0.120826105
pcaec364.smc160	0.214273044	0.137266889	0.120831799
lysopcac204.histidine	0.085343267	0.05467358	0.120840275

pcaac422.c2	0.089867216	0.057584389	0.120921054
pcaec361.pcaec382	-0.023045478	0.014768012	0.120948917
pcaac362.pcaac402	0.101651555	0.065150682	0.121007157
pcaac384.smc161	-0.057984564	0.037171822	0.121089074
histidine.c102	0.054921595	0.035214351	0.121152837
pcaec443.smc202	-0.092730398	0.059456846	0.121155554
putrescine.pcaec300	-0.009684101	0.00621036	0.121221555
pcaec386.kynurenine	-0.009013019	0.005780191	0.121233861
creatinine.pcaac320	-0.097877814	0.062771894	0.121241531
serine.lysopcac160	-0.019001934	0.012186685	0.121247302
smc240.histidine	0.060627079	0.038883386	0.12125551
pcaac380.pcaec443	0.05639333	0.036176924	0.121345966
pcaac240.c51dc	-0.16265863	0.104398852	0.121528498
pcaec343.pcaec300	0.005307777	0.003406809	0.121542501
lysopcac280.pcaec364	0.009770767	0.006271443	0.121545673
pcaec425.c181	0.02033006	0.013059954	0.121855561
pcaec360.c51	-0.175021686	0.112440555	0.121879521
pcaac421.c51dc	-0.161810395	0.103956971	0.121893214
c102.c14	-0.059182091	0.038028154	0.121950905
pcaec365.pcaec404	-0.014083215	0.009049606	0.121961893
lysopcac170.pcaec363	0.021500302	0.013819989	0.12207716
phenylalanine.pcaec343	0.084734001	0.054473747	0.122133751
lysopcac204.pcaac380	0.098060519	0.06304658	0.122165984
glutamicacid.lysopcac181	-0.098992316	0.063676275	0.122344046
pcaec381.pcaec404	0.030197745	0.019425445	0.122361363
pcaec342.lysopcac161	0.120116187	0.077282747	0.122433498
pcaec424.pcaec343	0.091908864	0.059162041	0.12260826
c102.pcaec300	0.009261129	0.005961737	0.122628023
glutamine.pcaec444	-0.131748295	0.08481891	0.122661146
smohc141.c181	0.024046194	0.015484425	0.122747458
pcaac421.smc202	-0.078402277	0.050494917	0.122807485
smc160.pcaec300	0.013074934	0.008421872	0.122850315
pcaec384.smc261	0.054590538	0.03516375	0.122857749
acetylornithoine.pcaec402	-0.054166417	0.034890636	0.122858586
pcaec386.pcaac406	0.088561476	0.057052094	0.122899577
asymmetricdimethylarginine.pcaec38	0.044230342	0.028506796	0.123071752
c2.pcaac405	0.027396734	0.017659237	0.123110278
lysine.pcaec342	0.140009808	0.090248272	0.123116518
pcaec446.lysopcac203	-0.02032238	0.013099711	0.123122255
pcaec321.smohc161	-0.039328115	0.0253659	0.123344776
pcaac403.pcaec321	0.063124566	0.040725148	0.123445078
pcaec301.pcaec322	0.069079027	0.044578128	0.123540465
pcaac322.pcaec404	-0.028629968	0.018483299	0.123697319
pcaec301.pcaec300	-0.009304941	0.006007392	0.123709077
pcaac321.c5	-0.011222727	0.007245886	0.123726878

c51dc.pcaac402	0.02289326	0.014780976	0.123728915
lysopcac203.smc261	0.053928991	0.03482303	0.123770746
prolinr.lysopcac140	-0.019228201	0.012417846	0.123825077
pcaec364.pcaac384	-0.086174725	0.05565571	0.12384413
pcaac342.prolinr	0.034635625	0.022378912	0.124003819
c51dc.c14	-0.034795563	0.022488617	0.12410936
pcaac361.arginine	0.045064694	0.029130745	0.124174871
pcaec404.acetylornithoine	-0.097098954	0.062768023	0.124182378
pcaec362.pcaac405	-0.064057686	0.041416249	0.124247331
c16.pcaec405	-0.089706847	0.058006634	0.124292479
pcaac344.methionine	-0.034305364	0.022183964	0.124314005
c14.smohc241	0.0889942	0.057577289	0.124496564
alphaaminoadipicacid.pcaac323	0.151149922	0.097801867	0.124539309
pcaac343.pcaac240	-0.02511351	0.01625142	0.124577747
pcaec321.smohc221	0.02499435	0.016179656	0.12470122
pcaec360.pcaec300	-0.019866713	0.012862257	0.124756068
h1.pcaec444	-0.102799455	0.066557745	0.124770368
pcaac402.pcaac404	-0.023886568	0.015467164	0.124812674
pcaec363.smohc221	0.02189588	0.014180805	0.124882883
pcaec383.pcaac403	0.02194744	0.014214584	0.124893038
pcaec445.pcaac386	0.028665303	0.018568073	0.124944719
isoleucine.c2	-0.052699093	0.034137865	0.124964467
pcaec401.pcaec406	-0.064062995	0.041506373	0.125028502
methionine.smc160	-0.107961383	0.069950542	0.125041505
pcaec343.pcaec404	-0.011324217	0.007337742	0.125068734
pcaac402.pcaec343	0.114318841	0.074076451	0.125075717
c182.c2	0.073434222	0.047588789	0.125113465
c18.histidine	0.063692859	0.041286738	0.125211439
lysopcac160.smc261	0.055949248	0.03626732	0.125212637
pcaac381.c2	0.089888124	0.058289513	0.125356549
pcaec322.c14	-0.028726762	0.018629021	0.125369701
smohc241.pcaec300	-0.006804747	0.004413226	0.125404926
isoleucine.pcaec424	-0.025902035	0.016799831	0.12542808
alphaaminoadipicacid.smohc161	-0.01472366	0.009553182	0.125567252
pcaac380.pcaac362	-0.054956246	0.035658308	0.125577071
smohc222.arginine	-0.066615672	0.04322981	0.125631669
pcaac386.pcaec361	-0.015953406	0.010354491	0.125690756
pcaac361.pcaac402	-0.025989449	0.016869437	0.125715077
glutamicacid.pcaec300	-0.04458803	0.028946677	0.12578153
pcaac342.c51dc	-0.131432174	0.085343819	0.125859241
pcaac421.pcaac343	0.025647751	0.016659965	0.125992698
pcaec341.kynurenine	-0.009909763	0.006437112	0.125995262
pcaec300.c0	-0.000455309	0.000296058	0.126378719
methioninesulfoxide.spermidine	-0.077030077	0.050089417	0.126391401
pcaec301.pcaac386	-0.026382542	0.01715625	0.126408783

pcaec342.pcaec300	0.005504912	0.003579888	0.126420321
pcaec365.kynurenine	-0.012247477	0.007965241	0.126449095
leucine.pcaec422	-0.049294215	0.032063139	0.126499146
c18.smohc241	0.086711382	0.056404316	0.126521796
pcaac424.arginine	-0.100798155	0.065578181	0.126582914
spermidine.kynurenine	-0.051114234	0.033259302	0.126638791
pcaec445.pcaec361	-0.012237519	0.007963587	0.126676992
pcaac380.c14	-0.058297385	0.037938295	0.126688591
pcaec445.pcaac421	0.099047174	0.064487254	0.126864628
pcaec404.pcaac404	-0.014142692	0.009208208	0.126874346
pcaec402.c141oh	0.10772249	0.070145202	0.126916485
c0.taurine	-0.058390698	0.038045904	0.127153075
pcaac420.c5	-0.01261684	0.008221083	0.1271654
pcaec343.smohc221	0.021659753	0.014114505	0.12719467
pcaac426.smohc161	-0.014760386	0.009620051	0.127253282
c0.smc181	-0.029797615	0.019425184	0.127343608
asparagine.pcaec363	0.013107158	0.008545194	0.12736944
pcaec444.smc202	-0.074276082	0.048425357	0.127378521
c162.pcaac365	0.053681583	0.035000547	0.127400831
c51dc.pcaec342	0.108610255	0.070828678	0.127477979
c102.c18	-0.065495637	0.042719322	0.127542025
pcaac406.pcaec361	-0.013623526	0.00888859	0.12765626
pcaec363.pcaec343	0.1042433	0.068013189	0.127657666
leucine.pcaac406	0.04812853	0.03140458	0.127697159
pcaec365.smohc221	0.030889793	0.02015837	0.12774066
pcaec383.c5	-0.029987912	0.01957017	0.127747585
pcaac425.lysopcac280	0.011140364	0.007270799	0.12777752
lysopcac203.pcaec445	0.082954976	0.054144374	0.127801983
c51.pcaac384	0.095696757	0.062467912	0.127844505
lysopcac161.lysopcac170	0.031231829	0.020390136	0.127899292
pcaec420.pcaac381	-0.023562595	0.015385895	0.127966247
pcaac363.serotonin	0.146783435	0.095849268	0.127976801
prolinr.pcaac425	-0.093183145	0.060848409	0.127976891
h1.pcaec403	-0.064771712	0.042298954	0.128005097
lysopcac160.lysopcac182	-0.053570591	0.035011752	0.128304031
smohc241.pcaec443	0.063652349	0.041602569	0.128319994
pcaec380.pcaec422	0.077576306	0.050716317	0.128418414
leucine.pcaec424	-0.034102459	0.022301668	0.128534456
smc180.pcaac341	0.035679466	0.023334156	0.128553727
pcaac320.histidine	0.057542477	0.037638727	0.128617087
smohc141.pcaac384	-0.055843908	0.036529231	0.128633096
smc161.pcaac281	0.002207944	0.001444448	0.128675882
glutamicacid.pcaac406	0.092850827	0.06075475	0.128746326
c0.lysopcac161	-0.089555063	0.058606678	0.128800897
c142.lysopcac170	-0.019907855	0.013030021	0.128856515

c12.smohc221	-0.054960693	0.035978496	0.128917782
c102.c162	0.134173361	0.087843421	0.128963323
lysopcac181.pcaac425	-0.101711306	0.066592156	0.128972848
pcaec361.pcaec404	-0.067277255	0.044060697	0.129085793
smc261.pcaec342	-0.104288906	0.068305572	0.129116308
smc180.histidine	0.067753335	0.04437815	0.12913423
lysopcac180.lysopcac182	-0.07355199	0.048178375	0.129151007
c181.pcaec405	-0.057758912	0.037838063	0.129196624
smc180.pcaac384	-0.082748333	0.054216007	0.129247674
smc202.phenylalanine	-0.056081124	0.036749156	0.129302233
pcaac386.c14	-0.087175862	0.057125288	0.129303539
phenylalanine.pcaec300	0.01683307	0.011031208	0.129327665
lysopcac280.pcaac402	-0.111418169	0.07301866	0.129343501
c3.pcaac240	-0.038071574	0.024953594	0.129391115
serine.pcaec444	0.153720665	0.100754751	0.129391999
pcaac404.pcaac386	0.041112271	0.026947796	0.129408194
pcaac404.pcaec360	0.00551892	0.003617849	0.129447111
pcaec322.pcaac362	-0.047089864	0.030874569	0.129514677
lysine.pcaec443	-0.067419557	0.044205865	0.129532645
c182.transhydroxyproline	-0.040660455	0.026662781	0.129566975
asymmetricdimethylarginine.smohc2	0.013036651	0.008551668	0.129699705
histidine.pcaac365	-0.069777041	0.045774511	0.129722799
pcaec361.pcaec300	-0.07187107	0.047160035	0.129818146
lysopcac204.pcaac406	0.044802255	0.029398402	0.12982152
pcaec342.pcaec322	-0.12823135	0.084143412	0.129823312
smc181.smc180	-0.135726386	0.089090195	0.129945758
c102.pcaac386	-0.018912786	0.012415472	0.12998265
smc181.pcaec404	-0.01119741	0.00735115	0.130008918
smohc161.c141	-0.036523361	0.023981055	0.130061413
smc181.prolinr	0.02321713	0.015249829	0.13020078
pcaac361.c0	-0.001396671	0.000917661	0.130316388
pcaac404.pcaac384	0.064961945	0.042684877	0.130339946
pcaac361.pcaac240	-0.020187307	0.013264958	0.130350877
serotonin.pcaac343	-0.042024356	0.027622306	0.130466238
lysopcac182.lysopcac181	0.081317278	0.053457543	0.130525329
pcaec382.smohc221	-0.088817126	0.058390037	0.130539271
pcaac361.pcaec363	-0.026753466	0.017589779	0.130572918
pcaac425.lysine	-0.118785496	0.078099821	0.130578435
pcaec404.pcaac403	-0.138724397	0.091245395	0.130729116
pcaec421.pcaec424	-0.043501969	0.028616726	0.130775642
pcaac383.pcaac406	-0.071298381	0.046913207	0.130867434
lysopcac204.smohc221	-0.038419364	0.02528494	0.130952132
smc180.smc261	0.085711486	0.056414009	0.130983955
pcaec363.pcaec445	0.052224909	0.034373938	0.130987167
pcaec364.tyrosine	-0.060347172	0.039725665	0.131042294

pcaec446.c18	-0.031661045	0.020842293	0.131047565
arginine.lysopcac161	0.091397373	0.060189837	0.13119648
smohc241.histidine	0.064626877	0.042561825	0.131211993
ornithine.pcaac385	0.042139921	0.027754854	0.131245581
pcaac385.spermidine	0.082268254	0.054193303	0.131305555
c181.pcaac405	0.074280126	0.048940682	0.131379577
h1.pcaac424	0.018370035	0.012107249	0.131501246
lysopcac161.pcaec364	-0.024520838	0.016163235	0.131551904
phenylalanine.spermidine	-0.085069033	0.056077153	0.131570837
acetylornithoine.smohc161	-0.023544358	0.015522219	0.131617094
pcaec382.pcaac402	-0.085856536	0.056605009	0.131629933
smc161.pcaec404	-0.01015484	0.006697018	0.131741812
pcaec365.lysopcac281	0.027245754	0.017968645	0.131748951
glutamicacid.pcaec445	-0.042687649	0.028155815	0.131792504
pcaec342.lysopcac180	0.112198379	0.074004883	0.131799493
pcaac360.kynurenine	-0.013022279	0.008590193	0.131836677
pcaac366.c5	-0.019108599	0.012606148	0.131869848
lysopcac161.pcaac240	-0.022831157	0.015063101	0.131898903
acetylornithoine.pcaac365	0.055919254	0.03690695	0.13204039
pcaec362.c0	-0.001061825	0.000700833	0.13205365
pcaec360.pcaec383	-0.156934981	0.103603491	0.132135604
pcaac422.smohc221	-0.121195225	0.080037123	0.132269261
pcaec406.c18	-0.040835014	0.026968445	0.132284489
c0.c5	-0.02481518	0.016394211	0.132416951
c8.lysopcac280	-0.013880621	0.009170899	0.132443391
glutamicacid.pcaec422	-0.090064882	0.05951349	0.132493717
pcaec403.pcaec343	0.083081332	0.054911336	0.132580919
pcaac320.transhydroxyproline	-0.031097089	0.020556096	0.13263606
pcaac386.pcaec300	0.014497485	0.009584911	0.132701981
smc181.pcaec321	-0.084783215	0.056054604	0.132707272
serine.phenylalanine	-0.04662651	0.030844308	0.132920307
c18.pcaac281	-0.002371207	0.00156862	0.13292585
pcaec360.pcaac402	0.076185011	0.050399934	0.132936523
pcaec322.arginine	-0.063734866	0.042165101	0.132950366
lysopcac281.lysopcac180	0.0742778	0.049144162	0.13298298
leucine.pcaec401	0.096603413	0.063920063	0.133011161
glutamine.pcaac384	-0.100382112	0.066427164	0.133050717
putrescine.c2	0.056091453	0.037132361	0.133198212
smc261.pcaec300	-0.015836786	0.010484465	0.133218922
c141.spermidine	0.080356274	0.053210207	0.133303936
lysopcac180.pcaec443	-0.092814129	0.061497006	0.133538684
pcaac320.pcaec383	-0.1014004	0.067187959	0.133549245
c10.c141	0.079451547	0.05264717	0.133567783
lysopcac170.smohc161	-0.018928814	0.012544644	0.133623012
pcaac365.c2	0.074458731	0.049371618	0.133824345

smohc221.pcaac404	0.013007387	0.008625484	0.13385247
pcaac321.pcaac403	0.005461767	0.003622096	0.133882146
prolinr.pcaac281	0.001382831	0.000917069	0.133887625
pcaec362.pcaec444	-0.118930624	0.0788993	0.134017727
glutamicacid.spermidine	-0.077870428	0.051669373	0.134089859
pcaac321.pcaec405	-0.082498757	0.054745158	0.134123349
glutamine.pcaec380	-0.064726541	0.042954352	0.134146868
c142.pcaac406	0.046547513	0.030894268	0.13419724
c101.pcaac341	0.007900559	0.005244018	0.134219378
c181.transhydroxyproline	-0.02726724	0.01810052	0.134258091
histidine.c2	-0.092465521	0.061380647	0.134259664
smohc241.pcaac381	-0.02589788	0.017199523	0.134438035
pcaec301.pcaac425	-0.088331285	0.058684134	0.134575063
smc241.c51dc	-0.130537297	0.086738942	0.134640362
pcaec423.pcaec382	-0.023541407	0.015642987	0.134647289
pcaec343.pcaac404	0.008371026	0.005563168	0.134697281
pcaec341.prolinr	0.050458857	0.033539826	0.134768489
pcaec382.c5	-0.029938276	0.019908511	0.134936503
c2.spermidine	0.079538846	0.052905793	0.135036294
pcaec386.c51dc	-0.14092084	0.093777574	0.135214478
lysopcac281.pcaec445	0.042948122	0.028582039	0.13523701
c181.pcaac365	0.042319069	0.028163554	0.135239084
serotonin.c141	-0.037662581	0.025066382	0.135266036
pcaac342.pcaac385	-0.04401475	0.029296426	0.135297109
c181.pcaac426	0.076000621	0.050591429	0.135335839
pcaac383.pcaac380	-0.054448752	0.036250604	0.135396087
pcaec301.pcaac384	0.066412293	0.044215969	0.135399073
h1.pcaac344	0.09733183	0.064802495	0.135404584
pcaac420.c141	-0.03660114	0.024373004	0.135473851
creatinine.serotonin	0.088675417	0.059077031	0.135653308
ornithine.pcaac365	-0.029430064	0.019610327	0.135723177
pcaac426.pcaec363	0.047171696	0.031443526	0.135862678
smc180.c181	0.014909909	0.009938916	0.135875434
pcaac424.asparticacid	0.075867166	0.05057502	0.135891686
phenylalanine.pcaec424	-0.074016276	0.04934285	0.135904913
pcaec443.spermidine	-0.075247466	0.050176015	0.13600083
pcaac321.pcaac240	-0.019910781	0.013280101	0.136098126
pcaec341.pcaec300	0.016049	0.010708174	0.136236206
pcaac363.pcaac281	0.006652028	0.004438612	0.136259214
glutamine.pcaec322	-0.049475471	0.033015316	0.136288199
pcaec406.pcaec360	-0.00744308	0.004967643	0.136352755
creatinine.pcaac363	0.025212904	0.016828915	0.136384618
asparticacid.smohc161	0.029068559	0.019403958	0.136414681
pcaec446.lysopcac170	0.022371374	0.014933481	0.136416099
arginine.lysopcac280	0.024810122	0.01657207	0.136666412

pcaac404.lysopcac280	0.00811672	0.005422691	0.136743914
pcaac422.pcaac343	0.017956159	0.011997041	0.136767541
smc180.lysopcac280	0.011867319	0.007929409	0.136792193
pcaac426.pcaec444	0.184485616	0.12329566	0.136879224
pcaac300.smohc222	0.005887803	0.00393555	0.136939183
smc160.pcaac425	0.094191287	0.062962484	0.136956206
pcaac424.c181	0.022813922	0.015250899	0.136978065
pcaec444.pcaac404	0.00456132	0.003049215	0.136979802
c10.lysopcac181	0.105019298	0.070214291	0.137032704
methionine.pcaec364	-0.012791286	0.008552948	0.137073252
c51.pcaec361	-0.010307259	0.006892367	0.137094407
lysopcac181.pcaec363	0.029195267	0.019528471	0.137211601
pcaac404.pcaec444	0.105987849	0.070894714	0.137213399
pcaec425.pcaec300	0.013323037	0.008913515	0.137292381
pcaac401.pcaec424	-0.06283018	0.042038401	0.137321226
pcaec406.pcaec443	0.071371296	0.047762143	0.137395164
pcaac320.serotonin	0.120923816	0.080931263	0.137434909
arginine.smohc222	-0.012475836	0.008352123	0.137545205
prolinr.pcaec300	-0.076573706	0.051281191	0.137681077
c2.lysopcac204	0.043082401	0.028861177	0.137803079
lysopcac170.pcaec404	0.02507629	0.016802074	0.137879943
lysopcac204.pcaec363	0.025605309	0.017158559	0.137925977
pcaec404.pcaec402	-0.138243643	0.092644779	0.137948465
pcaac281.smohc221	0.038476943	0.025787423	0.137976901
methionine.pcaac425	-0.081389689	0.054547844	0.137977635
lysopcac170.pcaec406	-0.050000518	0.03353151	0.138221023
citruline.c5	-0.011454016	0.007681456	0.138227423
smc181.lysopcac280	0.016746444	0.011233135	0.138310868
pcaec424.smc261	0.068539674	0.045987056	0.138414631
pcaac321.spermidine	0.079563896	0.053400208	0.13853477
pcaac384.c18	-0.049490069	0.033219434	0.138577366
pcaac381.smohc221	-0.057963657	0.038911493	0.13862059
lysopcac180.prolinr	-0.049433801	0.033187026	0.138640614
pcaec364.pcaec445	0.066943144	0.044958146	0.138783052
pcaec444.smc181	0.023712765	0.015925341	0.138786891
lysopcac204.lysopcac161	-0.092687843	0.062259288	0.138854394
pcaac421.pcaec385	-0.072302992	0.048570403	0.138885126
pcaac384.smc181	0.063648785	0.042758911	0.138904145
c51.pcaec385	-0.095164548	0.063933002	0.138916229
pcaec446.pcaec443	0.03872032	0.026013025	0.138918048
pcaac380.kynurenine	-0.010998269	0.007389219	0.138938129
pcaac341.pcaac300	-0.160028328	0.107584324	0.139189039
pcaec425.pcaec343	0.103036454	0.069276766	0.139229293
pcaac405.c51dc	-0.141852229	0.095381427	0.139257248
c12.c141	0.11433901	0.076920273	0.139454887

pcaec341.pcaec445	0.048882301	0.032892214	0.13954083
lysopcac281.c141oh	0.105305395	0.070860294	0.139550642
pcaec421.pcaac281	-0.004754765	0.003201243	0.139765201
citruline.pcaec385	0.096352626	0.064877203	0.139800476
pcaac321.pcaec403	0.079463976	0.053517868	0.139890907
c5.pcaec382	0.027105529	0.018256027	0.139908894
pcaec343.pcaac365	0.049055122	0.033045925	0.139985983
pcaac364.pcaac385	-0.04336593	0.029225623	0.140150375
pcaac405.lysopcac280	0.005338247	0.003597963	0.140189244
c51dc.pcaac322	-0.085057762	0.057329654	0.1401959
pcaac362.pcaec384	-0.057047189	0.038452435	0.140217848
lysopcac161.c51dc	-0.169097579	0.114014338	0.140337883
pcaac386.c18	-0.049494461	0.033376477	0.140393911
lysopcac204.pcaec444	-0.110010378	0.074187605	0.140406414
pcaac364.smc161	-0.050204432	0.03385671	0.14041095
pcaec386.pcaec404	-0.012636982	0.008524835	0.14053779
pcaac341.c162	-0.086660478	0.058473863	0.140626748
pcaac240.lysopcac182	0.095439301	0.064409612	0.14070179
lysopcac161.lysopcac160	-0.029272204	0.019759752	0.140794924
acetylornithoine.pcaac402	0.038693811	0.02612796	0.140920099
pcaec405.pcaec343	0.095017562	0.06416334	0.140937424
pcaac380.pcaec404	-0.013710703	0.009258599	0.140939676
pcaec342.arginine	-0.055619751	0.037566462	0.141017474
phenylalanine.pcaec363	0.10995312	0.074268161	0.141039146
c162.pcaec382	-0.011834613	0.007994268	0.14106595
pcaec403.pcaec405	0.206791812	0.139689863	0.141072237
pcaec342.smc241	0.106178021	0.071743372	0.141177245
smc240.pcaac425	0.109481024	0.073977106	0.141187539
pcaec385.tyrosine	-0.070910188	0.047925248	0.141275891
pcaac384.pcaac402	0.055574576	0.037565448	0.141327411
serine.pcaac406	0.033640527	0.022749017	0.141497802
pcaec425.c141oh	0.10193314	0.068938701	0.141541323
methionine.pcaac341	-0.016681955	0.011283358	0.141581094
c2.pcaac240	-0.02453238	0.016593372	0.141584325
pcaac320.pcaac402	-0.074966848	0.050710339	0.141613688
pcaec384.pcaec404	-0.018679684	0.012636216	0.14163212
pcaac362.glycine	-0.042188612	0.02854161	0.141664604
lysopcac182.pcaec384	-0.051241252	0.034672007	0.141733778
pcaec424.pcaec404	-0.036467868	0.024676388	0.141744667
pcaac362.pcaac240	-0.020152686	0.013639884	0.141841616
smohc221.lysopcac161	0.11403992	0.077193737	0.141884785
pcaac425.pcaac402	0.071747652	0.048574658	0.141954799
pcaac365.pcaac425	-0.133145068	0.090148326	0.141982595
pcaec384.arginine	-0.045081519	0.03055679	0.142416885
smc202.pcaac364	0.043136468	0.029239493	0.142431545

methionine.lysopcac161	0.067342496	0.045654484	0.142494429
lysopcac181.smohc221	-0.075741758	0.051350798	0.142510473
pcaac366.pcaec404	-0.025166147	0.017061998	0.142511696
c3.pcaac405	0.093262414	0.063253075	0.142659329
serotonin.pcaec301	0.122087398	0.082820235	0.142742039
asymmetricdimethylarginine.pcaec3(	0.003063753	0.002078463	0.142762901
phenylalanine.c0	0.001235065	0.000838003	0.142824249
glutamicacid.pcaac384	0.061679323	0.041851673	0.142840263
smc202.prolinr	0.016978602	0.011520895	0.142850372
c182.pcaec341	0.035155171	0.023856684	0.142883751
c182.smohc221	0.019386079	0.013155895	0.142892472
c141oh.pcaec300	0.023962289	0.016263652	0.142946657
c18.smc160	0.080404279	0.054601894	0.143164793
glutamicacid.pcaac343	0.03189512	0.021662981	0.14322459
pcaac364.pcaac341	-0.036447314	0.024755427	0.14323457
asparagine.pcaac386	0.029600649	0.020105113	0.143234926
pcaac402.spermidine	-0.073564771	0.049967653	0.143247518
pcaac380.pcaac404	0.011323227	0.00769203	0.143294739
c141.c14	0.071992142	0.048906277	0.143302767
creatinine.pcaac321	-0.033575625	0.022811534	0.143349278
alanine.pcaac420	0.122100564	0.082963716	0.143385985
pcaec361.smohc161	-0.024816361	0.016864254	0.14343949
pcaac360.pcaac404	0.020922157	0.014221102	0.143528893
pcaac323.pcaec343	0.105773488	0.071905836	0.143584269
asparticacid.pcaac425	0.107993171	0.073417635	0.143599623
alphaaminoadipicacid.pcaac402	-0.071211014	0.048419844	0.143665608
methioninesulfoxide.smc161	0.110689668	0.075272542	0.143714375
pcaec425.pcaac323	-0.088639704	0.060287058	0.143775053
pcaec384.lysopcac204	-0.042512574	0.028914928	0.143783215
pcaac420.pcaac343	0.031136411	0.021185429	0.143933386
pcaac384.pcaec404	-0.020682354	0.014073321	0.143958951
pcaac363.pcaac384	-0.121314305	0.082565857	0.144043085
lysopcac181.pcaac381	-0.055774179	0.037964362	0.144092959
pcaec341.pcaec405	-0.050255213	0.03421024	0.144122363
pcaec360.kynurenine	-0.014588974	0.009932274	0.144167202
pcaac344.smohc161	0.019021834	0.01295684	0.144371411
c10.c181	0.037113084	0.025280763	0.144386474
lysopcac140.methionine	-0.038369493	0.026137555	0.144400924
kynurenine.valine	0.098327942	0.067002902	0.144527294
kynurenine.c8	0.051935841	0.035406931	0.144714895
pcaec322.c51dc	-0.152910743	0.104266202	0.144792414
c14.pcaac403	-0.005984753	0.004080892	0.144795434
pcaec380.glycine	0.054862627	0.03741052	0.144803058
c51dc.acetylornithoine	0.151557621	0.103357761	0.144847281
pcaac426.pcaac384	0.082392389	0.056189827	0.144852133

smc260.histidine	0.053612026	0.036562318	0.144852961
alanine.prolinr	0.072014661	0.049138922	0.145067162
pcaac403.c141	-0.078627726	0.053652254	0.145074097
prolinr.pcaec404	-0.051050353	0.034843193	0.145172366
pcaec363.alphaaminoadipicacid	0.024805301	0.016930765	0.145184202
pcaec383.c141oh	0.122399991	0.083551738	0.145222833
acetylornithoine.pcaec342	-0.087626766	0.059826395	0.145298168
c14.smc260	0.046863171	0.03199976	0.14535242
pcaac323.pcaec342	0.103649894	0.070786237	0.145412114
smc260.pcaac420	0.09175549	0.062666527	0.14543382
pcaac321.c141	-0.040902809	0.027936673	0.145450369
citruline.pcaac363	0.016135816	0.011021208	0.145465742
smc180.spermidine	0.077718231	0.053090032	0.145513394
prolinr.smc160	-0.082333992	0.056267555	0.145687118
pcaac380.c18	-0.042377838	0.028964409	0.145730383
lysopcac280.pcaac364	0.078365471	0.053567542	0.145777414
c181.pcaec383	0.108358529	0.074086151	0.145866692
pcaac343.pcaac405	0.048568187	0.03320797	0.145881897
kynurenine.pcaac404	-0.008497656	0.005810417	0.145898242
pcaec382.pcaec343	0.06881462	0.047065416	0.146002321
pcaac320.pcaec300	0.038979167	0.026663252	0.146057023
asparticacid.pcaec300	0.018249122	0.012487541	0.146199299
smc202.arginine	-0.031768004	0.021738718	0.146207627
pcaec362.smc241	0.076853494	0.052592848	0.14622522
lysopcac182.smc261	0.038651007	0.026451526	0.146249967
asymmetricdimethylarginine.pcaec36	-0.027629994	0.018911612	0.146303288
smc161.prolinr	0.0209077	0.014317384	0.146496642
pcaec445.prolinr	0.029735454	0.020363345	0.14651245
pcaec446.smohc221	0.022988561	0.015743339	0.146522143
isoleucine.pcaec401	0.111382621	0.076282445	0.146542537
pcaac421.lysopcac280	0.009376012	0.006421908	0.146578277
citruline.pcaec403	0.043564948	0.029864639	0.146923686
pcaec364.pcaac406	0.099910235	0.068497188	0.146963116
isoleucine.spermidine	-0.078651674	0.053940353	0.14709527
c18.smohc161	-0.015778342	0.010823764	0.147197965
pcaac425.pcaec383	-0.118606577	0.081369046	0.147228939
c142.pcaec422	0.028024117	0.019227671	0.147269895
pcaec360.pcaec404	0.03599579	0.024701066	0.147333912
pcaac341.pcaac403	0.007425198	0.005095687	0.147362292
lysopcac181.c2	0.064250043	0.044096484	0.147395446
smohc161.pcaec322	0.109798259	0.075366243	0.147442491
pcaac381.smohc161	-0.044297973	0.030415615	0.147563984
pcaec406.c2	0.070008991	0.048071395	0.147582637
pcaec384.smc181	0.018541162	0.012731926	0.14760509
pcaec422.c141oh	0.108078945	0.074221695	0.147635256

creatinine.lysopcac204	-0.028452399	0.01954798	0.147814106
pcaac365.spermidine	0.084546311	0.058091174	0.147844195
pcaec423.c141	-0.050995904	0.035041798	0.147877154
pcaac424.pcaac402	-0.189015164	0.129882166	0.147878809
c12.smc181	-0.025618373	0.017610434	0.148032208
smc160.c5	-0.00872162	0.005995414	0.1480355
pcaac406.pcaac323	-0.130872966	0.090001304	0.148199332
pcaac343.pcaac300	-0.113219173	0.077862681	0.148209082
c18.pcaac406	0.091636987	0.063027167	0.148253207
pcaec342.c51dc	-0.139038707	0.095639414	0.148294182
pcaec300.histidine	0.075826409	0.052167483	0.148366694
pcaec343.pcaac385	-0.029554394	0.020345858	0.148621378
pcaac365.tyrosine	0.058180082	0.040054122	0.148638934
pcaec322.pcaec384	-0.072495238	0.049910476	0.148647603
histidine.pcaec363	0.014861468	0.010232583	0.148685957
asymmetricdimethylarginine.pcaec42	0.014192235	0.009772023	0.14869537
lysopcac161.pcaac403	0.011980754	0.008249489	0.148703906
pcaac322.pcaac240	-0.024335805	0.016757894	0.148732518
lysopcac281.pcaac404	0.005400374	0.003719066	0.148766193
pcaec445.arginine	-0.04043492	0.027846809	0.148774402
histidine.pcaec444	-0.140063561	0.09646004	0.148777581
arginine.alanine	0.055046638	0.037910892	0.148787729
h1.c0	-0.001295961	0.000893181	0.149079778
serotonin.pcaac402	0.025396125	0.017503826	0.149096366
pcaec386.c18	-0.044811313	0.030886547	0.149111367
c101.lysopcac181	0.058684108	0.040450522	0.149131744
pcaac281.pcaac365	-0.050965443	0.035131898	0.14915236
c101.pcaec300	0.014026474	0.009670187	0.149208629
pcaec421.lysopcac181	0.080869073	0.05576175	0.14927143
pcaac240.spermidine	-0.078506762	0.054133417	0.149275564
c12.pcaac384	0.066923793	0.046148717	0.149294819
arginine.pcaac406	0.055162897	0.038039489	0.149302732
pcaec443.pcaec321	0.038854679	0.02680321	0.149447661
c182.c102	0.0619804	0.042763421	0.149517036
pcaac420.pcaec385	-0.067418439	0.046521518	0.149570163
asparticacid.pcaac364	0.080769174	0.055737103	0.149592081
serine.acetylornithoine	-0.092023182	0.063507415	0.149618546
c102.transhydroxyproline	-0.029519211	0.020373852	0.149656887
isoleucine.pcaec340	0.042692911	0.029478795	0.14982958
pcaac383.asparagine	0.011006603	0.007599994	0.149835152
citruline.pcaac362	-0.050043991	0.034556807	0.149855379
smc241.pcaec300	-0.02637549	0.018216392	0.149930007
alphaaminoadipicacid.c14	-0.036045996	0.024896858	0.149953919
pcaac422.pcaac365	0.086540915	0.05978122	0.150005873
pcaec301.pcaac405	0.032175268	0.022226759	0.150015812

arginine.smc202	0.104150713	0.07198594	0.150231462
pcaec300.pcaec341	-0.115320067	0.079708115	0.150242849
smc241.c141	-0.035710643	0.024689266	0.150348036
pcaec404.pcaec343	0.071772437	0.04962663	0.150391465
isoleucine.pcaec300	-0.012339596	0.008534867	0.150520824
glycine.pcaac406	0.04750453	0.032857721	0.150526968
glutamine.c181	-0.064994772	0.044960246	0.150571595
smc241.spermidine	0.073437116	0.050810348	0.150652093
c0.smohc161	0.018691657	0.01293298	0.150665371
methionine.lysopcac204	-0.028822226	0.019943337	0.150683587
pcaec406.pcaec364	0.032577376	0.022545622	0.150754328
putrescine.pcaac365	0.035994052	0.024910407	0.150758028
pcaac421.c18	-0.041703153	0.028865269	0.150810887
pcaec445.smohc221	0.031079094	0.02151299	0.150834862
pcaec446.pcaec404	-0.009292077	0.006433213	0.150912131
c10.pcaac343	0.02162507	0.014972038	0.150919796
lysopcac170.spermidine	-0.078350727	0.054250977	0.150958205
c2.pcaec384	0.065366939	0.045261343	0.150962644
lysopcac203.c18	0.052924266	0.036649251	0.151000904
pcaac362.pcaec360	0.006462566	0.004479069	0.151348895
kynurenine.smohc221	0.018739016	0.012989289	0.151401111
pcaac421.lysopcac180	0.079598942	0.055176867	0.151411517
smohc241.glycine	0.052009476	0.03605334	0.151423935
pcaac300.pcaac405	0.037078487	0.025717902	0.151658196
pcaec341.c51dc	-0.121988992	0.08461837	0.151686702
pcaec343.lysopcac281	0.050681929	0.035156372	0.151693155
pcaec402.lysopcac161	0.053513547	0.037121695	0.151705459
lysopcac281.acetylornithoine	-0.08072971	0.056020703	0.151846849
lysopcac203.transhydroxyproline	0.045742929	0.031744897	0.151879407
asparticacid.pcaec342	0.104464912	0.072497472	0.151881714
pcaec385.histidine	0.060543207	0.04201826	0.151900641
pcaac426.pcaac405	-0.030526164	0.021192422	0.152027688
glutamine.pcaec422	0.042352079	0.029404157	0.152051704
pcaec385.smc160	0.170884972	0.118642696	0.152054639
c0.pcaec341	0.046118724	0.032026136	0.152139042
putrescine.pcaac403	-0.006335578	0.004399776	0.152155025
pcaac341.pcaec403	0.071846473	0.04989602	0.152170127
pcaac386.pcaec364	-0.03845389	0.026707606	0.152202195
pcaec443.pcaac402	-0.079719926	0.055369183	0.152208329
acetylornithoine.pcaec382	-0.012003061	0.008337152	0.152231267
pcaac405.smohc161	0.014027122	0.00974515	0.152319564
c2.smc160	0.068848307	0.047838451	0.152379427
pcaec301.pcaec445	-0.04668461	0.032445858	0.152474745
smc180.pcaec343	0.073690215	0.051224524	0.152552153
pcaec322.glutamine	-0.025588935	0.017789504	0.15259295

lysopcac204.pcaec300	-0.009039976	0.006285328	0.152638918
smc161.c14	-0.028792801	0.020029219	0.152844784
pcaec445.pcaec363	-0.086696508	0.060311325	0.15286089
pcaac421.acetylornithoine	-0.100358807	0.069835685	0.152977869
glycine.pcaac366	0.060183107	0.041879854	0.152985987
lysopcac203.c51dc	-0.16986639	0.118229308	0.153067787
pcaac383.leucine	0.015688876	0.010920441	0.153096592
lysopcac180.smc261	0.061216405	0.042625069	0.153236421
h1.pcaec445	0.091804103	0.063927583	0.153263586
serotonin.kynurenine	-0.017581634	0.012245726	0.153356696
smc260.pcaec300	-0.016353401	0.011395156	0.153532347
lysopcac204.c14	0.04797716	0.033432286	0.153550511
c142.spermidine	0.075231007	0.052432459	0.153618014
asymmetricdimethylarginine.methior	-0.030224575	0.021067866	0.153671612
arginine.pcaac365	-0.060582436	0.042236029	0.153743073
pcaec401.lysopcac280	0.013649074	0.009522224	0.154024252
pcaec444.pcaac380	-0.070083479	0.048898231	0.154064047
c3.pcaec361	-0.009235578	0.006443891	0.154070235
kynurenine.transhydroxyproline	-0.020184963	0.014086152	0.154145865
histidine.smc181	0.038533767	0.026904656	0.154354508
alanine.c3	0.054971289	0.038397512	0.154525216
spermidine.lysopcac280	-0.0609234	0.042563117	0.154602613
pcaec406.pcaec382	-0.018696423	0.013068659	0.154813062
pcaec386.pcaac384	-0.068855388	0.048136045	0.154869604
pcaec425.c141	-0.045639649	0.031907033	0.154880587
pcaec425.lysopcac280	0.016664223	0.011651608	0.154934064
pcaac364.pcaec360	0.005277441	0.003690385	0.154978886
pcaec406.pcaac405	-0.049289802	0.034468238	0.154991796
pcaec406.pcaac380	-0.140931873	0.098569564	0.155059352
c16.c5	-0.019991374	0.013987782	0.155222244
smc181.pcaac384	-0.075276749	0.052679117	0.155289683
lysopcac280.citruline	-0.027148055	0.019001642	0.155360285
pcaec341.pcaac322	0.090191193	0.06312756	0.155362614
c181.pcaec380	-0.035547875	0.024881909	0.155376951
smc160.lysopcac180	0.082751321	0.057983124	0.155808401
lysopcac140.lysopcac180	0.04537407	0.031795336	0.155835822
pcaec401.pcaec363	0.031245038	0.021894652	0.155837065
lysopcac140.tyrosine	0.056941269	0.039903527	0.155862723
pcaec405.c162	0.082447032	0.057793677	0.1559773
pcaec384.pcaec382	-0.037825759	0.026515164	0.155978598
pcaec341.smohc221	0.05124731	0.035925768	0.156005374
pcaec321.pcaac384	-0.077465156	0.054314438	0.156075281
phenylalanine.pcaec360	0.006440535	0.004515895	0.156087574
pcaac365.pcaac362	-0.061502709	0.043127018	0.156119075
pcaec386.pcaec342	-0.196365561	0.137707527	0.156154249

lysopcac140.pcaac361	-0.078801887	0.055270658	0.156216382
lysopcac160.pcaec384	-0.049087909	0.034432625	0.156251955
lysopcac160.arginine	0.073936711	0.051888393	0.156454995
pcaac364.smc160	0.100969483	0.070862823	0.156472257
histidine.glycine	0.040292353	0.028278755	0.156481092
smc261.pcaac420	0.091049624	0.063918734	0.156587507
pcaac342.smohc221	0.030424268	0.021359886	0.156614782
pcaec365.pcaac323	-0.137324579	0.096416259	0.156636755
pcaec364.smc161	-0.058587333	0.041136487	0.156657099
pcaec360.pcaec382	-0.010437942	0.007329846	0.156710622
c102.prolinr	0.038179276	0.02681278	0.156743021
pcaec322.pcaac322	0.068084584	0.047820785	0.156793951
c141.pcaac341	0.011492908	0.008072405	0.156798776
smc202.smohc241	0.114436328	0.080387236	0.15684634
c14.pcaac365	0.039349665	0.027644657	0.156890875
serotonin.smohc161	-0.007518136	0.005282285	0.156930236
pcaec405.pcaec380	0.096641148	0.067908611	0.156978662
smohc161.citruline	0.022361513	0.015713676	0.156991789
lysopcac170.pcaac381	-0.044551052	0.03131635	0.157121283
histidine.pcaac406	0.034985969	0.024595132	0.15716119
pcaec322.pcaec445	0.089095554	0.062636534	0.157176785
pcaec444.pcaac240	-0.01863213	0.013099143	0.157185024
pcaec444.pcaac420	0.168180161	0.118253377	0.157240441
pcaec386.smc180	-0.028743826	0.020217653	0.15738029
pcaec362.pcaec300	0.006210934	0.004368611	0.157380558
c18.lysopcac204	0.038893213	0.027363338	0.15748383
lysopcac182.tyrosine	-0.070713464	0.049755829	0.157528064
pcaec406.pcaac403	-0.008217787	0.005782526	0.157547901
prolinr.glycine	-0.020476843	0.01441046	0.157597385
c141oh.valine	-0.071383552	0.050235924	0.157598692
pcaec424.arginine	-0.074476721	0.052423893	0.15768652
pcaec444.smc261	0.029777747	0.020970682	0.157887904
c3.pcaec322	0.087414643	0.061565599	0.157919464
asymmetricdimethylarginine.smc240	-0.035223853	0.02480967	0.157948165
kynurenine.c5	-0.009867764	0.006950805	0.157978784
lysopcac280.lysopcac281	0.030945938	0.021804814	0.158104641
lysopcac280.smohc221	-0.028004174	0.019734461	0.158155767
histidine.pcaec322	-0.059291212	0.041788184	0.158213448
smohc222.pcaec360	-0.005714482	0.004028204	0.158281378
c142.pcaac420	0.120401011	0.084883941	0.158339214
pcaac344.pcaec424	-0.008275047	0.005835618	0.158454323
lysopcac204.lysopcac170	0.029239218	0.020622996	0.158520567
smc161.pcaac365	-0.05648099	0.039847368	0.158626534
pcaec343.c14	-0.031306114	0.022089592	0.158684783
alphaaminoadipicacid.valine	0.078177354	0.055170484	0.158748864

pcaec340.kynurenine	-0.008376827	0.005913214	0.158861429
pcaac386.tyrosine	0.063232542	0.044645078	0.158946123
ornithine.pcaec364	0.021805533	0.015396449	0.15896605
pcaac403.pcaec343	0.099759014	0.07045721	0.15907985
pcaac320.pcaac386	0.055742204	0.039379004	0.159182107
pcaac363.smc161	-0.064697996	0.045709736	0.159217657
pcaec301.citruline	-0.009663896	0.006835428	0.159690325
pcaac343.pcaac402	0.077260209	0.054669512	0.159858197
lysopcac281.pcaec342	0.118474322	0.083833592	0.159862521
spermidine.c12	0.202395676	0.143231396	0.159903721
serotonin.pcaec405	-0.058238577	0.04122427	0.160004206
c2.arginine	0.072669758	0.051449751	0.160087628
asymmetricdimethylarginine.smc261	0.0428534	0.030362386	0.160394418
smc180.prolinr	0.055538582	0.039358168	0.16047996
c182.lysopcac160	0.012649609	0.008964382	0.16048301
pcaec425.pcaec404	-0.02477428	0.017562893	0.160628119
pcaac366.lysopcac181	0.045236803	0.032070998	0.160652562
pcaac404.asparticacid	0.03710114	0.026308845	0.160742441
glycine.c162	0.077395172	0.054892739	0.160825206
pcaec361.pcaec343	0.112686247	0.079930091	0.160861914
citruline.pcaec360	0.00797786	0.005659674	0.160924667
smohc222.prolinr	0.047067441	0.033393493	0.160959421
pcaac300.pcaac402	-0.041659821	0.029559975	0.16100287
smohc221.pcaec445	0.03924302	0.027847867	0.161043893
pcaec363.pcaec404	-0.010767322	0.007641167	0.161065456
smohc141.pcaec364	-0.044705169	0.031728688	0.161105994
pcaac403.spermidine	-0.070635424	0.050141553	0.161183419
smohc222.c141	-0.030991267	0.022001064	0.16121131
smc181.c0	-0.003008756	0.002136467	0.161311779
glycine.valine	-0.06864545	0.048745205	0.161322228
pcaec364.pcaac366	-0.044569796	0.031650518	0.161341421
prolinr.pcaac322	0.059113142	0.041981888	0.161377585
pcaec406.c141oh	0.130792292	0.092890353	0.161387685
pcaac365.c51dc	-0.157615126	0.111954737	0.161441462
pcaec340.c0	-0.000603463	0.000428747	0.161542842
pcaec422.pcaac402	0.118349279	0.084098495	0.161612093
smohc161.prolinr	0.050179991	0.035658594	0.161622666
citruline.c14	-0.039473784	0.028050807	0.161625505
smc261.pcaac344	-0.087657145	0.062298325	0.161675789
pcaec363.pcaec300	0.004835105	0.003436408	0.161685176
glutamicacid.pcaec444	0.132281657	0.094038182	0.161786592
pcaac343.c162	-0.082602296	0.058723994	0.161804696
pcaec382.pcaec363	0.049808377	0.035415829	0.161873316
pcaac402.smohc221	-0.09182119	0.065292741	0.161899187
c2.lysopcac180	0.101047132	0.071859804	0.161937611

smc180.lysopcac170	0.019088584	0.013575005	0.161941688
smc261.pcaac404	0.006529935	0.00464622	0.16215724
c51dc.pcaac363	0.041642686	0.029633161	0.162203651
pcaec444.histidine	0.054803759	0.039002259	0.162242205
c162.lysopcac204	0.031815503	0.022643126	0.16225965
pcaac405.arginine	0.029263179	0.020828653	0.162300069
creatinine.kynurenine	-0.026142966	0.018610793	0.162367613
pcaec406.pcaac381	-0.029302617	0.020860827	0.162382119
pcaec340.smc160	0.106558597	0.075872831	0.162451912
histidine.pcaec361	0.007687935	0.005474271	0.162469989
c51dc.pcaac406	0.072763723	0.051814929	0.16249245
pcaac405.pcaec385	0.109522653	0.077998525	0.162533217
glutamicacid.c18	0.1155032	0.082265816	0.162574489
c2.pcaac422	0.090310424	0.064324771	0.162588878
smohc222.c181	0.016329037	0.011633629	0.162698682
lysopcac182.pcaac320	-0.088249031	0.062903161	0.16289852
smc240.smc261	0.043465865	0.030984478	0.162930568
pcaac322.pcaac405	0.034059083	0.024281641	0.16297791
lysopcac281.c2	-0.094767814	0.067574685	0.163053003
methionine.pcaec363	0.028109596	0.020043996	0.163059286
c12.alanine	0.091201178	0.065044851	0.163138912
pcaac361.pcaec446	-0.036105149	0.025774066	0.163525155
glycine.smc261	0.017828885	0.012729687	0.163601966
pcaac386.glycine	0.028971019	0.02068542	0.16360872
pcaac240.smohc221	0.146441688	0.104580832	0.163692323
lysopcac280.methionine	-0.038409394	0.027430874	0.163706591
prolinr.pcaec360	0.004957063	0.00354145	0.163855422
pcaec365.alanine	-0.027264095	0.01947901	0.163873867
c0.kynurenine	0.017207157	0.012296068	0.163952273
smohc241.pcaec342	-0.101098231	0.072244445	0.163955882
pcaec322.pcaac420	0.102318309	0.073116617	0.163957652
lysine.smc261	0.055267654	0.039498196	0.163999575
asparagine.pcaac421	0.046903379	0.033526725	0.164077426
pcaec385.alanine	-0.019939763	0.014253503	0.164091519
pcaec361.pcaec360	-0.009845622	0.007039935	0.164211018
asparticacid.kynurenine	-0.012242636	0.008756235	0.164323795
lysopcac161.pcaec406	-0.034757902	0.024860041	0.1643294
pcaac364.smc240	0.031519579	0.022544336	0.164337861
serine.lysopcac280	0.016293004	0.01165401	0.164354445
pcaac406.lysopcac161	-0.094298468	0.067459073	0.164413768
pcaec422.lysopcac280	0.011253945	0.00805313	0.164533593
pcaec421.pcaac343	0.021669828	0.015506663	0.164536461
pcaec445.lysopcac170	0.015379738	0.011006696	0.164580013
smc180.pcaec445	0.050972986	0.03647972	0.1645833
smc240.pcaac381	-0.024002164	0.017178955	0.164617001

lysopcac160.pcaac406	0.041457544	0.029672842	0.164625926
pcaac323.c18	-0.020745586	0.014851415	0.164709461
lysopcac140.c18	0.051274555	0.036707122	0.164715571
pcaac383.kynurenine	-0.009662779	0.006918304	0.164763073
smc261.pcaec364	0.014208394	0.010174331	0.164824125
pcaac420.pcaec342	-0.130337018	0.093337942	0.164852685
smc161.pcaec341	0.046096351	0.033012516	0.164873584
c18.glutamicacid	-0.044991778	0.032224003	0.164906673
methionine.smohc221	-0.031771077	0.02275554	0.164915256
alanine.pcaac381	0.015183024	0.010876414	0.164985028
prolinr.smohc161	-0.015536306	0.011130512	0.16502357
pcaac360.lysopcac203	-0.034387895	0.024636275	0.165025595
c0.pcaec360	0.006654101	0.004768148	0.165113393
smc241.lysopcac180	0.068826473	0.049321145	0.165130129
pcaac405.pcaac402	0.052023959	0.037297287	0.165319772
pcaec301.pcaac380	0.068168349	0.048882086	0.165409801
pcaec380.citruline	-0.019374673	0.013894272	0.165443113
pcaac405.smc240	0.032477235	0.023295482	0.165531356
smc261.smohc221	-0.059278052	0.042530323	0.165639812
c102.pcaac406	0.045706696	0.032795167	0.16566425
smohc161.pcaec425	-0.028660222	0.020564478	0.165672116
smc160.glutamine	-0.02430956	0.017443114	0.165680586
pcaac422.pcaac323	-0.088465334	0.063479579	0.16569414
smc240.smohc222	0.008888536	0.006379607	0.165793584
pcaec381.pcaac403	0.012640166	0.009072411	0.165799648
serine.pcaac405	-0.061372804	0.044059504	0.165890331
pcaac323.kynurenine	-0.004692295	0.003369	0.165940672
pcaac365.kynurenine	-0.012923886	0.009286019	0.166251327
pcaec404.c141oh	0.106527643	0.07655343	0.166315178
c16.spermidine	0.071615117	0.051473151	0.166386605
pcaac365.pcaec361	-0.022715938	0.016327608	0.166401948
histidine.pcaec422	0.052941915	0.038057124	0.166445013
glycine.pcaac426	-0.034211335	0.024595357	0.166490357
glycine.pcaec322	-0.03933886	0.028298243	0.166737189
pcaac323.pcaac364	0.042312704	0.030444474	0.166834176
lysopcac161.pcaec363	0.026975854	0.019414128	0.166936026
pcaec446.pcaac405	-0.065215126	0.046939684	0.166983703
c141.lysopcac180	0.063072287	0.045411693	0.167116935
lysopcac170.lysopcac180	-0.143768881	0.10351889	0.167141784
pcaec300.smc160	0.104049928	0.07493104	0.167205138
c2.valine	-0.078583135	0.056595052	0.167233415
pcaec443.acetylornithoine	-0.06131207	0.044157629	0.167243762
pcaac384.pcaac381	-0.034420555	0.02479444	0.167318203
c181.pcaec341	0.051759724	0.037288141	0.167359323
pcaec444.lysopcac180	0.051482838	0.037099829	0.167486204

pcaac385.pcaac403	-0.00875369	0.006309323	0.167565997
smohc141.pcaec361	-0.016045324	0.011565186	0.167578192
pcaec446.glycine	-0.071006301	0.051204155	0.167776631
pcaec382.arginine	-0.149382601	0.107727722	0.167795237
smc161.phenylalanine	-0.058304708	0.042059906	0.167928624
pcaac383.pcaec343	0.07493798	0.054060515	0.16794178
pcaac342.serotonin	0.127817914	0.092210909	0.167953816
pcaec406.pcaac425	0.10944353	0.078959674	0.167977814
lysopcac161.c2	0.091730514	0.066182772	0.167993244
pcaac405.spermidine	0.074923268	0.054056654	0.167994528
smc261.smc240	-0.025643714	0.01850723	0.168119065
pcaac281.pcaac381	-0.045637058	0.032942773	0.168198853
smc160.arginine	-0.04155973	0.030013299	0.168392007
pcaec365.c141oh	0.112615745	0.081332534	0.168415531
c102.pcaec423	-0.065083373	0.047008699	0.16845738
pcaec401.pcaac323	-0.102642418	0.074147133	0.168515109
smc241.pcaec321	-0.093337821	0.067428003	0.168529857
pcaec421.c51dc	-0.174380273	0.125994641	0.168600038
histidine.asparticacid	0.043693716	0.031588736	0.168851852
pcaec321.c14	-0.037965188	0.02744928	0.168883158
lysopcac281.phenylalanine	-0.070052866	0.050650931	0.168898866
lysine.pcaac404	-0.00541687	0.003916923	0.168933092
pcaec445.pcaec404	-0.012076684	0.008733099	0.168956504
pcaec423.smc261	0.040029824	0.028949857	0.168997495
pcaec301.pcaec382	-0.011671746	0.008441917	0.169038906
c141.smohc221	-0.052205788	0.037763781	0.16908918
pcaac341.lysopcac280	0.020541289	0.014859117	0.169097456
pcaac380.pcaac381	-0.037206157	0.026916019	0.169127392
pcaec402.transhydroxyproline	-0.03644629	0.026367367	0.169144376
lysopcac181.glycine	-0.015831046	0.011455443	0.169230985
smohc221.smc181	0.022542914	0.01632082	0.169454934
alphaaminoadipicacid.prolinr	0.033301904	0.024114237	0.169525704
pcaac321.lysopcac181	0.059041902	0.04275748	0.169571855
pcaec364.lysine	-0.090286373	0.065389687	0.169606291
c5.kynurenine	-0.013824496	0.010012418	0.169608801
pcaec405.pcaac300	-0.058605341	0.042455763	0.169715981
c102.pcaec301	0.118872764	0.086122999	0.169752412
leucine.c2	-0.046699262	0.033836881	0.169794896
pcaec386.pcaec321	-0.069600779	0.050436181	0.169841546
pcaec420.pcaac385	-0.065298739	0.047321061	0.169862621
pcaec444.pcaec360	-0.014879932	0.010784015	0.169891773
pcaac384.prolinr	0.036425532	0.026400203	0.169913087
pcaec321.prolinr	0.0229929	0.016666362	0.169957721
pcaec365.pcaac366	-0.051149237	0.037086775	0.170087448
alanine.phenylalanine	-0.058940475	0.042742269	0.170149996

pcaac421.pcaec342	-0.120836207	0.087654809	0.170281622
prolinr.pcaac420	0.124033682	0.089975922	0.170289445
c162.glycine	-0.030393497	0.022075074	0.17081234
pcaac402.acetylornithoine	-0.063740045	0.046296306	0.170824556
prolinr.pcaac381	0.022395615	0.016267576	0.170849873
pcaec386.smohc221	0.026471508	0.01923122	0.170916696
smc241.tyrosine	-0.067747472	0.049221701	0.170951306
glutamicacid.smohc221	-0.141569379	0.102880253	0.171048361
lysopcac170.prolinr	-0.056044616	0.040732028	0.171086959
pcaec362.smohc221	0.024765448	0.017999604	0.17110127
pcaec322.kynurenine	-0.007580537	0.005512793	0.171350868
c142.c181	0.024584789	0.017885702	0.171515249
smc240.pcaac404	0.004814272	0.00350385	0.171687107
pcaec364.arginine	-0.063908452	0.046517148	0.171726187
creatinine.lysopcac203	-0.014434945	0.010507168	0.171741639
c18.pcaec382	-0.00922409	0.006715712	0.171837693
pcaac404.c5	-0.012185881	0.008873687	0.171914796
histidine.pcaac425	-0.089196218	0.064959903	0.171965534
prolinr.citruline	0.021747772	0.015841761	0.172053551
isoleucine.pcaac406	0.045377524	0.033067605	0.172223504
pcaac344.pcaec383	-0.091822917	0.066942267	0.172407387
asparticacid.pcaec401	0.072964382	0.053202458	0.172477527
smohc221.pcaac366	-0.088753325	0.064723536	0.172533296
alphaaminoadipicacid.kynurenine	-0.006037708	0.00440335	0.172566163
lysopcac181.spermidine	-0.079908224	0.058290203	0.172657486
isoleucine.pcaac343	0.034914228	0.025477812	0.172810295
ornithine.smc261	0.050510016	0.036866914	0.17290813
pcaac322.prolinr	0.03495446	0.025514745	0.172936973
pcaac386.smc261	0.034076669	0.024875466	0.172961978
pcaec425.pcaec380	0.089026451	0.064989292	0.172970495
prolinr.pcaec405	0.077456384	0.056551929	0.173036785
pcaac365.pcaec384	0.053390182	0.038984857	0.173080356
pcaac240.pcaec343	0.1017064	0.074293194	0.173243726
pcaac405.pcaec361	-0.010817641	0.007904727	0.173394715
h1.alanine	-0.033526139	0.024500488	0.173431004
c4.pcaac300	0.053794222	0.039314046	0.173451568
pcaec301.c5	0.01190161	0.008698447	0.173475084
smc240.transhydroxyproline	0.036764987	0.026880949	0.173646419
serine.pcaec385	-0.049385739	0.036112715	0.173694045
c3.pcaac323	0.081097964	0.059306027	0.173723862
pcaec406.smc261	0.047349485	0.034640906	0.173906042
c142.pcaac403	-0.005487648	0.004015118	0.173943488
pcaac406.pcaac425	0.106180738	0.077697542	0.173992249
pcaec406.smohc221	0.027098826	0.01983084	0.174020886
pcaec301.c2	0.078428639	0.05739611	0.174037774

isoleucine.pcaec422	-0.042745493	0.031282434	0.174040164
c5.pcaec445	0.112385546	0.082261962	0.17411737
pcaec405.smohc221	0.06508186	0.047655671	0.174280911
pcaac366.pcaec422	0.042303471	0.030983005	0.174372448
c14.pcaec424	0.01620408	0.011872864	0.174553376
pcaac281.glycine	0.109017248	0.07988758	0.17460555
c141.c8	0.063546381	0.046571999	0.174654855
c141.c2	0.066938846	0.049070317	0.174759941
lysopcac204.smc240	0.045311042	0.033218804	0.174798705
pcaac344.c2	0.079683374	0.05843193	0.174899867
pcaac322.glycine	0.036244202	0.02658333	0.174987011
c5.lysopcac160	0.015829358	0.01161673	0.175233443
pcaac365.smc241	0.14830278	0.108933331	0.175619434
pcaac404.smohc221	0.065766009	0.048337351	0.175886079
pcaec405.pcaac381	0.041121399	0.030228153	0.175947715
pcaec300.pcaac420	0.09975376	0.073339311	0.176010808
c141oh.pcaac403	-0.004505422	0.003313957	0.176212329
c101.pcaac425	-0.089018443	0.065478684	0.176220812
prolinr.pcaac402	-0.032163183	0.023659933	0.176254965
pcaac320.c8	0.056313892	0.041431005	0.17630981
pcaac385.lysopcac161	-0.078405571	0.057693549	0.176379455
pcaec340.pcaac365	-0.072230525	0.053152085	0.176398451
lysopcac280.smohc161	0.01304886	0.009602832	0.176425358
creatinine.lysopcac180	-0.064496741	0.047471157	0.176489978
acetylornithoine.pcaec300	-0.01880647	0.013842315	0.176499255
smc181.pcaac364	0.051529851	0.037934757	0.176575445
pcaec423.lysopcac203	-0.024342181	0.01792582	0.176715116
smohc241.smc240	-0.037310601	0.027477126	0.176734436
smc180.c0	-0.000712366	0.000524714	0.176813695
smc202.pcaec404	-0.007566598	0.005573535	0.176824535
lysopcac281.pcaec380	0.063786649	0.046988805	0.176858756
smohc222.pcaec341	0.067914518	0.050030521	0.176866463
pcaac322.tyrosine	0.051837644	0.038188866	0.176885374
lysopcac204.asparticacid	0.026106598	0.019232773	0.176885499
pcaec445.pcaac402	0.041172234	0.030334563	0.176926777
pcaec384.c2	0.062988806	0.046413836	0.176977062
pcaac426.pcaec364	0.020462621	0.015080292	0.177040876
pcaac321.c162	-0.08759564	0.064555877	0.177045609
methionine.pcaac362	0.028289386	0.020851402	0.177103504
pcaac406.pcaec445	0.084297087	0.0621358	0.177120931
serine.pcaac281	0.003975554	0.002930496	0.177135047
c101.pcaec424	0.015352292	0.011316682	0.177137355
pcaec423.pcaac343	0.023838057	0.017571849	0.177138043
pcaec365.pcaec444	0.107850075	0.079505686	0.177168895
c0.smc180	-0.018346726	0.01352673	0.177224878

citruline.pcaac403	-0.017645605	0.013012268	0.177306354
pcaac366.pcaac385	-0.053609014	0.039539783	0.177385639
pcaec420.pcaac405	-0.043277813	0.031928391	0.177499908
pcaec380.pcaec424	-0.034397723	0.025381917	0.177582201
pcaac362.smohc221	0.024043269	0.017741671	0.177588431
pcaac321.pcaac405	0.074283408	0.054822834	0.177656501
lysopcac160.acetylornithoine	-0.09791565	0.072268004	0.177680587
pcaac386.pcaec322	-0.059767032	0.044120328	0.177763
lysopcac204.smohc241	0.101012111	0.074567817	0.177763938
pcaec362.pcaec361	-0.011702288	0.008639131	0.17778504
c3.pcaec444	-0.084145502	0.06212362	0.177811421
pcaec384.smc161	-0.058939575	0.04351811	0.177848294
c5.pcaec360	0.008693657	0.006419583	0.177889288
pcaac406.c162	-0.051413934	0.037965221	0.177890039
lysopcac161.c162	-0.084449008	0.062365476	0.177934276
pcaec365.pcaac384	-0.056877508	0.042007056	0.177966031
c2.pcaac341	-0.007683864	0.005676931	0.178117092
smc240.pcaec363	-0.026123771	0.01930109	0.178129064
c10.c51dc	0.141409218	0.104517666	0.178293876
smohc141.pcaac402	-0.081039302	0.059898117	0.178299334
pcaac300.pcaac381	-0.043593492	0.032225992	0.178366023
smc260.citruline	0.016543957	0.012230368	0.178381426
citruline.pcaec300	0.005253365	0.003883953	0.178417438
pcaec385.c18	-0.034853689	0.025774356	0.178519401
pcaac322.pcaec424	-0.007741923	0.005726052	0.178586333
pcaec443.pcaac281	-0.003835796	0.002837727	0.178694235
pcaac343.pcaec380	-0.085586138	0.063322663	0.178734676
c2.pcaec364	-0.010921966	0.008082835	0.178841187
pcaec405.pcaec300	0.01613923	0.011944741	0.178871992
lysopcac140.asparagine	0.009797573	0.007252895	0.178970425
pcaac281.lysopcac181	0.039746178	0.02943739	0.179180243
pcaec384.pcaac402	0.027215931	0.02016032	0.179250164
smc180.lysopcac160	-0.017590859	0.013031422	0.17928054
c141.pcaac403	-0.005005061	0.003707957	0.179300987
lysopcac181.lysopcac170	0.056444286	0.041820595	0.17934566
lysopcac281.lysopcac170	0.028154214	0.020864457	0.179438644
acetylornithoine.pcaac323	-0.111696224	0.082801385	0.17957342
c51.smc181	-0.02258874	0.016749508	0.179683837
pcaec402.c2	0.079811082	0.059185914	0.179728758
pcaec360.pcaac426	0.077961726	0.057818636	0.179759857
smc260.c14	0.089026807	0.066043755	0.179883895
leucine.pcaac240	0.017669279	0.013111237	0.179997316
pcaac426.c14	0.049548079	0.036766795	0.180001422
methionine.c2	-0.068708586	0.0509887	0.180035261
phenylalanine.pcaec401	0.059285822	0.043996817	0.180042709

phenylalanine.lysopcac161	0.09931028	0.073705677	0.1800789
pcaac405.pcaac323	-0.1288742	0.095657582	0.180125409
lysopcac160.c51dc	-0.144760378	0.107450006	0.180128716
glycine.c0	-0.000611739	0.000454153	0.180207961
pcaec363.citruline	0.019739373	0.014655453	0.180237483
c14.valine	-0.071093124	0.052783284	0.18024039
pcaac360.pcaac380	0.14112558	0.104798686	0.180321403
smohc222.smohc221	0.063592039	0.047224844	0.180339028
pcaec322.lysopcac160	-0.018700713	0.013889757	0.180407517
pcaac361.kynurenine	-0.006056654	0.004498991	0.180453276
serotonin.pcaec445	0.054800843	0.040708116	0.180464616
pcaac300.pcaec384	0.060595567	0.045014982	0.180487004
ornithine.prolinr	0.042219054	0.031363819	0.180491201
arginine.pcaec445	0.043605612	0.032399449	0.180565793
pcaac320.pcaac404	0.007794892	0.005791754	0.180570513
pcaec364.pcaec404	-0.017844066	0.013260182	0.180626038
h1.pcaac406	-0.116858671	0.086842701	0.180642713
acetylornithoine.lysopcac180	0.077954728	0.057932354	0.18064903
c2.c102	0.055792874	0.041465266	0.180675968
pcaec445.c5	-0.012164463	0.00904382	0.180828809
citruline.lysopcac203	-0.010705953	0.007960328	0.180875376
serotonin.smc181	0.029138973	0.021667744	0.180909128
pcaac300.tyrosine	0.053073329	0.039481637	0.181088132
lysine.lysopcac160	-0.018788312	0.013977727	0.181118048
pcaec425.pcaac300	-0.042204109	0.031409602	0.181276677
pcaec446.c141	-0.026837634	0.019979998	0.18141998
smc180.c162	-0.119414172	0.088911094	0.181468794
c181.smohc221	0.030802341	0.02295342	0.181832082
c16.smc202	-0.017197775	0.012819763	0.181976074
smc261.smohc161	-0.020760915	0.015479025	0.182065437
lysopcac161.pcaec300	0.030741788	0.022922674	0.182104477
pcaec364.pcaac404	0.00527564	0.003935138	0.182253152
kynurenine.glycine	-0.023199144	0.017304864	0.182264578
pcaec424.lysopcac181	0.080130692	0.059783889	0.182353634
pcaec446.lysopcac280	0.007045191	0.005256575	0.182378436
c182.lysopcac204	0.027098188	0.020219362	0.182395584
citruline.pcaac344	-0.062195312	0.046412629	0.182446928
c0.pcaac404	0.005820024	0.004343929	0.182526402
pcaec300.pcaac403	-0.029889667	0.022309121	0.182529848
pcaac240.pcaec322	0.066090464	0.049331811	0.182556746
pcaac426.lysopcac280	0.006954504	0.005191167	0.182567341
pcaec401.pcaec424	-0.010058371	0.007509413	0.182646924
c14.pcaec322	-0.043434824	0.03243368	0.182726802
creatinine.pcaac365	0.038066053	0.028431709	0.18283389
smc161.lysopcac160	-0.032648584	0.024387116	0.18286491

pcaec425.pcaec385	-0.073760993	0.055097713	0.182875641
c141.smc260	0.041673044	0.031128819	0.182876241
smohc222.pcaec443	0.090269942	0.067446255	0.182983657
smc161.smc202	-0.051854423	0.038746636	0.183017237
pcaac404.pcaec424	-0.008628007	0.006447323	0.183037995
smohc222.smc202	-0.095858516	0.071643762	0.183116884
pcaac240.isoleucine	-0.019639703	0.014681757	0.183212501
c8.pcaac300	-0.056068612	0.041919566	0.183266465
asparagine.pcaac322	0.057163871	0.042740506	0.183287562
lysopcac140.lysopcac160	-0.021857504	0.016343312	0.183309073
putrescine.transhydroxyproline	-0.034438122	0.025750748	0.183319992
pcaac361.c51dc	-0.133523677	0.099866723	0.183432371
lysopcac161.pcaec424	-0.012957626	0.009694759	0.183581788
c16.pcaac426	0.072618155	0.054351537	0.183737272
isoleucine.pcaac344	0.073996688	0.055402433	0.18388766
pcaec401.pcaac385	-0.049391885	0.036982299	0.183909486
c14.lysopcac204	0.030050235	0.022500452	0.183914548
pcaac366.pcaec424	-0.008502466	0.006366729	0.183942748
c4.citruline	0.011035201	0.008264162	0.183989992
c101.pcaec380	-0.079194486	0.05932478	0.184113052
lysopcac203.pcaec424	-0.00685	0.005132905	0.184244996
pcaec422.lysopcac170	0.013209271	0.009898771	0.18427491
creatinine.pcaec340	0.068054355	0.051000742	0.184293105
lysopcac280.pcaac381	0.039108144	0.029310256	0.184325067
pcaac341.pcaac322	0.098897023	0.074131816	0.184394351
pcaac383.pcaec380	0.041338624	0.030988452	0.18441688
lysopcac180.glycine	-0.019930692	0.014943665	0.184508156
c51dc.leucine	0.026988359	0.020237211	0.184547761
smc202.pcaac420	0.071598588	0.053691666	0.184576112
creatinine.pcaec380	-0.050872245	0.038161084	0.184714116
pcaec340.smc181	0.04009803	0.030080014	0.184729394
pcaac240.glutamicacid	-0.071983385	0.054004863	0.184775207
pcaac362.c102	0.047461427	0.03560992	0.184804946
c2.pcaac404	0.002407668	0.001806882	0.18490848
c141.c102	0.043308263	0.032503422	0.184933691
lysopcac140.c14	0.057930871	0.043495919	0.18511475
c181.tyrosine	-0.075932391	0.057013257	0.185125138
pcaac403.acetylornithoine	-0.060129707	0.045153114	0.185175242
smohc221.pcaec363	-0.034188568	0.025674309	0.185194578
smc261.citruline	0.007666901	0.005758073	0.185234297
pcaac385.pcaac425	-0.111424755	0.083684349	0.185239532
lysopcac181.phenylalanine	-0.080958478	0.060803791	0.185245332
pcaec405.spermidine	-0.067548502	0.050732875	0.185250789
pcaec404.pcaac343	0.020419281	0.015338741	0.185326727
lysopcac203.pcaac381	-0.035762801	0.0268649	0.185331192

c16.lysopcac160	0.030812513	0.023147276	0.185350363
citruline.c18	-0.028341437	0.02129581	0.185450469
pcaec321.pcaec425	-0.030864061	0.023194429	0.185509146
isoleucine.pcaec301	0.048808905	0.036685799	0.18557787
serotonin.smc180	0.014376131	0.010808573	0.185706093
pcaac405.pcaac281	0.002729433	0.002052416	0.185773006
lysopcac181.pcaec444	-0.1370623	0.103088236	0.185871913
pcaac360.pcaec402	0.068470563	0.051503552	0.185914377
prolinr.pcaec364	-0.0194184	0.014607988	0.18595835
isoleucine.c18	0.04511772	0.033959054	0.186191454
serine.smohc221	-0.050942893	0.038353819	0.186308881
glutamine.citruline	0.007511296	0.005655489	0.186339417
pcaac342.pcaac420	0.067248439	0.050634631	0.186349617
pcaec342.pcaec445	0.04394214	0.033090761	0.186410177
pcaac323.glycine	-0.041588115	0.031330708	0.186586956
kynurenine.lysopcac204	0.021958902	0.016545229	0.186648629
c16.pcaec363	-0.005341274	0.004024703	0.186675654
pcaec444.pcaec343	0.10704674	0.080661024	0.186676988
c102.pcaac381	-0.022544643	0.016989166	0.186715699
c51dc.pcaec382	-0.010673315	0.0080433	0.186721997
pcaac366.kynurenine	-0.015675219	0.011813136	0.186738759
pcaac363.kynurenine	-0.01044559	0.007872418	0.18676257
lysopcac280.prolinr	-0.029927996	0.022559386	0.186837641
asparagine.c5	0.012747411	0.009610089	0.186893892
c51dc.histidine	0.088828978	0.066970062	0.186914687
pcaec342.pcaec343	0.134476757	0.101384916	0.186914774
pcaec420.lysopcac204	-0.036779764	0.027733678	0.186987805
lysopcac204.pcaac381	-0.02800866	0.021125376	0.187102372
pcaac402.c181	0.032221864	0.024307864	0.187187148
h1.citruline	0.010591681	0.007991186	0.187237808
pcaac344.c5	-0.011635941	0.008780862	0.187327931
c142.leucine	0.009337237	0.007047073	0.18738314
pcaac281.spermidine	0.070508724	0.053217386	0.187403645
c0.pcaec422	-0.024633977	0.018594064	0.187433039
lysopcac182.asparticacid	0.021102442	0.015929937	0.187474976
pcaec322.c141oh	0.105662931	0.079772351	0.187523822
c141oh.smohc221	0.066654132	0.050332695	0.187618094
c182.taurine	-0.045761665	0.034557072	0.187630218
c2.pcaac425	0.046210082	0.03489765	0.187654771
pcaec401.pcaac403	0.02797772	0.021136055	0.187808446
c16.pcaec380	-0.04624368	0.034936402	0.187822766
pcaec340.lysopcac181	0.041271024	0.031181	0.187841964
c5.smohc161	-0.017300919	0.013071605	0.18785707
pcaac281.c51dc	-0.125392785	0.09474127	0.187864045
acetylornithoine.pcaac380	-0.059994567	0.045336487	0.187934033

smc261.pcaec404	0.018746148	0.014166041	0.187934553
lysopcac280.pcaec404	0.015518419	0.011727176	0.187944151
lysopcac161.pcaac385	0.034496444	0.026078194	0.188103001
lysopcac280.pcaac404	0.004872137	0.00368405	0.188206956
pcaac323.c5	-0.022120247	0.016727054	0.188230652
smc260.smohc221	-0.043269131	0.032725966	0.188316389
c51.c5	-0.006657403	0.005035532	0.188342921
smc160.histidine	0.049142852	0.037175323	0.188397358
pcaac362.pcaec340	0.079421884	0.060081444	0.188403206
pcaac240.pcaac380	0.066784735	0.050530515	0.188480393
pcaec301.pcaec444	0.127032239	0.096122482	0.188515282
pcaac322.pcaec383	-0.083085599	0.062880191	0.188593225
smc160.c2	0.051236992	0.038778982	0.188618188
c0.pcaac344	0.075817061	0.057386595	0.188649271
c162.pcaac406	0.053773899	0.040708201	0.188716908
pcaac323.pcaac320	-0.099595113	0.075417805	0.18884385
c16.smohc241	0.106349703	0.080533125	0.188846291
putrescine.pcaac421	0.023013178	0.017430489	0.188942108
pcaec341.lysopcac204	-0.024001926	0.018179925	0.188955288
pcaac406.pcaec360	0.003460061	0.002621283	0.189040514
pcaec445.histidine	0.04632384	0.035094543	0.189045412
pcaec401.lysopcac203	-0.025344512	0.019205899	0.189162478
lysopcac182.pcaac406	0.032506463	0.02463616	0.189215717
pcaec361.arginine	-0.081992098	0.062141936	0.189225334
lysopcac180.pcaac406	0.032967677	0.024987454	0.189246479
pcaac281.pcaac384	-0.091885726	0.069644629	0.189252462
lysopcac182.smc180	0.040309234	0.030558	0.189334284
c142.pcaec341	0.053215128	0.040343953	0.189357525
asparagine.pcaec445	0.113673722	0.086191024	0.189416887
lysopcac140.pcaec406	-0.042751327	0.032415782	0.189421898
c8.pcaac386	0.023181601	0.017587042	0.189667498
lysopcac182.pcaec420	0.049049122	0.037218862	0.189751072
pcaec425.pcaac343	0.015023709	0.011400683	0.189773236
c10.smc202	-0.05445774	0.041325067	0.189773466
c182.c0	-0.000724585	0.000550038	0.189924483
c3.smc202	0.0664904	0.050474376	0.189933659
phenylalanine.arginine	0.054703456	0.041529883	0.189968045
asparticacid.pcaac406	0.070937159	0.053854244	0.18996825
pcaac343.pcaec382	0.017332588	0.01315863	0.189969386
asparagine.pcaac344	0.035553804	0.026994104	0.190005454
pcaec424.pcaec364	0.009507084	0.007219587	0.190088803
pcaec382.lysopcac161	0.05033526	0.038224542	0.190093796
pcaec420.pcaac426	-0.050376141	0.038257637	0.190117385
c10.pcaac300	-0.054351113	0.041278413	0.190138988
c3.prolinr	0.015659	0.011892872	0.190147244

kynurenine.c18	-0.039636129	0.030107941	0.190215318
prolinr.pcaac405	-0.059330577	0.045080294	0.190335335
pcaac281.pcaec322	-0.083659175	0.063572952	0.190386693
lysopcac182.lysopcac203	-0.013120365	0.009971073	0.190424396
citruline.pcaac321	-0.02894615	0.022000365	0.190468128
pcaec443.lysopcac204	-0.08746875	0.066484527	0.190497129
smohc241.pcaec404	0.011002653	0.008363348	0.190512416
pcaec362.citruline	0.010821861	0.008226501	0.190543299
pcaac424.citruline	-0.007670361	0.005831311	0.190581122
pcaec341.phenylalanine	-0.050353186	0.038282844	0.190608312
pcaac362.kynurenine	-0.007503305	0.005705651	0.190684735
pcaac424.smohc221	-0.114588235	0.087144438	0.190732528
pcaec362.smc181	0.012278578	0.009338303	0.190753068
pcaac425.c14	0.062886147	0.04783251	0.190802094
smohc222.pcaac405	-0.046377599	0.035279443	0.190848047
pcaec300.c2	0.062373655	0.047447974	0.190851118
pcaec381.pcaec424	-0.02287157	0.017402362	0.190948335
smc202.pcaec364	-0.026991281	0.020538989	0.190992344
h1.glycine	-0.034782507	0.02646883	0.191010909
lysopcac170.pcaec364	0.019609765	0.014928719	0.191189914
pcaac320.pcaac323	-0.052480778	0.039958225	0.191246584
pcaec363.leucine	0.011352366	0.00864493	0.191316832
c4.pcaec384	0.085571985	0.065167033	0.191338435
asymmetricdimethylarginine.pcaec44	0.066793969	0.050871881	0.191383346
methioninesulfoxide.asparticacid	0.090799963	0.069165904	0.191450242
pcaac401.h1	0.006441506	0.004907841	0.191548533
h1.pcaec300	-0.017568787	0.013386753	0.191579407
pcaac322.pcaac404	0.003195411	0.002434864	0.191594085
alanine.pcaec342	0.078355569	0.05970827	0.191611052
pcaac321.pcaec424	-0.01134716	0.008646921	0.191620875
pcaac341.histidine	0.04743251	0.036146501	0.191637008
smc161.pcaec445	0.042348859	0.032273281	0.191648438
methioninesulfoxide.phenylalanine	-0.131435934	0.100176999	0.191701798
pcaac401.arginine	-0.073484757	0.056008357	0.191703499
pcaac344.glycine	0.036012472	0.027450402	0.191744367
alanine.smc240	0.046898198	0.035748065	0.19174499
pcaac426.smc261	0.06963204	0.053077243	0.191747886
pcaec300.pcaec405	-0.069298571	0.052826392	0.191775777
pcaec321.valine	0.089327166	0.068098012	0.191800389
glutamine.pcaec446	0.099762577	0.076062392	0.191852692
methionine.smc261	0.033585487	0.025612582	0.191953759
pcaec380.pcaec301	0.128993132	0.098402096	0.19209213
putrescine.pcaec383	-0.054048096	0.041233205	0.192121558
pcaec321.smc180	-0.021868961	0.016692257	0.192345626
c141oh.smc202	-0.049310672	0.037640102	0.192368973

pcaec443.citruline	-0.009986617	0.007623266	0.192381997
pcaec300.lysopcac181	0.046781761	0.035717431	0.192464407
c2.leucine	0.007024422	0.005363133	0.19246877
lysopcac203.pcaac344	0.061849874	0.047222334	0.192469482
pcaac384.pcaac404	0.015435877	0.011785565	0.192480139
phenylalanine.smc160	-0.061247538	0.046775249	0.192590447
smohc141.histidine	0.060311465	0.046069064	0.192673965
c14.pcaec362	0.024443265	0.018678152	0.192842152
c2.pcaec424	0.018241735	0.013940747	0.192888197
asymmetricdimethylarginine.c162	-0.085634475	0.065454255	0.192958834
alphaaminoadipicacid.pcaec382	-0.008566752	0.006550478	0.193129534
pcaac385.valine	-0.064343919	0.04920028	0.193132852
pcaac364.prolinr	0.052678716	0.040283116	0.19316119
pcaec362.putrescine	0.048961118	0.037442667	0.193189278
lysopcac161.asparticacid	0.031107817	0.023790607	0.193210216
pcaec362.leucine	0.017770357	0.013590627	0.193217699
pcaac406.pcaac405	-0.063382171	0.04847542	0.19322904
c162.pcaec362	0.011710272	0.008956741	0.193258128
pcaec403.c0	0.001348778	0.001031711	0.193293487
pcaec446.pcaac323	-0.075880852	0.058062289	0.193440504
c10.lysopcac280	-0.010536898	0.008064406	0.193540074
pcaec321.lysopcac281	0.02160563	0.016537677	0.19358897
lysopcac181.lysopcac280	0.01405301	0.010757162	0.193610128
putrescine.pcaac425	-0.08153799	0.062443081	0.193810001
citruline.pcaec405	-0.05673485	0.043457454	0.193901844
pcaac363.putrescine	0.027025659	0.020702709	0.193939087
pcaac405.c102	0.032353157	0.024784343	0.193949162
c182.lysopcac161	-0.094153099	0.072147038	0.194074797
creatinine.citruline	0.019012455	0.0145709	0.194140385
glycine.pcaec424	0.00815576	0.006251773	0.19423285
pcaec405.pcaac404	-0.009937167	0.007618725	0.194315487
pcaec382.c141oh	0.112830573	0.086522908	0.194401836
asparticacid.c5	-0.008569841	0.006571925	0.194417669
isoleucine.lysopcac180	-0.059255744	0.045463466	0.194634407
pcaec364.c8	-0.055724345	0.042770914	0.194809554
pcaac422.citruline	-0.010238449	0.007860919	0.194948061
c51.smc180	-0.008618282	0.006618806	0.195070511
smohc241.c162	-0.087155794	0.066937463	0.195084839
pcaec382.acetylornithoine	-0.095796547	0.073598894	0.195236566
pcaac322.kynurenine	-0.013289682	0.010210466	0.195246308
pcaac323.prolinr	0.008703701	0.006687724	0.195290688
pcaac361.histidine	0.053118953	0.04081812	0.195320457
pcaac385.smc161	-0.051112895	0.039281648	0.195377425
lysopcac204.tyrosine	-0.044381758	0.034116113	0.19547541
c182.pcaec404	-0.009405148	0.007229713	0.195475778

c14.citruline	0.009386999	0.007215839	0.195480569
lysopcac281.lysopcac160	-0.044512941	0.034218069	0.195489898
pcaac361.pcaec343	0.104543555	0.080370392	0.195520186
histidine.c8	-0.060988056	0.046888844	0.195546686
pcaac403.pcaec382	-0.034100515	0.026219539	0.195586951
c51dc.smohc241	0.060987398	0.046903747	0.195692644
pcaec322.pcaac366	-0.056887115	0.043752217	0.195711791
pcaec443.pcaac362	-0.051685125	0.039752438	0.195724121
pcaac402.citruline	-0.010356741	0.007966543	0.195773823
pcaec385.pcaec404	-0.009583411	0.007372409	0.195817308
c141oh.pcaec360	0.008810278	0.006777834	0.195829615
asparagine.pcaac402	0.05562373	0.042792649	0.195837531
smc160.pcaec425	-0.024591627	0.018919047	0.195840554
pcaec341.pcaac386	0.015283879	0.011759589	0.195888167
pcaec402.pcaec444	0.160157363	0.123243348	0.19594755
acetylornithoine.pcaec405	-0.050459699	0.038836078	0.196023418
pcaac360.smc202	-0.04598587	0.035393776	0.196035471
pcaac344.smc240	0.04132377	0.03180763	0.196065042
lysopcac160.asparticacid	0.019791913	0.01523677	0.196140531
pcaec405.citruline	-0.008725413	0.0067181	0.196197136
pcaec381.pcaac381	0.032973741	0.025406228	0.19651615
smc241.smc160	-0.167062018	0.128721811	0.196518657
serotonin.methionine	0.031773923	0.024482743	0.196533823
pcaec300.lysopcac160	-0.020503573	0.015800804	0.196595706
c2.smohc222	-0.061939443	0.047732854	0.196596183
pcaac281.pcaac343	-0.041088269	0.031665228	0.196611205
pcaec343.citruline	0.011630621	0.008963707	0.196631681
c101.c51dc	0.142720742	0.110013255	0.196706633
pcaac366.smohc161	-0.016617026	0.012809761	0.196737163
asparticacid.pcaac420	0.099297455	0.076546711	0.196737882
smohc161.pcaec361	-0.017832075	0.013747033	0.196757007
methionine.pcaac426	-0.035753743	0.027573867	0.196930274
pcaac321.smc240	0.0398625	0.0307451	0.196966351
pcaec365.transhydroxyproline	-0.066708306	0.051456291	0.197014726
asparagine.c182	0.073278726	0.056534801	0.197096103
pcaec444.pcaec342	-0.116555598	0.089927597	0.197118553
pcaac386.c2	0.077274966	0.059622137	0.197127635
pcaac365.pcaec405	-0.052714036	0.040674808	0.197158936
c2.tyrosine	0.066646093	0.051429278	0.197196342
lysopcac203.histidine	0.082449187	0.063625663	0.197206815
smohc161.pcaac421	-0.03038901	0.023451394	0.197213127
c142.pcaec382	-0.011388917	0.008789002	0.19721831
pcaec340.pcaec382	-0.011517248	0.008888266	0.197229731
pcaec342.pcaac420	0.051406691	0.039689796	0.197425513
c0.c3	-0.026924421	0.020787764	0.197427764

c162.leucine	0.008558706	0.006608066	0.197432728
pcaec420.smc160	-0.064586604	0.049867093	0.197438221
pcaec362.pcaac322	0.070716804	0.054607648	0.197498989
pcaec361.lysopcac161	0.062540556	0.04829834	0.19753963
pcaac320.pcaec361	-0.0110247	0.008514551	0.197564741
smc240.leucine	0.007676886	0.005929597	0.197611072
serine.pcaec364	0.013914495	0.010748601	0.197656597
pcaac404.smc161	-0.040660786	0.03141508	0.197736552
pcaec343.pcaec382	-0.011819112	0.009132401	0.197775262
lysopcac161.pcaac426	-0.094232189	0.072815448	0.197800009
asparticacid.c0	0.000667799	0.000516052	0.197823854
pcaec383.c0	0.001174609	0.000907809	0.197878901
pcaac384.pcaac425	0.08371804	0.064705167	0.197898191
lysopcac160.histidine	0.064036548	0.049494664	0.197909024
histidine.pcaec445	0.069213704	0.05349651	0.197911997
methioninesulfoxide.pcaac364	0.132755085	0.102614596	0.197937262
c181.taurine	-0.067625298	0.052272055	0.197939733
pcaec422.c4	-0.038319834	0.029626944	0.198045316
pcaac403.pcaec364	0.005712957	0.004417331	0.198082033
pcaac363.pcaec300	0.004665617	0.003608164	0.198162349
smc180.lysopcac180	0.082681794	0.063944011	0.198175441
pcaec402.pcaec322	0.080034555	0.061899773	0.198197556
pcaec424.pcaec382	-0.023167224	0.017918175	0.198206032
pcaac342.citruline	0.016255805	0.012573148	0.198222217
smc241.smc180	-0.013674422	0.010578033	0.198284248
pcaec341.pcaec322	-0.065646219	0.050796679	0.198417409
smc181.tyrosine	-0.073055995	0.056532304	0.198433057
lysopcac161.smc181	-0.02081437	0.016108174	0.198476418
asparticacid.lysopcac180	0.08190722	0.063395489	0.198530995
methionine.kynurenine	-0.014867385	0.011507272	0.198532652
pcaec401.lysopcac180	0.075957679	0.058797283	0.198581744
acetylornithoine.phenylalanine	0.071687467	0.055502933	0.198671267
pcaac422.pcaac405	-0.072816263	0.056387353	0.198754118
pcaec343.pcaac386	-0.01491433	0.011551525	0.198838643
pcaac406.pcaac403	0.034881372	0.027018182	0.198866394
pcaec301.pcaec342	0.107741691	0.083456512	0.198880654
c8.spermidine	0.067483909	0.0522762	0.198908755
pcaec301.pcaac320	-0.080039102	0.062016992	0.199016397
c10.lysopcac204	0.024083756	0.018666884	0.199159417
c141.smc202	-0.044385318	0.034403561	0.199176349
smohc222.pcaac404	0.007619725	0.005907459	0.199276474
pcaac321.kynurenine	-0.01711509	0.013273127	0.199412887
pcaac403.prolinr	-0.034566277	0.026810193	0.199467715
asparagine.asparticacid	0.042229782	0.032759246	0.19953738
pcaec386.pcaec444	0.122753409	0.095230814	0.199567091

smohc221.histidine	0.060143251	0.046668351	0.199661367
pcaac323.lysopcac280	0.010337337	0.008021692	0.199683736
lysopcac160.pcaec363	0.019294463	0.014976444	0.199805923
phenylalanine.transhydroxyproline	-0.039354763	0.030548826	0.199827752
lysine.pcaec340	0.04179561	0.032444534	0.199841797
kynurenine.pcaac420	0.112493593	0.087332814	0.199881733
pcaac385.smc240	0.03564092	0.02766937	0.199882447
kynurenine.pcaec380	-0.049159042	0.038167485	0.199923395
methioninesulfoxide.c162	0.07645856	0.059363093	0.199923671
lysopcac204.pcaac404	-0.007697906	0.005978014	0.200020343
pcaec422.c51	-0.119841961	0.093085708	0.200112793
pcaac364.pcaac323	-0.107833934	0.083781049	0.200232506
c18.pcaec362	0.048921082	0.038016338	0.200318899
pcaac363.lysopcac160	-0.033144304	0.025763534	0.200444879
isoleucine.pcaac240	0.016816819	0.013072539	0.200465039
isoleucine.pcaac402	-0.071822357	0.055832684	0.200478185
prolinr.c16	-0.028300299	0.022000232	0.200485801
pcaac344.transhydroxyproline	0.027987707	0.021758668	0.20051541
pcaec342.lysopcac204	-0.029898112	0.023246822	0.200571927
pcaac383.pcaec424	-0.004805171	0.003736297	0.200585037
glutamicacid.arginine	-0.115991907	0.090208077	0.200672812
pcaec424.asparticacid	0.033061218	0.02572724	0.200937323
pcaac405.methionine	-0.038388933	0.029873259	0.200939601
glutamicacid.pcaec382	-0.017364912	0.013515486	0.20102465
c182.pcaac343	0.023668529	0.018422433	0.201041817
pcaec365.pcaac421	-0.042689284	0.033228421	0.201057417
lysopcac161.glycine	-0.037071523	0.028859439	0.2011158
c2.pcaec341	0.050352076	0.039201951	0.201159981
pcaec403.pcaec383	0.320259827	0.249340517	0.201160078
c4.pcaec361	-0.011218054	0.008734423	0.201187195
pcaac404.kynurenine	-0.018809686	0.014647265	0.201247328
c14.acetylornithoine	0.100586531	0.078346812	0.201357193
pcaac402.pcaec424	-0.022941667	0.017872275	0.201432873
pcaec403.lysopcac161	0.05654535	0.044052753	0.20145484
leucine.pcaec300	-0.015555896	0.012119182	0.201457034
lysopcac280.spermidine	-0.066839181	0.05207295	0.2014599
pcaac281.leucine	0.008889467	0.006926226	0.201501051
pcaac406.pcaac420	-0.052660386	0.041042777	0.201637017
pcaac343.prolinr	0.025736578	0.020059705	0.201658704
c5.citruline	0.007031681	0.005480959	0.201683123
pcaec383.arginine	-0.117651424	0.091724085	0.201774791
acetylornithoine.pcaac386	0.05795755	0.045186234	0.201785043
pcaac405.c162	-0.061405114	0.047877966	0.201821188
smc261.lysopcac203	-0.013274794	0.010350788	0.201836116
pcaec425.pcaec422	-0.03727964	0.029069613	0.201858746

pcaec321.pcaac402	-0.06992216	0.054533322	0.201940908
lysopcac204.leucine	-0.032075941	0.0250183	0.201973187
c141.smc181	-0.027950419	0.021800548	0.201973905
lysopcac140.smc180	0.025535823	0.019918956	0.202012756
pcaec321.glycine	0.096708031	0.075443139	0.202054577
pcaac300.histidine	0.061412329	0.047911383	0.202081351
c4.kynurenine	-0.014564957	0.011366422	0.202217093
pcaec420.pcaac320	-0.122488216	0.09564773	0.202491648
pcaac386.pcaac403	-0.022583797	0.017638575	0.202580695
lysopcac203.asparticacid	0.026590604	0.020772101	0.202669249
pcaac341.smc240	0.030633915	0.02393963	0.202837431
asparticacid.tyrosine	-0.066588985	0.052038549	0.202845539
citruline.pcaec443	0.037121855	0.029014061	0.202903508
asparticacid.c102	0.051201055	0.04001873	0.202909228
creatinine.transhydroxyproline	0.027478426	0.021478152	0.2029306
asymmetricdimethylarginine.citruline	0.009268652	0.007245173	0.202958608
pcaac420.pcaac406	0.049996631	0.039083831	0.202983656
pcaec425.pcaac281	-0.00693617	0.0054228	0.203032745
pcaec365.smc181	0.026909931	0.021040867	0.203081543
asparagine.smc180	0.084558118	0.066117214	0.203090015
c101.pcaac381	-0.033927718	0.026529537	0.203106323
pcaac320.smc161	-0.049883359	0.039010228	0.203155741
pcaac322.pcaac421	0.019407131	0.015178087	0.203189771
c16.smc160	0.122875968	0.096103035	0.203204716
pcaac321.c18	0.053529408	0.041877191	0.203323623
lysopcac140.smohc221	-0.042771391	0.033481384	0.203597716
pcaec340.pcaac322	0.078312663	0.061305359	0.203614714
pcaec384.prolinr	0.032326508	0.025309488	0.203674852
smohc222.pcaac425	0.083498041	0.065377729	0.203704854
methionine.citruline	0.005798693	0.004542755	0.203948836
pcaec362.pcaac344	0.096749798	0.075796014	0.203956139
smohc221.citruline	0.007079303	0.005546406	0.20398187
smohc141.pcaac405	-0.060653749	0.047531452	0.20408778
pcaec403.citruline	-0.006267882	0.004912258	0.204126055
pcaac320.lysopcac181	0.03770699	0.029560423	0.204259019
pcaac341.kynurenine	-0.018186364	0.014257615	0.204271665
c51.pcaec340	0.038508945	0.030190267	0.204276164
kynurenine.smc181	-0.013201058	0.010350193	0.204311767
pcaec341.pcaac404	0.011332309	0.008885335	0.204328039
pcaac386.lysopcac161	-0.077638317	0.060883261	0.204396802
pcaac363.pcaec424	-0.006838584	0.005365467	0.204624292
smc180.pcaac322	0.083489722	0.065506995	0.204638092
pcaec322.pcaac240	-0.017049825	0.013378427	0.204669653
lysopcac281.leucine	0.042095538	0.033032213	0.204686746
pcaac381.pcaec364	0.006338589	0.004974736	0.204765305

c10.smohc221	-0.070367025	0.0552468	0.204931712
pcaec401.smc241	0.049496145	0.038864088	0.204972318
lysopcac182.pcaec424	-0.012837981	0.010081599	0.205029948
pcaec386.pcaac380	0.166080198	0.130429333	0.205055594
c181.lysopcac170	0.021600383	0.016964384	0.20507552
c18.pcaec404	0.054064615	0.04247361	0.205209733
alanine.pcaec420	-0.039435938	0.030986866	0.205292096
histidine.citruline	0.005112329	0.004017977	0.205399059
histidine.lysopcac160	0.03848999	0.030251451	0.205409253
smc181.kynurenine	-0.004195678	0.003297645	0.205412696
pcaec342.citruline	0.009710458	0.007632369	0.205431271
pcaac322.c0	-0.0008839	0.000694757	0.205441643
pcaac363.pcaac406	-0.039679268	0.031189139	0.205452132
smohc161.lysopcac160	-0.028291597	0.022238551	0.20546176
lysopcac181.c3	-0.076317758	0.059998357	0.205528923
pcaec421.pcaac405	-0.054307089	0.042698067	0.205568282
arginine.smc160	-0.093677366	0.073653066	0.205572921
pcaac405.pcaec446	-0.046699016	0.036719407	0.20560588
c51.pcaec322	0.052957128	0.041645315	0.205661719
lysopcac203.c51	-0.081570328	0.064160022	0.205755711
glutamicacid.pcaac424	0.039004058	0.030680899	0.205782685
lysine.prolinr	0.038554521	0.030330553	0.205831203
c14.transhydroxyproline	-0.022241688	0.017498462	0.205859342
pcaec401.smc260	-0.034095155	0.026833064	0.206010344
phenylalanine.pcaec382	-0.037759591	0.029722377	0.206092029
c141oh.pcaec380	-0.028566276	0.022488208	0.206138719
c182.citruline	0.00723823	0.005699304	0.206230323
pcaac362.citruline	0.004575581	0.003603003	0.206260439
pcaec342.smc161	-0.075456304	0.059440758	0.206437416
c3.pcaec364	-0.018064352	0.014237078	0.206655257
pcaec425.pcaec444	0.098051823	0.077286341	0.20670576
creatinine.pcaec444	-0.090644058	0.071459015	0.206779144
pcaec445.tyrosine	-0.040753159	0.032127856	0.206782041
pcaec405.acetylornithoine	-0.056785673	0.044767792	0.20678872
pcaec401.pcaac405	-0.053673064	0.042316189	0.206812901
c182.leucine	0.020250292	0.015965499	0.206813974
lysopcac280.pcaac420	0.094524656	0.074532339	0.20686433
c18.c16	-0.063111876	0.04976716	0.206897721
smohc222.pcaec404	-0.017974228	0.014177081	0.20700668
creatinine.pcaec363	0.018600029	0.014671713	0.207038509
methioninesulfoxide.smc202	-0.091783055	0.072399273	0.207043192
pcaec362.histidine	0.061808882	0.048756441	0.207052973
glutamine.pcaac426	-0.055908404	0.044113914	0.207175029
pcaac425.pcaac405	-0.032082182	0.025315292	0.207196396
pcaac365.arginine	0.04918911	0.038822623	0.207297141

histidine.pcaac405	-0.064661849	0.051034788	0.207299518
c4.lysopcac280	-0.013967752	0.011025368	0.207349835
pcaec343.pcaec422	0.035891067	0.028333007	0.207391154
c14.leucine	0.00713631	0.005633625	0.207399403
pcaec343.pcaec341	0.074958504	0.059177369	0.207420734
c16.pcaec343	0.058437498	0.046157644	0.207646782
smc180.transhydroxyproline	-0.024547191	0.019389301	0.207655456
smc181.pcaac425	0.099547631	0.078631377	0.207660238
smc161.pcaec385	-0.049750135	0.039322102	0.207949059
smohc161.pcaec341	0.099575457	0.078718318	0.208033409
pcaec421.citruline	-0.006470834	0.005115579	0.208044941
asymmetricdimethylarginine.smohc2	-0.017143162	0.013553756	0.208080509
smc240.c162	-0.089000361	0.070367508	0.208092652
c14.prolinr	0.042422422	0.03354733	0.208178129
pcaec364.citruline	0.006648179	0.005257779	0.208216811
pcaac380.smohc221	0.028213841	0.02231779	0.208309829
lysopcac140.smc240	0.074879963	0.059235036	0.20833489
smohc221.lysopcac280	0.014756791	0.011674019	0.208350951
serine.c2	-0.065167364	0.051557871	0.208388827
ornithine.lysine	0.104570386	0.082736388	0.208412807
smc161.smc260	-0.032900963	0.026032962	0.208441057
pcaac360.pcaac381	-0.049712185	0.039335525	0.208448484
phenylalanine.prolinr	0.05155158	0.04079567	0.208500551
pcaec381.citruline	-0.005965979	0.004723124	0.208683439
c8.smohc221	-0.063060552	0.049931475	0.208755333
lysopcac204.glycine	-0.046510328	0.036831453	0.208810375
c102.lysopcac180	0.072956663	0.057776376	0.208826931
lysopcac181.pcaac380	0.051500211	0.040791833	0.208909138
pcaec385.c14	-0.038767039	0.030710502	0.208971736
pcaec361.pcaac385	-0.040180214	0.031831054	0.208986847
lysopcac140.pcaec422	0.041045309	0.032521437	0.209057137
lysine.pcaec322	-0.100141897	0.079347737	0.209070194
c10.phenylalanine	-0.048471795	0.03840801	0.209084762
c141.lysopcac204	0.043355763	0.034355765	0.209105698
pcaac386.pcaec382	-0.009973614	0.007904418	0.209172977
pcaac322.putrescine	0.039997403	0.031700058	0.209184386
pcaac360.pcaac421	-0.025744693	0.020404927	0.209204261
pcaac401.citruline	-0.006962376	0.005518579	0.209227726
pcaec341.pcaac365	-0.045112068	0.0357583	0.209242632
pcaec301.glutamicacid	-0.063312018	0.05018805	0.209273955
serine.lysopcac161	0.060787343	0.048195362	0.20935521
smc161.citruline	0.00592979	0.004702511	0.209457828
pcaec340.pcaac281	-0.004091784	0.003246049	0.209616272
smc160.leucine	0.006418985	0.00509281	0.209667125
c51.lysine	0.098670388	0.07830133	0.209762247

glutamine.pcaac281	0.001464824	0.001162588	0.209822592
pcaec404.citruline	-0.004940801	0.003921844	0.20987773
smc241.citruline	0.007827015	0.006213856	0.209952867
pcaac344.smc241	0.028644341	0.022743189	0.210002509
c10.pcaec424	0.0140145	0.011128177	0.210037835
c181.citruline	0.006526283	0.005182452	0.210061831
smohc221.pcaac403	-0.007537993	0.005986072	0.210079389
alphaaminoadipicacid.acetylornithoir	-0.074594937	0.059242792	0.210121109
serine.pcaec322	-0.041544631	0.032994837	0.210126242
smc241.lysopcac280	0.010531791	0.00836525	0.210173857
pcaac360.pcaec343	0.092722637	0.073659245	0.210241501
pcaec321.alphaaminoadipicacid	0.034242541	0.027202915	0.21024981
serine.pcaac322	0.041816157	0.033219631	0.210251101
c141oh.pcaac406	0.044089848	0.035028362	0.210282995
c162.citruline	0.006770236	0.005379973	0.210382245
c8.pcaec424	0.013256119	0.010534362	0.210398477
c14.pcaec301	-0.051330053	0.040792005	0.210410495
pcaac380.pcaac343	0.035761161	0.028436569	0.210684383
glutamine.pcaec340	0.043215178	0.034364633	0.210694652
pcaec425.smohc221	0.044846572	0.035662003	0.210695774
pcaac426.histidine	0.073866758	0.058741261	0.210714314
pcaac384.pcaec445	0.053481182	0.04253137	0.210729098
pcaac363.citruline	0.006307623	0.005018034	0.210895768
c51dc.c162	-0.095692566	0.076130196	0.210907094
pcaac384.pcaec361	-0.010301661	0.008196039	0.210925955
pcaac365.pcaec365	-0.038334208	0.030499356	0.210933656
pcaac361.smc260	-0.024804162	0.019737322	0.210995837
c102.lysopcac204	0.034137887	0.027167383	0.211045477
isoleucine.citruline	0.003761821	0.002994294	0.211134355
pcaac402.c141oh	0.106267075	0.084588091	0.211149289
methionine.pcaec382	0.011538266	0.009185032	0.211180264
pcaec384.alanine	-0.026155412	0.020827864	0.211329792
smc260.smc240	-0.053981443	0.043005064	0.211530485
smc202.lysopcac160	-0.016418869	0.01308179	0.211581543
pcaec321.histidine	0.054428148	0.043366583	0.21158961
pcaac422.smc161	0.1475806	0.117622906	0.211726601
pcaac385.c2	0.05852547	0.046646591	0.211739546
lysopcac161.arginine	0.0543146	0.043297959	0.211818868
pcaac320.arginine	-0.06462462	0.051523191	0.211875269
pcaec380.pcaac281	-0.003591762	0.002863997	0.21193842
pcaac406.pcaac361	-0.073035444	0.058240634	0.211966995
prolinr.pcaec301	0.067919288	0.054180269	0.212129825
pcaac404.c16	0.030622896	0.024428573	0.212133818
pcaec364.smc180	-0.014582371	0.011633904	0.212181416
smohc222.citruline	0.003688279	0.00294283	0.212227559

c181.leucine	0.012672719	0.010112207	0.212263929
pcaec321.pcaac420	0.095545547	0.076243005	0.212277944
smc181.pcaec445	0.041734919	0.033306254	0.212316318
pcaec385.pcaac365	0.034629493	0.027636252	0.212323432
pcaec403.acetylornithoine	-0.05348596	0.042687585	0.212353559
pcaec362.phenylalanine	-0.040960869	0.032691708	0.212360521
pcaac321.pcaec341	-0.111915145	0.089325939	0.212381755
pcaec301.pcaec361	0.020497273	0.016360867	0.212404272
citruline.smohc221	0.020072635	0.01602533	0.212500964
pcaec360.arginine	-0.064548984	0.051539368	0.21255026
c101.lysopcac160	0.015146807	0.012094801	0.212579553
pcaec383.citruline	-0.004784795	0.003821039	0.212621897
smc241.pcaac322	0.050757179	0.040544543	0.212744259
lysopcac181.lysine	0.081715012	0.06527519	0.212756156
pcaec380.smohc221	0.06632985	0.052987416	0.212774413
c0.pcaec403	0.039718344	0.031733114	0.212834977
pcaac361.citruline	0.003314168	0.00264798	0.212854554
spermidine.pcaac320	0.120515348	0.096295035	0.212876857
pcaac344.isoleucine	-0.041472445	0.033139899	0.212908282
c4.pcaac405	0.055659776	0.044496658	0.213111943
c51.citruline	0.001931416	0.001544124	0.213133192
smc202.citruline	0.006131155	0.004901876	0.2131477
smc241.c5	-0.008128641	0.006499347	0.213180966
alphaaminoadipicacid.pcaac406	0.03800711	0.030389078	0.213181875
h1.smc202	0.083528188	0.066787567	0.21319224
pcaec322.c8	0.052538551	0.042013364	0.213241287
pcaec404.asparticacid	0.06179191	0.04941394	0.213250189
pcaec343.pcaac323	-0.114727721	0.09179134	0.213476031
methionine.lysopcac280	0.010320663	0.00825786	0.213503931
pcaac320.lysopcac280	0.013868581	0.011096986	0.213517591
pcaec360.pcaac300	-0.048951485	0.039171448	0.213549847
pcaec383.lysopcac181	0.061864087	0.049505623	0.213562595
pcaac424.pcaec380	0.085499851	0.06843503	0.213664488
c181.lysopcac204	0.031641007	0.025328435	0.213711624
c8.lysopcac182	0.093729839	0.075035543	0.213744229
smc161.leucine	0.009954324	0.007969236	0.213760752
pcaac281.pcaac425	0.101517106	0.08127374	0.213767152
smc180.citruline	0.006609387	0.005292018	0.213818621
pcaec384.pcaac384	-0.05354768	0.042875222	0.213824522
pcaac420.pcaac240	-0.020206404	0.016180045	0.213850702
smohc221.smc202	-0.075711466	0.060627337	0.213867632
serine.smc261	0.032258243	0.025832743	0.213891736
pcaec360.pcaac425	-0.098814947	0.07913572	0.213912814
glycine.citruline	-0.01239612	0.009927576	0.213920695
pcaec425.pcaac381	0.023574789	0.01888185	0.213961904

lysopcac181.pcaac426	-0.071257786	0.057094185	0.214132714
pcaec443.lysopcac280	0.013063639	0.010468376	0.214191024
pcaec360.pcaec446	-0.063177972	0.050627501	0.214196976
pcaec420.pcaac406	-0.069260829	0.055509501	0.214258701
lysopcac281.kynurenine	-0.007741436	0.006204523	0.214266242
pcaac424.pcaac300	-0.069545473	0.055742723	0.214300336
lysopcac204.pcaec405	-0.037628714	0.030162782	0.214334648
asparagine.citruline	0.004637356	0.003717804	0.214401829
c2.citruline	0.023100612	0.01852436	0.214510638
putrescine.pcaac405	0.018303892	0.014678514	0.214530525
pcaac321.arginine	0.046127829	0.036993861	0.214559893
pcaac401.pcaac406	0.051126289	0.041003121	0.214566192
pcaac364.c5	-0.006538714	0.00524544	0.214688903
c14.lysopcac170	0.034248802	0.027477144	0.21472739
c141oh.arginine	0.035873688	0.028787721	0.214837695
pcaec301.smohc221	-0.028436178	0.022822027	0.214892
smc240.alanine	-0.058018071	0.046565198	0.214907939
asymmetricdimethylarginine.c2	-0.063215632	0.050740357	0.214940356
pcaec445.smc161	-0.056534811	0.045385366	0.215014773
pcaec342.leucine	0.009155258	0.00735189	0.215149758
pcaac381.citruline	-0.006706453	0.005387661	0.215337741
c141oh.prolinr	0.032115667	0.025802161	0.215371111
pcaec300.pcaec343	0.071382356	0.05736243	0.215473585
pcaec383.pcaac344	0.089139426	0.0716346	0.215490764
smc181.pcaec444	0.088237242	0.070909733	0.215491727
c12.leucine	0.008742376	0.007026173	0.215528953
leucine.citruline	0.002425631	0.001949784	0.215605183
glutamine.pcaec343	-0.059228104	0.047622374	0.215732687
pcaec405.pcaec382	-0.084569722	0.068003064	0.215764614
smohc141.pcaac404	0.005798049	0.004663317	0.215869227
pcaac343.c51	-0.124459799	0.10010499	0.215883453
pcaac361.c102	0.031279587	0.025163781	0.215976228
pcaac365.methionine	-0.034409705	0.02768308	0.21599577
pcaec382.citruline	-0.005444566	0.004381436	0.216121646
smohc221.pcaac421	-0.032428676	0.026099482	0.216173688
c10.pcaac406	0.048053222	0.038676426	0.216195938
citruline.methionine	-0.028070484	0.022593866	0.216213631
pcaec445.pcaec360	0.008715625	0.007015869	0.21625812
lysopcac180.pcaec321	0.042786078	0.034450765	0.216377441
smohc241.pcaec444	-0.101446832	0.081694154	0.216436456
smc181.asparagine	0.018008626	0.014502625	0.216450705
pcaec425.citruline	-0.008857516	0.007133966	0.216506514
pcaac402.pcaec322	0.059567373	0.047980825	0.21654879
pcaec386.pcaec445	0.063740955	0.051344453	0.216565307
pcaec341.citruline	0.004339969	0.003496061	0.216583609

pcaac341.smc161	-0.042295146	0.034079306	0.216697098
smc261.alanine	-0.015180244	0.012232784	0.21674588
c3.c2	-0.04780384	0.038533802	0.216885327
pcaec362.pcaec443	0.051167582	0.041246624	0.216900532
lysopcac204.citruline	-0.034070781	0.027466987	0.216937803
pcaec405.pcaec383	0.143897241	0.116027634	0.217022058
citruline.pcaac323	-0.077642862	0.062612182	0.217072938
c102.c4	-0.03682864	0.029704217	0.217152022
c51dc.isoleucine	0.034178796	0.027567808	0.217165767
smc160.prolinr	0.035246178	0.028430026	0.217186563
c0.c182	-0.086453579	0.069749308	0.217283119
pcaac421.pcaec404	-0.01289474	0.010403425	0.21729027
pcaac404.asparagine	0.010077318	0.00813099	0.217326891
pcaec420.pcaac422	-0.055751612	0.045001257	0.217504585
glutamicacid.lysopcac182	-0.059033762	0.047651404	0.217513028
c101.pcaac386	0.016518539	0.013334446	0.217542634
pcaec322.citruline	0.005337261	0.00430883	0.217582251
lysine.pcaec401	0.026671586	0.021532749	0.217592446
pcaec385.citruline	0.003723104	0.003007947	0.217923711
pcaec401.pcaec405	-0.066551551	0.053774965	0.217983673
prolinr.pcaac365	-0.045806665	0.037025313	0.218139778
pcaec382.pcaec342	0.079506895	0.06427309	0.218197039
pcaec423.citruline	-0.007279859	0.005885441	0.218230387
pcaac341.citruline	0.009173462	0.007417652	0.218311787
smc181.pcaec341	0.057718131	0.04667743	0.218376567
pcaec446.pcaec341	0.053151711	0.042990741	0.218442998
pcaec380.arginine	-0.062699054	0.050715113	0.218462743
smc161.glutamine	-0.023799935	0.019253529	0.218524084
pcaec422.citruline	-0.017458885	0.014123873	0.21852693
smc180.pcaac404	0.010783597	0.008725174	0.218604106
pcaec425.tyrosine	-0.051421632	0.041606969	0.218614399
pcaec423.pcaec361	-0.045467083	0.036791226	0.218642815
serine.kynurenine	-0.006278853	0.005081417	0.218703309
pcaec421.spermidine	-0.063376814	0.051305582	0.218840104
methionine.pcaac381	0.018884125	0.015295395	0.219082375
c182.pcaac426	0.051622871	0.041814898	0.219108796
methioninesulfoxide.pcaac365	0.082430433	0.066776029	0.219155356
pcaec381.prolinr	-0.023359243	0.018924617	0.21919265
c101.glycine	-0.026404353	0.021392931	0.219220519
pcaec300.leucine	0.008097332	0.00656175	0.219308089
acetylornithoine.lysopcac280	-0.010188511	0.008257047	0.219346405
pcaec321.citruline	0.004978778	0.004035103	0.219365262
creatinine.pcaac281	-0.003344013	0.00271027	0.219378655
lysopcac181.c0	-0.000641517	0.000520072	0.219495591
asparticacid.c8	0.069788037	0.056579695	0.219521201

smc181.lysopcac160	-0.016498125	0.013376397	0.219547533
glutamine.pcaec445	0.062738723	0.050871065	0.219579827
isoleucine.lysopcac203	0.015269837	0.012384154	0.219682016
pcaac401.c141oh	0.101449154	0.082282877	0.219712742
c18.pcaac381	-0.010950833	0.008881977	0.21971427
smc261.arginine	-0.032416725	0.026294014	0.219740942
pcaac364.c141	-0.0401725	0.032593213	0.219857715
c101.c8	0.076063679	0.061716061	0.219881531
smc241.histidine	0.046878259	0.038038065	0.219909064
c8.pcaec443	0.057937908	0.047015584	0.219942946
pcaec402.smohc222	0.008951867	0.007266386	0.220075875
pcaac363.smc241	0.074521123	0.060497744	0.220133893
c182.kynurenine	-0.015079707	0.012242296	0.220144673
pcaac402.lysopcac161	0.074987917	0.060887741	0.220217128
phenylalanine.citruline	0.002041115	0.001657456	0.220255088
pcaec406.citruline	0.00471572	0.003829836	0.220315972
c12.acetylornithoine	0.103287116	0.083902894	0.220420232
smohc141.leucine	0.012002157	0.009752628	0.220559269
pcaac381.pcaac343	0.012086993	0.009822289	0.220593184
pcaac383.glycine	0.057655646	0.046857829	0.220641679
lysopcac160.citruline	-0.00972432	0.007904447	0.220717701
pcaac344.prolinr	0.047913862	0.038948633	0.220737575
leucine.pcaac404	-0.017200256	0.013981962	0.220739847
c162.c182	-0.078511838	0.063826894	0.220777248
pcaec403.c181	-0.031946335	0.025978349	0.220906178
pcaec300.glycine	0.054139425	0.044040117	0.22105878
pcaac385.pcaac341	-0.007298603	0.005937204	0.221066619
smc181.lysopcac170	0.010820111	0.008802338	0.221092085
pcaec301.kynurenine	0.013891209	0.011302516	0.221164811
pcaac323.pcaac281	0.001786817	0.001453891	0.221182537
pcaac322.lysopcac161	0.070758394	0.057580364	0.221229698
pcaac420.spermidine	0.064659284	0.05264247	0.221450712
lysopcac280.pcaec341	-0.064909203	0.052849815	0.221484379
pcaac380.citruline	0.003635734	0.002960374	0.221502662
alanine.citruline	0.004685173	0.003814953	0.221512318
asymmetricdimethylarginine.pcaac40	0.026208813	0.021343765	0.221575878
pcaac365.pcaac401	0.023613222	0.019231795	0.221619156
pcaac323.pcaac381	0.031165599	0.0253833	0.221627668
c141.smohc222	0.005260415	0.004284608	0.221647291
pcaac404.pcaac344	0.078073595	0.063610253	0.22178676
kynurenine.pcaac425	-0.09195659	0.074928828	0.221832415
lysopcac170.pcaec420	0.042525433	0.034651705	0.221842774
lysopcac161.c8	0.055883076	0.04553688	0.221850283
alphaaminoadipicacid.citruline	0.002443585	0.00199125	0.221866585
prolinr.smc261	0.030690392	0.025016854	0.222006205

pcaac405.asparagine	0.013806087	0.011254262	0.222023277
pcaec342.glutamine	-0.01598475	0.013031528	0.222068914
c12.pcaac406	0.049317581	0.040208266	0.222094401
pcaac406.citruline	0.002563079	0.002089863	0.222139288
c141oh.leucine	0.007497141	0.006113942	0.222213045
c10.lysopcac160	0.018340029	0.014957141	0.222237312
pcaac385.kynurenine	-0.006517529	0.005317651	0.22243668
c12.smohc241	0.091543224	0.074692509	0.222451536
pcaec301.c4	-0.012122299	0.00989102	0.222457065
pcaac384.leucine	0.007642615	0.006236776	0.222522835
c2.smohc221	0.089386281	0.072944584	0.222527096
acetylornithoine.smc181	0.030646899	0.025015391	0.222631524
c142.c51dc	0.119894255	0.097877336	0.22269826
pcaec301.smc240	0.02803794	0.022891026	0.22273575
lysopcac180.lysopcac204	0.030530551	0.024927242	0.222757402
pcaec425.smc202	-0.061903159	0.050543277	0.22276905
c51dc.pcaac381	-0.025479553	0.02080545	0.222805839
c5.pcaec444	-0.113253631	0.092481086	0.222822239
pcaac384.smc240	0.031735904	0.025916066	0.222840691
pcaec301.c12	0.051117342	0.041748692	0.222900461
smohc241.pcaac421	-0.017100996	0.013966927	0.222905606
smohc222.c141oh	0.090560358	0.073966142	0.222921896
pcaac322.lysopcac170	-0.011215379	0.009160745	0.222945219
pcaac425.pcaac422	-0.056171056	0.045887047	0.223009515
creatinine.pcaac426	-0.042294342	0.034550998	0.223010368
pcaec384.pcaec342	-0.113207024	0.092483648	0.223024414
smc240.pcaec322	-0.06476573	0.052915019	0.223069046
smohc141.pcaac421	-0.0263878	0.021559736	0.223076
putrescine.pcaec321	-0.04532322	0.037030868	0.223079052
c141.leucine	0.005327599	0.004353115	0.223105998
pcaec342.c14	-0.028225278	0.02306455	0.223146501
smohc222.kynurenine	-0.003935241	0.003216263	0.223224242
smc181.citruline	0.003027049	0.002474939	0.223399123
c16.lysopcac204	0.032445525	0.026528734	0.223416768
lysopcac182.pcaec423	-0.040943525	0.033477233	0.223419524
pcaac320.c141oh	0.083405726	0.068196572	0.223422018
smc261.pcaac300	0.070821252	0.057909564	0.22344324
pcaac383.citruline	0.004166231	0.003407642	0.223574671
pcaec422.pcaec343	0.083976546	0.068691966	0.223614211
pcaec384.pcaac365	0.033852739	0.027691973	0.223627086
c142.pcaec383	0.060499542	0.049499869	0.223724525
alphaaminoadipicacid.pcaec342	-0.062972605	0.051528599	0.223771948
pcaac341.pcaec446	-0.040430497	0.033094617	0.223932761
pcaac425.lysopcac160	-0.021024393	0.017216552	0.224117823
pcaec402.pcaac421	-0.046895775	0.038403315	0.224130844

pcaec446.pcaac321	-0.031571945	0.025857487	0.224183892
lysopcac161.pcaec360	0.003640922	0.002982667	0.224298869
c5.pcaac344	0.092616407	0.075875358	0.224319547
pcaec365.citruline	0.004148546	0.003399495	0.224431599
pcaac240.c141oh	0.11835304	0.096996349	0.224492696
pcaac322.phenylalanine	-0.04528162	0.037119251	0.224600192
lysopcac161.pcaec422	-0.065582148	0.053761152	0.224606232
pcaac421.citruline	-0.007004744	0.00574262	0.224643216
lysopcac180.alanine	0.050048297	0.041031373	0.224652746
pcaac425.smohc221	-0.051773072	0.042460039	0.224811681
c181.lysopcac160	0.012834189	0.010525928	0.22482815
pcaac300.pcaac421	0.018319814	0.015028295	0.224930829
c10.pcaec362	0.021095221	0.017306878	0.224979663
c141.pcaac381	-0.011039515	0.009057025	0.22498064
c181.phenylalanine	-0.047492708	0.03896478	0.224990976
pcaec403.pcaac381	0.033628565	0.027591214	0.225009226
smc181.pcaec360	-0.008906085	0.007307876	0.22505366
creatinine.lysopcac181	0.038079801	0.031248515	0.22508594
pcaec446.alanine	-0.010860539	0.008912597	0.225105257
kynurenine.pcaac405	0.036077965	0.029612749	0.225194259
lysopcac203.pcaec361	0.005547039	0.004553159	0.225210163
serine.pcaac404	0.005552626	0.004558028	0.225238727
c4.leucine	0.007488605	0.006147796	0.225281143
pcaac380.pcaec445	0.05518338	0.045323926	0.225494405
pcaac424.pcaec301	0.08822399	0.072467833	0.225536224
pcaac405.citruline	0.003400567	0.002793454	0.22556973
lysine.pcaec445	0.045679993	0.037529099	0.225624979
smc241.pcaac384	-0.075967301	0.062417305	0.225663458
putrescine.c141oh	0.111653218	0.091766451	0.225806272
pcaec385.glycine	-0.05000981	0.041108427	0.225873199
pcaec386.citruline	0.003092464	0.002542045	0.225876341
glutamicacid.kynurenine	-0.016976478	0.01395825	0.225987711
lysopcac182.c3	-0.072684141	0.059762841	0.225996419
pcaec405.pcaec363	0.034272993	0.028186563	0.226101157
c4.spermidine	0.066112636	0.054376399	0.226139298
pcaac381.pcaec343	0.077310566	0.063588091	0.226150961
smc240.pcaec404	0.016028241	0.013184751	0.226203191
pcaac403.pcaec424	-0.011769945	0.00968201	0.226208544
pcaec424.citruline	-0.00245986	0.002023655	0.226245869
asymmetricdimethylarginine.pcaac38	0.026508553	0.021812098	0.226336501
pcaac360.pcaec340	-0.074728943	0.061500496	0.226419831
alphaaminoadipicacid.lysopcac161	-0.076499064	0.062961144	0.226448242
lysopcac140.smohc161	0.01472355	0.012119321	0.22650063
pcaec423.pcaac365	0.069478038	0.05719653	0.226560612
putrescine.pcaec382	0.018372624	0.015124966	0.226561769

lysopcac140.c101	-0.011284607	0.009291703	0.226652884
pcaac300.c3	0.072913647	0.060038097	0.226662684
pcaac422.pcaac300	-0.074114946	0.061031997	0.226698525
pcaec425.pcaec402	-0.048781291	0.040170833	0.226704657
smc261.leucine	0.00666352	0.005488329	0.226788561
smc261.lysopcac161	-0.05116679	0.042149269	0.226858208
pcaec443.smohc221	-0.080938317	0.066682232	0.226915759
pcaec420.c0	-0.000685521	0.000564872	0.226994324
lysopcac140.kynurenine	-0.013154354	0.010839689	0.227012947
pcaac344.pcaec364	-0.011314138	0.009325093	0.227102806
pcaac386.pcaac380	-0.069311734	0.057127254	0.227107971
lysine.citruline	0.002345231	0.001933158	0.227155855
pcaac322.pcaac425	-0.105205796	0.086728957	0.227201502
pcaac365.pcaec342	-0.124348334	0.102516602	0.22723319
c101.smohc161	0.00703471	0.005799757	0.227243155
ornithine.citruline	0.002714043	0.002238217	0.227372842
pcaac362.pcaec300	0.005623601	0.004637717	0.227377464
pcaac300.pcaac343	-0.042622545	0.03515287	0.227411154
pcaec384.pcaac385	-0.029701461	0.024502685	0.22753301
pcaec385.pcaec364	-0.031447169	0.025943807	0.227550359
pcaec422.isoleucine	-0.021594986	0.017817428	0.227592874
smc240.glutamine	-0.029520071	0.024363983	0.227740675
serotonin.pcaec380	0.061155591	0.050475713	0.227757093
phenylalanine.glycine	-0.024620227	0.020322654	0.227801902
alphaaminoadipicacid.lysopcac203	0.019832313	0.016372142	0.227848496
pcaac385.lysopcac160	0.02459982	0.020308395	0.227860799
pcaac380.pcaec342	-0.138848385	0.114635772	0.227898781
glycine.pcaac402	0.077875785	0.064296141	0.227902122
pcaec446.tyrosine	-0.041019575	0.033868756	0.227929411
pcaac365.pcaec424	-0.004853585	0.00400807	0.22799816
pcaec365.pcaec406	0.062566912	0.051677684	0.228089447
h1.spermidine	0.061567854	0.050852744	0.228091631
c4.prolinr	0.022356995	0.018466806	0.228110797
pcaac363.pcaac420	0.065417708	0.054037022	0.228129677
asparagine.smohc222	0.006406719	0.005292224	0.228136541
pcaac384.pcaec405	-0.063530749	0.052483516	0.228175348
c182.pcaec322	0.06019957	0.049734309	0.228200647
pcaac362.pcaec445	0.088372621	0.07301458	0.228231713
smohc141.pcaec445	0.028204819	0.023305052	0.228268905
lysopcac181.smc181	-0.017109916	0.014140041	0.228349934
pcaec340.leucine	0.008889397	0.007347366	0.228410241
c102.pcaec405	-0.075599829	0.062492343	0.228459906
pcaec421.smohc222	0.016076741	0.013292423	0.228566638
lysopcac182.pcaac425	-0.069702798	0.057638521	0.228626831
c162.c5	-0.006176602	0.005108737	0.228734797

pcaac404.smohc222	-0.005533088	0.004577284	0.22881621
pcaec321.c8	0.04421376	0.036576167	0.228816713
citruline.pcaec342	0.064724344	0.053555848	0.228921724
pcaac363.arginine	0.066752253	0.055252485	0.229078094
pcaec342.kynurenine	-0.007383866	0.006111922	0.229086716
pcaec445.lysopcac203	-0.011723599	0.009706752	0.229213643
c14.smc202	-0.030390223	0.025165986	0.22928518
phenylalanine.c102	0.025241112	0.020903748	0.229323169
c141.smohc161	0.010097638	0.00836279	0.229339892
pcaac281.glutamine	-0.015443914	0.012794126	0.229469907
c102.tyrosine	0.066678779	0.055238495	0.229470925
pcaac406.pcaac385	-0.032208272	0.026683105	0.229486658
methioninesulfoxide.pcaec403	0.085520833	0.070851146	0.229493073
methioninesulfoxide.leucine	-0.031182846	0.025834429	0.22950177
pcaac300.pcaac344	-0.101459736	0.084066094	0.229548745
lysopcac203.citruline	-0.00430216	0.00356485	0.229578185
serine.asparticacid	0.030244725	0.025064425	0.22963512
pcaec445.c18	-0.025966392	0.021522821	0.229719939
pcaac386.citruline	0.003683657	0.003053855	0.22980695
c4.pcaec422	-0.036167184	0.029995515	0.22999103
pcaec401.pcaec445	0.039983958	0.033167134	0.230077001
pcaec422.smohc221	-0.099495712	0.082534696	0.230087701
smc261.spermidine	0.062915727	0.052193342	0.230112994
smc180.leucine	0.007349399	0.006097373	0.230150422
ornithine.pcaac384	-0.056076868	0.046530431	0.230217015
pcaec383.pcaec342	0.088009925	0.073032606	0.230250984
c10.h1	0.006605041	0.005481394	0.23028345
lysopcac161.lysopcac181	0.110800614	0.091955965	0.230307135
c5.leucine	0.016782737	0.013928911	0.230324891
pcaec360.glycine	0.040232308	0.033392952	0.230352106
pcaec321.leucine	0.005423833	0.004501822	0.230354378
citruline.pcaec322	-0.054966052	0.045623618	0.230368456
c141.pcaec424	0.01217759	0.010107822	0.230369572
serine.pcaec361	0.006102796	0.005065561	0.230372252
c10.serotonin	0.085318078	0.070818547	0.230379961
c141oh.pcaac404	0.003021711	0.002508594	0.230456187
pcaac405.pcaec424	-0.004326846	0.003592813	0.230547867
lysopcac181.pcaec341	0.0436551	0.036252828	0.230594547
lysopcac161.smc180	0.017004982	0.014124921	0.230704492
pcaac384.asparticacid	0.02742558	0.022781764	0.230727651
pcaec343.pcaac240	-0.013052175	0.010842212	0.230731336
smohc141.smohc221	0.035925641	0.029845598	0.230774886
h1.leucine	0.008882109	0.007379582	0.230817549
pcaac361.pcaec361	-0.012650786	0.010511814	0.230865041
pcaec382.pcaec361	0.016863112	0.01401214	0.230872096

pcaac344.smohc222	0.006122545	0.005087718	0.230898072
asymmetricdimethylarginine.c141oh	0.096026499	0.079801343	0.230928011
c101.lysopcac204	0.017431023	0.014486207	0.230941574
pcaec405.pcaec443	0.153119174	0.127252854	0.23094815
pcaac384.citruline	0.002441711	0.002029306	0.230964531
citruline.phenylalanine	0.03808629	0.03165435	0.230976758
pcaec384.citruline	0.009490447	0.007887962	0.230991098
c2.pcaac381	-0.005598856	0.004654058	0.231049168
pcaec404.pcaac381	0.0366611	0.030478317	0.23110577
glutamicacid.c102	0.048256086	0.040118065	0.23110833
smohc222.pcaec445	0.026391072	0.021941123	0.231123213
pcaac342.pcaac300	-0.069604901	0.057871597	0.231148686
pcaac300.lysopcac280	0.008448473	0.007024342	0.231150565
pcaac385.citruline	0.00252703	0.002101317	0.231208015
lysopcac182.pcaac426	-0.062931816	0.052340732	0.231302428
citruline.serotonin	0.046018441	0.038276747	0.231338331
smc161.arginine	-0.05702635	0.047433396	0.231344231
lysopcac140.putrescine	0.035320771	0.029380308	0.231362972
ornithine.pcaec380	-0.040057609	0.033321193	0.231372958
pcaac321.pcaec446	-0.036157013	0.030083761	0.231484171
c141.c4	-0.036181646	0.030105627	0.231505317
smohc221.leucine	0.007762529	0.006458981	0.231506811
c51.c102	0.026658263	0.022181666	0.231508472
pcaac420.pcaec404	-0.011674145	0.009714155	0.231527331
c162.pcaac362	0.03113332	0.025909683	0.231587944
pcaac343.serotonin	0.121461727	0.101093368	0.231637711
isoleucine.pcaac405	0.054973337	0.045766606	0.231758934
pcaec405.c14	-0.05697928	0.047439959	0.231791799
pcaac404.citruline	0.007299326	0.006077365	0.231797397
lysopcac170.pcaac384	-0.040983399	0.034127149	0.231860964
smc261.tyrosine	-0.06091985	0.050734531	0.23191741
pcaec425.lysopcac180	0.038515993	0.032077106	0.23192731
pcaec322.pcaac402	-0.070073642	0.058371792	0.232028097
smc161.pcaac402	-0.035792915	0.02981851	0.232071441
pcaac323.citruline	0.002293829	0.001911146	0.232118605
c142.pcaec380	-0.054947763	0.045789807	0.232210405
pcaec401.pcaec322	-0.084007892	0.070008805	0.232225149
h1.lysopcac182	-0.035703152	0.029754914	0.232246075
pcaac405.lysine	-0.08390423	0.06994008	0.232342476
lysopcac181.asparticacid	0.01901304	0.015849393	0.232362591
pcaac405.histidine	0.050452221	0.04206069	0.232400063
pcaec380.pcaac362	-0.044589505	0.037174694	0.232420017
pcaec444.pcaac300	-0.057426	0.047876841	0.232422335
c12.lysopcac181	0.080316442	0.066961051	0.232423277
pcaec363.lysopcac204	-0.020830608	0.017367031	0.232429524

lysopcac281.c8	0.052365642	0.043666228	0.232510417
kynurenine.pcaec343	0.04254913	0.03548702	0.23259558
lysopcac203.c101	-0.036148438	0.030151483	0.232638702
pcaac366.smohc222	0.011410705	0.009520402	0.232771028
pcaec384.pcaec361	-0.014008242	0.011688104	0.232789899
pcaec444.spermidine	0.061485741	0.05130255	0.23279428
pcaec300.pcaac381	-0.027594308	0.023027079	0.232852917
pcaec384.pcaec402	-0.053944408	0.045016032	0.232854463
creatinine.pcaac421	0.009492933	0.007921763	0.232855159
c18.pcaec361	-0.015848124	0.01322891	0.232988707
c182.pcaec423	-0.024653775	0.020579831	0.233001749
pcaac320.lysopcac170	0.012994605	0.010847379	0.23300533
smc160.pcaac384	-0.05259881	0.043910627	0.233039568
kynurenine.serotonin	0.076894137	0.064200927	0.233097752
pcaec443.pcaac381	0.02518389	0.021038015	0.233348141
pcaec300.c162	-0.076266971	0.063719287	0.233404305
pcaec404.pcaac300	-0.063757204	0.053269461	0.23341997
c18.leucine	0.006980617	0.005833029	0.233474894
pcaac363.leucine	0.008439794	0.007053238	0.233535226
alanine.pcaac380	-0.040749741	0.034058361	0.233580449
c12.pcaec424	0.007573847	0.006330528	0.233606744
glycine.c2	0.064634765	0.054025284	0.233614709
putrescine.c8	-0.036879765	0.030827421	0.233634165
c4.pcaec360	0.002718775	0.002273882	0.233897215
pcaec445.c16	-0.050844489	0.042531148	0.233970686
lysopcac161.citruline	0.008077441	0.006757043	0.233991757
lysopcac161.kynurenine	-0.008535665	0.007140894	0.234026395
pcaac426.c51dc	-0.127357966	0.106567763	0.234116952
lysine.pcaec405	0.049698751	0.041589984	0.2341636
pcaac365.pcaec444	0.115912311	0.097001413	0.234169198
pcaac424.pcaec343	0.066111973	0.055329292	0.234197591
pcaec424.pcaec402	-0.050560391	0.04231562	0.234213976
smc261.smc260	-0.046648765	0.039043351	0.234231813
smc202.pcaec361	-0.006975924	0.005838689	0.234238905
pcaec423.pcaac323	-0.071674812	0.059997482	0.234295604
pcaec361.pcaac405	-0.03835131	0.032105187	0.234326189
lysopcac140.pcaec404	0.02128242	0.017816792	0.234340672
pcaec421.pcaac406	0.047183013	0.039514826	0.234518463
pcaac362.c162	-0.06602762	0.055301302	0.234556318
lysopcac204.c51	-0.063073883	0.052835015	0.23462337
putrescine.citruline	-0.003848146	0.003224441	0.234763421
pcaec362.pcaec363	0.030352091	0.025435754	0.234820341
pcaac342.pcaac406	-0.045679152	0.038282734	0.234851344
pcaac361.lysopcac160	-0.033543926	0.028117874	0.234941069
smc241.leucine	0.005850388	0.00490405	0.234942765

c14.c162	-0.091676824	0.076847607	0.234943421
pcaec446.pcaec385	-0.084564713	0.070926617	0.235210747
lysopcac170.pcaec422	-0.028894997	0.02423543	0.235219347
pcaec443.pcaac343	0.012413902	0.010412361	0.235233066
c162.smc180	-0.010147499	0.008511476	0.235238393
histidine.pcaac426	-0.023506872	0.019717854	0.235258734
pcaac344.pcaac281	-0.003420439	0.002869637	0.235344806
pcaec364.pcaac424	-0.032483405	0.027256496	0.235412824
pcaac402.pcaec380	0.15660935	0.131437674	0.235513358
c102.lysopcac160	0.01641996	0.013781905	0.235550876
pcaac403.pcaec380	0.135644541	0.113880783	0.23566994
putrescine.pcaac381	-0.03880031	0.032576153	0.235687624
alanine.pcaac281	0.002534174	0.002127815	0.23572297
pcaac401.pcaec343	0.087996875	0.073913751	0.23589515
serotonin.prolinr	-0.025179347	0.021150323	0.235910747
pcaec424.pcaac300	-0.052510709	0.04410834	0.23591107
pcaec301.asparagine	0.014226856	0.011950796	0.235927248
c141.pcaec405	-0.030722931	0.025808196	0.235934875
c51.kynurenine	-0.00452152	0.003798732	0.235998483
c3.pcaec342	-0.067960069	0.057110098	0.236110988
asymmetricdimethylarginine.acetylor	0.037596323	0.031595296	0.236130276
serine.citruline	0.005459543	0.00458881	0.236201881
pcaac425.pcaac323	-0.053491991	0.044962068	0.236216631
pcaec386.pcaec443	0.054139506	0.045512419	0.23627902
pcaec386.pcaac421	-0.031862727	0.026785605	0.236282127
c142.pcaac281	0.001402762	0.001179274	0.236295278
asymmetricdimethylarginine.methior	0.017644318	0.014834145	0.236324127
serotonin.citruline	0.005793492	0.004871065	0.236351929
serine.c141	-0.03117607	0.026224638	0.236571607
pcaec420.c2	0.043752432	0.036806402	0.236607082
lysopcac160.leucine	-0.009878551	0.008310326	0.236610984
pcaec300.transhydroxyproline	-0.031051514	0.026122855	0.236624992
phenylalanine.pcaac321	0.024449706	0.02056932	0.236634066
pcaac422.glycine	-0.019756999	0.016623102	0.236682167
pcaac341.lysopcac160	-0.020811395	0.017510549	0.236690204
pcaec424.pcaec444	0.162585407	0.136817785	0.236757237
c8.pcaac240	-0.016254222	0.013679007	0.236786631
pcaec363.smc161	-0.056463541	0.047522384	0.236831469
pcaec340.citruline	0.00515456	0.004339544	0.236962893
c102.pcaac281	0.002201457	0.001853504	0.236996024
pcaac383.pcaac281	0.00378164	0.003184257	0.237044014
histidine.smc240	0.06723738	0.056633895	0.237191918
pcaac360.pcaec424	-0.013029184	0.010978117	0.237347682
pcaec424.c14	-0.058640605	0.049413118	0.237383362
lysopcac281.pcaac420	0.108885224	0.091753955	0.237396285

pcaec322.pcaac426	0.075113774	0.063301434	0.237437247
c181.pcaac421	-0.016197932	0.013651535	0.237467258
pcaec341.leucine	0.005794591	0.004883819	0.237483334
smc261.pcaac425	-0.090731736	0.076477717	0.237525082
pcaac364.pcaac380	-0.050569286	0.042625813	0.237536003
pcaac426.methionine	-0.035909132	0.030269501	0.23755159
pcaec446.pcaac380	-0.084673814	0.071378061	0.237567993
pcaac341.pcaac385	-0.029875895	0.025186309	0.237598068
lysopcac203.pcaec425	0.01745902	0.014720035	0.237646825
pcaec301.pcaec424	-0.007819579	0.006594231	0.237745559
lysopcac203.pcaac322	-0.084396981	0.071172224	0.237748572
pcaac361.smc161	-0.048077063	0.040555631	0.237887895
lysopcac204.lysopcac160	0.014759472	0.012455001	0.238059596
c18.c5	-0.014174022	0.011961709	0.23808883
pcaac322.citruline	0.00540901	0.004565259	0.238139912
pcaac402.h1	0.005755264	0.004857571	0.23814662
pcaac240.phenylalanine	-0.070965469	0.059897756	0.238156947
lysopcac140.leucine	-0.010716815	0.009046122	0.238192684
pcaec340.histidine	0.060168986	0.050796959	0.238266149
c16.pcaac403	-0.00674234	0.00569223	0.238273426
pcaac421.smohc221	0.043561652	0.036778876	0.238297427
pcaec364.c141oh	0.113892802	0.096162512	0.238314
lysopcac170.pcaec342	-0.082360505	0.069546253	0.238362628
c18.citruline	-0.001946241	0.001643466	0.238372674
pcaec380.lysopcac181	0.055129889	0.046557331	0.23841234
c5.prolinr	0.033516018	0.028306382	0.238445674
asparagine.smohc221	-0.057406643	0.048486728	0.238476625
acetylornithoine.glutamicacid	-0.063949579	0.054014583	0.238490195
pcaac343.asparagine	0.015391745	0.013001298	0.238517631
c101.methioninesulfoxide	0.025211793	0.021299286	0.238584811
c51dc.pcaac421	-0.009536376	0.008056704	0.238598513
putrescine.smc180	0.040346464	0.034091784	0.238674044
acetylornithoine.pcaac405	0.052439357	0.044311956	0.238694823
pcaac383.histidine	0.049290531	0.041654778	0.23873544
asymmetricdimethylarginine.glycine	-0.054966716	0.046452463	0.238743656
c14.pcaac381	-0.005851791	0.004945641	0.238770295
pcaac240.pcaac364	0.065526235	0.055379536	0.238770694
c10.c8	0.189306482	0.159994616	0.238776923
pcaac321.pcaec401	0.050118761	0.042358752	0.238780096
pcaac341.leucine	0.006344687	0.005362471	0.238792899
pcaec383.pcaec363	0.028414994	0.024016526	0.238801391
pcaec444.citruline	-0.002071136	0.001750582	0.238813509
pcaac364.pcaac344	0.07344431	0.062098437	0.238973422
pcaec382.pcaac422	-0.168084563	0.142132082	0.239018379
pcaac361.prolinr	0.054262273	0.045885022	0.239027706

smohc241.pcaec364	-0.024649413	0.020844619	0.239043234
c51.pcaec443	0.043862633	0.037094125	0.239068029
pcaac240.pcaec424	-0.005617601	0.004750746	0.239068588
citruline.c182	0.067828948	0.057363857	0.239081837
pcaec364.leucine	0.006678581	0.005649469	0.239189607
pcaac321.pcaac322	0.098354492	0.083199662	0.239193875
pcaac342.pcaec300	0.006490897	0.005491034	0.239217632
smc181.leucine	0.009744895	0.008244151	0.239238428
lysopcac170.asparticacid	0.01801538	0.015241501	0.239255166
pcaec380.c18	-0.050192702	0.042470633	0.239323883
c3.leucine	0.014176244	0.011995288	0.239325295
pcaec383.pcaec361	0.013097562	0.011082891	0.239339422
creatinine.smc161	-0.076278594	0.064561499	0.239456029
acetylornithoine.pcaec425	-0.009222869	0.007806936	0.239503004
lysopcac204.lysopcac203	-0.009494377	0.008038284	0.239591602
asparticacid.citruline	0.006235152	0.005279646	0.239657099
pcaac366.smohc221	0.055524106	0.047020876	0.239712494
pcaec361.pcaac403	0.019816101	0.016784315	0.239794628
pcaac240.pcaac404	0.015680982	0.013282167	0.239805677
glycine.asparticacid	0.031997097	0.02710497	0.239851614
lysopcac182.pcaec445	0.041726854	0.035356469	0.239975353
lysopcac140.pcaec424	-0.003919777	0.003321657	0.240018668
pcaac385.pcaec424	-0.004562489	0.003866951	0.240097747
pcaac320.pcaac380	-0.081579242	0.069150921	0.240153124
pcaac401.pcaac365	0.083764364	0.071018084	0.240251527
pcaac401.pcaac405	-0.069568472	0.058987558	0.240292781
c4.pcaac322	0.083984869	0.071212222	0.240298741
pcaac366.c141	-0.062485174	0.05298536	0.240326128
pcaac424.c162	0.063432146	0.053789094	0.240332502
c16.leucine	0.00877978	0.007445215	0.240341936
c51.pcaec403	0.042788192	0.036287658	0.240386554
pcaac343.citruline	0.008866061	0.007519452	0.240408614
glutamine.smc260	0.027900165	0.023663998	0.240436483
pcaac385.lysopcac181	0.026260334	0.02227573	0.240490739
c8.leucine	0.00955413	0.008105647	0.24056058
pcaac342.pcaec424	-0.005037121	0.004273803	0.240598969
serine.lysopcac180	0.041594016	0.035301031	0.240732999
pcaec424.acetylornithoine	-0.087965568	0.07466107	0.240759898
c101.citruline	-0.003109484	0.002639264	0.240774033
pcaec446.citruline	-0.003769806	0.003199778	0.240780771
pcaac361.pcaac406	0.04117398	0.034950097	0.240807355
c16.c51dc	0.067224904	0.05706867	0.24085265
glutamine.pcaac321	-0.012839676	0.010900727	0.240889123
pcaec361.pcaec424	-0.006904993	0.005863968	0.241025936
c142.pcaac384	0.052774308	0.044823201	0.241081967

pcaec404.lysopcac161	0.046181088	0.039225405	0.241106708
pcaac362.pcaac320	-0.055905991	0.047492554	0.241175593
smc160.smohc221	0.039826174	0.033839184	0.241266277
asparticacid.pcaac381	0.025300396	0.021497724	0.241281216
pcaac426.pcaec424	-0.00580883	0.004936298	0.241332547
c2.pcaac323	0.049101161	0.041725805	0.241332768
pcaac403.pcaec322	0.061220253	0.052032347	0.241403201
lysopcac170.pcaac406	0.041306348	0.035107674	0.241410713
pcaac420.pcaac405	-0.051595156	0.043855778	0.241445899
smohc161.leucine	0.009173512	0.007798726	0.241521508
smohc141.pcaac381	-0.027051053	0.022997838	0.241537484
pcaec343.smohc161	-0.008866475	0.007538172	0.241550626
pcaec361.citruline	-0.020956194	0.017822627	0.241706221
leucine.pcaec301	0.045873228	0.03901514	0.241721931
pcaac405.smc161	-0.04551975	0.0387173	0.241755724
pcaec361.pcaac322	0.058428431	0.049709392	0.241873318
pcaac341.pcaec401	0.064049074	0.05449326	0.241890265
pcaac321.lysopcac280	0.015436199	0.013133761	0.241910547
pcaec380.c14	-0.066340282	0.05644546	0.241913745
lysopcac140.citruline	0.005149152	0.004381878	0.24199242
lysopcac182.citruline	-0.003584163	0.003050248	0.242017013
c142.lysopcac280	-0.010819308	0.009207904	0.24203196
pcaec406.smc161	0.131509801	0.11192716	0.242049298
pcaac365.pcaec382	-0.014476909	0.012321445	0.242058405
pcaec423.pcaec424	-0.023201427	0.019747496	0.242070856
c51dc.serotonin	0.08807389	0.074962624	0.24207113
pcaec360.smc160	0.10189711	0.086747573	0.242176709
pcaac406.smohc221	0.029252211	0.024906358	0.242237193
pcaac362.arginine	0.06074353	0.05172259	0.242268175
c12.pcaac300	-0.058638551	0.049930588	0.242271637
methionine.pcaac421	0.018711992	0.015933396	0.242276886
pcaac406.c0	0.002458318	0.002093688	0.242369331
c18.pcaec402	-0.022571222	0.019226433	0.242444513
smc202.glutamicacid	0.052517903	0.044742168	0.242515726
c16.smohc161	0.032315338	0.027531246	0.242523842
serine.pcaac365	-0.043875257	0.037388576	0.24263401
smc181.pcaec343	0.065672859	0.055964542	0.242642405
histidine.c12	0.084204503	0.071760403	0.242666712
h1.lysopcac181	0.102946151	0.087739618	0.242705649
c162.pcaec300	-0.027782426	0.023678773	0.242709312
lysopcac204.c51dc	-0.116149883	0.098999849	0.242738167
c18.pcaac365	0.038205229	0.032568842	0.242806989
pcaac383.lysopcac280	0.006037327	0.005146727	0.242814447
serine.glutamicacid	0.034876993	0.029732647	0.242823222
citruline.kynurenine	-0.037522394	0.031994437	0.242919708

c3.pcaec341	0.04382082	0.037366091	0.24293399
pcaec444.transhydroxyproline	-0.028797677	0.024557189	0.242959938
pcaec403.smohc221	-0.068845855	0.058717504	0.243033986
pcaac281.lysopcac160	-0.013204709	0.011263899	0.243109668
c101.pcaac240	-0.010374829	0.008851822	0.243208801
smc260.pcaac402	0.079655774	0.067969361	0.243256557
smohc221.arginine	-0.035413181	0.030221539	0.243316712
lysopcac281.citruline	0.006725901	0.005739976	0.243325344
smc260.leucine	0.005552084	0.0047388	0.24338228
lysopcac180.serotonin	-0.078907241	0.06735711	0.243440854
c8.serotonin	0.095519632	0.081538465	0.243444416
pcaac343.lysopcac170	-0.011450348	0.009774635	0.243457364
lysopcac281.pcaec405	-0.092172445	0.0786857	0.243471222
lysopcac180.serine	-0.047651982	0.040689962	0.243591767
pcaec402.pcaec385	-0.062638301	0.053492003	0.243637746
pcaac364.pcaec424	-0.00500722	0.004276253	0.243656979
methionine.pcaac384	-0.046154233	0.039419325	0.243690416
pcaac364.citruline	0.004118425	0.003517499	0.243695958
pcaac281.pcaec405	-0.056887447	0.048596059	0.243784347
pcaac383.pcaec446	-0.039983543	0.034162039	0.243868517
c162.smc160	0.062302935	0.053231938	0.243869869
smc161.pcaac406	0.021465079	0.018339908	0.243870836
phenylalanine.pcaac403	0.009146963	0.007815983	0.243916272
pcaec421.pcaec423	0.157582008	0.134652837	0.2439186
smc240.h1	0.006100988	0.005215083	0.244083026
pcaec420.pcaac321	-0.042371921	0.036222093	0.244120117
spermidine.c102	0.122822996	0.105020778	0.244228348
pcaac341.pcaac404	0.003558329	0.003042647	0.244239109
c101.c16	0.041980792	0.035903738	0.244329385
pcaec300.smohc222	0.004844484	0.004143987	0.244417784
isoleucine.leucine	0.043360031	0.037091392	0.244431521
pcaec421.c141oh	0.110400573	0.09445157	0.24449022
citruline.pcaac240	-0.024336088	0.020821527	0.244516111
pcaac323.c14	-0.015182295	0.012990743	0.244553688
pcaac360.c141oh	0.098161518	0.084002933	0.244614881
pcaac344.c102	0.032714336	0.027996365	0.244626079
pcaac425.pcaac343	0.019209985	0.016439643	0.244628038
glycine.pcaac322	0.054633042	0.046756025	0.244646248
serine.c14	0.065376342	0.05595733	0.244704799
smohc141.pcaec443	0.070994341	0.060770003	0.244736323
glutamicacid.citruline	0.014550172	0.01245533	0.244759651
lysopcac140.pcaec443	0.073700205	0.063090176	0.244766075
methionine.lysopcac160	-0.03771867	0.032288901	0.244770228
pcaec444.leucine	0.005948648	0.005092532	0.244790344
c162.pcaac381	-0.006733881	0.005765433	0.244845475

smc181.pcaac341	-0.00508331	0.004352244	0.244845687
lysopcac161.pcaac320	-0.112672399	0.096474996	0.24487871
pcaac385.pcaec383	-0.066185185	0.056683214	0.244982993
pcaac420.pcaec422	-0.030403542	0.026044699	0.245092724
pcaac300.pcaac403	0.009730251	0.008338148	0.245255359
smohc222.leucine	0.012905609	0.011060579	0.245313586
lysopcac161.leucine	-0.018719382	0.016043853	0.245332821
pcaac421.pcaec405	-0.081065594	0.0694807	0.245344118
pcaac385.smc241	0.065977188	0.056551172	0.245365958
pcaec382.pcaac381	0.04526354	0.038799747	0.245401183
pcaac380.leucine	0.007135725	0.00611805	0.245503436
lysopcac204.lysine	0.050701616	0.043474531	0.245544751
c3.pcaac300	0.049162825	0.042156231	0.245557557
lysopcac140.pcaec341	0.061328267	0.052589039	0.245567937
serotonin.lysopcac161	-0.072479409	0.062155305	0.245599331
pcaac343.lysopcac280	0.01285031	0.011020653	0.245631932
c5.pcaac402	0.049679346	0.042615217	0.24573486
c142.c102	0.054319607	0.046599362	0.245772199
c16.c162	-0.129227962	0.110862442	0.24577717
pcaec361.pcaec445	0.0395078	0.033895428	0.245809996
pcaec402.smc260	-0.037725952	0.032368376	0.245834257
c8.c16	0.049794031	0.042727954	0.245892797
pcaac406.pcaec301	0.06972014	0.059834818	0.245958703
pcaac426.citruline	0.004705319	0.004038473	0.245993709
c51.pcaac426	0.033327798	0.028605206	0.246004941
pcaec425.c51dc	-0.113421543	0.097364834	0.246078659
asparticacid.pcaac405	-0.037908168	0.032542234	0.246087309
c10.leucine	0.0101701	0.00873061	0.246092521
smohc222.phenylalanine	-0.042300828	0.036322277	0.246205945
methioninesulfoxide.pcaec364	0.004575194	0.003928742	0.246227433
creatinine.leucine	0.009139016	0.007849481	0.246332912
lysopcac182.pcaac365	0.04036704	0.034673501	0.246364615
c181.pcaac381	-0.008121575	0.006977023	0.246428573
methioninesulfoxide.histidine	-0.098404885	0.084539537	0.246442999
pcaac424.pcaec402	-0.113899837	0.097858009	0.246475544
lysopcac203.putrescine	0.041314225	0.035495939	0.246481807
c182.pcaac381	-0.008645062	0.007427899	0.246502138
lysopcac204.c142	0.032850065	0.028225017	0.246502273
alanine.c102	0.038664042	0.033236385	0.246728066
pcaac360.lysopcac180	0.076326729	0.065642579	0.246947276
serotonin.pcaac344	0.057760311	0.049679424	0.24698847
pcaec340.pcaac381	-0.050040031	0.043042017	0.247018812
pcaac362.transhydroxyproline	0.021713329	0.018679104	0.247077905
pcaac406.smc261	0.04039529	0.034750841	0.247083293
arginine.pcaec422	0.02594983	0.022325808	0.247124524

pcaac342.transhydroxyproline	0.019782762	0.017024162	0.247239499
pcaac404.leucine	0.017626344	0.015168874	0.247252793
pcaec402.pcaac323	-0.072872758	0.062722958	0.247328871
acetylornithoine.citruline	0.011923169	0.010264888	0.247438501
c51dc.citruline	-0.00324161	0.00279081	0.247446097
pcaec381.pcaec361	0.011016416	0.009486536	0.247552074
smc181.pcaac404	0.008375667	0.007212648	0.247560687
pcaac401.pcaac343	0.013727618	0.011822552	0.247604798
pcaec322.leucine	0.011038976	0.009507164	0.247611461
asymmetricdimethylarginine.pcaac38	-0.081110864	0.069857778	0.247626138
pcaec425.pcaac402	-0.099640855	0.085838867	0.247746181
pcaec384.leucine	0.006674168	0.005750987	0.247853112
pcaac402.lysopcac170	-0.039655247	0.034172278	0.247883506
c8.pcaec425	-0.056986426	0.049114617	0.247955106
pcaac420.pcaec405	-0.056998417	0.049134668	0.248048181
pcaec382.pcaac300	-0.077961038	0.06722899	0.248214809
pcaac366.tyrosine	0.054118912	0.046673543	0.248261131
smohc221.lysopcac281	0.019921594	0.017181387	0.248274602
smohc141.c141	-0.024579202	0.021198799	0.248284735
pcaac424.pcaac405	-0.041312921	0.035633435	0.248315408
smohc161.transhydroxyproline	-0.01770967	0.01527624	0.248352483
pcaec443.tyrosine	-0.053293606	0.045978639	0.248433804
pcaec322.pcaec444	0.121036154	0.104447426	0.24854397
ornithine.pcaec300	0.019193993	0.01656402	0.248563241
methioninesulfoxide.pcaac385	-0.054798517	0.047290201	0.248565363
pcaec381.smohc222	-0.017265084	0.01490221	0.2486517
c3.pcaec340	0.026447512	0.02282797	0.248652223
alanine.tyrosine	0.065479721	0.056522437	0.248686464
pcaac360.pcaac322	0.064609566	0.055775246	0.248719661
pcaac344.pcaec445	0.062762151	0.054181905	0.248732429
pcaec341.pcaec364	-0.023478045	0.020268702	0.248740627
asymmetricdimethylarginine.pcaac40	0.068679209	0.059294638	0.248768961
pcaac363.pcaac421	0.024279081	0.020961607	0.24877134
pcaec405.c141oh	0.081293172	0.070186647	0.248780116
citruline.pcaec341	-0.03294295	0.028446305	0.248848366
histidine.spermidine	-0.065803325	0.056821815	0.248852588
pcaec321.smc161	-0.064115379	0.05536526	0.248861121
pcaec402.pcaec380	0.083491986	0.072100657	0.248882044
lysopcac170.serotonin	-0.10398479	0.089798447	0.248887009
putrescine.smohc161	0.009554354	0.008251222	0.248906466
pcaec405.pcaac281	-0.005157738	0.00445504	0.248988235
c141oh.pcaac381	-0.010191221	0.008803047	0.249003895
ornithine.isoleucine	0.023355476	0.020175493	0.249034956
pcaec382.lysopcac204	-0.111410832	0.096245173	0.249052427
c102.pcaac365	0.023865757	0.020618234	0.249079224

histidine.lysopcac204	-0.0141402	0.012216181	0.249083183
glycine.lysopcac280	0.009254672	0.007996893	0.249170301
pcaac426.kynurenine	-0.010943135	0.009455952	0.249173865
pcaec364.alanine	-0.022226722	0.019207784	0.249215426
pcaac385.pcaec341	0.068189041	0.058930429	0.249240586
pcaec422.pcaec322	0.066141881	0.057161446	0.249242361
glutamine.prolinr	0.027947925	0.024154841	0.249272681
alphaaminoadipicacid.pcaac383	-0.011156839	0.009643553	0.24931747
lysopcac140.histidine	0.057289134	0.049523583	0.249365092
citruline.c142	-0.041373697	0.035765491	0.249365148
pcaac421.pcaac405	-0.045592741	0.039418325	0.249433054
pcaec340.pcaac404	0.010697365	0.009249482	0.249474413
pcaac342.pcaac421	0.014104106	0.012196093	0.249511928
pcaac422.pcaec424	-0.013387421	0.011576699	0.249525668
prolinr.pcaac421	0.012913211	0.011167053	0.249543586
pcaac300.citruline	0.004472776	0.003868072	0.249557696
pcaac240.pcaec342	0.076864774	0.066474505	0.249568995
citruline.leucine	0.007175411	0.006207677	0.24973673
pcaec341.smc161	-0.05128136	0.044368742	0.249774957
pcaec301.pcaac421	0.01156385	0.010008124	0.249918911
pcaac363.smohc222	-0.036991604	0.032017227	0.249951792
pcaac341.smohc222	0.006800204	0.005885838	0.249958115
c8.pcaac406	0.044730076	0.038719981	0.250011431
pcaec383.pcaac381	0.03747038	0.032439129	0.250060914
smc261.lysine	0.072273005	0.062570787	0.250076554
smohc241.pcaac344	-0.060732875	0.052585347	0.250125823
c142.lysopcac160	0.013220611	0.011450638	0.25027479
methioninesulfoxide.lysopcac181	0.079702796	0.069043934	0.250354927
kynurenine.arginine	0.025787707	0.022339578	0.250366083
pcaec362.glutamine	-0.014790077	0.012812584	0.250370488
isoleucine.c3	0.037077138	0.032123421	0.250423985
serotonin.pcaec404	0.018917791	0.016390691	0.250436208
pcaec423.c18	-0.032712756	0.028348111	0.25052321
citruline.pcaac322	-0.038975764	0.033777886	0.250556687
pcaac366.c16	0.024945839	0.021619538	0.250568052
putrescine.lysopcac204	-0.022858567	0.019817736	0.250738223
pcaec446.histidine	0.043248823	0.037497943	0.25076876
pcaec385.leucine	0.005425182	0.004704167	0.250806999
pcaec301.smc202	0.036582493	0.03172477	0.250868699
pcaec340.c101	-0.010576634	0.009174795	0.251003044
pcaac365.citruline	0.004522026	0.003923417	0.251092741
lysopcac140.c102	0.03430227	0.029766015	0.251164804
pcaac422.pcaac425	-0.104458463	0.09066221	0.251256954
alanine.pcaac426	-0.044499082	0.038623274	0.251273634
pcaac385.smc261	0.061834181	0.053672362	0.251299775

pcaec404.pcaec364	0.006094801	0.005290368	0.251304456
pcaec421.pcaac424	-0.151662442	0.131666993	0.251383177
pcaec445.pcaac380	-0.072839582	0.063267576	0.251616614
pcaec382.pcaec380	0.110807827	0.096251171	0.251640338
lysopcac182.leucine	-0.006926604	0.006019859	0.251890703
pcaec365.pcaec360	-0.002994449	0.00260271	0.251937253
pcaec444.arginine	-0.034289979	0.029804686	0.251946477
pcaec341.lysopcac160	-0.024944628	0.02168179	0.251947325
pcaac402.pcaac343	0.013843932	0.012034148	0.251988396
pcaec385.pcaec341	0.060777079	0.052833011	0.251998881
lysopcac140.pcaec401	-0.069010872	0.059994193	0.252027308
leucine.c18	0.041921395	0.036445627	0.25204687
arginine.pcaec444	0.095546077	0.083067328	0.252055218
pcaec386.lysine	-0.093808385	0.081574527	0.252159037
smc180.pcaec361	-0.010591163	0.009209957	0.252160185
pcaec420.lysopcac203	-0.00844398	0.007343417	0.252200439
pcaec386.pcaec360	-0.004200457	0.003653048	0.252208897
pcaac365.lysopcac281	0.018386646	0.015993156	0.252288107
pcaac343.smohc222	0.009217794	0.008019212	0.252367523
pcaac321.citruline	0.006341288	0.005517913	0.252468289
lysopcac180.pcaec340	0.054723043	0.047625109	0.25254257
smc161.asparagine	0.013824727	0.012035061	0.252679578
pcaac344.citruline	0.003287481	0.002862183	0.252725743
spermidine.citruline	0.021581947	0.018790803	0.25274803
pcaec360.citruline	0.007608731	0.006624734	0.252749678
pcaac320.pcaac421	0.016338633	0.014227878	0.252823801
pcaec300.lysopcac204	-0.018154344	0.015811734	0.252904829
pcaac406.pcaac380	-0.059217055	0.051577703	0.252922527
pcaac320.citruline	0.005941445	0.005176265	0.253041339
pcaac240.smc160	0.071958526	0.062691783	0.253045593
pcaec381.c18	0.051702943	0.04504801	0.253080374
pcaac385.pcaec384	0.07559809	0.065885961	0.253212757
pcaec446.c14	-0.037625644	0.032793458	0.253235832
c12.lysopcac140	-0.00332106	0.002894963	0.253304385
pcaec384.pcaec380	0.040784762	0.035553841	0.25332863
pcaec443.asparticacid	0.052649464	0.045900861	0.253370274
c10.glycine	-0.033222988	0.028964511	0.253370968
pcaec361.lysopcac204	-0.041344393	0.036045499	0.253378481
pcaac385.pcaec342	-0.102796686	0.089626006	0.25340079
pcaac420.citruline	-0.007897662	0.006887013	0.253484962
pcaac422.arginine	-0.016308482	0.014221909	0.253498086
pcaec363.pcaec342	0.198604575	0.173212673	0.253548173
arginine.pcaec322	-0.058955376	0.051424324	0.253607747
pcaac424.c141oh	0.079641734	0.069477706	0.253672572
c51.prolinr	0.031089732	0.027122675	0.253684298

c12.phenylalanine	-0.032562873	0.028412905	0.253768482
pcaac343.pcaec300	0.00533998	0.004660059	0.253832457
leucine.pcaac281	-0.002981391	0.002602295	0.253925951
pcaac240.pcaac421	-0.037097605	0.032383651	0.253971986
pcaec300.pcaec364	-0.034197783	0.029856233	0.254034136
lysopcac281.pcaec424	-0.011928867	0.010414991	0.254058784
arginine.pcaac384	-0.039702017	0.034668282	0.254124136
creatinine.smohc221	0.050509206	0.044109247	0.254166959
prolinr.pcaac384	-0.051243171	0.044752957	0.254195948
c12.pcaec383	0.059735729	0.052171018	0.254206188
lysopcac140.smc161	0.096668932	0.084440035	0.254278391
pcaac240.tyrosine	-0.062097928	0.054245643	0.254306979
smohc222.pcaac281	0.001888437	0.001649691	0.25432041
pcaec364.kynurenine	-0.014361156	0.012545644	0.254324204
smohc222.pcaac421	-0.015755873	0.013766779	0.25441813
alphaaminoadipicacid.pcaac426	0.050237051	0.043904619	0.254522852
glutamine.pcaec401	0.025349554	0.022155131	0.254542355
kynurenine.pcaec342	0.089604579	0.078313556	0.254545349
serotonin.serine	0.037045025	0.032380132	0.254591053
c3.taurine	-0.051960829	0.045421001	0.254625783
pcaac341.serotonin	0.105309763	0.092063459	0.25466718
pcaac383.putrescine	0.022054291	0.019280629	0.254677459
asparagine.pcaac426	-0.023611604	0.020642764	0.254693001
pcaec385.pcaac386	0.02378255	0.020796986	0.254801454
pcaac422.pcaac240	-0.02104954	0.018408387	0.25483536
asymmetricdimethylarginine.pcaac38	0.012085364	0.010569471	0.254857544
pcaec300.pcaac406	-0.033247195	0.029091483	0.255094206
creatinine.phenylalanine	0.039175468	0.034280965	0.255124709
c12.smohc222	-0.005079822	0.004445405	0.255150802
pcaac300.pcaac321	-0.031406232	0.027487284	0.255208731
prolinr.pcaec322	-0.060168394	0.052661491	0.255218236
c4.pcaec403	0.07999722	0.07001758	0.255226371
pcaac384.c8	-0.042505247	0.03721429	0.255373385
lysopcac140.c141	-0.037812585	0.033116138	0.255521653
pcaac365.pcaac321	-0.039886182	0.034935944	0.255572529
pcaac365.transhydroxyproline	0.033342103	0.029214284	0.255738378
pcaac361.pcaac404	0.004119581	0.003609633	0.25574672
lysopcac182.valine	-0.082120466	0.071960683	0.255783711
pcaec381.lysopcac170	-0.017371726	0.015223442	0.25581207
smohc141.lysopcac160	-0.021852991	0.019158428	0.256007203
pcaec445.pcaac321	-0.021506466	0.018861804	0.256187211
lysopcac280.pcaec405	0.088317757	0.077457311	0.256187423
glycine.smc240	0.040859571	0.035835122	0.256188177
pcaec424.pcaec385	-0.063321389	0.055539733	0.256229886
c102.lysopcac281	0.012563255	0.011019366	0.256231061

c10.pcaac281	-0.003799328	0.003332628	0.256258911
pcaec404.leucine	-0.007513109	0.006591819	0.25637398
asymmetricdimethylarginine.pcaec44	0.052558575	0.046113679	0.256374654
c5.lysopcac182	0.084006254	0.073707271	0.256388305
arginine.pcaac341	0.005515992	0.004840078	0.256420992
alphaaminoadipicacid.pcaec340	0.044605382	0.039142829	0.25646049
pcaac422.lysopcac203	-0.022242722	0.019523146	0.256566131
pcaac405.lysopcac170	0.00824025	0.007232759	0.256568071
pcaac425.citruline	0.006480266	0.00568886	0.256643056
pcaec424.pcaac381	0.020017336	0.017573369	0.256660832
pcaec446.smc261	0.039054276	0.034291365	0.256734345
pcaac386.pcaac405	-0.037035497	0.032521883	0.256779382
pcaec386.pcaec424	-0.00935739	0.008217126	0.256787791
methionine.alanine	0.019881044	0.017462119	0.256888693
pcaac380.pcaec385	-0.060093461	0.052784688	0.256913737
c8.glycine	-0.038946344	0.034218171	0.257032966
h1.isoleucine	0.025876492	0.02274163	0.257170441
smc241.pcaac362	-0.044155666	0.038816708	0.257297013
pcaec404.smohc222	-0.012322916	0.010836922	0.257471736
smohc241.pcaec445	0.038676295	0.034013209	0.25748294
lysopcac180.asparticacid	0.014117602	0.012417046	0.257542497
methionine.pcaec401	0.025537153	0.022461344	0.257548928
smohc241.leucine	0.006626261	0.005828933	0.257611423
pcaec322.c2	0.053520251	0.047085497	0.257664288
glycine.pcaec404	0.02951221	0.025965908	0.257700029
pcaac422.pcaec384	-0.062257315	0.054788211	0.257803599
pcaec360.leucine	0.007199375	0.00633588	0.257820407
pcaac402.pcaac426	0.103512226	0.091099196	0.257832136
pcaec322.valine	0.060679392	0.053404563	0.257847678
smohc141.c162	-0.084959071	0.074788799	0.257945485
creatinine.pcaac405	0.013766303	0.012119093	0.257973859
phenylalanine.c141oh	-0.11490634	0.101160659	0.257990056
pcaec423.pcaac405	-0.049973908	0.043999393	0.258029009
smc161.pcaac384	-0.0573389	0.050487512	0.258063092
pcaac406.leucine	0.00635621	0.005596924	0.258081169
creatinine.pcaac420	0.091667311	0.080729655	0.258154736
pcaec343.lysine	-0.064886355	0.057145171	0.25816298
pcaac342.glycine	0.038315547	0.033744849	0.258169856
pcaac386.arginine	-0.031171955	0.027457241	0.258235751
c141.tyrosine	0.090067744	0.079354753	0.258356654
pcaac404.pcaec446	-0.036590134	0.032242257	0.258419701
pcaac363.c0	0.004954441	0.004365824	0.25843072
c14.c2	0.063237849	0.055725689	0.258438166
c5.pcaac404	0.007123432	0.006277309	0.258444611
pcaac300.phenylalanine	-0.036674303	0.032319916	0.258470897

pcaac343.kynurenine	-0.008729541	0.007693108	0.258473174
pcaac421.c141	-0.028111731	0.024777366	0.258535503
c14.c101	-0.01176159	0.010366691	0.25854267
serine.leucine	-0.008777944	0.007737159	0.258558609
pcaac381.pcaec420	0.043994064	0.03877798	0.258561197
glutamine.histidine	0.037123548	0.032728749	0.258658144
c181.kynurenine	-0.008774199	0.007739375	0.258896619
lysopcac170.lysopcac160	0.013482716	0.011893458	0.258931937
c141oh.pcaec362	0.013166036	0.011614275	0.258938798
lysopcac203.pcaac425	-0.077269362	0.06817303	0.259013258
pcaac323.c102	0.024704316	0.021796183	0.259015827
pcaac386.lysopcac181	0.020868049	0.018413719	0.259072645
pcaec405.pcaec424	-0.036016469	0.03178151	0.259087615
lysopcac181.pcaec420	0.053773093	0.047451097	0.259096183
prolinr.spermidine	-0.064785076	0.057169616	0.259106083
lysine.pcaac381	0.021892676	0.01932223	0.259180421
isoleucine.pcaac321	0.033509221	0.029575297	0.259187397
pcaac380.pcaac361	-0.073835788	0.065170206	0.259206381
pcaec301.c3	0.04113721	0.036314698	0.2592778
pcaec363.glutamine	-0.011686223	0.010317296	0.259325986
pcaec445.alanine	-0.009070081	0.008007637	0.25932763
c8.pcaec382	-0.010634625	0.009390403	0.259402783
smc161.smc240	-0.067034779	0.059199515	0.259463824
lysopcac170.pcaac383	0.015624676	0.013798586	0.259469921
pcaac360.citruline	0.006383629	0.005638099	0.259515273
ornithine.pcaac421	-0.009188938	0.008118982	0.259702213
c2.pcaec340	0.047973799	0.042390971	0.259738229
pcaec424.pcaac405	-0.046338434	0.040954406	0.259836546
pcaac404.histidine	0.042631217	0.037678893	0.259848697
serotonin.c102	0.048036489	0.042457425	0.259861781
lysopcac181.leucine	-0.007604562	0.006722621	0.259951391
smc202.pcaec382	0.007220252	0.006383769	0.260017324
alanine.pcaec364	-0.022222209	0.019649461	0.260059518
c141.arginine	0.036919863	0.032646484	0.260073622
pcaac384.pcaec424	-0.003782136	0.003345527	0.260238645
c12.pcaec445	-0.037083485	0.032802998	0.260244749
alphaaminoadipicacid.c5	-0.016554226	0.014644811	0.260290515
lysopcac181.lysopcac203	-0.010378831	0.009183144	0.260364852
pcaec384.pcaec363	0.029848032	0.026412577	0.260421821
c2.lysopcac160	0.008970735	0.007939458	0.260495847
alphaaminoadipicacid.pcaec343	-0.062342504	0.055179761	0.260531606
pcaec381.lysopcac161	0.050455562	0.044661567	0.260563669
h1.pcaec406	0.016364164	0.014490739	0.260751389
arginine.citruline	0.014169148	0.01254719	0.260757963
smc240.arginine	-0.032224931	0.028544712	0.260901059

histidine.pcaec382	-0.024312595	0.021536877	0.260920446
pcaac342.lysopcac160	-0.032013102	0.028360538	0.260959011
pcaec384.pcaec445	0.032677038	0.028952044	0.261013471
c12.pcaac381	-0.009862771	0.008738635	0.26102234
pcaac402.pcaac365	0.090135553	0.079867419	0.261053888
prolinr.pcaac240	0.013011978	0.011529711	0.261055629
asparticacid.pcaac426	0.071126467	0.063024424	0.261058399
lysopcac204.c8	-0.057591946	0.051032517	0.261066698
asparagine.pcaec364	-0.018977543	0.016817599	0.261109069
pcaec385.pcaec424	-0.008342039	0.007393484	0.261166856
pcaec445.c3	-0.017042339	0.015104849	0.261178114
methioninesulfoxide.pcaac320	-0.122035321	0.1081787	0.261253564
pcaac363.pcaac341	0.009191831	0.008149142	0.261312247
pcaec361.histidine	0.077339017	0.06858368	0.261434933
pcaec340.c8	0.043310223	0.038410991	0.261481888
pcaec321.pcaac361	0.088341043	0.078361525	0.261564175
pcaac281.pcaec424	-0.006044858	0.005362315	0.261592464
lysopcac140.pcaac405	0.012645106	0.011217432	0.261597664
pcaec403.pcaac343	0.014649074	0.012996208	0.261636466
pcaec401.c2	0.056097458	0.049776855	0.26172151
pcaac240.pcaac343	0.02328372	0.02066041	0.261724031
c142.h1	0.004743993	0.004209695	0.261745983
lysopcac181.arginine	0.062643377	0.055598744	0.26183691
glutamicacid.leucine	-0.007585343	0.006733655	0.261930736
histidine.c141	0.037256791	0.03307476	0.261947828
citruline.prolinr	0.015396645	0.013668418	0.261948812
pcaec424.lysopcac170	-0.017755607	0.015765557	0.262038071
creatinine.pcaac385	0.033086854	0.029379171	0.262049316
pcaac366.lysopcac281	0.014821488	0.013161464	0.262080323
smc180.pcaac366	-0.058325472	0.051815029	0.262282714
c18.pcaec342	-0.06770741	0.060151639	0.262297783
pcaec363.c51dc	-0.120683855	0.107234218	0.26237757
pcaac426.pcaac420	-0.080682293	0.07172335	0.262594269
smc260.pcaec445	0.0687591	0.061129719	0.262637893
c182.lysopcac182	0.017608499	0.015655308	0.262656548
pcaac341.pcaec424	-0.00329531	0.002929888	0.262673476
pcaec381.leucine	-0.004938032	0.004391003	0.262733713
ornithine.smc181	-0.024183887	0.021505462	0.262747789
pcaec386.pcaec385	-0.104100499	0.092591697	0.262853425
lysopcac280.lysopcac203	0.023113069	0.020558014	0.262858137
pcaac383.smohc161	0.020800676	0.018501817	0.262872796
glutamicacid.pcaec384	-0.066837221	0.059455109	0.262909831
glycine.tyrosine	0.055606983	0.049465909	0.262916255
smohc161.pcaac405	-0.058138281	0.051718514	0.262924119
smc161.pcaec443	0.047470543	0.042230205	0.262940683

pcaac281.serotonin	0.099551853	0.088570901	0.262987428
pcaac380.pcaec321	0.046870981	0.041701773	0.262997014
pcaac426.pcaec380	0.061326552	0.0545687	0.263045795
smc161.pcaec322	-0.084575244	0.075258232	0.263062961
smc161.histidine	0.053987421	0.048041187	0.263074338
smohc221.pcaac381	-0.031383933	0.027927319	0.263075123
serotonin.pcaec300	-0.008828469	0.00785678	0.263115967
kynurenine.pcaac343	-0.02465443	0.02194197	0.263139429
lysopcac203.smohc222	0.021816696	0.019419611	0.263217024
c0.pcaec402	-0.033126463	0.029490791	0.263282462
c0.pcaac363	0.026153081	0.023283964	0.263307308
c101.valine	-0.053030781	0.047216807	0.263345099
smohc222.smc240	-0.070022586	0.062348492	0.263366131
pcaac385.lysopcac280	0.005091674	0.004534103	0.263413185
smc202.c51dc	-0.116342132	0.103653144	0.263648107
pcaec362.kynurenine	-0.006352458	0.005659808	0.263663872
pcaec423.pcaec405	-0.056661665	0.050497981	0.263800491
lysopcac140.lysopcac203	0.015173942	0.013524363	0.263837387
pcaec340.pcaec364	-0.033953903	0.030263646	0.26385178
smohc221.pcaec444	-0.10771784	0.096012568	0.263861529
smc181.lysopcac180	0.056938016	0.05075187	0.263871683
pcaac426.pcaec342	-0.090115723	0.080329958	0.263901506
glutamicacid.pcaec425	0.021198849	0.018897482	0.26391759
pcaac322.pcaec361	-0.012548846	0.011186614	0.263921078
pcaac380.pcaac421	-0.025296391	0.022559274	0.264109043
lysopcac203.pcaac426	-0.066675759	0.059463682	0.264127962
pcaac422.pcaac421	-0.04168089	0.037173589	0.264142933
pcaac342.leucine	0.005274531	0.004704451	0.264173074
pcaac365.pcaec446	-0.050068391	0.044657801	0.264182553
pcaac323.smohc241	0.066237716	0.059083356	0.264211142
smc240.c0	-0.000517232	0.000461438	0.264286227
c2.smohc141	0.016912041	0.015091022	0.264389422
pcaac422.pcaac403	-0.024248191	0.02163894	0.264426649
pcaec300.lysopcac170	0.015944492	0.014228961	0.264433078
c101.asparagine	-0.005631954	0.005026177	0.264450774
c8.citruline	-0.004536319	0.004048761	0.264494412
c5.pcaec380	0.063311402	0.056517952	0.264588538
kynurenine.tyrosine	0.067286023	0.060075436	0.264662556
lysopcac281.lysopcac161	0.071290947	0.063658166	0.26471475
pcaec382.prolinr	-0.025253506	0.022550203	0.264724655
asymmetricdimethylarginine.pcaac42	0.008360593	0.007465753	0.264733141
pcaec342.pcaac385	-0.020252413	0.018085435	0.264750291
lysopcac170.leucine	-0.006102494	0.005450018	0.264792379
smc181.phenylalanine	-0.045692954	0.040808105	0.264799568
c142.pcaec362	0.015815352	0.014124617	0.264800255

pcaac366.isoleucine	-0.037318046	0.033340724	0.26497365
pcaac322.pcaac321	-0.053527681	0.047834444	0.265089941
c8.pcaec444	-0.09309965	0.083199852	0.265103304
alanine.pcaac405	0.026949271	0.02408401	0.265111273
c0.pcaec404	-0.014655692	0.013099989	0.265201984
lysopcac182.c141oh	0.092451696	0.082658664	0.265321283
pcaec384.pcaac380	-0.065027389	0.058141098	0.265335979
lysopcac204.pcaec424	-0.005326141	0.004762271	0.26535183
pcaec381.pcaec385	-0.068042987	0.060844326	0.265390535
pcaac300.pcaec424	-0.003535486	0.003161679	0.265425464
pcaec322.smohc161	-0.014674152	0.01312522	0.265518557
kynurenine.lysopcac181	0.053463567	0.047822309	0.265539415
c141oh.histidine	0.046971606	0.042017364	0.265562197
lysine.pcaac421	0.030368926	0.027165982	0.265565014
pcaac342.lysopcac161	0.062406321	0.05582477	0.265567633
pcaec360.pcaec343	0.083742319	0.074925622	0.265662966
spermidine.c162	0.04491307	0.040188404	0.265709685
smc202.lysopcac280	0.007670025	0.00686408	0.265772696
c101.leucine	0.005059795	0.004529155	0.265880893
lysopcac180.leucine	-0.00429131	0.003841692	0.265933918
smohc241.pcaec341	0.065177894	0.058352369	0.265961879
smohc222.pcaec364	0.042169081	0.037753854	0.265971725
pcaac426.pcaec301	0.081713167	0.073158419	0.26597733
pcaac364.pcaec363	0.029486234	0.026407316	0.266122731
c182.smohc241	0.067605959	0.060554052	0.266181125
pcaec343.pcaec384	-0.027984126	0.025068148	0.266238429
pcaac426.smc240	0.029007453	0.025992422	0.266377218
smc261.smc180	-0.008912643	0.007986349	0.266382283
smohc241.alanine	-0.019457492	0.017436319	0.26641099
pcaec361.pcaac425	0.09165951	0.082148384	0.266469673
c10.pcaac384	0.089827533	0.080511362	0.2664984
c0.smohc222	-0.009530016	0.008542146	0.266526483
lysopcac161.pcaac365	-0.038263452	0.034297184	0.266527568
c51.lysopcac161	-0.052103878	0.046703389	0.266531991
pcaec362.pcaec341	0.09409944	0.084348297	0.266543972
pcaac341.pcaec364	-0.013952345	0.012506958	0.266560662
c142.c3	0.04350715	0.039012607	0.266713955
pcaec364.pcaac362	-0.025246501	0.022638936	0.266725434
lysopcac280.smc160	0.083220077	0.074665004	0.266982158
methioninesulfoxide.smohc222	0.010987602	0.009859037	0.267028883
c10.citruline	-0.005044609	0.004527492	0.26713703
pcaec443.leucine	-0.007981934	0.007163783	0.267141505
acetylornithoine.pcaec383	-0.066943916	0.060084116	0.267157132
phenylalanine.pcaac322	0.045864635	0.041171004	0.267228358
histidine.smohc161	0.009674059	0.008686442	0.267359591

smohc222.pcaac420	0.054150523	0.048628156	0.267416569
pcaac404.lysopcac203	0.018104274	0.016259313	0.267455961
lysopcac281.lysopcac203	0.018808154	0.016892311	0.267479899
c8.pcaac384	0.077963604	0.070030466	0.267537161
pcaec424.leucine	-0.010891887	0.009784387	0.267575982
pcaac365.c12	0.058186636	0.052287954	0.26773819
pcaac386.c141	-0.034491874	0.031006006	0.267903424
pcaec322.c18	-0.021857445	0.019650678	0.267957289
lysopcac281.pcaec404	-0.071017719	0.063851824	0.267988479
pcaec384.pcaec405	-0.070382814	0.06328318	0.268005026
acetylornithoine.c16	-0.047937874	0.043106466	0.268051081
pcaac323.pcaac405	0.040529441	0.036450603	0.268128373
pcaec403.leucine	-0.005916148	0.005320857	0.268137632
alanine.c182	0.054264362	0.048805131	0.268146668
pcaac385.c102	0.026710825	0.024023843	0.268151501
c101.serotonin	0.065654294	0.059050141	0.268154361
histidine.smc261	0.020860941	0.018763529	0.268179399
c182.c5	-0.008261043	0.007430749	0.268198123
pcaac322.histidine	0.050124451	0.045086871	0.268201161
pcaec425.asparticacid	0.030022161	0.027005393	0.268210045
smohc222.smc161	0.11687559	0.105161162	0.268344991
pcaec300.pcaac421	0.010488409	0.009438988	0.268437425
smc240.spermidine	0.058408507	0.052573231	0.268517254
c4.lysopcac204	0.045994069	0.04140297	0.268562351
c162.smohc221	-0.060812123	0.05474376	0.268578586
pcaac404.pcaac365	-0.027330922	0.024604957	0.268604774
smc161.kynurenine	-0.004296103	0.003867881	0.268637754
pcaec422.pcaac343	0.017202735	0.015491142	0.268733831
pcaec406.pcaac343	0.028632028	0.025789453	0.268848047
c0.lysopcac140	0.003612527	0.003253981	0.268863317
asparagine.pcaac425	-0.065598423	0.059096744	0.268935994
pcaac425.c4	-0.016860389	0.01519021	0.268964559
pcaec382.pcaec404	0.03957636	0.035660617	0.269026926
smohc221.pcaac384	-0.045682525	0.041172884	0.269145651
smohc241.smc261	0.026531446	0.023912397	0.269146755
acetylornithoine.pcaec404	0.023004341	0.020737539	0.269240313
glutamicacid.pcaac402	-0.082193235	0.074095024	0.269246393
pcaec384.pcaec424	-0.03299844	0.029750533	0.269299489
pcaac384.c162	-0.061594253	0.05554189	0.269386342
pcaec343.c18	-0.0276504	0.024933707	0.269391806
c8.pcaac343	0.013785222	0.012431398	0.269414593
pcaac321.pcaec444	0.064168035	0.057878003	0.269511849
pcaac406.smohc161	-0.014371672	0.01296336	0.269528863
pcaac404.pcaac323	-0.120784394	0.108963322	0.269593867
pcaac420.leucine	0.007110437	0.006414639	0.269600936

c3.pcaec422	-0.027478837	0.024790185	0.269606942
lysopcac160.pcaec424	-0.004035274	0.003641069	0.269688682
pcaac320.leucine	0.004354018	0.003929471	0.269785361
prolinr.c2	-0.054281349	0.048989855	0.269798079
c102.pcaec380	-0.049301352	0.044495346	0.269798349
pcaec422.pcaec424	-0.005710557	0.005153917	0.269801849
serotonin.pcaec424	0.003743678	0.003379497	0.269905748
glutamicacid.isoleucine	-0.031243257	0.028207556	0.269966774
c181.pcaec301	-0.03548263	0.03204331	0.270090206
putrescine.phenylalanine	0.025142167	0.022709277	0.270176979
pcaac402.pcaac281	-0.004480382	0.004047291	0.270230505
creatinine.smc241	0.069646229	0.062919031	0.270269024
asparagine.pcaec360	0.006329959	0.005719177	0.270322026
acetylornithoine.pcaac421	0.012134956	0.010966217	0.270416449
c16.pcaec341	0.059780826	0.054023395	0.270417883
pcaec406.leucine	0.00604011	0.005459249	0.270492661
pcaac386.pcaec365	-0.020839978	0.018836584	0.270511161
lysopcac281.pcaec444	0.125353746	0.113316111	0.270565471
pcaec384.pcaec403	-0.037481606	0.033886425	0.270623916
ornithine.transhydroxyproline	-0.031137238	0.028158459	0.270757082
pcaac426.c4	-0.013579875	0.012281117	0.2707717
arginine.pcaac421	0.013194177	0.011932348	0.270773377
c2.lysopcac140	-0.002945367	0.002664046	0.27083773
pcaac343.pcaac364	0.072044706	0.065164053	0.270841895
pcaac365.pcaac361	-0.068565344	0.062022052	0.270880847
pcaac424.pcaac426	0.094780093	0.085751469	0.270971982
pcaac385.pcaac381	-0.051234901	0.046356205	0.270991244
glutamine.leucine	0.004360597	0.003945847	0.271048763
h1.kynurenine	-0.011611398	0.010510472	0.271206266
lysopcac181.smohc241	0.060980236	0.055202771	0.271243707
pcaec361.pcaac344	0.098825966	0.08947523	0.271309599
lysopcac203.pcaac361	-0.069146935	0.06261345	0.271378759
pcaac240.lysopcac140	0.00319409	0.002892713	0.271448484
c8.asparagine	-0.005465509	0.004949898	0.271456652
pcaec382.spermidine	-0.055618509	0.050372635	0.271467345
c181.pcaac403	-0.003933348	0.003562369	0.271468683
lysopcac281.pcaec383	-0.050712012	0.045930886	0.271487845
pcaec422.c51dc	-0.121002747	0.109599456	0.271508931
pcaec341.pcaec383	-0.057185991	0.051800613	0.271544173
pcaac342.c162	-0.066890116	0.060604583	0.271652157
glutamine.pcaec383	-0.044212474	0.040058332	0.271657139
lysopcac160.pcaac405	-0.076531261	0.069346716	0.271699974
asparagine.lysopcac160	0.008744577	0.007926215	0.271853854
pcaac344.pcaac321	-0.048847061	0.044277815	0.271876582
lysopcac281.pcaac381	-0.049293303	0.044691064	0.27197004

pcaec443.c141oh	0.073374493	0.066524028	0.271970746
methioninesulfoxide.lysopcac140	0.002925989	0.002653076	0.272018621
histidine.kynurenine	-0.011157641	0.010120018	0.272163606
histidine.lysopcac280	0.020391433	0.018497844	0.272234446
lysopcac182.phenylalanine	-0.0590673	0.053585639	0.272265161
pcaec381.pcaec402	-0.096700198	0.087727234	0.272271512
lysopcac203.pcaac421	-0.016062	0.014571764	0.272277472
pcaec341.pcaec424	-0.005762059	0.00522773	0.272302703
pcaec343.pcaac362	-0.013768697	0.012493223	0.27235346
pcaec362.lysopcac160	0.021617515	0.019615369	0.272363435
c8.lysopcac204	0.02088905	0.018954622	0.272369743
pcaac343.pcaac404	0.004881126	0.004429238	0.272383553
pcaec364.c14	-0.046667091	0.042355015	0.272477106
c182.pcaac421	-0.008818672	0.008004945	0.272544206
pcaac385.pcaec343	-0.09488325	0.086136741	0.27259228
pcaec446.pcaac366	-0.064772127	0.058806101	0.272631138
pcaac384.pcaac380	-0.047319088	0.042964093	0.272669666
serotonin.pcaac380	0.057851679	0.052545044	0.272830749
pcaec401.c18	-0.036305099	0.032979125	0.272892001
isoleucine.pcaac281	-0.003434389	0.00311983	0.272902867
c10.asparagine	-0.004743826	0.004309571	0.272929119
pcaac384.pcaec384	0.072883562	0.066217035	0.272967468
pcaec445.lysopcac182	0.032655347	0.029670668	0.273003617
smc161.spermidine	0.05761393	0.052351724	0.273037117
pcaac364.pcaec343	-0.065601613	0.059610302	0.273040761
pcaec406.c5	-0.007484702	0.006801279	0.273050935
pcaac405.valine	-0.040972377	0.037241141	0.273178219
pcaac424.leucine	-0.017654841	0.016047485	0.273190693
c101.smc181	-0.027188476	0.024713523	0.273197997
pcaec341.c2	0.048089793	0.043713581	0.273213031
c5.c51dc	0.212672126	0.193329007	0.273238294
smc181.c14	-0.023133938	0.021037412	0.27341016
pcaac425.pcaac281	0.004108588	0.003736956	0.27350105
pcaec423.leucine	0.013788176	0.012545357	0.273666944
lysopcac181.pcaac421	-0.007562658	0.006882044	0.273740394
pcaec383.c51dc	-0.13630122	0.124037546	0.27375185
pcaac420.c51dc	-0.119261547	0.108536613	0.273776492
lysopcac182.c4	-0.035638228	0.032433867	0.27378399
asparagine.glycine	0.028372131	0.025821553	0.273792525
c12.pcaec360	0.006013813	0.005473191	0.2737928
pcaac424.smc261	0.069384112	0.063154964	0.273855285
pcaec382.pcaac403	0.013725924	0.012494851	0.273901517
putrescine.c141	0.026571316	0.024188696	0.273912407
pcaac383.smohc221	0.045267557	0.041212853	0.273963266
pcaac422.pcaec406	-0.078164443	0.071173326	0.274031898

pcaac365.c16	0.032803922	0.02987196	0.274064822
pcaec363.pcaac420	0.04476728	0.040771866	0.274132942
pcaec322.pcaec385	-0.045897278	0.041807857	0.274211157
pcaac406.tyrosine	0.043860582	0.039953964	0.274227118
pcaac362.pcaec424	-0.002992206	0.002725735	0.274234502
glycine.glutamicacid	0.044259359	0.040318031	0.274236702
pcaac406.ornithine	-0.014527286	0.013234037	0.274251729
lysopcac181.pcaec384	-0.035294321	0.0321554	0.274297071
pcaac383.c16	0.041388311	0.037711688	0.274351199
pcaec343.leucine	0.010718283	0.009768403	0.2744613
pcaec384.pcaec321	0.059213733	0.053966387	0.274464109
leucine.pcaac321	0.028515926	0.02599165	0.274514149
pcaec424.smohc221	0.047851532	0.043617256	0.274531869
c101.taurine	-0.042648767	0.038878855	0.274580827
pcaac344.asparagine	0.008400829	0.007658541	0.274599408
c0.lysine	0.081011681	0.073854693	0.274606647
lysopcac182.lysopcac280	0.009021024	0.008224344	0.274623169
smc261.c51dc	-0.11239698	0.102480663	0.274669175
pcaac406.glycine	0.044967341	0.041000639	0.274675922
pcaec384.spermidine	0.057282654	0.052237142	0.274745095
creatinine.c51	-0.117184946	0.106880147	0.274820955
pcaec386.leucine	0.007131852	0.006505025	0.274844522
smc180.pcaac421	-0.027441743	0.025031287	0.27487186
pcaac363.transhydroxyproline	0.016350899	0.01491546	0.27489775
alphaaminoadipicacid.smc180	-0.021482549	0.019599907	0.274978297
pcaac323.c51	-0.08473491	0.077318785	0.275038209
alanine.leucine	-0.007984973	0.007286265	0.275047968
lysopcac160.c51	-0.070896039	0.064692737	0.275050216
smc260.pcaac405	-0.030155095	0.027518664	0.275086637
pcaec383.pcaac425	0.107202781	0.097832853	0.275099859
lysopcac160.lysine	0.075511662	0.068911676	0.275099992
pcaac365.pcaac385	-0.033819473	0.030865593	0.275131899
pcaac323.pcaac385	0.026167341	0.023882176	0.27513906
pcaec443.pcaec422	0.09653323	0.088117128	0.275215226
citruline.pcaac386	0.021477728	0.019606194	0.275238843
pcaec420.pcaac365	0.035724057	0.032613707	0.275276738
pcaac361.pcaec401	0.03335835	0.030460772	0.275383444
isoleucine.pcaec364	-0.019100716	0.017445675	0.275495459
smc161.pcaac343	0.018254961	0.016673905	0.275515582
pcaac402.pcaac405	-0.058840919	0.05375901	0.27564263
pcaec343.kynurenine	-0.005380633	0.004917652	0.275810667
smc161.c5	-0.005921312	0.005411903	0.27581904
pcaec321.lysopcac160	-0.0112839	0.010314273	0.275871101
pcaac342.putrescine	0.023385615	0.021376166	0.275872917
pcaac361.pcaec424	-0.002625876	0.002400406	0.275905428

glutamine.c12	0.085586156	0.078269692	0.276103105
h1.arginine	0.045537605	0.041647177	0.276130934
pcaac402.asparticacid	0.071427029	0.065326839	0.276145989
smc261.pcaac405	-0.018668681	0.017075269	0.276173365
c51.lysopcac280	-0.011632378	0.010641251	0.276250729
pcaec445.citruline	-0.006911786	0.006323084	0.276266671
h1.pcaac405	0.071886524	0.065765911	0.276282844
asymmetricdimethylarginine.lysopca	-0.002028188	0.001855991	0.276408805
pcaac405.c18	-0.041239448	0.037741613	0.276452698
pcaec341.pcaac323	-0.080379637	0.073568817	0.276496869
pcaac380.alanine	-0.014311594	0.013100264	0.276545651
pcaac380.alphaaminoadipicacid	0.012652557	0.011584203	0.276651175
lysopcac203.pcaec340	0.039339767	0.036028477	0.276790227
smc202.pcaac343	0.022617517	0.020715359	0.276827028
c10.pcaec365	-0.02236531	0.020492152	0.277008872
pcaac364.phenylalanine	-0.046	0.042148342	0.277019888
alphaaminoadipicacid.pcaec301	0.047044405	0.043106979	0.277038547
smc160.pcaac402	0.080408447	0.073687167	0.277094256
pcaec300.pcaec322	-0.059173601	0.05422819	0.277101889
pcaac403.pcaac405	-0.048429379	0.044388954	0.277177774
pcaec403.arginine	-0.103772266	0.095115999	0.27718467
pcaac424.pcaec321	0.028245997	0.02589647	0.277307356
pcaec423.c141oh	0.085350755	0.078265576	0.277395214
pcaec402.citruline	0.019894905	0.018243636	0.277401942
lysopcac181.pcaec424	-0.006231033	0.005714043	0.277417309
lysopcac280.pcaec380	0.037615738	0.034495008	0.277420818
glutamine.pcaec385	0.051641426	0.04736828	0.277533975
pcaac362.putrescine	0.021116727	0.019371043	0.277574755
pcaac425.pcaec424	-0.013396619	0.012291419	0.277663364
c142.lysopcac181	0.045695644	0.041929951	0.277710417
c162.pcaec383	0.06560804	0.060203481	0.277726924
pcaac404.smc240	0.021415961	0.019657453	0.277864789
pcaec381.pcaec364	0.005243793	0.00481322	0.277865333
pcaec443.lysopcac140	0.001748647	0.001605253	0.277921833
smohc241.spermidine	0.058601509	0.053796372	0.277924849
c16.pcaac421	-0.046386663	0.042583601	0.277930436
c51dc.c182	0.058688375	0.053880274	0.277961785
kynurenine.leucine	0.028391128	0.02606527	0.277963843
lysopcac281.glutamine	-0.013185814	0.012105792	0.277971205
pcaac381.smc161	0.086898187	0.07978623	0.278005324
pcaac240.pcaac402	0.063641501	0.05843706	0.278039171
pcaec362.pcaec445	0.022333594	0.020511469	0.27813855
pcaec365.leucine	0.007875477	0.00723322	0.278156947
c5.pcaac386	0.047984644	0.044072072	0.278163945
c0.citruline	-0.00528562	0.004855073	0.278206464

pcaec301.pcaac402	0.042891648	0.03940772	0.278326345
pcaac426.smc160	-0.076377192	0.070176136	0.278345285
pcaec365.pcaec445	0.050109797	0.046046048	0.278393721
lysopcac280.pcaec383	-0.085356454	0.078442727	0.278445072
pcaec362.pcaac404	0.003882286	0.003568607	0.278549569
pcaac381.pcaec382	-0.008049818	0.007399452	0.278552127
pcaec382.pcaac365	-0.052690833	0.048440145	0.278614841
pcaac383.c14	0.03087814	0.028387249	0.278616827
methioninesulfoxide.lysopcac170	-0.012694589	0.011670729	0.278624733
c12.citruline	-0.008721326	0.008018437	0.278655421
c141.pcaac384	0.058140579	0.053456432	0.278670209
pcaec383.acetylornithoine	-0.065098077	0.059854648	0.278680244
c2.pcaac426	0.049874535	0.045867078	0.278782189
pcaec321.pcaec365	0.023946998	0.022022922	0.278783983
c18.lysopcac160	0.009687958	0.008909699	0.278791633
smc240.pcaac240	-0.011222983	0.010324094	0.278915996
c8.pcaac421	0.037989088	0.034946652	0.278919394
lysopcac160.asparagine	0.014163491	0.013030053	0.278951535
h1.pcaac361	0.101359067	0.093271948	0.279075612
pcaac422.pcaec301	0.080204774	0.073829795	0.279233241
pcaac386.smc160	-0.063978605	0.058897553	0.279267451
c8.pcaec360	0.008640526	0.007954443	0.279275297
kynurenine.lysine	-0.095691011	0.088098124	0.279303869
pcaac420.lysopcac280	0.006390183	0.005883662	0.279346807
pcaec322.pcaac281	0.001621267	0.001492905	0.279394365
c51.glycine	-0.024001664	0.022102303	0.279414821
c142.prolinr	0.043055849	0.039649063	0.279419891
pcaec362.pcaec422	0.020061499	0.018474511	0.279429603
smc160.lysopcac181	0.048875113	0.045021828	0.279568263
c51dc.glutamicacid	-0.063330066	0.058339218	0.279585137
pcaac364.glycine	0.019368487	0.01784243	0.279593568
pcaac405.smc181	-0.031693839	0.029201806	0.27967796
c16.methionine	0.012017726	0.011073059	0.279689491
pcaec381.pcaec342	0.076193572	0.070215699	0.279767297
smc261.pcaac381	-0.038804446	0.035760705	0.279776883
pcaac404.pcaec340	0.062970104	0.058040289	0.279854698
pcaec301.c162	0.077402091	0.071350206	0.279906929
pcaac380.pcaec424	-0.005514246	0.005083316	0.27992725
pcaac322.alphaaminoadipicacid	0.037837822	0.034882425	0.279948832
putrescine.c102	-0.029641535	0.027327261	0.279965234
c142.citruline	-0.008057527	0.007429852	0.280056746
c182.pcaac425	0.048908891	0.045102674	0.280096515
c51.tryptophan	0.021069165	0.019430921	0.280131352
pcaac405.asparticacid	0.028683649	0.026456143	0.280182142
pcaec341.glutamine	-0.007875015	0.007263738	0.280200494

pcaec401.kynurenine	-0.012014346	0.011083314	0.28026744
c162.pcaec424	0.011627667	0.010727562	0.280310412
pcaec425.lysopcac204	-0.031852286	0.029387339	0.280322756
smc160.smc180	-0.015641317	0.014432816	0.280386839
kynurenine.lysopcac140	-0.000641721	0.00059215	0.280394962
pcaac403.pcaec383	-0.170383198	0.15723133	0.280424955
putrescine.lysopcac203	-0.007589859	0.007004022	0.280426622
pcaac281.c5	-0.007696062	0.007102043	0.28042765
pcaec322.pcaec364	0.038390582	0.03543127	0.280479772
creatinine.pcaec360	0.002366139	0.002183992	0.280533382
lysopcac170.pcaec340	0.046488287	0.042913568	0.280577978
pcaac403.pcaac365	0.080834848	0.074619878	0.280583306
pcaac385.pcaec363	0.024786878	0.02288281	0.280618178
pcaac403.pcaec404	0.033284235	0.030732276	0.280693849
c4.smc202	0.061653207	0.056926725	0.280698679
c12.c5	-0.008698727	0.008034553	0.280859168
pcaec421.c4	-0.025524205	0.023575884	0.280869901
leucine.pcaac343	0.022018521	0.020339601	0.280912372
glycine.pcaac381	-0.016009861	0.014791401	0.280986797
lysopcac204.pcaac421	-0.007884208	0.007284281	0.280994382
c4.pcaac386	0.025471105	0.023540313	0.281144206
pcaac343.smc241	0.020438224	0.018890419	0.281181692
pcaec401.lysopcac140	0.001126781	0.001041587	0.281245389
pcaec343.pcaac405	-0.027746696	0.025656542	0.281389541
c141.h1	0.008512908	0.007871818	0.281400839
pcaac344.smc181	0.016233899	0.015011556	0.281407195
pcaec343.pcaec445	0.045594575	0.042166819	0.281467664
pcaec402.c5	-0.006421374	0.005939405	0.281530892
isoleucine.pcaac421	0.021090017	0.01950771	0.281546747
c162.pcaac404	0.00169498	0.001568061	0.281622915
lysopcac160.lysopcac280	0.012066472	0.011164002	0.281668385
c14.pcaac406	0.046534743	0.043068584	0.281826889
pcaec405.pcaac405	-0.043131959	0.039919733	0.281832593
pcaac421.pcaac406	0.035288398	0.032663886	0.281884962
smc241.pcaac420	0.066911744	0.061937664	0.281903238
prolinr.c3	0.051816848	0.047969504	0.281949317
pcaec383.spermidine	-0.054639823	0.05058805	0.281998329
isoleucine.pcaec382	-0.035774973	0.033129108	0.282099584
pcaec363.c14	-0.024870447	0.023031588	0.282110472
pcaec321.lysopcac170	0.011831302	0.010956559	0.282111957
pcaac420.lysopcac161	-0.078286339	0.072503545	0.282146855
pcaec343.pcaac344	0.071627254	0.066346244	0.282218338
pcaec444.c141	-0.015232992	0.014110194	0.282229066
lysopcac170.pcaac421	-0.008325769	0.007715513	0.282441661
pcaac403.pcaac343	0.014933846	0.013839414	0.282447886

pcaac322.pcaac406	-0.049253215	0.045645064	0.282462428
pcaac420.smc202	-0.052139405	0.04832333	0.282497253
lysopcac160.pcaac364	0.054905361	0.050889188	0.282519303
histidine.c14	0.042337532	0.039254234	0.282685079
pcaac422.smc202	-0.055990381	0.051926178	0.282808645
c181.pcaec362	0.01833757	0.017006606	0.282811866
pcaac383.smc181	0.028050229	0.026015323	0.282830582
pcaac404.tyrosine	-0.065581077	0.060827248	0.282860178
pcaec321.pcaac322	0.05895815	0.054684781	0.282863496
pcaac320.pcaec424	-0.003755473	0.003483531	0.282899226
lysopcac140.pcaac402	-0.036467203	0.033828836	0.282931887
c12.lysopcac160	0.015514145	0.014394112	0.283011853
asymmetricdimethylarginine.pcaec4(	0.050289994	0.046676366	0.283186711
smc241.kynurenine	-0.004014638	0.003726218	0.283193779
pcaac420.c14	-0.040553433	0.037641775	0.283216534
c3.pcaec380	0.034676109	0.032197438	0.28338044
pcaac386.leucine	0.004552586	0.004227269	0.283392249
pcaac365.pcaec300	0.033831484	0.031421035	0.283500197
lysopcac180.pcaac421	-0.005535385	0.005141585	0.28355509
pcaec446.leucine	0.004265943	0.003962483	0.283558692
pcaac360.c141	-0.046021955	0.042758412	0.283673596
lysopcac140.pcaac321	-0.023269236	0.021620784	0.283709821
pcaac344.pcaac365	-0.042434159	0.0394323	0.283761921
c51dc.arginine	0.040574481	0.037705798	0.283782519
pcaec321.pcaac426	0.058749436	0.054614671	0.283948638
arginine.pcaec301	0.042556667	0.039569353	0.284043284
c16.valine	-0.06225383	0.057886443	0.284064754
pcaac385.lysopcac170	0.008254154	0.007676009	0.284122309
c4.c18	-0.054743175	0.050911609	0.284148727
pcaac323.pcaec382	0.007889366	0.007338083	0.284207994
c14.pcaac366	-0.040580444	0.03774858	0.284255825
pcaac341.c51	-0.063303613	0.058888149	0.284273025
pcaac341.pcaac421	0.013444843	0.012508791	0.284339498
pcaec423.smc260	-0.031821205	0.029608386	0.284382025
pcaac403.lysopcac161	0.064210073	0.059747733	0.284404281
pcaac361.pcaac425	-0.079188794	0.07369984	0.284497649
c12.pcaac405	0.058785874	0.054711955	0.284504759
citruline.c4	-0.016533392	0.015387616	0.284504894
pcaec403.pcaec364	0.005553262	0.005169347	0.284591156
alanine.smohc221	0.044550252	0.041470527	0.284593251
pcaac323.c8	0.038992764	0.03629736	0.28459504
alanine.pcaec445	-0.033753694	0.031421508	0.28461128
phenylalanine.kynurenine	-0.004487128	0.004178157	0.284733382
pcaec403.lysopcac180	-0.057343691	0.053396812	0.284748193
glutamicacid.pcaec322	0.062600974	0.058300179	0.284813471

pcaec401.pcaac421	-0.012212685	0.011374709	0.284858043
lysopcac161.pcaac404	0.008306071	0.007737269	0.284927542
pcaac343.pcaec342	0.106013352	0.098770752	0.28501117
c102.asparagine	-0.016291327	0.015178968	0.285031034
h1.lysopcac140	-0.00077509	0.000722177	0.285037158
c2.smc241	0.058336775	0.054359878	0.285086571
smc240.smc181	0.011956512	0.01114229	0.285124005
pcaac406.kynurenine	-0.005454273	0.005083148	0.285152607
glutamine.pcaac363	0.015444422	0.014393576	0.285153947
c18.pcaec406	-0.012263236	0.011429251	0.285171215
pcaec362.arginine	-0.027505758	0.025635771	0.28518235
smc240.citruline	-0.00913638	0.008515973	0.28522359
pcaac426.c16	0.021992802	0.020500818	0.285257306
pcaac322.pcaac361	-0.05600354	0.052204915	0.285263132
pcaac362.lysopcac161	0.053645948	0.050010401	0.285293513
smohc241.arginine	-0.022405123	0.020888039	0.285323257
lysine.pcaac384	-0.031999566	0.029833879	0.28534015
pcaac321.pcaec382	0.013567751	0.012649801	0.285351418
c16.pcaac381	-0.014726392	0.013730146	0.285354683
smohc161.pcaec404	-0.019185337	0.017891909	0.285474469
glutamine.pcaac381	-0.019439221	0.018129217	0.285488794
c12.pcaac404	0.002733645	0.002549468	0.285496859
pcaec321.arginine	-0.044073546	0.041105642	0.285514462
histidine.smohc221	-0.049835652	0.046481327	0.285530955
c16.pcaec424	-0.012640604	0.011790523	0.285560662
smc181.pcaac344	-0.055801011	0.052049972	0.285575215
pcaec382.leucine	-0.008687502	0.008105613	0.285699509
pcaac360.pcaec385	-0.058912415	0.054971502	0.285743513
pcaec385.c5	-0.008113093	0.007570665	0.285762075
lysopcac281.lysopcac204	-0.015149383	0.014136587	0.28576433
leucine.lysopcac203	0.011287851	0.010536487	0.2859134
smc261.pcaac421	-0.01177577	0.01099358	0.285985509
h1.pcaac403	-0.00710915	0.006637872	0.286053335
glutamicacid.pcaac405	-0.046714895	0.043626146	0.286142018
pcaac402.smc260	-0.049297101	0.046047428	0.286244243
smohc241.pcaac385	-0.020841809	0.019475184	0.286423405
methioninesulfoxide.c14	0.082099027	0.076732776	0.286530323
creatinine.tyrosine	0.057608536	0.053844269	0.286541088
pcaac344.pcaec385	0.037458827	0.035018815	0.286645652
pcaac322.asparagine	0.006513813	0.006090791	0.28674639
smc260.lysopcac203	-0.024270922	0.02269887	0.286834269
c51.pcaec445	-0.056404474	0.052767195	0.286980699
pcaac402.pcaec364	0.004528518	0.00423672	0.287006365
c16.pcaac425	0.071022451	0.066454349	0.287066125
lysopcac204.pcaac426	-0.055409608	0.051849082	0.287097339

pcaec383.c2	-0.079051906	0.073973125	0.287103561
lysopcac280.pcaec424	-0.002391253	0.002238657	0.287325023
smohc141.arginine	-0.030412252	0.028474392	0.287373534
pcaec380.pcaec402	0.069386598	0.064972776	0.287428727
pcaec322.pcaac381	0.02984351	0.027946888	0.287459376
lysopcac181.pcaac385	0.025596224	0.023970147	0.287471802
c51dc.lysopcac160	0.016587053	0.015535981	0.28755432
pcaec421.pcaec364	0.005302881	0.004967176	0.287585505
smc160.lysopcac281	0.020516598	0.019217982	0.287590786
pcaec445.smc261	0.025769545	0.024146725	0.287755531
lysopcac280.pcaac426	0.062086446	0.058183305	0.287810916
glutamine.pcaec300	0.018803251	0.017621565	0.2878219
smc260.pcaac421	0.02241705	0.02100943	0.287848741
c51dc.pcaec341	-0.081495484	0.076416835	0.28809161
pcaec385.pcaec443	0.039293471	0.036844986	0.288094377
pcaec421.phenylalanine	-0.064818086	0.060781087	0.288110143
c0.c142	-0.042104818	0.039509395	0.288437749
creatinine.prolinr	0.033701547	0.031626939	0.288480585
smc202.pcaec341	0.058430425	0.054833799	0.288482752
asparagine.lysopcac204	-0.006931558	0.006505595	0.288534557
pcaec425.acetylornithoine	-0.04677778	0.043907216	0.288578955
pcaac384.valine	-0.0527282	0.049497634	0.288628924
lysopcac204.lysopcac140	0.00318636	0.002991323	0.288658773
c18.pcaac405	0.051599157	0.048443557	0.28868633
spermidine.methionine	0.008426133	0.007912021	0.28875901
arginine.smc161	0.049679112	0.046656798	0.288849641
smc202.pcaec423	0.05448496	0.051176982	0.288912589
pcaec401.pcaac420	-0.07541414	0.070836991	0.288922856
pcaec365.c141	-0.031725315	0.029799812	0.288923143
smc202.kynurenine	-0.003683994	0.003460548	0.288943493
smc180.pcaec422	-0.037961854	0.035664646	0.289014838
citruline.asparagine	-0.00540144	0.005075603	0.289111695
pcaac424.pcaac343	0.012653044	0.011889864	0.289115861
lysopcac280.c14	0.034105613	0.032050062	0.289139249
pcaac405.pcaac384	0.045387875	0.042653954	0.289157425
glutamicacid.pcaec424	-0.033024567	0.031039059	0.289214881
smc202.pcaec360	-0.003983474	0.003744214	0.289245026
pcaac360.leucine	0.013011973	0.012230435	0.289245139
serine.c16	0.02905982	0.027315684	0.289267685
pcaec365.pcaec424	-0.011678389	0.010977924	0.28928772
prolinr.smc181	-0.008465767	0.007958574	0.289322778
pcaac422.asparticacid	0.031089446	0.029233546	0.289432857
c4.pcaec340	0.039872642	0.037504121	0.28958268
c3.serotonin	0.093616107	0.088061853	0.289619464
pcaac363.lysopcac140	-0.003129732	0.002944323	0.289664987

pcaec360.pcaec424	-0.002659068	0.002501581	0.2896723
asparagine.pcaec361	0.005546192	0.005217982	0.289697369
smc260.lysopcac161	-0.05003246	0.047073249	0.28971356
pcaac425.pcaac366	-0.028288267	0.026617999	0.289765254
pcaac240.c102	-0.050368413	0.047394743	0.2897684
acetylornithoine.lysopcac140	-0.000360933	0.000339632	0.289778488
smc260.lysopcac160	0.025856168	0.024333929	0.28985265
pcaec424.pcaac323	-0.066149046	0.062264141	0.28992603
pcaec343.pcaac366	-0.021617682	0.020351064	0.289996341
pcaac402.pcaec402	-0.155290041	0.146196053	0.290011888
c10.pcaec360	0.006730276	0.006336339	0.290026801
c4.pcaac344	0.031470141	0.02962864	0.2900351
smc261.kynurenine	-0.003410708	0.003212108	0.290181682
c182.pcaec382	-0.007159192	0.006742353	0.29018386
pcaec401.c51dc	-0.124076735	0.116855671	0.290197045
pcaac403.kynurenine	-0.011745737	0.011063105	0.290238325
pcaec360.smohc221	-0.040593907	0.038244083	0.290356289
leucine.pcaac403	0.011228913	0.010580961	0.290449213
ornithine.leucine	-0.015960741	0.015041983	0.290520716
h1.pcaec361	0.023015225	0.021691395	0.29054308
smohc161.pcaec445	0.02354547	0.022200318	0.290741753
lysopcac203.lysopcac182	-0.026990191	0.025448606	0.290748605
c51dc.pcaec424	-0.009277532	0.008747786	0.29075706
c18.pcaac386	0.0156997	0.014804673	0.290803297
pcaac403.c141oh	0.082213499	0.077530573	0.290828069
c51dc.pcaac404	0.010725189	0.010115003	0.290862567
pcaec300.kynurenine	-0.011295892	0.010658467	0.291098397
c141.c12	0.070074949	0.066122597	0.291112688
asparticacid.pcaac281	0.003226265	0.003044412	0.291130724
pcaec444.pcaec362	0.014468458	0.013652995	0.291133234
pcaac403.isoleucine	-0.017455787	0.016472504	0.29114927
pcaec443.pcaec301	0.104805731	0.098906822	0.291172544
pcaac421.pcaec340	-0.047220888	0.044565228	0.291195528
lysopcac160.serotonin	-0.09706056	0.091603651	0.291204386
phenylalanine.pcaec301	0.031363175	0.029602359	0.291244546
lysopcac204.phenylalanine	-0.048641033	0.045913692	0.291281209
prolinr.lysopcac181	0.044022738	0.041554701	0.29128529
pcaec446.pcaec405	-0.032812811	0.030974748	0.291308777
smohc222.c51dc	-0.10748442	0.101477977	0.291377316
arginine.pcaec402	0.032610817	0.030790373	0.291407177
pcaac421.serine	0.052523637	0.049598096	0.29147018
pcaac341.phenylalanine	-0.038032089	0.035914366	0.291478828
c141.histidine	0.040198262	0.037967326	0.291572548
citruline.lysopcac160	-0.008894574	0.00840103	0.291577725
smc160.citruline	-0.011979239	0.011315746	0.291629221

citruline.pcaec301	0.061802946	0.05838045	0.291634
pcaac364.pcaec422	0.022007968	0.020789417	0.29163854
c12.smc260	0.034569385	0.03265626	0.291652301
pcaac343.lysopcac160	-0.021579305	0.020386152	0.291677753
pcaec384.asparticacid	0.039781252	0.037589041	0.291771809
pcaec443.c141	-0.035375687	0.033429497	0.291818461
pcaec444.lysopcac160	-0.009878875	0.009335519	0.291825133
pcaac424.c5	-0.015260779	0.014424137	0.291916039
pcaac240.pcaec301	0.087606872	0.082815339	0.291981861
pcaec401.c14	0.056705649	0.053607839	0.29201436
lysopcac281.c102	-0.038188424	0.036102208	0.292014416
pcaac384.c141	-0.024422697	0.02308877	0.29202012
pcaec421.lysopcac140	0.001673548	0.001582349	0.292083086
c2.glutamicacid	-0.058628941	0.05543786	0.292116648
smc202.pcaac403	-0.007455253	0.007051093	0.292226965
pcaac420.pcaac421	0.07769111	0.073482799	0.292249404
methioninesulfoxide.pcaec380	0.096747575	0.091508525	0.292257267
creatinine.pcaac386	0.018402755	0.017408498	0.292320331
pcaec365.pcaec340	0.060267431	0.057027264	0.292454695
pcaac362.pcaac381	-0.031270377	0.029589881	0.292466026
smohc161.histidine	0.049225952	0.046590009	0.292564068
pcaac406.pcaec424	-0.002539851	0.002403975	0.292589598
lysopcac160.pcaec322	-0.047217813	0.0446932	0.292604915
pcaac323.c51dc	-0.105099213	0.099485684	0.292633191
pcaec360.pcaac364	0.047709976	0.045170452	0.292726291
pcaec422.pcaac426	0.074726168	0.07075559	0.292773671
lysopcac161.c0	-0.000717722	0.000679692	0.292848941
pcaac323.asparagine	0.010343032	0.009797043	0.292949782
pcaac425.lysopcac181	0.026845356	0.025428892	0.292962088
pcaac323.glutamine	-0.011579311	0.010968424	0.292965689
pcaec386.lysopcac203	-0.014227558	0.013480392	0.293088156
c8.phenylalanine	-0.044954234	0.042614897	0.293330237
smc260.c181	0.014392338	0.013643447	0.293332335
creatinine.c51dc	0.109081575	0.103442476	0.293503667
c142.pcaec404	0.022068343	0.020929009	0.293538449
pcaec443.pcaec343	0.065463863	0.06209686	0.293637074
pcaac426.lysine	-0.100081498	0.094948707	0.293711523
pcaec443.pcaac300	-0.047665759	0.045221568	0.293715776
pcaac342.kynurenine	-0.01213375	0.011511788	0.293725339
c16.pcaac365	0.039355152	0.037339795	0.29375036
pcaac424.pcaec403	0.129312191	0.122693759	0.293764377
methioninesulfoxide.c18	0.061009765	0.057893089	0.293813502
c51dc.methionine	-0.034126123	0.032383657	0.29382631
pcaac323.pcaac361	-0.035729208	0.033907102	0.293857688
c141oh.c51dc	0.100245184	0.095168438	0.294037205

serotonin.pcaac425	0.075642334	0.071815046	0.29406056
pcaec423.pcaec421	-0.00645906	0.006132576	0.294086145
pcaec443.c162	0.055617767	0.052814169	0.294156209
lysopcac180.pcaec424	-0.003311264	0.003144914	0.294242642
pcaac361.lysopcac181	0.023698978	0.022511015	0.294298555
pcaac366.pcaac420	-0.07221842	0.068601589	0.294321501
c3.c101	0.015614362	0.014832406	0.294322805
pcaec361.pcaac420	0.113552258	0.107870894	0.294346206
smc160.pcaec445	0.037918303	0.036026468	0.294417401
c3.pcaec343	-0.080091669	0.07609996	0.29444429
serotonin.smc161	-0.055647641	0.052884477	0.294537723
putrescine.leucine	0.006348199	0.006033034	0.294541948
lysopcac204.pcaec402	-0.025343604	0.024085748	0.294549129
pcaec384.pcaec444	-0.075253432	0.071519851	0.294558568
c10.pcaec383	0.042221351	0.040131614	0.294618596
pcaac342.smc240	0.045476517	0.043227824	0.294642605
smc160.pcaac421	-0.026294599	0.024996695	0.294686747
smohc221.pcaac365	0.048265481	0.045894392	0.294805169
phenylalanine.c141	-0.020655987	0.019642509	0.294836194
c0.pcaec340	0.038846716	0.036944921	0.294890932
serine.prolinr	-0.035587641	0.033846384	0.294904939
leucine.pcaec382	-0.021982737	0.020908252	0.294930292
c51.pcaac300	0.051132235	0.048633627	0.294936837
lysopcac182.pcaac381	-0.017450196	0.01660002	0.295010382
c101.pcaec445	-0.030505201	0.029021736	0.295056012
smc181.pcaac343	0.017328485	0.01648918	0.295154524
pcaec425.phenylalanine	-0.044066961	0.041938318	0.295220377
c14.pcaec383	0.055770261	0.053081094	0.295263876
pcaec360.pcaac323	-0.061449936	0.058488715	0.295278779
glycine.c141oh	0.076545136	0.072859141	0.295296277
pcaec402.prolinr	-0.031319088	0.029812509	0.295321732
pcaac404.arginine	0.026101817	0.024849198	0.295379551
pcaec360.smc240	0.041115301	0.039144793	0.295411548
c142.pcaec402	-0.0369719	0.035200338	0.295416571
pcaec383.pcaec421	0.01210384	0.011525089	0.295467555
pcaec421.pcaac323	-0.139427037	0.132782885	0.295549515
lysopcac180.lysine	0.06616337	0.063010557	0.295550176
lysopcac160.smohc241	0.074633161	0.071089344	0.295635426
smc241.pcaac421	-0.011968541	0.011402259	0.295720752
pcaac341.pcaec404	-0.031747178	0.03024797	0.295766596
asparagine.pcaec424	-0.009799307	0.009336906	0.295784909
pcaac343.c2	0.074218706	0.070725732	0.295847423
alphaaminoadipicacid.lysopcac204	0.023384063	0.02228518	0.295882975
lysopcac281.pcaec446	-0.032085289	0.030578487	0.295898337
pcaec364.pcaac365	0.028923098	0.027565085	0.295903321

pcaec443.pcaec322	0.053285667	0.050783912	0.295904664
pcaac425.pcaec340	-0.046864176	0.044674919	0.296023226
smc181.c16	0.036807312	0.035092277	0.296083755
methionine.glutamine	0.01243382	0.011855531	0.296126916
acetylornithoine.smc240	-0.010084253	0.009615539	0.296141839
pcaec386.c102	0.023173885	0.022097424	0.296156156
lysopcac161.pcaec446	-0.040776725	0.038885642	0.296193971
pcaac360.smc161	0.101062704	0.096388743	0.296258733
serotonin.smohc221	-0.030868987	0.029443576	0.296295033
lysopcac203.leucine	-0.012938572	0.012344735	0.29643599
h1.c142	0.037594695	0.035870813	0.296457295
smc180.kynurenine	-0.003842319	0.003666208	0.296467313
pcaec365.tyrosine	-0.050673878	0.048352574	0.296480343
arginine.c162	-0.074455271	0.071048382	0.296506141
pcaac421.pcaac362	-0.010755678	0.010263642	0.296511653
pcaac426.valine	-0.05182549	0.049462923	0.296592123
pcaec425.c162	0.057484145	0.054864561	0.296600396
pcaac300.pcaec383	-0.078327405	0.074760617	0.296617351
smohc222.pcaec340	0.061711434	0.058923216	0.296796449
c2.lysine	0.053213735	0.050813259	0.296832457
lysopcac281.histidine	0.063715452	0.060842909	0.29684564
pcaac362.lysopcac180	0.054892936	0.05241973	0.29686017
pcaac366.pcaec406	-0.03991165	0.038114983	0.296879806
pcaac426.lysopcac140	0.001486659	0.001419746	0.296883278
pcaac365.pcaec362	-0.045890421	0.043826205	0.296897278
creatinine.pcaac344	0.036443712	0.0348168	0.297068379
alphaaminoadipicacid.pcaec445	0.029885635	0.028554606	0.297120926
pcaec301.lysopcac280	-0.008904671	0.008508139	0.297124293
pcaac365.c18	-0.052846468	0.050496724	0.297158099
pcaec340.lysopcac204	-0.042047963	0.040181229	0.297192465
pcaac406.c14	-0.049961435	0.04774417	0.297200431
pcaac386.smc181	0.019605042	0.018735037	0.297201882
pcaec301.lysopcac140	0.00134297	0.001283652	0.297306148
c142.lysine	0.053178058	0.050832847	0.297340604
smohc141.lysopcac181	0.041214509	0.039397621	0.297349357
c142.pcaec424	0.009126112	0.008724088	0.297365304
prolinr.valine	0.057226013	0.054713984	0.297443524
pcaac405.pcaec420	-0.043308349	0.041412779	0.297507662
smc161.smc180	-0.012721832	0.012165337	0.297520656
pcaec444.asparticacid	0.022205574	0.02123609	0.297562863
c14.smc160	0.062236943	0.059520522	0.297569421
pcaac381.c16	0.020555837	0.019662869	0.297672731
smc180.pcaac402	-0.060853242	0.058225423	0.297802605
alanine.pcaac323	0.089996105	0.086113958	0.297825767
c141oh.pcaac322	-0.050936763	0.048742134	0.297851656

lysopcac204.smc180	0.010843658	0.010376461	0.297851934
lysopcac280.pcaac421	0.014153145	0.013544578	0.297895234
methioninesulfoxide.citruline	0.010806004	0.010341705	0.297911323
pcaac386.methioninesulfoxide	0.022432741	0.021473918	0.298024242
lysopcac182.pcaac421	-0.00853782	0.008173529	0.298061567
pcaac366.pcaec401	-0.049323862	0.04722335	0.298102662
glutamine.pcaac365	-0.048474728	0.046412373	0.298123363
smc261.lysopcac280	0.008798402	0.008424614	0.298154206
pcaec402.pcaec424	-0.020839195	0.019956261	0.298211819
smc202.c18	-0.022207404	0.021267444	0.29823319
glutamine.pcaac425	-0.065945145	0.063158551	0.298268451
pcaec443.pcaec402	-0.084169118	0.080626615	0.298353025
asparticacid.c141oh	0.083828755	0.08032142	0.298477929
pcaac381.pcaac405	-0.028046494	0.026875364	0.298519382
pcaec445.asparticacid	0.020037688	0.019204505	0.298607761
pcaac380.pcaec405	-0.064584276	0.06193958	0.298924661
pcaec321.pcaac380	-0.085907255	0.082390065	0.298928585
pcaac402.leucine	-0.013066536	0.012534555	0.299043244
smc160.alanine	-0.015792375	0.01514942	0.299043339
pcaec420.c18	-0.019977585	0.019166139	0.299091111
prolinr.smohc222	-0.004574087	0.004388992	0.299167238
c142.asparagine	-0.006569171	0.006304009	0.299218144
pcaac343.putrescine	0.03729894	0.03579352	0.299219975
c181.pcaec322	0.054787005	0.052577179	0.299233052
smc161.alphaaminoadipicacid	0.017811371	0.017097591	0.299363698
pcaac343.pcaac361	-0.045676291	0.043851967	0.299430931
lysopcac161.lysopcac182	-0.021889781	0.021017788	0.299483417
pcaac405.leucine	0.029659647	0.028488914	0.299665539
pcaac386.pcaac406	-0.072095531	0.069251959	0.299680839
pcaac425.pcaac421	-0.02305336	0.022144378	0.299686961
pcaec382.pcaac343	0.015424966	0.014817721	0.299717958
lysopcac280.creatinine	0.006492774	0.006237305	0.299728465
pcaac385.leucine	0.006778194	0.006512969	0.299837503
pcaac364.pcaac421	0.026993469	0.025938495	0.2998608
pcaac405.glutamicacid	-0.038772147	0.037260255	0.299905033
pcaec405.pcaec402	-0.127829072	0.122859608	0.299964353
pcaac361.c101	-0.013614598	0.013091701	0.30019909
pcaec445.c14	-0.036576467	0.035172904	0.300215975
pcaac386.pcaec424	-0.002907669	0.002796168	0.300229104
c4.pcaec382	-0.014345297	0.013797577	0.300312182
phenylalanine.pcaac344	0.07627948	0.073367942	0.30031811
c18.phenylalanine	-0.042210441	0.040599909	0.300325371
pcaac342.ornithine	0.054080736	0.05202219	0.300370681
pcaec423.pcaec342	-0.093580148	0.090022631	0.300394997
lysopcac204.alanine	-0.020722845	0.019936813	0.300437552

pcaac401.acetylornithoine	-0.034113964	0.032821516	0.300459834
glutamine.c14	0.045522952	0.043800373	0.300483035
pcaec445.c51dc	-0.099935526	0.096157142	0.300498841
pcaec403.pcaec342	0.070900316	0.068220074	0.300501464
asparagine.pcaec383	-0.020009606	0.019254092	0.300524194
pcaac363.pcaec446	-0.037130336	0.035729258	0.300535955
pcaec446.pcaac281	0.001700559	0.001636461	0.300556982
pcaac406.lysopcac280	0.00387514	0.003729409	0.30059968
c51dc.pcaec404	-0.010844681	0.01043893	0.300695696
pcaec422.acetylornithoine	-0.070601163	0.067962984	0.300719383
citruline.lysopcac140	0.002089894	0.002011994	0.300765715
histidine.smc202	-0.042162846	0.040600054	0.300870277
pcaac341.smc261	0.042323173	0.040762512	0.300965653
pcaac323.smohc222	-0.007086558	0.006825445	0.300979959
citruline.lysopcac281	0.031107073	0.029961664	0.300992339
smohc241.citruline	-0.007153026	0.006890277	0.301036769
pcaec420.pcaec341	-0.048807029	0.047026236	0.30115981
pcaac406.pcaec365	-0.02013671	0.019404221	0.301215072
pcaec421.lysopcac280	0.011880719	0.011449792	0.301267359
isoleucine.pcaac404	-0.016598051	0.015996225	0.301273532
c142.pcaac343	0.021797106	0.021008442	0.301311895
pcaec404.c181	0.017694656	0.017054658	0.301318424
c142.pcaac381	-0.012830792	0.012366825	0.301322661
pcaac322.pcaac420	0.066482269	0.064080177	0.301337235
arginine.smc261	0.027371252	0.026396171	0.30159048
smc260.spermidine	0.05334458	0.051444689	0.301594877
pcaac341.arginine	0.040576536	0.039140255	0.301704027
smohc161.pcaac365	0.052308311	0.05047635	0.301890936
smohc141.pcaec425	-0.013457525	0.012986437	0.301899333
pcaec301.lysopcac160	0.014433323	0.013928745	0.301922407
lysopcac161.c5	-0.018203883	0.017567553	0.301924203
smohc222.pcaac322	0.043811474	0.042280593	0.301930809
isoleucine.pcaac381	0.019825764	0.019134956	0.301979969
pcaac383.phenylalanine	0.047951449	0.046285935	0.302035149
asparagine.c101	-0.019983791	0.01929299	0.302117593
smc260.lysopcac180	0.048351014	0.046711038	0.302441693
c141oh.lysopcac140	-0.001892749	0.00182861	0.302457349
c10.pcaac381	-0.016642043	0.016079093	0.30248713
pcaac425.pcaac384	0.050305205	0.048612029	0.302571321
smohc221.pcaac320	0.104669044	0.101163832	0.302655813
smc180.pcaec322	0.066507922	0.064281299	0.302660532
pcaec443.pcaec361	0.018369205	0.017754425	0.302666045
pcaec300.phenylalanine	-0.02657655	0.025690946	0.302738416
c102.c3	0.034119796	0.032985047	0.302770772
pcaac426.glycine	-0.043864779	0.042420768	0.302939238

smc240.lysopcac170	-0.011433212	0.011057072	0.302949539
c141.pcaec362	0.015771939	0.015255293	0.303020068
pcaec380.lysopcac204	-0.084071434	0.08133024	0.303095618
asymmetricdimethylarginine.pcaec36	-0.005361768	0.005187317	0.303130206
alanine.lysopcac161	0.041459232	0.040113441	0.303167779
smohc241.c5	-0.006123001	0.005924796	0.303212608
smc202.pcaec401	-0.021592718	0.020896214	0.303269415
pcaac300.glutamine	-0.010370148	0.010036638	0.303317109
c141.asparagine	-0.00643325	0.006226576	0.303334341
prolinr.lysopcac281	-0.015538093	0.015039508	0.303353281
c16.c12	-0.026402083	0.025559964	0.303448799
pcaac240.c4	-0.012562371	0.01216187	0.303456205
pcaec340.pcaec424	-0.003663341	0.003547293	0.303557057
pcaac403.c4	-0.020816612	0.020158331	0.303584617
c162.pcaac422	0.056946078	0.055164474	0.303752263
c51.c182	-0.031530379	0.030546796	0.303797534
pcaac426.pcaac404	0.008427204	0.008164631	0.303815944
c162.pcaec444	-0.085508386	0.082850651	0.303853812
pcaec424.pcaec421	-0.004122754	0.003995438	0.30395341
asparagine.pcaec342	-0.050181652	0.048633296	0.303966426
pcaac343.pcaac381	0.017764136	0.017217107	0.303996757
pcaec300.pcaec424	-0.005243265	0.005082276	0.304041577
pcaec383.leucine	-0.011478311	0.011126643	0.30407447
pcaac404.lysopcac140	0.002177689	0.002111025	0.304087153
lysopcac140.pcaac344	0.057228	0.055486624	0.304178261
pcaac364.leucine	0.004416014	0.004281744	0.304189948
pcaac386.pcaac402	0.065489386	0.063504077	0.304234777
pcaec361.pcaac343	0.01900225	0.018428509	0.304295215
pcaec361.pcaec444	0.177311826	0.171974248	0.304340184
c18.pcaec403	0.032410771	0.031435925	0.304352563
pcaac362.pcaec401	0.047646683	0.046217655	0.304395103
serine.pcaec343	0.053729766	0.05213845	0.304581384
c4.pcaac384	-0.036768692	0.035684961	0.304652246
pcaec381.pcaac364	0.049870361	0.048401144	0.304658976
pcaac406.lysopcac140	0.001347857	0.001308237	0.304692013
glycine.prolinr	-0.037798434	0.036687648	0.304695522
pcaec301.c8	-0.049689808	0.04823101	0.30470992
serine.pcaec424	0.005495168	0.005334085	0.304732027
lysopcac181.c51	-0.049831716	0.048372949	0.304751714
pcaac404.prolinr	0.046209351	0.044857157	0.304757435
pcaac366.pcaac362	-0.058287978	0.056583873	0.304770533
pcaec386.alanine	-0.013063722	0.012682033	0.304779686
histidine.pcaac381	0.018711176	0.01816473	0.304786249
smohc221.kynurenine	-0.003526618	0.00342364	0.304788233
c182.pcaac365	0.026144057	0.025383274	0.304838173

pcaac403.pcaec402	-0.107210925	0.104096172	0.30486153
pcaac401.pcaec380	0.075638053	0.07344247	0.304873973
pcaec341.c8	0.034428961	0.033431115	0.304896176
citruline.glutamine	-0.02083907	0.02024626	0.305161869
ornithine.smc161	0.028236965	0.027452695	0.305495251
c18.pcaec424	-0.004124279	0.004010057	0.305534621
alphaaminoadipicacid.arginine	-0.043653097	0.042448951	0.305589454
lysopcac181.citruline	0.024753057	0.024071674	0.305617811
lysopcac182.pcaec403	0.039280952	0.038203256	0.305663194
lysopcac170.lysopcac140	-0.001558866	0.001516293	0.305725285
smc160.pcaac386	-0.010808173	0.010513283	0.305738294
h1.lysopcac160	0.040685722	0.03957577	0.30573972
pcaec403.pcaac300	-0.054013992	0.052545061	0.305782185
creatinine.pcaec424	-0.003837916	0.003733735	0.305807044
pcaec404.pcaec300	-0.037602958	0.03658248	0.305810485
c162.lysopcac140	-0.001111188	0.001081198	0.305884362
pcaac320.glutamine	-0.007756701	0.007548003	0.305925708
pcaac421.isoleucine	-0.018658947	0.018157619	0.30594432
pcaec360.pcaac421	0.015423387	0.015011107	0.306012234
lysopcac203.pcaac320	-0.042838128	0.041694517	0.306029404
pcaec300.pcaac240	-0.01694991	0.016498362	0.306057118
lysopcac281.smc160	0.094302343	0.091795076	0.306083154
pcaec380.smc161	0.074009846	0.072062297	0.306218164
lysopcac161.pcaec384	-0.038622023	0.037606693	0.306230965
c102.c141	-0.044905005	0.043728739	0.30627764
pcaec445.pcaec405	-0.030166686	0.029379857	0.306332985
pcaec363.c3	-0.036119698	0.035179827	0.306363492
smohc222.lysopcac160	-0.025663877	0.024998419	0.306408637
smohc141.pcaac344	-0.067620666	0.065870743	0.306433981
pcaac240.pcaec364	0.006452688	0.006287714	0.306588193
smc261.pcaac240	-0.008731814	0.008510079	0.306673628
lysopcac280.glutamicacid	-0.029499861	0.028751472	0.306685834
lysopcac280.lysopcac160	-0.017787017	0.017336153	0.306696372
c102.leucine	0.008759421	0.008538032	0.306732748
pcaec420.citruline	0.009110094	0.008880217	0.306753064
pcaac381.acetylornithoine	-0.03115394	0.030369837	0.306784998
pcaec342.pcaac403	-0.003772927	0.00367898	0.306917605
citruline.valine	0.070226614	0.068489928	0.307001904
c181.lysopcac280	0.020426971	0.019922108	0.307008949
serine.c3	-0.0257829	0.025147193	0.307038271
asparagine.smc241	0.016766704	0.016354012	0.307059206
pcaac300.c14	0.034282865	0.033443056	0.307117149
pcaec321.serotonin	0.080106145	0.078155828	0.307191145
c182.pcaec405	-0.037585396	0.036672381	0.307218263
asparticacid.pcaec385	-0.058277477	0.056874496	0.307325728

pcaac385.pcaec322	-0.054133711	0.052834685	0.307364009
pcaec365.pcaac385	-0.015663034	0.015287833	0.307384787
c12.pcaec362	0.013556043	0.013233207	0.30745371
pcaec363.pcaac385	-0.020311305	0.019829024	0.307488495
pcaec365.glycine	0.026903485	0.02626652	0.307522315
pcaec401.smohc221	-0.068888821	0.067259051	0.307531185
pcaac344.c14	0.047608874	0.046501722	0.307729974
pcaac385.pcaec446	-0.040597479	0.039656241	0.307764756
acetylornithoine.c4	-0.015600401	0.01523893	0.307771686
c141.pcaac281	0.001903817	0.001859742	0.307781649
smohc241.pcaec322	0.066982838	0.06543594	0.307809505
asparticacid.pcaec364	0.006380675	0.006233519	0.307824921
pcaac300.pcaec403	0.066889843	0.065348758	0.307836538
smc261.pcaac323	-0.056151094	0.054859423	0.307854118
h1.pcaac402	0.03099348	0.030280997	0.307861683
lysopcac281.pcaec360	-0.006782078	0.006626264	0.307868474
lysopcac182.pcaac343	0.017649706	0.017246284	0.307926292
pcaec401.pcaec383	-0.126355616	0.123480295	0.307976293
pcaac240.c16	0.011156709	0.010904587	0.308053987
pcaec424.pcaac343	0.01183163	0.011567558	0.308191575
pcaec381.pcaac405	-0.047416697	0.046360237	0.308210698
pcaac344.pcaac361	-0.053755704	0.052560735	0.308235704
pcaec421.pcaac384	0.037402279	0.03657102	0.308238073
h1.smohc161	0.016776181	0.016403696	0.308248735
pcaec401.histidine	0.071977932	0.070380321	0.308252348
c12.lysopcac170	-0.017342296	0.016957397	0.308253164
pcaac344.pcaec404	-0.020909516	0.020447433	0.308299985
pcaac421.pcaec422	-0.030942437	0.030260075	0.308322918
lysopcac181.serine	-0.031451845	0.030760324	0.30835544
lysopcac203.prolinr	-0.019922442	0.019487393	0.308429115
c141oh.smc261	0.063776848	0.062390711	0.308479845
pcaec380.pcaac385	-0.043709888	0.042761961	0.308503188
pcaec382.pcaac405	-0.045104805	0.04413031	0.308543415
asparticacid.smc240	0.058963792	0.057704657	0.308666919
lysopcac161.lysopcac280	0.00784582	0.007679057	0.308715846
pcaac381.lysopcac203	-0.053274964	0.052146472	0.308751602
c182.c14	-0.020737922	0.020302908	0.308852855
pcaec300.glutamine	-0.007736272	0.007574236	0.308868523
pcaec425.pcaac406	0.031228636	0.030576022	0.308891654
c18.pcaac403	-0.003804592	0.003725503	0.308945809
c141.pcaac404	0.00307594	0.003012047	0.308953687
c18.pcaec422	-0.019264541	0.018865729	0.308988149
smc241.c51	-0.049984545	0.048951427	0.309004461
lysopcac180.smc160	-0.047863977	0.0468802	0.309061116
pcaac404.pcaac240	-0.011596608	0.011360614	0.309161194

putrescine.lysopcac140	-0.001302685	0.001276278	0.309200106
c18.pcaec341	0.039491095	0.038692555	0.309224884
c181.glutamicacid	-0.05352647	0.052446542	0.309247108
pcaac361.pcaec340	0.060404433	0.059201221	0.309373175
c51.c141oh	-0.079047273	0.077486574	0.309459417
pcaec301.smc160	0.045658244	0.044783194	0.309743861
lysopcac160.c16	0.026580753	0.02607379	0.309789398
pcaec321.glutamine	-0.012503638	0.012265481	0.309801921
lysopcac140.pcaac384	-0.077223164	0.075754426	0.309815514
pcaac343.pcaac380	-0.040029925	0.039275804	0.309904181
c162.pcaac380	0.030485876	0.029922055	0.310073358
pcaec362.smc260	0.042633364	0.041850606	0.31013929
pcaec362.c18	-0.02586941	0.02540112	0.310266018
pcaec386.pcaac385	-0.023085274	0.022667594	0.310270544
pcaec322.smc261	0.03864936	0.03795975	0.310393334
pcaac406.c3	0.025969209	0.025510676	0.310484581
c51dc.tyrosine	0.043070147	0.042316263	0.310559721
c181.lysopcac180	0.025298376	0.024855864	0.310565561
asparagine.histidine	0.060397755	0.05934902	0.310628324
pcaec300.pcaac426	0.06459283	0.063480859	0.310701273
acetylornithoine.pcaac343	-0.016200176	0.015922294	0.310731732
asymmetricdimethylarginine.lysopca	-0.0183033	0.017990684	0.310767642
pcaec403.c5	-0.026831286	0.026373976	0.310785234
smc180.pcaac344	-0.059910279	0.058897595	0.310854146
asymmetricdimethylarginine.smc161	0.058493525	0.057506955	0.310872304
lysopcac170.citruline	0.026119332	0.025680256	0.310899718
pcaec444.pcaac360	-0.03342741	0.032867234	0.31092542
pcaec341.lysopcac280	0.00947918	0.009323564	0.311092717
pcaec420.pcaac421	-0.00858965	0.008449443	0.311138741
pcaec360.c141oh	0.090116267	0.088661606	0.311227298
pcaac364.pcaec401	0.0303024	0.02982427	0.311405326
c181.lysopcac140	-0.000847787	0.000834451	0.311429176
c102.pcaec403	0.054003622	0.053157619	0.311460641
kynurenine.pcaec405	-0.054347699	0.053500753	0.311500721
pcaac383.pcaec342	0.084232964	0.082924967	0.311527907
prolinr.pcaec382	-0.013193567	0.012989014	0.311539799
c51.pcaac321	0.009122378	0.00898164	0.311577131
lysopcac204.pcaac343	0.02315209	0.022798045	0.311643547
c2.pcaac365	0.044522173	0.043845352	0.311687731
pcaac381.pcaec340	-0.037913117	0.037337564	0.31169802
c102.lysopcac181	-0.070263914	0.069198358	0.311705763
c16.tyrosine	-0.070132286	0.06908628	0.311828266
pcaec420.pcaec424	-0.00438376	0.004318894	0.311885838
pcaac426.leucine	0.010474175	0.010319852	0.311916897
pcaec322.pcaec343	0.080614568	0.079434068	0.311960863

pcaec360.pcaec401	0.066528739	0.065561589	0.312012929
pcaec443.pcaec382	0.01826691	0.01800206	0.31203171
pcaec445.lysopcac280	0.006475142	0.006383471	0.312198763
pcaec361.pcaac366	-0.027813679	0.027420434	0.312207977
c4.c101	0.035026038	0.034532567	0.312232355
pcaac240.smc261	-0.064241262	0.063346894	0.312313839
c141oh.smc260	0.025962195	0.025600868	0.312316091
c162.c51dc	0.085033473	0.083850126	0.312316662
smc181.pcaac421	-0.036031389	0.035532701	0.31235375
lysopcac140.serine	-0.056930504	0.056144098	0.312366919
pcaec301.pcaac364	0.051043167	0.050340527	0.312390308
lysopcac203.lysopcac204	-0.03314822	0.032692978	0.312405979
glycine.pcaec361	0.006198481	0.006114095	0.312464419
pcaec422.c2	0.053711908	0.052991766	0.312565326
pcaec445.pcaac404	0.004656319	0.004594127	0.312590263
pcaac240.methionine	-0.041425165	0.040872433	0.312596883
pcaec424.pcaec300	0.011666039	0.01151087	0.312617398
pcaec405.pcaec340	-0.038994367	0.038479555	0.312665614
pcaac365.pcaac364	-0.052492544	0.051802371	0.312692091
glutamicacid.prolinr	-0.049188112	0.048542588	0.312704043
pcaac384.c102	0.021744695	0.02145949	0.312707714
phenylalanine.pcaac323	0.082307011	0.081255095	0.312871654
pcaec384.pcaac403	-0.002937364	0.002900453	0.312976462
pcaec380.c16	0.018599481	0.018368839	0.313057156
lysopcac161.pcaac300	-0.036235469	0.035787069	0.313069776
c2.pcaec342	-0.047133389	0.046556754	0.313138353
pcaac320.pcaec446	-0.036690747	0.03624358	0.313161128
pcaec380.c51	-0.128837599	0.1272718	0.31317781
pcaac321.leucine	0.007315399	0.007227426	0.31324002
pcaac403.leucine	-0.013391312	0.01323072	0.31325639
pcaec406.pcaac421	-0.0246346	0.024340444	0.313281517
pcaac406.methioninesulfoxide	0.029890766	0.029544268	0.313451586
smc202.c181	-0.011794883	0.011660135	0.313533448
pcaac380.pcaac405	-0.030834684	0.030490092	0.31365479
pcaec362.pcaec424	-0.006890622	0.006816907	0.313887607
pcaec363.kynurenine	-0.005663249	0.005603235	0.313936685
pcaac361.asparticacid	0.019038982	0.018840488	0.314020241
pcaac240.lysopcac203	0.025441359	0.025178788	0.314071393
pcaec360.smc180	-0.03210246	0.031772033	0.314084925
pcaec405.leucine	-0.042713222	0.042282877	0.314190952
putrescine.pcaec446	-0.063562357	0.06292822	0.314238965
methioninesulfoxide.pcaac344	0.066655056	0.065990328	0.314240898
pcaec340.pcaac386	0.010627463	0.010522069	0.314267901
c14.tyrosine	0.044646141	0.04420595	0.314295969
pcaac385.pcaac404	0.028220211	0.027942335	0.314302228

smc202.pcaac365	0.039504838	0.039121459	0.314371415
pcaac420.pcaec340	-0.023958318	0.023725862	0.314372433
pcaac381.pcaac300	-0.051397218	0.050909906	0.314480123
pcaec421.c16	0.014031432	0.013902095	0.314608425
c102.lysopcac203	-0.022304938	0.022101677	0.314659443
c0.pcaec362	0.026510927	0.026273723	0.314739923
putrescine.c181	0.011864417	0.011760137	0.314816837
pcaac422.lysopcac281	0.015936393	0.015796784	0.314830907
smc202.pcaac405	-0.026692806	0.026459353	0.314837933
pcaac405.lysopcac140	0.002510253	0.002488376	0.314852843
c14.pcaec340	0.040619142	0.040267242	0.314878062
glycine.lysopcac170	-0.017098145	0.016950077	0.314879778
pcaac321.c8	0.035276313	0.034971402	0.31488775
pcaac344.pcaac402	0.049904052	0.049474704	0.314907215
pcaec340.pcaec342	-0.124094084	0.123036359	0.314946072
c10.creatinine	-0.020102182	0.019930888	0.314947242
pcaac386.smohc221	0.029354301	0.029105619	0.314971286
c102.histidine	0.051678584	0.051248683	0.315045663
pcaac344.pcaec361	-0.01320872	0.013099438	0.315067692
c51dc.pcaec405	-0.034407404	0.034125797	0.315110938
ornithine.lysopcac280	-0.019644915	0.019487462	0.31519336
pcaac344.putrescine	0.048179787	0.047815356	0.315412496
pcaac361.pcaec341	0.065579878	0.065098894	0.315524057
pcaac344.kynurenine	-0.007150452	0.007098328	0.3155458
pcaec342.tyrosine	-0.039333046	0.039054277	0.315643969
lysopcac181.pcaac365	0.04734013	0.047006938	0.315667836
smc241.smc181	0.029590308	0.029382326	0.315672475
creatinine.c182	0.03052926	0.030316536	0.315702022
pcaec300.pcaec383	-0.043731434	0.04343647	0.315810263
lysopcac203.methionine	-0.034673037	0.034439289	0.315811925
lysopcac170.pcaac403	-0.007652255	0.007601187	0.315844852
spermidine.creatinine	0.038771551	0.038513023	0.31584761
pcaac402.isoleucine	-0.013791829	0.01369993	0.315849852
lysopcac203.serine	-0.040086307	0.039825351	0.315924342
pcaec300.pcaac385	-0.043456465	0.043176669	0.315958955
pcaec381.acetylornithoine	-0.03882367	0.038576904	0.315998978
pcaec402.pcaec445	0.032853401	0.032656716	0.316178149
smc241.pcaec443	0.046731628	0.046453699	0.316197272
pcaac361.c8	0.028451692	0.028282831	0.316203248
lysopcac180.pcaec420	0.034537497	0.034336992	0.316266099
pcaac381.pcaec424	-0.003821006	0.003799608	0.316365576
pcaec382.pcaec385	-0.072413006	0.072022483	0.316466078
lysopcac161.c3	-0.050651868	0.05037871	0.316466153
c162.smc260	0.024798209	0.024667945	0.316533979
ornithine.pcaec401	0.014237199	0.014163592	0.316574155

asparagine.smc202	-0.042464917	0.042246089	0.316582339
smohc221.pcaac362	-0.037602429	0.037409235	0.316589781
pcaec445.leucine	0.004266531	0.004244736	0.316604094
methioninesulfoxide.lysopcac180	-0.070699289	0.07033917	0.316611166
pcaac424.pcaac381	0.039647907	0.0394513	0.316676518
smc261.pcaec445	0.104183608	0.103711504	0.316883554
pcaec343.glutamine	-0.011884963	0.011836904	0.317119787
lysopcac161.c16	0.034718895	0.034580138	0.31714256
pcaec386.pcaec405	-0.055913037	0.055692613	0.317168864
phenylalanine.glutamicacid	0.044895169	0.044719946	0.317187889
creatinine.smc180	0.019747347	0.019670736	0.317199215
c181.valine	-0.050856797	0.050660463	0.317208428
methioninesulfoxide.creatinine	0.012701415	0.012656169	0.317352767
lysopcac181.valine	-0.0690927	0.068846874	0.317354881
pcaec446.lysopcac182	0.034215201	0.034099176	0.317435625
pcaac401.putrescine	-0.053791319	0.053617531	0.317513159
smc261.pcaec405	-0.021718197	0.021655578	0.317681259
pcaac381.pcaec385	-0.038802616	0.038690805	0.317682074
pcaac401.c102	-0.028723351	0.028642818	0.317719677
pcaac365.glycine	0.030768069	0.030682123	0.317724704
isoleucine.lysopcac204	-0.013728998	0.013693281	0.317817451
smc160.pcaac281	0.002062025	0.002056777	0.317844843
lysopcac160.pcaac323	-0.08486942	0.08465578	0.317858179
lysine.lysopcac161	0.070444577	0.070277245	0.317926777
serine.pcaec380	0.039804968	0.039711454	0.317939369
h1.pcaec424	-0.005104512	0.005093293	0.31801254
serine.pcaac323	0.080682048	0.080517099	0.318086696
smc240.pcaec301	0.041348641	0.041268373	0.318136547
pcaec420.pcaec445	0.042508801	0.042428859	0.31816584
pcaec301.lysine	-0.071225312	0.071095304	0.318192556
smc261.pcaec422	-0.029299882	0.029247146	0.31820485
pcaac385.pcaac321	-0.01881299	0.018780025	0.318227836
lysopcac170.smc240	-0.036389268	0.036334359	0.318345363
pcaac365.pcaac405	0.078735738	0.078630251	0.318427054
smc161.pcaac421	-0.020710101	0.020684335	0.318473228
lysopcac161.histidine	0.073164182	0.073086988	0.318564486
pcaec445.pcaec385	-0.071244153	0.071173411	0.318594467
c142.pcaac405	-0.05173778	0.051688791	0.318616721
pcaac405.lysopcac181	0.02026195	0.020245068	0.318671586
pcaec422.pcaac421	-0.051722728	0.051688613	0.318755358
smc261.pcaec385	-0.040844129	0.040820561	0.318795183
arginine.glutamicacid	0.040790903	0.040768293	0.318806151
pcaac381.leucine	-0.024338661	0.024325401	0.318810724
c12.asparagine	-0.009055561	0.009050944	0.318827632
asymmetricdimethylarginine.pcaec44	0.02305897	0.023047216	0.318827647

pcaac401.pcaec364	0.004837785	0.004835404	0.3188361
lysopcac182.pcaac383	0.011500807	0.011497834	0.318948828
pcaac343.pcaec364	-0.00803686	0.008036386	0.31904502
pcaac281.lysopcac280	0.005637835	0.005637675	0.319059778
pcaec384.pcaec341	0.054292454	0.054291369	0.319063854
serotonin.pcaac323	-0.069965085	0.069969144	0.319101463
pcaac384.pcaac421	-0.017574648	0.017578568	0.31918103
pcaac323.lysopcac204	-0.029395246	0.02940729	0.319271005
smc181.arginine	-0.020607752	0.020618258	0.319319233
c142.pcaac321	0.021482298	0.021496646	0.319395443
lysopcac180.pcaec343	0.058211752	0.05825399	0.319423237
c2.prolinr	0.017293916	0.017307312	0.319446844
c51.asymmetricdimethylarginine	0.01415816	0.014170133	0.31948107
c102.pcaec446	-0.025103374	0.025130414	0.319592602
pcaac365.c4	-0.021492227	0.021515751	0.319600953
c5.pcaec383	0.085700279	0.085809654	0.319688483
pcaac240.pcaac381	0.05660503	0.056682956	0.319736832
histidine.lysopcac140	0.002528729	0.002532247	0.319743839
c4.c142	-0.046538895	0.046610385	0.319813611
pcaec422.lysopcac180	0.047576359	0.047654568	0.319865483
pcaec420.c141oh	0.081430609	0.081577265	0.319941113
c0.pcaac386	0.031474272	0.031531371	0.319947445
pcaec340.pcaac421	0.011915703	0.011938168	0.319981705
pcaac240.pcaac425	-0.132693706	0.132960944	0.320043597
pcaac321.c14	0.053607198	0.053716555	0.320056119
pcaac421.pcaac300	-0.053494427	0.053607984	0.320095972
pcaac300.pcaac404	0.003034382	0.003040836	0.320098048
c5.pcaec340	0.074404819	0.074564507	0.32010724
pcaec420.pcaec404	-0.014188316	0.014219176	0.320121094
pcaac300.c101	-0.006368164	0.006382715	0.320173942
c8.pcaec383	0.032742551	0.032817379	0.320174168
pcaac362.c18	-0.030971258	0.031042284	0.320178011
pcaec403.pcaec322	0.039002317	0.03909329	0.320196875
pcaac383.prolinr	0.040349211	0.040461414	0.320412465
c51dc.pcaec403	0.048517537	0.048655917	0.320446786
smc181.c102	0.020573032	0.020632036	0.320454424
smc160.pcaac405	-0.026395188	0.026471471	0.320464991
c4.pcaac425	-0.081371862	0.081617802	0.320528638
arginine.c0	0.000481436	0.000482911	0.320549401
pcaec402.pcaec384	-0.051766744	0.051928777	0.320580378
pcaec322.pcaec382	-0.040327365	0.040460552	0.320663334
pcaac406.pcaac421	-0.008615262	0.008645276	0.320750408
smc241.pcaec363	-0.030861537	0.030969183	0.320752414
pcaac344.phenylalanine	-0.043692505	0.043848148	0.320788065
smc202.pcaec385	-0.030634064	0.030743794	0.320797554

pcaac240.asparagine	0.0126978	0.012745106	0.320866511
asparticacid.pcaec382	-0.010889477	0.010931434	0.320927736
pcaec386.pcaec384	0.072515871	0.072798335	0.320948027
pcaec401.asparticacid	0.030012613	0.030132447	0.320994895
serotonin.pcaec342	-0.068192672	0.068466326	0.321004575
kynurenine.pcaec340	0.028066533	0.028180375	0.321025331
serine.pcaec341	0.027909391	0.028024067	0.32105063
c101.pcaac300	-0.022551882	0.022644584	0.321051466
putrescine.valine	-0.056861169	0.057105938	0.321144657
serine.pcaac403	0.005088103	0.005110475	0.321188945
pcaec301.pcaac404	-0.017588674	0.017668227	0.321249435
lysopcac180.lysopcac280	0.005820118	0.005847449	0.321332437
smc202.smohc161	-0.010629571	0.010679591	0.321337169
smc241.pcaec385	-0.054159524	0.054419568	0.321383095
c4.serotonin	0.069856092	0.070198165	0.321428864
pcaec383.pcaac405	-0.047123848	0.047367234	0.321557441
alanine.acetylornithoine	0.03078718	0.030948264	0.321589744
smc240.lysine	0.038322664	0.038523297	0.321591268
acetylornithoine.leucine	0.017997803	0.018093914	0.321641529
pcaec405.lysopcac204	-0.055773943	0.056073581	0.321656987
lysopcac160.pcaac421	-0.011348238	0.011409496	0.321669306
c142.pcaec421	-0.001760992	0.001770626	0.321704109
c12.pcaec421	-0.00238503	0.002398464	0.321781842
pcaec300.c8	0.044692779	0.044945997	0.321797659
pcaac403.pcaec363	0.030919703	0.031098463	0.321853124
lysopcac281.c162	-0.070476756	0.070900374	0.321963046
pcaac323.transhydroxyproline	0.023010504	0.023149161	0.321970269
pcaec404.pcaac405	-0.046136871	0.046420023	0.322023656
pcaac383.smc160	-0.066395632	0.066813322	0.32209731
pcaec301.lysopcac180	-0.048857871	0.049165376	0.322098723
pcaec380.pcaac323	-0.036395799	0.036627803	0.322137337
smc261.smc181	-0.010347443	0.010416741	0.3222919
pcaac281.smc181	0.034328235	0.034561614	0.322340427
pcaac380.pcaac403	-0.00717737	0.007226538	0.322365314
pcaec381.lysopcac204	-0.071814943	0.072311255	0.322394318
creatinine.c3	0.024819389	0.024991679	0.32240905
pcaec385.pcaac281	0.001253947	0.001262663	0.322413459
c162.c4	-0.033784431	0.034020862	0.322436094
lysine.lysopcac203	0.022774908	0.022935766	0.322467079
lysopcac182.smohc161	-0.008751219	0.00881322	0.322477578
pcaac386.pcaec341	-0.058597406	0.059031643	0.322633459
pcaac363.glutamine	-0.006769567	0.006820255	0.322670348
smc181.spermidine	0.053053676	0.053452022	0.322680281
c101.histidine	0.035706532	0.035975804	0.322696025
pcaac421.pcaac403	-0.010876582	0.010959834	0.322750097

pcaec421.c51	-0.108917706	0.109780307	0.322877123
pcaac426.pcaac323	-0.060410895	0.060893377	0.322909133
pcaac281.phenylalanine	-0.035400523	0.035684879	0.322931066
pcaec422.smc260	-0.023945145	0.024138361	0.32294856
lysopcac180.lysopcac140	-0.002430939	0.002450625	0.322962589
pcaac380.pcaec402	0.05505961	0.055512489	0.323023237
pcaac300.asparticacid	-0.03147654	0.031738045	0.323062776
c101.c102	-0.033418639	0.033698386	0.323092932
pcaec445.glycine	-0.030192596	0.03044956	0.323159787
pcaac361.c4	-0.011334691	0.01143319	0.32324546
serotonin.pcaec340	0.051001077	0.051446403	0.323265369
pcaac361.putrescine	0.019574049	0.019747403	0.323324926
pcaec402.pcaec382	-0.042489605	0.042877053	0.323450286
pcaec340.pcaec445	0.041744694	0.042126854	0.323467511
c102.asparticacid	-0.036701955	0.037038627	0.323476325
pcaac386.c162	-0.043581137	0.043984057	0.323510782
lysopcac140.pcaac240	0.016864209	0.017026809	0.323700134
pcaac421.leucine	0.016653624	0.016815553	0.323739115
pcaac362.glutamine	-0.009224321	0.009314037	0.323740414
lysine.pcaec300	0.018859448	0.019047277	0.323851797
pcaac385.asparticacid	0.027937069	0.02822048	0.323940222
pcaec340.pcaac366	-0.024958439	0.025214485	0.32399476
pcaec385.pcaac424	-0.011297491	0.011414795	0.32405408
pcaac426.pcaac425	-0.12620561	0.127523787	0.324083414
pcaec384.pcaac323	-0.097207325	0.098227752	0.324108587
arginine.c182	0.024913653	0.025181967	0.324238497
smc240.pcaac385	-0.014752026	0.014911115	0.324245388
c0.prolinr	0.012145009	0.012276533	0.324267004
pcaec364.lysopcac160	-0.007999464	0.008089936	0.324495995
lysopcac161.pcaec405	-0.032962635	0.033336069	0.324505171
pcaec443.lysopcac160	-0.021239688	0.021482098	0.324545248
pcaac385.methionine	-0.020174834	0.020407257	0.324596411
serotonin.pcaac406	-0.04796068	0.048523959	0.324703261
pcaec383.pcaac343	0.024583999	0.024875466	0.324756317
c4.lysopcac170	0.028035095	0.028384607	0.325047335
pcaac401.pcaac300	-0.04719747	0.047791695	0.325106024
pcaec380.pcaac405	-0.042088663	0.042622815	0.325154077
lysopcac204.pcaac323	-0.060907664	0.061681811	0.325163151
lysopcac170.pcaec424	-0.003859139	0.003908724	0.325229193
glycine.pcaec382	0.007894087	0.007996537	0.325290657
c5.pcaec443	0.093809957	0.095028621	0.325296711
glutamine.methionine	-0.024055951	0.024375515	0.325436332
pcaec361.glutamine	-0.008839524	0.008957458	0.325463668
smc261.smc161	0.059318564	0.060131012	0.325632386
methioninesulfoxide.pcaec424	0.007599692	0.007704439	0.325673627

smc202.pcaec421	-0.001342913	0.001361481	0.325694328
c12.creatinine	-0.027078507	0.027453437	0.325703571
pcaac420.smohc221	0.037357224	0.037885051	0.325838193
pcaac321.pcaac421	0.010848217	0.011002151	0.325866999
smohc241.c51dc	-0.09088576	0.092183348	0.325908512
c141oh.pcaec361	-0.008110468	0.008226789	0.325939394
pcaec322.c5	-0.004407302	0.004470732	0.325963184
pcaec405.pcaac323	-0.039634071	0.040208354	0.326009504
pcaac380.smc160	-0.047925135	0.048620418	0.326018082
c162.pcaec402	-0.021244059	0.021555976	0.326101173
c101.c12	0.035518931	0.036064122	0.326417839
pcaac321.lysopcac170	0.017591425	0.017862125	0.326436279
pcaac362.lysopcac280	0.005303906	0.005386007	0.326479609
pcaac300.pcaac385	-0.027086207	0.02750748	0.32651457
smc261.lysopcac160	0.024190916	0.024567724	0.326525659
alanine.pcaec446	0.044075755	0.044772187	0.326632144
pcaac381.prolinr	-0.023856129	0.024233965	0.326649865
lysopcac280.lysopcac161	0.084343982	0.085680445	0.326653313
smc261.pcaac402	-0.022523478	0.022882244	0.326692751
c51dc.pcaac384	-0.035664572	0.036233808	0.326708096
smohc161.smc161	0.069298033	0.070404699	0.326712283
pcaec445.ornithine	-0.020599424	0.020930561	0.326762272
creatinine.lysopcac182	0.023194634	0.023570974	0.32683357
lysopcac170.kynurenine	-0.00761012	0.007733908	0.326852988
kynurenine.pcaac421	0.026755088	0.027192034	0.326883862
pcaac402.prolinr	-0.019081574	0.019403464	0.327138865
c16.histidine	0.058234627	0.05922026	0.327165456
creatinine.smohc161	-0.027689965	0.028160924	0.327204838
pcaec360.pcaec364	0.047160113	0.047966611	0.327248914
pcaac366.c14	-0.053678102	0.054613233	0.327400451
c102.pcaac320	-0.059791102	0.060835507	0.327422478
pcaac403.c16	0.012223318	0.012437354	0.327442805
citruline.pcaac300	0.049348351	0.050215548	0.327472412
pcaec420.leucine	0.00556168	0.005661037	0.327610543
pcaec364.pcaac380	0.052560752	0.053501422	0.327625795
pcaec322.pcaec405	-0.038453416	0.039142009	0.327630711
pcaec343.pcaac343	0.030334748	0.03088174	0.327689745
glutamicacid.pcaac281	-0.001583705	0.001612329	0.327709893
pcaac366.pcaec365	-0.024963405	0.025414737	0.327712412
pcaec380.pcaec300	0.013910536	0.014162254	0.327719872
isoleucine.lysopcac160	0.014156198	0.014412367	0.327720051
lysopcac161.prolinr	0.041265532	0.042019261	0.327800281
pcaac420.c18	-0.025881891	0.02636267	0.327947246
pcaac402.pcaac406	0.053654549	0.054652092	0.32795487
c141oh.pcaec301	-0.029780668	0.030338165	0.328015511

pcaac341.c2	0.057848467	0.058932092	0.328021202
c0.pcaac425	-0.059266288	0.060385305	0.328091714
pcaec380.lysopcac140	0.001402887	0.001429508	0.32813653
pcaec321.pcaac421	0.008746461	0.008912916	0.328162633
smc240.smohc141	0.036121677	0.036829431	0.328428619
pcaec343.pcaec363	-0.052570792	0.053608312	0.32849578
pcaec383.c16	0.012306889	0.012550728	0.328532435
pcaac420.pcaac362	-0.011144527	0.011366588	0.328585547
glutamicacid.smc241	-0.054747277	0.055850675	0.328693656
pcaec343.methionine	0.03062667	0.031245851	0.328723263
smc181.lysopcac181	0.038581842	0.039362957	0.328736779
pcaac422.glutamine	-0.010507616	0.010720577	0.328747025
pcaec445.pcaac381	0.015003314	0.015308775	0.328790583
c141.pcaac240	-0.008613372	0.008789297	0.328821278
pcaac422.c141oh	0.080532797	0.082191723	0.328903853
c182.pcaec402	-0.016302472	0.016642066	0.329013133
pcaec386.pcaac381	-0.032341088	0.033015992	0.329030841
pcaac323.pcaec421	-0.001308223	0.001335536	0.329035418
pcaec321.c3	-0.026248066	0.026796104	0.329035985
glutamine.asparticacid	0.034945619	0.035681075	0.329114589
c181.pcaac344	-0.027826132	0.028415363	0.329175837
pcaec360.transhydroxyproline	-0.038746135	0.039567924	0.329191929
pcaac385.smc181	-0.044574097	0.045520809	0.329205852
pcaac322.pcaac380	-0.031607267	0.032280244	0.329230762
lysopcac181.smc261	0.039112278	0.0399459	0.329241014
pcaac405.alanine	0.056054853	0.057251789	0.329259587
pcaac402.creatinine	0.013154325	0.013435223	0.329260145
pcaec301.lysopcac281	0.007992882	0.008164836	0.32933527
lysopcac161.pcaac402	-0.02613225	0.026696049	0.329364287
pcaec361.lysopcac181	0.028487457	0.029115682	0.329589682
smc260.pcaec384	0.053469476	0.054655634	0.329651523
lysopcac204.pcaac403	-0.00293986	0.003005089	0.329653342
pcaac366.creatinine	0.008889031	0.009086867	0.329685605
pcaec422.transhydroxyproline	-0.031447805	0.032148479	0.329697115
pcaac361.smohc221	0.036968471	0.037798147	0.329773602
pcaec361.phenylalanine	-0.042331305	0.043282907	0.329791075
smc160.smc240	-0.049530819	0.050654159	0.329885211
pcaec385.lysine	-0.062613512	0.064040291	0.329935857
c51.smohc222	0.003276675	0.003351589	0.329971609
pcaec405.pcaec422	-0.042788749	0.043768887	0.32999209
pcaac384.lysopcac280	0.00395749	0.004048556	0.330041394
asymmetricdimethylarginine.smc180	-0.019654612	0.020108827	0.330087912
pcaac422.leucine	-0.023975836	0.024530838	0.330106077
pcaac386.lysopcac140	0.000878195	0.000898689	0.330194512
pcaac425.pcaec446	0.096080833	0.098336623	0.330261331

pcaec300.smc180	-0.011613407	0.011892662	0.330528665
pcaac420.pcaec382	-0.027833491	0.028506533	0.330592221
pcaac404.pcaac422	-0.031472521	0.032250724	0.330848771
c182.pcaac422	0.023727501	0.024315536	0.330875311
asparagine.pcaec341	0.051409101	0.052684372	0.330886355
pcaac422.creatinine	0.009204355	0.009432844	0.330894651
pcaac402.lysopcac180	-0.065696623	0.067328478	0.330901837
c12.pcaec301	-0.052397787	0.053704268	0.330946337
pcaec360.histidine	0.042373538	0.04343989	0.33105522
lysopcac204.prolinr	-0.023498912	0.024096481	0.33117937
pcaec401.c141oh	0.0785824	0.080602467	0.331309384
pcaac344.pcaec382	0.008864276	0.009095582	0.331491551
pcaec385.lysopcac140	-0.003021249	0.003100471	0.331551453
c10.lysine	0.051865709	0.053228603	0.331577563
pcaec340.arginine	-0.029327719	0.030101731	0.331631309
pcaec421.isoleucine	-0.011694977	0.012007042	0.33176831
pcaec403.c141oh	0.071087073	0.072989288	0.33180362
lysopcac280.c51dc	-0.092371075	0.094858128	0.33188135
pcaec364.putrescine	-0.075218593	0.077260201	0.331983514
pcaac360.pcaac344	-0.063064418	0.064776425	0.331985675
pcaec401.c16	0.016467809	0.016915687	0.332009245
pcaec404.pcaec363	0.034224973	0.035158367	0.332044492
pcaec423.pcaac300	-0.042567698	0.043734374	0.332107902
pcaac364.alanine	-0.013915497	0.014297617	0.332132501
pcaec322.smc180	-0.021157324	0.021741163	0.332195867
pcaec386.lysopcac170	0.01350758	0.013881243	0.332227795
c10.smohc241	0.062706354	0.064455433	0.332335601
ornithine.putrescine	-0.036838441	0.037866436	0.332341375
methionine.c8	-0.035795012	0.036797833	0.332393019
pcaec444.pcaac421	0.009846109	0.010122929	0.332439436
pcaec421.smohc221	-0.081483363	0.083777338	0.332457231
pcaec383.glutamine	-0.012871925	0.013234469	0.332463201
c101.glutamine	0.00972583	0.010001644	0.332553886
pcaac321.pcaec380	-0.054266078	0.055809632	0.33259381
creatinine.ornithine	0.028848789	0.029674425	0.332675901
c5.pcaac321	0.046930284	0.048278705	0.332728838
pcaec321.pcaac381	0.01747793	0.017980552	0.332740598
pcaac320.smc260	-0.020669235	0.021264525	0.332760856
pcaac422.pcaac362	-0.012214511	0.012567053	0.3327898
pcaec381.pcaac365	-0.048242054	0.04963782	0.332822551
pcaac426.smohc221	-0.046179465	0.047516223	0.332829332
alanine.pcaec444	0.06374922	0.065607703	0.332925782
pcaec446.c51dc	-0.089763311	0.092381397	0.332932106
smc261.isoleucine	0.020016606	0.020602085	0.332971014
pcaec364.pcaac403	-0.003031196	0.003121007	0.333148441

lysopcac182.pcaec341	0.030318111	0.031218158	0.333175583
asymmetricdimethylarginine.pcaac42	-0.032668784	0.033645669	0.333276594
smc240.pcaec424	-0.002502791	0.002577859	0.333319149
pcaac323.pcaac300	-0.032795526	0.033785316	0.333406599
pcaec424.smc202	-0.065681886	0.067668018	0.333433724
ornithine.pcaac383	0.00986847	0.010167486	0.333462483
pcaac300.lysopcac204	-0.013201674	0.013602725	0.333499228
pcaac320.pcaac425	-0.086141989	0.088762555	0.333519207
pcaac404.c14	0.022705472	0.023397709	0.33355017
pcaac362.smohc222	0.018244883	0.018803024	0.333598743
c18.pcaec385	-0.04697738	0.04842381	0.333691444
pcaec424.pcaac421	0.011158601	0.011503348	0.333740663
c8.pcaec445	-0.043956477	0.045315815	0.333754391
pcaac385.arginine	0.025427959	0.026217557	0.333814062
smohc161.pcaac322	0.04803886	0.049546261	0.333966558
pcaec300.c12	0.040058733	0.041315858	0.33396809
pcaec385.pcaec360	0.003380153	0.003486959	0.334068893
lysopcac170.tyrosine	-0.056279886	0.058060393	0.334086991
pcaec321.pcaac323	-0.034931495	0.036037859	0.334103677
pcaec342.alanine	-0.014813863	0.01528576	0.334188984
pcaec385.smohc241	0.056475003	0.058277431	0.334217182
serine.pcaac321	0.019076893	0.019687003	0.334248048
smohc221.lysopcac182	-0.018724596	0.019324979	0.334286453
smc161.pcaac364	0.036460755	0.037630369	0.334293367
pcaec445.kynurenine	0.014292694	0.014751676	0.334309366
alphaaminoadipicacid.pcaac404	0.012638942	0.01304569	0.33434165
pcaec425.pcaac421	0.012754398	0.013164875	0.334342096
pcaac361.glutamine	-0.006100973	0.006299186	0.334484683
lysopcac182.pcaac385	0.02316778	0.023926826	0.334612634
pcaec406.pcaec424	-0.005235622	0.005407569	0.334649407
pcaac383.methionine	-0.025023014	0.025844895	0.334650878
pcaac402.c16	0.011787702	0.012179481	0.334833306
lysopcac281.smohc161	0.011367893	0.011747714	0.334915099
methionine.pcaec424	0.009987488	0.01032149	0.334929263
c0.pcaac402	0.039852054	0.041189992	0.334990104
smc261.histidine	0.039946505	0.041288539	0.335000895
glycine.pcaec401	0.017447411	0.018034759	0.335032644
lysopcac161.pcaec425	0.00720565	0.007448709	0.335064285
asparagine.pcaec380	-0.055226742	0.057098073	0.335135432
pcaec403.pcaec404	0.038557073	0.039870462	0.335218803
pcaec380.creatinine	0.008651556	0.008946521	0.335232962
pcaac360.pcaac361	-0.042444604	0.043895407	0.335273574
asymmetricdimethylarginine.pcaac38	-0.022182659	0.022943362	0.335325558
c162.pcaec322	0.052660797	0.054471777	0.33537064
ornithine.pcaac240	0.006947486	0.007188788	0.335530236

pcaec443.isoleucine	-0.013540229	0.014011072	0.335549444
c16.pcaac321	0.01460175	0.015109813	0.335559254
pcaac384.creatinine	-0.007019648	0.00726733	0.335787012
pcaac403.creatinine	0.009561214	0.009902136	0.335960317
pcaac321.transhydroxyproline	-0.019839124	0.020547744	0.335988939
serine.arginine	-0.054886019	0.05685278	0.336042518
pcaac421.tyrosine	-0.048464192	0.050202361	0.336057151
pcaec321.pcaec424	-0.005996065	0.006211186	0.336062759
pcaec401.isoleucine	-0.016778198	0.017381012	0.336086633
smc261.lysopcac182	-0.031155137	0.032275775	0.336105777
lysopcac182.alanine	-0.019854989	0.020571185	0.336153076
pcaec383.isoleucine	-0.01217423	0.012614661	0.336202305
c142.pcaec301	-0.049699175	0.051498152	0.336211624
pcaec402.pcaac322	0.042474859	0.044015554	0.336246844
c5.pcaec422	-0.02876477	0.029810446	0.336283807
methioninesulfoxide.lysopcac204	-0.122427128	0.126890449	0.336332277
pcaac402.pcaac300	-0.049244959	0.051045712	0.336383525
pcaec423.c101	-0.028488315	0.029535438	0.33647131
c51dc.lysine	-0.065932823	0.068357912	0.336482913
kynurenine.lysopcac203	-0.020548438	0.021309695	0.336606323
pcaec301.pcaec406	0.01149935	0.011926128	0.33663711
pcaac322.smc240	0.038125819	0.039541796	0.336649349
alanine.pcaac321	-0.030228027	0.031351261	0.336658215
c0.pcaec361	-0.008921708	0.009255531	0.336778131
c18.pcaec380	-0.013448624	0.013952488	0.336800816
smohc161.lysopcac170	0.012636029	0.013111226	0.336866121
smc160.c51dc	-0.08279329	0.085908323	0.336874309
pcaac402.c4	-0.023150947	0.024029098	0.337016897
glycine.pcaec363	0.010406754	0.010801745	0.337027939
pcaec322.pcaec340	-0.047164554	0.048955151	0.337032385
smc241.transhydroxyproline	-0.030473796	0.031631345	0.337041733
pcaec446.smc161	-0.062975481	0.065382582	0.337152028
c14.pcaac426	0.055903478	0.058048292	0.337218601
pcaec423.pcaec404	-0.017979582	0.018678637	0.337456942
smc160.transhydroxyproline	-0.024647505	0.025609612	0.337528391
pcaec420.pcaac424	-0.010273844	0.010675226	0.337544
pcaec406.lysopcac161	-0.082130784	0.085339962	0.33754662
c101.phenylalanine	-0.037813607	0.039291317	0.33754884
c12.lysopcac280	-0.009318677	0.009683543	0.337583827
ornithine.pcaac380	0.045022486	0.046791309	0.337645568
smc181.pcaec383	0.055776428	0.057995769	0.337878264
serotonin.methioninesulfoxide	0.025585299	0.026610074	0.338000179
pcaac344.pcaec300	0.012366243	0.01286192	0.338013972
pcaac300.creatinine	0.006384815	0.006641126	0.338042117
pcaac364.pcaec404	-0.020098612	0.020907198	0.338082405

pcaac343.c8	0.031278773	0.032537688	0.338090419
pcaec300.pcaac366	-0.022379067	0.023281009	0.338115744
pcaec362.pcaac420	0.055379854	0.057617664	0.33816454
histidine.pcaec383	-0.040268444	0.041896401	0.338173429
smohc222.lysopcac180	0.05091397	0.052972409	0.338174421
pcaec300.pcaac365	-0.043993424	0.045784418	0.33830432
spermidine.c8	0.01110292	0.011555254	0.338317999
lysopcac281.c4	-0.01055437	0.010985623	0.338373569
pcaac401.c141	-0.031684801	0.032979562	0.338375215
glutamicacid.pcaec385	-0.072698575	0.075669796	0.338378293
lysopcac181.c162	-0.077873454	0.081058257	0.338390662
c12.pcaec405	-0.028132007	0.02928552	0.338439885
serotonin.smc240	0.041266619	0.042964262	0.338502227
smohc221.spermidine	0.049981364	0.052042964	0.338552612
methioninesulfoxide.c4	-0.02176105	0.022664674	0.33868088
pcaac381.pcaac426	0.076433257	0.079609518	0.338695287
pcaac420.alanine	-0.009593231	0.009994436	0.338818062
pcaac422.pcaac406	0.038576917	0.040191662	0.338834751
methioninesulfoxide.c16	0.01851943	0.019296615	0.338884711
creatinine.pcaac384	0.049201692	0.051266617	0.33888593
pcaac344.pcaac363	0.017957468	0.018711355	0.338892058
methionine.pcaec444	-0.0914368	0.095277473	0.338902105
pcaec423.c51	-0.076270128	0.079480449	0.338942703
asymmetricdimethylarginine.pcaac36	-0.036793474	0.038344953	0.338977699
pcaec420.lysopcac280	0.005902721	0.006153366	0.339114087
h1.pcaac384	-0.0606104	0.06318462	0.339118267
smc260.smc202	-0.031094907	0.032419144	0.339171541
pcaac323.c3	0.033257821	0.03467617	0.339199292
c101.creatinine	-0.00476132	0.004965248	0.339283798
lysopcac281.pcaac426	0.059795092	0.062378788	0.339458726
smc261.pcaac362	-0.010767905	0.011233793	0.339485111
pcaec401.methionine	-0.03695786	0.038557075	0.339487393
smohc221.c51dc	-0.092068426	0.096055846	0.339504926
pcaec424.lysopcac161	0.043303783	0.045181307	0.339526953
pcaec402.tyrosine	-0.047892287	0.049971386	0.339552291
pcaec340.c162	-0.061226879	0.063887278	0.33957052
putrescine.asparagine	-0.00402098	0.004195816	0.339584166
pcaac365.lysopcac140	0.001258398	0.001313246	0.339632239
smohc241.smc181	0.018343729	0.019143474	0.339637962
putrescine.pcaec385	0.058670647	0.061235079	0.339689264
pcaac360.pcaac300	-0.044980807	0.046946962	0.339690208
pcaec404.pcaec321	-0.036169301	0.037750547	0.3396934
c101.c142	-0.058083362	0.060666962	0.340045127
lysopcac140.pcaec446	0.091257755	0.095320674	0.340063774
pcaec342.alphaaminoadipicacid	0.010515567	0.010984536	0.340098884

c181.smc160	0.052622336	0.054970033	0.340106488
lysine.smc181	0.010705206	0.011182847	0.340108139
pcaec341.pcaac421	0.01061905	0.011092938	0.340112083
pcaac366.citruline	0.010678867	0.011158519	0.340245586
glutamine.pcaac343	-0.016148842	0.01687553	0.340283987
c4.lysopcac160	0.00553851	0.005787753	0.340285145
lysine.c12	0.06124116	0.064001773	0.34032007
smc160.smc181	0.022991997	0.024029334	0.340338286
pcaac323.smc261	0.033760075	0.035283471	0.340341463
creatinine.c102	0.036385973	0.038034111	0.340420559
pcaac363.pcaac321	-0.018393913	0.019228583	0.340458109
methioninesulfoxide.pcaac380	0.054764271	0.057272223	0.340650446
kynurenine.lysopcac182	0.036143624	0.03780026	0.340668583
pcaac383.glutamine	-0.009282648	0.009713365	0.340928659
asymmetricdimethylarginine.c5	-0.007909402	0.008276926	0.34095922
ornithine.pcaac381	0.013313921	0.013933377	0.340986953
c12.c181	0.017018423	0.017812624	0.341051412
pcaac320.pcaac366	-0.012439675	0.013023105	0.341158828
pcaac344.lysopcac281	0.00725621	0.007598514	0.341284339
pcaec425.pcaec363	0.027511283	0.028810183	0.341302474
c102.pcaac420	-0.05167182	0.054111786	0.341305724
pcaac420.pcaec443	0.036803334	0.038545247	0.34135621
alanine.pcaec401	0.012694202	0.013296626	0.341414245
c4.pcaac380	0.01925073	0.020164475	0.341418324
acetylornithoine.pcaac322	0.050753546	0.053163095	0.341422899
smc240.lysopcac280	0.005365776	0.005620658	0.341434843
lysine.c182	0.055929979	0.058589907	0.34146089
phenylalanine.c2	-0.050835095	0.053256344	0.341493644
lysopcac170.smohc241	0.049085272	0.051426931	0.341528761
c182.lysopcac181	-0.036114505	0.037841196	0.34157725
pcaac240.pcaac365	0.062489434	0.065477258	0.341578045
pcaec421.creatinine	0.012381545	0.012974743	0.341622413
c12.c102	0.031434158	0.032945176	0.341695596
smc161.c16	0.026549961	0.027833291	0.341818219
pcaec402.leucine	-0.020145541	0.021119475	0.341822117
pcaac405.pcaac406	0.042314311	0.044362185	0.341845894
pcaec383.creatinine	0.007950881	0.008336239	0.341878311
c181.pcaac386	0.012273319	0.012868288	0.341882581
pcaec381.c141oh	0.058812385	0.061667706	0.341916072
pcaec321.alanine	-0.011473123	0.012030411	0.341926947
pcaec363.tyrosine	-0.039785408	0.041718195	0.34193014
smohc161.smc240	-0.018039027	0.018917912	0.341994817
pcaec422.pcaec401	0.07081957	0.074300141	0.342190099
pcaec445.pcaec403	-0.031602057	0.033163268	0.342307097
pcaec365.creatinine	-0.01822395	0.019128166	0.342405537

pcaac343.glutamine	-0.007000633	0.007348732	0.34245454
smc260.pcaec424	-0.004665376	0.004897383	0.342457135
alphaaminoadipicacid.serotonin	0.069896351	0.07338604	0.342547455
pcaac366.pcaec300	0.013942889	0.014639627	0.342567721
pcaac366.pcaac323	-0.033444278	0.035118506	0.342608668
pcaac386.pcaac321	-0.015894609	0.016695698	0.342764347
pcaac364.methionine	-0.018122029	0.019036594	0.342795006
pcaac281.pcaec385	-0.044106889	0.046338226	0.342851002
prolinr.pcaec425	0.013293753	0.013970185	0.342985665
phenylalanine.pcaac365	-0.040448747	0.04251073	0.343028791
pcaac425.lysopcac203	-0.045200958	0.047510031	0.343077731
pcaac403.pcaac380	0.058832018	0.061837746	0.343080205
histidine.pcaec423	0.025490227	0.026797927	0.343177219
c142.histidine	0.042472345	0.044660052	0.343271906
pcaec423.smc160	-0.039891856	0.04195116	0.343323679
pcaac366.smc161	0.058277203	0.061288915	0.343349702
lysopcac170.phenylalanine	-0.04101406	0.043134183	0.343355888
c162.pcaec404	0.028635072	0.030118489	0.343406941
pcaac360.c16	0.024007938	0.025252421	0.343421604
pcaac380.pcaac385	-0.026271357	0.027638188	0.343509006
pcaec360.pcaec421	-0.001382864	0.001454964	0.343559516
c182.c51dc	0.083507121	0.087861098	0.343560009
smc241.pcaac425	0.079454018	0.083599377	0.343575582
pcaac344.alanine	-0.02874681	0.030250543	0.343637985
smc261.pcaec423	0.025593838	0.026938369	0.343740262
pcaac360.glycine	0.03774042	0.039727459	0.343793627
c101.lysopcac140	-0.001399982	0.001473801	0.343829441
c12.pcaec401	-0.018791793	0.019784008	0.343862267
pcaac300.smohc221	-0.028947998	0.030482454	0.343956795
putrescine.pcaec384	0.011581905	0.012196476	0.343982221
pcaac364.pcaac422	-0.036572028	0.0385178	0.344046531
pcaec342.smc160	0.101522784	0.10696337	0.344222747
lysopcac281.asparagine	0.007004903	0.007380892	0.344261672
pcaec362.pcaec340	0.05182116	0.054604223	0.344275393
c141.c5	-0.007360262	0.007755595	0.344278441
glutamicacid.pcaec380	0.045479059	0.047924087	0.344301165
pcaec386.pcaec383	0.060065331	0.063297087	0.344320513
pcaac281.lysopcac170	0.009556836	0.010071485	0.344342146
c4.pcaec341	0.044803372	0.047222339	0.344405715
smc240.pcaec382	-0.006614094	0.006971895	0.344454056
pcaec385.pcaec342	-0.091123916	0.096057716	0.34447558
pcaac406.isoleucine	0.023192464	0.024450305	0.344517112
histidine.smohc222	-0.005083117	0.00535891	0.344527077
pcaac364.pcaac321	-0.020062831	0.021157987	0.344677369
pcaac364.c141oh	0.063117964	0.066564792	0.344687863

c10.pcaec341	0.051491502	0.054308687	0.344734519
lysine.pcaac320	0.09228692	0.097341431	0.344760913
pcaec301.pcaac361	-0.044944759	0.047411042	0.344808372
kynurenine.isoleucine	0.015652355	0.016517538	0.344991264
pcaac425.c141oh	0.068997334	0.072818504	0.345039778
pcaec444.pcaac381	0.013219179	0.013951832	0.345058974
lysopcac203.pcaec402	-0.023343666	0.024638188	0.345073299
pcaec443.creatinine	0.009029596	0.009530781	0.34509594
pcaec422.pcaec382	-0.010969696	0.011578723	0.34510247
pcaac401.lysopcac170	-0.025424887	0.026837141	0.345114809
pcaec446.kynurenine	0.007641252	0.00806701	0.34519328
pcaec383.lysopcac203	0.013951557	0.014729039	0.34519735
pcaec423.pcaac420	-0.092142524	0.097279916	0.345209885
pcaac381.pcaec401	-0.065070673	0.068708632	0.34527955
serine.pcaac364	0.052523069	0.055467305	0.345347045
isoleucine.asparagine	0.006996694	0.007389759	0.345402844
pcaac383.creatinine	0.006326589	0.006682445	0.345434343
citruline.lysopcac204	0.035564696	0.037578582	0.345606454
pcaac300.leucine	0.005941841	0.006279384	0.345689163
smc161.pcaec424	-0.005871252	0.006204805	0.345690794
histidine.pcaec421	0.002366851	0.002501433	0.345713391
pcaac343.smc240	0.034810316	0.036793802	0.34576743
c181.pcaec342	-0.05576392	0.058941928	0.345772233
pcaec361.pcaac384	-0.040563757	0.042889903	0.345933687
smc240.pcaac420	0.039799506	0.042084528	0.345964557
pcaec443.pcaac405	-0.041288992	0.043660069	0.345970485
smc160.pcaac420	0.052139563	0.055135235	0.345983439
pcaac323.smohc141	-0.039336555	0.041600817	0.346031811
glutamicacid.pcaec343	0.056191364	0.059431169	0.346075158
pcaec343.lysopcac204	-0.018652237	0.019732651	0.346196729
ornithine.alanine	0.01791416	0.018954531	0.346265429
c162.lysopcac160	0.01113158	0.011778769	0.34629475
pcaec361.smohc222	-0.009216431	0.009752658	0.346313756
pcaac422.lysopcac204	0.043994853	0.046557641	0.346345677
arginine.pcaec380	0.039667825	0.04198475	0.346416621
pcaac366.lysopcac140	0.001378548	0.001459071	0.346418015
pcaec321.pcaac321	-0.026278324	0.027814836	0.346445
c182.c181	0.011359353	0.012024685	0.346490686
pcaec402.lysopcac280	0.012302277	0.013023523	0.346516035
lysopcac203.creatinine	0.006010775	0.006364129	0.346588467
pcaac401.pcaec363	0.031143816	0.032976184	0.346610741
pcaec385.lysopcac161	-0.06907679	0.073153342	0.346692037
lysopcac182.smc202	-0.034510237	0.036551938	0.346758944
c141oh.pcaec341	0.037417815	0.039640428	0.346866782
pcaec365.alphaaminoadipicacid	0.007457264	0.007900238	0.346867588

spermidine.leucine	0.042459858	0.044984978	0.346898948
smohc161.pcaac381	-0.026479686	0.028058593	0.346969867
pcaec425.pcaec443	0.054761092	0.058034538	0.347037795
lysopcac204.c141	0.02475188	0.026232041	0.347048265
pcaac322.methionine	-0.01973076	0.020911136	0.347059238
pcaac323.pcaac406	0.022585565	0.023936744	0.347059573
pcaac403.asparticacid	0.042723019	0.045279854	0.347069498
pcaec403.isoleucine	-0.009882024	0.010474486	0.347117878
pcaac424.pcaac422	-0.117326223	0.124362539	0.34712639
pcaec402.kynurenine	-0.005216986	0.005530086	0.347145969
smc240.c141	-0.038376169	0.040679628	0.347149475
pcaec301.c18	0.040908355	0.043365835	0.347171995
pcaac321.lysopcac160	-0.01514107	0.016050876	0.347179174
lysopcac181.glutamine	-0.009053972	0.009598571	0.347207123
pcaac320.pcaac422	-0.022586994	0.023947029	0.347235631
pcaac300.c18	0.028999575	0.030746817	0.347252584
pcaac343.pcaac420	0.084999884	0.090133335	0.347317416
pcaac405.kynurenine	-0.006495799	0.006890879	0.347511006
pcaac403.h1	0.003378226	0.003583887	0.347537119
pcaac403.pcaec385	-0.064525694	0.068461004	0.347586835
asymmetricdimethylarginine.pcaec38	0.043358984	0.046009579	0.347651664
smohc222.pcaac300	0.05238995	0.05561213	0.347820275
smc202.smc261	0.030917654	0.032820722	0.347842467
pcaac320.lysopcac161	-0.07905404	0.083934192	0.347923506
pcaec406.pcaac384	-0.032943846	0.034979634	0.347952406
c5.pcaac385	0.10165651	0.107971293	0.348098616
pcaac320.pcaec444	0.080630232	0.085661175	0.348223646
asymmetricdimethylarginine.leucine	-0.005346574	0.00568063	0.348262155
pcaac361.pcaec342	0.089887018	0.095510175	0.348297281
methionine.pcaac344	0.040027122	0.042533568	0.348324709
ornithine.c142	-0.054658106	0.058090238	0.34840339
c0.spermidine	-0.050985603	0.05421709	0.348668972
histidine.lysopcac161	0.058848236	0.062585854	0.348728814
smc161.pcaac381	-0.024109793	0.02564461	0.34879506
lysopcac180.c12	-0.045208453	0.048090295	0.348833974
pcaec424.pcaac365	0.043246747	0.046006843	0.348868482
pcaec360.pcaac381	0.031387053	0.033399492	0.349001589
pcaec385.pcaac321	-0.017138789	0.018243133	0.349145367
lysopcac203.lysine	0.053646312	0.057105966	0.349170059
pcaac342.glutamine	-0.008410115	0.008952538	0.349172944
serotonin.pcaec363	-0.017202329	0.018314952	0.349255139
pcaec422.asparticacid	0.023590688	0.025125789	0.349432752
pcaec321.pcaec340	-0.066656328	0.070994391	0.349436673
pcaac322.pcaac402	0.042800901	0.045601694	0.349597499
pcaec424.c3	-0.024828298	0.026460797	0.34973896

c51.pcaec382	-0.007682303	0.008187429	0.349739141
pcaac322.c51dc	-0.074022501	0.078891561	0.349750934
citruline.pcaec365	-0.014716432	0.015685747	0.349790603
pcaec384.c5	-0.004216206	0.004493975	0.349797515
pcaec385.kynurenine	-0.006586712	0.007024278	0.350045369
alphaaminoadipicacid.pcaec422	-0.02309903	0.024636608	0.350105257
pcaac344.c3	0.033575462	0.035811658	0.350122126
pcaac240.pcaac322	0.052077229	0.055545923	0.350124213
pcaec445.pcaac361	0.034239802	0.036521363	0.350136835
pcaec361.pcaec402	-0.09215098	0.098297207	0.350165019
smohc141.smc240	-0.024863975	0.026527517	0.35025882
c16.c2	0.076849384	0.081992229	0.350265743
lysopcac170.lysine	0.055416491	0.059129332	0.350300742
pcaec341.smohc222	0.004166536	0.004445776	0.3503101
pcaac300.pcaec301	-0.047250785	0.050428814	0.350417712
pcaac425.lysopcac140	0.000907277	0.000968515	0.350524395
pcaac281.pcaac321	-0.019204314	0.020502597	0.35057291
pcaec381.smc202	-0.061073154	0.065213967	0.350661581
smc161.pcaac344	-0.045955884	0.049077471	0.350717725
pcaac420.pcaac300	-0.031076767	0.033187903	0.350720967
smc180.pcaec424	-0.004371407	0.004668492	0.350733559
smohc141.pcaac420	0.056381376	0.060220761	0.350794587
pcaec381.pcaac300	-0.048434539	0.051736253	0.350826912
lysopcac181.lysopcac161	-0.120132395	0.128357723	0.350961882
pcaec385.c102	0.019121522	0.02043157	0.350981131
pcaec340.lysopcac170	0.011366199	0.012146554	0.351045865
smc161.pcaac405	-0.040706214	0.043506628	0.35110875
serotonin.pcaec444	-0.053190527	0.056851079	0.351119484
serine.pcaec422	-0.02432347	0.026001308	0.351191631
pcaac426.pcaac422	-0.028345214	0.030301091	0.351201417
c141oh.pcaac321	0.015432329	0.016498952	0.351252658
pcaac320.pcaec404	-0.017155768	0.018342392	0.351275808
pcaac363.lysopcac280	0.006571138	0.00702565	0.351275961
c182.pcaac240	-0.011142505	0.011913295	0.351279412
pcaec342.creatinine	0.004553601	0.004868682	0.351287572
pcaac344.arginine	0.037461565	0.040060563	0.351370137
pcaac281.c101	-0.007505082	0.008027002	0.351444011
c51dc.kynurenine	0.006281716	0.006718824	0.351462823
pcaec381.pcaec322	0.045044085	0.048179076	0.351469148
pcaec446.c3	-0.013108314	0.01402292	0.351547552
ornithine.pcaec446	0.015594046	0.016683313	0.351582768
ornithine.asparticacid	0.017344295	0.018561752	0.35173625
pcaec342.c101	-0.005955337	0.006373419	0.351740429
pcaec321.smohc241	-0.088642099	0.094870176	0.351766487
c12.smc180	-0.007860744	0.008414149	0.351829377

pcaac384.lysopcac170	0.006814437	0.00729424	0.351833286
citruline.threonine	-0.038148021	0.040837021	0.351868581
pcaac362.pcaec363	-0.024524625	0.02625766	0.351947725
smc241.pcaac281	0.001063009	0.001138328	0.352032424
phenylalanine.pcaac421	0.013608519	0.014574687	0.35209664
h1.phenylalanine	0.047081917	0.050426747	0.352117049
methionine.pcaec404	0.011230076	0.012028228	0.352130475
pcaec340.smohc161	0.019863258	0.021275326	0.352137943
pcaec444.glutamine	-0.007672756	0.008219187	0.352195128
pcaec444.pcaec380	0.032030692	0.034319922	0.352308428
pcaec380.acetylornithoine	-0.026712107	0.028622444	0.352328987
pcaac402.pcaec301	0.065931411	0.070649286	0.352347595
smc202.lysopcac204	-0.026102994	0.027975395	0.352425522
lysopcac160.pcaec383	-0.090174481	0.096662101	0.352521302
pcaac422.glutamicacid	0.049587379	0.053165171	0.352613573
c18.c141	0.017992554	0.019291231	0.352625795
methionine.pcaec383	-0.049129778	0.052682867	0.352689312
pcaec423.lysopcac180	0.053634968	0.05751977	0.352738499
pcaac321.pcaec301	-0.06975196	0.074811133	0.352783482
pcaec405.lysopcac182	-0.031839378	0.034152822	0.352841184
pcaec383.methionine	-0.032740251	0.035120144	0.35285472
pcaac366.c18	-0.039279994	0.042138892	0.352896075
pcaac240.c181	0.015749978	0.016896695	0.352907236
creatinine.pcaec300	0.011666905	0.012521041	0.353087308
pcaec403.creatinine	0.010432979	0.011198353	0.353154795
pcaac281.lysopcac281	-0.02544367	0.027317246	0.353277819
pcaec446.pcaec422	-0.024908061	0.026743776	0.353306146
pcaac403.smc260	-0.038521552	0.041364276	0.35334907
pcaac385.pcaec401	0.025718468	0.027616568	0.353352351
pcaec362.pcaac421	0.021347891	0.022923833	0.353360789
pcaec421.pcaec363	0.026255301	0.028196433	0.353410418
pcaec385.pcaac425	-0.066723928	0.071677712	0.353548926
pcaec380.methionine	-0.033748483	0.036257006	0.353587765
smc161.c101	-0.007847037	0.008431534	0.353657587
glutamicacid.smc161	0.069200438	0.074361928	0.353702798
pcaec384.glutamine	-0.009684057	0.010408585	0.353805055
pcaec361.c101	-0.027347107	0.029395048	0.353836465
pcaec342.phenylalanine	-0.034099897	0.036653617	0.353837553
acetylornithoine.pcaec406	-0.014219028	0.015284589	0.353859739
pcaec340.glutamine	-0.006639622	0.007137621	0.353888803
pcaac281.pcaec341	0.098437089	0.105839267	0.353974873
h1.pcaec383	-0.076739506	0.082519997	0.354032476
smohc141.c2	-0.052118123	0.056047395	0.354061707
pcaec361.pcaec383	-0.081882482	0.088077893	0.354182402
pcaec321.lysopcac280	0.007512784	0.008081787	0.354216185

histidine.pcaac321	0.026902898	0.028943974	0.354274277
pcaec421.pcaac425	-0.141154396	0.151900064	0.354389665
pcaec360.lysopcac170	0.016910186	0.018198631	0.354419268
smohc161.pcaec422	-0.01907386	0.020528106	0.354441296
pcaec425.pcaac425	-0.073639657	0.07926354	0.354498092
lysopcac182.creatinine	0.004803181	0.005170981	0.354588989
pcaec343.pcaac403	-0.003048956	0.003282442	0.354591085
pcaac381.pcaec405	0.075559166	0.081373413	0.354756185
lysopcac182.pcaec405	-0.030046523	0.032363137	0.35482358
kynurenine.lysopcac280	-0.00718656	0.007741688	0.354887889
pcaec422.leucine	-0.028475712	0.030689497	0.355109396
smohc141.c14	-0.02338034	0.025198694	0.355122688
pcaec301.lysopcac170	0.007813353	0.008421545	0.355152666
smc240.pcaec360	-0.009141466	0.009853392	0.355169824
pcaac360.pcaac385	-0.012644191	0.013629917	0.355205438
c141.pcaec301	-0.045623136	0.049188634	0.355291007
leucine.smc160	-0.041728775	0.044992573	0.355319241
pcaec340.c12	0.040122034	0.0432662	0.355386181
c3.lysopcac160	0.0077444	0.008351395	0.355392161
pcaec382.pcaec424	-0.047084141	0.050778461	0.355429326
glycine.pcaac386	-0.019999423	0.021571486	0.355493069
pcaac404.pcaac402	-0.049338507	0.053217324	0.355497952
pcaac240.leucine	-0.009168149	0.009890186	0.355559501
putrescine.pcaac424	-0.020337777	0.021939678	0.355563936
c2.lysopcac161	-0.055575325	0.059954669	0.355579632
pcaec340.pcaac385	-0.021293804	0.022971819	0.355580876
pcaec403.lysopcac170	-0.014892696	0.016068008	0.355632308
pcaac426.pcaac403	-0.016397221	0.017691481	0.355638077
c162.pcaec421	-0.001497498	0.001615763	0.355657548
c0.pcaec380	0.042033778	0.045353695	0.355660523
putrescine.pcaac344	0.055115649	0.059470797	0.355676626
pcaac322.leucine	0.005304429	0.005724136	0.355723487
asparticacid.pcaac342	0.048155505	0.051968122	0.355745361
smc202.lysopcac140	-0.000971634	0.00104878	0.355845438
phenylalanine.smc181	-0.016229226	0.017522222	0.355966507
lysopcac170.pcaec361	0.004637272	0.005006878	0.355980892
alphaaminoadipicacid.pcaac421	0.006689615	0.007222882	0.355986409
lysopcac140.pcaac281	-0.000781544	0.000843921	0.356029448
c181.pcaac422	0.022522676	0.02432423	0.356107539
smohc221.pcaec424	-0.01649908	0.01781974	0.356132424
c10.spermidine	0.047678592	0.051499764	0.356176774
spermidine.lysopcac161	-0.137386039	0.148411921	0.356225756
pcaec423.pcaec340	-0.048207075	0.052077445	0.356239785
pcaac384.kynurenine	-0.004560252	0.004926649	0.356266125
pcaac343.arginine	0.037287679	0.040314057	0.356628682

pcaec322.pcaec321	0.066973051	0.072409707	0.356634805
asymmetricdimethylarginine.smc241	0.025143786	0.027186821	0.356669071
pcaac320.smohc221	0.047835974	0.051733871	0.356771296
pcaac380.pcaac281	0.001200382	0.001298417	0.35685338
pcaac420.h1	0.007489148	0.008101036	0.356868259
pcaac425.pcaac300	-0.038418752	0.041560949	0.356905785
lysopcac170.smc181	0.01231146	0.013319085	0.356930799
c101.pcaec383	0.03691866	0.039941272	0.356943062
pcaec342.c18	-0.021027629	0.022749813	0.356955809
h1.valine	-0.053354726	0.057733635	0.35703141
lysopcac140.pcaac426	-0.034300757	0.0371197	0.357080799
pcaec300.creatinine	0.004271626	0.004623123	0.357126556
pcaac385.pcaec365	-0.02767693	0.029956403	0.357159168
pcaec401.pcaec365	-0.022774999	0.024653474	0.357212148
asparagine.pcaec401	0.014975415	0.01621246	0.357267638
pcaac406.pcaec444	0.113586875	0.122974662	0.357286897
pcaac240.pcaac384	0.02646868	0.028657844	0.357313109
alanine.lysopcac203	0.017046727	0.018462624	0.357468912
glutamine.lysopcac180	0.04855128	0.052585821	0.357486093
smc180.smohc221	0.067472906	0.073088742	0.357544693
pcaac385.pcaac384	0.079662187	0.086298148	0.357575791
c8.pcaec365	-0.020200739	0.021888523	0.357686095
pcaac240.pcaec382	-0.013671213	0.014813905	0.35770075
histidine.pcaac323	0.084262913	0.091308683	0.357715236
serotonin.c18	0.049903424	0.05408026	0.35775145
lysopcac203.pcaac403	-0.005643905	0.006116445	0.35776355
c4.c141	-0.024867189	0.026953391	0.357837845
phenylalanine.pcaac381	0.01490381	0.016155088	0.357865771
isoleucine.pcaec361	0.005184674	0.005620026	0.35787108
c182.pcaac344	-0.023960782	0.025975042	0.357913481
pcaec385.glutamine	-0.009774276	0.010596027	0.357917009
creatinine.lysopcac280	-0.004862674	0.005271592	0.357926009
pcaac383.c102	0.02042102	0.022138659	0.357934017
pcaec443.lysopcac170	-0.010330942	0.011202498	0.358045563
pcaec424.c51	-0.08773611	0.095143242	0.358072716
pcaac300.pcaec446	-0.03437141	0.03727988	0.358158321
pcaac422.asparagine	-0.007760722	0.008418314	0.358208882
ornithine.pcaac361	-0.065262445	0.070795616	0.358231
lysopcac140.lysopcac182	-0.012505355	0.013565628	0.35823193
pcaec342.pcaec405	-0.040867078	0.0443326	0.35823827
putrescine.glycine	0.018084599	0.019618468	0.358245551
pcaec425.spermidine	-0.047130703	0.051131125	0.358273377
pcaac406.pcaac344	-0.041569796	0.045099287	0.358284799
pcaac323.c182	0.036911993	0.040048207	0.358311055
pcaec384.pcaac281	-0.001504964	0.001632848	0.358315479

pcaec404.isoleucine	-0.009079413	0.009850985	0.358318032
citruline.pcaec424	0.002609624	0.002832485	0.35850318
pcaac321.creatinine	0.008051888	0.008740031	0.358531377
pcaac380.pcaec365	-0.019201988	0.020849622	0.358682205
pcaec360.pcaac343	-0.031522133	0.034231107	0.358741067
pcaac321.asparagine	0.006077981	0.00660057	0.35875957
pcaac420.pcaac404	0.004598655	0.004994439	0.358796815
c181.pcaac383	0.01309629	0.014223944	0.358814313
pcaec301.pcaec383	-0.05639659	0.061254337	0.358827835
lysopcac281.methionine	-0.028881447	0.031374748	0.358913106
pcaec364.pcaec380	0.037641355	0.040892712	0.358934472
pcaac364.lysopcac281	0.012374646	0.013444748	0.358977757
serine.c51dc	-0.086410942	0.093898375	0.359054349
pcaac421.pcaac385	-0.044478037	0.048336763	0.359101351
serotonin.pcaac403	-0.002579757	0.002803673	0.359119603
c162.pcaac321	0.011971145	0.013013027	0.359223415
pcaec361.putrescine	0.034833726	0.037865588	0.359225808
pcaec361.pcaec380	0.046150383	0.050168062	0.359233781
pcaec402.pcaac381	0.024058213	0.026160087	0.359370333
pcaac426.lysopcac204	-0.020896189	0.022724802	0.359433432
pcaec342.pcaec385	-0.048433328	0.052674294	0.359457028
smc181.c18	-0.02141476	0.023309595	0.359861899
pcaec425.glutamine	-0.009144713	0.009954449	0.359890164
smohc221.pcaac420	0.033409657	0.036368973	0.359903246
lysopcac170.glutamine	-0.010695989	0.011644588	0.359951938
pcaac322.glutamine	-0.013662916	0.014875476	0.359978712
lysopcac161.glutamine	-0.007951558	0.008657807	0.360009844
pcaec322.lysopcac280	0.007330161	0.007983501	0.360146834
pcaec381.pcaac426	0.066943557	0.072912043	0.360158491
smc202.lysopcac182	-0.023702619	0.025816996	0.360179319
pcaec444.pcaec402	-0.03027444	0.032980363	0.360256436
smohc141.c101	-0.009171973	0.009993231	0.360326815
smohc221.c5	-0.005143544	0.005604574	0.360367582
leucine.lysopcac180	-0.039387537	0.042927113	0.36046984
methioninesulfoxide.ornithine	0.05589913	0.0609256	0.360493998
c142.smc161	-0.039892503	0.04348412	0.360543202
pcaec364.histidine	0.045993568	0.050137046	0.360567731
pcaac422.acetylornithoine	-0.031118397	0.033925953	0.360626392
lysopcac281.pcaac421	0.012031539	0.01311712	0.360629124
pcaec402.pcaec404	0.017723807	0.0193234	0.360639253
methioninesulfoxide.lysopcac182	-0.038717233	0.042213653	0.360663627
alanine.pcaec424	-0.013509621	0.014734049	0.360807312
smohc161.arginine	-0.017623354	0.019222969	0.360865692
c142.c5	-0.004489597	0.004899893	0.361138334
c182.asparticacid	0.011289212	0.012321215	0.361150116

c4.pcaec301	0.071476742	0.078014285	0.361171574
serine.c102	0.021950495	0.02395978	0.361203687
pcaec421.histidine	0.090210237	0.098475691	0.361241933
h1.pcaec341	0.049168348	0.053683354	0.361331012
pcaec342.pcaac366	-0.01768703	0.019314951	0.361424351
pcaac425.histidine	0.068885905	0.075229056	0.361442576
pcaec341.lysopcac161	0.04750744	0.051882087	0.361443204
pcaac422.pcaec343	0.051736168	0.056512164	0.361544458
pcaac362.pcaec301	0.057089526	0.062360842	0.361553116
pcaac320.pcaac321	-0.021976008	0.024007601	0.361602073
pcaac425.isoleucine	-0.023263053	0.02541683	0.361662375
lysopcac180.smc260	-0.028240944	0.030855752	0.361664857
c10.valine	-0.058968756	0.064430928	0.361681942
c141oh.pcaac422	0.024337593	0.026594982	0.361736676
acetylornithoine.pcaec365	-0.01385627	0.015143859	0.361811766
pcaec382.smc261	0.057644146	0.063000818	0.361812605
pcaac366.pcaec402	0.030528718	0.03336773	0.361842491
glutamicacid.smc240	0.036888149	0.040322615	0.361890688
pcaec360.asparticacid	0.063545266	0.069465774	0.361919175
methioninesulfoxide.pcaac421	0.026901463	0.029409587	0.361947112
h1.pcaac363	-0.012326353	0.013476603	0.361983269
isoleucine.pcaac322	0.040473458	0.044251199	0.361993066
pcaec406.pcaac321	-0.021348061	0.023340809	0.361996069
pcaac386.asparticacid	-0.041585193	0.045467504	0.362001506
smc260.smc181	-0.010623479	0.011615767	0.362022178
pcaac365.smc160	-0.058340883	0.063794454	0.362053891
putrescine.c5	0.00863357	0.009440767	0.362061506
c142.smc202	-0.036164732	0.039549463	0.36210394
smohc161.c0	-0.000557491	0.000609682	0.36211543
pcaac404.pcaec342	0.077031239	0.084248534	0.362148157
pcaec406.lysopcac170	0.019558579	0.021392472	0.362179252
pcaec403.pcaac405	-0.040963119	0.044814622	0.362292782
putrescine.smc161	-0.05382959	0.058904345	0.362402469
pcaac384.pcaec401	0.019656607	0.02151195	0.362452025
pcaec384.c142	0.036716205	0.040184828	0.362488487
ornithine.pcaec422	-0.021292912	0.023305232	0.362503945
pcaac320.c162	-0.059523322	0.065156541	0.36256179
kynurenine.pcaec424	-0.006356787	0.006959858	0.362662991
pcaac360.pcaec445	0.040494859	0.044342803	0.362729683
pcaac344.pcaac403	-0.009796987	0.010731301	0.362880166
pcaac425.smc240	0.019922872	0.021822969	0.362882427
smc241.asymmetricdimethylarginine	0.03150522	0.034510892	0.362895408
smohc222.pcaec322	-0.098825419	0.108261356	0.362929699
pcaec342.pcaac344	0.082112954	0.089965806	0.3629969
smc181.lysopcac182	-0.018234879	0.01998727	0.363200477

pcaac365.alanine	-0.016944374	0.01857572	0.363277069
c51dc.pcaac323	-0.097600364	0.107006854	0.363321156
pcaec383.smc261	0.063597237	0.069728307	0.363332959
smc240.serine	-0.02288312	0.025091562	0.363378768
c182.serotonin	0.056089151	0.061513431	0.363465368
smohc221.asymmetricdimethylargin	0.039598494	0.043441098	0.363609694
smohc141.isoleucine	0.022687555	0.024890633	0.363638472
pcaec363.transhydroxyproline	0.015981437	0.017535396	0.363695191
alphaaminoadipicacid.pcaec383	0.032378986	0.03552794	0.36370294
smc160.c101	-0.006333586	0.006950454	0.363765394
smohc161.pcaac404	0.004424128	0.004855183	0.363781253
pcaac323.arginine	-0.022989985	0.025236258	0.363900602
smc180.lysopcac182	-0.021813082	0.023944911	0.363911544
pcaac343.pcaac421	0.019125076	0.020995664	0.363944838
pcaac240.pcaac403	-0.012156032	0.013347502	0.364034837
spermidine.lysopcac140	0.005334408	0.005857635	0.364065576
alphaaminoadipicacid.lysopcac140	0.00077209	0.000848008	0.364171575
smohc222.histidine	0.049730411	0.054633613	0.364287799
lysopcac280.pcaac320	-0.075351722	0.082782794	0.364297738
smc181.pcaac322	0.044878173	0.049314416	0.364398846
lysopcac280.c4	-0.009387954	0.010317654	0.364477345
pcaac365.pcaac402	0.058689238	0.064512697	0.36456181
c162.lysopcac280	-0.011846332	0.013022332	0.364581786
c162.c101	-0.023502955	0.025838483	0.364625474
pcaac364.lysopcac140	0.00116462	0.001280557	0.364702794
acetylornithoine.spermidine	0.046546972	0.051187078	0.364762385
c51dc.creatinine	0.008326704	0.009156918	0.364770368
acetylornithoine.smc261	0.019579376	0.021532004	0.364780748
pcaec382.isoleucine	-0.014921756	0.016410092	0.364786817
c51dc.c141	0.019484373	0.021428272	0.364797398
c3.pcaec402	-0.041106678	0.045217197	0.364897077
lysopcac161.pcaec421	-0.001742433	0.001917158	0.365018909
pcaac424.creatinine	0.013581775	0.01494646	0.365106848
c2.pcaec361	-0.005573945	0.006134918	0.365177564
pcaec380.isoleucine	-0.018885426	0.020787926	0.365219747
pcaec443.pcaac366	-0.054421361	0.059906124	0.365238957
pcaec443.prolinr	-0.017016069	0.018736078	0.365368425
pcaac344.histidine	0.043571695	0.047982647	0.365434809
pcaac363.lysopcac204	-0.011930031	0.013138929	0.365477328
glycine.pcaec360	-0.005958731	0.00656283	0.365498237
alphaaminoadipicacid.pcaec446	-0.038529535	0.042453878	0.365703183
pcaec384.smc241	0.085226706	0.093924246	0.365789488
lysopcac161.smc240	0.023284909	0.025662286	0.365810165
pcaec363.creatinine	0.00414776	0.004571419	0.365828456
pcaec402.smc161	0.073443443	0.080954536	0.365884292

pcaec444.pcaec424	-0.007128364	0.007858083	0.365926761
smc160.pcaec404	-0.011006289	0.012133916	0.365963441
pcaac321.smc161	-0.026816679	0.029566346	0.365999282
pcaec420.prolinr	0.02477124	0.027312597	0.366024166
prolinr.pcaec420	-0.019488618	0.021492552	0.366125065
pcaac322.creatinine	0.007128404	0.007861677	0.366142591
smc261.pcaec383	0.056208201	0.061990674	0.36614676
pcaac386.ornithine	-0.013642123	0.015045896	0.366157137
c181.pcaac240	-0.014335794	0.015814547	0.366265983
pcaec405.pcaec403	-0.026452502	0.029190832	0.366425186
pcaac383.pcaac422	-0.022289175	0.02460338	0.366558382
glutamicacid.pcaec362	0.021591083	0.023835923	0.366620836
creatinine.pcaac323	-0.062726488	0.069262538	0.366719734
pcaec382.lysopcac180	-0.0735003	0.08116964	0.366782527
pcaec421.c14	0.064823133	0.071607144	0.366916552
smohc161.spermidine	0.046778514	0.051674948	0.366924566
pcaec401.lysine	0.062756222	0.069329494	0.366954947
c101.pcaec360	0.004854882	0.005363952	0.367004405
pcaec444.pcaec385	-0.036821784	0.040691216	0.367103006
lysopcac281.c51	-0.059111455	0.065327791	0.367136537
pcaac404.pcaec401	0.019899235	0.021994484	0.367192721
c16.pcaec383	0.05944173	0.0657008	0.367194723
pcaec403.serine	0.074908562	0.08280835	0.367264524
c8.pcaac381	-0.015314105	0.016929638	0.367279244
pcaac342.smc181	0.009706467	0.010730892	0.367299705
c4.pcaec401	0.037073987	0.040989617	0.367332596
smc260.pcaec321	-0.03246172	0.03589075	0.367339694
pcaec340.tyrosine	0.036313164	0.040150613	0.367358473
lysopcac181.pcaac343	0.008796412	0.00972709	0.367412683
pcaec321.smc160	0.066390302	0.07341559	0.36741958
glycine.c5	0.009171244	0.010143618	0.367508651
pcaac403.pcaac300	-0.047734148	0.052801084	0.367562574
lysopcac181.pcaec445	0.040750187	0.045075992	0.367564802
pcaec322.pcaac344	-0.068024015	0.075245277	0.367566225
c162.pcaac322	-0.034830804	0.038529644	0.367582187
pcaac344.smc160	0.043867497	0.048538614	0.367706481
pcaac362.pcaac344	-0.036822844	0.040760038	0.367896438
pcaec342.pcaac322	0.044509989	0.04926981	0.367903201
pcaac281.creatinine	0.003794878	0.004202042	0.36805629
pcaec380.pcaac322	0.057730965	0.06393748	0.368148748
c8.smohc241	0.064958872	0.071946677	0.368176853
c3.c141	-0.035437774	0.039258095	0.36827639
pcaec322.pcaec365	0.029319738	0.032483888	0.368326014
alphaaminoadipicacid.pcaec405	0.024762149	0.027434628	0.368329108
pcaac420.pcaac403	-0.004823037	0.005344371	0.368400937

lysopcac160.pcaec361	0.00435814	0.004830164	0.368494021
c0.pcaec301	0.052770267	0.058508752	0.368681797
pcaec381.creatinine	0.009938015	0.011018908	0.36868992
pcaec361.tryptophan	0.07021688	0.077854713	0.368694812
pcaec342.pcaec382	-0.012122807	0.013443182	0.368755812
smohc241.lysopcac170	0.011648511	0.012917368	0.368761055
histidine.lysine	0.082045568	0.090986144	0.368779189
c10.pcaac421	0.038413768	0.042611136	0.368906784
h1.smc161	0.066775279	0.074072015	0.36890925
pcaec341.pcaec343	0.050052751	0.055523421	0.368920043
lysopcac181.pcaec340	0.033260446	0.036898211	0.368951895
pcaec423.pcaac362	-0.009350608	0.010373634	0.368967086
histidine.pcaac384	-0.055947657	0.062076051	0.369023265
lysopcac203.asparagine	0.00900923	0.009997989	0.369114294
smc241.pcaec424	-0.00246253	0.002732993	0.369149356
pcaac362.acetylornithoine	0.022536409	0.025013861	0.369192359
pcaec340.c51dc	-0.09160083	0.101671254	0.369195387
pcaec361.lysopcac203	0.018999578	0.021089135	0.369213009
pcaac385.c162	-0.048328025	0.053651561	0.369288307
c182.histidine	0.056415679	0.062630286	0.369289692
acetylornithoine.pcaec361	-0.011846708	0.013154833	0.369403012
pcaac383.pcaec401	0.032932845	0.036571652	0.3694334
leucine.alanine	0.0095581	0.010614862	0.369463554
histidine.pcaec401	0.018754496	0.02083195	0.369553401
lysopcac203.pcaac405	0.02015323	0.022386342	0.369568724
pcaac426.pcaac402	0.057293446	0.063649844	0.369627988
c3.pcaac422	0.046178196	0.051306822	0.369678272
c182.spermidine	0.050979932	0.056642844	0.369686667
pcaec446.asparticacid	0.01809316	0.020104812	0.369730394
c162.c3	0.047746622	0.053056955	0.369745863
pcaec380.pcaec365	-0.028215456	0.031357768	0.369810046
pcaec343.c162	0.063206119	0.070248556	0.369832243
pcaec381.pcaac343	0.008320567	0.009248486	0.369875576
pcaec381.lysopcac180	-0.061666418	0.068543995	0.369878858
asparticacid.pcaac360	0.055543369	0.061756483	0.370021302
c4.pcaec380	0.034730834	0.038618216	0.370050585
asparticacid.leucine	-0.016683448	0.018551961	0.370080287
pcaec421.pcaac344	0.073232285	0.081442163	0.370127202
smohc222.smc160	-0.154659766	0.172016041	0.370176512
smc241.pcaac402	-0.033914951	0.037721166	0.370179053
creatinine.c8	-0.050732899	0.056451467	0.370389595
c2.pcaec445	-0.054860703	0.061045446	0.370396566
pcaac405.pcaac420	-0.038224303	0.042534389	0.370406142
smohc241.pcaec340	0.054836699	0.061027016	0.37046131
lysopcac182.c5	0.00625271	0.006958627	0.370466191

pcaac404.pcaac364	0.046637769	0.051904447	0.370478849
pcaac362.asparticacid	0.01124887	0.012521408	0.370563947
pcaac366.leucine	-0.009404577	0.010469276	0.370600316
asparagine.leucine	0.005124597	0.005704887	0.370611292
smohc161.pcaec343	0.036342227	0.040469113	0.370748373
lysopcac140.phenylalanine	-0.025774934	0.0287057	0.370812555
pcaac344.pcaac364	0.068031893	0.075780619	0.37089494
leucine.pcaac381	0.018871562	0.021021423	0.37090447
lysopcac180.smohc241	0.044628776	0.04971476	0.370922148
pcaec423.c16	0.019060096	0.021234403	0.370971142
pcaac300.pcaac425	-0.073074522	0.081419519	0.371023394
smc161.pcaec444	0.074125936	0.082591544	0.371026524
c51.pcaac425	-0.058969603	0.065704444	0.371027776
smohc241.pcaac403	-0.014664454	0.016344082	0.37116848
c10.lysopcac203	-0.006403182	0.007137807	0.371250092
histidine.alanine	0.02845809	0.031726923	0.371308563
prolinr.alanine	0.015143508	0.016884557	0.371353542
lysopcac140.glutamine	-0.009861513	0.01099607	0.371387333
pcaac341.lysopcac170	0.009485776	0.010580692	0.371549015
kynurenine.smc202	0.049247241	0.054938414	0.371607263
lysopcac161.pcaac361	-0.093793031	0.104642321	0.371654014
pcaec364.pcaec405	-0.037386518	0.041725704	0.371820748
pcaec380.glutamine	-0.008652776	0.009663228	0.372126102
c5.pcaec341	0.05997693	0.066981491	0.372130273
serotonin.asparticacid	0.035235093	0.039353198	0.372167625
pcaac381.pcaec383	0.083519021	0.093280927	0.37217076
spermidine.c16	0.084855389	0.094786627	0.372236831
pcaec403.pcaec385	-0.055692506	0.062211753	0.372245645
leucine.pcaec342	-0.066848766	0.074680577	0.372288014
pcaac323.lysopcac140	-0.000906423	0.001012684	0.372319603
isoleucine.prolinr	-0.034737177	0.038810663	0.372334414
pcaac322.smc181	0.011328836	0.012658063	0.372362312
pcaec406.pcaac406	0.042997508	0.048051143	0.372448461
glutamicacid.lysopcac140	0.001273027	0.001422868	0.372521645
asparticacid.pcaec363	-0.02570198	0.028727253	0.372522095
pcaac240.c51	-0.088853307	0.09932494	0.372584874
pcaac323.pcaec322	-0.059122717	0.066096081	0.372625059
pcaec385.pcaac380	0.053629098	0.059955431	0.372632417
c14.pcaec300	0.041209948	0.046072999	0.372650397
pcaac424.lysopcac161	0.044210746	0.04943287	0.37269821
pcaec403.pcaac426	0.061275117	0.068528976	0.372810282
smohc221.pcaac405	-0.023652038	0.02645811	0.372920201
pcaec401.creatinine	0.019144212	0.021416942	0.37295282
c16.pcaac343	0.025087544	0.028065889	0.372953579
smc240.pcaec342	-0.072592185	0.081212163	0.37296516

c8.valine	-0.054410526	0.060890126	0.373110823
lysopcac281.creatinine	0.004502789	0.005039348	0.373142397
glycine.pcaac421	-0.010758371	0.012041257	0.373178157
pcaac343.creatinine	0.004504548	0.00504196	0.373203252
alanine.pcaec402	0.039089755	0.043755049	0.373221965
pcaec363.lysopcac203	-0.02095317	0.023454865	0.37324169
pcaec382.glutamine	-0.011158208	0.012490726	0.373252684
ornithine.pcaec402	-0.02330089	0.026086704	0.373311375
pcaac403.pcaec301	0.057968952	0.064899669	0.373311729
smc181.lysopcac140	-0.001013025	0.001134164	0.373321108
pcaac402.pcaec384	-0.044323485	0.049624845	0.373331734
pcaec422.c101	-0.029757414	0.033316927	0.373336651
pcaac342.pcaac321	-0.024191702	0.027086173	0.373349246
pcaec444.smc161	-0.043035917	0.048202622	0.373523016
c8.creatinine	-0.007660548	0.00858159	0.373597985
pcaec363.c101	-0.006107197	0.006842348	0.373658649
pcaac424.prolinr	-0.017163593	0.0192304	0.373677168
pcaac401.c16	0.012683671	0.014211347	0.373688349
lysopcac140.pcaec363	0.014034212	0.015727287	0.373771186
pcaac362.c182	0.03843748	0.043076396	0.373791646
pcaec406.pcaec405	-0.056497155	0.063318401	0.373812382
methionine.c141oh	-0.077081822	0.086401905	0.373886964
c182.smc202	0.038562682	0.043228119	0.373917419
smc260.kynurenine	-0.002870308	0.003217771	0.373947643
pcaac426.asymmetricdimethylarginir	-0.014830598	0.016627311	0.373987904
pcaac384.pcaec383	-0.0430266	0.048242439	0.374019574
c10.pcaac403	-0.002801482	0.003141218	0.374039309
smohc161.isoleucine	0.017062914	0.019132354	0.374044785
pcaac341.glutamine	-0.005419674	0.006078875	0.374192663
pcaec420.spermidine	0.04587054	0.051453119	0.374223181
h1.pcaec382	0.007095413	0.007959191	0.374237883
creatinine.c14	-0.030884292	0.034644709	0.374246677
c181.smc181	-0.02089902	0.023443972	0.374253259
pcaac405.lysopcac161	-0.06054901	0.067930318	0.37430956
pcaec384.pcaac405	-0.034658326	0.038886318	0.374345356
methionine.c16	-0.019645719	0.022047095	0.374448676
methioninesulfoxide.pcaec301	0.042057014	0.047198848	0.374459115
pcaac425.asymmetricdimethylarginir	-0.019190814	0.021542166	0.374572069
pcaac383.smc161	-0.034610546	0.038855299	0.374622349
pcaec384.pcaac361	0.027451127	0.030820423	0.374662499
c142.smohc241	0.046678205	0.052411917	0.374703593
pcaec365.pcaac322	0.041347306	0.046432255	0.374765738
smc160.smohc241	-0.071968597	0.080821763	0.374779689
lysopcac140.pcaac385	0.013230875	0.014860562	0.374846981
pcaac240.pcaec383	-0.066871305	0.075110899	0.374865082

smohc221.creatinine	0.005031005	0.005650952	0.374869175
pcaec383.pcaac385	-0.02181166	0.024507211	0.37502081
pcaac364.pcaac402	0.040135984	0.045097493	0.375035393
smohc141.valine	0.060368213	0.067832116	0.37504478
pcaec301.c16	0.018576444	0.020873633	0.375054001
lysopcac182.pcaac321	-0.017309342	0.019452525	0.375119758
pcaec382.creatinine	0.007741737	0.008700382	0.375124707
c141.lysopcac160	0.024455904	0.027485127	0.375140168
pcaec405.tyrosine	-0.055603034	0.062496636	0.375188529
lysopcac280.threonine	-0.020979388	0.02358409	0.375263247
smc202.pcaec340	-0.015967321	0.017951152	0.375300497
c51dc.pcaec343	0.040875016	0.045956142	0.375328288
c181.pcaac343	0.018142388	0.020401738	0.375423766
smohc221.pcaec423	0.018088462	0.020347218	0.375567012
pcaec383.lysopcac180	-0.054765423	0.061615854	0.375657598
pcaac360.smc261	0.027161245	0.030559262	0.375665448
glutamicacid.pcaac300	-0.059000431	0.066384018	0.375682071
c181.pcaac380	0.043376807	0.048816429	0.375791772
smohc241.pcaac405	-0.023062742	0.025960768	0.375899376
c141.pcaec422	0.017840787	0.020087481	0.376014327
lysopcac203.c141oh	0.102916767	0.115895657	0.376090544
pcaac403.pcaac426	0.081984889	0.092324784	0.376094334
pcaec380.lysopcac281	0.014845776	0.016718978	0.376118827
c4.pcaac422	0.027593259	0.031076227	0.376139044
pcaec341.c101	-0.004079936	0.004597674	0.376423483
arginine.asparticacid	-0.020255086	0.022829938	0.376517494
c182.pcaec342	-0.044902768	0.050615581	0.376561847
c51dc.lysopcac140	-0.000693513	0.00078181	0.376600474
c141oh.c4	-0.012613722	0.014221342	0.376656323
pcaec402.lysopcac170	0.042808278	0.04826596	0.376673684
pcaec381.pcaec405	0.098288079	0.11082302	0.376691164
c141.c3	0.045329502	0.051112426	0.376709145
smc241.lysopcac203	-0.008424112	0.009499287	0.376732535
pcaec421.smc261	0.05558226	0.06267755	0.376742306
lysopcac160.tyrosine	-0.041060458	0.046309275	0.376817221
c162.kynurenine	-0.004260662	0.004805919	0.376877676
pcaec363.pcaac240	-0.010002236	0.011287093	0.377081027
pcaac380.pcaec340	-0.032730181	0.036934726	0.377082653
pcaac403.pcaac422	-0.063470433	0.071639022	0.377183118
pcaec301.pcaec384	0.028421913	0.032081313	0.377205738
asparticacid.pcaec383	0.078770473	0.088940869	0.377358077
serine.putrescine	-0.014998765	0.016937847	0.377429061
pcaec406.valine	-0.058438775	0.066005871	0.377515312
pcaac403.c51	-0.09246292	0.104446591	0.37756482
pcaac240.smc240	0.04331462	0.048929048	0.37757094

smc260.pcaec342	-0.055522814	0.062727807	0.377632686
c142.pcaac300	-0.031426574	0.035510561	0.37771139
pcaec383.pcaec445	0.037332411	0.042188684	0.377765532
c142.pcaec445	-0.037999634	0.042949311	0.377838735
c10.pcaac240	-0.01350563	0.01526615	0.377880232
pcaec405.c51dc	-0.09825884	0.111101103	0.378024845
lysine.pcaec360	0.004411987	0.004989408	0.378099339
pcaac425.pcaec301	0.044234018	0.050026654	0.378132508
lysopcac140.taurine	-0.047786988	0.054058618	0.378253182
pcaec341.serotonin	0.06609577	0.074771688	0.378262207
lysopcac281.c5	-0.005664972	0.00640892	0.378288013
asparticacid.pcaac343	0.011267637	0.012748683	0.378337696
smohc161.kynurenine	-0.003512151	0.003974015	0.378363821
c51dc.glycine	-0.017623458	0.019944267	0.378441116
c2.pcaec380	-0.033840337	0.038297133	0.378446215
smohc241.pcaec422	-0.017295294	0.019573138	0.378447242
c14.c102	0.02045002	0.023144522	0.378471283
alphaaminoadipicacid.transhydroxyp	-0.020019913	0.022657834	0.378473181
pcaec360.glutamicacid	-0.027383737	0.030996907	0.378549192
pcaec301.glycine	0.023175955	0.026234438	0.378558472
pcaac424.pcaac323	-0.056465761	0.063929571	0.378648757
pcaec380.pcaec363	0.021165104	0.023963055	0.378654427
pcaec364.transhydroxyproline	-0.033688953	0.038142877	0.378658973
pcaac406.c182	0.021688602	0.024558113	0.378700028
c8.lysopcac181	0.05369141	0.060796923	0.378714646
pcaec405.isoleucine	-0.012440762	0.014089397	0.378789753
pcaac401.pcaac322	0.044668743	0.050599274	0.378893903
pcaec420.pcaac403	-0.006639144	0.007521798	0.378969504
c2.smc180	-0.004145728	0.004698297	0.379111908
c102.pcaec424	0.009398749	0.010651502	0.37911307
lysopcac180.valine	-0.03224567	0.036549416	0.37918776
methionine.pcaac406	0.045449354	0.051516181	0.379195316
c8.pcaec362	0.011537025	0.013079385	0.379280168
smc180.pcaac380	-0.056061555	0.063556779	0.379283731
creatinine.pcaec401	0.035998188	0.040814341	0.37932248
c4.pcaec445	0.062756286	0.071166724	0.379418339
arginine.pcaac405	-0.037413949	0.042432989	0.379473419
kynurenine.pcaac281	0.000862866	0.000978652	0.379490138
c182.smc181	-0.017643531	0.020014754	0.379577032
pcaac323.lysine	-0.045119664	0.051187325	0.379611851
c141.c101	-0.011532019	0.013084652	0.379677762
pcaac420.pcaac422	-0.037660466	0.042732656	0.379696781
c3.pcaec384	0.037081233	0.042078963	0.379736887
smc240.pcaec340	0.022728877	0.025794739	0.379783186
citruline.pcaec401	0.021166755	0.024023875	0.37982214

smc181.pcaac281	0.001256707	0.001426486	0.379870839
pcaec340.ornithine	-0.028253803	0.032071242	0.379876937
pcaec343.pcaec385	-0.042637329	0.048413994	0.380032349
pcaac360.c18	-0.034770707	0.039481869	0.380035927
pcaec341.pcaac420	0.055121369	0.062595992	0.38008225
pcaac240.pcaac406	0.038360413	0.043564847	0.380111108
serine.pcaac343	0.010664512	0.012112356	0.380149083
c181.c14	-0.028611702	0.03249642	0.380153675
c182.tyrosine	-0.037797012	0.042930073	0.380167167
methionine.pcaac366	0.027086526	0.030766764	0.380193809
kynurenine.citruline	0.03213179	0.036500728	0.380235491
smc241.lysine	0.040424064	0.045934566	0.380381009
pcaac421.lysopcac160	-0.013109748	0.014898077	0.380420646
c51.pcaec421	0.002468513	0.002805448	0.380454421
lysopcac160.pcaac403	0.017484007	0.019871233	0.380473115
smohc141.pcaac380	-0.038532061	0.04379719	0.380517029
alphaaminoadipicacid.leucine	-0.010314653	0.011726786	0.380626824
alanine.smc260	0.016936848	0.019257632	0.380677002
pcaec383.smohc221	-0.078914519	0.089736075	0.380720615
pcaec340.c102	0.020852062	0.023715826	0.380807098
smc240.c16	0.021057796	0.023951372	0.380837984
pcaec403.prolinr	-0.011553713	0.013141517	0.380845039
lysopcac281.pcaec322	0.058333904	0.066351759	0.380853201
lysopcac161.pcaac323	-0.100616572	0.11446765	0.380942714
pcaac341.asparticacid	0.037738925	0.042945251	0.381065591
pcaec360.pcaec340	-0.016835698	0.019158524	0.38107137
ornithine.taurine	-0.026390108	0.030037274	0.381168066
lysopcac170.c142	0.025357675	0.028862274	0.381170002
glycine.serotonin	-0.066950114	0.076205098	0.381182674
pcaac426.alphaaminoadipicacid	0.012075677	0.013745239	0.381191572
histidine.pcaec424	-0.038251526	0.043545686	0.381252285
pcaac426.pcaec401	0.023081172	0.026275829	0.381254687
pcaac403.glutamine	-0.014509659	0.016518451	0.381269476
pcaec404.c51dc	-0.098463894	0.112095978	0.381270497
pcaec443.pcaec405	0.088688856	0.100973452	0.381297969
ornithine.serine	-0.017040504	0.019403362	0.381359634
c51dc.c102	-0.025011849	0.028484153	0.381428466
lysine.kynurenine	-0.004000243	0.004555996	0.381471553
pcaec322.transhydroxyproline	-0.031906017	0.036344964	0.381553247
pcaac380.lysopcac160	0.040551569	0.046199852	0.381620232
pcaec300.pcaec446	-0.041354633	0.047118938	0.381662204
smc260.pcaac240	-0.006657181	0.007586765	0.38176594
pcaac360.lysine	-0.079452114	0.090559165	0.381832315
pcaac381.pcaec422	-0.02478111	0.028246828	0.38185635
arginine.pcaac322	0.033905603	0.038656391	0.381966629

c51.pcaec402	-0.014957143	0.01705372	0.381989209
pcaec360.c101	-0.009327893	0.010635992	0.38201538
pcaac425.smc261	0.0524189	0.059774506	0.382052163
pcaac402.pcaac380	0.051614319	0.058857482	0.382055868
citruline.c3	-0.021558062	0.024583809	0.382064584
lysopcac203.tyrosine	-0.044417549	0.050653258	0.382079162
pcaac366.pcaec361	-0.024267571	0.027675367	0.382094771
ornithine.pcaac424	0.019409531	0.022137493	0.382145377
pcaac380.asparticacid	-0.033905051	0.038678633	0.382247375
pcaec301.tyrosine	0.030695988	0.035017874	0.382248969
lysopcac181.lysopcac182	-0.040138254	0.045789591	0.382249091
pcaac405.smc202	0.020850864	0.023789359	0.382304187
pcaec384.pcaec422	-0.038054447	0.04342465	0.3823831
serotonin.pcaec401	0.022510285	0.025690882	0.382456431
h1.lysopcac170	-0.020522896	0.023424144	0.38248601
smohc241.pcaec382	-0.013923304	0.015891685	0.382488801
pcaac425.pcaac406	0.047134515	0.053800226	0.382507828
asymmetricdimethylarginine.pcaec36	-0.002248628	0.002567176	0.382609469
pcaac323.pcaec405	-0.044451885	0.050750627	0.382623839
pcaec386.pcaac240	-0.01458851	0.016657101	0.382664558
lysopcac281.pcaec362	0.010366955	0.011837892	0.382702431
pcaac426.c162	-0.042270669	0.048269102	0.382710009
pcaec363.pcaec382	-0.01731549	0.019773726	0.382735837
pcaec386.asparticacid	-0.030141055	0.03442205	0.382762649
pcaac363.creatinine	0.004010083	0.004580073	0.382807131
c51.smc240	-0.04190054	0.047856507	0.382809735
asparticacid.putrescine	-0.017393216	0.019868483	0.382879048
pcaac380.lysopcac161	-0.070845809	0.08093168	0.382900432
serine.pcaec362	0.013329585	0.015233112	0.383083565
pcaec382.pcaec401	-0.070837838	0.080956864	0.383101425
c3.c18	-0.027350986	0.031264584	0.38320114
pcaac421.lysopcac161	-0.058845828	0.067272971	0.383250584
smc181.ornithine	-0.054642687	0.062468444	0.383254637
pcaac321.c0	-0.000663411	0.000758489	0.383295603
pcaec301.lysopcac181	-0.023462955	0.026827731	0.383333934
c8.c101	0.017718811	0.020261735	0.383378505
citruline.c8	0.03310476	0.037857182	0.383395766
pcaac426.pcaac421	-0.017392269	0.019889696	0.383411131
c0.c14	-0.028960855	0.033126251	0.383508327
pcaec425.c14	-0.031859007	0.036442067	0.383519094
pcaac383.pcaec341	-0.038951868	0.04455771	0.383545089
pcaac384.glutamicacid	-0.029453707	0.033693324	0.383555272
kynurenine.pcaec420	-0.040389543	0.046210024	0.383624472
pcaec402.c51dc	-0.09981805	0.114208681	0.383649349
pcaac422.smc260	-0.043423381	0.049685336	0.383665245

pcaac426.pcaec422	0.017997531	0.020594881	0.383710851
c18.valine	-0.021727753	0.024867481	0.383787971
kynurenine.pcaec444	-0.091398655	0.104612842	0.383818787
pcaec381.pcaec445	0.051379041	0.058812693	0.383862328
glutamicacid.pcaac240	-0.011202321	0.01282348	0.383876301
pcaec382.pcaec362	0.011429176	0.01308518	0.38394935
lysopcac204.methionine	-0.03169515	0.036291631	0.384002762
pcaec401.lysopcac281	0.01653238	0.018931508	0.38404225
pcaac320.smc240	0.030882304	0.03536491	0.38405646
pcaec421.smc240	0.029525925	0.033812635	0.384070265
c8.pcaec341	0.037806532	0.043297501	0.384092631
c10.pcaec421	-0.00219445	0.002514046	0.384258005
pcaac384.pcaec342	-0.067872564	0.077759318	0.38426972
pcaec405.pcaec385	-0.05497079	0.06298308	0.384306537
glutamicacid.c51	-0.043900541	0.050306185	0.384371601
pcaac320.lysopcac281	0.008126534	0.009312415	0.384377606
lysopcac160.pcaac425	-0.070321435	0.080600777	0.384480761
pcaac321.smc260	-0.018189213	0.020848874	0.384499524
lysopcac280.smc161	0.067574064	0.077456917	0.384512048
acetylornithoine.lysopcac181	-0.042967093	0.049256667	0.384565409
pcaac386.c3	0.019790811	0.022688202	0.384573532
pcaec381.pcaac380	0.036492036	0.041837286	0.384605096
pcaec362.acetylornithoine	0.056720929	0.06504315	0.384706409
putrescine.pcaac426	-0.034265844	0.039300533	0.3847924
serotonin.valine	-0.043317638	0.049685715	0.384824888
c162.pcaac364	0.027282343	0.031295244	0.384857532
serine.pcaac426	-0.024685738	0.028328307	0.385051599
pcaec421.tyrosine	-0.055217185	0.063371479	0.385100791
pcaac424.phenylalanine	-0.051865963	0.059525428	0.385101334
lysopcac182.c51	-0.044709299	0.051312852	0.385110305
phenylalanine.pcaec420	-0.030887147	0.035449556	0.385115438
prolinr.tyrosine	-0.058317935	0.066950367	0.38524401
c3.pcaec382	0.010519699	0.012078808	0.385320341
h1.putrescine	0.020134567	0.023119064	0.385328172
pcaec422.pcaec383	-0.078828645	0.090513329	0.385328743
pcaec443.c16	0.013211002	0.015169579	0.385338887
pcaac385.ornithine	-0.016313938	0.018734818	0.385396604
pcaac366.pcaec421	0.003446264	0.003958491	0.385495305
c141.pcaac300	-0.021856512	0.025106476	0.385521235
pcaac405.ornithine	-0.010619779	0.01219968	0.385551855
pcaac323.serotonin	0.069943411	0.080352355	0.385572408
pcaec365.smohc241	-0.057063789	0.065562564	0.385619897
pcaac380.pcaac240	-0.012557549	0.014429418	0.385672922
smc181.pcaac406	0.019594041	0.022516537	0.385709657
pcaec425.c51	-0.064102466	0.073675704	0.385788155

pcaec365.c5	-0.005617462	0.006456821	0.385819911
pcaec425.pcaac240	-0.013617535	0.015653715	0.385863821
glycine.pcaac281	0.002082582	0.002394127	0.385892504
prolinr.pcaac385	0.004225262	0.004859099	0.386063608
pcaac425.c51dc	-0.090548897	0.10414188	0.386107429
c3.pcaac421	0.016865881	0.019397832	0.386109524
pcaac322.serotonin	0.055295771	0.06359879	0.386123443
c4.pcaec446	-0.05221138	0.060053665	0.386142437
pcaac381.pcaec403	-0.030879266	0.035517666	0.386145824
c8.lysopcac203	-0.006247746	0.007186365	0.38615509
serotonin.ornithine	-0.054554375	0.062760411	0.386231715
pcaac360.spermidine	0.045259823	0.052077988	0.386324578
pcaec420.asparagine	-0.003530948	0.004063629	0.386413356
smohc221.pcaac386	0.010551401	0.012145457	0.386501674
pcaec405.pcaac366	-0.019545228	0.022498218	0.386505644
lysopcac140.lysopcac170	-0.014367912	0.016539289	0.386522877
smc181.pcaec322	0.069022946	0.079459706	0.386555905
lysopcac280.pcaac380	0.022675916	0.026109785	0.386648585
smc161.smohc241	-0.051003171	0.058727096	0.38665173
c51dc.pcaec383	0.046368873	0.053394576	0.386683634
lysine.smohc141	-0.029599404	0.034090904	0.386776263
arginine.c51	-0.061574691	0.070922749	0.386806535
smc181.histidine	0.043677847	0.050315124	0.386865428
pcaac341.smc260	-0.017918364	0.020643964	0.386928005
pcaec300.pcaac405	0.025265445	0.02911545	0.387039021
pcaec445.pcaac323	-0.053382077	0.061538875	0.387210761
pcaac321.lysopcac203	0.013924573	0.016053606	0.387250698
pcaec383.pcaac300	-0.045056831	0.051949698	0.387285214
lysopcac160.pcaac343	0.022334008	0.025756554	0.387392792
pcaac405.pcaec401	0.018192876	0.020981034	0.387397641
c141oh.taurine	-0.02378013	0.027433447	0.38755092
pcaac406.lysopcac203	-0.010295468	0.011879436	0.387641984
lysopcac140.alanine	0.016993582	0.019608562	0.387654075
pcaec361.pcaac421	0.012364202	0.014267762	0.387685637
pcaec404.pcaec401	-0.05874125	0.067785744	0.387691538
pcaac363.pcaec301	0.057078249	0.065870767	0.387720846
pcaec380.c2	0.060776646	0.070140514	0.387731896
c141oh.transhydroxyproline	0.020909701	0.024131539	0.387737333
pcaac404.pcaac405	-0.087363231	0.100844952	0.387833529
pcaac322.pcaec300	0.007393918	0.008535063	0.387840717
pcaec322.pcaec363	-0.027481182	0.031723509	0.387855691
glycine.c182	0.04464275	0.051536018	0.387871066
lysopcac170.lysopcac181	0.066894222	0.077228417	0.38790224
spermidine.c4	-0.053961308	0.062299885	0.387919945
smohc241.pcaec424	-0.005243785	0.006054266	0.387932851

pcaac424.isoleucine	-0.012157225	0.014038422	0.388006081
pcaac383.asymmetricdimethylarginir	-0.021211881	0.02449556	0.388032642
pcaac381.pcaec380	0.064619371	0.074637808	0.388128473
c181.pcaec402	-0.015087019	0.017427293	0.388161537
pcaac386.serotonin	0.076652354	0.088562673	0.3882691
pcaac366.pcaac405	-0.044134389	0.051003264	0.388373285
pcaac344.glutamine	-0.007676588	0.008876607	0.388654277
pcaac343.c102	0.030859086	0.035685369	0.388685151
lysopcac160.pcaec444	0.124510067	0.144006943	0.388763394
asparticacid.pcaec421	-0.002773227	0.003207876	0.388821425
pcaac403.pcaec361	0.007336047	0.008485941	0.388827623
c5.pcaec446	-0.058801374	0.068020683	0.388844588
smc261.pcaec363	-0.019766763	0.022866074	0.388847359
pcaac386.pcaac365	0.050871184	0.058867413	0.389007511
asymmetricdimethylarginine.pcaac32	-0.020429087	0.023640626	0.389015021
isoleucine.c101	0.005201779	0.006019628	0.389023481
smc161.pcaec365	0.042179292	0.048811691	0.389030892
pcaac385.pcaac281	0.001387662	0.001606102	0.389101702
lysopcac181.c142	0.030944303	0.035817529	0.389129296
smc261.asparagine	-0.008145754	0.009429697	0.389185404
c12.pcaac240	-0.009715971	0.011247528	0.389190243
c3.pcaec301	0.039298901	0.045504721	0.389304751
lysopcac181.pcaac364	0.036761965	0.042567638	0.389309952
pcaec423.pcaac385	-0.015776914	0.018268533	0.389310821
smc260.smc180	-0.007682143	0.008896844	0.389389209
c14.pcaac405	0.035332632	0.040928192	0.389490422
lysopcac170.pcaec383	0.099592545	0.115365818	0.389494503
spermidine.c141oh	0.237526795	0.275184767	0.389561422
pcaec362.smohc161	-0.016196567	0.018766133	0.389604993
methionine.putrescine	0.025278563	0.029290924	0.389636453
pcaec300.c101	-0.006603175	0.007652321	0.38970139
pcaac360.pcaec361	-0.009805568	0.011365001	0.389762727
pcaac365.pcaec360	0.003943267	0.004571585	0.389886665
smc240.pcaec444	-0.093678604	0.108638095	0.39002947
smohc221.pcaec361	-0.012358304	0.014334411	0.390115735
c102.pcaac384	0.032719215	0.037955908	0.390176172
pcaec380.pcaec401	-0.042760721	0.049606658	0.390196234
pcaac384.smc241	0.043918263	0.050961431	0.390306725
pcaac402.pcaec404	0.03512461	0.040763457	0.390375327
pcaec381.histidine	-0.040127619	0.046571556	0.39039476
pcaac424.pcaac366	-0.023397773	0.02716147	0.390505098
pcaac421.pcaec322	0.06011411	0.069793336	0.39056906
kynurenine.pcaec341	-0.035309354	0.040995158	0.390574794
smohc222.smc260	-0.029636964	0.034410343	0.390588387
c5.pcaac421	0.020692324	0.024032827	0.390741043

pcaac320.c101	-0.007603542	0.008832182	0.390802478
pcaac402.pcaec385	-0.06295639	0.073130701	0.390811039
c16.pcaac385	-0.046084992	0.053537476	0.390852877
pcaac240.citruline	-0.03141965	0.03650858	0.390956487
pcaac321.glutamine	-0.008246827	0.009584773	0.391066714
pcaec380.pcaac403	0.007644622	0.008884892	0.391068029
pcaec423.c14	-0.045993089	0.053462549	0.391134324
lysopcac280.pcaac383	-0.014997311	0.017433054	0.391137585
pcaac422.lysopcac140	0.000829599	0.000964749	0.391339878
pcaec402.pcaec405	-0.058138048	0.067613792	0.391371204
kynurenine.pcaec403	0.025004993	0.02908146	0.391387116
serotonin.c181	-0.011977059	0.013930451	0.391414894
pcaec446.pcaac406	0.026148137	0.030412792	0.391415459
pcaac384.c2	0.045032464	0.05238088	0.39144977
pcaec405.glutamine	-0.008317515	0.009675524	0.39148652
c18.pcaac426	0.037126425	0.043196095	0.39157405
c0.pcaac384	0.051862411	0.060343441	0.391591472
pcaac365.glutamine	-0.00675701	0.007862021	0.391593912
kynurenine.pcaac320	-0.037559265	0.043705939	0.3916415
histidine.phenylalanine	-0.022119507	0.025739843	0.391649205
c102.serotonin	0.015813556	0.018402208	0.391660013
pcaac426.ornithine	-0.017066189	0.019861856	0.391706655
pcaac240.pcaac300	-0.03569204	0.041539141	0.391709773
smc181.c101	-0.023121017	0.026912078	0.39176866
c14.smc181	-0.022904853	0.026663658	0.391825114
smc260.pcaac425	-0.061139315	0.071174131	0.391835326
lysopcac280.pcaec401	0.02610112	0.030386467	0.391856474
pcaec386.c141oh	0.074326892	0.086540006	0.391910725
asymmetricdimethylarginine.c102	0.015610769	0.018176877	0.391936861
c8.pcaec421	-0.002854851	0.003324173	0.391942515
pcaac424.serine	0.075657018	0.08810799	0.392014162
kynurenine.asymmetricdimethylargi	-0.016972834	0.019769874	0.392104917
c18.smc240	-0.032029553	0.037307918	0.392105562
pcaac401.isoleucine	-0.01250741	0.014570392	0.392163969
c0.lysopcac204	0.028697322	0.033433007	0.392196818
pcaac341.asparagine	0.006022663	0.007017434	0.392257344
pcaec383.pcaec382	0.027484942	0.032029748	0.392332302
pcaec406.ornithine	-0.014269727	0.016629569	0.39233953
pcaec405.pcaac421	0.013393792	0.015609685	0.392366975
c181.pcaac320	-0.038713161	0.04512611	0.3924525
smc261.c16	-0.028956134	0.033754455	0.392475618
lysopcac140.arginine	0.037216128	0.043388966	0.392538211
pcaec402.pcaac343	0.011907974	0.013884572	0.392588767
smc160.pcaac381	-0.02820011	0.032881655	0.39259772
pcaac281.smohc141	0.023784146	0.027733648	0.392615719

pcaac381.serine	0.036775048	0.042881839	0.392616513
pcaac300.c5	-0.009179884	0.010705016	0.392649065
lysopcac180.c14	0.023077586	0.026912676	0.392666897
leucine.pcaac322	0.039174538	0.045687369	0.392694884
c4.pcaac421	0.013232697	0.015432712	0.39269664
pcaac342.pcaac343	-0.022487351	0.026228296	0.392737832
c0.serotonin	0.060256003	0.070284454	0.392767376
pcaac404.lysine	-0.054621896	0.063721454	0.392832529
smohc241.h1	0.004827203	0.005631839	0.392871403
spermidine.histidine	-0.02407036	0.028084701	0.392906778
glutamicacid.putrescine	-0.057073272	0.066592185	0.392910327
pcaac404.glutamicacid	-0.044039741	0.05140217	0.393069207
pcaec406.lysopcac203	-0.011385401	0.013289567	0.393097241
pcaec444.lysine	0.056071471	0.06545662	0.39315063
pcaac405.lysopcac160	-0.01882134	0.021975511	0.393234094
pcaac425.c16	0.021512907	0.025118666	0.393243911
smc160.smc260	-0.030432484	0.035535274	0.39327086
pcaec445.pcaec382	-0.011308369	0.013204759	0.393279775
lysopcac161.pcaac405	0.032308548	0.037728928	0.393308624
pcaec363.pcaac366	-0.013031043	0.015222236	0.393463188
c141.pcaec421	-0.001786027	0.002086449	0.39348557
c0.pcaac240	-0.014845232	0.017345277	0.393566331
pcaac363.c18	-0.022424506	0.026201366	0.393573726
c181.histidine	0.050890833	0.059469088	0.393628784
c141oh.pcaac383	0.005787403	0.006763174	0.393645183
pcaac424.lysopcac140	0.001240578	0.001449825	0.393671823
c162.pcaac323	0.047031525	0.054984017	0.393841341
lysopcac182.lysopcac160	0.008288288	0.009690158	0.393861544
pcaec424.isoleucine	-0.010151486	0.011869035	0.393883043
c2.phenylalanine	-0.04087507	0.047796267	0.393936993
glycine.pcaec341	0.029160065	0.034099999	0.39397002
pcaac404.pcaac421	0.014795737	0.017302431	0.393975064
c10.pcaac321	0.017316345	0.020250601	0.393987197
pcaac343.pcaec446	-0.030792157	0.03601006	0.393989411
pcaec343.serotonin	0.070808262	0.082812453	0.394019799
c102.pcaac425	-0.060599971	0.070878616	0.394053576
pcaec343.pcaac322	0.039578767	0.046300578	0.394141916
pcaac240.c0	-0.002793668	0.003268847	0.394245872
pcaac365.creatinine	0.007398703	0.008657904	0.394286371
smc180.c5	-0.003818233	0.004468103	0.394290257
acetylornithoine.taurine	-0.030346596	0.035513854	0.394319656
smc202.pcaac323	0.103159122	0.120733908	0.394356354
c16.c101	-0.006418805	0.007512587	0.394371244
pcaec406.methioninesulfoxide	0.019830565	0.023210226	0.39438097
pcaec360.smc161	-0.043510065	0.050934511	0.394465792

pcaac363.c102	0.027421307	0.032104132	0.394520495
c0.pcaec383	0.052995612	0.062056931	0.394604746
pcaac403.smc202	-0.061499825	0.072030996	0.394708051
c14.kynurenine	-0.003977845	0.004659797	0.394787942
pcaec383.prolinr	-0.015635254	0.01832015	0.394901879
smc181.smohc161	0.017029931	0.019954445	0.394904795
pcaac425.smc202	-0.03117102	0.036524316	0.394909476
smohc221.pcaec422	0.028511932	0.033410121	0.394931537
pcaec300.methionine	-0.023527719	0.027572272	0.394976395
pcaac402.pcaec342	0.063650137	0.074593639	0.394986946
c0.c51	-0.042385202	0.049673326	0.39499404
c141.smohc241	0.060593269	0.07102801	0.39509855
pcaec403.c16	0.013338956	0.015636973	0.395126318
pcaec341.pcaec301	-0.03124496	0.03663074	0.395164175
lysopcac204.pcaec380	-0.04717532	0.055308664	0.395177834
pcaac421.lysopcac140	0.000883972	0.001036392	0.395185438
pcaec341.c162	-0.04801339	0.056298712	0.395240539
pcaec425.pcaac322	0.045783755	0.053684569	0.395242679
c181.pcaec382	-0.00669849	0.007855348	0.395297389
alphaaminoadipicacid.pcaac381	0.018090611	0.021216714	0.395336916
pcaec342.pcaec363	0.085906444	0.10075625	0.395360101
smc160.pcaec385	-0.042355711	0.049681033	0.395395255
pcaac401.lysopcac140	0.00123356	0.001446939	0.395407503
smc160.pcaec424	-0.003461368	0.00406013	0.395409892
asparticacid.lysopcac161	0.054901822	0.064399636	0.395414799
leucine.pcaac421	0.03121602	0.036620754	0.395472421
pcaac404.lysopcac182	-0.017960248	0.021070152	0.395478465
pcaac321.pcaac281	-0.005673507	0.006658306	0.395648671
pcaec423.creatinine	0.009344908	0.010967121	0.395654529
c18.asparticacid	0.011443271	0.013430542	0.395682506
phenylalanine.pcaec364	-0.013973396	0.016402482	0.395752223
pcaec341.pcaec384	-0.071498695	0.083930975	0.395770167
pcaec405.pcaac322	0.058159669	0.068278909	0.395814106
leucine.c101	0.005513852	0.006473458	0.395832126
pcaac342.pcaac341	0.011579759	0.013595503	0.39584785
lysopcac280.leucine	-0.016849338	0.019782785	0.395857442
pcaac240.pcaec422	-0.068630232	0.080580814	0.395870013
pcaec425.pcaec340	-0.01545814	0.018151112	0.395902359
c102.pcaec341	0.045558075	0.053502565	0.395970977
c162.pcaec405	-0.018629337	0.021878322	0.395978965
pcaec362.pcaec402	-0.029370238	0.034508588	0.396199303
c162.pcaac344	-0.028245179	0.033188588	0.396226128
c3.glutamine	0.011602271	0.013633937	0.396262796
pcaec340.pcaec322	-0.05084003	0.059743745	0.396271894
alphaaminoadipicacid.c18	-0.03238249	0.038057225	0.396315537

smc181.smc261	0.041035391	0.048234875	0.396397643
pcaac343.pcaac322	0.08645452	0.101629084	0.396427712
h1.creatinine	-0.001393157	0.001637936	0.396499827
smc240.pcaac405	-0.018435017	0.021675704	0.396535484
pcaec404.pcaec445	0.039241937	0.046143535	0.396568836
smohc222.creatinine	0.004237153	0.004982663	0.396598021
citruline.creatinine	0.006415786	0.007545302	0.396640753
pcaac380.pcaec382	-0.010769572	0.012667267	0.396703346
smc180.arginine	-0.016121879	0.018962993	0.396710673
lysopcac281.pcaec385	-0.033230713	0.039087432	0.396717447
glutamicacid.creatinine	0.008653054	0.010178495	0.396735532
pcaac404.c102	0.018771918	0.022085583	0.396828844
smc240.creatinine	0.005650028	0.006647807	0.396858758
pcaec385.pcaac421	-0.043258142	0.050903315	0.39691343
pcaec343.creatinine	0.013021781	0.015324296	0.396948057
acetylornithoine.isoleucine	0.012758902	0.015015027	0.39695096
smc261.pcaec340	0.040361077	0.047501141	0.396981829
methionine.pcaac402	-0.023965203	0.028210365	0.397075385
pcaec382.pcaec445	0.068109093	0.080176851	0.397093071
pcaec380.c141	-0.050373825	0.059299655	0.397096689
c51.pcaac385	0.037773072	0.044471237	0.397150535
pcaac401.pcaac385	-0.026981088	0.031767885	0.397185159
pcaac401.pcaac380	0.042615726	0.050180559	0.397225012
pcaec340.pcaac426	0.052300545	0.061586516	0.397239985
creatinine.glutamicacid	-0.036336564	0.042789445	0.397254488
pcaec403.pcaec361	0.005396298	0.006354633	0.397256437
pcaec444.pcaac385	-0.012641575	0.014890128	0.397367705
lysopcac281.c16	0.012229812	0.014405646	0.397384766
asparagine.pcaac384	-0.027810342	0.032758968	0.397396595
pcaec402.smc261	0.044073387	0.051926888	0.397496289
smc260.c101	-0.032042364	0.037752579	0.397503024
lysopcac280.histidine	0.042147031	0.049675082	0.397665083
glutamine.c2	0.035648642	0.042023386	0.397747934
c181.pcaec401	-0.036588381	0.043131656	0.397753229
pcaec385.pcaac403	-0.001969916	0.002323039	0.397922119
pcaec401.pcaec403	0.039315958	0.046365436	0.397940214
kynurenine.lysopcac161	0.043361114	0.05114562	0.398029647
pcaec443.pcaec445	0.047473609	0.056006983	0.398118401
c182.pcaac405	0.041345443	0.048778649	0.398131613
smc181.pcaac365	-0.041083628	0.048476013	0.398192275
pcaac365.ornithine	-0.026107199	0.03080786	0.39823901
pcaac424.c16	0.014635694	0.017271088	0.398244552
c142.pcaac421	0.014983819	0.017682069	0.398249124
pcaac320.pcaec340	-0.055265472	0.065219521	0.398263361
smc240.pcaec402	-0.03053119	0.03603408	0.398313186

pcaac426.pcaac385	-0.019726969	0.02328534	0.398370085
lysopcac181.lysopcac180	0.080286851	0.094771574	0.398382472
pcaac240.pcaac281	-0.0026025	0.003072249	0.398417012
lysopcac203.glutamine	-0.008688712	0.010257645	0.39844592
lysopcac181.c14	0.039327671	0.046433034	0.398485563
pcaac404.pcaec380	0.029774405	0.035158745	0.398552105
leucine.lysopcac160	0.014904033	0.017600931	0.398597142
pcaec420.histidine	0.040254247	0.047544815	0.398661712
c8.smc202	-0.037885283	0.04474815	0.39867589
pcaec421.pcaec382	0.017890192	0.021131028	0.398677069
c142.pcaac341	-0.003777333	0.004461608	0.398677575
pcaec364.c5	-0.006841319	0.008081274	0.398714327
pcaec386.pcaac405	-0.023846161	0.028170951	0.398760904
methionine.leucine	0.007184334	0.008488408	0.398822343
pcaec402.glutamine	-0.019371722	0.022888083	0.398823881
c10.c162	-0.070889325	0.083762745	0.398855156
smohc222.smohc241	-0.109472655	0.129357706	0.398873127
smohc141.phenylalanine	-0.036759832	0.043440719	0.398913025
pcaec380.pcaac300	-0.042266845	0.04995367	0.398960761
pcaac366.asparticacid	-0.022856292	0.027017047	0.399030635
smc260.lysopcac280	0.009130248	0.01079427	0.399115758
pcaec403.glutamine	-0.012793492	0.015125624	0.399130375
lysopcac181.threonine	0.030520718	0.036084736	0.39913534
h1.pcaac381	0.028097811	0.033231061	0.399290105
pcaec402.creatinine	0.007898559	0.009341787	0.399301367
h1.smohc221	-0.041367937	0.048928282	0.399316509
c14.c5	-0.003052039	0.003610049	0.399345641
putrescine.pcaec362	-0.008187682	0.009685748	0.399398875
pcaec360.c18	0.032974802	0.039011541	0.399440815
lysopcac180.pcaac322	-0.065922425	0.077998575	0.399486908
c141.lysopcac203	-0.006452075	0.007634303	0.399504758
pcaac365.lysopcac181	0.020798021	0.024610874	0.399542688
pcaec360.glutamine	-0.007545867	0.008929722	0.39956844
c5.pcaec403	0.08231814	0.097428157	0.399633536
c12.c101	-0.017336858	0.020520861	0.399672762
creatinine.alanine	-0.013180492	0.015603384	0.399739872
pcaec443.histidine	-0.055238427	0.065402605	0.399811925
lysopcac280.pcaac406	-0.031192182	0.036934487	0.39984719
lysopcac182.threonine	0.019138431	0.022663462	0.399883574
creatinine.smc181	-0.023148319	0.027413794	0.399915777
pcaac240.asparticacid	0.046975615	0.055635663	0.399949441
c16.smc180	-0.01266955	0.015008027	0.400037774
pcaac386.pcaec301	0.054724527	0.064828703	0.400062563
serine.pcaac384	-0.045946605	0.054436456	0.400117867
pcaac384.pcaec380	0.029689493	0.035179944	0.400178409

pcaac343.pcaec341	-0.064143532	0.07601095	0.400211869
citruline.pcaec386	0.008913733	0.010563729	0.400249063
pcaac362.creatinine	0.00350427	0.004153219	0.400281221
pcaec380.pcaac404	-0.006438729	0.00763142	0.40030048
pcaec420.smc202	-0.027575392	0.032685763	0.400334848
pcaec300.putrescine	0.020008241	0.023716567	0.400341329
pcaec406.pcaec445	0.03302507	0.039150119	0.40039154
pcaac422.c16	0.014150181	0.016776385	0.400442444
pcaec300.asparticacid	-0.020914339	0.02479741	0.400470367
pcaec365.smc202	-0.038696524	0.045882077	0.40048003
c8.lysopcac160	0.016347752	0.019383483	0.400482937
pcaec365.c102	0.015566607	0.018462906	0.400626076
pcaac366.acetylornithoine	-0.030682415	0.036400882	0.400751853
c10.pcaec382	-0.009310448	0.01105138	0.400993668
h1.c141	0.012237136	0.014529046	0.401114134
pcaac240.c8	-0.045666737	0.054228197	0.401187558
acetylornithoine.smc161	-0.064312754	0.076377935	0.401236884
asymmetricdimethylarginine.pcaec34	0.033425249	0.039704272	0.401336139
histidine.arginine	0.017988296	0.021368609	0.401361711
glutamicacid.pcaac421	0.01499458	0.017816902	0.401482426
pcaec420.pcaec365	-0.014019787	0.016660236	0.401527694
pcaec341.asparagine	0.005225637	0.006209851	0.401530054
pcaec343.pcaec364	0.011435364	0.013589776	0.401552123
pcaac365.pcaac420	-0.046819697	0.055679407	0.401880433
alphaaminoadipicacid.pcaec380	0.023038255	0.027402453	0.401960284
pcaec301.pcaec403	-0.008241797	0.00980422	0.402015679
pcaec425.pcaac404	-0.00617649	0.007348256	0.402071262
serine.pcaec402	-0.030703492	0.036528924	0.402078365
kynurenine.taurine	-0.036341461	0.043251361	0.402238639
alanine.c2	-0.053291018	0.063431408	0.402295849
histidine.pcaac402	-0.034982923	0.041640114	0.402301782
pcaec341.pcaac366	-0.01032905	0.012295456	0.402332498
pcaac425.pcaec422	0.022736045	0.02706622	0.402363247
smc240.lysopcac182	-0.036029583	0.042897013	0.402422817
pcaac362.pcaec444	-0.079463809	0.094611339	0.402429292
pcaec421.pcaac300	-0.042561306	0.050677019	0.402453391
pcaec380.pcaec406	-0.021630832	0.025760045	0.402536906
smohc222.pcaac320	0.065170813	0.077612225	0.402540795
pcaec401.acetylornithoine	-0.024967127	0.029744673	0.402717621
lysopcac160.glutamine	-0.006912793	0.008237354	0.402818711
glutamicacid.pcaac425	-0.043902886	0.052329392	0.402946632
pcaec422.smc261	0.033045054	0.039396762	0.403056217
lysopcac281.pcaec363	0.016797716	0.02002924	0.40312123
pcaac401.lysopcac280	0.013285623	0.015841864	0.403132164
pcaac323.pcaac403	-0.003592394	0.004284236	0.40320239

pcaec421.asparticacid	0.031715455	0.0378247	0.403218759
pcaec404.glutamine	-0.00858186	0.010234983	0.403220016
lysine.pcaec422	0.033058869	0.039427608	0.403227299
pcaec404.pcaac323	-0.047326656	0.056448225	0.403261886
pcaec404.pcaec385	-0.055296561	0.065961042	0.4033104
c51.pcaec406	-0.00757253	0.009034367	0.403383167
pcaac342.pcaec384	-0.01195655	0.014266838	0.40345345
pcaec420.smohc141	-0.019686546	0.023493438	0.403513065
lysopcac140.glycine	0.035333107	0.042166117	0.403518185
c101.pcaec382	0.015492478	0.018489274	0.403536744
pcaec425.pcaec403	-0.029252736	0.034916294	0.403604429
lysopcac181.smc160	-0.050364307	0.060120281	0.403643844
pcaec362.pcaec364	-0.009318381	0.011123935	0.403665261
pcaec301.pcaac381	0.018706017	0.022330916	0.403673071
kynurenine.pcaec363	-0.018625027	0.022238336	0.403759623
lysopcac170.acetylornithoine	-0.028005174	0.033441513	0.403805272
c51dc.pcaac420	-0.050959901	0.060863195	0.403890055
pcaac385.alanine	-0.013625929	0.016276087	0.403952363
pcaec421.c0	-0.001425584	0.001702882	0.403961014
pcaec341.c3	-0.03954766	0.047240646	0.403964411
pcaec402.pcaec383	0.103506203	0.123656304	0.40402362
lysopcac170.pcaac366	0.019601318	0.023417426	0.404027944
pcaac240.pcaac323	0.096282921	0.115031402	0.404042262
pcaec405.pcaac406	0.030900406	0.036933923	0.404251788
pcaec300.pcaac386	0.007569443	0.009051548	0.404465121
pcaac381.c141	-0.035792311	0.042805029	0.404514918
smc180.tyrosine	-0.052718202	0.063050634	0.404540625
pcaec402.lysopcac160	-0.025707589	0.030746249	0.404542709
pcaec341.smc160	0.071730255	0.085789957	0.404546248
pcaec420.pcaec301	0.048663399	0.058202199	0.404549356
pcaac361.creatinine	0.003505924	0.004193869	0.404630683
pcaec386.pcaac361	-0.061938513	0.074094612	0.404645293
histidine.pcaec341	0.029540584	0.035339803	0.404666008
pcaec343.c101	-0.005793249	0.006932292	0.40478429
c16.pcaec300	-0.015675326	0.018758109	0.404803523
c51.pcaec362	0.006251496	0.007481093	0.404812791
smohc221.pcaec364	-0.015127597	0.018103865	0.404834676
pcaac300.pcaac240	-0.023008691	0.027537075	0.40486114
pcaac343.c18	-0.029232257	0.034987322	0.404885393
pcaec321.pcaec382	-0.013523276	0.016188246	0.404960402
smc240.c2	0.034811637	0.041675845	0.405005804
pcaac381.pcaac380	0.041801872	0.050052537	0.405081755
phenylalanine.pcaec380	0.037460727	0.04485889	0.405127024
alanine.smc261	0.014066829	0.01684591	0.405155001
c182.lysopcac140	-0.000729255	0.000873348	0.405165256

c141.c162	-0.072109026	0.08635872	0.405174748
smc202.c16	0.025090549	0.030067218	0.405462233
pcaec382.asparticacid	0.051466746	0.061675168	0.405462824
leucine.arginine	0.043530674	0.052170491	0.405512324
pcaac360.asparticacid	-0.026580921	0.031859037	0.405548015
pcaec405.lysopcac161	0.039177922	0.04696527	0.405626533
creatinine.pcaec421	-0.01404019	0.016832998	0.405683739
pcaec365.c162	0.050452697	0.060512704	0.405871052
lysopcac140.lysopcac161	0.0736742	0.08837371	0.405920105
c3.phenylalanine	0.020163851	0.024187405	0.405928875
c51.pcaac421	0.01039896	0.01247617	0.406010352
pcaec342.pcaac365	0.02594985	0.031133967	0.406019236
pcaac344.serine	-0.029247288	0.035090806	0.406028008
pcaac405.pcaec383	-0.037485405	0.044982844	0.406110985
smc161.lysopcac170	0.005976714	0.007173756	0.406218164
smc261.pcaec365	0.023511421	0.028226075	0.406312469
pcaec341.c102	0.017256605	0.020725305	0.40650001
smohc221.pcaac300	0.03902816	0.04687585	0.406527481
smc240.pcaec364	-0.014503442	0.017419958	0.40653282
pcaac363.methionine	-0.020995437	0.025222373	0.406624426
pcaac422.pcaac385	0.035682042	0.042866195	0.406628967
c12.pcaec341	0.056014717	0.067307599	0.406733207
c3.pcaec446	-0.060693206	0.072935882	0.406775454
lysopcac161.lysopcac204	-0.028397225	0.034130074	0.406840297
smc202.lysopcac180	0.028832566	0.034661163	0.406946438
c14.pcaec401	-0.025060472	0.03012923	0.406988408
pcaec365.pcaac320	0.046608522	0.056036595	0.406996628
spermidine.lysopcac182	-0.041617588	0.050036301	0.406998649
c2.lysopcac170	0.00993704	0.01194955	0.407091564
c162.pcaac426	0.030417798	0.036582305	0.407144114
pcaac384.lysopcac161	-0.047040837	0.056574242	0.407144559
c0.pcaec444	-0.034874702	0.041953269	0.407264786
h1.pcaac421	0.015651711	0.018829444	0.407286726
pcaec401.pcaac300	-0.038591074	0.046426411	0.407289517
pcaec424.pcaec362	0.006537371	0.007865036	0.40731035
c0.pcaec445	-0.034906016	0.041995123	0.407311404
pcaac343.pcaec340	-0.034842524	0.041919193	0.407316498
glutamine.smohc221	0.045996088	0.055341133	0.407342189
smc181.pcaac402	-0.037916492	0.045625978	0.407403473
pcaac360.transhydroxyproline	0.04855471	0.05842872	0.407415293
smc240.putrescine	0.0154701	0.018619134	0.407492141
pcaac422.pcaac361	-0.036213834	0.043586836	0.407507748
c16.smohc141	0.027423776	0.033008246	0.407523218
pcaec405.pcaec321	-0.024485333	0.029475321	0.407584964
phenylalanine.pcaac405	-0.02697681	0.03247994	0.407662616

arginine.pcaac404	-0.005837879	0.007028787	0.407663355
c16.pcaac422	0.02110193	0.025408178	0.407691397
pcaec406.smc181	0.019322077	0.023265542	0.407700019
smc161.pcaac420	0.047971582	0.057763199	0.40770846
pcaec425.pcaac405	-0.042495047	0.051171575	0.407733521
pcaac405.pcaec322	-0.045818157	0.055187307	0.407853181
pcaec423.lysine	0.06277925	0.075648805	0.408051715
pcaac342.pcaac320	-0.050850202	0.061285771	0.408138955
methioninesulfoxide.pcaac343	0.010875854	0.01310966	0.408204722
pcaac341.pcaac361	0.049077863	0.059173909	0.408330209
glutamicacid.pcaac381	0.014897079	0.017961756	0.408333506
c181.pcaec404	-0.009797331	0.011814244	0.408387854
phenylalanine.pcaac385	0.019745669	0.023810929	0.408394669
asparticacid.lysopcac203	0.005853323	0.007058421	0.408395319
smc241.pcaec382	-0.006946701	0.008378677	0.4084941
pcaec424.pcaac240	-0.011025717	0.013299102	0.408514139
pcaac366.glutamine	-0.007746976	0.009344601	0.408528202
pcaac341.pcaec380	-0.040887173	0.049319564	0.408532079
lysopcac180.kynurenine	-0.005466555	0.006594085	0.408541449
pcaec381.isoleucine	-0.01328924	0.016031433	0.408575183
pcaac422.pcaac381	0.028330631	0.03417906	0.408609277
creatinine.pcaec385	0.022439343	0.027074582	0.408660686
pcaac421.smc161	0.069905403	0.08436436	0.408764702
smc180.pcaac362	-0.028885744	0.034860808	0.408770741
smohc141.pcaac300	0.046288476	0.055864649	0.40878183
serotonin.c14	0.03114119	0.037586504	0.408816848
lysopcac181.pcaec405	-0.023788531	0.028714314	0.408853538
pcaac401.pcaec362	0.006165849	0.007442624	0.408856115
c162.histidine	0.040261295	0.048598956	0.408862554
pcaec340.pcaac425	0.068226685	0.082381331	0.409008191
lysopcac180.acetylornithoine	-0.029291085	0.035370197	0.40903778
leucine.pcaac344	0.046037404	0.055593765	0.409052026
pcaac402.tyrosine	-0.040549087	0.048971258	0.409100353
pcaac426.pcaac240	-0.010281224	0.012416984	0.409112311
smc241.pcaec445	0.040065848	0.04839628	0.409183708
arginine.leucine	0.021423848	0.025881085	0.409234657
smc260.phenylalanine	-0.033998492	0.041072686	0.409243751
creatinine.c162	0.074067598	0.089482873	0.409263335
c141.valine	-0.046162388	0.055775331	0.409308768
creatinine.c16	-0.013327623	0.016110121	0.409515534
pcaac342.phenylalanine	-0.023242371	0.028098568	0.409577558
smohc141.creatinine	0.003463474	0.004187293	0.409596552
pcaec343.tyrosine	-0.037498751	0.045339509	0.409638071
lysopcac180.c2	-0.041656033	0.050371093	0.409684812
pcaac385.c4	-0.014786106	0.017880621	0.409712047

ornithine.pcaac322	0.031189681	0.037719282	0.409737539
pcaec424.lysopcac160	-0.017884536	0.021628858	0.409741212
pcaac363.pcaac422	-0.01149093	0.013897128	0.409756241
c181.lysine	0.042912524	0.051905228	0.409817726
pcaac366.c2	0.058479881	0.070737808	0.409837156
alphaaminoadipicacid.pcaac281	-0.00189934	0.00229751	0.409847463
smohc241.asymmetricdimethylargin	0.016351752	0.019783329	0.409933912
c4.smc180	-0.012326365	0.014914768	0.409983791
c16.taurine	-0.054542903	0.066001043	0.410017215
pcaec404.lysopcac170	-0.01217476	0.014732996	0.410036705
pcaac365.smc240	0.026946578	0.032618006	0.410169099
lysopcac160.c18	0.048436373	0.058631501	0.410175115
c141oh.pcaac240	-0.008301102	0.010049206	0.410214458
methioninesulfoxide.pcaec385	-0.054639214	0.06616969	0.410385051
pcaec321.smc181	0.037515296	0.045433661	0.410400862
kynurenine.c141oh	0.085362671	0.103390911	0.410449238
pcaec301.pcaec363	0.034276134	0.04151758	0.410477014
pcaec403.pcaec321	-0.030425222	0.036859197	0.410554325
c3.pcaac381	0.013540086	0.016406849	0.410652828
smc261.pcaec420	0.018669731	0.022625373	0.410710784
c162.pcaac320	-0.039383092	0.047730033	0.410736847
smc202.pcaec365	0.01881405	0.022803164	0.410770035
pcaec421.prolinr	-0.013042995	0.015809222	0.410791904
pcaec342.pcaec384	-0.05480504	0.066429602	0.410800495
pcaec380.pcaac420	-0.060177491	0.072942074	0.410803592
spermidine.lysopcac160	-0.04051689	0.049117946	0.410868145
pcaac383.smc241	0.015755297	0.019102968	0.410943523
pcaac322.c14	0.03575687	0.043354629	0.410945402
c5.transhydroxyproline	-0.031777468	0.038539391	0.411063153
pcaec360.pcaac420	0.091540009	0.111021321	0.411073771
pcaec446.pcaec424	-0.010000065	0.012128909	0.411099023
serotonin.pcaec402	-0.020196283	0.024496081	0.411105718
pcaec341.pcaec361	-0.006842	0.008300459	0.411206566
c10.smc261	0.04316846	0.052372341	0.411224191
pcaec340.pcaec384	0.070601237	0.085660267	0.411258237
leucine.methioninesulfoxide	0.011669912	0.014160035	0.41129007
pcaec423.glutamine	-0.008205881	0.009958268	0.411356526
pcaac321.serotonin	0.061355159	0.074461626	0.41138106
pcaac341.pcaac323	-0.04414292	0.053573105	0.411385705
pcaec340.pcaac323	-0.056341698	0.068394185	0.411496888
c142.smohc222	-0.003163244	0.003839966	0.411502778
pcaac381.phenylalanine	0.030316663	0.036805226	0.411538604
lysine.pcaec363	0.021086339	0.025599743	0.411545405
pcaac385.lysopcac203	-0.007191787	0.008732085	0.411595569
asparticacid.pcaac321	0.014167971	0.017203682	0.411630605

isoleucine.alanine	0.007825527	0.009504436	0.41173693
pcaec420.pcaac425	-0.053161765	0.064577513	0.411811266
lysopcac181.acetylornithoine	-0.023185444	0.02816472	0.411819955
pcaac385.glutamine	-0.00474739	0.005767001	0.41182536
pcaec363.pcaac405	-0.028216995	0.034286863	0.411956327
pcaac383.lysopcac140	-0.001175783	0.001428791	0.411982709
pcaec423.pcaac320	-0.076508293	0.092977082	0.412010448
pcaac405.smc241	0.019309623	0.023466896	0.412025979
pcaec444.pcaec422	0.035148244	0.042726485	0.412145892
c16.pcaac364	0.054290717	0.065998269	0.412160315
pcaec384.lysopcac281	0.020759698	0.025236601	0.412163398
pcaec425.isoleucine	-0.008216643	0.009990353	0.412245644
histidine.pcaec340	0.024550422	0.029854697	0.412317877
pcaac322.c101	-0.005165596	0.006281736	0.412323795
lysopcac161.pcaec343	0.064571935	0.078524412	0.412325591
pcaac361.lysopcac170	0.005161792	0.006277476	0.412350983
c0.c12	-0.022248211	0.027058405	0.412375197
pcaec446.smc160	-0.064908426	0.078948959	0.412416244
smohc221.pcaec322	-0.074029918	0.090046904	0.412433623
pcaec404.lysopcac204	-0.048663001	0.059202997	0.412523027
smc241.pcaec341	0.067508985	0.082136905	0.41255733
pcaec342.pcaec444	-0.070077055	0.085272831	0.41261968
pcaec364.pcaec340	0.052084719	0.063386199	0.412672863
ornithine.asparagine	0.012039435	0.014653639	0.412731897
c12.pcaec423	0.026142789	0.031820976	0.412755717
phenylalanine.pcaac425	-0.047918163	0.058328969	0.412779933
pcaac322.pcaec421	-0.004357322	0.005304181	0.412795537
c12.smc261	-0.026264484	0.031978834	0.412897586
lysopcac180.citruline	0.025628721	0.031211485	0.412998186
c2.pcaac384	0.059664981	0.072663924	0.413010889
pcaac405.smc180	0.022467234	0.027363601	0.413036969
pcaac384.c16	0.022965543	0.02797156	0.413054484
pcaac240.pcaac420	0.073213344	0.089173118	0.413058502
pcaac421.pcaec402	-0.0568905	0.06929838	0.413101022
c182.smc240	-0.025050643	0.030515695	0.413123612
c14.histidine	0.035173758	0.042847801	0.413129396
pcaec363.smc260	0.024849836	0.030276285	0.413203748
pcaac380.c101	-0.02067998	0.025196421	0.413214125
pcaec402.pcaec361	0.012408325	0.015123149	0.41336468
smc181.pcaec364	0.022347534	0.02723699	0.413365143
pcaec404.creatinine	0.008402631	0.010241529	0.413386621
h1.pcaac426	-0.032230646	0.039287319	0.41342296
methioninesulfoxide.pcaec401	0.029730968	0.036242206	0.413446762
pcaec301.pcaac406	-0.045975558	0.056057519	0.413555423
pcaac406.smc260	-0.019876899	0.024235842	0.413558222

pcaac364.serotonin	0.06691754	0.081595684	0.413577263
pcaec363.pcaac362	-0.007581962	0.009245323	0.413591465
pcaec405.smc202	-0.047327293	0.057717416	0.413650193
pcaec421.pcaac385	-0.025086672	0.030594585	0.41365697
lysopcac181.kynurenine	-0.00434076	0.005294977	0.413760691
pcaec364.lysopcac181	0.021150821	0.025809934	0.413933844
smohc161.tyrosine	-0.050624391	0.061781139	0.413972898
c141oh.c142	0.035775448	0.043664038	0.414018708
pcaac406.smc161	0.043792703	0.053454118	0.414062251
arginine.pcaac425	-0.056756449	0.069327239	0.41439386
kynurenine.c162	-0.077394111	0.094559994	0.414512675
lysopcac182.pcaec360	-0.006830925	0.008348395	0.414645585
pcaec421.pcaac404	-0.007893255	0.009650566	0.414831163
glutamine.pcaec424	-0.012708715	0.015538635	0.414846806
pcaec363.smc241	0.015135257	0.018508635	0.414925419
methionine.h1	-0.001752347	0.002143047	0.414954314
c102.smohc161	0.007625077	0.009327078	0.415050567
ornithine.histidine	-0.035300378	0.043182335	0.415077765
lysopcac180.glutamine	-0.007594326	0.009290663	0.415110667
smohc222.lysopcac182	-0.015555038	0.01903074	0.415139736
lysopcac160.pcaec382	-0.005401678	0.006608919	0.415158116
lysopcac182.pcaec402	-0.016477815	0.020166019	0.415285425
lysopcac203.alanine	0.036641661	0.044844626	0.415301161
pcaec383.asparagine	0.008075151	0.009883406	0.415323384
phenylalanine.lysopcac181	0.02762156	0.033807104	0.415327422
pcaac320.lysopcac204	-0.010097936	0.012359284	0.415328446
pcaac380.smc202	-0.026411324	0.032326214	0.415332676
pcaec382.pcaec384	-0.028479685	0.034859331	0.415353255
pcaec380.pcaec340	-0.043047887	0.052690956	0.415353651
pcaac323.pcaac363	0.013637686	0.016693032	0.415364695
lysopcac280.c101	-0.010710993	0.013111965	0.415411294
c16.asparagine	0.007891044	0.00966037	0.415434073
pcaec380.pcaec343	0.048765764	0.059702311	0.415452067
pcaac425.c102	0.022232212	0.027221663	0.415511916
c0.glutamine	0.016668925	0.020410629	0.415529932
c141.transhydroxyproline	0.015808584	0.019360235	0.415603678
pcaec402.c51	-0.046862317	0.05739184	0.415613056
pcaec404.tyrosine	-0.044999106	0.055123922	0.415730697
lysopcac181.pcaac320	-0.042917469	0.0525753	0.415742946
asparagine.kynurenine	-0.006856734	0.008400369	0.415778731
pcaec364.ornithine	-0.026925558	0.032987919	0.41578852
pcaac361.pcaac420	-0.038509214	0.047188824	0.415878789
c181.pcaac300	-0.019045032	0.023338534	0.415897391
pcaac323.pcaac425	-0.060027284	0.073562224	0.415912696
pcaac425.lysopcac182	-0.00997156	0.01222052	0.415934568

pcaac364.c162	-0.044272436	0.054263652	0.415987067
c51.pcaac281	0.00152766	0.001872532	0.416015979
pcaec402.arginine	-0.028788743	0.035288221	0.416020758
pcaec422.pcaec445	0.039021161	0.047831704	0.416029964
c142.lysopcac203	-0.008347217	0.010232911	0.416074733
alanine.lysine	-0.040609802	0.049793046	0.416160768
citruline.smc260	0.011095417	0.013605851	0.416208158
isoleucine.pcaac385	0.049893825	0.061187378	0.416243453
pcaac421.smohc241	-0.049644237	0.060891564	0.416321876
smohc241.smc180	-0.012596621	0.015452194	0.416373072
pcaec402.pcaac422	-0.043185921	0.052996891	0.416557301
phenylalanine.asparagine	0.005632665	0.006912364	0.416562085
glycine.c16	-0.029229448	0.035872025	0.416586284
c0.glycine	-0.024041792	0.029510878	0.416671931
lysopcac204.pcaec420	0.015951426	0.019581868	0.416714016
pcaac321.pcaec364	-0.012553453	0.015411957	0.416756802
pcaec424.lysopcac180	0.037120295	0.045575699	0.416786051
pcaac344.pcaec405	-0.027875147	0.034230131	0.416860395
pcaac365.pcaac384	0.048405847	0.059444095	0.416881276
smc260.lysopcac170	0.01072807	0.013174555	0.416884963
asparticacid.pcaac385	0.023859396	0.029302062	0.416911066
c12.pcaac420	0.066739488	0.081975034	0.416975279
pcaec363.putrescine	0.018052701	0.022174099	0.416980695
pcaac321.pcaec406	-0.02762967	0.033937717	0.416984045
lysopcac160.pcaac300	-0.027621066	0.033933354	0.417069039
pcaec321.pcaec343	0.066256692	0.081402597	0.417092748
lysopcac280.pcaac365	-0.045742134	0.056204766	0.417144328
lysopcac181.smc180	0.00942651	0.011584884	0.417234262
smc160.lysopcac280	0.005962412	0.007328105	0.417265243
c51dc.pcaac425	-0.059665413	0.073338654	0.417308627
pcaec321.pcaec401	0.039125225	0.048094451	0.417338417
methionine.serotonin	0.071988806	0.088493723	0.417348443
c141oh.c8	0.040742352	0.050090489	0.417414427
pcaec300.citruline	0.024474981	0.030093627	0.417460429
pcaec321.pcaac365	-0.031307225	0.03849789	0.417503415
pcaac366.pcaac361	-0.024847339	0.030555063	0.41751511
smc202.pcaec362	0.024941916	0.030675667	0.417580264
c12.c2	0.033883719	0.041674161	0.417592665
pcaec322.smc260	-0.023546175	0.028971723	0.41778322
lysopcac204.pcaec341	0.027458817	0.033786419	0.41779004
c12.c3	0.029889557	0.036778226	0.417801765
lysopcac204.pcaac405	0.030189039	0.037157812	0.417940294
smc202.putrescine	0.028069988	0.03455346	0.417992126
c12.pcaac320	-0.059182327	0.072852776	0.417997218
c3.c4	0.008375069	0.010310151	0.418021354

smc260.smc161	0.058890188	0.072499293	0.418036535
pcaec364.pcaac343	-0.012490427	0.015377025	0.418041021
lysopcac180.pcaac420	-0.043683907	0.053780848	0.418052931
pcaac380.lysopcac170	0.017280777	0.021276254	0.41808044
c2.c141	-0.03274586	0.040318786	0.418100912
citruline.pcaac404	0.006464977	0.007960153	0.418104591
pcaac322.asparticacid	-0.033305908	0.041011056	0.418131525
smc260.smc261	0.019391361	0.023878395	0.418149888
pcaec322.c101	-0.00658919	0.008114627	0.418192348
pcaac361.pcaac343	0.010929775	0.013460496	0.418206547
pcaac422.prolinr	-0.024918704	0.030692007	0.418259992
pcaec422.pcaec301	0.057394034	0.070696392	0.418292781
pcaec301.lysopcac161	-0.057847769	0.071264014	0.418349616
pcaec420.pcaec421	0.002041359	0.002514947	0.418377186
pcaac322.pcaec341	-0.054586234	0.067275463	0.418552618
pcaac401.leucine	-0.031291126	0.038565216	0.418553709
lysopcac281.pcaac403	-0.015996604	0.019718092	0.418620526
pcaec405.pcaec322	0.049615679	0.061164501	0.41866691
smc161.lysopcac280	0.00829104	0.010221833	0.418708886
serine.valine	-0.035793162	0.044128727	0.418710552
arginine.pcaac240	0.010182678	0.012559093	0.4188975
lysopcac160.pcaac386	-0.015390723	0.018983667	0.418924011
pcaec405.smohc222	-0.006810009	0.008401556	0.419021212
pcaec422.pcaac420	-0.072618077	0.089605392	0.419103877
creatinine.smc160	-0.051337031	0.063347221	0.419111807
pcaac406.methionine	-0.022576934	0.027860435	0.419139704
isoleucine.c8	-0.018705368	0.0230836	0.419155045
pcaac320.pcaec403	0.048444703	0.059790165	0.41920437
isoleucine.creatinine	0.007904235	0.009758914	0.419373349
smohc161.pcaec342	-0.078265477	0.096640787	0.419425256
c5.methionine	0.056901404	0.07026837	0.419475116
pcaac405.pcaac425	-0.047340382	0.058465319	0.419506798
pcaac425.glycine	-0.01665295	0.020568312	0.4195505
prolinr.pcaec383	-0.025245068	0.031181874	0.419569929
serine.pcaac360	-0.025368588	0.031335072	0.419579252
pcaec361.c16	0.020043674	0.02476047	0.41962963
pcaec445.smc202	-0.038813676	0.04794804	0.419634457
pcaec421.methionine	-0.031757256	0.039232389	0.419651227
pcaac421.pcaac404	0.004907805	0.006063256	0.419669232
c141oh.pcaec406	-0.006204164	0.007665076	0.419684774
c14.smc180	-0.005951575	0.007353618	0.419723143
c12.pcaec384	-0.022533538	0.027842125	0.41972728
pcaac383.lysopcac203	0.011050159	0.0136535	0.419729904
smc161.c18	-0.01619535	0.020013022	0.419779972
lysopcac140.pcaec445	0.046308527	0.057229967	0.419822894

pcaec424.h1	0.002506755	0.003098245	0.419867063
asparagine.c16	-0.037140232	0.045911285	0.419942815
alphaaminoadipicacid.pcaac362	0.007114351	0.008795097	0.41997542
lysine.c102	0.018640038	0.023045734	0.42001641
lysopcac182.pcaec342	-0.047843252	0.059161475	0.420096004
c5.lysopcac203	0.072818663	0.090058512	0.420164087
pcaac322.c3	0.029955056	0.037051978	0.420227284
pcaec321.smohc141	0.022161233	0.027417475	0.420325788
kynurenine.pcaec401	0.019144646	0.023685627	0.420330071
lysopcac170.pcaec402	-0.016634663	0.020580306	0.420330351
smc241.creatinine	0.004214409	0.005214828	0.420400226
ornithine.lysopcac203	0.010457645	0.012942419	0.420483787
pcaec420.pcaec446	0.032774815	0.04057114	0.420585619
pcaac406.h1	-0.001778017	0.002201	0.420593365
lysopcac161.c101	-0.006265733	0.007756937	0.420629854
smc261.lysopcac281	-0.009380973	0.011615278	0.420697424
pcaec424.creatinine	0.006591498	0.008161855	0.420721974
pcaec321.c12	0.029699355	0.036774922	0.42072199
c142.pcaec361	-0.008083829	0.010010044	0.420737071
isoleucine.pcaac384	-0.042001508	0.052033908	0.42095336
lysopcac170.lysopcac203	-0.021603938	0.026766476	0.420992526
smohc221.pcaac385	-0.016025713	0.019857498	0.421044696
c51dc.asparagine	0.007328263	0.009084087	0.421229327
c5.c101	0.015236389	0.018889056	0.421280501
pcaec342.smc181	0.00925657	0.011477332	0.421347341
alanine.smc161	0.066402146	0.082346323	0.421423317
pcaac384.pcaec422	0.020017372	0.024829502	0.421528746
pcaac422.methionine	-0.033721566	0.04183164	0.421567368
pcaec301.smc161	-0.046992298	0.058295618	0.421580235
phenylalanine.lysopcac203	0.005801193	0.00719718	0.421618421
pcaec364.pcaec401	0.04224586	0.052414017	0.421637893
pcaac361.pcaac341	-0.005422176	0.006727486	0.421654852
asymmetricdimethylarginine.c101	0.00511515	0.006347229	0.421704492
pcaac366.c51	-0.085561212	0.106178379	0.421740137
lysopcac280.tyrosine	-0.036071644	0.044763728	0.421741361
kynurenine.pcaac380	-0.031649964	0.039286453	0.421857891
c2.c101	-0.00910501	0.011301892	0.421859107
lysopcac170.pcaac344	0.035333431	0.043859147	0.421862985
pcaec365.pcaac321	-0.016639337	0.020656681	0.421916469
pcaec421.pcaec301	0.056020591	0.069557425	0.421992464
glutamine.acetylornithoine	0.017094894	0.021227085	0.422022457
pcaec381.pcaec362	0.007764848	0.009642759	0.422069798
pcaec383.pcaec422	0.046999745	0.058389877	0.422254979
pcaec401.pcaec364	0.006762187	0.008401644	0.42229232
pcaac341.putrescine	0.021526656	0.026746766	0.42231109

pcaec422.c16	0.014634245	0.01818496	0.422361532
pcaac342.pcaac402	0.044211317	0.054946199	0.422427806
glutamine.pcaac424	-0.00870201	0.010816673	0.422502341
pcaac362.c4	-0.008221139	0.0102205	0.422572699
smohc241.pcaac366	-0.011210243	0.013937762	0.422613076
pcaec424.pcaac385	-0.028298634	0.035184915	0.422626917
serine.smc240	0.037200071	0.046253122	0.422633579
c162.prolinr	0.022030667	0.02739512	0.422685026
lysopcac161.creatinine	0.003224988	0.00401089	0.42275649
pcaec382.pcaac426	0.066222604	0.082366448	0.422790004
pcaec421.pcaec380	0.078096299	0.097145322	0.422840457
pcaec381.c4	-0.014676696	0.018256638	0.422841585
pcaec321.pcaec383	-0.038025268	0.047301005	0.422847547
c181.c51dc	0.066210373	0.082369733	0.422893933
pcaec443.glutamine	-0.008817199	0.010969348	0.422903019
lysopcac170.pcaec380	0.022042793	0.027423355	0.422907079
c18.lysopcac161	-0.059237776	0.073700339	0.42292495
pcaac401.pcaec340	-0.033840589	0.042104282	0.422943718
pcaec420.pcaac361	-0.034764602	0.043255657	0.422962142
pcaac364.asparticacid	0.04271203	0.053150989	0.423021253
pcaac361.lysine	0.045627782	0.056787609	0.423088461
smc160.tyrosine	-0.039821197	0.049570137	0.423175369
pcaec446.pcaec445	-0.122686613	0.152750125	0.423259078
pcaec362.c101	-0.012239988	0.015239566	0.423266778
pcaec421.pcaac422	-0.123715452	0.154069378	0.423374091
pcaec385.asparagine	0.021630703	0.026939458	0.423401624
glutamicacid.pcaec446	-0.056838218	0.070805852	0.42351927
smohc161.pcaac420	0.0464759	0.057898887	0.423533868
glycine.creatinine	0.009651961	0.012024334	0.423537163
pcaec386.pcaac320	0.064949268	0.080914922	0.423546592
pcaac424.pcaac425	0.091076388	0.113465147	0.423549064
pcaec383.transhydroxyproline	0.047402884	0.059056448	0.423555321
pcaec404.pcaac421	0.012351937	0.015393219	0.423695701
pcaec404.putrescine	-0.046371953	0.057798672	0.423768195
creatinine.lysopcac281	-0.01062302	0.013243099	0.423852475
pcaac383.c4	-0.014437378	0.018000722	0.423916303
pcaec402.pcaec364	0.004258098	0.005309147	0.423924238
alanine.lysopcac180	-0.030117036	0.037552401	0.423941745
pcaac406.c2	-0.044662417	0.055707786	0.424099493
pcaac321.pcaac384	0.039810371	0.049662513	0.424162066
pcaac281.pcaac405	0.026501133	0.033061676	0.424191825
c141.pcaec445	-0.030365703	0.037886563	0.424235932
pcaac386.lysopcac203	-0.009945982	0.012410779	0.424288647
prolinr.pcaec401	0.013427229	0.016755252	0.424302687
pcaac362.smc180	-0.006939117	0.008659479	0.424327131

pcaac344.pcaac404	0.003260126	0.004068663	0.424358869
kynurenine.c3	0.025578893	0.03192283	0.424361281
pcaac422.c141	-0.029382515	0.036669869	0.424362084
pcaac384.pcaec406	0.010308624	0.012867388	0.424435874
lysine.tyrosine	-0.02694779	0.033638366	0.424459298
pcaec365.pcaec341	0.042517746	0.053079667	0.424508483
pcaec425.glutamicacid	0.043019091	0.053718983	0.424624032
pcaac362.h1	0.002308712	0.002883235	0.424670552
c8.h1	0.005375313	0.006714198	0.424755793
putrescine.kynurenine	0.006203712	0.007749	0.424759624
c141oh.lysopcac280	0.008397748	0.010490883	0.424818354
pcaec445.phenylalanine	-0.036371294	0.04543751	0.424825352
h1.pcaec421	0.007098031	0.008868531	0.42488704
smc161.pcaec364	0.016862957	0.021075279	0.425020889
pcaac383.pcaac240	-0.011778751	0.014724076	0.425115774
h1.smc260	0.023466872	0.029336366	0.425139503
lysopcac161.smc161	-0.052714173	0.065901783	0.425159352
pcaec382.lysopcac181	0.047413983	0.059285381	0.425235298
c3.pcaec420	-0.027464625	0.034345167	0.42528931
c181.c162	-0.069456321	0.086858079	0.425296201
asparticacid.c16	0.031065511	0.038850924	0.425322162
pcaac403.citruline	0.01894964	0.023702093	0.425389019
pcaac383.serine	-0.018806478	0.023524239	0.42541283
c102.pcaec402	0.034819622	0.043556381	0.425433692
c51dc.c141oh	0.068020981	0.085092736	0.425456935
pcaac366.pcaac322	0.054190492	0.067807946	0.425571669
glycine.c8	0.024144254	0.030215937	0.425640542
pcaec445.pcaec421	-0.001005108	0.001258171	0.425751782
pcaac361.smc240	0.025068758	0.03138279	0.425785601
pcaac240.pcaac424	-0.013274264	0.01662264	0.425924672
lysopcac161.pcaac381	0.01465429	0.018353445	0.425991928
asparticacid.pcaac422	0.019554756	0.0244918	0.426008373
glutamicacid.pcaec405	0.043794852	0.054865986	0.426127227
pcaec342.lysopcac160	-0.010684486	0.013388197	0.426221114
pcaec444.creatinine	0.004057723	0.005084725	0.426238709
smohc222.c14	-0.020918485	0.026214144	0.426260409
citruline.pcaac422	-0.038631025	0.048415524	0.426306054
lysopcac180.pcaec423	-0.023313977	0.029225895	0.426415548
serotonin.histidine	-0.044975223	0.056386658	0.426470358
lysine.lysopcac140	0.000918477	0.001151619	0.42651042
smc260.creatinine	-0.006489069	0.008138338	0.426630046
pcaec301.pcaac240	-0.011105837	0.013928865	0.426641854
lysopcac161.asparagine	0.006907323	0.008663713	0.426673537
smc260.pcaec365	0.018592269	0.023324186	0.426758312
pcaec404.pcaec362	0.005966329	0.007486854	0.426883754

c4.pcaec424	0.008631126	0.010831724	0.426924085
pcaec446.pcaac300	-0.028232859	0.035431636	0.426930668
pcaac361.pcaac322	0.054672142	0.068627812	0.427034444
pcaac344.asparticacid	-0.020528038	0.025775372	0.42716553
lysopcac204.acetylornithoine	-0.01713817	0.021519286	0.4271718
pcaac343.pcaec362	0.026651802	0.033473708	0.427292678
pcaec401.c101	-0.013349599	0.016769195	0.427363549
c181.h1	0.002308015	0.002899248	0.427366517
smohc241.pcaec405	-0.022518077	0.028287526	0.427384449
serine.pcaac401	-0.018839528	0.023666817	0.427390992
pcaec445.pcaac362	-0.005078097	0.006379505	0.427408127
pcaec381.glutamine	-0.010207197	0.01282343	0.427420588
smohc221.pcaec342	-0.092738886	0.11652872	0.427498503
ornithine.pcaac403	0.003370394	0.004235644	0.427570371
c51.pcaec446	-0.047031232	0.059116194	0.427656684
pcaac381.c101	-0.015461434	0.019435114	0.427674961
glycine.pcaac343	0.015806121	0.019870395	0.427721536
pcaac401.pcaac426	0.066460913	0.083557244	0.427760383
pcaac386.pcaec343	-0.045194497	0.056832786	0.427861836
pcaec385.lysopcac170	0.005979351	0.007520131	0.42792334
glutamicacid.lysopcac180	-0.078359774	0.09855932	0.427958551
pcaac384.smc180	0.030635494	0.038533479	0.427967893
pcaac364.asparagine	0.007378964	0.009283526	0.428078512
c14.lysopcac160	0.01436883	0.018078541	0.428104406
pcaac386.pcaac425	0.070225371	0.08835788	0.428114249
pcaac342.alphaaminoadipicacid	0.007929175	0.009977812	0.42817373
pcaac321.pcaac381	0.014851304	0.018689458	0.428200062
smc240.pcaec421	0.00135829	0.001709704	0.428302382
smc240.pcaac362	-0.007415929	0.009334567	0.428302432
smohc161.pcaec403	-0.019304449	0.02430294	0.42837967
lysopcac203.pcaac422	-0.045712096	0.057564607	0.428510152
pcaac420.glutamicacid	0.044369747	0.055876488	0.428528974
smc240.c8	-0.02772307	0.034916219	0.428575377
pcaac364.creatinine	-0.006435584	0.008105861	0.428602205
smohc221.pcaac380	-0.037183866	0.046834589	0.42860325
c141oh.pcaac426	0.036255339	0.045666701	0.428619681
pcaac404.asymmetricdimethylarginin	-0.015879278	0.020007851	0.428770325
pcaac384.methionine	-0.012302858	0.015501686	0.428773754
lysopcac182.pcaac420	0.072020375	0.090772831	0.428909048
smohc161.phenylalanine	-0.030602069	0.038573413	0.428948036
asymmetricdimethylarginine.pcaec42	-0.004313621	0.005437284	0.428950918
lysopcac161.smc260	-0.017583652	0.022164807	0.428966701
pcaac401.pcaec322	0.034910529	0.044011989	0.429029996
pcaac385.lysopcac281	0.013570171	0.017108497	0.429042694
pcaac386.valine	-0.036957592	0.046594648	0.429048871

pcaec343.smc181	0.009422837	0.011880181	0.42905841
pcaec422.creatinine	0.016299975	0.020551611	0.429077031
lysopcac170.methionine	-0.023110268	0.029139149	0.429090766
pcaac364.pcaec445	0.034343975	0.043305474	0.429112345
pcaac342.asparticacid	0.021233541	0.0267781	0.429181366
pcaac424.pcaec404	0.047630838	0.06007089	0.429200988
pcaec383.pcaac366	-0.014502397	0.018294731	0.429318027
pcaec445.pcaec402	-0.018039683	0.022759149	0.429361359
citruline.c101	0.007710281	0.009727496	0.429365288
lysopcac204.asparagine	-0.004078754	0.005146102	0.429386542
c102.smohc141	-0.01742415	0.021985249	0.429417199
pcaac322.serine	-0.016818788	0.021222373	0.429437814
pcaec422.lysopcac281	0.007286818	0.009195159	0.429461222
pcaec364.pcaac320	0.064736835	0.081705128	0.429542335
c10.c16	0.027947288	0.035280198	0.429641426
pcaec363.pcaac421	0.008481556	0.010708027	0.429686535
pcaec425.pcaec384	-0.043738265	0.055224273	0.429723146
pcaec382.pcaec383	-0.167526613	0.21155074	0.42978915
pcaac425.h1	0.006127979	0.007740764	0.429933116
c14.isoleucine	-0.006514656	0.008229327	0.429939702
pcaec386.pcaac321	-0.015028777	0.018985695	0.429971519
pcaec301.pcaac426	0.04718419	0.059621776	0.430083356
pcaac365.pcaec322	-0.058289177	0.073660309	0.430122767
pcaec342.pcaac362	-0.009462515	0.011959356	0.430181653
pcaec301.lysopcac204	-0.025411027	0.032117455	0.430200422
pcaac384.pcaec301	0.036977782	0.046739935	0.430230433
pcaec300.c5	-0.005237545	0.006620395	0.430239832
pcaec402.c16	0.01442985	0.018240934	0.430270699
c12.isoleucine	-0.005403696	0.00683181	0.430333898
c12.smc160	0.0584051	0.073857681	0.430439793
pcaac383.pcaec301	0.034657845	0.043828847	0.430454195
lysopcac180.pcaec363	0.014533151	0.018379227	0.430463689
lysopcac180.pcaec384	-0.029373309	0.037148309	0.430483509
pcaec362.asparticacid	0.016350922	0.020679057	0.430485936
pcaec443.pcaec362	0.006103684	0.007719361	0.430486839
pcaec385.pcaac405	-0.028465949	0.036004755	0.430534361
putrescine.pcaac343	-0.013534077	0.017119	0.430550819
asparagine.pcaec385	0.039711571	0.050245065	0.430684899
c4.arginine	-0.042435919	0.053693248	0.430695194
pcaac300.c141oh	0.050192247	0.063515457	0.430755259
smc260.arginine	-0.021123403	0.026731974	0.430781034
pcaac405.pcaac426	0.038557267	0.048795922	0.430791782
pcaac386.kynurenine	-0.005166701	0.006539464	0.430846347
smc261.pcaec402	0.044819171	0.056733477	0.430895857
leucine.smc261	-0.04085458	0.051715111	0.430897059

pcaec385.lysopcac203	-0.008430116	0.010671508	0.430913514
pcaac426.spermidine	0.044997907	0.056963135	0.430923372
pcaac424.lysopcac170	-0.006427523	0.008139244	0.431070412
putrescine.smc181	0.015554613	0.01969974	0.431134794
glycine.pcaac344	0.022215431	0.028137027	0.431158273
smohc221.serine	-0.014264345	0.018070544	0.431259823
c102.creatinine	0.014093424	0.017858489	0.431374976
lysopcac161.c102	0.021758286	0.027573984	0.431424319
pcaac426.c102	0.024672451	0.031267921	0.431436881
lysopcac160.transhydroxyproline	0.0492669	0.062438098	0.431445063
c142.c18	-0.023766485	0.030129768	0.431589408
asparticacid.arginine	-0.047034677	0.059641568	0.431695269
pcaac342.pcaac363	-0.014664589	0.01859536	0.431699292
pcaec322.tyrosine	-0.032967456	0.041804371	0.431700955
pcaec445.methionine	0.017305996	0.02195324	0.431876329
pcaac381.c8	0.026524671	0.033649108	0.431899101
pcaec401.pcaec360	-0.002953631	0.003747403	0.431952759
c181.smohc141	0.04332444	0.054968008	0.431955675
pcaac380.c141oh	0.069321774	0.087958967	0.431991022
pcaac422.isoleucine	-0.015897533	0.020172024	0.432000853
pcaac341.pcaac281	-0.002567059	0.003257445	0.432023528
alanine.pcaac385	0.011911644	0.015116282	0.432057473
alphaaminoadipicacid.c51	0.04090163	0.051906424	0.432065114
citruline.glycine	0.039726317	0.050427286	0.432178074
creatinine.asparagine	-0.004120518	0.005231525	0.432272383
arginine.c141	-0.028723394	0.036474311	0.43235156
smohc222.c162	-0.055772024	0.070825398	0.43237406
pcaac422.pcaec401	-0.00993579	0.012619111	0.432430854
creatinine.histidine	0.032965847	0.041871536	0.432460817
pcaac341.pcaac422	-0.018944647	0.024063903	0.432487137
methioninesulfoxide.pcaac426	0.094879404	0.1205226	0.432505098
kynurenine.c101	-0.009263876	0.011769291	0.432569628
pcaac281.putrescine	0.015130017	0.01922329	0.432602103
lysopcac280.glutamine	-0.007519306	0.009554265	0.432635146
serotonin.smc261	-0.022378987	0.028443014	0.432757399
pcaac360.pcaac403	-0.007714806	0.009806446	0.432811818
pcaec403.c2	-0.06872388	0.087374958	0.432909742
lysopcac182.pcaec380	-0.026391845	0.033559034	0.43297358
c16.c182	0.062886397	0.079966496	0.432985891
pcaec445.pcaac403	-0.003428579	0.004360113	0.433019946
c141oh.c18	-0.038245441	0.048645308	0.433102079
pcaac424.pcaec385	-0.043358466	0.055153853	0.433145049
isoleucine.smohc221	-0.025793501	0.032814482	0.433201388
glycine.pcaec405	-0.031129901	0.039612971	0.43331172
pcaac381.pcaec404	0.013535732	0.017225867	0.433353637

pcaac281.kynurenine	-0.003699493	0.004708464	0.433393449
alanine.pcaec385	0.029235975	0.037209868	0.433397178
c14.pcaac281	-0.00068202	0.000868081	0.43342122
pcaac342.pcaac383	-0.005265046	0.006701673	0.433439984
pcaac420.pcaac385	-0.031751246	0.040415394	0.433445252
pcaec341.lysopcac170	0.006803573	0.008661936	0.433542311
smohc141.pcaec424	-0.008850023	0.011268337	0.433581908
pcaec384.lysopcac161	0.051031413	0.064978298	0.4335981
pcaac364.c12	0.028997048	0.036923865	0.433622008
pcaec446.pcaec402	-0.019192511	0.02444217	0.433679669
pcaac421.alanine	-0.007456987	0.009498518	0.433768954
pcaec425.pcaec421	-0.001055478	0.001344499	0.433788844
lysopcac182.lysine	0.041385815	0.052742345	0.433996489
pcaac344.pcaec384	0.022172388	0.02825783	0.434015945
pcaac323.pcaec360	0.003919149	0.004996122	0.434137264
pcaac386.pcaec445	0.053484091	0.06818605	0.434168604
pcaac343.lysopcac281	0.012081755	0.015404598	0.434220802
pcaac381.pcaec402	0.033603471	0.042846525	0.434232545
pcaac343.pcaec421	-0.003650751	0.004655107	0.434249448
pcaec422.pcaac406	0.027525541	0.035098606	0.434256325
pcaec360.c5	-0.007263839	0.009262497	0.43426464
pcaec445.pcaac300	-0.028156062	0.035905617	0.434294837
pcaac322.pcaec340	-0.036549518	0.046611363	0.434315613
asparagine.creatinine	-0.008496479	0.010836256	0.434347407
pcaec446.lysopcac160	-0.005248375	0.006694463	0.434400799
smohc161.c181	0.009933705	0.012671	0.434410099
c51.pcaac320	-0.04109665	0.052421494	0.434413605
pcaac405.pcaec301	0.041138264	0.052480342	0.434463987
smc160.lysopcac182	-0.027174474	0.034669264	0.434498585
pcaac404.pcaec322	-0.043773024	0.055847889	0.434516259
pcaac401.pcaac240	-0.014184978	0.018098471	0.434529986
pcaac403.pcaac381	0.024934304	0.031815921	0.43456591
pcaac322.pcaec382	0.008256925	0.010536992	0.434619822
pcaec383.pcaac365	-0.038558223	0.049207327	0.434635055
smc260.pcaec380	0.021672215	0.027660583	0.434682826
creatinine.pcaec362	-0.01454877	0.018569738	0.434705475
h1.c51	0.047605701	0.060767398	0.43473945
asymmetricdimethylarginine.putresci	0.032602751	0.041625328	0.434836268
lysine.lysopcac204	-0.014748369	0.018831276	0.434870379
pcaec384.c16	0.02147606	0.027422919	0.434895008
c10.histidine	0.037979117	0.048496372	0.434900605
pcaac362.pcaec343	0.051966057	0.066360925	0.434930444
smc260.c141oh	0.060678094	0.077488821	0.434945698
smohc241.pcaec360	-0.006158333	0.007865384	0.434998068
c14.pcaac404	0.001663304	0.002124492	0.435026288

pcaac406.pcaec363	-0.018662241	0.023839256	0.435074194
pcaec382.c8	0.023597593	0.030146694	0.435119646
pcaec383.pcaac421	0.011594565	0.014812871	0.435133164
pcaec383.pcaec362	0.006873486	0.008782779	0.435207132
serotonin.alanine	-0.008589631	0.010975729	0.435211294
c51dc.pcaec402	0.026989383	0.034487111	0.435216581
lysopcac281.c14	-0.031735092	0.040552169	0.435227615
pcaec340.pcaac403	0.015901004	0.020319571	0.435244137
lysopcac180.pcaac365	0.038357274	0.049022461	0.435304682
pcaec365.asparticacid	-0.019898423	0.025434065	0.435357147
pcaec343.transhydroxyproline	0.021970312	0.028086109	0.435418589
lysopcac180.putrescine	0.026295691	0.033623234	0.435523568
smc160.pcaac380	-0.035167965	0.044970823	0.435553849
pcaec403.pcaec362	0.00521373	0.006667432	0.435581739
serotonin.lysopcac180	0.062440828	0.079855404	0.435608749
methioninesulfoxide.pcaec343	0.077291076	0.098853675	0.435638068
lysopcac170.pcaac343	0.014851874	0.018999696	0.435745561
c51.valine	0.045243714	0.057881182	0.435760054
serine.c142	0.024703788	0.031607497	0.435810043
pcaac323.pcaec340	-0.013367389	0.01710393	0.435834066
asparagine.pcaec420	-0.024106999	0.03084956	0.435893628
c0.pcaec405	-0.029802283	0.038143431	0.435961521
pcaec322.creatinine	0.004497129	0.005756223	0.435995232
pcaec362.pcaac321	-0.024939397	0.031922739	0.436007891
pcaec405.asparticacid	0.03960102	0.050690719	0.436016197
pcaac422.lysopcac181	0.04633996	0.059319338	0.436035766
creatinine.pcaac343	0.034194614	0.043777973	0.43609618
pcaec340.lysopcac161	0.042494761	0.054407149	0.43612003
pcaec404.pcaac380	0.054909085	0.070311086	0.436182251
pcaec422.c8	0.026445084	0.033863413	0.436188752
lysopcac204.spermidine	-0.042255214	0.054112199	0.436219437
prolinr.c5	0.01150218	0.014733063	0.436322751
c142.pcaac386	0.009308771	0.011924721	0.436368076
prolinr.leucine	-0.013361664	0.01711753	0.436394027
prolinr.c101	0.006814193	0.008729862	0.436407144
pcaec443.pcaac421	0.010167305	0.013026512	0.436438051
citruline.lysopcac280	-0.006921563	0.008868264	0.436450823
pcaac361.c51	-0.039635429	0.050785037	0.43646958
smohc141.pcaac322	0.039331309	0.050409983	0.436602284
smc160.pcaac385	-0.015199406	0.019480715	0.436602411
pcaac424.tyrosine	-0.036337249	0.046575261	0.436628738
pcaec444.pcaec383	-0.025469219	0.032647254	0.436658017
pcaec381.ornithine	0.033432584	0.042856801	0.436677793
pcaec420.c51dc	-0.081974146	0.105091507	0.436720898
pcaec423.pcaec362	0.007813368	0.010017127	0.436735817

smohc221.c102	0.019755999	0.025332206	0.436808653
ornithine.arginine	0.012438334	0.015949124	0.436809297
c2.c141oh	-0.084671292	0.108604327	0.436952908
smohc221.c0	-0.000482692	0.000619177	0.4369887
pcaac365.lysopcac170	0.006713337	0.008614057	0.437119652
alanine.pcaac300	-0.056249154	0.072183274	0.437173718
isoleucine.pcaec405	0.039848687	0.051152186	0.437310207
pcaac424.c4	-0.014274362	0.018327137	0.437402607
pcaec385.valine	-0.048847437	0.062717128	0.437409404
pcaec402.smc160	-0.044191297	0.056747253	0.437476493
pcaac344.lysopcac161	0.055083856	0.070737968	0.437497706
pcaac421.taurine	-0.042898876	0.055098639	0.437567955
pcaec340.pcaec343	0.045996955	0.059078222	0.437571509
arginine.pcaac343	0.016907932	0.021716975	0.437582577
pcaac424.pcaec361	0.008971758	0.011524074	0.437603255
histidine.pcaec402	-0.023068619	0.029631295	0.437603824
lysopcac280.pcaec361	0.006499592	0.00835102	0.437734722
leucine.pcaec405	0.037809502	0.048593087	0.437861212
pcaac322.smc160	0.040833127	0.052482154	0.437888024
pcaac380.methioninesulfoxide	0.012674324	0.016291609	0.4379303
pcaac365.lysine	0.054720285	0.070340436	0.437948756
pcaec383.phenylalanine	-0.046895982	0.060290526	0.438008466
c102.taurine	-0.0114624	0.014736911	0.438026845
pcaac383.pcaac425	0.062460885	0.080307353	0.438043976
lysopcac182.serine	-0.008056082	0.010359778	0.438127494
alanine.pcaec380	0.029387831	0.037791765	0.438130761
pcaac322.pcaec385	0.030081841	0.038685402	0.438144498
pcaac386.methionine	-0.020013671	0.025739419	0.438175306
pcaac360.pcaac384	-0.031072388	0.039967981	0.438244334
ornithine.c16	-0.032131066	0.041330479	0.438252476
spermidine.c51dc	0.11792118	0.151725894	0.438381447
acetylornithoine.pcaac384	-0.034574708	0.044495927	0.438480185
pcaec363.c18	-0.017627417	0.022685772	0.438483487
c102.ornithine	0.013162808	0.016941295	0.438518395
lysopcac280.lysine	-0.051254249	0.065979087	0.438600834
pcaac420.ornithine	-0.013223184	0.017022331	0.438607746
smohc221.pcaec343	0.051555117	0.066368726	0.438616908
pcaec364.lysopcac170	0.005729966	0.007377145	0.438663777
pcaec382.pcaac363	-0.019887881	0.025607463	0.438707729
smc240.c14	0.01617337	0.020830123	0.438826858
c3.lysopcac203	0.020424629	0.026309671	0.438900397
lysopcac181.pcaec421	-0.001747384	0.002251021	0.438931814
pcaec404.pcaac366	-0.013612343	0.017535942	0.438937178
pcaac380.lysopcac140	0.001087455	0.001401047	0.438984796
pcaec406.lysine	-0.06023391	0.077605733	0.438996621

pcaec422.pcaac362	-0.006815059	0.008780699	0.439003653
pcaec300.pcaec321	0.056760014	0.073133353	0.439017903
pcaac341.pcaec382	-0.006964635	0.008974815	0.439074881
pcaec443.pcaac426	0.054988244	0.070879979	0.439207944
pcaac421.lysopcac204	0.029007576	0.037394082	0.439247591
pcaac300.serine	-0.026036644	0.033567126	0.439287242
pcaac281.c12	0.038003994	0.048997947	0.439307734
pcaac426.pcaec404	-0.010692427	0.013786483	0.439337735
pcaec422.lysopcac160	-0.016511277	0.021293108	0.439423095
smc241.pcaac405	-0.015604242	0.02013017	0.439576953
pcaac361.c12	-0.043476093	0.05608961	0.43960548
pcaec380.lysopcac280	0.010193358	0.013151332	0.439627082
lysopcac161.ornithine	-0.033521216	0.043260655	0.439754095
c16.smc161	-0.044075511	0.056889062	0.439815142
lysopcac203.pcaec343	0.04761094	0.061456561	0.439846754
c162.lysine	0.041162015	0.053138476	0.439900306
c141oh.c162	-0.068213605	0.088066886	0.439930962
pcaec405.c16	0.014394175	0.018583869	0.439939054
c51.c162	0.051333937	0.066280949	0.439975616
lysopcac181.creatinine	0.003530573	0.004559134	0.440031388
pcaac426.asparticacid	0.021155302	0.027320184	0.440059919
pcaec403.histidine	-0.04098343	0.052928994	0.440081935
pcaec361.pcaac365	-0.037468621	0.048393474	0.440117398
c2.pcaec343	-0.030796846	0.039776655	0.440120472
c10.pcaec401	-0.008702411	0.011240917	0.440162623
phenylalanine.pcaac380	0.06046956	0.078108706	0.440163135
pcaac322.lysopcac281	0.006848214	0.008847025	0.440223347
lysopcac181.transhydroxyproline	0.034270304	0.044279073	0.440287004
smc181.valine	0.074527667	0.096295831	0.440296884
smc161.pcaac322	0.03651184	0.047177289	0.440306653
pcaac323.lysopcac180	0.025666397	0.033168677	0.440373848
pcaec360.pcaac405	-0.025205379	0.032576065	0.440418127
c102.smc202	0.042929008	0.055486464	0.44045047
pcaec402.c8	0.024208691	0.031290229	0.440451767
acetylornithoine.asparagine	-0.00621278	0.00803025	0.440457675
alanine.lysopcac140	0.001064222	0.001375587	0.44047108
glycine.smohc241	-0.050359351	0.065097853	0.440503343
pcaec420.glutamine	-0.005316563	0.006873903	0.440593568
pcaac425.tyrosine	-0.044029097	0.056927331	0.440602587
alanine.pcaec343	-0.044922862	0.058083368	0.440606088
alphaaminoadipicacid.c182	-0.019377242	0.025054131	0.440609235
pcaec383.pcaec401	-0.0709734	0.091770293	0.440629324
pcaec360.c4	-0.01120688	0.014491553	0.440654228
serotonin.lysopcac140	0.003213728	0.004155727	0.440662243
pcaec446.ornithine	-0.011895636	0.015389364	0.440867139

lysopcac140.isoleucine	-0.01701061	0.022010749	0.440953136
c0.pcaec424	0.006315503	0.008173155	0.441023348
lysopcac182.c182	0.066589025	0.086180253	0.441047806
c141.smc161	-0.031523467	0.040801052	0.441081687
pcaec402.pcaac405	-0.028846857	0.037340836	0.441132224
c141oh.phenylalanine	-0.031008391	0.040141134	0.441158314
pcaac386.pcaac421	-0.0123599	0.016001267	0.441188768
lysopcac204.c101	-0.014177444	0.018354681	0.441198724
pcaec343.pcaec424	-0.006243815	0.008083818	0.441217167
citruline.glutamicacid	0.024949203	0.032305058	0.441266897
pcaec384.pcaac422	-0.027044707	0.035018962	0.441274388
alphaaminoadipicacid.smc261	-0.008160185	0.010567297	0.441319425
pcaac422.asymmetricdimethylarginir	0.00912917	0.011822837	0.441347219
kynurenine.histidine	0.053326276	0.069064356	0.441370634
pcaec381.smc260	-0.023025698	0.029821618	0.441376608
pcaec301.transhydroxyproline	0.019380536	0.02510147	0.441392317
c5.lysopcac204	0.03300633	0.042751225	0.441411152
pcaec342.serotonin	0.062943767	0.081540582	0.441484347
pcaac322.pcaac344	-0.061934928	0.080238365	0.441510955
smohc222.pcaac323	0.068675032	0.088971642	0.441517567
pcaac420.acetylornithoine	-0.031415565	0.040712181	0.441650564
glycine.putrescine	-0.00848508	0.010997922	0.44172942
pcaec425.pcaec365	0.022567059	0.029250259	0.441729507
asymmetricdimethylarginine.pcaac32	0.072129722	0.09349312	0.441740675
pcaec405.creatinine	0.007414401	0.009611369	0.441786574
c4.pcaec363	0.008862633	0.011488796	0.441789315
pcaac422.pcaec402	-0.036535198	0.047361679	0.441793373
pcaec423.asparagine	-0.006969413	0.009035054	0.441813269
smc161.creatinine	0.00425319	0.005514577	0.441879208
c141oh.smohc222	-0.007036846	0.009124662	0.441922459
c5.c4	0.033053423	0.042865016	0.441972647
pcaec423.lysopcac161	0.054719591	0.07096498	0.441988058
c0.c162	-0.065218393	0.084588041	0.44202753
pcaec322.pcaac425	-0.071231157	0.092389912	0.442043971
pcaec340.smohc221	-0.045714797	0.059298865	0.442080716
c0.pcaec401	-0.023355183	0.030296397	0.442099713
c142.pcaec401	-0.02501926	0.032458896	0.44215381
c162.asparagine	-0.004599895	0.005968005	0.442176809
pcaec405.pcaac380	0.0651311	0.084507198	0.442202043
phenylalanine.pcaac406	0.035621491	0.046231649	0.442329958
pcaec380.pcaac366	-0.05403293	0.070130504	0.442352148
smc160.pcaec405	-0.017906729	0.023245355	0.442427099
pcaec421.pcaac322	0.057444384	0.074573259	0.442443467
pcaec341.pcaac403	0.010435014	0.013551369	0.442605529
pcaac425.pcaac362	-0.012410574	0.016119054	0.442665852

alphaaminoadipicacid.pcaec363	-0.00902472	0.011723625	0.442750187
pcaac381.c3	0.040772571	0.052971578	0.442799094
pcaac404.smc241	0.015181354	0.019723646	0.442801074
pcaac344.pcaac300	-0.086499423	0.112384343	0.442817732
pcaec423.pcaac403	0.027311426	0.035485031	0.442826341
lysopcac280.c18	0.022031953	0.028628108	0.442867054
pcaec363.pcaec385	-0.044919491	0.058373222	0.442908112
phenylalanine.lysopcac182	-0.010657231	0.013853431	0.443048656
pcaec341.pcaec401	0.038874849	0.050535474	0.443064018
pcaac401.pcaac362	-0.01627217	0.021157487	0.443159359
serotonin.pcaec420	-0.048559099	0.063138303	0.443163198
pcaac320.pcaac420	0.054707247	0.071162774	0.443357763
c5.serotonin	0.102577506	0.133445697	0.443404305
pcaec384.c18	-0.019320649	0.025138239	0.443467876
pcaac361.lysopcac161	0.061791578	0.080409636	0.443536671
pcaec361.methionine	-0.024895066	0.032401395	0.443611678
pcaac323.pcaec380	-0.053836541	0.070074401	0.443644996
ornithine.pcaec443	-0.052494033	0.068336434	0.443707958
smc261.serine	-0.015065477	0.019612477	0.443715481
c162.arginine	0.021749875	0.028314426	0.44371699
pcaac240.kynurenine	-0.062310202	0.081120968	0.443741047
pcaac366.pcaac421	-0.056391429	0.0734178	0.443756026
pcaec422.pcaac365	0.049998168	0.065094354	0.443757035
lysopcac160.pcaac344	0.049116047	0.063951327	0.443795697
phenylalanine.c14	-0.025248516	0.032876963	0.443826781
pcaac385.pcaac365	0.029480742	0.038390471	0.443857343
pcaac341.c101	-0.004040663	0.005262135	0.443882768
lysopcac161.c142	0.021031356	0.027389312	0.443887531
pcaec380.lysopcac182	-0.018448116	0.024026583	0.443914883
pcaac403.pcaac384	0.024327406	0.031684328	0.443924
pcaac381.c5	-0.016663697	0.021706431	0.443995583
smohc241.lysopcac280	0.006863653	0.008941179	0.444019077
phenylalanine.alanine	0.013118329	0.017090678	0.444062277
acetylornithoine.pcaac240	-0.012591434	0.016406581	0.444127292
alphaaminoadipicacid.c101	0.004201401	0.005474932	0.444170948
c12.pcaec322	-0.024183128	0.031517831	0.444232987
smohc241.pcaac362	-0.006424755	0.008374757	0.444308135
pcaac342.pcaec405	-0.03510119	0.045755634	0.44431556
serotonin.pcaec364	-0.018848181	0.024570473	0.444337879
pcaec381.c101	-0.047167223	0.061502399	0.444450572
pcaec423.asparticacid	0.020888362	0.027242271	0.444541796
c5.pcaac381	0.021677755	0.028277488	0.444633449
c181.asparticacid	0.010933963	0.014264474	0.444687487
c18.smc261	0.030069985	0.039234439	0.444746151
smohc161.pcaac385	-0.013397089	0.017480291	0.444750308

methionine.smc241	0.010152165	0.013247869	0.444801718
pcaec381.pcaec321	-0.027486479	0.035870627	0.444835734
pcaec405.pcaac422	-0.07092889	0.092576075	0.444894037
ornithine.valine	0.047115417	0.061495251	0.444897111
pcaac281.pcaac420	0.04281184	0.055885347	0.444955184
asparticacid.pcaec343	0.048937218	0.063881508	0.444957101
smc241.c101	-0.007861709	0.010264926	0.445064833
kynurenine.creatinine	0.00587684	0.007673626	0.445083521
c5.c3	0.027265211	0.035603853	0.445116355
lysopcac140.creatinine	0.004089448	0.005340268	0.445127012
c16.lysopcac140	-0.000545675	0.000712599	0.445140193
c51dc.pcaec322	0.056957124	0.074382182	0.445150571
c162.smohc161	0.00301259	0.003934469	0.445176915
pcaec421.pcaac240	-0.0148727	0.0194276	0.44526401
smc260.alanine	-0.012498741	0.016330826	0.445381697
pcaac360.serine	0.03509984	0.045865886	0.445426419
smohc161.pcaac344	-0.047687782	0.062316798	0.445440387
c18.smohc221	-0.041464066	0.054192489	0.445512734
methioninesulfoxide.pcaec421	0.002942245	0.003845688	0.445542104
pcaac403.pcaec421	0.006727321	0.008793026	0.445543048
pcaec420.lysopcac180	0.034483249	0.045072304	0.445548667
glycine.pcaec446	0.035767769	0.046752884	0.445564318
pcaac403.pcaec362	0.007074811	0.009247763	0.445569868
smohc222.pcaac381	-0.02721729	0.035577511	0.44557909
glutamicacid.smc202	-0.053153271	0.069486864	0.44562303
smohc141.glutamine	-0.011761661	0.015376744	0.445647098
pcaac425.glutamine	-0.005687578	0.007435904	0.445658266
c142.pcaac364	0.025537825	0.033389637	0.445680605
pcaac323.pcaec341	0.031272994	0.040898326	0.445793792
pcaac360.acetylornithoine	-0.035174128	0.046000277	0.445794868
lysopcac180.pcaec301	0.02606931	0.034093659	0.445802152
lysopcac204.c18	-0.020111883	0.026304371	0.445834671
pcaec364.c102	0.014739425	0.0192787	0.445857705
pcaac402.pcaec446	0.056026671	0.073283255	0.445870992
smc180.pcaec444	0.059601876	0.077981788	0.445999873
smc202.pcaec343	0.047975491	0.062771138	0.446007516
pcaec386.c5	-0.004662259	0.006101255	0.446093433
pcaec401.alphaaminoadipicacid	0.010338172	0.013530205	0.44613297
pcaec424.glutamine	-0.00827267	0.010827094	0.446138808
c4.methioninesulfoxide	0.012474513	0.016326824	0.446151258
pcaec443.serotonin	-0.054998234	0.071986427	0.446176065
pcaac381.asparticacid	-0.022924244	0.030006404	0.446193896
c181.pcaec406	-0.005140609	0.006728965	0.446209316
methioninesulfoxide.asparagine	0.014599685	0.019113985	0.446286697
pcaec401.phenylalanine	-0.033216828	0.043488388	0.446294424

pcaac322.pcaac320	-0.031741175	0.041558943	0.44632193
pcaec382.c16	0.013893986	0.018193154	0.446363351
leucine.pcaec383	0.04462586	0.058435757	0.446374783
c10.pcaec322	-0.032961049	0.043163769	0.44640221
lysine.pcaac322	0.029862209	0.039110715	0.446460185
pcaec340.putrescine	0.019071225	0.024986546	0.446620851
pcaac425.prolinr	-0.027667946	0.036260156	0.446751496
pcaac365.pcaec383	-0.05433016	0.07120647	0.446778366
acetylornithoine.pcaec423	-0.027554981	0.036115222	0.446790572
pcaac366.pcaec363	0.016103441	0.021106261	0.44679314
pcaec402.acetylornithoine	-0.018884161	0.024752152	0.446816788
c182.pcaec445	-0.042200605	0.055328324	0.446935377
pcaac342.pcaac422	-0.01083477	0.01420534	0.446938597
pcaec444.tyrosine	-0.027241965	0.035720474	0.446987515
c141oh.c14	0.030666452	0.040217213	0.447060251
pcaac364.lysopcac161	-0.050634556	0.066437933	0.447290604
pcaec343.c51dc	-0.080930236	0.106211922	0.447388042
pcaac402.pcaec362	0.006343146	0.008326943	0.447511605
smc241.pcaec405	-0.015811889	0.020763246	0.447647917
pcaec404.pcaac406	0.02939857	0.038607057	0.447678192
arginine.methionine	-0.014908441	0.019581677	0.44775871
smc160.pcaec402	-0.027706044	0.03639799	0.44784762
pcaac323.c162	-0.053786142	0.070670502	0.447915318
smc181.pcaac366	-0.043468788	0.057124873	0.447998703
pcaac360.pcaac426	0.044362657	0.058299818	0.448000712
alanine.pcaec383	-0.025414168	0.033401543	0.44804346
c10.pcaec445	-0.062373811	0.081977892	0.448047461
pcaac323.c16	0.028912819	0.038001601	0.448065182
pcaec365.acetylornithoine	-0.032427142	0.04262097	0.448068518
pcaac380.pcaac321	-0.020950304	0.027539563	0.448122826
smc261.creatinine	0.005216176	0.006857421	0.448166438
arginine.c2	-0.037698112	0.049569646	0.448257875
pcaec301.pcaec340	-0.047669442	0.062687187	0.448302197
smohc222.serine	-0.012762537	0.016784404	0.448333686
pcaac240.serotonin	-0.088669557	0.116619119	0.448361119
pcaec364.pcaac385	-0.011206429	0.014739492	0.448382037
pcaec423.pcaac322	0.044687586	0.058777728	0.448393112
pcaac424.pcaec363	0.024544548	0.032288525	0.448463253
lysopcac204.smc161	-0.053332745	0.070163434	0.448487993
arginine.creatinine	0.008661744	0.011395914	0.448516082
pcaec385.smc181	-0.014563541	0.019161115	0.448526462
smohc241.c102	-0.019771268	0.026015355	0.448569667
pcaec364.pcaec343	0.058146827	0.0765141	0.448590747
lysopcac181.c4	-0.017426623	0.022934968	0.44866314
pcaac385.pcaec340	0.046764812	0.061547008	0.448666113

pcaac422.pcaec362	0.007368798	0.009698243	0.448675166
pcaec363.pcaac343	-0.0139569	0.018369312	0.448682987
lysopcac181.pcaec422	-0.027821062	0.03661715	0.448690182
pcaac424.glutamine	-0.008941473	0.011771039	0.448789059
pcaec301.asparticacid	0.041573106	0.054729305	0.448790907
pcaec403.asparticacid	0.048110468	0.063342995	0.448844635
alanine.spermidine	0.041967318	0.055271534	0.448981469
lysopcac160.alanine	0.039049007	0.051429426	0.448993332
pcaac360.pcaec322	0.076764787	0.101107878	0.449015486
pcaac401.c4	-0.010290491	0.013553814	0.449018056
pcaec405.pcaec423	0.030459004	0.040123332	0.449076292
pcaec422.pcaec421	0.003712711	0.004890802	0.449084264
c142.asparticacid	0.018380619	0.024214106	0.44910445
pcaac360.smohc241	-0.042309425	0.055739503	0.449122843
pcaac322.pcaec380	-0.036289627	0.047813512	0.449166791
pcaec425.pcaac422	-0.016446366	0.02167474	0.449287446
c2.lysopcac281	0.006835169	0.009008856	0.449325407
pcaec384.smohc241	0.044378591	0.058493856	0.449342451
pcaec360.putrescine	0.025172165	0.033179902	0.449360987
pcaac401.smc260	-0.023415411	0.030864969	0.449370907
asymmetricdimethylarginine.asparag	-0.006168876	0.008134659	0.44954721
smohc222.c5	-0.002930377	0.003864761	0.449615769
pcaac365.lysopcac160	0.024182081	0.031897411	0.449681011
asparticacid.isoleucine	-0.017800668	0.023489486	0.449863709
c5.c102	0.026622413	0.035135813	0.449931787
pcaac380.glycine	-0.054120012	0.071430945	0.44995878
c10.c5	-0.006693885	0.008835501	0.449984208
lysopcac180.creatinine	0.003780568	0.004990554	0.45002458
creatinine.c18	-0.020190143	0.026654889	0.450072242
pcaac381.c12	0.035570374	0.046985915	0.450323914
pcaac385.c12	0.033289333	0.043987539	0.450475154
pcaec443.lysopcac161	0.043224489	0.05712044	0.45051377
smc202.c14	-0.011354009	0.015006149	0.450574494
leucine.c8	-0.017710689	0.023408389	0.450591187
lysopcac203.lysopcac280	0.004996088	0.006603443	0.450595706
pcaac363.pcaec401	0.0500306	0.06612751	0.450601994
c3.isoleucine	0.008789571	0.011617592	0.450604076
pcaec406.histidine	0.036419533	0.048139377	0.450622433
c2.pcaac403	-0.003294724	0.004355334	0.450660299
c2.pcaec402	-0.026270934	0.034732813	0.450724764
pcaec402.pcaec422	-0.021004627	0.027770832	0.450734623
c0.pcaec364	0.017474392	0.023103818	0.450742695
glycine.lysopcac182	-0.015453057	0.020431557	0.450748267
pcaac386.threonine	0.01924525	0.025448744	0.45080629
pcaec342.c3	-0.018466278	0.024418869	0.450809799

smc180.creatinine	0.005625721	0.007439574	0.450834497
glycine.c142	0.031773306	0.042032311	0.450991523
histidine.pcaec446	0.065640022	0.086853727	0.451094364
pcaec341.pcaac321	-0.021522575	0.0284805	0.451129353
lysopcac281.pcaac362	0.016016459	0.021195093	0.451145407
c8.pcaec401	-0.006277233	0.008307244	0.451166106
pcaec403.c101	0.013208452	0.017480879	0.451189654
alphaaminoadipicacid.smc181	0.019969101	0.0264289	0.451199222
pcaec445.pcaac420	0.075424339	0.099843999	0.451292708
pcaac380.smc180	-0.012960994	0.017158664	0.451328817
pcaec300.pcaec301	-0.032016698	0.042398736	0.45146524
pcaec365.glutamine	-0.01094833	0.014501896	0.451569751
pcaec424.phenylalanine	-0.033851463	0.04484749	0.451656872
pcaec386.pcaec382	-0.010554309	0.013984184	0.451705361
smc261.pcaec361	-0.007646391	0.010133163	0.451789743
pcaec404.pcaac426	0.050092167	0.066393293	0.451858195
c182.pcaac320	-0.025140783	0.033330455	0.451970487
citruline.ornithine	0.027074005	0.035897521	0.452021947
pcaec380.smohc241	-0.045395277	0.060191573	0.452035523
kynurenine.pcaac365	0.03814405	0.050584183	0.452100902
lysopcac180.pcaec364	0.009312635	0.012349941	0.452105428
pcaac402.glutamine	-0.010065558	0.013348588	0.452110798
c181.pcaec421	-0.000658264	0.000873	0.452128196
smohc222.tyrosine	-0.044623264	0.059180134	0.452128613
h1.asparticacid	-0.007785987	0.010329184	0.452271696
pcaac323.pcaec401	-0.017803819	0.023619699	0.452280891
citruline.pcaac364	-0.024608514	0.032649032	0.452305561
acetylornithoine.pcaac362	-0.005003458	0.006638546	0.452324082
h1.pcaec343	0.035715753	0.047389684	0.452346163
serotonin.asymmetricdimethylarginir	-0.019696131	0.026134465	0.452355193
pcaac365.pcaec445	-0.031849135	0.042267675	0.452436238
pcaec404.pcaec422	-0.042757076	0.056746171	0.452454779
c141.pcaec406	-0.029256634	0.03882897	0.452457835
c14.lysopcac140	-0.000553854	0.000735217	0.452550118
pcaac424.smc202	-0.047251207	0.062727542	0.4525761
smohc222.c102	0.015715413	0.02086729	0.452674665
kynurenine.lysopcac160	0.012889845	0.017115933	0.452687767
c8.pcaac321	0.013162767	0.017481533	0.45277026
pcaec406.pcaec340	-0.046082301	0.061205124	0.452792393
pcaec402.pcaac362	-0.006398458	0.008499788	0.452874581
pcaec425.pcaac366	-0.007965433	0.010581428	0.452876787
alphaaminoadipicacid.c16	0.027390881	0.036386782	0.452879633
pcaec365.histidine	0.040902104	0.054340492	0.452921446
c4.valine	0.052124496	0.069284923	0.453148755
pcaac385.smc180	0.027380582	0.036395395	0.453155906

alphaaminoadipicacid.pcaec424	-0.012838431	0.017067155	0.453203045
smohc241.pcaac365	0.039519438	0.052542699	0.453257485
putrescine.asymmetricdimethylargini	-0.00842779	0.011205975	0.453293106
pcaac341.lysopcac140	0.000739997	0.000984064	0.453352888
pcaac342.pcaec342	-0.06720585	0.08937238	0.4533557
smc160.c14	-0.017614769	0.023425129	0.453364812
smc241.smc260	-0.024990896	0.033239754	0.453438431
pcaac404.pcaac426	0.045362928	0.060351999	0.453557375
c182.pcaac300	-0.018233863	0.024260807	0.453594697
pcaac381.c181	0.020675881	0.02751218	0.453630413
arginine.pcaac364	0.030468664	0.040554868	0.453763853
asparticacid.smc261	0.04805429	0.063966609	0.453796609
kynurenine.pcaec421	0.001166439	0.001552894	0.453857529
pcaac320.alanine	-0.007403376	0.009856351	0.453864241
lysopcac180.pcaec445	0.037904557	0.050468192	0.453905695
pcaac380.pcaac344	-0.029453034	0.039215558	0.453907807
pcaac401.prolinr	-0.017786048	0.023681526	0.453909751
lysopcac140.pcaac320	-0.044508153	0.059261938	0.453916016
pcaec405.pcaec401	-0.023156561	0.030838176	0.453997291
creatinine.pcaec342	0.043182909	0.057519893	0.454092234
alphaaminoadipicacid.methioninesul	0.009725261	0.012955686	0.454147202
smohc141.pcaec340	0.063560873	0.084680272	0.45418165
pcaec321.pcaac320	0.081566185	0.10871377	0.45437039
glutamicacid.pcaac422	0.023880735	0.031829929	0.454384457
pcaac406.pcaac321	-0.013124125	0.017494312	0.454424427
c14.pcaec445	-0.04867347	0.064883278	0.454438882
c102.lysopcac140	0.001356471	0.0018083	0.454459117
pcaec384.asparagine	0.008136579	0.010847304	0.454479855
pcaac406.c141	-0.015183235	0.020245762	0.454572984
pcaac365.pcaec386	0.01098134	0.014643453	0.454591989
pcaac425.c8	0.031580176	0.042115989	0.454637915
c51.creatinine	0.00496752	0.006625504	0.454686535
c18.pcaac422	0.024084668	0.032123925	0.454695424
pcaac402.smc161	0.083178258	0.110950416	0.454727738
c0.c4	0.011081169	0.014783965	0.454817122
pcaec380.pcaac381	0.027450931	0.036624866	0.454831228
creatinine.smc240	0.0244742	0.032657138	0.454883712
smc180.pcaac281	0.001495736	0.001996022	0.454925963
lysopcac281.smc261	0.032070047	0.042806714	0.455031574
pcaac403.pcaac404	-0.012353047	0.016493848	0.455172037
c0.pcaac361	-0.030169105	0.040282989	0.455183842
pcaec365.pcaec380	0.038796114	0.051810928	0.455260372
lysopcac180.pcaac343	0.009223685	0.012319122	0.455304057
pcaec422.pcaec362	0.007117844	0.009506957	0.455322455
c8.pcaac403	-0.001829386	0.002444538	0.455527959

pcaac401.smohc221	-0.071961849	0.09616896	0.455570554
smc161.pcaec405	-0.017454494	0.023326462	0.45557988
pcaac344.pcaec363	0.017842428	0.023847588	0.455630511
pcaac425.acetylornithoine	-0.037251137	0.049797922	0.455714586
pcaac364.c18	-0.027903525	0.037307224	0.455778963
smohc161.smohc141	0.130860577	0.174981911	0.455831212
c4.c3	0.083372396	0.111485203	0.455842207
pcaec342.pcaac321	-0.016382995	0.021911575	0.455930571
lysopcac182.c162	-0.050031318	0.06692457	0.45599615
pcaec446.lysopcac140	0.001501291	0.002008596	0.456083263
pcaec342.pcaac421	0.010827362	0.014487151	0.456116936
pcaac362.serine	-0.008041212	0.0107617	0.45621947
isoleucine.c51dc	0.092103666	0.123272741	0.456251292
c14.arginine	0.020169205	0.026994966	0.456255279
smc160.creatinine	0.004306178	0.005763515	0.456256841
pcaac361.pcaec422	0.009949997	0.013317379	0.456257199
pcaec422.arginine	0.0387829	0.051918073	0.456342508
serotonin.pcaac421	0.007886747	0.010561861	0.456512481
smc260.pcaec385	-0.021403374	0.028663607	0.45651875
c101.lysopcac203	-0.004854501	0.006502148	0.456584672
putrescine.pcaac321	-0.008782593	0.01176582	0.456674801
c101.pcaac422	0.00746864	0.010006287	0.456707926
lysopcac161.glutamicacid	0.032784987	0.043927435	0.456738313
asymmetricdimethylarginine.lysopca	0.007097766	0.009511436	0.45680411
pcaac281.c3	-0.014354183	0.019236003	0.456816448
methionine.c51dc	0.072003763	0.096494435	0.456827524
pcaec446.glutamine	-0.012425592	0.016652374	0.45683995
methionine.pcaac240	0.013139829	0.017609948	0.456849521
pcaec444.pcaec364	0.012240416	0.016404662	0.456852399
smc241.serine	-0.01601503	0.021464373	0.456872381
lysopcac182.pcaac380	0.032920977	0.044128029	0.456925516
pcaec321.pcaac366	-0.005055763	0.006776868	0.45692607
citruline.pcaac405	0.018964239	0.025421953	0.456958297
glutamicacid.c101	0.009307524	0.012482231	0.457149142
kynurenine.c51	-0.037362335	0.050114232	0.45722051
smc240.lysopcac203	-0.007232554	0.009702345	0.457280433
pcaec444.lysopcac161	0.032110428	0.043076667	0.457291765
lysine.pcaac240	0.008437532	0.011319977	0.457327028
pcaec404.pcaac422	-0.059968111	0.08047374	0.457434247
creatinine.pcaec405	-0.031449107	0.042207245	0.457480636
pcaec300.glutamicacid	-0.028485396	0.038232282	0.457510886
smc241.pcaec420	0.010060303	0.013502926	0.457520062
pcaec423.lysopcac280	0.0066432	0.008917661	0.45757876
pcaac320.pcaac344	-0.053795013	0.072215843	0.457596173
smc261.pcaec446	-0.013024234	0.017484759	0.457613717

c18.taurine	-0.023959371	0.032167238	0.457645665
c182.methionine	-0.020976294	0.02816889	0.457751715
c16.smohc222	-0.007305111	0.009811339	0.457814236
pcaac403.tyrosine	-0.038877257	0.052215355	0.457815424
arginine.glutamine	-0.004680175	0.006286361	0.457851146
pcaec384.glycine	0.019559952	0.026275784	0.457903541
lysopcac281.pcaac323	0.084009095	0.112855012	0.457910409
pcaec401.pcaac424	-0.00573786	0.007709029	0.457967434
arginine.pcaec340	-0.035862689	0.048183241	0.457970969
c141.pcaac343	-0.016073815	0.021596181	0.457975967
pcaac320.pcaac362	-0.005807068	0.007803788	0.458069536
c14.pcaec321	-0.039220766	0.052718669	0.458172637
pcaac361.pcaac364	0.03495417	0.046991621	0.458248115
smohc141.kynurenine	-0.002977694	0.004003546	0.458292885
smc241.pcaec444	0.064755871	0.087067382	0.45830482
smohc222.pcaec385	-0.034115232	0.045880885	0.458415453
pcaac404.smc202	0.03128136	0.042097733	0.458714522
glycine.pcaac425	-0.039515329	0.05318279	0.458747999
putrescine.pcaec401	0.01061472	0.014286273	0.458753035
c3.pcaac322	0.024250222	0.032640695	0.45878741
smc181.pcaec422	-0.039423693	0.053064981	0.458794653
smc261.c162	0.046297374	0.06231926	0.458810365
lysopcac161.spermidine	-0.04730961	0.063682975	0.458818674
pcaec364.creatinine	-0.023294252	0.03136023	0.458876997
c5.pcaac422	0.024317072	0.032739185	0.45890394
pcaec422.ornithine	-0.021274457	0.028643407	0.458913854
smc180.smc160	-0.070529582	0.09496034	0.45891878
asparticacid.smohc141	-0.023517901	0.031664566	0.458922944
pcaec405.pcaec362	0.007955192	0.010711734	0.458958189
pcaac421.pcaac240	-0.012990384	0.017495185	0.45904841
pcaec446.putrescine	-0.029374537	0.039561644	0.459055383
pcaec403.pcaec445	0.044688577	0.060186779	0.459056698
pcaac424.pcaac362	-0.012273184	0.016534368	0.459186514
pcaec321.pcaac386	-0.009217716	0.012419383	0.459234349
ornithine.pcaec424	0.002946572	0.003970499	0.459287205
pcaac365.phenylalanine	0.016467879	0.022195068	0.459380961
pcaec403.smc260	-0.024488529	0.033010969	0.459460165
smc202.lysopcac281	-0.007650549	0.010314888	0.459538891
c4.asparticacid	-0.023646323	0.031900637	0.459811036
glutamicacid.c142	0.037930123	0.05117173	0.459821467
c10.pcaec384	-0.026389845	0.035603801	0.459835579
pcaac401.smc161	0.060142454	0.08114596	0.459862619
pcaac323.pcaac421	0.01846513	0.024914653	0.459879854
c4.smc161	0.063550363	0.085765621	0.459975473
pcaac365.pcaec422	0.018504312	0.024974466	0.460004422

pcaac321.alphaaminoadipicacid	0.007251842	0.009787508	0.460004958
pcaec424.tyrosine	-0.028785351	0.038858758	0.460101454
pcaac425.pcaec362	0.006464663	0.00872957	0.460234999
pcaec364.glutamine	-0.009946404	0.013434291	0.460339838
c0.pcaec382	0.004569353	0.006171702	0.460341494
pcaec340.pcaec383	-0.048413859	0.065408097	0.460456498
citruline.c12	-0.024814922	0.033528624	0.460498756
smc180.pcaac364	0.029192884	0.039457985	0.460658565
pcaec421.transhydroxyproline	0.027007411	0.036505458	0.460676032
pcaec404.c51	-0.063183534	0.085406349	0.460687771
asparagine.pcaac321	-0.01374648	0.018586443	0.460809898
pcaec405.pcaac362	-0.009886161	0.01336727	0.460820468
pcaec362.smc240	-0.01926435	0.026051889	0.460892403
c8.lysine	0.041011319	0.055462069	0.460900101
pcaac404.pcaec301	0.040191236	0.054371876	0.46105526
c0.putrescine	-0.018926321	0.025604552	0.461063524
pcaac366.lysopcac280	0.006922313	0.009364937	0.461066259
c51dc.smc261	0.032173696	0.043530306	0.461104493
c51.asparticacid	0.010913647	0.014766373	0.461118041
c2.smc260	0.012778035	0.017290881	0.46116865
pcaac321.lysopcac182	-0.01330538	0.018004498	0.46116937
pcaec421.pcaec362	0.00686815	0.00929439	0.461197573
pcaac380.pcaec383	0.052493469	0.071040885	0.461220189
c8.pcaac323	0.077932702	0.105469416	0.461224016
pcaec404.pcaec322	0.030440427	0.041198561	0.461249247
pcaec425.pcaec362	0.00706901	0.009568272	0.461294192
pcaac321.valine	0.040549931	0.054887891	0.461306007
smc260.c4	0.015770881	0.021353794	0.461442614
smc161.c51	-0.028842772	0.03905838	0.461502468
pcaac321.asparticacid	-0.023584185	0.03193882	0.461523843
c101.pcaac384	0.02377829	0.032203219	0.461545117
c102.c12	-0.040208041	0.054458672	0.461581639
pcaac300.pcaec401	0.046413267	0.06287404	0.461658938
c18.lysopcac281	0.014599754	0.019782478	0.461767993
c18.smc180	-0.008804652	0.011931718	0.461825236
pcaec406.alanine	-0.00857215	0.011616864	0.461833839
pcaec300.pcaec363	0.031592672	0.042815672	0.461851599
c5.pcaec364	-0.024849954	0.033677755	0.461852771
c16.ornithine	-0.017039732	0.023094173	0.461875546
pcaac425.smc160	-0.037114208	0.050304161	0.461900284
smc240.lysopcac204	0.019441973	0.026352668	0.461921442
pcaec445.glutamine	-0.016956891	0.022985449	0.461944593
serotonin.pcaac364	-0.02876069	0.039008301	0.462202832
smc240.asparagine	-0.004859357	0.006591076	0.462223183
pcaec404.pcaec340	-0.034433061	0.046713572	0.462315685

pcaac323.pcaac426	0.048444521	0.065724204	0.462329293
pcaac404.pcaac341	0.005476548	0.007431233	0.46240467
lysopcac182.lysopcac140	0.006169453	0.00837211	0.46243996
pcaec363.c102	0.014212159	0.019289607	0.462517169
pcaac425.ornithine	-0.022583159	0.030655398	0.46257779
pcaac384.pcaac422	-0.029798405	0.040455685	0.462643897
pcaec364.pcaac420	0.023086422	0.031343205	0.462644141
pcaec401.pcaac320	-0.076635647	0.104044209	0.462644445
c10.c101	0.012895754	0.017508747	0.4626663
pcaac364.glutamine	-0.005203801	0.007065691	0.462692684
c10.pcaac404	0.002885353	0.003918281	0.462757035
lysine.pcaac323	0.059065936	0.080220464	0.462809996
pcaec381.pcaec384	-0.027278033	0.037051929	0.462861089
putrescine.lysopcac281	-0.008670536	0.011783693	0.463105469
pcaec300.smohc141	0.023349165	0.031737292	0.46317023
pcaec342.pcaec364	-0.010468093	0.014229031	0.463179707
pcaec405.lysopcac170	-0.006677128	0.00907666	0.463209138
pcaec362.pcaec383	-0.043058683	0.058536759	0.463241576
h1.lysopcac180	0.073081018	0.09935988	0.463281185
pcaac402.pcaec361	0.006476664	0.008806467	0.463326277
pcaec322.pcaac365	-0.035889055	0.048803608	0.463366932
acetylornithoine.tyrosine	0.034710101	0.047207245	0.46343153
pcaec365.smc161	-0.037451525	0.050937945	0.463451197
pcaac364.pcaac343	-0.01247706	0.016970647	0.4634658
pcaac401.pcaec385	-0.037857789	0.051495236	0.463492184
c101.c18	-0.020490429	0.02787556	0.463554605
smohc221.c16	0.019888864	0.027059021	0.463584966
pcaac362.pcaac321	-0.015976693	0.021737541	0.463606951
c5.pcaac384	-0.029814399	0.040567039	0.463631339
c16.lysine	0.040717927	0.055403152	0.463632946
c141oh.c16	0.025360763	0.034508194	0.463644339
pcaec384.pcaac421	0.016832926	0.02290497	0.463654837
pcaac320.smc160	0.043088025	0.058632133	0.463664061
pcaac380.smc261	0.022859843	0.031115495	0.463791805
lysopcac140.prolinr	-0.024687216	0.033604981	0.463820652
pcaec361.creatinine	0.003198727	0.004354908	0.463892841
pcaac344.pcaec403	0.046922793	0.063890476	0.463944635
smc160.serine	-0.011729559	0.01597284	0.463994125
glutamine.c101	-0.00668821	0.009108161	0.464015053
pcaec385.pcaec403	-0.024116302	0.032846784	0.464077999
pcaec363.arginine	-0.027803018	0.037869146	0.46408973
pcaac300.pcaac366	-0.010503165	0.014306581	0.464112422
pcaac426.pcaac406	0.051516823	0.070172926	0.464117409
smohc221.putrescine	0.017736368	0.024162253	0.464171019
lysopcac204.pcaec342	-0.044214413	0.060235181	0.464185008

c0.pcaec406	0.005891062	0.008025685	0.464187221
pcaec322.alphaaminoadipicacid	0.017657059	0.024055669	0.464198056
c182.c3	0.051267512	0.069847404	0.464207258
pcaec425.histidine	0.0501541	0.068343267	0.464290696
glutamine.c141oh	0.052073489	0.070968394	0.464351263
c16.pcaec423	0.026962682	0.036746533	0.464356379
pcaec341.ornithine	-0.025285828	0.034463756	0.464389381
pcaac321.lysopcac204	-0.007097766	0.009674023	0.464389428
kynurenine.c4	-0.01095711	0.014934532	0.464399795
asymmetricdimethylarginine.pcaec3(	0.032078156	0.043733213	0.464509021
pcaac422.pcaac384	0.026132086	0.035627536	0.464519006
c18.kynurenine	0.002692684	0.003671736	0.464595247
isoleucine.pcaac383	-0.006647222	0.009065796	0.464677122
smc180.smc161	0.056605475	0.077207455	0.46471306
pcaac405.pcaec380	0.02687668	0.036666501	0.464808545
asparagine.c18	0.027087882	0.036957259	0.464840187
c4.pcaec364	-0.01803764	0.024616972	0.464973799
lysopcac181.smc260	-0.020970495	0.028624628	0.465052028
asymmetricdimethylarginine.pcaec3ξ	0.028371665	0.038733031	0.465119194
c5.smc180	-0.020779838	0.028370189	0.465143205
smc240.pcaac421	-0.017255537	0.023559285	0.465157098
lysopcac170.pcaec445	0.014736919	0.020121718	0.465182471
alanine.lysopcac204	-0.01191506	0.016268773	0.465182685
c101.lysopcac182	0.025092341	0.034263457	0.465214928
asymmetricdimethylarginine.pcaec4(	-0.019904574	0.027183602	0.465280692
pcaac420.isoleucine	-0.010030212	0.013699389	0.465318603
c16.pcaec402	-0.021693694	0.029635442	0.465407631
smc181.smc161	0.056788323	0.077580392	0.465423034
pcaec406.pcaec365	-0.015332819	0.020954912	0.465598265
smohc141.lysopcac161	0.041154742	0.056245145	0.465599626
pcaec406.tyrosine	0.039267041	0.053677353	0.465699854
glutamine.pcaac421	-0.005361234	0.007329166	0.465727399
pcaac385.pcaac406	0.018181482	0.024861864	0.465844887
pcaac300.putrescine	0.015510966	0.021212123	0.465886795
pcaec422.pcaac405	-0.049174642	0.067260687	0.465963445
pcaac343.serine	-0.012950258	0.017716501	0.466044856
arginine.putrescine	0.027408407	0.037510282	0.466215971
smc181.pcaac405	-0.047628714	0.065189676	0.466260473
glycine.smc202	-0.030922911	0.042324582	0.466262876
pcaac422.pcaec380	0.071026948	0.097216442	0.466267241
c12.tyrosine	0.024415158	0.033419573	0.466292645
pcaac364.lysopcac204	-0.008654723	0.011848299	0.466355653
spermidine.isoleucine	0.052923404	0.072482334	0.4665416
pcaec300.tyrosine	0.025789485	0.035328572	0.466642999
ornithine.pcaec382	-0.005717685	0.007833577	0.466700857

pcaec381.c162	0.057637191	0.078985343	0.466807144
smc240.pcaac402	0.04859924	0.066612432	0.466891216
smc241.alanine	-0.006659306	0.009127795	0.466902562
acetylornithoine.c182	-0.05218056	0.071528247	0.466935271
smc202.pcaec342	0.046102922	0.063199557	0.466952406
pcaec300.pcaec444	0.072526074	0.099423169	0.46696045
pcaac403.pcaac421	0.022503812	0.030849938	0.46696533
c101.pcaec301	-0.028068163	0.038481514	0.467006237
pcaec364.pcaec341	0.066075836	0.090611377	0.467110547
lysopcac161.pcaec402	-0.019532756	0.026789797	0.467177948
pcaec381.c102	-0.03164933	0.043409615	0.467193821
pcaec422.pcaac320	-0.073408253	0.100685538	0.467194625
pcaec300.smc261	0.034373774	0.047150005	0.467227705
smc202.pcaac426	-0.033620221	0.046127048	0.467330637
c182.pcaec421	-0.000929005	0.001274711	0.467370156
lysopcac140.smc160	0.044146904	0.060583132	0.467429253
pcaec444.c101	-0.018847292	0.025875012	0.467613557
smohc241.isoleucine	0.010165932	0.013959455	0.467705266
pcaac403.pcaac366	-0.016540493	0.022715591	0.467760855
serine.pcaec401	0.040247075	0.055284962	0.467860182
pcaec382.pcaec301	0.062191071	0.085434852	0.467895309
pcaac385.pcaec301	0.042071666	0.057820273	0.468082851
pcaec420.c4	-0.023746427	0.032636144	0.468093231
pcaac404.pcaac366	-0.010023055	0.013775325	0.468094414
lysopcac161.pcaac380	0.029417238	0.040430487	0.468099884
pcaac384.c5	-0.003092125	0.004250131	0.468138983
pcaec381.pcaac385	-0.014939839	0.020535004	0.468142634
lysopcac161.pcaec423	0.021880393	0.030078058	0.468189435
smohc141.transhydroxyproline	-0.014121375	0.019420857	0.468390558
acetylornithoine.c162	0.050208556	0.069058546	0.46843994
pcaac383.alphaaminoadipicacid	0.002759261	0.003795293	0.468453081
pcaac323.glutamicacid	0.030482501	0.041930486	0.468480413
lysopcac160.pcaac240	-0.011873382	0.016333237	0.468499369
pcaec322.glutamicacid	0.039993934	0.055022517	0.468548928
pcaac405.asymmetricdimethylarginin	-0.012495357	0.017192551	0.468595252
pcaac425.pcaec382	0.007488663	0.010304383	0.468621849
pcaac425.pcaac424	-0.005583834	0.00768394	0.468656368
c142.pcaac404	0.001443311	0.001986487	0.468732397
smc202.creatinine	0.003980398	0.005478504	0.468742203
h1.lysopcac204	0.029232252	0.040235506	0.4687542
pcaac321.pcaac343	-0.025414994	0.034984748	0.468796658
pcaec401.pcaec301	0.044527376	0.061294548	0.468802783
c141oh.lysopcac182	-0.015800889	0.021751074	0.468807328
pcaec365.pcaac406	0.029338312	0.04039307	0.468881463
pcaac403.lysopcac140	0.001249253	0.001720216	0.468943769

lysopcac180.lysopcac203	-0.008567	0.011798278	0.469002127
pcaec321.pcaac362	-0.002933978	0.004040835	0.469027139
pcaec420.pcaac343	0.032320296	0.044532089	0.469214475
pcaec420.pcaec364	0.008839678	0.01217979	0.46922024
pcaec446.pcaec343	-0.042126516	0.0580567	0.469315641
pcaac421.lysopcac170	0.025176306	0.034702015	0.469382917
lysopcac204.pcaac344	0.03976749	0.054814102	0.469384388
h1.pcaec423	0.039728367	0.054791786	0.469640277
lysopcac182.putrescine	0.035589808	0.04908428	0.46964236
c12.histidine	0.034589392	0.047710447	0.469697251
pcaac360.c101	-0.013973	0.019273927	0.469707441
c51.pcaec401	-0.011239658	0.015504983	0.469745898
lysopcac170.serine	-0.040357222	0.055694368	0.469921251
pcaac240.smc202	-0.044793067	0.061825614	0.469990263
pcaac421.pcaac420	-0.156257747	0.215686557	0.470014633
pcaac425.pcaec322	-0.041183799	0.056847064	0.470014748
pcaec402.pcaac366	-0.010339981	0.014273188	0.47003474
pcaec340.pcaec403	-0.007049073	0.00973121	0.470069083
pcaac342.c0	-0.000333703	0.000460719	0.470111763
pcaac281.pcaac360	-0.031711156	0.043782866	0.470127478
pcaec404.lysopcac203	0.009905854	0.013677158	0.470139545
arginine.pcaec362	0.008770956	0.012110292	0.470143362
lysopcac181.pcaac300	-0.027153326	0.037494504	0.470181016
smohc241.serine	-0.011876655	0.016400463	0.470198742
pcaec401.glutamine	-0.004613292	0.006371086	0.470240116
pcaac365.c102	0.022173645	0.030622473	0.470240795
pcaac361.pcaec444	0.058426927	0.080724715	0.470434929
c0.pcaac321	0.020440962	0.028246085	0.470499663
pcaac381.pcaac361	-0.035915858	0.049637622	0.470568784
lysopcac160.c14	0.022089729	0.030539235	0.470714653
pcaec386.methioninesulfoxide	0.008717016	0.012051956	0.470737051
c182.pcaec406	-0.004656012	0.006437322	0.470738496
smohc221.pcaec341	0.033521009	0.046351936	0.470799325
histidine.c16	0.025443263	0.035192734	0.470931262
c18.pcaac366	-0.031387695	0.043417579	0.470957742
pcaac386.histidine	0.027399472	0.037901789	0.470969305
lysopcac160.pcaac281	-0.003011157	0.004166342	0.471075407
asparagine.lysopcac280	0.005655298	0.007825487	0.47111032
glutamine.c16	-0.023220919	0.032138082	0.471196649
pcaac380.c102	0.019915381	0.027564513	0.471218205
smohc141.lysopcac180	0.046140064	0.063874051	0.47130438
pcaac365.putrescine	0.031787911	0.04400674	0.471315522
smohc241.pcaac323	-0.053566801	0.07418651	0.471490808
c14.serotonin	0.024891678	0.034475114	0.471513549
pcaac321.pcaac426	0.051499695	0.0713296	0.471527388

lysopcac181.pcaec380	-0.023870437	0.03307451	0.471698448
c0.pcaac383	-0.005339155	0.007397875	0.471699873
h1.acetylornithoine	0.053002257	0.073439638	0.471701621
c51.lysopcac170	-0.005297728	0.007340798	0.47171941
pcaec424.lysopcac204	-0.018792251	0.026040063	0.471729177
pcaac366.prolinr	0.029619243	0.041048587	0.471791347
leucine.tyrosine	-0.048601771	0.06735643	0.471794108
pcaac424.pcaec362	0.00717233	0.009941057	0.471840284
pcaac406.arginine	-0.02494647	0.034578166	0.471861205
pcaec363.pcaec401	0.049125333	0.068095015	0.471878488
pcaec382.smc260	-0.027379934	0.037954246	0.47189697
alanine.c101	0.006974019	0.00966899	0.471968283
lysopcac170.pcaec401	-0.039665724	0.054995668	0.471983661
pcaac342.asparagine	0.004185964	0.005804369	0.47203099
pcaac385.c5	-0.004725133	0.006553171	0.472110326
c4.h1	-0.000896442	0.001243381	0.47215548
c141oh.pcaac421	-0.007386928	0.010245963	0.472162724
creatinine.c12	-0.012429227	0.017240511	0.472180091
asymmetricdimethylarginine.pcaec42	0.002019929	0.002801878	0.472187427
lysopcac161.pcaec445	0.069337723	0.09618088	0.472193488
pcaac422.pcaec383	0.038390789	0.053253446	0.472195021
prolinr.acetylornithoine	0.030142434	0.041815434	0.472233316
pcaac360.c14	-0.06061864	0.084096032	0.472244537
pcaac425.smohc141	-0.01044804	0.01449762	0.472338788
pcaec361.c162	-0.052480896	0.07282442	0.472352934
pcaac403.c51dc	-0.096968373	0.134574111	0.47240954
kynurenine.pcaac403	-0.01040513	0.014445294	0.47255962
c141.smc240	0.018500054	0.025686437	0.472612548
asparagine.pcaac360	-0.017760083	0.024659285	0.472617235
smohc141.serine	-0.008924381	0.012392291	0.472655925
smc241.lysopcac182	-0.028467073	0.039529391	0.472659791
isoleucine.pcaec420	-0.026809224	0.037228703	0.472676434
pcaac343.pcaac426	0.055791688	0.077476744	0.47268478
asparticacid.glycine	-0.010014344	0.013907025	0.472694793
c8.ornithine	0.023327437	0.032399346	0.472753313
c3.glycine	0.015876669	0.022051095	0.472754885
pcaec443.pcaac404	-0.008535976	0.011858968	0.472879971
asparticacid.pcaac322	0.015415746	0.021418817	0.472917824
c8.smohc141	0.034994888	0.048627988	0.472969468
c142.pcaac422	0.011590538	0.016109169	0.473058834
pcaac426.tyrosine	0.040614976	0.056459546	0.47314186
acetylornithoine.c18	-0.032181423	0.044753562	0.473315951
lysopcac203.smc202	-0.032753313	0.045549859	0.473325547
pcaec385.lysopcac280	0.006199448	0.008622007	0.47334952
isoleucine.lysopcac170	-0.005506364	0.00765815	0.473353192

c51dc.c4	-0.009653466	0.013426037	0.473358857
pcaec363.pcaec403	0.029022531	0.0403725	0.473446088
pcaac322.c2	0.046907154	0.065257396	0.473487054
pcaac381.pcaac404	-0.006134586	0.008534505	0.473489543
pcaec405.asparagine	0.011124036	0.015477307	0.473530091
smc181.pcaec385	-0.030236164	0.042071769	0.473561699
lysopcac280.pcaec382	-0.005047316	0.007024381	0.473646502
pcaac281.pcaec365	0.015569785	0.021669395	0.473663445
pcaac364.c14	0.024322325	0.033852637	0.473687075
pcaec384.tyrosine	-0.044072519	0.061346155	0.473719657
c18.c101	-0.002009544	0.002797544	0.473780485
c2.pcaac385	-0.021937571	0.030542663	0.47382014
acetylornithoine.smohc241	-0.042706626	0.059463296	0.473856085
smc241.lysopcac181	0.034056167	0.047425198	0.473916726
phenylalanine.c101	0.007774022	0.010825872	0.473920483
c12.pcaec385	-0.031745045	0.044216538	0.474013657
pcaec446.pcaac361	0.03650336	0.050860073	0.474151182
arginine.serine	0.032410922	0.045159615	0.474166124
pcaec402.pcaec425	-0.022320245	0.031102534	0.474204688
asparticacid.lysopcac281	-0.008977842	0.012510878	0.474223996
methioninesulfoxide.c2	0.095415138	0.132968908	0.474241264
pcaec301.pcaec401	0.015700406	0.02188696	0.474385233
c5.tyrosine	0.066316651	0.092468009	0.474481067
pcaac420.lysopcac160	-0.013313958	0.018567242	0.474553477
pcaec365.pcaac405	-0.018712454	0.026099333	0.47461281
pcaac342.h1	0.001135477	0.001583778	0.474630391
lysopcac281.smohc141	0.019761318	0.027563631	0.474635
pcaec404.pcaec342	0.048085047	0.067098737	0.474821597
pcaac386.pcaac424	-0.009734958	0.013585823	0.474869893
pcaec421.glutamine	-0.006025949	0.008410289	0.474903983
prolinr.pcaec403	0.029513202	0.041192682	0.474922527
asymmetricdimethylarginine.pcaac34	0.020507093	0.028627161	0.474994026
pcaec382.pcaac385	-0.016629	0.023213504	0.474994426
glutamicacid.smohc161	0.010504761	0.014665088	0.475018698
pcaec406.pcaec403	-0.010549518	0.014731751	0.475143789
pcaec404.c16	0.012906762	0.018024587	0.475170479
glycine.pcaac405	0.010460258	0.014608774	0.475194182
arginine.pcaec365	-0.012757015	0.017819321	0.475265917
pcaac420.lysopcac182	-0.015136133	0.02114317	0.475279191
pcaac366.pcaac402	-0.032697459	0.045674298	0.475281928
alphaaminoadipicacid.lysopcac182	-0.013232685	0.018486886	0.475340701
pcaec322.pcaac424	-0.007391999	0.01032861	0.475405777
pcaec382.pcaac366	-0.011941529	0.016687538	0.475458933
c3.c162	-0.06143096	0.085848236	0.475470836
kynurenine.smohc141	-0.021357649	0.029847679	0.47548408

h1.ornithine	-0.038586137	0.05392895	0.4755181
pcaec341.arginine	-0.025626288	0.035822175	0.475594763
pcaec445.lysopcac160	-0.004444987	0.006213548	0.47559777
smc241.pcaac385	-0.008967689	0.012536076	0.475609841
c5.creatinine	0.00967131	0.01352065	0.475641465
pcaec365.pcaec382	-0.006338845	0.008862624	0.475681897
ornithine.creatinine	0.006120091	0.008558693	0.475780722
lysopcac181.pcaec402	-0.015496276	0.021672074	0.475804602
spermidine.pcaac281	-0.006947028	0.009715796	0.475810865
pcaec361.pcaac386	-0.018598052	0.026010762	0.475817244
pcaac320.pcaac300	-0.055991045	0.078321895	0.475897355
pcaec405.lysopcac203	0.015468748	0.021638957	0.475914135
pcaac424.pcaec424	0.026056134	0.036454973	0.475980677
lysopcac180.pcaec341	0.022543263	0.031542869	0.476018832
pcaec446.pcaac381	0.013424251	0.018784102	0.476034991
pcaac344.smc261	0.023877538	0.033419179	0.47614232
c141oh.citruline	-0.02139724	0.029948127	0.476148058
h1.tryptophan	-0.022409858	0.031367503	0.476177399
pcaac323.pcaac344	-0.029079795	0.040708123	0.476226958
lysopcac140.pcaec385	0.022217903	0.031105343	0.476269714
pcaec384.c14	-0.024801722	0.034722979	0.476272984
pcaec301.pcaac323	-0.038188901	0.053471165	0.476320965
pcaec365.pcaec322	0.057678673	0.080762952	0.476335594
pcaac361.taurine	0.032232448	0.045138528	0.476393529
pcaec421.lysopcac170	-0.029915301	0.041898744	0.476447777
smc181.pcaec424	-0.009647673	0.013512483	0.476452797
lysopcac181.pcaac323	-0.063914272	0.089520878	0.476466888
asparticacid.smc260	0.023254014	0.032575589	0.476535647
leucine.c3	0.016363222	0.022922702	0.476538325
lysopcac181.serotonin	-0.062489269	0.087544292	0.476564065
asymmetricdimethylarginine.pcaec32	-0.031709648	0.044432443	0.476651582
pcaec423.smc240	-0.028992663	0.040631592	0.476719425
pcaec301.smc261	-0.021199806	0.029714883	0.476786698
pcaec361.pcaac426	0.045363202	0.06358666	0.476807124
lysopcac281.c12	0.031999145	0.04485766	0.476843514
pcaec343.lysopcac203	-0.01659234	0.023260404	0.476855044
smohc241.creatinine	0.005204996	0.007297598	0.476905655
c12.lysine	0.03533213	0.049537222	0.476907916
pcaac381.pcaec342	0.061012442	0.085548314	0.476939543
arginine.pcaec405	0.032425519	0.045465796	0.476944424
asparagine.lysopcac180	0.02967913	0.041628128	0.477084028
pcaec403.pcaac365	-0.032808287	0.046017835	0.477091019
pcaac381.glutamine	-0.009003858	0.012629597	0.47710949
lysine.leucine	0.015128658	0.02122359	0.477167787
pcaec381.pcaac320	-0.050067091	0.070245087	0.47721351

lysopcac203.c16	0.0378912	0.053166025	0.477246141
pcaac381.ornithine	-0.019232563	0.026985712	0.477247055
pcaec445.pcaac363	0.005948352	0.008346509	0.477258655
smc180.putrescine	0.01502484	0.021082453	0.477261794
pcaac405.pcaac240	-0.007927194	0.011124033	0.477293684
leucine.smc202	0.032506538	0.045617158	0.477308581
pcaec321.pcaac422	-0.008616179	0.012093272	0.477381091
serine.pcaec363	0.014428179	0.020252956	0.477429452
pcaec301.prolinr	-0.014380568	0.020187179	0.477452426
glycine.histidine	0.021213758	0.029780363	0.477465356
c102.pcaac300	-0.063574623	0.089262548	0.477539355
methioninesulfoxide.pcaec361	0.003548864	0.004983541	0.477603333
c10.lysopcac170	-0.015919248	0.022357437	0.477654704
pcaac404.pcaec402	-0.020443097	0.028712498	0.477679819
smc161.pcaac240	-0.005965922	0.008379188	0.477679903
asparagine.pcaac366	0.014556529	0.020446672	0.477720828
asymmetricdimethylarginine.aspartic	0.01466028	0.020595155	0.477779521
pcaec422.lysopcac140	0.000855684	0.001202108	0.477786851
lysopcac281.pcaec422	-0.011763072	0.016527579	0.477845977
pcaac406.pcaec386	0.008738762	0.012279231	0.477879106
pcaac402.serine	0.068031089	0.095595034	0.477885863
lysine.methioninesulfoxide	0.010633008	0.014941502	0.477896193
lysopcac140.smc241	0.01253376	0.017612488	0.477897422
c18.pcaec301	-0.015396687	0.021639703	0.477983105
pcaac402.pcaac425	0.098992322	0.139133335	0.477988822
smohc222.smc180	-0.035574698	0.050003887	0.478022058
pcaac386.smc241	0.016652919	0.023409884	0.478068949
smohc241.c14	0.018219485	0.025613341	0.47809044
pcaac363.pcaac383	-0.00507397	0.007134254	0.478161769
smohc141.putrescine	0.014820227	0.020841503	0.478236117
pcaac420.pcaec380	0.034703646	0.048805752	0.478258091
pcaac386.pcaac364	-0.026610196	0.037427287	0.478302965
c0.c101	0.010970838	0.015432021	0.478346154
pcaac300.ornithine	-0.014503091	0.020400691	0.478347501
pcaec343.pcaec383	-0.036484943	0.051322333	0.478355987
pcaac406.valine	-0.030170785	0.042440661	0.478358834
pcaac342.creatinine	0.004487917	0.006313841	0.478412736
asymmetricdimethylarginine.c4	-0.010257483	0.014430996	0.478419501
pcaac425.pcaec401	0.019811343	0.027876775	0.478493397
pcaec403.pcaac421	0.011419431	0.016071261	0.478571119
c102.serine	0.011263186	0.015854809	0.478666392
pcaac401.lysopcac161	0.047442281	0.066784776	0.478678702
smc240.pcaec380	0.028992011	0.040815336	0.478712175
pcaac366.putrescine	0.037363373	0.0526026	0.478728459
pcaac341.alphaaminoadipicacid	0.003629999	0.005110704	0.478741699

c182.pcaec362	0.009257607	0.013034677	0.478769257
isoleucine.methioninesulfoxide	0.012573675	0.017705108	0.478804485
pcaec404.pcaac362	-0.007554686	0.010638509	0.478832766
smc181.pcaec340	-0.020699529	0.029150282	0.478850919
c10.tyrosine	0.041564533	0.058539125	0.478892368
pcaec301.smohc161	0.003941721	0.005552084	0.478939615
pcaac385.pcaac421	-0.01991726	0.028056625	0.478975763
serotonin.pcaac361	0.030208619	0.042557813	0.479018794
pcaac342.smohc222	-0.004330076	0.00610117	0.479088708
asparagine.c141	-0.0252387	0.035563442	0.479108084
pcaec401.pcaec444	0.097826764	0.137869495	0.479182537
pcaec401.pcaac322	0.042022061	0.059223477	0.479188657
pcaac420.pcaac281	0.001170649	0.001649952	0.479217003
lysopcac170.smc180	0.006148826	0.008666538	0.47922594
pcaac320.asparagine	0.003341291	0.004710205	0.479298798
c5.lysopcac170	-0.018988323	0.026769308	0.479323992
glutamicacid.pcaac426	0.075758382	0.10680259	0.479324565
lysopcac204.threonine	0.022717725	0.032031756	0.479390036
pcaac366.pcaac425	-0.076389221	0.107709042	0.479394242
pcaec382.h1	0.002126018	0.002997864	0.479419461
c181.c101	-0.003741082	0.005276691	0.479540064
smc202.pcaac384	-0.024388763	0.034406099	0.479622055
lysopcac181.c141oh	0.0496896	0.070109957	0.479691253
pcaac300.pcaec364	-0.009043635	0.012760859	0.479714159
pcaac420.pcaac381	0.026658794	0.037616817	0.479718867
citruline.pcaac426	-0.027836431	0.039278557	0.479719294
pcaac365.lysopcac204	-0.010737459	0.015151378	0.479728024
c4.pcaec444	-0.033234134	0.04692067	0.479959423
pcaec386.ornithine	-0.01278521	0.018050628	0.479964094
pcaac342.lysopcac280	0.00832987	0.011762374	0.480037046
pcaac281.pcaec343	0.047529578	0.067116831	0.480047881
pcaac426.glutamine	-0.004574865	0.006460455	0.480065351
h1.pcaec321	-0.009725232	0.013734566	0.480095806
lysopcac140.pcaac300	-0.032693224	0.046172657	0.480108067
c5.pcaec424	0.013293121	0.01877646	0.480168269
pcaec401.pcaec380	-0.053507921	0.0755835	0.480190615
pcaac300.smc160	0.031343346	0.044299321	0.480435594
pcaec380.lysopcac170	-0.005434921	0.007682436	0.480490068
pcaec422.pcaec423	0.069009829	0.097581906	0.480644013
pcaac426.pcaec405	-0.030630367	0.043320304	0.480725659
pcaec382.pcaec402	-0.102123532	0.144435595	0.480734848
c10.smc161	-0.031074729	0.043953144	0.480769321
citruline.pcaec444	0.064052041	0.090629033	0.48092237
pcaec425.pcaec401	-0.017635866	0.024954009	0.480931604
pcaac403.pcaac362	-0.009710225	0.013740623	0.48096547

c12.pcaec422	0.021399765	0.030285298	0.481011561
pcaec340.asparagine	0.003746525	0.005302428	0.481035093
pcaac365.pcaac386	-0.017728775	0.025097928	0.481148954
pcaac321.pcaac404	0.00220669	0.003124088	0.481172083
c101.smc260	0.01728434	0.02447339	0.481231818
lysopcac161.threonine	0.012934806	0.018316038	0.481262322
c0.pcaac381	0.013148421	0.018620641	0.481312111
h1.pcaec446	-0.040887969	0.057905565	0.481315895
pcaec424.pcaec340	-0.026328023	0.037292463	0.481394561
pcaac380.smohc141	-0.023745715	0.033636017	0.481411173
pcaac362.pcaac426	0.028438735	0.040284692	0.481421723
lysopcac160.pcaac426	-0.040225683	0.057004621	0.481599923
pcaac300.pcaac422	-0.024462257	0.034666025	0.481600809
pcaac300.lysopcac182	-0.010334011	0.014644722	0.481605549
smc181.c5	-0.002941505	0.004168768	0.481631587
asparticacid.pcaac362	0.004989868	0.007072745	0.481692929
pcaac402.pcaec363	0.019972705	0.028330002	0.482005911
pcaac384.pcaac343	-0.013554592	0.019228258	0.482049999
pcaac381.lysopcac161	0.035476832	0.050328611	0.482066744
smc240.pcaac343	0.012944922	0.018364923	0.482086421
pcaac363.pcaac381	-0.03554483	0.050432118	0.482127814
pcaec365.ornithine	-0.016203367	0.022991254	0.482154614
pcaec380.pcaec383	0.074884362	0.106262224	0.482185202
lysopcac204.glutamine	-0.006718211	0.009534095	0.482223536
pcaec402.h1	0.001681479	0.002386285	0.482228742
pcaec443.smc181	0.004842059	0.006871877	0.482243136
smc161.tyrosine	-0.035582944	0.050502307	0.482267328
smc241.valine	0.047030781	0.066757568	0.482316625
pcaec403.tyrosine	-0.03424264	0.048612041	0.482375313
pcaac424.putrescine	-0.051116329	0.072575031	0.482426606
pcaac406.pcaec382	-0.004677867	0.006641827	0.482438752
c2.c4	-0.021380704	0.030359366	0.482470001
smc161.smohc222	0.007489823	0.010635333	0.482478883
smohc221.pcaec362	0.024419555	0.034677782	0.482513158
smc160.putrescine	0.018665092	0.026506138	0.48251594
pcaac361.serine	-0.009719112	0.013804262	0.482586662
pcaac343.glycine	-0.059585451	0.084646872	0.482671266
pcaec321.pcaec385	-0.027850463	0.039572353	0.482760597
pcaac422.c18	-0.023426582	0.033288416	0.4827855
c8.asymmetricdimethylarginine	0.005584816	0.007935949	0.482791284
smc260.putrescine	0.009537257	0.013554617	0.482865547
h1.lysopcac161	0.025503032	0.036246794	0.482879641
pcaac343.c16	0.020436212	0.02905012	0.482949797
pcaec421.pcaec401	0.030761327	0.043738664	0.483063373
c102.kynurenine	-0.003344727	0.004755856	0.483070989

smc180.c102	0.015629894	0.022224149	0.483072084
pcaec405.pcaac402	-0.06724316	0.095616573	0.483088134
pcaec386.pcaac322	0.031581683	0.04491531	0.483162633
pcaec406.c16	0.032484037	0.046200887	0.483183933
c14.spermidine	0.037777517	0.053730335	0.483189789
pcaac421.pcaac366	-0.012268911	0.017451366	0.483227271
serine.pcaec445	0.027645953	0.039326647	0.483259391
pcaac240.asymmetricdimethylarginir	-0.021157253	0.030103984	0.483369344
pcaec446.pcaac422	-0.009559762	0.013603828	0.48341898
smc202.transhydroxyproline	0.016331614	0.023242348	0.483455997
pcaec321.lysopcac204	-0.01276255	0.018163341	0.483463426
pcaec383.glutamicacid	0.057378287	0.081665289	0.483495233
pcaec385.pcaec444	-0.051302491	0.073017959	0.48349655
c18.pcaac361	0.031186723	0.044389941	0.483520253
asparticacid.pcaec422	-0.027901285	0.039717058	0.483558506
pcaac406.pcaac362	0.029024233	0.041331686	0.483728873
leucine.lysopcac182	-0.016386245	0.023335331	0.483741099
lysopcac160.creatinine	0.003161987	0.004503087	0.483756928
glutamicacid.pcaec423	0.026283453	0.037432119	0.483768698
pcaac383.pcaac366	-0.032553645	0.046377541	0.483915513
c51dc.c142	-0.027239544	0.038811209	0.483964981
pcaac341.creatinine	0.003988598	0.005683026	0.483967021
pcaec420.transhydroxyproline	0.012049852	0.017170225	0.484002108
arginine.kynurenine	-0.018145598	0.025857194	0.484017998
asparagine.smc160	0.044512193	0.063437415	0.484074647
pcaec423.lysopcac170	0.007650301	0.010903374	0.484090648
pcaac366.c12	0.035388794	0.050440414	0.484121255
pcaec380.smc261	0.024613685	0.035082791	0.484125893
pcaec421.pcaec361	0.006305404	0.008987615	0.484140005
pcaac281.pcaac240	-0.007973168	0.011369261	0.4843105
c18.pcaac343	0.010288485	0.014673109	0.48438022
pcaec425.pcaac362	-0.004369719	0.006232013	0.484384399
pcaac405.pcaac365	-0.027320059	0.038964365	0.48439555
pcaec341.pcaac385	-0.017019101	0.024276655	0.484462086
c8.pcaec342	0.039416867	0.056225813	0.484463502
pcaec383.pcaec423	0.017144223	0.02445693	0.484494115
pcaec363.c51	-0.035717302	0.05095589	0.484525865
c0.c2	-0.042479653	0.060605208	0.484539155
pcaac383.alanine	0.01472292	0.021008236	0.484606078
pcaac360.lysopcac280	0.004756395	0.006790089	0.484808799
pcaac366.lysopcac161	0.040956847	0.058473797	0.48484614
pcaac380.c51dc	-0.068156165	0.097311707	0.484871398
pcaac321.pcaec340	-0.047104711	0.0672575	0.484887892
smc241.h1	0.00195887	0.002798134	0.485075071
glutamicacid.pcaec403	0.023627482	0.033753413	0.485112561

pcaec386.pcaec402	0.027816012	0.039739278	0.485137501
lysopcac160.pcaec402	-0.012950267	0.018503946	0.485198232
pcaac342.c101	-0.011025906	0.015757504	0.485286018
pcaec381.pcaac321	0.008434015	0.01205345	0.485289909
smohc161.lysopcac140	-0.00041531	0.000593544	0.485294236
pcaec340.taurine	0.034254255	0.048957496	0.485317646
pcaec402.pcaac425	0.058455941	0.083561531	0.485390938
pcaac420.pcaec406	0.009063322	0.01295739	0.485443473
phenylalanine.lysopcac204	-0.016875186	0.024132803	0.48557295
pcaac300.glutamicacid	-0.02420623	0.034620342	0.485618068
c51dc.pcaec363	0.017425646	0.024923329	0.485631073
c51dc.pcaac366	-0.013413949	0.019186441	0.485651744
pcaec383.pcaec340	-0.032326069	0.04624072	0.485685755
smohc241.serotonin	0.049699036	0.071098359	0.485725777
smc161.serine	-0.017382511	0.024867573	0.485735081
smc261.c51	-0.025127423	0.03594893	0.485752254
pcaec425.pcaec424	0.026314101	0.037653684	0.485833392
pcaac240.pcaac405	-0.031826013	0.045543441	0.485858112
smohc161.c101	-0.00996042	0.014254399	0.485885868
lysopcac160.pcaec341	0.024009757	0.034361217	0.485895362
glycine.pcaec383	-0.02832521	0.040538853	0.485912966
pcaec420.smc240	0.013792638	0.019740363	0.485922454
lysine.serotonin	0.064877415	0.092861622	0.48595748
asparticacid.smc161	0.037758126	0.054054788	0.486038682
smc240.smc241	0.008712949	0.012477069	0.486162636
pcaec381.pcaac421	0.011083566	0.015873509	0.48620874
smc261.c181	-0.011668455	0.016716171	0.486339042
pcaac240.pcaec423	0.027030915	0.038725199	0.486348513
pcaec340.pcaec301	-0.029872964	0.042801006	0.486391346
alanine.smc241	0.009465665	0.013564246	0.486460329
c16.pcaac380	0.038390845	0.055016502	0.486481154
pcaec342.transhydroxyproline	0.0183155	0.026254605	0.486602777
pcaac426.methioninesulfoxide	-0.035058792	0.050258882	0.486631988
pcaac320.phenylalanine	-0.028709401	0.041157784	0.486643975
isoleucine.smc160	-0.024987261	0.035827042	0.486708532
pcaec402.smc240	-0.020906816	0.029976982	0.486716239
glutamine.lysopcac204	-0.011163352	0.0160096	0.486802257
methionine.c101	0.005589888	0.008018479	0.486905441
pcaac361.pcaec300	0.011370332	0.016314763	0.487024299
pcaac240.pcaec405	-0.049210965	0.070610723	0.487025434
pcaac424.pcaac403	0.011237482	0.016124699	0.48703927
pcaac366.smohc241	-0.035726644	0.051279015	0.487164248
pcaec443.pcaac422	-0.051246232	0.073566265	0.487233633
pcaac363.serine	-0.00868695	0.012472322	0.487296912
kynurenine.pcaac322	-0.03290232	0.047240959	0.487309121

pcaac385.pcaec362	-0.015705432	0.022550979	0.487332458
pcaec444.pcaac366	-0.004529848	0.006505064	0.487384934
pcaec406.glutamine	-0.007768871	0.011157718	0.487434509
pcaac344.creatinine	0.01657881	0.023815893	0.487530607
c51.lysopcac182	0.009201682	0.013220119	0.487585275
smohc141.pcaac321	-0.010642465	0.015290365	0.487592744
pcaac365.lysopcac203	-0.014338881	0.020603689	0.487646847
pcaec380.pcaac402	-0.034268656	0.049249708	0.487723763
pcaec322.c4	-0.008793994	0.012639232	0.48775172
pcaec403.pcaac321	0.005727193	0.008231464	0.487752442
pcaac321.pcaac422	-0.045867923	0.065926047	0.487765141
alphaaminoadipicacid.smc161	0.028084499	0.040366385	0.487770362
c12.pcaec382	-0.008860833	0.01273615	0.487780808
pcaec402.pcaac321	0.011745611	0.016884077	0.487818894
histidine.c3	0.034352939	0.049386608	0.487862509
lysopcac280.asparagine	0.005367167	0.00771603	0.487866026
c2.c14	0.050870913	0.073135169	0.487873943
lysopcac140.pcaac403	0.004403005	0.00633054	0.487908821
c181.pcaac425	0.029146416	0.041908828	0.487937571
smohc141.lysopcac281	-0.013934631	0.02003793	0.487974581
pcaac406.lysopcac170	0.004393256	0.006319471	0.488111465
pcaac322.pcaac383	-0.003967211	0.005707549	0.488181616
lysine.pcaec365	-0.010343313	0.014883001	0.488248002
pcaec322.pcaac323	-0.034876418	0.050188132	0.488286319
pcaec403.transhydroxyproline	0.047243516	0.067998766	0.48837595
pcaac421.glutamine	-0.006949099	0.010002893	0.488414227
smc260.smc160	-0.045249252	0.06513694	0.488433054
smc161.putrescine	0.017603952	0.025342323	0.488453344
ornithine.pcaac343	0.007635335	0.010992994	0.488504927
c5.arginine	-0.042279314	0.06088864	0.488625331
serine.c51	-0.028134112	0.040527784	0.488736534
c141.pcaec446	-0.016158903	0.023285793	0.488895894
pcaac341.lysopcac204	-0.007630974	0.010997188	0.488918431
c4.pcaac361	-0.041556867	0.059888878	0.48892009
ornithine.pcaec445	0.022291411	0.032129457	0.488982473
pcaac381.lysopcac140	0.000845346	0.001218445	0.488988413
pcaac342.pcaac366	-0.007904497	0.011393377	0.488994862
lysopcac281.pcaec401	0.018327139	0.026417606	0.489015386
pcaac420.pcaac380	-0.064691171	0.093250958	0.489024882
pcaec365.pcaac425	0.045057135	0.064958522	0.489089076
putrescine.c16	-0.020704829	0.02985684	0.489188575
phenylalanine.pcaac383	-0.006430397	0.009275195	0.489301483
pcaac343.ornithine	0.034040361	0.049103134	0.489331223
pcaac406.c16	0.01924347	0.027761598	0.489377155
pcaec301.pcaec341	-0.035215643	0.050806263	0.489397685

smohc222.spermidine	0.035917074	0.051823035	0.489437875
pcaec404.pcaec403	-0.045554843	0.065740774	0.489516102
pcaec386.pcaec365	-0.019299574	0.027851689	0.489519655
pcaec423.pcaec363	0.01565895	0.022597832	0.489519977
c5.pcaec301	0.076817337	0.110874902	0.489589715
c102.pcaec322	0.046741931	0.067470949	0.48962581
pcaac344.smohc141	0.031255761	0.045119872	0.489653369
smc260.pcaec362	-0.010557853	0.015245438	0.489779616
pcaec380.c141oh	0.048944489	0.070676581	0.489787048
smc261.pcaac406	0.033489485	0.048360748	0.489799838
pcaec385.serotonin	0.060685746	0.087650614	0.489883375
smc202.pcaac421	0.018626491	0.026904254	0.489904953
creatinine.pcaec404	0.016482353	0.023811165	0.489976323
smc180.pcaec403	-0.019518856	0.02820088	0.490023093
pcaac404.glutamine	-0.004237435	0.00612301	0.490076611
pcaac341.c141oh	0.056796393	0.082076888	0.49011468
smc180.pcaac360	0.037068152	0.053569744	0.490133146
smc161.pcaec384	-0.057433144	0.083008407	0.490173893
pcaac363.tyrosine	0.039303221	0.056805991	0.49018038
pcaec425.creatinine	0.005272654	0.007622688	0.490292969
glutamine.pcaec301	-0.030051268	0.04344916	0.490332567
pcaec381.smc161	0.058121352	0.084074237	0.490540597
pcaec365.c4	0.01985033	0.028716421	0.490575827
pcaec444.kynurenine	0.002808218	0.00406346	0.490678123
pcaec301.c102	-0.020352668	0.02945074	0.490687785
pcaac421.lysopcac182	-0.050490504	0.07308097	0.49080712
pcaac320.pcaac405	0.03156232	0.045691076	0.490874753
smohc161.serine	-0.015043725	0.021779155	0.490897837
asparticacid.pcaac366	0.008083802	0.011703721	0.490920412
c101.pcaec420	0.022425814	0.03247685	0.491037399
pcaec403.pcaac380	0.040076278	0.058039909	0.491051011
pcaec422.tyrosine	-0.031339617	0.045389353	0.491071891
serotonin.alphaaminoadipicacid	0.005865248	0.008495712	0.491124782
smohc141.pcaac323	0.06389775	0.092555194	0.491126618
asymmetricdimethylarginine.creatinin	0.003219099	0.004663028	0.491145081
c2.pcaec420	0.012442337	0.01802398	0.491159993
alanine.glutamine	0.010123006	0.014665643	0.491202721
lysopcac281.smohc222	-0.003596888	0.005213274	0.491394011
pcaec301.pcaac344	0.048937315	0.070944998	0.491491583
c2.c18	-0.038224557	0.055418232	0.491520022
smohc161.alanine	-0.017186334	0.024920915	0.491590253
c141.pcaec365	-0.017194297	0.024935593	0.491644566
ornithine.phenylalanine	-0.031579703	0.045797731	0.491645202
lysopcac181.pcaac362	-0.009752807	0.014143974	0.491651099
pcaec405.c0	-0.001180186	0.001711875	0.491730633

lysopcac140.pcaac404	0.004696632	0.00681255	0.491732066
pcaec424.pcaec401	-0.036654876	0.053180122	0.491826046
c141.lysopcac280	0.006632034	0.009621989	0.491826584
h1.pcaac383	-0.006032552	0.008752493	0.491839123
pcaac404.spermidine	0.03663264	0.053158367	0.491911486
pcaec446.pcaac385	-0.027874226	0.040451642	0.491941278
glutamicacid.pcaac363	-0.009236339	0.013403996	0.491942374
c102.lysopcac170	0.013593748	0.01972892	0.49197189
pcaac402.pcaac362	-0.008457647	0.012276014	0.492015426
pcaec421.smc260	-0.016416274	0.023828229	0.492024706
pcaac401.pcaac424	-0.007156847	0.010389183	0.492066946
pcaac380.creatinine	-0.006170862	0.008959313	0.492135867
phenylalanine.pcaec340	0.025815803	0.037485117	0.492179993
pcaac323.pcaec362	0.012407778	0.018017835	0.492215178
pcaec365.c8	-0.027294319	0.039637905	0.492244861
pcaec423.pcaec445	0.027288151	0.039638607	0.492350165
c4.tyrosine	0.037333873	0.054237524	0.492402434
pcaec404.pcaec421	-0.005120255	0.007440621	0.492522613
smc240.c51dc	-0.060606416	0.088087288	0.492599192
pcaec301.tryptophan	-0.023672216	0.0344111	0.49266383
pcaac421.c3	-0.017111648	0.024874897	0.492673592
pcaec322.pcaac343	0.024551645	0.035702209	0.492817803
pcaec402.pcaec362	0.009829365	0.01429579	0.492885645
pcaec343.pcaec365	0.016970696	0.024684361	0.492924915
c8.pcaec384	-0.01241752	0.018063312	0.49296496
leucine.asparagine	0.005197717	0.007561608	0.493003777
pcaec321.creatinine	0.007214413	0.010496044	0.493026743
pcaec321.pcaac424	-0.01527016	0.022219018	0.493083005
pcaac365.pcaec401	0.037942789	0.055210158	0.493091429
asparticacid.c51dc	0.074666935	0.108711047	0.493345442
lysopcac160.serine	-0.023135383	0.033687759	0.493395188
pcaac322.pcaec301	-0.039041693	0.056856992	0.49345461
pcaac420.smc261	0.01929863	0.028109169	0.493520469
pcaac320.pcaec425	-0.010271671	0.014964507	0.493619581
c5.smc261	-0.041684839	0.060732253	0.493639403
smc202.pcaec322	-0.07005674	0.10207662	0.493674257
lysopcac203.pcaac321	-0.021754005	0.031698053	0.493691146
pcaac320.pcaac385	-0.021963653	0.032004093	0.493698683
pcaec361.asparticacid	0.019608011	0.028575269	0.493754137
pcaec380.pcaec421	0.005358445	0.007811531	0.493893721
c101.c5	-0.012019714	0.017522908	0.493908313
c18.pcaac322	-0.034317922	0.050034406	0.493943776
smohc222.lysopcac204	-0.017823455	0.02599151	0.494034961
pcaec365.pcaec301	0.050300219	0.073352821	0.494042238
c8.lysopcac180	-0.038754315	0.056522189	0.49409383

pcaec360.lysopcac140	0.000876752	0.001278752	0.494104715
smc260.pcaec322	-0.046552886	0.067906302	0.494157915
asparticacid.pcaec380	0.050807574	0.074113578	0.494163721
smohc141.pcaec402	-0.031443337	0.045877678	0.49426642
pcaac300.asparagine	0.003511959	0.005124623	0.494305802
pcaec444.c162	-0.036900297	0.053846606	0.494321684
serotonin.c4	-0.010856128	0.015843612	0.494372069
c2.creatinine	-0.004634233	0.006764759	0.494466549
pcaec363.pcaac344	0.046596581	0.068034241	0.494564802
citruline.pcaac320	-0.035227349	0.051434632	0.494566969
smc181.pcaec362	0.011207159	0.016369744	0.494736272
pcaec443.pcaec342	0.050319461	0.073517966	0.494846524
pcaac342.c141oh	0.048456577	0.070799409	0.494865758
c142.pcaac385	0.004437796	0.006484555	0.494901424
pcaac402.lysopcac140	0.001490635	0.002178145	0.494903941
pcaac365.c162	-0.043301973	0.063283162	0.494968488
pcaac381.pcaac422	0.028792244	0.042081311	0.495001463
c181.c5	-0.003684174	0.0053859	0.495104918
lysopcac180.c16	0.023013885	0.03366137	0.495326745
pcaec423.pcaac424	-0.005948355	0.008701652	0.495389152
c101.serine	0.006165608	0.009020487	0.495437952
pcaac364.c8	-0.02533252	0.037064888	0.495467924
pcaac380.serine	0.032621733	0.047739238	0.495551311
pcaec386.creatinine	-0.012211343	0.01787157	0.495581864
pcaec363.pcaac321	-0.017972805	0.026303812	0.495585367
c12.lysopcac203	-0.007973419	0.011669914	0.49560544
alanine.pcaac364	0.028852482	0.042229506	0.495615103
pcaec322.pcaec403	-0.006932527	0.010147756	0.495660268
pcaec381.pcaec301	0.050600528	0.074110631	0.495905001
citruline.pcaac383	0.009649527	0.014133162	0.495912781
pcaac341.pcaec363	-0.01670345	0.024465075	0.495919748
lysopcac180.c51	-0.036542066	0.053528339	0.495969586
lysopcac204.smc160	0.036944321	0.054118176	0.495974334
methioninesulfoxide.glutamine	-0.019594635	0.028706798	0.496025953
smc240.asparticacid	0.012647317	0.018532527	0.496113753
pcaec362.c14	-0.020986009	0.030756724	0.496186994
pcaec363.glycine	-0.021506294	0.031523058	0.496239012
lysopcac160.glycine	-0.035686072	0.052313976	0.496294684
pcaec402.pcaec421	0.002795955	0.004099026	0.496325859
pcaec363.pcaac386	0.009139323	0.013401012	0.496398231
c16.c0	-0.000578574	0.000848407	0.496419971
pcaac343.pcaec405	-0.035211745	0.051634554	0.496427233
pcaac366.pcaac403	0.005928531	0.008693818	0.49643768
pcaec385.threonine	0.017132597	0.025126925	0.496489857
pcaac365.lysopcac161	-0.058776825	0.086210306	0.496526387

pcaac383.pcaac403	0.002714655	0.00398174	0.49653153
c162.smc261	-0.014158278	0.020766908	0.496534637
pcaec364.h1	-0.001300059	0.001907904	0.49676445
pcaec300.smc202	-0.019833477	0.029114798	0.49688443
glycine.leucine	0.012199926	0.01790941	0.496893515
smc180.lysopcac161	0.038109146	0.055954882	0.496977272
pcaec341.glycine	0.024816282	0.036439235	0.497000779
ornithine.pcaac300	0.020264778	0.02976455	0.49712434
c141.pcaac420	0.038724374	0.056887548	0.497198854
pcaec321.asparagine	0.003336327	0.004902424	0.497307166
smohc161.creatinine	0.003915526	0.00575362	0.497315791
pcaac403.lysopcac160	0.030113277	0.044250343	0.497323257
smohc161.pcaac380	-0.031536093	0.046343494	0.497345267
arginine.pcaac300	-0.026625803	0.039129214	0.497362604
c4.c102	0.029509913	0.04337196	0.497404822
pcaec445.serotonin	0.074460067	0.109438199	0.49740917
pcaec381.lysopcac181	-0.040714639	0.059841262	0.497413756
pcaac406.pcaac384	-0.024831222	0.036498462	0.497439772
pcaec300.lysopcac203	0.009931893	0.014599267	0.497462055
smc261.asymmetricdimethylarginine	0.013693178	0.020128914	0.497478949
histidine.pcaec425	0.002575906	0.003786608	0.497483202
c3.methioninesulfoxide	0.007376136	0.010843941	0.497520583
pcaec363.pcaac425	-0.054243601	0.079750519	0.497547063
isoleucine.pcaec380	0.02180759	0.032064701	0.497581184
asymmetricdimethylarginine.smc160	-0.051478978	0.075703997	0.497649747
pcaec365.pcaec403	-0.01501204	0.022076914	0.497659378
pcaac364.pcaac403	-0.002520249	0.003707462	0.497792426
pcaec362.serine	-0.011237265	0.016531331	0.497806191
pcaac405.c5	-0.007208771	0.010605242	0.497818216
pcaac403.pcaac323	-0.064726941	0.095238882	0.497887222
pcaec382.pcaac404	-0.009784263	0.014399338	0.497971381
pcaac404.glycine	0.058571098	0.086202451	0.497992973
pcaac344.pcaac380	-0.020088717	0.029567598	0.498020224
pcaec380.smc181	0.006210493	0.009142429	0.498090989
pcaac300.lysopcac281	0.006532379	0.00961649	0.498100646
smc260.transhydroxyproline	-0.016467264	0.024245918	0.498171703
pcaac363.pcaac322	-0.042498888	0.062576917	0.498190875
leucine.creatinine	0.006520303	0.009600964	0.498201254
pcaac300.pcaec420	0.028186434	0.041508351	0.498248896
lysopcac182.serotonin	-0.05751782	0.084756765	0.498522029
pcaac421.pcaec362	0.008939857	0.013173778	0.498529753
pcaec401.pcaac366	0.018545285	0.027331829	0.498584449
pcaec365.pcaec362	-0.014894309	0.021951226	0.498587635
pcaec386.pcaac424	-0.006822634	0.010056283	0.498634062
smc240.c51	-0.031504498	0.046436901	0.498639212

citruline.pcaec404	-0.005907843	0.008712007	0.498835221
pcaec300.pcaac362	-0.009845963	0.014520101	0.498857117
pcaec443.c4	-0.020098754	0.029641449	0.498875712
pcaec365.lysopcac203	-0.008672065	0.012790807	0.498920253
smc261.c101	0.005921948	0.008736261	0.49900467
pcaec386.smc240	0.023880336	0.035236393	0.499093503
pcaec386.pcaec446	-0.038409498	0.056683315	0.499158208
pcaac342.c102	0.039148119	0.057798665	0.499345906
pcaec382.pcaac323	-0.037369578	0.055173686	0.499352714
lysopcac181.c18	-0.020046527	0.029597666	0.499357171
smc241.pcaac240	-0.00620992	0.009169176	0.499382765
ornithine.pcaac323	0.036471363	0.053862381	0.499470793
pcaac380.pcaac384	-0.023068824	0.034084423	0.499664971
smohc161.pcaec424	-0.005786552	0.008550816	0.499721362
isoleucine.smohc241	0.028899426	0.042717187	0.499845302
c102.pcaec362	0.013019967	0.019252289	0.500002071
putrescine.pcaec443	0.03748274	0.055436072	0.500089394
smc181.smohc222	-0.004139007	0.006122212	0.500139687
pcaac406.lysopcac181	0.011223816	0.01660184	0.500143175
pcaac406.phenylalanine	0.015622546	0.023111388	0.500200795
lysopcac204.isoleucine	-0.014793349	0.02188634	0.500232747
alanine.pcaec361	0.005132396	0.007594725	0.500316776
pcaec300.c51	-0.044673133	0.066106271	0.50032102
alphaaminoadipicacid.pcaac363	-0.013704574	0.020281638	0.500361646
prolinr.c18	0.035465659	0.052487979	0.50037576
pcaec422.pcaac385	-0.020041449	0.029669677	0.50050565
lysopcac204.pcaec382	-0.005193935	0.007691253	0.500620803
glycine.pcaac424	-0.004123299	0.00610588	0.50062354
pcaec383.pcaec301	0.045894849	0.067967617	0.500657684
asymmetricdimethylarginine.taurine	-0.022627713	0.033513646	0.500699946
citruline.lysopcac170	-0.009485366	0.014048813	0.500704378
asparticacid.lysopcac181	-0.049917621	0.073946361	0.500780576
lysopcac160.pcaec401	-0.047817492	0.070836388	0.500787156
pcaac405.glutamine	-0.004089835	0.006059467	0.500845334
methioninesulfoxide.pcaac240	-0.013964742	0.020692137	0.500888352
pcaac360.pcaec383	0.053179538	0.078818422	0.500997369
c5.c8	-0.052891509	0.078406871	0.501081007
putrescine.isoleucine	0.008465031	0.012548742	0.501084433
lysopcac160.pcaec343	0.044503475	0.065976568	0.5011082
pcaec421.h1	0.001999932	0.002964983	0.501119229
prolinr.c142	0.046046316	0.068289693	0.501270036
smohc221.pcaec340	0.017646735	0.026172231	0.501285871
pcaec403.pcaec301	0.035902665	0.053253396	0.5013294
pcaec363.glutamicacid	0.018872075	0.028001119	0.501462317
pcaec421.pcaec385	-0.048857657	0.072494856	0.501480828

lysine.pcaec425	0.003810917	0.005654736	0.501488939
ornithine.pcaec385	-0.029033648	0.043085016	0.501530097
methionine.c51	-0.03928744	0.058302681	0.50154007
pcaac300.pcaec445	0.030999259	0.046004633	0.501555274
pcaec404.prolinr	-0.009630566	0.014295125	0.501639765
pcaac404.smohc161	0.008162624	0.01211884	0.501733231
arginine.glycine	0.019713936	0.029271442	0.501772123
c18.pcaac362	-0.019067877	0.02831653	0.501837911
c0.pcaec446	-0.047146424	0.070025272	0.50190511
pcaec363.pcaec446	-0.018047282	0.026805241	0.501906951
lysopcac281.asymmetricdimethylarg	-0.009644845	0.014328039	0.501989119
pcaac401.pcaec420	0.024015433	0.035676998	0.501995383
pcaac425.phenylalanine	0.020773147	0.030862267	0.502022571
smc161.smohc141	0.031029988	0.046100893	0.502024711
arginine.pcaac381	0.016535937	0.024574021	0.50214234
pcaac360.pcaac383	0.003617281	0.005375684	0.502146336
pcaec365.pcaec383	0.034797514	0.05171724	0.502181373
pcaec445.pcaac366	-0.007308001	0.01086196	0.502203429
c0.pcaec385	-0.025115393	0.037334973	0.502268574
smc160.pcaac344	-0.030542301	0.045402292	0.502268736
pcaec301.pcaac343	-0.013372755	0.019879623	0.502279752
c142.serotonin	0.025405584	0.037775444	0.502371266
pcaec363.serine	-0.009692701	0.014412454	0.502383776
c142.c101	-0.011360547	0.016893235	0.502403872
pcaec381.pcaec422	-0.046247631	0.068777008	0.502443368
pcaac362.pcaec341	0.035597892	0.052946735	0.502503404
smohc241.lysopcac281	-0.00960644	0.014289809	0.50255158
prolinr.pcaac422	-0.005142267	0.007650686	0.50263193
serine.pcaac420	0.031360576	0.046662385	0.502668412
pcaac341.prolinr	0.032212084	0.047935491	0.502722871
pcaac386.pcaac384	-0.026433099	0.039341214	0.50278309
pcaac405.pcaac403	-0.003432558	0.0051088	0.502784312
pcaac386.isoleucine	0.013276831	0.019763735	0.502856638
pcaec445.pcaac424	-0.008430853	0.012550793	0.502881238
pcaec406.lysopcac181	0.019407377	0.028891822	0.502889536
asparagine.smc161	-0.050612283	0.075349686	0.502906649
smohc241.pcaec363	-0.012733536	0.018972533	0.503250949
lysopcac181.pcaec322	-0.037390256	0.055713142	0.503273448
c12.c162	-0.07139366	0.10641671	0.503421575
c8.smc180	-0.009188586	0.013697517	0.503463742
smc260.pcaac422	-0.007846079	0.011696951	0.503490059
methionine.pcaec420	-0.021827341	0.032541048	0.503500577
pcaec424.pcaac422	-0.010691057	0.015939226	0.503516141
smohc141.spermidine	0.034660352	0.051677194	0.503535153
asparticacid.c4	-0.024227709	0.036125618	0.503571438

pcaec423.acetylornithoine	-0.015968172	0.023809947	0.503571721
ornithine.pcaac426	0.03619361	0.05397835	0.503654575
pcaac402.pcaac384	0.020537017	0.030631043	0.503690596
smc260.isoleucine	0.006446316	0.009614812	0.503695277
pcaec385.lysopcac181	0.024531718	0.036593523	0.50374143
lysopcac281.pcaac422	-0.007517143	0.011213365	0.503748213
c14.c0	0.000519382	0.000774978	0.503865313
pcaac403.c162	0.046081914	0.068774918	0.503960413
pcaec423.pcaec301	0.041947431	0.062611641	0.504009579
lysopcac170.lysopcac281	-0.010879757	0.016241528	0.504066405
pcaac341.pcaec384	-0.02050732	0.030614786	0.504080741
asparagine.pcaec425	0.004351139	0.006497646	0.504208956
pcaac343.pcaac422	-0.013010883	0.01943065	0.504235857
pcaec364.pcaac344	0.032102815	0.047949458	0.504294689
asparticacid.acetylornithoine	-0.031908232	0.047682782	0.504508553
smc180.pcaec364	0.021438842	0.032038283	0.504517429
glutamicacid.pcaec363	-0.028469999	0.042548722	0.504547956
c181.smc180	-0.008542977	0.012769195	0.504602024
pcaac402.pcaac381	0.03305162	0.049411694	0.504683082
c4.smc160	0.039067191	0.058411845	0.504733761
pcaec380.pcaac361	-0.022110335	0.033067054	0.504842912
lysopcac280.c16	0.009070254	0.01356733	0.504916126
pcaec361.kynurenine	-0.004014356	0.00600491	0.504931478
pcaec406.pcaec363	0.022843513	0.034175139	0.504987055
c8.serine	0.009310086	0.013928733	0.504997332
asymmetricdimethylarginine.smc260	0.012802012	0.019154163	0.505023848
smohc161.c5	-0.002277106	0.003407431	0.505081427
c101.acetylornithoine	0.038605643	0.057777213	0.505142149
c3.smc240	-0.021714166	0.032498977	0.505162445
pcaec300.alanine	-0.008936974	0.013378745	0.505258698
pcaec401.pcaec384	-0.032587463	0.048789345	0.505307221
lysopcac204.pcaac366	0.011666132	0.017466877	0.50532087
pcaec301.pcaec446	-0.035502919	0.053157812	0.505335209
pcaec404.pcaec361	0.004235223	0.006341678	0.505359522
lysopcac170.smc161	0.03234543	0.048435291	0.505380175
pcaec321.pcaec446	-0.039307138	0.058861093	0.505387928
pcaec423.pcaac240	-0.006696758	0.010028809	0.505415176
lysopcac204.pcaac321	-0.015950751	0.023893289	0.505522845
pcaec402.c101	0.019991414	0.029946254	0.505526964
pcaac360.methioninesulfoxide	0.009380497	0.014051732	0.505531907
pcaec300.lysopcac280	0.005026218	0.007529928	0.505576381
pcaac405.c182	0.015263489	0.022866796	0.505578343
pcaac281.pcaec383	-0.027325586	0.040949335	0.505701488
pcaac364.pcaec406	0.008119712	0.012168988	0.505737143
c5.c2	-0.069525706	0.104216569	0.505812984

lysopcac181.histidine	0.029702432	0.044525096	0.505834014
methioninesulfoxide.tyrosine	-0.035016986	0.052495895	0.505866993
asparagine.pcaec340	0.034175254	0.051235197	0.505876849
acetylornithoine.pcaac403	-0.002036167	0.003052953	0.505925813
c3.smc261	-0.026649492	0.039958244	0.505936304
pcaac343.lysopcac203	0.016061597	0.024085091	0.505977507
c142.creatinine	-0.011856919	0.017782653	0.506041167
pcaec381.c51	-0.049836234	0.074765261	0.506168226
c5.lysopcac161	-0.089353704	0.134050373	0.506169033
lysopcac182.glutamine	-0.009876919	0.014820792	0.506261397
smc261.pcaec424	-0.003982453	0.005976189	0.506284651
c102.smc161	0.059433751	0.089209462	0.506386342
asymmetricdimethylarginine.pcaac42	0.00741981	0.011140304	0.50650995
c18.c3	-0.023750746	0.035665357	0.506573518
pcaac363.smc160	-0.040906192	0.061429327	0.506590511
pcaac300.smc202	-0.016042024	0.024092551	0.506626588
pcaac386.pcaec384	0.024324676	0.036534037	0.506652957
c51.pcaec384	-0.017318166	0.026013156	0.506692554
c10.pcaec380	-0.035379387	0.053149283	0.506747229
leucine.pcaec361	0.003889128	0.00584252	0.506748128
phenylalanine.smc240	-0.027910756	0.041941246	0.50686701
c51dc.pcaac343	-0.013409551	0.020152029	0.506900995
smc240.prolinr	-0.016657468	0.025039879	0.507016939
pcaac404.pcaec422	0.006193335	0.009310477	0.507040487
pcaac300.c12	0.026466088	0.039794112	0.507120275
lysopcac181.pcaec403	0.020447508	0.030749974	0.507193807
ornithine.pcaac320	-0.02690274	0.040458432	0.507201778
pcaac401.pcaac361	-0.038934976	0.058557779	0.50723316
c102.pcaac405	0.043957187	0.066123033	0.507309489
c51.tyrosine	-0.023170259	0.034854649	0.507316272
smc240.smc180	-0.005175072	0.00778504	0.507330553
putrescine.smc261	0.013038463	0.019615008	0.507347754
c5.h1	-0.00244687	0.003681692	0.507420531
acetylornithoine.pcaac320	-0.042416662	0.063822532	0.507421416
pcaec443.pcaac322	0.031711806	0.047724457	0.507501916
c51.smc160	-0.041876182	0.063025633	0.507531307
c4.pcaac240	-0.012405541	0.018676765	0.507663838
pcaac405.pcaac321	-0.021134775	0.031819328	0.507671071
lysine.pcaac365	-0.024468427	0.036841088	0.507703294
asparagine.pcaac364	-0.018997685	0.028605962	0.507732064
alphaaminoadipicacid.c12	-0.023520437	0.035423196	0.507816428
pcaac360.pcaac362	-0.005862487	0.008829894	0.507846885
pcaac344.pcaec380	-0.032569295	0.049055178	0.507849733
pcaac281.asparagine	0.003409314	0.005136858	0.507999942
lysopcac160.c8	0.033093451	0.049868277	0.508050473

methionine.glutamicacid	0.020487852	0.030879958	0.508146
pcaec322.pcaac364	0.032396062	0.048828523	0.508147076
pcaec340.pcaac362	-0.010334083	0.015577139	0.508180599
c12.serotonin	0.016154455	0.024352403	0.508213527
pcaec300.pcaac422	-0.006990655	0.0105385	0.508224646
pcaec422.glutamine	-0.00455374	0.006865245	0.508250876
pcaac406.pcaac281	0.00095678	0.001442821	0.508360866
c8.arginine	-0.006293753	0.009492994	0.508451352
pcaec402.pcaec403	-0.021749461	0.032807887	0.508486712
asymmetricdimethylarginine.pcaec40	-0.00197223	0.002975553	0.508564936
c51.smc161	-0.054502033	0.082264956	0.508752183
pcaac366.pcaac300	-0.031636235	0.047753998	0.508774371
pcaec424.pcaac362	-0.003188227	0.004813628	0.508870097
pcaac323.pcaec446	-0.015744106	0.02377092	0.508874772
pcaac281.arginine	-0.029671932	0.044808865	0.508962566
pcaec422.pcaac404	0.005680606	0.00857887	0.508979437
smc160.serotonin	0.048858291	0.073789968	0.509002428
c101.pcaec446	-0.018426033	0.027829873	0.509022232
c51.pcaec365	-0.014200912	0.021449814	0.509049398
pcaec381.pcaac422	-0.036311771	0.054855088	0.509109989
pcaac342.pcaec446	-0.027981092	0.042270227	0.509110412
pcaec386.smohc241	-0.054346243	0.082103254	0.509130789
smc261.c14	0.021784688	0.032914594	0.509175882
c141oh.pcaac405	-0.032320592	0.04883769	0.509213525
pcaec300.pcaec385	-0.029915644	0.045204606	0.509221829
c3.lysopcac204	0.027769008	0.041961268	0.509225636
pcaac366.ornithine	-0.021096217	0.031878379	0.509228953
c142.smc180	-0.013114156	0.019818478	0.509266251
pcaec322.c3	-0.020627441	0.031174827	0.50929422
pcaac402.pcaac322	0.045053152	0.06810021	0.509357103
pcaac384.pcaec444	-0.04306437	0.065097388	0.509378701
pcaac405.pcaec425	0.007029959	0.010627579	0.509413827
pcaec381.c141	-0.020531136	0.031038074	0.509414064
c10.isoleucine	-0.008818931	0.013332495	0.509427369
pcaac406.asparagine	-0.006539456	0.009886492	0.50943237
isoleucine.c141	-0.020252314	0.03062009	0.509462747
c4.isoleucine	0.00724524	0.010955033	0.509491074
pcaec381.tyrosine	-0.024646876	0.037272652	0.509556789
c14.taurine	-0.017617961	0.026645819	0.509600594
pcaac421.pcaec446	0.044765918	0.067713844	0.509655626
pcaac341.pcaac321	-0.029775386	0.045041565	0.509681024
serotonin.creatinine	-0.004428151	0.006698957	0.509708932
lysopcac182.c142	0.055778585	0.084398278	0.509788088
prolinr.transhydroxyproline	0.02132595	0.032270027	0.509812272
c10.c2	0.027463078	0.041563271	0.509879942

pcaec340.c2	0.033961956	0.051402134	0.509907131
pcaec361.pcaac422	-0.01545102	0.023385637	0.50991064
pcaec386.glycine	0.026719666	0.040441193	0.50991149
smohc141.c5	-0.002165041	0.00327695	0.509922047
pcaac360.pcaec404	-0.008472034	0.012825704	0.510009359
pcaec386.pcaac403	-0.002416348	0.003658125	0.510014688
lysopcac140.pcaac420	0.039376646	0.059623112	0.510089482
pcaec322.smc161	-0.053725564	0.081351444	0.510097622
pcaac384.glutamine	-0.005104158	0.007730602	0.510199639
methionine.creatinine	-0.003102974	0.004699743	0.510206282
pcaac361.pcaac366	-0.007124077	0.01079146	0.510260333
alanine.c8	-0.020246207	0.030672479	0.510312464
pcaec401.c182	0.014279281	0.021635536	0.510367017
pcaec363.pcaac403	-0.002097973	0.003178957	0.510389724
pcaac322.pcaac366	-0.029562931	0.044802175	0.51045479
c181.smc240	-0.013951485	0.021146986	0.510529084
lysopcac160.c142	0.017916702	0.027157476	0.510532293
c3.pcaac404	0.004051152	0.006142319	0.510651416
c4.smc261	0.016940953	0.025686236	0.5106599
smc181.pcaec405	-0.015243697	0.02311588	0.510715772
pcaac403.lysopcac182	-0.046814467	0.070994952	0.51074227
kynurenine.c12	-0.018642187	0.028273595	0.51077801
smohc221.pcaec380	0.023029211	0.034929705	0.510808801
c182.c142	0.030530722	0.046313113	0.510858406
methioninesulfoxide.c51	-0.057754358	0.087610088	0.510860784
lysopcac160.smc260	-0.02008689	0.030471157	0.510867476
serine.lysopcac203	0.005426486	0.008232913	0.5109244
smohc241.lysine	0.02500694	0.037941198	0.510939608
pcaac381.pcaac385	-0.006897336	0.010464963	0.510945277
pcaec423.pcaac360	-0.022298093	0.033832078	0.510949778
pcaac404.alanine	0.013129269	0.019924785	0.511039298
smc261.glycine	-0.011435019	0.017360835	0.511214711
h1.pcaac321	-0.018680523	0.028377277	0.511455503
asparagine.putrescine	0.020204986	0.030701538	0.511571876
pcaac404.pcaec382	0.003211897	0.00488055	0.511577267
pcaac341.c8	0.022462484	0.034133221	0.511589278
lysopcac140.pcaac322	-0.023829576	0.036212126	0.511606952
c141oh.pcaec421	-0.001974991	0.00300175	0.511676551
smc160.pcaac240	-0.005473382	0.008319803	0.511722962
pcaac406.threonine	0.017104228	0.026000531	0.511743818
pcaec360.asparagine	0.006560872	0.009973336	0.511743985
pcaec341.creatinine	0.002205141	0.003352845	0.511839355
glycine.lysopcac204	-0.008991866	0.013673026	0.511875839
asymmetricdimethylarginine.pcaac3[illegible]	0.005779045	0.008788368	0.51191214
pcaac323.pcaac240	-0.006612576	0.010056971	0.511955128

pcaec384.lysopcac140	-0.00061927	0.000941905	0.511984527
pcaec363.pcaac322	0.034215397	0.052058341	0.512122211
pcaac323.threonine	-0.021390399	0.032552301	0.512213442
pcaac300.pcaec422	0.013014326	0.019807991	0.512267683
pcaac366.lysopcac170	-0.005068336	0.007715512	0.512345913
c8.c4	0.003401436	0.00517818	0.512360869
pcaac341.pcaac366	-0.004750129	0.00723331	0.512474133
lysopcac181.c102	0.026874523	0.040924764	0.512487482
pcaac421.asparagine	-0.005648442	0.008601972	0.512510694
c162.pcaec446	-0.017327176	0.026387948	0.512518894
methioninesulfoxide.smc240	-0.026475191	0.040321716	0.512540306
lysopcac182.pcaac403	-0.001494456	0.002276093	0.512546892
c141.lysine	0.024839423	0.037831747	0.512554834
pcaec402.pcaec401	-0.024860176	0.037865482	0.512578486
pcaec385.h1	-0.00130403	0.001986261	0.512587589
pcaec363.smc181	0.010269723	0.015642726	0.512592457
pcaac420.pcaac366	-0.012857917	0.019586343	0.512620605
pcaec444.pcaac362	-0.002539998	0.003869925	0.512704363
pcaec425.pcaec342	-0.055356995	0.08434688	0.512730875
lysopcac280.isoleucine	-0.011652668	0.017755703	0.512746368
pcaac381.glutamicacid	-0.029196949	0.044491135	0.512769167
pcaac366.smc160	-0.022679749	0.03456317	0.512807267
serotonin.pcaac240	0.011277368	0.017192919	0.512968615
pcaac362.pcaac420	0.037526274	0.05721278	0.512983878
pcaec445.glutamicacid	0.037452285	0.057111372	0.513067837
pcaec361.pcaac362	-0.010945484	0.01669218	0.513100595
pcaac322.pcaec362	0.014690443	0.022404717	0.51312625
phenylalanine.pcaec422	-0.02213975	0.033769034	0.513166304
pcaac425.c0	-0.000537996	0.000820637	0.513191466
methioninesulfoxide.c5	0.012742949	0.019439012	0.513222779
pcaac401.pcaec402	-0.085049634	0.129764266	0.51329874
alanine.lysopcac281	-0.013659939	0.020841767	0.513301762
pcaec322.smohc241	-0.03398298	0.051855794	0.513350231
pcaec380.smc160	-0.01756233	0.02680136	0.513387867
pcaec404.c162	0.049517709	0.075578562	0.513449233
pcaec382.pcaac425	-0.078018616	0.119079544	0.513450045
pcaec420.lysopcac140	0.000446246	0.000681139	0.513471622
pcaac320.creatinine	0.004131341	0.006306062	0.513477458
smohc221.pcaec301	0.032152155	0.049084899	0.513545786
smc202.smc160	-0.041281849	0.063023577	0.513551695
smohc161.pcaec402	-0.027512392	0.042004668	0.513576177
pcaec322.lysopcac204	-0.01896998	0.028963593	0.513592021
pcaac343.pcaac366	-0.006940049	0.010599266	0.513715655
pcaec342.histidine	0.035451652	0.054149012	0.513755108
pcaac300.pcaec380	-0.028199944	0.043076392	0.513790927

pcaec342.c51	-0.031767766	0.048530403	0.513825919
pcaac240.prolinr	-0.015641385	0.023897429	0.513873092
c182.smc160	0.034619377	0.05289489	0.513890994
lysopcac180.pcaac344	-0.027056196	0.041340252	0.513902512
c12.c51dc	0.070291337	0.107440231	0.514056109
lysopcac182.pcaec383	-0.031338286	0.047914146	0.51417547
pcaac424.smc260	-0.022918137	0.035044226	0.514222528
smc241.smohc241	-0.039967324	0.061126719	0.514308462
pcaec385.c4	0.024191974	0.036999815	0.514310625
pcaec444.valine	0.031402105	0.048032001	0.514352849
pcaec343.pcaac420	0.041901421	0.064094024	0.514369162
lysopcac204.smohc141	0.021151239	0.032354483	0.514378709
pcaec445.pcaac422	-0.005155576	0.007887147	0.514421316
c2.c5	-0.003159275	0.004833306	0.514434932
leucine.pcaec420	-0.034248228	0.052399403	0.514465315
smc180.pcaac420	0.03177088	0.048623476	0.514589581
pcaac344.alphaaminoadipicacid	0.002823155	0.004320809	0.514602835
pcaac401.c0	-0.00066922	0.001024269	0.514616648
pcaec403.phenylalanine	-0.035010477	0.05359563	0.514700355
pcaec420.threonine	0.007231312	0.01107061	0.514722664
leucine.lysopcac204	-0.01248445	0.019116766	0.514810194
pcaec365.lysopcac170	0.006392175	0.009788561	0.514834561
pcaec300.pcaec403	-0.012664735	0.019401111	0.514989453
c142.taurine	-0.019515573	0.029898042	0.515019343
pcaac366.lysopcac182	-0.008819438	0.013511503	0.515020643
pcaec446.pcaac403	-0.001940212	0.002972481	0.515027556
pcaec402.pcaac426	0.044977273	0.068920308	0.515108851
pcaac321.phenylalanine	-0.026459651	0.040549273	0.515152202
pcaac401.pcaec383	-0.079508308	0.121869556	0.515233103
glutamicacid.tyrosine	-0.037030738	0.056764847	0.515266316
pcaec301.smc241	0.017135752	0.026268936	0.515287771
pcaec424.spermidine	-0.033420625	0.051237667	0.515322023
lysopcac182.asparagine	-0.003925633	0.006018709	0.51534022
c12.pcaac321	0.01009506	0.015479258	0.515386184
pcaec342.lysine	-0.032206634	0.04939057	0.515441689
pcaac402.pcaac385	-0.014025406	0.021514809	0.515560767
smohc241.lysopcac203	-0.006100114	0.009357911	0.515579049
methionine.smohc141	-0.025457615	0.03905369	0.515582321
kynurenine.pcaec422	0.01547384	0.023738664	0.515595658
c16.serine	0.014529913	0.022290972	0.515603216
smc260.asymmetricdimethylarginine	0.011667319	0.017901556	0.515655066
c142.smc260	0.012613913	0.019354476	0.51566652
pcaac360.pcaac405	-0.029189418	0.044792368	0.515711896
alphaaminoadipicacid.pcaec341	0.015906778	0.024411937	0.515751885
pcaec321.tyrosine	-0.022990539	0.035286517	0.515790137

c142.pcaac240	-0.006434264	0.009876969	0.515853021
pcaec420.pcaac322	0.027770965	0.04263365	0.515888477
pcaec363.serotonin	0.052009004	0.07986478	0.515999655
pcaec342.pcaec401	0.042364803	0.065061954	0.516043221
pcaec422.c18	-0.022526511	0.034598527	0.516083518
glutamine.serine	-0.006024757	0.009254522	0.516132514
pcaac361.pcaec384	-0.032716616	0.050259261	0.516164592
pcaac401.lysine	0.04880343	0.074973462	0.516173756
pcaac366.smc181	0.005881694	0.009036247	0.516201228
pcaac420.pcaec403	-0.012310172	0.018917049	0.516301132
glutamine.isoleucine	0.010696147	0.016437185	0.516311549
asparagine.pcaac420	0.041718219	0.064117271	0.516358995
lysopcac204.glutamicacid	-0.023085789	0.035482356	0.516376787
pcaec384.pcaec343	0.03711002	0.057046226	0.516442365
pcaec362.pcaec380	0.018153571	0.027906842	0.516454818
pcaac322.smohc222	-0.002773285	0.004263471	0.516474286
glycine.pcaac380	-0.02868017	0.044098533	0.516545251
smc260.pcaec446	-0.010975682	0.016876951	0.516564567
arginine.pcaec425	0.003701161	0.005692314	0.516649745
pcaec422.pcaac403	-0.006511932	0.010015756	0.516671949
pcaac383.pcaac361	-0.03732637	0.057422567	0.516761587
c8.smc261	0.022916403	0.035259187	0.516818449
smc161.pcaac425	0.046098256	0.070936436	0.516875334
c5.pcaac343	0.018045036	0.027768843	0.516889945
pcaac421.pcaec401	-0.016384111	0.025213106	0.516893259
pcaec386.pcaec403	-0.011705168	0.018014657	0.516936534
pcaac362.pcaec446	-0.021093761	0.032469376	0.517005493
pcaac386.smc161	0.040037808	0.061631816	0.517019684
c2.pcaec446	-0.034137257	0.052558116	0.517093394
pcaac342.lysopcac170	-0.004630006	0.007128824	0.517117696
c181.serotonin	0.047246263	0.072750301	0.5171475
pcaac281.serine	-0.006283011	0.009674823	0.517155097
smc160.lysopcac170	0.005899462	0.009085899	0.51723246
pcaec401.c51	-0.067322965	0.103689011	0.517245919
lysopcac203.pcaac364	0.023884123	0.03679213	0.517319243
smc261.pcaac422	-0.014315835	0.022054844	0.517359378
smc261.smohc141	0.04075157	0.062794706	0.517447377
glutamine.pcaac405	-0.036857634	0.056800899	0.517494638
pcaec343.smc260	-0.018864474	0.029075719	0.517550477
lysopcac170.arginine	-0.029376082	0.045277279	0.517551144
pcaec365.pcaac403	-0.001597058	0.002461626	0.5175655
pcaac421.pcaec386	-0.008177275	0.012605572	0.517616143
pcaac384.c14	0.013467639	0.0207628	0.517655129
smc240.pcaac366	-0.010150805	0.015649347	0.517656343
lysopcac160.pcaec405	-0.019478412	0.030029807	0.517659504

smc241.pcaac343	0.012126645	0.018701739	0.517796521
pcaec443.pcaac385	-0.009410773	0.014514759	0.517838101
pcaac406.histidine	0.024305445	0.037492929	0.517897042
lysopcac280.smc241	0.027509462	0.042435556	0.517898975
pcaac404.c3	-0.034593854	0.053364147	0.517901685
serotonin.pcaac384	-0.027476733	0.042385785	0.51790603
pcaec342.c102	0.014486884	0.022353948	0.518025462
c51dc.valine	-0.021322026	0.032901029	0.518027152
pcaac383.pcaac385	-0.010018641	0.015462259	0.51810712
pcaac322.lysopcac203	0.007758388	0.011974957	0.518144028
pcaac381.glycine	-0.007407946	0.011434134	0.518146933
pcaac240.pcaec406	0.004194524	0.006474641	0.518173427
pcaec364.lysopcac281	0.008154912	0.012588836	0.518205401
pcaac365.smc181	0.020467509	0.031598807	0.51824332
pcaec443.pcaec385	-0.03533892	0.054558407	0.51824592
pcaac344.pcaac322	0.05732926	0.08851975	0.518299243
pcaec406.pcaec385	-0.0457678	0.070675225	0.518340908
c182.pcaec301	-0.026136946	0.040364551	0.518377574
pcaac422.lysopcac180	-0.03899105	0.060238252	0.518533494
pcaec386.alphaaminoadipicacid	0.002290228	0.003538339	0.518546295
pcaec405.lysopcac280	0.010218876	0.015788576	0.518564625
smc241.pcaec423	0.012451877	0.019238954	0.518571187
pcaac321.pcaac364	0.041953083	0.06482588	0.518607629
smc180.pcaec380	0.029257047	0.045211553	0.5186407
lysopcac281.pcaec364	0.006230142	0.009628047	0.518661179
pcaac281.smc240	-0.023256126	0.035941759	0.518682061
lysopcac204.kynurenine	-0.006355083	0.009822815	0.518732778
methionine.serine	-0.013182986	0.020376782	0.518739579
lysopcac160.pcaec301	0.022208177	0.034332682	0.518809771
pcaac365.c5	-0.005048939	0.007806691	0.518879008
c141oh.smc160	0.04477309	0.06923991	0.518948747
pcaec362.creatinine	0.002320195	0.003588248	0.518966534
pcaac360.pcaec342	-0.078157119	0.12087515	0.518976666
ornithine.smc240	-0.006512895	0.010073637	0.519018698
pcaec322.c51	-0.03031589	0.046896679	0.519075912
smohc241.c141	-0.021215288	0.032821697	0.519114667
pcaac424.c101	0.010536554	0.016301159	0.519122131
pcaac323.c12	0.029335408	0.045389699	0.519165641
phenylalanine.pcaec406	0.002454983	0.003798784	0.519195335
isoleucine.pcaac403	0.01003765	0.015533853	0.51924439
c14.creatinine	0.004858825	0.007520008	0.519282733
c182.pcaec424	0.005883746	0.009106919	0.519311998
serotonin.pcaac404	-0.002321599	0.003593606	0.519336722
pcaec342.lysopcac203	-0.011977741	0.018542216	0.519378444
smohc241.pcaac420	0.033200562	0.051400306	0.519410664

pcaec360.alphaaminoadipicacid	0.002836695	0.004392079	0.519446408
pcaac381.tyrosine	-0.02518532	0.039002571	0.519531163
c10.c4	0.004535291	0.007024216	0.519576225
pcaac385.pcaac363	0.004971793	0.007701901	0.519664798
smc181.asparticacid	0.014982577	0.02321301	0.519722388
c141.isoleucine	-0.005862389	0.009083683	0.519763114
pcaac323.creatinine	0.00340397	0.005274785	0.519793493
pcaec385.pcaac420	0.025906085	0.040144617	0.51979975
arginine.pcaec420	-0.012167881	0.018858582	0.519865529
pcaec445.lysopcac204	-0.008549161	0.013250668	0.51988493
smc240.asymmetricdimethylarginine	0.00912246	0.014139465	0.519891384
pcaac366.pcaac404	-0.004536997	0.007032392	0.51990442
c101.pcaac406	0.014026773	0.021742665	0.519924013
pcaac364.pcaac361	0.020735417	0.032143544	0.519948927
pcaec401.prolinr	-0.014771685	0.022902097	0.520010563
smc160.kynurenine	-0.002300232	0.003566355	0.520017979
pcaec444.isoleucine	-0.007398785	0.011472641	0.520065993
pcaac406.glutamine	-0.005140151	0.007971639	0.520131982
asymmetricdimethylarginine.pcaac24	0.006067851	0.009411832	0.52019674
phenylalanine.c12	-0.021101022	0.032733987	0.520250604
pcaac365.pcaec420	0.016241839	0.025201136	0.520336457
pcaec385.lysopcac204	-0.016028367	0.024872536	0.520380438
c12.pcaac426	0.026595116	0.041270015	0.520382316
smc181.smc260	-0.017492825	0.027145994	0.520394838
pcaec380.pcaac421	-0.065435066	0.101566151	0.520483686
asparagine.h1	-0.002902738	0.00450596	0.520523022
pcaec362.lysopcac140	-0.000522362	0.000810993	0.520586153
pcaec360.pcaec406	-0.033201393	0.051556374	0.520663566
smc261.pcaec403	-0.011990827	0.018621508	0.520701569
c162.creatinine	0.005752853	0.008935019	0.520746153
smc180.pcaec404	-0.012273825	0.019064043	0.520768072
pcaec446.alphaaminoadipicacid	0.003303167	0.005130744	0.520782284
c51.smc241	-0.010809954	0.016795086	0.520886262
lysopcac180.lysopcac170	0.005727263	0.008898314	0.52088833
pcaac320.pcaac343	-0.030483247	0.047365108	0.520923545
pcaec424.pcaec383	-0.043861127	0.068159983	0.520973742
leucine.kynurenine	-0.002785132	0.004328414	0.521005673
pcaec385.pcaec380	0.02730108	0.042429963	0.521015086
asparagine.pcaac362	0.008389735	0.013038942	0.521016318
pcaec363.lysopcac180	0.041733555	0.064861253	0.521021913
smc241.lysopcac281	0.008261539	0.012840069	0.521028093
smc260.pcaec443	-0.030795825	0.047863427	0.521033404
pcaec445.creatinine	0.004138383	0.006432042	0.521039397
pcaac421.ornithine	-0.011973523	0.018610417	0.52105468
pcaac426.serine	-0.018470693	0.028711094	0.52108573

pcaec341.pcaac422	-0.009811392	0.015251331	0.521095849
pcaec444.pcaec420	0.017256054	0.026827919	0.521161343
c101.smc161	-0.031831638	0.049492527	0.521195021
pcaec423.smc181	0.004814787	0.007486153	0.521195949
pcaec421.pcaac362	-0.00621052	0.00965804	0.521272007
smc161.c141oh	0.044628038	0.069411072	0.521329424
pcaec402.lysine	0.046177265	0.071825305	0.521356542
pcaec423.isoleucine	-0.012082008	0.018793786	0.521381344
c16.pcaac383	0.005421512	0.008433645	0.521400178
c162.pcaec406	-0.014565827	0.022664836	0.521517565
smc181.c4	0.008260015	0.012853274	0.521532434
pcaac405.serine	-0.011358072	0.017674367	0.521538462
c51.pcaac342	-0.010258115	0.015969629	0.521718458
pcaac381.taurine	0.028222166	0.043939915	0.521758282
c18.glycine	-0.010249709	0.015961014	0.52183493
pcaac386.pcaec406	-0.011934868	0.018587035	0.521876602
pcaac403.smc261	0.037739814	0.058783063	0.521933991
c14.c141	0.024682881	0.038445869	0.521935312
pcaac385.glutamicacid	-0.023449679	0.036525688	0.521942643
prolinr.lysopcac203	0.010069714	0.015685958	0.521973638
pcaec405.histidine	0.052191077	0.081313544	0.522043237
serotonin.pcaac320	0.059427631	0.092595491	0.522076527
pcaac362.c12	-0.03614438	0.056320971	0.522103249
pcaac321.histidine	0.029691656	0.046266708	0.522107868
pcaac342.acetylornithoine	0.008705373	0.01356602	0.522137389
pcaac383.c12	-0.027268474	0.042494515	0.522144085
pcaac366.pcaec362	0.00855141	0.013327476	0.522180603
pcaac366.c141oh	0.040245614	0.062728704	0.522216521
pcaac366.lysopcac204	-0.036968628	0.05762399	0.522237788
smohc222.pcaec361	-0.007039092	0.010972649	0.522261554
pcaec380.serine	0.034578112	0.053909718	0.522329405
putrescine.pcaec405	-0.026097956	0.040690517	0.52234943
pcaec444.pcaac406	-0.01837292	0.028646939	0.52236218
pcaac362.pcaac422	-0.00746761	0.011645137	0.522422362
lysopcac180.c181	-0.020359524	0.031752192	0.522463579
pcaec403.pcaec402	-0.074110041	0.115589165	0.522496081
pcaec360.pcaac344	-0.025868828	0.040349032	0.522511662
smc241.pcaec401	0.030942787	0.048265154	0.522528785
pcaec301.pcaec360	-0.002433897	0.003796848	0.522573507
c141.smohc141	0.014019751	0.021872354	0.522606182
pcaac424.smc240	0.024321517	0.037946183	0.52262742
pcaec406.alphaaminoadipicacid	0.002955104	0.004613039	0.522853871
c141oh.pcaec403	0.033762054	0.052715197	0.522942392
asymmetricdimethylarginine.pcaac32	0.049568282	0.077406096	0.523003849
c51dc.taurine	-0.029118353	0.045473319	0.523021573

c162.pcaac420	0.051737629	0.080805472	0.523063997
pcaec384.pcaac420	0.033443328	0.052244025	0.523152747
pcaac320.prolinr	0.023810264	0.037199613	0.523197715
pcaac386.pcaac420	-0.031309879	0.048916648	0.523198752
pcaec343.pcaac421	0.011729502	0.018325568	0.523201207
pcaac402.pcaac323	-0.063693368	0.099520732	0.523240825
c0.tyrosine	-0.02050335	0.032039388	0.523279067
pcaec401.glycine	0.018137364	0.028350341	0.523398188
lysopcac170.creatinine	0.002648895	0.00414137	0.523489046
lysopcac182.pcaac424	-0.002424191	0.003790395	0.523525761
pcaac420.pcaec421	-0.000960051	0.001501233	0.523560185
pcaac281.pcaac380	-0.024458636	0.038246748	0.523568301
pcaec420.pcaac384	-0.027026158	0.042262668	0.523578159
pcaec443.pcaac380	0.033730468	0.05275466	0.523641038
c102.pcaac426	0.042037531	0.065747725	0.523645873
smc261.lysopcac170	0.011525143	0.018031881	0.523790188
pcaac323.c101	-0.01018614	0.015937546	0.523806496
pcaac403.pcaac321	0.007405685	0.011587783	0.523828726
pcaac300.c51	-0.030305217	0.047419446	0.523832527
asparagine.smc260	0.010827082	0.016941463	0.523832905
pcaac240.lysopcac204	-0.018390976	0.028778284	0.523852615
lysopcac204.pcaec361	0.002029591	0.003176269	0.52389906
pcaec341.pcaac361	0.01932991	0.030257046	0.523982923
pcaec301.arginine	0.014556771	0.022790072	0.524063038
c14.pcaec422	-0.014940615	0.023392584	0.524090783
pcaec424.pcaac366	-0.005169379	0.008094058	0.524108151
prolinr.methioninesulfoxide	-0.013141656	0.020577316	0.524118357
pcaac362.c51dc	-0.05964718	0.093400702	0.524138952
pcaac281.pcaec446	-0.01519722	0.023804096	0.524260397
c10.serine	0.009070755	0.014208661	0.524281536
c141oh.c101	-0.002960068	0.004637968	0.524392451
pcaec423.arginine	-0.025638211	0.04017937	0.524477696
pcaac344.pcaec301	-0.043955849	0.06888728	0.524484335
pcaac424.pcaac380	0.036613152	0.057383314	0.524509256
lysopcac281.threonine	-0.014699174	0.023042013	0.524584554
pcaec342.c12	0.027794451	0.043569919	0.524585667
pcaac406.pcaec342	-0.054973194	0.086175675	0.524590508
pcaec423.c162	0.037278939	0.05844515	0.524639326
smohc222.putrescine	0.019334641	0.03031468	0.524669781
pcaec423.h1	0.001650438	0.00258775	0.524675739
c14.pcaec446	-0.016316128	0.025584976	0.524718433
pcaec401.smc181	0.004609818	0.007228786	0.524731458
lysopcac181.smc241	-0.056953359	0.089317569	0.524765741
pcaec406.pcaec422	-0.014419126	0.022615296	0.524809381
pcaec401.c4	-0.019724972	0.030942208	0.524877568

prolinr.lysopcac161	0.040308427	0.063234195	0.524897812
serine.smc161	0.037611283	0.059006534	0.524922416
pcaec444.pcaac422	-0.004999304	0.007843666	0.524948659
c141oh.glutamicacid	-0.024956622	0.039163046	0.525025985
pcaec362.pcaec360	-0.003552167	0.005575107	0.525091881
smohc221.smc240	-0.028930436	0.04540755	0.525104525
smohc221.pcaec384	0.012215691	0.019173485	0.525113981
pcaac343.valine	0.022605077	0.035485252	0.525170075
asymmetricdimethylarginine.histidin	-0.02242825	0.035208789	0.525183226
pcaac321.pcaac366	-0.005066849	0.007954381	0.525195341
alanine.c162	-0.046265344	0.072645904	0.525278122
pcaac343.lysopcac182	-0.008385927	0.013167724	0.525282216
smohc161.pcaac402	0.033828569	0.053118892	0.525287677
pcaec384.pcaac320	0.049277544	0.077379054	0.525296235
pcaec385.ornithine	-0.015800297	0.024811763	0.525313398
c12.lysopcac281	-0.011871576	0.018644877	0.525369369
lysopcac281.serotonin	0.060794066	0.095492007	0.525421434
glycine.phenylalanine	-0.017963874	0.028218534	0.525448675
pcaac402.pcaac363	-0.010840948	0.01702953	0.5254496
lysopcac180.lysopcac160	0.013293516	0.020882701	0.525460452
c3.pcaec424	-0.011060161	0.017379864	0.525591965
pcaac300.lysopcac170	0.009719676	0.015273739	0.525600187
smc180.alanine	-0.014500922	0.022788136	0.525618858
lysopcac182.pcaac362	-0.003727705	0.005858405	0.525642335
c142.isoleucine	-0.007624678	0.011984734	0.525708059
pcaac381.alanine	-0.009878239	0.015527153	0.525713205
asymmetricdimethylarginine.c51dc	-0.05767814	0.090674623	0.525772411
pcaac365.serine	-0.019702422	0.030979823	0.525853116
lysopcac203.smc241	0.008815051	0.013862627	0.525911533
lysopcac180.pcaec422	-0.020156916	0.03169971	0.525921512
alphaaminoadipicacid.alanine	0.006191933	0.009738575	0.525957661
pcaec403.pcaec363	0.022561214	0.035490255	0.526031359
asymmetricdimethylarginine.pcaac34	0.00954784	0.015023556	0.526146449
acetylornithoine.c3	0.021131118	0.033251297	0.526164042
pcaac405.pcaac380	-0.02266431	0.035664856	0.526175318
c18.c102	-0.014673997	0.023107436	0.526465693
pcaec322.c12	0.021411848	0.033723711	0.526539749
pcaac320.alphaaminoadipicacid	0.003041855	0.0047917	0.526606189
pcaac365.pcaac322	0.038406505	0.060502056	0.52661972
smohc222.ornithine	-0.016825717	0.026510741	0.526698558
phenylalanine.serine	-0.00838618	0.013213718	0.526710503
pcaec363.pcaac281	0.000903548	0.001423969	0.526794749
pcaec365.pcaac344	0.021349845	0.033653596	0.526877667
pcaec362.methionine	-0.022979092	0.036223036	0.526892152
lysopcac180.tyrosine	-0.025839458	0.040743255	0.527006482

smc260.pcaec406	0.001982405	0.003126114	0.527044729
pcaec321.pcaac300	-0.038160099	0.06018417	0.527101913
smc240.pcaec420	0.007886849	0.012438983	0.527110061
creatinine.pcaac424	-0.007433227	0.011725146	0.527166571
pcaac421.c162	0.029326316	0.046260111	0.527174461
lysopcac203.smohc241	0.040908425	0.064530877	0.527179822
alphaaminoadipicacid.serine	0.009388378	0.01480977	0.527182609
lysine.pcaac426	-0.023618941	0.037261218	0.527219545
c14.pcaec343	0.028359672	0.044742344	0.527239401
pcaec406.pcaac420	-0.052457892	0.082783756	0.527350283
c182.c8	-0.023716089	0.037432341	0.527416384
smc260.pcaec364	0.008862592	0.013989358	0.527447841
pcaac422.pcaec385	-0.02747935	0.043375438	0.527448198
smc161.pcaec362	0.006712392	0.010597135	0.527518225
c4.phenylalanine	0.01140896	0.018013868	0.527565427
pcaac362.pcaac425	0.05541719	0.087516061	0.527643606
pcaec363.lysopcac140	-0.0006192	0.000977917	0.52766968
pcaec382.pcaec405	0.090762143	0.143358173	0.527714272
c181.c142	0.021235087	0.033541749	0.527727504
asparticacid.alanine	0.01784739	0.02819087	0.527729494
pcaac403.pcaac344	0.045820614	0.072376582	0.527732734
smc160.c18	-0.01417515	0.022394857	0.527811957
pcaac360.pcaec365	-0.013854992	0.021889459	0.527819647
pcaec386.lysopcac280	0.003966541	0.006267692	0.527883207
pcaec362.transhydroxyproline	0.011232166	0.017749068	0.527898673
pcaac384.pcaec385	0.031933857	0.050462525	0.527903954
pcaec342.pcaec340	0.070771339	0.111848846	0.52795755
pcaec341.pcaec446	-0.025285901	0.03996825	0.528016941
pcaac386.pcaac344	-0.048315033	0.076373481	0.52803934
c182.smc261	-0.018417076	0.029112626	0.52803971
c181.pcaac420	-0.061230814	0.096798214	0.528074442
pcaec402.pcaac385	-0.008845937	0.013984532	0.528080893
smohc161.lysopcac161	0.030546705	0.04829566	0.528118478
putrescine.pcaac366	-0.003439412	0.005437984	0.528127926
c51.pcaec424	0.004364697	0.006901627	0.528169364
pcaac240.pcaec446	0.045095602	0.071308412	0.528178073
pcaac321.c102	0.023002187	0.036374417	0.528197354
pcaac380.asparagine	-0.005947284	0.009405202	0.528218767
c2.c12	-0.049919606	0.078950789	0.528252763
pcaac386.glutamine	-0.005935241	0.009387291	0.528268524
smc161.pcaac424	-0.009122042	0.01442879	0.528302541
lysopcac203.pcaec446	-0.018715225	0.029604987	0.528332891
citruline.pcaec384	0.004728513	0.007480968	0.528392966
pcaec401.pcaec362	0.009187106	0.01453955	0.528524792
pcaec401.c141	-0.019476668	0.03083381	0.528657771

pcaac383.c182	0.022540089	0.035689283	0.528723797
pcaec363.lysopcac160	-0.009030679	0.014299655	0.528745565
pcaac421.pcaec380	0.042431919	0.067197779	0.528799658
lysopcac170.c16	0.019031726	0.030142808	0.528840669
pcaac405.pcaac422	-0.011312	0.017918384	0.528891738
pcaac362.glutamicacid	-0.017530895	0.02777478	0.528974275
pcaec362.pcaac422	-0.011817983	0.018726044	0.529027438
lysopcac160.methionine	-0.015927613	0.02524408	0.529128014
pcaec402.pcaec342	0.042682592	0.067658432	0.529187009
lysopcac161.pcaac424	-0.0026434	0.004190641	0.529231038
pcaec423.smc161	0.049937787	0.079180642	0.529299298
c18.pcaac240	-0.010082286	0.015991599	0.529434812
prolinr.pcaac364	0.02356365	0.037378466	0.529478413
pcaac384.c141oh	0.054683547	0.086748613	0.529503931
pcaac381.smc260	-0.02214286	0.03513086	0.529550515
prolinr.threonine	0.028996491	0.046005736	0.52956135
c16.creatinine	-0.006476871	0.010278808	0.529666379
spermidine.c5	0.023773247	0.037732204	0.529710068
kynurenine.c16	-0.01680483	0.026673862	0.529736685
pcaac421.c16	0.012788079	0.020299685	0.529767166
lysopcac160.smc240	0.028924297	0.045923976	0.52985484
c182.pcaac385	0.009750915	0.015482477	0.529872268
smc181.pcaec403	-0.018502687	0.029392446	0.530066993
c3.pcaac366	0.017527387	0.027846483	0.530116393
pcaac386.c182	0.016407039	0.026069505	0.530163114
pcaac402.putrescine	-0.031400862	0.049895355	0.530178227
pcaec386.glutamine	-0.010189841	0.016193288	0.530224638
alanine.histidine	-0.029095805	0.04623862	0.53023112
pcaec365.pcaec363	-0.018223462	0.028966581	0.530317982
pcaec361.pcaec362	0.010383501	0.016506932	0.530371096
pcaec420.pcaec340	0.022611087	0.035947406	0.530393395
pcaac385.lysopcac204	-0.007906135	0.012569779	0.530409765
pcaac341.pcaac360	-0.02320278	0.036894147	0.530460819
glutamine.smc241	0.010416727	0.016570715	0.530642794
alphaaminoadipicacid.smc202	-0.019449698	0.03094268	0.530675955
pcaec380.smc260	-0.016418482	0.026123595	0.530727816
smc202.c4	0.008707561	0.013856316	0.530776314
prolinr.c182	0.030122272	0.047939825	0.530830586
acetylornithoine.pcaec343	0.013965939	0.022230204	0.530891596
lysopcac280.pcaac384	0.02028508	0.032289202	0.530898485
pcaac341.pcaac381	0.010655479	0.016962625	0.530935802
alphaaminoadipicacid.glutamine	0.004135384	0.006587621	0.531212182
glutamine.kynurenine	-0.007433976	0.01184409	0.531276207
pcaec300.taurine	0.016955985	0.027018047	0.531323777
c141oh.smc180	-0.005523921	0.008802564	0.531352814

pcaac402.lysopcac204	0.051561714	0.082167346	0.531362455
c142.pcaec406	-0.002652992	0.004227897	0.53137808
pcaec364.lysopcac204	-0.011354554	0.018103377	0.531567843
pcaec361.pcaac323	-0.047678803	0.07602774	0.531621843
c141oh.spermidine	0.035670964	0.056881235	0.531628861
lysopcac280.pcaac321	0.009462146	0.015088426	0.531628994
arginine.pcaec385	0.019340729	0.030840988	0.531630059
smc161.pcaac361	-0.02718497	0.0433498	0.531632704
alphaaminoadipicacid.smc241	-0.011100477	0.017702619	0.531668209
pcaac405.smohc222	-0.003804058	0.006066795	0.531683545
citruline.pcaec340	0.01874353	0.029895019	0.531716878
pcaec383.c101	0.011548377	0.018421422	0.531768544
pcaec443.lysopcac180	-0.035849275	0.057188092	0.531790258
phenylalanine.c51	-0.020583587	0.032837606	0.531814042
pcaac343.methionine	-0.01631336	0.026030439	0.531896705
pcaac366.c3	0.013640194	0.021765484	0.53190588
pcaec322.lysopcac161	0.038377386	0.061239558	0.531914244
phenylalanine.leucine	-0.010963698	0.017496445	0.53194828
pcaac343.lysopcac204	-0.010634431	0.016972797	0.531992064
pcaac380.phenylalanine	0.016115674	0.025726727	0.53208345
pcaec386.c12	0.023846464	0.038068546	0.532089324
histidine.c182	0.036684202	0.0585814	0.532219794
methioninesulfoxide.pcaec363	0.019802015	0.031628069	0.532297693
pcaec365.lysopcac161	-0.049099327	0.078423341	0.532303835
c10.smc180	-0.008305741	0.013270591	0.532437767
pcaec362.pcaac403	-0.001734728	0.002771758	0.532449099
pcaac421.smc261	0.017744231	0.028355234	0.532498256
pcaec380.lysopcac161	0.031403837	0.050184071	0.532504947
pcaac366.smc241	0.006849869	0.010946498	0.532514167
smc160.pcaac360	-0.036321259	0.058046886	0.532537803
c141oh.smohc141	0.008655234	0.013833979	0.532585203
lysopcac180.smohc222	0.002984385	0.004770184	0.532596539
pcaac402.pcaac321	0.006522666	0.010426146	0.53261379
pcaec340.pcaac420	0.051666036	0.08258788	0.532625567
pcaec340.pcaec422	-0.019344354	0.030922366	0.532632454
ornithine.pcaac405	0.016401894	0.026223294	0.532702883
pcaec403.pcaec340	-0.028147641	0.045006988	0.532745005
pcaac381.pcaec386	-0.03094811	0.049488657	0.532776653
histidine.lysopcac182	-0.011226741	0.017952971	0.532787097
h1.taurine	-0.031692551	0.0506813	0.532794564
lysopcac181.pcaac424	-0.001952296	0.003122126	0.532808057
smohc161.c14	-0.019532031	0.031238984	0.532850103
pcaac426.pcaac365	0.030815645	0.049292904	0.53291005
c101.pcaec365	-0.008510127	0.013612956	0.532913106
pcaac405.pcaec406	0.00321246	0.005139494	0.532975441

pcaec384.pcaac381	-0.019644117	0.031428311	0.53298104
pcaac403.pcaec384	-0.027356889	0.043774806	0.533046011
glutamicacid.pcaec361	0.001722155	0.002755707	0.533049141
pcaec403.smc161	0.049087233	0.078560322	0.533118686
methionine.lysopcac281	-0.006725248	0.010763666	0.533134869
pcaac341.pcaec361	-0.007056844	0.011294441	0.533137067
pcaec364.threonine	0.016123089	0.025806872	0.533168047
pcaec381.pcaac322	0.026849379	0.042975669	0.533169257
pcaec361.smc241	0.014263586	0.022832396	0.53320161
smc180.smc240	-0.01461552	0.023397235	0.533227509
isoleucine.arginine	0.032458012	0.051962918	0.533247591
smc181.pcaac420	0.035828853	0.057360686	0.533256833
pcaec364.methioninesulfoxide	0.004500244	0.007207042	0.533388188
pcaac426.serotonin	-0.048209812	0.077212839	0.53341934
pcaec422.pcaac300	-0.028312427	0.0453617	0.533568156
histidine.smc241	0.006177883	0.009898634	0.533590154
c14.pcaac383	0.002443516	0.003917009	0.533781905
pcaac361.pcaac344	-0.037754355	0.060525433	0.533811467
lysopcac160.ornithine	-0.042180924	0.067624916	0.533830095
c101.smc180	0.009410654	0.015088345	0.533859372
pcaac425.pcaac385	-0.014668165	0.023517922	0.533860677
pcaac420.tyrosine	-0.029566036	0.04741587	0.533961733
lysopcac281.pcaac342	0.006014879	0.009649822	0.534113842
pcaac381.c14	-0.025261401	0.040532453	0.534163722
c51dc.pcaac422	0.009096091	0.014598861	0.53427535
c0.pcaec421	0.001248676	0.002004189	0.534298491
c5.pcaac300	0.046330476	0.074364235	0.534306516
lysopcac181.pcaac360	-0.023021481	0.036955605	0.534353228
pcaac323.asymmetricdimethylarginir	0.015069349	0.024192599	0.534391749
lysopcac160.pcaec420	0.023687401	0.038040983	0.534529202
pcaec360.pcaec445	-0.02799863	0.04497135	0.534590101
smc260.threonine	-0.021033532	0.033786946	0.534625427
pcaec445.smc241	-0.045416909	0.07296386	0.534675679
pcaac405.c16	0.014389001	0.023118211	0.534707005
pcaac366.pcaec322	-0.044758887	0.071917955	0.534739386
smohc141.pcaac385	-0.012705759	0.020416019	0.534751027
methioninesulfoxide.serine	0.097718923	0.157054459	0.534846031
pcaac321.lysopcac281	0.006720169	0.010801453	0.534874543
leucine.lysopcac170	-0.004819651	0.007747041	0.534891763
pcaec362.alanine	-0.006943386	0.011161493	0.534920598
pcaac323.pcaac362	0.011846428	0.0190434	0.534926299
c12.pcaac343	0.009205764	0.014798875	0.534937382
pcaac364.pcaec362	-0.008256044	0.013273915	0.534992001
lysopcac203.smohc141	0.011932261	0.019186167	0.535028076
acetylornithoine.pcaec443	0.019007179	0.030565126	0.535068503

pcaec363.pcaec340	-0.04068965	0.065433399	0.535075079
pcaec343.putrescine	0.016500934	0.026537298	0.535105735
pcaac321.lysine	0.037612873	0.060494764	0.535136801
pcaec322.pcaec341	0.040224766	0.064702645	0.535181137
pcaec322.pcaac405	-0.028157059	0.045296443	0.535226479
pcaec423.smc241	0.0101661	0.016358541	0.535332886
c14.pcaac422	0.010916796	0.017568783	0.535385721
glycine.smc160	-0.045307321	0.072928468	0.53546287
pcaac360.pcaac424	-0.005570687	0.008967722	0.535504789
pcaac421.smc181	0.006191702	0.009968359	0.535542547
smc260.lysopcac281	-0.008228981	0.0132486	0.53555218
pcaac341.threonine	0.011518595	0.018545694	0.535570633
smohc141.smc241	-0.045009951	0.072475184	0.535605545
c162.pcaec386	-0.006230684	0.01003304	0.535620615
pcaec402.pcaac384	-0.022511754	0.036250551	0.535628526
lysopcac140.ornithine	-0.015152118	0.024399925	0.535637779
pcaac421.prolinr	0.013391707	0.021565674	0.535648991
pcaec321.pcaac385	-0.016381584	0.026383587	0.535696676
smc241.putrescine	0.017473743	0.028144582	0.535725672
pcaec322.serotonin	0.043645797	0.07030735	0.535772046
pcaec425.smc260	-0.020436323	0.032920872	0.535781679
arginine.pcaac426	-0.02786808	0.044897667	0.535826837
pcaac381.creatinine	0.008740065	0.014082291	0.535866158
pcaec342.pcaac343	-0.012215543	0.019688388	0.535996229
pcaac402.c51dc	-0.081057333	0.130672955	0.53608624
c8.pcaac422	0.009719033	0.015668828	0.536105025
lysopcac140.pcaec342	0.04453358	0.071797816	0.536114573
lysopcac170.valine	-0.036092706	0.058203858	0.536216459
pcaac343.c101	-0.003314778	0.00534554	0.536220944
c5.isoleucine	0.014698858	0.02370677	0.536269366
pcaec422.kynurenine	-0.003453733	0.0055703	0.536270297
pcaec383.pcaac322	0.035734395	0.057640985	0.536322012
pcaac361.transhydroxyproline	-0.01386592	0.022367651	0.536347047
smc240.lysopcac281	0.006312212	0.010184162	0.53641455
pcaec300.arginine	-0.023953177	0.038647886	0.536432247
c51.pcaac380	0.02253927	0.03636876	0.536456634
pcaec340.pcaac422	-0.018505181	0.029861867	0.536489415
pcaec406.pcaec446	0.075725423	0.122217761	0.536554036
pcaac406.pcaac366	0.03152412	0.050880248	0.536566807
pcaac406.pcaec322	-0.03096637	0.049987557	0.536628031
c16.pcaec425	-0.009982328	0.016114817	0.536648587
pcaac402.pcaec321	-0.02789819	0.045040872	0.536683433
lysine.pcaac386	0.010865483	0.017544547	0.536741805
pcaac361.phenylalanine	0.018560982	0.029973002	0.536775716
pcaec362.lysopcac170	-0.004892882	0.007901682	0.536799608

pcaac323.smc241	0.00684475	0.011053962	0.536804819
pcaec444.pcaac424	0.008332829	0.013459545	0.536877409
pcaac402.smc202	-0.044859586	0.072470738	0.536942489
pcaec382.c18	0.031333258	0.050619517	0.536947189
c51dc.pcaec445	0.024043289	0.038845971	0.536984313
smc160.asymmetricdimethylarginine	0.012656637	0.020451942	0.537044338
lysine.pcaac341	0.001916563	0.003097167	0.537067938
smohc221.smc241	0.007222821	0.011675586	0.537189992
pcaac424.pcaac321	0.007662	0.01238602	0.537206653
pcaec444.pcaec363	-0.020906171	0.033803382	0.537296492
pcaec341.c51	-0.024057809	0.038903901	0.537344599
pcaec404.c101	0.010895893	0.017621262	0.537379301
pcaac321.threonine	0.01216137	0.019667925	0.537381059
c141.pcaac425	-0.029995477	0.048521296	0.537475147
asparticacid.pcaac323	0.060748464	0.098272975	0.5374959
pcaac401.lysopcac160	-0.027833601	0.0450277	0.537506719
pcaec422.pcaac424	-0.009336607	0.01510545	0.537538707
pcaac401.pcaec342	0.052367777	0.084726109	0.537546712
pcaac300.alanine	-0.013089502	0.021177768	0.537550371
pcaac420.lysine	-0.054888775	0.088819411	0.537613327
leucine.smohc221	-0.028688279	0.046427724	0.537658727
pcaec424.pcaac406	0.019933169	0.032260476	0.537678854
glutamicacid.pcaec301	0.016415916	0.026569413	0.537699814
pcaac386.pcaec402	0.017379377	0.028139337	0.537852062
c12.pcaac322	-0.016453306	0.02664051	0.537861119
acetylornithoine.c51	0.025438916	0.041191719	0.537881645
c2.pcaec403	0.016874261	0.027324701	0.537899742
serine.pcaec301	0.028342512	0.045896538	0.537909942
phenylalanine.smc161	0.036311257	0.058808436	0.537963003
pcaec300.pcaec342	0.058866758	0.095346569	0.537997222
pcaec361.smc181	0.002920936	0.00473157	0.538042444
spermidine.lysopcac180	-0.063439868	0.102775442	0.538083332
glycine.pcaec422	0.015803206	0.025603169	0.538103368
smc180.methioninesulfoxide	0.004784899	0.007752258	0.538109787
pcaec300.pcaec422	-0.017510704	0.028375296	0.538185854
pcaec424.smc181	0.003953014	0.006406541	0.538240494
pcaec403.pcaac366	-0.007650477	0.012399211	0.538250106
pcaec322.asparagine	0.005376438	0.008714017	0.538267124
lysopcac281.pcaec361	0.018181759	0.029469659	0.538281569
c162.glutamicacid	-0.027665184	0.044842858	0.538300711
methioninesulfoxide.smc261	0.017977966	0.02914983	0.538427532
pcaac362.lysopcac160	-0.025039139	0.040600927	0.538447231
pcaac424.pcaec322	0.031146813	0.050507866	0.538474172
pcaec343.c102	0.011397435	0.018487217	0.538585321
pcaac362.pcaec405	-0.028109159	0.045597696	0.538613922

pcaac322.pcaec363	0.016370763	0.026556889	0.538626277
pcaec300.smc181	0.020287447	0.032910748	0.538628208
c14.pcaec385	-0.024139201	0.039160509	0.538642294
pcaac281.citruline	0.041104988	0.066685209	0.538651217
pcaec405.pcaec406	0.020071295	0.032563123	0.538665848
pcaec360.smc181	0.016742784	0.027168941	0.538753972
pcaac362.pcaac384	-0.026089935	0.042339096	0.53877596
arginine.pcaec300	0.010055266	0.016318544	0.538793923
pcaac363.c4	-0.008529997	0.013845022	0.538847074
pcaec383.pcaac422	-0.076045384	0.123447458	0.538907304
ornithine.lysopcac204	0.013796325	0.022401204	0.538999389
smohc221.pcaec421	0.000681666	0.001107291	0.539169502
pcaac240.putrescine	-0.016193837	0.026312139	0.539277846
pcaac360.pcaac366	-0.009097785	0.014784249	0.539331221
lysopcac180.smc240	-0.02990309	0.048600741	0.539390187
pcaac322.pcaac343	-0.012654259	0.02056958	0.539447904
c101.pcaec361	-0.004455718	0.00724375	0.539501112
c3.putrescine	-0.018396163	0.029907595	0.539509008
pcaec341.pcaac425	-0.043987385	0.071518351	0.539541755
smohc222.glycine	-0.052588593	0.085503227	0.539543251
pcaac365.c51	-0.031293544	0.050883002	0.539568453
lysopcac170.pcaac240	0.005745191	0.009341965	0.539583174
pcaac321.pcaac385	-0.021255323	0.034563096	0.539593581
lysopcac280.pcaec362	0.007047031	0.011459319	0.539600736
pcaac424.c51	-0.047556153	0.077336274	0.539623044
pcaac364.pcaac320	0.046150408	0.075053316	0.53963965
pcaac405.pcaac404	0.016552082	0.026919184	0.539653561
arginine.c8	-0.02610683	0.042463084	0.539698435
asparagine.pcaac424	-0.007591506	0.012347969	0.539707944
pcaec383.pcaac362	-0.006724373	0.010939813	0.539792298
c141oh.methionine	0.013961631	0.022716392	0.539834369
c101.c4	0.00852088	0.013865197	0.539870149
pcaac300.pcaec341	-0.040563651	0.066016894	0.539941397
putrescine.h1	-0.001256245	0.002044527	0.539941829
pcaac323.pcaac383	-0.002976579	0.004845047	0.539999717
alphaaminoadipicacid.lysopcac170	-0.003437662	0.005595758	0.540013856
c181.pcaec443	-0.038084974	0.061999334	0.540048844
pcaec404.pcaec383	0.146575267	0.238628726	0.540075555
h1.pcaac385	0.014187183	0.023100376	0.540132255
pcaec365.threonine	0.023518663	0.038295239	0.540140889
pcaac420.prolinr	0.013023465	0.021208168	0.540182286
pcaec423.phenylalanine	-0.024659588	0.040159185	0.540203239
pcaac424.ornithine	0.031102225	0.050652721	0.540214639
smohc161.lysopcac182	-0.015201905	0.024760937	0.540268432
pcaec364.pcaec383	0.023278471	0.037919899	0.540309088

prolinr.glutamicacid	0.032979686	0.053736583	0.540412279
pcaac360.c102	0.019562373	0.031876767	0.540439484
pcaec444.pcaac403	0.024962096	0.040677064	0.540454217
c10.asymmetricdimethylarginine	0.005803012	0.009457463	0.540503229
pcaac420.taurine	-0.025988515	0.042355254	0.540507544
pcaec444.pcaec423	0.007415158	0.012086339	0.540552754
pcaac386.c51dc	-0.057497007	0.093727258	0.540595848
pcaac363.pcaac425	-0.053629259	0.087423285	0.54060018
smohc222.acetylornithoine	-0.033030371	0.053845663	0.540611316
creatinine.pcaec383	0.034858643	0.056827914	0.540624308
pcaac421.c102	-0.016975531	0.027680109	0.540711049
pcaec446.pcaac404	0.002326508	0.003794204	0.540777862
c5.pcaec342	-0.054310952	0.088582554	0.540819351
pcaec386.serine	0.04278766	0.069802146	0.540902598
smc160.smohc141	0.044385816	0.072416971	0.540945316
pcaac384.pcaac281	0.000822386	0.001342281	0.541104749
pcaac322.pcaac422	-0.0136579	0.02229536	0.541163421
arginine.c51dc	-0.059325451	0.096852016	0.541197865
asymmetricdimethylarginine.pcaec36	-0.008129483	0.013272998	0.54123366
pcaec384.pcaac386	0.007794081	0.012725603	0.5412405
pcaac344.lysopcac204	-0.009273199	0.015141435	0.541262831
pcaac364.c16	0.012320048	0.020118291	0.541301147
pcaac342.smohc141	0.009180961	0.014992826	0.541316647
lysopcac170.lysopcac182	-0.017342187	0.028320514	0.541318445
pcaec342.methioninesulfoxide	0.004907513	0.008016556	0.541438455
leucine.phenylalanine	0.0508581	0.083090891	0.541500707
smc181.lysopcac204	0.019768812	0.032301318	0.541543838
pcaec420.alphaaminoadipicacid	0.00223431	0.003650766	0.54154473
methioninesulfoxide.lysopcac161	0.083192954	0.135942629	0.5415712
smc180.pcaac323	-0.058986592	0.096391754	0.541587434
pcaec384.c12	0.026072843	0.042606479	0.541588062
pcaac363.pcaec382	0.00429548	0.007019681	0.541605174
glycine.pcaac360	-0.025259282	0.0412835	0.541651506
pcaac386.pcaec380	-0.035574992	0.058148416	0.541686424
alanine.pcaac383	0.003293267	0.005383198	0.541705247
c51.smohc221	0.025879501	0.042303633	0.54171308
pcaec403.lysopcac182	-0.025887082	0.04232008	0.541751758
pcaec403.h1	0.001320096	0.002158185	0.541770175
pcaac424.c0	0.000620025	0.001013684	0.541779804
pcaec386.smc241	0.030575726	0.04999913	0.54186539
pcaac365.c101	-0.003214412	0.00525789	0.541980895
alanine.c141	0.016460922	0.026925663	0.541982997
pcaec443.pcaac363	-0.009018222	0.014752488	0.542012803
lysopcac203.smohc161	-0.013679497	0.022383352	0.542115596
c101.methionine	0.031083531	0.050863084	0.542131705

lysopcac204.pcaac424	-0.002456456	0.004019819	0.542155076
pcaac401.pcaac384	0.020945544	0.034276712	0.542164384
glutamicacid.h1	0.002919958	0.00477849	0.542170496
pcaac384.pcaac385	-0.00916005	0.014993159	0.542245898
creatinine.glycine	-0.018948009	0.031018019	0.542297123
c181.c141oh	0.078263888	0.128120463	0.542303268
c16.pcaec445	-0.039138677	0.064071255	0.542303268
asparticacid.pcaec445	0.036110805	0.059120666	0.542345108
smohc241.pcaec343	0.030154311	0.049371283	0.54236633
glutamine.pcaac404	-0.003944919	0.006459031	0.542370336
lysopcac203.pcaec301	0.02554787	0.041832736	0.542400284
pcaec401.tryptophan	-0.003364054	0.005509093	0.542451884
c181.pcaac362	0.00408604	0.006693149	0.54255466
c4.smohc161	0.009327707	0.015279806	0.542568631
pcaac386.pcaac401	0.01640213	0.026869513	0.542584061
c142.pcaac362	0.002669927	0.004373931	0.542596163
pcaec342.smc260	0.020634128	0.033804068	0.542605685
alanine.asymmetricdimethylarginine	-0.007259878	0.011894495	0.542637048
leucine.pcaac384	-0.03023729	0.049542964	0.542657905
pcaec406.c101	-0.032656612	0.053510377	0.542683667
ornithine.threonine	0.012340982	0.020224693	0.542744304
lysopcac280.lysopcac180	-0.026837257	0.043982931	0.542757116
smohc241.c16	-0.032138764	0.052675817	0.54279059
c162.alphaaminoadipicacid	-0.004279343	0.007014136	0.542804448
pcaec364.c3	-0.009388565	0.015391134	0.54287341
pcaac281.smc241	0.017705772	0.029026261	0.542877913
lysopcac170.c162	0.040612811	0.066586224	0.542919751
c16.pcaac424	0.003833846	0.006286096	0.542942922
pcaec364.lysopcac161	0.053906122	0.088390387	0.542962057
pcaec363.pcaec383	-0.031449659	0.051574657	0.54301165
c141oh.asparagine	-0.00736984	0.012087006	0.543049018
pcaac322.pcaec405	-0.022786792	0.037372968	0.543061727
pcaac360.c51dc	-0.067262344	0.110318979	0.543065285
c8.pcaac322	0.0191788	0.031458353	0.543098954
pcaac384.ornithine	-0.012925232	0.021209726	0.543267888
pcaac344.pcaac383	-0.004081683	0.006697945	0.543273027
pcaec362.c102	0.019108125	0.031362247	0.543353524
pcaac320.pcaec364	0.007195359	0.011810464	0.543377064
pcaac404.pcaec406	0.005617508	0.009220866	0.543389582
pcaec364.c18	-0.017921079	0.029422002	0.543463795
pcaac366.smc261	0.023899714	0.039244988	0.543541114
pcaac384.c142	0.019230364	0.031582321	0.543601409
pcaec425.pcaec445	0.043914478	0.072122441	0.543607104
pcaec361.pcaac364	0.027771356	0.045612099	0.543625895
pcaec360.taurine	0.021954284	0.036058616	0.543632196

pcaec301.pcaac321	0.006981566	0.011467002	0.543638907
smc181.smc160	-0.055226497	0.090743748	0.543798446
pcaac420.tryptophan	0.009288398	0.015265335	0.543887666
acetylornithoine.pcaac420	-0.042862947	0.070445163	0.543891017
pcaec385.pcaac363	0.00482926	0.007937083	0.543901394
pcaec300.smohc221	0.037879493	0.062258053	0.543911608
pcaec405.lysopcac160	0.050614017	0.083193097	0.543934858
pcaac300.smc240	0.032188882	0.052911007	0.543956759
pcaec424.pcaec301	0.031813588	0.052297593	0.543983529
lysopcac181.pcaec423	-0.020346989	0.033449401	0.544001268
c51dc.pcaac365	0.01311339	0.02156389	0.544116015
pcaac422.smc160	-0.020511617	0.033740025	0.544239515
prolinr.pcaac300	0.027040515	0.044491991	0.544351868
pcaac424.lysopcac281	0.007915312	0.013023722	0.54435205
pcaec321.c101	-0.004128759	0.006795208	0.544459589
citruline.arginine	0.015626957	0.025719773	0.544468317
pcaac421.pcaec420	0.016330275	0.026877679	0.544473466
pcaac344.pcaac240	0.017234736	0.02836766	0.544492538
c8.smohc222	-0.001241162	0.002043401	0.544590678
pcaac323.methionine	-0.02215679	0.036483444	0.544649933
pcaec445.pcaec322	-0.034780179	0.05727013	0.544656747
kynurenine.pcaac361	-0.017707161	0.029159041	0.544682591
smc180.lysopcac181	0.021697528	0.035737023	0.544760123
pcaec386.c101	-0.00641789	0.010570843	0.5447687
pcaac426.lysopcac182	-0.008385688	0.013814375	0.544838219
c181.smc261	-0.037238675	0.061347767	0.544849316
pcaac404.lysopcac204	-0.006486029	0.010687585	0.544938226
glutamicacid.serine	0.017185296	0.02831795	0.544942038
pcaec385.pcaec402	-0.028445976	0.046879842	0.544997976
pcaec383.pcaac321	0.006766558	0.011152702	0.545041413
pcaac386.serine	0.026197975	0.043184234	0.545083219
asparticacid.smc202	0.031917344	0.052611933	0.545083231
pcaec300.pcaec420	0.014199739	0.023408882	0.545122795
c102.pcaac422	0.010475307	0.017271617	0.545183755
glutamicacid.asparticacid	0.051622887	0.085153103	0.545361159
pcaac404.c162	-0.036432413	0.060096895	0.545366615
smc240.pcaac424	0.004426849	0.007302504	0.545378677
serine.pcaac380	0.027908062	0.046038967	0.545395995
c18.c142	0.022399179	0.036951662	0.545401516
smohc221.acetylornithoine	-0.041521911	0.068500828	0.545416873
c14.pcaec406	-0.002822567	0.004656713	0.545432364
leucine.pcaac405	0.040253744	0.066415711	0.545459436
pcaac420.glycine	-0.02090678	0.034495373	0.545467882
lysopcac203.pcaac383	0.003307924	0.005458588	0.545515044
pcaac402.pcaac366	-0.012385383	0.020438536	0.54552948

pcaac421.pcaec383	0.046525132	0.076789898	0.545599793
pcaac425.leucine	0.010798781	0.017823734	0.545606691
lysopcac280.pcaac405	-0.033038017	0.054537192	0.545657371
pcaec322.ornithine	-0.01275876	0.021062879	0.545685526
isoleucine.pcaec383	0.042577769	0.070298337	0.545734349
lysopcac280.pcaac300	-0.022154965	0.036595382	0.545912626
pcaec446.pcaec384	0.013738916	0.022694642	0.545927088
pcaec364.pcaec406	0.031186994	0.051525248	0.545996987
c141.pcaec383	0.047133163	0.077872441	0.546006794
pcaac361.pcaac380	-0.022851117	0.037755131	0.546017272
pcaec365.lysopcac160	-0.008355707	0.013806964	0.546060138
c4.lysopcac182	0.028936504	0.047818646	0.546093576
pcaec361.c51dc	-0.066094349	0.109237072	0.546143886
pcaec420.pcaec300	-0.028007126	0.046289155	0.546148495
alanine.pcaac361	-0.014077629	0.023267652	0.546159863
putrescine.pcaac422	-0.008769529	0.014494996	0.546177208
pcaac361.smc241	0.02590401	0.042820224	0.546214224
asparagine.serine	-0.002417887	0.003997032	0.546232407
histidine.c18	0.021117865	0.034913689	0.546273102
pcaac364.pcaac322	0.028219259	0.046657051	0.546297042
pcaec420.lysopcac170	-0.007692785	0.012719268	0.546303338
c0.pcaac362	0.015665521	0.025907891	0.546403643
pcaec364.pcaec321	0.02958113	0.048922412	0.546409073
pcaec401.c8	-0.021924551	0.036260732	0.546420835
pcaec404.pcaec301	0.029694752	0.049112559	0.546427301
c14.c142	0.040284741	0.0666365	0.54648149
pcaec340.creatinine	0.003028067	0.005010135	0.546585139
pcaec405.pcaec301	0.045597685	0.075444627	0.546586576
prolinr.serine	-0.004188374	0.006930719	0.546630233
smc161.asparticacid	0.012598009	0.020848919	0.546675207
pcaec340.pcaac380	-0.027073354	0.044820951	0.546820322
putrescine.alanine	-0.00634445	0.010505716	0.546905778
asparagine.pcaac422	-0.002650164	0.004388914	0.546954342
pcaac363.lysopcac170	-0.004196963	0.006950841	0.546970742
c102.putrescine	-0.008697735	0.014404922	0.546973116
pcaac362.c141oh	0.049261436	0.081586079	0.546977032
pcaec382.c51	-0.046326414	0.076730842	0.547006795
asymmetricdimethylarginine.lysopca	-0.017991027	0.029802298	0.547055186
glycine.pcaec421	0.001092702	0.001810311	0.547108248
pcaac240.c2	-0.068821854	0.114022345	0.54711953
lysopcac204.c5	0.010888707	0.018045137	0.547230354
c14.pcaac322	-0.018583041	0.030799626	0.547271609
pcaec444.phenylalanine	-0.025351857	0.042026131	0.547346209
lysopcac280.asymmetricdimethylarg	-0.011092155	0.018387663	0.547347126
pcaec384.ornithine	-0.018359287	0.030435048	0.547354084

citruline.isoleucine	0.008468714	0.014038984	0.547354134
pcaec444.lysopcac170	-0.014991804	0.024853874	0.547374363
pcaac420.pcaac363	0.005784011	0.009589953	0.547417818
pcaac364.pcaec444	-0.041634109	0.069034097	0.547442693
asparticacid.pcaac421	0.024557819	0.040728325	0.547527788
smohc141.smc202	0.039568651	0.065633041	0.547587206
c102.pcaec343	0.03143473	0.052152762	0.547675884
c141.citruline	0.035039528	0.058149234	0.547784679
pcaac240.smohc241	0.036705017	0.060917845	0.547815371
pcaac341.pcaec422	0.006962891	0.011557076	0.54785149
c142.pcaec446	-0.018810321	0.031227865	0.547932267
isoleucine.pcaec423	0.008342504	0.013850599	0.547956213
kynurenine.pcaac381	-0.019597093	0.032544347	0.54805909
pcaec402.asymmetricdimethylarginir	0.015695415	0.026067247	0.548094466
pcaec364.pcaac361	0.054417817	0.090378723	0.548096982
smc202.pcaac300	-0.021651931	0.035961107	0.548107419
pcaec364.serotonin	0.04265699	0.07086281	0.548191817
pcaac384.pcaac321	-0.011040671	0.018341384	0.548199607
pcaec446.glutamicacid	0.034870881	0.057934419	0.548233739
asparticacid.pcaac344	0.033763256	0.056100396	0.548277781
pcaec423.pcaac366	-0.010579029	0.017584357	0.548424087
lysopcac181.pcaec364	-0.01189241	0.019770383	0.548483419
citruline.pcaec421	0.000987219	0.001641213	0.548489118
smohc141.pcaec343	0.028662576	0.047665352	0.548614333
serine.isoleucine	-0.011711262	0.019480635	0.548717125
pcaec364.alphaaminoadipicacid	0.001861786	0.003097939	0.5488492
arginine.pcaec406	-0.008102919	0.013483133	0.548854906
c8.pcaec363	-0.00830921	0.013827113	0.548875557
pcaec386.pcaac281	0.000702016	0.001168223	0.548881925
glutamine.smc240	0.028054839	0.046691983	0.548933237
pcaec401.pcaec342	-0.057023428	0.094944154	0.549098946
pcaec360.pcaec403	-0.00604918	0.010072072	0.549105733
smc260.methionine	0.017242366	0.028709989	0.549118481
lysine.pcaec421	0.000703719	0.001171894	0.549167363
acetylornithoine.c51dc	0.066012268	0.109930268	0.549170342
c141oh.pcaec342	-0.036001428	0.059955164	0.549183423
c12.glutamine	-0.011163295	0.018593279	0.549235516
lysopcac203.valine	-0.027981031	0.046619095	0.549360909
pcaec341.pcaac320	0.048628545	0.081021781	0.54937045
leucine.pcaec423	0.008443265	0.014067835	0.549376239
c51dc.c12	-0.01963624	0.032720195	0.549413639
acetylornithoine.pcaac366	-0.006683822	0.011137515	0.549419054
pcaec382.pcaac380	0.036165629	0.060274188	0.549485064
acetylornithoine.pcaec341	0.035681165	0.059475541	0.549543845
pcaac405.pcaec341	0.036625958	0.06105565	0.549578268

c4.alanine	0.004865019	0.008110318	0.549593337
pcaac323.lysopcac161	0.034513676	0.057544414	0.549647186
pcaec444.pcaec421	-0.001167247	0.001946229	0.54966503
c182.lysopcac170	0.007685558	0.01281507	0.549678033
smc261.pcaec301	0.029528095	0.049243347	0.549739128
c18.pcaac323	-0.037992943	0.063366129	0.549777769
pcaec380.pcaec362	0.008376152	0.013971259	0.549811482
c141oh.pcaac384	0.030625002	0.051086999	0.549851208
lysopcac182.pcaac404	0.004146694	0.006918382	0.549913823
pcaec406.smohc141	-0.009180123	0.015317533	0.549948487
isoleucine.phenylalanine	0.03930799	0.065598348	0.55001432
c51dc.pcaac281	0.00114255	0.001906759	0.550022028
c51dc.pcaec380	-0.024280693	0.040522739	0.55003784
c2.pcaec363	-0.006155698	0.010275626	0.550123355
lysopcac204.pcaec362	-0.009890646	0.016510349	0.550123932
smohc222.pcaec424	-0.010552908	0.017617039	0.550150699
pcaec322.pcaac422	-0.013011776	0.021724209	0.550193459
asparagine.tyrosine	-0.019443704	0.032464871	0.550218498
creatinine.c101	-0.006162239	0.010289889	0.550252832
methionine.lysopcac203	0.012468109	0.020820491	0.550269566
pcaec362.c2	0.035292078	0.058943446	0.550331829
pcaac360.smohc221	-0.030759925	0.051386051	0.550425057
smohc141.smc260	-0.020655274	0.034507475	0.550445446
pcaac281.pcaac426	0.021107358	0.035269726	0.550524261
pcaec403.lysopcac203	0.006332185	0.010581946	0.55056435
lysopcac160.c141oh	0.057173225	0.09556069	0.55063275
pcaec360.pcaac366	-0.005052149	0.008444502	0.550643153
pcaac323.serine	-0.011500971	0.019224125	0.550656102
pcaac343.pcaac320	-0.021433415	0.035827548	0.550668621
c2.glutamine	0.013038961	0.021799216	0.550734723
c162.c14	-0.016676986	0.027881689	0.550738053
prolinr.pcaec406	0.001177929	0.001969615	0.55079374
lysopcac204.pcaac386	0.013403909	0.02241311	0.550801413
pcaac404.pcaec420	-0.019120811	0.031976067	0.550845456
pcaac322.isoleucine	-0.010478982	0.017525748	0.550880947
glutamine.pcaec362	-0.005586588	0.009343752	0.550896669
pcaac366.pcaec343	0.03925434	0.06565446	0.550898419
c0.pcaac365	-0.028398416	0.047497948	0.550902244
lysopcac182.smc160	-0.035954576	0.06015209	0.551008327
c0.c8	0.020919556	0.03500142	0.551041894
pcaac402.c162	0.044543395	0.074531188	0.551061606
pcaec381.pcaac366	-0.009666944	0.016175308	0.551069485
pcaac380.pcaac300	-0.025890033	0.043336702	0.551215914
pcaec383.c12	0.041890348	0.070122631	0.551235128
smohc241.pcaac422	-0.01071417	0.017937762	0.551295076

smc180.isoleucine	0.011274568	0.018878351	0.551344889
alanine.pcaec406	0.001672357	0.002800627	0.551401713
pcaec383.pcaec364	0.002829428	0.004738444	0.551411743
arginine.pcaac424	-0.003122575	0.005229875	0.551449466
c141oh.c5	-0.004009895	0.006716025	0.551450344
pcaec446.pcaac363	0.004089495	0.006849616	0.551466051
pcaec360.pcaec384	-0.009269256	0.015526609	0.551498281
smc160.asparticacid	0.011141216	0.018662833	0.551510397
pcaac380.lysine	-0.055576021	0.09310201	0.551534765
pcaac361.pcaac323	-0.045151246	0.075639779	0.551542917
pcaec401.smc160	-0.020763894	0.034786284	0.551560048
pcaec404.pcaec384	-0.021971313	0.036817432	0.551649936
smc241.methionine	0.014413936	0.024155779	0.551687428
pcaac365.pcaac424	-0.006638007	0.011124436	0.551689134
asparticacid.pcaec340	-0.018369042	0.030788273	0.551742647
asparagine.smohc161	0.006650394	0.011148296	0.551799643
smc260.serine	-0.006518827	0.010928473	0.551826101
pcaec343.pcaac426	0.037310375	0.062557025	0.55187769
pcaec443.lysopcac281	0.006859056	0.011501736	0.551925765
pcaec423.pcaec365	-0.011618251	0.019484329	0.551967518
pcaac422.pcaec342	0.043213252	0.072486759	0.552056242
alphaaminoadipicacid.lysopcac160	0.00532462	0.008931787	0.552063527
leucine.pcaac383	-0.004914941	0.008244749	0.552072178
arginine.pcaac361	-0.021314147	0.035754446	0.552074868
pcaec422.pcaac422	-0.015391069	0.025818788	0.55207941
c101.smohc241	0.028946337	0.04856486	0.552135598
putrescine.pcaec406	0.002317473	0.003888344	0.552155099
pcaec382.pcaac362	-0.005953332	0.009991386	0.55226095
pcaec421.pcaac366	-0.009697575	0.016276204	0.552283171
c14.pcaac425	0.020239329	0.033972076	0.552316086
pcaec380.tyrosine	0.034513153	0.057936556	0.552354515
h1.histidine	0.02007708	0.033704526	0.55237247
smc161.pcaac386	-0.006547026	0.010992133	0.552418381
pcaec342.pcaec360	-0.003126884	0.005251009	0.552503433
pcaac425.pcaec406	0.013419883	0.022541761	0.552602185
c4.methionine	-0.014809166	0.024877967	0.552643436
pcaac402.serotonin	-0.054704612	0.09192436	0.552755232
pcaec322.pcaec401	0.035602263	0.059829169	0.552781434
c16.pcaac404	0.003230884	0.005430838	0.552881994
pcaec360.pcaec361	-0.004776463	0.008029681	0.552924357
lysopcac160.pcaac322	-0.039601975	0.066575012	0.552926655
c102.pcaec401	0.030819289	0.051816729	0.552975016
c101.pcaec402	0.019380833	0.032587775	0.553006873
lysopcac204.smc202	-0.019358413	0.03255189	0.55302894
alphaaminoadipicacid.pcaac424	0.003594316	0.006044427	0.553058579

pcaec364.pcaac321	-0.013464016	0.022643075	0.553078532
pcaec385.smc240	0.012394744	0.020846176	0.553104138
pcaac405.c4	-0.007591786	0.012769159	0.553131104
pcaec404.smc240	0.017444999	0.029346755	0.553195685
pcaac365.alphaaminoadipicacid	0.00173874	0.002925146	0.553217337
isoleucine.smc240	-0.013129836	0.022089877	0.55323663
pcaec340.pcaec444	-0.065475503	0.110182942	0.553329265
arginine.pcaec424	0.00642925	0.010821117	0.553398858
ornithine.lysopcac140	0.004550512	0.007659648	0.553432406
arginine.pcaec423	0.008503222	0.014315007	0.553486676
c3.h1	-0.00073878	0.001243951	0.553559379
smohc241.pcaec401	-0.012734437	0.021444281	0.55359947
c2.c8	0.025905625	0.043624045	0.553599575
c14.pcaac240	-0.005776716	0.009730031	0.553692004
c182.phenylalanine	-0.01735083	0.029226125	0.553708211
c182.pcaec360	-0.003464163	0.005836928	0.553831373
pcaec424.c4	-0.007267736	0.012246672	0.553861341
pcaec342.pcaac405	-0.022861989	0.038536377	0.553987073
glutamicacid.acetylornithoine	-0.021313971	0.035928083	0.55399874
pcaac281.pcaec301	-0.026010774	0.043851162	0.55405175
c2.pcaec385	0.029283484	0.049384195	0.554176951
pcaec360.creatinine	0.003906477	0.006588209	0.554192297
c181.pcaec422	-0.011380337	0.019202503	0.554393279
pcaec405.pcaac426	0.045707923	0.077129837	0.554418986
smc180.smc202	-0.029024909	0.048983276	0.554460906
asparticacid.asparagine	0.003590439	0.006059805	0.554491984
c101.pcaec421	-0.00121122	0.002044465	0.554533544
pcaac323.isoleucine	-0.007539826	0.01272848	0.554586804
pcaac364.pcaac420	0.033042161	0.055783667	0.554608118
pcaac386.pcaac361	-0.033792937	0.057051697	0.554611763
glutamine.pcaac366	0.008308847	0.014031146	0.554711714
citruline.pcaec420	-0.008843353	0.014933995	0.554717739
c14.pcaec382	-0.010824854	0.018282286	0.55476272
phenylalanine.pcaac404	0.00325093	0.005490748	0.554776767
pcaac360.pcaec382	-0.007815232	0.013201546	0.554830517
pcaac422.smc261	0.017925489	0.030280218	0.554835118
pcaac366.pcaac424	-0.010545585	0.017814686	0.554852898
prolinr.pcaec424	0.008698768	0.014696374	0.554893783
pcaac343.pcaac344	-0.036657256	0.061932191	0.554897469
c10.pcaac405	-0.028746569	0.048572102	0.554937871
ornithine.asymmetricdimethylarginin	-0.008049361	0.013601699	0.554965944
pcaac422.h1	0.001317669	0.002226603	0.55497024
pcaec341.pcaac424	-0.014517868	0.024539675	0.555088084
c181.pcaec385	-0.029396909	0.049692303	0.555107631
pcaec383.pcaec385	-0.035834477	0.060580718	0.555149381

c102.pcaac361	-0.021248465	0.03592486	0.555180464
isoleucine.c14	0.015265249	0.025809815	0.555192724
phenylalanine.smc202	0.025002938	0.042279822	0.555248303
leucine.pcaec380	0.020686057	0.034981052	0.555260172
lysine.pcaec424	0.004608433	0.007793077	0.555260672
putrescine.pcaac384	-0.046345135	0.078378444	0.555294129
pcaac422.pcaac420	0.081769176	0.138292962	0.555310473
c10.pcaac422	0.009579362	0.016201706	0.555322958
pcaec362.valine	0.025376033	0.042919925	0.555332994
pcaac322.lysopcac182	-0.006376278	0.010784586	0.555333901
pcaec403.pcaac385	-0.014198736	0.024015964	0.555346742
pcaac421.c14	-0.024386538	0.041257162	0.555436642
pcaac381.pcaac321	0.013147085	0.022246195	0.555506715
pcaec363.c4	-0.009361201	0.015840854	0.555525592
alphaaminoadipicacid.pcaec385	-0.024576723	0.041603979	0.555674544
serotonin.pcaec403	-0.03793536	0.064219896	0.555687714
pcaac341.smohc161	-0.008130139	0.013763715	0.555698887
pcaac321.c4	-0.010500784	0.017779537	0.555754367
pcaec423.pcaac422	-0.013119306	0.022215109	0.555789688
pcaec343.smohc241	-0.020394494	0.034535557	0.555804173
acetylornithoine.pcaec380	-0.025657279	0.043455811	0.555880265
c5.pcaac240	-0.014003072	0.023717273	0.555884035
c141oh.pcaac300	-0.021933815	0.037155842	0.555949004
c12.methionine	0.021656909	0.036689677	0.555980338
c141oh.pcaec384	-0.013478394	0.022838521	0.556055201
pcaac321.glutamicacid	-0.041763061	0.070774495	0.556104814
pcaac383.lysopcac182	-0.008212978	0.013918303	0.55610587
pcaac421.pcaec382	0.010973372	0.018597207	0.556125853
c51dc.pcaac362	-0.006783413	0.011496332	0.556128962
pcaac403.pcaec403	0.049303251	0.083566327	0.556169572
lysopcac181.asparagine	-0.002793585	0.004735579	0.556219921
lysopcac203.c3	-0.022945123	0.038895731	0.556220238
c181.pcaac363	0.004366341	0.007401912	0.556233558
pcaec301.pcaac362	-0.002514849	0.004263328	0.556243232
pcaac385.pcaac422	-0.013973006	0.023699038	0.556428455
asparagine.pcaec402	-0.015741179	0.026698624	0.556438218
c51.lysopcac281	0.008205771	0.01391822	0.556449731
pcaac361.pcaec405	-0.026818904	0.045491351	0.556471056
pcaec446.pcaac343	0.012245468	0.020774689	0.556535799
pcaac424.pcaec445	0.034515407	0.058569234	0.556624196
lysine.c162	-0.042824993	0.072673421	0.556643948
pcaac344.smc161	-0.023617523	0.040084987	0.556706637
c102.pcaec342	0.033403354	0.056697829	0.556732911
pcaec362.pcaac361	0.015554893	0.026404402	0.556762804
pcaec380.pcaec445	0.02977566	0.050544156	0.556763034

pcaac343.c182	0.01778213	0.030185744	0.556770804
smc202.c8	0.020082671	0.034091156	0.556772743
pcaac403.pcaac364	0.034404976	0.058406572	0.556791077
pcaec401.taurine	0.029006907	0.049245995	0.556817425
c8.kynurenine	0.004475703	0.007599596	0.556871673
pcaec403.c4	-0.012839455	0.021803924	0.556924974
pcaac402.c2	-0.062148374	0.105549428	0.556959488
methioninesulfoxide.pcaac384	0.02730868	0.046385363	0.557008577
c51.ornithine	0.01089783	0.018512975	0.557059301
pcaac422.pcaec405	-0.05183674	0.088070042	0.557108586
pcaac300.smc181	0.008573356	0.014566648	0.557125114
pcaac406.putrescine	0.013021239	0.022128109	0.557200592
pcaec380.pcaac424	-0.011038001	0.018759721	0.557240411
smc260.pcaac364	0.016965304	0.028833731	0.55724338
pcaec421.leucine	-0.038557174	0.065532205	0.557252799
c18.pcaac425	0.029133412	0.049520951	0.557296354
pcaec341.valine	0.028820768	0.048990691	0.557305765
c162.glutamine	-0.016292012	0.027700061	0.55739459
phenylalanine.lysopcac140	-0.0007099	0.001207034	0.557409176
pcaac361.lysopcac203	-0.038277401	0.065087242	0.557437558
lysopcac140.c182	0.020933749	0.035599717	0.557479344
c16.methioninesulfoxide	0.011849871	0.020151799	0.557479953
ornithine.smohc222	-0.002279971	0.00387772	0.557522451
arginine.prolinr	0.024110451	0.041009354	0.557550082
c16.smc260	0.01372973	0.023353989	0.557569531
pcaec401.lysopcac204	-0.018351359	0.031215907	0.557577391
pcaec445.pcaec424	-0.011772197	0.020024762	0.557579299
pcaac323.lysopcac182	-0.007501981	0.012762396	0.557621318
lysopcac170.pcaac321	-0.013677679	0.023271038	0.557664033
pcaac321.lysopcac140	0.000733233	0.001247573	0.557682814
c182.c4	0.007450722	0.012678047	0.557709908
pcaac385.pcaec422	0.010423034	0.017738855	0.557780021
smohc141.pcaec384	-0.044797516	0.076240861	0.557782236
smohc161.methioninesulfoxide	0.005824397	0.009912935	0.557798056
ornithine.pcaac341	0.001495211	0.002545076	0.55784055
pcaec386.pcaac363	0.004490063	0.007642882	0.557846394
c4.lysopcac140	0.001663451	0.002831512	0.557849809
c0.pcaac366	0.024578531	0.041845455	0.557925786
ornithine.pcaac404	-0.003631021	0.006181899	0.557926498
serine.pcaec382	0.005785687	0.009850358	0.557930024
glutamicacid.pcaac403	0.003782692	0.006440201	0.557931302
pcaec444.smc260	-0.020912084	0.03560809	0.557979201
pcaac281.pcaac322	0.030907204	0.052628444	0.557987758
pcaac321.pcaac300	-0.053927132	0.091828656	0.557996903
c3.pcaec403	0.02821196	0.048044661	0.558033999

pcaec342.serine	-0.009500047	0.016180839	0.5580914
glutamine.pcaac422	-0.005952714	0.010140281	0.558145392
pcaec341.putrescine	0.014149675	0.024107254	0.558205447
histidine.tyrosine	-0.033424266	0.056946882	0.5582116
pcaac386.pcaac426	-0.030838715	0.052545282	0.558238189
putrescine.smc241	0.004429119	0.00754761	0.558287664
pcaac320.lysopcac160	-0.012301872	0.020965359	0.558322937
c3.creatinine	0.007020806	0.011965494	0.558334318
c2.pcaac362	0.003696787	0.006301143	0.558380678
c10.arginine	-0.00733531	0.01250354	0.558398428
smohc221.prolinr	0.018766393	0.031989407	0.558408262
pcaec340.serine	-0.015748149	0.026849222	0.558477745
pcaec322.pcaec383	-0.035505357	0.060539989	0.558519605
c3.pcaec421	0.000444483	0.000757927	0.558541217
pcaac363.pcaac426	-0.031433414	0.053604401	0.558574278
pcaec362.pcaec425	-0.001748226	0.002981866	0.558647987
pcaec380.smc241	0.007753978	0.01322713	0.558693837
pcaac240.threonine	-0.011248579	0.019189495	0.558716245
c12.c14	0.027833084	0.047490308	0.558786595
lysopcac280.pcaec421	0.002543965	0.004341035	0.558821359
smohc141.pcaec385	-0.027518338	0.046962021	0.558859762
c141.serotonin	0.018851489	0.032178716	0.558948775
methioninesulfoxide.pcaac322	0.021556772	0.036802063	0.559007896
c5.c12	-0.021297202	0.036362225	0.559043551
lysopcac161.smc202	0.025301298	0.043209852	0.55914486
pcaac425.pcaac426	0.051978121	0.088773306	0.559164595
c141oh.pcaac362	0.002178047	0.003720026	0.559179812
pcaec321.pcaec422	-0.019940001	0.034065892	0.559284564
pcaac426.phenylalanine	0.01556582	0.026595249	0.559318391
pcaec424.pcaec405	-0.057825391	0.098806393	0.559349499
pcaec380.kynurenine	-0.005363568	0.009165461	0.559380273
pcaac344.c18	-0.022263036	0.038045455	0.559396408
glutamicacid.pcaac361	-0.027111922	0.046334431	0.5594191
pcaec403.pcaac362	-0.004342105	0.007421024	0.559437175
pcaec421.pcaec343	0.042998479	0.07349426	0.559470409
pcaec420.smc241	0.009520642	0.016273163	0.559475311
lysopcac203.pcaac420	0.045722792	0.078157475	0.559504233
pcaec384.pcaec360	0.003038338	0.005194356	0.559556546
smc241.pcaac360	-0.021371934	0.036543751	0.559623006
c141.kynurenine	-0.002551605	0.004363508	0.559670976
c5.lysopcac181	-0.038512229	0.065875118	0.559761558
pcaac384.lysopcac203	-0.005323307	0.009105521	0.559762062
pcaac361.pcaac360	-0.022502885	0.038496967	0.559820733
pcaac361.c182	0.012901615	0.022072217	0.559832982
pcaec444.alanine	-0.005799642	0.009922116	0.559834097

pcaec423.lysopcac281	0.004134287	0.007074007	0.55988982
leucine.lysopcac161	-0.043529262	0.074493569	0.559955372
lysopcac161.pcaec342	0.050017243	0.085609382	0.560013249
pcaac384.pcaec402	-0.013146362	0.022505861	0.560093023
pcaac404.pcaec385	0.032274164	0.055254117	0.560110724
lysopcac160.pcaac402	-0.034415727	0.058936249	0.560215281
c12.pcaac362	0.008828636	0.015119951	0.560243435
pcaac380.h1	-0.001832121	0.003137858	0.560263665
c18.lysopcac180	0.033503142	0.057387659	0.560312177
prolinr.pcaac321	-0.013616087	0.023323632	0.560321994
putrescine.pcaec365	-0.009805305	0.016796643	0.560337795
pcaac406.pcaec402	0.016642675	0.028510867	0.56036116
pcaec361.isoleucine	-0.009758295	0.016717883	0.56037924
pcaec424.smc161	-0.034092173	0.058430348	0.560538355
smc241.pcaec403	-0.011306483	0.019382594	0.560629033
pcaec342.glutamicacid	0.014877867	0.025505239	0.560632972
pcaec424.pcaac342	0.015814954	0.027117292	0.560713775
pcaec424.taurine	-0.02970364	0.050934492	0.560735346
c3.smc160	-0.040793053	0.069953992	0.560756963
c8.smc161	-0.029004737	0.049740365	0.560769347
c14.glutamine	-0.006551159	0.011235222	0.560790486
pcaec385.pcaec383	0.030430729	0.052192983	0.560823173
glycine.c12	0.021818581	0.037422409	0.560828118
pcaec343.pcaec446	-0.015027069	0.025779229	0.560909562
pcaec300.serine	-0.005491687	0.009421836	0.560940331
h1.smc261	0.015536588	0.026658279	0.560982389
pcaec364.smc181	0.011586636	0.019881514	0.560996478
pcaec404.pcaac321	0.005418664	0.009297899	0.560996878
pcaac422.pcaec404	0.013509339	0.023185028	0.561069833
pcaec322.pcaec446	-0.019212538	0.03297383	0.561079853
phenylalanine.valine	0.043585165	0.074806452	0.561094002
pcaec361.c12	0.027108325	0.046538806	0.561195046
pcaac422.pcaac320	-0.025088574	0.043071843	0.561199477
pcaac422.pcaac366	-0.012344609	0.021193178	0.561200627
lysopcac203.smc180	0.003649684	0.006266502	0.56124673
lysopcac181.pcaac386	0.014839202	0.025481308	0.561283841
c16.threonine	0.014099173	0.024210968	0.561290481
smc261.pcaec406	0.003406779	0.005850286	0.561303665
lysopcac204.lysopcac280	0.00394984	0.006783171	0.561321775
c101.c3	0.020461281	0.035153832	0.561489737
pcaec361.pcaac381	0.02035479	0.0349786	0.561576087
pcaec424.pcaac360	-0.02061491	0.035426553	0.56158658
alanine.creatinine	-0.002286902	0.003930241	0.561608407
c102.alanine	-0.012493464	0.021471428	0.561614119
c12.pcaac344	-0.024532347	0.042166259	0.561657239

pcaec443.pcaac364	0.024108848	0.04144552	0.561724871
smohc161.putrescine	0.014429069	0.024805048	0.561725432
pcaac366.c101	-0.004701594	0.00808375	0.561784815
c142.smohc161	0.002648466	0.004553691	0.561786151
pcaac424.pcaec342	0.043831526	0.075365517	0.561801321
pcaac366.pcaec301	0.054211734	0.093222787	0.561839725
pcaec404.transhydroxyproline	-0.043260594	0.0743967	0.561868882
pcaec343.isoleucine	0.009381049	0.016134145	0.561898862
pcaac366.c51dc	-0.064648743	0.111188069	0.561902083
pcaec406.smc160	-0.029311864	0.050413717	0.561908536
pcaec446.taurine	-0.016695142	0.028716234	0.561937406
pcaac240.pcaec401	0.015499113	0.026661956	0.561980392
smc261.methionine	0.010063103	0.017310821	0.561980907
pcaac341.pcaec301	-0.034672163	0.059651537	0.562030346
smc160.lysopcac203	-0.005853389	0.010070765	0.562043252
c2.kynurenine	-0.006623215	0.011398313	0.562148186
pcaec422.lysopcac203	-0.026162785	0.045030531	0.562194344
pcaec422.lysopcac182	-0.023984468	0.041285825	0.562237311
pcaac380.pcaec363	-0.01836986	0.031623693	0.562269552
lysopcac281.c101	-0.004803042	0.008268737	0.562283933
lysopcac161.pcaec321	-0.022102244	0.038063216	0.562415493
pcaec301.pcaac366	-0.004168211	0.007179094	0.562461162
pcaec385.pcaac300	-0.021941493	0.037791594	0.562469345
lysine.pcaac405	0.030391891	0.052355073	0.56253406
smohc161.pcaac403	-0.002633525	0.004536688	0.56253452
pcaec361.pcaec385	-0.027451066	0.047299775	0.562622838
pcaec385.c12	0.023242437	0.040050784	0.562649342
smc260.c51	-0.017559203	0.03025951	0.562674263
c182.pcaac420	-0.044923292	0.077416454	0.562678392
pcaec420.pcaac364	-0.024513967	0.042245576	0.562683726
spermidine.lysopcac203	0.048707036	0.083953894	0.562756939
pcaec425.pcaac380	0.038465755	0.066325198	0.562896283
pcaec342.pcaac386	0.008278795	0.01427607	0.562929746
pcaac300.methionine	-0.018117515	0.031247742	0.563000192
acetylornithoine.lysopcac281	-0.009490485	0.016369426	0.563022651
pcaac424.pcaac322	0.027437413	0.047326757	0.563039315
pcaec425.lysopcac170	-0.003729346	0.006433048	0.563057751
pcaac386.pcaac240	-0.008670861	0.014960651	0.56315124
smohc241.pcaec406	0.002793007	0.004819803	0.563213124
pcaac365.pcaec363	0.014672493	0.02532845	0.563345479
lysopcac204.pcaac300	-0.023190023	0.040033535	0.563361692
pcaec360.lysine	-0.051414136	0.088769521	0.563414239
pcaec422.pcaec342	-0.050389054	0.087032546	0.563561499
pcaec363.valine	0.023260556	0.040176104	0.563563524
pcaec425.smohc141	-0.008412674	0.014534737	0.563675981

c5.pcaec401	0.036624441	0.063282323	0.563710316
pcaac405.pcaec382	0.002095893	0.0036216	0.563728173
lysopcac182.pcaac422	-0.00662718	0.011452702	0.563771119
c10.pcaac323	0.056285594	0.097274168	0.563789995
isoleucine.pcaec421	0.000531703	0.000918994	0.56382831
lysopcac161.pcaac343	0.016876128	0.029169555	0.563840868
glycine.alanine	-0.006049542	0.010456445	0.563844909
c16.pcaec406	-0.003871052	0.006691178	0.563855714
histidine.serine	-0.008805083	0.015220346	0.5638714
c10.pcaac344	-0.024925863	0.043091646	0.563917848
pcaac364.pcaec385	0.026424546	0.04568598	0.563947005
c4.asparagine	-0.005029061	0.008695237	0.563964161
smc202.c12	0.019084451	0.033001798	0.564020969
pcaac300.smc161	-0.024511613	0.042397773	0.564122443
ornithine.pcaec383	-0.028193252	0.048771061	0.56416357
pcaec384.smc180	0.006778377	0.011726638	0.564191143
alanine.pcaac240	-0.00611222	0.010575508	0.564239913
lysopcac160.putrescine	0.020875446	0.036121502	0.564264826
asparticacid.pcaac300	-0.024669468	0.042689888	0.564296355
c8.transhydroxyproline	0.017955073	0.031076036	0.564361926
lysopcac180.pcaac362	-0.015406342	0.026665123	0.564366968
glutamicacid.pcaec421	0.00585589	0.010136706	0.564420594
pcaec386.smc202	-0.017734989	0.030701261	0.564439728
pcaec341.serine	-0.007965386	0.013789191	0.5644457
pcaec383.pcaac424	0.013620459	0.023579258	0.56445146
smohc222.c4	-0.011646276	0.020162189	0.564462392
pcaec386.lysopcac281	0.006862758	0.01188137	0.564477841
pcaec364.pcaec363	-0.024812613	0.042961169	0.564509847
c51dc.h1	-0.002809902	0.004865831	0.564565644
pcaec361.asparagine	0.00448493	0.007769893	0.564738891
pcaec384.pcaec406	0.005914368	0.010246814	0.564757788
pcaac386.lysopcac170	0.005597594	0.00969854	0.564779703
acetylornithoine.pcaac361	0.011127427	0.019281231	0.564811047
pcaac281.c0	-0.000237967	0.000412354	0.564823969
pcaec386.lysopcac161	-0.044141681	0.076539466	0.565076416
prolinr.serotonin	0.017526351	0.030390862	0.565089785
pcaec341.alphaaminoadipicacid	0.002053973	0.003561935	0.565125328
c16.pcaec403	0.014369755	0.024920792	0.565144328
pcaec361.smc202	0.027303157	0.047352052	0.565156378
smc202.histidine	0.02693886	0.046728333	0.565223578
pcaac422.pcaac401	-0.025190089	0.043699617	0.565265503
pcaec343.pcaac321	-0.014557309	0.025255463	0.56528902
c16.smc181	0.016767837	0.029096772	0.565372668
smc202.pcaac406	0.012837442	0.022276486	0.565373062
smc261.putrescine	0.021920018	0.03804168	0.565418292

lysopcac180.pcaec383	-0.057007567	0.098941109	0.565441067
pcaac425.pcaac321	0.007760188	0.013472517	0.56555928
pcaec364.smc202	-0.024088389	0.041820217	0.565561093
pcaac341.glutamicacid	-0.018887553	0.032798852	0.565654426
c18.pcaec401	-0.017751077	0.030830316	0.565717284
citruline.tryptophan	0.002320457	0.004030988	0.565792996
c5.tryptophan	0.005399268	0.009381404	0.565877838
pcaec444.lysopcac204	-0.007586017	0.013182673	0.565928531
pcaac422.pcaac424	-0.004831774	0.008396546	0.56593247
pcaec425.asymmetricdimethylarginir	0.010130592	0.017605026	0.565939401
c102.glutamine	-0.015614241	0.027140513	0.566024536
pcaec343.acetylornithoine	-0.042545084	0.073966801	0.566105181
pcaec406.transhydroxyproline	-0.03331875	0.057929134	0.566123827
pcaec300.c4	-0.004607845	0.008014502	0.566275883
isoleucine.pcaac320	-0.058618039	0.101957694	0.566284903
lysopcac180.asparagine	-0.004433822	0.007713047	0.566337584
pcaec405.pcaec445	0.031478231	0.054764101	0.566371434
alanine.pcaac403	0.004901613	0.00852772	0.566378823
lysopcac161.c12	0.034821335	0.060584608	0.566399324
pcaec423.glutamicacid	0.028324151	0.049280409	0.566399783
c102.pcaec406	-0.002761388	0.004804821	0.566428643
arginine.pcaac403	0.003132621	0.005451534	0.566483151
pcaec421.smc202	-0.039188437	0.068199865	0.566496329
putrescine.pcaec361	0.005301448	0.009226562	0.566514034
c4.lysopcac281	-0.009906707	0.01724318	0.566552098
c162.pcaec384	0.010410619	0.018121123	0.566570379
pcaec340.alphaaminoadipicacid	0.013516453	0.023528576	0.566592118
c18.pcaec363	0.008041392	0.013999324	0.566630402
smc260.pcaac384	-0.046472846	0.080925349	0.566728099
c162.asparticacid	0.009115866	0.015874225	0.566736378
smohc161.pcaec383	0.029563037	0.051482058	0.566747324
pcaec405.c51	-0.044773462	0.077978862	0.566791351
pcaac383.pcaec382	0.002763555	0.004813156	0.566796277
pcaac421.pcaec421	-0.001837822	0.003201126	0.566829736
smohc222.pcaac406	0.014547758	0.025346446	0.566938338
pcaac341.pcaac380	-0.026154487	0.045574039	0.566983194
lysopcac280.lysopcac170	0.020031578	0.034910034	0.567040084
alphaaminoadipicacid.h1	0.00402395	0.00701295	0.567051694
smohc221.glutamine	-0.009786918	0.017065935	0.567262091
pcaac425.smohc241	-0.027072384	0.047208895	0.567273841
smc160.pcaac323	-0.040478469	0.07058992	0.567292811
pcaac421.creatinine	-0.012658342	0.022077448	0.567340439
c141.pcaac426	-0.022632485	0.039473982	0.56734636
lysopcac160.c5	-0.006484531	0.011311111	0.567389116
pcaec322.c16	0.01892204	0.033007977	0.567410739

lysopcac181.pcaac321	-0.013130581	0.022906348	0.567429422
c14.lysopcac182	-0.013981411	0.024391206	0.567438671
pcaac385.serine	-0.013292927	0.023190237	0.567440706
c141.pcaac421	0.008965514	0.015641811	0.567465017
pcaec444.pcaec340	-0.015749475	0.027477674	0.56746683
pcaec445.pcaac426	-0.035695122	0.062294461	0.567579679
pcaac323.asparticacid	0.018449118	0.032199001	0.567603045
lysopcac280.pcaac422	-0.013045603	0.022769951	0.567630799
lysopcac181.putrescine	0.030665617	0.053555346	0.567856266
pcaac422.pcaec445	-0.055911576	0.097651503	0.567879488
kynurenine.asparagine	-0.00629711	0.011001092	0.567983938
pcaac240.glutamine	-0.006134306	0.010717935	0.568029449
pcaec403.pcaac322	0.030195626	0.052770787	0.568121911
c101.smc240	-0.017468902	0.030529573	0.568126902
c18.prolinr	0.016870245	0.029486579	0.568169502
histidine.pcaac422	-0.009113532	0.015931084	0.568218981
arginine.asparagine	-0.004279788	0.007481651	0.568233685
pcaec445.alphaaminoadipicacid	0.002226043	0.003891778	0.568268702
pcaac360.smc181	0.004412297	0.00771422	0.568280063
lysine.smc180	0.004570455	0.007991324	0.568308628
lysopcac181.c16	-0.03053858	0.053399332	0.568333204
pcaac404.pcaec363	-0.021861737	0.03823363	0.568398928
pcaec423.tryptophan	-0.008105602	0.014175877	0.568402293
smohc222.pcaac426	-0.019331319	0.033808648	0.568403769
c4.pcaec362	-0.006642658	0.011617383	0.568403876
pcaec300.c18	0.015091547	0.026400738	0.568506935
isoleucine.pcaac361	-0.023267462	0.040709272	0.568562094
c101.pcaec385	-0.013032347	0.022803399	0.568591105
pcaac343.glutamicacid	0.034749159	0.060809143	0.568633472
c16.pcaac366	-0.007389144	0.012931968	0.568674281
c142.transhydroxyproline	0.010000901	0.017504115	0.568701441
pcaac341.c102	0.019781018	0.034623239	0.568717482
lysine.smc241	0.003754664	0.006572888	0.568776199
pcaac361.pcaac422	-0.008414713	0.014731504	0.568796354
smohc222.c101	-0.003806715	0.006665329	0.568852804
c141oh.alanine	0.036337438	0.063625861	0.568859981
c4.smohc241	0.015326434	0.026844313	0.568977131
pcaac422.c51	-0.062552329	0.109586271	0.569067303
pcaec406.c102	0.015872085	0.027806727	0.569070175
pcaac386.pcaec362	-0.007758247	0.013592821	0.569096903
serine.methioninesulfoxide	0.00733907	0.012859098	0.569117754
citruline.pcaac366	-0.036610574	0.064147184	0.569119191
pcaac401.pcaec301	0.041307049	0.072385449	0.569168975
smc202.pcaac385	0.012017283	0.02105995	0.569190246
pcaec405.c12	0.029140265	0.051074796	0.569245408

c181.pcaac366	0.006547092	0.011476854	0.569299814
pcaac426.pcaec386	0.005950986	0.010432559	0.569324214
pcaac425.transhydroxyproline	0.013884049	0.024349497	0.569476949
smohc221.pcaec405	-0.022508123	0.039475369	0.569488542
pcaac322.h1	0.001333953	0.002339641	0.569507791
phenylalanine.threonine	0.008868776	0.015555543	0.569519236
pcaec361.pcaec342	0.060827335	0.106701661	0.569564419
pcaec384.pcaac322	0.028475592	0.049954789	0.569592809
smohc221.lysine	0.020153252	0.035372015	0.569779458
smc240.smohc241	-0.024509247	0.043020836	0.56980982
pcaec381.arginine	-0.04128231	0.072488732	0.569949678
lysopcac181.c182	0.026541017	0.046605922	0.569964791
lysopcac160.pcaec425	0.002241321	0.003936673	0.570055068
pcaac385.smohc241	0.02561791	0.045004849	0.570135158
pcaac281.lysopcac161	0.03447559	0.060577452	0.570209366
pcaec362.pcaac365	0.022883822	0.040210608	0.5702207
pcaac405.pcaec386	0.004875335	0.008566783	0.570221836
lysopcac170.pcaec446	0.019982991	0.035113887	0.570226326
c4.pcaac362	0.006630179	0.011650794	0.57023681
c5.histidine	0.046327265	0.08141858	0.570287011
lysopcac280.pcaac366	-0.003534607	0.006213122	0.570359661
lysopcac204.c162	-0.035744061	0.062837129	0.570398339
alphaaminoadipicacid.pcaac422	0.009280671	0.016318612	0.570479355
smc241.pcaac422	-0.01359446	0.023910731	0.570591967
pcaac403.pcaac322	0.042462647	0.074688731	0.57060714
lysopcac180.c4	-0.011678091	0.020541961	0.570626643
pcaac380.lysopcac280	0.003223152	0.005670168	0.570666632
pcaec360.pcaac424	-0.005041854	0.008869779	0.570673165
pcaec404.c4	-0.012255027	0.021560745	0.570697041
pcaec365.smc240	0.012627169	0.02221565	0.570700169
pcaec421.smc241	0.007058229	0.012418332	0.570713015
pcaec360.lysopcac160	-0.01345377	0.023671026	0.570717942
pcaec300.pcaec425	-0.003730699	0.006564021	0.570723816
pcaec422.prolinr	-0.013613504	0.023960826	0.570858609
c12.smohc141	0.019774441	0.034804743	0.570860683
pcaec383.smc161	0.042208021	0.074290691	0.570865284
prolinr.pcaec380	0.020300328	0.035730793	0.570865453
smc180.pcaec405	-0.013624286	0.02398038	0.570867862
pcaac320.lysopcac182	-0.009445087	0.016625988	0.57090254
pcaec420.ornithine	-0.012374235	0.021784956	0.570952893
pcaec382.tyrosine	-0.028615377	0.050380678	0.570976103
pcaac321.pcaec402	0.016986241	0.029908327	0.571002945
pcaec443.smohc241	-0.028545607	0.050265519	0.571034859
h1.pcaac343	-0.020213488	0.035595014	0.571049937
c3.pcaac362	0.00931772	0.016408113	0.571050875

pcaac383.taurine	0.014908439	0.026254637	0.571072904
pcaac403.lysopcac280	0.005734534	0.010099218	0.571086847
lysopcac182.alphaaminoadipicacid	-0.004238818	0.007466195	0.571144349
c8.pcaec422	0.016961843	0.029878775	0.571175524
smc261.pcaac360	-0.018907461	0.033308318	0.571201828
citruline.acetylornithoine	0.018807902	0.033134206	0.571216644
pcaac323.putrescine	0.02462226	0.043380348	0.571242227
pcaec445.c162	-0.029913558	0.052706108	0.571266797
pcaec421.lysopcac180	0.041777804	0.073613615	0.571284196
smohc161.lysopcac281	-0.008085032	0.014246231	0.57128925
smc180.pcaec341	0.046394986	0.081751018	0.571292684
asymmetricdimethylarginine.pcaec4(	0.025541195	0.045008299	0.57131859
lysopcac280.serine	-0.012750944	0.022475282	0.5714172
pcaec382.pcaac321	0.009287318	0.016371164	0.571440581
pcaec446.c16	-0.027628556	0.048711053	0.571511431
pcaac360.prolinr	0.021949136	0.038702591	0.571558564
c10.pcaec363	-0.007938889	0.014001376	0.571636729
c16.pcaec385	-0.024450186	0.043123721	0.571657146
pcaac424.pcaec446	0.039084736	0.06895121	0.571746172
lysopcac181.pcaec361	0.002094865	0.003695802	0.571761964
alphaaminoadipicacid.c10	-0.01307916	0.023080678	0.571864657
pcaec406.pcaac424	-0.006422239	0.011333903	0.57188614
pcaac386.putrescine	0.014052374	0.024799745	0.571890153
putrescine.pcaec402	-0.033916276	0.05986046	0.571920524
pcaac363.alphaaminoadipicacid	0.001261628	0.002226734	0.571925013
pcaac320.putrescine	0.014048513	0.0248018	0.57202744
smc160.pcaec420	0.00807641	0.014259486	0.572056316
c3.c51dc	0.065934799	0.116484572	0.57229334
pcaec382.smc241	0.01526851	0.026985796	0.572456639
pcaec403.pcaac363	-0.012555642	0.022192535	0.572482616
pcaac365.pcaac403	-0.00792554	0.014009307	0.572500097
glutamicacid.lysopcac160	0.018842174	0.033309803	0.572547153
pcaec425.pcaec406	0.003227458	0.005705853	0.572563807
pcaec365.methioninesulfoxide	0.005740823	0.010151599	0.57265246
pcaac401.pcaac321	0.005684657	0.010052325	0.572654136
pcaec300.asparagine	0.00245466	0.00434182	0.572758594
glutamine.pcaec382	-0.011301288	0.019991134	0.572784165
c12.spermidine	0.02932561	0.051889882	0.572895482
lysine.isoleucine	0.014797152	0.026183906	0.572913733
methioninesulfoxide.pcaac362	-0.00787359	0.013933792	0.572949252
pcaac323.pcaac322	0.023010203	0.040725759	0.572995308
lysopcac203.smc160	0.057828398	0.102353808	0.573007748
pcaec420.pcaac383	0.003272779	0.005793056	0.573032691
pcaac425.c2	0.039566348	0.070050832	0.573117586
c142.pcaec384	-0.008959747	0.015865587	0.573182061

pcaec421.pcaec405	-0.062506739	0.110699996	0.573235293
lysopcac140.pcaec343	0.054728924	0.096929209	0.573250283
c51dc.glutamine	-0.00934614	0.01655706	0.573350085
smc161.valine	0.036001252	0.063800042	0.573484495
pcaac365.pcaec421	0.001039475	0.00184217	0.573495192
pcaec405.pcaac365	0.025532964	0.045254425	0.573534268
smohc222.lysopcac170	0.005620268	0.009962518	0.573580391
c2.pcaac366	0.005957317	0.010561535	0.573637
pcaec385.pcaac381	-0.015907623	0.028203159	0.573651196
smc241.pcaec404	0.007562603	0.013409004	0.573680049
pcaac405.pcaac366	-0.009221268	0.01635192	0.573726666
pcaac404.alphaaminoadipicacid	0.001526074	0.002706653	0.573796274
pcaac384.pcaac341	-0.006392121	0.011337247	0.57380112
arginine.pcaac386	0.003770487	0.006689587	0.573923834
pcaec363.methioninesulfoxide	0.005562603	0.00987205	0.574036084
pcaec321.pcaec384	-0.007990078	0.014180813	0.574054407
pcaec343.smc161	-0.033425603	0.059326024	0.574068402
spermidine.lysopcac204	-0.022446338	0.03985176	0.57418818
c181.pcaac404	0.002371299	0.004210385	0.574217579
pcaec384.tryptophan	0.001891792	0.003358995	0.574218038
lysopcac204.pcaec445	0.025367665	0.045069666	0.5744539
asparticacid.serotonin	0.050180443	0.08915589	0.574464199
pcaac403.pcaec342	0.042592473	0.075675193	0.574468713
pcaac386.alanine	-0.006932567	0.01231932	0.574532038
asparagine.threonine	0.009490557	0.016865045	0.574534814
pcaac401.glutamine	-0.007107709	0.012631572	0.574562949
smc180.c8	0.01961978	0.03487109	0.574601331
c51.pcaac364	0.031478683	0.055968763	0.574740137
prolinr.pcaac363	-0.013973808	0.024847334	0.574771685
smc260.pcaac362	-0.002660113	0.004730609	0.574817354
c182.pcaec425	-0.003208485	0.005706042	0.574833157
pcaec443.smc261	0.026047341	0.046323917	0.574839289
c101.smc202	0.019776962	0.035176743	0.574886934
smc240.pcaac384	0.030604024	0.054436459	0.574900367
glutamicacid.histidine	-0.030242411	0.053794404	0.574908595
pcaac361.lysopcac204	-0.006919245	0.012308728	0.574938274
pcaec406.lysopcac280	0.00286949	0.005105654	0.57501935
pcaec381.pcaac363	-0.009077745	0.016154183	0.575072439
lysopcac140.pcaec405	-0.015035838	0.026759581	0.57511168
glutamine.lysopcac160	0.012528623	0.022299953	0.575154704
pcaac424.lysopcac280	0.005832357	0.010385092	0.575300271
smc260.c18	0.01567358	0.027910788	0.575333383
c2.threonine	0.013219051	0.023540827	0.57534878
pcaec321.smc260	-0.011979176	0.021333272	0.575356771
lysopcac204.pcaac363	0.006794716	0.012102393	0.575417762

pcaec382.lysopcac160	0.038051693	0.067778905	0.575435882
phenylalanine.smohc241	0.03212859	0.057234113	0.575473424
c10.smc240	0.01502043	0.026760175	0.575511465
pcaec362.pcaac323	0.058631973	0.104462717	0.575529222
pcaec404.smc260	-0.01973226	0.03515861	0.575553883
pcaac361.alphaaminoadipicacid	0.002690605	0.004794237	0.57556672
pcaec404.phenylalanine	-0.02803682	0.049965596	0.575630677
pcaec384.c102	0.010793996	0.019239196	0.57568544
pcaac360.c162	0.025251395	0.045008068	0.575685723
pcaec363.pcaac323	-0.041045122	0.073162024	0.575702557
pcaac341.c4	-0.01232971	0.021980968	0.575763857
pcaac342.pcaec425	-0.002970978	0.005297268	0.575815239
pcaec360.valine	-0.036521249	0.065130412	0.575890491
pcaec300.pcaac384	-0.028525013	0.050871481	0.575899562
pcaec301.pcaec365	0.018027345	0.03215925	0.576009807
h1.pcaec384	0.012681504	0.022624331	0.576036889
pcaac342.serine	-0.0071931	0.012834736	0.576094689
isoleucine.smc180	0.018337215	0.032727634	0.576191546
pcaac366.asparagine	0.005885646	0.010508087	0.576321601
pcaac281.pcaac422	-0.005970285	0.010659322	0.576326007
smc202.c101	0.01054699	0.018833589	0.576387438
pcaac240.h1	0.001330508	0.002376536	0.576494607
smohc161.pcaec380	0.017274857	0.030856746	0.576502421
c0.c18	-0.026336682	0.047051277	0.576567914
c51dc.c16	-0.0212733	0.038010829	0.576622379
pcaac406.pcaac402	-0.031553617	0.05637997	0.576625198
pcaac361.pcaec322	-0.029975603	0.053560926	0.576629122
pcaec446.pcaac424	-0.005327111	0.009519947	0.576684053
ornithine.c141oh	0.045531956	0.081374626	0.576710242
pcaac322.smohc141	0.00637588	0.011398229	0.576819418
lysine.serine	0.01365827	0.024419232	0.576853638
h1.c3	0.022577758	0.040366253	0.576854786
serine.pcaec425	0.001192395	0.002132055	0.576890589
serotonin.pcaac281	-0.00092235	0.001650059	0.577087964
acetylornithoine.histidine	0.035220744	0.063022538	0.577169969
pcaec360.lysopcac280	-0.00983627	0.017604645	0.57725708
c181.arginine	0.019187229	0.034343488	0.57728799
alanine.c16	-0.015895851	0.028454272	0.57731562
smohc221.methioninesulfoxide	0.003629044	0.006497027	0.577367056
lysopcac160.c0	0.001264424	0.002263765	0.577381319
arginine.smc241	0.003394362	0.00607713	0.577382711
asparagine.ornithine	-0.015956368	0.02857424	0.577470542
lysopcac160.pcaac365	-0.030869245	0.055292215	0.577555737
h1.serotonin	-0.034937562	0.062581949	0.577571938
pcaac383.asparticacid	0.01057481	0.018942234	0.577573812

c182.pcaec443	0.034520345	0.061838429	0.577595424
pcaec425.pcaac360	-0.019450299	0.03484898	0.577665844
pcaac342.pcaec360	0.001967431	0.003525416	0.577707073
ornithine.pcaec341	0.015383908	0.027569246	0.577748466
pcaac383.lysopcac204	-0.00698494	0.012517925	0.57775845
c12.smc241	-0.010923076	0.019577001	0.577786084
c51.c8	-0.015142089	0.027140609	0.577814583
asymmetricdimethylarginine.glutami	0.02123507	0.038064558	0.577843722
pcaec446.pcaec382	-0.006352769	0.011389776	0.577918118
serine.asparagine	0.005103554	0.009150757	0.577946215
c5.lysopcac180	-0.045438397	0.081491394	0.578037575
smc260.pcaec405	-0.014771752	0.026494919	0.578074167
c141.pcaac364	0.019127442	0.034307388	0.578074331
pcaec384.alphaaminoadipicacid	0.001498024	0.002686974	0.578086363
pcaac421.glutamicacid	0.032200622	0.057757763	0.578087741
pcaac421.asymmetricdimethylarginir	0.011000291	0.019732683	0.578119104
pcaec383.lysopcac204	-0.026153936	0.046923622	0.578182485
pcaac361.asparagine	0.007986777	0.014329908	0.578197689
kynurenine.smc240	-0.009217018	0.01653775	0.578210044
arginine.smc260	0.010061858	0.018057727	0.578296477
c162.c2	-0.021247821	0.038135163	0.578319498
lysopcac180.pcaec425	0.002243948	0.00402859	0.578432333
isoleucine.pcaec403	-0.030033248	0.053925065	0.578474327
pcaac383.c142	-0.023672781	0.042513044	0.578548193
histidine.taurine	-0.012283153	0.022063311	0.578624997
pcaec386.h1	-0.001269178	0.002279764	0.578630674
pcaec403.pcaec424	0.010374446	0.018635344	0.57863516
pcaec404.pcaac385	-0.017408898	0.031272143	0.578647291
c16.arginine	0.019527361	0.035090815	0.578790132
spermidine.c142	-0.068555663	0.123196799	0.57879553
pcaac404.pcaac380	-0.021070592	0.037866941	0.578819456
pcaac323.pcaec444	0.038334821	0.068908014	0.578900491
smohc141.pcaec365	0.013595321	0.024446853	0.579037738
pcaec341.pcaac362	-0.004113025	0.007396362	0.5790581
smohc161.valine	0.041145723	0.073993005	0.579066074
lysopcac161.smohc241	0.035852177	0.064478811	0.579097072
pcaac281.methionine	-0.015815352	0.028444788	0.579116585
alphaaminoadipicacid.smc240	-0.007906585	0.014222663	0.579176064
lysopcac160.c101	-0.008048907	0.014478866	0.579181006
pcaec406.pcaac361	-0.040885415	0.073558641	0.579240018
c141.pcaac362	0.005945543	0.010697323	0.579255998
pcaac386.alphaaminoadipicacid	0.00125398	0.002256228	0.579263722
pcaec423.pcaac406	0.020467618	0.036826673	0.579266108
smohc222.glutamicacid	0.016267005	0.029269062	0.579271355
pcaac341.pcaec342	-0.054344448	0.097789234	0.579301599

pcaec423.pcaac281	-0.001605284	0.002888618	0.579303817
smohc241.pcaac402	0.039254666	0.070642059	0.57933318
pcaec380.pcaec342	0.043510223	0.078303557	0.579348987
ornithine.pcaec425	0.002128629	0.003830879	0.579356279
kynurenine.serine	-0.017788113	0.032013436	0.579359664
pcaec403.pcaec423	0.014884107	0.026791791	0.579426431
pcaec422.pcaac366	-0.008258993	0.014866778	0.579435828
smc202.lysopcac170	0.005090209	0.009164318	0.579501064
pcaac426.pcaac321	-0.026680973	0.048038976	0.5795251
pcaec380.lysopcac160	0.010209426	0.018386232	0.579611722
pcaec405.lysopcac140	0.001612118	0.002903847	0.579686273
pcaac281.pcaac403	0.004221949	0.00760576	0.579732198
pcaac365.leucine	0.004455896	0.008027833	0.579761563
isoleucine.smc202	0.025847774	0.046575465	0.579823252
c12.pcaac422	0.009499519	0.017120377	0.579890957
pcaac323.pcaac342	0.004200441	0.007570937	0.57992843
pcaac364.glutamicacid	-0.020928068	0.037729715	0.580015283
pcaac422.smohc241	-0.039131028	0.070551618	0.580042575
pcaac341.transhydroxyproline	-0.010486581	0.018907247	0.580050118
c2.c0	0.001944873	0.003506659	0.580056977
pcaec424.pcaec445	0.039067467	0.070440485	0.580061187
pcaac361.pcaac405	0.037456656	0.067544711	0.580109338
c101.pcaac421	0.020473576	0.036923711	0.580152272
pcaac424.pcaac363	-0.006448392	0.011629891	0.580163194
lysine.pcaac383	0.004165423	0.007513034	0.580191097
serine.pcaac240	-0.005422795	0.009781684	0.580220931
pcaec381.pcaac362	-0.005413624	0.009765612	0.580239194
pcaac405.smc261	-0.015045085	0.0271435	0.580291249
c3.pcaac424	0.003044687	0.005493505	0.580322316
c3.c142	-0.027185508	0.049060231	0.580396568
pcaec383.h1	0.001019681	0.001840287	0.58042186
pcaec300.pcaec362	0.010121991	0.018268006	0.580425364
pcaec300.ornithine	-0.015087806	0.027230611	0.580430658
histidine.pcaec406	0.002442185	0.004408338	0.580487279
citruline.asymmetricdimethylarginine	-0.00907929	0.016389659	0.58050616
pcaec443.c3	-0.023304676	0.042069838	0.580514647
pcaac384.arginine	0.013409842	0.024212336	0.580588867
pcaac364.valine	-0.024422461	0.044101625	0.58063425
pcaac380.ornithine	-0.008657799	0.015638083	0.580730705
asparticacid.pcaac383	0.004653371	0.008407424	0.580834464
pcaac323.lysopcac203	0.013598377	0.024569326	0.58084398
alanine.lysopcac182	-0.006201409	0.011205752	0.580882662
pcaec321.pcaec403	-0.011654951	0.021061109	0.580900197
c3.pcaec365	-0.009217074	0.016656667	0.580921263
lysopcac161.pcaec383	-0.06043821	0.109225873	0.580937715

pcaec401.pcaac344	-0.020317425	0.036721331	0.580968881
pcaec382.pcaac421	0.011981657	0.0216588	0.58102782
c8.c5	-0.004677403	0.008455887	0.581059864
pcaac240.smc241	0.010886008	0.0196825	0.581109637
pcaac361.glutamicacid	-0.015723348	0.028434939	0.581192827
c18.putrescine	0.01294263	0.023407646	0.581217096
lysopcac161.pcaec403	0.014683457	0.026559157	0.581261251
c12.alphaaminoadipicacid	-0.001904369	0.003444962	0.581302464
pcaec443.pcaec340	-0.024415263	0.044169622	0.581327567
smohc241.pcaec385	-0.022163317	0.040097107	0.581341491
smc202.pcaec446	-0.01056518	0.019115283	0.58136368
pcaec444.smc241	0.012764745	0.023097368	0.581404148
c162.pcaec341	0.027978172	0.05062968	0.581434979
c142.pcaac320	-0.027954523	0.050591609	0.581470236
pcaac360.pcaec403	-0.009471709	0.017142779	0.581493244
c18.smc202	-0.020667756	0.037407382	0.581502881
smc180.serine	-0.009808611	0.017755904	0.581564837
c51dc.smc160	0.036101576	0.065360112	0.581609447
pcaac405.pcaac383	-0.00238683	0.004321498	0.581632208
lysopcac280.pcaac403	0.006203139	0.011235633	0.581782639
pcaac240.pcaac321	0.005884231	0.010660126	0.581857876
pcaec403.pcaac406	0.028141978	0.050986793	0.581884301
pcaec383.lysopcac182	-0.01988835	0.036043475	0.58199269
serine.taurine	-0.014992852	0.027174385	0.582033988
c10.c3	0.022886598	0.041485335	0.58206687
alphaaminoadipicacid.pcaac240	0.004341794	0.007872323	0.582171309
c101.ornithine	0.016357066	0.02966159	0.582219333
pcaec381.pcaac425	-0.057866443	0.104945179	0.582259852
pcaac384.pcaac403	-0.001659104	0.003011518	0.58258619
pcaec385.pcaac364	0.027582056	0.050067532	0.582601508
pcaac420.pcaac425	-0.047598912	0.086417784	0.582667884
pcaec383.smc241	0.008879139	0.01612369	0.582743745
pcaec405.taurine	-0.034013075	0.061792121	0.582911582
pcaac344.pcaac422	-0.018894171	0.034327849	0.582938919
pcaac343.pcaec343	0.053894914	0.097924635	0.582960953
pcaec342.lysopcac182	0.010529934	0.019133637	0.582984964
smc240.phenylalanine	0.021261812	0.038650975	0.583148115
ornithine.c141	-0.010759993	0.019563079	0.583204447
glutamicacid.valine	0.043177153	0.078507937	0.583234207
lysopcac170.pcaac385	-0.015299348	0.027818716	0.583238187
c101.alphaaminoadipicacid	0.004696519	0.008541209	0.583306705
methionine.pcaec342	-0.041989981	0.076378463	0.583377696
c3.c182	-0.021303573	0.038751679	0.583388811
pcaac360.alanine	-0.006743753	0.012267785	0.583411687
smc180.pcaec406	0.002841232	0.005169126	0.583451456

pcaac323.pcaec403	-0.006858728	0.012483112	0.58359767
pcaac380.pcaac366	-0.03018035	0.054945297	0.583707544
smc181.glutamicacid	0.018084842	0.032932418	0.583796362
pcaec443.pcaac321	0.006164234	0.011226162	0.583833834
pcaac406.pcaec341	-0.021526834	0.039210356	0.583893029
lysopcac160.pcaac362	-0.003445846	0.006276975	0.583922344
pcaec361.c14	-0.038284511	0.069753988	0.58400144
leucine.c141	-0.012939196	0.023575144	0.584002412
asparticacid.lysopcac140	0.000539279	0.000982596	0.584015318
pcaac406.alphaaminoadipicacid	0.00146711	0.002673316	0.584037432
smohc141.pcaec403	-0.012548043	0.022867772	0.584089587
lysopcac161.methionine	-0.019345432	0.035260847	0.58414706
creatinine.pcaec384	-0.031366745	0.057174863	0.584165533
c4.glutamicacid	0.021651678	0.039470422	0.584204043
pcaac342.pcaac362	-0.003749534	0.006835683	0.584225192
pcaac300.pcaac361	-0.031016861	0.056553308	0.584273176
serine.c182	0.017117307	0.031212588	0.584302719
alphaaminoadipicacid.lysopcac180	-0.027887944	0.050862155	0.584375254
c16.asymmetricdimethylarginine	0.014884474	0.027153213	0.584469888
pcaec382.pcaec422	0.02138763	0.039017583	0.584478489
lysopcac160.taurine	-0.022895394	0.041772006	0.584512622
smc180.asparagine	0.004210171	0.007681721	0.584531288
c0.serine	-0.014711591	0.026844217	0.58455919
pcaac422.smohc222	0.001866383	0.003405789	0.584581694
pcaec424.c8	0.017652722	0.032213652	0.584591325
smohc222.pcaec365	0.019009673	0.034692887	0.584623741
methioninesulfoxide.serotonin	-0.065521853	0.119579934	0.584629213
pcaac422.pcaac364	0.03019571	0.055109965	0.584640227
alanine.c14	0.017856223	0.03259599	0.584717705
pcaec423.c3	-0.014404717	0.026297521	0.584748368
lysopcac161.pcaec340	-0.015741143	0.028737429	0.584749721
lysopcac281.pcaec406	0.001608101	0.002936049	0.584782719
c51.pcaec301	0.023770501	0.043400836	0.584791282
lysopcac204.serotonin	-0.040982133	0.074828368	0.584801611
pcaec423.pcaac404	0.002984133	0.005448927	0.584819851
isoleucine.alphaaminoadipicacid	0.008982091	0.016403124	0.584868451
histidine.lysopcac203	0.012639048	0.023083018	0.584893583
c142.lysopcac140	-0.00096135	0.001755813	0.584909507
pcaac363.c182	0.012610775	0.023033732	0.584931857
pcaac424.pcaac385	-0.009741641	0.017793832	0.584944659
lysopcac281.serine	-0.005610229	0.010248682	0.584987909
acetylornithoine.pcaec364	-0.015056823	0.027516325	0.585134345
smc260.acetylornithoine	0.012643381	0.023106473	0.585145863
pcaac401.c51dc	-0.063282222	0.115666658	0.58519423
phenylalanine.lysopcac170	-0.007417553	0.013558216	0.585207551

c14.pcaec361	-0.00435055	0.007952928	0.585242904
pcaec383.c102	-0.015117574	0.027639235	0.58529558
pcaec382.smc202	0.044936695	0.082159572	0.585306913
isoleucine.smc261	-0.028494602	0.052100491	0.585326039
c101.pcaec341	0.02595489	0.047463769	0.585381102
pcaac240.pcaac422	-0.013632082	0.024929751	0.585392088
kynurenine.glutamicacid	0.028849366	0.052758469	0.585392211
lysopcac140.valine	-0.028164609	0.05150628	0.585392694
c3.threonine	0.01411978	0.025824577	0.585434809
c3.pcaac342	-0.004694236	0.008585687	0.585438971
pcaac381.lysopcac181	-0.014858234	0.027175832	0.585443752
c14.alphaaminoadipicacid	-0.001263153	0.002310538	0.585479627
pcaec365.methionine	-0.011376413	0.020810603	0.585498687
lysopcac181.c5	0.005460606	0.009989664	0.585525175
c141oh.smc161	-0.0184614	0.033774701	0.585539693
acetylornithoine.pcaac383	-0.001210667	0.002214941	0.585548684
pcaac384.pcaec340	0.028307906	0.051808861	0.585685793
glutamicacid.pcaac344	0.038100162	0.069735127	0.585710348
serotonin.asparagine	-0.010044882	0.018386636	0.585738513
c51.pcaec342	-0.032918359	0.060269961	0.585829291
pcaac420.pcaac344	-0.018220548	0.033359979	0.585830659
asparagine.pcaec406	0.000613917	0.001124062	0.58584445
pcaac364.pcaac404	0.005087856	0.009316658	0.585883271
pcaec422.smc241	0.010811667	0.019801803	0.58595802
methioninesulfoxide.pcaec383	-0.049505526	0.090673085	0.585968901
pcaec403.smc261	-0.036691474	0.067204622	0.585976895
c182.pcaac366	0.005457712	0.009998639	0.586059846
ornithine.pcaac425	-0.034398412	0.063034802	0.586156131
lysopcac140.c142	0.02480424	0.045454144	0.58616091
lysopcac140.pcaec425	0.001864676	0.003417104	0.586167143
pcaac386.lysopcac281	0.006241842	0.011440955	0.586248754
putrescine.pcaec445	-0.036147284	0.066272342	0.58634101
pcaec360.serotonin	0.054093949	0.099179568	0.586355547
arginine.c3	-0.022583752	0.041410341	0.586389286
pcaac300.pcaac362	-0.004650342	0.008533255	0.586662318
pcaec444.serine	-0.003862345	0.007087321	0.586663371
pcaac403.pcaac320	-0.037766536	0.069303423	0.586677531
asparticacid.methionine	-0.008502386	0.015603642	0.586709921
smc181.c8	0.018720359	0.034356831	0.586721758
pcaec380.glutamicacid	0.032748034	0.060110147	0.586776519
glycine.pcaac404	-0.002658612	0.004881037	0.586857888
glutamicacid.c141oh	-0.052225827	0.095900772	0.586926418
pcaec405.pcaac364	0.023381426	0.042950523	0.587064625
lysine.h1	-0.001653203	0.003037082	0.58709301
c141oh.smohc241	0.023539134	0.043253743	0.587181371

creatinine.smc260	0.005594441	0.01028074	0.587211026
lysopcac203.pcaec405	-0.021472949	0.039464508	0.587251675
pcaac421.asparticacid	0.021805744	0.040077833	0.587267433
pcaec446.c102	-0.011848253	0.021779393	0.587317401
smc181.pcaec401	-0.010517026	0.01933325	0.587334991
pcaec385.methioninesulfoxide	0.006144065	0.011294664	0.587339814
c12.pcaec406	-0.002509754	0.004614527	0.587407284
pcaac363.acetylornithoine	0.005557516	0.010219259	0.587443992
pcaac406.pcaec384	0.011685621	0.021496527	0.587596771
c12.pcaec365	-0.012309811	0.022658307	0.587819721
alanine.pcaec300	0.007268434	0.013380821	0.587876241
pcaec401.leucine	0.02618497	0.048207668	0.587895266
pcaec382.pcaec341	-0.025902642	0.04768914	0.587905046
asparagine.pcaac323	0.0411307	0.075727807	0.587916946
pcaec420.c182	0.010897459	0.020066037	0.587957374
pcaec383.glycine	0.013407851	0.024689067	0.587965199
smc202.pcaac363	0.0029058	0.005350708	0.587965271
serotonin.lysopcac182	-0.025092242	0.046206095	0.587977492
c5.phenylalanine	0.026361899	0.048546145	0.587993161
pcaec363.pcaec384	-0.01549777	0.02854234	0.588029468
pcaec341.c182	0.017401299	0.032048792	0.58803776
c4.c162	-0.050831898	0.093625988	0.588063584
pcaac362.pcaac421	-0.014863999	0.027378114	0.588070256
citruline.pcaac361	-0.019214384	0.03539235	0.588083192
smc202.pcaec444	0.038368043	0.070674993	0.588094513
lysopcac204.c12	0.031780964	0.058564031	0.588238505
ornithine.pcaec421	0.000602337	0.001110009	0.588258016
lysopcac161.pcaac344	0.024936129	0.045969138	0.588387318
c101.pcaac362	0.001573202	0.002900174	0.588389334
phenylalanine.pcaac422	-0.007804302	0.01438877	0.588432158
acetylornithoine.pcaac381	0.010208543	0.01882269	0.588456451
c10.pcaac383	0.002583828	0.00476567	0.588578704
c4.creatinine	0.008614636	0.015889674	0.58859382
pcaac360.glutamine	-0.007956166	0.014676226	0.588621681
acetylornithoine.tryptophan	0.001008236	0.001860196	0.588695437
pcaac320.pcaac360	-0.029378878	0.054211034	0.588743358
pcaec401.pcaac406	0.015712453	0.028993448	0.588746246
histidine.pcaac364	0.031755514	0.058602862	0.588783815
pcaec443.pcaac425	0.059056173	0.108988927	0.588798725
pcaac341.pcaac384	0.025316444	0.046723067	0.588808635
c2.smohc161	0.004704959	0.008684689	0.58886846
pcaac401.histidine	-0.041192755	0.076049722	0.588935623
pcaac381.pcaec363	0.02299884	0.042467962	0.589003064
smohc222.pcaec405	-0.011147509	0.020584334	0.589006044
c18.c51dc	0.040960853	0.075642224	0.589037175

alanine.taurine	-0.02061173	0.038066403	0.589064668
pcaac405.smohc241	0.019002089	0.035098141	0.589112118
methioninesulfoxide.pcaac425	-0.055078698	0.101741703	0.589140014
pcaec301.pcaac385	0.005562917	0.010276049	0.58914708
pcaac281.pcaac361	-0.022859219	0.042230654	0.589183783
pcaac425.asparagine	-0.005971403	0.01103203	0.589194637
pcaac401.pcaac323	-0.048953742	0.090470824	0.589317581
c12.pcaac364	0.016158298	0.0298685	0.589399051
alphaaminoadipicacid.spermidine	-0.028669853	0.052998533	0.589416684
glutamine.pcaac403	-0.00312346	0.005775314	0.589503373
pcaec445.pcaec406	0.001093913	0.002022729	0.589516433
spermidine.c51	-0.052687742	0.097423954	0.58951752
smc161.asymmetricdimethylarginine	0.031371343	0.058009684	0.589527128
methionine.pcaec406	0.003311211	0.0061236	0.589572092
c5.methioninesulfoxide	0.007454901	0.013787976	0.589605238
pcaac422.pcaec420	0.010858162	0.020082893	0.589614931
lysopcac280.pcaac424	0.019817911	0.036655177	0.589621333
pcaac362.pcaac383	-0.002361958	0.004369035	0.589651957
smc261.c5	-0.002946036	0.005450328	0.58971297
pcaac342.c3	-0.039724429	0.073494338	0.589722983
histidine.ornithine	-0.034050348	0.063004796	0.589770753
pcaec342.acetylornithoine	0.009229479	0.017082456	0.589874349
arginine.pcaac383	-0.002751005	0.005093037	0.589970442
serotonin.glutamicacid	0.017077993	0.031617983	0.589980582
pcaec445.serine	-0.003841799	0.007112918	0.589994177
pcaac383.pcaac424	0.003509129	0.006497816	0.590041079
prolinr.taurine	-0.013848858	0.025645782	0.590070082
c3.lysopcac180	-0.021508791	0.039831269	0.590075356
pcaac426.smc161	-0.030504811	0.05649161	0.590081739
pcaec421.pcaac321	0.006807464	0.012606794	0.590084878
leucine.pcaec384	0.014128747	0.026165888	0.590095604
pcaec421.pcaac426	0.048981771	0.090733485	0.590182204
pcaec446.smohc141	-0.014082718	0.026088819	0.590212003
pcaec381.c16	0.015329926	0.028402181	0.590249352
pcaec340.acetylornithoine	0.011136687	0.020633509	0.590253994
pcaec385.asparticacid	0.026381566	0.04887869	0.590255631
smc161.pcaec446	-0.011807111	0.021876045	0.590260903
pcaec444.pcaec384	-0.017924619	0.033210692	0.590263331
lysopcac170.pcaec301	0.017631705	0.03266979	0.590283883
pcaec386.smohc141	-0.054782228	0.101508313	0.590292327
pcaec382.smc160	-0.045705577	0.084691693	0.590300615
pcaec445.tryptophan	0.001111266	0.002059284	0.590323141
c181.pcaac385	0.017351588	0.032154541	0.590327521
pcaec301.pcaac322	0.029067429	0.053866398	0.590334548
pcaac363.c2	-0.041103696	0.076218323	0.590562876

pcaec363.acetylornithoine	0.011305687	0.020964719	0.590574483
pcaec425.c8	0.019612798	0.036373396	0.590619068
pcaac404.pcaec300	0.015525052	0.028794214	0.59064288
smc160.c51	-0.023419733	0.043438385	0.590659605
smc161.pcaac422	-0.014438463	0.026781091	0.590672903
pcaec420.putrescine	0.014364048	0.026646635	0.590722631
pcaec424.c51dc	-0.054659478	0.10140549	0.590748645
c102.pcaac341	-0.002436911	0.004521255	0.590768546
smohc161.asparticacid	0.011878761	0.022038945	0.590768866
lysopcac280.pcaec443	0.037694682	0.069943985	0.590812105
pcaac424.c2	-0.049311281	0.091509602	0.59085488
pcaac344.lysopcac182	-0.00671987	0.012471541	0.590888067
smohc141.lysopcac182	-0.011970026	0.022216737	0.590910184
lysopcac181.lysopcac281	-0.012397413	0.023011039	0.590927257
pcaac384.pcaec365	-0.014347645	0.026633432	0.590962546
pcaac344.c101	-0.001746981	0.003243231	0.590999336
pcaec405.pcaac424	-0.008760678	0.016264546	0.591011835
leucine.lysine	0.057592736	0.106927193	0.591025686
pcaac426.pcaec406	0.004996399	0.009276773	0.591042176
pcaac342.tyrosine	0.025679216	0.047681078	0.591063111
pcaac362.c3	-0.035021964	0.06503475	0.591097826
pcaac403.pcaac406	0.030506592	0.056660338	0.59116652
pcaec300.h1	0.000975416	0.001811839	0.59120451
c12.prolinr	-0.019279393	0.035813147	0.591220849
asparticacid.c51	-0.043172157	0.08020448	0.591259876
methionine.pcaac405	0.026653757	0.049517798	0.591266719
lysopcac182.pcaac405	0.011876879	0.022065813	0.591279283
alphaaminoadipicacid.pcaec423	0.006140029	0.011408104	0.591300994
pcaec422.smc160	-0.023288239	0.043270319	0.591309857
smc181.pcaac300	-0.018843709	0.035014516	0.591333979
pcaac402.c0	0.000722057	0.001341862	0.591380483
pcaac362.lysopcac203	-0.009787902	0.018190764	0.591401864
smohc241.lysopcac180	0.022725595	0.042235859	0.591405921
smohc221.alanine	-0.015893276	0.029544882	0.591493579
pcaec365.smc260	-0.014148988	0.026309005	0.59158754
pcaec385.c8	-0.027771213	0.051639836	0.591596905
glutamicacid.asparagine	0.006794212	0.012635914	0.591663019
smc260.pcaec361	-0.002621927	0.004877138	0.591728638
pcaec405.asymmetricdimethylarginir	0.012206144	0.02270745	0.591767307
pcaac344.serotonin	0.04046436	0.075283406	0.591798585
glutamicacid.methioninesulfoxide	0.015114632	0.028122445	0.591823221
pcaec343.asymmetricdimethylarginir	0.01800554	0.033504957	0.591863641
pcaac422.pcaec423	-0.044185078	0.082223546	0.591878694
pcaac281.c4	-0.005622144	0.01046313	0.591912037
glutamicacid.c12	-0.057263636	0.106573501	0.591921071

pcaac362.pcaac405	0.026062028	0.048511581	0.591978086
pcaac281.smohc222	-0.003139207	0.005843371	0.591983475
smc260.lysopcac140	-0.001121525	0.002087787	0.592012351
pcaec341.alanine	-0.006555209	0.012203555	0.592031417
c181.lysopcac203	-0.007459947	0.013888279	0.592042374
asparticacid.spermidine	-0.0283526	0.052785584	0.592050685
c2.histidine	0.027824011	0.051806571	0.592087043
lysopcac182.pcaac360	-0.013734369	0.025574795	0.592119692
lysopcac180.histidine	0.017837565	0.033216668	0.592134223
h1.pcaec402	0.013460599	0.025067065	0.592150051
lysine.ornithine	-0.025407277	0.047322046	0.592206523
pcaec401.c162	-0.033336315	0.062104315	0.592290668
smc261.pcaac363	0.003397913	0.006330422	0.592304501
smc181.transhydroxyproline	-0.011719727	0.021836531	0.592343415
pcaec424.c141	-0.011069612	0.020626152	0.59236018
smc181.pcaac321	0.009872285	0.018397171	0.592400656
pcaac420.smc240	-0.032884507	0.061284962	0.592425536
c141oh.glutamine	-0.005064498	0.00943873	0.592437945
pcaac404.valine	-0.021201547	0.03951366	0.5924401
pcaac422.pcaac321	0.008011616	0.014932969	0.592479547
c12.pcaac385	0.002031642	0.003786842	0.592482968
smc180.c101	-0.017887273	0.033344403	0.592524477
pcaec405.c101	0.009189889	0.017132095	0.59254267
lysopcac280.c0	-0.00034446	0.000642154	0.592542884
lysopcac160.pcaec422	-0.033171953	0.061850516	0.592604013
pcaec342.c4	-0.006466611	0.01205881	0.592651183
phenylalanine.pcaac363	-0.008094824	0.015096143	0.592677443
pcaec362.pcaac386	0.008609167	0.016057363	0.592723845
pcaac401.c18	-0.019008606	0.035458726	0.592774681
pcaec321.pcaec406	0.001690795	0.003154366	0.59281568
methionine.tryptophan	-0.00314912	0.00587735	0.592961327
pcaac363.pcaec343	0.039210186	0.073190936	0.593017549
pcaac341.pcaec444	0.036501186	0.068140985	0.593054137
pcaac364.alphaaminoadipicacid	0.000852717	0.001592108	0.593110241
pcaec401.pcaac363	-0.021937381	0.040962569	0.593139998
pcaec444.pcaec322	-0.023673879	0.044215337	0.593225935
glutamicacid.glutamine	-0.00948588	0.017722422	0.593346567
lysopcac181.pcaec382	-0.003776818	0.007056282	0.593350255
pcaac361.smc180	-0.005993901	0.011198527	0.593351589
pcaac365.pcaac363	0.005915059	0.011054334	0.593455354
acetylornithoine.pcaac404	0.004894256	0.009147844	0.593505131
leucine.pcaec421	0.000419656	0.000784781	0.59369478
pcaec321.ornithine	-0.014971527	0.02799883	0.593710434
pcaec300.pcaec365	0.009419073	0.017615509	0.59372168
pcaec342.lysopcac281	0.015880002	0.029699726	0.593734308

pcaac383.pcaec361	-0.004177615	0.007813821	0.593762415
pcaec402.pcaac383	0.002536821	0.004745545	0.593814521
pcaac425.tryptophan	0.0062831	0.011756306	0.593899859
pcaac341.lysopcac281	0.007533822	0.01409961	0.593980408
pcaec300.lysopcac161	0.025741479	0.048177874	0.593999394
lysine.spermidine	-0.02839631	0.053146873	0.594000802
pcaec383.lysopcac160	-0.019262949	0.036058754	0.59406202
pcaac402.c101	0.020700437	0.038749633	0.594062134
c162.lysopcac281	0.004673546	0.008749265	0.594093569
smc161.pcaac363	0.002796907	0.005236693	0.594139398
pcaec385.tryptophan	0.00127366	0.002384904	0.594171879
pcaac401.smc241	0.008839109	0.016553797	0.594232753
pcaec300.pcaec382	0.005557227	0.010407599	0.59423554
pcaac425.valine	-0.021002931	0.039345522	0.59433985
asymmetricdimethylarginine.pcaec36	0.008797684	0.016482378	0.594370471
pcaec402.pcaec406	0.008168733	0.015306021	0.594418042
lysopcac170.c141	-0.007955285	0.014908703	0.594482954
pcaec425.c142	0.022094646	0.041407765	0.594491936
pcaac240.pcaec380	0.038051385	0.07132193	0.594541011
pcaec420.pcaac342	-0.009181834	0.017210174	0.594543741
pcaec386.threonine	0.01520542	0.028504364	0.594592137
pcaec444.tryptophan	0.001032398	0.001935747	0.59466713
smc261.lysopcac140	-0.000620318	0.001163115	0.594672814
pcaac322.pcaac403	-0.008408377	0.015767698	0.594713501
pcaec343.lysopcac182	0.018856279	0.035368592	0.594803061
c162.pcaec423	0.020285525	0.038060325	0.59490852
h1.smc160	-0.04104028	0.077011995	0.594960891
c51.pcaac322	0.032908787	0.061763928	0.595024403
pcaec300.pcaec361	-0.005630636	0.010570743	0.595130296
pcaec445.pcaac425	-0.033752528	0.063366973	0.595137574
smc240.pcaac422	-0.014093727	0.026463062	0.595186343
pcaec425.c3	-0.011667324	0.02190807	0.59520212
c4.pcaac341	-0.002941185	0.005523071	0.595223614
pcaac402.pcaac364	0.025546369	0.047974871	0.595245946
pcaac360.lysopcac161	0.032457495	0.060957237	0.595267653
c18.spermidine	-0.031564695	0.059293084	0.59534568
pcaec301.lysopcac203	0.008993038	0.016893194	0.595348144
pcaec365.pcaec446	-0.036307933	0.06821259	0.595396952
pcaec383.smc181	0.002121309	0.003985366	0.595397963
methionine.c182	0.024896261	0.046780535	0.595454543
methioninesulfoxide.pcaac420	-0.045732404	0.08593812	0.595480576
pcaac385.c182	0.010331045	0.019416819	0.595541505
kynurenine.threonine	0.007676358	0.014431026	0.595633041
lysopcac160.c2	-0.03847573	0.072335354	0.595651437
c142.serine	0.007186129	0.013511101	0.595678486

pcaac363.pcaec444	-0.038860045	0.073063703	0.595680743
smohc221.pcaec406	0.002787039	0.005240807	0.595728744
ornithine.c182	0.017660299	0.033211271	0.595756073
glycine.asymmetricdimethylarginine	-0.006681161	0.012564637	0.595765
pcaec342.pcaec301	-0.020045132	0.037700362	0.595797654
smc180.c16	0.022346946	0.042030745	0.59580807
pcaec444.pcaac361	0.015196394	0.028582353	0.595815272
pcaec424.pcaac344	0.029812921	0.056080643	0.595858424
lysopcac203.spermidine	-0.03360823	0.063230463	0.595919536
citruline.smc241	0.002387301	0.004492952	0.596041126
pcaac240.c5	-0.008499623	0.01600035	0.596130112
smc181.pcaec365	0.023363502	0.043984799	0.596159572
pcaec362.c12	0.021655732	0.040771925	0.596179595
c8.pcaac383	0.002543258	0.004788583	0.596203439
lysopcac170.pcaec341	0.015640597	0.029452444	0.596246922
lysopcac203.pcaec443	0.035664994	0.067161651	0.596256374
methionine.pcaac281	0.002909172	0.00547928	0.596319659
pcaac240.lysopcac281	0.020522732	0.038666023	0.596438129
pcaec300.pcaac300	0.043472015	0.081914577	0.59648635
pcaec443.c0	-0.001007102	0.0018982	0.596585044
glutamine.pcaec405	-0.030669339	0.057810004	0.596610632
pcaec405.c8	0.019038183	0.035892665	0.596679552
putrescine.c51	-0.046150617	0.087018334	0.596724337
lysopcac160.valine	-0.016749309	0.031583281	0.59674722
pcaec340.alanine	-0.008024125	0.015133263	0.596810153
lysopcac180.c182	0.014641783	0.027614625	0.596818821
lysopcac204.lysopcac281	0.008595547	0.016212972	0.596855917
lysopcac140.pcaac360	-0.021503362	0.040583865	0.597073552
pcaac402.c51	-0.044138482	0.083315481	0.597125247
serotonin.isoleucine	-0.006320072	0.011930389	0.597145743
c141.asparticacid	0.014611269	0.027585583	0.597197727
smc180.pcaac385	-0.006465878	0.01220984	0.597272136
glutamine.pcaec361	-0.002675764	0.005052867	0.597278484
c12.pcaec446	-0.017596983	0.03324193	0.597411736
pcaac300.pcaac360	-0.013628036	0.025751122	0.597508579
alanine.c4	-0.004393852	0.008302991	0.59753066
pcaec343.serine	-0.007021032	0.013267901	0.597540934
pcaac421.pcaec406	-0.009576244	0.018097733	0.597564273
lysopcac180.glutamicacid	-0.018144077	0.03429899	0.597663282
asymmetricdimethylarginine.c12	0.013366935	0.02527594	0.597772013
pcaac401.pcaac366	-0.018572559	0.035120132	0.597779521
pcaec406.pcaac322	0.025463501	0.04815709	0.597828149
phenylalanine.pcaec443	-0.041168945	0.077861664	0.597838113
citruline.smc181	-0.007200785	0.013623742	0.597975069
pcaec446.tryptophan	0.001369726	0.002591501	0.597975822

lysopcac180.pcaac321	-0.018218125	0.034468999	0.597982186
pcaec446.pcaec406	0.001853728	0.003507858	0.59804205
pcaac404.pcaac281	0.001708885	0.003234322	0.598104741
pcaec362.smohc241	0.051764915	0.097976346	0.598117532
smc202.pcaac361	-0.019811579	0.037503338	0.598172337
lysopcac181.c101	-0.003738481	0.007077086	0.59817948
pcaec443.smc240	0.018020853	0.034118505	0.598226066
serine.lysopcac170	-0.016310724	0.030881133	0.598230557
lysopcac280.c8	-0.019369882	0.036678918	0.598289092
lysopcac160.pcaac422	-0.024254318	0.045936093	0.598352595
isoleucine.pcaec406	0.015457213	0.029275641	0.598361211
pcaac321.pcaec420	-0.029072092	0.055065672	0.598385976
smc180.asparticacid	0.012058033	0.022846469	0.598502041
methioninesulfoxide.pcaac321	0.008384921	0.015887589	0.598515838
smohc161.lysopcac180	0.033457081	0.06339603	0.598528472
pcaac403.pcaec401	0.027040923	0.051246538	0.598586524
pcaec404.pcaec406	0.013646811	0.025862808	0.598587753
pcaac385.tryptophan	-0.003386001	0.006417883	0.598638426
pcaec405.lysopcac180	0.030484667	0.057793966	0.598719425
lysine.c51dc	-0.055532354	0.105284422	0.598733708
pcaec343.pcaac380	-0.032896572	0.062374154	0.598763974
pcaac344.lysopcac280	0.002885547	0.005471281	0.598769728
methioninesulfoxide.pcaec402	-0.068485537	0.129859404	0.598781227
pcaec445.smohc241	0.031893664	0.060478115	0.598797443
smohc221.pcaec446	-0.009567568	0.018143053	0.598810025
prolinr.pcaac406	0.013144745	0.024928882	0.598845202
c102.pcaac385	0.001508135	0.002860336	0.598867095
pcaec383.ornithine	0.020412567	0.038717155	0.598891597
leucine.pcaac424	0.005400635	0.010244366	0.598920518
c101.smohc222	-0.001549698	0.002939755	0.598940573
methionine.threonine	0.011020958	0.020907636	0.598958635
pcaec342.smc202	-0.024835643	0.047115324	0.598959651
pcaac424.pcaec406	0.024373919	0.046259456	0.599117973
pcaec420.c3	0.01009106	0.019155632	0.59918899
pcaec365.tryptophan	0.001485598	0.002820386	0.599228979
pcaec386.smc261	0.030167226	0.057276857	0.599259664
smc202.c2	0.023134488	0.043926429	0.599278313
c181.lysopcac181	-0.01962911	0.03727137	0.599285658
pcaac424.smc241	0.010542279	0.020018967	0.599312875
pcaac300.c4	-0.005332478	0.010126975	0.599349448
pcaac281.transhydroxyproline	-0.01447321	0.027486935	0.599358487
pcaac425.pcaec342	-0.041900586	0.079576748	0.599362373
c101.pcaec322	-0.026612056	0.050555865	0.59946905
asymmetricdimethylarginine.pcaac36	-0.031453281	0.059760791	0.599517136
c51.lysopcac160	0.017452856	0.033174603	0.599675669

pcaec420.c102	-0.01201957	0.022853067	0.599773324
pcaec444.c51	-0.026086186	0.049604531	0.59981963
pcaac386.c141oh	0.035526786	0.067564886	0.599865243
pcaac300.pcaec386	0.004384652	0.008339046	0.59987878
methioninesulfoxide.h1	0.002317569	0.004408159	0.599915233
pcaac344.pcaac343	-0.014186757	0.026984612	0.599922245
pcaec342.putrescine	0.013400448	0.025489538	0.599930292
smohc222.isoleucine	0.011514524	0.021905334	0.599981671
pcaec446.c101	-0.011330357	0.021556491	0.600007318
serine.c162	0.034272782	0.065222399	0.600102033
methionine.smc260	0.008828464	0.016802477	0.600136211
pcaac322.acetylornithoine	0.003665854	0.00697759	0.600171541
pcaec364.valine	-0.026769671	0.050960223	0.600220161
pcaac341.pcaac364	0.033022979	0.062875385	0.600283987
c5.c141oh	-0.078244521	0.148984749	0.600303665
pcaac426.h1	-0.001664353	0.003169115	0.600307675
pcaec363.tryptophan	0.000823148	0.001567407	0.600317269
alphaaminoadipicacid.lysine	0.039800887	0.07581602	0.600454832
lysopcac203.c0	-0.001085585	0.002067931	0.600458238
lysine.taurine	-0.015717838	0.029943089	0.600484644
lysopcac281.prolinr	0.014979035	0.028535953	0.600488622
glutamine.smc181	-0.005327227	0.010148982	0.600499331
c3.pcaac385	0.035462842	0.067587328	0.60064211
pcaac384.lysopcac160	0.010584566	0.020173209	0.600650632
c0.smc240	0.031646609	0.060316451	0.600656232
lysine.pcaec420	-0.02261374	0.043109916	0.600736808
pcaec444.pcaac402	-0.040933905	0.078043105	0.600775821
arginine.pcaac321	0.00797552	0.015206006	0.600779816
h1.pcaac366	0.010734365	0.020469168	0.600836509
arginine.pcaac344	0.033824667	0.064510978	0.600900372
c12.serine	0.007788166	0.014856042	0.600957136
pcaec364.tryptophan	0.00119205	0.002273971	0.600976028
asymmetricdimethylarginine.serine	0.005513179	0.010518129	0.601014455
pcaec340.pcaec446	-0.018961054	0.036176538	0.601038029
pcaec380.c162	0.027329032	0.052144368	0.601053627
lysopcac182.pcaec362	-0.008248638	0.015738904	0.601061246
pcaec385.smohc141	-0.023202907	0.044278594	0.601110768
pcaec403.c51	-0.039509548	0.075401944	0.601135083
methioninesulfoxide.smc241	0.008907835	0.017000679	0.601146465
lysopcac180.pcaec361	0.002251609	0.004297374	0.601160009
pcaac366.smc240	0.019306695	0.036851003	0.60118605
phenylalanine.c5	-0.01442052	0.027530441	0.601262081
pcaec365.pcaec422	-0.014335975	0.027370588	0.6012827
c0.asparticacid	0.010392251	0.019841413	0.601287809
pcaac385.pcaec406	0.003754148	0.007167847	0.601299826

c12.pcaec402	-0.023857442	0.045551984	0.601304895
c12.smc240	0.014151775	0.027023042	0.601338272
c3.smc241	0.022749825	0.043448635	0.601400821
pcaec361.taurine	0.033415444	0.063832528	0.601481729
smc181.creatinine	0.004792389	0.00915509	0.601494884
methionine.pcaac422	-0.011444367	0.021869196	0.601603998
c51.histidine	0.028613467	0.054688699	0.60167597
methionine.pcaec425	0.001068229	0.002041847	0.601702374
pcaac420.smc160	-0.020186623	0.038592261	0.60176747
pcaac322.pcaac381	0.02967312	0.056736225	0.601818177
c2.pcaac361	-0.020885235	0.039940107	0.601878877
c142.arginine	0.012184275	0.02330985	0.60202088
pcaec341.smc181	0.011340417	0.02169727	0.602051168
pcaac420.glutamine	-0.006240579	0.011940532	0.602070186
pcaac322.pcaac364	0.027005859	0.051673002	0.602075926
pcaac344.c12	0.02273468	0.043501628	0.602085086
pcaec385.alphaaminoadipicacid	0.000874033	0.001672434	0.602088854
lysopcac180.c8	0.02048168	0.039192753	0.602104743
c0.h1	-0.002460086	0.00470797	0.602140827
lysopcac203.lysopcac140	-0.001389652	0.002659862	0.602199086
pcaac281.pcaac383	0.002803814	0.00536668	0.602201868
lysine.pcaac366	-0.006086177	0.011649356	0.602202609
pcaac426.smc241	0.004510823	0.008637524	0.602349663
lysopcac281.smc161	-0.029689031	0.056855785	0.602387362
lysopcac161.pcaac362	-0.002422927	0.004640074	0.602392343
pcaac361.c2	-0.029288596	0.056092262	0.602408875
c51.pcaec383	0.026406844	0.05057875	0.602448275
c102.pcaec365	-0.01172366	0.022457936	0.602494213
pcaec445.pcaec386	-0.002319384	0.004443207	0.602508572
pcaac424.transhydroxyproline	0.023366368	0.04478702	0.602706439
pcaac321.smc180	-0.005004374	0.009595117	0.602822858
pcaac426.pcaec365	-0.012862607	0.024664376	0.602856745
pcaac405.pcaac343	-0.01094635	0.020991429	0.602883031
smc181.methioninesulfoxide	0.004663091	0.008942861	0.602908024
c102.methioninesulfoxide	0.007290074	0.013983433	0.602974091
pcaec364.methionine	-0.010774939	0.020669288	0.602998147
pcaec444.pcaec406	0.001048354	0.002011595	0.603099849
serotonin.lysopcac203	-0.005361877	0.010295957	0.603364189
pcaac386.creatinine	-0.009988797	0.019182379	0.603397054
pcaac384.alphaaminoadipicacid	0.000738518	0.001418339	0.603421815
methioninesulfoxide.pcaac342	-0.015371804	0.029526628	0.603480119
pcaec365.pcaec402	0.014946259	0.028710156	0.603491813
pcaac404.pcaac425	-0.026155751	0.050250741	0.603551972
putrescine.pcaac322	0.01802979	0.034644208	0.603605844
smc241.methioninesulfoxide	0.004772028	0.009169978	0.603626965

c51.c16	0.016445926	0.031604213	0.603644725
lysopcac204.pcaec301	0.014389697	0.027653967	0.603660663
pcaec424.pcaac321	0.005156014	0.00990899	0.603668606
pcaec363.asparagine	0.006882294	0.013226788	0.60367348
c141oh.c182	0.018643117	0.035830802	0.603687483
c181.alanine	-0.012956683	0.024904975	0.603732673
pcaac425.alphaaminoadipicacid	-0.001235687	0.002375243	0.603738677
pcaec363.h1	-0.00221633	0.004260331	0.603746506
pcaec421.pcaac402	-0.086748518	0.166764474	0.603773635
pcaac403.lysopcac181	0.028127844	0.05407911	0.60381661
c142.pcaec322	-0.023410235	0.045012756	0.603847197
c5.pcaec425	0.005158042	0.009918443	0.603871151
lysopcac140.pcaec380	-0.021166853	0.04070263	0.603877436
pcaac363.smc181	0.0070161	0.013491968	0.603888564
glycine.pcaec340	0.012491103	0.024024949	0.603956926
pcaec300.pcaec406	0.001414179	0.00271999	0.603957913
putrescine.ornithine	-0.019293551	0.037112725	0.603997699
pcaec405.c142	0.022316912	0.042928705	0.604000198
ornithine.smohc141	-0.011210054	0.021564037	0.604007422
pcaac386.smc240	0.012384634	0.023824343	0.604020226
pcaac405.h1	-0.000706807	0.001359751	0.604037593
pcaec362.serotonin	0.052247841	0.100525968	0.604080273
serine.pcaac381	0.013691812	0.026344165	0.60409158
c51dc.pcaec406	-0.001954133	0.003760487	0.604146833
smohc222.c18	-0.013548445	0.026074835	0.604181941
pcaac240.pcaac366	-0.005665814	0.010905031	0.604208969
methionine.pcaac380	0.022376695	0.043070687	0.604226632
pcaac386.pcaec421	0.001244107	0.002394963	0.604272517
c181.pcaec425	-0.002890801	0.00556512	0.604285307
arginine.c16	0.012887487	0.024814446	0.604351795
pcaac404.serine	-0.015205431	0.02928319	0.604421049
pcaac401.spermidine	-0.026508375	0.051051825	0.604428143
c102.tryptophan	-0.00372279	0.007169988	0.604446227
serine.pcaac383	-0.003291385	0.006339795	0.604484943
methioninesulfoxide.pcaac363	-0.00916164	0.017648989	0.604526628
pcaac424.h1	0.001374237	0.002647902	0.60460445
smc241.asparagine	-0.003586334	0.006910868	0.604638972
c51dc.pcaec421	0.000575463	0.001108948	0.604649102
lysopcac161.phenylalanine	-0.017784565	0.034273142	0.604662957
pcaec360.pcaac406	-0.032675107	0.062982711	0.604740797
pcaac403.pcaec420	0.026224186	0.050554527	0.604785402
pcaac381.threonine	-0.018073118	0.034846639	0.604843378
pcaac343.pcaac323	-0.035202266	0.067874913	0.6048524
pcaac420.pcaec386	-0.002482518	0.004786905	0.604872017
pcaac343.pcaac362	-0.003266473	0.006298963	0.604895031

smc260.glycine	-0.017056956	0.032894416	0.604920503
glutamicacid.lysine	0.064398914	0.124207625	0.604961244
c102.pcaec363	-0.010396615	0.020061714	0.605132584
pcaac381.pcaac342	0.012060041	0.023272316	0.605144846
pcaac341.pcaac362	-0.002229627	0.004302527	0.605145359
c141oh.kynurenine	-0.002172217	0.004191964	0.605164425
pcaac344.c4	-0.00858021	0.016558207	0.605165251
pcaec340.c182	0.011598197	0.022383791	0.605188306
prolinr.pcaec421	0.003688687	0.007120197	0.605252226
pcaec423.serine	-0.010287844	0.019862862	0.605332962
pcaac384.pcaec341	0.025165972	0.048591566	0.605357639
pcaec420.smc161	-0.04280994	0.082660591	0.605363201
citruline.smc161	0.023162663	0.044725188	0.60537133
pcaec425.pcaac424	-0.005447525	0.010518796	0.605373884
pcaac361.smc181	-0.012460155	0.024060051	0.605379145
glutamicacid.lysopcac170	-0.005847552	0.011292443	0.605412929
pcaac321.smohc241	-0.016377986	0.031629303	0.605425622
glycine.pcaac362	-0.015579041	0.030097279	0.605556197
c0.methioninesulfoxide	0.007479578	0.014451448	0.605595815
pcaac323.pcaac360	0.018307143	0.035381367	0.605695146
smc161.pcaec380	0.015850284	0.030635392	0.605721979
pcaec405.pcaec421	-0.00139629	0.00269903	0.605759785
lysine.pcaac361	-0.020464598	0.039560996	0.605786333
lysopcac203.pcaec423	0.012331495	0.023842846	0.605851296
c5.lysopcac140	0.001706351	0.003299413	0.605872884
smc202.lysopcac161	0.031612068	0.061132483	0.605914942
phenylalanine.pcaec421	0.000706915	0.001367588	0.60605497
pcaec382.transhydroxyproline	0.031610128	0.061157841	0.606086301
pcaac380.pcaac322	0.025370866	0.04908782	0.606096713
pcaac344.leucine	0.003203135	0.006197575	0.606103431
smc161.pcaac380	-0.024967399	0.048318196	0.606178698
c0.lysopcac203	0.006972026	0.013493658	0.606206266
pcaec422.tryptophan	-0.001826168	0.003534599	0.606230037
lysopcac280.pcaac360	0.026314738	0.050941412	0.606290126
c141.pcaac385	0.002647967	0.005126825	0.606343144
pcaec422.alanine	-0.008721733	0.016886849	0.606351345
lysopcac170.histidine	0.01941195	0.037586956	0.606369691
kynurenine.pcaac384	0.016873787	0.03267263	0.60637263
c4.lysopcac181	-0.025226373	0.048846536	0.606378768
pcaec424.smc241	0.007492818	0.014510805	0.606434542
asparticacid.pcaec406	-0.00327478	0.006342959	0.606487094
smc202.leucine	0.010423134	0.020190075	0.606511806
smohc221.pcaac424	0.003535367	0.006848245	0.606516132
pcaac383.methioninesulfoxide	0.008932742	0.017304196	0.606534179
pcaec401.tyrosine	0.024716904	0.047886187	0.606575191

pcaec385.pcaec406	0.014718688	0.028516804	0.606588082
pcaac363.valine	0.025037678	0.048526002	0.606711159
pcaac343.pcaac360	-0.018684485	0.036213138	0.606714948
pcaac344.c141oh	0.030593417	0.059302254	0.606763092
smc181.c51dc	-0.048093931	0.093232103	0.606789558
pcaec322.smc241	-0.018409171	0.035697208	0.606892824
c101.pcaec406	-0.00132899	0.002577612	0.606972115
pcaec424.pcaec406	0.002119263	0.004110387	0.606973799
pcaec363.smc202	-0.026502651	0.051406158	0.606996895
asparagine.pcaec343	-0.029568266	0.057360014	0.607044507
pcaac406.c102	0.012153881	0.023581134	0.607099344
lysopcac140.tryptophan	-0.001012294	0.001964074	0.607100482
pcaac385.pcaec444	-0.037213496	0.072222346	0.607199735
pcaec385.pcaec382	-0.009721667	0.018873177	0.607309803
c18.pcaac380	0.026884086	0.052206088	0.607410304
pcaec385.pcaac342	0.014267785	0.027707158	0.607418205
c10.smohc222	-0.001180778	0.002293025	0.60742228
pcaec360.acetylornithoine	0.006641552	0.012898802	0.607454852
pcaac300.smc260	-0.01209462	0.023489593	0.607457739
pcaec425.pcaec383	0.034790435	0.067569937	0.607466316
smohc241.pcaac406	0.024951563	0.048471499	0.60754484
glutamine.pcaac380	-0.028308094	0.054992513	0.607548345
smohc141.pcaec360	-0.003900415	0.00757873	0.607624927
smc160.pcaec343	0.032013605	0.062225739	0.607748688
pcaac281.pcaac362	0.002171763	0.004222012	0.607807803
pcaac365.glutamicacid	-0.020521281	0.039905152	0.607904768
pcaac424.c51dc	-0.057975971	0.112772196	0.608011662
pcaac363.phenylalanine	-0.019382767	0.037708471	0.608068728
glycine.smc180	0.011433785	0.022245118	0.608086506
pcaac366.pcaec383	0.052328874	0.101816966	0.608114621
smc260.pcaac366	-0.004093851	0.007965878	0.608133502
c51.pcaec444	0.032640031	0.063519845	0.608180725
pcaec360.pcaec301	-0.02508996	0.048831691	0.608216134
lysopcac180.c102	0.014145221	0.027530686	0.608220676
lysopcac182.c51dc	0.054361913	0.105811006	0.608244451
c2.alanine	-0.022371661	0.043545431	0.608251259
pcaac320.pcaac342	0.006463009	0.012580415	0.608264295
pcaec386.lysopcac140	0.000581584	0.00113222	0.608312053
pcaac381.pcaec406	-0.007373071	0.014354644	0.60833345
ornithine.pcaec361	-0.006850744	0.013338078	0.608342917
pcaac381.histidine	-0.028075761	0.054666552	0.608371518
c182.pcaec384	-0.003843159	0.007484957	0.60846291
pcaec380.c51dc	-0.056302021	0.109671298	0.608519004
pcaac362.threonine	0.012424915	0.024204173	0.608541935
c141.pcaec402	-0.018247355	0.035556107	0.608638787

smc260.glutamine	0.011718487	0.022837474	0.608690084
pcaec383.pcaec341	-0.017387253	0.033896386	0.608810329
pcaac421.pcaac381	0.024036673	0.046861911	0.608829455
pcaec301.threonine	-0.009477722	0.018478185	0.608837456
pcaec382.pcaac360	-0.032922358	0.06418719	0.608839172
c182.lysopcac203	-0.007198306	0.014037381	0.608920201
pcaec382.ornithine	0.015790051	0.030795579	0.608960607
c162.pcaec364	-0.007092132	0.013832004	0.608963467
c162.pcaec380	-0.024326351	0.047453551	0.609032201
pcaec406.pcaec383	0.035313459	0.068898112	0.609094234
smc181.alphaaminoadipicacid	0.003624827	0.007072964	0.609133073
pcaac401.methionine	-0.024743446	0.048292015	0.609216172
pcaec425.pcaac342	0.00345103	0.006735832	0.609238698
pcaac363.pcaac320	-0.013952973	0.02723443	0.609246135
pcaec405.smc241	0.010836783	0.021152209	0.609249276
glycine.methionine	0.011097824	0.021665942	0.609318728
smohc222.pcaac385	-0.008299948	0.016213166	0.609526739
pcaac362.c2	-0.029826729	0.058266826	0.60954588
pcaec404.asparagine	0.005836299	0.011404575	0.609649388
pcaec340.c18	0.013288524	0.025971715	0.609717114
pcaac424.pcaec405	0.046318781	0.090535616	0.609748739
c12.taurine	-0.009939981	0.019430169	0.609772322
leucine.lysopcac281	-0.00547114	0.01069514	0.609786813
creatinine.tryptophan	0.001464986	0.002863805	0.609787955
asymmetricdimethylarginine.pcaec38	0.003896721	0.007617949	0.609811586
c51.pcaac383	-0.001405376	0.002747614	0.60983173
pcaac406.pcaec362	-0.00666384	0.013031673	0.609924135
pcaac365.pcaac341	-0.014084689	0.027549588	0.609999927
pcaac322.pcaec401	-0.012572475	0.024591895	0.610002691
pcaec321.pcaec341	0.030089471	0.058856619	0.610010543
isoleucine.serotonin	0.041865033	0.081906143	0.610079828
pcaec362.tryptophan	0.001124253	0.00219999	0.610155182
c141.smc241	-0.012129447	0.0237401	0.610224849
pcaac421.pcaec425	-0.002707682	0.005299565	0.610225759
pcaac300.pcaac323	-0.01501218	0.029395731	0.610388303
serine.glutamine	-0.00504762	0.009884657	0.61041662
pcaec300.prolinr	0.014358764	0.028119076	0.610424024
pcaec342.tryptophan	0.000663362	0.001299227	0.610465479
pcaac323.smc180	0.005522276	0.010816766	0.610502288
pcaac320.c12	0.015012073	0.029405571	0.61051022
pcaec445.pcaec446	0.060809259	0.119117637	0.610524518
c8.pcaec380	-0.03250903	0.063682048	0.610529982
lysine.pcaec384	0.005633224	0.011035992	0.610563931
methionine.lysopcac182	-0.00912536	0.017881524	0.610646172
pcaac405.alphaaminoadipicacid	0.000800744	0.001569101	0.610648156

pcaec381.kynurenine	0.005217531	0.01022482	0.610675845
c5.pcaac366	-0.023345301	0.045769122	0.610825555
asparticacid.phenylalanine	-0.032983074	0.064667617	0.610844135
pcaec406.pcaec425	-0.001684484	0.00330322	0.610905439
pcaec422.pcaec406	0.003467972	0.006800991	0.610926932
methioninesulfoxide.pcaec405	0.051361185	0.100726375	0.610936392
pcaec340.lysopcac182	-0.007209091	0.014138389	0.610945605
pcaac321.c101	-0.001940849	0.00380669	0.610975326
c141.pcaec385	-0.023155478	0.045420336	0.611008935
pcaec443.smc241	0.005182572	0.010167996	0.611085567
spermidine.acetylornithoine	-0.053612717	0.10521982	0.611200141
putrescine.tryptophan	0.000918246	0.001802385	0.611248452
pcaac342.pcaec301	0.029233739	0.057385198	0.611270465
pcaec362.h1	0.000649641	0.001275462	0.61133462
c141oh.pcaec322	-0.016586341	0.032570328	0.611398369
lysopcac204.pcaec425	0.00201158	0.003950222	0.611408706
smc180.lysopcac140	-0.000361458	0.000709815	0.611411174
smohc222.pcaec401	0.031730342	0.062326714	0.61150322
isoleucine.kynurenine	-0.003111607	0.006112237	0.611516294
pcaac343.smc161	-0.02383213	0.046818229	0.61154635
pcaac422.pcaac344	-0.020450382	0.040190901	0.611689071
pcaec364.pcaec425	0.002714014	0.005334347	0.611724189
pcaac380.threonine	0.011496623	0.022603743	0.611839578
pcaec300.acetylornithoine	0.004657583	0.009158603	0.611887532
pcaac321.putrescine	0.015957271	0.031385801	0.611974276
alanine.pcaec386	0.004766286	0.009374917	0.611984721
lysopcac182.histidine	0.017633021	0.034704765	0.612209817
pcaec365.pcaec425	-0.015888916	0.031274827	0.612241119
pcaac366.pcaac240	-0.010138398	0.019956994	0.612261701
smc202.asymmetricdimethylarginine	0.008452445	0.016640027	0.612299233
pcaac320.pcaac426	0.022218758	0.043747448	0.612349364
lysopcac160.pcaec364	0.004394855	0.008653594	0.612364944
citruline.lysopcac182	-0.009623958	0.018950926	0.612385161
pcaac422.pcaec365	-0.012001527	0.023633018	0.612390054
ornithine.lysopcac160	0.004199848	0.008272938	0.612507451
asparagine.glutamine	0.014124279	0.027825804	0.612552583
pcaec360.pcaec365	-0.013539525	0.026674358	0.612560039
lysine.pcaac424	0.004949317	0.009751715	0.61259699
smc181.putrescine	0.013156252	0.025925659	0.612647581
pcaec421.lysopcac161	0.038154808	0.07518984	0.612657603
pcaec444.asymmetricdimethylarginir	0.008607266	0.01696204	0.612660036
pcaec300.smc161	-0.036221889	0.071383788	0.612672811
pcaec361.serine	-0.010724624	0.021138835	0.612730414
lysopcac204.pcaec383	0.041828995	0.082456056	0.612768145
c3.tryptophan	0.000552202	0.001088559	0.612775161

pcaac300.pcaec404	0.006735879	0.01328003	0.612816896
pcaec322.lysopcac181	0.022209245	0.043786715	0.612819995
pcaec360.tryptophan	0.000741886	0.001462799	0.612851851
kynurenine.pcaac300	0.017497425	0.034512766	0.612981017
c181.spermidine	0.0296388	0.05846517	0.613006311
pcaec340.pcaac320	0.038078285	0.075124941	0.613063623
glycine.c141	0.017586173	0.034696578	0.61307059
pcaac426.pcaac380	-0.021486981	0.042393105	0.613074304
smc161.pcaac360	-0.017888647	0.035295665	0.613093939
pcaec425.taurine	-0.022037269	0.043482042	0.6131007
pcaec364.smohc241	0.026293582	0.051891232	0.613175895
pcaec341.lysopcac203	0.006924807	0.013666818	0.613188548
pcaac360.ornithine	-0.008960722	0.017686557	0.613221664
c5.smc241	0.015905583	0.031399635	0.613282666
pcaac426.pcaec384	0.007318454	0.014448188	0.613298195
c14.pcaec365	-0.009667802	0.019086637	0.613304446
c51.phenylalanine	0.018929588	0.037375285	0.613338467
c0.pcaac424	0.002411983	0.004762622	0.613361735
c14.serine	0.007847879	0.015496597	0.613371681
pcaec401.pcaac360	-0.013613274	0.02688114	0.613372552
kynurenine.pcaec383	0.033960959	0.067063412	0.61338936
pcaac403.c14	0.02708166	0.053478795	0.613389879
lysopcac281.pcaec384	0.013829564	0.027310616	0.613403479
lysopcac180.pcaac424	-0.002902064	0.005732328	0.613485973
lysopcac203.glutamicacid	-0.019980705	0.039467772	0.613492286
pcaec425.pcaec386	-0.002001021	0.003953596	0.613581058
ornithine.pcaac422	-0.010231244	0.020217214	0.613623709
smohc222.pcaac366	0.004496322	0.008885208	0.613637829
glycine.pcaec385	0.018724706	0.037004799	0.613664635
c142.c162	-0.049892603	0.098639161	0.613803429
lysopcac203.pcaec421	0.001385152	0.002738498	0.613804973
c12.pcaac424	0.003325806	0.006576192	0.613855905
c102.threonine	0.01316186	0.026026357	0.613871031
pcaac424.pcaac364	0.023671604	0.046812655	0.61390322
alphaaminoadipicacid.pcaec421	0.001123706	0.002222313	0.613916961
pcaac360.smc241	0.041925331	0.082916676	0.613927516
pcaec321.tryptophan	0.000517382	0.001023251	0.613932298
methionine.tyrosine	-0.026313417	0.052047487	0.613974105
pcaec404.c8	0.015207008	0.030090058	0.614101863
pcaec406.c142	0.026295902	0.052050013	0.614226957
pcaac385.pcaac366	0.002469751	0.004888662	0.614230256
pcaec340.glutamicacid	-0.016948799	0.033552454	0.614269807
lysopcac203.pcaec342	0.043654349	0.086422739	0.614282153
c141.smc180	-0.00573309	0.011350398	0.61429996
pcaac426.lysopcac203	-0.013648323	0.027022207	0.614315576

c18.pcaac404	0.002031968	0.004023496	0.614352342
pcaec405.smohc141	-0.008245625	0.016328514	0.614382005
lysopcac161.h1	-0.001305363	0.002584992	0.614385959
smohc141.c12	0.027551957	0.054563217	0.614401905
pcaec425.smc161	-0.025362826	0.050228793	0.614408079
smc180.pcaec446	-0.010000843	0.019807912	0.614446336
putrescine.pcaac362	-0.001636867	0.003242183	0.61446435
pcaac420.pcaec425	-0.001848364	0.003662887	0.614636794
pcaec405.putrescine	-0.031435459	0.062297913	0.614651228
smohc241.methionine	0.012454075	0.024683621	0.614686798
lysopcac140.pcaac380	-0.01757055	0.034825671	0.61470044
pcaac381.pcaac344	0.036692088	0.072731468	0.614729558
pcaec383.threonine	-0.010133823	0.020089533	0.614767486
pcaac401.asparagine	-0.007096797	0.014070996	0.614821135
pcaec445.threonine	0.008244435	0.016347754	0.61484942
lysopcac180.pcaec386	0.004019978	0.007971545	0.614867034
pcaec340.pcaec404	-0.011667917	0.023143622	0.614963974
pcaec385.pcaec422	-0.014540054	0.028842889	0.61499225
lysopcac203.smc240	0.021374394	0.042400752	0.614997942
c2.c51dc	0.041874656	0.083086668	0.615079562
pcaac403.smc241	0.015172674	0.030105428	0.615081663
pcaec322.tryptophan	0.000884048	0.00175421	0.615100378
c51dc.pcaec385	-0.02734547	0.054261765	0.615102692
pcaec403.pcaec401	-0.031798914	0.063104063	0.615132376
lysine.c16	0.022157555	0.043978308	0.615190643
c4.pcaac366	0.010042644	0.019935389	0.615239387
pcaec362.smc160	0.050876635	0.100997828	0.615253233
c3.pcaac384	-0.015350356	0.030476913	0.615301087
pcaec363.histidine	0.027861306	0.055328199	0.615376317
pcaec402.phenylalanine	-0.020786729	0.041299709	0.615551674
lysopcac170.smc241	0.009592811	0.019064126	0.615641169
glutamine.lysine	-0.032515441	0.064637482	0.61574172
lysopcac170.pcaac322	0.027795094	0.055255287	0.615750592
pcaac402.methionine	-0.017604904	0.03500405	0.615814694
glutamicacid.pcaac364	0.03457195	0.068753767	0.615886151
c5.glutamicacid	0.025276671	0.050269399	0.615895234
pcaec406.lysopcac140	0.000912891	0.001815602	0.615909958
asparagine.asymmetricdimethylargin	-0.00613118	0.012197868	0.616021913
c162.isoleucine	-0.007472175	0.014867057	0.616052849
pcaec382.c102	-0.018720488	0.037253022	0.616106719
lysopcac281.pcaac385	0.019506278	0.038820438	0.616140542
pcaac384.asymmetricdimethylarginin	-0.006351675	0.012644293	0.616238132
asparticacid.serine	0.011200954	0.022311731	0.616458766
asymmetricdimethylarginine.kynuren	0.002181208	0.004345468	0.616508476
pcaec403.c8	0.017281816	0.034434373	0.616559752

glycine.pcaac240	-0.004984785	0.009933783	0.616612789
pcaec425.putrescine	-0.021659132	0.04317494	0.616712065
pcaec405.lysopcac281	0.007289184	0.014530725	0.616726423
pcaec341.threonine	0.011183455	0.022298051	0.616793286
pcaec321.c51	-0.022744769	0.0453511	0.616805753
lysopcac160.pcaac384	0.023999145	0.047854451	0.616822183
pcaac380.pcaac342	0.008297753	0.016545827	0.616823318
pcaac424.pcaac421	0.013797286	0.027513284	0.616840156
c101.smc261	0.016216961	0.032339934	0.616857093
pcaac425.arginine	-0.01398281	0.027885473	0.616868353
alanine.pcaec340	-0.025960003	0.051781428	0.616937815
lysopcac180.smc180	0.003645496	0.007272333	0.616976593
c142.lysopcac180	-0.014389099	0.028706388	0.616999107
pcaac361.c16	0.014814304	0.029556733	0.617023586
lysopcac180.pcaac404	0.002001979	0.00399453	0.617048747
acetylornithoine.prolinr	-0.017420459	0.03476222	0.617082508
smohc161.pcaac360	0.024305624	0.048503384	0.617096572
pcaec343.tryptophan	0.000852345	0.001700935	0.617102364
pcaac406.smc241	0.006458093	0.012888007	0.617109648
pcaac425.pcaac364	0.026706881	0.053297679	0.617112647
lysopcac170.pcaec384	-0.012178126	0.024304009	0.617122638
pcaec363.pcaec341	-0.040456195	0.08074022	0.617128659
putrescine.serine	-0.006687614	0.013349457	0.61719951
pcaec301.pcaec402	0.014985835	0.02991499	0.61721208
c51dc.pcaec420	0.025603021	0.051109222	0.617212188
smohc222.pcaec384	0.013302581	0.026556358	0.617232066
c141oh.lysopcac160	0.005749996	0.011482848	0.617353069
pcaec363.c162	-0.039210419	0.078314194	0.617399152
pcaec425.alanine	-0.005411619	0.010809469	0.617429987
c51dc.pcaac344	-0.018968009	0.037888471	0.617436642
pcaec421.c102	-0.01588843	0.031748504	0.617563653
pcaac363.asparticacid	0.011256889	0.022495316	0.617589076
pcaac424.pcaac365	0.026572773	0.053104499	0.617605839
c8.pcaac281	-0.012965353	0.025910925	0.617609189
pcaec360.tyrosine	-0.025748625	0.051464263	0.617652184
glycine.serine	-0.011383216	0.022752297	0.617659116
pcaac383.pcaec444	0.038077276	0.076111519	0.617678714
smc202.pcaac362	0.001961751	0.003921406	0.617689555
smc202.pcaac381	0.017377438	0.03473839	0.617710602
smc240.pcaac401	-0.00855635	0.017106624	0.617752502
pcaec300.tryptophan	0.000656769	0.001313246	0.617799311
pcaec385.pcaac361	0.031576974	0.063145659	0.617831141
pcaec321.pcaac360	-0.026426926	0.052849555	0.617848683
pcaec300.lysopcac182	-0.007975875	0.015951409	0.617869733
serotonin.smc160	0.036415374	0.072829753	0.617872439

lysopcac182.pcaec422	0.009061791	0.018124096	0.617887184
ornithine.pcaec386	0.003159897	0.006321046	0.617946763
c16.asparticacid	0.011444125	0.022895499	0.617988406
pcaec360.c12	0.020036424	0.040090415	0.618031118
kynurenine.c51dc	0.048129297	0.096304531	0.618044735
arginine.valine	0.017200736	0.034423962	0.618106666
pcaac420.c8	0.015969373	0.031960594	0.618117285
pcaec384.serotonin	0.04550247	0.091080833	0.618169856
pcaac385.alphaaminoadipicacid	0.00057004	0.001141128	0.618199812
citruline.pcaec380	0.015662414	0.031356724	0.618234457
pcaac321.tryptophan	0.000888756	0.001779365	0.618243074
pcaec403.putrescine	-0.033058279	0.066197076	0.618304408
c51.pcaac365	-0.016487505	0.033016682	0.618320494
lysopcac280.smc261	-0.024504198	0.049076391	0.618363825
c18.pcaac385	-0.016394582	0.032836763	0.618386332
pcaec364.c4	0.013909176	0.027861924	0.618426493
pcaec446.pcaec403	-0.011680837	0.023398706	0.618433091
pcaac421.pcaec445	0.023306159	0.046692864	0.618483094
pcaac360.c8	-0.022997639	0.046077648	0.618505087
c101.pcaec384	-0.008070995	0.01617213	0.61853182
pcaec445.pcaac360	-0.019225014	0.038525668	0.618566817
pcaec402.isoleucine	-0.011610138	0.023268368	0.618603175
pcaec444.pcaac365	0.023521569	0.047142734	0.618619268
pcaec342.pcaac240	-0.006015472	0.0120566	0.618624657
lysopcac180.pcaac320	-0.014645321	0.029353146	0.618625156
pcaac365.c3	0.010633623	0.021312967	0.618630698
histidine.c4	-0.005397522	0.010819713	0.61867807
pcaac380.pcaec403	-0.010406043	0.020862009	0.618717789
lysopcac180.smohc141	-0.012562382	0.025186338	0.61873594
lysine.pcaec406	-0.004288036	0.008598744	0.618803381
pcaec341.tryptophan	0.000561902	0.001126819	0.618816704
pcaec443.h1	0.001044913	0.002095781	0.618875327
pcaec363.c182	0.020163857	0.040451853	0.618955246
pcaac281.pcaac363	0.004783113	0.00959699	0.619003282
pcaac361.lysopcac281	-0.010692593	0.021455723	0.619032113
kynurenine.tryptophan	0.001084146	0.002175732	0.619078439
pcaec343.pcaac425	0.037325837	0.074909358	0.619085667
pcaac380.pcaec406	-0.007583839	0.0152205	0.619096403
pcaac281.pcaec363	0.01280611	0.025704459	0.619137853
pcaec401.pcaec421	-0.003270441	0.00656525	0.619181146
pcaec443.c8	-0.01992192	0.039993241	0.619189602
c162.lysopcac182	-0.029785329	0.059799033	0.619218902
c14.pcaec425	-0.00409728	0.008226996	0.619262355
pcaac343.c4	-0.005887807	0.011825566	0.619361262
pcaac363.tryptophan	0.002387587	0.00479545	0.619362674

arginine.pcaac402	-0.019221032	0.038605728	0.619366645
serotonin.pcaac366	0.000799298	0.001605537	0.619396221
pcaec446.pcaec386	-0.002039773	0.004097585	0.619424049
pcaac365.smohc221	0.023371773	0.046951275	0.619431681
c3.alphaaminoadipicacid	0.001220372	0.002451676	0.619443988
pcaac362.smohc141	0.009723018	0.019535976	0.619494772
pcaac364.pcaec423	0.005310083	0.010669579	0.619504399
pcaec420.pcaec380	-0.026036207	0.05231503	0.619506646
pcaec341.lysopcac140	-0.000363842	0.000731378	0.61965164
c4.alphaaminoadipicacid	0.001444334	0.002903532	0.619676139
pcaec382.pcaec340	-0.022414557	0.045074154	0.619787482
smc181.smc202	-0.025428944	0.051137909	0.619801348
smc260.c5	-0.004285851	0.008624409	0.620024887
glycine.lysine	-0.023825221	0.04794522	0.620037803
leucine.lysopcac140	0.000697151	0.001403061	0.620070885
pcaac365.histidine	0.029712016	0.059799421	0.620083262
lysopcac140.lysopcac280	0.002465556	0.004962292	0.620085367
pcaec361.pcaec384	-0.016908848	0.034043912	0.620212467
pcaec404.methionine	-0.020439612	0.041156564	0.620245465
pcaec401.asparagine	-0.004608341	0.009279437	0.620253987
c182.pcaac362	0.002932585	0.00590551	0.620277991
pcaec421.pcaac363	-0.007322565	0.014747711	0.620321832
pcaec386.tryptophan	0.00097046	0.001954657	0.62034704
asymmetricdimethylarginine.pcaac36	0.002586937	0.005211938	0.620444045
glutamine.c18	-0.028923704	0.058274573	0.620453554
pcaac403.lysopcac281	0.006954071	0.014013471	0.620519027
creatinine.pcaec420	-0.025858454	0.05211597	0.620568589
pcaac321.pcaec423	0.004169327	0.008405338	0.620665828
pcaec322.pcaac363	0.002661982	0.005367141	0.620704898
alanine.lysopcac280	-0.008642452	0.017425352	0.620710299
c142.pcaec425	-0.00603116	0.012160352	0.6207109
pcaac426.lysopcac281	0.002822079	0.00569096	0.620767928
pcaec404.h1	0.001027712	0.00207279	0.620822191
pcaec422.pcaac240	-0.005564538	0.011223177	0.620824527
smohc241.putrescine	0.015240828	0.030741952	0.620853575
c5.pcaac361	-0.042349833	0.085439124	0.62091961
smc180.c4	0.005580444	0.011259869	0.620967376
lysopcac182.tryptophan	-0.000418713	0.000845151	0.621090068
pcaac380.pcaac363	0.008548726	0.017255743	0.621102073
kynurenine.pcaac321	-0.006534283	0.013189872	0.621110374
pcaac403.smc160	-0.026552954	0.053602023	0.621131018
pcaac363.threonine	0.01247184	0.025180526	0.621183967
pcaec365.pcaac342	-0.00621831	0.012557074	0.621249655
smc240.pcaec341	0.019619797	0.039623019	0.621279317
pcaac403.methionine	-0.019604573	0.039597926	0.621329063

ornithine.glycine	-0.007923324	0.01600441	0.621342891
serine.lysopcac182	-0.009927034	0.0200577	0.62144668
histidine.tryptophan	-0.000846093	0.001710125	0.621565617
pcaac341.smc181	0.006241633	0.012615724	0.621568882
alphaaminoadipicacid.taurine	-0.013540728	0.027368879	0.62156973
pcaac323.acetylornithoine	0.008801408	0.017792653	0.621628723
pcaac380.smohc241	-0.036093163	0.07296743	0.621641201
pcaec321.c18	-0.010291436	0.020807541	0.62167385
lysopcac204.c102	0.015723289	0.03179663	0.621748349
pcaec362.pcaac381	-0.014113765	0.028544652	0.621783769
citruline.pcaec406	-0.015312987	0.030970623	0.621790286
lysopcac161.pcaac366	-0.003863536	0.007814539	0.621813005
pcaec402.pcaec301	0.02859697	0.057849839	0.621864138
c3.lysopcac170	-0.011236032	0.022730854	0.621880691
pcaac342.methionine	-0.014174122	0.028678088	0.621921761
c8.isoleucine	-0.009793212	0.01981441	0.621923353
pcaac422.c8	0.017208564	0.03481862	0.621932103
pcaec360.pcaac322	0.016561813	0.033511433	0.621946683
pcaec300.c182	0.011463622	0.023199903	0.622010161
pcaec443.pcaac384	0.01756407	0.035552437	0.622074185
c162.pcaac405	-0.019677209	0.039836631	0.622134188
alanine.tryptophan	-0.000586317	0.001187052	0.622148948
spermidine.c182	0.073724827	0.149284272	0.622199227
pcaec321.lysine	-0.030909297	0.062593145	0.622229144
pcaec361.valine	0.013158694	0.026647235	0.622230512
alanine.pcaac362	-0.002644175	0.005355741	0.62230265
pcaec405.pcaac342	0.024154395	0.048944006	0.622441928
lysopcac180.tryptophan	-0.00024647	0.000499508	0.622501312
pcaec364.smc240	0.008799081	0.017836433	0.622575909
citruline.lysine	-0.02943378	0.059677569	0.622651439
c141.pcaec343	0.035594542	0.072178418	0.622698544
pcaac363.smc240	0.016418021	0.033293361	0.622708842
glycine.smc241	0.004652092	0.009436924	0.622825084
pcaac424.c8	0.020178492	0.040934582	0.622840711
prolinr.tryptophan	-0.00073436	0.001489913	0.622880968
pcaac381.tryptophan	0.015669221	0.031792039	0.622896162
pcaec424.c18	-0.01426549	0.028950556	0.622975532
acetylornithoine.serine	0.021711093	0.044061945	0.622984936
citruline.c51dc	-0.047246886	0.095902792	0.62304578
pcaac362.pcaec380	0.021889243	0.044436172	0.62308403
pcaec401.citruline	0.022075197	0.044822457	0.623152123
lysopcac180.pcaac366	0.015995741	0.032497076	0.623351019
pcaac420.pcaec445	0.035158859	0.071436973	0.623389642
smohc221.pcaac422	-0.009424829	0.019156141	0.623506507
creatinine.pcaac381	-0.027389988	0.055672053	0.623515167

pcaec361.c4	-0.006826681	0.013876778	0.623542039
asymmetricdimethylarginine.isoleuci	0.010191949	0.020719406	0.62357478
pcaec364.asparagine	0.003999107	0.008131141	0.623629399
arginine.pcaec403	-0.009818557	0.019963579	0.623631242
isoleucine.pcaec425	0.002032616	0.004133269	0.623669285
pcaec363.pcaac363	0.011522678	0.023432512	0.623690885
c4.tryptophan	0.000632403	0.001286213	0.623733856
pcaec406.pcaec401	-0.011732934	0.02386351	0.623740602
glutamine.pcaec406	0.001283859	0.002611548	0.623782832
pcaec322.smc181	-0.014435086	0.029364956	0.623806284
pcaec425.h1	0.001372481	0.002792257	0.62383755
smc261.pcaac343	0.00736969	0.014994467	0.62386362
pcaac401.lysopcac181	0.028626284	0.058243564	0.62386454
c162.c51	-0.030178102	0.061405724	0.623891658
pcaac341.tryptophan	0.001092649	0.00222375	0.62396208
pcaec362.pcaec343	0.030222307	0.061509412	0.623969096
pcaec406.pcaec420	0.01803495	0.036707794	0.623992532
putrescine.prolinr	-0.008093533	0.016473837	0.624003117
serine.serotonin	-0.030139729	0.061358321	0.624064967
pcaac401.tyrosine	-0.020771642	0.042287855	0.62407351
pcaec362.c182	0.013332128	0.027142911	0.624083187
pcaec385.c142	0.019483084	0.039673955	0.624155652
pcaec404.alphaaminoadipicacid	-0.001663496	0.003387475	0.624160868
pcaac361.pcaac362	-0.003160176	0.006435258	0.62416111
lysopcac160.tryptophan	-0.000275678	0.000561469	0.624216696
pcaec364.glutamicacid	0.010929224	0.022262569	0.624265822
pcaec403.asparagine	0.006356197	0.01294781	0.62427624
lysopcac280.smc181	0.00270817	0.005516713	0.624280593
putrescine.pcaac406	0.022399937	0.04563363	0.624307519
c182.threonine	0.008050931	0.016403196	0.624342728
c18.lysopcac140	-0.000608022	0.001238839	0.624353425
smc161.smc241	0.068692183	0.139963217	0.624362033
pcaec420.smc260	-0.008965469	0.018270282	0.624414368
pcaac404.pcaac406	0.018862709	0.038453128	0.624538216
lysopcac161.pcaac363	0.006668278	0.013594946	0.624567036
pcaac384.tryptophan	-0.003640575	0.007422753	0.624591945
smohc241.smc241	0.026055847	0.05315343	0.62477604
pcaac321.alanine	0.014005529	0.028580788	0.624894375
c16.lysopcac181	0.035900528	0.073264592	0.624909203
smohc161.tryptophan	0.000900216	0.001837546	0.624987523
c162.pcaec422	-0.009286695	0.018956705	0.624995505
lysopcac182.pcaec361	0.002109398	0.004307289	0.625109942
pcaac365.lysopcac280	0.003825122	0.007810814	0.625114413
pcaac421.pcaec301	0.033274405	0.067948621	0.625129779
alphaaminoadipicacid.putrescine	0.009877496	0.020182695	0.625338215

serine.tryptophan	-0.000308938	0.000631282	0.625353883
pcaac422.tryptophan	-0.000819192	0.001673984	0.625364604
lysopcac203.pcaac362	-0.002851405	0.005827363	0.625402534
pcaec446.serine	-0.005985291	0.01223283	0.625425235
lysopcac160.smc180	0.002392534	0.004889916	0.625426469
histidine.alphaaminoadipicacid	0.010021752	0.020483577	0.625441535
pcaec321.isoleucine	-0.010333123	0.021121981	0.625474042
smc180.pcaec365	0.016955693	0.034663668	0.625518522
pcaac425.lysopcac204	0.02152917	0.044017593	0.625550372
smc260.pcaac363	0.005536654	0.011320661	0.625570583
c142.ornithine	0.011254346	0.023012442	0.625584904
c2.pcaec404	0.011559916	0.02363971	0.625620708
pcaec403.smc240	0.022993442	0.047023095	0.625636416
asparticacid.pcaac384	-0.026612888	0.054428505	0.625658021
pcaec385.lysopcac182	0.015194843	0.031080534	0.625704018
glutamine.pcaec384	0.012020041	0.024586772	0.625706666
pcaac300.taurine	0.009273837	0.01897275	0.625766463
smohc222.lysine	0.024750046	0.050637322	0.625785458
smohc241.pcaac425	0.03086461	0.06315757	0.625841009
lysopcac203.smc161	-0.041235318	0.084393103	0.625899017
smohc161.c102	0.012569961	0.025730068	0.625954158
smohc141.pcaac422	-0.006678411	0.013671368	0.625979444
c4.histidine	0.025029413	0.051239362	0.625990741
pcaec360.pcaec444	0.054521268	0.111626972	0.626030575
pcaac300.pcaec402	0.012134265	0.024849058	0.626104654
pcaac381.c51dc	-0.052237547	0.106986772	0.626145036
lysopcac181.tryptophan	-0.000202913	0.000415609	0.626167351
putrescine.c51dc	-0.044258752	0.090657935	0.626192174
pcaec405.smc260	-0.015572149	0.031901149	0.626232769
c141.c0	-0.000529653	0.001085122	0.626255743
pcaac281.smc180	-0.005774617	0.011833192	0.626328786
lysopcac161.serotonin	-0.049628561	0.101716515	0.626393139
pcaec360.pcaec342	-0.049286467	0.101034374	0.626457964
pcaec404.smc241	0.013103411	0.026864585	0.626501065
ornithine.smc241	0.004329603	0.008878522	0.626577875
lysopcac280.h1	0.000622285	0.001276375	0.626654584
c8.prolinr	-0.018975907	0.038925986	0.626692501
pcaec343.pcaac422	-0.007043646	0.014449907	0.626716693
pcaac380.tryptophan	0.000779978	0.001600128	0.626720434
methionine.alphaaminoadipicacid	0.001263315	0.002593673	0.626983046
c14.pcaac300	-0.015212719	0.031233935	0.626996044
pcaac364.tryptophan	0.001881226	0.003862539	0.627005575
pcaac403.alphaaminoadipicacid	-0.00105458	0.002165349	0.627018597
smc181.pcaac385	-0.006743021	0.013846455	0.627046968
glutamine.pcaac383	0.00656227	0.013475595	0.627054664

pcaac403.pcaac385	-0.0151421	0.03109461	0.627058769
pcaac281.pcaec425	-0.003367314	0.006915039	0.627068328
pcaac366.arginine	-0.023490616	0.048246372	0.627115228
pcaac342.tryptophan	0.000794545	0.001632057	0.627152059
pcaac384.pcaac365	0.016873882	0.03466437	0.627192939
pcaac362.c16	-0.016612695	0.034127992	0.627194769
pcaec405.c141	-0.018504626	0.038020324	0.627246173
methionine.lysopcac140	0.000464966	0.000955552	0.627323369
pcaec321.pcaec363	-0.015279096	0.031403769	0.627363885
pcaac383.tryptophan	0.0012613	0.002592664	0.627398492
pcaec340.tryptophan	0.000630449	0.001296144	0.627458278
smohc221.tryptophan	0.000946844	0.00194663	0.627459708
smohc141.tryptophan	0.000787303	0.001618667	0.627467772
pcaac383.transhydroxyproline	0.00835939	0.017187501	0.627485874
c2.serine	0.021387047	0.043975574	0.627503732
c101.smc241	-0.005170572	0.010631788	0.627509287
smc241.pcaec421	0.002111957	0.00434301	0.627539426
pcaec384.methionine	-0.007848101	0.016139556	0.627556225
pcaec342.pcaac363	0.009821387	0.020197824	0.627559972
smc260.tyrosine	-0.042232372	0.086853353	0.627567394
pcaac402.pcaac422	0.069283822	0.142502485	0.627606572
pcaac426.pcaec360	-0.001864891	0.003835804	0.627616373
c14.c12	0.032057931	0.065948464	0.627668787
smohc241.tryptophan	0.000976421	0.002008804	0.627693689
smc240.kynurenine	-0.002212814	0.004553567	0.627777753
alanine.serine	-0.004804861	0.009888439	0.627809437
pcaac366.tryptophan	-0.015878449	0.032679338	0.627823886
c4.pcaec423	0.005757027	0.011851753	0.627918152
c5.pcaec385	-0.034903244	0.071856482	0.627930664
pcaec422.pcaac380	0.02493975	0.051347372	0.627951214
smohc161.pcaec401	-0.010243624	0.02109215	0.627983701
asparticacid.pcaec322	0.016037732	0.033023488	0.627993808
pcaec405.pcaac385	-0.015301674	0.031521158	0.62813872
lysopcac203.tryptophan	-0.000278122	0.000572942	0.628148561
smc202.smohc141	-0.012721216	0.026206564	0.628153145
lysopcac180.pcaac363	-0.006575495	0.013546424	0.628164821
pcaec405.h1	0.001137471	0.002343712	0.628218156
lysopcac204.tryptophan	-0.000175677	0.000362011	0.628251926
lysopcac170.tryptophan	-0.000282707	0.000582601	0.628273952
pcaac342.threonine	0.009331366	0.019230807	0.628288176
asparagine.alphaaminoadipicacid	0.000767474	0.001581849	0.628326754
smc160.pcaac321	-0.007143372	0.014724845	0.628363413
c181.pcaec343	0.020458904	0.042174576	0.628379914
pcaac386.lysopcac280	0.001999079	0.004121381	0.628415053
pcaac381.pcaec445	0.028634454	0.059035259	0.628422755

c142.alanine	0.014817604	0.030550273	0.628434238
pcaac403.c182	-0.023632415	0.048731481	0.628485119
pcaec406.isoleucine	0.013915609	0.02869785	0.628521171
pcaac320.threonine	0.00975486	0.020120254	0.628572754
pcaec360.pcaac362	-0.002424573	0.005001362	0.628604848
smc261.c4	0.0083544	0.017234104	0.628620937
citruline.c102	-0.014573922	0.030064408	0.628623135
pcaac363.pcaac402	-0.02511777	0.051821428	0.628664263
pcaac385.c142	0.011588219	0.023909007	0.628677322
pcaac320.tryptophan	0.000540421	0.001115051	0.628691639
asparagine.c12	0.037157048	0.076667842	0.628698808
pcaec362.asparagine	0.005335829	0.011009956	0.628708011
c5.pcaac380	0.046143579	0.095217323	0.628724595
pcaec404.c14	-0.03115352	0.064288474	0.628741421
c51dc.c18	-0.015928415	0.032876004	0.628804944
pcaec420.serine	-0.02034414	0.041991173	0.628814585
pcaec422.pcaec385	-0.023889063	0.049315651	0.628867475
pcaec386.tyrosine	-0.024306508	0.050178315	0.628873673
c12.pcaec343	0.033315611	0.068777967	0.628880015
lysopcac182.taurine	-0.018221601	0.037618252	0.628888322
c16.pcaec401	-0.018869763	0.038965767	0.628970975
pcaac343.tryptophan	0.002882795	0.005954735	0.629075022
pcaac402.pcaec406	-0.018550501	0.038318169	0.629075386
pcaec381.pcaec403	0.072041517	0.148815756	0.629088769
c102.pcaac321	0.014024533	0.028971613	0.629103134
pcaac344.lysopcac140	0.001064762	0.00220026	0.629211345
pcaec383.valine	0.011019376	0.022783005	0.629394741
serotonin.pcaec341	-0.024413123	0.05047532	0.629396128
pcaac362.pcaec402	-0.008470761	0.017513984	0.629401462
spermidine.c101	-0.002320512	0.004798675	0.629460606
pcaac341.serine	-0.010605256	0.021937422	0.629560647
pcaec406.tryptophan	0.000752105	0.00155581	0.629571409
serotonin.lysopcac281	-0.007125448	0.014740419	0.629586794
c101.arginine	-0.011514668	0.023825118	0.629654584
pcaac401.smc261	0.016915138	0.035003738	0.6296981
pcaec403.smc241	0.008370103	0.017321578	0.629712135
pcaac420.pcaec362	0.00567462	0.011744484	0.629744
lysopcac203.pcaec401	-0.022370719	0.046310642	0.629825722
smc240.methioninesulfoxide	0.003516097	0.007279395	0.629852132
smc160.pcaac422	-0.008419795	0.01743337	0.629887935
lysopcac204.ornithine	-0.013352365	0.027650938	0.62994451
pcaac364.lysopcac170	0.003739872	0.007747676	0.630072904
lysopcac160.pcaac381	-0.016729159	0.034664928	0.630152752
putrescine.pcaac380	0.016718366	0.034650107	0.630227187
pcaac386.c101	-0.003846985	0.007973228	0.630229505

h1.glutamine	0.002826416	0.005858142	0.630237434
lysopcac161.tryptophan	-0.000340065	0.000705056	0.63034558
pcaec446.pcaec444	-0.053224201	0.110363793	0.630389842
pcaac381.pcaec362	0.00870496	0.018051703	0.630416535
pcaec444.smc180	-0.005045145	0.010463151	0.630445999
pcaec341.pcaac381	0.012515509	0.025956101	0.630447647
lysopcac182.methioninesulfoxide	-0.011300196	0.02343863	0.630491155
pcaec381.pcaac240	0.010443923	0.021669417	0.630599143
pcaac362.c142	-0.019385386	0.040222008	0.630603638
pcaec444.ornithine	-0.013791051	0.028617532	0.630639427
pcaec420.smc180	0.003903798	0.008100905	0.630648361
pcaac424.pcaac384	0.019490511	0.040445896	0.630652346
pcaec406.taurine	-0.028684429	0.0595431	0.630757695
methioninesulfoxide.pcaec406	-0.025576908	0.05309258	0.630758131
smc241.acetylornithoine	-0.007495223	0.015559065	0.630768442
phenylalanine.lysine	0.040500728	0.084074443	0.630770214
pcaec380.c101	-0.010128679	0.021027128	0.63079065
lysopcac170.isoleucine	0.012596716	0.026152765	0.630816652
lysopcac280.pcaac362	-0.001776887	0.003689327	0.630838051
pcaec321.c102	0.009945985	0.020653389	0.63088209
pcaac425.pcaac381	-0.035824482	0.074398623	0.630914673
smc160.tryptophan	0.000722858	0.001501428	0.630966941
leucine.pcaec425	0.001267598	0.002632987	0.630979217
smc202.c102	0.011923248	0.024776679	0.631121723
phenylalanine.tryptophan	-0.000175259	0.000364221	0.631148921
pcaac383.pcaac320	-0.019747563	0.041039424	0.631152523
c10.pcaec301	-0.026200154	0.054453708	0.631180692
smc180.tryptophan	0.000753463	0.001566148	0.631217641
pcaec380.tryptophan	-0.002367286	0.004920716	0.631222988
c4.c182	-0.01671932	0.034755813	0.631247628
smc181.pcaac361	-0.016693902	0.034716194	0.631377474
smohc222.smc241	0.00783634	0.016297345	0.631400493
pcaac343.pcaec445	0.027214937	0.056620095	0.631525823
pcaac363.pcaec341	-0.021356455	0.044437954	0.631574178
smc181.smc240	-0.012251817	0.025496507	0.631617542
pcaec424.c16	0.010669587	0.022204229	0.631623756
pcaac322.tryptophan	0.000763391	0.001588874	0.631666133
pcaac402.tryptophan	-0.000498724	0.001038029	0.631671448
ornithine.tryptophan	-0.000983371	0.002047213	0.63174724
smohc161.c4	0.006394371	0.01331212	0.631750295
lysopcac180.c18	-0.0178757	0.037228516	0.63187834
lysopcac204.putrescine	-0.010118534	0.0210737	0.6318866
pcaec321.pcaac425	-0.041292104	0.086005726	0.631915717
pcaac383.isoleucine	0.016030975	0.03339049	0.63191767
isoleucine.tryptophan	-0.000312707	0.000651441	0.631975929

pcaec381.glutamicacid	-0.036471524	0.076001414	0.632078303
methioninesulfoxide.pcaac361	-0.03231269	0.067337784	0.632092366
c162.smc240	-0.011890764	0.024780722	0.632106858
pcaac381.c102	-0.014307112	0.029818862	0.632134192
c8.pcaac426	0.018458837	0.038478446	0.632192271
c16.glycine	0.018888348	0.03937499	0.63220267
lysopcac280.c142	0.017515263	0.036512871	0.632204896
pcaac365.pcaac344	-0.035797049	0.074627245	0.632221244
pcaec382.phenylalanine	-0.029799414	0.062124248	0.632223787
leucine.tryptophan	-0.000327401	0.000682554	0.632226248
pcaec421.tryptophan	-0.000517935	0.001079825	0.63224303
smohc221.smc160	0.039599299	0.082574849	0.63230751
smohc241.acetylornithoine	-0.008500403	0.017726294	0.63232173
h1.pcaec380	-0.024178493	0.050435903	0.632425211
glutamicacid.c4	-0.011099452	0.023156857	0.632478079
c18.glutamine	-0.008671958	0.018095545	0.632537898
smc181.c142	-0.026595271	0.055496872	0.632545406
c8.pcaec406	-0.001947647	0.004064747	0.632591743
pcaec322.serine	-0.009515374	0.019860958	0.632631701
smohc222.pcaac422	-0.00881079	0.018394548	0.632709938
pcaac381.c162	0.027405209	0.057215833	0.632716855
lysopcac280.c2	-0.039126642	0.081688824	0.632722303
methioninesulfoxide.c101	0.010202811	0.021305347	0.632783911
methioninesulfoxide.tryptophan	-0.00027123	0.000566388	0.632789955
pcaac426.threonine	0.006218016	0.012985407	0.632811254
pcaec383.pcaec322	0.023439636	0.048950702	0.632814637
pcaec406.pcaac240	-0.007380189	0.015414415	0.63285493
pcaec340.methionine	-0.013068269	0.027307944	0.633020271
pcaac323.alphaaminoadipicacid	0.002069158	0.004324201	0.633052473
pcaec362.pcaac281	-0.00077538	0.001620463	0.633062415
pcaac281.pcaac342	0.001844358	0.003854809	0.633088038
c8.pcaac420	0.04069903	0.085065698	0.633098056
pcaac344.pcaec401	0.038124426	0.079694995	0.633142872
histidine.pcaac404	-0.001940101	0.004055735	0.633156467
pcaac281.tryptophan	0.000670694	0.001402075	0.633157807
c142.pcaac426	0.02146926	0.044886749	0.633200206
smc181.c51	-0.014550527	0.030422963	0.633217175
pcaec423.pcaac380	-0.028743071	0.060101678	0.63324113
glutamine.pcaac342	-0.009008227	0.018838533	0.633283609
pcaac424.pcaec340	-0.022554282	0.047168651	0.63329665
lysopcac181.pcaec343	0.03358726	0.070242453	0.633297151
lysopcac182.c102	-0.014998065	0.031372483	0.633366367
smohc241.pcaec420	0.00610238	0.012767084	0.633427962
c181.pcaac364	0.015875072	0.033215998	0.633458532
pcaac403.tryptophan	-0.000505733	0.001058461	0.633554143

pcaec364.c12	0.017329306	0.03627105	0.633573635
glutamicacid.tryptophan	-0.000147599	0.000308962	0.633606512
pcaec401.smc240	0.0095432	0.019983125	0.633722322
pcaec403.ornithine	0.013921276	0.029151373	0.633730619
pcaec421.c8	-0.022047926	0.046170388	0.633742962
pcaec445.c51	-0.028893467	0.060508075	0.633756935
c162.tryptophan	0.00386116	0.008086638	0.633785457
c8.tryptophan	0.000905446	0.001896426	0.633803551
smohc222.methioninesulfoxide	0.003831003	0.008024019	0.633808303
methionine.lysopcac180	-0.020148198	0.042201809	0.633820316
c162.pcaac363	0.002998492	0.006280903	0.633839369
pcaac281.lysopcac204	-0.008145532	0.017063586	0.633864031
pcaac420.c51	-0.027913847	0.058476023	0.6338697
pcaec421.ornithine	0.030881493	0.06469533	0.633882586
pcaec403.tryptophan	-0.000354019	0.000741741	0.633922083
c18.lysine	0.020648815	0.04327186	0.633988566
pcaac383.pcaac420	0.025529163	0.053513407	0.63407875
c8.pcaec343	0.032513213	0.068165627	0.634140788
pcaac360.tryptophan	0.002701753	0.0056649	0.634172865
c182.c101	-0.003893127	0.008163591	0.634200956
pcaac420.lysopcac140	0.000730211	0.001531373	0.634239713
smc180.lysine	0.019205306	0.04028927	0.63434561
pcaec341.pcaec385	-0.021645822	0.045423279	0.634451817
smc160.pcaac343	0.007972673	0.016732913	0.634501082
alphaaminoadipicacid.pcaac344	0.018518812	0.038870912	0.634535387
pcaac240.tryptophan	-0.000721725	0.001514928	0.634542271
lysopcac180.pcaec380	0.015541126	0.032623264	0.634561352
spermidine.glycine	0.06087319	0.127784903	0.63456802
pcaec386.histidine	0.023418402	0.049162024	0.634582837
pcaac366.pcaec445	0.029175715	0.061252406	0.634605551
kynurenine.pcaac342	-0.007676035	0.016116102	0.634622258
c14.c3	0.011702904	0.024573166	0.634656833
pcaac402.alphaaminoadipicacid	-0.000868594	0.001823923	0.634674083
pcaac300.tryptophan	0.00055696	0.001169547	0.634677546
smohc221.lysopcac204	-0.014687187	0.030842846	0.634694498
lysopcac203.threonine	0.008073127	0.016955051	0.634726853
pcaac320.pcaec382	-0.004922681	0.010340305	0.634784771
smc240.pcaec423	0.006888592	0.014469995	0.634789611
c0.lysopcac280	0.00091595	0.001924257	0.634831191
glycine.threonine	0.008447751	0.017751696	0.634914885
serotonin.pcaac422	0.020999456	0.044137807	0.634995831
leucine.pcaec341	0.015986035	0.033608619	0.635079292
smc180.lysopcac204	0.010037638	0.021107092	0.635146982
smohc241.methioninesulfoxide	0.003680436	0.007739922	0.635178393
pcaec446.serotonin	0.047463786	0.099823526	0.635204294

phenylalanine.c51dc	0.047294077	0.099472031	0.635222737
smc260.tryptophan	0.000962824	0.002025464	0.63528778
citruline.smohc241	-0.013833565	0.029107678	0.635362176
c10.pcaac420	0.037913449	0.079789121	0.63542202
pcaac341.pcaac420	0.034485195	0.072586823	0.635480126
c51dc.pcaac405	0.031608472	0.06653918	0.635518113
c181.lysopcac182	0.00867369	0.018261628	0.635566064
smc161.pcaec340	-0.016204841	0.034118185	0.635570373
c51.alanine	-0.00968334	0.020387834	0.635574081
asparagine.pcaec301	-0.030645197	0.064525704	0.635593057
serotonin.pcaac362	0.000452139	0.000952321	0.635702602
lysopcac181.pcaac403	-0.007530219	0.015862882	0.635751737
acetylornithoine.threonine	0.010973614	0.023119413	0.635792676
glutamine.pcaec404	-0.01097266	0.023125297	0.635907889
pcaec362.glycine	0.014987159	0.031603847	0.63609838
smc240.pcaec406	0.003507559	0.007397231	0.636132252
putrescine.alphaaminoadipicacid	0.00127006	0.002678651	0.636153694
pcaec421.pcaac380	0.020928634	0.044142562	0.63617293
pcaac363.pcaec420	-0.014512752	0.030613194	0.636205707
alanine.methionine	-0.007381764	0.015573362	0.636254922
lysopcac281.taurine	0.01311277	0.027665236	0.636268632
spermidine.ornithine	0.067045164	0.141456929	0.636281712
lysopcac160.c141	-0.00985	0.02078301	0.636293696
pcaac424.alphaaminoadipicacid	-0.000532919	0.001124636	0.636354601
c141oh.putrescine	0.010229127	0.021590345	0.636409198
smohc141.pcaec380	0.013974694	0.029496831	0.636418533
glutamine.tryptophan	0.000284121	0.000599886	0.63652095
pcaec361.pcaec363	0.0175094	0.036970224	0.636533195
acetylornithoine.pcaac424	0.004567237	0.009646552	0.636640058
smc160.pcaec365	0.015436623	0.032606369	0.636664542
pcaec404.lysopcac281	0.007336251	0.01549725	0.63668804
pcaec402.tryptophan	-0.001241062	0.002622445	0.636790636
pcaac240.pcaec445	-0.031041126	0.065592486	0.636793639
pcaac362.c101	-0.018072108	0.038188356	0.63679793
asparticacid.tryptophan	-0.000447366	0.000945509	0.636860512
c10.tryptophan	0.001498087	0.003166274	0.636867189
c3.histidine	0.025748916	0.054423772	0.636881295
ornithine.acetylornithoine	-0.010024976	0.021193439	0.636949821
smc181.pcaec443	0.035365286	0.074770678	0.636977763
smc202.methioninesulfoxide	0.007887608	0.016676537	0.636982718
pcaec446.pcaec420	0.009816854	0.020761709	0.63708364
pcaec445.pcaec383	-0.015620825	0.033040352	0.637122314
arginine.tryptophan	-0.000380495	0.000804905	0.637164699
pcaec321.pcaac363	0.003041426	0.006434289	0.637186278
pcaac426.smohc141	-0.004514221	0.009550852	0.637214174

pcaac240.serine	-0.010458106	0.022132341	0.637303269
leucine.pcaec403	-0.03214962	0.068073079	0.637477678
lysopcac281.h1	0.000730652	0.001547341	0.637536704
pcaac366.pcaac406	0.019676817	0.04167506	0.637571982
pcaec382.tryptophan	-0.000800619	0.001696256	0.637683566
pcaec404.tryptophan	-0.000359813	0.000762356	0.637695242
pcaec364.isoleucine	-0.006496509	0.013769876	0.637826245
lysopcac180.alphaaminoadipicacid	-0.001469476	0.00311474	0.637833453
h1.alphaaminoadipicacid	-0.001442617	0.003058133	0.637869092
prolinr.smc180	-0.009101457	0.019294463	0.637881744
lysopcac160.phenylalanine	-0.021441832	0.045461111	0.637925361
c14.glutamicacid	-0.017018968	0.0360847	0.637934428
c182.smc241	-0.002086335	0.004423921	0.637960193
pcaac424.tryptophan	-0.000636879	0.001350471	0.637964125
arginine.spermidine	-0.025555071	0.054189494	0.637971649
pcaec360.alanine	-0.005039443	0.010686525	0.63798401
pcaac322.pcaac426	0.034176094	0.072473183	0.637984717
pcaac364.lysopcac280	0.003037405	0.006441072	0.637985145
c162.pcaec385	-0.023484997	0.049808218	0.638027692
c10.pcaec406	-0.001986937	0.004214255	0.63804795
ornithine.pcaac363	0.005673084	0.012034297	0.638098067
pcaec425.alphaaminoadipicacid	-0.00098836	0.002096808	0.638130374
pcaec403.alphaaminoadipicacid	-0.000676688	0.001435648	0.638142787
smc240.pcaec383	-0.026851597	0.056969933	0.638155325
pcaec361.pcaac321	0.008622619	0.018295388	0.638176071
smohc241.ornithine	-0.009930914	0.021071451	0.638178218
pcaec446.methionine	0.010543676	0.022376702	0.638254571
pcaac384.pcaec343	-0.02829127	0.060049377	0.638294786
smc202.pcaac360	0.018462071	0.039198079	0.638393901
arginine.methioninesulfoxide	0.005057281	0.010738026	0.638411703
pcaec444.lysopcac280	0.003158975	0.006707531	0.638418758
pcaac404.transhydroxyproline	0.010739896	0.02280838	0.638478865
pcaac380.valine	-0.027694833	0.058817562	0.638489619
smohc222.lysopcac281	0.009729328	0.020663371	0.63849738
pcaec381.tryptophan	-0.000560106	0.001189691	0.638532602
leucine.c51dc	0.060553579	0.128624123	0.638547027
c5.glutamine	0.013915526	0.02956275	0.638595365
pcaec382.lysopcac203	0.006164837	0.013098354	0.638633876
serine.c141oh	-0.04158592	0.088365356	0.638665239
pcaec341.c12	0.018251576	0.038784039	0.638678317
pcaac385.pcaec385	0.020200463	0.042928749	0.638704752
c51dc.pcaac361	-0.026326761	0.055948325	0.638706882
pcaec322.alanine	-0.004657119	0.009899918	0.638803044
c14.smc261	-0.010453232	0.022222442	0.638823849
pcaac364.threonine	0.010339323	0.021982883	0.638863461

c8.smc260	0.011134815	0.023674768	0.638871332
pcaec401.pcaac281	-0.000953312	0.002027113	0.638902295
smc260.pcaec422	-0.007382201	0.015698208	0.638918795
prolinr.pcaac366	-0.010465483	0.022257831	0.638964558
pcaac403.pcaac240	-0.009457404	0.020118188	0.639036566
pcaec422.h1	0.001161357	0.002471138	0.639124837
c142.pcaec403	0.012201446	0.025963177	0.639136548
pcaac320.ornithine	-0.010136145	0.021573914	0.639221047
pcaec402.pcaac300	-0.01952475	0.041564665	0.639284785
smc181.pcaec446	-0.009683357	0.02061555	0.639308047
c141oh.serine	0.010644876	0.022662683	0.639309403
pcaac321.pcaac323	-0.013954895	0.029710063	0.639314244
pcaac425.pcaec425	-0.009452074	0.020127789	0.639385118
pcaac322.lysopcac204	-0.012856796	0.027380548	0.639416274
smohc141.lysopcac140	-0.000299051	0.000636958	0.639459421
pcaac425.smc241	0.005958604	0.012691672	0.639466318
alphaaminoadipicacid.c8	-0.017804242	0.037924408	0.639482487
c181.asymmetricdimethylarginine	0.008378047	0.017847553	0.639513754
c18.tryptophan	0.001187679	0.002530127	0.639519389
pcaac425.pcaac344	-0.024027813	0.051214526	0.63970095
pcaac422.pcaec421	0.00321615	0.006855279	0.639708649
pcaec403.serotonin	-0.04148291	0.088428563	0.639735176
pcaec300.pcaec402	0.011064887	0.023588129	0.639753
pcaec362.c51	0.029593174	0.063087002	0.639754432
pcaec321.pcaac281	0.000914403	0.001950656	0.639981339
pcaac321.pcaec422	0.007107218	0.015162724	0.640007891
pcaec381.h1	0.001249817	0.002666619	0.640036435
acetylornithoine.smc241	-0.008846036	0.018875271	0.640059358
pcaec383.tryptophan	-0.000733665	0.001565667	0.640103465
pcaac240.pcaac362	-0.003029792	0.006465981	0.640118757
phenylalanine.alphaaminoadipicacid	-0.000601378	0.001283437	0.640123148
pcaec402.c162	-0.036088675	0.077034314	0.64018924
pcaac344.tryptophan	0.001440979	0.003077115	0.640322085
c3.c12	-0.012207246	0.026075329	0.640419046
pcaec421.pcaec402	-0.025820847	0.055155847	0.640425956
c142.pcaac323	0.0301908	0.064496496	0.640457038
pcaac424.smohc141	-0.008031228	0.017157337	0.640461968
smc240.tryptophan	0.001715322	0.003664542	0.64046669
spermidine.lysopcac170	0.032963025	0.070430641	0.640513219
pcaec423.pcaac421	-0.0262308	0.056048426	0.640526521
pcaac404.smc181	0.006566319	0.014030637	0.640529238
pcaac424.pcaec384	-0.01449208	0.030968993	0.64056069
pcaac342.alanine	-0.005760101	0.012310706	0.640604157
c181.pcaec360	0.003048293	0.006517243	0.640722689
c4.smc260	0.00568793	0.01216094	0.640727083

creatinine.c141oh	0.0431052	0.092172147	0.640771004
pcaec382.pcaec364	0.001805886	0.003862191	0.640827285
smc260.pcaec363	-0.009355909	0.020010839	0.640854943
c141oh.smc181	-0.006195976	0.013252757	0.640868176
pcaac405.lysopcac182	-0.004344145	0.009293458	0.640926884
c3.alanine	0.00372072	0.007959787	0.640928032
histidine.pcaac360	-0.00999581	0.021386641	0.640966514
smc181.pcaec380	0.023150017	0.049550116	0.641096091
pcaec446.asymmetricdimethylarginin	0.009470623	0.020275312	0.641169491
pcaec421.serotonin	-0.045670231	0.09777426	0.641171286
glutamicacid.alphaaminoadipicacid	-0.000385823	0.000826106	0.641214498
lysopcac281.tryptophan	-0.0016692	0.003574217	0.641233589
glutamicacid.c3	0.022135597	0.04740032	0.641247035
c51dc.lysopcac203	0.005905423	0.012646	0.64125633
pcaac321.pcaac362	-0.002208426	0.004730147	0.641325044
c2.tryptophan	0.000386466	0.000827912	0.641387119
pcaec340.pcaec425	-0.001486627	0.003185245	0.641439422
pcaec381.lysopcac203	0.004484632	0.009610081	0.641484996
pcaec424.pcaec425	-0.005578467	0.011956038	0.641540374
glutamine.c8	-0.016129489	0.034571413	0.641558845
pcaac240.pcaac385	-0.015268596	0.032727165	0.641568644
pcaac386.c16	0.017289596	0.037060367	0.64158059
pcaac384.c4	-0.006299819	0.013505943	0.641635768
smc160.glycine	-0.011393135	0.024425385	0.641636779
methioninesulfoxide.lysopcac281	0.00675918	0.01449096	0.641640603
arginine.phenylalanine	0.016267698	0.034876938	0.641647581
smc260.pcaac344	-0.022671701	0.048611687	0.641681581
c141oh.pcaac385	0.003096181	0.006639524	0.641723005
creatinine.serine	-0.009179258	0.019684997	0.641736134
pcaac401.tryptophan	-0.001216397	0.002608607	0.641740408
pcaac362.alphaaminoadipicacid	0.002912896	0.006247124	0.641757137
lysopcac160.pcaec342	-0.040903006	0.087726789	0.641773882
smc261.smc241	-0.044643622	0.095762571	0.641819381
lysopcac281.pcaec300	-0.023836458	0.051134072	0.641844114
pcaac402.pcaec401	0.027743774	0.05951623	0.641845046
smc261.smohc241	-0.024356275	0.052250255	0.641850925
pcaac344.pcaec365	-0.012029303	0.025813346	0.641947702
pcaec342.asparticacid	0.016969628	0.036417367	0.641972511
c10.ornithine	0.012410387	0.026634491	0.64199
lysopcac160.pcaac404	0.001935996	0.00415521	0.642012625
pcaac322.smc180	-0.0031698	0.006805271	0.642108332
phenylalanine.ornithine	-0.023463983	0.050378149	0.642128946
pcaac384.pcaac362	0.001015382	0.002180091	0.642132723
pcaec405.alphaaminoadipicacid	-0.001340348	0.002877856	0.642137403
pcaac344.glutamicacid	-0.021604362	0.046389637	0.642158851

c16.tryptophan	0.001172291	0.002517833	0.642244613
c141.glutamine	-0.004418821	0.009491397	0.642269241
pcaec445.pcaec380	0.02032174	0.04365427	0.6423015
pcaac365.lysopcac182	-0.005035839	0.010818635	0.642328123
smc161.pcaac385	-0.006111539	0.013129777	0.642332847
pcaec360.lysopcac204	-0.010878517	0.023373439	0.642368154
glycine.taurine	-0.012378082	0.026597083	0.642389376
pcaec423.lysopcac160	-0.015913586	0.034194248	0.642392706
pcaec443.c51dc	-0.050568298	0.108666627	0.642417842
pcaec341.pcaac364	0.016742751	0.035982232	0.642451069
lysopcac161.pcaec443	0.034247676	0.073612926	0.642498276
smc240.pcaec446	-0.010130079	0.021774791	0.642512193
pcaec422.serine	-0.010634726	0.022861113	0.642535082
leucine.pcaac361	-0.018584619	0.039951573	0.642542092
pcaec343.pcaac360	0.017067595	0.03669278	0.642563549
prolinr.pcaac380	-0.020228828	0.043507606	0.642705961
kynurenine.alanine	-0.020550902	0.044201927	0.642718088
c10.c18	-0.01201012	0.025832788	0.64272851
smohc221.pcaec420	0.005573312	0.011988933	0.642761552
pcaac405.pcaac361	-0.05762533	0.123963235	0.642770965
c142.smc241	-0.004470746	0.009617855	0.642785305
smc180.asymmetricdimethylarginine	0.007104598	0.015284628	0.642798426
pcaec380.c12	0.023362527	0.050262533	0.642805438
c142.lysopcac281	0.004564432	0.009821358	0.642851514
pcaac342.smc180	-0.002722283	0.005859301	0.642949093
smc261.tryptophan	0.000696192	0.001498697	0.643004358
pcaac425.pcaac361	-0.016109136	0.034690999	0.643126437
smc161.lysine	0.019435181	0.041859284	0.643171239
pcaac426.glutamicacid	-0.024457926	0.052679011	0.643182519
c3.pcaec383	0.025648234	0.05526128	0.643293653
serotonin.smc202	0.015209474	0.032772249	0.643315484
c102.lysopcac182	0.039619364	0.085369682	0.643318615
smc161.serotonin	0.030346736	0.065391599	0.643329155
smohc141.taurine	0.021697144	0.046762124	0.643391534
smohc222.tryptophan	0.000699345	0.001507419	0.643430436
c14.tryptophan	0.000534714	0.001152762	0.643487858
c2.pcaac300	0.0246192	0.053076503	0.643495897
pcaac365.pcaec403	-0.006605429	0.014241988	0.643527345
c142.pcaac366	0.003431726	0.007399986	0.643564584
pcaec444.putrescine	0.015656216	0.033762625	0.643588279
pcaac386.pcaec386	0.005620932	0.012123725	0.643648055
lysopcac160.pcaac320	-0.014460863	0.03119517	0.64369795
isoleucine.c182	0.021656148	0.04672054	0.643723519
pcaec406.pcaec384	0.012564904	0.027108401	0.643737306
pcaec424.pcaec386	-0.00254561	0.005493069	0.643797169

pcaec444.smohc141	0.014890159	0.032133765	0.643826995
pcaac420.c101	0.011192863	0.024155704	0.643839381
smohc161.pcaec340	0.032186854	0.069472695	0.643882916
smc180.c51	-0.013749875	0.0296796	0.643900994
c4.glutamine	0.003989145	0.008612369	0.643964637
pcaec421.serine	0.034008679	0.073423124	0.643964883
smc202.pcaac380	0.011901774	0.025695471	0.643966238
pcaac386.tryptophan	0.000633011	0.001366983	0.644047732
pcaec404.pcaac360	-0.021832066	0.047147759	0.644058753
leucine.smc240	-0.012342104	0.026656796	0.644098782
smc180.pcaec401	-0.00826227	0.017845073	0.644098955
lysopcac280.pcaac363	-0.011997952	0.025916355	0.644135405
lysopcac160.pcaac363	-0.005134635	0.011095107	0.644253615
pcaec341.smc180	0.007443604	0.016088479	0.644337269
pcaac300.pcaec342	-0.034955692	0.075557678	0.644359355
pcaec363.pcaec405	-0.023441721	0.050672931	0.644379044
smohc161.asparagine	0.004052604	0.008761714	0.644431097
lysopcac182.pcaac300	-0.015282012	0.033043364	0.644468305
methioninesulfoxide.lysopcac160	0.010500392	0.022711434	0.644571342
smohc222.lysopcac280	0.003436745	0.007434062	0.644601648
arginine.smc181	0.002751062	0.005951759	0.644651947
pcaac361.tryptophan	0.001346613	0.00291338	0.644659234
pcaac425.creatinine	0.007838219	0.016958944	0.644679712
smohc161.pcaec362	-0.007909328	0.017113227	0.644688029
pcaac386.smc260	-0.014888685	0.032215029	0.644695533
pcaac381.lysine	-0.033891378	0.073335707	0.64471386
pcaec405.c181	0.008082593	0.017490127	0.644726036
c14.smohc141	0.009648662	0.020881632	0.644767954
pcaac426.tryptophan	0.001228062	0.002658176	0.644818077
pcaec425.pcaac363	0.004895784	0.010597256	0.644824175
pcaec420.smohc241	-0.023793863	0.051516942	0.644910831
c162.smohc141	0.005774576	0.012503191	0.644922736
smohc161.asymmetricdimethylargin	0.009167782	0.019855423	0.645009521
pcaec322.pcaac361	-0.026442568	0.057269302	0.645012109
smohc241.c18	0.010401353	0.022531662	0.645076851
c101.pcaac383	0.001469555	0.003183465	0.645085086
c102.pcaac362	0.00126553	0.002742182	0.64516853
smc241.tryptophan	0.001422661	0.00308269	0.645172055
pcaac403.smc161	0.048108641	0.104245281	0.645175656
pcaec322.histidine	0.026129194	0.056626429	0.645221154
c12.tryptophan	0.001653876	0.003584265	0.645224078
leucine.lysopcac280	-0.004047487	0.008771758	0.645227234
pcaec300.pcaac380	-0.017797725	0.038577447	0.645278796
smc240.pcaac406	0.025964666	0.056284012	0.645304118
c4.pcaac321	-0.005637489	0.012222315	0.645353858

pcaec380.spermidine	-0.023772023	0.051545069	0.645394309
pcaec421.lysopcac203	0.041031765	0.088984727	0.645450754
lysopcac180.pcaac364	0.018955018	0.041110012	0.645472128
lysopcac180.c142	0.015054428	0.032654648	0.645515694
pcaec363.pcaac422	-0.007193298	0.015603451	0.645524728
lysopcac204.pcaec343	-0.02880838	0.062495226	0.645551594
lysopcac180.pcaec402	-0.007422129	0.01610409	0.645612123
c5.threonine	0.013668725	0.029663926	0.645682689
pcaec406.smc241	0.007652621	0.016609363	0.645714693
lysopcac140.pcaac425	-0.037832182	0.082111975	0.645716478
isoleucine.transhydroxyproline	0.014435911	0.031335915	0.645756784
smohc222.pcaac362	0.002436609	0.005289167	0.645759184
pcaac362.tryptophan	0.00134086	0.002912222	0.645941195
c141.pcaac363	0.004680309	0.010167365	0.646011552
pcaac404.smc160	0.037090399	0.080580854	0.646039252
asparagine.tryptophan	0.002639199	0.005734519	0.646080675
pcaec443.smc260	-0.013843768	0.030083754	0.646120683
c141oh.pcaec382	0.00427048	0.009282131	0.646191441
smohc141.c8	0.018722156	0.040694095	0.6461949
methioninesulfoxide.pcaec446	0.045754718	0.099461792	0.646228919
pcaec422.pcaac360	-0.013244246	0.028792603	0.64625418
alanine.pcaac424	0.003184629	0.006925327	0.646350979
c162.pcaac421	0.01781025	0.038741298	0.646444063
smc181.tryptophan	0.000842755	0.001833185	0.646444536
lysopcac161.pcaac421	0.017796226	0.038714522	0.646475765
c142.tryptophan	0.001370111	0.002981247	0.646548848
c16.smc241	-0.006133284	0.013346264	0.646567189
smc240.pcaac386	0.01693853	0.036859876	0.646575916
c162.lysopcac203	-0.004965137	0.010805335	0.646597826
citruline.alphaaminoadipicacid	0.000693473	0.001509394	0.646647645
pcaec383.pcaac420	0.048688496	0.105985867	0.646684614
pcaac406.smohc141	-0.005652454	0.012305081	0.646704196
smc181.pcaac360	0.019708518	0.042912665	0.646767885
pcaac341.pcaec445	-0.011492467	0.025025871	0.646801516
pcaec365.c12	0.018553156	0.040407864	0.646856157
smc260.pcaec383	-0.018162523	0.039559254	0.646874206
pcaec382.alphaaminoadipicacid	-0.000597963	0.001302934	0.647007448
lysopcac170.pcaec421	0.002601176	0.005667927	0.647012382
pcaac360.pcaec406	-0.006404921	0.013957009	0.647030659
lysopcac160.pcaac424	-0.001836375	0.004002919	0.647134221
pcaec384.pcaac321	-0.009502171	0.02071619	0.647188429
smc161.pcaac426	0.020093725	0.043810245	0.647209755
isoleucine.pcaec444	-0.033280797	0.072563353	0.6472161
pcaac386.pcaec444	0.056207974	0.122569567	0.647262323
pcaac384.smc260	-0.010667623	0.023265716	0.647310759

pcaac321.taurine	0.005901432	0.012872047	0.64734211
pcaec420.tyrosine	0.027305964	0.059560018	0.647347489
pcaac405.tryptophan	0.002591777	0.005654543	0.647425018
smohc222.pcaac365	0.022109259	0.048238914	0.647442799
c51.leucine	0.015366599	0.033530167	0.647468971
pcaec363.pcaec360	0.002461625	0.005371507	0.647481249
lysopcac170.pcaec444	-0.031304719	0.068310214	0.647482495
pcaec421.pcaac381	0.02046689	0.044674844	0.647584825
asparticacid.alphaaminoadipicacid	-0.000209546	0.000457401	0.647590183
phenylalanine.pcaec365	0.010335516	0.02256085	0.647593997
smohc241.pcaec446	-0.011942986	0.026074125	0.647649538
lysopcac182.prolinr	-0.011875107	0.025925961	0.647649952
pcaec423.ornithine	-0.015677393	0.034230637	0.647683041
pcaac342.arginine	-0.017002441	0.037125531	0.647698365
smc181.pcaec406	0.007317775	0.015980588	0.647737897
smohc222.pcaac343	0.009290855	0.02028945	0.647738558
creatinine.pcaec406	0.002755226	0.006017253	0.647758569
pcaac361.pcaac426	0.023052276	0.050354633	0.647822443
c14.pcaac380	-0.017291607	0.037774607	0.647851889
acetylornithoine.pcaac342	-0.001717788	0.003753078	0.647892319
methionine.pcaac403	0.001720243	0.003758779	0.647921632
pcaac341.isoleucine	-0.009451956	0.020653282	0.647929521
leucine.pcaac365	-0.014586538	0.031877436	0.647977687
pcaec322.smohc141	0.006045165	0.013214395	0.648059246
pcaec424.tryptophan	-0.000531964	0.001162864	0.64806525
pcaac401.pcaec405	-0.046557845	0.101780075	0.648082466
lysine.tryptophan	-0.001132974	0.002477015	0.648111642
smohc141.pcaec301	-0.021639701	0.047319105	0.648169567
smc160.h1	0.000704363	0.001541106	0.64835885
citruline.asparticacid	0.012908628	0.028252095	0.648460326
c16.c8	0.008340814	0.018260191	0.648555581
c51.smc260	-0.007556037	0.016543713	0.64858726
pcaac383.pcaac342	0.004440783	0.0097232	0.64859539
pcaac402.pcaac424	-0.007087559	0.015518426	0.648596375
smohc141.smc161	0.040903952	0.089579144	0.648664729
glutamine.alphaaminoadipicacid	0.000375239	0.000821825	0.648687006
pcaec341.c14	-0.016965537	0.037157906	0.648696377
pcaec423.alanine	-0.005098375	0.011166475	0.648696889
pcaec343.lysopcac161	0.036537809	0.080051271	0.64880351
pcaac405.pcaec384	0.016831273	0.03687601	0.648804429
serine.threonine	0.007119637	0.015600091	0.648836357
smc241.threonine	0.012837256	0.028132508	0.6488868
smohc141.pcaac362	0.002056921	0.004507928	0.648904168
pcaec362.pcaac406	-0.015260342	0.033448068	0.648939938
isoleucine.pcaec384	0.010898103	0.023888082	0.648957714

pcaac300.smohc141	0.012086172	0.026496207	0.649006354
pcaec406.pcaec321	0.01961239	0.043001692	0.649051634
pcaac384.pcaac366	0.00167289	0.00366861	0.649111208
histidine.pcaec343	-0.030443709	0.066770452	0.649150845
smc161.acetylornithoine	-0.0094574	0.020743094	0.649162145
pcaac385.pcaac362	0.001323705	0.002903353	0.649167217
pcaac342.lysopcac281	0.004074179	0.008941132	0.649350874
pcaac344.pcaac320	-0.041670858	0.091453139	0.649361212
pcaec321.acetylornithoine	0.00799997	0.017557429	0.649366035
lysopcac281.smc260	-0.009273392	0.020352325	0.649368178
pcaac422.pcaec403	-0.006231169	0.013676197	0.64938354
pcaec363.pcaac406	-0.024583648	0.053963119	0.649424837
c2.c162	-0.032710865	0.071807425	0.649444873
pcaac405.transhydroxyproline	0.013739117	0.030163907	0.649483572
pcaec382.smc240	0.023562145	0.051734862	0.649513445
methioninesulfoxide.glutamicacid	0.037959591	0.083366633	0.649590394
smohc222.pcaec406	0.002226305	0.004889837	0.649619756
pcaec301.smohc141	0.00211804	0.004652798	0.649672562
pcaac425.c101	0.004336649	0.009528114	0.649727273
pcaec421.lysopcac281	0.004686608	0.010297518	0.649743211
smc202.tryptophan	0.000883953	0.001942589	0.649801808
histidine.pcaac342	-0.001407354	0.003093215	0.649843122
pcaec424.alphaaminoadipicacid	-0.000969116	0.00213008	0.649853134
pcaec422.c12	-0.026723562	0.058739404	0.649864477
pcaac281.ornithine	-0.010048478	0.022090311	0.649914361
smc161.lysopcac140	-0.000690543	0.001518153	0.64993164
pcaec446.pcaec421	-0.000571875	0.001257345	0.649953623
pcaac404.pcaac362	-0.003754965	0.008255974	0.649960073
arginine.pcaac363	-0.005958777	0.013102091	0.649975948
c141.pcaac383	0.006098143	0.01340882	0.649983038
alphaaminoadipicacid.isoleucine	-0.00502718	0.011056619	0.650061925
pcaac406.glutamicacid	-0.013249489	0.029145423	0.65011703
methionine.c12	-0.021837935	0.048039625	0.650129646
smc180.pcaac405	-0.02251915	0.049538898	0.650134391
c18.pcaac321	-0.010853691	0.023878087	0.650155203
c0.tryptophan	-0.001032975	0.002272879	0.650203531
c101.lysopcac280	-0.008962452	0.019720751	0.650211206
pcaac381.isoleucine	-0.008058532	0.017731947	0.650214132
serine.pcaec404	0.00963439	0.021200483	0.650229936
c3.smc161	0.028478108	0.062675702	0.650279909
smc261.methioninesulfoxide	0.006322674	0.013915269	0.650281967
methioninesulfoxide.isoleucine	-0.011473338	0.025252055	0.65029409
smc161.smc181	-0.008459473	0.01861965	0.650310038
c141oh.pcaec343	-0.025185418	0.055450013	0.650403363
alphaaminoadipicacid.c4	-0.007703694	0.016967128	0.65052109

pcaec425.smc261	0.024602546	0.054191566	0.650552864
methionine.smc240	-0.007007079	0.015435135	0.650569189
pcaac402.c18	-0.024220277	0.053362406	0.650631399
pcaec443.c2	0.036758841	0.080994473	0.650659372
lysopcac181.prolinr	-0.017812626	0.039255725	0.650720507
c8.histidine	0.022674861	0.049973102	0.650732979
pcaac383.pcaac323	0.028893065	0.063691169	0.65080343
smohc241.taurine	0.018006198	0.039693549	0.650812671
smc160.c8	0.013685359	0.030170667	0.650835766
pcaac365.tryptophan	0.00156764	0.00345711	0.650939272
citruline.pcaec383	-0.02975637	0.06563044	0.650983234
lysine.asymmetricdimethylarginine	-0.006206697	0.013689452	0.650983315
c102.pcaac366	0.00209195	0.00461459	0.651025552
serotonin.taurine	0.008382226	0.018491734	0.651052895
pcaec424.lysopcac281	0.004754119	0.010489403	0.651099706
pcaac364.c102	0.011772039	0.025974987	0.65111687
lysopcac170.methioninesulfoxide	0.023233692	0.051288555	0.651265689
c162.pcaec361	-0.004756736	0.010503177	0.651347659
pcaec301.glutamine	-0.004369703	0.009650319	0.651406152
lysopcac181.pcaac240	-0.005455428	0.012048675	0.651421642
smohc161.c8	0.015135726	0.033429467	0.651433447
smc180.ornithine	-0.01565923	0.034612457	0.651684869
pcaec405.smc240	0.012605009	0.02786216	0.651692067
pcaac426.pcaac320	-0.047509671	0.105021246	0.651709662
c51.pcaac422	0.013951389	0.030841004	0.651721578
pcaac362.pcaec425	-0.002383918	0.005270204	0.651740359
c141oh.tryptophan	0.000879477	0.001944351	0.651750686
pcaac364.pcaac240	-0.009409729	0.020803122	0.651751777
glutamicacid.pcaac321	0.003672641	0.008119608	0.651755619
pcaac366.pcaec382	-0.005783937	0.012789236	0.651803847
c8.pcaac362	0.002411592	0.005332934	0.651834798
c2.pcaec362	0.008780313	0.019416817	0.651839025
pcaac406.tryptophan	0.000686421	0.001518118	0.651874093
c141oh.pcaac342	0.002386046	0.005278032	0.651932705
pcaac424.smc181	0.002358724	0.005219059	0.6520238
pcaac363.h1	0.000616883	0.001364974	0.652028728
smc241.lysopcac170	0.00501912	0.011109945	0.652150411
pcaec420.c16	0.014311868	0.031680764	0.652161586
c181.lysopcac161	-0.027598022	0.061091999	0.652166864
c102.c51dc	-0.044228905	0.097910974	0.652180919
smohc221.h1	0.000596244	0.001319929	0.652181644
pcaac240.transhydroxyproline	-0.023921904	0.052958025	0.652189029
pcaac320.tyrosine	0.021089448	0.046696621	0.652251973
lysopcac280.tryptophan	-0.000856811	0.001897375	0.652287693
c0.phenylalanine	0.010705348	0.023708562	0.652314872

smohc141.asparagine	0.002915312	0.006456473	0.652319304
pcaec365.pcaec343	-0.041451819	0.091805649	0.652330869
pcaac360.pcaec362	0.010421652	0.023084048	0.652367926
alphaaminoadipicacid.pcaec406	-0.001960044	0.004344328	0.652578162
c14.pcaac424	0.002550243	0.005652552	0.652582731
lysopcac281.pcaac424	-0.004173792	0.009251414	0.652593406
smohc161.smohc221	0.031237124	0.069257785	0.652683237
smc180.pcaac422	-0.008620707	0.019121411	0.652817226
serine.pcaec403	-0.016209891	0.035959153	0.652856258
lysopcac281.pcaac321	0.007104497	0.015762252	0.652897753
lysopcac203.lysopcac180	0.019687106	0.043685908	0.652953367
pcaec443.tryptophan	-0.001048631	0.002327098	0.652977485
pcaac341.lysopcac203	0.008646226	0.019190419	0.653026786
pcaec422.pcaac383	0.003803246	0.008443675	0.653115705
pcaec381.alphaaminoadipicacid	-0.00045095	0.001001268	0.653148851
c181.pcaec384	-0.007952987	0.017659601	0.65317052
c18.tyrosine	-0.01984152	0.044063767	0.653212336
c5.alanine	0.004349856	0.009662262	0.653284822
alphaaminoadipicacid.pcaac366	0.0065645	0.014581808	0.653289155
smc181.acetylornithoine	-0.008775387	0.019495377	0.653330667
c5.pcaec406	-0.003933151	0.008737955	0.653333476
alanine.pcaec425	0.001110893	0.002468069	0.653345448
c141.tryptophan	0.001154433	0.002565061	0.65337825
c101.pcaac366	0.002203215	0.004895481	0.653385153
pcaec446.lysopcac281	0.002800385	0.006223089	0.653422182
pcaec386.acetylornithoine	-0.011238743	0.024982286	0.653516284
smc181.pcaac363	0.002043519	0.004542763	0.653536412
methioninesulfoxide.pcaec362	0.008387022	0.018647863	0.653595898
pcaec342.pcaec446	-0.013906024	0.030919012	0.653596871
pcaec341.smohc161	0.009705986	0.021581743	0.653614943
pcaec405.phenylalanine	-0.016663076	0.037055537	0.653652987
lysopcac180.pcaec421	-0.003076943	0.006843008	0.653675107
pcaec341.pcaac380	-0.02733181	0.060805271	0.653783227
lysopcac170.putrescine	0.018262832	0.040635361	0.653830253
pcaec383.pcaac363	-0.009418881	0.020957432	0.653832317
lysine.smc202	0.018546286	0.041269644	0.653858387
c10.taurine	-0.017685002	0.039363341	0.653942537
lysopcac140.lysine	0.035771104	0.079621337	0.653950203
pcaec420.methionine	-0.012412725	0.027633137	0.653999288
pcaec340.pcaac406	-0.020320258	0.045236845	0.653999296
smc202.glutamine	-0.006958084	0.015492377	0.654047893
pcaac420.pcaec401	-0.012464188	0.027753149	0.6540627
c141.methionine	0.013972455	0.031112702	0.654075133
c182.pcaec403	0.012301503	0.027394171	0.654101168
pcaac384.pcaac361	0.01730159	0.038532584	0.654132511

pcaac342.c182	0.016405501	0.036539816	0.654158358
pcaec422.pcaac381	0.011096624	0.024715621	0.654161189
pcaec301.pcaac360	0.013166131	0.02932687	0.654181187
lysopcac181.pcaac404	0.002541027	0.005660212	0.654192946
pcaac281.pcaec406	0.001243823	0.002770888	0.654220392
pcaac421.pcaac342	0.002806285	0.006252167	0.654249061
pcaec365.pcaac424	-0.006119549	0.013634365	0.654261462
pcaac344.pcaac421	0.024068229	0.053641095	0.654364103
c162.pcaac425	0.016365842	0.036475541	0.654371455
c16.serotonin	0.048424821	0.107937377	0.654401529
smc260.asparagine	0.007498677	0.016714673	0.654408502
pcaac322.pcaec342	0.0346698	0.077281294	0.65441581
asparagine.c51dc	0.04443683	0.099065589	0.654457968
lysopcac182.spermidine	-0.025664789	0.057221828	0.654490881
lysopcac182.isoleucine	0.020610836	0.045953723	0.654491672
pcaec402.alphaaminoadipicacid	-0.000528215	0.00117791	0.654548301
pcaac421.tryptophan	-0.001426538	0.003181242	0.654557173
pcaec403.smc181	0.002003773	0.00446855	0.654560847
alanine.pcaec421	-0.000843657	0.001881511	0.6545776
pcaac404.smc180	0.00441079	0.009838696	0.65463737
pcaec405.smohc241	-0.02594069	0.05788451	0.654756017
smc241.pcaec406	0.002872794	0.006411366	0.654804508
acetylornithoine.alphaaminoadipicaci	-0.001444985	0.003225179	0.654837355
c141.taurine	-0.010189978	0.022744081	0.654840916
pcaec443.alphaaminoadipicacid	-0.000652873	0.001457538	0.654912234
lysopcac280.pcaec406	0.002983573	0.006661685	0.654954001
pcaac401.c101	-0.011676608	0.026074405	0.654991328
pcaac421.transhydroxyproline	-0.014577913	0.032555836	0.655017886
pcaec342.h1	0.001941034	0.00433498	0.655033013
c8.c2	0.020346724	0.045443773	0.655052333
pcaac281.smc260	-0.010632029	0.023746974	0.655061412
spermidine.c3	0.053888161	0.120391629	0.655143647
pcaac383.acetylornithoine	0.004468503	0.009986525	0.655254471
pcaec343.pcaec406	0.001692027	0.003781718	0.655276382
pcaac386.pcaac342	-0.008304347	0.018561609	0.655297396
c101.pcaec401	-0.009519579	0.021278213	0.655302799
pcaac240.pcaec404	-0.033962581	0.075934602	0.655393002
prolinr.c4	-0.004421521	0.0098862	0.655406889
c16.pcaec361	-0.004855227	0.010856492	0.655423384
pcaac300.smohc161	0.009362443	0.020935306	0.655430893
pcaec361.lysopcac180	0.032749984	0.073242943	0.655478769
pcaac320.c51	-0.018112065	0.040512124	0.655525
pcaac281.asparticacid	0.007774322	0.01739002	0.655540205
pcaac402.pcaec420	0.016394431	0.036676846	0.655583269
pcaac320.pcaec406	0.00306108	0.006849086	0.655629432

c0.smc241	-0.008308847	0.018595139	0.655704104
glutamine.smc202	0.020029845	0.04482728	0.655708656
pcaec384.pcaec386	-0.003152486	0.007055857	0.655732192
pcaec421.pcaac365	0.030238045	0.067681445	0.655746516
pcaec321.pcaac343	-0.016309603	0.036509192	0.655778331
c10.alphaaminoadipicacid	-0.0005991	0.001341165	0.655796316
pcaec401.pcaec340	-0.026427462	0.05916356	0.655807964
lysopcac160.pcaec445	0.035703805	0.079935339	0.655826847
pcaec425.pcaac364	0.013637195	0.030535506	0.655868141
pcaec404.pcaac240	0.007703776	0.01725368	0.655940746
pcaec384.isoleucine	-0.00712926	0.015968642	0.655974401
pcaec425.serotonin	0.043424031	0.097300277	0.656093185
smohc141.asparticacid	0.009405641	0.021075354	0.656095084
pcaec360.smohc141	0.025610315	0.057402491	0.656190801
pcaec360.pcaec385	-0.021716715	0.048682903	0.656240041
acetylornithoine.asymmetricdimethyl	-0.009280076	0.020804096	0.656251107
pcaac406.pcaac401	0.00591031	0.013250799	0.656276663
c5.c142	-0.043076751	0.096580611	0.656287904
pcaac321.c16	0.010403532	0.023328706	0.65633441
acetylornithoine.pcaec360	0.003463249	0.00776807	0.656422831
pcaac364.smc260	-0.008827712	0.019800956	0.656429219
pcaac385.transhydroxyproline	0.013134879	0.029463286	0.656441875
c0.pcaac364	-0.009541392	0.02140373	0.656458603
pcaec363.pcaec444	-0.038135505	0.0855546	0.656485327
c10.pcaac362	0.00276363	0.006200758	0.656523025
pcaec321.lysopcac181	0.015584415	0.034969807	0.656551057
smohc141.ornithine	-0.007664614	0.017204721	0.656665405
pcaac323.pcaec365	0.012197085	0.027378816	0.656666318
serine.smc241	0.005022097	0.011273465	0.656676417
pcaec340.pcaec402	0.024614089	0.055260359	0.656719045
pcaec365.phenylalanine	0.009126368	0.020494154	0.656794492
alanine.pcaec363	0.006639514	0.014910516	0.656812492
pcaac425.pcaec404	-0.006945568	0.01559874	0.656831221
pcaec301.pcaec362	0.004294821	0.009645587	0.656832589
pcaac343.pcaac385	0.019973717	0.044860493	0.656848547
c8.alphaaminoadipicacid	-0.00036962	0.000830159	0.656848908
smc202.acetylornithoine	-0.015662277	0.03518804	0.656948312
smc240.methionine	0.009976715	0.022416078	0.656971858
pcaac341.taurine	0.008033442	0.018050625	0.656985545
pcaac344.lysopcac203	0.006446464	0.014488136	0.657059699
c2.pcaac421	0.02159434	0.048533444	0.657067234
pcaec301.h1	0.001423958	0.00320038	0.657069602
pcaac300.smc180	-0.003672367	0.008253902	0.657076122
smohc222.valine	0.033815297	0.076012046	0.657117485
serine.pcaac362	-0.005434388	0.012219104	0.657205736

glutamicacid.phenylalanine	0.025664139	0.057716678	0.657269148
prolinr.pcaec362	-0.00422612	0.009505337	0.657306873
pcaec386.phenylalanine	0.00977209	0.021980056	0.657318392
smohc222.pcaac363	0.002109785	0.00474548	0.657318896
lysopcac181.c8	0.021436705	0.048217042	0.657319269
pcaac404.tryptophan	-0.001681897	0.003784399	0.657433721
pcaac344.pcaac362	0.003155403	0.007102589	0.657554937
pcaec444.pcaec386	-0.002743431	0.00617549	0.657566371
spermidine.asymmetricdimethylargir	-0.036346673	0.081819771	0.657578309
lysopcac170.pcaac422	0.03830635	0.086242075	0.657618744
pcaac420.smohc141	-0.00531276	0.011961348	0.657627247
isoleucine.pcaac424	0.004937171	0.011116108	0.657638034
pcaac342.lysopcac203	0.006719898	0.01513123	0.657665285
arginine.smc180	0.001876245	0.004224895	0.657676228
c141oh.pcaac363	0.004176013	0.009409439	0.65787932
c18.pcaec420	0.016196722	0.036498409	0.657912466
c5.smohc241	0.031443055	0.07085554	0.657914138
c102.pcaac363	0.005856299	0.013197393	0.657925827
c51dc.tryptophan	0.001408829	0.003175046	0.657945459
c10.smc260	0.013066302	0.029450083	0.657976607
pcaac421.smc241	0.004181816	0.00942588	0.657993644
leucine.serotonin	0.033996177	0.07663236	0.658012176
c4.smc241	0.023061978	0.051996528	0.658082664
lysopcac140.pcaac366	-0.008931276	0.020137814	0.658098161
methionine.histidine	-0.028572423	0.064424116	0.658100063
lysopcac160.pcaac321	-0.010744735	0.024227535	0.658109001
c181.smc241	-0.004768252	0.010752697	0.658141893
pcaac424.pcaac360	-0.023616894	0.05325823	0.658146139
c162.lysopcac181	-0.023591391	0.053208413	0.6581924
pcaec384.pcaec446	0.034656294	0.078176887	0.658243578
arginine.pcaec341	-0.01504078	0.033930246	0.658258595
pcaec421.smc181	0.001888634	0.004261259	0.65831267
c0.pcaac426	0.014677455	0.033116589	0.658316179
pcaec402.spermidine	-0.022486816	0.050738114	0.658324645
pcaac321.pcaec363	-0.011249323	0.025388044	0.658395684
c162.smc241	0.004928601	0.011123898	0.658418101
c12.asymmetricdimethylarginine	0.004732211	0.010681156	0.658433414
pcaec405.lysine	-0.037999606	0.085777118	0.658461562
leucine.c4	0.008815214	0.019899238	0.658469836
c12.pcaec363	-0.00632519	0.014279559	0.658497551
pcaec445.c8	-0.013057952	0.029479624	0.658501683
pcaac383.pcaec386	0.003904309	0.008814435	0.658504247
pcaac421.h1	0.001130217	0.002551613	0.658506215
c51.pcaac366	0.005680213	0.012826128	0.658563572
lysopcac170.pcaac426	-0.026479393	0.059791924	0.658566168

c8.c162	-0.03217206	0.07265874	0.658620974
c8.pcaac404	0.00169171	0.003821344	0.658680697
c8.smc241	-0.004946663	0.011173962	0.658684125
kynurenine.pcaec402	-0.018142864	0.040999492	0.658814891
pcaac362.smc202	-0.01113041	0.0251533	0.658823124
pcaec384.lysine	-0.021536266	0.04867285	0.658847055
leucine.smc181	0.00515852	0.011658551	0.658849302
smc161.tryptophan	0.000903193	0.002041312	0.65885612
kynurenine.pcaac344	-0.012195066	0.027572312	0.658973986
pcaac384.transhydroxyproline	-0.014150248	0.031994806	0.658993384
ornithine.pcaec362	-0.002867097	0.006483317	0.659022386
glutamicacid.pcaac404	-0.004125335	0.009332369	0.65915303
c162.taurine	-0.021527749	0.048701091	0.659158463
c5.pcaac342	-0.003415897	0.007728971	0.659214906
pcaec381.taurine	0.025378295	0.057425652	0.659234441
pcaec404.smc181	0.002017126	0.004565568	0.659321379
smc261.ornithine	-0.010938364	0.02475975	0.659344908
pcaec341.taurine	0.008713832	0.019725023	0.659355504
c51dc.pcaac320	-0.016094224	0.036431845	0.659357567
pcaec401.lysopcac170	0.003606884	0.008164888	0.659362643
c102.c182	-0.03605285	0.081619097	0.659387715
pcaac364.pcaac425	0.033584865	0.076042181	0.659430866
c4.pcaec342	-0.01815919	0.041122244	0.659481807
smohc161.c12	0.025586965	0.057969241	0.659627501
pcaec443.pcaec404	0.016062964	0.036401369	0.659710553
methioninesulfoxide.alphaaminoadip	-0.000358389	0.000812207	0.659725774
pcaec383.serine	0.034382128	0.077941942	0.659817974
pcaac422.pcaec422	0.010250173	0.023237843	0.659837166
c18.lysopcac203	-0.005792248	0.013133051	0.65987665
pcaec341.acetylornithoine	0.005453951	0.012366742	0.659895392
pcaac386.lysopcac160	0.007704436	0.017472884	0.659953856
c51dc.pcaac364	-0.018763604	0.042562617	0.660018612
lysopcac170.smohc222	0.004798147	0.01088442	0.660033058
pcaec380.lysopcac203	-0.012562422	0.028498674	0.660047335
pcaac342.c51	-0.037587957	0.085295588	0.660140125
pcaec404.lysopcac160	-0.029782576	0.067587025	0.660157147
pcaac365.pcaac383	0.003751752	0.00851585	0.660225126
c12.pcaac383	0.001775272	0.004030564	0.66030359
pcaac362.pcaac363	-0.007433576	0.016878134	0.660322411
pcaac361.alanine	0.028117723	0.063863269	0.660428155
smc202.c5	-0.002034749	0.004621527	0.660430841
smc181.smc241	0.011412075	0.025927962	0.660525386
lysopcac140.c4	-0.007879252	0.017901917	0.660533257
glutamine.smohc141	0.013444023	0.030555736	0.660642307
pcaec340.pcaac342	0.002175559	0.004945223	0.660680048

lysopcac170.pcaac424	-0.00147651	0.003356366	0.660693161
pcaec363.pcaac364	0.016088911	0.036573988	0.660702455
smohc222.pcaec386	0.006185045	0.014060481	0.660711003
glutamine.pcaac323	-0.022045049	0.05011906	0.660736245
smc160.pcaec446	-0.008408005	0.019117636	0.660772393
pcaec422.c3	-0.008471008	0.019264202	0.660827033
pcaec421.lysopcac204	-0.025508662	0.058015675	0.660857572
smohc161.pcaec385	-0.022727288	0.051692028	0.66087095
pcaec360.smc241	-0.00408802	0.009298509	0.660888713
pcaec380.c3	0.011165806	0.02540498	0.660982745
pcaac323.tryptophan	0.002404202	0.005471013	0.66103245
c8.pcaac361	0.018413356	0.04190847	0.661085158
lysopcac204.c16	0.017463869	0.039754047	0.661137827
pcaec406.c182	0.009776556	0.022257521	0.661174369
methioninesulfoxide.smohc141	-0.01062875	0.024201471	0.66122459
lysopcac281.pcaac402	-0.028406221	0.06468375	0.66124077
c141.c51dc	0.050227237	0.114385035	0.661275987
serine.pcaac425	-0.025083658	0.057126169	0.661286442
pcaec382.pcaac322	0.022815197	0.051962305	0.661301023
glycine.pcaac384	-0.024186468	0.055100054	0.661385334
pcaec383.smc202	0.029991724	0.068335971	0.661435279
putrescine.pcaac342	-0.002016075	0.004593758	0.661445166
smc241.glutamine	-0.005871706	0.013380234	0.661472864
pcaac343.pcaec301	-0.035496641	0.080892288	0.661487813
pcaac421.smc260	-0.012543606	0.028586232	0.661498525
lysopcac203.pcaac424	-0.003620943	0.008252368	0.661514831
c4.c12	-0.01281564	0.029209647	0.661536129
c16.lysopcac280	-0.007851786	0.017896225	0.661541216
pcaec341.pcaec380	-0.012984236	0.029594448	0.661541833
pcaec403.c51dc	-0.048681679	0.110960087	0.661547247
kynurenine.pcaec365	0.008298951	0.0189191	0.661602777
lysopcac203.pcaec380	0.019062131	0.04346432	0.661664221
c16.kynurenine	-0.006691779	0.015259898	0.661699685
glutamine.pcaac322	0.012737225	0.029048929	0.661732747
pcaec424.c101	0.008656143	0.019743355	0.661762844
prolinr.pcaec384	0.004182672	0.009540229	0.661768968
acetylornithoine.pcaec401	0.007813057	0.017823969	0.661826259
pcaac281.pcaec402	-0.018407724	0.041999162	0.661867925
pcaac240.smohc141	0.009792896	0.022343631	0.661869451
pcaac366.smohc141	-0.008586435	0.019593205	0.661905909
leucine.pcaac363	-0.007349881	0.016778379	0.66203513
pcaac240.pcaac320	-0.039129716	0.089329002	0.662046839
asparticacid.pcaec424	0.007891479	0.018016495	0.662065879
lysine.pcaac425	-0.035552951	0.081183317	0.662123668
putrescine.pcaac383	0.00116589	0.002662916	0.662202893

pcaac322.pcaac360	-0.013856394	0.031657762	0.662297802
serine.alphaaminoadipicacid	-0.000344854	0.00078799	0.662337868
c141.pcaac320	-0.034341784	0.078470786	0.662338011
histidine.smc161	0.032986272	0.07538456	0.662384664
pcaec321.c4	-0.003989422	0.00911765	0.662401943
c141oh.pcaec363	-0.004927855	0.011265516	0.662489602
pcaac344.pcaac323	-0.027680159	0.06328495	0.662517777
c16.smc240	-0.008437105	0.019289899	0.662521142
c51.c4	0.00402824	0.009212443	0.66261073
pcaac344.smc202	0.014670162	0.033551146	0.662620175
c51.acetylornithoine	-0.00828657	0.018955444	0.662683319
c181.threonine	0.010074752	0.023050614	0.662748118
pcaec423.alphaaminoadipicacid	-0.001306184	0.002989949	0.662901868
h1.c12	0.006425317	0.014708969	0.662922458
pcaec321.pcaac342	0.001951905	0.004469724	0.663020194
pcaec403.pcaac384	0.016752751	0.038363543	0.663027884
pcaac420.pcaac342	0.001502323	0.003440827	0.663076622
pcaec322.pcaac406	0.013659952	0.031287707	0.663094803
c2.alphaaminoadipicacid	0.000551918	0.001264329	0.663139166
asparticacid.pcaac380	0.022721006	0.052049856	0.663143842
smc180.pcaec423	0.006785874	0.015551712	0.663275046
pcaac240.pcaec340	-0.018941355	0.043435128	0.663462337
lysopcac203.pcaac366	-0.004245576	0.009737171	0.663510409
phenylalanine.smc261	0.00778197	0.017852842	0.663598765
pcaac360.smc240	-0.025091164	0.057568213	0.663630813
smohc141.smc180	0.040856514	0.09375041	0.663667074
pcaac404.c4	-0.005778852	0.013262184	0.663711809
c10.pcaec402	0.029458713	0.067622186	0.66378586
pcaac320.serine	-0.009792085	0.02247966	0.663814275
pcaac364.pcaec365	-0.010807449	0.024813015	0.663844609
c3.pcaac380	-0.025769686	0.059166787	0.663853702
pcaec402.smc181	0.00197244	0.004528886	0.663867238
c8.pcaec420	0.007559576	0.017358234	0.663882206
lysopcac181.smc202	-0.016863177	0.038722534	0.663893998
pcaec405.tryptophan	-0.001598218	0.00367018	0.663913583
c182.smc180	-0.006348241	0.014579737	0.663946185
pcaec402.valine	0.011265992	0.025875682	0.663965113
lysine.pcaec403	-0.009978878	0.022921974	0.663999897
smohc141.pcaac366	0.003305316	0.007592916	0.664018315
pcaec405.smc181	0.003353091	0.007706987	0.664195046
smohc241.c51	-0.014410402	0.033125349	0.664227767
c3.pcaac364	-0.008999461	0.020689767	0.664267458
pcaac381.pcaac383	0.002038573	0.004687391	0.664315189
c8.pcaec446	-0.025026765	0.057547858	0.664329358
pcaec401.c3	0.011638619	0.026762626	0.664331403

asparagine.pcaac341	-0.002279671	0.005242077	0.664334279
pcaac402.phenylalanine	-0.031524901	0.072503684	0.664388735
pcaac383.valine	0.024051319	0.055327971	0.664460865
pcaec362.smohc222	0.003360164	0.007732174	0.664558943
pcaac402.smc241	0.015903235	0.036597583	0.664577607
pcaac281.lysine	0.019084909	0.043919563	0.66457833
ornithine.c5	-0.004487427	0.010327872	0.664611288
smc261.lysopcac180	0.022847799	0.05259661	0.664683727
pcaac320.pcaac361	0.024710009	0.056903571	0.66479472
lysopcac204.pcaec421	-0.003043087	0.0070079	0.664799677
pcaec424.kynurenine	0.001773081	0.004083243	0.664801943
smohc141.lysopcac170	0.005088448	0.011718742	0.66481571
isoleucine.lysopcac182	-0.01127839	0.025975017	0.664825201
pcaac344.acetylornithoine	0.003899954	0.008983526	0.664882118
pcaec405.pcaac321	0.006315091	0.014547324	0.664893866
c142.pcaec300	0.012301149	0.028340528	0.664936393
pcaac401.serine	0.037785833	0.087064849	0.664973732
pcaec424.c162	0.028568482	0.065843904	0.665056645
pcaec406.pcaec386	-0.009394468	0.021659577	0.665164731
c10.pcaec420	0.007643027	0.017621903	0.665171721
smc260.smohc161	-0.004912252	0.011328103	0.665236221
c3.arginine	-0.017168838	0.039602217	0.665310007
c181.pcaac321	0.012408959	0.028629051	0.665377287
pcaac402.pcaac320	-0.039582644	0.091328241	0.665398025
pcaec423.pcaec406	0.002501488	0.005772228	0.665430383
c2.pcaec401	-0.01641033	0.037883387	0.665565171
pcaac385.pcaac405	0.025319021	0.058453374	0.665587705
pcaec360.c182	0.016305387	0.037644131	0.665590224
pcaec383.alphaaminoadipicacid	-0.000728352	0.001681606	0.665602326
c51dc.pcaac380	0.020251246	0.046760367	0.665633585
lysopcac161.pcaec361	0.002440259	0.005636013	0.665712972
pcaec381.serotonin	-0.03820146	0.088232889	0.665723466
pcaec404.pcaec423	0.007477123	0.017271133	0.665749304
c141.c182	0.016612229	0.038378952	0.665806307
pcaac364.pcaac281	-0.000654001	0.001511216	0.665866357
asparticacid.lysopcac170	0.010421001	0.024081675	0.665887223
pcaac421.lysopcac281	0.006515538	0.015060964	0.665977672
c10.pcaec446	-0.016390128	0.037888337	0.665992616
alphaaminoadipicacid.ornithine	0.012427232	0.028736818	0.666094449
serotonin.pcaac383	-0.001585305	0.003667175	0.666205991
c102.valine	-0.02098813	0.048551437	0.666212869
pcaac384.smc261	0.016028635	0.03708189	0.666239556
pcaac381.pcaac406	0.010560771	0.024433024	0.666251292
c51.c14	-0.014840489	0.034342915	0.666328749
pcaec362.lysopcac204	-0.010324239	0.023892187	0.666335181

pcaec425.smc240	0.011077477	0.02563653	0.666349962
pcaec443.pcaac365	0.02327725	0.053876316	0.666384478
methioninesulfoxide.pcaac424	0.007803535	0.018063014	0.666407983
pcaec445.asymmetricdimethylarginir	0.007251805	0.016787422	0.666436151
pcaec403.c162	0.033792908	0.078230301	0.666444491
pcaec444.c142	0.01496886	0.034655149	0.666465853
pcaac363.ornithine	0.015497315	0.035880555	0.666482896
lysine.creatinine	-0.004919764	0.011394913	0.666601212
pcaec406.c12	0.015934978	0.036910583	0.666624729
pcaec300.c102	-0.017764488	0.041151021	0.666645273
alanine.smc181	0.004287301	0.009931877	0.666659281
smohc141.c3	-0.007461918	0.017290114	0.666731367
pcaac322.glutamicacid	-0.018032862	0.041787562	0.666756572
pcaec360.pcaec362	0.010940751	0.025353048	0.666757069
pcaac322.c182	0.017492649	0.040544104	0.666821247
pcaac364.pcaac406	-0.012772147	0.029613854	0.666935709
pcaac402.ornithine	0.019982843	0.04636839	0.667175986
lysopcac140.alphaaminoadipicacid	-0.000956796	0.002220334	0.667200568
pcaec322.h1	0.000534403	0.001240402	0.667268776
smc161.h1	0.000431575	0.001001773	0.667283123
pcaec422.histidine	0.03140578	0.072902353	0.667296331
pcaac402.c102	-0.015131775	0.035125929	0.667300642
c12.smc161	-0.013972963	0.032442279	0.66736171
pcaac323.pcaac420	0.026521623	0.06157957	0.667371583
smc161.pcaec422	0.013456382	0.031249637	0.66742922
c141oh.lysopcac203	-0.005011396	0.011638917	0.667455988
glutamine.c51dc	-0.042131737	0.097863823	0.667498365
asymmetricdimethylarginine.c51	0.027941488	0.064903559	0.667502805
pcaac364.lysine	-0.025378838	0.058958925	0.667545116
pcaec322.methioninesulfoxide	0.005697888	0.013237508	0.667555564
pcaec340.pcaac405	0.020013279	0.046504347	0.667615204
pcaac420.histidine	0.023417465	0.054417015	0.667629253
serotonin.threonine	0.007706397	0.017908962	0.66764665
smc260.pcaac381	0.017459456	0.040574439	0.667648646
pcaac386.pcaec425	-0.001491116	0.003465491	0.667671199
pcaac320.pcaac403	0.009130234	0.021224935	0.667751192
c141.pcaec384	-0.015399146	0.03580356	0.667797982
smc180.c142	-0.026782751	0.06230079	0.667947992
smc240.alphaaminoadipicacid	-0.001473606	0.003428701	0.668026773
pcaac281.pcaac366	0.003056121	0.007111025	0.668036508
pcaac300.pcaac380	-0.016512482	0.038425202	0.668066805
isoleucine.pcaec446	-0.014168878	0.032988096	0.668223318
isoleucine.threonine	0.008730417	0.020326427	0.668226393
pcaac425.pcaec403	-0.007747395	0.0180424	0.668307144
pcaac422.phenylalanine	0.010192776	0.023737681	0.668312338

pcaec443.pcaac323	-0.022959767	0.053473392	0.668329884
lysopcac160.lysopcac170	0.008362307	0.019479525	0.668388719
c162.pcaec343	0.020622905	0.048040234	0.668390881
glutamine.h1	-0.000588898	0.001371841	0.668396821
c5.pcaec420	-0.010631857	0.024773676	0.668481312
pcaac281.c14	-0.01085799	0.025303703	0.668519575
phenylalanine.pcaec425	0.000680106	0.001585037	0.668539722
pcaac401.asparticacid	0.020471579	0.047713614	0.668559963
smohc141.smohc241	-0.044961314	0.104846421	0.668720322
smohc221.isoleucine	0.007718274	0.018000099	0.668749074
pcaec405.pcaec386	-0.00310146	0.00723562	0.66886005
smohc141.serotonin	0.031748982	0.074073747	0.668877931
pcaac404.pcaec365	0.00925121	0.021588635	0.668943947
pcaac386.pcaac366	0.015264106	0.035621109	0.668950704
asparagine.smc240	0.034114349	0.07961477	0.668965371
methioninesulfoxide.smc181	0.001467606	0.003425046	0.668965592
glutamine.serotonin	0.019874553	0.046386578	0.668992885
pcaec360.c51dc	-0.049157254	0.114732631	0.668996022
pcaac364.kynurenine	-0.002853778	0.006661132	0.669016492
lysopcac182.acetylornithoine	-0.01249099	0.029159833	0.669059662
lysopcac140.pcaec444	-0.031042635	0.072468082	0.669059669
pcaec423.smohc241	-0.026152883	0.061058148	0.669085361
lysopcac182.pcaec401	-0.018530733	0.04326472	0.669097707
smohc161.pcaec406	0.00278611	0.006505417	0.669123217
alanine.kynurenine	-0.002909649	0.006793965	0.669127424
pcaec363.smohc141	0.004043405	0.009443205	0.669191462
pcaec361.smc260	-0.011338394	0.026481146	0.669200764
pcaac421.smc160	-0.017800738	0.041574306	0.669202041
pcaac383.tyrosine	0.024426301	0.057053364	0.669228215
pcaac404.putrescine	0.019331642	0.045157727	0.669256682
lysopcac281.c18	0.012391435	0.028951152	0.669314613
c141oh.pcaac366	0.002683915	0.006271783	0.669370466
serine.pcaec365	0.011050041	0.025830896	0.66948015
lysopcac160.pcaec360	-0.001874093	0.004381241	0.669501919
alanine.pcaac343	-0.007089778	0.016577332	0.669556276
smc202.pcaac366	0.002825852	0.006608008	0.669584301
pcaac300.c102	0.015519291	0.036291052	0.669588954
pcaec421.lysopcac160	0.021665921	0.050664884	0.669590548
pcaac342.smc160	0.026842721	0.062784868	0.669661031
pcaec385.pcaac422	-0.02291905	0.053612909	0.669692722
pcaec444.serotonin	0.030207032	0.070665789	0.669713025
pcaec362.pcaac320	-0.035713174	0.083547718	0.66971659
glutamine.threonine	0.015148056	0.035439635	0.669735326
acetylornithoine.pcaac406	0.034454039	0.080634195	0.669840245
pcaac343.acetylornithoine	0.003703927	0.008669308	0.669870898

pcaac300.pcaec343	0.028938599	0.067737424	0.669891752
pcaec322.c162	-0.027338811	0.063995501	0.66990508
smohc141.pcaec406	0.001522721	0.003564761	0.669933902
glutamicacid.methionine	-0.01068965	0.025028742	0.669980628
pcaec380.smohc141	-0.007826884	0.018328647	0.670027756
pcaac404.pcaec341	0.023568654	0.055192451	0.670030172
lysine.lysopcac180	-0.020422405	0.047825341	0.670034669
spermidine.c14	0.067205943	0.157408255	0.670083704
prolinr.asparticacid	0.011842001	0.027740215	0.670130052
putrescine.tyrosine	0.015802223	0.037021272	0.670164655
pcaac360.pcaec341	-0.021210376	0.049697574	0.670202971
pcaec340.pcaec361	-0.004825774	0.011308957	0.670252051
c3.pcaac361	-0.027987923	0.065593042	0.670274575
pcaac385.pcaac383	-0.001362916	0.003194878	0.670344715
pcaac240.alphaaminoadipicacid	-0.001063483	0.002493468	0.670407581
alanine.c141oh	-0.040433912	0.094802826	0.67040908
smohc161.pcaac321	-0.007730242	0.018124924	0.670414493
c101.asparticacid	-0.012018445	0.028179928	0.670420551
smohc241.c142	0.016156101	0.03789551	0.670534443
c181.pcaac342	0.002114179	0.004959285	0.670552997
pcaec361.pcaac342	0.002817061	0.006610753	0.670679596
pcaac323.lysopcac160	-0.004841857	0.011362635	0.670688421
pcaec364.pcaac421	-0.016905521	0.039680272	0.67074478
citruline.taurine	-0.011922238	0.027985458	0.670765147
pcaec300.c3	-0.008530919	0.020025977	0.670781735
c3.pcaec362	-0.001942261	0.004559519	0.670791444
lysopcac204.smc241	0.018237194	0.042816217	0.670819058
lysopcac181.taurine	-0.015882549	0.037288898	0.670825524
pcaec300.pcaac363	0.003328986	0.007817382	0.670889753
putrescine.creatinine	0.005854965	0.01375031	0.670917382
pcaac380.pcaac424	-0.003208197	0.007534748	0.67093126
pcaac363.pcaec380	0.018269573	0.042917223	0.670999347
pcaac384.pcaac323	-0.027592442	0.064834636	0.671080391
pcaec322.lysine	-0.028692194	0.067419377	0.671083272
pcaec443.pcaac406	0.01855333	0.043598106	0.671100915
pcaac322.c51	-0.026236201	0.061658484	0.671133792
pcaac240.c141	-0.027386977	0.064366249	0.671149614
pcaec380.pcaec361	-0.010746165	0.025256202	0.67114993
citruline.serine	-0.019280148	0.045314164	0.67115638
pcaec443.lysopcac182	-0.023934323	0.056253408	0.671159282
pcaec386.pcaac362	-0.009598546	0.022561697	0.671186785
alanine.pcaac422	-0.004581459	0.01077003	0.671220126
lysopcac204.alphaaminoadipicacid	0.001423537	0.00334648	0.671224737
pcaec341.pcaec425	-0.00142295	0.003345144	0.671228692
pcaac401.smc181	0.00177143	0.004164705	0.671253763

pcaec443.c181	-0.009600765	0.022575103	0.671299026
pcaac342.lysopcac204	-0.008243024	0.019383215	0.671309952
pcaec406.asparagine	-0.005442737	0.012799315	0.671331376
pcaac342.pcaec406	0.001613201	0.003795113	0.671450155
pcaec365.serine	0.038944245	0.091656865	0.671582172
creatinine.pcaac342	-0.001551205	0.003651039	0.671600459
smohc221.smohc141	0.02120991	0.04992847	0.671644581
pcaac420.pcaac321	-0.014774034	0.034778545	0.671646629
pcaec401.pcaac361	-0.025610138	0.060287697	0.671649808
c51.pcaec405	-0.020938797	0.049299872	0.671704789
pcaec343.c3	-0.012071778	0.028423369	0.671712081
smc202.serotonin	0.032111268	0.075621748	0.671772481
pcaec403.c12	0.033488518	0.078876217	0.671815788
arginine.c18	-0.025665932	0.060453978	0.671828367
c18.pcaec386	-0.002658421	0.006261935	0.671840429
lysopcac160.pcaec386	0.002645325	0.006231245	0.671848292
pcaec403.threonine	-0.010112933	0.023825317	0.671894999
pcaec423.pcaec425	-0.001350415	0.003181732	0.671919629
pcaac406.pcaec446	-0.0247596	0.058347981	0.671980759
serine.pcaac342	0.002312352	0.005450009	0.672024194
pcaec363.pcaec380	0.008463696	0.019952319	0.672088145
asymmetricdimethylarginine.pcaac36	0.003500703	0.008253511	0.672123808
pcaac383.pcaac300	0.024835615	0.058560004	0.672154127
pcaac363.pcaec405	-0.024359818	0.05743838	0.672155524
pcaac281.h1	0.000515849	0.00121651	0.672201534
smc240.smc161	0.046290995	0.109170402	0.672212526
pcaac422.pcaac380	0.025253654	0.059571559	0.672288062
pcaec380.smc240	0.015524001	0.036631158	0.672381956
c182.smc161	-0.01942389	0.045839574	0.672422698
c0.pcaac322	-0.018795987	0.044359367	0.672433946
pcaac366.c102	0.014203	0.033520917	0.672445021
pcaec446.pcaec340	-0.010287547	0.024281872	0.672469532
asymmetricdimethylarginine.tryptopl	-0.003851682	0.009091254	0.6724717
asymmetricdimethylarginine.pcaec44	-0.037758992	0.089138295	0.672521768
c101.pcaec422	0.010127864	0.023910677	0.672543131
pcaac406.pcaec425	-0.001730545	0.004085676	0.672548142
pcaac386.smohc141	-0.006872695	0.016234065	0.672703624
pcaec360.pcaac321	0.013962937	0.032988914	0.672768191
c0.pcaec423	0.012671217	0.029938046	0.672778031
pcaec422.glutamicacid	-0.020854436	0.049281596	0.672835615
pcaac322.smc161	-0.025111913	0.059349915	0.672873899
pcaec406.pcaac300	-0.020444889	0.04832191	0.672887392
pcaec421.acetylornithoine	-0.014576643	0.034452391	0.672889169
c2.pcaac320	-0.012876219	0.030435795	0.67291359
putrescine.arginine	0.006676853	0.015782274	0.672914652

prolinr.pcaac342	-0.002394079	0.005659661	0.672952994
pcaac406.smc240	0.009636906	0.022782257	0.672958148
pcaec385.serine	-0.028063614	0.066347502	0.672973636
pcaac322.lysopcac280	0.002625017	0.006207173	0.673030907
asparagine.pcaac342	-0.001699892	0.004020244	0.673079953
pcaac386.pcaac322	0.021846501	0.051677861	0.673144908
pcaac341.c18	0.014275035	0.033770942	0.673175622
pcaac240.pcaac363	-0.005180839	0.012256983	0.673187989
phenylalanine.histidine	-0.030232539	0.071525932	0.673191852
pcaec446.pcaac342	0.000947705	0.002242205	0.673201277
pcaac363.pcaac362	0.007270912	0.017206846	0.67327932
glycine.lysopcac180	-0.018363951	0.043459404	0.673283054
pcaec445.pcaec365	0.007047484	0.016686725	0.673438373
c5.pcaec384	0.019188887	0.045453626	0.67356704
pcaec364.pcaec384	0.044769874	0.106072351	0.673636095
pcaac421.smohc141	-0.010798712	0.025585606	0.673641334
creatinine.pcaec301	-0.022400942	0.053075929	0.673646709
smc161.c102	0.008600298	0.020378843	0.673671269
smc202.serine	-0.016585541	0.039304382	0.673703314
c4.lysine	0.028922296	0.06855508	0.673770909
leucine.pcaec364	-0.005288876	0.012536557	0.673776587
c14.smc241	-0.028040112	0.06646763	0.673787583
pcaec341.pcaac342	0.00088079	0.002088399	0.673866077
pcaec445.pcaac342	0.000846797	0.002007893	0.673879958
pcaec420.c51	-0.038300057	0.090829131	0.673925699
smohc221.phenylalanine	-0.018768256	0.044522203	0.674015489
pcaec406.pcaec380	0.019940235	0.047311457	0.674074377
c182.pcaac384	0.016043055	0.038066688	0.674089903
pcaac403.pcaac363	-0.006178835	0.014663577	0.674143118
pcaac323.phenylalanine	-0.010910432	0.025902454	0.674260146
pcaec404.lysopcac280	0.00296296	0.007037688	0.674405154
c162.pcaec403	0.009127638	0.021685626	0.67448234
pcaec383.c4	-0.008616262	0.020471104	0.674488541
pcaec404.lysopcac182	-0.014608248	0.034708111	0.674495946
pcaec445.c142	0.009803629	0.02330318	0.674633936
pcaac300.lysopcac160	-0.006155351	0.014635867	0.674730858
serine.c4	0.008301305	0.01974851	0.674887669
pcaac381.alphaaminoadipicacid	-0.000622107	0.001480201	0.674935389
pcaac380.smc241	0.007427839	0.017679427	0.675041219
c16.alanine	0.009870716	0.023502558	0.675154537
serine.lysopcac140	0.000560421	0.001334427	0.675164615
pcaec360.pcaec322	0.020684643	0.049264336	0.675237773
serotonin.pcaec384	0.007163411	0.017062193	0.675259169
smohc222.h1	0.000489692	0.001166697	0.675343893
pcaec361.glycine	0.008187543	0.019510391	0.675398206

pcaec425.c18	-0.012671137	0.030194951	0.675402695
smc240.pcaec384	0.00842451	0.020076224	0.675415831
pcaac366.pcaac381	0.017603048	0.041956946	0.675471195
asymmetricdimethylarginine.pcaac40	-0.001323338	0.003154996	0.675549993
pcaec423.histidine	0.030543352	0.072819309	0.67555136
pcaec383.pcaac240	0.006552178	0.015624108	0.675607453
pcaec386.valine	-0.023231132	0.055406969	0.675666912
c141oh.pcaec385	-0.014530399	0.034657159	0.675681965
smc180.pcaac406	-0.012419642	0.029624586	0.67570153
smohc161.pcaac300	0.018101329	0.043190148	0.675793726
smc261.acetylornithoine	-0.006387097	0.015240832	0.675815475
pcaec386.smc161	-0.029267941	0.069839803	0.675819465
c102.phenylalanine	0.016077075	0.038366823	0.675846181
smc180.c2	0.021324272	0.050891151	0.675859733
pcaec380.alphaaminoadipicacid	-0.001120548	0.002674293	0.675866931
pcaac321.asymmetricdimethylarginir	-0.011996234	0.02863402	0.675908487
pcaac403.pcaec422	0.026036899	0.062165624	0.675995445
lysopcac181.c141	-0.008121433	0.019392338	0.676021079
lysopcac181.pcaec446	-0.011561648	0.027607574	0.676028812
lysopcac160.threonine	0.006949221	0.01659383	0.676030221
pcaac405.lysopcac203	0.007972915	0.019047034	0.676170629
methionine.asparticacid	0.015307619	0.036577036	0.676234335
pcaac385.smc202	0.010896403	0.026038284	0.676254236
pcaec384.pcaac240	0.006031324	0.014414061	0.676285493
c4.pcaec421	0.000301677	0.000721001	0.676299109
pcaec423.pcaac344	-0.020864776	0.049868104	0.676310397
pcaac420.c3	-0.009607856	0.022966227	0.676348342
serotonin.pcaec382	0.004084353	0.009763495	0.676361592
c102.alphaaminoadipicacid	0.001501791	0.003590858	0.676436485
kynurenine.c102	0.018640912	0.044577751	0.676480294
pcaac405.pcaac344	0.020193117	0.048295227	0.676515296
pcaec363.pcaec301	-0.016377819	0.03917245	0.676532011
pcaec341.pcaec363	-0.017805562	0.042590869	0.676557389
c181.serine	0.009055978	0.02166597	0.676614929
pcaec421.alphaaminoadipicacid	-0.002060767	0.004933441	0.676810231
pcaac361.pcaac381	-0.011675931	0.027952224	0.676812891
phenylalanine.c8	0.013951992	0.03340484	0.676846808
pcaac422.lysopcac161	0.023097365	0.055303881	0.67686083
pcaec420.tryptophan	0.002853339	0.006832704	0.67689311
glycine.pcaac383	-0.001438394	0.003445251	0.676965878
pcaac380.acetylornithoine	-0.006528935	0.015639769	0.676997499
lysopcac170.pcaac323	0.024722896	0.0592248	0.677008883
pcaac420.pcaac361	-0.022208914	0.053203793	0.677016611
c181.glutamine	-0.007581195	0.01816575	0.677086986
pcaec343.methioninesulfoxide	0.004963377	0.01189348	0.677098201

pcaac366.methioninesulfoxide	0.008952605	0.021453924	0.677116194
pcaec401.pcaac426	-0.029679022	0.071123539	0.677120722
pcaac406.pcaec343	-0.027900005	0.066871963	0.677174014
smc260.pcaac343	-0.011688927	0.028036471	0.677390464
pcaac422.c162	0.022484975	0.053941389	0.677447216
c5.pcaac365	0.00843879	0.020247021	0.677483106
pcaac343.tyrosine	0.021063852	0.050547846	0.677541856
c101.c141oh	-0.035875469	0.086129618	0.677674932
pcaec443.phenylalanine	-0.024117023	0.057905766	0.677705149
smohc241.pcaac281	-0.000725009	0.001741178	0.677776229
smc202.pcaec405	-0.008722526	0.020951621	0.677829179
isoleucine.pcaac365	-0.011878458	0.028532351	0.677830671
pcaec421.putrescine	-0.018104245	0.043486885	0.677830963
pcaec385.smc241	-0.010095806	0.024251794	0.677848454
pcaac360.pcaac240	-0.006022596	0.01447354	0.677980063
lysopcac170.c182	0.010011425	0.024062255	0.678014607
pcaac386.pcaec401	-0.011173472	0.026855451	0.678017329
smc261.pcaec421	0.000878633	0.002112805	0.678162451
pcaec384.c8	-0.016763411	0.040311582	0.678173344
smc261.pcaac320	0.012995424	0.031250777	0.678175366
glutamicacid.pcaec406	-0.00351566	0.008454391	0.678178892
pcaec380.h1	0.00066881	0.001608475	0.678204096
phenylalanine.methioninesulfoxide	0.00739235	0.017778546	0.678205694
pcaec364.pcaac323	-0.029450853	0.070837508	0.678241797
pcaac365.pcaec406	0.00408642	0.009829707	0.678264282
pcaec342.pcaec341	0.046685819	0.112305104	0.678276125
pcaac421.pcaac344	-0.011852452	0.028516815	0.678330836
alanine.glycine	-0.014987869	0.036067741	0.67839109
smohc161.ornithine	-0.00827502	0.01991942	0.678480916
smc160.methioninesulfoxide	0.003813194	0.00917938	0.678492665
asymmetricdimethylarginine.pcaac3(	0.026459693	0.063695611	0.678492828
pcaec443.pcaac342	0.00489129	0.011775275	0.678508868
pcaec446.pcaac344	-0.018121135	0.043627043	0.678524735
pcaac421.pcaec365	-0.012734567	0.030659235	0.678529445
c2.pcaec425	-0.001429579	0.003442187	0.678563822
pcaec361.alphaaminoadipicacid	-0.000605201	0.001457315	0.678583262
pcaac343.isoleucine	-0.006997274	0.016850588	0.678605596
smc160.acetylornithoine	-0.008369171	0.020157514	0.678653328
glutamicacid.c141	-0.016117911	0.038821447	0.678659201
leucine.valine	0.028791098	0.069356704	0.678706151
lysopcac182.lysopcac204	-0.006022074	0.014507217	0.678711576
putrescine.serotonin	0.030455913	0.073391955	0.678808477
c16.pcaac384	-0.004676512	0.011271618	0.678869422
pcaec361.pcaac300	-0.026009524	0.062692249	0.678881465
prolinr.pcaec385	-0.016677078	0.040206048	0.678944237

c51dc.c51	-0.0125525	0.030267444	0.678995911
spermidine.methioninesulfoxide	-0.006533045	0.015755348	0.679042522
asparticacid.pcaec402	-0.041311912	0.099670514	0.679167373
pcaec382.c14	0.02838382	0.068494137	0.679230912
pcaac342.lysopcac140	0.000327953	0.000791533	0.679282892
acetylornithoine.pcaec445	0.018657211	0.045059918	0.679482537
c102.c16	-0.009339493	0.022563364	0.679577852
lysopcac170.c18	-0.020931594	0.050576596	0.679624337
pcaec301.creatinine	-0.003745409	0.009050278	0.679635094
c4.c8	0.014727151	0.035588207	0.679652241
smc260.pcaac300	0.02579292	0.062337233	0.679693761
acetylornithoine.pcaac300	0.029213076	0.070604205	0.679698121
pcaec446.lysopcac204	-0.008986372	0.021723966	0.679768736
pcaac361.pcaac384	-0.030974918	0.074881382	0.679774969
c142.pcaec342	0.018293375	0.044231382	0.679825764
pcaec362.smohc141	0.012571817	0.030409021	0.679942445
c12.lysopcac180	0.015970766	0.038632535	0.679958509
pcaec321.serine	-0.004563359	0.011039272	0.679978212
c141.pcaec403	0.009303845	0.022508278	0.6799949
pcaac402.smc181	0.001514501	0.003664384	0.680030712
c14.pcaac384	0.024791731	0.059998655	0.680102609
smohc141.pcaac383	0.001969218	0.00476767	0.680226261
pcaac361.c141oh	0.026781974	0.064861693	0.680318827
pcaac240.lysopcac180	0.023124447	0.056009902	0.680352009
alanine.pcaac404	-0.001972542	0.004777798	0.680357568
smohc161.pcaec386	0.003909494	0.009469574	0.680363455
pcaec403.lysine	0.031735387	0.076869823	0.68036496
pcaec443.c51	-0.026274048	0.063644007	0.680377772
pcaac425.pcaec365	-0.011495453	0.027851185	0.680438258
methioninesulfoxide.c102	-0.019240438	0.046630125	0.680531366
pcaec404.pcaec386	-0.002039116	0.004941943	0.680534258
c10.pcaec342	0.021590908	0.052336713	0.680589636
glutamine.phenylalanine	-0.013354344	0.032379934	0.680671471
pcaec322.c102	0.009921694	0.024058122	0.680687105
smc180.pcaec386	0.002873827	0.006968692	0.680697353
pcaac240.c18	-0.01850251	0.044867135	0.680702236
pcaec340.c4	-0.00377986	0.009168823	0.680799533
pcaec381.pcaec406	0.006308254	0.01530241	0.680808412
arginine.c14	-0.009368703	0.022726933	0.680815826
pcaac421.pcaac281	-0.000974053	0.002363253	0.680861851
pcaec420.lysopcac161	-0.025911076	0.062870908	0.680887255
c16.pcaac342	-0.004982102	0.012089328	0.680904809
pcaac420.pcaec343	0.038617857	0.093710459	0.680911963
pcaec383.pcaac406	-0.023130772	0.056130818	0.680919837
pcaec402.lysopcac140	-0.000344883	0.000836937	0.680926526

pcaac386.pcaec422	0.007790357	0.018905866	0.680939262
glutamine.pcaec425	0.002285931	0.005548618	0.680996448
leucine.pcaac422	0.013824406	0.033556778	0.681004829
pcaac320.lysopcac140	0.000297055	0.000721143	0.681039526
c0.pcaac385	0.009121011	0.022146681	0.681096293
pcaac422.alphaaminoadipicacid	-0.001621309	0.003937242	0.681138253
pcaac342.smc202	-0.010008141	0.024304891	0.681148032
pcaec444.c51dc	-0.042274832	0.102681349	0.681196158
leucine.pcaec444	-0.030461071	0.07400212	0.681258079
pcaec321.smc241	-0.003498572	0.008499854	0.681273078
pcaac342.pcaac426	0.019147859	0.046540897	0.681407435
lysopcac160.pcaec380	0.017734869	0.043112955	0.681452677
c102.methionine	-0.021391444	0.052010637	0.681502725
pcaec382.c101	0.00817174	0.019870837	0.68153705
pcaec404.smohc141	-0.007491034	0.018219642	0.68160385
c101.pcaac321	0.006101684	0.014842334	0.681641511
pcaec444.pcaac342	0.001422302	0.003460011	0.681664456
pcaac384.pcaec382	0.001707146	0.004154109	0.681748645
pcaac384.smc160	0.037667658	0.091671168	0.681788055
smohc161.lysopcac181	0.017392333	0.042330888	0.68181254
pcaac323.pcaec385	-0.016440516	0.04002082	0.681861691
pcaec422.pcaac322	0.025398329	0.061841462	0.681933688
pcaec403.valine	0.010429208	0.025406049	0.682079773
smohc141.lysopcac280	0.002930566	0.00714253	0.682228289
pcaac380.c16	0.016986716	0.041403661	0.682248144
smc241.alphaaminoadipicacid	-0.001262894	0.003078486	0.68227657
smohc221.pcaec386	0.006247788	0.01523147	0.682307739
asparagine.c51	-0.02670132	0.065097443	0.682318564
pcaec380.pcaac240	-0.006599	0.016091704	0.682382627
pcaec382.c51dc	-0.055176814	0.134557774	0.682402164
c18.c8	0.018869818	0.046018723	0.682412312
pcaac381.pcaac360	0.019545612	0.047668706	0.682424221
pcaec322.pcaec406	0.002391203	0.005832455	0.68245942
pcaec365.c142	0.012681523	0.030941421	0.682551834
pcaec342.pcaac381	-0.012444684	0.030364166	0.682557823
pcaec446.pcaac364	-0.014851944	0.036240601	0.682581716
alphaaminoadipicacid.smohc141	-0.003577356	0.008732306	0.682688582
smc202.smc240	-0.012097574	0.02953433	0.682731367
pcaec343.pcaec425	-0.001053211	0.002572235	0.682846222
pcaac383.c18	0.011027171	0.026939999	0.682941605
c0.creatinine	0.007933269	0.019383547	0.682974468
citruline.pcaec425	0.004530584	0.011075484	0.683131345
pcaac424.serotonin	-0.03438139	0.084058014	0.683163854
pcaec446.pcaec423	-0.009724464	0.023777632	0.683196404
lysopcac180.pcaac240	-0.005767981	0.014104239	0.683212186

pcaac401.alphaaminoadipicacid	-0.000642253	0.001570836	0.683280481
pcaac320.pcaec401	0.019295922	0.047200066	0.683316453
pcaec342.smohc141	0.002378543	0.005821149	0.683468421
phenylalanine.pcaac240	-0.004469725	0.010946935	0.683684736
smohc241.alphaaminoadipicacid	-0.000832991	0.00204039	0.68372674
lysopcac182.c101	-0.002925508	0.00716669	0.683757425
pcaec420.glycine	0.017304647	0.042393405	0.683770012
lysopcac204.pcaac364	0.015287479	0.037457379	0.683815409
pcaec342.pcaac422	-0.007017459	0.017195296	0.683834856
asparticacid.pcaac404	-0.003996556	0.009794139	0.683869632
lysopcac181.pcaac344	-0.021039239	0.051561026	0.683877357
pcaac341.lysine	0.024964042	0.061182685	0.683892559
c141oh.alphaaminoadipicacid	0.001115015	0.002733608	0.683990195
serotonin.pcaec343	0.032204744	0.07897711	0.68407676
c102.c10	-0.007028421	0.017236859	0.68408979
lysopcac181.pcaac422	-0.014763688	0.036210575	0.684117454
pcaac385.c16	0.011282966	0.027674322	0.684126511
lysopcac160.alphaaminoadipicacid	-0.001069419	0.002623575	0.684189682
pcaac300.pcaac406	-0.01561889	0.038321185	0.684219355
pcaac403.c0	0.000449141	0.001102035	0.684235983
pcaec422.smc181	0.002707508	0.006644345	0.684283796
pcaac384.pcaac322	0.015697283	0.03852196	0.684284751
pcaec380.pcaac320	-0.031153072	0.076458375	0.684312443
glutamicacid.smohc141	-0.006077167	0.014918287	0.684376782
lysopcac203.pcaac323	0.037583789	0.092301035	0.684506096
glutamine.lysopcac203	-0.002344316	0.005758091	0.68454478
pcaec385.lysopcac281	0.004202247	0.010323213	0.684593546
histidine.pcaec380	-0.016364902	0.040202541	0.684598188
lysopcac180.c5	-0.004835505	0.011881391	0.684656797
pcaec360.lysopcac281	-0.00630631	0.015495677	0.68466353
c51.smohc241	-0.013227148	0.032517624	0.684812727
lysopcac182.pcaac386	0.013050226	0.032090561	0.684885946
asparagine.c162	-0.023831626	0.058606056	0.684906221
pcaec380.ornithine	-0.018167274	0.044677366	0.684912372
pcaac383.threonine	0.007184601	0.017670887	0.684952083
c4.pcaec406	-0.003612421	0.008886568	0.685006971
pcaec382.c4	-0.008212629	0.020204008	0.685020458
smohc141.pcaec422	-0.007363816	0.018116892	0.68503788
c8.pcaac380	0.017726843	0.043613652	0.685044963
asymmetricdimethylarginine.lysopca	0.006219029	0.015302125	0.685071082
pcaac405.pcaac381	-0.013795595	0.033948372	0.685104946
c5.taurine	-0.014256792	0.035090562	0.685166659
glycine.c102	0.024106698	0.059353656	0.685263479
pcaec365.h1	-0.000566661	0.001395228	0.685271476
pcaec422.c162	0.021591316	0.053163653	0.685281267

pcaac403.pcaac424	-0.00744982	0.018343551	0.685282579
c12.pcaac421	0.004664162	0.011484602	0.685285797
pcaec361.pcaec406	0.002619844	0.006451861	0.685331859
smc160.lysopcac161	-0.025743099	0.063399774	0.685343766
pcaec363.lysopcac170	-0.002555724	0.006295341	0.68539744
kynurenine.pcaac422	-0.014663576	0.036122004	0.685415763
smohc221.alphaaminoadipicacid	-0.001433947	0.003532776	0.685450687
pcaec404.pcaac364	0.013759488	0.033902955	0.685486556
phenylalanine.pcaac384	-0.028909056	0.071253154	0.685578962
phenylalanine.pcaac366	0.013051904	0.032171667	0.68559918
prolinr.c8	0.010463021	0.025790915	0.685606005
pcaac401.pcaec404	-0.013569185	0.033448964	0.685619174
lysopcac280.pcaec422	-0.008777896	0.021639238	0.685634665
pcaac404.pcaec423	0.004013225	0.009894156	0.685657785
c10.transhydroxyproline	0.011128748	0.027441889	0.685714201
c8.pcaec301	-0.020527727	0.050623328	0.685743039
pcaec423.pcaac402	0.029468033	0.072689695	0.68581962
pcaec364.pcaac363	0.003314905	0.00818099	0.685965453
prolinr.pcaac362	-0.005599106	0.013820092	0.686004741
lysopcac181.c51dc	-0.043946052	0.108500824	0.686087449
pcaec403.lysopcac140	0.000828764	0.002046504	0.686134485
h1.smc181	0.00205903	0.005084489	0.686136371
pcaec341.c4	-0.004523091	0.011171053	0.686186992
pcaec362.pcaec421	0.00110729	0.002734795	0.686190306
smc180.pcaec383	0.019104231	0.047191288	0.686237251
pcaec363.lysine	-0.023029543	0.056889085	0.686245024
pcaac403.lysine	0.029428717	0.072697508	0.686248139
c10.asparticacid	-0.008538333	0.021098085	0.686331339
prolinr.lysopcac204	-0.005194017	0.012843084	0.686533596
pcaec444.acetylornithoine	-0.023674324	0.058558152	0.686631819
pcaac424.pcaec423	0.033514801	0.082909648	0.686671872
pcaec386.methionine	-0.008153972	0.02017563	0.686732937
pcaac402.histidine	-0.034879491	0.08630539	0.686739703
pcaec425.c12	0.01598021	0.039558963	0.686872545
pcaac240.smohc161	-0.017020243	0.04213465	0.686880193
c14.smohc161	0.003290362	0.00814553	0.686881553
pcaac323.h1	0.000433922	0.001074272	0.68689965
pcaac366.phenylalanine	0.012451707	0.030827142	0.686901164
pcaac300.acetylornithoine	0.003704814	0.009177578	0.68707666
smohc241.c4	0.00462506	0.011458189	0.687101895
pcaac405.serotonin	-0.029166507	0.072273927	0.687169126
pcaac240.pcaec341	-0.01244986	0.030854573	0.687208561
c12.pcaac425	-0.017891396	0.044341021	0.687212924
h1.methioninesulfoxide	0.014580142	0.03613466	0.687213428
pcaec444.lysopcac182	0.015217253	0.037715583	0.687228668

pcaac380.pcaaec446	-0.039696956	0.098389337	0.687232938
c181.pcaaec420	0.00569915	0.01412545	0.687233879
pcaac425.pcaac322	-0.025238511	0.062556637	0.687245783
asymmetricdimethylarginine.c3	-0.016139153	0.04000502	0.687262208
pcaaec342.c8	-0.014785934	0.036655634	0.687301878
c10.pcaac426	0.014761029	0.036596995	0.687326978
pcaaec425.tryptophan	-0.001261906	0.003128767	0.687338953
pcaaec386.pcaac300	-0.018299951	0.045372923	0.68733926
c181.smc202	-0.015525316	0.038496925	0.687365687
pcaac401.pcaaec446	0.026653092	0.066094261	0.687386503
pcaaec386.glutamicacid	0.014094506	0.034953079	0.687399754
pcaaec401.arginine	-0.018898098	0.046874018	0.687453225
lysopcac180.threonine	0.00974271	0.024165916	0.687459634
c51.alphaaminoadipicacid	-0.004250602	0.010544901	0.687506344
pcaaec382.putrescine	0.022840817	0.056685323	0.687620039
pcaac422.lysopcac280	0.003755578	0.009320515	0.687622818
creatinine.alphaaminoadipicacid	0.000732194	0.001817254	0.687640857
c16.pcaac344	0.003944784	0.009795644	0.687790796
c142.pcaac322	-0.020290463	0.050409736	0.687935493
citruline.pcaac406	0.023556309	0.058527349	0.687955327
pcaaec420.pcaac281	0.000523321	0.001300273	0.687965443
pcaac385.lysopcac182	-0.004221178	0.010488298	0.687968784
pcaac402.pcaaec421	0.002849966	0.007082175	0.688006531
pcaac383.pcaaec421	0.000500114	0.001243183	0.688100964
smc202.pcaac322	0.012689668	0.031544996	0.688110813
c5.serine	0.011998117	0.029830774	0.688159226
pcaaec443.smc160	-0.010819137	0.02690068	0.688172302
c5.pcaac320	-0.038385934	0.095465552	0.688242948
pcaaec424.lysopcac280	0.002332883	0.005802254	0.688262775
lysopcac203.smc260	0.01260834	0.03135927	0.688265683
isoleucine.tyrosine	-0.02514768	0.062554419	0.688301061
c3.pcaaec405	0.015023732	0.037372888	0.688313813
pcaac426.pcaac362	0.002192636	0.005455739	0.688387328
pcaac425.c18	-0.015054643	0.037460558	0.688398706
pcaac342.pcaac361	0.016548613	0.041181776	0.688425688
smc261.pcaaec322	0.028905459	0.07193249	0.688426872
arginine.pcaaec446	0.034706895	0.086376516	0.688450443
pcaac425.lysopcac281	0.0025066	0.006238458	0.688458768
smohc161.acetylornithoine	-0.007408159	0.018439533	0.688490993
pcaaec341.pcaaec340	-0.018803141	0.046803291	0.68849533
c162.c141oh	0.046532143	0.115837129	0.688528433
phenylalanine.putrescine	-0.008687857	0.021627567	0.688528631
pcaac240.pcaaec363	0.010541392	0.0262437	0.688550416
pcaaec342.pcaac342	0.001385424	0.003449558	0.68858679
pcaaec362.isoleucine	0.008245011	0.020532164	0.688629433

smohc221.c182	-0.010593289	0.026382771	0.688660738
pcaec360.pcaac341	0.00553048	0.013774685	0.688680584
pcaec420.lysine	-0.025145673	0.06263834	0.688720076
pcaec363.asparticacid	0.011305326	0.028174374	0.68885188
asparticacid.pcaac320	0.014582705	0.036342485	0.688855523
pcaac421.pcaac360	-0.014581972	0.036341873	0.688865366
arginine.alphaaminoadipicacid	-0.000486111	0.001211531	0.688870578
c8.smc160	-0.020745561	0.051715039	0.688933633
methioninesulfoxide.pcaac406	0.031912597	0.079562057	0.688969039
c51dc.threonine	-0.021050704	0.05248234	0.688970877
glutamine.arginine	0.010470949	0.026109051	0.689010527
pcaac321.pcaac360	-0.014296214	0.035658222	0.689101156
c12.c10	0.014703208	0.036682887	0.689177608
lysopcac170.pcaac361	0.012478185	0.031136431	0.689222337
pcaac403.pcaec406	0.007541681	0.018820377	0.689251305
pcaac425.pcaec405	-0.021747449	0.054288407	0.689345262
smc181.c181	0.003599599	0.008989229	0.689460174
smohc222.threonine	0.014082345	0.035168846	0.689470164
smc260.pcaac385	-0.006388642	0.015959186	0.689550813
pcaec386.pcaec363	-0.013169402	0.032905726	0.689620707
leucine.smc180	0.003794168	0.009485698	0.689788095
pcaec423.pcaac342	0.001590756	0.003977224	0.68980423
smc241.c16	0.012752862	0.031888127	0.689834544
c51dc.pcaec384	0.009510571	0.023785857	0.689896016
lysopcac281.lysopcac181	0.009327937	0.023331471	0.689925995
asparticacid.valine	0.012878255	0.032213039	0.689938282
pcaec361.serotonin	0.038182795	0.095521907	0.689979271
methioninesulfoxide.lysopcac280	-0.007480402	0.018713896	0.689981988
lysopcac280.taurine	0.030445059	0.076166593	0.689987688
pcaec341.pcaac343	-0.01246196	0.031179414	0.690010612
c18.creatinine	0.003184931	0.00796948	0.6900433
smc260.pcaac383	-0.001617661	0.004048177	0.690071675
pcaac360.lysopcac140	0.000388684	0.000972685	0.690073988
c182.tryptophan	0.003067602	0.007677005	0.690085828
pcaec301.pcaac365	0.015287381	0.038260143	0.690099792
smc161.pcaac342	0.000526127	0.001316784	0.690106608
pcaec383.pcaec425	-0.001801987	0.004510214	0.69012146
c3.c8	-0.011022623	0.027592713	0.690164672
pcaec322.pcaac342	0.000924662	0.002314791	0.690177685
smc240.pcaec405	-0.018075227	0.04525403	0.690207981
pcaac420.pcaac383	0.002411304	0.006039441	0.690323698
c141oh.lysopcac281	0.006458591	0.016177153	0.690336906
c10.lysopcac182	0.03197003	0.080080331	0.690349432
pcaac383.pcaec406	0.002918101	0.007309797	0.690364456
c8.pcaac366	0.003605781	0.009034485	0.690431368

glycine.pcaec444	0.031451652	0.078839133	0.69056259
methioninesulfoxide.lysine	-0.04053071	0.101623637	0.690638308
smc161.pcaec421	-0.000633252	0.00158812	0.690703473
pcaac364.smc180	0.00735897	0.018457883	0.690742472
pcaec360.pcaac383	0.00278412	0.006983504	0.690756413
pcaac426.pcaec402	-0.011409149	0.02862141	0.690791691
smc180.acetylornithoine	-0.006852856	0.017193702	0.690832199
pcaec385.pcaec386	-0.00288276	0.007233807	0.690873125
kynurenine.pcaac366	-0.008472828	0.021262116	0.690886359
pcaac362.alanine	-0.012097931	0.030362632	0.690920234
pcaec301.alanine	-0.0053443	0.01341768	0.691026996
pcaac384.smc202	0.015743187	0.039531051	0.69106687
pcaec444.prolinr	-0.006758003	0.016971538	0.691105373
pcaac240.pcaec362	0.00299848	0.007531052	0.691140234
pcaec421.pcaec403	0.028570282	0.071766403	0.691175373
pcaac343.lysine	-0.024625152	0.061871966	0.6912484
pcaec445.pcaec340	-0.0068481	0.017208542	0.69128817
pcaec362.pcaec365	-0.006112209	0.015361932	0.69133778
lysopcac281.lysopcac140	-0.000437488	0.001099554	0.691339882
pcaec361.pcaec425	-0.000960035	0.002413078	0.691362559
c3.pcaac363	-0.004578739	0.011511071	0.691420113
c14.alanine	0.008713769	0.02190724	0.691428036
pcaac365.smohc141	-0.007206569	0.018120742	0.691472365
pcaec424.serine	-0.005645441	0.01419607	0.691487731
c162.pcaac342	0.000515568	0.001296497	0.691497696
smc181.pcaac342	0.000512651	0.001289177	0.691501659
c10.lysopcac281	-0.005411726	0.013614739	0.691624576
pcaec445.pcaac406	0.010629391	0.026745752	0.691673672
pcaec423.pcaec401	-0.017918973	0.045106668	0.691795635
pcaac422.smc241	0.01823758	0.045910012	0.691804095
ornithine.pcaec406	0.002308718	0.005812847	0.691856451
c10.c102	0.010634566	0.026776519	0.691867425
pcaac343.smohc141	0.017742035	0.044679626	0.691915708
c141oh.pcaac281	-0.000731562	0.001842502	0.69194987
asparagine.lysopcac281	-0.003850062	0.009702623	0.692127737
smc160.smc161	0.052677938	0.132755878	0.692130137
pcaec406.glycine	0.016942112	0.042703293	0.6921764
lysopcac181.pcaec383	-0.036334357	0.091595308	0.692218019
smc180.pcaec384	0.018663385	0.047055126	0.692258862
c141.c16	0.01267021	0.031945005	0.692260617
pcaec381.lysopcac182	-0.017425943	0.043935537	0.692260969
pcaec406.c51dc	-0.04007455	0.101046427	0.692282908
pcaec422.lysopcac161	0.02439846	0.061524613	0.692305862
pcaec402.asparagine	0.003185301	0.00803267	0.692321269
lysopcac181.pcaac383	0.003767006	0.00950173	0.69238639

pcaec402.pcaac380	0.022356473	0.05639191	0.692391216
pcaac424.lysopcac203	0.005071038	0.012792796	0.692428237
h1.transhydroxyproline	-0.007075639	0.017855407	0.692519177
c102.smc260	0.012401351	0.031296145	0.69253112
arginine.pcaec364	-0.002879897	0.00726993	0.692619338
pcaac404.c182	0.009809685	0.024763611	0.692623147
c16.isoleucine	-0.009091492	0.02295245	0.69264664
asymmetricdimethylarginine.pcaac32	-0.011614796	0.029323585	0.692654406
pcaac403.c2	-0.038167961	0.096362878	0.692657978
acetylornithoine.c12	-0.018309337	0.046231016	0.692691541
pcaec361.pcaac383	0.0016851	0.004256652	0.692813421
pcaac323.pcaac365	-0.016052272	0.040551963	0.692835514
pcaac401.pcaac360	-0.02233111	0.056419095	0.692862638
lysopcac140.pcaac323	-0.030991941	0.078302027	0.692868287
pcaac384.pcaac363	0.001989214	0.005027132	0.692945136
pcaac385.asymmetricdimethylarginir	-0.006420308	0.016229004	0.693010295
methioninesulfoxide.pcaac300	-0.023427266	0.059220835	0.693021662
asparticacid.methioninesulfoxide	0.010366084	0.026205411	0.693037294
pcaec364.pcaec360	0.001339762	0.003387626	0.693098694
c5.putrescine	-0.01831523	0.046319467	0.693154558
c102.citruline	-0.016429134	0.041554459	0.693189263
pcaac362.pcaec406	0.001220483	0.003087016	0.693192133
pcaec404.taurine	-0.02552664	0.064574962	0.69323453
h1.c16	0.011560924	0.02925112	0.693287755
pcaac424.lysopcac181	0.02507734	0.063457296	0.693321369
smc240.acetylornithoine	-0.014508696	0.036716362	0.693342237
c181.pcaec340	-0.005047258	0.012774275	0.693375365
h1.c2	0.010792523	0.027319137	0.693417755
pcaac361.serotonin	0.027800143	0.070380262	0.69345783
c142.c16	0.012991996	0.03289278	0.693471799
pcaec322.lysopcac170	0.003771723	0.00954933	0.693477457
pcaac363.pcaac405	0.02609928	0.066082648	0.693494709
pcaec382.valine	0.007597213	0.019236091	0.693497116
c182.smohc141	0.010047464	0.025441022	0.693507546
pcaac360.lysopcac281	0.004849436	0.0122793	0.693510445
pcaac342.smohc241	-0.017398982	0.044058399	0.693525483
smohc141.c142	0.018893407	0.04784835	0.693560172
pcaac323.pcaac422	-0.00803806	0.020356986	0.693563904
pcaac384.pcaec446	-0.013860677	0.03510714	0.693596531
smohc161.lysopcac280	0.003252447	0.008239283	0.693642187
c162.pcaac300	-0.015128922	0.038337901	0.693736627
smohc241.pcaac322	0.012464886	0.031595374	0.693813453
c10.smc241	-0.010387711	0.026330449	0.69381563
kynurenine.pcaac364	0.021134661	0.053572428	0.693820729
pcaec321.asymmetricdimethylarginir	0.009650038	0.024461848	0.693830211

pcaac361.pcaac320	-0.022318369	0.056580171	0.693858017
spermidine.c141	0.043608277	0.110555482	0.693864413
pcaec420.pcaac344	0.018640099	0.047257062	0.693869204
smc160.lysine	0.016648419	0.042212651	0.693903394
smc240.pcaac360	-0.008585071	0.02176862	0.693915021
lysopcac170.alphaaminoadipicacid	-0.000831968	0.002109599	0.693919104
pcaec405.serine	0.025350472	0.064282402	0.693927743
smohc241.lysopcac204	0.007615989	0.019315892	0.69398286
histidine.pcaac424	0.010985598	0.027873894	0.694106842
c162.pcaec363	-0.003162163	0.008024382	0.694142564
pcaac386.c12	0.01595222	0.040483618	0.694163161
smohc161.pcaec443	0.02551672	0.064764858	0.694200828
glycine.c4	-0.003788533	0.009616192	0.694212537
c10.pcaec422	0.010417156	0.026441225	0.694212751
pcaac406.pcaac343	0.012574608	0.031918312	0.694221475
c142.pcaec365	-0.007633679	0.019378021	0.694241573
c0.pcaac343	0.005658131	0.014363381	0.694246985
smc181.h1	0.000362134	0.000919636	0.694356161
glutamine.pcaac362	0.003426744	0.008703912	0.694413474
pcaac406.smc202	0.007984636	0.020282529	0.694436249
c142.pcaec420	0.005647361	0.014351729	0.694564232
c142.c182	-0.035331179	0.089803281	0.694614548
pcaac421.pcaec403	-0.009176881	0.023327123	0.694635909
lysopcac182.pcaec406	0.001161007	0.002951563	0.694670058
pcaac362.c0	-0.000425532	0.001081826	0.694675694
kynurenine.pcaec406	0.00391831	0.009961925	0.694688177
pcaec365.asymmetricdimethylarginir	0.014997243	0.03813214	0.694711762
smohc221.pcaec365	0.010246255	0.026053283	0.694723455
lysopcac182.pcaac342	-0.001698532	0.004320782	0.694851154
pcaec322.pcaec425	-0.001118476	0.002847185	0.695051373
c182.c51	0.018962591	0.048273464	0.695065892
pcaac343.pcaec384	-0.010749942	0.027366484	0.695067299
lysopcac280.c51	-0.040518818	0.103155586	0.695082687
pcaec383.c8	0.01464886	0.037294725	0.695087753
smc202.pcaac342	0.000468227	0.00119211	0.695098496
pcaec360.c162	-0.031182468	0.079406693	0.695156413
pcaac341.acetylornithoine	0.004335682	0.011043496	0.695224699
smc260.c51dc	-0.03492466	0.08895918	0.695231011
pcaac281.smc161	0.038256457	0.097453905	0.695254944
smc160.methionine	0.008813234	0.022451637	0.695267086
c142.smohc141	0.009895609	0.025209229	0.695269938
pcaec381.smc181	0.001641627	0.004182557	0.695303549
glutamine.pcaac240	0.00612633	0.015612073	0.695365311
pcaec386.pcaac344	-0.025200595	0.064220406	0.695366676
smohc221.smc161	0.040374145	0.102908736	0.695424377

smc260.pcaac321	-0.007127011	0.018167825	0.695455428
c141.pcaac405	-0.020663595	0.05268499	0.695512336
pcaac281.smohc241	-0.023605377	0.060204245	0.695602287
isoleucine.smc241	0.006011616	0.015336738	0.695685843
c141oh.pcaec402	-0.01131444	0.028867645	0.695710155
smohc241.prolinr	-0.005860962	0.014956514	0.695765519
pcaac424.lysopcac182	-0.034704041	0.088598696	0.695889062
phenylalanine.smc180	-0.004890835	0.012487188	0.695911896
smc261.pcaec425	0.001905271	0.004866608	0.696036813
pcaec403.pcaac425	0.040371837	0.103126891	0.69605268
lysopcac170.pcaec321	0.013293335	0.033959422	0.696074576
pcaac422.smc240	0.012873242	0.032891376	0.696119598
pcaac380.pcaec384	0.011216344	0.028658379	0.696123719
pcaac364.pcaac342	-0.00185644	0.004744857	0.69621798
smc241.pcaac323	-0.023309095	0.059591251	0.696294296
alphaaminoadipicacid.pcaec402	-0.010392608	0.026579618	0.696405261
pcaec420.pcaac380	0.018243998	0.046666105	0.69644333
pcaac426.pcaac366	0.003590375	0.00918393	0.696448129
lysine.threonine	0.006126751	0.015672572	0.696462274
pcaec403.lysopcac280	0.002590603	0.006627825	0.696502201
creatinine.glutamine	-0.00368648	0.009433949	0.696576117
c2.pcaec422	-0.015191927	0.038887433	0.696652297
ornithine.lysopcac180	0.014260639	0.036503937	0.696655156
pcaac361.lysopcac182	-0.004127668	0.01056793	0.696711179
creatinine.pcaac406	0.008861244	0.022691389	0.696765046
pcaec405.methionine	-0.014772411	0.037830324	0.696779834
pcaac420.pcaec420	0.010895096	0.027901378	0.696783752
putrescine.pcaec403	-0.00663973	0.017004853	0.696802211
lysopcac281.lysine	-0.021848631	0.055956929	0.696806997
pcaec403.pcaac240	0.007125941	0.018252408	0.696839024
pcaec383.pcaac342	0.006629681	0.016982012	0.69685132
smohc241.kynurenine	-0.002001323	0.005126792	0.696872584
asparticacid.pcaac424	0.006181337	0.015835652	0.696889129
pcaec425.pcaac365	0.01578632	0.040456788	0.696993197
lysopcac203.c12	-0.025951388	0.066509669	0.697002268
pcaec341.pcaac363	0.003298803	0.008454833	0.697018507
pcaac425.pcaac240	-0.005512758	0.014131192	0.697059028
pcaac404.pcaac381	0.011584799	0.029700142	0.69709889
smc260.pcaac342	0.000640143	0.001641545	0.697169036
isoleucine.smc181	0.008531296	0.021877875	0.697178462
pcaec445.isoleucine	-0.006211312	0.015930003	0.697206527
pcaec402.methioninesulfoxide	0.004091268	0.010492799	0.697207143
c2.pcaac363	0.002271839	0.005827799	0.697269135
serine.glycine	-0.00766084	0.019659634	0.69738305
lysopcac203.pcaac360	-0.010031587	0.025744786	0.69739669

pcaec422.pcaec361	-0.007048198	0.018090437	0.697430649
c141oh.pcaec365	0.008293455	0.021293257	0.697520441
glutamicacid.pcaac320	-0.036371143	0.093384771	0.697528703
pcaec383.smc260	-0.012017587	0.030856311	0.697533728
prolinr.ornithine	0.012262261	0.031488139	0.697566644
c2.pcaec382	0.004032343	0.010357775	0.697654304
pcaac421.lysopcac181	-0.024476292	0.06287344	0.697662665
c141.pcaac342	0.000575739	0.001478955	0.697668035
c142.pcaac342	0.000709927	0.00182366	0.697668239
pcaec383.pcaac364	0.016362161	0.042044678	0.697761054
lysopcac182.smohc141	0.003214056	0.008260749	0.69782426
pcaac342.lysine	-0.017822785	0.045808508	0.697827317
lysopcac140.c51	-0.023523856	0.060462849	0.697833609
histidine.pcaac420	0.016802645	0.043187681	0.697835023
pcaac384.c182	0.00867869	0.022310155	0.697878784
c18.pcaac320	-0.012752264	0.03278371	0.697893572
c3.pcaec445	-0.032146435	0.082643146	0.697895705
pcaec340.pcaac360	-0.014313837	0.036801934	0.697922517
pcaec384.phenylalanine	-0.012525904	0.032218167	0.698039603
pcaec382.threonine	-0.010178749	0.026181027	0.69803995
alphaaminoadipicacid.pcaac403	0.003171223	0.00815832	0.698093863
phenylalanine.asymmetricdimethylar	-0.013790013	0.035478791	0.698113832
methionine.pcaac361	-0.012324245	0.031718335	0.698210226
serotonin.pcaec425	-0.001398725	0.003600213	0.698240563
serotonin.phenylalanine	-0.02103554	0.054152602	0.698286561
smohc241.pcaec365	0.009997731	0.025737922	0.698290787
c10.smohc161	-0.003561199	0.009167925	0.698292574
c51dc.lysopcac180	0.023776358	0.061216054	0.69832244
ornithine.smohc221	0.015607336	0.040201791	0.698452224
pcaec300.pcaac323	-0.017141521	0.044167557	0.698542872
pcaec420.h1	0.000779993	0.002010155	0.698599042
pcaec401.c142	0.011377935	0.029341893	0.698787326
pcaac361.glycine	-0.006084126	0.015692778	0.698838056
isoleucine.lysopcac281	-0.003328776	0.008586733	0.698865565
phenylalanine.pcaec444	0.039287075	0.101363867	0.698924957
pcaec422.methionine	-0.019519608	0.050363893	0.698934661
prolinr.asymmetricdimethylarginine	-0.0046977	0.012122997	0.698985066
pcaec402.smc202	0.034026822	0.087835441	0.699066565
c141oh.lysine	0.018882883	0.048751234	0.699111958
lysopcac170.c2	-0.024372656	0.062936421	0.69916588
c141oh.pcaec404	0.008943228	0.023095805	0.699191976
c141.lysopcac140	-0.000606063	0.001565568	0.699267736
pcaec422.pcaec363	0.010816705	0.027942842	0.699282027
pcaac402.lysopcac281	0.005732775	0.014813885	0.69936659
pcaac240.pcaec403	0.027572937	0.071253321	0.699378445

c0.smc160	0.022151335	0.057252909	0.699428184
putrescine.c4	-0.002736599	0.007073866	0.699459788
glutamicacid.pcaec402	-0.028466065	0.073589555	0.699488108
smc202.pcaec403	-0.004375153	0.011310666	0.699492181
pcaec424.c102	-0.007255706	0.018760114	0.69953222
pcaac426.putrescine	0.009048166	0.023396214	0.699551463
isoleucine.pcaac342	-0.001442771	0.003731071	0.699585106
pcaac365.asparticacid	-0.017578581	0.04546049	0.699594388
smc261.h1	0.000809693	0.002094477	0.699663295
h1.c182	0.010826199	0.028008658	0.699703537
pcaec402.lysopcac203	0.007516707	0.019447504	0.699716672
pcaec406.pcaac342	0.001974343	0.005108121	0.699718153
smc260.pcaac365	0.020690047	0.053531294	0.699723244
glycine.smc181	-0.020223112	0.05232771	0.69974789
pcaac420.asymmetricdimethylarginir	0.006899416	0.017855472	0.699797366
pcaac300.pcaac420	0.030293617	0.078410815	0.699840631
pcaac342.pcaec444	-0.030771972	0.079662169	0.699887923
pcaec321.smc202	0.015480563	0.0400839	0.699944696
pcaac344.c51	-0.022971906	0.059481606	0.699946312
pcaec342.pcaac425	0.030509386	0.079013976	0.700002015
alphaaminoadipicacid.c51dc	-0.042612191	0.110366161	0.700022781
smohc161.pcaec420	0.006479901	0.016783383	0.700028746
smc241.c18	-0.008802656	0.022800874	0.700046211
pcaec404.c12	0.02409461	0.062414544	0.700064711
pcaac341.asymmetricdimethylarginir	0.010834241	0.028065711	0.700072368
pcaac363.pcaec425	-0.001732091	0.004487116	0.700084801
pcaac424.pcaec401	0.026551843	0.068798366	0.700141905
pcaac323.pcaec406	0.002162986	0.005605266	0.700180737
pcaec403.pcaac360	-0.016835737	0.043629505	0.700184382
c141oh.pcaac344	-0.018764289	0.048633092	0.700218269
pcaac365.c14	-0.025802174	0.066875192	0.700224169
pcaec406.acetylornithoine	-0.009239501	0.023947497	0.700226052
smc260.pcaec402	0.026564219	0.068856817	0.700251172
pcaac426.pcaac383	0.002643677	0.006852977	0.700264915
pcaec402.pcaac342	0.001862332	0.004827698	0.70027296
smc202.c162	-0.039900589	0.103456225	0.700334808
pcaec421.pcaec342	0.033534924	0.086954202	0.700345263
pcaec445.c101	-0.004193359	0.010873849	0.700363637
smohc222.c16	0.012528191	0.032489586	0.70038631
lysopcac280.alphaaminoadipicacid	-0.000601692	0.001560604	0.700427359
lysopcac170.pcaec343	0.024902684	0.064602622	0.700483522
alanine.pcaec341	-0.014625521	0.037942984	0.70049412
pcaac240.smc181	0.002074981	0.0053837	0.700524645
pcaec424.alanine	-0.00387272	0.010048333	0.700531997
pcaac420.pcaec383	0.026488753	0.068734259	0.700554201

c51dc.pcaec425	0.001663847	0.004318455	0.700621819
pcaac403.ornithine	0.015845846	0.041135081	0.700675626
smohc141.pcaec420	0.005984015	0.015535603	0.700700862
ornithine.pcaec365	0.01009574	0.026219531	0.700800046
pcaac365.pcaec341	-0.034426319	0.089430191	0.70087004
pcaac385.acetylornithoine	0.01509915	0.039226255	0.700890204
smohc141.pcaac342	0.000447068	0.001161677	0.700947251
pcaac361.pcaec406	0.001538114	0.003997223	0.700985429
alanine.ornithine	0.016770711	0.043596476	0.701070698
arginine.pcaac320	0.018956483	0.049307094	0.7012355
lysine.pcaac342	-0.001339325	0.003485806	0.701409381
c182.serine	0.008183647	0.021301807	0.701443484
pcaac344.pcaec343	-0.032299683	0.084083149	0.701470412
lysopcac140.c8	0.011952406	0.031119427	0.701513275
pcaac366.pcaec341	-0.009150611	0.023824667	0.70151359
pcaec445.lysine	0.031186354	0.081240478	0.701664709
c12.pcaac281	-0.00025301	0.000659209	0.70171588
pcaec301.acetylornithoine	0.003687892	0.009612296	0.701822202
lysopcac160.pcaec340	0.021726548	0.056635849	0.701856054
pcaac342.pcaec382	-0.003886307	0.010132007	0.701893728
pcaac363.pcaec421	0.001634983	0.004262632	0.701897781
smc241.c182	-0.007022134	0.018308572	0.701911359
smc160.pcaec444	0.032918609	0.085830371	0.701920602
pcaec365.pcaac363	0.002784228	0.007261531	0.702001473
c181.pcaec386	-0.001814113	0.004731837	0.702028982
pcaec444.c16	0.011018529	0.028748856	0.702114801
histidine.pcaac362	-0.002728096	0.007118734	0.702144944
smc240.pcaac383	-0.00100297	0.002617224	0.702151512
smohc161.pcaac422	-0.009158667	0.023900838	0.702169575
lysopcac170.smohc141	-0.007572713	0.019762292	0.702172835
pcaac322.c8	0.011973821	0.031250246	0.702195576
c14.pcaac321	0.008335168	0.021755391	0.702216363
c141.methioninesulfoxide	0.004764826	0.012436556	0.702216736
ornithine.pcaac342	-0.001457729	0.003805424	0.702264542
asparagine.arginine	0.007401675	0.019322333	0.702266625
pcaac386.pcaec363	0.008540849	0.022303656	0.702361491
pcaac323.pcaec301	-0.020407082	0.053297854	0.702396594
pcaec421.c162	0.027387444	0.071556606	0.702507078
lysopcac180.c141oh	0.026269591	0.068637692	0.702514329
smohc222.pcaec380	0.01630984	0.042616903	0.702529217
pcaac341.alanine	-0.007583471	0.019815482	0.702532107
smc161.pcaac366	0.011038829	0.028846028	0.702549298
smohc161.pcaac342	0.00046984	0.001228057	0.702618333
pcaac360.taurine	0.01613395	0.042185792	0.702720387
pcaec321.c16	0.01154845	0.030197645	0.702735942

smc241.pcaec446	-0.007811992	0.020431716	0.702796954
pcaec444.alphaaminoadipicacid	-0.000606341	0.00158596	0.702818091
pcaac363.lysopcac182	-0.005653352	0.01478752	0.702827125
pcaac364.pcaec341	0.025762052	0.067387987	0.702835389
pcaac240.taurine	0.014225459	0.037212887	0.702851698
creatinine.pcaec402	-0.01292944	0.033829146	0.702906568
pcaac380.pcaac383	0.002888713	0.007559569	0.702959531
smohc222.pcaac342	0.000430244	0.001126779	0.703175165
smc180.pcaac342	0.000530676	0.00138982	0.703178546
pcaec364.pcaac381	-0.012601378	0.033008997	0.703233931
c141oh.isoleucine	-0.002848412	0.007461365	0.703234679
smc260.lysine	0.02025663	0.053065967	0.703256443
smc161.pcaac323	0.027334679	0.071621372	0.703308331
alanine.pcaac384	0.015159279	0.039721237	0.703318444
serotonin.h1	-0.001519301	0.003981736	0.703373242
smc202.h1	0.000313796	0.000822504	0.703413599
pcaec386.pcaec340	-0.021687277	0.056862354	0.703497643
pcaec342.pcaec402	-0.016646708	0.043658073	0.703573304
pcaec363.smohc161	-0.004234369	0.011106133	0.703597933
pcaac341.pcaec365	-0.009018323	0.023661764	0.703693585
lysine.pcaac343	0.006400833	0.016795848	0.703722247
pcaec342.pcaec383	-0.020625346	0.054125015	0.703742603
pcaac405.creatinine	0.013131328	0.034459561	0.703745402
c101.pcaac342	0.000536884	0.001409018	0.703768153
c8.pcaac405	-0.022429825	0.058869298	0.7037856
pcaec300.pcaec360	0.002144517	0.00562877	0.703799203
prolinr.smohc141	-0.005862413	0.015388701	0.703826258
pcaac403.pcaac425	-0.05167769	0.135676999	0.703876618
c16.pcaac341	-0.002136238	0.005609015	0.703898577
pcaec382.lysine	0.035133171	0.092248599	0.703902132
smohc161.smohc222	-0.002365695	0.006211627	0.703904802
leucine.threonine	0.008999856	0.023631022	0.703905066
putrescine.pcaec342	0.020658282	0.054259791	0.703993974
pcaec425.pcaec423	0.005118131	0.013443139	0.70399745
smc241.pcaac426	-0.016875168	0.044327559	0.704021101
smc160.pcaac342	0.000503323	0.001322497	0.70410054
pcaac401.pcaec361	0.006763613	0.017773709	0.704133754
pcaac403.glycine	-0.006082823	0.015986164	0.704159544
glutamine.putrescine	0.011735185	0.030846557	0.704209948
smohc161.threonine	-0.01102714	0.028993963	0.704292922
methionine.methioninesulfoxide	0.004902155	0.012892377	0.704358577
asparagine.acetylornithoine	0.008188051	0.021539935	0.70443502
pcaec360.pcaac363	-0.005134904	0.013509281	0.704458366
lysopcac204.creatinine	-0.004628844	0.012178763	0.704478289
pcaac300.pcaec444	0.027520535	0.07241492	0.704504567

pcaac384.lysopcac281	0.004197494	0.011048078	0.704585889
pcaac385.c14	-0.009344457	0.024598571	0.704624187
pcaec360.pcaec425	-0.000842031	0.002216638	0.70463143
pcaec443.asparagine	-0.008105224	0.021340645	0.704680372
lysopcac140.spermidine	-0.023408975	0.061642505	0.704716224
c141oh.pcaec405	-0.016341461	0.043032808	0.704723285
lysine.phenylalanine	0.010747411	0.02830179	0.704724111
c12.pcaec425	-0.002218778	0.005843097	0.704736593
pcaec364.glycine	0.01267315	0.033376766	0.704756243
pcaac361.pcaec385	-0.020906738	0.055062674	0.704763585
c4.pcaec383	0.020202618	0.053209005	0.704767774
c5.pcaec421	0.000562803	0.001482331	0.704775332
pcaac344.c16	0.00825084	0.021735794	0.704832422
pcaec322.pcaec342	-0.040178138	0.105853154	0.704856161
glutamine.c162	-0.024081605	0.063453098	0.704890724
pcaac321.pcaec342	0.032528818	0.08572855	0.704948851
serotonin.tryptophan	-0.001381488	0.003641448	0.70499396
pcaac386.pcaec360	0.001619166	0.004268344	0.705020614
acetylornithoine.pcaac281	-0.001195099	0.003150867	0.705058191
pcaec422.pcaec446	-0.011242631	0.02965506	0.705190588
c141oh.pcaec424	0.009496724	0.025050001	0.705192581
smohc241.lysopcac140	-0.000268466	0.000708343	0.705269867
pcaac386.pcaac281	-0.000505407	0.001333686	0.705307563
creatinine.pcaec403	0.008778466	0.023166393	0.705325677
phenylalanine.glutamine	0.00669776	0.017677682	0.70536184
lysopcac140.c141oh	0.036697587	0.096890323	0.705456948
lysopcac170.c102	0.010908161	0.028801316	0.705468519
pcaac421.pcaac363	0.004010202	0.010590307	0.705521196
lysopcac161.pcaac420	0.022321456	0.058958791	0.705575277
smc202.pcaac422	-0.016578011	0.043791539	0.705595779
pcaec380.valine	0.016752448	0.044262818	0.705662295
smc161.pcaac362	0.006812267	0.018006804	0.705781253
pcaac322.smc260	0.007856349	0.020767566	0.70579404
pcaec361.pcaec364	0.002141396	0.005660665	0.705797642
pcaec423.asymmetricdimethylarginir	0.005694075	0.015061289	0.705970946
smc241.lysopcac160	0.01333591	0.035292476	0.706113192
pcaec361.lysine	0.026511382	0.070165368	0.706133166
pcaac363.c3	-0.008938818	0.023659764	0.706158891
lysopcac160.c182	0.010710901	0.028358724	0.706242929
pcaac341.tyrosine	-0.022987522	0.060875458	0.706300558
pcaac426.c18	-0.015124445	0.040074017	0.706450911
pcaac426.pcaac361	-0.016159473	0.042827222	0.706521319
pcaec300.pcaac383	0.001165983	0.003090223	0.706524508
pcaec363.pcaec362	0.006716748	0.017802272	0.706536553
pcaec342.c162	-0.029051795	0.07700279	0.706547575

smohc222.pcaec446	-0.007202661	0.019092379	0.706569206
c4.pcaac365	-0.023024081	0.061041323	0.706617212
pcaec301.pcaac342	-0.001224223	0.003246453	0.706686125
pcaac385.smohc141	-0.004632178	0.012284941	0.706711339
acetylornithoine.lysine	-0.03048827	0.08086073	0.706722308
smc260.pcaec301	0.016405224	0.043512761	0.706741361
lysopcac161.alphaaminoadipicacid	-0.001834664	0.004866427	0.706753783
smc261.lysopcac181	0.018784363	0.049835289	0.706809614
pcaac361.pcaec380	-0.018937125	0.05024994	0.706861721
pcaac363.pcaec406	0.001385626	0.003677497	0.706916248
c14.pcaac361	0.013836055	0.036733731	0.707010477
pcaac342.c8	0.014704719	0.039059495	0.707150087
pcaec384.lysopcac170	0.003148825	0.00836413	0.707151612
pcaec382.c0	0.000399575	0.00106139	0.707154239
pcaac365.threonine	0.01193237	0.031697492	0.707168291
serotonin.pcaac342	0.001113284	0.002957362	0.707168603
c10.methionine	0.012075048	0.032079631	0.707195487
asparticacid.c141	-0.01509704	0.040118831	0.707270017
pcaac402.pcaec365	-0.006773642	0.018000373	0.707271827
pcaec443.c5	-0.007913994	0.021033185	0.707303962
pcaec422.asparagine	0.003634028	0.009658913	0.707323697
pcaac403.smc181	0.001471741	0.003911813	0.707328065
pcaac380.pcaec425	-0.000848892	0.002256623	0.707366453
smohc221.pcaac342	0.001206152	0.00320651	0.707381873
pcaec361.pcaec446	-0.011947075	0.031765692	0.707424377
c5.pcaec343	-0.037452386	0.099605513	0.707493194
glycine.pcaec402	-0.016278894	0.043302738	0.707548793
pcaec405.glutamicacid	0.021509563	0.05723441	0.707635547
alanine.pcaac406	-0.014258748	0.037942786	0.707649804
pcaec385.phenylalanine	0.008766995	0.023340392	0.707784288
lysopcac280.pcaec386	-0.002408158	0.006412535	0.707840277
pcaac322.pcaec445	-0.015435032	0.041120187	0.707970395
smc160.pcaec406	0.001725464	0.004597783	0.708031262
pcaec381.valine	0.009227128	0.024587678	0.708036699
pcaec385.glutamicacid	0.011376697	0.030338891	0.708249819
arginine.pcaec421	-0.003300631	0.008804622	0.708333245
lysopcac160.pcaac366	-0.003925425	0.010475366	0.708441366
glutamicacid.glycine	-0.008844935	0.023605779	0.708467686
pcaec364.smohc141	0.016309787	0.043537378	0.708525512
lysopcac140.pcaec301	-0.016633462	0.044406679	0.708558569
c102.acetylornithoine	0.002931795	0.00782762	0.708578084
smc261.pcaac321	0.008526961	0.022769903	0.708623383
pcaec404.ornithine	0.012015971	0.032087766	0.708632146
pcaac320.smc241	-0.003601961	0.00961919	0.708644212
pcaec384.pcaec423	0.005195292	0.013874677	0.708652821

pcaac360.smc260	-0.011426387	0.030518489	0.708679177
c182.pcaec401	-0.015214013	0.040636494	0.708690905
c8.pcaac342	0.000202321	0.000540398	0.708691373
phenylalanine.c18	0.013334924	0.035622778	0.70873237
pcaac386.c4	0.005692309	0.015207695	0.708756498
pcaac323.pcaec363	-0.007863542	0.021009674	0.708773204
acetylornithoine.asparticacid	0.004267699	0.011406861	0.708882741
pcaac404.lysopcac281	0.004034781	0.010784931	0.708898736
pcaec341.pcaec406	0.001525593	0.004077901	0.70889912
asparticacid.lysopcac280	-0.002569134	0.006867815	0.708920753
serine.c5	-0.003860662	0.010320547	0.708926633
lysine.pcaac321	0.01563553	0.041808215	0.708995679
lysopcac204.pcaac342	-0.001310113	0.003504021	0.70906537
pcaec381.smc241	0.010405332	0.027833372	0.709098491
c4.pcaac342	0.000630715	0.001687292	0.709128826
c102.pcaac360	0.008739029	0.023379163	0.709134208
lysopcac161.smc160	-0.013742935	0.036766405	0.709137966
pcaac366.pcaec384	0.006784372	0.01815021	0.70913818
lysopcac280.smc240	-0.016647091	0.044541882	0.709175368
pcaec420.asymmetricdimethylarginir	-0.007193139	0.019250017	0.709228037
pcaec384.pcaec362	0.017038063	0.045600024	0.709248598
c10.pcaac342	0.000186473	0.000499073	0.709251045
histidine.pcaac300	-0.016600705	0.044432527	0.709267528
serotonin.spermidine	0.020571052	0.055079012	0.709366606
pcaec363.pcaac342	0.002113384	0.005658905	0.709382099
pcaec301.smc180	0.008771299	0.023494401	0.709475666
c16.pcaec420	0.010334141	0.027680677	0.709476839
c10.pcaac361	0.012447498	0.033342262	0.709483659
pcaec403.c18	0.018079672	0.048445527	0.709579468
pcaec425.lysopcac140	0.000683185	0.001830913	0.709621878
lysopcac280.valine	0.010016705	0.026846061	0.709638522
prolinr.kynurenine	0.003229053	0.008654329	0.709640118
c141.pcaec382	0.004453847	0.011937163	0.709644984
pcaec386.lysopcac204	-0.01014263	0.02718495	0.709652703
lysopcac180.phenylalanine	0.017854327	0.047857539	0.709671163
smohc141.c102	0.011370487	0.030489862	0.709779265
glutamine.lysopcac161	-0.014460181	0.038789147	0.709881352
pcaec402.pcaac281	-0.00105952	0.002842212	0.709888147
c101.pcaec425	-0.002725502	0.007313058	0.709955276
pcaac365.pcaec380	-0.018189086	0.048814541	0.710009978
pcaac380.pcaec420	0.010856149	0.029137961	0.710038671
lysopcac170.pcaec322	0.017601704	0.047253575	0.710100203
prolinr.histidine	-0.018007496	0.048345996	0.710117559
smc240.taurine	0.01226769	0.032939816	0.710150148
lysopcac181.smc240	0.019780214	0.05311508	0.710168301

pcaec383.lysine	0.026024435	0.069887478	0.710188164
pcaec361.h1	0.000434563	0.001167114	0.710215361
c182.pcaec365	-0.009275913	0.024914358	0.710235896
pcaac320.h1	0.00048628	0.001306197	0.710254663
lysopcac204.pcaec385	0.010525178	0.028275994	0.710296657
pcaec360.pcaec402	-0.015467126	0.041561961	0.710359009
lysopcac181.methioninesulfoxide	-0.022090523	0.059360022	0.710360072
pcaac365.pcaac422	-0.011085004	0.029787755	0.710368884
pcaec382.smc181	0.00120671	0.003243203	0.710413071
smc241.pcaac381	0.012878765	0.034614168	0.710418377
pcaac405.lysopcac281	0.004011057	0.010785711	0.710552137
h1.serine	0.0121491	0.032671826	0.710577183
smc241.pcaac342	0.000424822	0.001142478	0.710584217
pcaac381.pcaac322	0.018359722	0.049375628	0.71058816
pcaac300.pcaac426	0.021987618	0.059139788	0.710623172
c102.pcaac342	-0.006058152	0.016304246	0.710787843
leucine.putrescine	0.009528882	0.025654547	0.710890689
pcaec446.smc241	0.005417257	0.014585531	0.710903637
pcaec385.smc180	-0.006128397	0.016501109	0.710918566
pcaec404.smc161	-0.022790112	0.061406306	0.711109283
pcaec301.serine	0.012181289	0.032829942	0.711179345
pcaec443.c14	0.012787801	0.034473454	0.711250493
pcaec385.pcaec425	0.00391357	0.010550363	0.711253873
prolinr.pcaac424	0.003988308	0.010753449	0.711294993
lysine.valine	0.022061789	0.059484456	0.711297353
pcaec380.asparticacid	-0.012562043	0.033875569	0.711337683
prolinr.pcaec402	-0.008940347	0.024113991	0.711393776
spermidine.pcaac240	-0.01293404	0.034886315	0.711397691
pcaac360.pcaac420	-0.053077301	0.143182466	0.711435823
pcaec382.lysopcac182	-0.017759517	0.04791304	0.711462199
alphaaminoadipicacid.pcaac342	-0.000727783	0.001963614	0.711482333
pcaec364.pcaec301	0.02409191	0.065022628	0.711570455
smohc221.pcaec403	-0.007673489	0.020711326	0.711584239
pcaac362.pcaec342	-0.035168169	0.094923798	0.71159079
pcaec420.alanine	-0.002933484	0.007917933	0.711592524
pcaac385.c3	0.008422488	0.02273736	0.711637897
pcaac426.c8	0.013652593	0.03685714	0.711642237
pcaec406.threonine	0.011315716	0.030551448	0.711669654
pcaac402.smc240	0.020989547	0.056671608	0.711677722
smohc241.pcaac383	0.002060236	0.005563065	0.711699637
asparticacid.pcaec361	0.002869189	0.00774829	0.711731113
c0.c141oh	-0.033900803	0.091565245	0.711777987
pcaec445.pcaac322	0.019124478	0.05165674	0.711788591
phenylalanine.pcaec405	0.019964894	0.05396049	0.71196056
pcaac404.pcaec425	0.002028887	0.005484045	0.711982222

alphaaminoadipicacid.c102	0.00607518	0.016421518	0.71198922
pcaac404.isoleucine	-0.009362248	0.025308028	0.712004455
pcaac384.lysopcac204	-0.004556355	0.01231786	0.712029449
pcaac421.pcaac426	0.028399079	0.076786022	0.712067597
pcaac401.taurine	-0.018831897	0.050950107	0.712240472
pcaec363.pcaac320	-0.023089013	0.062483283	0.712308444
lysopcac204.lysopcac181	-0.020973982	0.056762734	0.712323594
histidine.pcaec420	-0.008409617	0.022765148	0.712394346
pcaec385.pcaac385	-0.011731394	0.031757473	0.712395633
c181.smc260	0.007408881	0.020058884	0.712432204
prolinr.creatinine	0.003343794	0.009053156	0.712436198
pcaac364.c142	0.006138592	0.016621354	0.712459726
pcaec405.smc261	0.025286246	0.068482183	0.712520158
smohc141.glycine	-0.015021587	0.040688434	0.7125593
pcaec446.acetylornithoine	-0.016789013	0.045497889	0.712692602
pcaac422.lysine	0.026018127	0.070522076	0.71274504
c12.pcaac342	0.000671453	0.001820026	0.712753132
c2.smc181	-0.00415486	0.011264769	0.712818823
pcaac402.pcaec403	0.047853118	0.129743567	0.712824969
pcaac401.lysopcac204	-0.027459866	0.074463668	0.712869445
pcaec386.pcaac383	0.002053803	0.005569412	0.712872369
pcaec386.c8	-0.01501719	0.040724885	0.71288533
pcaac360.pcaec420	0.007919892	0.021492373	0.71307091
smc181.serine	-0.016517243	0.044826529	0.713091368
lysopcac181.pcaac405	0.012024356	0.032637414	0.713126934
pcaec381.pcaec341	-0.012236281	0.0332147	0.713143955
asymmetricdimethylarginine.pcaac42	-0.001583663	0.004299265	0.713175841
pcaec361.c8	0.012734933	0.03459022	0.713317921
pcaac381.lysopcac170	0.013051196	0.035451462	0.713335038
pcaac381.pcaec425	-0.000545207	0.001481501	0.71343368
pcaac366.lysopcac203	0.01566682	0.042574745	0.713453171
smc181.pcaec386	0.00551577	0.014989813	0.71346506
pcaec386.pcaec422	-0.006067348	0.016494377	0.713557707
pcaec422.taurine	-0.018595861	0.050577177	0.713684485
glutamine.ornithine	-0.008009799	0.021788977	0.713732953
pcaac323.pcaec420	-0.009235601	0.025124815	0.713747123
glutamine.pcaec421	0.002191443	0.00596303	0.71380957
smc260.pcaec425	0.000854169	0.002324905	0.71388799
pcaac361.pcaac363	-0.003805847	0.010360535	0.713931681
smohc222.pcaec423	0.004979994	0.013557656	0.713947345
pcaac322.pcaec425	-0.002210582	0.006018231	0.713951425
pcaec404.lysopcac140	0.000660717	0.001799025	0.713988978
pcaec380.pcaac321	0.006884193	0.0187465	0.714017438
pcaec420.pcaec382	-0.004132271	0.011258557	0.714160081
smc160.lysopcac160	-0.008025985	0.021876489	0.714276674

pcaec361.smc240	0.014742872	0.040191947	0.71432549
creatinine.pcaec443	0.017541204	0.047832781	0.71439418
c141oh.lysopcac180	-0.012990871	0.035443155	0.714537371
pcaec406.pcaac426	-0.030691113	0.083747129	0.714577056
lysopcac203.pcaac380	0.024269907	0.06622834	0.714588662
c5.c18	-0.031149748	0.085011151	0.714617293
c4.pcaac403	0.00375779	0.010258368	0.714695455
smc260.h1	0.000350526	0.000957286	0.714805769
pcaac384.pcaec362	-0.003768033	0.010292142	0.714849404
pcaec401.h1	0.000377167	0.001030266	0.714864862
lysopcac181.alanine	-0.007569965	0.020684275	0.714946906
pcaec380.pcaac342	-0.00209928	0.005736195	0.71495139
c5.glycine	-0.016151677	0.0441388	0.714982315
lysopcac204.pcaac240	0.00566023	0.015468686	0.714992623
c51dc.pcaac342	0.000777562	0.00212544	0.715051839
isoleucine.putrescine	0.010510782	0.028731339	0.715056286
pcaac366.pcaec403	-0.006945198	0.018993225	0.715177483
pcaec384.putrescine	-0.019253974	0.052693885	0.715381633
c2.pcaec322	-0.018577389	0.050842797	0.715384716
pcaec362.lysopcac182	0.010584519	0.028974464	0.715447061
putrescine.pcaec301	-0.019133494	0.052381661	0.715472548
acetylornithoine.pcaac385	0.003291387	0.009011786	0.715501918
asparagine.methioninesulfoxide	-0.004085091	0.011190368	0.715633854
citruline.smc180	-0.008586407	0.023526432	0.7156977
pcaec340.pcaac383	0.001426848	0.003909836	0.715720437
smohc222.pcaec420	0.00508586	0.013938045	0.715755811
c142.pcaec363	-0.003372199	0.009244161	0.715828949
pcaac320.pcaac381	-0.013936766	0.038224329	0.715968765
putrescine.pcaec363	0.008530303	0.023401177	0.716028416
pcaac420.smc241	0.00370865	0.010175069	0.716058612
pcaec382.lysopcac280	0.003048192	0.008364044	0.716091407
pcaec381.asparagine	0.004613009	0.012658047	0.716096638
phenylalanine.smc241	0.003489412	0.009575282	0.716107196
prolinr.smc241	0.002064265	0.005666049	0.716179397
pcaec443.smc161	0.028674351	0.078714833	0.716209508
pcaec420.c142	0.008459286	0.023229182	0.716295228
alanine.pcaac342	-0.000950155	0.002609544	0.71633894
pcaac366.pcaac342	-0.001056729	0.002902419	0.716355368
smc261.pcaac342	0.000441402	0.00121262	0.716413959
prolinr.lysopcac182	-0.006689545	0.018383736	0.716505664
glutamicacid.lysopcac161	0.020258597	0.055678131	0.716529466
pcaec445.c182	-0.014195226	0.039018152	0.716560119
pcaec406.pcaac363	0.00301043	0.008278238	0.716675518
h1.pcaec386	0.003302352	0.009081295	0.716684921
lysopcac170.pcaec386	0.003613006	0.009937218	0.716729679

pcaac426.c182	0.008643719	0.023774292	0.716736429
alanine.alphaaminoadipicacid	0.002345504	0.006451352	0.716741165
pcaac405.lysopcac204	-0.004315311	0.011872239	0.716807264
spermidine.glutamicacid	-0.010164392	0.027968832	0.716852413
pcaac404.lysopcac170	0.004061319	0.011176595	0.716883246
smc241.pcaac320	0.014256858	0.039249928	0.716990905
pcaec421.pcaec406	-0.02067816	0.056932082	0.717009686
smohc141.acetylornithoine	-0.007819623	0.021531992	0.717042875
c181.asparagine	-0.011894647	0.032756613	0.717073412
lysopcac280.pcaec445	-0.037203867	0.10247033	0.717112432
pcaac404.pcaec445	-0.00773415	0.021307611	0.717182257
lysopcac281.tyrosine	-0.020909957	0.057618447	0.717235991
c101.lysopcac161	-0.017977075	0.049541407	0.717261462
pcaac385.c51	-0.011871366	0.032716828	0.717274656
pcaec362.pcaac343	0.009518263	0.026235239	0.717310042
smc202.pcaac425	-0.021895079	0.060378241	0.71743868
leucine.smc241	0.006123858	0.016887309	0.717439544
pcaec381.lysopcac140	0.00062147	0.001713804	0.717443202
lysopcac182.smc260	0.010724679	0.02957584	0.717450407
smc161.pcaac300	-0.016151018	0.044543546	0.717470391
pcaac420.c16	0.010180031	0.028080447	0.717513964
pcaac404.pcaec421	0.00035618	0.000983019	0.71766159
lysine.pcaec361	0.005184458	0.014314182	0.717767912
citruline.pcaac384	-0.022464384	0.062025799	0.717777027
lysopcac182.pcaec365	0.006304358	0.017411583	0.717851344
c18.pcaec384	-0.007881941	0.021774244	0.717921271
pcaec406.pcaac366	0.00777416	0.021478346	0.717944552
h1.c141oh	0.021143812	0.058417334	0.717951443
pcaac381.smc181	0.001609328	0.004447547	0.718024474
lysopcac180.pcaac300	-0.011772256	0.03253711	0.718051367
c14.pcaec342	-0.020444539	0.056523883	0.718135457
pcaac402.pcaac344	0.024136292	0.066733224	0.718145947
lysopcac160.smc241	-0.014773289	0.040848498	0.71816291
prolinr.arginine	0.006496831	0.017963958	0.7181639
pcaac401.c51	-0.03257342	0.090067465	0.718166449
pcaec340.pcaac361	0.012662255	0.035012233	0.718169037
smc181.pcaac240	-0.003114041	0.008611824	0.71820754
histidine.pcaec362	0.004207059	0.011634624	0.71820937
lysopcac203.pcaac342	-0.001201476	0.003323548	0.718279607
smc202.pcaac321	0.009023218	0.024962514	0.718304462
c18.c4	-0.010308971	0.028521386	0.718322091
pcaec384.methioninesulfoxide	0.003776786	0.010450163	0.718350234
smc161.pcaec420	0.004123086	0.011409093	0.718367646
lysine.pcaec341	0.010625325	0.029408678	0.718432538
pcaac422.pcaac322	0.024156153	0.066881181	0.718521177

pcaec300.pcaac425	-0.029669729	0.08214901	0.718528981
c14.pcaac344	-0.023436072	0.064915142	0.718635916
smc260.pcaec382	-0.005035795	0.013949317	0.718650606
pcaec425.smc181	0.004107398	0.011378051	0.718660688
asymmetricdimethylarginine.smc181	-0.006849601	0.018975945	0.718683631
pcaec402.glutamicacid	0.02273191	0.062985082	0.718723065
lysopcac170.pcaac342	0.003595316	0.009964966	0.718807945
smohc241.pcaac342	0.000691126	0.001915622	0.718816428
smc241.lysopcac161	-0.025258092	0.070017678	0.718850356
pcaec362.pcaac342	0.000700347	0.001941455	0.718854511
pcaac404.pcaec386	0.002386146	0.006614721	0.718854865
c142.c14	-0.014874253	0.041242645	0.718914888
smc202.lysine	-0.018941989	0.052526701	0.718941651
smohc141.methioninesulfoxide	0.003350614	0.00929156	0.718947669
pcaac320.pcaec363	-0.008915129	0.024723413	0.718957909
smohc221.c51	-0.011095217	0.030769797	0.718962892
asparagine.alanine	-0.015417398	0.042757088	0.718967971
smohc222.pcaac344	0.024210049	0.067144073	0.718977248
asparticacid.smohc241	0.019677591	0.054580608	0.719010962
smc180.smc260	-0.01197033	0.03320987	0.719069542
smc180.h1	0.00036912	0.001024198	0.719104127
pcaac425.pcaec386	0.005922871	0.016440989	0.719214771
leucine.pcaac342	-0.001298195	0.003603709	0.71922342
phenylalanine.pcaac420	0.02023738	0.056192661	0.719294996
c141oh.pcaec422	0.00492226	0.013669762	0.719339003
asparticacid.pcaec365	0.007152784	0.019871261	0.719434184
pcaac401.c3	-0.010155264	0.02821452	0.719453424
pcaac362.pcaac385	-0.00521356	0.01448524	0.719459525
methioninesulfoxide.c141oh	0.035465306	0.098541582	0.719474674
pcaac403.pcaac342	-0.005735184	0.015942869	0.719600334
pcaac320.pcaec386	0.003310063	0.009201664	0.719607188
c10.smohc141	0.011066455	0.030773244	0.719690372
smc240.c182	-0.014096601	0.039218138	0.719818741
pcaec405.c18	-0.01399663	0.038944838	0.71985201
pcaec422.pcaac321	0.004650731	0.012942812	0.719902025
pcaec321.methioninesulfoxide	0.004962389	0.013813427	0.719965759
pcaac364.smc202	0.011868775	0.033038308	0.719966536
pcaac404.smohc141	-0.003962136	0.011030123	0.719990631
pcaac343.smc202	0.013667288	0.038050943	0.720010471
pcaac421.pcaac425	0.034049704	0.094800261	0.720018571
pcaec422.smc240	0.012496418	0.034793808	0.720031058
pcaec361.lysopcac280	0.0045707	0.012728861	0.720086879
pcaec365.pcaec384	-0.009371894	0.026103604	0.720127742
pcaec343.smohc141	0.002650714	0.007392167	0.720458149
c51dc.alanine	0.003951277	0.011019209	0.720460684

pcaec420.smc181	0.004072362	0.011360695	0.720550371
pcaac422.smohc141	-0.014009946	0.039089387	0.72058975
pcaec365.lysopcac140	0.000306296	0.000854642	0.720602446
pcaec340.pcaac424	-0.00663338	0.018512324	0.720653595
pcaac341.c16	0.011172889	0.031181136	0.720653847
pcaec445.asparagine	-0.003654494	0.010202102	0.720737555
lysine.pcaec444	0.047306437	0.132097372	0.720806252
glutamicacid.taurine	-0.011362105	0.031730069	0.720829829
c5.lysopcac280	0.006261761	0.017489305	0.720869125
smc202.pcaec384	-0.011421616	0.031901475	0.72087352
serine.pcaec421	0.00077176	0.002155887	0.720910846
c3.pcaec386	0.004162933	0.011630092	0.720935367
pcaec301.pcaec380	0.017389517	0.04858585	0.72095911
serotonin.pcaac420	0.026375688	0.073695478	0.7209682
glutamicacid.lysopcac203	0.000926637	0.00258947	0.721007723
phenylalanine.pcaac342	-0.001659409	0.004637394	0.721019631
lysopcac170.c14	0.017427775	0.048705007	0.721026419
smc202.pcaec383	0.021697405	0.060639813	0.721037728
c3.smc181	0.006191378	0.017304761	0.721055069
smohc141.glutamicacid	0.011826052	0.033055331	0.721069518
lysopcac140.pcaac424	-0.001714739	0.004794171	0.7211394
pcaec380.smc202	-0.020541713	0.057433487	0.721147504
pcaec383.putrescine	0.026846732	0.075068801	0.721171816
pcaec444.pcaac425	0.021206594	0.05930156	0.7211885
smc181.c141oh	0.023804713	0.066587827	0.721272552
pcaac360.pcaec401	-0.015640037	0.043750031	0.721277899
pcaac300.pcaec423	0.002113151	0.005911249	0.721282912
c102.pcaec382	0.005624571	0.015734269	0.721288071
smc202.pcaec406	0.004870298	0.013624901	0.721300802
pcaec444.smc160	-0.020267044	0.056718091	0.721395046
c5.lysine	-0.043832015	0.122702413	0.721475207
pcaac344.pcaac366	0.004283662	0.011992112	0.721486778
pcaec424.pcaec384	-0.008139002	0.022787519	0.721514652
pcaac384.glycine	0.008486429	0.023760493	0.721517459
pcaac402.lysine	0.02555327	0.071544934	0.72151866
pcaac384.smohc241	0.021563825	0.060383913	0.721557387
pcaac386.pcaec446	0.018253932	0.051119988	0.721581257
pcaec380.pcaac406	0.014898376	0.041726764	0.721606712
pcaec361.pcaac361	0.010997177	0.03080748	0.721667621
pcaac364.isoleucine	-0.01032254	0.028923094	0.721718695
pcaac381.putrescine	0.018558143	0.052005627	0.721754078
pcaac420.pcaec446	0.033279672	0.093273337	0.721792532
smc180.pcaac424	0.003699851	0.010369858	0.721798742
smohc161.h1	0.000494945	0.001387268	0.721807949
pcaac366.lysopcac180	0.018619252	0.052193601	0.721839507

smohc222.pcaec421	0.000389318	0.00109147	0.72187171
pcaec380.phenylalanine	-0.015009717	0.04208525	0.721902326
pcaac365.pcaac380	-0.016293112	0.045696282	0.721975579
pcaac424.pcaac320	-0.018691366	0.052436962	0.722048951
pcaec340.pcaec406	0.003116363	0.008742985	0.722058236
pcaac344.pcaec425	-0.002842048	0.007977535	0.722196538
pcaac363.pcaac342	0.02798207	0.078546861	0.722203752
pcaac422.spermidine	-0.018227629	0.051173672	0.722245061
pcaac344.c182	0.010199053	0.028635314	0.72226082
lysopcac182.pcaac366	-0.003493186	0.009810208	0.722330921
c18.pcaec322	0.019431283	0.054584908	0.722401102
pcaec422.pcaac361	-0.017245053	0.048444954	0.722408998
pcaac381.c4	-0.007026037	0.019752689	0.722612117
pcaac420.creatinine	-0.005172142	0.014541567	0.722627426
lysopcac280.c12	0.017309116	0.048666042	0.722633766
pcaac361.valine	0.015245475	0.042865783	0.722645191
c0.asymmetricdimethylarginine	0.001735273	0.004879165	0.722650041
smc261.pcaac365	-0.010144373	0.028523615	0.722651039
smohc221.c12	0.018083642	0.050861578	0.722727268
c101.tryptophan	0.003486234	0.009807265	0.722780775
pcaec444.pcaac364	-0.011223879	0.031576103	0.722795556
lysopcac280.arginine	0.011722825	0.032986095	0.72284635
c8.lysopcac140	-0.000509549	0.001434143	0.722912292
smc241.pcaac363	0.002232485	0.006283863	0.722931669
pcaac401.pcaec365	-0.006986655	0.019666269	0.722940572
putrescine.pcaec380	-0.015653459	0.044064	0.722953346
asparagine.pcaac281	-0.000606814	0.001708212	0.722960888
lysopcac160.pcaac342	-0.001336041	0.003761867	0.723020531
pcaac321.acetylornithoine	0.002446961	0.00689075	0.723054774
pcaac364.pcaac363	0.002520233	0.007097512	0.723070648
pcaac420.pcaec384	0.005938036	0.016723329	0.723079465
smohc241.lysopcac182	-0.006665702	0.01877468	0.723108014
pcaec421.pcaec322	0.019623428	0.055294605	0.723218737
kynurenine.pcaec362	-0.003499986	0.009868469	0.723387021
pcaac364.pcaec301	0.026341733	0.074280652	0.723416263
lysopcac204.pcaac422	0.02966613	0.083687129	0.723517831
pcaec381.pcaac323	-0.021174365	0.05975316	0.723611002
alphaaminoadipicacid.pcaac380	0.011706329	0.033042519	0.723673191
smc202.c142	0.009692089	0.027367387	0.723772901
lysopcac140.lysopcac281	0.004091576	0.011556605	0.723848301
pcaec365.smc241	-0.005498321	0.015530781	0.723862683
pcaec403.pcaec422	-0.019004997	0.05371196	0.724008916
pcaec365.pcaec385	-0.029473836	0.083299186	0.724009466
pcaec404.kynurenine	0.002719397	0.007685717	0.724014165
pcaec384.pcaac342	0.003140668	0.008878003	0.724063802

c8.pcaec322	-0.014209101	0.040166338	0.724065231
pcaac383.smc180	-0.003668156	0.010370101	0.724089303
pcaec385.c51	-0.008485795	0.023990225	0.72409334
c2.pcaac342	0.001482972	0.004192702	0.724105033
creatinine.pcaac383	0.001494857	0.004226488	0.724116659
smc261.glutamicacid	0.012537278	0.035449567	0.724133485
smohc221.taurine	0.008178317	0.02312779	0.724171545
c141.pcaac422	0.016828985	0.047591732	0.724173608
pcaac402.pcaac342	-0.002189024	0.00619077	0.724186128
pcaec401.smohc241	0.01674073	0.047346396	0.724197329
serotonin.c10	-0.005813865	0.016457833	0.724437843
smohc222.pcaec403	-0.006611749	0.018716786	0.724442231
c2.asparagine	0.008773922	0.024841394	0.724483137
lysopcac280.c5	-0.004623004	0.01309333	0.724570529
citruline.pcaac421	0.00616124	0.017451117	0.724588283
lysopcac203.lysopcac160	0.00676428	0.019160055	0.72460051
pcaec360.pcaec341	-0.020909481	0.059237339	0.724647392
pcaec446.asparagine	-0.003403739	0.009644918	0.724702059
prolinr.pcaec342	0.029184981	0.082707899	0.724729634
pcaac380.glutamine	-0.004918766	0.013941037	0.724760825
pcaec340.smc161	-0.028457897	0.08065988	0.724770471
pcaec406.lysopcac182	-0.005017116	0.014220915	0.724781796
pcaac403.c101	0.019485268	0.055271418	0.724976693
phenylalanine.lysopcac180	0.014840178	0.042105744	0.725042277
prolinr.lysopcac160	-0.008489383	0.024087803	0.725053708
c14.pcaec402	-0.011261511	0.031976811	0.725246351
pcaac365.methioninesulfoxide	0.006370451	0.018092383	0.725299212
pcaec340.lysopcac140	-0.000236754	0.00067264	0.725396449
c3.asymmetricdimethylarginine	0.004331349	0.012306436	0.72541051
c8.c102	0.009731606	0.027650805	0.725419097
pcaec361.pcaec340	0.020324538	0.057753899	0.725441764
lysopcac180.smc161	0.020592506	0.05852461	0.725483411
pcaec322.pcaec301	-0.01790749	0.050909373	0.725564471
smc202.pcaac404	0.001977245	0.005622227	0.725615977
smc202.pcaac424	-0.002394353	0.006808395	0.725621191
pcaac404.ornithine	-0.003553109	0.0101043	0.725646053
pcaac342.pcaac381	0.009803229	0.027879408	0.725656202
pcaac380.histidine	0.015358754	0.043693579	0.725745399
smc181.threonine	-0.012206906	0.034729347	0.72576319
isoleucine.pcaec404	0.006341556	0.018044805	0.725802783
pcaec385.pcaac322	0.01381549	0.039324005	0.725884577
pcaac362.smc260	-0.010674323	0.030395042	0.725988059
smc241.pcaec422	-0.011256657	0.03205737	0.726021976
pcaac406.pcaec340	0.02203717	0.062761843	0.726034941
pcaac381.pcaac281	0.000779248	0.002219893	0.726105268

pcaec402.pcaec420	0.005631757	0.016043898	0.72611123
smohc161.serotonin	0.024809313	0.070678973	0.726117001
lysopcac203.pcaec420	0.008578497	0.024440374	0.726129789
c8.taurine	-0.013164689	0.037506908	0.726132223
pcaec424.pcaac380	-0.018246382	0.051992694	0.726171487
c102.pcaec425	-0.001564125	0.004458597	0.726268962
glutamicacid.c16	0.020038302	0.057140288	0.726362534
smc241.serotonin	0.020173664	0.057529705	0.72637817
histidine.smc260	0.004747551	0.013539003	0.726384031
pcaac281.alphaaminoadipicacid	-0.001169741	0.003337195	0.726489644
pcaec424.asymmetricdimethylargini	0.004184135	0.011940636	0.726568144
pcaac365.pcaac342	-0.001049842	0.002996967	0.726650381
c2.pcaac322	-0.015630786	0.044621829	0.726655608
smohc161.lysopcac204	0.007764611	0.022166227	0.72665894
pcaec360.pcaac361	0.015533688	0.044348083	0.726675973
c3.lysopcac280	0.001933494	0.00552176	0.726757116
c5.asymmetricdimethylarginine	0.008362086	0.023884518	0.726797668
c162.pcaec445	0.024280045	0.069352134	0.726802797
c142.threonine	0.00799537	0.022841161	0.726844616
c102.pcaac383	-0.001369205	0.003912335	0.726897745
pcaac402.lysopcac160	0.010077669	0.028799975	0.726936577
pcaac365.pcaec385	0.015027767	0.042964876	0.727049431
pcaac240.c12	0.025966675	0.074245204	0.727069299
pcaec401.pcaac402	-0.018437534	0.052724601	0.727104522
pcaec364.lysopcac203	-0.004626762	0.013233185	0.727150819
pcaac405.glycine	-0.011434241	0.032708192	0.727188168
smohc241.pcaac320	0.017978335	0.051460949	0.727356381
pcaec403.alanine	0.006793258	0.019447541	0.727391486
smc180.glycine	0.017341429	0.04964609	0.727399664
pcaec402.methionine	-0.007451834	0.021343816	0.727525363
pcaec421.pcaac342	-0.003957846	0.011339723	0.727606482
pcaac323.pcaec386	-0.002530894	0.007251507	0.727612906
asymmetricdimethylarginine.pcaec40	-0.015228801	0.043644234	0.727677237
ornithine.pcaec363	0.005331042	0.015278575	0.727683024
pcaec340.pcaec341	-0.04536405	0.130024547	0.727708751
pcaac300.pcaec382	0.002647703	0.007589529	0.727728038
pcaac420.pcaac424	-0.00336467	0.009645036	0.727737516
pcaac341.pcaec406	0.002475197	0.007098112	0.727840872
putrescine.pcaec425	-0.000493768	0.001416083	0.727860808
smohc161.c162	-0.02988486	0.08571171	0.727874437
pcaac344.lysopcac170	-0.003512947	0.010076082	0.727893211
pcaec382.pcaec406	-0.014787447	0.042432815	0.728006548
c141oh.pcaec386	-0.000753096	0.0021612	0.728028163
smohc241.pcaec403	-0.004443245	0.012753042	0.728069594
pcaec361.pcaec365	-0.005952739	0.017091569	0.728160701

pcaec300.serotonin	0.026585261	0.076340091	0.728188711
creatinine.pcaec341	-0.023122459	0.066424779	0.728299457
lysopcac203.c4	-0.007524686	0.021617707	0.728314555
pcaec446.smc240	0.023773519	0.068311383	0.72836161
c14.pcaac385	0.001926788	0.005541636	0.728604407
pcaec364.pcaac342	-0.003036716	0.008734426	0.728620029
smc202.threonine	-0.008952136	0.025752968	0.728662303
smc241.pcaec361	-0.004274164	0.012296814	0.728686781
smc160.pcaac365	0.018658889	0.053701813	0.728783775
creatinine.pcaec386	0.004756921	0.01369179	0.728802442
pcaec364.pcaec342	-0.039395159	0.113463	0.728968539
pcaac404.lysopcac161	0.026101989	0.075195494	0.729032531
pcaac401.pcaec425	-0.001136731	0.003275548	0.729097221
c12.pcaec403	0.006189112	0.017837125	0.729139322
c51dc.pcaec446	-0.015965949	0.046024145	0.729195909
pcaac281.pcaac406	-0.00992221	0.028607087	0.72924035
pcaec381.pcaac383	-0.004048342	0.011672101	0.729244329
c18.c162	-0.026440851	0.076235235	0.729249392
smc161.smc160	-0.036262142	0.104577654	0.729312319
c181.pcaec423	-0.00219391	0.006327165	0.729315269
pcaac381.pcaac320	0.032523935	0.093835501	0.729419227
smohc241.c101	0.006500789	0.018769872	0.729617299
smohc222.smc181	0.016459536	0.047535808	0.729681887
pcaec360.pcaac380	0.009914682	0.028640824	0.729743701
pcaec380.pcaec341	-0.006364796	0.018389337	0.729788434
smc161.c8	0.011170786	0.032280904	0.729836482
c10.c142	0.019670451	0.056849606	0.729867064
pcaec342.methionine	-0.013408086	0.038765679	0.729967086
alanine.smohc222	0.001350223	0.003903895	0.729974324
pcaac363.pcaac424	0.004101886	0.011860689	0.729994389
pcaec300.pcaac321	-0.010704267	0.030955234	0.730024736
methioninesulfoxide.c3	0.013869174	0.040114709	0.73007006
pcaac405.acetylornithoine	0.005714027	0.01653133	0.730137202
c141oh.c3	-0.016531383	0.047830268	0.730153975
pcaac360.pcaac342	-0.002932988	0.008487107	0.730187254
creatinine.pcaec446	-0.024191541	0.070018451	0.73024666
c2.smc261	0.011477119	0.033219012	0.730249564
asparagine.pcaec403	-0.006176626	0.017878686	0.730267756
c141.pcaec342	0.016825649	0.048710495	0.730307373
c8.methioninesulfoxide	0.004178422	0.012098279	0.730343484
pcaec423.smc202	-0.013988915	0.040508118	0.730371373
pcaec381.c2	-0.037449242	0.10846252	0.730418264
pcaac323.taurine	-0.006776857	0.01963442	0.730509542
c16.pcaec342	0.019042662	0.0551884	0.730587255
asparagine.pcaac300	-0.028170574	0.081679141	0.730703638

pcaec446.pcaec380	0.013504101	0.039154673	0.730705104
pcaac422.methioninesulfoxide	0.00452588	0.01313002	0.730850681
pcaec301.pcaec386	-0.001611169	0.004674636	0.730877019
pcaac364.pcaac383	-0.001438492	0.004174632	0.730938956
pcaac321.tyrosine	0.021514252	0.062451703	0.731002867
pcaac344.pcaec402	0.006501331	0.018877656	0.731078918
smc260.alphaaminoadipicacid	-0.000631226	0.001832994	0.731097184
lysopcac281.smc202	0.012405365	0.03603327	0.731167123
smohc221.pcaac321	0.006804967	0.019767758	0.731189296
pcaec340.pcaac321	-0.009256623	0.026890732	0.731200387
pcaec403.glutamicacid	0.029227177	0.084928918	0.731270958
c162.pcaec420	0.005530502	0.01607415	0.731327293
c3.c10	-0.004356182	0.012663807	0.731383661
smc160.pcaac403	-0.001155939	0.003360763	0.731410343
c51dc.serine	0.004487634	0.013048174	0.731427668
pcaac422.taurine	-0.016302201	0.047402269	0.731439867
pcaec362.pcaec401	-0.01001083	0.029115624	0.731501147
pcaec381.smc160	-0.019208213	0.055896832	0.731646187
smc161.pcaec406	0.002595165	0.007553058	0.731680442
lysopcac281.c0	0.000268184	0.000780598	0.731701867
pcaec401.pcaac342	-0.001755431	0.005109822	0.731718514
lysopcac281.pcaac300	-0.015934473	0.04638358	0.731721201
pcaac420.smc260	-0.010464183	0.030466724	0.731776919
pcaec361.tyrosine	-0.016609374	0.048359744	0.731783039
pcaac323.methioninesulfoxide	0.005775393	0.01681918	0.731837945
serotonin.glycine	-0.010168159	0.029614199	0.731858372
pcaac425.smc161	0.023825305	0.069411622	0.731939089
pcaec424.pcaec363	0.011101446	0.032352862	0.732021798
pcaac385.pcaac424	-0.002802114	0.008167987	0.732078791
pcaec381.pcaac342	-0.00414136	0.012072	0.732082908
lysopcac203.c142	0.015164051	0.044209366	0.732120157
pcaec341.smc202	0.012060291	0.035165888	0.732158549
glutamicacid.pcaac342	-0.001260854	0.003677413	0.732225907
kynurenine.pcaec425	0.000813495	0.002372735	0.732236037
acetylornithoine.c142	0.028762677	0.08389339	0.732238337
pcaec342.pcaec425	-0.000793625	0.00231499	0.732259124
pcaac425.pcaac420	-0.039665971	0.115723104	0.732299693
arginine.serotonin	0.030891026	0.090128535	0.732316277
c0.alanine	0.00267866	0.007817996	0.732403905
lysopcac140.c5	0.003217721	0.009393571	0.732465758
pcaac425.pcaec361	0.004249627	0.012406329	0.73247176
pcaac321.pcaac406	-0.014766576	0.043112585	0.732490574
smohc161.pcaac364	0.013179188	0.038483628	0.732527919
pcaac426.c2	0.024436297	0.071360332	0.732548122
smc241.pcaec343	0.022458267	0.065588492	0.73256584

asparagine.pcaac381	0.006420219	0.018750807	0.732576908
pcaac361.h1	-0.000582998	0.001702917	0.73261003
serine.pcaac422	0.01934609	0.056512007	0.73262274
pcaac380.putrescine	0.00942011	0.027519508	0.732644736
smc160.pcaac322	0.01487376	0.043458355	0.732684856
pcaac281.smc202	0.011121029	0.032504271	0.732769431
lysopcac281.valine	0.012585064	0.036789546	0.732812939
c14.pcaac323	0.018608512	0.054399417	0.732820975
pcaec406.lysopcac204	-0.024667933	0.072115051	0.732827211
pcaec361.pcaec322	0.020773965	0.060739997	0.732864003
pcaac365.c182	0.007727549	0.022594396	0.732866133
pcaac420.methionine	0.008815813	0.025777917	0.732881791
c102.pcaec404	-0.004184909	0.012237258	0.73288929
pcaac364.acetylornithoine	0.006622669	0.019371043	0.732961311
c51dc.smc241	0.005278029	0.015440728	0.733006279
pcaec321.pcaec386	-0.00424369	0.012415247	0.733015567
pcaec406.pcaac360	0.0144146	0.042172083	0.733021907
pcaec340.pcaac363	0.00287656	0.008415952	0.733026291
lysopcac161.pcaec365	0.008186486	0.023954122	0.733057511
pcaac343.alanine	-0.005177181	0.015153488	0.733138219
pcaac363.c8	0.010463778	0.030635029	0.733203481
lysopcac181.lysopcac140	-0.000349007	0.001022035	0.733263585
pcaac405.threonine	0.006831723	0.020008599	0.733295993
lysopcac280.pcaec425	-0.001653455	0.004842926	0.73331318
pcaec381.c8	0.011551166	0.033833256	0.733314618
pcaec424.pcaac426	0.021861987	0.064054699	0.733399261
leucine.smohc141	-0.003563261	0.010440764	0.733413012
pcaac425.pcaec445	-0.026342589	0.07719669	0.733445799
pcaec421.pcaac364	0.013660979	0.040046933	0.73353267
pcaec423.pcaac384	-0.011039705	0.032363135	0.733536006
pcaec404.pcaac344	0.019065686	0.055892645	0.733541386
pcaac342.c18	-0.00962701	0.028233884	0.733645777
histidine.c101	-0.005044256	0.014793905	0.733649599
asparagine.serotonin	-0.022236077	0.065235821	0.733733499
glutamine.pcaec423	-0.00330611	0.009704164	0.733858971
histidine.pcaac343	0.004976538	0.01460807	0.733873535
pcaac404.taurine	0.005933118	0.017430247	0.734082664
pcaec301.asymmetricdimethylarginir	-0.006813311	0.020016724	0.734091037
asparagine.pcaac380	-0.020190986	0.059333125	0.734152809
pcaac362.asymmetricdimethylarginir	-0.005038274	0.014806205	0.734165922
smc160.pcaac363	0.001868714	0.00549199	0.734180546
pcaac344.pcaac342	-0.000797669	0.002344285	0.734180927
asparticacid.h1	0.001649076	0.004846575	0.734184892
pcaac365.asparagine	0.003527009	0.010368134	0.734243615
leucine.glutamine	0.006282649	0.018469907	0.734260053

prolinr.putrescine	0.009104037	0.026775269	0.73436475
lysopcac170.pcaec423	-0.005031272	0.014801019	0.734431809
c2.methionine	0.005386682	0.015846592	0.734432213
pcaec422.alphaaminoadipicacid	-0.003983791	0.011720478	0.734452342
pcaec384.pcaac424	-0.006555832	0.019289677	0.734480821
c182.c141oh	0.030340774	0.089283099	0.734507532
pcaac404.pcaac424	0.001150685	0.003386869	0.734565935
pcaac384.acetylornithoine	0.012871477	0.037887962	0.734584107
smc180.smc241	0.012457345	0.036670376	0.734594117
c102.pcaac343	0.004525825	0.013322842	0.73459952
pcaac421.c51	-0.029936865	0.088156194	0.734686177
c8.pcaac344	-0.015669778	0.046156702	0.734759864
kynurenine.asparticacid	0.012411865	0.036571791	0.734840496
pcaec401.ornithine	-0.012856148	0.03788573	0.734873143
lysopcac180.transhydroxyproline	-0.013524861	0.039858886	0.734889321
smc260.lysopcac204	0.004585708	0.013523294	0.735055925
smc160.c3	-0.005503154	0.016230028	0.735074391
pcaac321.pcaac383	0.002293637	0.006767771	0.735199538
pcaac402.pcaec425	-0.001787388	0.005274484	0.735223187
lysopcac180.pcaac380	0.021185022	0.062518543	0.735234154
smc160.valine	0.017595307	0.051930391	0.735260442
smohc221.pcaac323	0.032682834	0.09648801	0.735336021
c18.smc241	-0.004433915	0.013090395	0.735342894
pcaec402.alanine	-0.003312063	0.009780117	0.735389817
smc241.pcaac344	-0.023145241	0.068366955	0.735471356
smc260.asparticacid	-0.012825989	0.037906244	0.735609255
pcaac402.threonine	-0.006442106	0.019041416	0.735639418
smohc221.pcaac322	0.017638265	0.052156775	0.735746863
pcaec384.c3	-0.004858348	0.014366737	0.735755426
pcaac321.lysopcac161	0.02051624	0.060682575	0.735812055
pcaac364.c101	-0.001052516	0.00311374	0.735863131
pcaac421.putrescine	-0.010530387	0.031162691	0.735943363
pcaec404.pcaac322	0.023832921	0.07054113	0.735986968
pcaac426.pcaec421	0.000671724	0.001988792	0.736064944
c182.c141	0.007838244	0.023210424	0.736103437
smc161.pcaec343	0.021944991	0.064984778	0.736110423
lysopcac160.lysopcac204	0.018937475	0.056084602	0.736136929
glutamine.pcaac406	0.009955452	0.029489121	0.736183274
methionine.arginine	0.008990483	0.026635124	0.7362247
histidine.glutamine	0.011587379	0.034329821	0.736233247
lysopcac161.acetylornithoine	-0.011299843	0.033485147	0.736287881
kynurenine.smohc241	0.017284576	0.051220199	0.736289466
smc161.glycine	-0.014979115	0.044399893	0.736355622
glutamicacid.threonine	0.009317094	0.027621327	0.736395532
putrescine.histidine	0.016635125	0.049317902	0.736403969

pcaac343.h1	0.000404739	0.001200228	0.73646827
pcaac401.methioninesulfoxide	0.00804934	0.023870409	0.7364747
acetylornithoine.ornithine	-0.007619317	0.022605362	0.736589029
pcaec301.pcaec385	0.014139665	0.041953717	0.736609992
pcaac401.pcaec403	-0.011988621	0.035571585	0.736611528
pcaec361.asymmetricdimethylarginir	0.008829568	0.026202576	0.736652702
pcaac300.prolinr	-0.008451652	0.025083685	0.736679078
smc240.pcaec401	-0.009009199	0.026741986	0.736712777
c162.methioninesulfoxide	0.003768698	0.011188124	0.736746889
asparagine.lysine	-0.022236581	0.066037594	0.736838645
smc261.alphaaminoadipicacid	-0.001363902	0.004051681	0.736913789
pcaec423.pcaec385	-0.013546974	0.040256076	0.736993701
smohc141.pcaac363	0.001426087	0.004238429	0.737034419
smc261.phenylalanine	-0.0094397	0.028059202	0.737068282
pcaec381.smohc241	-0.015774096	0.046894736	0.737104711
lysopcac160.pcaac380	0.027813658	0.082688775	0.737109859
glutamicacid.smc180	-0.004235535	0.012593153	0.737131876
pcaec301.alphaaminoadipicacid	-0.00130343	0.003876068	0.737177029
pcaac383.pcaac405	-0.017587407	0.052301087	0.737180038
pcaec386.lysopcac181	0.006170599	0.018356299	0.737266762
pcaec405.pcaec425	-0.001752597	0.005214525	0.737310352
pcaec362.asymmetricdimethylarginir	0.005189525	0.015449401	0.737456707
pcaac424.lysopcac180	-0.024689495	0.073509053	0.737482633
pcaec422.pcaac384	0.01126268	0.033532857	0.737482748
c3.lysopcac181	-0.011082132	0.03299664	0.737492965
pcaac404.smc260	-0.006738381	0.020067493	0.737546027
c142.phenylalanine	-0.009178849	0.027337834	0.737568306
pcaec403.pcaac364	0.011795874	0.035151098	0.737703763
pcaac240.smc161	0.027449832	0.081801981	0.7377127
pcaac425.serotonin	-0.023757608	0.070803132	0.737727595
pcaec443.pcaec401	-0.025121927	0.074875378	0.737748718
c162.h1	0.000855022	0.002548672	0.737778115
pcaec300.pcaac322	0.01882965	0.056137655	0.737821931
pcaec380.lysine	0.02790957	0.083219182	0.737855798
pcaec363.pcaec425	-0.000789306	0.002353836	0.737891126
smc261.pcaac426	-0.012534702	0.037383259	0.73790976
pcaac402.pcaec405	0.028945138	0.086340156	0.737952784
pcaac344.pcaac360	-0.011654842	0.034767242	0.737968254
pcaec386.pcaec380	0.018014657	0.053739409	0.737970028
smohc161.pcaec405	-0.007370022	0.021987854	0.737997425
c141oh.pcaec401	-0.015762884	0.047034326	0.738035293
lysopcac140.pcaac383	-0.003301299	0.009853754	0.738115234
c182.pcaac361	-0.016783088	0.050097514	0.738131138
pcaec443.pcaec446	0.02065674	0.061670667	0.738173269
smc180.smohc161	0.006135658	0.018320059	0.738201646

pcaec363.isoleucine	0.0051011	0.015232046	0.738218284
c5.pcaac405	0.039236786	0.117188765	0.738275243
pcaac420.alphaaminoadipicacid	-0.000965896	0.002886469	0.738416857
smc202.ornithine	0.008021942	0.023973577	0.738426523
pcaec322.asymmetricdimethylarginir	-0.012807927	0.038308554	0.738637331
leucine.alphaaminoadipicacid	-0.001134281	0.003393916	0.738732176
pcaac406.pcaac322	0.016492789	0.049350643	0.73874268
pcaec381.smohc141	-0.01039588	0.031110131	0.738766929
c2.pcaec301	-0.017265893	0.051672457	0.738784022
pcaec406.pcaec444	0.036720205	0.109913083	0.73882701
pcaec403.lysopcac281	0.004990274	0.01493933	0.738863196
pcaec443.pcaec425	-0.001484727	0.004445426	0.738897893
c16.c10	0.011499065	0.034436068	0.738946734
lysopcac161.pcaec444	0.036060484	0.108024358	0.73902725
smc260.smohc241	0.014146841	0.042382642	0.739049477
pcaec444.lysopcac140	-0.000383099	0.001147789	0.739062511
c162.lysopcac161	-0.024527745	0.07351033	0.739143327
h1.lysopcac281	-0.003615524	0.010836215	0.739152404
pcaac320.smc181	0.003250406	0.009742838	0.739176392
pcaec321.pcaec322	-0.041675216	0.12492874	0.739197563
lysopcac170.glutamicacid	-0.010132279	0.030383828	0.739284811
pcaac240.pcaac344	0.02059931	0.061781716	0.739326399
methionine.smc161	0.011571186	0.034706131	0.739338576
pcaec340.lysopcac203	0.003092277	0.009275112	0.739345754
c141.pcaec420	0.005232336	0.015695193	0.739363255
pcaec444.pcaac383	0.001033677	0.003101265	0.739411253
pcaac384.pcaac383	-0.000906062	0.00271853	0.739424089
acetylornithoine.smc202	-0.0149982	0.045002987	0.739439066
pcaec361.c182	0.010068518	0.030215329	0.7394735
lysopcac203.pcaac240	-0.003832519	0.011502048	0.739490308
smc202.pcaec363	-0.005373928	0.016128422	0.739495586
glutamicacid.lysopcac280	-0.002245218	0.006738461	0.739496889
pcaac425.pcaec343	0.018943611	0.056855301	0.739500312
lysopcac180.pcaac360	-0.007985578	0.023967955	0.739509819
pcaac380.lysopcac181	0.00691193	0.020747821	0.739537861
pcaac380.c3	0.007534408	0.022621796	0.739598375
pcaac426.lysopcac160	-0.019687835	0.059120991	0.739636383
c182.creatinine	0.00434396	0.013046167	0.739667233
smc161.c4	-0.005312584	0.015957679	0.739705787
c141oh.pcaac364	0.009700705	0.02913898	0.739709912
pcaac341.lysopcac182	-0.004450145	0.013368106	0.739724124
spermidine.h1	0.002907515	0.008736019	0.739779482
glycine.pcaac342	0.001856534	0.005578209	0.739779612
pcaec362.c162	-0.024743977	0.074355437	0.739809295
methioninesulfoxide.pcaac366	-0.007261958	0.021822706	0.739816

arginine.pcaac420	0.021851913	0.065668582	0.739823699
pcaac422.c14	-0.0129844	0.039030686	0.739890698
c3.smc180	-0.004289099	0.012893188	0.739896432
pcaec403.c102	-0.010807655	0.032493018	0.739933576
pcaac426.pcaec446	-0.020567195	0.061867811	0.740066865
alanine.pcaac344	0.015482391	0.046578033	0.740097678
pcaac380.c51	-0.015093554	0.045419472	0.740159612
c0.pcaac341	-0.000961975	0.002896264	0.740288605
h1.pcaac404	-0.002255795	0.006794095	0.74037943
pcaec404.threonine	-0.008574677	0.025827269	0.740395961
pcaec340.methioninesulfoxide	0.004151318	0.012509346	0.740504173
c0.pcaac403	-0.001778569	0.005361015	0.740577672
putrescine.pcaac420	-0.020937541	0.063111889	0.740582964
creatinine.pcaac364	-0.016434235	0.049563207	0.740712087
c10.pcaac366	0.003502492	0.010563019	0.740712703
pcaec446.pcaec425	-0.003358199	0.0101347	0.740881529
lysopcac170.pcaec425	0.00451316	0.013624827	0.740965299
c8.threonine	0.006504606	0.019640032	0.741006088
pcaec364.pcaec446	-0.016754564	0.050599187	0.741057374
smohc141.pcaec363	-0.020569821	0.062122132	0.741060483
pcaac422.pcaec363	0.00677591	0.020467957	0.74111279
pcaac320.methioninesulfoxide	0.004633575	0.01399701	0.741119809
pcaac385.pcaec445	0.013403858	0.04049928	0.741176336
pcaac360.pcaec425	-0.001043281	0.00315228	0.741179835
pcaac341.pcaac320	0.043181985	0.130506075	0.741239873
pcaac424.smc161	0.027927071	0.084426209	0.741310944
c14.pcaac342	0.000919098	0.002779492	0.741398069
pcaec402.c12	0.012673015	0.03833056	0.741433378
pcaec342.pcaac426	-0.00982702	0.02974145	0.741591184
pcaac421.pcaec424	0.014331344	0.043375761	0.74160259
pcaec380.pcaac425	-0.034255296	0.103690706	0.741632373
pcaec364.lysopcac182	0.007642384	0.023133699	0.741634746
glycine.kynurenine	-0.003513341	0.010635353	0.741643543
lysopcac280.glycine	0.006232125	0.01886548	0.741643609
pcaec420.pcaac366	0.00632728	0.019154636	0.741658019
c16.smc261	0.004077752	0.012346969	0.741705451
asparagine.lysopcac182	-0.007848607	0.023765913	0.741718277
creatinine.pcaac422	-0.006605235	0.020004558	0.741763423
pcaec361.pcaec421	0.000763939	0.00231367	0.741764677
smohc222.c51	-0.009084465	0.027514705	0.741777632
pcaac422.pcaac383	0.003121105	0.009454709	0.741820181
pcaec381.c0	0.000326754	0.000989886	0.741834283
lysine.smc160	-0.019391115	0.05874823	0.741850134
pcaac362.pcaac360	-0.007124063	0.021592836	0.741958947
smc240.glutamicacid	-0.006789083	0.020578186	0.741966994

pcaac341.pcaec341	0.030557331	0.092632961	0.741997999
pcaac320.pcaec422	0.006567912	0.019913608	0.742039518
smc260.pcaac323	0.019967562	0.060556835	0.742105649
c12.lysopcac182	-0.010201669	0.030941004	0.74211999
lysine.pcaac422	0.007631413	0.023148038	0.742146338
smc261.pcaec362	0.005453354	0.016549395	0.742266045
c141oh.pcaac380	0.016500581	0.050076483	0.742275305
c8.c12	0.027653808	0.083940237	0.742321447
pcaac405.pcaec422	0.003752826	0.011391846	0.742333104
pcaac422.alanine	-0.004907564	0.014897731	0.742343716
pcaec403.pcaac344	0.024492711	0.074358496	0.742366352
pcaec425.lysopcac161	0.018822157	0.057146981	0.742383602
pcaac424.lysine	-0.026275299	0.079778934	0.742393121
pcaac420.pcaec402	-0.016073656	0.048810095	0.742424242
lysopcac180.methionine	-0.008101654	0.024604307	0.742448529
pcaac322.pcaec444	-0.02282894	0.069342278	0.742491358
pcaec445.h1	-0.001950231	0.005923808	0.742492844
pcaec406.putrescine	0.012270477	0.037273955	0.742509466
pcaec382.histidine	-0.02276401	0.069150765	0.742511859
lysopcac161.pcaac384	0.018447533	0.056041747	0.742526235
asymmetricdimethylarginine.pcaec38	-0.013681757	0.041564402	0.742529825
pcaec446.c51	-0.013433527	0.040818678	0.742580812
c142.lysopcac182	0.011298013	0.034343144	0.742677457
pcaec402.pcaac363	0.002450411	0.007451865	0.74278481
lysopcac140.pcaac342	-0.000624095	0.001898207	0.74282294
pcaec322.pcaac385	-0.006145794	0.018694805	0.742851612
pcaac421.pcaac361	-0.017951643	0.054631131	0.742961807
pcaac402.lysopcac203	0.006408195	0.019508106	0.743043642
c101.pcaec423	-0.003670042	0.011174054	0.743078092
smc261.pcaac383	-0.001250201	0.003807073	0.743118838
lysopcac170.pcaec405	-0.010155959	0.030930246	0.743148066
c142.pcaac363	0.001983951	0.006043421	0.743199101
c142.c141	-0.014503949	0.044181529	0.743200552
pcaac421.methioninesulfoxide	0.005828086	0.017755531	0.74323092
pcaec343.pcaec386	0.002803202	0.008541179	0.743262643
c141.alanine	-0.006276804	0.019129455	0.74332002
pcaec360.pcaec420	0.006336782	0.019315239	0.743358367
asparagine.pcaac404	-0.002164823	0.006598917	0.743369162
lysopcac281.c142	0.011539529	0.035190482	0.743475623
pcaac342.pcaec402	-0.006766335	0.020640008	0.743543558
pcaac365.pcaec425	-0.001133282	0.003457205	0.743561201
pcaec443.pcaac344	0.015713049	0.047941178	0.743595861
c5.pcaac363	-0.005141007	0.015686634	0.743614837
pcaac362.c51	-0.019179973	0.058524817	0.743620816
pcaec421.pcaec365	-0.006340854	0.019348216	0.743621441

pcaec444.lysopcac281	0.003248761	0.009915862	0.743689465
kynurenine.pcaac360	0.008347153	0.02549214	0.743834744
histidine.smohc241	-0.026571265	0.081190811	0.743963695
c0.pcaac281	-0.000360786	0.001102579	0.744000724
lysopcac281.pcaac363	0.002427759	0.00741963	0.744010348
c141oh.pcaec446	-0.007164225	0.021896931	0.744031594
pcaac366.pcaac321	0.005783905	0.017680314	0.744062729
pcaac385.pcaac420	-0.017494953	0.053488772	0.744108777
pcaac361.pcaec425	-0.000729858	0.002232653	0.744241259
alphaaminoadipicacid.tyrosine	-0.01764845	0.053988448	0.744247659
smohc141.c18	-0.008121266	0.024843944	0.744249112
pcaec301.putrescine	-0.008625088	0.026397773	0.744366632
pcaac362.methionine	-0.009420557	0.028843319	0.744460255
c182.pcaac363	0.002443975	0.007483409	0.744479592
methioninesulfoxide.smc160	-0.009362438	0.02866963	0.744496863
smohc221.glutamicacid	0.006682382	0.020465248	0.744526679
smohc141.pcaec446	-0.006953894	0.021297538	0.744535559
pcaec384.pcaac363	0.00172983	0.005297995	0.744539391
pcaac380.glutamicacid	0.011905353	0.036465668	0.744558585
histidine.pcaec405	0.012819566	0.039267775	0.744570542
kynurenine.smc241	0.002596497	0.007955468	0.744635703
c16.pcaac323	0.031495038	0.096528162	0.744711741
pcaac385.tyrosine	0.016654115	0.051046853	0.74473188
c14.pcaac360	-0.007661373	0.023483057	0.744732239
pcaec321.putrescine	0.009821187	0.030104762	0.744745373
pcaec386.serotonin	0.028479207	0.087316485	0.744800458
glutamine.c102	0.010651067	0.032657929	0.744815872
ornithine.pcaac420	0.026756531	0.082076961	0.744926914
c10.prolinr	-0.013236712	0.040609402	0.744958095
smohc241.pcaac386	0.005442756	0.016698776	0.744969003
pcaec425.asparagine	0.004590148	0.014082926	0.744969272
pcaec403.pcaec386	-0.003172064	0.009735024	0.745042327
pcaac323.smc181	-0.004999855	0.015345554	0.745059322
isoleucine.ornithine	-0.011362792	0.034877383	0.745078392
pcaac281.c182	0.010222378	0.031378838	0.745093172
pcaac385.pcaec382	0.001709279	0.005249545	0.745219929
glutamine.c51	0.012419105	0.038151498	0.745283605
pcaac360.lysopcac170	-0.015691448	0.048206983	0.745298111
c18.pcaac424	0.002192043	0.006734475	0.745302502
pcaec444.pcaac386	-0.008657682	0.026606438	0.745376297
asparagine.pcaac403	-0.000555537	0.001707433	0.745402101
pcaec386.pcaec341	0.022123187	0.068010562	0.745457507
smc240.smc160	-0.022032413	0.067734919	0.745469874
pcaac424.pcaac342	-0.001816619	0.005585948	0.745516602
pcaec384.valine	-0.018465263	0.056787807	0.745554152

pcaac281.pcaac385	-0.016607369	0.051074459	0.745556045
pcaac420.pcaac360	-0.011765653	0.036187067	0.745575492
c8.c142	0.016848044	0.051826256	0.745611109
c102.smc160	-0.02505167	0.077068783	0.745634554
pcaac360.asparagine	-0.002426932	0.007468712	0.74571726
c3.pcaac383	-0.001451982	0.004468924	0.745747461
pcaac366.lysopcac160	0.004806978	0.014796052	0.745765592
pcaec424.pcaec423	0.013463465	0.041445278	0.745790741
arginine.ornithine	0.008620303	0.026542491	0.745847757
c181.pcaac322	-0.01821733	0.056103216	0.745895112
pcaec446.pcaac425	-0.022195736	0.068360923	0.745915067
pcaac403.transhydroxyproline	-0.017539202	0.054020733	0.745922
c142.pcaec405	-0.009228262	0.028423762	0.745928228
smc241.pcaec340	0.018082896	0.055728214	0.746066787
pcaac364.methioninesulfoxide	0.003588273	0.011058861	0.746076815
pcaac421.alphaaminoadipicacid	-0.001573585	0.004851069	0.74614583
smohc241.pcaac300	0.016914428	0.052150329	0.746175453
c141oh.pcaac425	0.01421498	0.043828081	0.746179115
pcaec423.pcaec386	-0.002922406	0.009011675	0.746212256
pcaac426.pcaac364	-0.019495462	0.060129956	0.746264356
pcaec420.acetylornithoine	-0.02136017	0.065909692	0.746369919
pcaac322.pcaac342	-0.000945761	0.002918404	0.746380905
putrescine.methioninesulfoxide	-0.005427556	0.016751254	0.746425409
pcaac381.pcaec365	-0.00548672	0.016933949	0.746426795
smc181.pcaec342	0.024319311	0.075078742	0.746494551
c5.c10	-0.011779357	0.036366711	0.746503959
pcaac360.pcaec386	0.003734991	0.011532737	0.746537971
pcaac320.pcaec445	0.013885125	0.042881255	0.746580162
c51dc.pcaac424	0.002837609	0.00876374	0.746591024
pcaec425.pcaec322	0.015641163	0.048332286	0.746721284
pcaac421.spermidine	0.016824503	0.051993204	0.746741582
c12.c182	-0.016884532	0.052191274	0.74680041
lysopcac280.kynurenine	-0.009262013	0.028644079	0.746924548
pcaec421.asparagine	0.005546664	0.017154086	0.746928055
pcaac426.pcaac342	-0.001064572	0.00329279	0.746958015
pcaac401.smc160	-0.018216289	0.056353392	0.746998046
pcaac402.pcaec445	0.021340963	0.066050654	0.747112177
pcaec341.pcaec420	0.007050867	0.021829513	0.747189985
pcaac364.pcaac362	0.001864429	0.005772356	0.747193212
c142.tyrosine	0.015621734	0.048365969	0.747195112
methioninesulfoxide.pcaac381	0.015414754	0.047727262	0.74720593
pcaac366.smc260	-0.006872374	0.021280035	0.747225954
pcaac360.methionine	-0.012743261	0.039460064	0.747232506
c10.pcaac380	0.013371486	0.041407	0.747242002
pcaac386.asparagine	-0.003222648	0.009980316	0.747262984

smc161.pcaac383	-0.000784715	0.002430257	0.747267992
lysopcac180.taurine	-0.007468269	0.023132082	0.747298573
c102.pcaec444	0.034613807	0.107235582	0.7473518
alphaaminoadipicacid.pcaec362	0.003872277	0.011997865	0.747378798
pcaec385.c101	-0.002866765	0.00888426	0.747430228
c101.lysopcac281	-0.002312736	0.007168554	0.747473116
pcaac380.pcaec421	-0.000507472	0.001573525	0.747560881
c14.asparagine	-0.003739959	0.011596765	0.747565424
ornithine.lysopcac161	-0.024971837	0.077464916	0.747668986
pcaec405.c2	0.021373899	0.066313742	0.74770557
smohc141.lysopcac204	-0.006690544	0.020761674	0.747751102
pcaac385.threonine	0.008263524	0.025646535	0.747786139
h1.pcaac360	0.011014331	0.034187453	0.747811475
pcaec322.putrescine	0.007586101	0.023557826	0.747928069
pcaec424.smohc141	-0.006451476	0.020034898	0.747934481
methioninesulfoxide.pcaec423	0.010230791	0.031773916	0.747953264
alanine.glutamicacid	0.013676376	0.042478575	0.74797421
asparagine.smohc141	-0.005266845	0.016364573	0.748061166
pcaec445.pcaec343	-0.019281978	0.059921447	0.748104074
pcaec365.pcaec386	0.002488315	0.007733114	0.748114332
c2.c182	-0.007897448	0.024544214	0.748121802
smohc221.pcaec383	-0.014999586	0.046620496	0.748141543
smc202.asparticacid	0.008936028	0.027775627	0.748153708
methioninesulfoxide.lysopcac203	0.001993666	0.006201009	0.748316417
prolinr.pcaec343	0.024213836	0.075342099	0.74840829
c162.pcaac341	0.001612648	0.005018619	0.748447785
smc160.pcaec340	0.022725898	0.070731079	0.748472685
smohc221.pcaec402	-0.020755391	0.064598857	0.748475306
pcaac406.acetylornithoine	-0.006996607	0.021779355	0.74851088
smc181.asymmetricdimethylarginine	0.004078433	0.012697212	0.748543074
lysopcac204.methioninesulfoxide	0.01050485	0.032715235	0.74862421
c51.pcaec425	-0.001574618	0.00490427	0.748645908
methionine.pcaec421	0.001167164	0.003636051	0.748701319
pcaec380.pcaec425	-0.000954045	0.002972824	0.748758321
pcaec445.pcaec420	0.00753365	0.023480589	0.748816106
smc240.pcaec343	0.01817606	0.05666136	0.748862854
asparagine.spermidine	-0.01641753	0.051188567	0.748906368
serine.ornithine	0.012140066	0.03787164	0.749033626
pcaec386.pcaac425	-0.028587181	0.089277649	0.749300514
pcaec362.smc180	0.002223467	0.006944583	0.749325118
c8.c51	-0.022077846	0.068963146	0.749350109
lysopcac203.pcaec385	0.017257493	0.053918037	0.749403631
lysopcac204.pcaac384	0.012666153	0.039578124	0.7494338
c10.alanine	0.00891745	0.027868741	0.749470696
smc261.pcaac366	-0.005951626	0.018602448	0.749502886

isoleucine.pcaec343	0.021701702	0.067845044	0.749552972
glycine.lysopcac281	0.002359837	0.007378303	0.74958086
smohc161.alphaaminoadipicacid	-0.002124232	0.006641836	0.749587493
pcaec364.pcaec422	0.011051322	0.034555125	0.749594151
pcaec420.pcaec342	-0.022851057	0.071477843	0.7496871
pcaec365.pcaac426	0.013750221	0.043012977	0.749700839
c101.pcaac420	-0.018207689	0.056960254	0.749716024
glutamine.pcaac300	0.022036882	0.068974774	0.749840193
pcaec423.kynurenine	-0.001929759	0.00604181	0.749909037
pcaec421.c18	-0.014115984	0.044203511	0.749954515
c141oh.pcaec420	0.005875438	0.018400658	0.749980938
pcaac342.methioninesulfoxide	0.003135101	0.00981892	0.749991572
pcaec384.pcaac362	0.00146343	0.004585606	0.750109802
pcaec420.pcaec425	-0.000665398	0.002085465	0.750163388
spermidine.c10	-0.006575047	0.020615355	0.750258302
pcaac403.c8	0.014877202	0.046652288	0.750291475
pcaec443.serine	0.017680028	0.055449772	0.750327672
pcaec342.valine	0.011978039	0.037576997	0.750394072
c102.lysopcac161	-0.013562937	0.04256691	0.75049514
pcaec322.pcaac321	-0.009190698	0.028848011	0.750522347
pcaac363.pcaec342	0.033058405	0.103778761	0.750555217
pcaec404.c3	-0.009053324	0.028421661	0.750563431
pcaec382.pcaec365	-0.005101183	0.016016298	0.750591106
c182.methioninesulfoxide	0.003030843	0.009518786	0.750661475
serine.pcaec384	-0.005665747	0.017795393	0.750679409
smc180.phenylalanine	-0.011829372	0.03716028	0.750716531
c10.pcaec405	-0.002969073	0.009329712	0.750788633
pcaac322.pcaac401	-0.007408199	0.023282127	0.750823417
pcaec401.alanine	-0.003580715	0.011254602	0.750851379
pcaac422.c102	-0.009461162	0.029744249	0.750905771
pcaec403.pcaac424	0.006392021	0.020100031	0.750961196
c101.pcaec363	0.003620474	0.0113863	0.750993692
lysine.glutamicacid	0.01049339	0.033004147	0.751013392
pcaec383.smc240	0.01240607	0.03902628	0.751052297
pcaac426.smc202	-0.009880904	0.031094198	0.751140782
alphaaminoadipicacid.pcaec403	-0.010214715	0.032145796	0.751149228
pcaec446.pcaec383	-0.015114268	0.047575783	0.751205114
smohc241.phenylalanine	-0.015574318	0.049025068	0.751210836
c4.pcaec385	-0.024969289	0.078606724	0.75123539
pcaac300.lysopcac203	0.003941972	0.012410077	0.751239516
pcaac426.lysopcac170	0.004556833	0.014348111	0.751278624
pcaec406.pcaec301	0.020972576	0.0660391	0.751288453
c3.c102	-0.005360309	0.016878847	0.751290439
pcaac420.pcaac322	0.01480848	0.046639608	0.75134105
smohc161.pcaac363	0.001341302	0.004226232	0.751442012

pcaec383.c141	-0.010050224	0.031667886	0.75145113
pcaac403.pcaac420	0.036242807	0.11423162	0.751518155
serotonin.pcaac322	0.016951374	0.053441904	0.751580378
pcaac401.pcaac320	-0.019078652	0.060156085	0.751610708
smc261.pcaec384	-0.017176825	0.05417937	0.751698704
smohc222.c12	0.013725395	0.043301333	0.75174588
smohc141.pcaec386	0.005302369	0.016729047	0.751759621
pcaec365.pcaec421	0.000232137	0.000732606	0.751828162
acetylornithoine.smohc141	0.006066172	0.019145029	0.751836979
lysopcac280.c3	-0.010777991	0.034017462	0.751849567
smc161.ornithine	-0.011678836	0.03686581	0.751882774
lysopcac180.pcaac342	-0.001354826	0.004278759	0.751998695
c0.methionine	-0.007703217	0.024337404	0.752091442
phenylalanine.c4	0.004663964	0.014739727	0.752164346
lysopcac281.alphaaminoadipicacid	-0.000734987	0.002322957	0.752179351
pcaac383.pcaac321	-0.007905155	0.02498492	0.752182721
lysopcac181.smc161	-0.023631991	0.074691848	0.752185659
pcaac383.serotonin	0.021262267	0.067206222	0.752200567
asymmetricdimethylarginine.lysopca	-0.002906307	0.009188411	0.752255152
pcaac402.smohc222	-0.006254507	0.019774847	0.752266827
smc260.taurine	0.012295142	0.038873949	0.752269615
pcaac343.pcaac321	-0.008900158	0.02814246	0.752291254
c101.pcaec362	0.002841497	0.008987208	0.752353698
pcaac361.threonine	0.00751516	0.023772765	0.752388931
smc180.pcaec420	0.004161001	0.013162825	0.752394342
smohc241.c8	-0.011264768	0.035636978	0.752409404
pcaac386.lysopcac204	-0.010481718	0.033161927	0.752425211
asparticacid.c142	0.010115621	0.032007675	0.752455138
pcaac426.transhydroxyproline	0.007023659	0.022224256	0.752456354
methionine.acetylornithoine	0.008372656	0.026494206	0.752469509
c142.pcaac344	-0.011071581	0.03503786	0.752491686
pcaec301.c182	0.010705895	0.03388091	0.752493976
pcaac365.pcaac366	-0.032541659	0.102988762	0.752503937
pcaac406.taurine	0.010893661	0.034504419	0.752697115
pcaec420.lysopcac281	0.001356226	0.004296153	0.752722809
smohc141.pcaac406	-0.009231981	0.029249889	0.752767839
pcaac405.smohc141	-0.003322252	0.010530048	0.752860605
pcaac404.acetylornithoine	0.001728452	0.005479268	0.752897693
spermidine.c2	-0.073121894	0.231838929	0.752938235
pcaac343.pcaac403	-0.004546754	0.01441854	0.752982755
pcaac321.pcaac344	-0.021993216	0.069759653	0.753035395
arginine.c4	-0.012632372	0.040078495	0.753096383
c14.threonine	0.005718456	0.018147386	0.753156196
pcaec343.pcaec421	0.000651241	0.002066769	0.753164417
c5.smc160	-0.035913851	0.113995466	0.753205836

pcaec360.pcaec363	-0.008143523	0.025852335	0.753239809
smohc241.pcaac361	0.014692149	0.046641894	0.753241704
pcaec301.phenylalanine	0.005464987	0.01734971	0.753248411
c12.valine	-0.013369962	0.04244786	0.753260811
c0.pcaac342	0.008519752	0.027056144	0.753323061
pcaac363.alanine	-0.005789675	0.018386752	0.75332958
pcaac383.pcaec300	0.00537516	0.017074022	0.753381012
c51dc.pcaec365	-0.006400055	0.020330014	0.753386223
pcaac300.isoleucine	-0.007178108	0.022805313	0.75342582
creatinine.taurine	0.012041943	0.038266423	0.753478118
pcaac366.pcaec380	-0.029867065	0.094930106	0.753527598
pcaac300.pcaac384	-0.023058235	0.073297446	0.753555794
pcaec361.leucine	-0.017224783	0.054762069	0.753590539
c182.pcaac360	0.005916802	0.018824537	0.753761261
pcaec405.transhydroxyproline	-0.022231681	0.070738316	0.75378601
ornithine.smc260	0.006149036	0.019567268	0.753808354
pcaac404.c141oh	0.033357856	0.106154824	0.75381847
putrescine.smohc141	0.002266981	0.007220416	0.754022779
pcaec342.pcaac361	0.023518634	0.074929135	0.754090848
alanine.pcaac402	0.013986143	0.044561667	0.754104459
smohc222.taurine	0.007063596	0.02251725	0.754228215
pcaac403.lysopcac180	-0.018633242	0.059408275	0.75426602
smohc241.threonine	0.006151583	0.019613343	0.754269406
c4.taurine	-0.021153571	0.067453726	0.754300896
pcaec342.pcaec406	0.001487283	0.004742942	0.754318359
c0.smc260	0.007874165	0.025114368	0.754353159
pcaac341.pcaec425	-0.000917409	0.002926666	0.754403592
pcaac402.glycine	-0.004367433	0.013933902	0.754423587
c162.pcaec342	0.015005511	0.047882114	0.754465192
pcaac362.ornithine	0.009303723	0.029687907	0.754465345
h1.pcaec340	-0.021015485	0.067063409	0.754478143
lysopcac180.smc241	0.009750851	0.031119684	0.754503555
pcaac321.pcaac380	-0.011888131	0.037942813	0.754516277
serotonin.c51dc	0.028643584	0.091428489	0.754537194
c101.c2	-0.013449457	0.042945266	0.754622693
pcaac320.c4	-0.004109497	0.013122457	0.754631387
pcaec385.pcaec363	0.00961351	0.030701899	0.754662436
pcaec361.smc180	0.001021248	0.003262198	0.754714783
pcaec341.methioninesulfoxide	0.004040838	0.012908547	0.754729484
lysopcac180.smc181	-0.00314976	0.010062895	0.754751047
pcaec341.asymmetricdimethylarginir	0.008324644	0.026597131	0.754764004
pcaac363.taurine	0.007265925	0.023215668	0.754775561
smc180.pcaac321	0.006464451	0.020661333	0.75485008
pcaec406.pcaac281	0.000469149	0.001500077	0.754946202
serotonin.putrescine	-0.007460581	0.023859832	0.754996419

kynurenine.smc261	0.009585586	0.03065635	0.755000281
pcaec321.threonine	0.008776429	0.02807072	0.755018796
smc180.pcaec402	-0.014473381	0.046293285	0.755025499
pcaec363.pcaac383	0.001836415	0.00587693	0.755151949
pcaac360.pcaac422	0.011633732	0.03723082	0.755154036
smohc221.lysopcac180	0.012586478	0.040283228	0.755173944
pcaac420.valine	-0.018926278	0.06057549	0.755180309
smc181.serotonin	0.020786986	0.066545167	0.755231197
c181.smc161	-0.017470438	0.055937479	0.7552716
lysopcac281.spermidine	-0.016604783	0.053166356	0.755274105
pcaac322.smohc241	-0.009141782	0.02927331	0.755294443
pcaec322.pcaac360	0.015958445	0.051102056	0.75529816
histidine.valine	-0.01771267	0.056737954	0.755375486
pcaac344.pcaec341	-0.02845299	0.09114712	0.755389328
serine.pcaac300	-0.01872619	0.060000615	0.755439048
pcaec422.pcaec425	-0.001162503	0.003728083	0.755648958
pcaac300.h1	0.00046548	0.001492868	0.755664468
pcaec300.pcaac360	-0.006143611	0.019704516	0.755676437
pcaac405.pcaac421	0.013069298	0.041917795	0.755678666
pcaec423.pcaac425	0.028084874	0.090112692	0.755769742
lysopcac280.smc260	-0.006655963	0.021361683	0.755830187
serotonin.c5	0.004432778	0.014229685	0.755881731
c14.pcaac363	0.001439007	0.004620303	0.75592969
smc241.pcaac424	-0.003612588	0.011609276	0.756135881
methionine.pcaec341	0.017666512	0.056795056	0.756229918
pcaac404.pcaac363	-0.002819414	0.009064432	0.756241896
pcaec361.c0	-0.000194721	0.000626274	0.756334659
asparticacid.pcaec446	-0.017979285	0.057842944	0.756402766
lysine.histidine	-0.030635732	0.098573707	0.756432548
methionine.pcaac342	-0.005417449	0.017433865	0.756468377
glycine.lysopcac140	0.000243579	0.000783958	0.756497508
pcaac386.c102	0.01000205	0.032195523	0.75652679
smohc241.smc202	-0.014743298	0.047463505	0.756558655
glutamicacid.lysopcac204	0.014526504	0.046782599	0.756644408
c5.pcaec365	-0.006604559	0.021272172	0.756668709
pcaac385.pcaac342	-0.001167166	0.00375977	0.75670158
alphaaminoadipicacid.smc260	-0.00515281	0.016601879	0.756747391
smc261.pcaac385	-0.003020736	0.009734772	0.756801569
c12.pcaec386	-0.000623248	0.002008562	0.756807691
histidine.pcaac366	-0.003688814	0.011892021	0.756886037
asymmetricdimethylarginine.alanine	0.006516127	0.021008067	0.756901042
pcaec364.c142	0.010252977	0.033056623	0.756907503
smc180.methionine	0.00519782	0.016764305	0.756991937
pcaec363.smc160	-0.031062888	0.100205502	0.757038203
pcaac361.acetylornithoine	0.005413846	0.017474088	0.757167561

c102.pcaec421	-0.001439108	0.004645656	0.757202796
pcaac361.pcaec420	0.010050795	0.03245099	0.757242758
pcaec382.pcaac384	0.013323048	0.043018611	0.757256426
pcaac384.taurine	0.005143878	0.016611654	0.757293969
c142.methioninesulfoxide	0.00393885	0.012723148	0.757349661
pcaac365.valine	-0.01703447	0.055027119	0.757362113
serine.pcaec405	0.013080971	0.042266869	0.757422672
pcaac404.pcaac385	-0.005464751	0.017663034	0.757495601
pcaac384.smohc141	-0.00996093	0.032198948	0.75752092
leucine.transhydroxyproline	0.010584105	0.034218233	0.757554249
pcaec364.pcaac322	0.014019119	0.045326733	0.757570594
pcaec381.putrescine	0.018726428	0.060547865	0.757576101
pcaac323.pcaec425	-0.000962639	0.003113846	0.757678653
serotonin.pcaac405	0.019593845	0.063397401	0.757742482
smohc221.ornithine	-0.010531874	0.034084063	0.757793211
pcaac366.lysine	0.026490104	0.085731146	0.757798205
alphaaminoadipicacid.pcaec365	-0.007480511	0.02421572	0.757858226
pcaac380.serotonin	0.029462959	0.095402826	0.757922339
pcaac384.isoleucine	-0.005929546	0.019200678	0.757927891
pcaec423.c4	-0.004247106	0.013754968	0.757966391
smohc141.pcaec362	-0.00396639	0.012848536	0.758015896
smc180.pcaec342	0.023758775	0.076984956	0.758082581
lysopcac180.lysopcac161	-0.015909114	0.051555473	0.758107905
pcaac240.pcaac342	-0.00169404	0.005489878	0.758113423
glutamine.taurine	-0.007878209	0.025533741	0.758139296
asymmetricdimethylarginine.pcaec42	-0.001906865	0.006180971	0.758166176
c10.c12	0.024912261	0.08076285	0.758199431
pcaec421.c2	0.028696129	0.093046278	0.758241053
c162.pcaec401	-0.011121282	0.036062493	0.758254655
smc241.pcaec301	0.01528268	0.049567917	0.758308681
pcaec341.lysopcac182	-0.004413381	0.014314811	0.758315746
pcaac365.pcaec404	-0.005392749	0.017495432	0.75836974
c2.pcaac424	-0.01095331	0.035536733	0.758379271
pcaec403.pcaac323	-0.020155863	0.065395591	0.75838732
pcaac385.phenylalanine	-0.010296393	0.033408865	0.758403252
pcaac405.pcaac385	-0.007405739	0.024033	0.75843718
pcaec401.pcaac383	-0.001951645	0.006334125	0.758462066
methionine.asymmetricdimethylargir	-0.004464727	0.014490495	0.758463412
pcaac362.pcaac300	-0.015033506	0.048797823	0.758491346
serotonin.smohc141	0.001669947	0.005421554	0.758534915
pcaac401.pcaac383	0.002364209	0.007678451	0.758624698
pcaac365.pcaac406	0.014895858	0.048380743	0.758635036
pcaac321.pcaec445	-0.006986482	0.022694023	0.758659755
pcaec421.pcaec404	0.014047113	0.045639268	0.758712859
lysopcac140.c16	-0.023565536	0.076576777	0.758749526

pcaac320.pcaac281	0.000859561	0.002794114	0.75882886
pcaec301.spermidine	0.015921735	0.051758003	0.758839566
asparagine.valine	-0.012559056	0.040834609	0.7588849
c4.pcaec386	-0.003357291	0.010916003	0.758886638
c5.smc181	-0.010293676	0.033471878	0.758905436
acetylornithoine.serotonin	0.014270245	0.046429668	0.759042287
c51.taurine	-0.007827855	0.025469838	0.759052633
pcaac424.c12	0.01794025	0.058399651	0.759159207
pcaec446.pcaac420	-0.04136219	0.134649881	0.759170323
pcaac424.pcaec420	0.008525556	0.027762472	0.759241748
pcaac360.c4	-0.003527282	0.011486425	0.759246756
pcaac386.pcaac363	0.003693041	0.012034429	0.759406008
c101.pcaac385	0.003701774	0.01206346	0.759417048
pcaac341.pcaec385	-0.013133536	0.042819976	0.759525862
pcaac342.pcaac403	-0.000829745	0.002705707	0.759564234
pcaec443.pcaec406	0.006808887	0.022203912	0.759573408
pcaec363.lysopcac182	0.011039441	0.036004976	0.75960669
c141.pcaac366	0.005486229	0.017898777	0.759678548
c141oh.pcaac323	0.021276634	0.069418646	0.759691284
pcaac363.pcaac344	0.0193018	0.063000691	0.759784703
smc161.pcaac403	0.001009223	0.003294193	0.759792417
pcaac383.c8	0.007928912	0.02588286	0.759812113
lysopcac280.pcaac344	0.014361053	0.046888396	0.759855208
pcaac364.pcaac381	-0.012304271	0.0401788	0.759888422
pcaac402.arginine	-0.020052458	0.065517643	0.760022076
histidine.smc160	-0.018286863	0.059754575	0.760044195
pcaec423.transhydroxyproline	-0.010011076	0.032735027	0.760204749
lysine.glutamine	0.006065571	0.01984577	0.760346111
pcaec425.pcaac384	0.016488688	0.053952341	0.760361059
c182.valine	-0.011941043	0.039081904	0.760419486
smc181.glutamine	-0.005404219	0.017689562	0.760446582
pcaac405.pcaec363	-0.007836364	0.025650906	0.760448702
leucine.c141oh	-0.0324736	0.106299715	0.760455939
prolinr.lysopcac280	-0.00224206	0.007339933	0.760479003
pcaec321.pcaec420	0.006852019	0.022431878	0.760480214
pcaec420.c141	-0.007478784	0.024484091	0.760483367
smc180.pcaec385	-0.013068772	0.042800275	0.760568228
pcaec425.lysopcac182	-0.010902966	0.035716142	0.760625981
pcaac300.serotonin	0.019869764	0.065107393	0.760688843
pcaec422.valine	-0.011146992	0.036537013	0.760762406
pcaec343.c16	0.01585523	0.051976462	0.760793798
pcaac342.asymmetricdimethylarginir	0.006350411	0.020818489	0.760800847
pcaac344.pcaec444	-0.021433317	0.070266349	0.760806525
h1.asymmetricdimethylarginine	-0.006687964	0.021931174	0.760865192
pcaac320.pcaac406	-0.011513295	0.037759408	0.760895931

pcaec365.valine	-0.021224983	0.069637897	0.760988098
pcaec386.pcaec406	-0.007395258	0.024268255	0.761034476
pcaec444.pcaec404	-0.004966118	0.016301537	0.761101821
pcaec406.pcaac383	0.001914119	0.006283594	0.761116594
lysopcac281.pcaac380	0.006238778	0.02048131	0.761126677
c162.pcaac343	0.008032566	0.026375811	0.761176441
c162.pcaac281	-0.00033427	0.00109765	0.761184326
pcaec402.pcaec363	0.011183391	0.036725417	0.761198677
pcaec361.pcaec405	-0.023156797	0.07604926	0.761211073
lysine.alphaaminoadipicacid	-0.001354528	0.004448555	0.761218774
lysopcac160.c4	0.00411147	0.01350524	0.761258358
pcaec363.pcaec406	0.001263808	0.004151443	0.761265195
asparticacid.pcaec362	0.004145224	0.013617268	0.761277917
pcaec361.glutamicacid	-0.013508373	0.044385347	0.761328337
pcaec322.pcaec422	-0.007640121	0.025105509	0.761345503
lysopcac203.pcaec341	0.012987222	0.042680713	0.761370322
pcaac343.pcaec363	0.008600056	0.028266469	0.761399548
pcaac401.c14	-0.013320615	0.043782309	0.76140173
alphaaminoadipicacid.pcaac364	-0.012355841	0.040618145	0.761440797
pcaac405.pcaac320	-0.02813151	0.092494701	0.761481289
kynurenine.c141	0.00751821	0.024724661	0.761530326
pcaec381.c181	-0.011060972	0.036380667	0.76156302
lysopcac161.c141oh	-0.033518008	0.110277525	0.761632971
pcaec361.transhydroxyproline	-0.012125253	0.039893829	0.761636214
pcaac380.pcaec386	-0.004601551	0.015142654	0.761680293
pcaec382.pcaec386	-0.00292605	0.009629994	0.76170504
pcaac363.pcaec340	0.016115972	0.053040871	0.761710285
lysopcac203.pcaec386	0.002099156	0.006909488	0.761735305
pcaac300.lysopcac161	0.019592263	0.06449014	0.761739306
pcaac343.asparticacid	0.010725784	0.035305162	0.761739549
serotonin.c142	0.011307563	0.03722316	0.761758185
glutamine.asymmetricdimethylargini	-0.002960832	0.009747394	0.761774463
smc260.pcaec420	0.003989839	0.01313607	0.761793215
histidine.pcaac361	-0.010179342	0.033518451	0.761822016
smc261.threonine	0.008775673	0.028903266	0.761876367
acetylornithoine.putrescine	0.00642354	0.021164286	0.761962826
pcaec360.c102	0.01574647	0.051900067	0.762045453
c101.pcaac424	0.001094132	0.003606562	0.762066034
pcaec444.pcaac343	0.003976822	0.013108822	0.762068178
pcaec362.pcaec406	0.001482272	0.004886313	0.762081997
smc260.lysopcac181	0.005842441	0.019262371	0.762114869
c51dc.c2	-0.017568961	0.057931524	0.762143269
c3.smohc241	-0.016681107	0.055028887	0.762247834
smc181.pcaac386	-0.002633416	0.008693309	0.762406866
c3.lysopcac281	-0.003556449	0.011741778	0.762434306

c18.smohc141	0.0054544	0.018009811	0.762458115
asparagine.lysopcac140	-0.005420431	0.017898835	0.762473358
lysopcac281.pcaec425	-0.00082851	0.002735985	0.762486607
pcaec380.pcaec385	-0.013022856	0.043005673	0.762488509
smc240.pcaac342	0.001990682	0.006575861	0.762558079
c141.pcaec425	-0.000981135	0.003242935	0.762695144
pcaec401.pcaec385	0.010921479	0.036101751	0.762714984
smc202.c0	-0.000265466	0.000877566	0.762727831
pcaec321.phenylalanine	-0.009469797	0.031305628	0.762733488
smc260.pcaac322	0.00692976	0.022909806	0.762744877
pcaac380.pcaac422	0.010797421	0.035698072	0.762756274
c51.pcaac361	0.020138259	0.066581448	0.762759749
glycine.pcaec343	-0.019030343	0.062933546	0.762814977
pcaec363.pcaac426	-0.009907386	0.032768061	0.762844661
phenylalanine.pcaec446	-0.029070658	0.096171042	0.762896456
pcaec424.pcaec365	0.006209528	0.02054231	0.762897115
smohc141.citruline	-0.010105054	0.03343464	0.76293275
arginine.smc240	0.009136385	0.030229865	0.762934748
pcaac361.c18	-0.011225173	0.037143215	0.762947804
c18.lysopcac182	-0.004751524	0.015723512	0.762963768
lysopcac170.glycine	-0.008208846	0.027166506	0.762982311
c182.pcaac323	-0.019678232	0.065147337	0.763066409
pcaac300.c182	0.011119421	0.036820205	0.763115846
pcaac320.asparticacid	0.014370059	0.047585145	0.763120502
pcaac384.threonine	0.006973972	0.02309372	0.763120953
pcaec300.c142	0.011920458	0.039477447	0.763143389
pcaec424.pcaac320	-0.009215673	0.030523635	0.763171465
smohc222.lysopcac203	-0.003752711	0.012433926	0.763252906
c16.pcaec301	-0.012584303	0.04171267	0.763345717
asparticacid.c3	-0.008738852	0.028967432	0.763354603
lysopcac182.methionine	0.007950355	0.026359011	0.763400559
serotonin.smc241	0.003071357	0.010183164	0.763405764
lysopcac281.smc241	0.005009454	0.0166159	0.763501403
glutamicacid.pcaac360	0.016501244	0.05475987	0.763613461
pcaac424.pcaec425	-0.001531066	0.005082763	0.763697989
pcaec444.c102	-0.004993892	0.016580468	0.763725299
pcaac364.asymmetricdimethylarginin	-0.004308437	0.01430683	0.763760167
c14.pcaac320	-0.013593569	0.045140312	0.763764046
c102.c51	0.004276663	0.014204192	0.763806489
c5.pcaac403	-0.001587154	0.005271624	0.763813836
pcaac341.smc180	-0.002215413	0.007359625	0.763853712
pcaec384.pcaec301	0.017569333	0.058372479	0.763881042
lysopcac180.pcaec362	0.006935244	0.023043019	0.763894109
smohc241.asparticacid	-0.008772853	0.029158335	0.763970161
pcaec343.smc240	0.009148243	0.030406251	0.763971914

asymmetricdimethylarginine.c142	-0.009191014	0.030548431	0.763972063
isoleucine.lysopcac181	-0.014360964	0.047737004	0.763996358
glycine.pcaec420	-0.010610539	0.035283812	0.764084098
smc202.pcaec386	-0.003094722	0.010291309	0.764089865
pcaac366.pcaec342	0.025001681	0.083158963	0.764137734
pcaac343.pcaec425	-0.000734857	0.002444367	0.764150211
pcaac365.pcaec343	-0.021650705	0.072028608	0.764186787
arginine.histidine	-0.016257849	0.054111127	0.764287106
putrescine.lysopcac280	0.002328704	0.007752235	0.764334037
phenylalanine.pcaec361	0.000964199	0.003210615	0.764391389
pcaac362.lysopcac140	-0.00048748	0.001623323	0.764405521
pcaec362.pcaec405	-0.009451888	0.031475648	0.764409548
pcaac420.asparticacid	0.009259279	0.030841408	0.764462641
pcaac404.pcaec364	-0.004608555	0.015353986	0.764514896
c18.pcaec421	0.000370932	0.001236002	0.764550973
pcaec364.asymmetricdimethylarginir	-0.007024705	0.023414807	0.764623333
pcaec401.valine	-0.0116348	0.038794163	0.764699526
phenylalanine.pcaec383	-0.013129949	0.043786182	0.764734524
c51.pcaec343	-0.014216366	0.047420959	0.764791124
pcaec425.c16	0.008070004	0.026927431	0.764864392
pcaac320.lysine	-0.018366725	0.061295472	0.764904031
phenylalanine.pcaec384	0.004739275	0.015817628	0.764921229
pcaac341.methioninesulfoxide	0.004982076	0.016631672	0.764971708
pcaac422.pcaec425	-0.000648907	0.002168281	0.765185601
pcaac406.pcaac300	0.018089887	0.0604527	0.765210137
smc161.c12	0.011537989	0.038559173	0.765219455
pcaac384.smohc161	0.007804957	0.02608653	0.765244747
pcaac364.ornithine	-0.00608538	0.020340248	0.765256645
pcaec340.pcaac300	0.025924197	0.08666132	0.76528364
pcaec446.pcaec322	-0.018982558	0.063461511	0.765302353
c181.alphaaminoadipicacid	0.000832153	0.002782295	0.765325601
lysopcac140.acetylornithoine	0.00319039	0.010667735	0.765340496
c102.smc240	0.012412051	0.041504691	0.765353716
c51dc.pcaec301	0.025567627	0.08549657	0.765356147
c4.putrescine	-0.017890602	0.059836936	0.765401301
c4.pcaac424	-0.004654389	0.015570749	0.765454973
pcaec420.pcaec406	0.004317515	0.014455535	0.76564017
pcaac281.lysopcac203	0.002731752	0.009146311	0.765642422
pcaac420.smc180	-0.003235601	0.010834127	0.765660403
kynurenine.pcaec322	-0.013784403	0.046159606	0.76567881
pcaec384.serine	-0.009637577	0.03227459	0.765688637
c18.pcaec383	0.023786226	0.079658435	0.765695616
pcaac321.pcaec343	0.024392435	0.081722572	0.765790176
h1.pcaec342	0.028617944	0.095883417	0.765799704
smohc141.h1	0.000360773	0.001209088	0.7658618

pcaec446.creatinine	0.006438555	0.02157922	0.765874139
pcaec385.lysopcac160	-0.00312628	0.010479947	0.765917995
pcaec425.pcaec361	-0.003334434	0.011177951	0.765922627
pcaec406.methionine	-0.00657403	0.022042866	0.765973028
smohc221.c18	-0.008609657	0.028871525	0.76599787
pcaec361.prolinr	-0.008955569	0.030033633	0.766013986
c162.threonine	0.007482208	0.025094858	0.766035021
pcaec402.serine	-0.01347106	0.045188478	0.766072142
isoleucine.c12	-0.008908745	0.029897998	0.766176482
glutamicacid.pcaec420	-0.011498579	0.038592839	0.766195812
serotonin.pcaec361	0.003947818	0.013252175	0.766231039
pcaac381.c142	0.008992309	0.030190805	0.766269427
pcaac364.c3	0.006566472	0.022051724	0.766325295
pcaec423.pcaac383	0.002930823	0.009843082	0.7663415
h1.pcaac342	-0.001665089	0.005592321	0.766348383
pcaac341.pcaec343	-0.024996828	0.083962138	0.766371316
pcaec401.pcaac362	0.004862776	0.016333695	0.766372144
c141.lysopcac161	-0.023929154	0.080380973	0.766385591
pcaac366.pcaac401	0.003326604	0.011176818	0.766433161
c102.pcaec340	0.017103565	0.057478433	0.766485999
pcaec423.lysopcac140	-0.00045577	0.001531908	0.766521662
c8.pcaac425	-0.019304948	0.064903201	0.76657951
smc202.taurine	-0.012538995	0.042168012	0.766643626
pcaec343.c4	-0.004031766	0.013559275	0.766654469
h1.smc241	0.004136296	0.01391533	0.766727837
pcaec363.pcaac300	-0.018990363	0.063904411	0.766788153
pcaac380.pcaac360	0.015174143	0.051067855	0.766812055
smohc241.asparagine	-0.002282746	0.007683264	0.766835313
isoleucine.pcaac300	0.009312259	0.031344907	0.766847766
smohc222.serotonin	0.020739389	0.069812907	0.766862256
pcaec420.creatinine	0.003940731	0.013269023	0.766926027
pcaec361.pcaac404	0.002068052	0.006965482	0.766992435
phenylalanine.taurine	-0.010339064	0.03482376	0.766994939
c141oh.smc240	0.008079906	0.02722345	0.767069093
putrescine.c18	-0.005842461	0.01969089	0.76713819
pcaec386.lysopcac160	-0.006914126	0.023308473	0.767193856
pcaec343.c12	-0.017435257	0.058784038	0.767222243
pcaac344.pcaec406	0.003181488	0.01072787	0.767249399
smc202.methionine	0.005073898	0.017109099	0.767250523
c141oh.pcaac320	-0.019564921	0.065986656	0.767298694
ornithine.lysopcac181	0.006677248	0.022528866	0.767383879
pcaec425.pcaac321	0.004469147	0.015085236	0.767480402
glycine.c3	0.010205866	0.034454663	0.76751715
pcaec380.pcaac422	0.023961247	0.080894382	0.767522762
pcaec403.pcaec384	-0.0079799	0.026940867	0.76752546

smc261.pcaec401	-0.008360263	0.0282256	0.767530175
pcaec381.c3	0.011451235	0.03866163	0.767532577
asparagine.c2	0.020178712	0.068144482	0.767589436
lysopcac180.pcaec360	-0.001326883	0.004482009	0.767642914
lysopcac180.c51dc	-0.029583791	0.099945425	0.767678704
pcaac364.taurine	0.003095788	0.010460398	0.767713949
pcaac360.serotonin	-0.021237741	0.071761437	0.767716984
leucine.glutamicacid	0.009659567	0.032643968	0.767749431
c141oh.c51	0.008677277	0.029331081	0.767800988
smc161.pcaec425	-0.003139658	0.010613116	0.767809289
c102.c142	-0.012373039	0.041827645	0.767823013
pcaec425.lysopcac181	-0.016850925	0.056972627	0.767851803
pcaac404.pcaac383	-0.001293155	0.004372629	0.767877537
c0.isoleucine	0.003296102	0.011146676	0.767904844
smohc241.pcaac360	-0.00699047	0.023665646	0.768147206
pcaec443.pcaec423	0.005822229	0.019710667	0.768147227
c51.spermidine	-0.015853766	0.05368251	0.76819303
pcaac421.c12	0.009514198	0.032217955	0.768206196
c2.ornithine	0.020712659	0.07017654	0.768325533
pcaac366.pcaac363	0.00493729	0.016729573	0.768346315
pcaec383.pcaec424	0.005466308	0.018522144	0.768346814
c14.lysopcac203	-0.00357771	0.012127223	0.768429198
lysopcac161.lysopcac140	-0.000381293	0.001292628	0.768459758
kynurenine.pcaec384	0.003850908	0.013059246	0.76853205
pcaec380.pcaec386	-0.003420711	0.01160052	0.768535238
pcaac360.lysopcac181	0.006909813	0.023436807	0.768572019
pcaac344.methioninesulfoxide	0.005946445	0.020177779	0.76866712
asparagine.lysopcac161	0.012659223	0.04296219	0.768699934
histidine.pcaec404	0.006497088	0.02205417	0.768747822
glycine.pcaec365	0.00620239	0.02106335	0.768849363
pcaec362.methioninesulfoxide	0.003049003	0.010354945	0.768860503
lysine.pcaec386	0.003064812	0.010410063	0.768891336
histidine.putrescine	0.006259906	0.021273192	0.769002594
histidine.acetylornithoine	0.00513343	0.017445853	0.769012775
pcaac343.lysopcac140	0.001088274	0.003700934	0.769161667
phenylalanine.smohc141	-0.003548558	0.012068468	0.769175768
smc240.pcaac365	-0.01735749	0.059040419	0.769207943
putrescine.lysopcac182	0.00388226	0.013205339	0.769209155
pcaec423.pcaec420	0.004655568	0.0158371	0.769228822
c102.smc180	-0.006103137	0.020777307	0.769400729
pcaac405.pcaac362	-0.002886319	0.009826977	0.76942105
pcaec402.pcaac320	0.024037475	0.081852971	0.769457176
lysopcac280.pcaac385	-0.008157706	0.02778118	0.769476262
glycine.pcaec406	0.002252175	0.007672539	0.76955585
lysine.pcaec383	-0.016238589	0.055323928	0.769570135

smohc241.c12	0.015400366	0.05247408	0.769595431
ornithine.pcaac321	-0.005653494	0.019263425	0.769596849
pcaac425.pcaac380	-0.016543993	0.056378151	0.769624647
pcaec322.pcaac383	0.000988704	0.003369691	0.769652245
pcaac364.pcaec340	0.016272181	0.055461525	0.769663576
pcaec424.glycine	-0.00392158	0.013372178	0.769764012
pcaac380.c142	-0.014205281	0.048439568	0.769768862
c182.putrescine	-0.006622404	0.022586667	0.769813158
isoleucine.glycine	-0.009997041	0.034106275	0.769878209
pcaec384.pcaec385	0.016105343	0.054957331	0.769925993
pcaac323.pcaac380	0.009746052	0.03325834	0.769934319
c0.histidine	0.014593646	0.04980693	0.769962173
pcaac401.pcaac281	-0.000611409	0.002087434	0.77004203
pcaec421.smc161	-0.026885585	0.091798569	0.770060173
smc202.smohc222	-0.002015692	0.00688275	0.770071197
pcaec421.pcaac420	0.038782349	0.132447881	0.770108763
pcaec444.c14	-0.012234857	0.041791336	0.77014807
smohc241.pcaec402	0.010739022	0.036683303	0.770156482
spermidine.alphaaminoadipicacid	0.004984629	0.017042276	0.770357472
smohc221.c4	-0.003072713	0.010505828	0.77036443
glutamine.methioninesulfoxide	-0.004198763	0.014357415	0.770388375
acetylornithoine.lysopcac170	-0.003880847	0.013270988	0.770399598
pcaac422.serotonin	-0.024630166	0.084233876	0.770421533
lysopcac280.asparticacid	-0.013453043	0.046011541	0.770435292
pcaac402.pcaac383	0.002099598	0.007181064	0.77043859
smc240.pcaec425	0.000835363	0.002857427	0.770462978
isoleucine.lysopcac161	-0.023730115	0.081171878	0.770465851
c181.pcaec365	-0.0059285	0.020284769	0.770527195
lysopcac182.arginine	-0.015223693	0.052096995	0.770561811
pcaec363.pcaec402	-0.012576204	0.0430384	0.770568939
pcaac342.smohc161	-0.005371025	0.018396042	0.770754
smc261.pcaec386	0.002772013	0.009495187	0.77077505
pcaec402.pcaec446	-0.008476473	0.029037047	0.770789949
arginine.pcaac360	-0.015644155	0.053592397	0.770797019
pcaac344.ornithine	-0.005718418	0.019590891	0.77081099
ornithine.pcaec384	0.004288367	0.014698077	0.77090854
pcaec364.pcaac240	0.003276181	0.011230753	0.770945626
alanine.arginine	0.004361845	0.014956148	0.77100129
pcaec381.pcaac361	-0.011297965	0.038739436	0.771003091
alphaaminoadipicacid.pcaec425	0.00069092	0.002369366	0.771029319
leucine.smohc241	0.011122397	0.038145354	0.771049294
putrescine.glutamine	0.006146183	0.021080159	0.77106218
pcaec444.c8	0.010834366	0.037159736	0.77106255
pcaec423.pcaec383	-0.020947306	0.071852096	0.771084144
pcaec362.c8	-0.011669215	0.040029702	0.771099228

pcaec405.pcaac384	0.011959042	0.041025972	0.771110371
pcaec340.pcaec385	-0.015752264	0.054044946	0.771135793
pcaec364.c51	-0.005920738	0.020321794	0.771224952
pcaac420.methioninesulfoxide	0.005187533	0.01781176	0.77130669
smc202.spermidine	0.015388824	0.05283976	0.771311537
pcaac424.pcaec341	-0.00532729	0.018299405	0.771401181
pcaec382.pcaec322	0.016880103	0.058008624	0.771496752
c51dc.pcaac240	0.005600058	0.019244685	0.771497178
c10.methioninesulfoxide	0.003649354	0.012542325	0.771519529
lysopcac161.smc261	0.014953632	0.051420143	0.771634342
pcaec364.pcaec444	0.023498062	0.080809406	0.771656451
pcaec445.pcaec443	0.009766762	0.03361123	0.771811727
lysopcac203.c182	0.010770032	0.037077821	0.77189517
pcaec405.pcaac320	-0.021070788	0.0725454	0.771911441
pcaec301.c141	-0.008736357	0.030083933	0.771949804
smc181.taurine	-0.01242864	0.042802586	0.771971316
pcaec422.pcaec380	0.029047037	0.100038704	0.771981429
pcaac380.pcaec401	-0.009663339	0.033281157	0.771983967
pcaec360.methioninesulfoxide	0.003528471	0.012155452	0.772041788
pcaec365.c16	0.008803823	0.030332366	0.772067547
pcaec401.pcaec425	-0.000854132	0.002943432	0.77211547
serotonin.acetylornithoine	0.005112053	0.017618458	0.772137694
pcaec300.pcaec421	0.000821063	0.002830181	0.772171064
c51.c18	-0.013357801	0.046052907	0.772213962
serotonin.pcaec362	-0.00816705	0.028159511	0.772233229
c3.pcaac360	0.007896719	0.027229701	0.772251747
smc260.pcaac401	-0.009138124	0.031515816	0.772290128
smc241.taurine	0.007533663	0.025988965	0.772346852
lysine.smc260	0.002686995	0.009271069	0.772387658
pcaec364.pcaec362	0.006599504	0.022774122	0.772422021
pcaac383.pcaac381	-0.010255852	0.035391993	0.772423521
smc261.asparticacid	-0.008489462	0.029304929	0.772488318
pcaec321.pcaac406	-0.009194119	0.031751054	0.772583797
pcaec342.taurine	0.011297504	0.039047913	0.772770705
smc180.valine	0.012030554	0.041582875	0.772777611
pcaec300.pcaac402	0.015194853	0.05254243	0.772871583
pcaac321.smohc221	-0.011556003	0.040009761	0.773148362
c14.ornithine	-0.007646417	0.026474723	0.773156038
c51.serine	0.007034019	0.024356312	0.773173575
pcaec361.methioninesulfoxide	0.003363273	0.011648827	0.773230503
pcaec321.pcaec380	0.011896373	0.041214527	0.773289218
pcaac281.methioninesulfoxide	0.002469891	0.008558397	0.773329283
serotonin.c162	-0.013770167	0.047715994	0.773334407
pcaec384.smohc141	-0.005602118	0.019414724	0.773361894
c101.asymmetricdimethylarginine	0.002596994	0.009000267	0.773364683

histidine.glutamicacid	0.008363582	0.028986159	0.77337174
pcaac381.smohc241	0.013631677	0.04724866	0.773392981
asparticacid.pcaac240	0.006267826	0.02172984	0.773443392
pcaec342.asparagine	0.002382365	0.008262399	0.773523693
pcaec443.smohc141	-0.00584714	0.020279082	0.7735272
c141.pcaac344	-0.015075801	0.052299252	0.77358311
pcaec404.serine	0.018326469	0.063576163	0.77358332
c162.pcaac384	0.010365055	0.035962451	0.773614844
smc181.smohc141	-0.006201525	0.021521233	0.773660948
smohc241.c182	-0.006047036	0.02098598	0.773670106
c2.lysopcac280	0.003538177	0.012281914	0.773720641
pcaac426.pcaec425	-0.001288846	0.004474319	0.77374061
pcaac406.pcaac240	-0.005051035	0.017543286	0.77384429
pcaac362.pcaec420	0.004955713	0.017212634	0.773849684
phenylalanine.pcaec362	0.002559	0.00889001	0.77389561
pcaac240.lysopcac160	0.007568865	0.026298852	0.773933139
smohc141.methionine	-0.003949829	0.013724785	0.773943901
c141.pcaac380	-0.012016991	0.041762109	0.773973968
pcaac365.pcaac320	0.016453642	0.057185005	0.773990892
c5.alphaaminoadipicacid	-0.002185554	0.007598185	0.774055705
pcaec382.kynurenine	0.002349875	0.008169778	0.774064471
histidine.pcaac383	0.008297412	0.028851261	0.774093109
c0.pcaac300	0.013957575	0.0485703	0.77426438
pcaac385.pcaac300	0.027341789	0.09514713	0.774268397
c141.glutamicacid	0.008851167	0.030802141	0.774274256
pcaac366.serotonin	-0.024482299	0.085202252	0.774283617
lysopcac182.h1	0.00071684	0.002495033	0.774311649
c101.pcaac361	0.010464237	0.03643362	0.77438262
pcaac361.pcaac385	-0.004315998	0.015027714	0.77439117
pcaac384.pcaac300	0.015893322	0.055381357	0.774561497
c16.pcaac363	0.004765437	0.016607833	0.774592449
pcaec380.pcaac365	0.013629425	0.047504846	0.774617762
pcaec421.pcaec425	-0.001449104	0.005050951	0.774624507
pcaec383.tyrosine	-0.016974538	0.059180199	0.774677362
methionine.pcaec423	0.002766063	0.009645525	0.77472039
pcaec406.c3	0.00501328	0.017484719	0.774757207
lysine.lysopcac182	-0.006143765	0.021445364	0.774939817
pcaec341.pcaac383	0.000974262	0.003400902	0.774949679
pcaac364.smohc141	0.013871799	0.048425572	0.774961571
pcaac320.taurine	0.005768596	0.02013787	0.77496229
pcaac425.c162	0.015387792	0.053725195	0.774991678
c51.pcaec422	-0.004752723	0.016595053	0.775009042
pcaec405.pcaac360	-0.015055687	0.052590804	0.775096254
pcaac281.lysopcac182	-0.004769786	0.016661856	0.775103948
lysopcac180.isoleucine	0.007013768	0.024505751	0.775150359

pcaec301.pcaec425	-0.000723048	0.002527321	0.7752394
c2.taurine	-0.015795211	0.055265764	0.775459456
c10.smc160	-0.015785868	0.055235518	0.77546912
c18.smc161	-0.016477833	0.057678736	0.775552427
pcaac385.creatinine	0.003207445	0.01122904	0.77558651
pcaac403.lysopcac203	0.004754718	0.016651588	0.775660629
pcaec365.putrescine	-0.017309984	0.060627149	0.775680538
c141.pcaec401	0.012427672	0.043539977	0.775744811
smc240.pcaac322	0.01846659	0.06470768	0.77578034
pcaec364.acetylornithoine	0.008311433	0.02912398	0.775783206
c4.serine	0.008331341	0.029197893	0.775814229
c5.pcaac281	-0.00144748	0.005073833	0.775857995
pcaec322.glycine	-0.018868055	0.066150611	0.77589966
c141.pcaac322	-0.014086422	0.049390761	0.775918888
pcaac403.pcaac360	-0.014247308	0.049970947	0.775989029
pcaac281.pcaec384	0.012522184	0.043934327	0.776058849
pcaec321.h1	0.000413773	0.001452033	0.776104428
pcaec364.pcaec420	-0.004435593	0.015570199	0.776168514
smc241.c14	0.011677346	0.040991148	0.776170236
pcaec383.pcaac426	0.017427925	0.061178898	0.776175256
alanine.isoleucine	0.005131362	0.018013655	0.776181898
pcaac404.c12	-0.011751548	0.041258091	0.776204391
c51.c142	-0.015043146	0.052845404	0.776331967
citruline.pcaac342	-0.001190287	0.004182104	0.776369406
pcaec386.pcaec425	-0.001446458	0.005083935	0.776444979
c142.pcaac383	0.001365384	0.004800464	0.776512192
c12.pcaec342	0.0150536	0.052927999	0.776520604
pcaec363.pcaac380	-0.022754725	0.080032632	0.77659591
lysopcac161.c4	0.003790385	0.013332318	0.776609367
pcaac360.lysopcac204	0.009184997	0.032307434	0.776609892
glycine.pcaec403	-0.00494516	0.017402672	0.776716065
glycine.c18	0.011884814	0.041828162	0.776736475
c181.smohc161	-0.003512885	0.012365826	0.77677788
pcaec405.threonine	-0.007125782	0.025083736	0.77677816
lysopcac182.pcaec421	-0.000651129	0.002292123	0.776783815
pcaac426.smc180	0.00284268	0.010008397	0.776816611
ornithine.c8	-0.01054143	0.037123823	0.776874911
c141oh.pcaec425	0.002035444	0.007168238	0.776874917
pcaac361.pcaec382	-0.001472436	0.005185925	0.776893193
smc160.phenylalanine	-0.011342932	0.039956331	0.776928458
lysopcac160.pcaac383	-0.002015292	0.007100778	0.776982282
lysopcac281.pcaac240	0.00337946	0.01190925	0.77701687
c5.pcaec386	0.003366626	0.011867528	0.777080948
pcaac425.methionine	-0.008625209	0.030412923	0.777142453
pcaac405.c141oh	-0.022497947	0.079344504	0.777185112

pcaec360.serine	0.016429381	0.057943765	0.777190874
pcaec406.serotonin	0.029903753	0.105495409	0.77725196
pcaec385.pcaac426	-0.009849577	0.034755406	0.77730035
pcaac420.c12	0.009568435	0.033772267	0.777357492
lysopcac170.ornithine	-0.006368841	0.022480041	0.777366161
pcaec424.pcaec422	0.013051886	0.046078347	0.777409545
pcaec381.pcaec401	-0.014532767	0.051338607	0.777545264
pcaec444.pcaac384	-0.014619526	0.051645146	0.777545489
pcaac365.smc161	-0.012682492	0.044814546	0.777604411
pcaac365.smohc241	-0.014068524	0.049714805	0.777615783
pcaec385.pcaac320	0.01294382	0.045743523	0.777630707
pcaec380.c8	0.009995948	0.035340822	0.777723142
isoleucine.smohc141	-0.003678629	0.013006882	0.777740338
c182.pcaec386	-0.004079849	0.014427782	0.777774366
pcaec364.smc261	-0.004055695	0.01434673	0.777840183
pcaec380.methioninesulfoxide	0.003838852	0.013586636	0.777951182
smc240.tyrosine	0.014418991	0.05104216	0.777992779
glutamicacid.smc260	0.015083745	0.053397026	0.777999602
alphaaminoadipicacid.tryptophan	-0.002498592	0.008849616	0.778109697
pcaec443.c101	0.00757917	0.026845497	0.778119943
isoleucine.c4	0.00834674	0.029565241	0.778127291
pcaec425.lysopcac160	-0.006395712	0.022662541	0.778204466
pcaac342.c12	0.009794584	0.034709931	0.778228332
pcaec403.methionine	-0.007528748	0.026684089	0.77825909
pcaac321.prolinr	-0.01116925	0.039598174	0.778319491
c12.transhydroxyproline	-0.006216837	0.022041846	0.778333064
lysopcac280.lysopcac140	0.000311186	0.001103621	0.778393589
c141oh.smc241	-0.004257473	0.015100468	0.778412558
alanine.c142	0.007180648	0.025469534	0.778421959
alphaaminoadipicacid.threonine	-0.012968146	0.046015388	0.778505306
pcaec420.pcaec423	-0.003205607	0.011374662	0.778506795
pcaac403.pcaec425	-0.001703845	0.006046109	0.778515629
pcaec363.pcaac381	0.007365924	0.026138501	0.778519331
pcaec444.h1	0.000837096	0.002971502	0.778592439
pcaec360.threonine	0.008153636	0.028953572	0.778666953
lysopcac182.pcaac384	-0.009980886	0.03544272	0.778670477
pcaec422.lysopcac181	0.017880203	0.063496991	0.778681762
pcaec363.pcaec421	-0.000735539	0.002612379	0.778706568
pcaac323.ornithine	-0.010064535	0.035758923	0.77878595
pcaec365.c3	-0.004986534	0.017720169	0.778824833
pcaec425.pcaec420	0.007339641	0.026086632	0.778861552
pcaec401.smc180	0.001408735	0.005010855	0.779029668
pcaac385.c101	-0.001331125	0.004736355	0.779100532
pcaec423.serotonin	-0.020866924	0.074259065	0.779133033
pcaac363.pcaac364	-0.021596635	0.076856047	0.779133482

pcaac421.smc180	-0.00202698	0.007213872	0.779146829
pcaec425.methionine	0.007841858	0.027924573	0.779269927
lysopcac161.pcaec362	0.005828044	0.020754012	0.779275726
pcaac426.alanine	-0.004591157	0.016354739	0.779346107
pcaec422.pcaec403	0.009463988	0.033720215	0.77939294
smc241.pcaec322	0.018246595	0.065024976	0.779433601
pcaac422.pcaac426	0.018177237	0.064806581	0.779528977
prolinr.pcaac404	0.001505313	0.005367979	0.77957489
smc240.pcaec445	-0.022324484	0.079610787	0.77957804
pcaac365.serotonin	-0.026706668	0.095238412	0.779578997
c182.pcaac380	0.011408522	0.040687199	0.779596782
methionine.prolinr	-0.005777149	0.020609892	0.779662866
c141.acetylornithoine	0.010234971	0.036519458	0.779700206
pcaac343.asymmetricdimethylarginir	0.008046943	0.028716246	0.779729353
pcaec364.c16	0.007919985	0.028282888	0.779878813
pcaac366.pcaec425	-0.000922376	0.003294987	0.779951152
putrescine.smohc241	-0.008853507	0.031640709	0.780042396
pcaac401.lysopcac281	0.002963622	0.010596576	0.780146888
c182.lysopcac281	0.001734915	0.006203452	0.780152965
pcaec404.histidine	-0.017007546	0.060822028	0.780184607
lysine.smc240	0.01026443	0.036750741	0.780436882
c3.pcaec425	-0.00147435	0.005279205	0.780454918
pcaec424.ornithine	0.007362735	0.02636955	0.780502039
lysine.pcaac420	0.014294697	0.051198142	0.780509758
pcaac366.c182	0.007242421	0.025944446	0.780549826
pcaac344.pcaac425	-0.026883525	0.096311932	0.780566185
pcaac322.pcaec406	0.00179079	0.006417067	0.780614475
pcaac366.pcaac422	0.018347162	0.06574596	0.780618497
pcaec405.pcaec342	0.021838574	0.078300869	0.780737607
pcaac403.pcaac361	-0.011340957	0.040664126	0.780747165
phenylalanine.pcaec385	-0.009569984	0.034323272	0.780804072
pcaac386.lysine	-0.020388413	0.073124871	0.780806152
pcaec380.asymmetricdimethylarginir	0.007416382	0.026614444	0.780925898
pcaec383.c51	-0.026976092	0.096854245	0.781031205
pcaec422.pcaec420	0.005289115	0.01899243	0.781059651
isoleucine.pcaac363	-0.004571732	0.016418365	0.781085018
pcaec384.pcaec364	-0.005558827	0.019965321	0.781106596
pcaec446.c162	-0.013011862	0.046739995	0.781134126
c16.pcaac420	0.034231404	0.122972009	0.781150005
pcaac403.serotonin	-0.027552489	0.098991649	0.781177585
lysopcac160.pcaac360	-0.005757767	0.020709377	0.781410739
pcaec340.pcaac343	-0.011321555	0.040732604	0.781471135
arginine.pcaac422	0.011754615	0.042292225	0.781478997
pcaac365.asymmetricdimethylarginir	-0.005306142	0.019092034	0.781489379
pcaec362.pcaac300	0.020668262	0.074368832	0.78149609

pcaac426.c3	0.009439227	0.033965219	0.781501464
c18.h1	0.001227924	0.004425673	0.781849106
smc202.lysopcac203	-0.004291064	0.015467987	0.781879
pcaac404.pcaac342	-0.001345416	0.004851639	0.781958762
smc260.pcaac360	-0.005875701	0.02119119	0.781990025
pcaac381.lysopcac280	-0.001629864	0.005879244	0.782026476
pcaac384.pcaac342	-0.001691125	0.006100892	0.782049674
pcaac361.c142	-0.011106131	0.040066488	0.782050084
lysopcac204.pcaec365	-0.004310771	0.015555569	0.782105065
pcaec382.pcaec425	-0.001231251	0.004443122	0.782110381
phenylalanine.pcaac343	0.006371092	0.023000354	0.782197803
pcaec381.pcaac406	0.017347758	0.062645589	0.782259621
pcaac422.pcaec340	-0.014976749	0.054086692	0.782272199
c8.methionine	0.008979716	0.032429287	0.782273134
smc241.glutamicacid	-0.007538581	0.027228838	0.782304754
lysopcac182.smohc241	0.010329936	0.037323866	0.782377766
lysopcac281.smohc221	0.013549723	0.048988605	0.782512338
pcaac342.pcaec380	0.013282239	0.048050084	0.782638326
pcaec404.smc202	-0.016677252	0.060345452	0.782685728
acetylornithoine.pcaec386	0.001879587	0.006803158	0.782748221
pcaac401.c2	0.019601264	0.070946888	0.782748736
c8.tyrosine	0.020102509	0.072771763	0.782779628
pcaac365.smc260	-0.007368045	0.026679661	0.782835879
lysopcac170.pcaac320	-0.00756706	0.027400741	0.782839345
pcaec401.lysopcac161	-0.016590723	0.060099184	0.782921261
pcaec384.smc202	0.015329724	0.055536648	0.782941765
smc181.pcaac380	0.015810093	0.057323907	0.783115136
smc161.lysopcac161	-0.013564408	0.049192889	0.783163862
pcaec340.asparticacid	-0.011728272	0.042537849	0.783183344
pcaec385.pcaac366	-0.007073211	0.025662196	0.783249356
phenylalanine.lysopcac280	0.005254199	0.019063769	0.783261549
c12.putrescine	0.007515453	0.027268519	0.78326356
pcaac341.c182	0.005959762	0.021625239	0.783276011
pcaac300.pcaac342	-0.001211573	0.00439792	0.783356602
pcaec385.creatinine	0.003876274	0.014071129	0.783364756
pcaac320.pcaec360	0.001530892	0.005557448	0.783372711
smc160.pcaac366	0.005337243	0.019379512	0.783418925
c101.kynurenine	0.001149812	0.004175737	0.78345803
pcaac344.pcaac381	0.011563649	0.042007679	0.783519966
pcaac424.pcaac420	-0.01826678	0.066381198	0.783592517
glutamicacid.c162	-0.021826102	0.079324319	0.783615381
pcaac363.isoleucine	0.005908058	0.021475117	0.783644834
pcaac405.pcaec421	0.000241832	0.000879346	0.783719993
lysine.pcaac362	-0.00190531	0.006930915	0.783807038
pcaec405.pcaec384	-0.008857397	0.032226221	0.783845044

c14.pcaec341	0.015642068	0.056932951	0.783925718
pcaec343.lysopcac170	-0.002193928	0.00798737	0.78397997
serotonin.pcaac426	-0.015029064	0.054726989	0.784022745
pcaec300.pcaec423	0.001748373	0.00636757	0.784056679
pcaec381.pcaec365	-0.005277156	0.019222684	0.784092773
pcaec365.pcaac240	-0.004433702	0.016152467	0.784121045
c8.alanine	0.008396193	0.030609906	0.784269864
pcaec406.phenylalanine	0.006021887	0.021959472	0.784322981
serine.lysopcac204	-0.006486237	0.023654973	0.784342539
pcaec365.pcaac361	0.024461009	0.089214931	0.784358766
pcaec404.pcaac384	0.010511851	0.038342441	0.784376871
pcaac381.c51	-0.010320909	0.037646764	0.784381285
arginine.pcaec384	0.002792444	0.010186505	0.784396291
pcaec365.pcaac420	0.013677137	0.049898961	0.784423202
lysopcac203.ornithine	-0.010183727	0.037179046	0.784566026
pcaac405.c8	0.009202072	0.033615142	0.784690696
pcaec385.pcaac240	-0.003922597	0.014330928	0.784715522
pcaec423.methionine	0.01250889	0.045727863	0.784841883
c14.pcaec386	-0.000662534	0.002422046	0.784847688
smc161.methioninesulfoxide	0.002012261	0.007361086	0.784984601
methionine.pcaac343	-0.00585328	0.02141212	0.784985896
kynurenine.c182	0.014367124	0.052557211	0.784986928
asparagine.pcaec382	-0.003586619	0.013120749	0.784992198
leucine.c14	0.00825216	0.030190574	0.785006847
c141.threonine	0.005459721	0.019974449	0.785007168
pcaac401.pcaac381	0.020606491	0.075403266	0.785046615
pcaec365.serotonin	0.019250162	0.07046836	0.785130411
smohc241.pcaac343	0.00552015	0.020214411	0.785202996
lysine.c3	-0.008434115	0.030894826	0.785268589
pcaec301.isoleucine	-0.003742623	0.013715585	0.785361135
pcaec402.pcaec365	-0.005787333	0.021209176	0.785364549
c142.pcaec386	-0.001071942	0.003928582	0.785373724
pcaec404.pcaec382	0.014261477	0.05227997	0.78542504
c12.pcaec340	-0.007459774	0.027351077	0.785462588
pcaac420.c102	-0.00628445	0.023046799	0.785508173
pcaec383.c162	-0.022675146	0.083158947	0.785515645
pcaec380.putrescine	0.014061919	0.051577052	0.785541196
c5.pcaec402	-0.019070758	0.069950054	0.785545125
pcaac360.smc160	-0.0190333	0.069819209	0.785564744
smohc141.pcaec401	-0.011066453	0.040595691	0.785569872
pcaac383.glutamicacid	-0.007734038	0.028376506	0.785608948
lysopcac280.methioninesulfoxide	0.004497013	0.016499748	0.785609207
c3.pcaec401	0.012151666	0.044587756	0.785622018
lysopcac170.pcaac364	0.010485562	0.038478812	0.785646173
pcaac403.smc240	0.01438323	0.05278747	0.785667579

glutamicacid.pcaec401	-0.017242519	0.063284835	0.785679384
pcaac344.pcaac420	0.018469063	0.067804877	0.785735748
methionine.pcaec403	0.00916595	0.033651889	0.785743393
pcaac362.serotonin	0.01914179	0.070309764	0.785840153
smc180.c182	-0.005025569	0.018467495	0.785931372
pcaec402.c18	0.008669463	0.031874446	0.786040795
pcaec425.c101	0.005087528	0.018705074	0.786041866
pcaac281.pcaec403	-0.00620979	0.022855322	0.786261719
pcaac403.pcaac402	0.063183192	0.23255354	0.786267002
prolinr.smc240	0.010705967	0.039404817	0.786267979
pcaec401.pcaec343	-0.017324723	0.063766847	0.786270486
pcaec341.smohc241	0.020985822	0.077262749	0.786325934
smc202.pcaec425	-0.000983198	0.003620103	0.786343003
smc180.pcaac363	0.001352404	0.004982293	0.786459571
c5.smohc141	-0.004165397	0.015345836	0.786465063
asparticacid.smc160	-0.009985244	0.036797335	0.786524309
smohc161.pcaec446	-0.005670185	0.020897431	0.786542517
pcaac360.pcaec363	-0.008541625	0.031493822	0.786633214
pcaac421.pcaac322	0.015774746	0.058172406	0.786666666
pcaec380.pcaac364	0.013672136	0.050424427	0.786690598
acetylornithoine.pcaec340	-0.017117881	0.063139042	0.786711384
pcaac323.valine	0.010262091	0.037854236	0.786726068
pcaac366.histidine	-0.01492254	0.055048788	0.786738703
pcaac366.h1	0.000419424	0.001547926	0.786830405
lysopcac204.transhydroxyproline	-0.010333852	0.03814331	0.786858984
pcaac320.pcaec341	0.017032912	0.062886904	0.786914116
methionine.pcaec362	-0.003098081	0.011439918	0.78694227
lysopcac204.taurine	-0.012952984	0.047850271	0.787030542
pcaec383.smc180	0.000737805	0.002725592	0.787032704
c141.creatinine	0.009772683	0.036119457	0.78713234
smc180.pcaac365	-0.014104143	0.052161279	0.787263396
pcaec443.c102	-0.008366281	0.030949847	0.787322908
c8.smc240	0.007726817	0.028587041	0.787343227
pcaac320.pcaac363	0.002310965	0.008553994	0.787442103
c18.pcaac344	-0.012135998	0.044923792	0.787454185
c51.c101	-0.001534016	0.005679943	0.787508287
pcaec364.pcaac426	-0.006266371	0.023202568	0.787511024
asparticacid.pcaac341	0.002129996	0.00788838	0.787553589
c142.pcaac425	-0.013502983	0.050015524	0.78758509
lysopcac170.pcaac362	0.004024137	0.014908368	0.787624364
citruline.pcaec445	-0.014375542	0.053263119	0.78764582
pcaec321.pcaac405	-0.00953693	0.035340512	0.787675343
histidine.pcaac363	-0.003524267	0.013067556	0.787799963
phenylalanine.creatinine	0.003962713	0.014694054	0.787811162
pcaac380.isoleucine	0.007865652	0.029172156	0.78785156

pcaec382.pcaac364	0.01517699	0.056309355	0.787928358
spermidine.alanine	-0.018543097	0.06885491	0.788098755
pcaec386.pcaac420	0.014793673	0.054958697	0.788197662
c0.alphaaminoadipicacid	0.002294478	0.008525302	0.788228829
pcaec423.pcaec384	-0.006839379	0.025413601	0.788240092
lysopcac140.serotonin	0.017530664	0.065151683	0.788277068
c51dc.alphaaminoadipicacid	0.001937277	0.007199823	0.788278439
pcaac385.methioninesulfoxide	0.003786584	0.014074924	0.78831092
c2.asparticacid	0.006500285	0.024171021	0.788389009
smohc141.lysopcac203	0.002803515	0.010426493	0.78842361
smohc222.pcaac424	-0.003845994	0.014304434	0.788436319
pcaac322.threonine	0.007017734	0.026115636	0.788551156
smc260.pcaec341	-0.012969861	0.048266814	0.78855572
pcaac424.c3	-0.010749614	0.04002002	0.788637016
lysopcac160.lysopcac203	0.003741024	0.013927966	0.788643073
pcaac383.pcaac426	0.011253433	0.041898474	0.788650675
pcaac365.taurine	0.004184927	0.015590964	0.788779813
pcaac403.smohc141	-0.004501691	0.016773443	0.788808944
pcaac363.methioninesulfoxide	0.004175548	0.015563453	0.788878233
smc160.pcaec423	0.003990243	0.014874359	0.788900264
smc261.pcaec321	-0.013248606	0.04938694	0.788901678
citruline.c141oh	0.024860745	0.092679705	0.788915233
pcaec364.c101	-0.001818086	0.006782437	0.789057948
serotonin.pcaec423	-0.004580556	0.017091478	0.789100638
asparagine.pcaec362	-0.003978068	0.014843677	0.789104356
pcaac384.serine	-0.009970252	0.037215353	0.78917387
pcaec343.smc241	-0.004214279	0.015733371	0.789212982
glutamicacid.pcaac385	-0.005938086	0.022170321	0.789225757
smohc222.pcaac403	-0.001272371	0.004751058	0.789249898
asparticacid.pcaec301	0.016691323	0.062356532	0.789351599
asymmetricdimethylarginine.pcaec34	-0.017027839	0.063627081	0.789394806
glutamine.pcaac360	-0.007844497	0.029315099	0.78941555
glutamine.pcaac364	-0.010938605	0.040880417	0.78942837
pcaac360.threonine	0.00642056	0.023996416	0.78943789
lysopcac281.pcaec386	0.005762073	0.02154504	0.789530192
pcaec404.asymmetricdimethylarginir	0.004472034	0.016733097	0.789673349
pcaec443.valine	0.00969909	0.03629948	0.78971975
putrescine.smc202	-0.007462266	0.027929406	0.789729956
pcaac366.pcaac426	0.023219106	0.086926118	0.789783746
smc202.asparagine	0.003526706	0.013208945	0.789875439
c10.pcaec385	-0.007916643	0.029656286	0.789911723
pcaac300.lysine	0.016804372	0.062950482	0.789912238
pcaec363.pcaac365	0.009396355	0.035209401	0.78997006
asparagine.taurine	-0.007413256	0.027778939	0.789973577
smc202.pcaec380	0.012500734	0.04685261	0.790016826

phenylalanine.c142	0.012722088	0.047688999	0.79004586
c182.pcaac321	0.006278223	0.023536492	0.790067193
c3.smc260	-0.007654849	0.028705338	0.790124303
pcaec402.pcaec340	-0.01099419	0.041236591	0.790168404
pcaec385.methionine	-0.005005841	0.018779984	0.790214946
pcaec421.pcaac360	-0.008057645	0.030237918	0.790274176
pcaac341.pcaec340	0.01876038	0.070406931	0.790288327
pcaac403.serine	-0.015159	0.056919747	0.790391384
smohc161.pcaac383	0.001017475	0.003822165	0.790482474
pcaac386.glutamicacid	-0.007862512	0.029543507	0.790536632
pcaec381.pcaec420	0.008230274	0.030926673	0.790545195
pcaec425.smc241	0.013028811	0.048962006	0.790561921
pcaec425.valine	0.011704308	0.043996605	0.790617948
pcaec343.smc202	-0.014292088	0.053740531	0.790680481
c8.pcaec402	-0.015118364	0.05684906	0.790686234
glutamine.pcaec420	-0.005936913	0.022326893	0.790709365
asparagine.pcaac343	-0.004513762	0.016977268	0.790738303
pcaec423.threonine	0.004483257	0.016864082	0.79075709
pcaac365.pcaac343	-0.006520278	0.024528932	0.790777526
pcaac381.pcaec443	-0.024543274	0.092346612	0.790813254
pcaac365.smc202	0.007576706	0.028508143	0.790813276
pcaac385.lysine	-0.015316684	0.057638654	0.790841758
pcaec446.c12	0.008666508	0.032624589	0.790913161
glycine.pcaac323	-0.020354041	0.076625281	0.790922804
pcaac363.pcaac360	-0.005390315	0.020295191	0.790949858
prolinr.pcaac383	-0.005231653	0.019702464	0.790998089
pcaac381.pcaac240	-0.005221717	0.019669288	0.791042115
pcaac406.pcaac342	-0.00258181	0.009725815	0.791054569
pcaac403.pcaec423	0.012541309	0.047245548	0.791062195
methionine.c102	0.004462802	0.016814735	0.79109245
lysopcac140.c3	0.009212438	0.03471455	0.791118064
lysopcac140.smohc241	0.013348077	0.050309327	0.791161675
glutamicacid.pcaec365	0.008616042	0.032482162	0.791212061
methioninesulfoxide.asymmetricdim	-0.005898647	0.022238161	0.791216436
pcaac281.taurine	0.008305251	0.031321439	0.791283336
c5.pcaac424	0.002383845	0.008991693	0.791318286
lysopcac160.smohc141	-0.004676747	0.017641646	0.791333225
alanine.smohc141	-0.009109196	0.034364012	0.791346627
smc241.pcaac403	0.001379563	0.005205357	0.791386633
pcaec343.c182	0.008447172	0.031895593	0.791532126
smohc241.pcaac380	0.007879443	0.029752698	0.791537509
pcaec380.pcaac380	0.015168658	0.057277814	0.791541482
pcaac362.tyrosine	0.010276027	0.038806781	0.791561688
pcaec420.c101	-0.001862169	0.0070325	0.791565603
pcaac385.pcaec402	-0.005848903	0.022092074	0.791599054

pcaec362.pcaac366	0.005352772	0.020235617	0.791775017
pcaac421.pcaac383	0.001213054	0.004585905	0.791778381
c181.pcaec446	-0.011390144	0.043071786	0.791833992
pcaac426.c12	-0.011040905	0.04175199	0.791838145
pcaec424.pcaac364	0.009118362	0.034482767	0.791844108
pcaac343.pcaec406	0.001384698	0.005236892	0.791859623
pcaac422.kynurenine	-0.002647192	0.010012675	0.791881269
pcaec383.pcaac360	-0.014374803	0.054399888	0.791989586
pcaac385.pcaac380	-0.010252661	0.038808128	0.792031676
pcaec301.smc260	-0.007670801	0.029045079	0.792099842
smohc141.lysine	0.012502712	0.047354262	0.792157349
lysine.pcaac363	-0.005436597	0.020593903	0.792183835
pcaec380.c182	0.007654168	0.029033641	0.792460382
ornithine.pcaec444	-0.016173904	0.06136821	0.792518762
pcaac342.isoleucine	-0.005810587	0.0220512	0.792557774
pcaec382.methionine	-0.006243307	0.023702165	0.792632968
c16.pcaec365	-0.006265893	0.02379025	0.792652872
pcaec380.leucine	-0.01052982	0.039981426	0.792662809
kynurenine.smc260	-0.005010599	0.019025422	0.792666224
c51.pcaac381	-0.010938843	0.041556905	0.792772141
pcaec360.pcaec386	-0.001005187	0.0038193	0.792802544
pcaac402.smc261	0.016982331	0.064527459	0.792807309
c14.pcaec403	0.005455448	0.020734108	0.792857509
pcaec383.pcaec406	-0.011827034	0.044954596	0.792877675
smc181.pcaec421	-0.000299328	0.001137774	0.79288262
pcaec385.isoleucine	-0.004438133	0.016871603	0.792904591
pcaac322.valine	0.014259438	0.054209483	0.792912429
smc181.lysine	0.011460761	0.043571236	0.792918749
lysopcac180.pcaac403	-0.003562327	0.013544008	0.792931351
lysine.c14	-0.008904175	0.033856473	0.792947444
lysopcac140.threonine	0.006325265	0.02405712	0.793001904
pcaec361.pcaac363	-0.003510885	0.013353149	0.793002894
c12.c51	0.004825318	0.018357525	0.793059281
smc180.pcaec421	0.000353022	0.001343594	0.793142328
smc240.lysopcac140	0.000219194	0.000834367	0.793171414
pcaec343.pcaec405	-0.014903048	0.056734063	0.793189881
smc261.c12	0.009545081	0.036344592	0.793232393
pcaac425.threonine	0.004031258	0.015350089	0.793237114
lysopcac182.smc161	-0.012370649	0.047126052	0.793329326
c141.pcaec340	-0.006765455	0.025776843	0.793359151
smc180.alphaaminoadipicacid	-0.003952756	0.015060271	0.793359166
ornithine.pcaac360	-0.009891915	0.037690716	0.793369031
pcaec424.pcaec446	-0.008849646	0.033732961	0.793450177
pcaac366.c4	-0.003532461	0.01346752	0.793488134
pcaac404.c18	0.009870141	0.037630954	0.793493523

pcaec444.threonine	0.005326211	0.020310026	0.793526145
lysopcac180.pcaac383	-0.000964035	0.003676123	0.793528505
pcaac381.pcaac364	0.011943066	0.045559231	0.793604387
c51.h1	-0.000666577	0.002543009	0.793621583
pcaac425.smc181	0.002166868	0.008267892	0.793651695
pcaac361.ornithine	-0.004463268	0.017032366	0.793679354
smc202.smc180	0.006927996	0.026440672	0.793699242
pcaec341.pcaec342	-0.029421473	0.112313748	0.793747356
lysopcac180.pcaec446	-0.008053649	0.03074875	0.793778108
c141oh.pcaec445	0.017436422	0.066605206	0.793878325
glutamicacid.c51dc	0.035444353	0.135395097	0.793880679
pcaec443.ornithine	0.01472727	0.056257413	0.793881436
pcaec383.pcaec365	-0.004824653	0.018431144	0.793894761
pcaec363.smc180	-0.002498575	0.00954624	0.79391966
pcaec301.pcaec420	-0.004246354	0.016237843	0.794092175
asymmetricdimethylarginine.h1	0.000242102	0.000925962	0.794130343
pcaec446.isoleucine	-0.005565116	0.021286052	0.794142557
pcaec425.pcaac320	-0.015629147	0.059793255	0.794187039
pcaec423.pcaec322	-0.016896655	0.064647457	0.794202688
prolinr.glutamine	-0.004826227	0.018466349	0.794213174
pcaec322.threonine	-0.010967681	0.041985082	0.794308937
smc180.lysopcac281	0.005278398	0.020210872	0.794356499
pcaec322.pcaec424	-0.013021775	0.049864637	0.794374794
lysopcac281.lysopcac182	-0.003174675	0.012157996	0.794393428
lysopcac160.c10	0.008079407	0.030951303	0.794456653
pcaec443.c142	0.010448831	0.040033182	0.794481162
alanine.pcaec365	0.003775599	0.014465876	0.794484136
c51.putrescine	0.003739324	0.014327903	0.794498337
pcaec340.c142	0.011017488	0.042222218	0.794530198
pcaac343.c141oh	0.014860664	0.056958771	0.79455977
pcaac406.pcaec421	0.000564643	0.002165143	0.794647732
c51.pcaac363	0.00150934	0.00578839	0.794674539
smohc221.lysopcac140	-0.00024621	0.00094486	0.794808869
pcaac404.threonine	0.004450194	0.017086926	0.794912037
lysopcac170.pcaac380	0.014071456	0.054028784	0.794912586
pcaec385.c3	-0.003890228	0.014937381	0.794918688
serine.smohc241	-0.015898226	0.061055625	0.79495453
lysopcac181.pcaec321	0.011999722	0.046092521	0.794992478
pcaec364.pcaec423	0.001673076	0.006428426	0.795052291
serine.pcaac421	-0.009974602	0.038339684	0.795127936
c51dc.pcaac360	0.007118418	0.02736314	0.795141551
smohc141.pcaac386	0.002278944	0.008760965	0.795158059
pcaac424.glutamicacid	0.018883163	0.072597437	0.795171091
pcaac401.pcaac404	0.001596862	0.006141211	0.795235659
pcaec343.lysopcac140	0.00020054	0.000771602	0.79533073

c141.pcaec443	0.022314779	0.085891332	0.795406184
asymmetricdimethylarginine.smohc1	-0.012833728	0.04940699	0.795442471
pcaec402.asparticacid	0.010291398	0.039626257	0.795476136
pcaac383.c3	-0.010683967	0.041148357	0.795527357
smohc222.transhydroxyproline	0.007684897	0.029600791	0.795548207
pcaac364.h1	0.000243909	0.000939511	0.795552642
pcaec300.pcaac364	-0.01191914	0.045912396	0.795557303
c4.pcaec420	-0.009490281	0.036565063	0.795604353
pcaec384.smc260	0.007429552	0.028642741	0.795726101
smc160.pcaac383	-0.000639578	0.002466151	0.795760339
pcaec422.pcaec404	-0.004000524	0.015429693	0.795812706
pcaec404.pcaec424	0.009670908	0.037305174	0.795840972
pcaec403.pcaec425	-0.001439328	0.005553686	0.795895978
c4.glycine	0.006455629	0.024917898	0.795965471
smohc161.c3	-0.003630342	0.014013664	0.795979852
h1.threonine	0.005800192	0.022394081	0.796019696
c0.acetylornithoine	-0.020617054	0.079609381	0.796041168
pcaac281.alanine	-0.005622054	0.021715742	0.796106382
lysine.pcaac403	-0.001912415	0.007388897	0.796160391
pcaac300.valine	-0.010341689	0.03996784	0.796216284
pcaac240.pcaec365	-0.003356785	0.012975325	0.796250907
pcaec420.c162	-0.014327332	0.055383847	0.796261517
arginine.lysopcac140	0.000234779	0.000907619	0.796273725
pcaac381.pcaec361	0.006273853	0.024259058	0.796317383
pcaac362.pcaac322	-0.015873226	0.061401413	0.796396955
pcaec443.pcaac420	-0.020785935	0.080466571	0.796549432
phenylalanine.pcaac424	0.004483907	0.017359241	0.796562346
alanine.pcaac366	-0.002318391	0.008975591	0.796563361
smc240.lysopcac161	0.014660548	0.056777939	0.796633464
smc202.pcaac386	0.001668046	0.006461195	0.796667961
c5.lysopcac281	-0.003974606	0.015403875	0.796773751
pcaac405.pcaac342	-0.000953894	0.003697631	0.79681385
pcaec403.pcaec341	-0.006806133	0.026388533	0.796855593
pcaac342.pcaec363	0.00686736	0.026627151	0.796864788
c102.glutamicacid	0.007664196	0.029720145	0.796887405
c141.alphaaminoadipicacid	0.002186469	0.008479973	0.796917986
pcaec421.kynurenine	0.002403976	0.009324013	0.796927843
smc241.phenylalanine	-0.007280407	0.028237804	0.796928975
pcaec301.methionine	-0.006436266	0.024968038	0.796963352
pcaac341.pcaac406	0.009487525	0.036805775	0.796969114
pcaec386.transhydroxyproline	0.012585817	0.048834321	0.797006003
lysopcac281.pcaac366	-0.008788578	0.034102338	0.797015974
lysopcac204.pcaac360	-0.006282478	0.024379064	0.797025359
pcaac426.pcaac343	0.008029884	0.03116616	0.797065562
pcaec384.pcaac406	0.008946603	0.034759715	0.797268358

pcaec445.taurine	-0.005567227	0.021630534	0.797273028
pcaac321.serine	0.014791508	0.057476919	0.797297105
smc241.ornithine	-0.006607634	0.025680563	0.797332501
pcaac380.smc260	-0.009923186	0.03858652	0.797435565
lysopcac280.pcaec385	-0.010604471	0.041235838	0.797436177
c2.pcaac360	-0.006274875	0.024411428	0.797528435
lysopcac181.pcaec301	0.006487535	0.025243136	0.797562862
pcaac421.phenylalanine	-0.009019042	0.035102565	0.797615192
c51dc.phenylalanine	-0.013317514	0.0518335	0.797619277
smohc221.c2	0.011639885	0.04530444	0.797621413
pcaec443.pcaec421	-0.00233096	0.009076835	0.797716011
smohc241.pcaec425	0.001043751	0.004067207	0.797852539
pcaec385.pcaac362	-0.004120902	0.016059479	0.797870594
pcaec322.pcaec362	0.009304726	0.036279916	0.797972344
pcaac420.smc181	0.003713825	0.014480609	0.797973558
phenylalanine.pcaac360	-0.006046259	0.023577226	0.797992021
pcaac425.pcaac342	-0.0013021	0.005077904	0.798007502
pcaec421.smohc141	-0.003926538	0.015318265	0.798080204
pcaac401.asymmetricdimethylarginir	-0.007855186	0.030646695	0.798092653
pcaac405.c14	0.005117346	0.019986045	0.798299407
lysopcac170.c4	0.005571381	0.021765981	0.798359958
c4.c141oh	-0.020558473	0.080325597	0.798381636
c5.acetylornithoine	0.023218899	0.090724895	0.798391508
smohc161.smc260	-0.009293032	0.036318145	0.798428472
pcaec301.pcaac281	-0.000447778	0.001751038	0.798549306
pcaec343.pcaac342	0.001881256	0.007361645	0.798682863
lysopcac203.c141	-0.005336786	0.02088626	0.798707343
pcaac383.lysine	0.01203189	0.047089761	0.798712676
pcaac341.smc160	0.016197767	0.063396542	0.798720737
pcaac426.pcaac344	0.017546063	0.068690112	0.798767945
pcaec342.smc180	-0.001918785	0.007515192	0.798858206
lysopcac180.lysopcac181	0.02179725	0.085380643	0.798878141
pcaac342.pcaac360	-0.0050573	0.019810226	0.798884039
prolinr.c12	-0.005417221	0.021230101	0.798976787
pcaac425.pcaec380	0.011188251	0.043870551	0.799083356
acetylornithoine.pcaac425	0.01237106	0.048531365	0.799175979
c14.pcaac420	-0.014477104	0.056804757	0.799215531
pcaac406.pcaac424	-0.004856504	0.019055973	0.799217502
pcaac401.pcaec386	-0.00210492	0.00826131	0.799265433
pcaec382.smc161	0.02284771	0.089726023	0.799384062
pcaac321.pcaac342	-0.002256224	0.008862657	0.799431953
histidine.pcaec385	-0.011457329	0.045008273	0.799444264
alanine.asparagine	0.003006776	0.011811943	0.79944938
pcaec401.pcaec402	0.007467132	0.029340237	0.799489809
smc202.pcaac240	-0.001904685	0.007484704	0.79950865

c14.pcaec404	0.008554792	0.033631554	0.799592659
pcaec300.lysopcac281	-0.002858757	0.011243637	0.799679373
pcaec401.pcaac365	-0.012181527	0.047932802	0.799770291
pcaac300.pcaec406	0.002739806	0.010781519	0.799783388
pcaec385.putrescine	-0.012444977	0.0489807	0.799815323
c141oh.methioninesulfoxide	0.004138849	0.016289636	0.79981574
pcaac421.valine	-0.012979789	0.05109637	0.7998566
pcaec301.pcaec421	-0.000702803	0.002766838	0.799868957
histidine.threonine	0.003816821	0.015029963	0.799916857
pcaec341.methionine	-0.006752969	0.02660101	0.79998326
smc260.pcaec421	0.000311726	0.001228215	0.800027107
creatinine.methionine	-0.010645728	0.041964387	0.800119385
lysopcac280.pcaac361	-0.015310438	0.060369058	0.800173992
c5.ornithine	0.008328431	0.03283986	0.800179096
pcaac381.methionine	-0.012703396	0.050112015	0.800261883
serotonin.lysopcac204	-0.008442596	0.033304327	0.800263055
pcaac426.taurine	0.00361191	0.014255178	0.800358033
c18.pcaac364	-0.009349787	0.036947287	0.800603132
c141oh.ornithine	0.003859091	0.015254852	0.800667007
lysopcac161.pcaac360	-0.006856082	0.027102192	0.800669415
pcaac341.smohc221	0.011993667	0.047414815	0.800684547
pcaec421.arginine	0.011136421	0.04403828	0.800739633
c3.glutamicacid	0.006433924	0.025455822	0.800841312
pcaac380.transhydroxyproline	0.009938176	0.039334418	0.800910802
smc240.c3	0.0074412	0.02947703	0.80107885
c182.lysine	0.015256539	0.060445712	0.801109658
isoleucine.pcaec342	-0.020122664	0.079725759	0.801111324
pcaec322.c182	-0.012443361	0.049321623	0.801194885
asymmetricdimethylarginine.alphaan	0.002006011	0.007952196	0.80121915
pcaec342.c16	-0.011935838	0.047316419	0.801221459
c141.pcaec386	-0.000684963	0.002715947	0.801264189
pcaac364.transhydroxyproline	-0.005436799	0.021582161	0.801486831
smc160.pcaec384	-0.021105123	0.083787238	0.801503973
methioninesulfoxide.pcaec341	-0.005103794	0.020262264	0.801506137
pcaac300.pcaec385	0.009972347	0.039599289	0.801548711
pcaac384.pcaec363	-0.006181551	0.024549112	0.801570317
c8.pcaac424	0.001653917	0.006568353	0.801572301
c3.c141oh	-0.024465671	0.097182642	0.80161203
pcaec381.pcaec425	-0.00081589	0.003243054	0.801742012
methionine.smc202	-0.012171494	0.048382514	0.801751936
c0.lysopcac281	-0.003215011	0.012781457	0.801775761
c51.pcaec420	0.004698354	0.018679297	0.801783312
c10.c141oh	0.029166132	0.115973613	0.801812616
lysopcac170.c5	-0.003596153	0.014299587	0.801814846
pcaec443.pcaac320	-0.016306599	0.064878174	0.801926451

asparticacid.lysopcac182	-0.011455717	0.045587436	0.801965578
pcaac381.smc241	0.005955497	0.023708207	0.802036027
leucine.prolinr	-0.010013322	0.039872771	0.802088459
citruline.pcaec362	-0.003625423	0.014437609	0.802105576
pcaec321.lysopcac203	0.002873017	0.011442428	0.802124929
lysopcac180.pcaec382	-0.001142858	0.004551742	0.802127388
glycine.tryptophan	0.002349675	0.009358315	0.802129345
pcaec443.c12	0.014107447	0.05618819	0.802132353
pcaac404.c142	0.008691302	0.034626989	0.802191784
smc202.alphaaminoadipicacid	0.002501041	0.009964404	0.802192094
pcaac320.c16	0.008549831	0.034067835	0.802217264
pcaec424.methionine	0.00799408	0.031855318	0.802229042
lysopcac170.lysopcac280	-0.001666441	0.006641334	0.802252252
lysopcac140.pcaac362	-0.003132578	0.01248741	0.802299067
c14.c51	0.006336814	0.025263587	0.80232291
pcaec382.smc180	0.000553929	0.00220852	0.802333388
pcaac343.pcaec424	-0.004414029	0.017600506	0.802352308
histidine.pcaec403	-0.004833311	0.019274432	0.802373197
pcaec402.pcaac406	0.014402549	0.05745216	0.802431153
pcaec445.pcaac344	-0.011196347	0.044665376	0.80244348
smc161.glutamicacid	0.008787387	0.035063049	0.80248591
pcaec406.asymmetricdimethylarginir	0.003747366	0.01495376	0.80250123
pcaac343.smc181	0.001809857	0.007230605	0.802726398
pcaac405.putrescine	0.010302391	0.041166107	0.802758166
smc181.c12	0.013495519	0.053948228	0.80284079
pcaac366.valine	0.010328633	0.041305078	0.802917683
c3.acetylornithoine	0.013423859	0.053695281	0.802961279
methioninesulfoxide.methionine	0.006617996	0.026474362	0.802979167
c101.pcaac344	-0.011788042	0.047171469	0.803040782
pcaec445.pcaac364	-0.007700702	0.030815548	0.803041605
pcaac386.acetylornithoine	-0.006273715	0.025111009	0.803085921
pcaec342.smc240	0.005723592	0.022909859	0.803092298
pcaac320.pcaac424	-0.002094633	0.008384762	0.803105207
asparagine.pcaac361	0.013867221	0.055518999	0.803135998
c12.c18	0.009415246	0.037703381	0.803178793
pcaac380.c182	0.007286055	0.029182189	0.803212881
c51.pcaac323	-0.011695157	0.046851919	0.803255431
smc160.pcaec403	-0.005337923	0.021385591	0.803267696
lysopcac180.c141	-0.003715897	0.014887477	0.803271433
isoleucine.valine	0.038443304	0.154022624	0.803274279
pcaec384.pcaac366	0.001917019	0.00768118	0.803291057
pcaec402.pcaac360	-0.015933698	0.063844179	0.803292425
serine.pcaec340	0.0127747	0.051210533	0.80338282
pcaec444.pcaac321	0.005641124	0.022614541	0.803388828
smohc241.pcaac363	0.001643579	0.006590509	0.803435934

pcaec423.smohc221	0.02148642	0.086157828	0.803436953
pcaac322.asymmetricdimethylarginir	0.003678645	0.014751583	0.803445846
glutamine.c142	-0.010782756	0.043241191	0.803453436
alphaaminoadipicacid.smc160	-0.008818926	0.035366469	0.803457029
pcaec300.pcaec380	0.010871041	0.043596465	0.803458873
c3.ornithine	0.005647259	0.022648953	0.803472283
asymmetricdimethylarginine.pcaec34	0.010977233	0.044040224	0.803537075
c10.pcaec386	-0.000865956	0.003474269	0.803541888
pcaac344.h1	0.000306436	0.001229507	0.803552174
glycine.isoleucine	0.004059055	0.016293592	0.803641283
pcaac380.taurine	-0.011197913	0.044959741	0.80368325
c16.lysopcac161	0.01529139	0.061398381	0.803693545
smohc141.alanine	-0.007534176	0.030274831	0.803842225
pcaac385.smc260	-0.005959094	0.023956408	0.80392871
lysopcac281.pcaac361	-0.010327091	0.041543835	0.804055606
pcaec380.c142	0.009748189	0.039215591	0.804058379
citruline.tyrosine	0.009813449	0.03948723	0.804102635
leucine.ornithine	-0.009328743	0.037539556	0.804116325
pcaec385.pcaec340	-0.006535872	0.026304529	0.80414327
pcaec301.pcaec422	-0.003661241	0.01473526	0.804144388
pcaec341.pcaac405	-0.01417643	0.057058764	0.804155854
smohc161.smc241	0.012212588	0.04918832	0.804287785
ornithine.c4	-0.001660074	0.006693514	0.80449622
c8.pcaec405	-0.002498043	0.010073093	0.80451214
pcaec423.c51dc	-0.025600999	0.103245338	0.80453449
pcaac342.glutamicacid	-0.008398314	0.033871497	0.804547171
pcaec446.pcaac322	0.011539412	0.046544969	0.804567785
pcaac341.pcaac363	-0.002576487	0.010392818	0.804574993
smc261.pcaac380	0.012335799	0.04976405	0.804593955
pcaac240.pcaec402	-0.015032332	0.060651497	0.804623358
pcaac365.pcaec402	0.005869824	0.02368435	0.804632716
pcaac361.smc202	0.005583539	0.02253053	0.804643938
pcaec383.pcaec386	-0.001863905	0.007521822	0.804660393
glutamine.pcaac385	0.002042576	0.008243379	0.804672699
pcaac365.pcaac421	-0.010083062	0.040702825	0.804718941
c3.kynurenine	0.010432643	0.042130433	0.804793571
c3.asparticacid	-0.006166818	0.024909462	0.80483832
pcaac341.pcaec362	0.008771761	0.035456335	0.804972029
pcaac383.pcaac421	-0.009279084	0.037507966	0.804977019
pcaac381.pcaec384	-0.008839143	0.035741004	0.805037795
pcaec446.pcaac426	-0.016110457	0.065143094	0.805039523
serine.pcaec406	-0.013900778	0.056212877	0.805055435
glycine.c101	-0.001803017	0.007295678	0.805173771
c182.alanine	-0.003551747	0.014376668	0.80523985
pcaac401.smc180	0.000700964	0.002837895	0.805276771

lysopcac181.pcaac342	-0.001780055	0.00720685	0.80528197
pcaec422.pcaac281	-0.000651892	0.002639663	0.805308757
leucine.pcaac300	0.008144007	0.032977403	0.80531104
smc260.valine	-0.011783552	0.047718687	0.805325943
pcaac343.smc160	-0.018051641	0.073109067	0.805344517
pcaec444.c18	-0.006055221	0.024537142	0.805449619
pcaac341.smc202	-0.006156469	0.024953224	0.805493936
lysopcac160.pcaec362	0.003866521	0.015674951	0.805533786
c10.pcaac424	0.001526791	0.006190444	0.805558563
prolinr.smc161	0.013463833	0.05459137	0.805564271
ornithine.pcaec403	0.007384843	0.029948551	0.805599037
pcaac363.pcaac361	-0.025515259	0.103503023	0.805651002
smc240.pcaac320	-0.010930228	0.044343171	0.805670505
pcaac342.smc241	-0.002133242	0.00865537	0.805691554
leucine.pcaec402	0.007756445	0.031473006	0.805704685
pcaac300.arginine	-0.013444031	0.054551349	0.805704895
leucine.taurine	-0.00781341	0.031707258	0.805723329
pcaac321.smohc141	0.005550292	0.022525572	0.805741699
h1.pcaec422	0.010564907	0.042881024	0.805759009
c181.pcaac424	-0.00147987	0.006007815	0.805800013
c18.pcaac360	-0.006391547	0.025954056	0.805846554
smc180.pcaac320	0.007505864	0.030485032	0.805884519
asparagine.phenylalanine	-0.006451451	0.026206429	0.805912751
c51dc.lysopcac281	-0.004154021	0.016874806	0.805921388
c10.threonine	0.005744348	0.02334047	0.805964608
pcaac281.pcaec401	0.013847399	0.056309668	0.806115975
smohc241.pcaec384	0.012235073	0.049753682	0.80611768
pcaac404.pcaac321	0.007435595	0.03025993	0.806263532
c16.pcaec422	-0.005336624	0.021719152	0.806274053
creatinine.arginine	-0.009110801	0.037085486	0.806305141
pcaec422.glycine	-0.004631929	0.018855448	0.80631717
c14.lysopcac280	-0.00232875	0.00948172	0.806356217
pcaac363.c101	-0.001893953	0.007711675	0.806362843
pcaac380.lysopcac204	-0.006740339	0.027453668	0.806423643
c0.c102	0.004988621	0.020321265	0.806446185
alphaaminoadipicacid.c162	-0.014759458	0.060139963	0.806499686
pcaec446.smc181	-0.005285539	0.021537856	0.806508621
pcaec381.lysopcac160	0.004139752	0.016872242	0.806545847
smc260.prolinr	0.002843365	0.011591588	0.806594656
lysopcac181.alphaaminoadipicacid	0.0023419	0.009548475	0.806618829
c2.pcaec365	0.005699199	0.023241389	0.806654842
acetylornithoine.pcaac364	-0.011350252	0.046289712	0.80666824
pcaac426.pcaac360	-0.005895045	0.024046326	0.806704282
smc181.methionine	0.003959378	0.016158962	0.806802371
lysine.pcaec402	0.006626752	0.027052747	0.806856482

pcaec385.asymmetricdimethylarginir	0.007401661	0.03023553	0.806977486
pcaac320.pcaec384	-0.003878492	0.015843737	0.806980233
smc202.smc260	-0.006856759	0.028011753	0.806991861
pcaac383.pcaec420	-0.008591764	0.035108936	0.807041474
smc241.c4	-0.006108759	0.024971	0.807105742
pcaac240.smohc222	0.001705633	0.006973118	0.807131178
lysopcac281.putrescine	0.011231244	0.04593757	0.807217694
pcaec383.pcaac383	0.001954085	0.008000361	0.807402927
lysopcac182.smc240	-0.01202019	0.049225394	0.807451497
pcaec425.glycine	-0.003483621	0.014267494	0.807468389
pcaac365.isoleucine	0.006717613	0.027515107	0.807485535
pcaec362.c16	0.009453666	0.038737598	0.80756207
smohc161.pcaac320	0.009406714	0.038556739	0.807618507
smc241.pcaec425	0.000944912	0.003873698	0.807649822
pcaec424.putrescine	-0.010392417	0.042612287	0.807686183
pcaec443.pcaac360	-0.01026864	0.042113397	0.807724849
pcaac364.arginine	0.008135042	0.033363188	0.80772501
pcaac380.pcaec343	0.021903343	0.089846825	0.807761724
pcaec443.c18	-0.010917377	0.044802071	0.807843039
lysopcac160.lysopcac140	0.000849226	0.003485199	0.80785361
pcaac360.pcaec405	-0.016293198	0.066870589	0.807864306
glycine.pcaac364	0.011550067	0.047416842	0.807916036
pcaac360.pcaec301	0.016136137	0.066246365	0.807922263
citruline.pcaec402	-0.007827939	0.032141132	0.807944475
pcaac360.phenylalanine	0.006266362	0.025732503	0.807967378
pcaec420.taurine	0.004429171	0.018190373	0.807990191
smohc161.pcaac361	0.009067493	0.0372405	0.807994128
asparagine.glutamicacid	-0.009920143	0.040745167	0.808007074
smc240.c102	0.0056738	0.023317145	0.808112448
smohc222.pcaac364	0.009540764	0.039238367	0.808253762
acetylornithoine.pcaec403	-0.010033639	0.041276534	0.808304392
c101.pcaec405	-0.005190027	0.021359193	0.808378143
c51dc.smc240	-0.005187825	0.021351935	0.808394026
methioninesulfoxide.c142	-0.006317359	0.026008592	0.808449946
pcaac424.c14	0.018190901	0.074899258	0.808468129
pcaec406.pcaec341	0.008200794	0.0337694	0.808487211
lysopcac204.pcaac281	0.00074833	0.003081661	0.808497704
c51.glutamicacid	0.014077213	0.057988229	0.808554516
histidine.methioninesulfoxide	0.002493431	0.010273666	0.80859987
smohc161.pcaac323	0.019240758	0.079286769	0.808621656
acetylornithoine.smc180	-0.012297976	0.050685133	0.808651218
pcaac364.pcaec402	-0.004891958	0.020162247	0.808655222
c10.pcaec361	-0.002016092	0.008310627	0.80868427
prolinr.pcaac360	-0.004011395	0.016536821	0.808698586
pcaac360.smc180	-0.001367536	0.005639045	0.808746209

c102.smohc241	0.015198173	0.062670526	0.808748375
serotonin.lysine	-0.012192393	0.050286442	0.808787229
pcaec444.c4	-0.003708111	0.015294943	0.808801568
leucine.c102	0.002978074	0.012291867	0.808925506
pcaac402.methioninesulfoxide	0.005831911	0.02407317	0.808942776
alphaaminoadipicacid.asymmetricdin	-0.014407941	0.059480488	0.80896442
c16.lysopcac182	-0.013568579	0.056016979	0.808969895
c4.pcaac402	-0.021057386	0.086948111	0.809000246
arginine.tyrosine	-0.014972466	0.061840233	0.809052879
c16.pcaac360	0.008572193	0.035412695	0.809091351
pcaac322.pcaec420	-0.007383826	0.030504891	0.809100406
c16.lysopcac203	0.003983613	0.01645956	0.809123252
pcaac405.pcaec362	-0.003759598	0.015537792	0.809169274
c51dc.smc161	0.015049406	0.062248396	0.809324723
lysopcac140.lysopcac204	-0.007081243	0.029289956	0.809324915
pcaec385.c182	0.005126628	0.021213451	0.809398226
pcaac363.lysopcac203	-0.004500716	0.018626363	0.809427015
lysopcac203.pcaec362	-0.004394348	0.018189274	0.809459066
pcaac322.pcaec446	-0.016811113	0.06965924	0.809657427
smc161.methionine	0.004840425	0.020065677	0.80973823
pcaac384.c12	0.011470841	0.047556556	0.809757475
c101.pcaec403	0.004237222	0.017567709	0.809765571
pcaec300.smc240	-0.006994305	0.029015514	0.809873683
c8.pcaec361	-0.001788024	0.007418561	0.809899722
methioninesulfoxide.taurine	-0.012961156	0.053794931	0.809964632
pcaac323.pcaec383	-0.013831992	0.057432899	0.810041158
lysopcac280.pcaac343	-0.004132748	0.017161758	0.81006126
smohc161.c18	-0.006743732	0.028008141	0.810087473
pcaec381.transhydroxyproline	0.016859169	0.070025049	0.810101737
alphaaminoadipicacid.glycine	0.009429355	0.03917806	0.810163347
c0.pcaec343	-0.012853742	0.053427793	0.810239091
isoleucine.pcaac425	-0.01879241	0.078135053	0.810292979
smohc241.pcaac240	-0.002436925	0.010134974	0.810343151
pcaac363.c51	-0.022326054	0.092865186	0.810369011
c162.lysopcac180	0.007299643	0.03036903	0.810406844
prolinr.c141oh	-0.029671639	0.123516269	0.81051527
c101.c51	-0.010832772	0.045109332	0.810576949
smc161.c142	-0.008073271	0.033622416	0.810599457
smohc241.pcaac424	0.001465964	0.006106222	0.810629304
prolinr.pcaec444	0.02109753	0.087946503	0.81077371
pcaac386.taurine	0.005400971	0.022515137	0.81078047
pcaec425.serine	-0.00453061	0.018891438	0.810825553
pcaec444.pcaec365	0.003529146	0.014716749	0.810840062
acetylornithoine.glutamine	0.003910891	0.016308899	0.810842924
pcaac386.pcaec383	-0.014445025	0.060239032	0.810847515

arginine.pcaac366	0.013535622	0.056455064	0.810875279
pcaac426.c142	0.006358339	0.026538916	0.811009699
pcaec443.pcaec420	0.005823836	0.024309677	0.811022733
pcaec363.pcaec423	0.003540748	0.01478105	0.811039914
c102.c141oh	-0.025641609	0.107134286	0.811199001
pcaec381.pcaec421	-0.002035448	0.008509394	0.811307923
c102.h1	0.001185756	0.004958294	0.811349783
alphaaminoadipicacid.pcaac384	-0.010476222	0.043815554	0.811386726
lysine.putrescine	0.010942874	0.045768899	0.8113933
pcaec402.pcaac404	0.003051413	0.012766812	0.811453962
putrescine.pcaec386	0.00136595	0.005715544	0.811471496
c16.pcaac361	0.007936804	0.033225112	0.811555851
lysopcac204.pcaac361	0.008195492	0.034308552	0.811558639
pcaac406.smohc241	0.009380373	0.039281706	0.81161946
pcaac401.pcaec406	-0.010705537	0.044852414	0.811707571
pcaec301.c101	-0.002368642	0.009924826	0.81172722
glutamicacid.pcaac420	0.013688286	0.057358402	0.811737597
c142.lysopcac161	0.017476253	0.073266433	0.811826362
pcaec340.smc260	-0.005605727	0.023501997	0.811833196
leucine.c12	-0.006989291	0.029303166	0.811836856
pcaec402.threonine	-0.006164798	0.025852264	0.811878634
glutamicacid.pcaac383	0.001570877	0.006587562	0.811879804
kynurenine.pcaac406	0.010847688	0.0454981	0.811911071
pcaac383.pcaac364	0.011385936	0.047782434	0.812014498
pcaac386.transhydroxyproline	0.004828659	0.020266263	0.812034702
c141.lysopcac170	-0.00367089	0.015407754	0.812043571
lysopcac140.pcaec384	0.006073231	0.025502341	0.812125168
c181.c8	-0.012859071	0.054050718	0.812308284
pcaec360.pcaec422	-0.004441014	0.018683883	0.812474916
c101.smohc141	0.004915372	0.020681155	0.812489069
pcaec342.pcaac364	0.009730348	0.040945597	0.812514652
c162.acetylornithoine	-0.003960691	0.016679328	0.812653846
c102.pcaac404	-0.001021351	0.004301249	0.81265903
pcaac403.threonine	-0.004451218	0.018745906	0.812662327
c5.smc240	-0.022178917	0.093417732	0.812688314
pcaac406.c51dc	-0.02335481	0.098404705	0.812752011
c182.pcaec343	0.012441776	0.05242874	0.812772118
pcaec361.lysopcac281	-0.006728249	0.028367993	0.812873398
c10.acetylornithoine	-0.005530218	0.023322629	0.812919368
asparticacid.pcaec425	0.001275423	0.005380502	0.812975738
pcaec404.smc180	0.000737061	0.00311022	0.813026106
pcaac341.methionine	-0.007182512	0.030314108	0.813060156
c141oh.asparticacid	0.007674329	0.032406576	0.813154855
c2.smohc241	0.008736065	0.03689406	0.813175067
pcaac362.pcaac323	0.013572568	0.057335266	0.81322539

lysopcac203.c8	0.008955858	0.037833465	0.813229227
pcaac381.lysopcac204	-0.013747226	0.058077532	0.813239436
pcaac320.pcaac384	0.014954383	0.063178942	0.813243978
c101.pcaec404	-0.004340145	0.018337025	0.813252755
pcaec363.lysopcac281	0.004058401	0.017146812	0.813254347
pcaac281.valine	0.007929535	0.033514696	0.813321488
pcaac402.pcaac361	-0.013539063	0.057223984	0.813322298
pcaec362.pcaac383	0.002238803	0.009462522	0.813322965
methioninesulfoxide.pcaec386	0.003021428	0.012770452	0.813324237
lysopcac182.pcaac401	-0.000875189	0.003699442	0.813341234
pcaac380.c8	-0.009014703	0.038106416	0.813346429
pcaec403.pcaec406	-0.009248658	0.039110674	0.813418067
pcaac363.c16	0.008128392	0.034378139	0.813443843
pcaec383.methioninesulfoxide	0.004269513	0.018062108	0.813491209
pcaec386.pcaac360	0.012092265	0.051163522	0.813517626
smc240.pcaac300	0.010932294	0.046258195	0.813527997
pcaec341.pcaec402	0.012947824	0.054793754	0.813551958
prolinr.pcaec423	0.002159685	0.009139709	0.813555172
c5.pcaac322	0.017820794	0.075420095	0.813562816
methionine.c141	0.006851005	0.029005378	0.813631991
pcaac341.c3	-0.006667616	0.028229008	0.813632335
pcaac360.alphaaminoadipicacid	-0.001132274	0.004795051	0.813681407
pcaec443.pcaec383	-0.032237782	0.136566189	0.813738841
pcaac240.pcaec421	-0.001812992	0.007681552	0.813770478
citruline.lysopcac180	-0.006829702	0.028951182	0.81385941
pcaec386.pcaec362	-0.003467408	0.014701692	0.813900532
putrescine.pcaac323	0.016136201	0.068499099	0.814119622
pcaac425.smc260	-0.006502489	0.027604475	0.814126468
pcaac362.pcaec385	-0.013477004	0.057216429	0.814138029
c14.lysine	0.010111725	0.042940248	0.814185033
spermidine.asparagine	-0.008107112	0.034428672	0.814191257
smc240.glycine	0.006802752	0.028891578	0.814204905
pcaac421.pcaec384	0.003723537	0.0158193	0.814265759
smc160.alphaaminoadipicacid	-0.001695559	0.00720632	0.814336705
pcaec363.ornithine	0.009939182	0.042244608	0.814345112
pcaac385.pcaac364	-0.014421983	0.061304223	0.814363898
prolinr.pcaac426	0.014871718	0.06322288	0.814383924
smc202.smc161	0.019635076	0.083529664	0.814507642
serine.pcaac361	0.012197968	0.051919132	0.814604786
c12.h1	0.000533846	0.002273538	0.814707836
pcaac342.pcaac425	0.020460067	0.087153061	0.8147454
c4.lysopcac203	-0.003321393	0.014151021	0.814783873
pcaec423.c182	0.006593977	0.028094103	0.814783979
pcaec443.pcaac383	-0.001647467	0.007019312	0.814788149
putrescine.lysopcac181	0.005929966	0.025270748	0.81482505

pcaac426.creatinine	0.003943839	0.01681179	0.814879008
pcaac362.lysopcac170	-0.001805317	0.007700272	0.814986871
pcaac343.c12	0.00810109	0.034560307	0.81502095
asparagine.methionine	-0.007720071	0.03293517	0.815022826
lysopcac140.pcaec423	0.001972789	0.008416815	0.815034709
pcaec404.pcaec425	-0.001933971	0.008258276	0.815190317
pcaec382.asparagine	0.003228903	0.013788804	0.815203773
lysopcac140.pcaec386	0.001836565	0.00784372	0.81522225
glutamine.glycine	0.004772698	0.020395675	0.815330111
pcaec381.c5	-0.003538296	0.015124281	0.815374564
pcaec445.smohc141	-0.003904203	0.016695399	0.815451274
c181.pcaec445	0.0160403	0.068597434	0.815464176
pcaec362.pcaec386	-0.002206454	0.009440396	0.81554759
smc240.pcaec362	0.003392526	0.014520886	0.815620345
pcaac364.c182	0.005093647	0.021807176	0.815662311
pcaac363.glycine	0.012333231	0.052803888	0.81567
pcaac323.lysopcac281	-0.004062507	0.017411921	0.815863088
smc181.pcaec423	0.002420923	0.010377592	0.81588935
pcaec322.lysopcac182	0.0055776	0.023932719	0.816067823
smc260.pcaec343	0.01238657	0.053152723	0.816080208
pcaac403.pcaec365	-0.004319298	0.018537701	0.81610861
pcaec342.pcaec380	0.005413557	0.023234634	0.81611292
pcaac426.c101	-0.000971903	0.00417311	0.816189205
pcaac403.pcaec340	-0.010017399	0.043015982	0.816205089
c162.phenylalanine	-0.006262608	0.026893912	0.816215012
pcaac281.pcaac421	0.014960755	0.064252488	0.816230672
pcaac343.pcaec444	0.018739773	0.080498507	0.816266842
lysopcac161.alanine	0.009577441	0.041154601	0.816327236
c142.pcaec343	-0.012946073	0.055638594	0.816355965
pcaec401.c102	-0.005485803	0.0235772	0.816361753
c181.tryptophan	0.00593732	0.025521721	0.816389776
pcaec386.lysopcac182	-0.002164799	0.009305981	0.816400161
pcaec362.pcaec420	0.003939909	0.016942424	0.816460259
c141.pcaac360	-0.006021293	0.025894208	0.816470044
pcaec343.pcaac406	0.008271252	0.035579591	0.816518509
pcaac405.tyrosine	-0.009818414	0.04223768	0.816530537
putrescine.pcaac361	-0.008718573	0.037513801	0.816566605
pcaac385.pcaec425	-0.00163889	0.007054945	0.816648734
c4.pcaec405	-0.005534238	0.023866093	0.816971765
pcaac383.pcaac362	0.006387855	0.027551834	0.817001528
pcaac403.pcaec445	0.019678267	0.084888664	0.817029416
pcaec365.lysopcac204	-0.005615379	0.024226169	0.817047147
pcaac240.c14	0.008528743	0.036798221	0.817062088
pcaac385.pcaac426	-0.011649522	0.050265956	0.817072024
creatinine.pcaac360	0.004407801	0.019030411	0.817179712

pcaec421.smc180	0.000673559	0.002908219	0.817190004
smohc222.smohc141	-0.02796556	0.120777787	0.817236475
pcaec301.pcaac422	-0.007392261	0.031928461	0.81725184
pcaec402.lysopcac182	-0.011155005	0.048202808	0.817335232
pcaac426.isoleucine	-0.007201351	0.031122768	0.817360717
asymmetricdimethylarginine.threonir	-0.005513771	0.023829688	0.817362928
c5.smc161	0.026013048	0.112457087	0.817414953
c2.methioninesulfoxide	0.003088364	0.013356199	0.817480499
smc260.pcaac380	0.004426163	0.019150915	0.817566261
pcaec424.pcaac425	-0.017780498	0.076935359	0.817574472
isoleucine.pcaac366	-0.003060313	0.013243741	0.817600367
pcaec340.c14	-0.007894981	0.034171049	0.817626103
smc260.c16	-0.01008248	0.043643349	0.817644031
asparticacid.c10	-0.002666729	0.011546909	0.817700176
pcaac405.pcaec342	0.021368165	0.092560409	0.817770747
pcaac344.pcaac385	-0.006750653	0.029242652	0.817776061
alphaaminoadipicacid.pcaec444	-0.01262683	0.054703772	0.817797494
lysopcac182.c10	0.004917244	0.021322628	0.817960621
pcaac384.methioninesulfoxide	0.002409524	0.010449956	0.81798709
putrescine.smc240	-0.007071731	0.030669871	0.817988296
pcaec385.pcaec365	0.010154808	0.044047949	0.818016191
acetylornithoine.lysopcac182	-0.016125111	0.069989104	0.818128843
lysopcac182.pcaec382	-0.001571213	0.00682313	0.81821973
pcaac281.pcaec340	-0.013681144	0.059439407	0.818303314
lysopcac170.taurine	-0.006147332	0.026713684	0.818342368
methioninesulfoxide.smohc241	-0.015048289	0.065402417	0.818366848
c141oh.pcaec340	0.009406636	0.040892108	0.818407314
isoleucine.lysopcac280	-0.001940506	0.008437448	0.818444674
leucine.pcaec385	-0.01109361	0.048240249	0.818461315
pcaac344.pcaec423	0.001484069	0.006453907	0.818474461
pcaec361.lysopcac160	0.01300349	0.05656655	0.818528274
pcaac344.taurine	0.002347831	0.01021387	0.818538068
pcaec382.asymmetricdimethylargini	0.003893644	0.016943201	0.818585511
pcaec425.pcaac385	-0.008439244	0.036724621	0.818591497
pcaec382.c3	-0.010457914	0.045515054	0.818614561
pcaec401.serine	0.012766494	0.05556312	0.818616612
pcaec424.transhydroxyproline	-0.009072061	0.039503837	0.818706177
lysopcac180.h1	-0.00027437	0.001194785	0.818714294
pcaec341.tyrosine	-0.010579579	0.046075028	0.818732372
pcaec382.pcaac320	-0.013274187	0.057823884	0.818774238
c2.lysopcac203	-0.002202723	0.009603791	0.81893137
c4.pcaec322	0.014657507	0.063911823	0.818947065
putrescine.pcaac281	-0.000229785	0.001002634	0.819069754
pcaec420.pcaac420	0.020676343	0.090245624	0.819123644
c102.smc241	0.004481374	0.019560422	0.81912964

lysopcac160.pcaec421	-0.001079977	0.004714179	0.819139599
lysopcac281.pcaac322	0.006438857	0.028112845	0.819182409
pcaac420.pcaec301	0.012505571	0.054619212	0.819242069
smohc221.pcaac426	-0.005259032	0.02296956	0.819244085
pcaac362.pcaac342	0.008688366	0.037957158	0.819288553
c0.c141	-0.010263584	0.044851631	0.819339075
pcaec422.spermidine	-0.01191954	0.052096209	0.819366631
c3.valine	0.013850875	0.060541085	0.819377408
glycine.pcaac422	-0.00640068	0.027981287	0.819405433
pcaec421.lysopcac182	0.018925145	0.082758297	0.819458847
pcaec443.pcaec424	0.007827717	0.034246858	0.81954596
pcaac363.asymmetricdimethylarginir	-0.004534503	0.01984553	0.819606127
creatinine.isoleucine	-0.004711081	0.020623274	0.819648565
pcaac403.methioninesulfoxide	0.007707114	0.033749661	0.819705925
smc161.lysopcac182	-0.005250353	0.023012324	0.819866719
lysopcac203.pcaac384	-0.00823987	0.036128106	0.819929011
pcaac343.histidine	0.012380126	0.054293263	0.819968139
smc241.pcaac321	-0.003915845	0.017177368	0.82001303
pcaec363.threonine	0.00654553	0.028714621	0.820024112
pcaec364.pcaec322	-0.02590684	0.113653305	0.82002791
lysopcac140.c10	0.00172833	0.007582216	0.820028723
methioninesulfoxide.pcaec425	-0.001327836	0.005826719	0.820073446
pcaac404.smohc241	0.009369812	0.041133712	0.820149796
c142.pcaec385	-0.010875906	0.047746915	0.820155023
pcaac360.smohc141	-0.006559634	0.028800059	0.820168823
pcaec301.lysopcac182	0.003298526	0.014484336	0.820195234
c142.c2	-0.010389032	0.045622736	0.820206438
pcaac362.c14	0.004912735	0.021574632	0.820212077
pcaac403.valine	0.005076593	0.022303542	0.820285862
pcaec361.smohc241	0.015311671	0.067276822	0.820302686
lysopcac281.pcaac383	0.000712844	0.003136535	0.820551774
smc241.pcaec386	0.002205919	0.009707261	0.820572613
c3.c16	0.006116992	0.026923775	0.820609494
pcaac343.alphaaminoadipicacid	-0.002033879	0.008956487	0.820696557
c51dc.methioninesulfoxide	0.006572468	0.028945098	0.820710357
pcaac401.glutamicacid	0.016511151	0.072746523	0.82078676
prolinr.pcaac344	0.015850816	0.069852833	0.820826305
c3.smohc141	-0.003567169	0.01573109	0.820949002
pcaec363.taurine	0.010340086	0.045625449	0.821049431
c4.pcaac381	0.007409039	0.032699927	0.821090687
pcaac322.pcaec402	0.005644201	0.024922165	0.82117099
c10.c51	-0.012570033	0.055507187	0.821182945
smohc241.pcaec421	0.000313342	0.001384137	0.821242864
pcaac405.c142	0.008650676	0.038216025	0.821256854
c5.pcaac383	0.001590827	0.007029882	0.821309297

pcaec420.pcaec361	0.003126679	0.013816998	0.821311441
lysopcac160.pcaec423	0.005460041	0.024134188	0.821354466
histidine.c51	-0.009463674	0.041830985	0.821355073
serotonin.c3	-0.001500414	0.006635279	0.821439849
c51.isoleucine	0.003664785	0.016213548	0.821513194
pcaac360.c51	-0.00924422	0.040950453	0.821738836
pcaac364.smc241	-0.003299912	0.014619201	0.821752104
pcaac424.kynurenine	0.002550676	0.011301914	0.821782498
c0.pcaac421	-0.008671943	0.038450711	0.821899973
alanine.smc202	-0.00793782	0.035204022	0.821941515
phenylalanine.pcaac364	-0.013693055	0.060733966	0.821957731
serine.pcaac385	-0.003347248	0.014847326	0.821969463
pcaac424.smohc241	-0.011164086	0.049523093	0.821979222
pcaac405.pcaac424	0.001088207	0.004827521	0.821990587
pcaec383.c3	-0.00795438	0.03529165	0.822011863
glutamicacid.c8	0.010125894	0.044972274	0.822191411
pcaec445.pcaac343	0.004151667	0.018443686	0.822237157
pcaac300.c16	-0.008630103	0.038340386	0.822243342
pcaec360.pcaac342	0.004963271	0.022080718	0.822486494
serine.histidine	-0.012272323	0.05464051	0.82262419
c141oh.serotonin	0.008580712	0.03821041	0.822652465
pcaec322.pcaac386	-0.002438229	0.010858883	0.822673429
c5.c162	-0.018345818	0.08172484	0.822716128
isoleucine.taurine	-0.006986998	0.031132666	0.822759755
methionine.pcaec385	0.008006886	0.035679447	0.822771305
pcaec381.pcaec340	-0.009059785	0.040409733	0.822937187
c181.c4	0.006864138	0.030618941	0.822951634
pcaac361.pcaac321	-0.009016216	0.040220624	0.82295978
pcaec321.pcaac344	0.01043364	0.046555691	0.823004842
lysopcac170.pcaac404	0.000568516	0.002536989	0.823020305
lysopcac203.isoleucine	0.006851847	0.030577512	0.82302792
pcaac364.pcaac384	0.015847407	0.07073798	0.823067961
pcaac281.pcaec420	0.003279546	0.014648407	0.823180979
pcaec343.lysopcac160	-0.005597408	0.025001419	0.823181384
pcaec386.asparagine	-0.001949672	0.008709479	0.823202562
asparticacid.glutamine	0.003824096	0.017086157	0.823236605
smc261.c142	0.009094564	0.040652808	0.82331384
smc180.taurine	0.010898329	0.048719351	0.823327014
c162.pcaec425	-0.001038729	0.004644635	0.823370029
lysopcac204.pcaec386	0.003052658	0.01365061	0.823379913
smohc161.taurine	0.010527048	0.047094913	0.823457299
glycine.asparagine	-0.001662342	0.007440234	0.823536754
lysopcac161.pcaec404	-0.006758363	0.030252293	0.823556965
pcaec300.isoleucine	-0.004473235	0.020028342	0.823599501
smohc222.c2	-0.010833919	0.048532772	0.823689832

pcaac406.smohc222	0.001479441	0.006628612	0.823719949
pcaac362.pcaac424	-0.002962495	0.013275927	0.823752807
lysopcac170.c8	-0.008917731	0.040005835	0.82393698
c5.pcaac426	0.011756745	0.052759605	0.823994952
pcaac404.pcaec405	0.009156081	0.041100449	0.824043778
smohc161.pcaec301	-0.011739537	0.052716521	0.824107055
pcaac405.pcaec343	-0.019157476	0.086029535	0.824112434
smohc161.pcaac406	-0.00807944	0.036293734	0.824168619
lysopcac170.lysopcac161	0.013990907	0.062857162	0.824191925
pcaec403.smohc141	-0.004984855	0.02240379	0.824255643
c141oh.c2	0.015126835	0.067987063	0.824259309
phenylalanine.acetylornithoine	-0.01867691	0.083956707	0.824288029
pcaec341.pcaec382	-0.00319013	0.014349476	0.824398364
pcaac281.pcaec386	-0.002749943	0.012372057	0.824434374
smc160.c12	0.010254624	0.046139289	0.824447442
pcaec382.c162	-0.021847027	0.098327835	0.8245003
pcaec343.taurine	-0.009941327	0.044752837	0.824536888
smc181.pcaac424	-0.004077731	0.018358451	0.824553268
pcaac364.pcaec421	0.000235059	0.001058514	0.824594238
pcaec383.c18	0.00864281	0.038948952	0.824721407
pcaac406.c142	0.008867517	0.03996775	0.824747962
pcaec365.pcaac343	0.00461437	0.02079899	0.82475673
serotonin.glutamine	0.002537941	0.011441373	0.824783254
methioninesulfoxide.threonine	-0.005624217	0.02536202	0.824832922
c16.alphaaminoadipicacid	0.001761713	0.007946089	0.824871232
c8.c18	-0.005699439	0.025718899	0.824951299
smc202.pcaec301	-0.011497452	0.051911125	0.825045853
c51dc.c3	-0.01411826	0.063753334	0.825070775
c51.pcaec423	0.002477035	0.011190455	0.825147551
lysopcac161.c182	0.009240908	0.041764096	0.82521601
lysopcac140.c2	-0.009279696	0.041957162	0.825288795
pcaac405.pcaec444	0.01701132	0.076927922	0.825317939
pcaac300.smohc241	-0.008896568	0.040254601	0.82541566
pcaac240.pcaec425	-0.000943628	0.0042705	0.825449225
c181.pcaec381	0.0111015	0.050264728	0.825529765
phenylalanine.pcaac362	-0.007611465	0.034480309	0.825617167
lysopcac204.smc181	0.003783795	0.017141218	0.825621578
pcaec362.lysopcac281	-0.005165405	0.023404833	0.82565597
c16.pcaec446	0.014267501	0.064683084	0.825751382
smohc221.methionine	0.004405193	0.019973123	0.825766456
smc241.pcaac361	-0.011891023	0.053919228	0.825783609
pcaac424.c102	-0.00687991	0.031203661	0.825822418
pcaac343.taurine	0.003019465	0.013698604	0.825871122
lysine.lysopcac280	-0.002132619	0.009677043	0.825903905
asparticacid.asymmetricdimethylargi	-0.003951614	0.017934073	0.825933506

c18.pcaac384	0.01023547	0.046457119	0.825949292
pcaac426.smohc241	-0.008439629	0.038308275	0.825959054
pcaec401.pcaac425	0.020702661	0.094000321	0.826011847
pcaec322.pcaec421	-0.000415845	0.001888215	0.826018426
pcaec342.c182	0.007779369	0.035328544	0.826042671
pcaac384.lysine	-0.01288289	0.058511258	0.826060309
lysopcac204.pcaec406	-0.013864053	0.063106251	0.826436628
pcaac386.lysopcac182	-0.00209461	0.009541599	0.8265686
smohc141.c0	0.000106913	0.000487046	0.826576287
c5.smc260	0.010043141	0.045758232	0.826600724
isoleucine.pcaac420	-0.011253011	0.051307081	0.82672203
lysopcac281.pcaec382	-0.005990484	0.027320877	0.826770765
pcaec384.acetylornithoine	-0.012790296	0.058334852	0.826776559
asparticacid.pcaac361	0.011110666	0.050697895	0.826855959
h1.pcaac420	-0.0229356	0.104670784	0.826881658
serotonin.pcaec421	0.001124299	0.005131682	0.826906118
smohc221.serotonin	0.015739508	0.071840598	0.826906531
pcaac341.pcaac426	0.011740393	0.053588097	0.826909246
lysopcac182.pcaec446	-0.005533375	0.025258428	0.826921216
c182.smc260	-0.003068186	0.014012182	0.827002689
pcaac361.pcaec365	-0.004732594	0.021614324	0.827009876
alphaaminoadipicacid.pcaac322	-0.009716033	0.044375044	0.827012801
pcaec384.pcaac426	-0.009306895	0.042524128	0.82708364
pcaac403.pcaac383	0.001218841	0.005569246	0.827091002
c181.pcaac323	-0.013437526	0.061410365	0.827119586
lysopcac203.pcaac300	-0.008199828	0.037480215	0.827148961
c51.methionine	-0.003416051	0.01561441	0.827150484
leucine.c142	-0.012878822	0.058881332	0.827189742
pcaec443.pcaec365	-0.005797766	0.026510224	0.827209805
pcaac402.smc160	-0.011378885	0.052053642	0.827287568
pcaac321.c12	0.006619675	0.030285875	0.827307941
smohc222.pcaac361	-0.009166269	0.041938109	0.827312951
pcaac383.c101	0.001442569	0.006600582	0.827324502
smc260.pcaec386	-0.001926952	0.00881692	0.827324601
pcaec383.pcaec403	-0.025018584	0.114477634	0.827329246
pcaec343.pcaac361	-0.012984384	0.059423016	0.827358753
prolinr.c102	-0.004615105	0.021129082	0.827423581
prolinr.pcaec363	0.004899711	0.022440231	0.82748527
pcaac364.pcaac366	0.002105915	0.00964665	0.827516098
pcaac240.ornithine	0.016959677	0.077713878	0.827572864
pcaec383.kynurenine	0.001489171	0.006824423	0.827588695
pcaec322.smc240	0.006615062	0.030315533	0.8275925
pcaec422.c182	0.004422612	0.020269621	0.827606375
pcaec445.c12	0.007043672	0.03229668	0.827681354
serotonin.leucine	0.005365538	0.024602287	0.8276827

pcaec385.smc260	0.005224794	0.023957161	0.827684243
pcaec382.alanine	0.004340179	0.019913429	0.827790524
smc180.pcaac240	-0.001963882	0.009011105	0.827799979
c162.serotonin	0.011890967	0.054578675	0.827855836
pcaec321.pcaec301	-0.010301921	0.047314532	0.827961343
c51dc.pcaac321	-0.009520729	0.043736786	0.828000447
pcaec425.leucine	-0.006350096	0.029202911	0.828183251
c51.arginine	0.003195214	0.01469709	0.828216535
lysopcac180.pcaec405	-0.007920992	0.036444349	0.828262956
pcaec385.pcaec322	-0.014365679	0.06612305	0.828331479
pcaec360.smc261	0.009343817	0.043014613	0.828356764
glutamine.smc161	-0.009476495	0.043626051	0.828359283
lysopcac182.pcaac361	-0.008509338	0.039176205	0.828370336
arginine.c101	0.001265665	0.005828681	0.828418985
c16.lysopcac281	0.002671133	0.012303637	0.828452554
smc261.valine	0.007953336	0.036635509	0.828458332
pcaac362.asparagine	0.003330743	0.015349507	0.828536245
pcaac381.pcaac420	0.022255123	0.102608119	0.828613326
lysopcac180.asymmetricdimethylarg	-0.00352427	0.016249709	0.828622901
pcaec403.smohc241	-0.012566928	0.057952852	0.828649815
pcaec362.alphaaminoadipicacid	-0.001317693	0.006080518	0.828758752
pcaac322.pcaac384	-0.012355058	0.057037839	0.828833262
kynurenine.methionine	-0.00585925	0.027052963	0.828854411
pcaec362.pcaac362	0.002634566	0.012166094	0.828881255
pcaec364.pcaec382	-0.006078443	0.028088467	0.828995133
c14.h1	0.000433537	0.002004401	0.829081435
asparagine.pcaac363	-0.001696955	0.007846134	0.829091869
pcaac366.asymmetricdimethylarginir	0.005108464	0.023625139	0.829130106
pcaac401.kynurenine	0.002315652	0.010711975	0.829173556
pcaec321.pcaec402	0.010359372	0.047927635	0.829195347
pcaec384.pcaac300	-0.011081258	0.051277549	0.829228496
pcaec341.pcaac300	-0.013809294	0.063936046	0.829319874
pcaac406.c101	0.002002504	0.009275037	0.829384833
pcaac404.h1	-0.000263666	0.001221311	0.829396611
smc261.taurine	0.005735815	0.026576591	0.829447427
pcaec382.lysopcac140	0.000398271	0.001845559	0.829464803
smohc221.pcaec300	-0.017428197	0.080802717	0.829551543
c16.pcaec386	-0.00132046	0.006122689	0.829568373
pcaac386.c51	-0.013624699	0.063186659	0.829599828
pcaec401.putrescine	0.009650911	0.044759861	0.829608308
pcaac421.pcaac380	-0.017986855	0.083471303	0.829709298
pcaac402.pcaac240	0.005106536	0.023698045	0.829710905
smohc161.pcaac240	0.001678812	0.00779736	0.829849623
lysopcac180.c10	-0.006699345	0.031116137	0.829852601
pcaac383.pcaac322	-0.012969436	0.060239585	0.829855511

c16.prolinr	-0.005492822	0.02551329	0.82985938
phenylalanine.pcaec423	0.001400631	0.006505824	0.829862341
h1.pcaec385	0.012517139	0.058155879	0.829904749
pcaac424.pcaac240	0.004464915	0.020749563	0.829946075
c182.c16	-0.009670362	0.044944232	0.829959749
pcaac403.glutamicacid	-0.016144569	0.07504481	0.829984029
leucine.pcaac366	-0.003614839	0.016803316	0.829988594
lysopcac161.pcaac342	-0.001406414	0.006538621	0.830014369
pcaac385.pcaec403	-0.004677788	0.021748485	0.830020354
pcaac401.glycine	-0.002977904	0.013850262	0.830081531
pcaac402.c182	-0.009829692	0.04572518	0.830107824
arginine.lysopcac180	-0.007491182	0.034856784	0.830154612
pcaec445.lysopcac281	0.001682465	0.007835655	0.83030561
pcaec362.pcaac424	-0.001745845	0.008131049	0.830310062
pcaac380.pcaac426	-0.010344618	0.048188294	0.830343226
pcaec425.pcaac344	-0.007470271	0.034814707	0.830419867
c51.pcaac420	-0.012734361	0.059352082	0.830432334
serine.c12	0.010628816	0.049563246	0.830515376
pcaec403.c182	-0.009201859	0.042914977	0.830537823
methioninesulfoxide.smc180	0.000499031	0.002327591	0.830555337
pcaec364.pcaec385	-0.024069875	0.112407196	0.830763212
pcaec420.pcaec385	-0.008994907	0.042014668	0.830795432
pcaac406.lysopcac182	-0.001772734	0.008281147	0.830811752
pcaec361.smohc221	-0.013738002	0.064177457	0.830816405
pcaec383.alanine	0.003719061	0.017373728	0.830816706
pcaec402.c3	-0.004436	0.020725876	0.83084034
pcaac426.pcaec341	-0.009485092	0.044320964	0.830858105
pcaec383.pcaec384	-0.005557544	0.025971362	0.830875049
pcaac421.pcaec341	0.00517272	0.024175639	0.830893142
c181.methioninesulfoxide	0.00221661	0.010364241	0.830965685
pcaec420.pcaec343	-0.009406203	0.044000648	0.831040966
pcaac343.pcaac342	-0.001547875	0.007241832	0.831066998
c16.c141oh	-0.011751692	0.054983848	0.831075453
kynurenine.h1	-0.000352317	0.001648573	0.831090666
histidine.asparagine	-0.004961654	0.023220054	0.831114511
pcaac240.lysopcac181	0.010217058	0.047821041	0.831136122
c14.c182	0.007035249	0.032936731	0.831177425
smc260.c102	-0.006247978	0.029263235	0.83124701
pcaec422.methioninesulfoxide	0.002539649	0.011897238	0.831281322
lysopcac161.smc241	0.012197057	0.05715459	0.831328579
pcaec422.lysine	0.015780424	0.073953783	0.831346052
smohc221.pcaac344	0.011025049	0.051684272	0.8313981
ornithine.c51dc	0.018359239	0.086066785	0.831399269
c141.putrescine	0.005957259	0.027927554	0.831401377
c181.isoleucine	-0.003456518	0.016204285	0.831403121

smc260.pcaac406	-0.005510922	0.025840053	0.831432984
pcaec406.serine	0.016397957	0.076923909	0.831510369
acetylornithoine.pcaac426	0.003666119	0.017205341	0.831581081
c0.arginine	-0.006715253	0.031518181	0.831597136
pcaec342.pcaac380	-0.01954876	0.091797612	0.83167862
asymmetricdimethylarginine.c10	-0.003758194	0.017655864	0.83175404
c101.isoleucine	0.004158302	0.019537978	0.831774586
methioninesulfoxide.glycine	-0.003101041	0.014574109	0.831816857
pcaac380.pcaac320	0.02384856	0.112086157	0.831822714
lysopcac280.smohc222	0.001444903	0.006791141	0.831828106
arginine.isoleucine	-0.005026793	0.02362847	0.831843593
smohc161.pcaec421	0.000223636	0.001051342	0.831865342
pcaec401.smc202	0.007095833	0.03335933	0.831870052
lysopcac180.pcaec342	-0.015855531	0.074552575	0.831895943
pcaac366.serine	0.012253271	0.057633351	0.831949292
c141.smc261	0.008527908	0.040112504	0.831955184
pcaac401.pcaac364	0.010455167	0.049210397	0.832065141
c0.smc161	0.015202286	0.071560269	0.832079293
c101.lysine	-0.00916425	0.043155103	0.832144848
smc240.pcaac363	-0.001667737	0.007854034	0.832156281
pcaac320.glutamicacid	-0.007616864	0.035884567	0.832219545
pcaac420.asparagine	-0.002225114	0.01048679	0.832279929
pcaec360.pcaac422	-0.010640246	0.0501722	0.832364071
pcaac322.lysine	-0.011305676	0.0533193	0.832393134
c162.valine	-0.009995163	0.047143666	0.832410453
pcaec424.glutamicacid	0.013603765	0.064181531	0.832455108
c0.ornithine	0.001900819	0.008969599	0.832485963
pcaec386.smc160	-0.027758614	0.131018222	0.832524617
pcaac364.pcaec384	-0.004915474	0.023205736	0.832561081
c101.pcaac426	-0.008079845	0.038152102	0.832593561
pcaac386.pcaac383	-0.002092748	0.009882278	0.832602938
c16.pcaec384	-0.004620215	0.021822559	0.832642175
lysopcac140.pcaec362	-0.003875275	0.018310612	0.832701691
c51.lysopcac203	0.001759746	0.008315222	0.832710645
c12.c141oh	-0.024739565	0.116921411	0.832740266
pcaac365.prolinr	0.006473308	0.030596423	0.832756356
smc240.lysopcac180	0.008163156	0.038618736	0.832906337
isoleucine.histidine	0.015302958	0.07241	0.832937868
smohc221.pcaac363	0.001356817	0.006420974	0.832959306
c14.asymmetricdimethylarginine	0.002624372	0.012420329	0.83296988
pcaac384.pcaec425	0.000761293	0.003603	0.832971684
c162.tyrosine	-0.007895555	0.037368948	0.832977684
pcaac426.c51	-0.010600922	0.050184148	0.833013547
c18.alphaaminoadipicacid	0.000747718	0.00354192	0.833118789
glutamicacid.smohc241	0.013556654	0.06425665	0.833218974

pcaec340.smohc222	0.000937729	0.004444722	0.833219573
pcaec343.pcaac381	-0.00660139	0.031298496	0.833265309
c10.kynurenine	0.001276659	0.006053281	0.833275905
c102.smc261	0.005132994	0.024338768	0.833280356
pcaac341.c14	0.013159049	0.062411792	0.83332357
creatinine.putrescine	0.004192459	0.019895678	0.833417226
pcaec402.smc180	0.000656139	0.003114178	0.833439096
pcaac422.pcaac342	-0.003053764	0.014494638	0.833448207
pcaec341.pcaec421	-0.000319547	0.001517177	0.833497014
pcaec420.glutamicacid	-0.009536921	0.045286094	0.833518013
pcaac383.ornithine	-0.004548912	0.021620953	0.833673025
c182.pcaec420	0.002912069	0.013841064	0.833673208
pcaac380.pcaec422	-0.003335363	0.015853371	0.833677225
pcaec381.glycine	-0.002185852	0.010393597	0.833740114
lysopcac140.smohc141	-0.005269898	0.025083446	0.833905936
smc181.lysopcac281	-0.002864724	0.013646391	0.834037793
pcaac281.c8	0.008155376	0.038854863	0.834062765
pcaac406.pcaac364	-0.007822903	0.037273389	0.834073889
h1.lysine	-0.008508087	0.040549441	0.834119813
glycine.pcaec380	-0.00966551	0.046071746	0.83414123
pcaec364.phenylalanine	0.005169852	0.024651123	0.834197167
pcaec364.pcaac425	-0.014887547	0.070989543	0.834201931
pcaec402.c182	-0.006126801	0.029220014	0.834230305
isoleucine.serine	-0.010047085	0.047929259	0.834273164
pcaac322.alanine	-0.00710044	0.033872779	0.834275033
histidine.pcaec342	0.014030343	0.066952835	0.834325816
pcaec404.c142	0.006542819	0.031223036	0.834329394
smc180.pcaec382	0.001574182	0.007514494	0.834380002
pcaec446.pcaac360	-0.006162679	0.029420286	0.834392201
pcaec445.pcaac384	-0.014122411	0.067430177	0.834417795
smohc241.pcaec362	-0.006089149	0.029080992	0.834458062
pcaec424.pcaec420	0.003920333	0.018727633	0.834498356
pcaac361.pcaac342	-0.00130936	0.00625909	0.83460799
pcaec341.pcaac406	-0.006628901	0.031691063	0.834624231
c18.smc181	0.004750527	0.022712606	0.834635464
c14.methioninesulfoxide	0.00330231	0.015788591	0.834635602
c182.alphaaminoadipicacid	0.000734086	0.003512105	0.834746214
pcaec423.smohc141	-0.005492978	0.026282608	0.834761252
c12.arginine	0.003517183	0.016844335	0.834910517
smc240.pcaac281	-0.000928833	0.004448578	0.834919785
c142.smohc221	0.012424048	0.059515917	0.834952142
c141oh.threonine	0.006276915	0.03008008	0.835013085
smc241.lysopcac204	0.005252627	0.025172414	0.8350189
pcaac365.c141oh	0.013310972	0.063798324	0.83503807
smc261.lysopcac204	-0.005887563	0.028219928	0.835045864

c142.c4	-0.003264357	0.015663948	0.835226682
pcaec404.smc160	0.009621821	0.046177143	0.835251399
c101.threonine	0.004300277	0.020643345	0.835293943
pcaec425.lysopcac281	0.001683442	0.008082612	0.835320076
pcaac362.c8	0.006244299	0.029998727	0.835419309
pcaac364.pcaac426	-0.010507311	0.050540442	0.835616511
c2.pcaec386	-0.000977785	0.004703474	0.835626877
histidine.asymmetricdimethylarginine	-0.0026365	0.012685946	0.835671586
pcaac343.smc260	0.004544073	0.021866946	0.835689369
serotonin.c8	-0.009222723	0.044388688	0.835715608
pcaec365.pcaac383	0.000842863	0.00405698	0.835727861
c10.lysopcac180	0.010708846	0.051547577	0.835735149
pcaac320.isoleucine	-0.005229975	0.025182305	0.835783713
c182.pcaac386	0.00190637	0.009179468	0.835789116
asymmetricdimethylarginine.pcaec42	0.000694357	0.00334607	0.835916459
c3.asparagine	0.001947029	0.009383375	0.835929293
smc181.pcaac422	-0.009269769	0.044682228	0.835958802
pcaac421.pcaac424	-0.002203109	0.010622369	0.836003275
ornithine.alphaaminoadipicacid	0.001279165	0.00616754	0.836003295
pcaac323.c4	-0.001785273	0.008614907	0.836137414
c162.pcaec301	-0.012963744	0.062557807	0.836139315
pcaac364.pcaec386	0.007155249	0.034529692	0.836145603
pcaec341.pcaac344	0.011083736	0.053501479	0.836187118
smohc222.pcaac386	-0.001918955	0.00927183	0.836343575
asparticacid.threonine	0.007048643	0.034066672	0.836389469
smc240.pcaac321	-0.003184337	0.015391037	0.836398624
smc240.pcaec386	0.001994962	0.009646334	0.836464994
pcaec362.pcaec403	-0.006191964	0.029945336	0.836492139
smohc222.c182	-0.004101181	0.019834869	0.836499401
c14.pcaec421	-0.000669302	0.00323794	0.836546144
c181.pcaec363	-0.003146969	0.015225937	0.83656292
pcaac383.pcaec402	-0.005651373	0.027379032	0.836775179
glutamicacid.smc160	-0.011233714	0.054448674	0.836849216
c12.pcaac366	-0.004690398	0.022740795	0.836898101
smc202.pcaac383	-0.000775384	0.003759547	0.836906578
putrescine.pcaec424	-0.012244471	0.059393733	0.836973837
glutamicacid.c5	0.002868254	0.013915273	0.83700104
lysopcac160.c162	-0.013814724	0.067038352	0.83704063
pcaac404.pcaec343	0.011573801	0.056175123	0.837072775
ornithine.serotonin	0.014843887	0.07204971	0.837078905
pcaac384.pcaac426	0.008817638	0.042802319	0.837090151
pcaec446.pcaac383	0.001215473	0.00590145	0.837126496
lysopcac204.pcaec360	0.000999232	0.004852611	0.837161908
citruline.pcaac360	-0.006394734	0.03106804	0.83722928
pcaec362.lysopcac280	-0.001585	0.007703094	0.837282675

pcaec406.pcaac364	-0.007871966	0.038275573	0.837357478
putrescine.c162	-0.013559089	0.065949154	0.837409238
glutamicacid.pcaac323	0.017432505	0.084802503	0.837435135
pcaac360.pcaac406	0.010809226	0.052585068	0.837442136
acetylornithoine.methioninesulfoxide	-0.003614333	0.017584818	0.837457585
lysopcac182.c8	-0.009094983	0.044271095	0.837534558
h1.pcaac300	-0.009487388	0.046195855	0.837585445
h1.pcaec363	0.005912053	0.028788029	0.837591807
leucine.pcaac425	-0.015556922	0.075755523	0.837598085
pcaec365.c101	-0.002138802	0.010419363	0.837664422
c51dc.pcaec401	-0.013841893	0.067443083	0.837690708
pcaec340.pcaec401	-0.00781951	0.03810603	0.837717327
pcaec301.c142	0.005032845	0.02453523	0.837777152
pcaec301.pcaac420	-0.011628372	0.056702356	0.83781604
smohc221.pcaac281	-0.000769568	0.003753854	0.837870635
pcaac401.pcaac425	-0.022342163	0.108985058	0.837874772
smc240.smc260	-0.007819454	0.038183354	0.838042489
pcaec360.ornithine	-0.005907008	0.028850217	0.838073333
pcaac320.pcaec423	0.00107914	0.005270876	0.838081986
methionine.pcaac383	0.000888691	0.004341964	0.838129993
pcaac381.lysopcac281	-0.006107521	0.029844519	0.838153491
pcaec444.pcaac320	-0.004382867	0.021434023	0.838280518
pcaac343.pcaec385	0.007008166	0.034275283	0.838291988
smohc141.pcaac320	0.014480969	0.070833524	0.838315684
pcaac420.serotonin	0.020621945	0.100915012	0.838383553
pcaec420.isoleucine	0.004133483	0.020227928	0.838386893
c14.pcaec423	0.001979368	0.009689261	0.8384342
smohc222.pcaec383	-0.010357537	0.050709089	0.838458091
pcaac323.smc260	0.004402818	0.02155636	0.838463735
acetylornithoine.pcaac321	-0.005764871	0.028229697	0.838490116
pcaac300.pcaac363	0.002262169	0.011079155	0.838513943
asparticacid.c182	0.007589904	0.037217217	0.838706688
pcaec340.pcaec380	-0.00792422	0.038863302	0.838734364
lysopcac280.pcaec340	0.00757401	0.037158466	0.838788809
pcaec404.pcaac320	-0.011099298	0.054456081	0.838795765
prolinr.c51	0.004939969	0.024238699	0.838808281
pcaec425.ornithine	-0.006006651	0.029486439	0.838883234
c18.pcaac420	0.020942552	0.102808576	0.83888683
acetylornithoine.methionine	0.006457619	0.031706059	0.838912472
pcaac422.c182	0.007743127	0.038018651	0.838916304
lysopcac170.lysopcac204	0.004637054	0.022769368	0.838926744
pcaec384.smc240	0.003298034	0.016195275	0.838935663
lysopcac281.smc240	-0.007624327	0.037444633	0.838955704
methionine.pcaec386	-0.000683205	0.00335769	0.839065791
smohc161.c142	0.010551596	0.051876096	0.839124232

pcaec401.smc161	-0.013536457	0.066562903	0.83915276
pcaac320.pcaec365	0.004444418	0.021876955	0.839315126
smc241.pcaec384	0.018615543	0.091764875	0.839544406
c18.asymmetricdimethylarginine	0.004199469	0.020710345	0.839614414
pcaec340.pcaec421	0.000231784	0.001143764	0.839709371
smc181.smohc241	0.014968737	0.073878506	0.839738208
pcaec444.pcaec403	-0.008257046	0.040753658	0.839741475
pcaec364.lysopcac280	0.001728208	0.008532826	0.839797879
asymmetricdimethylarginine.glutami	0.001786568	0.008821607	0.839809242
smohc161.pcaec365	0.004649984	0.022979017	0.839937174
lysopcac280.c162	0.012556647	0.06208511	0.840022148
isoleucine.lysine	0.020696112	0.102334618	0.840029429
smc261.pcaac401	-0.001123351	0.005557247	0.84010589
arginine.pcaec361	0.001416861	0.007009617	0.840114146
pcaec380.threonine	-0.005289084	0.026166766	0.840115075
pcaac321.pcaec425	-0.001321523	0.006538784	0.840134203
pcaac300.pcaec421	0.000398505	0.001972079	0.840159147
c12.pcaac360	-0.005498509	0.02721906	0.840208959
pcaec406.smc180	0.004367689	0.021626321	0.840246246
pcaec300.pcaac424	0.002985143	0.014782	0.840259725
smc161.lysopcac281	0.00185505	0.009190041	0.840330018
lysopcac280.pcaec301	0.011207243	0.055522166	0.840332088
pcaec446.threonine	0.002433449	0.012063467	0.840434413
pcaac281.tyrosine	0.007653195	0.037943999	0.840452696
smc240.pcaac426	-0.004996271	0.024777951	0.840495841
pcaec424.c12	0.008062222	0.039988553	0.840518145
lysopcac160.c3	-0.00560509	0.027814607	0.840593986
smohc241.glutamicacid	-0.005060929	0.025128406	0.84068241
smohc161.pcaac386	-0.001966783	0.009765851	0.840689274
pcaac341.c12	0.007960951	0.039530302	0.840693452
pcaac422.tyrosine	-0.008677411	0.043094944	0.840719141
alanine.pcaec362	-0.002480505	0.012324439	0.840788212
smc180.pcaec340	0.013908432	0.069122216	0.840828838
lysopcac204.pcaac383	0.001002836	0.004983924	0.840829649
asparticacid.smc181	0.001106047	0.005497975	0.84086119
pcaec402.ornithine	-0.005492356	0.027304509	0.840878072
c5.pcaac420	0.016081808	0.079968782	0.840917807
pcaec341.pcaec386	-0.000770645	0.003833551	0.840976126
creatinine.pcaac300	-0.006847507	0.034078946	0.841050742
c10.pcaac363	0.001717525	0.008548364	0.841060258
asymmetricdimethylarginine.pcaac36	-0.005751302	0.028630351	0.841089312
h1.pcaec420	-0.012900813	0.064223582	0.84109544
glutamicacid.pcaec443	0.016359163	0.081440435	0.841095996
pcaec343.pcaec444	0.017756441	0.088398919	0.841100339
pcaec365.pcaac360	0.007590835	0.037795385	0.841121343

pcaec363.c12	0.011024299	0.054896511	0.84113741
prolinr.pcaec386	-0.001554472	0.007742757	0.841180376
lysopcac180.c3	-0.00488983	0.024363233	0.841226658
pcaac424.pcaac406	0.011479361	0.057202726	0.841247528
histidine.pcaac421	0.005347415	0.026651821	0.841277822
glycine.pcaac385	-0.005091196	0.025384995	0.841340628
pcaac406.pcaec401	-0.008818132	0.043973769	0.841362193
smohc222.c0	-0.000206903	0.001032073	0.841407875
c142.smc261	-0.004422449	0.022063385	0.841431467
pcaec420.pcaec362	0.001775856	0.008864736	0.84152103
pcaac404.smc261	-0.006809675	0.033995465	0.841534184
c141.pcaac321	0.00514002	0.025665507	0.841566754
lysopcac203.pcaac343	-0.007163825	0.035783014	0.841619609
pcaac406.c12	-0.010033198	0.050183192	0.841830657
lysopcac281.smc181	-0.00164228	0.008221398	0.841967013
lysopcac182.smc241	-0.008633338	0.04322527	0.841988719
pcaec343.pcaec301	-0.008126496	0.040697039	0.842024793
pcaac421.smc240	0.007198559	0.036052382	0.842035181
c14.smohc221	0.015446972	0.077384959	0.842079954
kynurenine.pcaac383	0.000782598	0.003920793	0.842087857
pcaac424.c142	0.006923979	0.034692001	0.842101633
pcaac401.smohc141	-0.005158201	0.025848768	0.842126015
lysopcac140.smc261	0.005007358	0.025094823	0.842138177
c51.pcaac405	-0.006467415	0.032425181	0.842201356
histidine.pcaac403	0.001070486	0.005368706	0.842250444
pcaac381.smc180	0.000603295	0.003026266	0.842282278
pcaec384.pcaec383	-0.011506884	0.057769344	0.842412036
pcaec384.c4	0.006658196	0.033435783	0.84245329
pcaec445.c4	-0.002829925	0.014214743	0.842492378
pcaac341.valine	0.007854595	0.039465811	0.842539993
pcaac344.pcaec340	-0.008567361	0.043064292	0.842601888
pcaac383.smohc141	-0.003661656	0.018415655	0.842687474
pcaac344.pcaac426	0.014546854	0.073198821	0.842768011
c0.pcaac422	-0.005549865	0.027956263	0.842932863
pcaec340.c16	0.010631804	0.053577813	0.842997585
pcaac381.lysopcac182	-0.002962661	0.014953252	0.843238429
lysopcac181.lysopcac160	0.003151603	0.015912697	0.84329494
pcaac386.pcaec403	-0.003792597	0.019154448	0.843337898
pcaec405.glycine	0.005538802	0.027988273	0.843418792
pcaac240.pcaec385	-0.012613764	0.063739059	0.843419042
pcaec420.c14	0.005604422	0.02832422	0.843442582
pcaec403.smc180	0.00060275	0.003047974	0.843530518
pcaac421.pcaac384	0.005591113	0.028274114	0.843536361
pcaec425.pcaec341	0.003603587	0.018227391	0.843571462
histidine.lysopcac281	-0.001276003	0.006455188	0.843595588

isoleucine.pcaec363	-0.004953818	0.025079502	0.843709818
c2.pcaac420	-0.013561634	0.068663523	0.843722318
pcaac426.pcaac363	-0.002551528	0.012920504	0.843745419
pcaec386.asymmetricdimethylarginir	0.006271375	0.031773387	0.843824112
pcaac322.smc202	-0.004847231	0.024562676	0.843852936
pcaac360.pcaac425	0.01688165	0.085552739	0.843866084
pcaec406.pcaac344	-0.011901979	0.060326957	0.843892058
pcaec381.pcaec386	-0.001747342	0.008864107	0.844021434
lysine.pcaac402	0.013782026	0.069950624	0.844099921
pcaec401.smohc141	-0.002556753	0.012977708	0.844110802
smc261.pcaec343	0.00961974	0.048852661	0.844187189
pcaac320.c3	0.006633778	0.033696209	0.844220908
c4.pcaac420	0.007879091	0.040055395	0.844350011
lysopcac203.alphaaminoadipicacid	-0.00132671	0.006746301	0.844387123
smc240.pcaec361	-0.00206716	0.010513792	0.844420862
smc202.pcaac344	-0.007234076	0.036809188	0.844487312
serine.lysine	0.014369486	0.073212374	0.844688682
lysopcac182.pcaec301	0.006442601	0.032827396	0.844699961
pcaec341.pcaac426	0.009786965	0.049869103	0.844702903
smohc221.tyrosine	0.00941132	0.04796615	0.844738469
pcaac425.pcaec402	-0.008619818	0.043941736	0.844771925
pcaac364.tyrosine	0.011024038	0.056290089	0.845022982
pcaec444.pcaec446	-0.009067338	0.046321243	0.845096651
glycine.c51	-0.009555202	0.048815569	0.845103021
asymmetricdimethylarginine.c8	0.005989453	0.03061843	0.845200612
smc202.alanine	-0.001653336	0.008456018	0.845274166
pcaac384.pcaec322	-0.011696828	0.059834272	0.845301276
pcaac403.c3	0.009470895	0.048479741	0.845402301
c12.pcaec420	0.002645844	0.013547674	0.845448384
pcaec383.lysopcac280	0.001739135	0.008905843	0.845462912
c16.c141	0.002219999	0.011371275	0.845503187
pcaac360.pcaac363	0.003491658	0.017885003	0.845503506
c8.lysopcac281	-0.001807453	0.009259417	0.845524321
c2.pcaac344	0.011586841	0.059386145	0.845595764
methioninesulfoxide.valine	0.004000021	0.020508559	0.84564937
lysopcac140.pcaac421	-0.005291809	0.027140562	0.845699127
acetylornithoine.c101	-0.002134115	0.010947447	0.845727295
c101.pcaac405	0.009673261	0.049621422	0.845727761
c5.asparagine	0.004268228	0.021901367	0.845772416
pcaec385.pcaec421	-0.00016135	0.000828141	0.845811436
glutamicacid.pcaac366	-0.003724837	0.019121254	0.845837412
pcaec402.c4	0.003483589	0.017884745	0.845853812
pcaac425.pcaac363	-0.002199532	0.011295677	0.845897973
pcaec443.pcaec341	-0.003318466	0.017047232	0.845945105
c162.c18	-0.008575435	0.044056182	0.845957092

smc261.pcaac364	-0.009092111	0.046714628	0.845970224
pcaac402.pcaac421	-0.010503295	0.053972547	0.845990948
methioninesulfoxide.arginine	0.007625168	0.039187652	0.846009319
pcaac360.c3	0.003938151	0.020241743	0.846028824
pcaec342.pcaec424	0.004479968	0.023031476	0.84606077
pcaec362.pcaac426	0.010747658	0.055278867	0.846130211
pcaac426.asparagine	0.006192918	0.031853927	0.846138036
pcaec401.methioninesulfoxide	0.002058808	0.010591427	0.846162829
pcaac421.threonine	-0.00484569	0.024949123	0.846289099
pcaec386.c51	-0.005106803	0.026299619	0.846324269
pcaac381.pcaec301	-0.013823687	0.071232758	0.84641352
pcaac362.pcaac403	-0.000552212	0.002845566	0.846415924
pcaac405.pcaec445	-0.004457325	0.022972552	0.846441145
pcaec343.pcaec362	-0.004181243	0.021571961	0.846597922
pcaac426.pcaec445	0.009794834	0.050533771	0.846598059
pcaac320.pcaec362	-0.003689537	0.019039973	0.84663636
pcaec384.c182	0.003778503	0.019501068	0.846651771
pcaac385.pcaac322	0.007004396	0.036192829	0.846830607
lysopcac280.acetylornithoine	0.002610099	0.013488255	0.846846962
pcaec402.pcaac364	0.008989864	0.04647438	0.846903244
pcaec444.taurine	0.006435823	0.033278973	0.846939923
pcaec386.pcaac422	0.006833189	0.035345767	0.846991473
leucine.pcaac385	-0.00658133	0.034076598	0.847140497
c8.pcaac363	0.001630832	0.008444745	0.847152629
pcaec382.pcaac420	0.019805612	0.102594084	0.847207051
smc160.c16	-0.008511941	0.044106294	0.847254968
asparticacid.pcaec420	-0.007068945	0.036694633	0.84752426
h1.asparagine	0.002906119	0.015085928	0.847527953
pcaec402.pcaac361	-0.007103108	0.036873617	0.847530963
serotonin.pcaec365	0.004988567	0.02589908	0.847545262
pcaac402.smc180	0.000480072	0.002493042	0.847584995
pcaec341.c142	0.006447593	0.033493208	0.84763211
h1.pcaac362	0.002383122	0.012385773	0.847707569
pcaec383.asymmetricdimethylarginir	0.004012554	0.020862744	0.84776768
glycine.ornithine	-0.007748303	0.040290865	0.847784804
serotonin.pcaec383	-0.012499657	0.065028683	0.84785639
lysopcac170.pcaac360	-0.005158124	0.026844149	0.847908637
lysopcac281.pcaec403	-0.001884972	0.009811901	0.847939895
c141.pcaec363	-0.003582163	0.018649558	0.847965821
pcaac402.c12	0.015364639	0.08001538	0.848010027
pcaac381.smc160	-0.008370586	0.043607685	0.848063952
pcaec386.pcaac426	-0.007633601	0.039795412	0.848166335
pcaac366.alphaaminoadipicacid	-0.002070343	0.010793987	0.848178808
pcaac281.c16	0.007977292	0.041591674	0.848182698
alanine.pcaec301	-0.009106505	0.047488548	0.848212458

c2.smc161	-0.013234262	0.06902767	0.848242266
pcaec386.pcaec322	-0.018799045	0.098060998	0.848255044
pcaac362.lysopcac204	0.003288073	0.017153981	0.848276772
pcaec322.pcaac300	-0.011011306	0.057494796	0.848403092
c51dc.smohc141	0.003950876	0.020631281	0.848417942
smc180.pcaec301	0.010278768	0.053681317	0.848434953
pcaac405.pcaac322	-0.009556042	0.049925997	0.848492365
citruline.lysopcac161	-0.013189916	0.068935678	0.848545194
smohc241.smc161	0.014267908	0.074571284	0.848548391
smohc161.smc202	0.015253417	0.079754565	0.848609386
pcaec362.pcaec423	-0.001975446	0.010329694	0.848621016
lysine.c101	0.002144141	0.011212115	0.848625125
pcaec381.pcaec443	0.047505038	0.248437506	0.848640055
pcaec341.c16	-0.006202498	0.032439761	0.848651598
pcaac342.pcaec385	-0.009285134	0.04856967	0.848674295
pcaac322.c12	0.006704615	0.035093024	0.848767202
pcaac323.pcaac366	0.00639799	0.033488118	0.848767278
c102.asymmetricdimethylarginine	0.001683232	0.008810452	0.848769691
phenylalanine.pcaec341	-0.00702554	0.036782731	0.848807529
pcaac342.pcaac323	-0.010172897	0.053283226	0.848869983
pcaec340.pcaec420	0.00378829	0.019842975	0.848876044
smc160.smc241	-0.017880562	0.093670661	0.848896289
lysopcac280.lysopcac182	0.002045776	0.010720602	0.848943933
pcaac426.pcaac424	-0.002316368	0.012144558	0.84901712
smc260.glutamicacid	-0.006384173	0.033473824	0.849026236
lysopcac182.ornithine	0.003686692	0.019352611	0.849198534
pcaec422.smohc141	-0.003315867	0.017408063	0.849215927
pcaec401.pcaec382	-0.002571997	0.013506879	0.8492609
c3.pcaac403	-0.001228672	0.006453222	0.849280093
pcaac361.isoleucine	-0.002951784	0.015512233	0.849365517
lysopcac170.transhydroxyproline	-0.005380677	0.028279168	0.849379225
pcaac320.pcaec380	-0.009866506	0.051862461	0.849399784
lysopcac140.pcaec340	0.011170969	0.05874985	0.849477245
pcaac426.pcaec340	0.010824539	0.056938429	0.84950469
c141oh.tyrosine	0.009559035	0.050282638	0.849507401
asparticacid.taurine	-0.010364325	0.054531817	0.849543331
kynurenine.c142	0.010261093	0.053990186	0.849547518
pcaec385.c16	-0.005842082	0.030741921	0.849561942
lysopcac204.c4	0.001372992	0.007226358	0.849592075
pcaac401.lysopcac203	-0.004674368	0.024602452	0.84959347
glutamine.lysopcac280	0.000905809	0.004768836	0.849634679
c141oh.c141	0.006009334	0.031647914	0.849683517
pcaec361.pcaac424	0.00581401	0.03065031	0.849834015
pcaec420.pcaec403	-0.002460073	0.012969121	0.849835177
smohc222.asparticacid	0.004515937	0.023813984	0.849876741

pcaec425.pcaec446	0.009781376	0.051593611	0.849914917
serine.smc160	-0.009223285	0.048680554	0.850008413
lysopcac204.arginine	0.008142762	0.042977577	0.850008519
smohc222.c8	0.005786623	0.030549158	0.850043962
pcaec381.smc180	0.00053959	0.002848818	0.850053168
alphaaminoadipicacid.histidine	-0.009449677	0.049892851	0.850060095
pcaec381.phenylalanine	-0.013345012	0.07046231	0.850065745
methionine.smc180	-0.001150524	0.006074965	0.850069205
pcaec404.glutamicacid	0.013310885	0.070320105	0.850145759
asparagine.prolinr	0.00734402	0.03880128	0.850159273
c0.pcaec420	-0.009658489	0.051052766	0.850226709
pcaec422.c142	0.006014203	0.031806991	0.850306569
kynurenine.pcaac363	0.001206673	0.006382651	0.850329375
alphaaminoadipicacid.pcaec386	0.001133221	0.005996297	0.850382881
pcaac385.pcaec386	-0.002142277	0.011336656	0.850396766
glutamine.valine	-0.008981483	0.047540043	0.850431512
pcaec384.glutamicacid	0.006414382	0.033960893	0.85046988
pcaac366.pcaac360	-0.010521469	0.055713928	0.850491347
asymmetricdimethylarginine.c182	-0.006832468	0.036182487	0.850502708
pcaec420.methioninesulfoxide	0.009166579	0.048565423	0.850570415
pcaac362.pcaec365	-0.003572994	0.018930309	0.850572278
pcaac386.c142	0.00490946	0.026012098	0.850577815
creatinine.smc202	0.006036035	0.031981576	0.850579995
alanine.pcaac421	0.006345699	0.033622647	0.850581472
lysopcac161.pcaec341	0.005011142	0.026558453	0.850620095
lysopcac281.pcaec365	0.003813725	0.020220908	0.850683044
pcaec340.h1	0.000302978	0.001607841	0.850812183
smc161.c3	-0.0028417	0.015082466	0.850833426
serine.asymmetricdimethylarginine	0.003987108	0.021163734	0.85084704
pcaec301.valine	-0.010725685	0.056965878	0.850933714
h1.smc240	-0.003358602	0.017838889	0.850940325
pcaec421.asymmetricdimethylarginir	-0.004244324	0.022545952	0.850957551
pcaac405.pcaac364	0.009859015	0.052371842	0.850958994
c14.methionine	-0.00266885	0.014178377	0.850971921
c16.pcaac240	-0.004249619	0.022579004	0.85098969
arginine.pcaec383	-0.013681858	0.072720425	0.851042742
prolinr.c10	0.001888588	0.010039403	0.851062829
pcaac360.c12	0.007255105	0.038599988	0.851189132
pcaac402.kynurenine	0.002760106	0.014685832	0.851199069
smc241.pcaac406	0.009861525	0.052489139	0.851250713
lysopcac281.arginine	0.005611742	0.029872576	0.851267517
arginine.lysopcac182	-0.004864295	0.025900289	0.851304639
pcaec445.pcaec384	0.009673987	0.051559535	0.85144626
pcaec420.valine	-0.005732633	0.030559086	0.851474223
pcaec444.pcaac322	0.008123299	0.043328973	0.851561979

pcaec364.pcaac386	0.004883274	0.026057939	0.851623784
pcaac300.pcaac320	-0.015268737	0.081510746	0.851685494
pcaac401.pcaec384	-0.00503267	0.026868479	0.851696575
pcaac364.pcaac405	-0.008172789	0.04365291	0.851763496
smc202.smc241	0.004173532	0.022323055	0.851968092
lysopcac181.asymmetricdimethylarg	0.003438548	0.01839286	0.851976265
lysopcac281.methioninesulfoxide	0.002328791	0.01246114	0.852027817
smc240.pcaac403	-0.003777257	0.020214769	0.852049713
pcaac420.smc161	0.012225179	0.065431121	0.852062149
pcaec343.valine	0.007672132	0.041069248	0.852086242
pcaac425.c12	-0.008162012	0.04372717	0.852205161
c51.serotonin	0.013253405	0.071066007	0.852333023
lysopcac204.pcaac385	0.004706287	0.0252472	0.852400351
pcaec402.smohc141	-0.003990081	0.021413334	0.852456696
pcaac405.phenylalanine	0.004025463	0.021618225	0.852557919
lysopcac181.c10	0.005620576	0.030184894	0.852559506
lysopcac140.pcaec383	-0.013766821	0.073950728	0.852593027
ornithine.pcaec423	-0.000630241	0.003385863	0.852611022
pcaac363.pcaec402	-0.0045238	0.024306353	0.852629142
c14.lysopcac281	0.002038519	0.010961656	0.852744801
lysopcac203.lysopcac281	0.001793822	0.009646571	0.852755626
lysopcac170.pcaec385	0.004801828	0.025827063	0.852780673
glutamine.lysopcac281	-0.002118647	0.011396252	0.852792388
smc161.isoleucine	-0.003180558	0.017110889	0.85281443
pcaec406.pcaec402	0.011231338	0.060432103	0.852836876
smc160.pcaec383	-0.010030376	0.053976545	0.852854176
prolinr.smc202	0.006888119	0.037068808	0.852860903
c51.pcaec404	0.004144253	0.022315802	0.852947367
lysopcac204.lysopcac182	-0.005317532	0.028634332	0.852950962
pcaac321.c142	-0.004473398	0.0240943	0.852984352
c0.pcaec365	-0.006084108	0.032783239	0.853043973
pcaec341.pcaac360	-0.005384554	0.029014037	0.853045141
pcaac385.c8	-0.006297742	0.033942151	0.853077278
c141.prolinr	-0.004846077	0.026134518	0.853167427
smc180.pcaac361	-0.006954546	0.037522085	0.853232254
pcaec340.smohc141	0.006799208	0.03669083	0.853259313
pcaec443.taurine	0.008801109	0.047525234	0.853355365
c51dc.pcaac300	0.00683762	0.036927662	0.853375388
lysopcac161.c14	-0.00857722	0.046345335	0.853446335
pcaac384.c3	0.003781538	0.020461476	0.853649373
pcaac321.smc241	0.008439992	0.045668363	0.853650989
pcaec385.taurine	-0.005030916	0.027224179	0.853662408
putrescine.pcaac385	-0.004811284	0.026035971	0.853664103
acetylornithoine.c102	-0.003512503	0.01900947	0.853677615
pcaec360.smohc241	-0.012586526	0.068153114	0.853753041

pcaac341.smohc241	-0.008018302	0.043444886	0.853845033
pcaec403.pcaac361	-0.00713303	0.038701369	0.854043211
pcaec361.lysopcac182	-0.00276355	0.014995317	0.854055151
pcaac425.c51	0.008267032	0.044885097	0.854142936
pcaac422.c12	0.008997753	0.048946377	0.854419579
pcaac420.putrescine	-0.005355979	0.0291627	0.854552869
pcaac281.pcaac424	-0.002235507	0.012188485	0.854746377
lysopcac161.pcaac322	0.012145427	0.06631449	0.854951943
smohc221.pcaec385	-0.007198013	0.039303333	0.854958925
pcaac402.pcaec423	0.004518769	0.024680221	0.854995933
pcaac422.c4	0.001207615	0.006600065	0.855091916
ornithine.glutamine	0.003966973	0.021688765	0.855143335
pcaac380.tyrosine	0.008576392	0.046893948	0.855155405
pcaec380.pcaac360	-0.008779428	0.048005782	0.855160407
pcaec386.pcaac343	0.004146262	0.0226722	0.85516356
lysopcac203.pcaac385	0.004676742	0.02563414	0.855505626
pcaac380.pcaac364	0.007402215	0.040574687	0.855511579
pcaec404.serotonin	-0.017568638	0.096307736	0.855521321
pcaec361.c142	0.005437432	0.029837881	0.855669621
pcaac422.c3	0.009912648	0.054398401	0.855676959
pcaec301.c14	0.006010607	0.032997575	0.85573192
pcaec425.kynurenine	-0.00190598	0.010466478	0.855770824
creatinine.valine	0.009586607	0.052656047	0.855804053
pcaec380.lysopcac180	-0.011192909	0.061528274	0.855918409
alanine.threonine	-0.005656847	0.031096236	0.855918933
pcaac341.pcaec423	0.000759164	0.004173361	0.855924582
citruline.pcaac420	-0.008903273	0.048950872	0.855944323
smohc161.pcaec364	0.005104214	0.028066369	0.855959622
smohc161.pcaac362	0.00379859	0.020887393	0.855961065
pcaac422.pcaec386	0.002728913	0.015008376	0.85598804
pcaac366.pcaec340	-0.007423781	0.040844338	0.85604138
pcaec362.taurine	-0.011291755	0.062142791	0.856081555
smohc222.asparagine	0.002627406	0.014463315	0.856118028
c0.lysopcac160	0.000780177	0.004295887	0.856157105
kynurenine.methioninesulfoxide	0.005620511	0.03095187	0.856173886
pcaec300.alphaaminoadipicacid	-0.000750464	0.004137448	0.856334737
lysopcac170.c51	-0.012878383	0.071001086	0.856335117
c4.pcaac320	0.017182129	0.094750777	0.856368533
pcaec405.pcaac425	-0.019193812	0.105901686	0.856445654
c182.ornithine	0.003309738	0.018264233	0.856467287
pcaec406.smohc241	0.009634496	0.053200871	0.85655941
pcaac366.c162	-0.009575859	0.052893549	0.856603577
acetylornithoine.lysopcac161	-0.004692247	0.025921763	0.856622746
smc180.serotonin	0.011153487	0.061616123	0.856622752
c5.c181	0.004461256	0.024649398	0.856644113

pcaec342.pcaac281	0.000237571	0.001312808	0.856663007
pcaac281.c51	-0.005583947	0.030860699	0.856681763
pcaec423.valine	0.007350819	0.040626096	0.856683366
c8.acetylornithoine	-0.004144377	0.022907067	0.856696705
lysopcac203.serotonin	-0.014606301	0.080757902	0.856740605
pcaac342.c2	0.011097015	0.061366538	0.856766922
pcaac366.taurine	0.004316079	0.023876077	0.856815232
lysine.smc161	0.008661293	0.047916822	0.856825579
pcaec365.pcaac422	-0.010517031	0.058192574	0.85684811
pcaac361.c14	0.006989653	0.038676353	0.856853169
pcaac360.putrescine	0.006443566	0.035664591	0.85689263
pcaac421.c142	0.007001955	0.038787966	0.857012092
c2.smc240	0.009276505	0.051448506	0.857178316
c141oh.pcaac343	-0.004319235	0.023964641	0.857235458
glutamicacid.pcaec386	0.001761327	0.009778696	0.857325483
pcaec445.pcaec364	-0.002750033	0.015269713	0.857342374
pcaec383.histidine	-0.013201622	0.073314592	0.857365156
pcaac401.pcaac422	-0.032727634	0.181804902	0.857406679
lysopcac281.pcaec341	0.010818499	0.060108295	0.857431498
smohc141.pcaac361	0.008607026	0.047846354	0.857505618
c8.c141oh	-0.019870131	0.110507012	0.857568373
pcaec381.alanine	0.002568484	0.014285008	0.857573076
c2.pcaec423	0.003521938	0.019614753	0.857766796
pcaac361.smohc241	0.006705062	0.037343914	0.85777195
lysopcac280.lysopcac181	-0.00381999	0.02127891	0.85779464
pcaac401.pcaec421	-0.000728697	0.004060965	0.857857839
pcaac343.threonine	0.005152478	0.028721003	0.85789061
smohc141.pcaec405	-0.003773087	0.02103354	0.857900973
pcaec343.c8	0.005208331	0.029048229	0.857967508
pcaec384.pcaac343	-0.004254958	0.023743446	0.858041039
lysopcac280.pcaec363	0.003289756	0.018363843	0.858090013
pcaac403.smohc222	-0.003070495	0.017144064	0.85812412
pcaec322.phenylalanine	-0.003192907	0.017831548	0.858155577
c10.lysopcac161	0.011567866	0.06460772	0.858164742
c16.c4	0.004812621	0.026886263	0.858202735
c16.pcaac322	0.009603671	0.053681955	0.858280972
pcaec361.pcaec403	-0.008860262	0.049539193	0.858316898
pcaec446.pcaac384	0.010000358	0.055937767	0.858377309
pcaec340.phenylalanine	0.003060634	0.017131127	0.85846923
alphaaminoadipicacid.c3	0.006548014	0.036661188	0.858508665
pcaec362.threonine	-0.007347882	0.041154813	0.858560715
glutamicacid.pcaac362	-0.002662665	0.014920029	0.858623453
pcaec360.methionine	0.00634513	0.035578398	0.858717679
pcaec343.pcaac364	0.00699118	0.03920816	0.858743474
c182.lysopcac180	0.005553024	0.031173258	0.858880699

pcaec300.pcaec404	-0.00389074	0.021843257	0.858891166
glutamine.smohc241	0.011175477	0.062763248	0.858940708
smc160.pcaec380	0.004624816	0.0259807	0.858978313
pcaac406.pcaac360	0.005508222	0.03097013	0.859098815
pcaec343.pcaec322	0.013624012	0.076645064	0.859178264
smc261.c8	0.004722227	0.02656761	0.859186742
pcaec406.pcaec423	-0.000781566	0.004397432	0.859195616
serotonin.pcaac300	0.005078761	0.028578488	0.859211087
pcaac424.glycine	-0.002371945	0.013353541	0.859278535
serotonin.smc260	0.003881542	0.021854142	0.859290622
pcaac342.pcaec421	-0.001242498	0.007003248	0.85944259
histidine.creatinine	-0.001659632	0.009355412	0.859457739
asparagine.pcaac383	-0.00111954	0.006314776	0.85954326
pcaec424.c142	0.00661226	0.037306088	0.859578935
kynurenine.lysopcac180	-0.005763574	0.032524593	0.859607797
pcaac360.creatinine	-0.002619366	0.014784332	0.859635168
pcaec445.pcaac281	0.001238336	0.006989485	0.859635498
pcaac406.serotonin	0.015044726	0.084917242	0.859637092
pcaac320.pcaec385	-0.007509815	0.042388163	0.859638292
pcaac300.threonine	0.004177474	0.023599975	0.859760548
pcaac341.pcaac383	0.000842923	0.004762125	0.859765306
pcaac341.smc241	-0.003634028	0.020536243	0.859803645
pcaec424.pcaac420	0.014086448	0.079670833	0.859920247
pcaec301.pcaac363	0.001353206	0.007656726	0.859978103
pcaac406.pcaec403	0.005028994	0.02846705	0.86003621
pcaec341.smc260	-0.004071774	0.023053071	0.860062928
leucine.pcaec365	-0.003916158	0.022173625	0.86007293
pcaec445.smc240	0.007746762	0.043878261	0.860121616
pcaac403.c18	-0.00755453	0.04281444	0.860202417
pcaec422.pcaec340	0.008496643	0.048168592	0.860245022
pcaac321.isoleucine	-0.005553579	0.031484074	0.860245465
pcaac380.pcaec362	0.002174766	0.012330759	0.860264359
c8.pcaec385	-0.004461935	0.025306321	0.860305253
pcaac341.pcaac344	0.010434951	0.059183382	0.860306407
pcaec423.pcaac321	0.002998347	0.017007685	0.860323587
lysine.arginine	0.004084072	0.023168676	0.860337743
pcaac321.ornithine	-0.003081533	0.017493954	0.860437424
pcaac240.lysopcac161	0.011307176	0.064197627	0.860451311
c16.phenylalanine	-0.006946753	0.039459142	0.860515198
pcaec403.asymmetricdimethylarginir	0.002854613	0.016225837	0.860608647
pcaac321.smc261	-0.007370661	0.041909113	0.860653785
c141oh.pcaec423	-0.001493118	0.008491439	0.860680795
pcaac401.alanine	-0.002159653	0.012289278	0.860761806
methionine.pcaec365	0.002796516	0.015914085	0.86076881
pcaac383.pcaec384	-0.005119318	0.029148809	0.860846309

citruline.smohc141	0.001327427	0.00755965	0.86087237
pcaac365.smc180	-0.004063706	0.023150174	0.860917072
pcaac381.pcaac424	-0.003409087	0.019427385	0.860962831
pcaac364.smohc241	0.011355713	0.064780576	0.86110663
pcaac426.pcaec382	0.00119859	0.006843057	0.861217114
asparagine.pcaec365	0.001852015	0.010576079	0.861248912
pcaec423.spermidine	0.009047431	0.051671419	0.861263133
histidine.lysopcac180	0.006252173	0.035710641	0.861276284
c162.ornithine	-0.003638889	0.020797416	0.861362847
creatinine.c5	-0.00241399	0.013820873	0.861602564
pcaac322.pcaec365	-0.004011413	0.022969398	0.861619076
pcaac404.pcaec361	-0.001607227	0.00920383	0.861631427
pcaec300.pcaec384	0.009841009	0.056374064	0.861678182
pcaac384.pcaac320	0.010942346	0.062711274	0.861739789
pcaec423.c142	0.006335641	0.03631613	0.861763051
pcaec444.smohc241	-0.011864579	0.068016292	0.861779304
smohc141.pcaac403	-0.001206492	0.006917785	0.861805153
glycine.pcaec384	0.002651112	0.015207352	0.861862715
spermidine.asparticacid	-0.016625707	0.095374696	0.861871398
pcaac402.c3	0.010038052	0.057593919	0.861894733
pcaac361.pcaac424	0.001022217	0.005872536	0.862069523
pcaac404.pcaac320	-0.006500156	0.037375754	0.862189997
pcaec424.pcaac361	0.006169811	0.035487062	0.862231448
serotonin.pcaec386	-0.000632605	0.003639735	0.862275096
c4.pcaac343	0.003038686	0.017486712	0.862301834
pcaec403.smc202	-0.01153136	0.066360432	0.862303815
pcaec423.taurine	-0.006503336	0.037442867	0.862367889
isoleucine.acetylornithoine	0.005738252	0.0330463	0.862402494
c142.pcaac380	0.005307911	0.030573768	0.862428256
pcaac426.smc181	-0.002285973	0.013167666	0.862432158
pcaec422.pcaac342	-0.00243074	0.014007843	0.862493296
asymmetricdimethylarginine.pcaec42	-0.005764552	0.033230416	0.862536355
prolinr.lysine	0.009765254	0.056293825	0.862538546
smohc161.glutamicacid	0.00523788	0.030199074	0.86255761
c182.pcaac364	0.00626243	0.036116356	0.862596135
lysine.asparagine	-0.001942758	0.011214566	0.862722195
alphaaminoadipicacid.lysopcac281	0.007313124	0.042217818	0.862731313
glutamine.pcaac420	-0.007088066	0.040968619	0.86289729
histidine.pcaac341	-0.000546675	0.003160715	0.862938647
pcaac321.pcaec386	0.001344875	0.007775949	0.86294349
lysine.pcaec301	0.008985329	0.051973066	0.862997498
smc240.pcaac323	0.011412226	0.066023814	0.863024323
lysopcac160.h1	-0.000268349	0.001553017	0.863069926
pcaac364.pcaac300	-0.010921266	0.063233989	0.863132744
pcaec380.asparagine	-0.002223796	0.012878183	0.863158322

smc240.pcaec403	0.00493203	0.028563803	0.863167917
pcaac344.pcaec362	0.00511163	0.029608044	0.863186627
asymmetricdimethylarginine.arginine	0.012078126	0.069974911	0.863215465
pcaec300.pcaec445	-0.010043248	0.058211275	0.863274737
pcaec405.pcaac361	-0.006079001	0.035253757	0.86334961
c162.c12	0.010497733	0.060901104	0.863398338
phenylalanine.smc260	0.003805707	0.022081932	0.86342076
ornithine.smohc241	-0.010273711	0.059644474	0.863495845
pcaec300.pcaec401	0.008924038	0.051813147	0.86350695
pcaac365.smohc161	-0.002500849	0.014531035	0.863609736
lysopcac204.pcaac320	-0.005562224	0.032323836	0.86363006
glutamicacid.ornithine	0.005922703	0.03442392	0.863650572
pcaac425.alanine	-0.002132201	0.012396434	0.863690417
c162.pcaac240	-0.001538027	0.008942207	0.8636942
lysopcac182.pcaec386	0.001231175	0.007158875	0.863707993
pcaac343.pcaac383	-0.000591141	0.003439056	0.863777274
c8.pcaac364	-0.009312379	0.054196744	0.863828321
ornithine.c14	-0.003727758	0.021701063	0.863865798
smc240.pcaec385	-0.007312208	0.042574459	0.863886692
pcaac344.lysine	-0.010481438	0.061104236	0.864057209
pcaac323.pcaac321	0.003308291	0.019295286	0.864118262
pcaac402.valine	0.003710715	0.021642659	0.864119974
pcaac342.pcaec341	0.00737853	0.043057997	0.864191512
pcaec446.c8	-0.005263476	0.030715705	0.86419271
smc161.pcaac320	-0.007009862	0.040919058	0.864232446
c18.c14	0.0041684	0.024359857	0.864383777
glycine.pcaec445	-0.00751783	0.043944827	0.864417763
c18.serine	-0.006596628	0.038571935	0.864459241
pcaec404.valine	0.004480523	0.026222344	0.864580764
c5.smc202	0.013436118	0.07868549	0.864666569
methioninesulfoxide.pcaec422	-0.010348858	0.06062669	0.864713037
pcaac360.pcaac320	-0.007549112	0.044227906	0.864722007
pcaac363.pcaac403	0.000890488	0.005224812	0.864919948
pcaec423.pcaac364	-0.006204757	0.036412019	0.864943745
pcaac381.pcaac421	0.003649411	0.02142789	0.865016599
lysopcac281.pcaac344	0.007900453	0.046437011	0.865156772
smc260.methioninesulfoxide	0.001341306	0.007886905	0.865207973
glycine.smohc161	-0.001850841	0.010884534	0.865226982
smc260.serotonin	0.009697084	0.057054535	0.865291005
leucine.smc161	0.008084372	0.047580449	0.865331934
lysopcac160.methioninesulfoxide	0.004938879	0.029078396	0.865381001
pcaec361.pcaec341	0.009541987	0.056182986	0.865388345
lysopcac204.pcaec401	0.006232292	0.036728763	0.865508777
pcaec385.pcaec362	-0.002928685	0.017272618	0.865609036
lysopcac160.pcaac361	0.00879547	0.051873818	0.865610155

c3.pcaac321	-0.003000716	0.017699044	0.865621096
glycine.pcaac403	-0.000717597	0.004233367	0.865645752
isoleucine.lysopcac140	0.000333043	0.001965061	0.865667261
lysopcac180.lysopcac281	-0.002274107	0.013424172	0.86572883
c51.threonine	0.003782774	0.02234624	0.865826
pcaac402.lysopcac280	0.002252892	0.01332436	0.865982562
pcaac341.smohc141	0.002826217	0.016721216	0.866030353
pcaac240.pcaac361	0.003224069	0.019079998	0.866064461
smohc221.pcaac240	-0.00130889	0.007748573	0.866108575
smc181.pcaac383	-0.000471005	0.002789666	0.866172107
pcaec363.c16	0.00763709	0.045237242	0.866184806
pcaac365.smc261	0.005691225	0.033715271	0.866200892
pcaec402.pcaac403	0.001517574	0.008991908	0.866225602
pcaac405.taurine	0.003802151	0.022543364	0.866313294
c4.pcaac363	0.000863697	0.005122441	0.866351735
alanine.valine	0.008378692	0.049695411	0.866359219
pcaec385.pcaec401	0.006855871	0.040665362	0.866365916
pcaec381.c14	0.008280583	0.049121458	0.866380635
isoleucine.h1	-0.00021886	0.001298366	0.866386791
smc161.pcaec386	-0.00251872	0.014944795	0.866411043
pcaec343.pcaac300	-0.012926982	0.07670808	0.866421356
pcaec443.pcaec403	0.010101312	0.059946125	0.86643335
smohc141.c4	-0.002520242	0.014960866	0.866473275
lysopcac161.lysopcac203	-0.004605895	0.027346003	0.866493193
pcaec406.asparticacid	-0.006849367	0.040697926	0.866597295
pcaac322.lysopcac160	-0.0023425	0.013928933	0.866693531
phenylalanine.pcaec386	-0.000901836	0.005362501	0.866693946
pcaac421.pcaac364	0.007166712	0.042617203	0.866701656
c3.pcaac343	0.002220026	0.013204307	0.866729806
lysopcac181.ornithine	-0.006291529	0.037429716	0.866761036
pcaec405.kynurenine	-0.00153296	0.009121994	0.866791027
pcaec386.c142	0.006172959	0.03673417	0.866796435
pcaac380.smc161	0.012493669	0.074365471	0.866828188
pcaec406.lysopcac281	0.002003949	0.011930631	0.866857145
smohc241.pcaac401	-0.001216627	0.007255949	0.867087766
pcaac240.lysopcac280	-0.006011182	0.03587029	0.867159979
pcaec340.lysopcac160	0.008297844	0.04952179	0.867176964
pcaec404.c102	-0.004101161	0.024482771	0.867214108
smc241.asparticacid	-0.004606824	0.027503159	0.86722237
pcaac405.pcaec405	-0.005833798	0.034828517	0.867223158
pcaac321.smohc222	-0.000941417	0.005620419	0.867224095
smc160.c102	0.003301398	0.019730024	0.867358216
glycine.smc161	-0.0055252	0.033044946	0.867457207
methionine.pcaec340	0.009444428	0.056496614	0.867484323
glutamine.c10	0.001071904	0.006412504	0.867491854

pcaec383.pcaac323	-0.010766416	0.064475954	0.867629298
putrescine.asparticacid	-0.009035399	0.054111003	0.867632813
c162.serine	0.006019562	0.0360641	0.867684716
pcaec364.serine	-0.012449324	0.074599107	0.86770816
lysopcac180.pcaac385	0.002417851	0.014488519	0.867710124
serotonin.pcaac381	0.00451764	0.027085179	0.867778217
pcaec322.pcaec420	0.003458411	0.020738742	0.867804077
pcaac344.pcaec446	-0.007776257	0.046634542	0.867813515
pcaec322.asparticacid	-0.006004735	0.036011179	0.867815442
citruline.pcaac385	0.00104875	0.006291258	0.867852235
pcaac344.pcaec421	0.000434904	0.002610523	0.867933214
pcaac385.pcaec380	-0.010131969	0.06082013	0.867939012
glycine.methioninesulfoxide	-0.003214028	0.019296945	0.867964747
alanine.pcaac320	-0.002609386	0.015666866	0.867966203
pcaac386.phenylalanine	0.004288239	0.025748277	0.867973875
pcaac381.pcaac425	0.018426494	0.110693838	0.868037559
lysine.pcaac385	0.003640885	0.021892121	0.868157958
serine.smc181	0.001803043	0.010855399	0.868325921
lysopcac160.smc181	0.001206446	0.007264516	0.868343701
pcaac321.pcaec362	0.004141329	0.024937477	0.868347882
lysopcac203.asymmetricdimethylarg	-0.003057491	0.018415485	0.868379473
pcaac422.c101	0.003041208	0.018324638	0.868430917
c162.smc161	0.00978188	0.058954851	0.868463303
pcaec364.smc241	0.014835209	0.089466956	0.868544829
pcaec341.h1	0.000196621	0.001186459	0.868621001
pcaec424.smc180	0.000732954	0.004423844	0.868650933
pcaac300.pcaec425	-0.002238346	0.0135162	0.868712169
pcaec445.pcaac320	0.008409979	0.050812703	0.868787055
c101.pcaec342	0.007989746	0.04829496	0.868844357
pcaec364.pcaec365	0.00941349	0.056955038	0.86896777
pcaec380.c4	-0.002548385	0.015423407	0.869007756
lysopcac280.pcaac323	0.01244661	0.075337554	0.869021256
pcaec380.pcaac383	-0.002553162	0.015464889	0.869113272
creatinine.acetylornithoine	0.005669955	0.034348559	0.869131336
smc241.lysopcac140	0.000144998	0.000878422	0.869134822
pcaec380.serotonin	0.014917567	0.090413522	0.869192964
pcaac281.pcaac320	-0.006940309	0.042073306	0.86922056
pcaac341.ornithine	-0.00223474	0.013550799	0.869253371
pcaac424.valine	0.005019288	0.03044164	0.86927966
pcaac362.lysopcac281	0.001742749	0.010571347	0.86930037
kynurenine.glutamine	-0.004047582	0.024576295	0.869427166
lysopcac182.pcaec425	0.001004333	0.006098358	0.869431401
c4.pcaac364	-0.004470586	0.027148885	0.869447062
c2.asymmetricdimethylarginine	0.002475194	0.015035323	0.869481601
c8.pcaec423	-0.010792658	0.065588522	0.86953993

pcaac422.valine	0.006369098	0.038726839	0.869609766
pcaec405.methioninesulfoxide	0.002914276	0.017721051	0.869617116
smc241.pcaec402	-0.007486113	0.045529946	0.869641482
ornithine.pcaec342	-0.008811565	0.053605405	0.869675627
isoleucine.c141oh	-0.01941909	0.118198654	0.869743443
c102.c2	0.011436816	0.06961532	0.869748214
pcaac406.lysopcac204	0.004377819	0.026653397	0.869776458
putrescine.c101	0.002916908	0.017764125	0.869814012
smc180.pcaac383	0.000568382	0.003461678	0.869821521
pcaac422.pcaac404	-0.000940673	0.005729219	0.869824553
pcaac381.h1	0.000140162	0.0008539	0.869860622
smc261.serotonin	-0.008179372	0.049848505	0.86990637
c102.lysine	-0.004878975	0.029735539	0.869910833
pcaec401.asymmetricdimethylarginir	0.004353074	0.026579428	0.870148793
lysine.c141oh	0.015207342	0.092878078	0.870181489
pcaac342.pcaac344	-0.011661866	0.071227556	0.870187441
pcaac384.pcaac364	0.011348918	0.069341812	0.870235045
smohc221.pcaec425	0.001094647	0.006689638	0.870261014
kynurenine.lysopcac281	-0.001020005	0.006233977	0.870271223
phenylalanine.pcaac300	-0.004634577	0.028326995	0.870279369
smohc222.pcaec362	0.002509299	0.015339931	0.870303198
pcaac343.leucine	0.001127624	0.006893584	0.870306151
c51dc.smohc222	-0.001037263	0.006349912	0.870482932
smc180.pcaac381	0.005041055	0.030888746	0.87060117
c51dc.pcaec423	-0.003088037	0.018948304	0.870780895
kynurenine.ornithine	-0.004008811	0.024600605	0.870793378
pcaec383.lysopcac281	0.00257016	0.015772529	0.870796615
pcaec402.glycine	-0.002656238	0.016301312	0.870800856
histidine.h1	-0.000352701	0.002166731	0.870931242
pcaac383.pcaec405	0.008551082	0.05253619	0.870942873
alphaaminoadipicacid.c142	-0.005084781	0.031266629	0.871052194
lysopcac181.h1	0.00175856	0.010819647	0.871124912
c141.pcaac424	-0.001302689	0.008022834	0.871251581
pcaec406.creatinine	-0.004739943	0.029196629	0.871272946
pcaec381.serine	-0.007561556	0.04659769	0.871329822
c4.c16	0.003579822	0.022069646	0.871382874
pcaec362.glutamicacid	-0.005253632	0.03239815	0.87142005
pcaec321.lysopcac161	0.010612081	0.065532303	0.871594335
pcaec361.pcaec301	0.009588755	0.059232219	0.871635626
pcaac401.pcaec341	0.005121819	0.03164251	0.87165054
glutamine.pcaac361	0.005353434	0.033104355	0.871769426
pcaec422.pcaac344	-0.006270553	0.038800843	0.87185211
c4.acetylornithoine	-0.009136156	0.056540395	0.871869662
pcaec422.smohc241	-0.007523334	0.046576292	0.871916234
pcaec322.c142	-0.006992598	0.043290649	0.871916515

c162.pcaec365	-0.002921018	0.018091454	0.871970208
smohc222.glutamine	-0.001820332	0.011288647	0.872131448
pcaec386.c16	0.005458315	0.033851656	0.872140183
pcaec422.putrescine	-0.005828791	0.036158495	0.872172445
pcaac405.c12	0.008438428	0.052369943	0.872227488
pcaec406.c162	-0.008279758	0.051387971	0.872234277
pcaac361.asymmetricdimethylarginir	-0.003118685	0.019368461	0.872315835
c18.c10	0.004871743	0.030256614	0.872319452
glycine.pcaac420	0.012087517	0.075121608	0.872404468
pcaac363.smc180	-0.001205327	0.007493155	0.872442928
c4.lysopcac180	0.00827277	0.051449106	0.872491566
asparagine.pcaec386	0.0008603	0.005353306	0.872562903
c102.lysopcac280	-0.000644571	0.004012562	0.872614897
smc181.glycine	0.006316283	0.03932013	0.872615621
spermidine.lysine	0.004713118	0.02937604	0.8727701
pcaec362.c3	-0.003227548	0.020118453	0.872780835
pcaec342.smohc161	0.001117634	0.006967133	0.872790266
pcaac402.pcaac403	0.002000161	0.012473445	0.87283881
c2.pcaac364	-0.00480436	0.030010771	0.873047653
c5.pcaac425	-0.01603025	0.100171344	0.873094488
pcaec404.pcaec446	0.008009612	0.05005453	0.873102799
pcaec386.c182	0.004372098	0.027333782	0.873154335
asymmetricdimethylarginine.phenyla	0.004500103	0.028137648	0.873170409
pcaac402.pcaec422	-0.008366459	0.052321758	0.873192202
asymmetricdimethylarginine.seroton	0.012107627	0.075758108	0.873258608
smohc222.pcaac360	-0.006242655	0.039086671	0.873342277
pcaec363.c142	-0.006531764	0.040912369	0.873389926
pcaac361.tyrosine	0.005422716	0.033966959	0.873394456
asparticacid.c162	0.012370294	0.077490645	0.873402957
serotonin.c16	-0.005277948	0.033088919	0.873503563
c12.threonine	0.003946302	0.024749215	0.873547908
pcaec443.methionine	-0.004041864	0.025357725	0.873593382
smohc222.alphaaminoadipicacid	-0.002533976	0.015917951	0.873753769
histidine.pcaac380	0.008859254	0.055659927	0.873771283
smc181.pcaac381	0.005476891	0.034416507	0.873796407
lysopcac170.c101	-0.001244174	0.007824783	0.873899606
pcaac401.c8	0.006612358	0.041596494	0.873931084
serotonin.c101	-0.000845455	0.005319122	0.873945237
c14.c10	0.000667904	0.004203234	0.873979781
pcaec360.lysopcac181	-0.003976677	0.025035633	0.874028309
pcaec444.pcaec445	0.013392121	0.084339719	0.87406987
pcaec361.c51	-0.008980134	0.056577257	0.87412055
pcaac366.c8	0.005105637	0.032169756	0.874131681
alanine.methioninesulfoxide	0.003183622	0.020069722	0.874195477
pcaec445.pcaec301	-0.007314329	0.04611431	0.874207362

pcaec382.c12	0.01191218	0.075183449	0.874342213
lysine.lysopcac281	0.000965777	0.006099554	0.874425385
alphaaminoadipicacid.asparticacid	-0.00272964	0.017244266	0.8744593
pcaac362.methioninesulfoxide	0.001681926	0.010629518	0.874507277
smc240.pcaac344	-0.006399513	0.040454093	0.874538452
smohc141.c51dc	-0.015178339	0.095972232	0.874568765
pcaec380.c102	-0.005353628	0.03385631	0.874588845
pcaac420.pcaec365	-0.003731777	0.023602585	0.874603851
lysopcac161.pcaec382	0.001336672	0.008458377	0.874666367
c12.methioninesulfoxide	0.002103762	0.013314484	0.874685127
pcaec380.pcaac384	0.006345169	0.040166063	0.874710368
pcaec385.pcaec343	0.013156621	0.083334944	0.874786659
pcaac425.pcaac360	-0.004306268	0.027304799	0.874916732
smohc161.glutamine	-0.002172969	0.013782649	0.874957028
smc160.pcaec322	0.013019011	0.082596548	0.874986951
pcaec343.asparagine	0.001057895	0.006713358	0.875019288
c0.pcaac360	-0.003335561	0.021167787	0.875021983
pcaec445.pcaec425	0.000480412	0.003049154	0.875038917
arginine.c141oh	0.012607763	0.08003944	0.875067455
pcaec446.c142	0.004960701	0.031494974	0.875076638
pcaec403.lysopcac181	-0.008789395	0.05580959	0.875091378
pcaac420.pcaec363	-0.00492334	0.031266882	0.875112737
pcaac424.c18	0.008409997	0.053428631	0.875156487
pcaec381.pcaec383	-0.024214951	0.153938114	0.875237531
smohc241.pcaac321	-0.00314578	0.019999195	0.875243673
pcaec322.pcaac421	0.004931886	0.031369316	0.875302879
lysopcac161.isoleucine	-0.005817876	0.037018608	0.875349468
c10.glutamicacid	-0.007443966	0.047365458	0.875349963
pcaac364.putrescine	0.00525574	0.033445331	0.875362558
pcaec385.pcaac343	-0.003168893	0.020166028	0.87536573
asparticacid.pcaac365	0.00926426	0.058970493	0.875397385
pcaec421.taurine	0.005948957	0.037885812	0.875457632
pcaac300.pcaac383	0.000745173	0.004745883	0.87546452
pcaec340.asymmetricdimethylarginir	0.003301999	0.021038324	0.87551403
pcaac385.pcaac343	-0.002852589	0.018180733	0.875553277
pcaec383.taurine	0.010384791	0.066187653	0.875555264
pcaec421.pcaac403	0.005490572	0.035027009	0.875670651
pcaec380.taurine	0.007885503	0.050317325	0.875699808
c2.h1	0.001195822	0.007631022	0.875707804
smohc221.lysopcac170	-0.001742409	0.011127954	0.875806794
h1.pcaac323	-0.01655221	0.105713612	0.875809552
pcaec342.lysopcac140	-0.00013958	0.000891676	0.875840274
pcaac401.smohc241	-0.008377683	0.053535786	0.875879118
pcaec386.pcaec401	-0.006219912	0.039761574	0.875924188
pcaac402.asymmetricdimethylarginir	-0.004358147	0.027863583	0.875939987

pcaec301.pcaac403	-0.000623049	0.003986201	0.87602543
pcaac401.threonine	-0.003035074	0.019441485	0.876173545
pcaac425.pcaec385	0.007276307	0.046613074	0.876183875
pcaec386.smc260	-0.005361267	0.034347924	0.876194162
pcaec343.pcaec401	-0.007653642	0.049041476	0.876211779
pcaac405.methioninesulfoxide	0.004650805	0.029805827	0.876233722
smc260.ornithine	0.004385455	0.02810607	0.876237229
lysopcac160.pcaec385	0.004260355	0.027320494	0.876309929
pcaac401.pcaec445	-0.014357646	0.092102929	0.876351585
pcaac403.pcaec405	-0.017951152	0.115225078	0.876426253
pcaac344.pcaac406	-0.005889332	0.037816316	0.876470979
pcaac240.pcaec386	0.002583775	0.016606405	0.876585973
c18.pcaec425	0.001190207	0.007652971	0.8766386
pcaec380.histidine	-0.010571124	0.067991587	0.876674177
c3.lysine	0.013772784	0.088586435	0.87667741
pcaac380.pcaec322	0.0160262	0.103133481	0.876740384
pcaec405.pcaac240	-0.002819003	0.018153158	0.876821324
pcaec364.pcaac300	-0.007364882	0.047478368	0.876954405
lysopcac182.pcaac240	-0.001546801	0.009972936	0.876970892
pcaec340.smc202	0.005417333	0.034956865	0.877071534
pcaac406.pcaac363	0.001778614	0.011479938	0.877102634
leucine.c51	-0.003433649	0.022166391	0.877125478
pcaec301.pcaec405	-0.006585848	0.04252911	0.877163486
pcaac383.c51	-0.004833807	0.031222558	0.877192832
smc160.taurine	0.007366834	0.047589459	0.877207046
pcaec443.pcaac361	-0.005756838	0.03720345	0.877254541
glutamine.smc180	0.001067521	0.006901098	0.877294523
smohc141.alphaaminoadipicacid	-0.001655713	0.010704476	0.877305306
pcaac380.c4	-0.001669835	0.010800241	0.877355592
pcaac386.pcaec340	0.007086604	0.045865977	0.877437554
glutamine.lysopcac170	0.001323356	0.008566286	0.877455288
smohc161.smc160	0.017636142	0.11420756	0.877504391
pcaec402.smc241	0.009642449	0.062466913	0.877552345
pcaac403.histidine	0.012856603	0.083305202	0.877575617
alanine.serotonin	0.00997235	0.064631996	0.877604738
lysopcac160.smc160	-0.008343961	0.054092005	0.877635708
pcaac403.pcaec446	0.007852707	0.050941903	0.877718111
pcaec364.pcaec421	0.000121726	0.000789738	0.87773046
c0.smohc241	-0.0080378	0.052173776	0.877790532
c51dc.putrescine	0.00600741	0.038997525	0.877800203
pcaec322.pcaec386	0.004179836	0.027153836	0.877890125
pcaac405.isoleucine	0.003332035	0.021665861	0.878000094
pcaec424.smc240	0.004861697	0.031631355	0.878073499
c51dc.c101	0.004144946	0.026976163	0.878110098
pcaac321.methionine	-0.003604159	0.023480861	0.878234977

smohc222.pcaec425	0.003437481	0.022412093	0.878327324
pcaac406.pcaec423	-0.000373629	0.002437088	0.878379661
pcaec443.lysine	0.011392281	0.074348856	0.878444422
c142.valine	0.008782831	0.057350091	0.878509927
pcaac404.pcaac322	0.009732988	0.063586668	0.878571059
pcaec423.pcaac381	0.005273954	0.034474355	0.878637608
methionine.pcaac300	0.006492881	0.042442885	0.878639726
smc181.pcaec420	0.002185017	0.014283736	0.878645161
c8.c182	0.004969303	0.032494329	0.878679818
pcaac426.smc260	-0.003142299	0.020557708	0.878739402
pcaec381.pcaec363	-0.005863206	0.038368013	0.878769054
spermidine.pcaac300	-0.026698151	0.174818917	0.878844658
pcaac381.c182	0.0072436	0.04745681	0.878910193
lysopcac281.smc180	0.000866268	0.005676829	0.87894051
c181.acetylornithoine	-0.002019459	0.013235647	0.878956152
creatinine.pcaac362	0.003925221	0.025744072	0.879039892
methioninesulfoxide.pcaec322	0.008469203	0.055550763	0.879049387
pcaac343.pcaac406	-0.00522591	0.034304128	0.879142463
c142.alphaaminoadipicacid	0.001448343	0.009515922	0.879251478
smc160.c162	0.010890572	0.071560618	0.879263603
pcaec405.valine	0.005955609	0.039137549	0.879275753
putrescine.pcaac360	0.006360314	0.041803629	0.879294505
pcaac300.pcaac424	-0.001377197	0.009052593	0.87930591
pcaac425.pcaec421	-0.000259702	0.001707118	0.879309035
pcaac385.c141oh	0.011786518	0.077488609	0.879326741
ornithine.pcaac344	-0.00602638	0.039622578	0.879336088
pcaac323.smc161	0.007847049	0.051619422	0.879396875
pcaec424.pcaec342	-0.011948776	0.078751475	0.879624952
pcaac342.c14	0.004150685	0.02736438	0.879660845
smohc141.pcaec421	0.00016231	0.001071257	0.879793617
pcaac362.pcaec382	-0.000661138	0.004363622	0.879795237
isoleucine.pcaec386	0.001407958	0.009292981	0.87979815
pcaec422.pcaec386	-0.001445787	0.009548288	0.879868398
c102.pcaec386	0.001282607	0.00847082	0.879871325
pcaec341.lysine	0.007189797	0.047489704	0.879885288
kynurenine.alphaaminoadipicacid	0.000518174	0.003426438	0.880018223
pcaac424.smc160	-0.004059431	0.026857743	0.880083158
methionine.smohc241	0.010009392	0.066239338	0.880111634
smohc241.lysopcac181	-0.004479581	0.029652144	0.880141883
pcaac321.smc160	0.00814189	0.053903871	0.880162695
pcaec445.pcaac385	0.008170276	0.054125715	0.880237199
h1.glutamicacid	0.005623213	0.037288397	0.880352714
h1.pcaac320	0.001609676	0.010674663	0.880359945
pcaec342.smohc241	-0.004465499	0.029614876	0.880366587
pcaec403.smc160	-0.007268424	0.048226031	0.880421608

c101.pcaac323	0.009077398	0.060241768	0.880447575
pcaec361.lysopcac170	-0.003437859	0.022820054	0.880472795
methionine.pcaec343	-0.010348249	0.068696233	0.880483012
pcaec424.valine	0.00514199	0.034135105	0.880484087
pcaec405.pcaac344	0.007111087	0.047235709	0.880556299
pcaec360.lysopcac182	0.001900359	0.012625686	0.880579484
c0.lysopcac180	-0.009133548	0.06074864	0.880709746
pcaec384.pcaec322	-0.012256875	0.081549254	0.880748783
serine.lysopcac281	-0.001042082	0.00693384	0.880757462
pcaac402.pcaec340	-0.006230075	0.041457664	0.880768207
pcaac366.pcaec446	-0.008904819	0.059267952	0.880790893
lysopcac180.pcaac425	0.011032863	0.073440771	0.880805657
lysopcac280.pcaec446	0.010895918	0.072557632	0.880852025
c51dc.ornithine	0.004720017	0.031442175	0.880892782
pcaec401.pcaac364	-0.006436619	0.042883665	0.8809105
pcaec362.c51dc	-0.016058254	0.106987804	0.880911006
c142.glutamicacid	0.007299301	0.048636718	0.880923803
pcaac361.methioninesulfoxide	0.003075108	0.020497629	0.880967411
c162.asymmetricdimethylarginine	-0.001083107	0.007219706	0.88096874
smc180.glutamicacid	0.003680711	0.024539952	0.880994275
pcaec300.c16	-0.005583671	0.037233673	0.881014412
lysopcac203.acetylornithoine	-0.005729112	0.038265332	0.881205163
pcaec380.pcaac363	0.003481369	0.023253252	0.881209361
pcaec364.taurine	0.003197135	0.021358475	0.881229881
serine.pcaec423	0.000901853	0.006028991	0.881311151
c3.pcaac425	0.01213581	0.081237186	0.881467659
pcaec360.h1	0.000424836	0.002844507	0.881494511
pcaac343.pcaac384	-0.009761598	0.06538134	0.8815345
pcaac401.pcaac363	-0.003404191	0.022800953	0.881536181
pcaac360.pcaec380	-0.010380341	0.069541357	0.881561204
pcaac401.creatinine	0.004270383	0.028629748	0.88164756
glutamine.pcaec403	0.005150993	0.034535017	0.881652434
c181.pcaac361	-0.005153305	0.03455775	0.88167701
pcaac365.pcaac281	-0.000526847	0.00353455	0.881728485
pcaac385.smc160	-0.011665284	0.078286726	0.88176709
pcaec444.glycine	-0.00230254	0.01545367	0.881775649
methionine.taurine	-0.003780053	0.025379741	0.881820156
smohc221.threonine	-0.004524636	0.030402272	0.881910103
pcaec382.smohc141	-0.003457453	0.023232706	0.881915813
lysine.pcaec446	0.006375206	0.042840436	0.881920162
smohc221.lysopcac203	-0.00182525	0.012269424	0.881958491
prolinr.pcaac343	0.00246825	0.01659278	0.881966123
pcaec300.threonine	-0.002921039	0.019637024	0.881968376
lysopcac170.pcaac420	0.011418471	0.076775971	0.881989692
pcaac422.smc180	0.000699599	0.004704797	0.882009824

pcaec406.pcaec322	-0.01492636	0.100389311	0.882021026
methionine.pcaac404	-0.001249981	0.008410659	0.882073051
pcaec380.prolinr	0.004584798	0.03085078	0.882078266
pcaac383.pcaac360	-0.003392093	0.022830753	0.882106987
serotonin.pcaec385	-0.006259741	0.042143936	0.882141003
pcaac401.transhydroxyproline	-0.006458242	0.04349393	0.882177523
pcaec401.pcaec361	0.002762942	0.018610325	0.882195778
pcaec342.pcaac360	0.00561179	0.037809893	0.88222861
pcaac424.alanine	0.002403112	0.016194287	0.882251173
phenylalanine.pcaac426	0.006512511	0.043898453	0.882281536
pcaec421.pcaac361	-0.006863995	0.046297083	0.882355752
methioninesulfoxide.pcaec445	0.013606669	0.091952168	0.88257966
pcaec300.lysopcac140	-7.84E-05	0.000530029	0.88267373
lysopcac182.pcaec343	-0.009630696	0.065195878	0.882781409
pcaac322.c142	-0.002661459	0.018030747	0.882870174
smc260.pcaec403	-0.001173592	0.007950858	0.882870935
pcaec381.pcaac420	-0.012576985	0.085221878	0.88289177
pcaac421.methionine	0.00454118	0.030775351	0.882907769
alanine.asparticacid	-0.00553585	0.037606943	0.883188304
pcaac420.lysopcac204	-0.006337094	0.043051031	0.883190893
pcaac240.c3	0.004978447	0.03383096	0.883224803
c2.pcaac343	0.002423853	0.01647455	0.883247992
pcaec444.asparagine	-0.001600584	0.010879439	0.883253485
alphaaminoadipicacid.pcaac320	0.004721813	0.032095413	0.883255169
pcaec445.lysopcac140	-0.000340968	0.00231896	0.883320588
c142.c51	0.004391835	0.029871496	0.883328994
asparagine.pcaac385	-0.002189111	0.014904741	0.883447803
acetylornithoine.pcaac401	-0.002339087	0.015926163	0.883449973
smc160.pcaac364	0.00571396	0.038924787	0.883509712
lysopcac181.pcaec360	0.001029483	0.007016656	0.883568841
prolinr.pcaec446	0.006192282	0.042205586	0.883570994
smc241.c12	0.005194655	0.035413573	0.883595948
isoleucine.pcaec402	-0.006568151	0.044777762	0.883597604
pcaac341.h1	0.000187098	0.001275671	0.883610981
pcaac420.pcaec322	0.009184562	0.062636009	0.883636559
c141oh.pcaac424	0.000577292	0.003937384	0.883648877
pcaec363.pcaec386	-0.000967868	0.006601297	0.883649143
glycine.pcaac363	0.001853044	0.012642461	0.883684513
pcaac384.pcaac406	0.004207348	0.028711789	0.883712653
smc261.c182	-0.003131509	0.021374158	0.883734852
pcaac385.serotonin	0.014296916	0.097603034	0.88375761
c18.pcaec445	0.00837834	0.057205519	0.883773292
alphaaminoadipicacid.creatinine	-0.002111035	0.014426853	0.88387854
h1.pcaac422	0.010630095	0.072647242	0.883880063
asparticacid.lysine	-0.009884743	0.067565852	0.883901266

pcaec421.c142	0.004105212	0.02806805	0.883931715
c141oh.h1	0.000323096	0.002209538	0.883956657
pcaac321.smc181	0.001819648	0.012447658	0.883991014
pcaac362.lysine	-0.00647117	0.044270392	0.883999085
pcaac360.c142	-0.005663017	0.038776753	0.884103262
c142.pcaec340	-0.003061363	0.020965705	0.884122081
smohc221.c14	0.003685178	0.025244888	0.884153969
c0.smohc141	0.002621679	0.01796028	0.884158908
pcaac424.pcaec386	-0.001662066	0.011389792	0.884194353
smohc221.asparagine	0.001347916	0.009254601	0.884413214
pcaec420.pcaac362	0.001701304	0.011684032	0.884443849
pcaec420.pcaec386	0.001610921	0.011065408	0.884465618
c10.pcaec423	0.009429835	0.064794308	0.884502449
smohc241.lysopcac161	-0.009817475	0.067486588	0.884551263
acetylornithoine.smc160	-0.009532548	0.065531796	0.884557973
pcaec405.pcaac403	0.002360549	0.016232139	0.884589523
pcaac300.lysopcac140	0.000114952	0.000790897	0.884653456
pcaac365.pcaac381	0.003756389	0.025847301	0.884663913
smc181.pcaac403	0.000574683	0.003954369	0.884665052
pcaac321.pcaec365	0.004816691	0.03315324	0.884698866
smc260.c3	-0.003601638	0.024814275	0.884810678
pcaec401.pcaac240	0.002092564	0.014420005	0.884833075
lysopcac140.glutamicacid	0.004317017	0.029750684	0.884840076
pcaac380.lysopcac281	0.001190237	0.008204215	0.884863822
lysopcac160.c12	-0.009314887	0.064220654	0.884888482
pcaec401.pcaec446	-0.006731901	0.04642216	0.884912297
pcaac385.pcaac320	-0.010825824	0.074657011	0.88491809
pcaac406.asymmetricdimethylarginir	-0.002090392	0.014423879	0.884982447
prolinr.pcaec422	0.004377191	0.030219942	0.885046566
creatinine.pcaac240	-0.001957442	0.013539043	0.885256871
pcaac425.pcaec363	0.003835471	0.026537381	0.885293671
glutamine.creatinine	0.005879523	0.040687064	0.88531332
c10.c182	-0.004515971	0.031259958	0.885345574
pcaac424.pcaac344	0.008185678	0.056720655	0.885463296
pcaec424.smc260	-0.004173685	0.028924345	0.885478295
arginine.lysopcac203	0.00120955	0.008384718	0.885509952
lysopcac280.pcaec402	0.010263626	0.071162582	0.885532493
pcaec421.pcaac320	-0.006518443	0.045247582	0.885663461
methioninesulfoxide.pcaac360	-0.008925727	0.061961622	0.885670688
c0.smc202	-0.004920222	0.034179727	0.885750315
pcaec381.pcaec446	0.010921625	0.075872874	0.885754336
c142.pcaac360	0.002742442	0.01906791	0.885850019
c14.lysopcac161	0.00675913	0.046997219	0.885854108
pcaec363.lysopcac280	0.001547618	0.010766052	0.885909287
pcaac363.pcaac366	0.004004043	0.027866535	0.885959226

pcaec362.smc161	-0.010200349	0.071004737	0.885982179
pcaac364.pcaac424	-0.001190316	0.008287815	0.886009698
smc240.c101	0.001712212	0.011923515	0.886027669
c181.c141	-0.004956262	0.034517456	0.886037506
pcaac341.pcaac405	-0.005577715	0.038858328	0.886074835
pcaec405.c3	-0.003319483	0.023127897	0.886084713
asparagine.pcaac320	-0.002418493	0.016855323	0.88611767
pcaec322.pcaac380	-0.008311106	0.057941529	0.886153823
pcaec322.pcaec402	0.008093851	0.056452837	0.886205736
pcaec343.pcaec403	0.004166587	0.029066974	0.886228835
glutamicacid.smc181	0.002604133	0.018171714	0.886258316
c16.pcaec362	-0.001895574	0.013230151	0.886282096
pcaec342.pcaac406	-0.007814997	0.054551642	0.886296371
pcaec420.pcaac300	0.004702459	0.032826125	0.886300413
smohc141.pcaac426	0.005877647	0.041043056	0.886337226
lysopcac181.pcaec365	-0.002635306	0.018410498	0.886388765
pcaac321.pcaac363	-0.002403427	0.016794533	0.886415387
pcaec406.pcaac362	0.00186327	0.013022478	0.886436363
pcaec406.c8	0.005823232	0.040707425	0.886460193
glycine.glutamine	0.00273632	0.019131333	0.886478055
smc161.lysopcac204	0.004859995	0.033996272	0.886534426
pcaac364.pcaec446	-0.004269835	0.029871276	0.886546667
c8.lysopcac170	-0.002262349	0.015830872	0.886573298
pcaac381.pcaac384	-0.005907862	0.041340921	0.886574451
pcaec362.c142	0.006460182	0.045246387	0.886675406
pcaac383.smohc241	-0.006597674	0.046221566	0.886705107
pcaec444.pcaac323	-0.009689132	0.067879946	0.886705835
pcaac322.pcaac424	0.001472964	0.010320569	0.886720098
pcaec444.pcaac405	-0.007614188	0.053361964	0.886745117
c102.c101	0.00298864	0.020948542	0.886763755
pcaec301.pcaec423	-0.000622269	0.004365032	0.886848907
pcaac401.valine	0.003394154	0.023823281	0.886916393
pcaac362.smohc161	-0.003097288	0.021747019	0.886954694
c14.asparticacid	0.004138998	0.029083579	0.887041142
c51dc.c10	0.000819468	0.005759806	0.887072936
lysopcac140.pcaac341	0.000670419	0.004713071	0.887094078
pcaac281.asymmetricdimethylarginir	-0.002300049	0.016181423	0.887177219
pcaac420.pcaac364	0.004684055	0.032974617	0.887248939
pcaac404.pcaec362	0.001914102	0.013474869	0.887249385
pcaec423.pcaac363	-0.003569732	0.025137908	0.887283926
c5.pcaac360	0.005085907	0.035823464	0.887311178
c10.putrescine	0.004175052	0.02941247	0.887329321
smc240.ornithine	0.00376632	0.026533181	0.887329958
pcaac383.pcaac363	-0.001987089	0.014003002	0.88736393
c12.c142	-0.006125268	0.043173405	0.887386404

methionine.pcaac320	-0.002223717	0.015677578	0.887414292
c51dc.pcaac383	0.001585509	0.011178994	0.887423162
pcaac422.pcaec361	0.001797104	0.012677904	0.887485045
pcaec321.lysopcac182	0.002717354	0.019187037	0.887584683
pcaac380.pcaec423	-0.000436725	0.003085421	0.88764756
pcaec341.pcaac240	-0.001975391	0.013959522	0.887676099
pcaec383.c142	0.004035666	0.028519442	0.887678274
c18.pcaec300	0.005555897	0.039264322	0.887682914
pcaec401.pcaec341	-0.003280424	0.023195779	0.887743273
pcaac421.c101	0.006728179	0.047612074	0.887830685
citruline.pcaec343	0.009486256	0.067136039	0.887841289
pcaec384.pcaec340	0.009252428	0.065487357	0.887851771
c162.smohc241	-0.005899091	0.041762809	0.887878153
pcaec321.taurine	0.003150862	0.022310699	0.887898475
glycine.pcaec364	-0.003363142	0.023828659	0.887967826
lysopcac203.pcaec365	0.002392008	0.016956176	0.888021855
pcaac362.smohc241	-0.008044526	0.057064791	0.888099301
c4.pcaec365	-0.004673815	0.033159526	0.888116967
acetylornithoine.pcaec420	-0.0034558	0.024518076	0.888117195
c141oh.smohc161	0.00090565	0.006427982	0.888162329
glutamine.lysopcac182	0.00412793	0.029304913	0.888186362
arginine.h1	-0.000257084	0.001826553	0.888275437
pcaac385.isoleucine	0.003801766	0.027012637	0.888281857
smc260.c162	-0.010183827	0.072406436	0.888354539
c4.smohc141	-0.001902767	0.013529023	0.88835831
smc202.valine	-0.00821851	0.058440757	0.888368988
smc261.pcaac424	-0.001495953	0.010638162	0.88837558
pcaac360.pcaec422	-0.002530188	0.01799772	0.888405214
pcaec402.c14	0.00795365	0.056584605	0.88842236
pcaec424.lysopcac203	0.001660078	0.011817868	0.888493506
smc261.pcaec341	0.010502536	0.074811482	0.888560676
pcaec381.methionine	-0.002217995	0.015807659	0.88862005
prolinr.c141	0.006188144	0.044112008	0.888642816
pcaac404.phenylalanine	0.003726255	0.026570248	0.888675052
isoleucine.pcaec341	0.003663767	0.026143948	0.888756589
creatinine.pcaec343	-0.008633814	0.061656167	0.888840663
lysopcac203.pcaac401	-0.000554063	0.00395671	0.888841045
ornithine.pcaec343	-0.009381221	0.067021903	0.888887326
pcaac422.histidine	-0.009777589	0.069912924	0.88898089
lysopcac182.pcaec385	0.004400507	0.031497459	0.889094386
pcaec420.smohc221	0.013132424	0.094021925	0.889122646
pcaac344.smohc241	-0.005770456	0.041333082	0.889174223
pcaec362.pcaec446	0.004192511	0.030050388	0.889247265
smohc222.lysopcac161	0.008670111	0.062151562	0.889260331
phenylalanine.h1	0.000613754	0.00440056	0.889282241

c142.smc160	0.007122467	0.051082348	0.889314271
pcaec401.pcaec404	0.002695143	0.019332078	0.88932854
pcaac323.c2	-0.006547083	0.046977443	0.889365188
pcaac364.pcaec382	0.001357237	0.009745359	0.889441244
putrescine.acetylornithoine	0.003284574	0.023596158	0.88949691
pcaac380.lysopcac182	-0.001458142	0.010485391	0.889603638
pcaec321.glutamicacid	0.006051417	0.043533024	0.889648357
leucine.pcaec406	-0.002009197	0.014454367	0.889652086
pcaac386.smc202	0.003289915	0.023678615	0.889701333
lysopcac180.methioninesulfoxide	0.005509486	0.039655169	0.889705628
putrescine.pcaec423	-0.00054846	0.003948279	0.889724242
pcaec402.pcaec386	0.001839183	0.013253071	0.889832454
pcaec401.lysopcac182	-0.006514369	0.046964152	0.88988348
pcaac321.methioninesulfoxide	-0.006417694	0.0462778	0.88990856
pcaac403.smc180	0.000369137	0.002662831	0.889949586
pcaec382.pcaac342	-0.003133602	0.022622077	0.890033141
histidine.pcaec386	0.000909701	0.006570029	0.89007846
smohc161.lysine	0.006627466	0.047865777	0.890080678
pcaac381.pcaec423	-0.000614115	0.004435621	0.89008748
pcaac424.threonine	-0.003119802	0.022554397	0.890187915
alanine.smc180	-0.005411177	0.039127079	0.890208379
acetylornithoine.pcaec363	-0.002312025	0.016719636	0.890220601
pcaec444.smc240	0.003248132	0.023508944	0.890312244
pcaac320.lysopcac203	0.001467986	0.010625486	0.890319171
c141oh.pcaac420	0.014185701	0.102750522	0.890395992
smc261.prolinr	-0.004156068	0.030113672	0.890433065
c181.creatinine	0.001170861	0.008486313	0.89046629
smohc221.c101	-0.000955611	0.006926402	0.890469539
pcaec360.pcaac320	-0.005803105	0.042083597	0.890526106
pcaec342.pcaac300	-0.010438906	0.075735105	0.890573641
asymmetricdimethylarginine.pcaac38	-0.005069152	0.036797516	0.89063394
ornithine.lysopcac170	-0.002962711	0.021510414	0.890653016
c182.pcaec446	-0.007687265	0.055827145	0.890681567
pcaec423.methioninesulfoxide	0.00138843	0.010089446	0.89074902
pcaec361.threonine	-0.003302331	0.023998082	0.890752187
pcaec361.smohc141	-0.00333234	0.02421834	0.890761953
pcaec321.c162	0.007814247	0.056798059	0.890774779
pcaec423.c8	0.004970619	0.036135477	0.890794
acetylornithoine.pcaec424	-0.002299216	0.016721001	0.89083376
lysopcac160.lysopcac281	0.001281593	0.009322374	0.890857182
lysopcac140.c12	-0.008571909	0.062367259	0.890882869
pcaac344.pcaec386	0.001887358	0.013739454	0.890941793
pcaac402.pcaac420	0.017117488	0.124645589	0.890972158
serine.smohc141	0.008267234	0.060232972	0.891031301
smohc241.pcaec380	0.004881506	0.035573885	0.891057076

lysopcac161.pcaec420	0.005767505	0.042032685	0.891062491
pcaec404.c18	0.005254948	0.038299416	0.891068611
pcaac402.c141	0.00705965	0.051508661	0.891186529
pcaec445.smc260	-0.005983519	0.043685491	0.891257084
c182.asymmetricdimethylarginine	0.00147517	0.010771309	0.891268507
pcaac300.asymmetricdimethylarginir	-0.002938894	0.021467759	0.891312374
pcaec343.pcaec380	0.003184638	0.023273312	0.89136094
methionine.pcaac424	0.001022685	0.007474834	0.891376253
pcaec424.pcaac424	0.002251818	0.016478884	0.891509106
pcaac383.smc260	-0.002868147	0.020989545	0.891510859
pcaec406.h1	-0.000350339	0.002564148	0.891523974
lysine.pcaec343	-0.009507209	0.069593662	0.8915394
pcaec403.pcaac342	-0.004235392	0.03101503	0.891579591
smohc141.pcaac343	-0.00309245	0.022649761	0.891600025
pcaec365.isoleucine	-0.002396396	0.017555196	0.891621364
pcaec382.glycine	-0.002241995	0.016427092	0.891640955
smc260.pcaac424	0.001092369	0.008004427	0.89164961
lysopcac140.pcaac381	-0.005815107	0.04263653	0.891714857
pcaac362.pcaac366	-0.009595404	0.070362756	0.89172858
lysopcac140.pcaac343	0.003124485	0.02291249	0.891732131
pcaec406.glutamicacid	0.005512628	0.040452366	0.891804278
pcaac321.c10	0.000873631	0.006411166	0.891810115
pcaac403.putrescine	0.006765138	0.049655438	0.891830259
putrescine.lysopcac170	0.001642939	0.012062493	0.891861316
smc260.c182	0.004557642	0.03346915	0.891883289
lysopcac181.c12	-0.006956659	0.051104113	0.891920555
ornithine.c3	-0.003575328	0.026269419	0.891940218
smc161.alanine	-0.002373203	0.017438198	0.891948166
citruline.methioninesulfoxide	-0.001400445	0.01029504	0.891996465
lysopcac280.smc202	0.004271935	0.031408774	0.89201233
h1.c101	-0.003483022	0.025616301	0.89204541
pcaec446.pcaec301	0.007308941	0.053785812	0.892107907
pcaac323.pcaac341	-0.000542356	0.003993475	0.892170352
c51dc.lysopcac161	0.007646388	0.056374875	0.892309181
lysopcac281.pcaac320	-0.006742381	0.0497129	0.892315729
pcaec404.glycine	-0.00243469	0.017966759	0.892406937
c101.pcaac360	0.002655397	0.019605802	0.892463321
smc181.pcaac426	0.005514118	0.040718317	0.892477739
smc160.pcaec421	0.000281393	0.002080716	0.892621619
pcaac422.c51dc	0.013729101	0.101583274	0.892690702
pcaac281.isoleucine	0.002998028	0.022184234	0.892697742
pcaec444.pcaec405	0.00574383	0.04252191	0.892747429
pcaec341.pcaec362	-0.007589083	0.05618598	0.892754176
pcaec361.pcaec386	-0.00076694	0.005679418	0.892779665
pcaec321.c182	-0.005853096	0.043367168	0.892836798

lysopcac170.smc202	-0.004855937	0.035981403	0.892844065
pcaec381.pcaac360	-0.008118814	0.060217938	0.89294903
arginine.lysine	0.01160046	0.086100984	0.893022409
pcaec340.pcaac364	-0.005564802	0.041365721	0.893183374
pcaec443.asymmetricdimethylarginir	0.003375923	0.025101752	0.893212866
pcaec382.c182	0.0054761	0.04072229	0.893224921
pcaac300.alphaaminoadipicacid	-0.000869786	0.006469901	0.893255315
kynurenine.c10	0.003746097	0.027866674	0.893260429
pcaec364.pcaac383	0.000518293	0.003856373	0.893284341
pcaec381.lysopcac281	0.001819853	0.013570593	0.893518158
pcaec384.pcaec401	0.005452077	0.040687185	0.893599331
pcaec343.c51	-0.006501232	0.048520667	0.893607966
c3.c14	-0.001826528	0.013635864	0.893638466
c2.pcaec406	-0.00299005	0.022331369	0.893682409
pcaac360.glutamicacid	0.007125815	0.053237435	0.89371785
lysopcac281.pcaac406	0.004151605	0.031032245	0.893770031
pcaec425.smohc241	-0.006220269	0.046509713	0.89380342
pcaec362.pcaec385	-0.007039703	0.052644089	0.893818189
c10.pcaec425	-0.003551774	0.026575955	0.893878507
pcaec322.taurine	0.003761686	0.028162253	0.893937111
prolinr.pcaec361	0.001505353	0.011281627	0.894045932
pcaec301.leucine	0.001544736	0.011579621	0.894071814
histidine.smohc141	0.004316206	0.032355209	0.894072291
pcaec364.pcaac364	0.007123758	0.053439259	0.894147229
pcaac240.pcaac383	0.000738614	0.005542737	0.894184915
acetylornithoine.pcaac363	0.0010636	0.007982899	0.894203241
pcaac401.serotonin	-0.012830188	0.096358197	0.894269358
c18.pcaac421	-0.006560765	0.049349342	0.894431676
lysopcac180.pcaec444	0.012263549	0.092325696	0.894523358
c5.pcaac362	-0.003771598	0.028418606	0.894612876
pcaac360.asymmetricdimethylarginir	0.005262278	0.039653646	0.894620581
pcaec401.pcaec423	-0.004160635	0.031356503	0.894634733
asparagine.c142	0.003615355	0.027249938	0.89464597
pcaec340.pcaec423	0.000677769	0.005108944	0.894654403
c181.ornithine	-0.003020883	0.022786744	0.894726444
pcaac322.c141oh	0.008717616	0.065765657	0.894739222
pcaec381.c182	0.005575736	0.042085056	0.894793239
pcaec343.pcaac281	0.000169782	0.001282183	0.894849146
pcaec424.pcaec341	0.003064808	0.023145497	0.894850432
lysopcac180.pcaac361	-0.007597087	0.057400918	0.894900614
pcaec421.smohc241	-0.006384889	0.048243232	0.894903336
c142.smc240	-0.004303419	0.032524005	0.894929139
pcaec403.pcaac420	-0.010015478	0.075824839	0.895109244
c101.c162	-0.010265328	0.077748662	0.895152526
methioninesulfoxide.c51dc	-0.018761283	0.142097904	0.895153735

kynurenine.putrescine	-0.003016088	0.022853859	0.895199417
pcaec343.smc180	-0.001213715	0.009198801	0.895223169
glutamicacid.c10	-0.005843952	0.044316455	0.895281638
pcaec405.pcaec446	0.006587488	0.049960933	0.895294181
pcaac421.pcaec363	0.002960652	0.022458379	0.895313433
smc240.c142	-0.005805013	0.044050021	0.895349822
c3.pcaec423	0.001901636	0.014430642	0.895353531
pcaac420.pcaec341	0.00320483	0.024324699	0.895373684
smc260.pcaec340	0.006897374	0.052367674	0.895406493
pcaac406.pcaac320	-0.004725905	0.035912615	0.895498044
serotonin.smohc241	-0.003677442	0.02799777	0.895692958
smc161.transhydroxyproline	0.00380359	0.028958753	0.895695011
pcaec421.glutamicacid	-0.010593943	0.080664215	0.895703878
pcaac385.taurine	0.002137852	0.0162788	0.895708979
alanine.pcaec384	0.002830544	0.021553893	0.895711637
pcaec406.pcaec362	-0.002346425	0.0178937	0.89586379
glutamine.asparagine	-0.001694273	0.012922427	0.895879807
lysopcac140.smc181	0.003683412	0.028112123	0.895947144
leucine.pcaac364	-0.003488545	0.026624968	0.8959475
putrescine.methionine	0.003454574	0.026369431	0.895962136
pcaac341.pcaec420	-0.003921819	0.029957576	0.896036619
c51dc.asparticacid	-0.001948873	0.014904	0.896155582
pcaec421.pcaec445	-0.012409467	0.094905235	0.896159819
pcaec401.pcaac384	0.003869235	0.02959882	0.896186513
pcaec386.pcaac366	-0.004483485	0.034353721	0.896354818
lysopcac160.c102	0.004146569	0.031785359	0.896397559
pcaec384.pcaec420	0.002359809	0.018129452	0.896627296
pcaac426.prolinr	0.004759294	0.036595885	0.896717636
smohc161.pcaac281	0.000312239	0.002401023	0.896722107
pcaec403.pcaec421	-0.000931592	0.007165012	0.89674158
pcaec342.ornithine	-0.004446241	0.034217835	0.896805044
c4.pcaac360	-0.003532324	0.027192693	0.896836262
c8.pcaec386	-0.000648756	0.004994564	0.896842066
methioninesulfoxide.pcaac403	-0.001861509	0.014338791	0.896896656
lysopcac140.pcaec421	-0.0006581	0.005072624	0.896965894
pcaec422.smc161	0.012918467	0.099587962	0.896978884
methioninesulfoxide.c8	-0.009262785	0.071420996	0.89699975
pcaec300.smc260	0.002815642	0.021717132	0.897032945
pcaec382.pcaac406	0.010344125	0.079803052	0.897056666
lysopcac280.alanine	-0.001808235	0.013956584	0.897103436
pcaec380.pcaec403	-0.004939936	0.038164119	0.897199907
lysopcac281.pcaac360	0.005093846	0.03943339	0.89740786
lysopcac203.pcaec406	-0.002355662	0.018236513	0.897410344
h1.prolinr	0.004414246	0.034182677	0.897438699
c18.threonine	0.003657569	0.028341244	0.897503695

pcaec385.smc202	0.007877979	0.061060412	0.897531501
c14.c4	-0.002418797	0.018747592	0.897531754
c5.pcaec405	0.007713763	0.059812047	0.897573127
c182.h1	0.00014694	0.001139955	0.89762602
pcaac300.pcaec363	-0.003284488	0.025498767	0.897697147
pcaec425.pcaec382	0.002303758	0.017896043	0.897760084
pcaec423.pcaac426	-0.009238273	0.071766171	0.897762133
smc161.c162	-0.009366332	0.072820823	0.897845682
pcaac424.taurine	0.007099905	0.055206928	0.89785855
glutamicacid.serotonin	0.013184061	0.102551169	0.89789372
pcaac406.transhydroxyproline	0.003238214	0.025193466	0.897914999
ornithine.pcaac364	0.004548894	0.035400304	0.897942745
pcaec385.pcaac383	-0.000546059	0.004250442	0.89796464
spermidine.lysopcac281	-0.005567256	0.043355881	0.898014146
pcaec360.smc260	-0.002676789	0.020849826	0.898033179
histidine.pcaac385	0.001197087	0.009325204	0.898043517
pcaac406.pcaac404	0.001224222	0.009540722	0.898087518
glycine.pcaec425	-0.00057549	0.004486269	0.898117077
pcaec343.histidine	0.006316968	0.04927582	0.898181784
pcaac426.pcaec403	0.002752438	0.021475991	0.898207546
smc261.c18	0.003192263	0.024928368	0.898291294
glycine.pcaac300	-0.004532503	0.035403511	0.898317625
lysopcac204.pcaec403	0.004571768	0.035713003	0.898325544
pcaec423.pcaec446	-0.004353723	0.034039863	0.898415098
pcaac406.pcaec405	-0.006458426	0.050507013	0.898437889
pcaac384.pcaac420	-0.006410839	0.050172229	0.898513094
pcaac360.pcaec423	-0.000445474	0.003487018	0.898532547
pcaac342.pcaac405	-0.004580951	0.03588523	0.898608709
pcaec404.pcaac365	0.005862489	0.045928837	0.898618769
pcaec445.pcaac383	0.000785818	0.006165003	0.898759737
lysopcac140.pcaac422	-0.00817062	0.064109385	0.898772624
c8.asparticacid	0.002810219	0.022066766	0.898849523
c181.c182	-0.007153031	0.056170176	0.898853507
pcaac344.c8	-0.003551367	0.027901471	0.898903489
c141oh.pcaac361	-0.005180871	0.040708114	0.898914294
pcaac281.pcaec422	0.002974085	0.023394013	0.899023782
c12.pcaac363	0.001124767	0.008849988	0.899053524
pcaec361.c2	-0.008034392	0.063232383	0.899078145
pcaac420.c182	0.00369115	0.029056605	0.899100488
pcaac426.c141oh	0.011399871	0.089795982	0.899163727
pcaec342.isoleucine	0.0026724	0.021054203	0.899182283
serotonin.pcaac424	-0.003301837	0.026024794	0.899227135
pcaec384.pcaec421	0.000103297	0.00081496	0.899323656
alphaaminoadipicacid.methionine	0.003412463	0.026927709	0.899342425
pcaec321.pcaac383	0.000482328	0.003807532	0.899381615

pcaac403.pcaec341	-0.004124453	0.032561091	0.899388782
asparagine.lysopcac203	-0.00579247	0.045768197	0.899473401
pcaac426.pcaec362	0.001428128	0.011287457	0.899503119
pcaec363.pcaac360	0.004404219	0.034839169	0.899588198
lysopcac140.pcaac363	-0.001411556	0.011166452	0.899592347
citruline.smc240	0.003803458	0.030136807	0.899753579
pcaac405.smc160	-0.009597402	0.076054159	0.899765206
smohc161.smohc241	0.015926142	0.126221579	0.899777542
alphaaminoadipicacid.pcaac385	-0.003344385	0.02651325	0.899805922
pcaac406.pcaec406	0.006738486	0.053451758	0.899863952
c182.acetylornithoine	-0.001760543	0.013969203	0.899892717
methionine.spermidine	0.006656139	0.052820753	0.899905816
smohc222.pcaac383	0.000513003	0.004071734	0.899923619
pcaec364.smc260	0.003020388	0.023984223	0.899970137
pcaac320.pcaec301	-0.006570194	0.0522145	0.9000503
c16.c51	-0.005504138	0.043764973	0.900101671
pcaec422.serotonin	-0.010659045	0.084779426	0.900132507
alphaaminoadipicacid.pcaac365	-0.003021837	0.024052769	0.900206124
lysopcac203.pcaec382	-0.000903331	0.007191277	0.900220946
pcaac381.serotonin	-0.007361393	0.058634348	0.90027421
pcaec362.lysopcac203	-0.002633923	0.020985883	0.900304374
acetylornithoine.pcaec384	-0.003699381	0.029488834	0.900351031
serotonin.pcaec446	0.006501582	0.051849848	0.900396638
methionine.pcaac321	0.002491176	0.019869059	0.900406788
lysopcac180.pcaec322	0.006867728	0.054854018	0.90054868
c142.pcaac361	0.002786007	0.022262478	0.900593288
pcaac365.pcaac426	0.007488197	0.059921653	0.900733284
glutamicacid.pcaec340	0.006787334	0.054330861	0.900765305
putrescine.pcaec420	0.003469228	0.027778893	0.900795914
smohc141.pcaec383	-0.005617714	0.044982884	0.900797188
pcaac300.smc261	0.005936218	0.047536334	0.90080359
pcaac281.pcaac323	0.009530321	0.076350485	0.900846392
pcaec321.c142	-0.004878284	0.03914664	0.901010499
c51.glutamine	-0.001692922	0.013585359	0.901012029
c5.pcaec423	0.003168148	0.025435202	0.901056375
lysopcac182.c141	0.002383514	0.019141741	0.901086711
pcaac321.pcaec404	-0.003237779	0.026002909	0.901089214
c51.lysopcac140	0.000195028	0.001567989	0.901196085
asparagine.pcaec404	0.00211635	0.017021318	0.901232095
c101.glutamicacid	0.004471204	0.035975247	0.901271342
pcaac362.pcaac361	-0.009261337	0.074553928	0.901320529
lysopcac203.phenylalanine	-0.005891019	0.047434439	0.901344595
smohc241.glutamine	0.001252228	0.010085526	0.901369837
ornithine.lysopcac281	-0.001039081	0.008375007	0.901442273
methionine.pcaec384	0.002544285	0.020527769	0.901541624

pcaac361.pcaec445	0.004811263	0.038826351	0.901562246
leucine.asparticacid	-0.002236586	0.018070663	0.90167968
pcaec386.pcaac342	-0.002814946	0.022744544	0.901683874
serine.pcaec446	-0.003597932	0.029103458	0.901792913
pcaac424.smc180	0.000438528	0.00354741	0.901798031
pcaac424.c141	0.00535757	0.043346682	0.901814591
pcaec424.pcaac404	-0.000875462	0.007084264	0.90183018
pcaec403.c14	0.006817018	0.055217295	0.901925358
pcaec420.phenylalanine	0.002809181	0.022760618	0.90195312
pcaac383.lysopcac170	-0.001052658	0.008535539	0.902029325
pcaac322.taurine	0.002045705	0.016598034	0.902089905
pcaec301.pcaac383	-0.000607178	0.00492836	0.902128693
c2.c3	0.002112238	0.017144782	0.902129256
pcaec361.pcaac406	0.003886806	0.031557516	0.902156324
creatinine.h1	-9.15E-05	0.000743284	0.90222151
pcaec444.pcaec401	0.004251274	0.034548193	0.902245122
smohc222.pcaac321	-0.00257728	0.020946615	0.902255413
pcaec384.lysopcac182	-0.00434767	0.035349754	0.902295173
lysopcac161.transhydroxyproline	0.007000922	0.05693143	0.902310183
pcaec402.pcaec341	-0.004434008	0.036116352	0.902469078
pcaec361.smc160	0.011247401	0.091712106	0.902573382
isoleucine.pcaec385	-0.005758676	0.046957485	0.902575105
methioninesulfoxide.c12	0.012021431	0.098053074	0.902602519
putrescine.smc160	0.00522089	0.042612837	0.902667404
pcaac422.serine	-0.009392709	0.076703846	0.902718752
smohc222.pcaec444	0.010859939	0.088724946	0.902761556
pcaec425.pcaac383	0.000517209	0.00422721	0.902799289
acetylornithoine.pcaec362	-0.004712329	0.038516471	0.902804446
pcaec405.ornithine	0.005265408	0.043043025	0.902817774
pcaac386.smc180	-0.002619275	0.021416378	0.902838743
pcaac281.acetylornithoine	-0.003750758	0.030689257	0.902906032
ornithine.pcaec301	0.009920242	0.081201294	0.902944575
pcaec365.lysopcac280	0.000822678	0.006736419	0.902979819
c101.pcaac320	-0.005058152	0.041438531	0.903027217
pcaac364.c51	-0.00344047	0.028197429	0.903067047
pcaec363.phenylalanine	0.004168191	0.034167272	0.903082793
smohc141.c16	-0.006427032	0.052683707	0.90308349
pcaac344.pcaec420	0.003440267	0.02822032	0.903150964
smc160.c4	-0.001949488	0.015994308	0.903167641
isoleucine.c51	-0.003209358	0.026395502	0.903404034
acetylornithoine.glycine	0.003504956	0.028828969	0.903411722
pcaac300.transhydroxyproline	0.00352355	0.029000303	0.903472682
pcaec445.pcaec423	0.001639218	0.013498289	0.903521293
pcaac300.c10	0.000312485	0.002577456	0.903680252
pcaac364.lysopcac203	-0.001265114	0.010435694	0.903686912

smc160.pcaec362	0.001184582	0.00977165	0.903689334
pcaac322.methioninesulfoxide	0.002915719	0.024055247	0.903702863
lysopcac170.asymmetricdimethylarg	0.001673306	0.013808849	0.903728888
pcaec364.pcaec403	-0.004510985	0.037230755	0.903739561
phenylalanine.asparticacid	0.004779671	0.039451953	0.90374839
pcaec342.threonine	0.004028782	0.033258092	0.90376009
pcaec365.pcaac381	0.003657882	0.030222288	0.90384254
creatinine.pcaac366	0.004580405	0.037872713	0.903914099
pcaec383.pcaac384	0.003804304	0.031457364	0.903919513
lysine.pcaac300	-0.003683012	0.030474883	0.903983724
pcaec340.pcaec363	0.006236394	0.051641783	0.904056044
lysopcac160.smc161	-0.008739356	0.072391426	0.904086818
pcaac405.pcaac300	0.00968371	0.080249295	0.904128949
c181.c10	-0.001071242	0.008886628	0.904227747
pcaec381.pcaac404	-0.002262878	0.018808124	0.904410815
c162.lysopcac170	-0.002012304	0.016725553	0.904411338
lysopcac140.methioninesulfoxide	0.00187494	0.015597061	0.904492008
pcaac343.smc180	0.000598978	0.004985245	0.904540216
creatinine.pcaac361	-0.004755117	0.039598546	0.904593234
pcaac360.c182	0.003209181	0.026733896	0.904626013
asymmetricdimethylarginine.ornithin	0.001504693	0.012538073	0.904651137
pcaac363.lysine	-0.006023546	0.050246682	0.904754238
pcaac404.pcaac300	0.007350012	0.06131803	0.904764058
pcaec446.pcaac421	-0.007815379	0.065202752	0.90476749
c16.pcaac320	0.005329568	0.044475173	0.90479144
pcaac421.pcaac320	-0.004292436	0.035821771	0.904795272
pcaac343.pcaec423	0.000670183	0.00559544	0.904838338
pcaac420.pcaec424	-0.003050036	0.025492022	0.904938305
pcaec403.pcaec443	0.020087873	0.167934597	0.904961595
lysopcac170.c51dc	-0.012301973	0.102848683	0.904965458
pcaac381.pcaec341	-0.003453423	0.028874522	0.904974405
pcaac344.smc180	0.001113103	0.009306927	0.90497573
c51.smc261	-0.002862165	0.023967929	0.905120363
pcaec343.pcaec402	0.004423687	0.037052178	0.905140695
pcaec382.pcaac383	0.00177471	0.014872824	0.905192111
c141oh.pcaac360	-0.002111852	0.017698825	0.905195369
c142.asymmetricdimethylarginine	0.000946738	0.007938882	0.905249319
pcaec343.pcaec420	0.002346038	0.019681607	0.905291882
pcaec362.pcaec301	0.0067669	0.056772386	0.905296642
lysopcac280.pcaec403	-0.00172916	0.014518911	0.905372899
pcaec383.serotonin	-0.010972442	0.092167777	0.905411263
c0.threonine	-0.004398724	0.036978714	0.905486938
pcaac422.pcaac360	-0.004057062	0.034117775	0.90551811
lysopcac140.pcaec420	0.003391136	0.028531923	0.90556504
smc160.pcaac406	0.003890725	0.03276374	0.905646621

smohc222.c3	-0.001775246	0.01495734	0.905696947
smohc161.pcaec384	0.0077707	0.065492301	0.905725977
pcaec420.pcaec444	0.010357889	0.087299294	0.905728006
smc180.c3	-0.001611444	0.013585254	0.90575245
pcaac363.pcaec385	-0.005397908	0.045515884	0.905770785
methionine.c10	0.00060407	0.005098519	0.905861237
pcaec383.pcaec402	-0.008977393	0.075787066	0.90588028
pcaac386.pcaec423	-0.000333835	0.002819677	0.90592829
pcaec424.pcaec322	0.004837965	0.040897388	0.906007028
pcaac384.pcaac405	-0.007182855	0.060751522	0.906055949
pcaec380.pcaec382	-0.00302717	0.025621312	0.906121503
c142.methionine	-0.002401324	0.020332214	0.906157903
histidine.pcaec384	0.001909888	0.016176965	0.906191297
pcaec423.pcaec341	0.003200644	0.027111442	0.906196909
lysopcac160.lysopcac161	-0.008754051	0.074250064	0.906319909
pcaec445.pcaec362	-0.001588517	0.01348431	0.906394826
pcaec443.glutamicacid	0.007857141	0.066721761	0.906430457
smc180.pcaac300	-0.004397477	0.037343918	0.906433322
alphaaminoadipicacid.c141oh	-0.010493816	0.089133016	0.906452377
pcaec382.c142	-0.002792298	0.023754255	0.906596877
pcaec384.pcaac364	-0.004986117	0.042460491	0.906691632
pcaec361.pcaec420	0.002240244	0.019095123	0.906778143
smc241.c142	0.003654185	0.031154908	0.906801367
pcaac281.pcaec444	-0.012594679	0.107604335	0.90699485
pcaec423.pcaec402	0.004486864	0.0383446	0.90702014
h1.pcaac240	-0.001762759	0.015066098	0.907030039
c12.pcaac380	-0.004361152	0.037284332	0.907055043
h1.c102	0.000934759	0.007992643	0.907069017
lysopcac281.pcaac405	0.00768181	0.065716111	0.907115353
pcaec443.threonine	-0.002289659	0.019592182	0.907137408
pcaac424.histidine	0.009405989	0.080496821	0.907150683
pcaac401.smc202	-0.008382457	0.071745626	0.907161281
smc160.pcaec401	-0.003640637	0.031165922	0.907177996
c10.pcaac364	0.004921334	0.042131678	0.907182944
c162.pcaac361	0.003809404	0.032617853	0.907198352
smohc241.pcaec301	0.005723147	0.049005034	0.907199932
c141.serine	0.004120422	0.035293572	0.907231442
pcaec300.smohc241	0.006738289	0.057775617	0.907325175
pcaec321.smc240	0.002589	0.022213249	0.907385704
pcaac322.ornithine	0.003317169	0.028462984	0.907392674
pcaac342.pcaec365	-0.002602025	0.022342933	0.907459699
creatinine.c141	-0.002097729	0.018014019	0.907466583
arginine.smohc141	0.002414023	0.020745325	0.90753393
pcaec360.c142	0.004701172	0.040405709	0.907546167
methioninesulfoxide.pcaec382	-0.002367394	0.02037164	0.907656061

isoleucine.pcaac362	-0.000928993	0.007997571	0.907696333
pcaec322.lysopcac140	-0.000127833	0.001101048	0.90774259
pcaac365.pcaac360	-0.004076617	0.03511684	0.907753389
pcaac365.pcaec301	-0.008324531	0.071760531	0.907818993
citruline.pcaec382	-0.000862481	0.007445984	0.907955483
pcaec361.pcaac320	0.011356352	0.098107532	0.908016903
pcaec360.phenylalanine	0.002937802	0.025391517	0.908059629
c3.pcaac402	-0.005932155	0.051272573	0.90806098
pcaec404.smc261	0.006888781	0.059576747	0.908116139
smc160.asparagine	-0.000777607	0.006729476	0.908176445
pcaec424.smohc241	0.006247079	0.054143441	0.908312753
leucine.pcaac320	-0.011295313	0.097917382	0.908332221
c162.pcaac366	-0.00652245	0.056558594	0.908358757
pcaec425.threonine	0.002134231	0.018513186	0.908390685
isoleucine.c16	-0.005159042	0.04476023	0.908408194
pcaac425.c142	0.003725294	0.032333251	0.908442957
pcaac322.c10	0.000771448	0.006699947	0.908500851
lysopcac170.pcaec365	-0.002712201	0.023557024	0.908507952
leucine.pcaec446	-0.004174558	0.036265994	0.908526921
putrescine.lysine	-0.004936318	0.042918231	0.908600193
lysopcac140.pcaec402	0.006840417	0.059520813	0.908673007
pcaac403.smohc241	-0.006419408	0.05586711	0.908688687
pcaac342.pcaec362	0.001723213	0.014997668	0.908693618
pcaac424.methionine	-0.002816938	0.024543969	0.908794596
smc240.c12	-0.004327464	0.037806469	0.909037901
pcaac401.lysopcac180	-0.006274834	0.05483077	0.909056549
smc161.pcaec383	-0.007730799	0.067554133	0.909057674
lysopcac160.isoleucine	-0.002905127	0.025395553	0.90909209
pcaec322.lysopcac281	-0.004282139	0.037436899	0.909101774
pcaec364.pcaec424	0.002053174	0.017956921	0.909136558
pcaec341.lysopcac281	0.003012451	0.02636321	0.909193266
pcaac380.pcaec380	0.005826456	0.050993853	0.909200552
pcaec383.pcaac361	-0.004814019	0.042136177	0.909207618
pcaec381.pcaac424	0.005118561	0.044814213	0.909232706
pcaac380.c12	0.004594867	0.040249943	0.909279394
pcaac362.pcaec362	0.001708249	0.014964851	0.909285421
pcaac384.phenylalanine	0.004224263	0.037018607	0.90931617
pcaec406.pcaec343	-0.008969226	0.078752659	0.909490869
pcaec342.pcaac320	0.008049773	0.070696598	0.909512569
pcaac320.pcaac364	-0.007454369	0.065478259	0.909527372
lysopcac181.pcaac363	0.001489175	0.013080754	0.909527544
c16.pcaac300	-0.004383016	0.038528706	0.909594781
pcaec403.methioninesulfoxide	0.002058239	0.018109759	0.909678807
lysopcac160.glutamicacid	-0.003616723	0.031852793	0.909764774
smc260.smohc141	-0.001207532	0.010648833	0.909882868

pcaac385.pcaec420	-0.002651708	0.023387549	0.909894325
lysopcac280.pcaec384	0.005876188	0.051891614	0.91000634
smc261.c102	-0.002110606	0.018645519	0.910040557
c3.pcaac426	-0.003025199	0.026729431	0.91005466
smc180.pcaec362	0.003522563	0.03112494	0.91005755
pcaec423.lysopcac204	-0.003540005	0.031348433	0.910255741
pcaac380.c162	-0.005494162	0.048655312	0.910259211
pcaec341.pcaac281	0.000398859	0.003532808	0.910273997
pcaac321.smc202	-0.003560863	0.031542774	0.910283039
pcaec301.smc181	0.002704703	0.023962347	0.910296422
smc180.c18	-0.003093558	0.027427219	0.91036093
pcaac402.asparagine	-0.001977871	0.017542303	0.910394873
pcaac342.pcaec340	-0.005995221	0.05320291	0.910444478
smc160.isoleucine	0.001950327	0.017314744	0.910481128
pcaec404.c141	-0.003647041	0.032384963	0.910500429
pcaac420.kynurenine	0.001552797	0.013809121	0.910633405
pcaac320.pcaec420	0.00254473	0.022640402	0.91067257
c142.pcaec360	-0.000719902	0.006409306	0.910732953
serotonin.c12	0.004426103	0.039423414	0.910772937
c101.pcaec340	0.003081798	0.027450143	0.910774519
pcaac360.histidine	-0.006061751	0.053993405	0.910774951
alanine.c0	0.000189444	0.001688977	0.91085698
pcaec322.pcaec380	-0.004594577	0.041002214	0.910942379
c0.c51dc	-0.011123722	0.099367303	0.911030477
pcaec381.lysopcac280	-0.001039872	0.009306624	0.911197378
pcaec421.c101	-0.001326871	0.011876166	0.911204533
methioninesulfoxide.pcaac383	0.001485471	0.013296771	0.911211545
pcaec422.pcaac363	-0.002627684	0.0235298	0.911244755
pcaac401.c142	-0.003081354	0.02764353	0.911408821
lysopcac203.pcaec384	-0.002416834	0.021690947	0.911445337
smc180.pcaac386	0.001042561	0.009357834	0.911453867
pcaec341.smohc141	-0.003402187	0.030555801	0.911506997
c101.c182	0.004468734	0.040161346	0.911565466
pcaac322.pcaec343	0.011073239	0.099525863	0.911573075
c5.c16	0.005711631	0.051390957	0.911667365
pcaec382.c141	-0.004048857	0.036430263	0.91166805
alanine.c18	0.004952187	0.044580656	0.911712492
pcaac422.c142	0.004493287	0.040460018	0.911735268
arginine.c10	0.000950311	0.008564317	0.911809134
pcaec343.c142	0.002986243	0.026921057	0.911837441
pcaec342.pcaac323	-0.00819993	0.073974915	0.911899547
pcaac360.pcaac321	-0.003981807	0.035957334	0.911986984
lysopcac182.pcaac364	0.004247707	0.038370882	0.912015205
pcaac402.pcaac360	-0.006295984	0.056886444	0.912034976
pcaec382.c2	-0.011853934	0.107238474	0.912144423

pcaac404.pcaac360	0.00275632	0.024940028	0.91216038
pcaec381.c12	0.006986157	0.063252662	0.91221538
pcaec444.pcaec301	0.004702437	0.042581545	0.912227047
pcaec321.asparticacid	-0.003866011	0.035060467	0.912359023
pcaac323.pcaec402	-0.002650776	0.024070634	0.91247147
pcaec446.pcaec365	0.002536503	0.023037741	0.912489545
pcaec445.methioninesulfoxide	-0.001383588	0.012567565	0.912497524
pcaac366.pcaac320	-0.007564768	0.068714521	0.912499261
pcaac406.creatinine	-0.002823484	0.025658288	0.912537256
pcaac402.smohc241	-0.00680134	0.061824301	0.912561803
pcaec404.lysine	-0.008641275	0.078561872	0.912575691
pcaac281.pcaec423	0.000573721	0.005216239	0.912580305
smohc221.glycine	-0.005238011	0.047623858	0.912580542
c142.glutamine	0.003518058	0.031990109	0.912591488
acetylornithoine.c14	-0.00083317	0.007579639	0.912631961
leucine.pcaac362	-0.001129507	0.010286811	0.912727466
pcaec383.pcaec420	-0.003096688	0.028220566	0.912782804
lysine.c51	-0.003608527	0.032887184	0.912788502
pcaec343.phenylalanine	0.003606724	0.032872249	0.91279245
c2.c51	-0.007401834	0.067464389	0.912796205
lysopcac280.pcaec423	-0.000368225	0.003359517	0.912881796
c101.pcaac404	-0.000291478	0.002663263	0.913010566
pcaec364.pcaac360	-0.003315758	0.030303908	0.913031988
serine.c10	0.000452966	0.004140342	0.913042923
arginine.pcaec401	0.007695588	0.070388969	0.913101157
c8.c181	0.003379975	0.030936304	0.913159354
pcaec404.pcaac383	0.000559266	0.005121123	0.91319755
pcaec421.c182	0.003199775	0.029313166	0.913236663
pcaac404.creatinine	-0.002264355	0.020775073	0.913366848
lysine.pcaac364	0.00356421	0.032708103	0.913385542
pcaac406.pcaac365	-0.005466014	0.050186586	0.913430185
prolinr.pcaec365	0.001664309	0.015282589	0.913439351
c8.putrescine	-0.003223219	0.029615327	0.913491673
glycine.pcaac320	-0.001173049	0.010784876	0.913545691
pcaec381.methioninesulfoxide	-0.002167549	0.019932931	0.913566158
pcaac384.lysopcac182	0.002917417	0.026840721	0.913604499
pcaec322.smc202	0.006284205	0.057878355	0.913697602
c5.pcaec363	-0.002145971	0.019780173	0.913764944
lysopcac280.pcaec420	-0.00263819	0.024318386	0.913769381
pcaac240.c142	0.003380112	0.03116145	0.91378084
pcaac404.pcaac343	0.003099774	0.028615427	0.913896164
c16.c142	0.007987987	0.073801658	0.913966943
pcaac322.c18	0.003911286	0.036193707	0.914101946
histidine.c142	-0.003068813	0.028418588	0.914164701
pcaac384.pcaec420	0.001764408	0.016343468	0.914186951

pcaac322.pcaac362	-0.002990213	0.027730095	0.914286122
pcaac421.histidine	0.007842389	0.072728037	0.914286943
lysopcac180.pcaec365	-0.00241872	0.022479411	0.914472699
pcaec404.pcaac363	-0.001680057	0.015623812	0.914524384
pcaac362.valine	-0.00306435	0.028526543	0.914612137
c141oh.c12	0.005221843	0.048645244	0.914672018
pcaac363.smohc241	-0.005495701	0.05119974	0.914677503
pcaac361.pcaac383	0.000610967	0.005692067	0.914679023
lysopcac281.pcaec421	0.000304626	0.002838395	0.91468948
lysopcac181.lysopcac204	0.004661428	0.043436846	0.914696024
pcaec421.c141	-0.005616311	0.052350298	0.914721207
c14.c16	0.004081215	0.038079497	0.914806005
pcaec425.lysine	-0.009469228	0.088457414	0.914907153
pcaec343.alphaaminoadipicacid	0.00092054	0.008600254	0.914916753
alphaaminoadipicacid.pcaac361	-0.004653761	0.043492926	0.914945184
smc160.pcaac361	0.005582496	0.052220653	0.915023051
pcaec421.alanine	-0.001523041	0.014249588	0.915038018
lysopcac182.asymmetricdimethylarg	0.001832272	0.017193774	0.915289189
asymmetricdimethylarginine.pcaac34	0.002356653	0.022130377	0.915349765
c102.pcaec384	-0.003024656	0.028408061	0.91536382
pcaac322.pcaec423	0.000428282	0.004026815	0.915454342
pcaec424.pcaec403	-0.004918686	0.046332805	0.915610767
pcaec385.pcaac360	0.003915946	0.036941933	0.915735235
pcaec384.c162	-0.007615926	0.071938512	0.915842601
pcaec360.c14	-0.006862971	0.06489326	0.915929178
pcaec382.serine	-0.009315256	0.088197055	0.916039391
pcaec342.c142	0.00335815	0.031812961	0.916086503
smohc241.tyrosine	0.006741531	0.063877762	0.916103294
smc180.pcaac403	0.001048101	0.009954034	0.916296424
pcaec406.pcaec421	-0.000175522	0.001667012	0.916298755
pcaac405.smc260	-0.00207812	0.01976179	0.916403873
lysopcac181.pcaac361	-0.006520692	0.062057451	0.916469907
c51.pcaec380	-0.003231165	0.030757766	0.916488187
alphaaminoadipicacid.asparagine	0.000807697	0.00768964	0.916500089
h1.c18	-0.003138165	0.029916624	0.916610948
pcaec422.pcaac364	-0.00443274	0.042262262	0.916619295
methionine.asparagine	-0.00088845	0.00847076	0.916621035
smc161.pcaec403	-0.003110736	0.029667303	0.916645014
smc181.alanine	-0.001725412	0.016462187	0.916679369
pcaec342.asymmetricdimethylarginir	0.004413474	0.042197872	0.916854137
pcaac425.glutamicacid	0.005352026	0.05117664	0.916862448
c14.c141oh	-0.015087528	0.144324303	0.916894456
pcaec424.serotonin	0.008842904	0.084591648	0.916896589
glycine.pcaec386	0.001084541	0.010382626	0.91695925
pcaec341.smc240	0.002007468	0.019231435	0.917016722

pcaac403.c142	-0.003030044	0.029038437	0.917047414
pcaec362.tyrosine	0.005168927	0.0495404	0.917054022
pcaac402.smohc141	0.002631677	0.025231932	0.917084237
lysopcac161.putrescine	0.007028667	0.06738961	0.917084629
creatinine.c142	-0.004453224	0.042727567	0.917144271
isoleucine.pcaec365	0.002578934	0.024750465	0.91716508
pcaac363.c141oh	0.009702781	0.093193076	0.917230487
asparagine.pcaec405	-0.003995941	0.038394023	0.917260319
pcaec443.c182	-0.003892619	0.037404201	0.917266761
pcaec383.pcaec446	0.004531372	0.043651615	0.917473794
lysopcac161.pcaec386	0.000814978	0.007852344	0.917489504
c51dc.asymmetricdimethylarginine	0.002011248	0.019382432	0.917506377
putrescine.smc260	0.001674197	0.016141719	0.91754433
lysopcac160.asymmetricdimethylarg	0.002042768	0.019697343	0.917552906
lysopcac160.smohc222	-0.005009904	0.048323972	0.917580254
smohc221.pcaec360	0.000631592	0.006104901	0.917751946
pcaec301.taurine	0.002263371	0.02189184	0.917805582
c5.pcaec362	-0.001393557	0.0134804	0.917815305
pcaec404.smohc221	-0.009524147	0.092138749	0.917822512
methioninesulfoxide.c182	-0.006513108	0.063106936	0.917949229
histidine.pcaec365	-0.00224815	0.021791938	0.917983427
pcaec422.threonine	0.001992419	0.019320842	0.918016336
smc161.pcaec401	-0.004888734	0.0474285	0.918053485
pcaac240.c182	-0.003492048	0.033907582	0.918123704
pcaac361.pcaac421	-0.003550767	0.034481192	0.918131877
c51.methioninesulfoxide	0.001296753	0.012592957	0.918133806
pcaac402.taurine	-0.006323097	0.061430059	0.918167733
pcaac406.c141oh	0.006661911	0.064737127	0.918187167
c12.pcaac401	-0.00024501	0.002383124	0.918263726
pcaac322.pcaec384	-0.004832591	0.047056658	0.918353391
creatinine.pcaec365	-0.002754272	0.026826297	0.918374533
c18.pcaac300	-0.004315095	0.042058961	0.91843337
pcaac406.pcaac422	-0.003475433	0.033960864	0.918639291
smc202.pcaec402	-0.004558439	0.044579194	0.918703904
smc160.pcaac424	-0.000796425	0.007788855	0.918706247
glycine.pcaac365	-0.006450169	0.063097015	0.91872659
c181.c12	0.006241404	0.061077149	0.918756197
lysine.c142	-0.004974516	0.048772053	0.918909609
h1.c8	0.001441546	0.014164039	0.919084006
serotonin.pcaac360	0.002036052	0.020012754	0.919113632
pcaec445.acetylornithoine	-0.00600394	0.059045479	0.919156718
smc161.pcaec423	0.000706382	0.006947073	0.919158919
c2.pcaec383	-0.009281499	0.09135684	0.919225814
lysine.pcaec423	0.000757946	0.007466904	0.919296084
c10.pcaac320	0.004339484	0.042759266	0.919312736

pcaec380.pcaec322	-0.007531529	0.07432413	0.919433826
glycine.pcaac361	0.002252783	0.022234706	0.919445903
pcaec403.kynurenine	0.000796215	0.007865505	0.919516855
pcaac384.pcaec421	7.88E-05	0.000778289	0.919526109
pcaac425.pcaac320	-0.010254491	0.101325976	0.919537293
c0.c16	0.004333844	0.04282987	0.919549618
c162.pcaac424	-0.000608544	0.006019	0.919615827
lysopcac281.smohc241	-0.005710241	0.056486048	0.919625881
pcaac300.pcaac364	0.005677779	0.056180804	0.919648503
lysopcac204.c182	0.002298994	0.0227512	0.919659009
pcaac421.lysine	-0.007339628	0.072657029	0.919684295
smc241.pcaec362	0.001217082	0.012051217	0.919704078
smohc161.pcaac426	0.003873674	0.038372535	0.919738439
pcaac420.smohc241	-0.004855274	0.048118336	0.919775157
pcaac424.pcaec421	-0.002756504	0.02733232	0.919815835
prolinr.methionine	-0.001676961	0.016645083	0.919897687
asparticacid.histidine	0.005341862	0.053070553	0.919970816
pcaec420.kynurenine	-0.000953222	0.009472653	0.91999225
pcaac342.c142	-0.001972873	0.019605647	0.91999305
pcaac300.pcaec340	-0.007442677	0.074021919	0.92005712
pcaac384.pcaec423	0.000730769	0.007269207	0.920071034
pcaac383.pcaec385	0.004769526	0.04744639	0.920074866
lysopcac281.pcaac343	-0.002811122	0.027984953	0.92013297
pcaac320.asymmetricdimethylarginir	0.004275737	0.042586825	0.92017317
c5.valine	-0.009252813	0.092322137	0.920313702
phenylalanine.serotonin	0.006849455	0.06836774	0.920343533
pcaac281.glutamicacid	-0.003120922	0.031157888	0.920359956
c12.c16	0.002706057	0.027032694	0.920408791
c102.pcaac344	-0.003721314	0.03718864	0.920438316
c3.pcaec363	-0.001383631	0.013833638	0.920475226
c182.lysopcac280	-0.00134407	0.013440559	0.920489679
pcaec403.pcaac320	-0.007415974	0.074206973	0.920540954
pcaec424.lysopcac140	0.000138321	0.001384462	0.920562375
pcaac422.pcaec322	0.005597395	0.056136334	0.920719746
smc160.lysopcac140	-7.16E-05	0.000717714	0.920734237
lysopcac161.c10	0.000906151	0.009115764	0.920962124
pcaac420.pcaac426	0.008843991	0.089046104	0.921029989
smc260.c142	0.004061454	0.04091804	0.921078299
alphaaminoadipicacid.pcaec420	-0.002109972	0.021262207	0.921096127
asparagine.pcaac406	0.001578995	0.015945282	0.921262486
pcaec301.pcaac401	0.000833961	0.008439934	0.921432468
pcaac342.pcaec386	0.000968316	0.009805953	0.921482846
c141.smc160	-0.006370601	0.064552348	0.92152952
smc260.c12	-0.00393002	0.039824184	0.921533207
lysopcac204.pcaac401	-0.000235029	0.002381812	0.921539297

pcaac424.pcaec365	0.002266796	0.023010145	0.921669024
putrescine.pcaec421	-0.000431579	0.004383073	0.921707028
smc180.c51dc	-0.009351986	0.094982766	0.92171109
lysopcac180.pcaac323	-0.005822	0.059196428	0.921797712
pcaac323.c141oh	-0.00545985	0.055522799	0.921809798
pcaac405.c3	0.003777826	0.038428844	0.921832179
ornithine.h1	-0.00019419	0.001977558	0.921919453
lysopcac204.pcaec384	-0.003666678	0.037351572	0.921943448
pcaec421.c3	-0.003778522	0.038501058	0.921963983
pcaac240.pcaec300	-0.004437698	0.045242624	0.922006848
pcaac321.c2	-0.007722595	0.078787173	0.922060921
pcaec382.pcaac344	0.00825611	0.08424937	0.922078611
c16.c14	0.004883729	0.049840823	0.922086213
c12.glutamicacid	0.003690349	0.037664672	0.922092126
pcaac385.lysopcac140	7.87E-05	0.000803234	0.922102587
leucine.h1	-0.000135352	0.001385042	0.922294028
pcaec383.smc160	-0.005447755	0.055750669	0.9223003
c182.c10	-0.000326742	0.003345332	0.92233621
pcaac386.pcaac404	0.000846319	0.008667257	0.922356475
pcaac383.pcaac344	-0.005301927	0.054344097	0.922422575
pcaec380.pcaec446	-0.004624834	0.047406863	0.922427269
serotonin.c182	-0.000943923	0.009677067	0.922438309
asparticacid.pcaec341	0.002764796	0.02835536	0.922467676
pcaac320.smohc241	-0.004476211	0.045919997	0.922488891
smc181.pcaac320	0.003739558	0.038377543	0.922518354
prolinr.alphaaminoadipicacid	0.001072489	0.011006633	0.922519202
pcaac381.transhydroxyproline	0.004407482	0.045234864	0.922522957
c141oh.lysopcac170	-0.002134116	0.021913946	0.922562047
pcaac426.pcaec385	-0.00439458	0.045130196	0.922570429
pcaac365.acetylornithoine	-0.008008164	0.082257156	0.922586588
pcaac406.c51	-0.00514806	0.05288821	0.922599827
smc180.smohc141	-0.001810028	0.018598582	0.922613922
pcaec300.pcaac342	-0.001292202	0.013288705	0.922677444
smohc161.c182	-0.001889039	0.01942948	0.922689472
pcaac323.pcaec384	0.001761533	0.018120761	0.922701073
prolinr.c14	-0.002830342	0.029127409	0.922732537
c14.pcaac421	0.001528955	0.015745075	0.922783415
phenylalanine.lysopcac281	-0.000603852	0.006219318	0.922794447
lysopcac180.pcaac422	-0.004922702	0.050710034	0.922808309
pcaac322.c16	-0.003169274	0.032647956	0.922809332
pcaac323.pcaec423	-0.000492553	0.005077438	0.922861723
lysopcac280.smc180	0.000371889	0.003836365	0.922917365
glutamicacid.pcaac341	0.000867029	0.008948537	0.922954911
lysopcac203.c10	0.000420181	0.004336698	0.922955735
lysopcac180.pcaac384	0.006045557	0.062454582	0.92302728

c5.c182	-0.005066415	0.052343686	0.923033557
c8.pcaac320	0.003594066	0.03713222	0.923033798
serine.pcaec342	0.007131754	0.073692921	0.923045199
pcaec343.pcaac363	0.001057909	0.01093837	0.923093703
pcaec446.c182	-0.002669204	0.027599111	0.92309534
pcaac386.asymmetricdimethylarginir	0.001247695	0.012902073	0.92310196
pcaac385.h1	8.94E-05	0.000924448	0.923107817
smc181.pcaac323	0.008570688	0.088667641	0.923137006
phenylalanine.pcaec322	0.005719787	0.059241645	0.923224775
smc260.smc241	-0.008395092	0.08698624	0.923256113
prolinr.smc260	0.001586542	0.016450357	0.923308722
pcaec425.pcaac426	0.006642072	0.068880852	0.923321232
pcaac322.lysopcac140	0.000122116	0.001266575	0.923332145
histidine.c10	0.000196811	0.002041807	0.92335125
lysopcac161.c18	-0.007151189	0.074192993	0.923354622
pcaac426.pcaac401	0.001031311	0.010702758	0.923375957
lysopcac280.serotonin	-0.009345205	0.097011054	0.923398192
pcaac424.methioninesulfoxide	0.001785496	0.018546312	0.923444989
pcaac321.c141oh	0.007524499	0.078188856	0.923474619
pcaec341.asparticacid	0.003012839	0.031314327	0.923492176
putrescine.lysopcac180	-0.005672486	0.058976937	0.923517029
pcaec363.pcaac424	-0.001106559	0.011511352	0.923559685
pcaec321.pcaac403	-0.00059204	0.006160423	0.923578582
pcaec405.pcaec365	0.002900572	0.03019328	0.923607894
h1.pcaec365	-0.003164099	0.032975197	0.923697401
c0.pcaec384	0.002694264	0.028079947	0.923700686
pcaac321.pcaac320	0.003166035	0.03302207	0.923758883
smohc161.pcaac366	0.003263536	0.034040316	0.923761808
arginine.smohc241	0.005203498	0.054345711	0.923860554
pcaec322.isoleucine	0.002201437	0.023009515	0.923918442
c18.arginine	-0.004349115	0.04548278	0.923961204
c182.pcaec385	-0.00342943	0.035906024	0.924048302
pcaac362.pcaec423	-0.000300858	0.00315013	0.924051963
pcaac405.pcaec402	-0.002349582	0.024602147	0.92405475
smc261.pcaec382	0.001522784	0.015953742	0.924096893
pcaac405.pcaac360	0.002475688	0.025946249	0.924123796
pcaec343.asparticacid	0.002647599	0.027815962	0.924308766
pcaac321.h1	-0.000165151	0.001738052	0.924437411
c51.asparagine	0.000521375	0.005489932	0.924478037
pcaec365.pcaac300	-0.004684608	0.049352221	0.924515538
histidine.serotonin	0.004646063	0.049013375	0.924618749
glutamicacid.pcaac365	-0.005686957	0.059995359	0.924620171
c14.pcaac341	-0.001281244	0.013545888	0.924782279
pcaac323.lysopcac170	0.000763274	0.00807477	0.924829622
smohc222.c142	0.002730233	0.028885216	0.924834125

pcaac406.pcaec420	0.005344973	0.056603813	0.924907301
smc202.pcaec420	-0.002429053	0.02572613	0.924913743
pcaac323.pcaac404	-0.001225517	0.012991194	0.924981346
pcaec342.pcaec421	-0.000255411	0.002708127	0.924998491
pcaac342.valine	0.002726119	0.028938181	0.92508402
lysopcac181.smohc141	-0.003833978	0.040739991	0.925160494
pcaac240.pcaec420	0.002311851	0.024603595	0.925275088
pcaac342.pcaec445	-0.004203508	0.0447597	0.925315639
creatinine.lysopcac140	8.78E-05	0.000935109	0.925337635
histidine.isoleucine	-0.001579217	0.016823881	0.925351511
pcaec341.pcaec423	0.000442807	0.004728892	0.925532947
ornithine.smc160	-0.005143395	0.054994523	0.925622666
pcaec321.lysopcac140	-6.20E-05	0.000663631	0.925695483
smc181.c2	-0.004619054	0.04943968	0.92569998
pcaec343.h1	0.000281189	0.003009976	0.925707223
lysine.pcaac360	0.001896011	0.020311852	0.925765819
pcaac343.c142	-0.001383257	0.014839243	0.925868037
lysopcac204.pcaec364	0.001817662	0.019500802	0.925873236
leucine.serine	0.003273285	0.035126411	0.92589203
pcaec421.pcaac383	0.000743512	0.007980543	0.925908166
pcaac401.pcaec401	0.007743954	0.083122758	0.925910278
lysopcac280.phenylalanine	0.003491057	0.037509826	0.925983549
pcaec385.pcaec446	-0.005751814	0.062070624	0.92630457
pcaec341.pcaec422	0.002614822	0.028220514	0.926311602
c18.pcaec365	0.002010992	0.021706194	0.926320144
c14.pcaec363	0.001487139	0.016054358	0.926331655
pcaec420.c12	-0.002478194	0.026760482	0.926351472
c2.lysopcac181	0.006281355	0.067868591	0.926394904
pcaac360.valine	-0.003793957	0.040993692	0.926396446
pcaac342.pcaec401	-0.00298666	0.032273716	0.926402956
pcaac425.pcaec423	-0.00036706	0.003970859	0.926484741
pcaec420.pcaec384	-0.00240459	0.026015006	0.926490738
pcaac366.smc180	0.000583303	0.006316706	0.926560448
h1.lysopcac203	0.006899841	0.074730392	0.926570903
asparticacid.smc180	0.000353072	0.003826096	0.926610324
pcaec384.c101	-0.000966206	0.010478363	0.926666185
pcaec403.lysopcac160	-0.002978525	0.032304696	0.926673024
putrescine.pcaec341	0.003368678	0.036565774	0.926732094
pcaac385.putrescine	-0.003025548	0.03284467	0.92673976
asparticacid.prolinr	0.002890952	0.031420614	0.926825994
citruline.c51	0.002450116	0.026706581	0.927037021
citruline.pcaac424	-0.001488997	0.016238298	0.92707302
pcaac420.pcaac320	-0.004434303	0.048383962	0.927111434
pcaac405.c101	0.000489809	0.005345541	0.927126303
smohc222.alanine	-0.001724159	0.018819567	0.927137578

lysopcac170.pcaac363	-0.001899152	0.020747183	0.927198965
pcaec446.valine	-0.006260832	0.068441344	0.927246935
pcaac342.pcaac281	0.000233186	0.002550446	0.92728489
pcaac385.pcaec421	0.000109664	0.001200939	0.927375412
pcaec443.lysopcac203	0.001172794	0.012871556	0.927534155
c10.pcaec343	0.005930943	0.065165724	0.927615015
pcaac321.pcaac420	0.008585626	0.094361521	0.9276362
acetylornithoine.pcaec446	-0.00134983	0.014843778	0.927676491
pcaec404.c0	-9.58E-05	0.001054412	0.927706631
histidine.leucine	-0.001105401	0.012162816	0.927717778
smohc221.pcaac361	-0.003197235	0.035231651	0.927824625
glycine.pcaec342	0.004380631	0.048272042	0.927824722
ornithine.c162	-0.006151039	0.067791707	0.927836175
alanine.lysopcac160	-0.000878124	0.009686077	0.927896444
c51dc.pcaec362	0.001932321	0.021321549	0.927920855
pcaec406.pcaac422	-0.005373323	0.059316223	0.927952475
pcaec403.pcaec365	-0.001856518	0.020503273	0.927984466
c182.pcaec422	-0.001551907	0.017150954	0.928033816
pcaac342.pcaac384	0.006039818	0.066813055	0.928102367
pcaec401.glutamicacid	-0.004304231	0.047686249	0.928211271
smc261.c3	-0.00157406	0.017443588	0.928230522
pcaec363.smohc241	-0.004135651	0.045832797	0.928233504
lysopcac140.asparticacid	0.003653024	0.040486041	0.928236846
pcaec322.smc160	-0.008212154	0.091093836	0.92829929
pcaac321.pcaac361	-0.006364233	0.070664308	0.928368767
pcaec386.pcaec301	-0.006318533	0.070293096	0.928507193
lysopcac161.pcaac321	0.002326753	0.025908806	0.928572939
c101.pcaac380	-0.003059906	0.034078101	0.928584466
pcaac365.c8	-0.003382208	0.037678996	0.928606053
pcaac364.pcaac365	0.003190945	0.035612538	0.928734579
pcaec405.lysopcac181	0.005753836	0.064296333	0.928823729
pcaec446.smc180	0.001382486	0.015466743	0.928906954
pcaec301.c10	0.002548698	0.028516039	0.928912266
c162.pcaac386	-0.000938556	0.010501943	0.928918554
acetylornithoine.pcaec301	0.003864687	0.04326025	0.928945553
glutamine.c5	-0.000889905	0.009962423	0.928953179
ornithine.c12	0.004118409	0.046158156	0.929034311
citruline.histidine	-0.003662569	0.041055682	0.929045468
alphaaminoadipicacid.pcaac300	0.002631132	0.029506581	0.929076219
pcaec405.c182	0.002318567	0.026011673	0.929104283
pcaac402.lysopcac181	0.0047245	0.053064524	0.929185612
c101.pcaec343	0.005091405	0.057221458	0.929229978
pcaec365.pcaac380	-0.007060306	0.079387021	0.929263229
lysopcac281.pcaec340	0.005855709	0.065912811	0.929338633
putrescine.pcaec340	-0.004190704	0.04718655	0.929361485

serotonin.pcaac385	-0.002909402	0.03281801	0.929487454
pcaec420.pcaac240	-0.000767987	0.008665163	0.929505958
pcaec343.pcaac320	0.006089364	0.068751104	0.929552081
pcaac402.glutamicacid	-0.007751567	0.087571867	0.929595327
pcaec322.pcaac403	0.000556602	0.006289125	0.929606665
pcaac403.asymmetricdimethylarginir	-0.001814325	0.020505334	0.929623881
asparticacid.pcaac401	0.00026738	0.003023423	0.92965919
smc161.pcaec301	0.004080389	0.046140261	0.929660523
pcaec361.lysopcac140	9.28E-05	0.001050952	0.929760166
pcaec405.pcaec420	0.002472772	0.028001787	0.929761112
lysopcac170.smc160	0.003895586	0.04411447	0.929762171
pcaac380.pcaec341	0.002960564	0.033529588	0.929769504
c14.pcaac364	0.002841553	0.032208162	0.929826972
methioninesulfoxide.pcaac422	-0.016368385	0.18554198	0.929831195
pcaac320.smc180	-0.000627547	0.007115005	0.929846104
pcaac385.pcaac344	0.006374239	0.072401657	0.929973477
pcaec402.taurine	0.004623697	0.052528289	0.929986938
pcaac343.pcaec401	0.005531544	0.062863324	0.930010582
pcaac361.pcaec421	0.000233508	0.002654894	0.93004176
c10.glutamine	-0.002461989	0.028008341	0.930082939
pcaec382.glutamicacid	0.008954599	0.101922889	0.93011894
arginine.pcaac385	-0.002482184	0.028289625	0.930209965
pcaac383.pcaec365	0.00208634	0.023800247	0.930274581
lysopcac161.taurine	0.002436255	0.027802141	0.930300058
pcaac363.smohc141	0.002783204	0.031787094	0.930356105
putrescine.c12	0.003107496	0.035506722	0.930387151
c18.isoleucine	0.00118542	0.013545878	0.930392757
c18.pcaac363	-0.000783738	0.008969444	0.930498109
smohc161.pcaac343	0.001474558	0.016879033	0.930512656
pcaec446.phenylalanine	0.003119654	0.035710445	0.930513155
pcaec425.c102	0.001823188	0.020870995	0.93051679
glycine.smc260	0.002200564	0.025203206	0.930550311
c14.putrescine	0.003183556	0.036528022	0.930676515
pcaec445.smc160	0.007057534	0.081043093	0.930732119
pcaac320.c182	0.00228908	0.026331352	0.930851215
serine.pcaec420	-0.002901203	0.03338557	0.930877956
pcaac321.c182	-0.001793363	0.020665787	0.930973646
c181.c16	-0.005247038	0.060472201	0.930982802
asparticacid.ornithine	0.002697611	0.031151807	0.931119359
pcaec364.pcaec402	-0.003150522	0.036411095	0.931174274
asymmetricdimethylarginine.pcaac42	-0.003804745	0.044001848	0.931220766
pcaec385.pcaac344	-0.004443924	0.051430469	0.931269474
smc241.pcaac300	-0.004159014	0.048227081	0.931403023
pcaec360.c16	-0.005223964	0.060610893	0.931442388
pcaac402.transhydroxyproline	-0.006367504	0.073896604	0.931458878

pcaec321.pcaec362	0.001949308	0.022632302	0.931489241
pcaac343.methioninesulfoxide	0.001554465	0.018087895	0.931639974
pcaec405.smc161	0.005747528	0.06688371	0.931645022
citruline.pcaec423	0.001452817	0.016909006	0.931655696
c3.pcaec406	0.00336719	0.039191503	0.931658368
alanine.c10	0.00035859	0.004174665	0.931673929
lysopcac181.pcaec362	0.002266896	0.0264084	0.931718953
smc181.lysopcac161	0.004737001	0.055237154	0.93178438
pcaac364.pcaec425	0.00062337	0.007272604	0.931818225
lysopcac180.pcaec401	-0.003071361	0.035833279	0.931820088
pcaec445.c102	-0.001834282	0.021414153	0.931863766
pcaac384.pcaac424	0.000838238	0.009793509	0.931916356
pcaec422.pcaac425	-0.007423689	0.08673507	0.931916989
pcaec384.lysopcac160	0.000987432	0.011543963	0.93195961
lysopcac281.pcaac425	-0.006178924	0.072238885	0.931961264
c12.pcaac323	0.004936661	0.057742085	0.931992667
leucine.pcaec363	-0.002525296	0.0295405	0.93199991
c12.asparticacid	0.003325848	0.038912755	0.932013044
pcaec360.pcaec423	-0.000322046	0.003771738	0.932080719
smohc222.pcaac240	-0.000620765	0.007271374	0.932091051
pcaac281.threonine	0.003096012	0.036305395	0.932165751
pcaec360.pcaec380	-0.004468525	0.052422126	0.932194081
serotonin.pcaec406	-0.001504964	0.017658257	0.932205163
glycine.arginine	0.001730014	0.020311184	0.932246253
smc241.pcaac401	-0.000326281	0.003832894	0.932285079
smohc141.tyrosine	-0.004647896	0.054711642	0.932423097
pcaac381.smc240	0.003874202	0.045628683	0.932459145
c141.c141oh	0.011444615	0.134836834	0.932482681
pcaec382.pcaec446	-0.004821948	0.05685204	0.932531689
lysopcac181.pcaac322	-0.00657933	0.077583	0.932541204
methionine.smc181	-0.000751904	0.008867742	0.932551433
lysopcac170.c12	0.003598435	0.042478533	0.932614142
pcaec361.pcaac240	0.001015648	0.012006905	0.932711874
pcaac422.pcaac363	0.001645488	0.019476092	0.932792095
pcaac363.c142	-0.002200919	0.026067609	0.932836866
c142.acetylornithoine	-0.001867218	0.022119196	0.93284876
pcaac406.asparticacid	0.002952956	0.034994041	0.932873881
pcaec402.lysopcac281	-0.001736214	0.020585971	0.932909485
pcaac422.threonine	-0.001957911	0.023219737	0.932924336
c10.pcaec403	-0.001814185	0.021551073	0.933035634
pcaec384.threonine	-0.00288642	0.03432778	0.933112315
pcaac401.pcaec423	0.005630828	0.06703359	0.93317897
pcaac422.putrescine	-0.002913456	0.03469161	0.933193668
pcaec343.lysopcac180	0.00511992	0.060976243	0.933206177
pcaac381.pcaec421	-0.000159607	0.001901257	0.933220231

pcaec364.c182	0.002148168	0.025591102	0.933225079
pcaac401.smc240	0.003844151	0.045869358	0.933332611
smc240.c4	-0.00098873	0.011823968	0.933479961
c181.methionine	-0.001793739	0.021451079	0.933480633
methionine.pcaec446	-0.006600229	0.078934385	0.933483279
methionine.valine	0.003472193	0.041533172	0.933496117
c51.smohc141	-0.00066209	0.007921181	0.933508591
pcaec403.c3	-0.003399252	0.040699798	0.933559904
c10.smc181	0.001795316	0.021499362	0.933571449
spermidine.arginine	0.004474012	0.053604704	0.933605191
smohc141.smc160	0.010580728	0.126934802	0.933690454
c141.lysopcac182	-0.001779741	0.021363448	0.933728415
smohc141.asymmetricdimethylargin	0.001633788	0.019622232	0.933764689
pcaec384.pcaec365	-0.00181197	0.021775392	0.933804558
lysopcac280.pcaac322	0.003077379	0.036996527	0.93382965
lysine.c10	0.000238676	0.002869472	0.933831698
pcaac401.phenylalanine	-0.003737215	0.044964868	0.933882124
c8.pcaec403	-0.001896919	0.02283289	0.933910482
pcaec360.lysopcac161	-0.005703274	0.068691117	0.933950606
c3.pcaec385	-0.003043391	0.036664465	0.933967488
pcaec406.pcaac401	0.00045258	0.005456577	0.934018616
ornithine.lysopcac182	-0.000950217	0.011457162	0.934023118
pcaac366.pcaec405	-0.003757575	0.045319397	0.934041593
pcaac421.pcaec343	0.006446931	0.077761652	0.934047056
pcaac363.pcaac385	-0.004911542	0.059243369	0.934048482
pcaac383.pcaec425	-0.001520763	0.018343724	0.934049114
pcaec404.pcaac402	-0.00968056	0.11681477	0.934075054
glycine.alphaaminoadipicacid	0.001956067	0.023608308	0.934087758
pcaec422.pcaec384	-0.002495614	0.030124342	0.9340967
pcaac425.pcaac383	-0.000375816	0.004552812	0.934333111
pcaac342.taurine	0.001873457	0.022700535	0.934346361
h1.pcaec301	-0.006039671	0.073210934	0.934372037
histidine.pcaac240	0.000948983	0.011505668	0.934385686
c162.pcaac383	-0.000422134	0.005121149	0.9344254
lysopcac161.valine	-0.004382219	0.053165019	0.934427684
pcaac406.c8	0.003462988	0.042024632	0.934445915
acetylornithoine.smc260	0.002445351	0.029675396	0.934446285
alphaaminoadipicacid.pcaac420	0.004135167	0.050239873	0.934521555
pcaac323.leucine	-0.000641463	0.007795643	0.934540309
pcaec381.smc240	0.005591925	0.068141588	0.934716137
isoleucine.methionine	-0.00262842	0.032100596	0.934861071
h1.pcaac322	-0.004725294	0.057712013	0.934863922
c3.tyrosine	0.003336037	0.040833696	0.935005998
c162.c142	-0.005271726	0.064567265	0.935046551
c162.putrescine	0.001059338	0.012982608	0.93508649

pcaac366.pcaac344	-0.004915339	0.060257914	0.935106382
pcaac405.c51	-0.001949535	0.023902688	0.935114585
pcaec423.tyrosine	-0.003831348	0.046977071	0.935117368
c18.smc260	-0.001670787	0.020493502	0.935141315
pcaec402.pcaec423	0.001787093	0.021923274	0.935150744
pcaec404.pcaac425	0.007337282	0.090146743	0.935248488
c8.pcaec340	-0.002850874	0.035067483	0.935324568
pcaec444.pcaec361	-0.000788331	0.009703905	0.935370843
c102.pcaec420	0.000945057	0.011635407	0.935383534
c16.pcaec322	0.003828565	0.047147731	0.935398548
asparagine.c10	0.00017603	0.002169385	0.935446884
pcaac424.asparagine	-0.001208975	0.014904359	0.935468448
c51.pcaac362	0.00060907	0.007518086	0.935549032
pcaac420.c4	-0.000888352	0.010978399	0.935625093
smc180.pcaec360	0.000848439	0.010488791	0.935647399
methioninesulfoxide.alanine	-0.002222968	0.027592128	0.93590523
glycine.spermidine	0.004324163	0.05370149	0.935939433
pcaec443.alanine	-0.001158998	0.014460144	0.936233957
smohc221.asparticacid	0.001922309	0.024029503	0.936355696
alphaaminoadipicacid.pcaac405	-0.003573461	0.044678426	0.93636844
pcaec361.c3	-0.002377257	0.02973558	0.936396429
pcaec404.pcaec420	0.001843147	0.023137088	0.936622335
pcaec382.pcaec421	-0.00036418	0.004572389	0.936633708
pcaac361.pcaec362	-0.002510785	0.031551732	0.936689978
pcaec360.isoleucine	-0.001075625	0.013522895	0.936718367
pcaec300.pcaec386	-0.000525849	0.006614379	0.93675022
pcaac363.glutamicacid	0.002958013	0.037211719	0.936757747
pcaec384.pcaac383	0.000284008	0.003574796	0.936792773
pcaac401.c182	0.002542681	0.032032251	0.936847168
pcaec385.pcaec423	0.000692763	0.00873136	0.936876461
pcaec341.pcaec365	-0.00123423	0.015578316	0.936967309
isoleucine.smc161	-0.00282449	0.03565152	0.93696929
isoleucine.asparticacid	0.00129111	0.016297498	0.936972143
pcaec404.pcaac420	-0.006098388	0.076992212	0.936982871
pcaac320.pcaac383	-0.000326003	0.004116025	0.93698645
smc181.pcaec402	-0.002674786	0.033784026	0.937010468
methionine.lysine	0.004804928	0.060695652	0.937017468
smohc222.asymmetricdimethylargin	-0.001591067	0.02010154	0.9370276
lysopcac140.smc202	0.002266003	0.028662808	0.937102444
pcaec444.c12	-0.002922147	0.037002862	0.937171042
lysine.c4	0.000514183	0.006523327	0.9372891
arginine.pcaac323	-0.008433506	0.107314674	0.937476034
acetylornithoine.kynurenine	-0.001636332	0.02082249	0.937477499
pcaac404.methioninesulfoxide	-0.002043827	0.026070275	0.937626788
lysopcac160.pcaac420	0.005444729	0.069470135	0.937644031

pcaac420.c142	0.003587447	0.0457749	0.937646896
pcaac361.pcaec301	-0.005177199	0.066091742	0.937677042
creatinine.methioninesulfoxide	-0.002157259	0.027542832	0.937684781
glycine.lysopcac161	-0.002449028	0.031268287	0.937685355
pcaec382.pcaec423	0.001635773	0.020889981	0.937700328
pcaec362.lysine	-0.003210117	0.041003605	0.937712664
leucine.pcaec343	0.004789302	0.061225638	0.93776417
alanine.smc160	0.004767887	0.060974535	0.93778725
lysopcac170.pcaec362	-0.001680591	0.021503186	0.937818424
lysopcac203.c5	0.001349226	0.017270092	0.937842609
pcaac362.pcaac343	0.002074589	0.02658008	0.937901752
smc180.pcaac426	0.002198593	0.028172437	0.937909662
lysopcac204.serine	-0.005750012	0.073691083	0.937919155
pcaac425.smc180	0.000443492	0.005701099	0.938108125
pcaac321.pcaac424	0.000467995	0.00601739	0.938121528
leucine.pcaac420	-0.003244998	0.041746295	0.938155125
lysopcac160.pcaec365	0.001516553	0.019521177	0.938189916
methionine.isoleucine	0.000857782	0.011047214	0.938222232
pcaec380.pcaec423	-0.000902065	0.0116258	0.93826607
prolinr.asparagine	-0.00141042	0.01818739	0.938299672
pcaac360.h1	0.000143258	0.001847943	0.938320509
pcaac405.pcaac401	0.000200035	0.002581351	0.938344971
pcaac424.pcaac383	-0.000429196	0.005541349	0.938375888
pcaac321.c3	0.001337683	0.017273835	0.938386653
lysopcac204.pcaac322	0.004334135	0.056090425	0.938521103
pcaac240.lysopcac170	-0.002316885	0.029998827	0.938551265
pcaec404.c2	0.005679843	0.073591777	0.938592631
smc240.isoleucine	-0.001263873	0.016381648	0.9386154
pcaac404.pcaac361	0.005263678	0.068229106	0.938619072
pcaac380.pcaac425	-0.006583959	0.085367392	0.938636639
pcaec402.c102	0.002074184	0.026901559	0.938654297
pcaac384.serotonin	-0.004664563	0.060570955	0.93872802
c141oh.creatinine	-0.001396883	0.018142067	0.938738356
asymmetricdimethylarginine.pcaac36	-0.00382055	0.049633166	0.938755146
lysopcac140.smc260	-0.001701037	0.022107636	0.938780822
asymmetricdimethylarginine.pcaec36	0.001612225	0.020973037	0.93883808
pcaac425.c182	0.002178495	0.028340387	0.938839976
smc160.ornithine	-0.001506274	0.019611409	0.938889927
pcaac366.alanine	-0.000954168	0.01244271	0.938986068
pcaec300.pcaec340	-0.008447913	0.110382702	0.939106762
lysopcac203.c2	0.005265496	0.068892588	0.939188091
c0.c10	-0.002384682	0.03120165	0.939190046
h1.pcaec322	0.002336105	0.030569998	0.939197855
pcaac422.ornithine	0.001696516	0.022216202	0.939240971
arginine.acetylornithoine	0.003037572	0.039874602	0.939388526

pcaec383.smohc141	-0.00151434	0.019879653	0.939390696
serine.smc180	-0.000584736	0.007680041	0.939421103
pcaac425.methioninesulfoxide	-0.002173492	0.028550934	0.939429266
pcaac344.threonine	0.001893142	0.024871181	0.93943636
pcaac360.tyrosine	0.00351177	0.04620344	0.939524669
pcaec445.pcaac405	-0.004896128	0.064491411	0.939594239
c16.glutamine	-0.001424793	0.018777266	0.939626347
pcaec300.c14	-0.002171323	0.028615882	0.939626666
lysopcac203.pcaac363	-0.000774832	0.010212275	0.93963114
isoleucine.smc260	0.000979609	0.012927548	0.93970715
pcaec386.c3	0.001473714	0.01945015	0.93971359
pcaac361.pcaac300	-0.006272465	0.082802303	0.939726672
pcaac384.c51	-0.001570091	0.020735827	0.939753316
pcaec300.c10	0.000132115	0.001745925	0.939791531
c0.asparagine	0.000868213	0.011484205	0.939847139
c2.c142	-0.003895495	0.051552104	0.939876022
c3.lysopcac140	-0.000143959	0.001905778	0.939896924
c101.lysopcac170	0.001456444	0.019281471	0.939898496
c101.c14	-0.002250756	0.029798159	0.939900517
pcaec402.c142	0.002391608	0.031664268	0.939903071
c101.pcaac364	-0.00243955	0.03232064	0.939943215
pcaec406.pcaac320	0.006789673	0.089985399	0.939964319
pcaac344.pcaec342	0.00649084	0.08606134	0.9399897
alphaaminoadipicacid.pcaec384	0.002118591	0.028131506	0.940077725
lysopcac161.pcaec301	0.002792345	0.037105986	0.940123009
pcaec300.pcaac361	0.005826966	0.077443508	0.940132323
pcaac381.kynurenine	0.000674499	0.00897874	0.940227371
arginine.pcaec363	0.001572163	0.020933148	0.94024152
h1.c51dc	-0.010794408	0.143739846	0.940247212
pcaac383.pcaec423	0.000319616	0.004256723	0.940256591
pcaac365.h1	0.00012235	0.001630589	0.940297022
arginine.pcaec386	0.000596649	0.007953991	0.940314119
alanine.pcaac363	-0.001116106	0.014901424	0.940404083
lysopcac140.pcaec365	0.001616487	0.021595671	0.940441373
pcaec340.pcaec365	-0.001172048	0.015667188	0.940475759
pcaec405.pcaec361	0.001185564	0.015860648	0.940523659
pcaec422.pcaec402	-0.002253566	0.030168493	0.940562898
pcaec444.c182	0.00255165	0.034180182	0.940599777
pcaac424.pcaec422	-0.009015655	0.120825979	0.940628402
pcaec445.pcaec401	0.002246855	0.03013544	0.940674699
pcaec403.c142	0.002376884	0.031897974	0.940709128
pcaac281.lysopcac140	-5.01E-05	0.000671978	0.940720739
leucine.glycine	-0.00253987	0.034114587	0.940760008
smc240.serotonin	0.004418502	0.05948144	0.940892991
pcaac421.c8	0.002574089	0.034707017	0.940986289

smohc241.pcaec361	-0.000588421	0.007938389	0.941020275
ornithine.c101	-0.000697889	0.009416126	0.941025868
pcaac383.smc202	-0.002109249	0.028486224	0.941082966
lysine.pcaec382	0.001021967	0.013805399	0.941097154
arginine.asymmetricdimethylarginine	0.001482397	0.020031428	0.941115476
pcaec301.serotonin	-0.003474913	0.047015151	0.941189405
lysopcac204.h1	0.000159923	0.002166296	0.941258779
c8.pcaac385	-0.001548364	0.020988782	0.941300177
pcaac363.pcaec365	-0.001707641	0.023150665	0.941307307
pcaac361.smohc141	-0.001134116	0.015376707	0.941312573
pcaac320.c102	0.002311895	0.031356824	0.941333872
pcaac403.lysopcac204	0.00440341	0.059796537	0.941404348
c101.lysopcac180	0.003363168	0.045716429	0.941463144
smc241.c3	-0.000983674	0.013375705	0.941482225
smc240.valine	-0.001667032	0.022683151	0.941521736
pcaec362.lysopcac180	0.005123514	0.069722507	0.941527866
pcaec322.lysopcac203	0.001191375	0.016217104	0.941543943
smohc222.pcaec363	-0.002582333	0.035158438	0.941556376
glutamine.c4	0.000579896	0.007901117	0.941599526
asymmetricdimethylarginine.c16	-0.001579354	0.021530743	0.941631842
pcaec423.pcaec300	0.002178278	0.029716711	0.941673162
pcaec364.pcaac281	0.000110274	0.001504625	0.941682107
leucine.isoleucine	0.003292249	0.0449452	0.941713784
lysine.c141	0.001543363	0.021078477	0.941737972
pcaec424.pcaac363	0.000903514	0.012343452	0.941755426
pcaac421.pcaec423	0.004531349	0.061925886	0.94177458
pcaec386.isoleucine	0.002700154	0.036912587	0.941793484
c0.pcaec342	-0.002749694	0.037609431	0.941823772
pcaec342.pcaac424	-0.000705699	0.009655716	0.941844193
pcaac320.c18	0.001831951	0.025068579	0.941850965
pcaec381.c142	-0.002181897	0.029876324	0.941887988
c141oh.lysopcac161	0.004897481	0.067144718	0.941960898
lysopcac170.threonine	0.002174784	0.029892448	0.94210828
pcaec343.threonine	-0.003623976	0.049901174	0.942211987
pcaac342.c4	-0.000771821	0.010629825	0.942223156
pcaec386.pcaec423	-0.000198836	0.00273897	0.94223402
smc161.lysopcac203	-0.000849113	0.011702471	0.942263314
pcaac361.smohc161	0.000865069	0.0119274	0.942287625
c4.threonine	0.002220343	0.030629004	0.942316483
pcaac366.pcaec385	0.002965736	0.040921334	0.942330329
pcaec401.pcaec386	-0.000947562	0.013074888	0.942332085
pcaac404.c51	-0.001897384	0.02618884	0.942349393
c142.c10	-0.000714879	0.00986878	0.942358671
pcaac362.phenylalanine	-0.002581628	0.035722974	0.942494023
pcaec342.pcaec362	0.001244079	0.017221145	0.942515194

pcaec384.pcaec425	-0.000448002	0.006204997	0.942547936
pcaac386.pcaac422	0.002499247	0.034632237	0.942575576
pcaac384.tyrosine	-0.003866798	0.053608141	0.942603017
lysopcac160.pcaec446	-0.003615497	0.050164639	0.942649226
pcaec385.c162	-0.005275761	0.073235164	0.942676228
pcaec405.serotonin	0.006584983	0.091441003	0.942696225
c18.pcaac342	0.000923014	0.012817607	0.942697856
alanine.pcaec423	-0.000305138	0.00423788	0.942704936
pcaac342.pcaec420	0.001181476	0.016439818	0.942812763
c141.c18	-0.001702545	0.023712384	0.942865928
glycine.smohc141	0.001155603	0.016103496	0.942896758
pcaac406.lysopcac281	0.00072922	0.01016899	0.942937131
pcaac405.c10	0.000290361	0.004049218	0.942938851
pcaac323.tyrosine	0.002757403	0.038456139	0.94294321
pcaec365.pcaac281	-8.65E-05	0.001206785	0.942989809
pcaac240.lysine	-0.004636637	0.064859095	0.943113747
lysopcac182.pcaac363	0.000647405	0.009064497	0.943166053
pcaac425.spermidine	0.00382505	0.053578737	0.94319053
pcaec405.smc180	0.000372349	0.005220462	0.943243131
c4.pcaac383	-0.000138378	0.001940464	0.943253737
pcaac381.pcaac401	0.000344435	0.004839336	0.943362986
c4.pcaec425	0.000418001	0.005883123	0.943460846
serine.pcaac320	-0.001535811	0.021625784	0.943487309
lysopcac170.c3	-0.001258951	0.017745095	0.943543852
pcaac425.asparticacid	-0.003436451	0.048440077	0.943547127
pcaac385.smohc161	-0.001018514	0.014362108	0.943567428
pcaac240.pcaec361	0.000873492	0.01232797	0.943616912
pcaac406.smc180	0.001003566	0.014165896	0.943625358
pcaac300.pcaec361	-0.000629473	0.008888449	0.943644885
c5.c51	-0.002881976	0.04069954	0.943651416
pcaec342.lysopcac170	-0.0004711	0.006659132	0.943703874
smc261.pcaec380	-0.002627405	0.037185786	0.943774444
pcaec424.prolinr	-0.001245354	0.017630692	0.943790786
pcaec446.lysine	0.004894419	0.069311232	0.94380707
pcaac320.methionine	-0.0019857	0.028131946	0.943830792
c162.pcaac401	-0.000144087	0.002042342	0.943858992
pcaac240.smc180	0.000258981	0.003672175	0.943878492
smohc221.pcaac406	-0.002357434	0.03343639	0.943894512
glutamicacid.asymmetricdimethylarg	0.001193889	0.016935221	0.94390059
smc241.pcaec365	0.002466879	0.035013855	0.943934765
pcaec383.pcaac404	-0.000672051	0.009554533	0.944026835
c5.pcaec381	-0.005420036	0.077069368	0.944036165
asparagine.pcaec384	-0.00177043	0.025192605	0.944076624
pcaac385.pcaac361	0.003788572	0.053933541	0.944100935
methioninesulfoxide.pcaac404	-0.000502291	0.00716311	0.944198927

leucine.methionine	0.002217341	0.03162425	0.944204198
smc161.taurine	-0.001733259	0.024740006	0.944248884
pcaac406.lysine	-0.005359097	0.07653165	0.944276202
h1.lysopcac280	0.000338201	0.004832298	0.944305645
pcaac402.lysopcac182	0.004467043	0.063923206	0.944389956
citruline.smc202	0.002479952	0.03549623	0.944402801
pcaec423.pcaec403	-0.002604447	0.037300875	0.944436613
asparagine.pcaac365	0.00353209	0.050597028	0.944448079
asparticacid.pcaec403	0.002762995	0.039600272	0.944476771
pcaec421.c12	0.003712938	0.053270415	0.944534217
smc261.transhydroxyproline	-0.001647968	0.023646646	0.944540901
pcaec321.pcaec423	0.000409378	0.005883637	0.944630219
smc181.pcaec425	-0.001701504	0.024458726	0.944640263
pcaec405.smc160	-0.002142038	0.03082724	0.944704715
smc261.glutamine	0.000752848	0.010837251	0.944717949
smc240.threonine	0.001440903	0.020741893	0.944718213
kynurenine.pcaac424	-0.000511078	0.007357366	0.944720902
smc161.c2	0.003150963	0.04537506	0.944738562
putrescine.lysopcac161	-0.002244741	0.03232745	0.944742551
c8.lysopcac161	0.004231075	0.060949922	0.94475743
pcaec383.asparticacid	0.002625481	0.037826882	0.944766217
lysopcac280.putrescine	0.002096066	0.030203166	0.944773307
c101.smc160	-0.003215092	0.04633609	0.944783269
pcaec421.pcaec420	-0.00149865	0.021607369	0.94480558
lysopcac170.smc260	0.002062054	0.029740064	0.94482335
pcaac421.kynurenine	0.000746804	0.010786488	0.944903423
c102.pcaec422	0.002015544	0.029113991	0.944907957
pcaac300.pcaec365	0.001670527	0.024134471	0.944917459
smc160.pcaac426	-0.002764576	0.039967787	0.944955068
c2.putrescine	-0.002180553	0.031555222	0.945008591
pcaac384.asparagine	-0.001090861	0.01578935	0.945019966
pcaac401.pcaec422	-0.002189856	0.031702126	0.945029855
prolinr.pcaec341	-0.001864474	0.027007471	0.945062043
pcaac406.pcaec380	0.002909654	0.042211752	0.945145881
asparticacid.pcaec384	-0.001868377	0.027107176	0.945149383
pcaac320.c14	-0.002679348	0.038881637	0.945161451
pcaac320.acetylornithoine	0.002643388	0.038427767	0.945258276
pcaec405.prolinr	-0.001447142	0.021045299	0.945278395
smc181.isoleucine	0.001149462	0.016721132	0.945294381
c51.c51dc	0.005488071	0.080038543	0.945433582
pcaac401.pcaac344	0.003956622	0.057788523	0.945513507
leucine.pcaac360	0.001282818	0.018736299	0.94551371
pcaec360.prolinr	-0.002066772	0.030199147	0.945536684
kynurenine.pcaec386	0.000298561	0.004365265	0.945571081
ornithine.methioninesulfoxide	-0.001200273	0.017577612	0.945658972

pcaec384.pcaac360	-0.002293455	0.033594715	0.945671584
citruline.pcaec363	-0.001525339	0.022346436	0.945679209
pcaec362.pcaac364	-0.002495726	0.03657243	0.945693584
smohc221.valine	-0.002020151	0.029605682	0.945697868
lysopcac204.pcaac425	-0.003971389	0.058283608	0.9457743
lysopcac180.c101	0.000489455	0.007184936	0.945787495
lysopcac281.pcaec420	0.001463546	0.02150044	0.945828746
pcaec362.smc202	-0.003749612	0.055085208	0.945829711
pcaec404.pcaac342	-0.002644114	0.038882585	0.945882779
putrescine.pcaac320	-0.001011347	0.014878705	0.9459064
pcaac360.lysopcac182	-0.000790245	0.011655276	0.946042501
pcaac323.pcaec424	-0.001583352	0.023356148	0.946050313
pcaec423.c102	-0.001622672	0.023971154	0.946128956
glutamine.lysopcac140	9.82E-05	0.0014512	0.946149925
pcaac402.pcaec341	-0.002010501	0.029718734	0.946162115
pcaec386.smc181	-0.001508689	0.0223015	0.946163157
acetylornithoine.valine	-0.000535755	0.00792754	0.946217276
arginine.lysopcac204	-0.002618614	0.038770007	0.946248483
c0.pcaec425	0.000416604	0.006175898	0.946316722
pcaec446.methioninesulfoxide	0.000876836	0.013012641	0.94637472
c0.pcaac320	0.002838035	0.042129709	0.946389965
asymmetricdimethylarginine.pcaac38	-0.000264687	0.003930179	0.946403426
pcaac323.alanine	-0.00091317	0.013564682	0.946425334
glutamicacid.pcaac401	0.000321003	0.004768544	0.946427727
pcaac322.pcaac385	-0.00098367	0.014613347	0.94643058
smc161.threonine	0.001429853	0.021244929	0.946438406
pcaec342.pcaec365	-0.0013618	0.020243055	0.946462883
pcaac366.spermidine	0.003622902	0.053884698	0.946493178
c51dc.pcaec386	-0.001170268	0.017423611	0.946547751
c141oh.pcaac401	-0.000159696	0.002378041	0.946556804
pcaac401.pcaac420	-0.008934874	0.13307652	0.946567438
c4.pcaac426	-0.002697868	0.040202775	0.946594749
c14.smc240	-0.001279512	0.019070193	0.946604007
pcaac421.c4	0.000780005	0.011626367	0.946608396
c142.pcaac401	-0.000136018	0.002028683	0.946641821
pcaec361.alanine	-0.00082414	0.01230022	0.946677725
asparticacid.smc241	0.001142575	0.017058855	0.946696529
c10.c14	-0.003790188	0.05660339	0.946710816
c8.glutamine	0.001321217	0.019751498	0.946765194
pcaec363.smc240	-0.0013706	0.020512162	0.946823308
smohc221.c8	-0.002254855	0.033804768	0.946915958
h1.smohc141	-0.00156751	0.023529782	0.946982833
c102.pcaec383	-0.003649972	0.054790468	0.946983801
smohc222.pcaac380	0.00293651	0.04411847	0.947029301
pcaec422.pcaec321	-0.002720525	0.040919644	0.947088957

c10.pcaac401	-0.00016869	0.002539157	0.94712811
c51.pcaac401	-0.000134825	0.002033158	0.947225189
pcaac344.asymmetricdimethylarginin	-0.000803907	0.012139178	0.94729593
smc260.pcaec401	-0.001873808	0.028301669	0.947308447
c141.pcaec361	0.000537768	0.008139486	0.947419214
putrescine.pcaac240	-0.001000174	0.015172974	0.94753912
pcaac240.methioninesulfoxide	-0.000987098	0.014975509	0.947542252
lysine.c18	-0.003022478	0.045871201	0.947561025
asymmetricdimethylarginine.smohc2	0.004003898	0.060944235	0.947714246
glycine.acetylornithoine	0.00252615	0.038476521	0.947748755
smc161.pcaec402	-0.001874534	0.028559775	0.947763727
c12.c4	0.000632704	0.009642138	0.9477771
lysopcac180.c162	0.004242279	0.064817972	0.94791183
asparagine.pcaec421	0.000780126	0.011928125	0.94794906
pcaec386.taurine	0.001901891	0.02911151	0.948005504
c51.pcaac344	0.002126915	0.032635669	0.948132476
lysopcac182.lysopcac161	-0.005579919	0.085671376	0.948164114
pcaec385.smc261	0.001751476	0.026900709	0.948182203
pcaec421.pcaec340	-0.003353019	0.051501458	0.948185054
smc241.pcaec383	0.004585806	0.070479135	0.948216182
pcaec321.methionine	-0.001406705	0.021647831	0.948283575
asparagine.c141oh	0.005218398	0.080478583	0.948394268
pcaec343.smc160	0.006317617	0.097483168	0.948421928
pcaec402.serotonin	0.004279951	0.066125484	0.948487557
pcaec340.pcaec362	-0.002024478	0.031307432	0.948535432
lysopcac280.lysopcac204	0.002029284	0.031389329	0.948547836
smc181.pcaac362	-0.002383112	0.036885664	0.948580235
smohc141.pcaec322	-0.005438557	0.084292752	0.948650339
pcaec422.c14	-0.00537369	0.083376652	0.948705244
pcaac401.lysopcac182	-0.004249069	0.066032665	0.948786939
pcaec443.glycine	-0.000754982	0.011754177	0.948879958
c2.isoleucine	0.000705412	0.010988601	0.94890859
pcaac320.pcaec402	-0.001969072	0.03068489	0.948927815
c181.pcaec403	0.001602494	0.024985958	0.948955611
lysopcac140.pcaec382	0.00052184	0.008157233	0.949085219
asparticacid.c18	0.002502589	0.039185571	0.949170751
c181.lysopcac281	0.000564174	0.008839197	0.949201546
pcaac420.pcaec423	-0.000462927	0.007253664	0.949206727
pcaac404.serotonin	-0.004393361	0.068914127	0.949261223
lysopcac180.ornithine	-0.001897759	0.029769026	0.94926264
histidine.prolinr	0.002335049	0.036638156	0.949275966
c4.c51dc	-0.007421272	0.116446537	0.949277195
smc260.pcaec423	-0.000194292	0.003051031	0.949317191
pcaac425.pcaec420	-0.002762429	0.043396413	0.949337156
pcaec446.c4	-0.001066103	0.016787548	0.949456483

c101.transhydroxyproline	0.001399326	0.022066468	0.949529153
pcaac424.pcaac361	-0.002438314	0.03845948	0.949540748
pcaec300.valine	0.003081532	0.048658399	0.949596112
pcaec365.lysopcac181	0.002127783	0.033643751	0.949663981
glutamine.pcaec342	-0.003104628	0.04911442	0.9496897
pcaac426.pcaac300	0.002178361	0.034474811	0.949709676
pcaac364.c4	-0.001087527	0.017213235	0.949715513
ornithine.kynurenine	0.000173423	0.002746066	0.949736424
pcaac384.h1	5.91E-05	0.000935429	0.94973976
pcaec384.h1	9.06E-05	0.001436365	0.949809225
pcaec446.pcaec401	-0.001935762	0.030734916	0.949872228
smc160.pcaec301	-0.002258366	0.035858539	0.949874333
pcaec405.c102	0.001563646	0.024831373	0.949881696
glycine.c10	0.000257103	0.004083536	0.949889346
glutamine.c141	0.001587586	0.025259704	0.949977125
pcaec406.c4	0.000712208	0.01133376	0.949985881
pcaac281.pcaac300	0.003856279	0.061411218	0.950021769
c141oh.asymmetricdimethylarginine	0.000715853	0.011415782	0.950090959
pcaac386.pcaec420	0.002899487	0.046241764	0.95009458
asparagine.pcaec423	-0.000570585	0.009106558	0.950131386
pcaac426.pcaac322	-0.004339676	0.069309967	0.95016627
pcaac321.pcaec385	-0.002873302	0.045937342	0.950217375
pcaec404.pcaac424	0.001505732	0.024082534	0.950236813
leucine.smc260	-0.00081572	0.013053576	0.950263556
pcaac421.pcaac321	0.001267882	0.020291573	0.950269125
c182.arginine	-0.002659839	0.04264409	0.950356721
pcaac384.pcaec403	0.003549636	0.056942504	0.950385161
smohc241.pcaac426	0.001765667	0.028325025	0.950386155
pcaec342.glycine	-0.002151134	0.034518108	0.950399617
arginine.c12	0.00267026	0.042871463	0.950426442
glycine.h1	0.000169768	0.002729456	0.950495551
smc241.prolinr	0.002017347	0.032493279	0.950585583
pcaec402.lysopcac181	-0.00347718	0.056027608	0.950603993
prolinr.pcaac361	0.001876567	0.030240918	0.950610333
kynurenine.smc180	0.00067754	0.010937385	0.950695156
pcaac342.pcaec423	0.000261347	0.004224934	0.950765825
pcaac403.arginine	-0.003551205	0.057444839	0.950796752
smohc221.smohc222	-0.000747352	0.012096276	0.950825156
ornithine.glutamicacid	0.002470886	0.04000044	0.950834858
pcaec420.asparticacid	0.001617074	0.026199163	0.950873892
c101.alanine	-0.000706738	0.011453354	0.950887055
lysopcac204.pcaec322	0.003219375	0.052238457	0.950948542
pcaac240.pcaec384	0.001939153	0.03147145	0.950958232
pcaac403.pcaec386	-0.000565037	0.009170876	0.950961558
pcaac323.c142	0.00107165	0.017443187	0.951101033

c8.smohc161	0.000540301	0.008795201	0.951105259
pcaac424.lysopcac160	-0.003301304	0.053769032	0.951131834
pcaac360.pcaac281	-8.80E-05	0.001433428	0.951145088
pcaac402.c14	0.004220311	0.068778791	0.951161424
lysopcac181.pcaac401	-0.00013782	0.002246502	0.951170963
pcaec380.pcaec405	-0.004820707	0.078610425	0.951190534
smc240.pcaac361	-0.002551059	0.041621041	0.951215563
serine.smc260	-0.001495644	0.02441538	0.951242811
pcaec420.pcaac404	0.000313101	0.005122318	0.951348855
pcaec421.pcaec384	0.001265968	0.020725725	0.951382954
acetylornithoine.c8	-0.002971129	0.048691643	0.95143278
smc240.c18	-0.001444576	0.023720156	0.95152698
c10.pcaac360	-0.00165575	0.02719339	0.951537165
alphaaminoadipicacid.pcaac360	0.001058439	0.017384752	0.951540958
pcaac386.pcaac320	0.001926153	0.031640599	0.951546682
putrescine.pcaec343	-0.00276239	0.045382868	0.951552585
pcaac363.pcaec362	-0.000916655	0.015091631	0.951655359
c101.pcaac343	-0.001243648	0.020501854	0.951718166
lysine.smohc241	-0.006230465	0.102719175	0.951722104
pcaac240.pcaac360	-0.003752438	0.061921469	0.951766131
c12.lysopcac161	0.003331815	0.054996517	0.951780162
prolinr.isoleucine	-0.000984919	0.016268966	0.951813983
c182.c162	0.006914072	0.114305832	0.951855532
pcaac381.c10	-0.000259601	0.0042964	0.95190686
pcaec403.pcaac422	-0.007107799	0.117783589	0.951967804
pcaac341.c10	0.000131803	0.002185396	0.951995955
pcaac320.pcaac322	0.00433309	0.071971592	0.952079655
pcaec361.c102	0.001847321	0.030684542	0.952081195
lysopcac170.h1	0.000405654	0.006738399	0.952083827
pcaec340.serotonin	0.004136761	0.068732301	0.952094758
acetylornithoine.pcaac360	0.000775775	0.012893142	0.952108266
isoleucine.c10	0.000417853	0.006949317	0.952140808
citruline.pcaac365	0.001975433	0.032862446	0.952153886
pcaec424.pcaec361	0.000553249	0.009220788	0.952242894
c141.pcaac323	0.003850686	0.064270999	0.952312013
lysopcac280.pcaac240	0.001833221	0.030634653	0.952369132
pcaec421.methioninesulfoxide	0.000793897	0.0132733	0.952392868
isoleucine.pcaac360	0.001387222	0.023194885	0.952396331
creatinine.pcaec322	-0.002969112	0.049648634	0.952400093
lysopcac204.pcaac362	0.000679834	0.011371992	0.952416858
c18.methionine	-0.002863642	0.047947685	0.952462252
c181.c51	-0.002784424	0.046641537	0.952482868
pcaec386.c162	-0.003035941	0.051081087	0.95269323
pcaec402.pcaac344	-0.005527702	0.093022099	0.952701359
pcaec364.pcaac422	-0.002600826	0.043794539	0.952730403

pcaac321.smohc161	0.001059483	0.017847781	0.952750148
creatinine.smohc141	-0.004547592	0.076607808	0.952750286
pcaac365.c10	0.000190093	0.003202539	0.952754379
pcaec363.c8	0.001826548	0.030791637	0.952783978
smohc221.pcaec401	-0.002079159	0.035090814	0.952838683
pcaac300.pcaac322	0.005377958	0.090896173	0.95290615
pcaec362.ornithine	0.001204239	0.020373321	0.952951716
pcaac424.lysopcac204	0.004838388	0.081977258	0.953021302
pcaec421.lysine	-0.004252023	0.072054932	0.953029454
prolinr.smohc221	0.00537495	0.091178254	0.953077904
pcaec380.alanine	-0.000696099	0.011809847	0.953084003
pcaec424.smc160	-0.002367727	0.040178483	0.953093557
c0.pcaec386	0.001173854	0.01995276	0.953171934
citruline.c10	-0.000178985	0.003044064	0.953198523
pcaac402.alanine	0.001279498	0.021768214	0.953214364
c162.pcaac360	-0.000759522	0.012924939	0.953225627
c16.acetylornithoine	-0.002549855	0.043508827	0.953351755
lysopcac281.glutamicacid	-0.003198156	0.054637541	0.953408541
pcaac380.methionine	0.002115291	0.036162631	0.953440509
smc240.pcaec365	-0.001721453	0.029446257	0.953466749
pcaec446.pcaac320	0.001782786	0.030526851	0.953514654
pcaec401.c12	0.002487702	0.042597354	0.953514804
pcaac344.c10	0.000228933	0.003920335	0.953518151
c10.pcaec340	-0.001707999	0.029254033	0.953526983
pcaac366.pcaec423	-0.000322256	0.005521293	0.953542102
pcaec421.pcaec341	-0.001346488	0.023084738	0.953572294
pcaac426.c10	0.000155241	0.002662785	0.953594348
pcaac240.glycine	-0.001586531	0.027242835	0.953645012
pcaec401.serotonin	-0.005644683	0.096977469	0.953669254
creatinine.smc261	-0.001073114	0.018444132	0.95368855
smc241.pcaac366	-0.002897022	0.049827552	0.953721062
c14.smc161	-0.00231515	0.039843565	0.953748856
c141.c10	0.000115748	0.001994226	0.953800084
arginine.c102	-0.002080548	0.035859676	0.953817918
pcaec425.c4	0.000661638	0.011411658	0.953849764
glutamine.pcaac320	0.001095824	0.018902858	0.953855934
smc241.smohc141	0.001056019	0.018220554	0.953866873
pcaec384.asymmetricdimethylarginir	0.001244643	0.021480101	0.953877676
kynurenine.pcaec446	0.00375883	0.064883515	0.953887227
pcaac386.pcaac343	-0.00106874	0.018466974	0.953934065
pcaac424.asymmetricdimethylarginir	-0.001438227	0.024881349	0.953989426
pcaac420.serine	-0.002175442	0.037638367	0.953993322
pcaec423.lysopcac182	-0.004311753	0.074612344	0.954001118
c51.lysopcac180	0.002162265	0.037441706	0.954031768
putrescine.pcaac363	0.000409656	0.00709632	0.954049415

pcaac381.asparagine	-0.00060563	0.010491687	0.954052025
c182.pcaec340	0.00105634	0.018314431	0.954089166
pcaac362.smc241	0.001704134	0.029617209	0.954199953
c10.pcaac322	0.002349269	0.040837313	0.954208815
smc241.pcaec380	-0.002485926	0.043259191	0.954257835
pcaec424.lysopcac182	-0.004567402	0.079523038	0.954282404
pcaec404.methioninesulfoxide	0.000815623	0.014206501	0.954300686
smohc141.pcaac365	-0.002542558	0.044296003	0.954310786
pcaec360.c3	0.004499753	0.078479525	0.95436057
pcaac381.lysopcac180	-0.00272421	0.04755447	0.954400761
pcaac426.pcaec343	-0.003626045	0.063299521	0.954402493
pcaac281.c102	-0.00140544	0.024553487	0.95443746
pcaec340.c10	9.83E-05	0.001717303	0.954449753
c8.c10	-0.000173801	0.003043293	0.954541055
methionine.c4	0.000525485	0.00920459	0.95455712
c2.lysopcac182	0.003820267	0.066918849	0.954558224
pcaec340.pcaec386	-0.000343645	0.006024559	0.954595871
lysopcac280.c182	0.001895386	0.03323006	0.954597802
pcaec383.smohc241	-0.004006674	0.070326842	0.954650364
pcaac343.c10	0.000216813	0.003806634	0.954662704
pcaec365.pcaec420	-0.000962413	0.016901817	0.954674828
lysopcac182.c18	-0.001785942	0.031376538	0.95469212
pcaec343.pcaec423	-0.001087341	0.019118159	0.954727816
lysopcac180.pcaec385	0.002079675	0.036582143	0.954747936
pcaac424.pcaec383	-0.008908604	0.156898847	0.954803745
glutamine.pcaec365	-0.001105667	0.019486534	0.954834951
pcaac406.alanine	-0.001546827	0.027274167	0.95485565
c8.smc181	0.001157879	0.020443927	0.954917009
c4.ornithine	-0.002170767	0.038380134	0.954978374
kynurenine.smc161	0.002796086	0.049457578	0.954997961
lysopcac281.asparticacid	0.002064808	0.036523955	0.95499959
pcaec360.pcaac365	0.002796753	0.049508202	0.95503321
pcaac425.serine	-0.002959309	0.052397081	0.955042909
pcaac341.pcaac342	-0.000964188	0.017091614	0.955095045
lysopcac181.pcaec425	0.000296267	0.005255067	0.955123337
lysopcac203.smc181	0.000502702	0.008943247	0.955256219
methioninesulfoxide.smc260	-0.002500891	0.044533085	0.955297729
pcaac366.pcaac364	0.002705212	0.048242468	0.955363496
pcaec403.pcaec446	0.002430501	0.043375815	0.955396718
pcaec381.threonine	0.001520421	0.027142616	0.955410665
pcaac365.pcaac300	-0.003144615	0.056196381	0.955457105
pcaac343.pcaac424	0.000693659	0.012396699	0.955459054
smc181.pcaec382	0.000644356	0.011515888	0.955460216
pcaec383.lysopcac140	-9.47E-05	0.001696182	0.955539745
pcaac426.pcaec423	-0.000243706	0.004370407	0.955611973

pcaec404.alanine	0.000881924	0.015828441	0.955647893
c51.pcaac360	-0.000852531	0.015308325	0.955669358
pcaac426.pcaec420	-0.003466613	0.06226284	0.95568018
smohc221.transhydroxyproline	0.001432272	0.025759085	0.955739406
smc241.pcaac383	-0.000180233	0.00324309	0.95576177
pcaec425.c182	-0.001470175	0.026461712	0.955774394
pcaec365.pcaec423	0.000305242	0.00549541	0.955785257
c8.c3	0.003143217	0.056791703	0.955943115
smohc161.c51	-0.001467567	0.026530066	0.9559664
smc241.c102	0.001221876	0.022106738	0.956002577
pcaec404.pcaec365	0.001131887	0.020483254	0.956012507
c14.pcaec420	0.001036053	0.018781365	0.956088252
lysopcac204.asymmetricdimethylarg	0.000998352	0.018100583	0.956094708
pcaac360.isoleucine	0.001571882	0.028529364	0.956141468
c2.pcaac383	0.000379005	0.006888901	0.956205264
pcaac386.pcaac300	0.003691241	0.067193383	0.9562706
pcaac425.pcaec384	0.001127658	0.020568455	0.956358032
isoleucine.pcaac422	0.00225393	0.041132322	0.956379974
pcaac425.lysopcac161	0.003691954	0.067516332	0.956471162
pcaac362.pcaec421	-0.000247219	0.004522567	0.956486215
pcaec382.methioninesulfoxide	0.000927646	0.016971837	0.956490587
smohc161.pcaec423	0.000676109	0.012409074	0.956628085
creatinine.pcaac322	0.001906732	0.034995777	0.956628419
alanine.c51dc	0.00505379	0.092860424	0.956677036
pcaac343.smohc241	-0.002307987	0.042415893	0.95668519
smc202.c10	0.000117336	0.002160274	0.956763086
smohc221.pcaec404	-0.000997509	0.018375462	0.9567873
serine.pcaec383	-0.003457439	0.063720647	0.956807602
pcaec300.pcaac344	0.004164889	0.07676768	0.956812502
smohc221.smc260	-0.001778627	0.032881508	0.956940642
pcaec382.pcaac240	-0.001152459	0.021311133	0.956951948
glutamicacid.transhydroxyproline	-0.002007536	0.037140116	0.956971663
pcaac362.pcaac364	-0.00292483	0.054126179	0.95698421
pcaec386.pcaec420	0.001236617	0.022885307	0.956985679
pcaec384.lysopcac203	-0.00056705	0.010496867	0.956997221
pcaec364.lysopcac140	-5.33E-05	0.000987636	0.957020467
asparticacid.pcaec386	-0.000429035	0.007966614	0.957129838
pcaec380.pcaac344	0.00294347	0.054676404	0.957145541
pcaac381.smc261	0.001746382	0.032473896	0.95719036
pcaac385.asparagine	-0.000329048	0.006126138	0.957242729
pcaec342.lysopcac280	-0.000517215	0.009632854	0.957258136
pcaec301.ornithine	0.000938691	0.017483685	0.957260744
lysine.pcaec362	-0.000489756	0.009122158	0.957261472
pcaec322.methionine	-0.000961725	0.017927862	0.957296835
pcaec421.pcaac421	0.004145871	0.07730414	0.957307562

lysopcac181.pcaac366	-0.0011398	0.021287356	0.95737684
serotonin.lysopcac160	-0.000592362	0.011064175	0.95738063
pcaec360.pcaac360	0.002026722	0.037869003	0.95739612
pcaac381.smc202	-0.00201136	0.03761074	0.957428688
pcaec361.smc161	-0.004080406	0.076459203	0.957517159
c141.pcaac361	0.001382156	0.025935936	0.957577562
citruline.c162	-0.005202753	0.097839395	0.95766877
pcaac281.pcaec382	0.000452474	0.008518895	0.957718304
pcaec362.c4	-0.000599257	0.01135237	0.957978584
pcaac422.smc181	-0.000364007	0.006898998	0.957998114
serine.pcaac363	0.000526455	0.009979104	0.958003381
pcaac240.alanine	0.001082385	0.020527222	0.958024425
asparticacid.pcaec423	-0.002072328	0.039325949	0.958050728
h1.smohc241	-0.000840449	0.015951881	0.958058421
c3.pcaac320	0.004661873	0.088514372	0.958073175
ornithine.pcaac362	-0.000412129	0.007856412	0.958240477
pcaac425.c141	-0.001843263	0.035144117	0.958247579
c18.asparagine	-0.000721041	0.013758031	0.95827935
putrescine.c10	0.00012377	0.002363019	0.958303971
c3.methionine	-0.001356424	0.025903201	0.958314052
histidine.pcaec301	0.0032718	0.062532818	0.958348879
smc160.glutamicacid	-0.001492516	0.02860114	0.958458245
smohc221.pcaac360	-0.002032271	0.038982761	0.958499018
putrescine.pcaec422	-0.00125718	0.024146282	0.958552612
smc260.c8	-0.001576963	0.030377422	0.958674158
c101.c10	-0.001094167	0.021091727	0.958702586
pcaac364.serine	-0.001774208	0.034212405	0.958716885
smohc221.smohc241	-0.00447649	0.086470222	0.95878804
ornithine.pcaec405	0.002318929	0.044801542	0.958795296
pcaac386.pcaac360	0.00133107	0.025750482	0.958850145
smohc161.c2	0.002912984	0.056369696	0.958861796
isoleucine.asymmetricdimethylargini	-0.001230292	0.023808596	0.958863497
asparticacid.c101	-0.000993153	0.019257244	0.958944097
isoleucine.pcaec362	-0.0004146	0.008039536	0.958946351
pcaec382.taurine	0.00250487	0.048632508	0.958997271
prolinr.smohc241	-0.001452378	0.028213809	0.959019962
pcaac361.lysopcac140	9.01E-05	0.001752941	0.959067146
pcaac300.methioninesulfoxide	0.001701726	0.033103506	0.959076703
smc202.isoleucine	-0.000965161	0.018776644	0.959079897
pcaec403.lysopcac204	0.003733819	0.072696941	0.959112325
pcaac344.c142	0.000742209	0.01452862	0.959331397
lysopcac182.pcaac323	-0.003735552	0.073354302	0.959459627
smc241.pcaac380	0.002568498	0.050450293	0.959470234
pcaac344.smc260	-0.000907985	0.017854947	0.95951641
pcaec446.pcaec363	-0.001730598	0.034051427	0.959540525

smohc222.lysopcac140	-8.53E-05	0.001678576	0.959562047
pcaec341.isoleucine	0.000668232	0.013155846	0.95956404
methionine.c142	-0.001476134	0.029070316	0.95957634
pcaec383.pcaec404	0.003457263	0.068189732	0.959637913
pcaec444.pcaec425	-0.000272507	0.005378124	0.959662717
h1.pcaac364	-0.000811118	0.016011605	0.959671716
alphaaminoadipicacid.glutamicacid	-0.002651802	0.052350006	0.959674024
pcaac281.c10	7.17E-05	0.001416307	0.959696284
lysopcac160.pcaec443	0.003599345	0.071126719	0.959714241
c102.arginine	-0.002022295	0.039964951	0.959716606
pcaec380.pcaac401	0.000189125	0.003748483	0.959834262
alanine.c12	0.001146076	0.022744466	0.959885613
pcaac426.lysopcac161	0.004101391	0.081459642	0.959917813
lysopcac204.pcaac420	0.003079843	0.061207663	0.959942321
glycine.pcaec423	-0.001036315	0.020615011	0.959980502
methionine.pcaac401	-4.61E-05	0.000917672	0.95998337
pcaac383.pcaac401	0.000178468	0.003553608	0.960018997
pcaac363.pcaac323	0.003470099	0.069176854	0.96006578
lysopcac204.c3	-0.000942689	0.018800525	0.960082547
c12.ornithine	-0.001574906	0.031419854	0.960096146
leucine.pcaec386	-0.000432582	0.008636909	0.960127337
smohc222.methionine	-0.000787561	0.015726728	0.960133268
pcaec361.pcaec423	-0.0002342	0.00468103	0.960169896
smc202.pcaac320	-0.002691813	0.053809805	0.960175662
c102.pcaec385	-0.002897079	0.057923581	0.960182851
c141.c51	-0.001186826	0.023742587	0.960205384
pcaec362.pcaac240	0.000431086	0.008628299	0.960225508
leucine.histidine	0.003642119	0.07292209	0.960238682
pcaec343.ornithine	0.00131603	0.026354103	0.960245767
pcaac420.threonine	0.001010298	0.020267718	0.960316395
c102.pcaac421	-0.000404315	0.008118718	0.96035398
serine.pcaac424	-0.000471766	0.009474312	0.960358934
pcaec420.pcaac363	-0.00069494	0.013959454	0.96036801
pcaec363.pcaac361	-0.003217709	0.064717584	0.960418533
histidine.smc180	0.001320935	0.026591172	0.960453167
glutamine.smc160	-0.00241154	0.04859011	0.960489316
pcaac426.acetylornithoine	0.001120588	0.022578813	0.960489475
glycine.pcaec301	0.002052554	0.041381946	0.960513207
citruline.pcaec446	0.004226831	0.085229832	0.960518691
pcaac402.pcaec386	-0.000507642	0.010241194	0.960538349
ornithine.pcaec322	0.002834953	0.057193322	0.960538921
c3.c51	0.001098537	0.022188388	0.960585323
c182.glutamicacid	-0.002539717	0.051301897	0.960588704
pcaec423.pcaac361	-0.002601252	0.052646832	0.960664938
lysopcac181.pcaac420	-0.003769557	0.076319558	0.960679033

pcaac406.pcaec422	0.000825296	0.016739506	0.960750192
c51dc.smc180	-0.000731045	0.014847569	0.960802376
alanine.pcaac360	-0.001563239	0.031784074	0.960845015
lysopcac161.asymmetricdimethylarg	0.001066998	0.021709648	0.960872478
pcaec420.pcaec402	-0.002064393	0.042036749	0.960903765
pcaac385.c10	0.000129008	0.002627838	0.960916809
smohc141.pcaac424	-0.000347096	0.007077369	0.960956358
asparagine.pcaac401	-3.64E-05	0.000743816	0.961005027
c3.pcaac401	8.42E-05	0.001720148	0.96101138
pcaac384.pcaec386	-0.000675475	0.013792737	0.96101196
pcaac343.pcaec386	0.000380406	0.007769793	0.961022799
pcaac321.pcaec361	-0.000510821	0.010436284	0.961033088
pcaac240.arginine	0.00150834	0.030820974	0.961039392
smc241.isoleucine	0.000884173	0.018105221	0.961121735
alanine.putrescine	-0.001477627	0.030259898	0.961124928
c181.putrescine	0.00152251	0.031180068	0.961126201
smc161.pcaac321	0.000970798	0.019889205	0.961141558
pcaec360.c8	0.002247691	0.046116547	0.961197989
glutamine.pcaec341	0.002080651	0.042735338	0.961239723
c162.c10	-0.000700879	0.014395702	0.961239919
pcaec424.pcaac383	0.000212381	0.004374538	0.961349056
h1.c4	-0.002722055	0.056072086	0.96135209
lysine.acetylornithoine	0.001365469	0.028130936	0.961356777
serine.pcaec386	-0.000270578	0.00557675	0.961373337
h1.pcaec362	0.000938558	0.019361617	0.96140812
c14.pcaac362	-0.001812851	0.037474165	0.961487014
pcaec403.pcaac383	0.000370499	0.007678706	0.961587104
pcaec385.pcaec420	0.001136361	0.023551452	0.96158714
creatinine.c10	-0.000126187	0.0026163	0.961602343
c51dc.pcaac401	0.000304665	0.006326013	0.961658332
pcaac343.pcaac401	-0.000133953	0.002783383	0.961685998
pcaac364.lysopcac182	0.001651307	0.034423012	0.961809205
glycine.c51dc	-0.005016379	0.104681789	0.961849576
pcaac385.pcaec423	-0.000168584	0.003528353	0.961961421
pcaac363.smc260	-0.001123314	0.023598784	0.962103962
pcaec340.pcaac240	0.000659	0.013845155	0.962106038
pcaec322.acetylornithoine	0.003049538	0.064077333	0.962111089
c51.c10	-0.00029501	0.006211444	0.962188244
pcaec446.smc202	-0.00234224	0.049371106	0.962230427
glutamine.pcaac344	0.00227659	0.048003661	0.962243303
methionine.pcaec402	0.001974715	0.041670834	0.962272665
pcaec380.smc180	0.000297131	0.006280278	0.962333637
c4.c51	-0.000941001	0.019895014	0.962344326
methionine.pcaec301	0.002636107	0.055739599	0.962348375
pcaec402.lysopcac204	0.002073717	0.043937276	0.962424788

pcaec443.kynurenine	-0.00058327	0.01245589	0.9627194
c141.asymmetricdimethylarginine	-0.00059276	0.012680522	0.962783959
acetylornithoine.pcaec422	0.001135614	0.024293483	0.962784051
smc160.pcaec386	-0.001165815	0.024946527	0.962794429
c162.pcaac385	-0.001364274	0.029275736	0.962899209
smc161.c10	6.81E-05	0.001460935	0.962905524
serine.h1	9.79E-05	0.002107266	0.963009945
c51.c3	-0.001078138	0.023231994	0.963053079
pcaac320.c10	7.56E-05	0.001629198	0.963061299
smc160.pcaac320	-0.003646359	0.078851468	0.963183606
pcaec365.pcaec405	-0.00209539	0.04536616	0.96322735
pcaec424.lysine	0.003353862	0.072786365	0.963315053
pcaac342.c10	9.79E-05	0.002125258	0.963334845
leucine.c10	0.000311324	0.006761099	0.963340396
c182.pcaac401	-9.40E-05	0.002043382	0.963370353
pcaac406.pcaec385	-0.002608403	0.056848517	0.963469999
pcaec341.c10	6.84E-05	0.001492476	0.963494763
asparticacid.pcaac363	-0.000393334	0.008588159	0.963536677
c141oh.pcaec383	-0.003918734	0.085870077	0.963667133
pcaec404.lysopcac180	-0.002500907	0.05485612	0.963703193
pcaac361.pcaac401	8.34E-05	0.001828966	0.963710069
pcaac342.c16	0.001646349	0.036153341	0.963744848
lysopcac181.pcaec342	0.003545156	0.077952144	0.963792041
pcaac426.arginine	-0.001167202	0.025682139	0.963816393
leucine.asymmetricdimethylarginine	-0.001551501	0.034179218	0.963860071
pcaec420.pcaac360	-0.001345302	0.029648946	0.963874984
lysopcac170.alanine	-0.001300997	0.028834915	0.964078322
pcaec301.c141oh	0.003104764	0.068835801	0.964090165
smc260.pcaac361	-0.001446608	0.032132723	0.964157089
pcaec382.lysopcac281	0.000685696	0.015242727	0.964184646
pcaac385.pcaac401	9.21E-05	0.002049898	0.964246811
putrescine.c182	0.001045108	0.02327394	0.964248691
c141oh.acetylornithoine	-0.001430579	0.031858858	0.964249492
pcaac380.asymmetricdimethylarginir	0.000948043	0.021129264	0.964277277
lysopcac182.kynurenine	-0.000186174	0.004155739	0.964332532
c2.pcaac380	-0.001242986	0.02777168	0.964365918
arginine.c142	-0.001398891	0.03125652	0.964367619
pcaac240.acetylornithoine	0.000795946	0.017812538	0.964423773
pcaec403.glycine	-0.000650268	0.014566116	0.964457279
pcaac365.pcaec423	-0.000167626	0.003758062	0.964487567
pcaec420.pcaac401	-5.31E-05	0.001191702	0.964513992
creatinine.asymmetricdimethylarginir	0.000664799	0.014930471	0.964549692
smc161.c182	0.000943948	0.021212126	0.964570327
c142.kynurenine	-0.000254011	0.005713294	0.964602734
pcaec381.pcaac344	-0.002777394	0.062490919	0.964614595

smohc222.pcaec422	-0.001518007	0.034218645	0.964680468
pcaac281.pcaac401	0.000102865	0.002318861	0.964682093
pcaec425.c10	8.34E-05	0.001884219	0.964745713
c18.pcaec423	-0.000211678	0.004795259	0.964854528
glycine.lysopcac160	0.000630093	0.014288854	0.964891429
pcaac363.pcaec423	0.000195681	0.004439795	0.964909269
c0.pcaac420	0.001394123	0.031651043	0.964931341
pcaac380.smc181	0.00084989	0.01935984	0.965048325
putrescine.taurine	-0.002153542	0.049145556	0.965111957
pcaac362.pcaac406	-0.00177894	0.040611587	0.965124606
pcaac363.lysopcac281	0.000583635	0.013338175	0.965162009
pcaac360.pcaac364	0.002257168	0.05177211	0.965288204
methioninesulfoxide.pcaac401	0.000564862	0.012973287	0.965334168
c14.pcaac401	-9.86E-05	0.002268117	0.96539393
lysine.pcaec364	-0.000546141	0.012581793	0.965440133
pcaac426.pcaec361	0.000670024	0.015482943	0.965545341
pcaac405.pcaec365	-0.001222793	0.028305423	0.96560503
pcaec445.c10	6.31E-05	0.001461423	0.965640134
pcaec301.histidine	-0.002612401	0.060545047	0.965646334
pcaac366.pcaac365	-0.002395923	0.05563908	0.965714915
lysopcac281.pcaac384	-0.001253752	0.029235751	0.965856272
pcaec341.glutamicacid	0.001420288	0.033133168	0.965870714
pcaec401.spermidine	0.00224722	0.052458419	0.965892943
pcaec380.pcaec420	-0.000912662	0.021341478	0.965951371
glutamicacid.c182	0.002286898	0.053675146	0.966077426
pcaac363.c10	8.78E-05	0.002063395	0.966124848
lysopcac161.pcaec401	0.002785137	0.065603005	0.966198269
lysopcac281.pcaac401	9.72E-05	0.002298438	0.966314785
lysopcac203.pcaac281	-0.000125905	0.002976327	0.966319529
pcaac381.pcaec446	-0.002217221	0.052462526	0.966350612
pcaec402.putrescine	0.002188651	0.051808576	0.966364938
pcaac364.c10	0.000104544	0.002480485	0.966443315
c5.c14	0.001597158	0.037905856	0.966452537
creatinine.lysopcac161	-0.00235409	0.055885647	0.966461701
pcaec386.pcaac364	-0.001925074	0.045924713	0.966625065
pcaac300.c142	0.000854344	0.020456512	0.966747696
c162.methionine	0.000827093	0.019835639	0.966800692
pcaec385.acetylornithoine	-0.002308544	0.055440792	0.966846463
methionine.c3	-0.002984405	0.071794356	0.966902963
methioninesulfoxide.putrescine	-0.002080392	0.050074383	0.966921083
pcaec381.lysine	0.00376204	0.090654658	0.966958835
lysopcac140.asymmetricdimethylarg	-0.000504613	0.01216946	0.966985141
pcaac321.pcaec421	-0.000342527	0.008266085	0.967007356
pcaac342.lysopcac182	-0.00056333	0.01359744	0.967014089
c14.c18	-0.002218036	0.053590139	0.967046133

c182.pcaac342	-0.001442216	0.034877799	0.96707663
smc261.pcaac361	-0.00208019	0.050344116	0.967101407
pcaec362.pcaac401	-0.000125869	0.003047147	0.967111158
pcaac362.smc240	0.002141918	0.05195181	0.967173401
c5.asparticacid	-0.000382648	0.009293757	0.967218213
c162.spermidine	-0.002185082	0.053168267	0.967278011
c3.serine	-0.00214645	0.052261045	0.96729853
lysopcac281.c10	7.89E-05	0.001922069	0.967307081
pcaec401.pcaac321	0.000894761	0.021798167	0.967317773
smc180.smohc222	0.000203815	0.004975205	0.96738253
ornithine.c102	-0.000484596	0.011830635	0.9673865
pcaec300.pcaac401	9.15E-05	0.002239538	0.967469173
acetylornithoine.pcaac344	0.001882809	0.046175087	0.967534294
lysopcac160.kynurenine	-0.000412824	0.010138939	0.967581019
pcaac420.c162	0.001840931	0.045369992	0.96769302
smc202.c141oh	0.002569217	0.063432045	0.967750714
lysopcac161.pcaac383	0.000414938	0.010252803	0.967776787
glutamine.pcaac401	-6.30E-05	0.001565563	0.967971184
pcaac363.smc202	-0.001140482	0.028362875	0.967983928
pcaec360.lysopcac203	-0.00089992	0.022463551	0.968102544
pcaec301.methioninesulfoxide	-0.000429608	0.010733385	0.968131115
pcaec343.pcaac401	-8.78E-05	0.002198047	0.968188455
pcaac422.pcaec446	-0.002260287	0.056664272	0.968239611
c2.glycine	0.000775283	0.019497532	0.968339848
c101.h1	-3.50E-05	0.000879576	0.968341846
pcaac364.pcaec420	0.000715106	0.017986395	0.968343824
pcaec401.pcaac422	0.003556013	0.089541094	0.96837915
pcaec386.pcaec421	3.88E-05	0.000976697	0.968386486
pcaec321.pcaec421	-7.40E-05	0.001867845	0.968457444
lysopcac203.methioninesulfoxide	-0.000690056	0.017426289	0.968470804
phenylalanine.pcaec403	0.003156657	0.079936984	0.96855776
lysopcac280.ornithine	0.004276866	0.109169732	0.968806858
alanine.c51	0.001552964	0.039710649	0.968862019
pcaec322.c10	7.29E-05	0.001863638	0.968873775
pcaec422.c102	0.000916101	0.02354382	0.969018415
pcaec340.smc180	0.001436313	0.036914499	0.969019446
pcaac385.pcaac360	-0.000822612	0.021150203	0.969031658
lysopcac204.c10	0.000409253	0.010543276	0.969093185
methioninesulfoxide.pcaec365	-0.000648287	0.01673506	0.96915549
pcaac402.pcaac401	0.00039417	0.010191719	0.969205422
pcaac341.pcaac343	0.001909008	0.049366727	0.969209843
serotonin.pcaac401	-5.28E-05	0.001367711	0.969284291
lysopcac170.c10	-0.000248652	0.006448136	0.969295869
c8.pcaac401	-6.99E-05	0.001814007	0.969311207
pcaac406.pcaac426	-0.00182819	0.047439699	0.969315523

pcaec340.valine	-0.00229471	0.059551764	0.969318789
pcaec340.c51	-0.001152968	0.029952408	0.969350382
pcaac403.taurine	-0.002730129	0.071020254	0.96939159
glutamicacid.pcaec341	-0.001474595	0.038374949	0.96940399
c16.pcaac362	0.00027801	0.007241729	0.96943264
asymmetricdimethylarginine.lysine	0.002737868	0.071386973	0.969462473
pcaec381.smc261	-0.002167221	0.056510411	0.969463795
lysopcac281.pcaac341	0.000346449	0.009036017	0.969471668
smohc141.pcaac401	8.49E-05	0.002223305	0.969599703
smc260.pcaac426	0.001321309	0.034730729	0.969707675
glutamicacid.lysopcac281	-0.000348381	0.009160658	0.969719015
pcaec425.pcaac420	0.00225485	0.059380435	0.969764537
pcaac384.pcaac240	-0.000656544	0.017311283	0.969802071
smc261.c10	6.07E-05	0.001603055	0.969839925
pcaac300.pcaac401	0.000108749	0.002874543	0.969876794
pcaec340.lysopcac281	0.000511988	0.013538108	0.969887601
pcaac383.c141oh	-0.003815976	0.100984367	0.969911888
c2.c10	-0.001232429	0.0326182	0.96991531
c182.pcaac322	-0.001640912	0.043454152	0.969932486
pcaac421.serotonin	-0.003694785	0.097874296	0.96994172
c101.pcaac322	0.001419021	0.037622301	0.969967785
pcaac320.valine	0.001199165	0.031821699	0.969994575
lysopcac181.methionine	-0.000988787	0.02629323	0.970056456
pcaac365.pcaec340	-0.001386782	0.036912975	0.970086044
lysopcac140.h1	-0.000115083	0.003074721	0.970197494
smc181.c10	6.58E-05	0.00175952	0.9702076
asparagine.c4	-0.000219026	0.005856678	0.970222385
pcaec386.c4	0.000703682	0.018868373	0.970304659
pcaac322.c4	-0.000297788	0.007993461	0.970336728
c16.c3	-0.002495755	0.067232138	0.970442194
pcaac362.isoleucine	-0.00078067	0.021041105	0.970457581
pcaec443.pcaec386	0.000484318	0.013061168	0.970474563
smohc222.c10	5.69E-05	0.001534411	0.970484184
pcaec406.smc240	0.00215697	0.058296096	0.970538662
smohc222.pcaac402	-0.002412915	0.065244068	0.970552471
pcaec342.pcaec423	0.000271176	0.007355304	0.970643879
pcaec423.prolinr	0.001155554	0.03136369	0.970663269
pcaac403.pcaac401	0.000287129	0.007798018	0.970681558
asparagine.smohc241	-0.002190136	0.059504594	0.970693133
pcaac342.pcaac401	-8.87E-05	0.002413597	0.970731805
pcaec445.pcaac401	-0.000179366	0.004883949	0.970757217
acetylornithoine.pcaec421	0.000261391	0.007131241	0.970813977
lysopcac203.pcaac365	0.002115039	0.057762269	0.970844266
histidine.pcaac320	0.000786659	0.021490587	0.970853386
pcaac360.pcaec421	6.71E-05	0.001838625	0.970942729

smc160.pcaac300	-0.00190587	0.052272817	0.970968595
pcaac404.c10	0.000126899	0.003482775	0.970987692
pcaec300.methioninesulfoxide	-0.00054833	0.015053295	0.970995777
pcaac404.c101	-0.000160585	0.004408919	0.970998293
c4.c10	0.000633816	0.0174155	0.971021364
lysopcac181.glutamicacid	0.001251366	0.034415995	0.971048235
glutamine.c3	-0.001245847	0.03434056	0.971112566
pcaac421.c182	-0.001065366	0.029373313	0.971119963
pcaec425.smc160	-0.001014838	0.027984742	0.971124672
lysopcac281.pcaec402	-0.002541163	0.070131932	0.971148493
isoleucine.c102	-0.000451	0.01245541	0.971168278
pcaac323.c0	2.96E-05	0.000818471	0.971188926
pcaac385.glycine	-0.000877451	0.024289453	0.971235456
smc241.c8	0.00118354	0.032766632	0.971239032
c141.ornithine	0.000898113	0.024928644	0.971312996
glutamine.glutamicacid	-0.002087234	0.058031361	0.971360757
h1.c10	-0.000117348	0.003273242	0.971453642
pcaec405.c10	9.34E-05	0.002620465	0.971627659
c101.pcaec386	-0.000257424	0.00722904	0.971645389
pcaac381.pcaac366	-0.00134015	0.037688145	0.971685826
leucine.lysopcac181	-0.001351619	0.038054063	0.97171808
lysopcac280.pcaac425	-0.003341165	0.094248214	0.971771957
acetylornithoine.pcaac422	-0.001758676	0.049653164	0.971797013
pcaec422.pcaac401	6.93E-05	0.001959044	0.971845223
c51dc.smc260	-0.000903037	0.02556929	0.971878184
pcaac383.pcaec362	-0.00045707	0.012978325	0.971957211
pcaec365.asparagine	-0.000258823	0.007355779	0.971982393
pcaec443.lysopcac181	0.001748593	0.049779925	0.972030016
putrescine.pcaac300	0.001147017	0.032663591	0.972038308
pcaac281.pcaec421	-4.00E-05	0.001138027	0.972040504
pcaac425.c3	0.00169785	0.04842152	0.972079785
serotonin.pcaac363	-0.00028738	0.008200217	0.972094533
c4.pcaec402	-0.001534505	0.043915591	0.97217673
smc260.c10	5.82E-05	0.001668051	0.972230839
pcaac341.pcaec421	-9.10E-05	0.002611102	0.972242421
pcaac405.pcaec423	-0.000104445	0.003004535	0.972319722
creatinine.pcaec423	0.000260557	0.007503928	0.972351354
pcaec422.lysopcac204	0.001208608	0.034912041	0.972434211
pcaec422.smc202	-0.001406881	0.040656304	0.972445682
pcaec446.smohc241	-0.002423019	0.070078416	0.972468306
histidine.pcaac401	-2.10E-05	0.000607646	0.972535045
c10.pcaac385	0.000550799	0.015985237	0.972563092
pcaac406.c4	0.000787742	0.022939053	0.972655509
lysopcac280.smohc141	-0.000429969	0.012521297	0.972656817
pcaec403.c141	0.001235143	0.035993199	0.972675102

h1.pcaec425	6.54E-05	0.001907964	0.972713142
serotonin.lysopcac280	-0.000534661	0.015609575	0.972725986
pcaac366.pcaec420	0.001059638	0.031118441	0.972885439
lysopcac161.serine	-0.001358451	0.039964337	0.972933333
pcaec343.pcaac424	-0.000303807	0.008944999	0.972955381
methionine.smohc161	0.000844423	0.024862961	0.972956002
pcaec423.putrescine	0.001277005	0.0376241	0.972973494
creatinine.lysine	0.002078657	0.06128628	0.97299257
pcaac341.pcaec386	0.00018326	0.005412489	0.973039071
pcaac424.pcaec382	-0.001293545	0.038231691	0.97305847
pcaec340.pcaac401	5.92E-05	0.001751892	0.973074582
ornithine.pcaac366	0.000427659	0.012672293	0.973127574
glutamicacid.pcaac322	-0.001117809	0.033140072	0.973141683
c2.pcaac401	-5.60E-05	0.001660978	0.97314726
pcaec402.pcaac420	0.004550331	0.135070641	0.973174561
pcaac384.pcaac344	-0.002082848	0.061906676	0.973209235
pcaac380.pcaec301	0.002072341	0.061597916	0.973210774
pcaec444.pcaac344	-0.001764674	0.052515172	0.973242538
c0.glutamicacid	0.001011274	0.030113895	0.973259645
pcaac281.smc160	-0.00316239	0.094180879	0.973262666
smohc141.c51	-0.000921488	0.027496962	0.973314778
pcaec445.smc180	0.000420351	0.012548939	0.973327077
smohc161.pcaec444	0.002984764	0.089637132	0.973485174
pcaac381.valine	0.00079095	0.023790972	0.973526974
pcaac323.c10	9.06E-05	0.002729055	0.97355382
lysopcac161.smohc141	-0.001397331	0.042194481	0.973629932
pcaec404.pcaac361	-0.001388519	0.042001934	0.973676079
c162.pcaec340	-0.000682002	0.020642405	0.973691668
smohc221.pcaac343	0.000556192	0.016891257	0.973780074
pcaec340.threonine	0.000714653	0.021783946	0.97387672
asparagine.c3	-0.001334667	0.040806065	0.973955403
pcaec424.c182	-0.00103428	0.031784023	0.974088087
pcaec341.c18	0.000756621	0.023477823	0.974337897
pcaac366.c10	9.79E-05	0.003042424	0.974374892
alanine.pcaec382	-0.000646511	0.020110017	0.97440027
pcaac341.pcaec402	0.001201847	0.037482977	0.974467837
pcaec360.c10	7.52E-05	0.002347202	0.974477152
smohc221.smc180	-0.000380244	0.011877101	0.9745068
pcaac421.pcaac401	-0.00013854	0.004339645	0.974578865
pcaac361.pcaec386	-0.000139702	0.00438896	0.974653689
pcaac404.pcaac420	0.001736751	0.054598747	0.974670378
glycine.pcaac401	-0.000120157	0.003779395	0.974683582
pcaac386.c10	7.47E-05	0.002357903	0.974763271
lysopcac161.pcaec380	-0.001535064	0.048556638	0.974825979
pcaec364.c162	0.002367246	0.07494449	0.974847656

pcaec405.alanine	0.000408554	0.012944392	0.974867076
pcaec382.smohc241	-0.002120493	0.067197046	0.974871787
phenylalanine.pcaac320	-0.001599231	0.050699527	0.974882157
lysopcac281.c3	0.00071848	0.022800303	0.974907204
pcaec422.phenylalanine	-0.001312763	0.041683249	0.974921592
lysopcac182.glutamicacid	-0.001261353	0.040065321	0.974930628
pcaac362.c10	7.40E-05	0.002353136	0.974953502
c18.serotonin	-0.002379244	0.07586302	0.975026195
pcaec423.smc180	-0.000162067	0.005167995	0.975028355
pcaac422.c10	7.29E-05	0.002325228	0.975035825
lysopcac181.pcaec401	-0.001389445	0.044342302	0.975048357
pcaec444.pcaac426	0.001982074	0.063263704	0.975051689
c4.asymmetricdimethylarginine	-0.000673063	0.021486006	0.975055441
pcaec385.pcaec384	0.001238307	0.039568992	0.975079917
lysopcac170.asparagine	0.000434003	0.013894073	0.975126353
phenylalanine.c16	0.000983258	0.031510819	0.9751524
isoleucine.pcaac364	-0.000842041	0.026993024	0.975159608
pcaec401.pcaac380	-0.001632571	0.052508589	0.975241816
c141.pcaec341	0.001383706	0.044550996	0.975267754
smc160.c142	-0.002046201	0.065886943	0.97526988
smohc141.pcaac364	0.001424445	0.045986589	0.975334376
pcaec343.pcaac383	0.000123012	0.003971864	0.975337866
alanine.pcaec403	0.000976447	0.031572452	0.975372613
pcaec420.lysopcac182	0.001077212	0.034889451	0.97541415
c0.pcaac401	3.94E-05	0.001277089	0.975464071
smc180.glutamine	0.000366525	0.011932684	0.97554067
pcaec424.pcaac322	0.001678526	0.054660668	0.975547017
pcaec365.pcaac364	-0.001643754	0.053559535	0.975561247
pcaac342.pcaac424	-0.000528129	0.017210434	0.975564187
smc202.pcaec422	-0.000614328	0.020035808	0.975584116
pcaac402.c8	-0.001356051	0.044311626	0.975630999
lysopcac281.c182	0.000891262	0.029155427	0.97565748
pcaec300.pcaac320	0.002504222	0.081981598	0.975675947
pcaec340.c3	0.000669343	0.022007226	0.975780575
smohc141.c182	-0.000747253	0.024612857	0.975823919
pcaec443.smc180	-0.000143387	0.004724521	0.975832378
pcaec363.pcaac401	-5.17E-05	0.001703884	0.97583995
pcaac422.lysopcac160	-0.001228876	0.040511309	0.975844713
lysine.pcaec380	-0.001365928	0.045074051	0.97586863
pcaec383.c14	0.001748178	0.057687884	0.975868651
pcaac362.taurine	0.001337456	0.044373958	0.975998811
smc241.pcaac364	0.001282889	0.042578551	0.976007268
c182.pcaec363	0.000313738	0.010440384	0.976070586
lysopcac280.pcaac401	5.47E-05	0.001825568	0.976118345
kynurenine.pcaec301	-0.001630773	0.054622749	0.97622599

pcaac281.pcaac364	-0.001286752	0.04323814	0.976302044
pcaec342.pcaac383	0.000395471	0.013289366	0.976303001
ornithine.pcaec420	-0.000872541	0.029332501	0.976312461
phenylalanine.isoleucine	0.000479971	0.016194449	0.976398896
pcaec444.pcaac401	-7.20E-05	0.002441555	0.976504289
c2.c16	0.001779022	0.060499229	0.976583826
pcaec424.c10	5.08E-05	0.001730261	0.976604099
kynurenine.pcaec385	0.001082852	0.036948623	0.976662412
pcaec365.pcaec401	-0.001143251	0.039108862	0.976721672
c101.pcaac363	-0.000314863	0.010813253	0.976812643
c162.c16	0.001804746	0.061989401	0.976816207
pcaac343.pcaec402	0.000612784	0.021085788	0.976857851
pcaac384.c101	0.000123114	0.004239285	0.976873993
lysopcac161.pcaec385	-0.000771227	0.026889034	0.977160081
pcaec381.pcaec423	0.000404835	0.014161022	0.977234812
pcaec343.lysopcac280	-0.000198791	0.006960708	0.977257835
citruline.pcaac381	0.001172773	0.041205443	0.977335404
smohc221.pcaec382	-0.000912531	0.032090799	0.977355868
pcaec444.c10	4.06E-05	0.001433695	0.977470417
acetylornithoine.creatinine	-0.000148872	0.005267367	0.977493444
h1.methionine	0.000287624	0.010195861	0.977535801
pcaac386.pcaac362	0.000634116	0.022571521	0.977628373
c141.pcaec423	-0.000185606	0.006616952	0.977662984
pcaec364.pcaec386	0.000522085	0.018620172	0.977672084
c181.pcaac360	0.000588632	0.02101161	0.977691261
pcaec383.pcaac380	-0.001794061	0.064164266	0.977734399
pcaec301.smohc241	-0.000650745	0.023285193	0.977745317
glutamine.tyrosine	-0.000993773	0.035591673	0.977765384
pcaec403.pcaec420	0.000798013	0.028586426	0.977769921
c5.spermidine	0.001474888	0.052868115	0.977784503
leucine.pcaec362	-0.000271463	0.009770926	0.977875862
pcaec340.smohc241	0.001732922	0.062393644	0.977882812
pcaac401.pcaac421	0.001842758	0.066432264	0.977910768
h1.smc180	0.000476542	0.017186291	0.977919418
pcaac343.c14	0.000964398	0.034786935	0.977923422
pcaac363.pcaac401	-5.65E-05	0.00204019	0.977941177
serine.c101	0.000306124	0.011076187	0.977991103
smohc221.pcaac383	-0.00012025	0.004352669	0.978000046
leucine.c16	0.001141916	0.04144453	0.978058864
pcaec402.pcaac365	0.00134303	0.04875378	0.978063377
pcaac344.pcaac424	0.000235242	0.008541443	0.978068105
pcaec444.glutamicacid	0.001363876	0.049622556	0.978112895
smohc222.pcaec343	-0.001864622	0.067900377	0.978131887
smc241.glycine	-0.000659355	0.024069015	0.978185053
pcaec425.pcaac403	0.000124031	0.00453235	0.978207791

asparticacid.c12	-0.001848858	0.067608454	0.978223124
arginine.threonine	0.000494215	0.018079947	0.978232334
pcaac363.c12	0.001023879	0.037512556	0.978264727
pcaec424.threonine	-0.000598973	0.02199116	0.97831035
serotonin.c51	-0.002381562	0.087759334	0.978389619
pcaac420.pcaac384	-0.000941902	0.03483017	0.978465028
smohc161.pcaac424	0.00021057	0.00778847	0.978470302
pcaac364.pcaac360	-0.0005082	0.018809254	0.9784842
pcaac381.asymmetricdimethylarginin	-0.000500671	0.018546983	0.978503204
lysopcac281.isoleucine	0.000432886	0.016068166	0.978546331
pcaec445.smc181	0.000451093	0.016773524	0.978584065
pcaac386.pcaec385	0.001357558	0.050681119	0.978669182
pcaec363.pcaec420	0.000556361	0.020795998	0.978695455
smc260.pcaac320	-0.00161287	0.06040928	0.978738627
pcaec404.c10	6.89E-05	0.002586204	0.97877568
pcaac383.lysopcac281	0.000208806	0.007849154	0.978815634
pcaec343.glycine	0.001023929	0.038495919	0.978818763
pcaec443.putrescine	0.001149622	0.043242345	0.978828967
pcaec382.pcaac361	-0.001376003	0.052167113	0.978995159
prolinr.pcaac320	-0.000651605	0.024745387	0.979030541
methionine.pcaac363	0.000285146	0.010895007	0.979158108
pcaec382.pcaec420	-0.00068703	0.026336928	0.979226556
smc180.c10	4.13E-05	0.00159232	0.979322658
putrescine.c142	0.001078225	0.041594016	0.979356792
pcaec446.lysopcac181	0.001908034	0.073745991	0.979396241
pcaac380.pcaac420	0.001890521	0.07340124	0.979489446
smc240.pcaac364	-0.001106468	0.042974721	0.979496635
smc260.lysopcac182	0.000260075	0.010124577	0.97954401
c14.c51dc	-0.002245647	0.087609611	0.979587823
pcaec404.lysopcac181	0.001420791	0.055583411	0.979644348
glutamine.pcaec402	0.001236703	0.048434143	0.979666425
pcaac386.c8	0.001052598	0.041258299	0.979683388
pcaec321.c10	4.01E-05	0.001573877	0.979722022
pcaac420.lysopcac181	-0.001064954	0.041859347	0.979740038
c10.lysopcac140	-3.02E-05	0.001188077	0.979747642
pcaac361.pcaec402	0.000583563	0.022972613	0.979770856
c5.pcaac364	-0.000908115	0.035756441	0.979775077
pcaec301.pcaec343	-0.001371111	0.054015067	0.979785725
smohc141.c10	4.07E-05	0.001604555	0.979800302
pcaec401.pcaac404	0.00018484	0.007305515	0.979851358
pcaac323.histidine	0.001090968	0.043139303	0.979860903
smc181.c3	0.000361187	0.014410891	0.98004081
pcaac361.c10	5.46E-05	0.002187306	0.980116916
lysopcac170.pcaac401	-7.12E-05	0.002864032	0.980197961
pcaec360.pcaac281	-3.86E-05	0.001554851	0.98024387

lysopcac281.pcaac365	0.001336325	0.05387934	0.980248837
c18.pcaec446	0.000706223	0.028477653	0.980251211
pcaac323.smc240	0.000714451	0.028831678	0.980266441
c18.pcaec343	0.001368702	0.055283907	0.980284258
lysopcac180.pcaac426	-0.001174823	0.047676311	0.980376646
smc241.c10	3.32E-05	0.001349932	0.980402238
pcaec406.pcaac404	0.000121084	0.004929585	0.980439525
c181.pcaac401	-3.66E-05	0.001493705	0.980471412
pcaac320.pcaac401	3.81E-05	0.001557386	0.980496199
ornithine.c10	0.000221931	0.009081111	0.980538162
c141.pcaac401	-4.27E-05	0.001750536	0.980566122
phenylalanine.c3	0.000638411	0.026187256	0.980586013
pcaec342.pcaec420	0.000380737	0.015698673	0.980686216
pcaec341.pcaec403	-0.001150583	0.04751043	0.980714376
pcaec443.pcaac240	0.000458811	0.018956827	0.980725943
smohc241.valine	0.000839249	0.034726149	0.980754057
c101.pcaac401	-5.28E-05	0.00218571	0.980764435
pcaec384.c51	0.000528205	0.021886385	0.98078087
pcaac420.lysopcac281	0.000202756	0.008408043	0.980796314
pcaac383.h1	-6.67E-05	0.00277699	0.980880871
c14.pcaac343	0.000531816	0.022277805	0.980989429
c0.pcaec363	-0.000213399	0.008962112	0.981037838
pcaac386.h1	5.71E-05	0.002407735	0.981101171
c16.putrescine	-0.00088021	0.037178052	0.981145867
pcaec405.c4	0.000326068	0.013778228	0.981153922
pcaac383.c10	4.17E-05	0.001765377	0.981175929
alanine.pcaac401	-4.90E-05	0.002074957	0.981201877
c181.pcaac384	0.001225346	0.051912943	0.981202942
pcaac384.putrescine	0.000599132	0.025396417	0.981213018
pcaac343.pcaec420	-0.000662087	0.028131453	0.981257389
pcaac344.pcaac401	-0.000123671	0.005266545	0.981299593
pcaec301.pcaac424	-0.000142564	0.006076954	0.981317661
phenylalanine.pcaec342	0.001686163	0.071962824	0.981340544
lysopcac170.pcaac300	-0.000965051	0.041324319	0.981402603
pcaec360.smc202	0.000859301	0.036997252	0.981503729
creatinine.pcaac401	-1.04E-05	0.000449799	0.981556466
smohc221.c10	3.84E-05	0.001657942	0.981563769
pcaec343.glutamicacid	-0.000926327	0.040122188	0.981613959
pcaec420.pcaec401	-0.001169222	0.050691858	0.981631758
c141.c142	-0.001153495	0.050048314	0.981645812
pcaac343.pcaac363	-0.000209314	0.009091586	0.981665594
pcaac425.c10	5.64E-05	0.002457137	0.981711291
pcaec423.c10	4.26E-05	0.001858093	0.981722974
pcaac383.smc240	-0.000718617	0.031358654	0.981750564
pcaec425.lysopcac203	-0.000469806	0.020532419	0.981778329

pcaec425.pcaac401	-4.88E-05	0.002140586	0.981830642
c182.isoleucine	0.000470831	0.020718409	0.981902511
acetylornithoine.c10	-8.37E-05	0.003687643	0.981924232
pcaec365.lysopcac182	-0.000444415	0.019617734	0.981959433
methioninesulfoxide.c10	0.000111803	0.00493643	0.981963605
pcaec361.ornithine	-0.001251567	0.055532119	0.982051821
pcaac404.pcaac403	-0.000117616	0.00522623	0.982077954
smohc141.pcaac360	0.000993957	0.044211225	0.982096176
lysopcac160.pcaac385	0.000575074	0.025582484	0.982098409
pcaec381.c10	7.78E-05	0.003467264	0.982125841
lysopcac181.pcaec406	0.000216756	0.009666599	0.982143038
pcaac281.c18	-0.000486583	0.02184382	0.982260579
smohc161.pcaac401	4.13E-05	0.001861401	0.98233114
smohc241.c3	0.000368367	0.016619674	0.982348992
pcaac425.putrescine	0.000605969	0.027417442	0.982399072
c18.pcaac383	-8.10E-05	0.003665104	0.982407317
pcaac420.c10	3.72E-05	0.001683077	0.982408345
pcaec361.pcaec422	-0.000691264	0.031296495	0.982410218
h1.pcaac401	-3.49E-05	0.001581429	0.982423199
pcaac406.c10	5.78E-05	0.002645324	0.982608991
lysopcac180.pcaec406	-0.000315862	0.014473941	0.982621036
pcaec384.c10	3.44E-05	0.001584577	0.982698811
pcaac401.c12	-0.000941563	0.04344579	0.982741032
pcaac365.c142	-0.000431185	0.019909255	0.982752646
pcaec402.c10	4.21E-05	0.001945751	0.982779617
c2.acetylornithoine	-0.000719531	0.033310348	0.982797804
pcaac342.smc260	-0.000496764	0.023069241	0.98285134
acetylornithoine.h1	0.000189767	0.008818431	0.982862684
pcaac366.c142	-0.000593752	0.027605089	0.982871096
pcaec361.pcaec401	-0.002255952	0.104895013	0.982872699
smohc161.glycine	-0.000667004	0.03104287	0.982888802
lysopcac180.pcaac401	5.36E-05	0.002495521	0.982891997
pcaec383.c182	0.000796405	0.037223655	0.982961582
smohc161.c16	-0.001194719	0.055927187	0.982987937
pcaec402.histidine	-0.001376589	0.064480433	0.982998363
c142.pcaec423	0.000248876	0.011673385	0.983021424
pcaac321.pcaec384	0.001018454	0.047808722	0.983035204
pcaec401.c10	4.99E-05	0.002343145	0.983047866
pcaec422.smc180	-9.75E-05	0.004583058	0.983049906
pcaec401.pcaec420	0.0003607	0.017005445	0.983108296
pcaac386.smohc241	-0.001080598	0.050952095	0.9831105
lysopcac280.smohc241	-0.000864158	0.040830915	0.983145376
pcaec363.pcaec365	0.000481352	0.022795944	0.98318409
histidine.c141oh	0.001861529	0.088191199	0.983190316
c0.pcaac323	0.001424646	0.067703755	0.983242495

c2.pcaec421	-0.000180042	0.00856037	0.983250694
c8.c14	0.000873894	0.04156086	0.983254838
pcaac363.pcaac300	-0.001773293	0.08436491	0.983260822
putrescine.pcaac401	2.65E-05	0.001261435	0.983290166
lysopcac182.lysopcac281	0.000224741	0.010717857	0.98330102
pcaec341.pcaec444	-0.002080526	0.099257835	0.983307407
pcaac362.lysopcac182	-0.00021449	0.010239404	0.983318018
citruline.h1	5.05E-05	0.002416258	0.98334725
c102.isoleucine	-0.000241454	0.011551764	0.983354305
kynurenine.pcaac426	0.0009365	0.044824893	0.983361887
pcaac362.smc181	-0.000470992	0.022603555	0.983405956
smc240.c10	3.06E-05	0.001473843	0.983473595
c8.pcaac360	-0.000559749	0.027014276	0.983498763
pcaec424.pcaac384	-0.001023058	0.049647807	0.983589686
methioninesulfoxide.pcaec384	-0.000572304	0.027805583	0.983608769
pcaec340.prolinr	-0.000843179	0.0410215	0.983630891
smc160.threonine	-0.000873662	0.042680663	0.983698429
pcaec446.c10	3.16E-05	0.001549223	0.983757253
pcaac363.c14	-0.000551583	0.027070552	0.983773256
pcaec362.pcaac363	0.000315726	0.015502114	0.983780502
lysopcac182.pcaac344	0.000796701	0.039159562	0.983797759
pcaac342.pcaec343	0.001492692	0.073454369	0.983816594
pcaec342.c10	3.18E-05	0.001572762	0.983895886
pcaec444.pcaac363	0.000237301	0.011781186	0.983959081
creatinine.pcaec361	-0.00018749	0.009312041	0.983965665
pcaec444.methioninesulfoxide	-0.000282687	0.01409409	0.984026986
pcaac406.smc181	0.000343415	0.017129045	0.984033713
smc180.smohc241	-0.001567561	0.078218321	0.984039952
c18.alanine	0.000216182	0.010833837	0.984108848
pcaec380.pcaec384	-0.000431391	0.021625276	0.984113493
asparagine.pcaac240	-0.000317106	0.015944407	0.984161471
ornithine.pcaac401	-2.36E-05	0.001188313	0.984215066
smohc241.smc160	0.001524436	0.077337603	0.984302239
pcaec380.pcaac426	-0.001630311	0.082777981	0.984315345
pcaac320.smc202	0.000606668	0.030846668	0.98433746
c141.lysopcac281	0.000166976	0.008499505	0.984354876
lysopcac160.pcaec406	0.000262462	0.013457163	0.984467827
pcaec384.taurine	-0.000731038	0.037527015	0.984486283
pcaec444.pcaec341	-0.000744773	0.038320669	0.98452214
pcaec361.c10	3.32E-05	0.001711188	0.984530169
smc160.pcaec422	0.000413271	0.021278615	0.984532814
smc260.pcaac403	0.00033611	0.017490386	0.98469607
c142.c12	-0.001596472	0.083143194	0.984708322
pcaac420.pcaac401	0.000102548	0.005342954	0.984715
putrescine.c3	0.000305503	0.015963621	0.984759303

smohc141.threonine	0.000675991	0.035368009	0.984778722
putrescine.threonine	0.00034542	0.018072983	0.984779156
pcaec321.pcaac364	0.000809475	0.04252995	0.984842422
pcaec420.c10	3.23E-05	0.001696908	0.984854949
kynurenine.pcaac401	3.87E-05	0.002033856	0.98485635
smohc161.c51dc	0.00182964	0.096234482	0.984858942
pcaec383.c10	5.57E-05	0.002937639	0.984909003
smohc161.smc180	-0.000505779	0.026759332	0.984947566
lysopcac161.pcaac401	-2.52E-05	0.001337111	0.984964488
pcaec362.c10	3.23E-05	0.001712872	0.984999929
pcaac364.pcaec403	-0.001210698	0.064315968	0.98500872
lysopcac180.smc202	0.0005827	0.030971505	0.985016797
pcaec343.pcaec340	0.001129013	0.060039236	0.985024351
phenylalanine.pcaac361	-0.000911383	0.048616011	0.985070565
pcaec365.smohc141	-0.001001839	0.053499515	0.985086819
smohc241.pcaec386	0.000106996	0.00572072	0.985105036
asymmetricdimethylarginine.lysopca	-0.000202547	0.010857679	0.985143718
pcaec365.glutamicacid	0.000708071	0.038009215	0.985164232
isoleucine.pcaac426	0.000853476	0.045840256	0.985172547
pcaac240.creatinine	-0.00010166	0.005473233	0.985207878
c8.glutamicacid	-0.000856738	0.04636322	0.985283753
pcaac401.c162	-0.001313676	0.071190178	0.98530428
pcaec360.pcaac384	-0.001237677	0.067137435	0.985318672
pcaac300.pcaec362	0.000349006	0.018956898	0.985338171
smc180.c14	0.00044089	0.024005556	0.985373458
c102.pcaac424	0.000177053	0.009656891	0.985398753
smohc221.pcaac401	-8.09E-05	0.004412928	0.985401812
pcaac365.pcaac404	-0.000135871	0.007431191	0.985438997
smc180.pcaac401	2.82E-05	0.001544284	0.985442331
pcaec423.pcaec380	-0.001522644	0.083442466	0.985467698
smc180.pcaec425	0.000107643	0.005935953	0.985558319
pcaec406.c10	3.52E-05	0.001942165	0.985560273
c102.pcaac380	-0.001085547	0.05991643	0.985571324
c182.smohc161	0.000116749	0.006455253	0.985596661
pcaac383.pcaec340	0.000835407	0.046278608	0.985623884
pcaec382.c10	6.42E-05	0.003565872	0.985659131
c51dc.pcaac385	-0.000301595	0.016773741	0.98568081
pcaac342.pcaac322	0.001209349	0.06733024	0.985695731
pcaac403.alanine	-0.000227443	0.012689322	0.985725582
pcaec446.h1	-0.000108529	0.006086666	0.985799939
pcaac406.pcaec383	-0.001363289	0.076461276	0.98580057
pcaac364.pcaac401	-3.02E-05	0.001696753	0.985844119
pcaec421.c10	5.21E-05	0.002935794	0.985858964
pcaac402.c142	0.000476702	0.026892337	0.985882982
pcaec421.valine	-0.000446191	0.025181753	0.985888949

pcaac281.pcaec380	-0.000584896	0.033081944	0.985919672
pcaec421.pcaec386	0.000146663	0.008308417	0.985941865
pcaec443.pcaec363	-0.000491195	0.027867291	0.985962686
pcaec380.c10	5.20E-05	0.002950572	0.985974204
asymmetricdimethylarginine.pcaac4(	-1.66E-05	0.000941959	0.985990047
methionine.ornithine	0.000691327	0.039353876	0.986009894
asparticacid.creatinine	0.000609506	0.034708098	0.986014685
pcaec340.pcaac344	0.001226832	0.070094864	0.986061239
methioninesulfoxide.pcaec420	0.001233883	0.070517544	0.98606516
acetylornithoine.pcaec385	-0.000969667	0.055915927	0.986189389
c142.c141oh	0.001975453	0.114098297	0.98621162
pcaac361.pcaec423	5.86E-05	0.003390507	0.986225827
pcaac281.pcaec362	0.000373278	0.021592721	0.986232636
ornithine.smc202	0.000588711	0.034063913	0.986236365
pcaec405.pcaac383	-9.25E-05	0.00535394	0.986240634
pcaac381.smohc141	0.000438395	0.025428191	0.986269831
smc260.pcaec444	-0.00175586	0.101859646	0.986271796
pcaec361.pcaac401	2.90E-05	0.001681045	0.986273112
pcaec445.valine	-0.000785202	0.045558261	0.986274116
smc181.pcaec384	0.000614845	0.036082645	0.986429568
pcaac381.pcaac362	0.000395755	0.023246097	0.986441782
pcaec385.c10	3.46E-05	0.00203473	0.986446225
pcaec340.lysopcac280	0.000133589	0.007859392	0.986463432
pcaac401.ornithine	-0.000516075	0.030504366	0.986526592
pcaac380.pcaac401	5.51E-05	0.003278416	0.986606202
pcaac404.c8	-0.000537505	0.031980581	0.986614849
c12.pcaec361	-0.000147878	0.008799857	0.986616995
pcaec361.c18	0.000489277	0.029154754	0.986634893
c10.pcaac425	-0.001093481	0.065423976	0.986689279
pcaec364.pcaac405	-0.000585083	0.035235378	0.986775912
c5.pcaac401	5.38E-05	0.00324349	0.986780577
pcaac344.lysopcac160	-0.000319314	0.019243844	0.986785423
smc202.c3	0.000257369	0.015559398	0.98682682
pcaac323.pcaac401	3.85E-05	0.00233165	0.986833801
c101.putrescine	0.000481048	0.029354965	0.986949271
c182.pcaac424	-7.49E-05	0.004583274	0.986990768
serine.creatinine	0.000281838	0.017257686	0.986993972
pcaac361.c3	0.000447482	0.027426133	0.987006114
pcaec406.smc202	-0.000625839	0.038531153	0.987064634
c102.pcaac364	-0.000787949	0.048667327	0.987105952
leucine.pcaac426	-0.000525064	0.032502265	0.987134475
smc180.lysopcac203	0.000193167	0.011971966	0.987150223
lysopcac203.h1	-7.36E-05	0.004587772	0.987224239
smohc222.pcaec402	-0.000684589	0.042777651	0.987254943
pcaac320.pcaec421	2.52E-05	0.001574597	0.987264502

pcaac402.pcaec383	0.003032931	0.189981771	0.987286079
lysopcac203.taurine	0.000815982	0.051241892	0.987318098
pcaac323.pcaac424	0.000118229	0.007484148	0.98741911
lysopcac161.pcaac422	-0.001063161	0.067674626	0.987488697
pcaec342.pcaac401	-3.06E-05	0.001950524	0.987495569
pcaec422.pcaec341	0.000380013	0.024236594	0.987513043
lysine.pcaec385	-0.00063745	0.040668833	0.98751715
isoleucine.glutamine	-0.000318676	0.020428848	0.987576759
pcaec364.c10	2.87E-05	0.001843686	0.987594847
c18.methioninesulfoxide	-0.000537287	0.034581942	0.987626667
pcaec420.c8	-0.000693614	0.044753844	0.987657098
kynurenine.acetylornithoine	0.000764493	0.049331035	0.987658064
pcaec343.c10	-3.17E-05	0.002046824	0.987660177
arginine.taurine	-0.000350743	0.022808301	0.987753089
pcaec365.c51	-0.000300495	0.019653308	0.987823221
lysopcac280.c10	-3.21E-05	0.002111833	0.987884667
c182.asparagine	-0.000142142	0.009352368	0.987895954
pcaec361.spermidine	-0.0007905	0.052030871	0.987900385
pcaac341.c142	0.000393344	0.02594912	0.98792797
pcaec342.pcaec386	-0.000137751	0.009142388	0.988000395
pcaec361.acetylornithoine	-0.000522542	0.034793971	0.988039523
lysopcac281.pcaec423	5.52E-05	0.003691562	0.988085028
pcaac366.smc202	0.000666095	0.044570223	0.988097922
pcaec382.pcaac401	-5.36E-05	0.003589314	0.988115078
pcaac403.c102	0.000578066	0.038791078	0.988132021
lysopcac182.pcaac322	-0.001099406	0.073861493	0.988145825
pcaac362.pcaec386	0.000116995	0.007957323	0.988290674
pcaec382.serotonin	0.001339612	0.09116379	0.988297231
pcaec424.histidine	0.000923253	0.062863928	0.988303625
smohc161.methionine	-0.000200896	0.013821317	0.988424092
pcaac281.c2	-0.000745668	0.05143166	0.988453584
lysopcac181.isoleucine	-0.000631509	0.04356322	0.988455062
c182.c12	0.000643528	0.044492471	0.988481033
pcaac240.histidine	-0.00099052	0.068624365	0.988504783
phenylalanine.pcaec402	-0.000716916	0.049701191	0.98851228
asparticacid.lysopcac204	-0.000545492	0.038106095	0.988599424
smohc221.c3	0.000273032	0.019400516	0.988791881
lysopcac181.pcaac384	0.000638645	0.045477144	0.988815944
pcaac384.c10	3.44E-05	0.002453708	0.988820067
methionine.phenylalanine	0.000416904	0.029709043	0.988824161
pcaac360.pcaec446	0.00076191	0.054417635	0.988849429
pcaec362.pcaac360	-0.000474502	0.033931072	0.988862846
smohc241.pcaec383	0.000631024	0.045293322	0.988904544
smc160.c10	1.84E-05	0.001318063	0.988904837
lysopcac204.pcaec446	-0.000677555	0.0486978	0.988919262

lysopcac204.pcaec423	0.00016745	0.012111458	0.988989131
pcaac401.pcaac342	0.000292378	0.021272755	0.98905403
pcaac342.pcaac364	-0.000995892	0.072714723	0.989092542
smohc221.lysopcac160	0.00056167	0.041097815	0.989115793
pcaec423.pcaac401	4.18E-05	0.003069731	0.989157286
c16.pcaec340	0.000766695	0.056455457	0.989184396
pcaac366.threonine	-0.000351053	0.025890739	0.989201537
pcaec402.pcaac401	3.17E-05	0.002343234	0.989213056
c12.kynurenine	8.44E-05	0.006241365	0.989234714
lysopcac181.pcaec386	-0.000265029	0.019723187	0.989298361
phenylalanine.c182	-0.000481	0.035885794	0.989325277
smohc241.pcaac364	0.000475876	0.035695226	0.989382606
c102.pcaac401	-3.70E-05	0.002780869	0.989404762
glycine.pcaec362	-0.000171805	0.012937199	0.989423799
pcaec443.c10	4.25E-05	0.003222012	0.989498162
pcaec406.smc260	-0.000522859	0.039694638	0.989509699
pcaec381.pcaac384	-0.000640809	0.04920316	0.989627795
alphaaminoadipicacid.smohc241	-0.000461142	0.035492246	0.989652484
pcaec406.c51	0.000583944	0.044990953	0.989663314
pcaac403.c12	0.000676622	0.052284776	0.989693622
lysopcac281.ornithine	-0.000317797	0.024689144	0.989748681
c182.glutamine	0.000251419	0.019587321	0.989777463
pcaec322.pcaac320	0.001473311	0.115430225	0.989834923
pcaec421.threonine	0.000216229	0.017093832	0.989925789
smc202.c182	-0.000267399	0.021142418	0.98992741
pcaac320.smohc141	0.000370379	0.029334201	0.989944405
citruline.pcaac401	1.28E-05	0.001016016	0.990003482
pcaec301.c51dc	-0.001076436	0.085864462	0.990015858
pcaac281.c142	0.000510345	0.041119044	0.990115451
pcaec404.pcaec405	0.002945675	0.237692378	0.990130253
pcaac240.smc260	0.00031784	0.025725027	0.990160145
pcaac421.pcaac422	0.000827498	0.067126268	0.990182275
smc181.lysopcac203	0.000186766	0.015178151	0.990200249
serine.pcaac366	-0.000238826	0.019421503	0.990206562
pcaac364.pcaec380	-0.000620266	0.050458144	0.990209994
lysopcac281.pcaec301	0.000846786	0.069077195	0.990237176
pcaac321.pcaac401	2.58E-05	0.002102309	0.990237628
pcaac404.pcaac401	2.34E-05	0.001911579	0.990250055
pcaec401.threonine	0.000319885	0.026215744	0.990282177
pcaec446.pcaac401	-7.87E-05	0.006470855	0.990307969
smc160.lysopcac204	-0.000305212	0.025165679	0.990341067
smc181.pcaac401	1.74E-05	0.001440684	0.990365877
smohc161.lysopcac203	-0.000148179	0.012282643	0.990392044
pcaec422.c10	2.61E-05	0.002166059	0.990413562
pcaac406.lysopcac160	-0.000248632	0.020717233	0.990442111

methioninesulfoxide.pcaec340	-0.000416721	0.034959967	0.990506815
c2.serotonin	0.00091739	0.077008395	0.99051247
smohc141.pcaac240	-9.44E-05	0.007929791	0.990514298
pcaec365.c182	0.00030595	0.025819587	0.990562892
pcaec424.asparagine	-0.000146028	0.012340294	0.99057575
glutamine.pcaec386	-0.000164732	0.013926782	0.990579691
pcaec443.pcaac401	4.90E-05	0.004151232	0.990605059
lysopcac161.lysopcac281	-0.000114794	0.009741845	0.990615418
pcaac300.c8	-0.000438441	0.037230897	0.990621257
pcaec386.c10	2.76E-05	0.002347716	0.990647528
ornithine.smc180	0.000174791	0.015023069	0.990733868
pcaac341.pcaac424	5.36E-05	0.004623407	0.990764974
kynurenine.pcaac362	0.000148598	0.012898745	0.990825071
leucine.c182	0.000361894	0.03156445	0.990868945
smohc221.c142	0.000428721	0.03739542	0.990869512
pcaec383.pcaac320	-0.000883912	0.07714368	0.990874706
smohc161.c10	1.89E-05	0.001657825	0.990917707
pcaec421.pcaac401	-4.45E-05	0.003925682	0.990981492
c4.pcaac401	-3.17E-05	0.002816759	0.991024816
smc202.pcaac401	-1.55E-05	0.001374766	0.991047961
alanine.smohc241	0.000294506	0.026288636	0.991077942
c0.leucine	0.000377176	0.033767237	0.991104153
pcaac381.c18	-0.00059278	0.053186303	0.991123696
pcaec382.pcaac424	-0.000171583	0.01556085	0.991218263
pcaac360.c10	2.92E-05	0.002653937	0.991227027
isoleucine.c142	-0.000818363	0.074529569	0.991255075
pcaec385.pcaac401	-1.72E-05	0.001578015	0.991309051
lysopcac203.kynurenine	5.18E-05	0.004775871	0.991368247
ornithine.c51	-0.000340216	0.03147464	0.991391396
pcaec383.pcaac401	3.02E-05	0.002813804	0.991444402
pcaec402.smohc241	0.000585735	0.054587569	0.991454318
pcaec300.lysine	0.00052021	0.048531781	0.991463274
pcaac240.c162	0.000696233	0.065261809	0.991503601
pcaec300.asymmetricdimethylarginin	-0.000149414	0.014073059	0.991544424
pcaac384.pcaac360	0.000189854	0.018000351	0.991600037
pcaec340.smc240	-0.0002485	0.023580054	0.991606939
lysopcac161.methioninesulfoxide	0.000366856	0.034838787	0.991613658
pcaec443.methioninesulfoxide	-0.000162016	0.015425852	0.991635322
pcaec401.pcaac401	-2.76E-05	0.002657454	0.991740132
methionine.pcaac364	-0.000353817	0.034317164	0.991788792
pcaec402.lysopcac180	0.000535584	0.05241913	0.991862749
smohc221.pcaac364	0.000427292	0.041835086	0.991865627
pcaac240.valine	-0.000298125	0.029436578	0.991934145
lysine.pcaac401	-4.10E-06	0.000408348	0.992000386
pcaac240.c10	4.03E-05	0.004019277	0.992015154

c16.pcaac401	-1.95E-05	0.001943608	0.992028527
pcaac425.taurine	-0.000325962	0.032763158	0.992076406
pcaec386.pcaac401	2.10E-05	0.002115171	0.992094123
pcaac322.pcaac363	-0.000230115	0.023234236	0.992112195
pcaac384.lysopcac140	4.33E-05	0.004373827	0.992115234
smc180.threonine	-0.000273925	0.027681846	0.992119074
pcaec384.creatinine	-8.79E-05	0.00888023	0.992121199
c3.pcaac420	0.000355448	0.036257469	0.992192366
smc181.c182	0.000195392	0.019970762	0.992207951
pcaac380.smc240	0.000496639	0.051346994	0.992296881
asymmetricdimethylarginine.tyrosine	-0.000388834	0.040497466	0.99235324
lysopcac281.lysopcac280	-0.000110371	0.011517551	0.992368021
pcaec361.pcaac380	0.000509543	0.053661423	0.992437597
pcaac363.pcaec386	-0.000124216	0.013108226	0.992452984
pcaac404.pcaec384	-0.000283282	0.030335747	0.992562858
smc160.pcaac362	-0.000109505	0.011745265	0.992574714
pcaac425.pcaec341	-0.000294861	0.03178019	0.992610726
smc180.pcaac343	-0.000157962	0.017034405	0.992614713
spermidine.glutamine	-0.00034568	0.037571991	0.992672579
pcaec405.pcaec341	-0.0001865	0.020276688	0.992674747
pcaec386.pcaec343	0.000753617	0.082421497	0.992717979
pcaec384.pcaac401	-1.40E-05	0.001529596	0.992736132
c3.pcaac365	-0.000473548	0.052223852	0.992778345
lysopcac204.smc260	-0.000312297	0.034503901	0.992791571
smc160.c182	-0.000205599	0.022728849	0.992795821
asymmetricdimethylarginine.smc202	0.000294716	0.032759372	0.992835099
pcaac421.glycine	-0.00021227	0.023706429	0.992868779
pcaec402.pcaac424	-8.85E-05	0.009914555	0.992889313
smc160.pcaac401	-1.63E-05	0.001823432	0.992893882
pcaac421.c10	1.80E-05	0.002020612	0.99290493
pcaac403.phenylalanine	0.000542887	0.061424593	0.99296103
pcaac405.pcaac363	-0.000101515	0.011531305	0.992988752
pcaec363.c10	1.42E-05	0.001624349	0.993053872
lysopcac204.smc261	0.000288721	0.033161547	0.993065963
pcaec404.pcaec341	-0.000218461	0.025218767	0.993100894
pcaec360.pcaac401	1.67E-05	0.001935502	0.993108649
c18.lysopcac280	-0.000256879	0.029835319	0.993142919
pcaec384.pcaac404	-8.00E-05	0.009491333	0.993285931
pcaac402.c10	-3.74E-05	0.004441519	0.993296517
c3.spermidine	0.000435933	0.051888003	0.993308935
c182.pcaac404	2.45E-05	0.002921523	0.99332067
pcaac422.lysopcac182	-0.000578048	0.06896316	0.993324421
prolinr.pcaac401	-1.24E-05	0.001482235	0.993343019
c18.acetylornithoine	0.000282014	0.03381841	0.993358592
pcaec341.pcaac401	1.06E-05	0.001279723	0.993381345

pcaec425.prolinr	0.000126349	0.015242413	0.993398217
methionine.pcaac385	0.00020036	0.024241353	0.993417397
prolinr.h1	1.35E-05	0.001646515	0.993460603
c14.pcaec384	-0.000356143	0.043813034	0.993526125
pcaac320.c142	-0.000164218	0.020250163	0.993541443
putrescine.pcaac364	-0.000341031	0.042749023	0.993646535
pcaac240.c101	-9.53E-05	0.011960274	0.993653003
pcaec322.pcaec423	-5.98E-05	0.007549575	0.993690834
pcaec365.taurine	-0.000234439	0.029615707	0.993695495
c18.c182	-0.00042342	0.053529828	0.993700321
pcaec342.pcaec403	0.000263756	0.033439338	0.993718155
pcaac424.c10	3.19E-05	0.004048975	0.993733052
c142.pcaac424	4.57E-05	0.005841781	0.993772226
lysine.asparticacid	-0.000254004	0.032517766	0.993778969
pcaec403.pcaac401	2.69E-05	0.00347238	0.993836055
c0.valine	-0.000630633	0.08227465	0.993895451
serotonin.c141oh	-0.000705623	0.092263729	0.99390905
pcaac360.pcaac401	-2.00E-05	0.002621134	0.99392102
lysopcac140.pcaac401	-0.000103649	0.013688845	0.99396968
pcaac322.pcaec386	-5.42E-05	0.007165421	0.993981167
pcaac366.pcaac384	0.000334572	0.044396891	0.993998223
pcaec425.pcaac361	0.000351304	0.046772684	0.994018161
pcaac322.arginine	0.000176397	0.023944854	0.994132907
alphaaminoadipicacid.pcaac401	-7.58E-06	0.001029747	0.994138399
smc180.c12	0.000421294	0.057308291	0.994145212
pcaec340.lysine	0.00040105	0.054681985	0.994158855
pcaac343.pcaec365	-0.000163804	0.02237358	0.994169149
pcaec340.isoleucine	-0.000163153	0.022819329	0.994305757
pcaec443.pcaac424	-0.000437208	0.061576356	0.994345188
c18.lysopcac181	-0.000218083	0.030918809	0.994382493
pcaac320.pcaec343	-0.000613632	0.08744735	0.994411374
pcaec360.asymmetricdimethylarginir	-0.000414913	0.059305437	0.994428059
lysopcac203.c102	-0.000189548	0.027098862	0.994429282
pcaec421.glycine	-0.000129001	0.018746032	0.994519401
lysopcac281.pcaac386	-0.000128407	0.018860154	0.994577662
leucine.pcaac401	-3.94E-06	0.000587919	0.994661899
c182.pcaac383	2.41E-05	0.003608103	0.994688889
c141oh.c10	-3.19E-05	0.004815193	0.994727416
pcaec405.pcaac420	-0.000516527	0.078270616	0.9947442
pcaec365.pcaac401	-1.09E-05	0.001672203	0.994823806
smohc222.pcaec301	-0.000369337	0.056853717	0.994826216
pcaec360.pcaac386	-0.000251659	0.038916404	0.994849796
pcaec420.serotonin	-0.000487837	0.075448586	0.99485046
pcaac366.pcaac383	-9.10E-05	0.014223219	0.994903845
methioninesulfoxide.pcaac323	0.000737328	0.115279395	0.99490606

pcaec425.pcaec301	0.000314643	0.050901077	0.995076929
pcaac341.lysopcac161	0.000461673	0.074917413	0.995092096
isoleucine.glutamicacid	-0.00027641	0.045095723	0.995118393
methionine.pcaac420	-0.00025412	0.041892305	0.995168859
pcaec381.pcaac401	1.90E-05	0.00316824	0.995212716
pcaec365.smc261	0.000147358	0.024878867	0.995282747
pcaac403.asparagine	8.41E-05	0.014456545	0.995368777
pcaac381.spermidine	0.000299606	0.05152876	0.995369301
pcaac341.pcaac401	7.58E-06	0.001309448	0.9953867
phenylalanine.c10	-0.000106352	0.018391293	0.99539446
isoleucine.pcaac401	-3.34E-06	0.000588077	0.995474493
pcaec445.lysopcac181	-0.000309894	0.055036987	0.995515607
pcaec365.c10	-1.42E-05	0.002532449	0.995525215
smohc141.pcaec423	-4.75E-05	0.008516213	0.995559579
pcaec446.smc260	-0.000207396	0.037282972	0.995569676
pcaac381.pcaac363	0.000118757	0.021736984	0.995648834
pcaac366.pcaac380	-0.000223132	0.041092542	0.995675417
asparagine.isoleucine	-0.000101221	0.018649982	0.995677493
pcaec446.pcaec362	-6.94E-05	0.012892276	0.995711367
pcaac406.serine	-0.000372477	0.069180388	0.995711929
pcaec403.c10	2.19E-05	0.004077584	0.995731768
pcaec384.lysopcac280	4.03E-05	0.007576411	0.995768813
phenylalanine.methionine	-0.000153257	0.029822131	0.995907146
lysopcac170.pcaec403	0.000163965	0.032050828	0.995925654
smc181.pcaec301	-0.000302823	0.05936389	0.995937327
c51.pcaac424	3.34E-05	0.006605387	0.995968043
pcaac366.pcaec386	-8.67E-05	0.017390092	0.99602845
smohc222.pcaac401	1.67E-05	0.003368454	0.996044992
pcaec425.smc180	-3.79E-05	0.007844093	0.996147803
pcaec422.asymmetricdimethylarginin	8.52E-05	0.017857546	0.996198059
serine.c8	-0.000197924	0.043288435	0.996358573
pcaec363.methionine	0.000218607	0.050003524	0.99651816
pcaac323.smc160	-0.000329051	0.075411445	0.996524864
pcaac384.pcaac401	-8.34E-06	0.0019311	0.996562159
pcaac424.c182	0.000164123	0.038067326	0.996566302
pcaac240.pcaac401	1.52E-05	0.003562447	0.996610676
c18.ornithine	-0.000102443	0.024504678	0.996670507
creatinine.threonine	0.000127891	0.030631984	0.996674862
kynurenine.pcaec423	6.15E-05	0.014965031	0.996726964
c4.smc240	-0.000157335	0.039883962	0.996858237
pcaac362.pcaac401	-1.11E-05	0.002840345	0.996898503
h1.pcaec404	-9.46E-05	0.024611866	0.996939167
leucine.acetylornithoine	0.000166403	0.043515966	0.996954494
methionine.pcaec380	0.000141974	0.037279309	0.996966887
pcaec361.pcaac360	-0.000252089	0.066514621	0.996981557

serine.methionine	-8.30E-05	0.022202238	0.997023471
pcaec424.pcaac401	-8.07E-06	0.002192257	0.997067
pcaec443.pcaec384	-6.19E-05	0.016895647	0.997083931
leucine.pcaec404	9.02E-05	0.024659916	0.997086501
glutamicacid.pcaec404	-0.000161817	0.044374711	0.997095737
pcaac403.c10	1.06E-05	0.002918176	0.997105462
pcaec381.asymmetricdimethylarginir	6.84E-05	0.018910808	0.997120186
smc241.c162	0.000240659	0.066642875	0.997123962
arginine.pcaac342	-7.75E-05	0.02173441	0.99716109
c4.pcaac385	-9.01E-05	0.025306613	0.997163841
pcaac406.pcaac383	-2.93E-05	0.008390799	0.99722353
c18.c12	0.000139981	0.040369973	0.997238421
pcaac422.pcaec341	7.60E-05	0.021962301	0.997244778
lysopcac182.c12	-0.000187289	0.05477299	0.997276714
pcaac424.pcaac401	-1.21E-05	0.003562143	0.99729482
pcaec422.pcaec365	-8.30E-05	0.025384031	0.997394404
smohc241.pcaec423	-1.94E-05	0.005947739	0.997403307
pcaec425.methioninesulfoxide	-4.80E-05	0.015914196	0.997598738
pcaec322.pcaac401	-5.73E-06	0.001932385	0.997638569
pcaec300.smc241	-0.000169081	0.058166421	0.997684899
methioninesulfoxide.pcaec342	-0.000281113	0.098455188	0.997726006
pcaec404.smohc241	-0.000174225	0.061341724	0.997737959
c181.c3	-0.000106093	0.037446042	0.997743548
pcaec405.pcaac401	6.01E-06	0.002184291	0.997808762
serotonin.pcaac321	6.20E-05	0.022690702	0.99782419
serotonin.arginine	-6.07E-05	0.022374974	0.997840711
pcaec404.pcaac401	6.32E-06	0.002362426	0.997869503
alphaaminoadipicacid.pcaec401	-0.00016801	0.063776336	0.997901923
alanine.h1	-9.53E-06	0.003665405	0.997929338
ornithine.c18	0.000127723	0.049869672	0.997960236
citruline.putrescine	-7.60E-05	0.031970261	0.998107877
pcaec403.taurine	-0.00012693	0.05365997	0.998116081
pcaec381.c51dc	0.000255133	0.108531665	0.99812778
pcaac366.glutamicacid	-0.000132338	0.05640044	0.998131256
lysopcac280.pcaec365	-4.30E-05	0.019564449	0.998250859
pcaac420.phenylalanine	6.46E-05	0.030658165	0.998322672
smc241.pcaac365	-9.47E-05	0.046284811	0.998369922
pcaac425.pcaac401	9.57E-06	0.005036599	0.998486554
pcaec301.c51	7.82E-05	0.041685352	0.998505869
phenylalanine.pcaac401	2.39E-06	0.001276253	0.998507831
c16.h1	5.60E-06	0.003070315	0.99854839
smohc241.c10	2.60E-06	0.001446518	0.998570159
pcaec321.pcaac401	-2.50E-06	0.001405012	0.998585124
smc161.pcaac401	3.01E-06	0.001719445	0.998607776
c12.pcaac361	4.79E-05	0.027539464	0.998615853

pcaec424.methioninesulfoxide	1.95E-05	0.011395936	0.998635095
histidine.c51dc	-0.000155278	0.092381364	0.998661331
pcaac343.c3	-0.000142523	0.085678653	0.998675168
putrescine.pcaec322	5.68E-05	0.034860132	0.998702567
lysopcac280.pcaac342	3.49E-05	0.0221975	0.998747791
pcaac322.pcaac323	5.84E-05	0.038981582	0.998807207
pcaac344.valine	7.18E-05	0.05015625	0.998859309
pcaec364.asparticacid	6.65E-05	0.048061685	0.998897781
pcaec405.pcaac363	-1.93E-05	0.014348479	0.998931165
pcaec421.pcaec446	7.12E-05	0.055068975	0.998970034
lysopcac181.pcaec385	2.95E-05	0.023150448	0.998985908
c12.smohc161	-9.81E-06	0.008355036	0.999064648
pcaac361.methionine	3.29E-05	0.028078717	0.999066891
pcaec386.putrescine	4.65E-05	0.041621725	0.999109886
c16.pcaec421	-2.70E-06	0.002460125	0.999125237
arginine.pcaac401	-1.37E-06	0.00127573	0.999146587
pcaac401.c10	-2.30E-06	0.002185677	0.999163378
lysopcac281.alanine	1.14E-05	0.011916327	0.999236238
pcaec385.pcaec301	-5.78E-05	0.067385184	0.999316704
pcaec423.glycine	-1.77E-05	0.021588713	0.999347762
pcaec402.pcaac240	8.69E-06	0.011156396	0.999379613
smohc141.pcaec444	-7.41E-05	0.098446283	0.999400746
pcaec423.c12	3.17E-05	0.043006843	0.999413298
smc181.c162	6.96E-05	0.097250598	0.99943023
pcaec363.asymmetricdimethylargini	-2.49E-05	0.037917963	0.999476188
arginine.lysopcac281	4.99E-06	0.007791123	0.999489936
lysopcac160.pcaac401	-1.06E-06	0.001718851	0.999509388
pcaec364.pcaac401	7.39E-07	0.001387124	0.999575729
lysopcac160.smc202	1.67E-05	0.031827457	0.999582355
pcaec421.smc160	2.53E-05	0.048781104	0.999587427
prolinr.c51dc	5.84E-05	0.118919973	0.999609027
c2.pcaac321	9.88E-06	0.020729806	0.999620306
c142.putrescine	7.44E-06	0.021989696	0.999730667
pcaac380.c10	6.18E-07	0.00218091	0.999774203
pcaec404.c182	-7.32E-06	0.029444728	0.999801964
c18.pcaac401	-2.90E-07	0.001462188	0.999842031
c18.c51	3.93E-06	0.032986943	0.999905036
c51.pcaec386	-8.76E-07	0.010396339	0.999932895

Supplementary Table 2. Associations between the percentage of the medial cartilage volume loss and each of the metabolite ratios

Metabolite ratios	Beta	Std. Error	Pr(>|t|)
pcaec321.pcaec405	0.302519002	0.069397412	2.54E-05
pcaec322.pcaec405	0.205934731	0.049220712	5.09E-05
pcaac420.pcaec405	0.25811661	0.061894355	5.37E-05
pcaec341.pcaec405	0.17959897	0.043240085	5.73E-05
methioninesulfoxide.pcaac342	0.147306711	0.037263053	0.000123061
c181.pcaac342	-0.024209001	0.006271614	0.000173965
lysopcac280.pcaac342	0.108143804	0.028056834	0.000177617
pcaec422.pcaac342	0.06794374	0.017716006	0.000190681
c18.pcaac342	-0.062114018	0.016210641	0.000193132
pcaec300.pcaac342	0.062948912	0.016846733	0.000272965
pcaac424.pcaec405	-0.426528023	0.114956359	0.000299953
pcaec420.pcaac342	0.080496243	0.021870132	0.000333572
pcaac422.pcaac342	0.06741439	0.018411801	0.000357132
pcaec340.pcaec405	0.265525396	0.07272787	0.000370739
pcaac386.pcaac342	0.085750855	0.023607982	0.000396057
pcaec445.pcaec405	0.136071156	0.037485646	0.000399328
citruline.pcaac342	0.019275855	0.005317748	0.000406637
c51dc.pcaec405	0.157785356	0.043541505	0.000408108
pcaac323.pcaac342	-0.034909173	0.009635174	0.000409109
pcaec342.pcaec405	0.204543447	0.056538502	0.000416861
c51.pcaac342	0.073531376	0.02033503	0.000419478
pcaac403.pcaac342	0.073256497	0.020282027	0.000425582
phenylalanine.pcaac342	0.021290039	0.005900006	0.000430725
pcaec446.pcaec405	0.142270081	0.039562536	0.000449895
smohc222.pcaec405	0.094089031	0.026216884	0.000461411
pcaec380.pcaac342	0.026114191	0.007303924	0.000483738
pcaac300.pcaac342	0.019944958	0.005600594	0.000508205
pcaac321.pcaac342	0.040184491	0.011285998	0.000509375
pcaac361.pcaac342	0.028338686	0.007970923	0.000518869
pcaac406.pcaac342	0.044030148	0.012387217	0.00052027
c8.asparticacid	0.099676283	0.028104893	0.000534759
pcaac365.pcaac342	0.013535064	0.003818707	0.000538806
pcaac360.pcaac342	0.038327377	0.010814114	0.0005392
pcaac343.pcaac342	0.032691456	0.009225017	0.000539981
smc241.pcaec405	0.093868184	0.026501953	0.000543456
pcaac421.pcaec405	0.314922321	0.088937913	0.000545367
c14.pcaac342	-0.012533664	0.00354191	0.00054964
c141oh.pcaac342	-0.023796118	0.006728457	0.000553533
c142.pcaac342	-0.008215831	0.00232448	0.000557686
isoleucine.pcaec405	-0.230768265	0.065309518	0.000559672
leucine.pcaac342	0.016220132	0.004593363	0.000564041
creatinine.c18	-0.120097741	0.034031613	0.000568363

smc161.pcaec405	0.105090885	0.029780672	0.000568711
pcaac366.pcaac342	0.013041919	0.003700085	0.00057677
pcaac424.pcaac342	0.025088869	0.007120659	0.000579509
pcaac426.pcaac342	0.014771387	0.004197907	0.000588878
pcaec362.pcaec405	0.141146325	0.040126693	0.000591392
pcaac341.pcaac342	0.076599722	0.02178282	0.000593432
pcaac402.pcaac342	0.027664858	0.007895861	0.000620217
c12.pcaac342	-0.008123622	0.002321653	0.000630219
c2.pcaac342	-0.018696112	0.005348497	0.00063782
pcaac425.pcaac342	0.022580488	0.006477797	0.000659548
c162.pcaac342	-0.005754372	0.001654803	0.000678937
isoleucine.pcaac342	0.0165474	0.004762365	0.000685379
pcaac405.pcaac342	0.016385703	0.004718467	0.000689953
smc261.pcaec405	0.096238496	0.027730118	0.000695009
pcaac404.pcaac342	0.021487722	0.006191627	0.000695228
lysopcac180.pcaac342	0.018947123	0.005461112	0.000697576
pcaec401.pcaac342	0.022597296	0.006523041	0.000710169
c101.pcaac342	-0.006226747	0.001799014	0.000717548
c141.pcaac342	-0.006533858	0.001888406	0.000720526
pcaec381.pcaac342	0.053305575	0.015412762	0.000724104
pcaec421.pcaac342	0.050069728	0.014478135	0.000724685
lysopcac140.pcaac342	0.008379378	0.002423479	0.000726462
smc202.pcaac342	-0.005262222	0.001522274	0.000728362
glutamicacid.pcaec405	-0.242544199	0.070186572	0.000731123
pcaac384.pcaec340	0.228686178	0.066195765	0.000733648
pcaec382.pcaac342	0.099741607	0.028874914	0.000734725
pcaec384.pcaac342	-0.039140463	0.011336757	0.000739089
leucine.pcaec405	-0.214471845	0.062162879	0.000745074
pcaec402.pcaec405	0.298327169	0.086543313	0.000752766
pcaac344.pcaac342	0.010303348	0.002994224	0.000768465
lysopcac203.pcaac342	0.014605878	0.004245268	0.000769947
acetylornithoine.pcaac342	0.01649448	0.004795393	0.000772191
lysopcac160.pcaac342	0.016519762	0.004805371	0.000777142
glutamicacid.pcaac342	0.016139855	0.004697557	0.000782367
ornithine.pcaac342	0.016676237	0.004862279	0.000798679
pcaac322.pcaac342	0.01278309	0.003728448	0.000801902
pcaac240.pcaac342	0.024043391	0.007013478	0.000802879
pcaac385.pcaac342	0.016443078	0.004803816	0.000817287
c10.pcaac342	-0.002181692	0.000637793	0.000823487
c8.pcaac342	-0.002359899	0.000690667	0.000834281
smc160.pcaec405	0.101655148	0.02975888	0.000836764
pcaec444.pcaec405	0.185325286	0.054328949	0.000850421
smc181.pcaac342	-0.00561549	0.001648133	0.000861951

pcaec301.pcaac342	0.014126763	0.004150486	0.000872311
alphaaminoadipicacid.pcaac342	0.008536588	0.002510567	0.000882302
lysopcac182.pcaac342	0.018782042	0.005524738	0.000884197
pcaec445.pcaac342	-0.008726378	0.002567658	0.000887333
c10.pcaec405	0.040492028	0.011928431	0.000899348
pcaec363.pcaac342	-0.024557032	0.007236323	0.000902388
smc161.pcaac342	-0.005702969	0.00168425	0.000925496
smohc241.pcaec405	0.122625855	0.036246438	0.000934696
c51dc.pcaac342	-0.009187703	0.002718728	0.000946384
lysopcac181.pcaac342	0.031129208	0.009216486	0.000952293
c102.pcaac342	0.070261273	0.020861064	0.00098309
pcaec425.pcaac342	-0.029021418	0.008622784	0.000990988
smohc141.pcaac342	-0.005002874	0.001486489	0.000991326
smc180.pcaac342	-0.005983325	0.001778457	0.000995411
smohc161.pcaac342	-0.005285639	0.001571494	0.00099837
alanine.pcaac342	0.011231085	0.00333916	0.000998374
pcaec386.pcaac342	0.097798941	0.029092542	0.001004389
smc160.pcaac342	-0.005687448	0.00169245	0.001008315
pcaec365.pcaac342	0.054015923	0.016075794	0.001009679
pcaec362.pcaac342	-0.008347605	0.002484452	0.00101015
c4.pcaac342	-0.007253462	0.002159318	0.001012831
pcaac364.pcaac342	0.020391827	0.006072705	0.001016891
pcaec446.pcaac342	-0.009632558	0.002870066	0.001022794
pcaac384.pcaac342	0.026190298	0.007806596	0.001027314
pcaec322.pcaac342	-0.00993963	0.002962871	0.001027876
glutamicacid.smohc221	-0.444476236	0.132515519	0.001029884
arginine.pcaac342	0.093172262	0.027805737	0.00104134
pcaec444.pcaac342	-0.014840447	0.004429296	0.001042411
smc241.pcaac342	-0.004897062	0.001462431	0.001049188
h1.pcaac342	0.023964666	0.007157208	0.001050065
lysopcac161.pcaac342	0.028006871	0.008367227	0.001053976
smohc222.pcaac342	-0.004825136	0.001442492	0.00106177
smc261.pcaac342	-0.00519217	0.001552317	0.001062521
lysine.pcaac342	0.014926026	0.004462554	0.001062731
pcaec341.pcaac342	-0.008935221	0.002674078	0.001074495
serotonin.pcaac342	-0.012650363	0.003786263	0.001075567
smohc241.pcaac342	-0.008190334	0.002452523	0.001081189
lysopcac204.pcaac342	0.014981143	0.004486292	0.00108205
pcaac420.pcaac342	-0.014710348	0.004406281	0.001085005
pcaec342.pcaac342	-0.014737506	0.004417199	0.001092621
pcaec343.pcaac342	-0.031384606	0.009424723	0.00111596
creatinine.pcaac342	0.015560575	0.004676469	0.001125669
smc260.pcaac342	-0.006981779	0.002102704	0.001152244

pcaec406.pcaac342	-0.021721579	0.006543174	0.00115473
prolinr.pcaac342	0.024059165	0.007250693	0.001160645
pcaec322.pcaec365	0.138303991	0.041714536	0.001171072
pcaac401.pcaac342	0.090289986	0.027237115	0.00117311
pcaec403.pcaac342	0.131459733	0.039718135	0.001193242
c5.pcaac342	0.032747713	0.009904975	0.001207631
smohc141.pcaec405	0.088842962	0.026944644	0.001243818
c8.pcaec405	0.042501449	0.012906553	0.001261261
asparagine.pcaac342	0.016955205	0.005153655	0.001274095
pcaec321.pcaec365	0.093164812	0.028336156	0.001282911
lysopcac182.c182	-0.363581885	0.110655379	0.001291923
pcaec385.pcaac342	-0.116717715	0.035533852	0.001296275
histidine.pcaac342	0.013011668	0.003966524	0.001314808
pcaec343.pcaec405	0.237951015	0.072729132	0.001352556
c3.pcaac342	0.035930108	0.011018098	0.001400937
alphaaminoadipicacid.pcaec405	-0.114877106	0.035276741	0.001421942
serine.pcaac342	-0.022738318	0.006992435	0.001443503
pcaec423.pcaac342	-0.016557078	0.005103297	0.001479033
smc260.pcaec405	0.110117562	0.034020822	0.001516172
pcaac281.pcaac342	-0.015974923	0.004949274	0.00156136
pcaec321.pcaac342	-0.018506934	0.005738302	0.001574445
c14.c142	-0.275872525	0.085608442	0.00158812
c18.c142	-0.152677319	0.047479537	0.001623742
c141oh.c142	-0.180346968	0.056172542	0.001650516
glycine.pcaac342	-0.022985491	0.007161757	0.001656465
pcaec364.pcaac342	0.035972185	0.011214848	0.001666812
c182.c142	-0.19080834	0.059536646	0.0016812
c142.smc202	-0.163170342	0.050916264	0.001682334
pcaec406.pcaec405	0.261162891	0.081505522	0.001684751
pcaac385.pcaec340	0.253286891	0.079171	0.001712139
putrescine.pcaac342	0.0188411	0.005901783	0.001750053
pcaec423.pcaec405	0.207807032	0.065131026	0.001760462
smohc161.pcaec405	0.090069593	0.028242417	0.001768636
c181.c142	-0.137288868	0.043134076	0.001804945
smc181.pcaec405	0.09460306	0.029730834	0.001809912
smohc221.pcaac342	-0.0131061	0.004119759	0.001814026
pcaec340.pcaac342	-0.020125137	0.006357002	0.001906186
pcaac421.pcaac342	-0.025442716	0.008037333	0.001907744
smc202.pcaec405	0.085133717	0.026933863	0.001936788
pcaec402.pcaac342	-0.019485616	0.006208551	0.002079728
asymmetricdimethylarginine.pca	0.240256132	0.076587861	0.002089756
pcaec443.pcaac342	-0.047414615	0.01514731	0.002135167
pcaac320.pcaec365	0.088008527	0.028129201	0.002145416

asymmetricdimethylarginine.pca	-0.060388887	0.019345645	0.002194606
pcaac281.pcaec405	0.195345552	0.062811631	0.002276309
methionine.pcaac342	0.069708627	0.022430849	0.002292977
serine.c18	0.176926993	0.056939753	0.002296206
pcaac362.pcaac342	0.151706165	0.048829667	0.002299271
pcaec363.c12	0.219377989	0.070618529	0.002301613
asymmetricdimethylarginine.c16	0.085703459	0.02770088	0.002395116
pcaec361.pcaac342	-0.026302094	0.008511342	0.00242277
pcaac383.pcaac342	-0.038416679	0.012525974	0.00260628
lysopcac281.pcaec405	0.31105166	0.101822041	0.002706706
c4.c182	0.136345984	0.044800127	0.002804776
c16.c142	-0.288916285	0.095062205	0.002841588
c162.smohc221	-0.214576065	0.070843206	0.002934313
lysopcac170.pcaac342	-0.03882812	0.012845598	0.002991276
pcaec404.pcaac342	0.151191905	0.050105016	0.003039575
lysopcac180.pcaec405	-0.14170388	0.046970993	0.003045803
kynurenine.c142	-0.209861501	0.069583209	0.003053899
lysopcac203.pcaec405	-0.153089095	0.050920578	0.003145252
smc180.pcaec405	0.092989403	0.030945558	0.003159764
smc240.pcaac342	-0.025392506	0.008480566	0.003265913
c101.c18	-0.107806426	0.036008754	0.003268969
lysopcac181.smc160	-0.232452627	0.077711259	0.003295795
c16.pcaac342	0.046614	0.01559725	0.003322529
histidine.pcaec405	-0.150787637	0.050663056	0.003450381
lysopcac281.pcaec365	0.077585669	0.026083058	0.003468609
arginine.smohc221	0.224170774	0.075580698	0.003560782
pcaec404.pcaac402	-0.446074989	0.150709377	0.003628137
pcaac405.c142	-0.145424259	0.049324145	0.003757349
prolinr.transhydroxyproline	-0.12288662	0.041710182	0.003781817
pcaac281.c12	0.186530699	0.06337323	0.003814509
pcaec300.pcaac402	-0.199354444	0.067835877	0.003868017
c101.c12	0.137255841	0.046711944	0.003873066
pcaac320.pcaac342	-0.047573233	0.016256927	0.004016471
c141.pcaec405	0.097898741	0.03349245	0.004057048
phenylalanine.c5	-0.103945324	0.035580858	0.004077017
c10.asparticacid	0.079560734	0.027257984	0.00410887
pcaec383.pcaac342	-0.063941179	0.021941281	0.004166379
pcaac320.pcaec405	0.21087895	0.072436155	0.004203654
c8.smc202	-0.168466175	0.057942618	0.004251668
kynurenine.c18	-0.113716119	0.039130247	0.004269267
pcaac381.c12	0.176116432	0.060822914	0.00440648
pcaec321.c12	0.137833509	0.047619576	0.004420896
pcaec425.pcaec405	0.323146488	0.111743893	0.004455788

smohc221.pcaac362	0.139827266	0.048515851	0.004587805
lysopcac203.c182	-0.138050461	0.047925975	0.004609811
pcaac342.pcaec365	0.083078125	0.028874393	0.004655039
alanine.pcaec405	-0.115491009	0.040343534	0.004861131
serine.smc240	-0.171397104	0.059932774	0.004902751
pcaac380.pcaac342	-0.061217176	0.021408705	0.004908146
pcaec364.pcaec340	0.234337952	0.082153452	0.005011405
lysopcac281.pcaac342	-0.035533303	0.012496072	0.00514511
c18.pcaec383	0.292714127	0.103047557	0.005190754
methioninesulfoxide.glutamine	0.105425989	0.037190867	0.005281219
pcaac341.pcaec365	0.08678175	0.030619347	0.005289259
citruline.c18	-0.078453699	0.027722891	0.005356631
prolinr.pcaac405	-0.165417177	0.058690385	0.005540438
alanine.c16	-0.103817529	0.036855918	0.005566949
c8.c51	0.250666784	0.089273146	0.005715798
creatinine.pcaec384	0.207959413	0.074066909	0.005718138
kynurenine.c16	-0.096921689	0.0345693	0.005786115
pcaec385.pcaec340	0.09535011	0.034051346	0.005845842
pcaac321.c18	0.152127948	0.054531257	0.006027153
lysopcac280.c14	0.116116961	0.041661152	0.006072399
lysopcac181.pcaac426	-0.206686391	0.074339429	0.006195223
creatinine.pcaec360	0.007809734	0.002841448	0.006793801
putrescine.pcaac426	-0.139879539	0.051063675	0.006976295
lysopcac181.lysine	0.232953783	0.085062007	0.006989995
c10.c51	0.196836376	0.07193972	0.007040612
pcaec361.pcaec405	0.269366772	0.09858513	0.007118069
c142.c12	0.293813157	0.107756497	0.007235344
c101.pcaec405	0.075344354	0.027691201	0.007355973
creatinine.c16	-0.0568974	0.020934815	0.007420357
c4.pcaec443	-0.120131581	0.044216877	0.007440887
pcaec420.lysine	0.220640717	0.081245832	0.007465523
c5.pcaec405	-0.210569423	0.077539166	0.007466911
c10.c182	0.110025483	0.040525969	0.007482203
smohc221.pcaec405	0.13892534	0.051236725	0.007557444
pcaac321.c14	0.189433956	0.069892452	0.007580755
pcaac343.pcaac405	0.116993388	0.04339846	0.007902511
pcaec341.pcaec365	0.054351536	0.020204331	0.008031793
alanine.c5	-0.051917455	0.019314617	0.008080011
lysopcac140.pcaec405	-0.093115416	0.034751967	0.008278721
pcaec361.c12	0.161644175	0.060449992	0.008406684
pcaec340.c141oh	-0.305121706	0.114160027	0.008436732
pcaac322.pcaac381	0.196916078	0.073679883	0.00844067
pcaec420.pcaac426	-0.133422748	0.04994767	0.008472705

smohc141.smc202	0.227493902	0.085268863	0.008551916
methioninesulfoxide.c18	0.201133435	0.075419077	0.008578116
prolinr.c142	-0.235968777	0.088765552	0.008787389
c8.smohc221	-0.173095885	0.065174457	0.008848319
pcaec381.pcaec402	-0.303544882	0.114354431	0.008884872
pcaec443.glutamine	0.037863895	0.014269584	0.008908745
c162.pcaec385	-0.171543978	0.06469599	0.008957248
pcaec383.glutamine	0.045591447	0.017237656	0.009126652
h1.c142	-0.12353854	0.046748849	0.009185122
c12.pcaec405	0.10069521	0.038144166	0.009256061
pcaac300.c18	0.105656325	0.04004365	0.009290647
smohc222.c12	0.148248588	0.056235012	0.009350628
smohc222.pcaec443	-0.232260468	0.088131628	0.009372958
pcaac403.glutamine	0.056566027	0.021507202	0.009512648
lysopcac140.smc240	-0.203376164	0.077357099	0.009540378
lysopcac181.c182	-0.158904173	0.060591167	0.009715034
c8.c18	-0.087576392	0.033403442	0.009736366
pcaec382.c18	0.172268321	0.065830072	0.009870721
pcaec363.pcaec405	0.17224798	0.065860787	0.009912874
pcaec364.pcaec322	0.3857027	0.147638236	0.009991843
pcaec343.c12	0.199161077	0.076378682	0.010130022
pcaec401.leucine	-0.163431736	0.062684362	0.010139346
pcaac362.c12	0.190638222	0.073272454	0.010293859
pcaac322.c5	0.037687175	0.014505367	0.010397702
pcaec381.c18	0.152588967	0.058806788	0.010496223
pcaac281.smohc222	-0.019687091	0.007600272	0.010625943
pcaec386.pcaec340	0.191443945	0.07392096	0.010639694
c141oh.smohc222	0.030693083	0.011882931	0.010844259
pcaec340.pcaec365	0.052561465	0.020359945	0.010884574
pcaac342.glutamine	0.030124577	0.011671357	0.010900881
pcaec424.glutamine	0.036379026	0.014100166	0.010931796
pcaec361.pcaec365	0.057294444	0.022221661	0.010983999
pcaac365.c12	0.17595435	0.06825942	0.011001736
pcaec403.c18	0.162303582	0.062992744	0.011037756
c162.pcaec360	-0.026955023	0.010462832	0.011046265
citruline.smc202	-0.118838407	0.046132859	0.011054211
asymmetricdimethylarginine.c51	-0.303644309	0.118024479	0.011154541
lysopcac140.smc202	0.095835414	0.037254316	0.011162478
prolinr.c16	-0.073942213	0.02876727	0.011227352
pcaec342.c12	0.145612896	0.056717713	0.011321371
pcaac300.smc240	-0.17680486	0.06886841	0.011322657
pcaec382.glutamine	0.041797753	0.016283205	0.011333902
acetylornithoine.c8	-0.16240628	0.0632935	0.011365114

pcaac321.pcaec382	0.042345504	0.016512375	0.011410561
pcaec403.glutamine	0.050541458	0.0197133	0.011430808
asparagine.spermidine	-0.170654766	0.066565933	0.011434892
glutamine.pcaac342	0.062711897	0.024511257	0.011599274
pcaec301.smc202	0.10599006	0.041443884	0.011632977
smohc141.c12	0.181425041	0.071004556	0.011707079
pcaac323.glutamine	0.036579271	0.014318219	0.011719343
pcaac384.c12	0.157922059	0.061843938	0.011757227
pcaec423.pcaec342	0.300036581	0.117504207	0.011761937
pcaac426.c12	0.138540568	0.054299926	0.011827031
pcaec425.glutamine	0.033122654	0.012982631	0.011829941
pcaac385.pcaec445	0.134304235	0.052680185	0.011890916
histidine.pcaec402	-0.098428467	0.038613427	0.011902382
lysopcac161.c182	-0.138327472	0.05431603	0.011979363
pcaac404.c182	-0.081950028	0.032220738	0.012088549
c10.c12	0.267003966	0.105063281	0.012155737
lysopcac170.smohc222	0.035993767	0.014164576	0.012164093
pcaec322.c12	0.111549329	0.043921208	0.012209021
pcaac383.smc202	0.09406456	0.037046714	0.012231696
pcaac361.c12	0.18529796	0.073109484	0.012384402
pcaec423.glutamine	0.03286473	0.012984493	0.012501312
pcaec361.glutamine	0.029559976	0.011692662	0.01260359
c18.smohc221	-0.178573151	0.070641017	0.012609868
pcaac300.c5	0.035276411	0.013962133	0.012655141
pcaac343.glutamine	0.02423035	0.009590672	0.012659539
smohc222.c16	0.106829161	0.042284665	0.012660281
pcaac321.lysopcac203	0.05289312	0.0209396	0.01267552
pcaac361.smc160	-0.241888319	0.095812781	0.012723531
pcaec384.pcaec365	0.071508536	0.028326665	0.012729359
pcaac281.smc202	0.106772467	0.042300606	0.01273936
pcaec340.smc202	0.114754557	0.045477592	0.012767572
c51dc.smohc222	-0.020798839	0.008261938	0.012973267
creatinine.c12	-0.056555568	0.022472434	0.013000178
pcaac402.pcaec405	-0.28229305	0.112384201	0.013170495
pcaac384.pcaec445	0.139786128	0.055656737	0.013180512
pcaac383.glutamine	0.031845216	0.012680326	0.013187227
prolinr.pcaec405	-0.185851889	0.074086657	0.013287947
c10.pcaec381	0.100122587	0.039916846	0.01329856
c10.smc202	-0.13571352	0.054111843	0.013307917
pcaac322.glutamine	0.048643471	0.019416381	0.013406759
lysopcac161.glutamine	0.028262657	0.011301585	0.013570977
pcaac381.glutamine	0.041132595	0.016466984	0.013677004
citruline.c14	-0.091780517	0.036770198	0.01374444

pcaec321.glutamine	0.039982312	0.016023836	0.013777048
smc181.pcaec381	0.088462599	0.03546759	0.013814047
smc180.pcaac362	0.113322169	0.045487643	0.013922556
c16.smohc221	-0.22116245	0.088790627	0.013939143
pcaac365.glutamine	0.025556289	0.010260692	0.013944117
pcaec444.glutamine	0.026689173	0.010732742	0.014094561
pcaac366.glutamine	0.030316876	0.01219429	0.014115494
smc161.smohc222	-0.034459406	0.013869372	0.014175694
pcaac386.c12	0.131001512	0.052728727	0.014180476
pcaec343.pcaec365	0.079956989	0.032188052	0.014195134
pcaec361.smohc222	-0.031634324	0.012736838	0.014209265
pcaac385.c12	0.142510669	0.057380436	0.014212185
lysopcac280.c181	-0.115365097	0.046470054	0.014251988
citruline.pcaec360	0.018419049	0.007420928	0.014272174
pcaac363.glutamine	0.022109759	0.008910501	0.014300003
c162.asparticacid	0.051277932	0.020691079	0.014417487
pcaac402.glutamine	0.043126269	0.017414743	0.01448873
pcaec404.glutamine	0.033050853	0.013359798	0.014586785
c10.phenylalanine	0.124337026	0.050297576	0.014660167
pcaec363.pcaec360	-0.017297491	0.006999213	0.014686866
pcaec340.smohc222	-0.01429462	0.00578827	0.01475629
kynurenine.pcaec365	-0.060800923	0.024652643	0.014886158
pcaec380.glutamine	0.031117108	0.012619782	0.014908591
c141.asparticacid	0.08865499	0.035957569	0.014916391
pcaec421.glutamine	0.02703564	0.010972326	0.014978635
pcaec381.glutamine	0.041231589	0.016737312	0.014999877
serine.c12	0.158957617	0.064553524	0.015041457
pcaac365.pcaac405	0.252644061	0.102776063	0.015211473
smohc222.pcaec385	-0.147161407	0.05987249	0.01522306
smc180.pcaec365	0.111042895	0.04518316	0.015235099
pcaac422.glutamine	0.034424358	0.014010816	0.015260777
pcaac343.c5	0.03439628	0.014018465	0.015397205
lysopcac160.glutamine	0.026389157	0.010756121	0.015406744
pcaac424.glutamine	0.0376612	0.015363767	0.015493375
arginine.c182	-0.080686127	0.032915918	0.0154942
c142.smohc222	0.012287252	0.005013898	0.015520638
pcaac363.c12	0.119714539	0.048860371	0.015541398
glycine.lysopcac203	-0.092678358	0.037859068	0.015630542
ornithine.pcaec405	-0.142853672	0.058356944	0.015632776
pcaec343.glutamine	0.037878871	0.01547486	0.015639464
pcaac406.c141oh	-0.206370211	0.084327782	0.015661473
pcaac380.pcaec405	0.19818914	0.081000696	0.015681457
lysopcac180.smohc222	0.015218054	0.00622253	0.015728194

smc261.pcaac362	0.035901959	0.014682263	0.015743936
c181.lysopcac204	0.081096722	0.033181194	0.015794309
pcaac405.pcaec405	-0.110893973	0.045373664	0.015796006
pcaac344.pcaac405	0.075391015	0.030859889	0.015838229
c142.asparticacid	0.077187165	0.031609677	0.01588559
pcaec322.glutamine	0.05699507	0.023348848	0.015921897
pcaec363.glutamine	0.032927341	0.013503806	0.016034006
pcaac240.glutamine	0.03408606	0.013979666	0.016038883
pcaac421.glutamine	0.031828266	0.013054413	0.016044698
pcaac320.smc202	0.097975031	0.040186272	0.016048978
lysopcac280.pcaec421	-0.013791832	0.005662706	0.016154155
pcaec405.glutamine	0.0307817	0.01263954	0.016162938
pcaac240.lysopcac203	0.080129367	0.032926753	0.016239465
pcaec343.c14	-0.070538947	0.028990304	0.016255502
c10.c18	-0.081936207	0.033683976	0.016285313
pcaec362.glutamine	0.040790557	0.016774685	0.016320858
kynurenine.pcaac342	-0.051100898	0.021015302	0.01632384
smc161.pcaec365	0.155027757	0.063771261	0.016350056
pcaac344.pcaec382	0.028892321	0.011892208	0.016414129
pcaec406.glutamine	0.035370358	0.014564007	0.016453303
c142.lysopcac203	-0.032452845	0.013365765	0.01647753
pcaac366.c12	0.159770129	0.065843257	0.016544441
pcaec381.smc240	-0.215450541	0.08879272	0.01654787
lysopcac204.smohc241	-0.23732355	0.097812907	0.016554109
methionine.c14	0.085982604	0.035471522	0.016655363
pcaec360.pcaac342	0.069755868	0.028778388	0.016659404
alanine.pcaec360	0.013542466	0.005588098	0.016679169
lysopcac182.pcaac426	-0.166058858	0.068565732	0.016747695
pcaec422.glutamine	0.02168764	0.008960915	0.016820424
lysopcac281.pcaac402	-0.204129575	0.084353493	0.016834528
pcaec444.methionine	-0.212342098	0.087797704	0.016896366
ornithine.pcaec382	-0.024733863	0.010228661	0.016916141
pcaec321.pcaac320	0.343250004	0.141968886	0.016929979
pcaac366.smc202	0.140364189	0.058086195	0.016988109
pcaac420.glutamine	0.037641254	0.01557701	0.016988957
phenylalanine.pcaac362	0.108605917	0.044944485	0.016989679
acetylornithoine.c51dc	0.34652099	0.143457439	0.01703216
pcaec446.glutamine	0.052522689	0.021746735	0.017045517
c102.c12	0.171728528	0.07111641	0.01706554
lysopcac140.lysopcac203	0.042745534	0.017707153	0.017098161
asymmetricdimethylarginine.smc	0.042869131	0.017767895	0.01715654
pcaac343.smohc222	-0.025336049	0.010502023	0.01716726
citruline.asparagine	-0.016020834	0.006642586	0.017196863

lysopcac203.pcaac405	0.070568217	0.029263651	0.017213753
c101.c182	0.126217979	0.052347348	0.017227343
pcaac405.smc202	0.074966541	0.031093152	0.017233328
alanine.pcaac323	0.271571445	0.112686897	0.017281648
acetylornithoine.c16	-0.135981656	0.056438267	0.017307865
pcaec383.c18	0.122333136	0.050775511	0.017312114
pcaac341.glutamine	0.019143309	0.007946234	0.017320729
smc181.pcaec365	0.138083062	0.057392397	0.017464869
c162.c142	-0.202409645	0.084165805	0.017513276
pcaec341.smohc222	-0.013967241	0.005813882	0.017628282
lysopcac203.glutamine	0.032183922	0.013406836	0.017713203
c10.lysopcac203	-0.022389574	0.009332359	0.017779522
c12.glutamine	0.058212406	0.024270932	0.017811762
lysopcac160.pcaec405	-0.094026266	0.03920854	0.017827364
pcaac383.c141oh	-0.315545371	0.131652213	0.017887949
pcaec340.c12	0.135587616	0.056582633	0.017912957
lysopcac280.glutamine	0.029909671	0.012484056	0.017933891
pcaec444.pcaec342	0.282354743	0.117959853	0.018036328
isoleucine.c14	0.080640326	0.033692651	0.018047702
pcaec420.lysopcac203	-0.023020463	0.009620195	0.018070312
c162.c182	0.200191878	0.083674259	0.018089984
pcaac321.glutamine	0.029972488	0.012530221	0.018113568
pcaac323.pcaac405	0.114097369	0.047739387	0.018208947
pcaac361.glutamine	0.019694163	0.008241183	0.01822224
lysopcac170.glutamine	0.036384501	0.015229489	0.018252862
pcaac401.glutamine	0.039376385	0.016488998	0.01830258
kynurenine.c12	-0.088141589	0.036924234	0.018347837
pcaac343.lysopcac203	0.075004939	0.031456776	0.018478054
pcaec342.glutamine	0.0407382	0.01708557	0.018478924
pcaac344.smohc222	-0.015900368	0.006669144	0.018488255
pcaec420.pcaac420	-0.280567264	0.117699896	0.018508412
lysopcac204.lysopcac203	-0.025104428	0.010535035	0.018547242
putrescine.lysopcac203	-0.021818734	0.00917286	0.018757452
pcaac341.smohc222	-0.018330302	0.007713174	0.018861439
lysopcac182.lysopcac203	-0.031083464	0.013085684	0.018916119
pcaec300.smohc222	-0.012895531	0.005431368	0.018970767
pcaac405.c141oh	-0.24568878	0.103510587	0.019005674
pcaac240.c182	-0.10493207	0.04422422	0.019046858
glutamicacid.glutamine	0.054892129	0.023137997	0.019064092
pcaac240.c14	0.113847861	0.048002733	0.019098031
pcaec340.glutamine	0.022138439	0.009338614	0.019150706
serine.glutamine	0.030588499	0.012904404	0.019162754
prolinr.c5	-0.045644473	0.019258504	0.019177567

pcaac401.c12	0.13412518	0.056667928	0.01933903
pcaec386.pcaac320	0.250211295	0.105790994	0.019425184
pcaac362.glutamine	0.02883173	0.012192461	0.019446824
c10.smohc221	-0.171324582	0.07248981	0.01951181
asparticacid.pcaac342	0.160692376	0.068000108	0.019527505
lysopcac280.lysopcac203	0.063648538	0.026939834	0.019552876
pcaec301.pcaac405	0.069013905	0.029214191	0.019566866
lysopcac140.glutamine	0.03396582	0.014385978	0.019633092
pcaac320.glutamine	0.023320896	0.009884212	0.019715956
histidine.c5	-0.057121581	0.024216134	0.019745842
pcaec423.smohc221	0.264993337	0.112422239	0.019832713
pcaec300.lysopcac203	0.044952504	0.019077807	0.019876513
asymmetricdimethylarginine.lys(	-0.091724786	0.038930762	0.019885332
pcaec341.glutamine	0.022391136	0.009517125	0.02005969
smohc141.asparticacid	0.06472103	0.027515991	0.020090597
pcaec342.c14	-0.071142517	0.03025561	0.020129084
smc161.c16	0.085663037	0.036433713	0.020138445
lysopcac204.pcaec405	-0.093030905	0.039575759	0.020164354
pcaac380.c12	0.123408161	0.052516619	0.02020687
smc161.c14	-0.061830228	0.026329149	0.020286819
pcaac344.pcaac402	-0.152030112	0.06479263	0.020388826
c12.phenylalanine	0.087401389	0.037249773	0.020391468
c102.pcaec443	-0.059882762	0.025528437	0.020424731
arginine.glutamine	0.019268184	0.008219148	0.020499756
pcaac342.smohc222	-0.018695265	0.007975585	0.020512545
pcaec420.pcaec443	-0.075992239	0.032424011	0.020531532
pcaec401.glutamine	0.019519227	0.008329128	0.020542775
pcaec384.glutamine	0.031872606	0.013625312	0.020769679
asparagine.pcaec421	-0.036401272	0.015565779	0.020805768
pcaac406.c12	0.153058268	0.065497628	0.020896475
alanine.c182	-0.149443627	0.063977462	0.02094876
lysopcac203.c14	0.098331868	0.042108644	0.020985641
pcaac365.smc202	0.086894021	0.037213266	0.020994603
smc161.c12	0.117494019	0.050337657	0.021043726
c141.smohc222	0.013121813	0.005622277	0.021055689
pcaec300.glutamine	0.023155334	0.00992231	0.021068268
c182.pcaec382	-0.020606229	0.008835439	0.021146205
c0.pcaac405	0.084267147	0.036154304	0.021225591
pcaec360.glutamine	0.027232576	0.011684632	0.021232777
pcaac362.pcaec361	-0.026403531	0.011332536	0.021273364
c182.pcaec361	-0.02098299	0.009007954	0.021300781
c4.c142	0.142123002	0.061055627	0.021389515
lysopcac181.glutamine	0.029248966	0.012568549	0.021422816

creatinine.pcaec385	0.082421431	0.035426224	0.021455202
pcaac344.glutamine	0.027021046	0.011617455	0.021491758
pcaac240.pcaec445	0.19915541	0.085684446	0.021580619
h1.pcaec382	0.024211848	0.010418886	0.021605306
pcaec404.smohc222	-0.03301321	0.014211783	0.021654887
lysopcac140.pcaac405	0.034166736	0.014709656	0.021666204
pcaec386.c12	0.115490825	0.049762833	0.021772687
pcaac380.pcaec342	0.349082851	0.150436554	0.021792699
kynurenine.pcaec360	0.010878092	0.004687934	0.021793857
smc260.pcaec381	0.049978091	0.021560563	0.021928603
c142.pcaec382	-0.026750646	0.01154321	0.02196207
pcaec424.pcaac342	-0.082123229	0.035442892	0.021983277
c16.c18	0.267226965	0.115446716	0.022114789
pcaac300.smohc222	-0.011988148	0.005179484	0.022124741
pcaec363.c14	-0.069812088	0.030195655	0.022269327
pcaac425.pcaac422	-0.139255411	0.060232755	0.022271387
pcaac321.smohc222	-0.016957665	0.007338405	0.02233644
pcaac404.pcaec322	0.168829456	0.073075205	0.022362559
pcaec406.smc240	-0.175778712	0.076109528	0.02240835
c162.pcaec381	0.030571614	0.013240046	0.022438228
pcaac404.pcaec405	-0.123855154	0.053670503	0.022514435
c8.pcaec402	-0.171321707	0.074241421	0.022518181
c4.pcaac405	0.134804932	0.058426089	0.022538647
c162.c101	-0.078064807	0.03383679	0.022548657
lysopcac181.lysopcac203	-0.027775008	0.012045653	0.022622629
asymmetricdimethylarginine.c8	-0.092140461	0.039983114	0.022699158
pcaac386.glutamine	0.028273245	0.012274786	0.02276419
isoleucine.pcaac402	-0.168888102	0.073339924	0.02279587
pcaac421.c12	0.096871026	0.042081051	0.022842394
pcaac320.transhydroxyproline	0.062299194	0.027063814	0.022846671
pcaac343.c182	-0.090816383	0.039464561	0.02288871
pcaac386.c14	-0.173029123	0.075223179	0.022947137
pcaac426.pcaec405	-0.130245786	0.05667076	0.023059372
lysopcac140.asparagine	0.021889143	0.009534064	0.023200312
pcaec380.leucine	-0.119881086	0.052223257	0.023220232
c102.c181	-0.015347464	0.006690528	0.023317032
c3.c181	-0.031184793	0.013597407	0.023344994
pcaec362.c12	0.122188094	0.05329976	0.023402272
pcaec386.c14	-0.110594352	0.048253398	0.023433153
c12.pcaac366	-0.068056798	0.029702905	0.023474685
lysopcac204.c142	-0.084855576	0.037042472	0.023503614
pcaac404.smc202	0.126182819	0.055088757	0.023517174
ornithine.pcaac405	0.078502048	0.0342964	0.023613065

pcaac361.c141oh	-0.194001489	0.084762061	0.02362217
creatinine.lysopcac203	-0.031618325	0.013816639	0.023643329
c181.pcaec385	-0.148536812	0.064984159	0.023804485
pcaac322.smohc222	-0.012741486	0.005577057	0.023871584
h1.pcaac322	0.172150729	0.075379788	0.023922762
putrescine.asparagine	-0.012550814	0.005498623	0.023997897
c8.lysopcac203	-0.021471005	0.009412402	0.024082766
phenylalanine.pcaec405	-0.160784582	0.070517462	0.024148055
pcaec301.c2	-0.172059173	0.075479589	0.024179889
smc241.pcaec385	-0.16256257	0.071339944	0.02423135
smc161.smc202	0.116047841	0.050941571	0.024270855
lysopcac281.lysopcac203	0.05046733	0.022165182	0.024343429
pcaac420.c12	0.100413887	0.044128924	0.024429986
pcaac343.c181	-0.01566672	0.006889668	0.024523891
pcaec361.lysopcac203	0.062817561	0.027630658	0.024553017
lysopcac180.glutamine	0.027644619	0.012166389	0.024631123
lysopcac140.c18	0.109654611	0.048293771	0.024732888
asparticacid.pcaac426	0.187744367	0.082716872	0.024785971
pcaac406.smohc222	0.019648257	0.008661356	0.024862545
c101.smohc222	0.008720339	0.003844474	0.024876175
pcaec402.glutamine	0.067979993	0.029980098	0.024924696
isoleucine.pcaac405	0.136281033	0.060105723	0.024934017
pcaec446.c12	0.096591017	0.042634535	0.025046921
c18.pcaac362	0.083929065	0.037056714	0.025089545
smohc141.pcaac322	-0.149488901	0.066006939	0.025098135
pcaec446.serotonin	-0.295536682	0.130528493	0.025135408
pcaac321.transhydroxyproline	0.060974182	0.026937374	0.025173395
histidine.c182	-0.17344891	0.076648445	0.025213786
pcaac384.transhydroxyproline	0.094642386	0.041832689	0.025246238
pcaac422.c12	0.144548029	0.063961363	0.025403563
alphaaminoadipicacid.pcaec361	-0.029656003	0.013127815	0.025461408
lysopcac180.smc160	-0.13878736	0.061489155	0.02558411
kynurenine.c181	-0.027096302	0.012007552	0.025616071
pcaec404.pcaec421	0.021972745	0.009739319	0.025649381
creatinine.c14	-0.10239827	0.045401236	0.025693067
pcaec360.serotonin	-0.292604873	0.129738364	0.025697013
lysopcac161.smohc221	0.225325195	0.09993182	0.02573286
c12.lysopcac204	0.053989175	0.023955653	0.025802287
glutamicacid.pcaec300	-0.08594189	0.03815236	0.025874497
pcaac344.c182	-0.084326924	0.037436704	0.025879485
leucine.lysine	-0.314976073	0.13987726	0.025925942
lysopcac281.glutamine	0.035766662	0.01588801	0.025966844
pcaac364.c12	0.108848502	0.048360316	0.025992307

pcaec385.glutamine	0.03125521	0.013889868	0.026029064
c162.pcaec382	-0.02369406	0.010530188	0.026036127
pcaec445.smohc221	0.063734219	0.028326706	0.026045161
pcaec403.pcaec381	-0.128020873	0.056911261	0.026076899
pcaac385.glutamine	0.016990197	0.007555122	0.026119364
c141.smohc221	-0.111744781	0.049694117	0.026131076
asparticacid.glutamine	0.050183959	0.022333471	0.026237287
smohc241.c12	0.154122424	0.068598694	0.026257204
glutamine.pcaec421	-0.017516923	0.007796789	0.026259904
pcaac344.pcaac381	0.123331274	0.054914771	0.026313561
pcaec406.c14	-0.112154739	0.049989337	0.026464688
lysine.pcaec405	-0.12256337	0.05463857	0.026491614
c51dc.pcaac323	-0.31447782	0.140279622	0.026582529
pcaac383.c12	0.124691345	0.055622868	0.026586809
smohc141.c16	0.154334551	0.068860787	0.026618228
putrescine.c16	-0.087574827	0.039091625	0.026685304
pcaec341.c141oh	-0.231507641	0.10336957	0.02672752
acetylornithoine.pcaac405	0.130364057	0.058212165	0.026737313
pcaec420.pcaec342	0.209287963	0.093458401	0.026743574
pcaac344.pcaec403	-0.187181885	0.083673956	0.026899379
pcaec444.c12	0.108145718	0.04836752	0.026974684
pcaec385.tyrosine	-0.141175681	0.063141645	0.026978666
c142.pcaec385	-0.139451686	0.062423708	0.027106751
pcaac344.smc202	0.098003162	0.04388724	0.027166603
pcaec343.pcaac320	0.200598976	0.089872583	0.027236276
c51.pcaec405	-0.143912737	0.06448655	0.027261137
pcaac322.pcaac405	0.071291313	0.031968558	0.027371541
lysopcac180.asparagine	-0.022489773	0.010095162	0.027526151
pcaec446.pcaec340	0.070758662	0.03176373	0.027534445
citruline.pcaac405	0.074112128	0.033301908	0.02768482
pcaac366.smc240	-0.107263901	0.048224998	0.027768975
pcaac380.c14	-0.111219944	0.050024547	0.027833182
smc180.asparticacid	0.066467354	0.029898983	0.027849984
pcaec360.pcaec402	-0.120843088	0.054364226	0.027865316
pcaec360.c181	-0.030947148	0.013924615	0.02789049
lysopcac280.pcaec381	0.058502475	0.026340222	0.027990618
pcaac422.glutamicacid	0.154864524	0.069728823	0.027996013
lysopcac182.pcaac405	0.064136425	0.02887953	0.028004877
c142.pcaec381	0.073126041	0.032940089	0.028064296
asparagine.c141	-0.103337126	0.046583166	0.028178143
phenylalanine.c16	-0.091368091	0.041199697	0.028223372
c182.smc202	-0.125640134	0.056684594	0.028308072
pcaec321.smc202	0.11622082	0.052438253	0.028317951

histidine.pcaec381	-0.016078081	0.00725462	0.028323734
pcaec365.pcaac320	0.162779515	0.073452913	0.028334044
pcaac300.glutamine	0.029182643	0.013174189	0.028402067
c101.asparagine	-0.0146238	0.006601904	0.028405542
pcaac426.smc202	0.090090232	0.040671472	0.028406691
pcaac323.pcaec381	0.021993711	0.009930056	0.028421388
pcaac320.pcaec300	0.077788539	0.035134357	0.028479934
smc202.pcaac405	-0.076820109	0.034726144	0.028611262
c141.glutamine	0.027456408	0.012420954	0.028730123
alanine.pcaec443	0.086877998	0.039312782	0.02877086
pcaac406.glutamine	0.02305879	0.010439971	0.028857282
lysine.pcaec381	-0.019662891	0.008908925	0.028971589
lysopcac204.lysine	0.126065904	0.057133185	0.029012607
pcaec341.smc202	0.101473402	0.046006845	0.0290779
asparagine.pcaec381	-0.019844935	0.0089989	0.029103223
c14.phenylalanine	0.079507217	0.036064162	0.029150446
lysopcac140.smohc222	-0.014375819	0.006521993	0.029179062
c162.smc202	-0.098122166	0.044528289	0.029223313
pcaec401.pcaac420	-0.204820239	0.093022765	0.029350474
acetylornithoine.c181	-0.023933438	0.010870385	0.029359067
pcaac341.lysopcac203	0.055294483	0.025115841	0.029368638
pcaac361.smc240	-0.090557277	0.041138099	0.029388784
creatinine.c181	-0.072317549	0.032857114	0.029412581
pcaec385.pcaac405	-0.103874206	0.047194809	0.029412871
smc202.c16	0.086705585	0.039423111	0.029529486
c18.pcaec381	0.021327006	0.009703232	0.02963366
pcaec401.lysopcac203	-0.055558009	0.02527808	0.02963767
pcaec301.c162	-0.205951608	0.093717624	0.029659319
pcaec445.pcaec340	0.04944773	0.022520414	0.029797563
pcaac361.transhydroxyproline	0.064270877	0.029296984	0.029938012
pcaec382.pcaac422	-0.409850177	0.186850343	0.029960338
lysopcac204.asparagine	-0.014795276	0.00674638	0.029989694
pcaac364.pcaec382	0.02795057	0.012747668	0.030024048
pcaec300.pcaec365	0.050556018	0.023065073	0.030076901
pcaac425.glutamine	0.021362979	0.009746907	0.030085482
c142.asparagine	-0.018143879	0.008278261	0.030086953
smc160.c16	0.126396298	0.057699933	0.030173522
asparagine.pcaac402	-0.12336788	0.056317927	0.030174866
pcaac384.c141oh	-0.24891986	0.113633768	0.030176099
pcaac344.asparticacid	-0.074007107	0.03379306	0.030215797
isoleucine.lysopcac203	0.035671314	0.016288559	0.030219341
c4.pcaec405	0.068368009	0.031224023	0.030246399
pcaac322.pcaec382	0.03023881	0.013813824	0.030288798

c3.pcaec405	-0.107073195	0.048914666	0.030292214
lysopcac140.c182	-0.101995549	0.046626217	0.030401185
pcaec420.c12	0.076549249	0.035006538	0.030461012
asparticacid.c181	-0.045413462	0.020775424	0.030520091
kynurenine.c14	-0.073647819	0.033706297	0.030590105
c8.asparagine	-0.014208142	0.006503936	0.03062323
serotonin.pcaec420	0.180811365	0.082772347	0.030630987
pcaec343.pcaac362	0.035855673	0.01641531	0.030642933
pcaec420.glutamine	0.019676976	0.009011829	0.030704646
pcaec301.c182	-0.096771906	0.044338672	0.03077227
smc161.pcaec381	0.020803766	0.00953455	0.030819546
acetylornithoine.pcaec381	0.013234211	0.00606767	0.030882378
methioninesulfoxide.pcaec381	-0.038467724	0.017644346	0.030952685
c141.lysopcac204	0.098496168	0.045206814	0.031057502
methioninesulfoxide.pcaec340	-0.099634963	0.045737062	0.031084911
c141.c12	0.189170542	0.086860762	0.031128238
c8.c182	0.092587824	0.042515395	0.031136711
pcaac424.pcaac422	-0.355432724	0.163229619	0.031155496
lysopcac160.smc240	-0.131016625	0.0601687	0.031156513
methionine.c181	0.026465892	0.012156329	0.031183892
pcaec322.smohc222	-0.017130818	0.007869708	0.031208557
smohc161.pcaec381	0.050866198	0.023368298	0.031215175
pcaec446.c16	0.138882293	0.063803616	0.031215409
pcaac343.smc240	-0.105115259	0.04829458	0.031228491
smc240.pcaec402	-0.102833729	0.047267704	0.031303601
pcaec340.c16	0.152472709	0.070108145	0.031360094
pcaac363.c5	0.02666799	0.012263556	0.031379183
citruline.pcaec382	-0.021179436	0.0097425	0.031429035
smc160.glutamine	0.04995943	0.022983129	0.03144273
pcaac424.c12	0.166123145	0.076434658	0.031469046
pcaec421.c12	0.151443827	0.069699267	0.031513823
pcaac365.smc240	-0.092834236	0.042784955	0.031747639
c101.lysopcac203	-0.018489534	0.008525101	0.031821401
c4.glutamine	-0.024457332	0.011277809	0.031837716
glutamicacid.c12	-0.302708512	0.139594935	0.031849092
c142.pcaec340	0.059483156	0.027435248	0.031876165
pcaac404.lysopcac203	0.046330033	0.021371042	0.031894861
c141.pcaec381	0.098147839	0.04529609	0.031979051
c8.pcaac381	-0.048142921	0.022218527	0.03198016
smc240.c142	0.124877947	0.057643571	0.032012148
smc160.c14	-0.066540489	0.030715225	0.032013151
c51dc.pcaac362	0.032628742	0.0150622	0.032021079
pcaec445.serotonin	-0.31037847	0.143448672	0.032222498

pcaec386.transhydroxyproline	0.138279403	0.063918659	0.032247973
pcaac341.c18	0.095616734	0.044221242	0.032336702
pcaec406.c12	0.104503033	0.048333885	0.032346796
pcaac361.smc202	0.063750464	0.029490127	0.032374406
c8.phenylalanine	0.121007384	0.055992539	0.0324234
c142.lysopcac204	0.054726631	0.025323514	0.032426133
phenylalanine.lysine	-0.237880009	0.110115698	0.032491294
putrescine.pcaac405	0.041719846	0.019317663	0.032538675
c141.smc160	-0.182401691	0.0844798	0.032582781
pcaac380.pcaac320	0.316741523	0.146706652	0.03259096
c51.pcaec382	-0.023199064	0.010749042	0.032651791
methioninesulfoxide.pcaac402	-0.342351839	0.15866932	0.0326995
lysopcac203.pcaec421	-0.007739667	0.003587179	0.032703618
c2.pcaec381	0.029302481	0.013585666	0.032761162
pcaec360.lysopcac161	0.193763753	0.089900488	0.032884927
smohc141.pcaec381	0.035863807	0.016639723	0.032885003
lysopcac180.lysine	0.178441393	0.082795193	0.03289285
glutamicacid.pcaec383	0.27488377	0.12766069	0.03305094
pcaec321.transhydroxyproline	0.071837303	0.033369686	0.033088302
c142.pcaec405	0.080074327	0.037215458	0.033179023
pcaac281.glutamine	0.036219073	0.016833844	0.033185351
histidine.c14	0.110980741	0.051594256	0.033228305
pcaac320.smohc222	-0.008934522	0.004153669	0.033231012
c10.lysopcac204	0.052831825	0.024580124	0.033361945
alphaaminoadipicacid.c181	-0.063656284	0.029622538	0.033398952
serine.pcaec402	-0.102984519	0.047934668	0.033437695
pcaac343.asparagine	0.036743713	0.01710385	0.033450932
pcaec322.c16	0.092915879	0.043255104	0.033465587
c4.lysopcac204	0.116883195	0.054435591	0.033539136
leucine.asymmetricdimethylargir	0.096052157	0.044737388	0.033552233
lysopcac280.c18	0.080618372	0.037552243	0.033567626
pcaec420.asparagine	-0.011449437	0.005333532	0.033579241
pcaec362.pcaec365	0.043188826	0.020118895	0.033580017
pcaec321.smohc222	-0.008277206	0.003856088	0.033592266
pcaac405.c182	-0.064297186	0.029979962	0.033743472
smohc161.asparticacid	0.061920318	0.028878512	0.033784793
pcaec381.pcaec405	-0.311870335	0.145478872	0.033818764
c141.asparagine	-0.017536271	0.008182099	0.033859789
phenylalanine.smc202	0.118748896	0.055413913	0.033884725
smc261.pcaec385	-0.114918501	0.053628117	0.033890124
pcaac426.pcaec445	0.141676324	0.066158084	0.034004614
pcaec340.c18	0.07286308	0.034029768	0.034031275
lysopcac140.pcaec361	0.024488251	0.011441874	0.03410748

glutamicacid.pcaec381	-0.04011061	0.018746994	0.03416146
pcaac344.c5	0.024748358	0.011568	0.034177518
c182.pcaec381	0.05411155	0.025311991	0.03430952
pcaec360.pcaec381	0.013352312	0.006247819	0.034364799
pcaac320.pcaec340	0.182873173	0.085602894	0.034432748
c141oh.lysopcac204	0.05874499	0.027500747	0.034447129
pcaec405.pcaec365	0.084427669	0.03952785	0.034465405
pcaac381.pcaac361	0.13895987	0.065107015	0.034596327
smohc222.asparticacid	0.066516244	0.031180613	0.034685936
pcaec405.pcaec421	0.00754494	0.003536939	0.034692022
pcaac240.smc240	-0.136982742	0.064240201	0.034761542
smc261.c182	0.059656565	0.027985219	0.034814762
smohc241.pcaec381	0.017773175	0.00834022	0.034873228
creatinine.pcaac426	-0.096908734	0.045481515	0.034897737
pcaac426.pcaac405	-0.05956028	0.027954882	0.034909658
serine.c181	0.044755604	0.021010888	0.03494926
pcaac381.pcaac342	-0.064959226	0.030498777	0.034967658
c181.c16	0.168613464	0.079174804	0.034989599
c16.lysopcac180	-0.262174386	0.123175926	0.035089102
lysopcac203.pcaec360	-0.013713972	0.006443232	0.035091148
kynurenine.pcaec381	0.014975663	0.007037344	0.035125144
pcaac366.lysopcac203	0.118647672	0.055769936	0.035174094
lysopcac182.c181	0.040506367	0.019040179	0.035176913
methioninesulfoxide.pcaec382	-0.05673035	0.026673812	0.035227431
pcaac320.smc240	-0.098710711	0.046432445	0.035305295
glutamicacid.c182	-0.149376036	0.0702785	0.035340304
pcaec343.smc160	0.271230674	0.127639777	0.035384234
pcaac425.c182	-0.078818631	0.037108304	0.035465387
serine.pcaec381	-0.020793524	0.009790683	0.035483149
pcaac323.pcaac362	-0.052998927	0.024970254	0.035596188
smc160.pcaec381	0.012207277	0.00575268	0.035636103
glycine.c181	0.060535863	0.02853547	0.035686622
lysopcac281.c181	-0.02285298	0.010774678	0.035723927
lysine.c2	0.125351667	0.059116434	0.035772775
asymmetricdimethylarginine.c12	0.070250318	0.033130626	0.035774131
pcaac300.pcaec382	0.021081167	0.009942314	0.035778935
alanine.c141	-0.074837033	0.035305475	0.03583455
c8.pcaec403	0.06337616	0.029898957	0.035836488
c12.smohc221	-0.100667336	0.047511701	0.035912823
pcaec420.smohc222	0.00753605	0.003556833	0.035916064
c162.smohc222	0.011306351	0.005337299	0.035949412
pcaec342.pcaac361	0.207827419	0.098153557	0.036035265
c16.pcaec381	0.030248917	0.014289156	0.036074656

pcaec445.asparagine	-0.028286186	0.013365931	0.036128633
prolinr.pcaac420	0.250999983	0.118639139	0.03618326
pcaec422.smc240	-0.096431225	0.045584597	0.03620274
pcaec300.c18	0.073205253	0.034613739	0.036247449
histidine.c2	0.171364155	0.081043264	0.036285777
c141.phenylalanine	0.127097832	0.060127724	0.036344879
pcaac322.c2	-0.180979247	0.085618911	0.036346701
pcaac421.pcaec421	0.008869236	0.004197117	0.036399206
pcaac364.pcaec445	0.120105124	0.056841444	0.036415601
lysopcac182.asparagine	-0.016671843	0.007894259	0.036510713
pcaac426.glutamine	0.017889267	0.008476178	0.036629629
c51.pcaec381	0.024976785	0.011839671	0.036712926
c4.pcaec445	-0.197171921	0.093466504	0.03671627
lysopcac182.c3	-0.165968798	0.078689289	0.036749627
pcaac321.c5	0.020188007	0.009572257	0.036763158
isoleucine.asymmetricdimethylar	0.065746691	0.031181327	0.036805747
pcaac405.glutamine	0.016758428	0.007949024	0.036831292
pcaac420.smc240	-0.169333616	0.080347609	0.036895074
c10.asparagine	-0.011945053	0.005669076	0.036935454
pcaec360.c162	-0.219111015	0.104058257	0.037058894
pcaec380.pcaec421	-0.021578851	0.010248406	0.037065623
pcaec301.pcaec381	-0.019378285	0.009204233	0.037084602
c162.c16	0.170852151	0.08118997	0.037174628
pcaec384.c12	0.117542434	0.055880162	0.037252483
glutamicacid.c18	0.228243873	0.10852068	0.037274175
glycine.pcaac405	0.040313601	0.019169791	0.037296737
lysopcac181.c142	-0.098901563	0.047040415	0.037340775
c141.pcaec340	0.070995009	0.03377037	0.03735802
smc261.pcaac405	-0.047205836	0.022462373	0.037423568
pcaac381.smohc221	-0.107786325	0.051379811	0.037756417
pcaac360.glutamine	0.040368633	0.01924449	0.037770976
pcaec421.ornithine	-0.177875464	0.084813133	0.037807768
c181.smohc241	-0.181006718	0.086309791	0.037815726
pcaac364.glutamine	0.019434521	0.009273685	0.037951966
smohc241.pcaac362	0.023038246	0.010993682	0.037958628
c14.c181	-0.059549449	0.028418691	0.037972818
pcaec343.lysopcac182	-0.097179998	0.04637769	0.037975389
pcaec364.transhydroxyproline	0.104949372	0.050106415	0.038054439
smc240.pcaac362	0.025655029	0.01225596	0.038168598
pcaac322.pcaec403	-0.211269605	0.100948037	0.038206237
alphaaminoadipicacid.prolinr	0.066464355	0.031809372	0.038515738
arginine.smohc222	0.023045089	0.01103026	0.038533795
c12.asparagine	-0.024861886	0.011900248	0.03854021

lysopcac140.pcaac361	-0.152354763	0.072941922	0.038583901
pcaec361.smc240	-0.11002288	0.052679002	0.038598594
asparticacid.pcaec401	0.146550725	0.07017446	0.038614709
pcaac424.c18	0.146057634	0.07000225	0.03878974
lysopcac182.pcaec381	-0.018453531	0.008845394	0.038811827
c14.pcaec381	0.029114089	0.013956682	0.038830074
pcaac404.glutamine	0.016761334	0.008035731	0.038846828
pcaec322.pcaec401	0.163690944	0.078483301	0.038862649
c3.pcaac405	0.1741951	0.083522712	0.03886975
smc181.c16	0.096256546	0.046158773	0.038894242
citruline.pcaac322	-0.09272788	0.044467092	0.038896196
pcaec322.pcaac362	0.08496981	0.040792288	0.039111324
c0.pcaec381	0.027546075	0.013224908	0.039119996
pcaac401.smc240	-0.12517403	0.060097395	0.039123766
pcaac385.smc240	-0.07594075	0.036470268	0.039178303
pcaac425.pcaec342	0.217280676	0.104364519	0.039208429
h1.lysopcac182	0.081584567	0.03918836	0.039216132
c12.pcaec382	-0.034799682	0.016716005	0.039219751
c181.glutamicacid	-0.143549059	0.068975403	0.039280629
pcaec301.c18	0.118617893	0.057001675	0.039300025
smohc222.c182	0.054081557	0.025991568	0.03932048
c3.pcaec443	-0.120869182	0.058131208	0.039459079
lysopcac281.pcaec381	0.014055688	0.006762555	0.039532843
pcaec425.smohc221	0.097648764	0.046995012	0.039589391
c51dc.pcaec381	0.010858279	0.005227736	0.039664525
c14.smohc221	-0.210598746	0.101410013	0.039697003
citruline.smohc221	0.043846891	0.021117531	0.039732584
pcaac406.asparagine	-0.026927372	0.012974912	0.039825192
methionine.pcaec361	0.023684001	0.011413107	0.03984244
citruline.pcaec365	-0.042757062	0.020619431	0.039986541
asymmetricdimethylarginine.c14	-0.049590025	0.023920084	0.040031444
smc180.pcaec381	0.024459725	0.011798556	0.040035465
lysopcac161.c181	-0.058045077	0.028012894	0.040132684
pcaac364.pcaac381	0.109084468	0.052667392	0.040216936
citruline.pcaec381	0.011526853	0.005566804	0.040269572
c182.pcaec340	0.049673931	0.023999598	0.040351275
c12.c141oh	0.317062157	0.153240994	0.040421662
pcaac403.pcaec421	-0.023889929	0.011547156	0.040435105
c141oh.glutamine	0.025604733	0.012381878	0.040528782
c142.c18	-0.081819161	0.039573024	0.04056424
lysopcac161.pcaac405	0.102469789	0.049574308	0.040617326
smc261.c16	0.091667548	0.044352557	0.040636562
phenylalanine.asparagine	0.018760227	0.009080417	0.040711617

glutamine.c181	0.122639901	0.059367213	0.040733146
pcaec423.lysopcac203	-0.048853676	0.023649012	0.040733541
pcaac381.pcaec402	0.116248116	0.056275529	0.040741719
pcaac323.c2	-0.127178637	0.061567497	0.040743394
pcaec380.smc240	-0.099194819	0.048036442	0.040809382
h1.c181	0.038833146	0.018824539	0.041010686
pcaac385.c141oh	-0.209502547	0.101560282	0.041016566
lysopcac170.pcaec381	-0.024719692	0.011985541	0.041053235
c182.c18	-0.079306595	0.038462106	0.041103335
c51dc.pcaec443	-0.088402104	0.042877514	0.041123186
c101.lysopcac180	-0.123528751	0.05991524	0.041124222
creatinine.c182	-0.082179407	0.039878957	0.041221421
pcaac281.pcaec381	0.014808117	0.007186658	0.041242735
c10.pcaec403	0.058197551	0.028245203	0.041248649
pcaec404.c18	0.103412404	0.050198281	0.041283728
c2.pcaac405	0.048094762	0.023346448	0.041286872
prolinr.c12	-0.057284846	0.027831296	0.041457717
pcaec425.pcaec421	0.003633771	0.001766112	0.041534873
c181.pcaac362	-0.018069678	0.008784286	0.041578932
asparagine.c181	0.034277574	0.016667288	0.04162465
pcaec340.pcaac362	0.042046759	0.020449176	0.041665589
c8.lysopcac204	0.051301513	0.024953122	0.041689405
smohc222.smc202	0.194293404	0.094513075	0.041707528
pcaec342.lysopcac182	-0.051607166	0.025105697	0.041720704
smc180.pcaec360	0.028250767	0.013747867	0.041786925
pcaac240.pcaec405	-0.190450197	0.09271358	0.041859735
pcaac364.pcaec300	0.039755386	0.019355155	0.041877738
c16.c182	-0.215760973	0.105049016	0.041886295
prolinr.pcaec382	-0.035098718	0.017088789	0.041886732
c162.pcaec404	0.081301361	0.03960726	0.042006236
pcaac300.transhydroxyproline	0.078011977	0.038013918	0.042054839
kynurenine.pcaac366	-0.057223202	0.027885305	0.042065034
smc260.pcaac405	-0.074330084	0.036227795	0.042099542
isoleucine.pcaec381	-0.013947894	0.00679922	0.042133672
c141.pcaec402	-0.095692454	0.046650228	0.042145619
creatinine.transhydroxyproline	-0.058081279	0.028320506	0.042187039
pcaec300.pcaec382	0.028004792	0.013656144	0.042201465
smc240.pcaec381	0.014550571	0.007095801	0.042213439
pcaac240.pcaec402	-0.163055927	0.079518091	0.042217166
pcaec382.smc240	-0.139135062	0.067864262	0.042252292
pcaac362.smc202	0.067625144	0.032994242	0.042310922
pcaec385.pcaec381	0.014911167	0.007275539	0.042321952
pcaac323.c16	0.102291532	0.04991752	0.042349922

c182.pcaec421	0.003430452	0.001674127	0.042360962
c141.lysopcac203	-0.02054765	0.010033259	0.042474769
phenylalanine.pcaac322	0.111009119	0.054211913	0.042501142
asymmetricdimethylarginine.c16	0.17673905	0.08633415	0.042554648
pcaac364.pcaec443	-0.052604674	0.025700131	0.042583245
c162.pcaec405	0.058851399	0.028754897	0.042603919
pcaec322.pcaec381	0.013892601	0.006789033	0.042636502
methioninesulfoxide.pcaac362	0.037412541	0.018286525	0.042678641
c0.c16	-0.114869454	0.056146067	0.042679019
serine.pcaac362	0.032792508	0.016029117	0.042688517
pcaac426.c182	-0.063774468	0.031180047	0.042733195
pcaec301.prolinr	-0.054205709	0.026512046	0.042812458
lysopcac140.c141oh	-0.259768579	0.12707938	0.042855009
c18.putrescine	-0.062758164	0.030719691	0.042976857
smc202.smc181	-0.059057651	0.028915402	0.043027168
leucine.c181	0.032489109	0.015908148	0.043040763
smc160.pcaec365	0.087329654	0.042780152	0.043134828
methionine.pcaec381	-0.012981692	0.006360035	0.043157395
pcaec423.serotonin	-0.198730738	0.09737848	0.043190428
pcaac322.c142	0.048241452	0.023639441	0.043199377
lysopcac204.glutamine	0.025550434	0.012521466	0.043218378
lysopcac281.smc202	0.096431983	0.047258692	0.043220129
pcaec444.c3	-0.065356725	0.032029852	0.043222054
c51.pcaec404	-0.05969417	0.029258992	0.043251834
pcaec445.pcaec381	0.016222823	0.007954721	0.043333025
pcaec444.pcaec421	0.005209025	0.002554841	0.043384794
pcaec422.c12	0.157083773	0.077065754	0.043443128
pcaac342.c181	-0.013280606	0.006515907	0.043455908
pcaec401.pcaec342	0.254015835	0.124636397	0.043468815
c2.pcaec401	-0.101215435	0.049700573	0.043626319
pcaec341.c12	0.103632447	0.050888473	0.043630617
c10.pcaec385	-0.07919849	0.038890675	0.043632963
c12.pcaec381	0.086003611	0.042234022	0.043641161
pcaac405.pcaec381	0.014026637	0.006889332	0.043678275
pcaac420.pcaec421	0.004012678	0.001971131	0.043705844
pcaec445.pcaec421	0.003364728	0.001653382	0.043773719
c141oh.pcaec340	0.109096869	0.05362301	0.043829003
c102.pcaec381	0.00619437	0.003045258	0.043871146
asparticacid.pcaec443	-0.138503938	0.068099533	0.043897509
leucine.pcaec381	-0.014585224	0.007173501	0.043962712
pcaec341.pcaec381	0.005677293	0.002792353	0.043967972
asparticacid.pcaac405	-0.087173291	0.04287699	0.043973626
pcaac362.pcaec381	0.008154264	0.004011155	0.043994632

lysopcac180.putrescine	-0.089812517	0.044182007	0.044006105
pcaec401.c12	0.113529946	0.055850482	0.044009905
c4.c18	-0.136156142	0.067033277	0.044171628
pcaac363.c181	-0.012927776	0.006364906	0.044178818
c14.lysopcac204	0.060290706	0.029692944	0.044243637
c162.pcaec402	-0.057587729	0.028363219	0.044254557
alanine.c142	-0.06781886	0.033404231	0.044266796
c0.c141	0.119402106	0.058818961	0.044292982
pcaec422.smc202	0.108132478	0.053308402	0.044454129
pcaec380.c12	0.133752866	0.065956212	0.04450876
pcaec361.pcaac362	0.044451043	0.02192116	0.044522756
pcaec364.glutamine	0.035779913	0.01765045	0.044587998
c141oh.c12	0.129271038	0.063787566	0.044645249
lysopcac204.c14	0.089499469	0.044165603	0.044659246
pcaec402.pcaac362	0.022627387	0.011168252	0.044701434
c101.smc202	-0.093544795	0.046178391	0.044734572
pcaec341.pcaec382	0.038121632	0.018819034	0.044737803
c14.glutamine	0.029873488	0.01475046	0.04478329
pcaec383.c12	0.186405696	0.09206884	0.044848257
pcaec301.glutamine	0.02563896	0.012663566	0.044849295
smc181.smc202	0.135842255	0.067116324	0.044916165
pcaec423.c12	0.114139188	0.056393789	0.044917729
c51dc.lysopcac204	0.045852138	0.022656934	0.044939632
smc202.pcaec385	-0.081872893	0.04045961	0.044959084
pcaac426.serotonin	-0.205044677	0.101393679	0.045095792
pcaec340.pcaec381	0.008148661	0.004032052	0.045231111
pcaac361.pcaec381	0.005558276	0.002751219	0.045302059
pcaac240.pcaec381	0.069088745	0.034207216	0.045363346
c102.c18	-0.114088976	0.056499054	0.045405948
smc261.pcaec382	0.042241204	0.020921927	0.045439182
pcaac320.pcaec381	0.004454293	0.002206389	0.045457721
pcaec342.pcaac362	0.031720248	0.015719149	0.045550453
smc240.smohc221	-0.083986627	0.041620819	0.045554389
citruline.c182	-0.152569668	0.075609234	0.045557407
pcaac240.asparagine	0.033843677	0.016774301	0.045586966
smohc141.pcaec385	-0.124386445	0.06166322	0.045629296
putrescine.smc181	0.052201824	0.025893425	0.045752477
pcaec420.pcaec382	0.02977643	0.014772061	0.045784281
pcaac341.smc240	-0.063650448	0.031582358	0.045820997
pcaec404.pcaac403	-0.2431403	0.12066849	0.045867207
pcaac321.alphaaminoadipicacid	0.02591322	0.01286149	0.045883454
pcaec425.pcaec365	0.077435683	0.0384437	0.045939709
pcaac385.pcaec381	0.01243522	0.006174802	0.045981891

phenylalanine.pcaec360	0.012014175	0.005966253	0.046000715
lysopcac140.prolinr	-0.088916654	0.04415864	0.046012986
pcaac321.pcaec365	0.087544622	0.043482989	0.046041294
c51dc.smc202	0.095632565	0.047509586	0.046083533
kynurenine.pcaac425	0.198886106	0.098806557	0.04608644
pcaac360.pcaec342	0.319549044	0.158767836	0.046108024
pcaac402.smc240	-0.149618935	0.074361935	0.046176814
lysine.c181	0.04073642	0.02025216	0.046238364
c3.pcaec403	-0.126895583	0.063090386	0.04625232
pcaec341.pcaec340	0.123506927	0.061419619	0.046301426
pcaac380.pcaec381	0.013575727	0.006751773	0.046320742
pcaac322.pcaec381	0.006931254	0.003448109	0.046377491
c0.c181	-0.053095703	0.026415277	0.046390671
smohc221.pcaec443	-0.139242053	0.069285205	0.046427543
prolinr.pcaec443	0.125019137	0.062211009	0.046437801
pcaec425.leucine	-0.076971403	0.038307684	0.046470448
pcaac342.pcaec381	0.005703975	0.002839083	0.04649233
pcaec321.c16	0.079609743	0.039627032	0.046504645
lysopcac140.c181	-0.058578578	0.029158671	0.04650642
smc160.pcaec385	-0.131236294	0.065332279	0.046528763
pcaac425.c12	0.115193229	0.057358466	0.046576857
creatinine.smohc161	0.074427377	0.037065232	0.046608418
spermidine.glutamine	0.098945225	0.04927855	0.046622887
pcaec446.pcaec381	0.009636979	0.004800363	0.046657753
pcaec424.c12	0.105305809	0.052456099	0.046663006
pcaac321.pcaec381	0.005151687	0.002566618	0.046696897
creatinine.lysopcac280	-0.013920795	0.006935604	0.046701176
pcaac343.pcaec381	0.006745604	0.003361137	0.046723619
ornithine.pcaec381	-0.056436085	0.028124601	0.04675561
pcaac424.pcaec422	-0.317914644	0.158479893	0.046822526
pcaac341.pcaec381	0.004652238	0.002319735	0.046878838
methioninesulfoxide.c181	0.064392924	0.032118702	0.0469505
smc160.asparticacid	0.049130933	0.024510736	0.046991259
smc241.pcaec381	0.01912127	0.009540729	0.047023029
lysopcac161.pcaec423	-0.079196214	0.039528227	0.047092161
pcaec422.pcaac362	0.023122929	0.011542605	0.047121254
pcaec386.glutamine	0.042607791	0.021270847	0.047138684
pcaec444.pcaec381	0.008124751	0.004056208	0.047145874
pcaac342.c5	0.02564361	0.012803114	0.047159074
lysopcac181.asparagine	-0.012455823	0.006219412	0.047179365
pcaac321.c181	-0.011826137	0.005905422	0.047194966
creatinine.pcaec381	0.029469993	0.01471635	0.047201017
pcaec384.pcaec340	0.172019339	0.085904557	0.047210475

pcaac363.pcaec381	0.005528655	0.002761965	0.04729068
c101.c51	0.118451097	0.059182177	0.047317373
pcaec446.asparticacid	0.052935921	0.026449253	0.04732294
pcaac240.pcaec365	0.034066605	0.017023925	0.047357073
c101.pcaec381	0.033121604	0.016558904	0.047452335
pcaac406.lysine	0.200783325	0.100390091	0.047473969
pcaec402.c18	0.083633438	0.041821883	0.047504399
pcaac421.smohc221	0.096963075	0.048490326	0.047517037
pcaac322.c181	-0.015738949	0.007872094	0.047550193
pcaac383.c181	-0.014604579	0.007304956	0.047557259
pcaac300.asymmetricdimethylar[illegible]	0.056297023	0.028162068	0.047582747
c101.pcaec421	0.005366544	0.002685262	0.047639624
smohc222.pcaec300	0.031551953	0.015788654	0.047653243
lysopcac203.pcaac380	0.173693434	0.086917833	0.047656588
pcaec383.arginine	-0.241876344	0.121039775	0.047661243
lysopcac160.c18	0.153958234	0.077103135	0.047830214
pcaac322.pcaac402	-0.119811724	0.060011809	0.047864766
c4.pcaec381	0.011034274	0.005527354	0.047883044
pcaec363.pcaec381	0.008973263	0.004495497	0.047910312
alanine.pcaec381	-0.01040427	0.005213707	0.047964845
pcaac384.pcaec381	0.011361278	0.005695458	0.048049253
pcaac344.spermidine	-0.142762643	0.071614804	0.048194857
pcaac343.smc202	0.099545662	0.049940549	0.048216588
c4.c181	-0.013719315	0.006883254	0.048232057
pcaac402.c12	0.209152929	0.104983681	0.048332024
pcaec445.spermidine	-0.136073821	0.068302473	0.048334061
pcaec321.pcaac426	0.14333798	0.071949172	0.048335422
pcaec341.lysopcac203	0.035751772	0.017945852	0.04833644
pcaac363.pcaac342	-0.205362938	0.103091046	0.04835292
pcaac300.pcaec381	0.008594985	0.004315445	0.048394486
pcaec321.pcaec381	0.006496671	0.003262013	0.048401771
smohc141.pcaec365	0.06394403	0.032107514	0.048407842
pcaac341.transhydroxyproline	0.049447209	0.024831969	0.048439811
pcaec362.lysopcac182	-0.075716462	0.03802984	0.048472861
methionine.glutamine	-0.031089816	0.015615475	0.04847432
lysopcac281.pcaac366	-0.089092653	0.044749417	0.04847863
pcaac405.pcaec445	0.060006694	0.030141685	0.048490028
c10.pcaac381	-0.042155794	0.021176407	0.048503891
pcaec343.pcaec381	0.009221961	0.004633362	0.048543711
c51dc.c181	-0.02382803	0.011976567	0.04863118
pcaac342.pcaec405	0.11967831	0.060156478	0.048642809
c5.lysopcac204	0.111802438	0.056208323	0.048684947
pcaec445.asparticacid	0.050308164	0.025294868	0.04870776

pcaac363.smc240	-0.086921465	0.043717684	0.048777467
pcaac342.smc240	-0.113207346	0.056941057	0.048788122
c14.pcaec382	-0.047738628	0.024016114	0.048830083
pcaac320.c12	0.076716768	0.038615924	0.048954887
c51.c181	-0.027498152	0.013842004	0.048964814
pcaac240.lysopcac170	-0.07816509	0.039358824	0.049033542
smc260.pcaac362	0.012339094	0.006213654	0.049051274
glutamicacid.pcaac405	-0.114127662	0.057476054	0.049067843
pcaec360.c18	0.101879752	0.051316241	0.049104316
c181.pcaec402	-0.045500948	0.022927221	0.049188716
lysopcac181.smohc222	0.008436916	0.004251657	0.04921112
putrescine.c14	0.115659887	0.058299008	0.049264657
pcaec365.pcaac361	0.232248169	0.117084534	0.049300139
pcaac404.pcaec381	0.007429096	0.003746082	0.049348679
smc202.c12	0.085945612	0.043352757	0.049426967
c3.pcaec381	0.015915408	0.008029381	0.049463778
pcaac383.pcaec381	0.006309171	0.003184073	0.049539477
c182.pcaac362	-0.015366736	0.007755397	0.04954553
glutamine.pcaec300	0.045996187	0.023216641	0.049574048
c102.tyrosine	0.144323369	0.072861683	0.049618031
pcaac420.pcaec381	0.015476715	0.007817315	0.049730068
pcaac281.pcaac322	-0.136882007	0.069140536	0.049733992
pcaac344.pcaec443	-0.055572302	0.028079286	0.049807277
pcaec300.pcaac362	0.037767863	0.019083934	0.049816524
pcaac425.pcaac405	-0.06611449	0.033410705	0.049839104
pcaac281.c16	0.107987152	0.054580655	0.049879432
c181.pcaec340	0.033173846	0.016771052	0.049930253
pcaec342.pcaec381	0.010889837	0.005506833	0.049990581
putrescine.c142	0.107910759	0.05457752	0.050026237
pcaac425.pcaec381	0.010590416	0.005356516	0.050036834
pcaec365.asparticacid	-0.066122806	0.033447796	0.050061202
pcaec423.pcaec381	0.012549293	0.006349855	0.050128051
pcaac320.c18	0.065006901	0.032894712	0.050139533
c101.lysopcac204	0.037752208	0.019108597	0.050201992
c4.smohc221	0.06361142	0.032203232	0.050242646
alphaaminoadipicacid.lysopcac2(	0.04265058	0.021598113	0.050308664
pcaac401.pcaec381	0.014981177	0.007586933	0.050324044
pcaec446.pcaec421	0.003259701	0.001651134	0.050367985
pcaac360.c12	0.099995604	0.050657627	0.05039909
smc261.asparagine	-0.024486962	0.012407414	0.0504422
lysopcac204.pcaec381	-0.034646357	0.017558121	0.050480955
pcaec342.c16	0.122542395	0.062103925	0.050487355
histidine.prolinr	-0.09483354	0.048078477	0.050569068

pcaec322.transhydroxyproline	0.094333377	0.04782704	0.050579226
pcaac322.smc202	0.063572119	0.032236708	0.05061899
c102.lysopcac203	-0.05740072	0.029110397	0.050643519
c16.lysopcac204	0.069014573	0.035001741	0.050652982
c16.pcaec405	-0.151332363	0.076781333	0.050744805
pcaec422.pcaec381	0.009112264	0.004623544	0.050757839
pcaec384.pcaec381	0.012486488	0.006335694	0.050760604
c181.asparagine	-0.0847359	0.043006258	0.050818537
pcaac364.pcaec381	0.006229713	0.00316199	0.050833367
pcaec362.pcaec381	0.015690081	0.007972002	0.051070124
pcaac384.pcaac405	-0.156894664	0.079729329	0.05110581
pcaec361.pcaec381	0.013907805	0.007068866	0.051148661
pcaec343.pcaec361	-0.01966058	0.00999307	0.051154702
pcaac364.spermidine	-0.134885936	0.068570939	0.05119207
pcaac364.c8	-0.095812456	0.048724719	0.051273375
c12.lysopcac203	-0.030166015	0.015341015	0.05127801
c181.pcaec421	0.002257404	0.001148053	0.051286744
pcaec404.pcaec402	-0.240982082	0.122566264	0.051304465
pcaac344.pcaec381	0.00713126	0.003627276	0.051319325
lysopcac181.smc240	-0.137102265	0.069741013	0.051334825
smohc221.pcaec381	0.019260378	0.009798593	0.051364069
c2.c181	-0.041524839	0.021132363	0.051438785
lysopcac170.lysopcac203	-0.069179859	0.035211054	0.051470167
c8.pcaec421	0.008593193	0.004374302	0.051499237
lysopcac170.c2	0.162308088	0.082642181	0.051556155
pcaac406.pcaec381	0.00853625	0.00434648	0.051560913
smc241.smohc221	-0.181035567	0.09218178	0.051566533
kynurenine.asparticacid	0.094285079	0.04801681	0.051603215
creatinine.pcaec365	-0.069131675	0.035208088	0.051611186
creatinine.smc202	-0.082409972	0.041978165	0.051652754
glycine.pcaac426	-0.063814675	0.032506149	0.051653428
c102.pcaec361	-0.031991874	0.016305734	0.05178928
pcaac320.pcaac360	0.139739356	0.071224119	0.051793365
pcaec382.c12	0.193580642	0.098682074	0.051829527
pcaec445.pcaac426	-0.160556858	0.081855261	0.05185155
pcaec402.pcaec365	0.054604037	0.027843338	0.051893492
pcaec424.pcaec381	0.01353022	0.006899903	0.051915632
pcaac366.smohc222	-0.024629378	0.012560336	0.051920796
arginine.pcaec381	-0.019255204	0.009821349	0.05196086
pcaec443.pcaac362	0.102915916	0.052494024	0.051963148
phenylalanine.pcaac405	-0.083773554	0.04273567	0.051993126
isoleucine.lysine	-0.263317062	0.134329773	0.051998299
pcaec365.transhydroxyproline	0.133187254	0.067947186	0.052006578

pcaac404.asparagine	0.021034142	0.01073125	0.05201549
citruline.pcaec385	0.16822543	0.085831539	0.05203134
pcaac363.pcaec443	-0.045510359	0.023223369	0.052063185
pcaec423.pcaac362	0.026757727	0.013655394	0.052084556
pcaac421.pcaec381	0.013547168	0.006914968	0.052130794
methionine.c141	-0.074592511	0.038076692	0.052142721
pcaec321.lysopcac161	0.168489945	0.086018269	0.052170878
c4.asparticacid	0.082111401	0.041953846	0.052358899
pcaac300.pcaac405	0.066560079	0.034010221	0.052373516
pcaec406.pcaec381	0.017460423	0.008922705	0.052398234
pcaac300.pcaec365	0.061982962	0.031677557	0.052418358
pcaec421.smc240	-0.087049841	0.044503541	0.052497269
lysopcac181.pcaac405	0.08381101	0.042859217	0.052559802
lysopcac280.pcaac402	-0.188995932	0.096651159	0.052565947
c181.valine	-0.130462506	0.066738763	0.05263994
pcaac323.pcaec365	0.070263952	0.035962733	0.052762324
pcaac380.pcaec361	-0.022304209	0.011418088	0.052808821
pcaac386.pcaec381	0.00649871	0.003328438	0.052920186
pcaec444.lysopcac203	-0.099198654	0.050814519	0.052957215
lysopcac161.pcaec381	0.035491281	0.018184901	0.053015043
smc202.pcaac344	0.094274435	0.048323523	0.053109714
pcaac300.smc202	0.06174952	0.031676312	0.05329157
pcaec342.pcaec360	-0.013445048	0.0069018	0.053453552
histidine.pcaac405	-0.131252192	0.067383816	0.053480127
c10.pcaec421	0.006444873	0.003309323	0.05352123
pcaac403.c12	0.133633941	0.06863408	0.053574557
prolinr.c182	-0.122695018	0.063021191	0.053594452
lysopcac170.asparagine	-0.035490165	0.018239071	0.053722191
pcaec382.pcaec381	0.026619681	0.013681664	0.053744697
pcaac421.lysopcac203	-0.056312726	0.028953645	0.053832129
pcaac381.c18	0.135766265	0.069819942	0.053881655
pcaac422.lysopcac203	-0.050068596	0.025750023	0.053895059
asymmetricdimethylarginine.c14	-0.204765187	0.105310943	0.053897878
pcaec386.pcaec381	0.019155499	0.009853155	0.053933026
pcaec384.citruline	0.020234671	0.010409519	0.053961726
c142.c101	-0.043163749	0.022213462	0.05405045
pcaec322.c2	-0.120562205	0.062104682	0.054277904
prolinr.pcaec360	0.009088988	0.004682356	0.054297477
c5.pcaec404	-0.063961181	0.03295601	0.054335391
smc241.pcaec443	-0.118794983	0.061209707	0.054337612
asparticacid.asparagine	0.015453054	0.007965649	0.054438787
asparticacid.pcaac366	-0.029858302	0.015391678	0.054446495
pcaec380.pcaac362	0.095161283	0.049059622	0.054470102

pcaac366.asparticacid	-0.068968355	0.035561446	0.054506111
smc202.pcaec423	-0.130832675	0.067464401	0.054521978
pcaac420.pcaac361	0.13551342	0.069892711	0.054572015
smc161.pcaec340	0.086890704	0.044829112	0.054647418
pcaac323.pcaec405	0.12947246	0.066814986	0.054707714
pcaec421.pcaec381	0.018248096	0.009417322	0.05471508
pcaac426.pcaec381	0.006946541	0.003585215	0.054735296
pcaac404.pcaac426	0.15380271	0.07939792	0.054789517
pcaec403.pcaec300	-0.041607258	0.021480652	0.05480781
lysopcac170.c181	0.051583728	0.026631389	0.054809315
pcaec401.pcaec381	0.013051714	0.006738711	0.054824855
lysine.c141	-0.053589802	0.027674227	0.054871152
pcaac323.c12	0.115564862	0.059682484	0.054886432
pcaec425.pcaec381	0.027070381	0.013993118	0.055106634
smohc161.valine	-0.18808189	0.097257596	0.055193095
pcaec365.pcaec381	0.014336058	0.007413216	0.055193279
pcaec341.pcaec300	0.027403208	0.014174028	0.055256903
pcaec322.lysopcac182	-0.060745234	0.031428797	0.055325364
pcaac425.pcaec406	0.057268209	0.029634692	0.05536484
histidine.spermidine	-0.144843263	0.074969503	0.055419997
c142.pcaec402	-0.089639983	0.046402787	0.055451031
pcaec446.asparagine	-0.024469848	0.012669329	0.055495434
pcaac281.pcaec365	0.055042532	0.028505198	0.055552778
smohc241.smohc221	-0.046199865	0.023929029	0.055585384
leucine.c14	0.076544073	0.039650928	0.055617279
pcaec384.tyrosine	-0.155736261	0.080699564	0.055694908
pcaec422.pcaec365	0.06430338	0.033329666	0.055759278
c0.pcaac366	-0.106130545	0.055013025	0.055774849
pcaec364.c12	0.091932676	0.047664538	0.055830719
pcaac425.pcaac320	0.256577617	0.133049299	0.055868784
pcaec360.smohc222	-0.018841774	0.009771607	0.055896865
kynurenine.histidine	-0.175197155	0.09088172	0.055955554
pcaac380.glutamine	0.035296979	0.018313623	0.056004225
pcaec300.pcaec381	0.014456157	0.007501108	0.05602422
alphaaminoadipicacid.pcaec381	0.029401246	0.015260267	0.056093357
c141oh.c181	0.079393122	0.041218644	0.056157283
c2.pcaec406	-0.056468477	0.029324872	0.056223946
c8.c12	0.212201399	0.110265952	0.056371243
glutamine.pcaec381	-0.066862018	0.034746792	0.056394799
c12.smc202	-0.097547435	0.050704213	0.056446905
pcaac342.pcaac381	0.070460585	0.036625409	0.056451418
pcaac343.pcaec365	0.05651432	0.029379189	0.05647668
pcaec363.pcaac361	0.16342196	0.084983417	0.056556625

creatinine.c2	0.105738406	0.05498955	0.056569892
pcaac341.smc202	0.062963135	0.032774981	0.056799623
pcaec365.pcaac362	0.069127147	0.035984102	0.056802959
c141.pcaac366	-0.045143192	0.023512461	0.056940276
prolinr.citruline	0.04021458	0.020945769	0.056944178
pcaec365.pcaec361	-0.025078691	0.01306384	0.056974183
c142.c141oh	0.287633512	0.149832881	0.056975122
c18.pcaec406	-0.028932101	0.01507172	0.056983694
creatinine.pcaac405	0.030689979	0.015989507	0.057015067
pcaec444.pcaec300	-0.044612741	0.023243311	0.057015408
pcaac364.pcaec365	0.062579642	0.032606966	0.05703698
smc181.asparticacid	0.058583777	0.030529727	0.057075485
smohc141.smohc222	-0.018260532	0.009518966	0.057149346
pcaac380.lysopcac203	-0.041333299	0.021556193	0.057259933
pcaac402.c182	-0.115133097	0.06005815	0.057316377
serine.prolinr	-0.085506259	0.04462796	0.057450797
c142.c182	0.226085926	0.11800136	0.057453268
alphaaminoadipicacid.pcaac362	-0.02218207	0.011577775	0.057458682
pcaec360.smc240	-0.098874333	0.051613944	0.057492941
citruline.lysopcac203	-0.020138492	0.010523055	0.057737868
pcaac403.pcaec381	0.020173808	0.010545665	0.057835213
pcaac344.pcaac384	0.351462914	0.183746169	0.057865208
lysopcac180.c2	0.126842717	0.066318072	0.057880901
leucine.pcaac362	0.025838089	0.013510463	0.057905654
creatinine.pcaac385	0.074130689	0.038762435	0.057907466
putrescine.pcaac406	-0.114708053	0.059984332	0.057925069
h1.pcaec420	0.161296454	0.084358984	0.057960727
pcaec386.pcaec361	-0.024270005	0.01269562	0.058004906
pcaac403.citruline	0.059646698	0.031201279	0.058006013
pcaac422.pcaac362	0.031653176	0.016561634	0.058062974
c10.pcaac366	-0.026518973	0.013878495	0.058119912
serotonin.smohc221	-0.074174392	0.038824662	0.058158732
pcaac323.pcaec340	0.043010985	0.022513554	0.058165
leucine.lysopcac203	0.02654264	0.013896061	0.058212368
smohc161.pcaec385	-0.129674209	0.067939507	0.058396018
asymmetricdimethylarginine.pro	0.026019201	0.013633423	0.058420158
c8.pcaec365	-0.055015826	0.028837672	0.058512717
alanine.asparagine	0.029594772	0.015517634	0.058591799
pcaec341.pcaac362	0.018547415	0.009725449	0.058600828
phenylalanine.c12	-0.082090383	0.043058822	0.058683234
histidine.c181	0.031656977	0.016605381	0.058688709
glutamicacid.lysopcac280	-0.016875486	0.008854118	0.058751653
pcaac320.pcaac362	0.019574147	0.010270475	0.058762385

pcaec364.pcaec381	0.015403783	0.008084549	0.058831771
c102.c14	-0.095980014	0.050387856	0.058898858
pcaac321.prolinr	-0.09909601	0.05202558	0.058907887
pcaac405.smc161	-0.097294192	0.051110178	0.059057433
pcaec363.pcaac362	0.023163087	0.012173429	0.059170442
c142.pcaac362	-0.010946386	0.005752932	0.059171597
pcaec365.glutamine	0.036317942	0.019087726	0.059179951
pcaec384.c142	-0.100732215	0.052943101	0.059184589
lysopcac182.alphaaminoadipicac	-0.018678799	0.009818361	0.059212889
c181.pcaec403	0.062416215	0.032819781	0.059298475
pcaac341.pcaec340	0.175876008	0.092504773	0.059367408
lysopcac181.pcaec443	0.165703606	0.087159548	0.05938213
pcaac403.pcaec443	-0.160433339	0.084415457	0.05946562
asparticacid.pcaec381	0.017499304	0.009208472	0.059488446
pcaac362.pcaac405	0.121178563	0.063789325	0.059578435
pcaec381.pcaec300	-0.020898274	0.011001091	0.05958026
lysopcac204.pcaac362	-0.028372976	0.014937895	0.05961454
c181.pcaac361	-0.086214239	0.045397253	0.059652831
lysopcac161.transhydroxyprolin	0.141973872	0.07478755	0.059751611
c162.lysopcac203	-0.026952203	0.01420468	0.059877213
pcaac401.pcaec421	0.010121936	0.00533507	0.059900075
pcaec401.pcaec421	0.016376266	0.00863186	0.059907617
pcaac365.smohc241	0.123925131	0.065324092	0.059921859
smc202.asparticacid	0.069236012	0.036499737	0.059947263
lysopcac203.c101	-0.075519178	0.039814153	0.059960368
pcaec402.citruline	0.045641835	0.024068745	0.060024429
histidine.c141	-0.082767557	0.043647989	0.060032434
pcaec446.tyrosine	-0.084781895	0.044728254	0.060134165
pcaec301.lysopcac203	0.04210544	0.022214213	0.06014226
pcaac406.pcaec444	0.307092542	0.162045149	0.060185408
lysopcac161.pcaec405	-0.083280072	0.043947231	0.0601992
pcaec420.pcaec381	0.007664246	0.004045859	0.060287191
pcaec425.pcaac402	-0.214644155	0.113314819	0.060302557
pcaec424.smohc221	0.109006473	0.057548706	0.060311679
pcaec364.lysopcac182	-0.057583227	0.030401589	0.060321609
kynurenine.pcaac364	0.133341851	0.070415582	0.060381161
pcaec382.smohc222	-0.051014348	0.026944429	0.060424586
pcaac321.smc202	0.078452362	0.041438441	0.060436711
pcaac426.pcaec443	-0.056611862	0.029902676	0.060439762
pcaec446.smohc221	0.039442049	0.020841951	0.060542819
smc161.asparticacid	0.051899582	0.027425188	0.060546833
pcaac344.c181	-0.015746604	0.008322833	0.060604484
c3.glutamine	-0.033972697	0.017957994	0.060629954

isoleucine.c181	0.028985017	0.015323933	0.060670292
lysopcac281.c18	0.071945399	0.038058882	0.060820522
pcaec360.c182	-0.09354888	0.049487388	0.060822242
smc241.pcaec402	-0.113064162	0.05981934	0.060857856
pcaec420.pcaec365	-0.041463486	0.021943476	0.06093003
creatinine.pcaec342	0.143073612	0.075720721	0.060939182
pcaac384.asparagine	-0.03918791	0.020743345	0.060981175
smc202.pcaec343	-0.156118212	0.082640161	0.060987573
acetylornithoine.pcaec421	-0.017698455	0.009368596	0.060988488
pcaec384.pcaac405	-0.096776488	0.051234308	0.061018967
serotonin.pcaec382	-0.024239731	0.012834915	0.06106256
prolinr.c181	0.038274824	0.020270418	0.061112152
pcaac381.smc240	-0.113170503	0.05994653	0.061159981
pcaac421.pcaac362	0.02554802	0.013537987	0.061257744
serine.smohc221	-0.09564486	0.050712192	0.061407412
c51.alphaaminoadipicacid	-0.026140102	0.013862034	0.061448093
pcaec422.pcaec300	-0.06862365	0.036404013	0.061540049
pcaac343.pcaec443	-0.050618485	0.026853664	0.06155125
c162.alphaaminoadipicacid	-0.017393487	0.009227737	0.061559602
pcaac341.c5	0.017900839	0.009504107	0.061754382
spermidine.c12	0.356727454	0.189552447	0.061964676
pcaec444.pcaac420	0.294540981	0.156511173	0.061968683
smohc222.pcaec381	0.027974402	0.01486717	0.062008951
pcaac384.glutamine	0.019137487	0.010173181	0.062070908
pcaac322.alphaaminoadipicacid	0.086583492	0.046027519	0.062077195
lysopcac203.c2	0.17018824	0.090519349	0.062213489
c141oh.pcaac366	-0.01550227	0.008245968	0.062233996
pcaec321.pcaac362	0.009995169	0.005319509	0.062373877
methioninesulfoxide.pcaec421	-0.009513821	0.00506369	0.062392148
pcaac381.pcaec300	-0.027740446	0.014766435	0.062421973
pcaac403.pcaac362	0.033977652	0.018086946	0.062427744
serine.pcaec401	0.13671685	0.072780434	0.06244008
pcaec301.asparagine	0.029646688	0.015783438	0.062459641
pcaec385.c12	0.098958866	0.05268841	0.062480473
acetylornithoine.pcaac366	0.027519984	0.014653111	0.062493159
pcaec420.histidine	-0.117594994	0.062634179	0.062577179
c162.pcaec340	0.050912201	0.027123105	0.062633857
pcaac402.c18	0.131707712	0.070168039	0.062640182
pcaec300.pcaac322	-0.138489383	0.073792437	0.062680329
pcaec420.lysopcac204	-0.068810225	0.036674102	0.062746714
pcaec402.c12	0.094517225	0.050385156	0.062797599
pcaac240.kynurenine	0.200368482	0.106819683	0.062815677
pcaac363.pcaec403	-0.149793568	0.079876079	0.062876498

serotonin.pcaac406	0.119988812	0.063986344	0.062890244
alanine.smc202	0.086736507	0.046266204	0.062959318
pcaac366.glutamicacid	0.138872699	0.07411029	0.063078949
pcaec445.valine	0.11217364	0.059863748	0.063085813
c4.c5	0.03462777	0.018480696	0.063098231
smohc241.pcaac405	-0.064079877	0.034210971	0.063188156
c10.c2	0.102450423	0.054701887	0.063215054
pcaac386.smc202	0.058261823	0.031116557	0.063286452
creatinine.asparagine	-0.012898435	0.006889945	0.063329263
pcaac422.pcaec300	-0.05804968	0.03101365	0.063373919
pcaac342.pcaec361	-0.022102845	0.011810699	0.063418635
c12.pcaec300	-0.023054079	0.012319061	0.063420074
creatinine.pcaac362	-0.063300686	0.033831907	0.063473147
lysopcac203.pcaec401	-0.113939961	0.060906618	0.063515489
pcaac364.transhydroxyproline	0.053065371	0.02836677	0.063521668
pcaec384.smohc222	-0.013407366	0.007167492	0.063536747
c0.smohc161	-0.032028258	0.017123814	0.06356291
c10.pcaac323	-0.239373712	0.127980459	0.063562918
c101.lysopcac182	-0.084371645	0.045112461	0.063582664
pcaac343.smohc221	0.046201224	0.024706863	0.063621294
pcaac341.c12	0.097144668	0.051953366	0.063639727
c182.lysopcac204	0.049998917	0.026743405	0.063676721
putrescine.pcaec381	0.045178506	0.024171317	0.063744462
pcaec362.pcaec340	-0.134553171	0.071990135	0.063750857
pcaec340.asymmetricdimethylar[illegible]	0.05167257	0.027648714	0.063772462
lysopcac204.c182	-0.055873181	0.029898261	0.063789158
c102.transhydroxyproline	0.050405688	0.026977562	0.063837806
pcaac385.pcaac420	-0.131364345	0.07031712	0.06387429
pcaac300.pcaac362	0.020963686	0.01122582	0.063975086
smc241.asparticacid	0.067487009	0.03614618	0.064030604
c142.pcaac366	-0.018168409	0.009732135	0.064060222
arginine.smc240	-0.074175097	0.039739213	0.064102538
pcaec420.citruline	0.021863691	0.011714524	0.064126776
pcaec383.asparagine	0.024296121	0.013019763	0.064166091
lysopcac140.asymmetricdimethy	0.029842322	0.015992464	0.064176173
pcaac281.pcaac366	-0.017448035	0.009351194	0.064199038
smohc161.pcaec365	0.056335483	0.030202442	0.064283575
lysopcac160.c181	-0.0704427	0.03776819	0.064301838
pcaec321.lysopcac203	0.028049438	0.015040624	0.064333074
pcaec301.asparticacid	-0.1343898	0.072074589	0.064378069
methioninesulfoxide.lysopcac28[illegible]	-0.045868682	0.02460766	0.06446194
smc240.pcaac366	0.038393743	0.020597527	0.064462288
pcaec363.citruline	0.03613277	0.01938698	0.064495248

c8.pcaac366	-0.022133879	0.011879927	0.064584762
lysine.smohc221	0.139562112	0.074911323	0.064599168
lysopcac180.c10	0.076174142	0.040899785	0.064680368
pcaec321.lysopcac182	-0.04696258	0.025217527	0.064702943
pcaec364.smc160	0.338921391	0.181993994	0.064707519
pcaec420.lysopcac182	-0.085377873	0.045852184	0.064741981
pcaac406.lysopcac203	-0.029147075	0.015654832	0.064765566
smohc161.c16	0.136842883	0.073500346	0.064773923
pcaec361.c18	0.07131528	0.038315871	0.06485246
lysopcac160.c10	0.075725181	0.040687056	0.064864548
c12.prolinr	-0.087685043	0.047116327	0.064882811
pcaec402.pcaec381	0.024593342	0.013215486	0.064894813
pcaac360.pcaec381	0.024025873	0.012912028	0.064925099
smohc241.glycine	0.08882929	0.047740991	0.06493727
pcaac385.pcaec405	-0.109871622	0.059051204	0.064942136
smc260.smc161	0.177620234	0.095511345	0.065075955
pcaec422.pcaac420	-0.219511268	0.11804602	0.065095498
pcaec425.pcaec342	0.206407007	0.111027902	0.065164626
pcaac365.pcaec381	0.014973051	0.008055512	0.065210568
smohc241.c182	0.051281517	0.02758952	0.065210821
pcaac422.pcaec342	0.177260557	0.095390987	0.065279279
isoleucine.pcaac362	0.019531537	0.010511541	0.065300529
c16.smohc222	-0.024000008	0.012920993	0.065395116
lysopcac182.c14	0.095934629	0.051658521	0.065445376
methioninesulfoxide.asparagine	0.046748352	0.025174892	0.06546666
leucine.pcaec385	0.117739835	0.063414491	0.065505742
pcaac343.pcaac402	-0.134367428	0.072374188	0.065520829
lysopcac203.c181	-0.042104109	0.022681502	0.065556161
c101.pcaec340	0.066972985	0.036079636	0.065565462
smc161.pcaec361	-0.017925282	0.00965688	0.065570584
pcaac342.pcaec443	-0.043616695	0.02349845	0.06558016
c10.smohc222	0.005599354	0.003016663	0.065581664
pcaac380.pcaac366	0.134156269	0.072295138	0.065648667
alanine.c12	-0.055458004	0.029893972	0.065723118
c182.pcaac361	-0.122165421	0.06587204	0.065805086
lysopcac281.pcaac403	-0.04817546	0.025978469	0.065826514
smc161.pcaec385	-0.096386508	0.051983887	0.065866364
pcaec383.lysopcac203	0.036010987	0.019422351	0.065875141
pcaac322.asparagine	0.014903856	0.008038768	0.065889948
pcaec443.pcaec381	-0.351071346	0.189433637	0.065995084
c142.pcaec365	-0.047226118	0.025484176	0.066011483
pcaac343.pcaec404	-0.025794953	0.01392426	0.066103286
pcaac323.pcaac384	0.152038031	0.082077621	0.066124936

c182.prolinr	0.036289224	0.01959095	0.066128194
smc261.pcaac366	0.04530367	0.024459821	0.066154134
c51dc.pcaac344	-0.09230056	0.049845675	0.066217805
pcaac385.c182	-0.047292977	0.025547775	0.066300055
pcaec405.pcaac402	-0.233046917	0.125905152	0.066327033
pcaac360.lysopcac203	-0.060356519	0.032611469	0.066355461
pcaec361.smc202	0.115328408	0.062315255	0.066362812
c182.glutamicacid	-0.124774305	0.067432404	0.066415572
pcaec322.lysopcac161	0.14914218	0.080609455	0.066441536
pcaac322.c182	-0.098662887	0.053328001	0.066451416
histidine.pcaac362	0.017320386	0.009361994	0.06645718
pcaac342.c182	-0.088911769	0.048063989	0.066487939
smc260.citruline	0.02992091	0.016183045	0.066627067
asparticacid.lysopcac280	-0.016697197	0.009031943	0.06665937
spermidine.c18	0.128061792	0.069274175	0.066668145
pcaec401.serotonin	-0.235535106	0.127474327	0.06680155
pcaac386.c181	-0.01405953	0.007610565	0.066850527
pcaac344.asparagine	0.018678711	0.010110981	0.066850737
pcaec443.pcaec300	-0.041691241	0.022575494	0.066941362
pcaec360.pcaec365	0.064806251	0.035095793	0.066969749
lysopcac180.pcaec300	-0.024650612	0.013351624	0.067012107
lysopcac280.pcaac362	0.008960018	0.004853674	0.067046502
pcaac424.pcaec381	0.03348622	0.018143826	0.06710927
c5.pcaac344	-0.185066473	0.100278679	0.067120536
smohc141.pcaac402	-0.146228968	0.079260675	0.067209785
alphaaminoadipicacid.pcaac426	0.106960998	0.05798828	0.067266065
pcaec402.pcaec340	-0.09999298	0.054220302	0.067314407
pcaac385.c18	-0.063749068	0.034567658	0.067316703
pcaac343.asymmetricdimethylar[illegible]	0.069619519	0.037759044	0.067375178
pcaec343.pcaac366	0.04952746	0.026861928	0.067375893
pcaac342.pcaac362	0.016585094	0.008995549	0.067387504
pcaac322.asparticacid	-0.099622028	0.054039883	0.067418206
pcaac386.pcaec443	-0.051131224	0.027736324	0.067420513
smohc141.c14	-0.061241769	0.03322855	0.067483452
phenylalanine.lysopcac203	0.017477469	0.009483384	0.067496628
pcaac422.pcaec381	0.033618234	0.01824741	0.067584956
smc160.c12	0.111743265	0.060663598	0.067635198
c101.tyrosine	0.145954814	0.079236988	0.067636901
pcaac426.spermidine	-0.138242084	0.075051611	0.067643278
pcaec383.pcaac362	0.026525738	0.01440082	0.067643289
pcaac424.c182	-0.092174475	0.050041664	0.067644104
c142.pcaec401	-0.078753118	0.042761638	0.067685027
pcaec405.pcaac422	-0.224509246	0.121958691	0.067805011

c51.lysopcac280	-0.025856545	0.014049695	0.067878767
putrescine.smohc222	0.005412454	0.002940981	0.067879766
lysopcac140.pcaec381	0.026837436	0.014584844	0.067919039
pcaec342.pcaac366	0.046863395	0.025468828	0.067928087
pcaac424.pcaec406	0.112004695	0.060873864	0.067940252
lysopcac204.pcaac405	0.090076661	0.048965089	0.06799012
lysopcac160.putrescine	-0.087444634	0.047543287	0.068041357
smohc221.pcaac405	-0.064148381	0.034883358	0.068089331
pcaac322.pcaec404	-0.045057771	0.024510351	0.068181685
smc180.c141oh	-0.17390482	0.094614518	0.06822331
c3.pcaac366	-0.067379509	0.036660608	0.068239415
pcaec420.pcaec300	0.111985954	0.060934271	0.068255894
c18.lysopcac204	0.066594098	0.036236911	0.068266795
lysopcac140.pcaec382	0.019705491	0.010723928	0.068298944
pcaec382.pcaec300	-0.035430178	0.019281883	0.068304902
smc181.pcaec340	0.070527012	0.038392651	0.068378097
pcaec423.smohc241	0.147544672	0.080323465	0.068394623
c0.leucine	0.081541401	0.044391937	0.068399038
c101.pcaac281	0.01075007	0.005853283	0.068438196
prolinr.pcaec381	-0.01683086	0.009164426	0.068444943
pcaec404.asparagine	0.02755132	0.015007486	0.068549744
pcaec443.smohc222	0.073407059	0.039986903	0.068558822
smc181.c141oh	-0.160779135	0.087581393	0.068560213
smc181.c12	0.130220818	0.070940064	0.068578466
ornithine.smohc161	-0.022504399	0.012259919	0.068584153
pcaec406.kynurenine	-0.022468136	0.012246348	0.068722338
pcaac385.smc160	-0.188760865	0.102931607	0.068846733
phenylalanine.smohc222	-0.010837726	0.00591088	0.068895428
pcaec420.c16	0.076422319	0.041681799	0.068903188
pcaec381.smohc221	-0.083321141	0.045450281	0.068937986
pcaac381.histidine	-0.131862989	0.071939899	0.068979383
pcaec301.pcaec300	-0.014587706	0.007967045	0.069272433
pcaac402.pcaec421	-0.017056466	0.00931679	0.069314858
pcaac281.asymmetricdimethylar[illegible]	0.038937684	0.021276266	0.069408683
c101.pcaac366	-0.011787603	0.006441146	0.069416532
pcaac381.pcaac405	-0.06491525	0.03547493	0.069440075
c141.smc181	-0.052761895	0.028834034	0.06944655
lysopcac140.tyrosine	0.09669987	0.052852362	0.069480476
asparticacid.c12	0.162609743	0.088890058	0.06952369
smc161.c2	-0.109113489	0.05965929	0.069582724
pcaec385.lysopcac182	-0.074765361	0.040900213	0.069725969
pcaac360.pcaac362	0.021248453	0.011628282	0.069829678
pcaec420.asymmetricdimethylar[illegible]	0.046262376	0.025323119	0.06989397

serine.pcaec360	-0.009760929	0.005343208	0.069907775
pcaac424.pcaec300	-0.055564487	0.030455844	0.070266312
lysopcac181.pcaec381	-0.030719453	0.016841673	0.07032916
pcaac421.smc240	-0.08646726	0.047411048	0.070365015
asymmetricdimethylarginine.pca	-0.012506326	0.006859217	0.07043977
c4.pcaec300	0.014318365	0.007854453	0.070489468
pcaec424.pcaec382	-0.043207001	0.023703658	0.070514228
glycine.pcaec381	-0.030243808	0.016592626	0.07052536
pcaac300.pcaac322	-0.217810559	0.119520249	0.070579064
lysopcac170.asymmetricdimethy	0.033089423	0.018158148	0.070591733
pcaec404.pcaec381	0.047270413	0.025941112	0.070602512
lysopcac203.pcaac425	-0.16404745	0.090060521	0.070708695
smc202.c2	-0.105293931	0.057817919	0.070769038
lysopcac182.pcaac366	0.023502554	0.012905549	0.070770515
histidine.pcaac366	0.028477516	0.015642601	0.070863787
phenylalanine.smohc221	0.098236646	0.053961783	0.070867577
pcaac384.pcaac362	-0.005222371	0.002868932	0.070893086
pcaac366.pcaec381	0.016567722	0.009101808	0.070901013
citruline.pcaec404	-0.020885453	0.011474904	0.070926869
smohc221.pcaec382	0.076784893	0.042197575	0.070995289
pcaec402.pcaec300	-0.027390014	0.01505464	0.071037948
pcaec402.lysopcac182	-0.115320477	0.063396661	0.071090539
lysopcac181.c181	0.054356868	0.029882687	0.071093923
c51.pcaac366	-0.030699425	0.016877806	0.071107123
pcaac364.pcaec403	-0.15379364	0.084572991	0.071176284
pcaac402.pcaec300	-0.040629703	0.022342859	0.071177437
lysopcac182.pcaec443	0.108372087	0.059614129	0.071265368
pcaac360.pcaac366	0.035384443	0.019467758	0.071311604
c162.lysopcac170	-0.03997439	0.021994877	0.071334865
c181.c18	-0.122059384	0.067166312	0.071361031
lysine.pcaac362	0.016562519	0.009116589	0.071441999
pcaac341.pcaac362	0.010285915	0.005663355	0.071522507
c141oh.pcaac362	-0.008894567	0.004897957	0.071560777
pcaec382.pcaac362	0.023890174	0.013155708	0.071563831
methionine.kynurenine	-0.02764334	0.015224033	0.071592412
pcaec380.pcaec381	0.025580888	0.014088477	0.071598372
alphaaminoadipicacid.glutamine	-0.015750076	0.008675235	0.071630748
pcaec300.pcaac403	-0.053609066	0.029528278	0.071631584
c102.pcaac366	-0.011023619	0.006072802	0.071673549
smc240.pcaec443	-0.092747542	0.051095498	0.071683463
pcaec384.pcaec443	-0.089808507	0.049479792	0.071702943
ornithine.pcaac385	0.066792523	0.036804017	0.071739755
lysopcac160.pcaec381	0.035923466	0.019798655	0.071797983

c3.lysine	-0.211363147	0.116504265	0.071833596
smc202.pcaac362	-0.009363778	0.005161485	0.071840689
c12.pcaac362	-0.036109171	0.01990805	0.07189739
pcaac385.pcaac362	-0.006930077	0.003820921	0.071909563
phenylalanine.pcaec381	-0.044632365	0.024617613	0.072017033
smohc161.smc202	0.190176873	0.104895418	0.07201883
pcaec365.alphaaminoadipicacid	0.018890204	0.010423044	0.072121912
pcaac405.pcaec444	0.183366177	0.10118333	0.072142521
c181.pcaec401	-0.103056779	0.056869782	0.072152414
pcaec363.pcaac366	0.036366507	0.020071814	0.072204301
asparticacid.pcaac362	-0.016879291	0.009318098	0.072261412
pcaac426.c14	0.088142633	0.048670945	0.07233325
c3.c16	-0.064133169	0.035413776	0.072337002
pcaac343.pcaec403	-0.154436086	0.085302516	0.07241721
pcaac384.pcaec300	0.017177275	0.009488079	0.072424316
glutamicacid.pcaec421	-0.024161089	0.013347005	0.072452457
c2.lysopcac204	0.069263764	0.038262752	0.072454281
pcaac380.pcaec340	0.088176283	0.048711896	0.072462814
c51.pcaac344	-0.077684373	0.042919954	0.07249033
pcaac364.c182	-0.051916731	0.028684428	0.07249896
smc241.pcaac405	-0.048001382	0.026531514	0.072609143
lysopcac280.pcaec300	-0.012350028	0.006827325	0.072657528
smc240.pcaac405	-0.051694361	0.028581437	0.072695436
pcaec363.lysopcac182	-0.085643298	0.0473677	0.072791839
phenylalanine.smc181	-0.041794799	0.023116154	0.072794719
pcaec382.pcaec443	-0.195705767	0.108250881	0.072817186
pcaec300.asymmetricdimethylar	0.033456288	0.018508094	0.072853771
pcaac386.pcaec300	0.022971551	0.012710402	0.072909197
pcaac363.pcaac381	0.120005566	0.06644698	0.073108177
pcaec363.smohc221	0.033970582	0.018811809	0.07314326
pcaec420.serotonin	-0.179186204	0.099228078	0.073144859
lysopcac182.lysopcac161	-0.203405009	0.112675389	0.073234663
smohc161.pcaec300	0.047574153	0.026355737	0.073258649
pcaec444.pcaac362	0.009201022	0.005097679	0.073279793
pcaec364.c14	-0.100945181	0.055951688	0.073404871
pcaac381.pcaac320	0.222714309	0.123467291	0.073454765
smc161.lysopcac182	-0.054601961	0.030271781	0.073471755
pcaac281.pcaec361	-0.02219825	0.012308112	0.07349991
pcaec421.pcaac362	0.022941727	0.012721561	0.073527132
pcaac386.pcaac401	0.063815993	0.035387238	0.07352901
pcaac424.pcaec402	-0.233241795	0.129339238	0.073533456
c2.pcaac366	-0.025073483	0.013906892	0.073593412
pcaec404.c12	0.148030784	0.082134355	0.073696652

c101.pcaac362	-0.006881846	0.003818505	0.073706848
pcaac364.pcaec340	0.131486163	0.072968098	0.073749256
c4.lysine	-0.162568176	0.090225083	0.073774715
pcaac344.pcaac362	-0.016843059	0.009348363	0.073789709
c102.asparagine	-0.036117604	0.020047835	0.073812005
pcaac342.c12	0.082265755	0.045665416	0.073824994
lysopcac181.pcaec360	-0.016623906	0.0092294	0.073872332
asymmetricdimethylarginine.pca	-0.006097185	0.003385946	0.073944781
c16.pcaec402	-0.070324105	0.039054508	0.073955513
c101.pcaac381	-0.063183929	0.035101213	0.074053198
pcaac425.lysopcac203	-0.112837592	0.062694907	0.074094627
pcaac401.pcaac362	0.050184367	0.027888056	0.07414158
c102.pcaec404	-0.028970814	0.016102372	0.07419384
smc181.pcaec421	0.002693055	0.001496878	0.074201879
pcaac321.lysopcac161	0.143626495	0.07984872	0.07426252
lysopcac280.c182	-0.078617169	0.043707839	0.074268146
pcaec404.ornithine	-0.075942556	0.042226593	0.074306965
pcaac426.pcaac366	-0.021729366	0.012086444	0.074406316
lysopcac181.pcaac366	0.050338661	0.027999751	0.07440699
serotonin.pcaec381	-0.060269113	0.033525786	0.074427873
pcaac322.pcaec443	-0.049578483	0.027585636	0.074497568
pcaac321.pcaac362	0.011190502	0.006226603	0.074505393
pcaac321.pcaec443	-0.03872512	0.021550018	0.074540555
smc161.pcaec421	0.00375488	0.002090117	0.074619594
pcaec423.asparagine	-0.021395944	0.011909875	0.074620399
ornithine.asparagine	0.034711299	0.019321827	0.074621439
pcaec380.pcaec342	0.185224007	0.103111345	0.074642487
smc241.citruline	0.014766479	0.008220494	0.074650276
pcaac404.pcaac366	0.032609302	0.018154157	0.074659275
smohc141.pcaac366	-0.017951788	0.009994143	0.074661433
pcaac360.asparticacid	-0.07546239	0.042011676	0.0746625
pcaac384.prolinr	0.062789918	0.034965789	0.074737845
pcaac384.pcaec405	-0.124619409	0.069402213	0.074760478
pcaec401.pcaec300	-0.043561732	0.024260745	0.074767992
c141.pcaec421	0.004940749	0.002751738	0.074778067
asymmetricdimethylarginine.pca	-0.10940621	0.060942664	0.074821231
pcaac322.pcaac403	-0.037253924	0.020761839	0.074963145
asparticacid.pcaac402	-0.131734473	0.073418637	0.074971817
smc180.valine	-0.098150092	0.054713814	0.075037687
pcaec445.pcaac362	0.015087769	0.008410832	0.075042762
pcaac385.lysine	0.136026236	0.075836219	0.075069313
c2.pcaac320	-0.071845091	0.040060899	0.075115469
smohc241.smc181	0.045308554	0.025265288	0.075129036

c162.c181	-0.02960323	0.016508076	0.075137866
lysopcac161.c141oh	-0.260148594	0.14510729	0.075211113
c102.pcaac362	-0.00647114	0.00360986	0.075238887
serine.c5	-0.024347176	0.013582564	0.075254533
smc160.pcaec300	0.020030511	0.011175158	0.07527346
lysine.lysopcac180	0.11281797	0.062951549	0.075317612
pcaec300.spermidine	-0.133986912	0.07476671	0.0753294
c162.pcaec421	0.003820603	0.00213204	0.075341019
lysopcac182.pcaac362	0.01382577	0.007717622	0.075427935
alphaaminoadipicacid.pcaec383	0.083959352	0.046875704	0.075484163
pcaac402.pcaac362	0.028960107	0.016176265	0.075616818
asymmetricdimethylarginine.smc	-0.077127012	0.04309316	0.075699226
acetylornithoine.ornithine	0.053240903	0.029748461	0.07571072
serine.pcaec404	0.04994817	0.027909115	0.075715641
lysopcac161.pcaac366	0.018411337	0.010288803	0.075750991
c8.spermidine	0.123780294	0.069183981	0.075800654
pcaec340.pcaac402	-0.107606086	0.06014583	0.075810418
glutamine.asymmetricdimethylar	0.0229417	0.01282667	0.075890206
pcaec363.c16	0.106385921	0.059514976	0.076059604
pcaec423.histidine	-0.171332812	0.095854133	0.076078435
pcaec401.c181	-0.027087624	0.01515591	0.076105702
pcaec444.asparticacid	0.050127047	0.028047061	0.076108461
histidine.lysopcac204	-0.028861356	0.016148622	0.07611079
alphaaminoadipicacid.c8	-0.08922396	0.049929343	0.076147966
pcaac380.kynurenine	-0.01750808	0.009798761	0.076186941
pcaec403.pcaac362	0.017464714	0.009774513	0.076187604
isoleucine.pcaec300	-0.020211965	0.011312927	0.076209533
pcaac406.smc202	0.04769017	0.026696796	0.076252215
pcaac405.pcaac366	0.038469584	0.021535955	0.076263165
pcaec386.pcaac405	-0.06637048	0.037155771	0.07626629
serotonin.pcaec402	-0.057700476	0.032304583	0.07628865
lysine.pcaac405	0.12316794	0.068958065	0.07629042
pcaac426.pcaac361	0.100681036	0.056368744	0.076292625
smohc141.c182	0.057829523	0.032378504	0.076303655
lysopcac180.valine	-0.086105182	0.048217593	0.076349879
pcaac424.ornithine	-0.119140144	0.066726111	0.076391019
lysopcac181.pcaec300	-0.03121731	0.017487338	0.076451646
pcaac426.smc240	-0.061307497	0.034348817	0.07649853
c3.smohc221	0.038837342	0.021766359	0.076591095
pcaac420.pcaac362	0.026772808	0.015005554	0.076605731
pcaec384.pcaec300	0.014807946	0.008299639	0.076609931
putrescine.pcaac366	0.012781488	0.007164327	0.076629798
pcaec422.smohc241	0.109317214	0.061278943	0.076649056

pcaac383.asparagine	0.017969858	0.010074386	0.076683444
isoleucine.asparagine	0.017396493	0.009753334	0.076695145
pcaec445.pcaec364	0.035833744	0.020090417	0.076698742
pcaec401.alphaaminoadipicacid	0.031813288	0.017837547	0.076718751
pcaac385.transhydroxyproline	0.069170592	0.038788645	0.076756702
pcaac380.pcaec402	0.13067134	0.073291754	0.076818248
pcaac366.smohc241	0.120480988	0.067580125	0.076835512
pcaec381.pcaac362	0.022929092	0.012861676	0.076842386
c101.c16	0.084609129	0.047468913	0.076897055
lysopcac161.smc181	-0.037978065	0.021320448	0.077079583
c12.smohc222	0.010466924	0.005876124	0.077085227
pcaac361.c5	0.01843689	0.010353484	0.077170688
pcaec424.pcaac362	0.01129243	0.006342956	0.077241703
pcaac424.pcaec443	-0.246991784	0.138745719	0.077263818
c0.creatinine	-0.045421938	0.025516718	0.077278694
serotonin.citruline	0.011463025	0.006441451	0.077363136
h1.pcaec381	-0.015741473	0.008845736	0.077365951
lysopcac181.smc241	-0.20940901	0.117682026	0.077383603
c10.pcaec404	0.045442874	0.025541431	0.077427222
c181.lysopcac180	-0.058394234	0.032824408	0.077459268
c12.lysopcac170	-0.039828568	0.022394726	0.077543051
c3.pcaec404	-0.021152062	0.011897611	0.077648425
pcaec362.pcaec300	0.010293118	0.005789786	0.077654182
pcaac402.pcaec381	0.056168028	0.031612128	0.077822518
c3.pcaec362	-0.01066536	0.006002761	0.077829968
asparagine.prolinr	-0.090707224	0.051056162	0.077850757
smohc141.pcaec300	0.017736816	0.009984615	0.07788416
pcaec362.citruline	0.01934365	0.010891544	0.077948732
pcaac320.pcaec382	0.024181296	0.013615736	0.077955871
smc202.citruline	0.011518934	0.006485999	0.077957904
pcaac342.pcaac366	0.026662666	0.015016375	0.078023589
h1.pcaac362	-0.028936122	0.016297973	0.078045093
pcaec340.transhydroxyproline	0.053658532	0.030227504	0.078092685
lysopcac160.pcaec365	0.045589435	0.025684426	0.078121275
glutamine.pcaac426	-0.103559388	0.058380402	0.078305772
pcaec406.pcaec402	0.141016723	0.079521757	0.078399317
glutamicacid.ornithine	-0.080317936	0.04529724	0.078429112
pcaec365.pcaac405	-0.060995717	0.034403947	0.078462907
pcaec421.c182	-0.068355722	0.038570119	0.078576574
pcaac363.asymmetricdimethylar[illegible]	0.046275731	0.026116607	0.078636007
kynurenine.lysopcac204	0.038809062	0.021908924	0.078720711
c182.pcaac366	-0.023323851	0.013170098	0.078789394
c51dc.asparagine	0.02121763	0.011980826	0.078790175

c182.c3	0.163041814	0.09208332	0.078852913
serotonin.pcaac366	-0.003742919	0.002114431	0.078921784
pcaec384.smc160	0.236980719	0.133889156	0.078955467
pcaec402.smohc222	-0.017014467	0.009613768	0.078984723
c162.lysine	0.123998937	0.070071586	0.07901795
alphaaminoadipicacid.pcaec403	-0.074871893	0.042312493	0.079035565
pcaec360.pcaec300	-0.03020622	0.01707067	0.079038557
pcaec405.pcaec381	0.08664347	0.048996736	0.079227802
c5.smohc221	0.120082478	0.067917144	0.079274592
h1.citruline	0.01870531	0.010582119	0.079348418
lysopcac180.lysopcac203	-0.027493928	0.015554192	0.079350416
pcaac361.pcaac362	0.014980518	0.008475058	0.07935395
pcaec300.smohc161	-0.041729853	0.02361303	0.079414852
pcaac341.alphaaminoadipicacid	0.011905732	0.006737147	0.079425164
pcaac403.pcaec300	-0.031504075	0.017829554	0.079461991
pcaec446.pcaec445	0.355949889	0.201465597	0.079488197
lysopcac203.pcaac366	0.022641733	0.012821474	0.079636315
smc161.smohc161	0.109142143	0.061822323	0.079721696
pcaec406.c141oh	-0.217264726	0.123112115	0.079830575
pcaac344.pcaec300	0.029958441	0.016981644	0.079932827
arginine.c18	-0.14042905	0.079602037	0.079937427
pcaac426.pcaac362	-0.01267185	0.007183294	0.079948674
lysopcac281.leucine	-0.07708215	0.043723712	0.080140825
c181.pcaac366	-0.026646006	0.01512036	0.080255576
lysopcac180.asymmetricdimethy	0.037687596	0.021386738	0.080266666
lysine.c182	-0.136254479	0.077355485	0.080400393
pcaac343.pcaac362	0.014613475	0.008297061	0.080421391
pcaac342.citruline	0.029303065	0.016646078	0.080578619
lysopcac140.pcaec365	0.050025942	0.028419105	0.080589827
c182.c162	0.264772612	0.150421343	0.080604509
pcaec406.pcaec340	0.142050022	0.080708759	0.080634152
pcaac343.pcaac366	0.024587208	0.013969811	0.080635504
c141oh.pcaec421	0.006963161	0.003956362	0.080641214
lysopcac140.pcaac365	-0.099004517	0.056255529	0.080655429
pcaac320.alphaaminoadipicacid	0.0111127	0.00631488	0.080679801
lysopcac204.c8	-0.118705041	0.067467684	0.080735811
c0.pcaac361	-0.093445965	0.053118984	0.080779019
pcaac406.pcaec446	-0.135162463	0.076833756	0.080783685
pcaac381.asymmetricdimethylar	0.042932943	0.024407095	0.080804033
pcaac240.pcaec421	0.017784287	0.010110655	0.080816058
pcaec425.pcaec402	-0.093472148	0.053147018	0.08085327
acetylornithoine.lysopcac170	-0.030716482	0.017469664	0.080933727
c2.pcaac362	-0.014593896	0.008302576	0.081022639

pcaac362.pcaec363	-0.060925271	0.034664421	0.081053677
pcaac321.smc240	-0.071546306	0.04070744	0.081053741
c182.pcaec402	-0.038625256	0.021977226	0.081064073
pcaec401.lysine	0.160793598	0.091509233	0.081129592
serotonin.pcaac362	-0.002203745	0.001254276	0.081154373
pcaec300.c12	0.095856258	0.054557517	0.081155877
pcaac240.asparticacid	-0.128972915	0.07340628	0.081156274
pcaac341.pcaac366	0.0167506	0.009533944	0.08116149
pcaac362.pcaac420	-0.132489204	0.075409669	0.081164478
c10.pcaec365	-0.047583456	0.027084512	0.081177274
lysopcac182.pcaec300	-0.016530232	0.009410803	0.081234557
putrescine.c51	0.201338544	0.11463371	0.081260687
glutamicacid.c2	0.153797999	0.087566525	0.081262075
smc161.pcaec300	0.007179779	0.004088196	0.081285099
pcaec360.pcaac366	0.019536866	0.011127571	0.081371658
h1.pcaec443	0.117024871	0.066656549	0.081384937
lysopcac204.smohc221	-0.058905212	0.033554574	0.081408074
pcaac320.pcaac366	0.030185299	0.017195601	0.081424631
lysopcac281.c102	0.0837327	0.047704844	0.081456202
pcaec365.pcaec300	0.011723849	0.00667942	0.081456984
c102.pcaac405	0.152974856	0.087159581	0.081475425
pcaac321.pcaac366	0.018395705	0.010483683	0.081546469
asymmetricdimethylarginine.glut	0.087995723	0.050150959	0.081560528
c16.pcaac405	0.09276181	0.052871378	0.081583959
smohc221.smc160	0.190806109	0.108762515	0.081608647
c182.pcaec383	0.153945501	0.087760305	0.081639323
c12.pcaec402	-0.105239365	0.060008409	0.081710739
c2.pcaac344	-0.137056773	0.078165979	0.081768703
lysopcac161.asymmetricdimethy	0.05008703	0.028571356	0.081830113
pcaec321.pcaec360	-0.016440286	0.009378886	0.081855399
ornithine.pcaac320	-0.09347581	0.053331616	0.08188573
c4.creatinine	-0.036690416	0.02093422	0.081898711
pcaac383.pcaec444	0.175707222	0.100259098	0.081919607
c162.c3	0.12272096	0.070032601	0.081952556
creatinine.smohc221	0.102200663	0.058328004	0.081981723
pcaec406.histidine	-0.111214657	0.063487324	0.082052484
pcaec342.pcaec300	0.008324052	0.004751894	0.082057354
smohc222.c162	0.163631484	0.09342266	0.082094543
pcaac403.c18	0.098703829	0.056353675	0.082096553
pcaec446.lysopcac203	-0.03046053	0.017391054	0.082096699
lysopcac203.c18	0.085118517	0.048599131	0.082107854
pcaec420.glutamicacid	0.104400254	0.059609902	0.08211649
pcaac361.smohc241	0.086077947	0.049153586	0.08214887

alphaaminoadipicacid.pcaec404	-0.026637692	0.015212274	0.082172738
asparagine.pcaac362	-0.030092935	0.017186326	0.082187358
lysopcac280.smohc222	-0.015646826	0.008939298	0.08229803
pcaec420.c5	0.031143593	0.017795332	0.082340054
pcaac404.pcaac405	-0.232905719	0.13308559	0.082349481
pcaec405.pcaec406	0.075098135	0.042915959	0.082376559
alanine.lysopcac203	0.042652199	0.024374286	0.082377115
serotonin.pcaec300	-0.01817595	0.010387932	0.082406079
pcaac341.c181	-0.009157082	0.005233618	0.082415142
c141.pcaac362	-0.02466069	0.014094737	0.082419912
lysopcac160.pcaac362	0.014469343	0.00827029	0.082434003
pcaec343.pcaec300	0.00791353	0.004523295	0.082442923
pcaec321.pcaec300	0.009257756	0.00529268	0.08250245
pcaac384.pcaec301	0.107814581	0.061656103	0.082592387
pcaac342.pcaec300	0.01270073	0.007263677	0.082612857
c16.pcaec365	0.054760146	0.031319013	0.082623781
pcaac344.transhydroxyproline	0.0503688	0.028809712	0.082647447
smc202.pcaec300	0.005627017	0.00321975	0.08276403
c181.lysopcac182	-0.042037267	0.024054746	0.082779699
c3.creatinine	-0.027534925	0.015768377	0.083015642
lysopcac203.pcaac362	0.013404388	0.007676493	0.083024607
c3.lysopcac204	0.096588133	0.055316359	0.083034381
pcaec301.pcaac362	0.009810066	0.005618388	0.083041398
pcaec385.prolinr	0.038885579	0.022270986	0.083049152
c101.smc160	-0.106472479	0.060987459	0.083085833
lysopcac160.pcaac366	0.024079921	0.013794508	0.08311951
asparticacid.pcaec423	-0.090353545	0.051760472	0.083120806
serine.pcaac366	0.044617658	0.025562217	0.083147971
alanine.pcaac405	0.055575149	0.031843506	0.08318201
methionine.spermidine	-0.1213403	0.069525847	0.083182697
pcaec425.serotonin	-0.223658705	0.128158227	0.083196361
pcaec381.smohc222	-0.034393229	0.019709968	0.083233065
pcaac360.transhydroxyproline	0.134522921	0.077096894	0.083252462
c18.lysopcac280	0.068512782	0.039269044	0.083279241
asparticacid.pcaec365	0.045650755	0.026166808	0.083296144
alanine.pcaac366	0.020613033	0.011816525	0.083327835
pcaec444.lysopcac180	-0.085762378	0.04917288	0.083384706
pcaec322.pcaec360	-0.01520427	0.00871786	0.083395594
smc181.pcaec300	0.007661073	0.004393094	0.083421274
c16.putrescine	-0.085333649	0.048934105	0.083428643
c12.c101	-0.047221788	0.027079984	0.083438935
pcaec385.pcaec361	-0.0190396	0.010918625	0.083441844
pcaac406.asymmetricdimethylarg	0.033107436	0.018986285	0.083444893

pcaec386.pcaac361	0.170469563	0.097772623	0.083484252
pcaac425.pcaec443	-0.075890832	0.043530328	0.083506903
pcaec384.pcaac362	-0.010524894	0.006037903	0.083553141
lysopcac203.pcaec300	-0.009837272	0.005643529	0.083558574
pcaec342.smohc221	0.034055702	0.019541859	0.083628928
pcaac344.alphaaminoadipicacid	0.009921478	0.005696077	0.083786328
asparticacid.citruline	0.012165454	0.00698457	0.083794654
ornithine.pcaac362	0.018007334	0.01034109	0.083869069
pcaac385.pcaec300	0.007958403	0.004570598	0.08389038
pcaac321.asparagine	0.0151723	0.008714871	0.0839344
pcaac240.pcaac362	0.01482749	0.008517711	0.083966638
pcaec383.pcaec300	-0.066969486	0.038476936	0.084014816
acetylornithoine.pcaac362	0.015239829	0.008756164	0.084022076
asparticacid.lysopcac203	0.016209708	0.009314035	0.08404246
lysopcac204.pcaac426	-0.119253043	0.068531142	0.084081867
isoleucine.c141	-0.07024548	0.040368545	0.084086254
c102.smohc221	0.055327077	0.031797344	0.084106315
pcaec423.pcaec421	0.014099457	0.008104775	0.084166677
pcaac422.smc240	-0.075343841	0.0433185	0.084228195
lysopcac281.smohc222	-0.011955545	0.006874053	0.084240792
pcaec301.pcaec361	0.037666751	0.021658564	0.084260597
pcaac363.pcaec300	0.008309803	0.004778266	0.084266316
pcaac366.pcaec443	-0.11537946	0.066363693	0.0843529
lysopcac160.pcaec300	-0.017613806	0.010135195	0.084478021
lysopcac203.pcaec381	0.033101308	0.019048264	0.08450023
pcaec385.pcaec300	0.008055911	0.004636076	0.08451839
lysopcac281.citruline	0.013193195	0.007593362	0.084552547
pcaac425.tyrosine	-0.130476944	0.075097532	0.084558033
lysine.pcaac366	0.026668501	0.015349455	0.084559773
pcaac364.pcaac362	-0.013206026	0.007601117	0.084567542
pcaac426.pcaac422	-0.06950153	0.040013092	0.084640111
pcaec383.ornithine	-0.088601082	0.051015966	0.08468216
h1.pcaec402	0.057360823	0.033031133	0.084711222
c16.c181	-0.054809652	0.031570032	0.084789147
lysopcac204.pcaac380	0.145340712	0.083719917	0.084805829
pcaac384.c182	-0.050993117	0.029384075	0.084918632
lysopcac140.pcaec421	-0.011587549	0.006677746	0.084945468
pcaac363.pcaec405	0.131273719	0.075658639	0.084975617
smc260.pcaac366	0.018209777	0.010496008	0.08500316
methionine.pcaec404	0.027551485	0.015883753	0.085066087
pcaec424.pcaec365	0.046913572	0.027050144	0.085110844
pcaec445.pcaec300	0.012218511	0.00704627	0.08516055
histidine.smohc141	-0.073854514	0.042594169	0.085183362

c0.glutamine	-0.046695245	0.026933305	0.085214571
methionine.c12	-0.10969361	0.063286039	0.085292536
pcaec363.pcaac363	-0.053501455	0.030873423	0.085358443
smohc222.c14	-0.059917619	0.034588281	0.085468989
asymmetricdimethylarginine.pca	-0.088498401	0.051087745	0.085473886
putrescine.lysopcac182	-0.030116781	0.017389008	0.085534399
pcaec401.smohc241	0.107989008	0.062355376	0.08555443
pcaac425.citruline	0.013029642	0.007524228	0.085579445
pcaac383.pcaac323	-0.145293798	0.083906426	0.085592773
c141oh.pcaec360	-0.015544534	0.008977246	0.08560509
pcaec386.pcaec300	0.011911421	0.00687923	0.085612836
pcaec405.asparagine	0.03533517	0.020412957	0.085700157
lysopcac280.pcaac344	-0.106871926	0.061745922	0.085732915
pcaec406.pcaec300	0.011455856	0.006619028	0.085748633
smc202.pcaec301	0.11826083	0.06834955	0.085840046
pcaec342.pcaec443	-0.073808826	0.042668008	0.085910989
pcaac342.smc202	0.055380526	0.032015634	0.085918813
c141.pcaec403	0.051287024	0.029649184	0.085918974
pcaac361.pcaac366	0.024608782	0.0142289	0.085973249
pcaec301.lysopcac280	-0.019443625	0.011245213	0.086051329
serine.citruline	0.010497963	0.006072071	0.086080895
pcaac404.c18	0.085668134	0.04955176	0.086086601
c181.pcaec383	0.16991784	0.098289461	0.086106765
leucine.pcaec403	-0.155028144	0.0896882	0.086147382
smc241.c142	0.070891501	0.041016229	0.086173706
pcaec404.pcaac362	0.024249362	0.014030933	0.086191364
c5.pcaac362	0.064661619	0.037414401	0.086195648
pcaec383.pcaec365	0.041939247	0.024270024	0.086236698
pcaec343.pcaac361	0.135188829	0.078242146	0.086272755
pcaac383.pcaac362	0.062682364	0.036278232	0.086273328
pcaec401.pcaec443	-0.092867853	0.053749754	0.086280726
pcaac422.pcaac366	0.04825812	0.027934776	0.086326353
smohc241.valine	-0.078966729	0.045716256	0.086363389
pcaec421.pcaec443	-0.135026351	0.078177205	0.086388536
c141oh.asparagine	-0.027519891	0.015933873	0.08639787
pcaec301.pcaac366	0.016343039	0.009462755	0.086405158
pcaec342.citruline	0.017455223	0.010107054	0.086415458
pcaac322.lysopcac203	0.027265818	0.015788965	0.086441228
serine.asparticacid	0.05727728	0.033171873	0.08647884
c51dc.tyrosine	0.096553812	0.055919041	0.086480757
pcaac364.citruline	0.008034038	0.004653873	0.086545508
pcaec380.pcaec443	-0.181063386	0.104895429	0.086578022
pcaac424.pcaac362	0.037647674	0.02181117	0.0865887

pcaac322.pcaec365	0.052200236	0.030242511	0.086591977
pcaac403.smohc221	-0.122019409	0.070694057	0.086598151
pcaec421.pcaac405	-0.097582354	0.056542951	0.086636316
pcaec384.glycine	0.059817213	0.034662021	0.086651045
pcaec364.pcaec300	0.016408676	0.009509006	0.086675359
pcaec363.c181	-0.017564147	0.010178971	0.086686284
pcaec360.pcaec340	0.04364347	0.025293298	0.086693027
phenylalanine.pcaec401	0.10060143	0.058304695	0.086702486
c142.pcaac405	-0.117839076	0.06829707	0.086712083
pcaec382.pcaec423	-0.04745217	0.027502443	0.086713844
pcaec385.pcaec421	0.001880322	0.001090419	0.086890358
alphaaminoadipicacid.pcaac366	-0.033120878	0.019211587	0.086962569
lysopcac204.smc160	-0.123012634	0.071369654	0.087036273
lysopcac203.lysopcac204	-0.074463839	0.043202704	0.087037452
pcaec405.pcaac405	-0.090957399	0.052779408	0.08708132
c4.asymmetricdimethylarginine	0.048737989	0.028287445	0.087153025
prolinr.pcaec362	-0.021576356	0.012523259	0.087162482
pcaac421.pcaac366	0.039654499	0.023016991	0.087174596
c51.lysopcac204	0.034722079	0.020156204	0.087208386
pcaac344.pcaec365	0.058588208	0.034011566	0.08721804
c102.lysopcac204	0.061959772	0.035972917	0.087253638
c181.pcaac405	0.111901007	0.064972712	0.087276084
pcaac404.pcaac362	0.018731148	0.010877714	0.08733103
methionine.prolinr	-0.046729367	0.027142112	0.087389039
pcaac281.pcaac362	-0.009577844	0.005563949	0.087433481
pcaac406.smc160	-0.107077548	0.062203432	0.087433878
pcaac363.pcaac362	-0.039021902	0.022668821	0.087437173
pcaec446.pcaec300	0.008715425	0.005063778	0.087484459
pcaac320.c5	0.01523211	0.008850859	0.087512824
c51.pcaec421	-0.006373923	0.003704024	0.087542872
pcaac322.pcaec300	0.019389462	0.011267851	0.087548871
smc161.citruline	0.010714816	0.006227077	0.087566304
pcaac323.smc202	0.109467681	0.063620327	0.087573869
asymmetricdimethylarginine.pca	0.004743668	0.002758112	0.087709388
pcaec423.pcaac366	0.039868527	0.023182099	0.087727365
lysopcac280.smc202	0.071117096	0.041355611	0.087754724
lysopcac180.pcaec362	0.05218333	0.030348555	0.08778782
pcaec406.pcaac405	-0.078607409	0.045718496	0.087803884
histidine.pcaac424	-0.063132331	0.036719748	0.087817915
pcaec321.c2	-0.075722637	0.044058528	0.087930989
c102.smc202	-0.125829753	0.073213836	0.087935014
pcaec300.smc202	0.065981974	0.038398038	0.087988488
glutamine.spermidine	-0.115468085	0.067197489	0.087993803

asparticacid.smohc222	-0.010066972	0.005859118	0.088024422
citruline.c181	-0.021670947	0.012613149	0.088033286
putrescine.c5	0.021418374	0.012467872	0.088076871
leucine.asparagine	0.017131947	0.009972995	0.088086324
pcaec362.pcaac366	-0.045770302	0.026649744	0.088151756
pcaec322.pcaec300	0.005397259	0.003142596	0.088156189
pcaec421.pcaec300	-0.052600235	0.030629212	0.088179911
lysopcac203.asymmetricdimethy	0.041632777	0.024249108	0.088261241
smohc241.pcaac366	0.031580878	0.01839437	0.088261484
serotonin.glutamicacid	-0.071534435	0.041674167	0.088327488
pcaec364.citruline	0.01195117	0.006962951	0.08835005
pcaec403.pcaec402	-0.261582707	0.152419387	0.088385073
c3.c5	0.060500317	0.035258132	0.088436194
glutamicacid.asymmetricdimethy	-0.038260569	0.022298372	0.088450757
pcaac360.lysopcac182	-0.026330309	0.015346341	0.088470462
pcaec343.smohc221	0.032143324	0.018743291	0.088620142
pcaac404.pcaec445	0.048133336	0.028068762	0.0886363
pcaac384.pcaac366	-0.008289564	0.004834045	0.088637546
pcaec421.pcaac366	0.036792792	0.021458335	0.088676961
pcaac420.pcaac366	0.04428348	0.025829572	0.088707623
isoleucine.pcaac366	0.029896202	0.017441305	0.088771221
pcaac380.c101	-0.056978227	0.033257704	0.088931504
pcaac380.pcaec421	0.003550601	0.002072658	0.088962425
pcaac405.pcaec300	0.012100877	0.007067596	0.089129245
lysopcac180.alphaaminoadipicac	-0.007027049	0.004104585	0.08915935
c162.citruline	0.012195546	0.007124792	0.089213786
c5.c14	0.085432376	0.049912097	0.089222831
pcaec386.kynurenine	-0.013142192	0.007678194	0.089228626
pcaac424.pcaac405	-0.080700713	0.047149254	0.089233129
pcaac404.transhydroxyproline	0.051444085	0.030056803	0.089240334
pcaec383.pcaec405	0.284039263	0.165967284	0.089267143
arginine.c2	0.111933833	0.065407729	0.089284339
leucine.pcaec300	-0.027463472	0.016053536	0.089391982
pcaec321.pcaac402	-0.123547911	0.072236381	0.089468847
pcaec341.transhydroxyproline	0.041679769	0.024377352	0.089571567
serotonin.pcaec405	0.093478915	0.054676505	0.089590446
smc161.asymmetricdimethylargi	-0.130712861	0.07646667	0.089639236
smc241.pcaec322	-0.146405543	0.085647292	0.089640887
pcaac320.pcaac426	0.098558758	0.057659418	0.089654712
pcaec360.putrescine	-0.074830457	0.043781981	0.089685391
c0.pcaec404	-0.029611339	0.017328358	0.089745602
pcaec321.pcaac366	0.01527988	0.008941721	0.089746915
alanine.pcaec362	-0.02773434	0.016230937	0.089764891

pcaec363.pcaec300	0.007785739	0.004557584	0.089844683
c10.smc160	-0.124278395	0.072755028	0.089868498
pcaec384.kynurenine	-0.038987922	0.022825661	0.089887453
asparagine.citruline	0.008405888	0.004923321	0.090020187
c3.c18	-0.070480329	0.041284082	0.090049587
pcaac343.pcaec300	0.010525316	0.00616572	0.090074418
pcaac363.citruline	0.011344376	0.006645765	0.090085977
pcaec445.pcaec446	0.26800274	0.157003825	0.090090824
h1.ornithine	0.121442374	0.071146515	0.090099897
alanine.pcaac322	0.113416495	0.066450703	0.090129191
pcaec321.smohc221	0.03667857	0.021490272	0.090133713
lysopcac280.smohc221	-0.044671859	0.026176865	0.090173348
c51dc.pcaac426	0.115249901	0.067535431	0.090178854
pcaec300.pcaac281	-0.008647335	0.005067551	0.090196982
pcaac404.pcaec300	0.064769442	0.037956845	0.090200003
pcaac323.pcaec300	0.003286367	0.001926087	0.090229262
c12.valine	0.095375801	0.055916682	0.090334404
c8.c2	0.102107982	0.05988577	0.090452397
pcaac380.pcaec300	0.013771462	0.008077063	0.090459341
pcaec384.pcaac366	-0.017247008	0.010117195	0.090512916
pcaec361.pcaec360	-0.015913878	0.009336446	0.090556423
pcaec443.ornithine	-0.126297715	0.074100903	0.090572904
prolinr.pcaac362	0.031036316	0.018209846	0.090578862
c101.pcaac405	0.111382775	0.065352916	0.090586818
creatinine.pcaec341	0.149141426	0.087510044	0.090596129
serine.pcaec300	-0.024564866	0.014414678	0.09061886
c141.pcaac320	-0.176191377	0.103406861	0.09067338
serotonin.smohc161	0.011939371	0.007007242	0.090674105
pcaec384.pcaec405	0.142627232	0.083710189	0.090681589
smohc161.citruline	0.035505471	0.020845155	0.090780119
pcaec445.c51	0.135804273	0.07974756	0.09084938
pcaac364.pcaac341	0.055944414	0.03285667	0.090895649
pcaac406.pcaec300	0.018249115	0.010718161	0.090904223
lysopcac170.pcaec421	-0.012716549	0.007469705	0.090945249
pcaac384.citruline	0.004573151	0.002686435	0.090964832
pcaac420.lysopcac203	-0.052595615	0.030899371	0.090993792
citruline.pcaec300	0.008763458	0.005148754	0.091013307
isoleucine.pcaec385	0.105248511	0.061841063	0.091038644
pcaac322.lysopcac280	0.013919181	0.008179495	0.091076821
smc181.pcaec385	-0.09445258	0.055508296	0.09109987
asparticacid.smc240	-0.129754424	0.076280329	0.091207745
pcaec341.pcaac366	0.027609475	0.016233596	0.09125657
pcaec424.pcaec402	-0.095258513	0.056015368	0.091291112

smc240.asparticacid	0.041572463	0.024447097	0.091304502
pcaac425.pcaec382	0.023110636	0.013596185	0.091440052
smc180.pcaec300	0.046833357	0.027558923	0.091515237
pcaac360.citruline	0.012674503	0.00745965	0.091574781
smc180.c162	0.200228273	0.117858443	0.091610093
pcaac300.pcaac281	-0.007980513	0.004698705	0.091692983
pcaac364.pcaac405	-0.097633571	0.057495945	0.091760029
alphaaminoadipicacid.valine	-0.124267746	0.073187273	0.091788963
pcaec401.pcaac405	-0.095161105	0.056054759	0.091845112
pcaac425.pcaec445	0.172656816	0.101707824	0.091858159
c162.pcaec401	-0.080629975	0.047509445	0.091941682
pcaac240.pcaac366	0.024394943	0.014375693	0.091975426
histidine.kynurenine	-0.022714025	0.013386746	0.092013897
c51dc.pcaec360	0.006704053	0.003951264	0.09202702
pcaec446.lysopcac182	-0.076467596	0.045072377	0.092052508
leucine.c141	-0.052731687	0.031081928	0.092054921
smohc221.citruline	0.012465674	0.007347767	0.092057401
smc240.putrescine	-0.041698717	0.02458267	0.092106867
creatinine.glutamine	0.021084432	0.01243124	0.092141113
c51dc.pcaec402	0.077200097	0.045520306	0.092166897
pcaac404.c8	-0.071428878	0.042117897	0.092170992
smc180.alphaaminoadipicacid	-0.033644808	0.019839105	0.092179276
pcaac320.pcaec443	-0.031359333	0.018491864	0.092186584
smc160.pcaec443	-0.071704637	0.042284792	0.092203703
pcaac361.pcaec365	0.048275071	0.028470771	0.092232906
pcaec423.pcaec443	-0.086724577	0.051150157	0.092254028
pcaac425.serotonin	-0.158162823	0.09328528	0.092256941
pcaec405.smohc221	0.107123299	0.063187897	0.092287548
pcaec365.citruline	0.007630018	0.00450139	0.092340318
smohc222.pcaac405	-0.079246557	0.046755081	0.092360622
putrescine.pcaac362	0.007242758	0.004273935	0.092416714
pcaec404.pcaec365	0.045704984	0.026976937	0.092494795
pcaec365.pcaac381	0.067435133	0.039805208	0.092513083
pcaec365.kynurenine	-0.017924071	0.010580446	0.092523201
lysopcac181.pcaac403	0.035418033	0.020908813	0.092551171
lysopcac140.pcaec300	-0.019434678	0.011476899	0.092656952
pcaec405.c12	0.11402695	0.067346738	0.092702939
pcaac385.pcaac366	-0.010906054	0.006444559	0.092864076
pcaec343.citruline	0.020098802	0.011877904	0.092896887
lysopcac204.pcaec300	-0.014107714	0.008340371	0.093015394
pcaac426.citruline	0.009041287	0.005345169	0.093017256
pcaac363.c182	-0.051367388	0.030369848	0.093035072
c181.histidine	-0.132824685	0.078533152	0.093049303

pcaec364.tyrosine	-0.089224567	0.052759833	0.093082643
lysopcac281.asparagine	0.016492573	0.009752983	0.093105067
c141oh.smc261	0.139479966	0.082485161	0.093116212
pcaec383.smohc222	-0.018293303	0.010818315	0.093118805
pcaec401.smc240	-0.044539097	0.026340927	0.093135339
smc202.pcaec340	0.040089447	0.023710999	0.093157822
smc180.citruline	0.011850681	0.007009504	0.093175861
pcaac362.c181	-0.015402264	0.009110319	0.093179619
smc261.glutamicacid	0.07896745	0.046710788	0.09319458
c162.pcaac366	-0.125893226	0.074495671	0.093313664
pcaec385.citruline	0.006731412	0.003983801	0.093360546
pcaac404.pcaac402	-0.118775682	0.070311438	0.093440433
pcaec364.spermidine	-0.115062983	0.068134812	0.093541288
c2.asparticacid	0.053771024	0.031844036	0.093575946
c182.pcaec405	-0.081869264	0.048486018	0.093587779
c5.c16	-0.114283981	0.067690238	0.093621457
pcaec382.smohc221	-0.130916612	0.077552617	0.093667087
pcaec425.lysopcac161	0.127101991	0.075298589	0.093691609
creatinine.pcaac363	0.037719388	0.02234636	0.093697346
pcaac380.pcaac405	-0.068027705	0.040308533	0.093748982
lysopcac203.smohc221	-0.039178938	0.023215828	0.09376421
pcaec365.lysopcac182	-0.043599768	0.025838929	0.093808217
c51dc.pcaec361	-0.023544238	0.013954569	0.093839254
pcaac381.c101	-0.043285842	0.025657501	0.093866728
lysopcac204.c2	0.087890654	0.052098079	0.093874609
pcaec404.pcaec406	0.057525592	0.034099126	0.093876962
c10.glutamine	0.062233586	0.036891537	0.093891766
c10.pcaec300	-0.034337098	0.020366552	0.094080566
pcaec424.pcaec342	0.174871946	0.103735769	0.094121399
pcaec341.pcaec443	-0.039595667	0.02348914	0.094129696
pcaec362.asymmetricdimethylar(	0.034316041	0.020357486	0.09413532
pcaac342.c142	0.04352429	0.025824531	0.094190658
pcaec445.pcaac366	0.024151655	0.014330455	0.094199905
asparticacid.pcaec322	-0.073362718	0.043534426	0.094233168
pcaac380.asparagine	-0.020904555	0.012406183	0.094263056
pcaec406.citruline	0.008545506	0.005072344	0.094318363
pcaac380.histidine	-0.096989304	0.057577558	0.094362248
smc202.pcaac366	-0.014671171	0.008709641	0.094366788
c182.c16	0.099730436	0.059209012	0.094385298
smc161.pcaec362	0.023540964	0.013978662	0.094446335
pcaac425.pcaac361	0.076986591	0.045730235	0.094556765
c101.c181	-0.028415297	0.01688151	0.094609941
pcaac281.pcaec300	0.014911753	0.008859462	0.094624659

c141.pcaec406	-0.086256638	0.051250891	0.094647387
smc181.c182	0.044271937	0.026305252	0.094651179
smc161.c142	0.074532815	0.044296575	0.094732933
pcaec384.spermidine	-0.116274114	0.069107851	0.094749277
lysopcac182.pcaac281	0.003176629	0.001889046	0.094923657
pcaac424.pcaec401	0.152434911	0.090671607	0.095007162
pcaac384.pcaac422	-0.089751175	0.053394487	0.095059465
smohc161.alphaaminoadipicacid	-0.014702446	0.008752141	0.095261876
c101.c10	-0.046659839	0.027783232	0.095348537
c10.lysopcac180	-0.114048905	0.067911493	0.09535807
pcaac240.pcaec423	0.085807418	0.051101342	0.095399825
pcaec340.smohc241	0.138001527	0.082188242	0.095413668
pcaac362.prolinr	0.048218844	0.028717538	0.095417158
pcaac383.transhydroxyproline	0.038044781	0.022659805	0.095440318
pcaac426.c181	-0.013440204	0.008006275	0.09548832
pcaec404.pcaec424	-0.08251129	0.049152525	0.09549422
pcaec424.ornithine	-0.05831692	0.034745451	0.095547805
serotonin.c141oh	0.203892567	0.121536538	0.095700586
asymmetricdimethylarginine.smc	0.019055309	0.011360055	0.095745184
putrescine.pcaec300	-0.013839835	0.008253087	0.095836794
c18.pcaec402	-0.042667996	0.025453941	0.095963449
smohc241.pcaec300	-0.009829084	0.005863745	0.095970477
acetylornithoine.pcaac323	0.184014282	0.109796197	0.096026307
smc180.smc202	0.108273443	0.064608353	0.096049759
acetylornithoine.citruline	0.022771628	0.013588372	0.096054794
glutamine.pcaac362	-0.019223699	0.011472179	0.096081768
pcaac383.c16	0.083607103	0.049895671	0.096089902
pcaac300.pcaec301	-0.111657926	0.066642738	0.096122934
lysopcac280.pcaac366	0.013728203	0.008194263	0.09614791
leucine.pcaac366	0.037085326	0.022138956	0.096192505
pcaec385.pcaac362	-0.035437955	0.021160537	0.096270625
pcaac383.citruline	0.007557856	0.004513383	0.096305155
c3.c142	-0.108343231	0.06470049	0.096306855
c141.pcaec300	-0.021897064	0.013076508	0.096306931
smohc221.lysopcac180	0.08889005	0.053084903	0.09631603
pcaac366.citruline	0.024693669	0.014748449	0.096348547
putrescine.pcaec404	0.031127988	0.018591622	0.096352715
pcaec403.asparagine	0.028582457	0.017071478	0.096356779
c162.smohc241	-0.092111243	0.055019305	0.096380222
lysopcac180.pcaec404	0.044991795	0.026888063	0.096550126
pcaac404.pcaac422	-0.071328679	0.042632717	0.096590124
pcaec380.glutamicacid	0.132616605	0.079271645	0.096621383
spermidine.c16	0.209462354	0.125232077	0.096689526

pcaec360.lysopcac182	-0.027818836	0.016633887	0.096723875
pcaac362.pcaec444	0.208979517	0.124957213	0.096726031
creatinine.serotonin	0.131210369	0.078462906	0.096755405
lysopcac204.c181	0.031107418	0.018602108	0.096756897
c2.c101	-0.024956088	0.014923864	0.096761896
pcaec386.citruline	0.005627989	0.003366848	0.096887841
lysopcac160.asymmetricdimethy	0.043375703	0.025949689	0.096899508
smohc221.c12	0.112049835	0.067034377	0.096900069
smc181.pcaec361	-0.016220518	0.00970427	0.096909048
pcaec422.pcaac366	0.032771874	0.019607104	0.096919723
pcaec423.pcaac420	-0.214876627	0.128572926	0.096956396
smohc141.pcaac281	0.034196649	0.020463534	0.096984368
pcaec384.pcaec322	0.179517926	0.107440177	0.097031445
c182.pcaec404	-0.016011181	0.009582928	0.09704362
pcaec343.smohc161	0.016675468	0.009980659	0.097048426
smc261.citruline	0.012755449	0.007634501	0.097051276
smohc222.citruline	0.006513272	0.003899003	0.097104182
kynurenine.pcaac344	-0.0607188	0.036349315	0.09711842
pcaac363.pcaec382	0.01546759	0.009260274	0.097139874
pcaec322.pcaec421	0.004155642	0.002487953	0.097142057
smc260.c182	0.073649226	0.044094885	0.097154221
c162.pcaec403	0.047737642	0.028586992	0.097220907
pcaac365.pcaac362	0.095570097	0.057235543	0.09724857
smc181.c162	0.213921384	0.128117693	0.097257056
c4.pcaac366	-0.043889775	0.026287175	0.097276622
arginine.pcaac281	-0.007587258	0.00454452	0.097293873
c162.lysopcac204	0.050159383	0.030044238	0.097298315
pcaac402.lysopcac280	-0.029306437	0.017555385	0.097327547
pcaec425.c12	0.08704868	0.052146158	0.097337314
pcaec381.lysopcac182	-0.096673042	0.057914117	0.097351757
pcaec360.citruline	0.01463554	0.008769407	0.097414669
pcaec403.pcaac281	-0.006808461	0.004081164	0.097547179
pcaac240.c162	-0.143425914	0.085977362	0.097563559
pcaac402.lysopcac204	0.180819524	0.108404739	0.097599049
creatinine.ornithine	-0.06543317	0.039228433	0.097599092
pcaec422.c181	-0.017228412	0.010330006	0.097639085
pcaec321.alphaaminoadipicacid	0.060113005	0.036044553	0.097651209
c3.pcaac320	-0.19446944	0.116612676	0.09766893
pcaac381.pcaac366	-0.082798106	0.04965177	0.097684164
pcaec385.spermidine	-0.1140066	0.068372068	0.097710452
smc261.tyrosine	-0.112025244	0.067190288	0.097742469
c3.pcaec300	0.008778997	0.005265514	0.097746542
pcaac385.citruline	0.004639484	0.002782929	0.097774507

glycine.asparticacid	0.059818432	0.035890467	0.097859854
pcaec322.citruline	0.009513483	0.005708354	0.097880695
methioninesulfoxide.citruline	0.022793991	0.013678865	0.097925358
h1.pcaec300	-0.029568615	0.017746951	0.097973644
pcaac360.pcaec300	-0.09320303	0.055944948	0.098002785
pcaec381.ornithine	-0.094271979	0.056587243	0.09800667
pcaec403.ornithine	-0.064025893	0.038437741	0.098057438
lysopcac161.pcaac362	0.010190661	0.006119221	0.098127605
pcaac383.pcaec443	-0.044137661	0.026504857	0.09814471
lysopcac160.pcaac405	0.152808358	0.091767362	0.098163861
pcaac405.pcaec322	0.12135525	0.072890481	0.098218356
pcaec361.transhydroxyproline	0.08751773	0.052576983	0.09828534
citruline.pcaec364	0.060020494	0.036067288	0.098372997
pcaec421.c18	0.096941272	0.058259266	0.098405509
pcaac344.pcaac366	-0.026295845	0.015806894	0.098484649
pcaec365.c12	0.088628184	0.053278115	0.098498135
c182.pcaec423	0.045335582	0.027255519	0.098527512
pcaac341.pcaec443	-0.032662751	0.019638488	0.098558243
pcaac405.pcaec340	0.150528477	0.090521708	0.098618744
c8.pcaec300	-0.03035108	0.018253359	0.098645306
alanine.pcaac362	0.011743108	0.007062502	0.09865059
lysopcac280.smc240	-0.097626387	0.058714158	0.098650659
pcaac320.pcaac402	-0.111957921	0.0673422	0.098694338
smc181.citruline	0.005449655	0.003278524	0.098753397
lysopcac170.pcaec300	-0.02093497	0.012594727	0.098758856
pcaac383.pcaec445	0.097116517	0.058435274	0.098809256
pcaec382.pcaec340	-0.098778356	0.059439742	0.098834732
pcaac405.pcaec363	-0.056174906	0.03380721	0.098874612
pcaac323.pcaac320	-0.166143246	0.099995382	0.098897896
pcaec425.pcaac362	0.013666727	0.008225564	0.098900893
pcaac364.pcaec362	-0.029091327	0.017513498	0.098984146
pcaac323.citruline	0.004204512	0.002531203	0.098985693
pcaac320.pcaac361	-0.124619421	0.075023938	0.098987816
asymmetricdimethylarginine.pcaa	0.006592409	0.003970005	0.09909002
pcaac300.pcaac366	0.03136093	0.01888657	0.099103309
pcaac405.pcaac422	-0.039257723	0.023642551	0.09910721
pcaac380.citruline	0.006510708	0.003921848	0.099179075
smc260.pcaec404	0.045359448	0.027330892	0.099274231
smohc161.c182	0.042472563	0.025600484	0.099392698
alanine.citruline	0.008384744	0.005054043	0.099399958
pcaec443.pcaec401	0.163718513	0.09869393	0.09943353
pcaac364.pcaac323	0.18415703	0.111053392	0.099549935
pcaac344.smc240	-0.069918183	0.042166441	0.099575632

arginine.leucine	-0.056681687	0.034185584	0.099593709
lysopcac203.pcaec404	0.020988662	0.012663085	0.099713052
pcaec401.pcaac362	-0.035677595	0.021528123	0.099756159
asparticacid.pcaac322	0.046852907	0.028274651	0.099794884
smohc222.pcaac361	0.091563824	0.055267328	0.099859678
pcaec322.pcaec385	-0.091662666	0.055327859	0.099864961
pcaec385.transhydroxyproline	0.088417101	0.053382507	0.099951028
smc261.c12	0.079333029	0.047900021	0.0999657
pcaac401.pcaec443	-0.101401479	0.061227854	0.099983556
pcaac323.pcaec404	-0.007364807	0.004448208	0.100075591
pcaac406.c181	-0.016193405	0.009782732	0.100151656
asymmetricdimethylarginine.pca	-0.019197071	0.011598035	0.10017291
smohc221.pcaac366	0.14211282	0.085864745	0.100197834
pcaac404.c51dc	-0.162555417	0.098242719	0.100288681
pcaec425.lysopcac180	-0.070297769	0.042487747	0.100306676
lysopcac140.pcaec445	-0.125061482	0.075588238	0.100313259
pcaec343.pcaac405	-0.056154849	0.033950165	0.100409335
pcaec405.lysopcac203	0.047242542	0.028565625	0.100452631
pcaac341.c182	-0.047132802	0.028502381	0.100489388
pcaac362.pcaec300	0.010156969	0.006143618	0.100568665
c181.prolinr	0.052596076	0.031813696	0.100569208
c51.glutamicacid	-0.126313617	0.076425376	0.100667465
pcaac384.pcaec443	-0.070816495	0.042849639	0.100687125
pcaec406.asymmetricdimethylarg	0.032571241	0.019708562	0.100693242
asparticacid.pcaac240	-0.047323474	0.028641469	0.100769366
c181.smc160	-0.120075177	0.072674285	0.100776252
smc240.pcaac365	0.128563624	0.077820574	0.100814145
c51dc.smohc221	0.042053606	0.0254567	0.10083165
lysopcac181.pcaec362	0.057475264	0.034798769	0.100896953
smohc141.c101	-0.021814676	0.013208317	0.100909228
smc180.c182	0.040184818	0.024341047	0.101048027
c51.pcaac426	0.062532685	0.037879156	0.101060438
pcaac360.smc240	-0.125296658	0.07591003	0.101112135
lysopcac140.pcaac402	-0.073865832	0.044765079	0.101218026
pcaec423.smc240	-0.088498502	0.053639985	0.101262364
alphaaminoadipicacid.kynurenin	-0.009635368	0.005842046	0.101374462
pcaac362.citruline	0.007875351	0.004775615	0.101423285
pcaec301.kynurenine	0.024695279	0.014975443	0.101428261
alphaaminoadipicacid.citruline	0.004350403	0.002638298	0.101450497
lysopcac281.pcaac281	0.005856991	0.003552024	0.101456037
smc181.pcaac366	0.124377685	0.075433742	0.101473019
smohc221.c142	0.081245916	0.049276919	0.10148769
pcaec380.pcaac366	0.152694558	0.092612762	0.101491796

pcaac403.smc240	-0.114706758	0.06957837	0.101521394
pcaec404.pcaac281	-0.006008832	0.003645684	0.101602244
pcaec385.smohc221	0.031514226	0.019122659	0.101643341
pcaec382.arginine	-0.235579671	0.142949586	0.101646028
lysopcac181.smc181	-0.030867932	0.018732196	0.101674157
pcaac421.serotonin	-0.212522839	0.12897358	0.101685073
pcaec363.pcaac406	-0.117258029	0.071163278	0.101699279
pcaec445.pcaac385	0.117518927	0.071329786	0.101738097
c16.pcaac281	-0.004819776	0.002925731	0.101772849
smc202.smc160	-0.137013481	0.083179236	0.101807184
pcaec341.alphaaminoadipicacid	0.007738022	0.004699466	0.101937517
pcaec404.pcaec405	-0.515717709	0.313221841	0.101954277
c181.c12	0.132475343	0.080488706	0.102079225
c182.lysopcac280	-0.029150545	0.017712234	0.102100146
c14.pcaac361	-0.079699704	0.048431829	0.102137281
kynurenine.smohc221	0.028379039	0.017246502	0.102160111
pcaac386.citruline	0.00665553	0.004045628	0.102238278
glutamicacid.citruline	0.027134631	0.016494973	0.102257356
pcaec383.pcaac281	-0.005565816	0.003383791	0.102294149
pcaec360.pcaac362	0.010849893	0.006596379	0.102298373
pcaac403.pcaec405	-0.249765028	0.151855638	0.102313384
pcaec424.pcaac366	0.017568495	0.010682128	0.102332112
serine.smc202	0.073490357	0.044695013	0.102414686
serine.pcaec340	-0.110987865	0.067500328	0.102415995
pcaec446.spermidine	-0.112652513	0.068517721	0.102440773
serine.pcaec363	0.043954516	0.026738769	0.102500192
pcaec341.asymmetricdimethylarg	0.057629372	0.035062366	0.102546748
methionine.pcaec300	-0.012260784	0.007460158	0.102572185
pcaac422.pcaac281	0.006074357	0.003696218	0.102593711
kynurenine.pcaec300	0.006856257	0.004172133	0.102604499
pcaac344.citruline	0.00622794	0.003789932	0.102617026
c12.c8	0.101056233	0.061502965	0.102653029
citruline.asparticacid	0.061219513	0.037259359	0.102662874
pcaec425.pcaac366	0.022954482	0.013973213	0.102728159
c12.pcaec421	0.005210934	0.003172129	0.102733187
putrescine.pcaec385	0.132969343	0.080967109	0.102828809
serotonin.c51	0.189898192	0.115652259	0.102888585
pcaac362.putrescine	-0.042096225	0.025638246	0.102897907
pcaac405.putrescine	-0.089094704	0.054262394	0.10289939
lysopcac161.lysopcac181	0.199998552	0.121823198	0.102943414
c51.pcaec361	-0.015027734	0.009156899	0.103062575
lysopcac181.pcaac281	0.003788201	0.002308406	0.103081666
pcaec446.pcaac362	0.06994509	0.042623285	0.103089893

asymmetricdimethylarginine.glut	-0.019073344	0.011627342	0.1032187
smc261.lysopcac203	-0.022508054	0.013722506	0.103251615
pcaec382.pcaac281	-0.007354073	0.004483816	0.10327076
pcaac365.citruline	0.008521297	0.00519552	0.103273499
glycine.pcaac300	-0.076526647	0.046659652	0.103277822
smc160.tyrosine	-0.107377752	0.065480364	0.103331266
c16.pcaac366	0.027978578	0.017062889	0.103354974
pcaec423.c14	-0.115838832	0.070646528	0.103362625
pcaec381.pcaac281	-0.007834952	0.004778604	0.103384644
phenylalanine.pcaac341	0.009254998	0.005644852	0.103393767
smc241.c182	0.039578155	0.024141268	0.10341603
pcaac386.pcaac405	-0.070589126	0.043062089	0.10345754
ornithine.pcaec362	-0.014013202	0.008550319	0.103526524
alanine.transhydroxyproline	-0.038123922	0.023268256	0.103622049
pcaac421.pcaac381	0.101280964	0.06181795	0.103638206
acetylornithoine.lysopcac203	-0.042735442	0.026087943	0.103689306
pcaec362.pcaec443	-0.089528304	0.054663173	0.103754578
pcaec402.pcaac366	0.030865978	0.018846573	0.103768651
pcaec301.pcaac344	-0.153390218	0.093660385	0.103773327
asparticacid.pcaec424	-0.038910184	0.02376053	0.103800578
c51dc.citruline	-0.006051912	0.00369608	0.103844634
pcaac402.pcaec402	-0.316771327	0.193463832	0.103848726
smohc141.alphaaminoadipicacid	-0.023097844	0.014108721	0.10389784
pcaec342.c181	-0.020108345	0.012290708	0.104122204
pcaac422.c182	-0.081987348	0.05011322	0.10412608
pcaec340.pcaac322	-0.132960495	0.081275579	0.104151325
pcaac362.asymmetricdimethylarg	0.031934707	0.019521718	0.104165266
pcaec360.smohc221	-0.082788699	0.050609691	0.104171209
ornithine.citruline	0.004850965	0.002965593	0.104187699
c4.pcaac281	0.01060574	0.006483728	0.104187939
pcaac361.pcaec343	0.174316429	0.106574545	0.104212701
smc260.pcaac363	-0.024423589	0.014932857	0.104227131
pcaec322.smohc221	0.021343954	0.013052522	0.104295152
pcaec421.pcaec405	-0.238841712	0.146065167	0.104308538
c51dc.smohc241	0.101692986	0.0621965	0.104338932
pcaac424.smc240	-0.08188751	0.050085695	0.104355602
pcaac380.glutamicacid	0.078604336	0.048078244	0.104360389
pcaac344.leucine	-0.013366867	0.008175992	0.104367309
phenylalanine.citruline	0.00359082	0.002196487	0.104386424
pcaec362.c141oh	-0.164430485	0.100601365	0.10445481
c142.smohc241	-0.113218097	0.06927592	0.104490326
lysopcac204.pcaec422	0.072930017	0.044634636	0.104568824
c2.pcaac422	0.139489026	0.085385149	0.104629444

smc260.c12	0.085742865	0.052488582	0.10464887
creatinine.asparticacid	0.08774499	0.053715488	0.104657064
ornithine.phenylalanine	0.098758574	0.060464426	0.104694997
smc160.pcaac405	-0.057180292	0.035014947	0.10475991
methionine.lysopcac204	-0.043243366	0.026484376	0.10480992
pcaac426.pcaac281	0.00619245	0.003793112	0.10485979
pcaec404.pcaac422	-0.173462606	0.106277701	0.104941466
pcaec342.alphaaminoadipicacid	0.023704677	0.01452581	0.10499697
lysopcac281.pcaac362	-0.045680464	0.027993016	0.105007175
pcaec365.c14	-0.07036272	0.043120519	0.105024759
smohc141.asymmetricdimethyla	0.042193534	0.025862624	0.105092423
pcaec340.citruline	0.009378386	0.005748872	0.105114506
smc260.pcaac281	0.005390935	0.003305719	0.105231426
histidine.pcaec423	-0.057774684	0.035436369	0.105318543
pcaac361.c181	-0.010023959	0.006148684	0.105343322
c141oh.pcaac405	-0.10507382	0.064471823	0.105448627
pcaec301.lysopcac182	-0.031118312	0.019094255	0.105457572
pcaac384.lysopcac280	0.008726525	0.005354654	0.105460273
pcaac362.smc160	-0.205558924	0.126141901	0.105486454
c141oh.pcaac322	-0.105093196	0.064498903	0.105529915
pcaac422.lysopcac182	-0.148096792	0.090895272	0.105544153
lysopcac280.pcaec404	0.025342479	0.015555214	0.105568988
pcaec446.pcaec364	0.050048943	0.030724227	0.105616022
pcaec404.lysopcac203	0.029416397	0.018061464	0.105677574
pcaac402.pcaac366	0.043930542	0.026974836	0.105700476
pcaec340.pcaac405	0.099883658	0.061335966	0.105723162
pcaac405.pcaec420	-0.089209921	0.054801474	0.105849138
pcaec300.c141oh	-0.187422687	0.115134628	0.10585289
pcaac360.pcaac361	0.094495364	0.058053454	0.105879762
citruline.pcaac320	-0.110537076	0.067909416	0.105883314
pcaac362.smc240	-0.111457544	0.068475559	0.10588657
pcaac320.c16	0.07310547	0.044913465	0.105887027
serine.c8	0.092869896	0.057056376	0.105889047
pcaec300.asparticacid	-0.053330679	0.032769081	0.105935163
lysine.pcaac281	-0.00475986	0.00292526	0.106001365
c18.citruline	-0.003542492	0.002177247	0.106024173
c16.c101	-0.016153243	0.009928403	0.106040733
putrescine.pcaac365	0.053808875	0.033082893	0.106144222
pcaac320.lysopcac161	0.180512777	0.110988809	0.106161498
pcaec363.c101	-0.014707672	0.009044919	0.106232845
pcaec301.c12	0.089964418	0.055328247	0.106245125
c51dc.pcaac366	0.041189092	0.025334335	0.106285915
c101.pcaec443	-0.115434196	0.071011915	0.106341321

c14.c101	-0.022315522	0.013728216	0.106349547
pcaac362.c182	-0.092553194	0.056943185	0.106384089
c182.pcaec300	0.006929313	0.00426333	0.106390792
isoleucine.pcaec403	-0.115650955	0.071158422	0.106405429
smohc141.smc160	0.271926772	0.167315587	0.106411291
glycine.pcaec380	-0.098706758	0.060736455	0.10642575
pcaac363.lysopcac280	0.015097321	0.009289962	0.106434928
smc260.lysopcac180	0.100445202	0.061809704	0.106445638
pcaec444.pcaac366	0.01395806	0.008589434	0.106455227
pcaec365.pcaac380	0.170042351	0.104642469	0.106464323
pcaac425.pcaac366	0.05724669	0.035229317	0.10646669
smohc161.tyrosine	-0.132644831	0.081632654	0.10648245
smc261.smohc141	-0.134687474	0.082896575	0.106511024
asparagine.glutamine	0.059646128	0.036711572	0.106520081
lysopcac170.alphaaminoadipicac	-0.004520217	0.002782235	0.106531024
pcaec403.pcaac366	0.026589392	0.016366062	0.106532095
pcaac320.c181	-0.007942745	0.004889603	0.106585813
smohc161.c12	0.124176629	0.076464026	0.106677091
histidine.citruline	0.008650158	0.005327343	0.106732487
creatinine.pcaec405	-0.090503089	0.055746652	0.106787457
pcaec406.pcaac362	-0.027857048	0.017166561	0.106941648
pcaec364.pcaec361	-0.025658147	0.015812092	0.106954314
pcaec381.pcaec365	0.041124739	0.02534495	0.106973713
c2.pcaec443	-0.140434676	0.086585338	0.107119165
pcaac365.transhydroxyproline	0.062753544	0.03869114	0.107121778
c51dc.glutamine	0.035438403	0.021849886	0.107123193
pcaec420.lysopcac160	0.046548046	0.028705071	0.107188974
pcaac421.pcaec362	0.028202241	0.017394152	0.107239006
lysopcac204.c18	-0.056328476	0.03474706	0.107295429
pcaec322.pcaec361	-0.012447614	0.007679256	0.107329856
lysopcac280.pcaec365	0.04180154	0.025788979	0.107336947
pcaec386.asparticacid	-0.0737489	0.045500427	0.107351419
pcaec363.pcaec361	-0.016811033	0.010372384	0.10737085
c181.pcaec381	0.107386328	0.066268842	0.107431955
phenylalanine.pcaac426	0.093775149	0.057869926	0.107435786
pcaac406.citruline	0.004486866	0.002769878	0.107558031
pcaec420.c8	-0.095561409	0.05899327	0.107559906
c18.pcaec401	-0.065909641	0.040688777	0.107564535
pcaac421.lysopcac182	-0.156299037	0.096501119	0.107604938
pcaec382.c181	-0.047006867	0.029037075	0.107777939
pcaac362.lysopcac280	0.011532592	0.007124863	0.107824557
pcaac320.citruline	0.011097398	0.006856031	0.107826168
pcaec405.pcaac362	0.028576409	0.017655761	0.107847997

pcaec361.pcaec340	-0.123263211	0.076165244	0.107883814
lysopcac161.pcaec340	0.061368316	0.037923022	0.107912238
isoleucine.pcaec423	0.029581724	0.018281995	0.107945477
pcaac383.smohc161	-0.039643848	0.024501325	0.10795647
pcaec321.serotonin	-0.1673293	0.103419261	0.107969057
pcaac363.smc202	0.060492734	0.037388165	0.107970262
pcaec360.transhydroxyproline	0.084680899	0.052340709	0.107988917
pcaac420.pcaec362	0.025065369	0.01549482	0.108035766
c10.lysopcac170	-0.0477608	0.029526191	0.108054592
pcaac421.pcaec300	0.019591375	0.012111621	0.108055805
pcaec380.smohc241	0.128608284	0.079509483	0.108065973
pcaec364.alphaaminoadipicacid	0.006613916	0.004089108	0.108081974
pcaac404.pcaec443	-0.049549586	0.030638034	0.108122968
putrescine.citruline	-0.00690968	0.004272532	0.108128204
smc181.pcaac360	0.091508903	0.056612021	0.108303578
pcaec321.citruline	0.008641957	0.005348686	0.108456299
lysopcac203.citruline	-0.007632345	0.004724229	0.108486869
lysopcac160.citruline	-0.016926474	0.010477333	0.108496017
h1.prolinr	-0.072789866	0.04506376	0.108554231
pcaec420.c51	0.193526056	0.119812418	0.10855897
pcaac341.lysopcac161	0.1594938	0.098759578	0.108617837
pcaec445.pcaec384	0.109779038	0.067976891	0.108623042
lysopcac181.pcaec405	-0.061276174	0.03794731	0.108661314
h1.pcaec340	-0.142803078	0.088437985	0.108670925
pcaac343.c2	-0.151147571	0.093608169	0.108680045
smc160.putrescine	-0.056516625	0.035004961	0.108712947
smc240.pcaac344	-0.086107699	0.053333591	0.108717355
pcaac420.smohc221	0.080908021	0.050119102	0.108759988
pcaec343.pcaec443	-0.058513758	0.036250027	0.108791166
pcaec423.pcaac361	0.112013811	0.06940289	0.108836054
pcaec406.c16	0.098472779	0.061014652	0.108845703
smohc141.c162	0.159863252	0.099054499	0.108851853
asparagine.lysopcac204	-0.013897078	0.008611551	0.108877918
pcaac403.ornithine	-0.087541088	0.054256449	0.10894376
pcaac381.lysopcac182	-0.031803995	0.019715298	0.109010158
asparagine.pcaec405	-0.081641258	0.050615939	0.109055348
pcaac405.prolinr	0.043627897	0.027049128	0.109064538
c181.pcaec361	-0.023866275	0.014797122	0.109067454
pcaac366.glycine	0.095303981	0.059095868	0.109110652
smc261.pcaac281	0.002665252	0.001653243	0.109233725
pcaec365.prolinr	0.044851019	0.02782103	0.109235773
methioninesulfoxide.pcaec300	-0.0227507	0.01411364	0.109270849
asparticacid.lysopcac180	-0.135529197	0.084081432	0.109289403

pcaac404.alphaaminoadipicacid	0.005757191	0.00357231	0.109347051
lysopcac281.pcaac365	-0.114470134	0.071029144	0.109351802
lysopcac280.prolinr	-0.048235472	0.029930393	0.109353011
pcaac321.pcaac426	0.151812282	0.094212574	0.109398365
pcaac321.pcaec300	-0.031239831	0.019388543	0.109426297
pcaac380.lysopcac182	-0.022273462	0.013823896	0.109431349
c10.c181	-0.054117627	0.033588834	0.109442231
isoleucine.c18	0.072588603	0.045055875	0.109463925
lysopcac204.lysopcac161	-0.133266792	0.082738337	0.10954636
pcaec301.smohc222	-0.009361459	0.005813372	0.109627102
c181.pcaec300	0.012766763	0.007928729	0.109658045
pcaec424.lysopcac280	-0.012321991	0.007653754	0.109715418
pcaec384.pcaec360	-0.0110374	0.006856393	0.109744025
smc240.pcaec300	-0.016421774	0.010202484	0.109790166
smohc241.glutamine	-0.021397591	0.013296832	0.109869136
c162.pcaec300	-0.05048577	0.031372966	0.109871813
pcaac385.isoleucine	0.057303958	0.035614318	0.109915115
lysopcac181.serotonin	-0.186018957	0.115627563	0.109967145
lysopcac180.pcaac323	-0.125541956	0.078043932	0.11000452
alphaaminoadipicacid.pcaec300	0.011336912	0.007049128	0.110077859
putrescine.ornithine	0.078759174	0.048975796	0.110110074
c0.pcaac365	-0.100816522	0.062700721	0.110159158
pcaac425.leucine	-0.0378326	0.023529443	0.110162918
citruline.pcaac281	-0.005046348	0.003138533	0.110166216
pcaec381.c182	-0.089206674	0.055486648	0.110200004
pcaac383.c51dc	-0.191292936	0.118993407	0.11022665
pcaac344.lysopcac280	0.011607452	0.007220427	0.1102283
pcaac362.asparticacid	0.026605475	0.016556371	0.110364859
pcaac406.lysopcac170	0.013411224	0.008346075	0.110380909
lysine.citruline	0.004117139	0.002562269	0.110393343
pcaec301.leucine	-0.024530385	0.015267229	0.110415023
c8.pcaac320	-0.078650099	0.048955821	0.110454999
pcaac300.lysopcac203	0.026291697	0.01636718	0.110495786
c5.pcaec362	-0.028549432	0.017772943	0.110501191
pcaac365.alphaaminoadipicacid	0.006202025	0.003861423	0.110543509
smc260.tyrosine	-0.184066569	0.114604548	0.110554075
lysopcac280.pcaac281	-0.004712732	0.002934605	0.110594713
pcaec362.alphaaminoadipicacid	-0.012875953	0.008017829	0.1105951
smohc241.c141	0.069595733	0.043337524	0.110598431
pcaac401.lysopcac161	0.141652131	0.088210024	0.110609284
pcaac320.pcaec445	0.090809309	0.056555735	0.110651134
pcaec341.pcaec421	0.003212139	0.002000551	0.110658466
pcaac323.smohc221	0.012867084	0.008013783	0.110660213

methionine.pcaec402	-0.088185317	0.054939075	0.110764058
pcaac323.pcaec361	-0.008616273	0.005369126	0.110844701
pcaec402.lysopcac203	0.041166475	0.025653601	0.110861413
lysopcac182.citruline	-0.006484848	0.004041676	0.110907872
putrescine.lysopcac280	0.016399218	0.01022401	0.111018993
pcaac300.c142	0.043256859	0.026970335	0.111046063
pcaec342.lysopcac204	-0.0494369	0.030833601	0.11116153
c12.lysopcac181	-0.142268267	0.088745533	0.111214888
pcaac405.citruline	0.005935834	0.00370283	0.111225975
pcaac404.smohc161	-0.025654561	0.01600425	0.111241205
asparagine.pcaec423	-0.019245466	0.012006521	0.111255909
pcaec406.c51	0.095075804	0.059317471	0.111275368
c102.pcaac344	-0.078570883	0.049032652	0.11136603
lysopcac181.pcaac381	-0.080833624	0.050446363	0.111377509
c182.pcaac281	0.008855004	0.005526918	0.111423559
arginine.pcaec443	0.11401115	0.071186759	0.111552273
pcaec381.lysopcac280	-0.019652197	0.012270877	0.111562559
lysopcac280.pcaac422	-0.048134132	0.030057019	0.111585645
smohc141.valine	-0.143616321	0.089691714	0.111631202
phenylalanine.c141	-0.041625816	0.026002177	0.111711617
pcaec364.pcaac320	0.172835383	0.10797162	0.11173615
putrescine.pcaec424	0.125355263	0.0783207	0.111782763
pcaec340.c101	-0.019454694	0.012155175	0.111785074
lysopcac160.c51	-0.137108974	0.08566822	0.111799009
pcaac405.c51dc	-0.202877277	0.126768378	0.111818356
pcaac404.c142	-0.07308109	0.045665054	0.111819524
lysopcac160.alphaaminoadipicac	-0.005539118	0.003461196	0.111824951
pcaac404.smc161	-0.066681686	0.041672341	0.111870523
pcaac364.pcaac366	-0.020351099	0.01272109	0.111948339
smohc141.pcaac362	-0.009515563	0.005948112	0.111955065
lysopcac170.pcaec404	0.035720657	0.022332575	0.112016167
pcaec343.c5	0.032384895	0.020251602	0.112095974
smc240.c2	0.08808586	0.055088869	0.112129359
pcaac405.pcaac362	0.020723861	0.012960837	0.112133237
pcaec342.pcaac406	-0.115003866	0.071931246	0.112168237
arginine.citruline	0.026572017	0.016620181	0.112173214
asymmetricdimethylarginine.c18	0.076281794	0.047712511	0.112173339
lysopcac140.smohc161	-0.02568324	0.01606508	0.11219112
pcaec446.c14	-0.069451622	0.043445222	0.112212858
pcaec446.valine	0.144239963	0.090242523	0.112266913
lysopcac182.pcaec405	-0.068411305	0.042804823	0.112299027
pcaec341.c181	-0.009847383	0.006163003	0.112386468
leucine.citruline	0.00412962	0.002585296	0.11249199

pcaac384.c8	-0.078735852	0.049300763	0.112558299
pcaec424.pcaac281	-0.00519676	0.003254027	0.112564356
pcaec446.pcaec361	-0.018242384	0.011423006	0.112572813
pcaac321.lysopcac280	0.027792999	0.017404206	0.112589216
c162.c102	0.098091107	0.061425767	0.112590978
smohc222.pcaac366	-0.01872549	0.01172627	0.112595686
pcaac363.smohc221	0.03070102	0.019225807	0.112599854
smc202.c182	0.044509687	0.027876792	0.112646202
lysopcac181.putrescine	-0.112873798	0.070698517	0.112669918
kynurenine.pcaec404	-0.015153835	0.009491888	0.112680495
c3.pcaac281	-0.009470014	0.00593208	0.112702131
smc160.valine	-0.109353481	0.068500333	0.112705128
serotonin.kynurenine	-0.025965356	0.016267388	0.112757237
alanine.smohc161	-0.015843684	0.009928466	0.112841329
c12.pcaec340	0.05756442	0.036073196	0.112845037
pcaac380.transhydroxyproline	0.082768192	0.051876128	0.112905479
citruline.pcaec423	0.035567912	0.022295878	0.112956519
c141oh.pcaec362	0.024542698	0.01538561	0.112978048
smohc161.c141oh	-0.150480992	0.094338899	0.112991804
pcaac360.asymmetricdimethylar[illegible]	0.083401622	0.052288603	0.113011018
lysopcac182.pcaec404	0.028577767	0.017923233	0.113138855
pcaec420.pcaac405	-0.067565314	0.042380854	0.11318642
c51.citruline	0.00326338	0.002047624	0.113298791
lysopcac182.c51	-0.108127251	0.067846699	0.113307887
glycine.pcaec361	0.012895467	0.008092045	0.113330624
pcaec444.citruline	-0.003696898	0.00232003	0.113359357
smc260.pcaac401	0.066225	0.041568764	0.113432644
pcaac383.pcaec300	0.035876872	0.022521583	0.113464912
pcaec381.pcaac422	-0.115403725	0.072446368	0.113475165
c5.pcaec361	-0.044352883	0.027846004	0.113511586
creatinine.pcaac361	-0.083159784	0.052216792	0.113557554
pcaec383.pcaec381	0.050440808	0.03167439	0.113581766
c0.citruline	-0.010238047	0.006429474	0.1136082
c18.c141	0.040633554	0.025517822	0.113608589
lysopcac203.alphaaminoadipicac	-0.014165774	0.008896864	0.113639781
pcaac420.pcaac381	0.079091734	0.049692346	0.113773108
pcaac342.alphaaminoadipicacid	0.020986693	0.01318705	0.113810404
pcaec382.pcaec404	0.075166551	0.047233088	0.113825313
pcaac385.pcaec446	-0.083522787	0.052490578	0.113870389
c16.pcaac361	-0.069717931	0.043820044	0.113913232
pcaac426.smohc221	-0.100027209	0.062870926	0.113916023
lysopcac140.lysopcac280	0.010417276	0.006549244	0.1140029
pcaac361.lysopcac203	0.136673727	0.085933399	0.114035872

lysopcac161.pcaac426	-0.153639157	0.096600773	0.114037304
pcaec342.c101	-0.01340767	0.008430849	0.11406975
pcaac360.c142	0.081316268	0.051135811	0.114094275
glutamine.citruline	0.011933842	0.007505315	0.114128062
c8.smc160	-0.108493275	0.068232681	0.114128876
pcaac401.pcaec401	0.174286734	0.109611059	0.114129229
smc261.asparticacid	0.06145903	0.038654021	0.11414486
c5.pcaec300	0.026860834	0.016896498	0.114201217
smc161.c182	0.044462962	0.027971143	0.114229806
pcaac403.pcaec423	-0.099042921	0.062315566	0.11428042
lysopcac280.lysopcac182	-0.022468729	0.014138449	0.114321878
pcaec301.pcaec443	0.091314856	0.057459999	0.11432262
pcaec341.asparagine	0.01304626	0.008209673	0.114335567
asymmetricdimethylarginine.asp	0.043235678	0.027207717	0.114343814
smc241.histidine	-0.080140645	0.050435758	0.114373484
pcaac364.alphaaminoadipicacid	0.003339385	0.002101613	0.114373962
smc161.c141oh	-0.14564755	0.091666235	0.11439042
acetylornithoine.pcaac361	-0.040442045	0.025455951	0.114431762
pcaac385.prolinr	0.033292909	0.020961474	0.114525901
serotonin.c162	0.099950366	0.062939981	0.114585577
pcaac425.pcaec300	-0.030717636	0.019343508	0.114589895
c4.pcaac320	-0.19842101	0.12495862	0.114615645
pcaec443.smc240	-0.071511939	0.045036347	0.114620642
glutamicacid.pcaac362	0.031232728	0.0196768	0.114753
pcaac420.pcaec300	0.02098936	0.013225852	0.114819186
pcaac300.pcaec300	-0.017965405	0.011320527	0.114823453
lysopcac170.pcaac366	-0.04912871	0.030958722	0.114838389
pcaac404.citruline	0.012782241	0.008056146	0.114898586
lysine.asymmetricdimethylargini	0.028661954	0.018065482	0.114917981
pcaec365.smc160	0.274471931	0.172999694	0.114920988
lysopcac170.pcaec365	0.049279218	0.031065667	0.114978547
pcaec420.c181	-0.014069595	0.008869576	0.114982199
citruline.lysopcac204	0.078831304	0.049716206	0.115129726
lysopcac281.phenylalanine	0.106635827	0.06725763	0.115162187
pcaec383.pcaac366	0.038335849	0.024180393	0.115178641
prolinr.lysopcac180	0.163205643	0.102956449	0.115228505
pcaec342.pcaac320	0.147785845	0.093231778	0.115239074
pcaec341.citruline	0.007348565	0.004636104	0.115255291
serotonin.c12	0.082383699	0.051990238	0.115363026
pcaec386.alphaaminoadipicacid	0.007404695	0.004673159	0.11538197
pcaec322.smc202	0.120933803	0.076327924	0.115408857
c51.kynurenine	-0.007976945	0.005035264	0.115450253
pcaac405.c141	-0.069510815	0.043891221	0.115566241

pcaac344.pcaec404	-0.042863779	0.027067144	0.115588414
c102.pcaac361	-0.075116036	0.0474353	0.115602548
glutamine.c18	-0.121795116	0.076917015	0.115622119
pcaac320.asymmetricdimethylar[illegible]	0.088912328	0.056162455	0.115698195
isoleucine.citruline	0.006287018	0.003971338	0.115704816
isoleucine.lysopcac161	-0.169508039	0.107077015	0.115716353
pcaec385.c14	-0.06448141	0.040733669	0.115727197
pcaec420.smc240	-0.041282088	0.026078527	0.115729198
pcaac366.pcaec405	-0.094594612	0.059765655	0.115782293
c18.valine	-0.052044493	0.032884843	0.115811985
pcaac361.pcaec445	0.081030988	0.051204589	0.115842546
c182.pcaec401	-0.084841724	0.053616293	0.115867113
pcaac406.pcaac405	-0.101772188	0.064324151	0.115915066
pcaac383.asymmetricdimethylar[illegible]	0.051238754	0.032391707	0.115990211
smc241.tyrosine	-0.103380782	0.065358235	0.116011197
pcaac421.c51	0.183965354	0.11630467	0.116011709
pcaec406.pcaec342	0.249799758	0.157929479	0.116020092
smohc221.pcaec362	0.072462004	0.045813972	0.116033755
pcaec424.citruline	-0.004243625	0.002683046	0.116036695
c8.lysopcac180	-0.118028574	0.074667136	0.116245825
pcaac343.citruline	0.015753889	0.009966567	0.11625872
lysopcac203.pcaac322	0.149078877	0.094340265	0.116361334
lysopcac161.lysopcac180	-0.092188496	0.058340077	0.116369075
pcaac385.c8	-0.070729686	0.044767673	0.116429519
smc202.pcaac281	0.002493686	0.001578431	0.116447079
h1.pcaac281	0.004434758	0.002807727	0.116531376
smohc221.pcaec340	-0.054596949	0.034572785	0.116598949
pcaec405.c5	-0.056969025	0.036075597	0.11660624
c14.citruline	0.015117238	0.009574776	0.116674179
smc261.pcaec362	0.034471455	0.021833847	0.11668626
glutamine.smohc221	0.115498571	0.073167392	0.116744935
isoleucine.creatinine	-0.020360412	0.012900834	0.11682027
pcaac322.asymmetricdimethylar[illegible]	0.030709775	0.019458811	0.116827325
pcaac343.leucine	-0.014348668	0.009092157	0.116840408
pcaec401.citruline	0.093364167	0.059164284	0.116860488
pcaac405.smohc161	-0.020433229	0.012948752	0.116870113
pcaac361.c182	-0.045980705	0.029145311	0.116955428
pcaac405.pcaac323	-0.200307477	0.126987374	0.117014171
c182.arginine	0.088686409	0.056240666	0.117122812
lysopcac204.pcaec340	0.125665637	0.0797157	0.117234799
alanine.pcaec423	-0.008810648	0.005589138	0.117242871
pcaec340.c181	-0.014028203	0.008900318	0.117298484
c181.citruline	0.010834616	0.006874225	0.117304129

creatinine.pcaec300	0.026105685	0.016565424	0.117352008
pcaac342.pcaec444	0.165623784	0.105118808	0.117427541
pcaec361.pcaac385	-0.066525022	0.04222349	0.117437057
smc241.glutamicacid	0.056591901	0.035920556	0.117453523
c142.pcaec360	-0.013317798	0.008453246	0.117455456
pcaac426.c141oh	-0.186563909	0.118433888	0.117503402
pcaac403.pcaac402	-0.483253917	0.306784661	0.117511691
pcaec421.citruline	-0.010689068	0.006785942	0.117521768
c18.pcaac405	0.101047446	0.064153631	0.117543203
c51.pcaec423	0.023249065	0.014761147	0.117558861
lysopcac182.smc160	-0.125102796	0.079434932	0.117583909
pcaec383.alanine	-0.03608509	0.022917249	0.117659433
leucine.pcaac402	-0.090484473	0.057472432	0.117702016
c4.citruline	0.017271017	0.010969947	0.117702934
pcaac380.smc240	-0.106600946	0.067719561	0.117758622
pcaec406.pcaec361	-0.020268728	0.012877161	0.117792619
pcaec382.c182	-0.084538874	0.053710689	0.117801523
c10.pcaec340	0.060726601	0.038582571	0.117808168
pcaac363.alphaaminoadipicacid	0.004626879	0.002940207	0.11787319
pcaec444.lysopcac280	-0.013931305	0.00885352	0.117902023
putrescine.alphaaminoadipicacid	0.005563272	0.003535692	0.117918819
pcaec381.pcaac366	0.033603065	0.021361023	0.118001828
pcaac420.pcaec340	0.049402381	0.031407775	0.11804054
putrescine.pcaec340	0.097885654	0.062235208	0.118063861
spermidine.pcaac320	0.200864917	0.127725872	0.118112268
alanine.c101	0.02009154	0.012776473	0.11813154
lysopcac204.smc202	-0.067595772	0.042987615	0.118153422
phenylalanine.asymmetricdimeth	0.073613203	0.046818349	0.118184105
pcaec384.smohc221	0.053478388	0.03401505	0.118211327
c8.pcaec424	-0.021967965	0.013973744	0.118236629
pcaec420.pcaac385	-0.09878399	0.062843706	0.118280532
pcaec425.pcaec340	0.037727991	0.024002703	0.118298374
pcaec382.pcaac366	0.034658589	0.022050217	0.118303001
lysopcac160.pcaec421	-0.009773394	0.006218716	0.118347761
smc160.alphaaminoadipicacid	-0.014940203	0.009506325	0.118348529
pcaec343.c101	-0.014405134	0.009166272	0.1183647
pcaec405.pcaac366	0.046758293	0.029754537	0.118380747
c102.c162	0.183600319	0.116839158	0.118397919
smc261.prolinr	-0.0624122	0.039719774	0.118416649
pcaec385.pcaec322	0.13699187	0.087225667	0.118595022
arginine.pcaec402	0.064039329	0.040775794	0.118600191
pcaec342.serotonin	-0.169261033	0.107778796	0.11861759
pcaac421.tyrosine	-0.104309439	0.066437829	0.118714711

pcaec422.citruline	-0.029406525	0.018731919	0.118754035
pcaac385.pcaec443	-0.070551741	0.044953849	0.118855503
serotonin.asparticacid	0.081693187	0.052055845	0.11887613
smc202.prolinr	0.024033379	0.01531527	0.118898153
c181.c101	-0.010938568	0.006972394	0.118992127
pcaac240.pcaac365	-0.135928275	0.086647242	0.11901192
c162.phenylalanine	0.055656111	0.035478514	0.119018338
glutamine.smc202	0.09281173	0.059167693	0.119042934
pcaac422.lysopcac280	-0.019293231	0.012300528	0.119074136
pcaac363.pcaec365	0.047864559	0.030535394	0.119302242
pcaac425.pcaec421	0.003529733	0.00225181	0.119302603
c51.c16	-0.06540288	0.041725883	0.119318422
pcaec342.pcaec361	-0.015199711	0.009697799	0.119343024
lysopcac280.asparticacid	-0.095145321	0.060706432	0.119350976
spermidine.citruline	0.039028051	0.024903437	0.119380616
pcaac281.pcaac405	0.068499642	0.043709316	0.119383394
ornithine.pcaac344	-0.081891314	0.052265382	0.1194598
pcaac403.pcaac422	-0.148463428	0.094760103	0.119484896
pcaac364.isoleucine	0.05979586	0.03816646	0.119488889
c12.alphaaminoadipicacid	-0.007126139	0.004548879	0.119522036
smc260.smc202	0.067200778	0.042903436	0.119579188
pcaac361.citruline	0.005500977	0.003512545	0.119633412
c101.citruline	-0.005479082	0.003498732	0.119650995
pcaec382.alanine	-0.041138802	0.026270004	0.119655782
lysopcac203.smc240	-0.087647007	0.055977826	0.119715255
acetylornithoine.lysopcac204	0.09095661	0.058092009	0.119717947
lysopcac170.pcaac281	-0.007500014	0.00479108	0.119793339
pcaec360.alphaaminoadipicacid	0.009081689	0.005801932	0.119822606
pcaec380.smohc221	0.109971001	0.070289146	0.119995053
pcaac403.pcaac366	0.046964059	0.030019902	0.120023428
pcaac281.asparticacid	0.03589166	0.022954462	0.120217912
histidine.pcaec361	0.011370943	0.007272451	0.120226998
pcaec420.glycine	0.087480841	0.055951689	0.120240477
pcaec343.pcaac360	0.075730184	0.048436677	0.120244773
pcaac281.smohc161	0.057045156	0.03648793	0.120266032
pcaec446.c181	-0.016000187	0.010234935	0.120291062
asymmetricdimethylarginine.pca	0.023166702	0.014819949	0.120310074
c2.pcaec300	0.049559803	0.031704588	0.120318581
c2.c51	0.139101545	0.088991114	0.120337332
serotonin.glycine	0.061078364	0.039078728	0.12036978
pcaec445.pcaec423	0.027829276	0.017805565	0.120370496
pcaac344.lysopcac203	0.02988319	0.019124123	0.120455761
pcaec360.c102	0.106978616	0.068481189	0.120557205

pcaac364.smohc222	-0.009538157	0.006106511	0.120603225
pcaac360.pcaac320	0.091125485	0.058344726	0.120631294
glycine.c162	0.113904309	0.072929325	0.120631537
lysopcac181.c18	-0.06105519	0.039106164	0.120767667
creatinine.pcaec364	0.049183119	0.031502371	0.120771526
pcaec422.pcaec380	-0.206075604	0.13199691	0.120780009
pcaec444.pcaec423	0.024923841	0.015964403	0.120780458
pcaec401.pcaec405	-0.111352556	0.071327465	0.120796475
pcaac361.pcaac281	0.005744106	0.003680236	0.12087848
pcaac383.c182	-0.073562899	0.047144978	0.1209831
pcaac300.citruline	0.00799996	0.005127079	0.120987831
creatinine.pcaac320	-0.130333019	0.083531782	0.121000665
pcaac343.c142	0.030540446	0.019574583	0.121017518
smohc161.c101	-0.029385819	0.018836016	0.12104647
pcaec360.pcaac405	-0.067179371	0.043063916	0.121068821
pcaac323.pcaac240	-0.020725053	0.013286795	0.121108729
smohc241.pcaec404	0.017313429	0.011101364	0.12116702
ornithine.c14	0.044641882	0.028628524	0.121220712
pcaac361.alphaaminoadipicacid	0.009871485	0.006331261	0.121264266
c16.pcaec382	-0.02551391	0.016364836	0.121287127
kynurenine.asparagine	-0.022650733	0.014528716	0.1212959
c51.c102	0.045854851	0.02941335	0.121307993
pcaac380.pcaac362	0.073957327	0.04744241	0.121330367
c102.smohc222	0.02181725	0.013999478	0.121437711
pcaec363.pcaec421	0.005371819	0.00344697	0.121441346
smc260.serotonin	-0.117298456	0.075268233	0.121444164
c182.asparticacid	0.025405207	0.016302572	0.121456029
c8.citruline	-0.008360447	0.005365116	0.121469833
pcaec300.pcaac405	0.060004542	0.038511558	0.121518588
acetylornithoine.pcaac381	0.038724517	0.024855659	0.121546067
pcaec381.citruline	-0.009761173	0.00626647	0.12161554
pcaac281.pcaec340	0.12213636	0.078422191	0.121677684
h1.pcaac323	0.217172393	0.139460504	0.121722459
c102.alphaaminoadipicacid	0.007380826	0.004739766	0.12172694
ornithine.pcaec360	-0.017593568	0.011298847	0.121751055
c102.pcaec403	-0.109576697	0.07038477	0.12181939
pcaec301.citruline	-0.01413571	0.009082313	0.121920143
pcaec322.smohc161	0.027068979	0.017392601	0.121931935
smc260.lysopcac182	-0.020785561	0.013355614	0.12193997
pcaec301.smohc221	-0.04711673	0.030275213	0.121948457
c102.lysopcac280	-0.008237747	0.005293577	0.12197293
pcaec382.pcaec402	-0.29703485	0.190876217	0.121976011
smohc222.c141oh	-0.152651434	0.09810324	0.122008424

lysopcac140.c5	0.01928814	0.012396654	0.122035399
c142.pcaec421	0.003647152	0.002344105	0.122043054
pcaac300.pcaec443	-0.05220994	0.033558129	0.122061319
c51dc.pcaec383	0.109877422	0.07062818	0.122082632
pcaac386.c51	0.129675647	0.083365996	0.122134588
c51dc.pcaec300	0.009597348	0.006170742	0.122182011
pcaac401.pcaac366	0.072123481	0.046375677	0.122205403
pcaac380.alphaaminoadipicacid	0.023865266	0.015347626	0.122257692
pcaac386.asymmetricdimethylarg	0.026453833	0.01702042	0.122434042
lysopcac140.pcaec380	-0.083517767	0.053746251	0.122508843
pcaec340.smohc161	-0.0437474	0.028154734	0.122534359
smc241.pcaac365	0.094868492	0.061057183	0.122547658
pcaac386.histidine	-0.077828999	0.050093944	0.122572423
pcaec445.lysopcac203	-0.019999798	0.012872789	0.1225752
lysopcac170.pcaac426	-0.12263058	0.078931817	0.122580564
c14.pcaac362	0.076790463	0.049435156	0.122644868
lysopcac140.pcaec423	-0.017249035	0.011105441	0.122681056
pcaec300.pcaec340	-0.226157264	0.145616906	0.122706903
c4.pcaec423	0.024302171	0.015647968	0.122716836
lysopcac203.lysopcac170	-0.057393009	0.036969266	0.122861188
pcaec322.c14	-0.038480864	0.024787425	0.122865257
pcaac386.smc240	-0.048838774	0.031459627	0.122867154
arginine.pcaec382	0.024393591	0.015715827	0.122929281
pcaec423.citruline	-0.012117912	0.007807226	0.122935673
pcaac383.alphaaminoadipicacid	0.007785842	0.00501635	0.122947023
lysopcac161.alphaaminoadipicac	-0.00996757	0.006423075	0.123008122
acetylornithoine.pcaec382	-0.017194189	0.011081204	0.123052209
pcaec362.c16	0.079309908	0.051113496	0.123054679
c8.pcaec382	-0.019310725	0.012445615	0.123062919
alanine.pcaec300	0.02741493	0.017670936	0.123109664
pcaec341.smohc161	-0.044185116	0.028491772	0.123256015
smc160.c101	-0.014262164	0.009196828	0.12326388
lysine.pcaec300	0.039098717	0.025217115	0.12333257
acetylornithoine.pcaec300	-0.028499097	0.018383583	0.12338924
c18.pcaec365	0.04439049	0.028636041	0.123409765
lysopcac180.pcaac281	0.004969802	0.003206401	0.123457455
pcaec405.pcaec342	0.160139246	0.10332538	0.123483593
pcaec322.pcaec362	0.074190231	0.047872734	0.123511244
lysopcac140.citruline	0.00900355	0.005809728	0.123511697
pcaec404.citruline	-0.008063099	0.005203678	0.123568563
c51.pcaec300	0.027325972	0.017635853	0.123579214
pcaec362.smohc221	0.037044591	0.02390905	0.123593514
pcaec383.citruline	-0.007853326	0.005069598	0.123664115

pcaec403.citruline	-0.010097727	0.006518802	0.123684905
pcaac343.lysopcac280	0.022630205	0.014610787	0.123720043
pcaac406.smc240	-0.046576921	0.03007481	0.123760337
pcaac421.alphaaminoadipicacid	-0.009914449	0.006402173	0.123783219
c182.citruline	0.011710391	0.007562887	0.123832441
ornithine.lysopcac203	0.026497884	0.017114837	0.123871371
smohc141.pcaec342	-0.23446747	0.151452316	0.123898607
leucine.pcaec340	-0.06016936	0.038867086	0.123909986
pcaec384.alphaaminoadipicacid	0.00549375	0.003548798	0.12391503
pcaec382.pcaec406	0.086688185	0.056004241	0.123956683
pcaec382.citruline	-0.008997235	0.005812689	0.123962701
kynurenine.pcaec443	-0.077225544	0.049891869	0.123963369
putrescine.creatinine	-0.028094888	0.018152106	0.123989894
pcaec364.pcaec385	0.229558858	0.148318087	0.123990199
c10.pcaec424	-0.022852569	0.014765598	0.124003888
prolinr.valine	0.112142713	0.072469309	0.124061445
pcaec446.citruline	-0.006565054	0.004242656	0.124075614
c141oh.pcaec381	-0.030272827	0.019567994	0.12415613
smc181.valine	-0.19695003	0.127316917	0.124187671
kynurenine.pcaac405	0.060756464	0.039278104	0.124211688
smc181.pcaec362	0.033458121	0.021632976	0.124260385
pcaac426.asymmetricdimethylarg	0.034024374	0.02199947	0.124266711
c8.pcaac406	0.079374956	0.051330347	0.124325675
lysopcac161.lysopcac280	0.015724272	0.010169293	0.124351035
pcaac322.pcaac321	-0.098021579	0.063394444	0.124359016
creatinine.pcaac420	0.165428027	0.107004725	0.124414338
pcaac323.pcaec423	-0.010352045	0.006698822	0.124567758
pcaac422.lysopcac161	0.112810147	0.073008202	0.124611788
smc241.putrescine	-0.057450032	0.037183066	0.124638832
lysopcac170.smohc221	-0.074126642	0.047978165	0.124651205
pcaac365.lysopcac170	0.017596003	0.011389621	0.124673524
pcaec423.pcaac363	-0.051233727	0.033166753	0.124718063
lysopcac160.pcaec404	0.032448707	0.021007899	0.124750948
glutamine.pcaac366	-0.028627386	0.01853494	0.124771847
prolinr.pcaec385	0.081971319	0.053077134	0.124802868
lysopcac180.c142	-0.066584987	0.043114686	0.124805765
c10.spermidine	0.105248993	0.068155951	0.124837551
pcaec322.c141oh	-0.163497824	0.105916257	0.124979482
pcaec443.citruline	-0.015619939	0.010120277	0.125033241
lysopcac140.pcaac362	0.025431814	0.016478616	0.125059381
methionine.citruline	0.009304358	0.006028863	0.125064244
smc261.pcaac320	0.063653895	0.041255835	0.125160208
asparticacid.alanine	-0.057465232	0.037247228	0.125184688

pcaac383.pcaac366	0.094559652	0.061296989	0.125223037
pcaac381.pcaec421	0.003869631	0.002508441	0.125223941
pcaec443.pcaec383	-0.277955507	0.180212405	0.125288671
pcaac362.pcaac281	0.003189692	0.002068172	0.125313256
pcaec404.pcaac366	0.035756943	0.023186565	0.125346138
lysopcac180.lysopcac170	0.018131813	0.011757573	0.125346489
lysopcac170.pcaac362	-0.030340495	0.019674328	0.125347189
c8.pcaec385	-0.05149121	0.033391276	0.125366739
c10.citruline	-0.009251077	0.006000297	0.125436186
leucine.pcaac405	0.135275368	0.087742207	0.125444303
lysopcac181.pcaec380	-0.067398936	0.043719406	0.125471388
pcaac381.pcaac425	-0.225158031	0.146057692	0.125484998
pcaac386.alphaaminoadipicacid	0.004593611	0.002980093	0.125518308
c16.pcaac362	0.014727251	0.009554378	0.125522733
pcaac403.pcaac405	-0.090646228	0.058818328	0.125593704
lysopcac182.lysine	0.107473549	0.069741756	0.125618178
lysopcac204.lysopcac181	-0.11545473	0.074927273	0.125650046
c0.ornithine	-0.018237724	0.011835991	0.125655029
smc240.lysopcac203	-0.019762966	0.012826751	0.125681423
glutamine.ornithine	0.044313946	0.02876153	0.1256875
pcaac420.pcaac422	-0.087103752	0.056539147	0.125722752
h1.pcaac366	-0.041636382	0.027033319	0.125821222
smc260.alphaaminoadipicacid	-0.003726243	0.00241943	0.125834548
pcaac343.pcaac403	-0.029307909	0.019030189	0.125848766
c51dc.pcaac320	-0.074076922	0.048101071	0.125860483
smohc141.c51	0.055866387	0.036278679	0.125885595
pcaac323.pcaac420	-0.12519551	0.081300934	0.125890693
c141.pcaec385	-0.092366169	0.05998285	0.125897075
lysopcac170.c5	-0.02905716	0.018870755	0.125916038
c0.pcaec300	0.011952554	0.0077646	0.126021726
pcaec406.pcaac366	-0.043638943	0.028351548	0.126059935
c51dc.pcaec423	0.038479024	0.025002404	0.126107643
pcaec422.pcaec443	-0.059107604	0.038406746	0.126113208
smc202.smohc221	0.020654921	0.013422458	0.126151562
pcaac361.pcaec405	-0.092474411	0.060096552	0.126168412
c142.citruline	-0.015146648	0.009844824	0.126223306
pcaac422.alphaaminoadipicacid	-0.007996043	0.005197966	0.126281186
pcaac322.citruline	0.009311359	0.006054151	0.126352079
asymmetricdimethylarginine.pca	0.093657277	0.060901258	0.126390609
pcaec360.c2	-0.104369802	0.067874293	0.1264302
pcaec446.pcaac405	-0.095838956	0.062367571	0.1266782
lysopcac204.smohc222	0.005609884	0.003650756	0.126688786
asymmetricdimethylarginine.pca	-0.059477762	0.03871216	0.126744102

pcaec383.lysopcac280	-0.018055648	0.011752154	0.126754169
lysopcac181.pcaec342	0.157999172	0.102852365	0.126801911
pcaac426.pcaac341	0.013340511	0.008685025	0.12683585
c0.pcaac342	-0.05484468	0.035711482	0.12690068
alanine.alphaaminoadipicacid	0.01307664	0.008516209	0.126967297
asymmetricdimethylarginine.pca	-0.00972688	0.006335182	0.126998024
pcaac381.citruline	-0.010974996	0.007148746	0.127032657
prolinr.creatinine	-0.018344045	0.011951021	0.127105898
c141.lysopcac182	-0.043264618	0.028188483	0.127131015
histidine.pcaec300	-0.024814204	0.016170458	0.127203184
pcaec340.pcaec443	-0.050864141	0.033147262	0.127215325
alanine.c181	0.017567804	0.011448972	0.127226695
pcaec342.pcaac360	0.076542425	0.049892235	0.127297653
serotonin.tyrosine	-0.115837429	0.075510053	0.127319471
pcaec363.pcaec404	-0.015577896	0.010154946	0.127330711
pcaec384.pcaac385	-0.049847505	0.032503145	0.127429014
lysopcac181.pcaac362	0.028669499	0.01869473	0.127443912
methionine.pcaec362	-0.023150423	0.015098572	0.127511573
c0.kynurenine	0.025047907	0.016339798	0.127597021
pcaac405.alphaaminoadipicacid	0.003176053	0.002072351	0.127684297
pcaac385.alphaaminoadipicacid	0.002309295	0.001507062	0.127750403
c141.c101	-0.026528466	0.017313774	0.127774752
pcaec383.pcaec424	-0.037454212	0.024447362	0.127819766
smohc221.c162	0.149060753	0.097321138	0.127917704
pcaec383.pcaec340	-0.093614896	0.061122723	0.127929776
smc241.alphaaminoadipicacid	-0.006225059	0.004064515	0.127936351
pcaec421.pcaec445	0.191770348	0.125234373	0.12800321
pcaec340.pcaac366	0.051106274	0.033389279	0.128169333
pcaac281.c51	0.062334794	0.040725573	0.128172683
asymmetricdimethylarginine.lys	0.021927991	0.014326737	0.128182942
c141oh.pcaec380	-0.045682984	0.029847456	0.128186781
pcaac384.c18	-0.067629763	0.044186723	0.128187713
pcaac363.pcaec404	-0.016896825	0.011040946	0.128228906
pcaac403.pcaac281	-0.012491007	0.008162405	0.128245895
pcaac385.kynurenine	-0.010795897	0.007055034	0.128263051
asparagine.pcaac426	-0.041873523	0.027368066	0.128319025
pcaac344.smc261	-0.067588201	0.04417906	0.12835475
pcaac240.pcaec301	0.167835786	0.109721416	0.128408152
lysine.smohc161	-0.017662424	0.011547402	0.128431807
pcaac240.smc261	-0.128330029	0.083900375	0.128433239
c5.lysopcac203	0.182191865	0.119116873	0.128440821
creatinine.pcaec404	-0.048114934	0.031474398	0.128644276
pcaac381.pcaac362	-0.046879866	0.030674037	0.128737694

c51.smohc222	-0.006782306	0.004437938	0.128754552
pcaec402.smohc221	-0.093164247	0.0609633	0.128767913
pcaec321.spermidine	-0.104695404	0.068520191	0.128830568
pcaac343.pcaac384	0.131818557	0.086280392	0.12886952
pcaec341.c18	0.047327516	0.030980112	0.128898972
c10.lysine	0.107669571	0.070480406	0.128904334
c5.citruline	0.011114354	0.007275681	0.128916576
pcaec300.c2	-0.096242593	0.06300334	0.12892254
glutamicacid.c181	0.0415248	0.027183881	0.128929214
pcaec402.pcaac320	0.165023145	0.10804284	0.128970476
pcaec383.pcaec406	0.090621154	0.059334754	0.12899531
c51.lysopcac203	0.016758208	0.01097416	0.129051355
c181.pcaec382	-0.015870543	0.010393013	0.12905656
pcaac406.pcaac402	0.113733977	0.074481326	0.129063132
pcaec384.smc241	0.189815051	0.124309935	0.12907888
pcaec386.histidine	-0.099135074	0.064925651	0.129090929
pcaac385.lysopcac203	-0.017634877	0.011550974	0.129140927
creatinine.alphaaminoadipicacid	0.003662775	0.002399395	0.129180632
pcaac420.alphaaminoadipicacid	-0.005816694	0.003810424	0.129185487
pcaec384.c8	-0.081247744	0.053227147	0.129207107
pcaac420.c51	0.11784716	0.077227201	0.129320171
alphaaminoadipicacid.smohc221	0.044138735	0.02893045	0.129393765
c14.alphaaminoadipicacid	-0.004655344	0.00305226	0.129511393
pcaac300.c182	-0.074118096	0.048603457	0.12957546
pcaec300.lysopcac204	-0.031969975	0.020965035	0.129584563
c18.pcaac421	-0.099240801	0.065125511	0.129853962
pcaec406.glutamicacid	0.081347597	0.053384512	0.129862437
pcaac300.prolinr	-0.050453575	0.033114179	0.129907564
glutamine.pcaec423	-0.019518775	0.012811057	0.129916662
pcaac384.alphaaminoadipicacid	0.002853888	0.0018735	0.129990648
serine.alphaaminoadipicacid	-0.001584845	0.001040566	0.130048388
pcaec385.alphaaminoadipicacid	0.003364606	0.00220916	0.130057395
lysopcac160.c182	-0.057023609	0.037441952	0.130066888
smohc222.alphaaminoadipicacid	-0.031993773	0.021007443	0.130070348
citruline.pcaec361	-0.017266975	0.011338437	0.130095967
c5.c2	0.209747535	0.137748958	0.13014378
smc261.alphaaminoadipicacid	-0.008144561	0.005348871	0.130146373
c12.citruline	-0.016180441	0.010626846	0.13016358
c141oh.pcaec385	-0.069668997	0.045763597	0.130221977
lysopcac182.smohc161	0.017768097	0.011671882	0.130238986
histidine.pcaac281	-0.00579371	0.003806157	0.130265227
c141oh.lysopcac203	-0.023393469	0.01536938	0.130293156
serotonin.pcaec404	0.033069595	0.021734581	0.130434221

pcaec420.pcaac361	0.087049295	0.057216013	0.1304605
pcaac362.c141oh	-0.163946465	0.10780767	0.130632009
pcaec445.pcaec383	-0.066354497	0.043637092	0.130665252
pcaac424.citruline	-0.011772159	0.007743734	0.130760911
pcaac343.pcaac281	0.006812988	0.00448224	0.130816419
pcaec403.alanine	-0.039025653	0.025675457	0.130825715
pcaac385.smc161	-0.079268369	0.052157628	0.130869594
lysopcac182.pcaec421	-0.004598302	0.003025707	0.130879763
pcaac383.lysopcac203	0.027445035	0.018060997	0.130922373
pcaec422.pcaac402	-0.169849396	0.111780782	0.130944546
pcaac420.c14	-0.07578906	0.049884473	0.13099357
methionine.alphaaminoadipicacic	0.005203573	0.003425751	0.131077393
c3.alphaaminoadipicacid	0.004918866	0.003238325	0.131078525
pcaac323.alphaaminoadipicacid	0.008672934	0.005711304	0.131178667
pcaac402.citruline	-0.016061897	0.010577906	0.131208274
pcaec445.tyrosine	-0.064721096	0.042647005	0.131419467
smohc161.pcaac366	-0.068171873	0.044924166	0.131447761
pcaec403.c12	0.15805292	0.104160706	0.131471195
asymmetricdimethylarginine.citrı	0.014594444	0.009618334	0.131480813
c102.asparticacid	-0.074427878	0.049054479	0.131507886
pcaac404.pcaac323	-0.219145435	0.144441674	0.131522913
phenylalanine.pcaec300	0.022272564	0.014681029	0.131546015
lysopcac160.asparticacid	0.030692288	0.020231348	0.131554183
pcaec444.c181	-0.013056767	0.008607617	0.131599759
pcaec423.asparticacid	0.054643587	0.036028729	0.131654827
pcaec365.pcaac366	0.074736349	0.049282166	0.131697297
pcaac366.pcaec301	-0.18675134	0.123178772	0.131798038
pcaec420.pcaac362	-0.023376762	0.015420753	0.13184135
pcaec363.c5	0.027242099	0.017970586	0.131841723
smc260.pcaec300	-0.022958011	0.015151052	0.132006596
pcaec443.pcaec424	-0.068481984	0.045205112	0.132097264
lysopcac170.c141oh	-0.14295415	0.09436964	0.132118738
pcaec425.pcaac361	0.093505339	0.061727244	0.132123101
c162.c2	0.076275277	0.050385579	0.132372198
pcaac344.smohc221	0.082019354	0.054186802	0.132420558
pcaec321.c14	-0.055209053	0.036478156	0.132460687
leucine.pcaac344	-0.111308653	0.073553262	0.132505276
smc161.prolinr	0.028815881	0.01904186	0.132509205
smc202.c181	0.023365662	0.015445918	0.132649079
c51dc.pcaac322	-0.115357377	0.076266848	0.13269756
pcaec420.alphaaminoadipicacid	0.00729745	0.004824722	0.132707046
pcaec444.serotonin	-0.14114766	0.093324213	0.1327243
c181.asparticacid	0.028530452	0.018866077	0.132770581

smohc222.lysopcac180	0.106064663	0.070146599	0.132826288
smc260.pcaec343	-0.106017493	0.070163961	0.133092509
pcaec382.pcaec401	0.161889841	0.107143674	0.133101695
pcaac401.citruline	-0.011062844	0.007325581	0.133304031
pcaac383.c18	0.053725968	0.03557702	0.133312936
c8.pcaec404	0.036458752	0.024143544	0.133325261
pcaec423.c181	-0.020996752	0.013904633	0.133332527
pcaac361.lysopcac170	0.012541358	0.008305412	0.133340682
h1.pcaec361	0.043405452	0.028745632	0.13335041
smohc222.pcaec362	0.030570239	0.020247615	0.133392481
c182.kynurenine	-0.024521965	0.016246687	0.133511773
smohc241.c16	0.105060078	0.069616443	0.13356905
pcaec405.lysopcac161	0.093771295	0.062142548	0.13360901
pcaec446.c51	0.081321668	0.053894305	0.1336245
pcaac363.pcaac366	-0.055473656	0.03678167	0.133809173
pcaac404.prolinr	0.089599773	0.059432755	0.133963979
pcaac281.c2	-0.102335268	0.067881206	0.133968656
pcaac401.pcaec300	-0.060869597	0.040377791	0.133984564
pcaec321.c181	-0.010633149	0.007054903	0.134061853
lysopcac203.putrescine	-0.070945922	0.047080063	0.134133389
c102.citruline	0.082690783	0.054876611	0.134151679
pcaec444.alphaaminoadipicacid	-0.003155809	0.002094333	0.13415606
pcaac341.citruline	0.01483095	0.009844665	0.134242168
c12.asparticacid	0.077372857	0.051360277	0.134248178
putrescine.pcaec364	0.051659708	0.034295867	0.13429364
smc180.smohc241	0.155483034	0.103236855	0.134348706
smc261.glutamine	-0.021536715	0.014303899	0.134458312
pcaec423.glutamicacid	0.098043885	0.065121651	0.134484942
c181.arginine	0.068320079	0.045380254	0.134497107
pcaac401.pcaec342	0.168588019	0.111982557	0.134501654
pcaec425.pcaac344	0.069186559	0.045958353	0.134518611
asparagine.pcaec361	0.010410273	0.00691536	0.134527463
c141.pcaac426	-0.078519752	0.052162783	0.134553416
pcaac361.pcaec443	-0.035029014	0.023273015	0.134591614
pcaac344.c12	0.086487707	0.057473752	0.13467211
c142.c181	-0.035767205	0.023769059	0.134682625
c16.pcaec300	-0.037350496	0.02482132	0.134683989
pcaac281.alphaaminoadipicacid	-0.00662931	0.004406663	0.134784665
smc240.alphaaminoadipicacid	-0.006812097	0.00452852	0.134814888
pcaec445.citruline	-0.012607162	0.008382039	0.134865571
c0.pcaec402	-0.058805895	0.039103284	0.134919027
pcaec364.c51dc	-0.16774149	0.111541421	0.134921973
c102.pcaac363	0.026213334	0.017431568	0.134938475

pcaac384.lysine	0.116124239	0.077242191	0.135043211
prolinr.pcaec402	-0.047867617	0.031843884	0.135090031
pcaac406.pcaec383	0.15166428	0.100921361	0.13519309
methionine.pcaec405	-0.082493202	0.054899521	0.13523823
acetylornithoine.alphaaminoadip	-0.006399277	0.004260061	0.135358221
c141oh.pcaec402	-0.057248793	0.03812424	0.135492076
pcaac420.citruline	-0.013715571	0.009133807	0.135494816
c18.asparticacid	0.026687818	0.017774127	0.135528114
pcaac385.pcaac422	-0.047027082	0.031320467	0.13553255
c182.smohc241	-0.120536608	0.080289384	0.135585204
leucine.lysopcac180	0.085320876	0.056834289	0.135599456
pcaec443.pcaac361	0.073724518	0.049109966	0.135601747
pcaac364.lysopcac280	0.012771804	0.008508629	0.135646107
kynurenine.c2	0.1063129	0.070828864	0.135661251
smc181.smc160	-0.180013114	0.119936933	0.13568312
pcaec423.lysopcac182	-0.147803665	0.098484174	0.135712107
pcaec322.lysopcac181	0.086800506	0.057849793	0.135799942
c102.pcaec300	0.011910215	0.007938104	0.135815684
pcaac363.pcaec445	0.10171546	0.067796083	0.135833977
pcaec364.pcaac366	0.063127856	0.042077922	0.135847923
pcaac341.pcaec382	0.017809779	0.011872208	0.135883428
pcaec424.pcaac405	-0.081462528	0.054309354	0.135922573
pcaac322.smohc221	0.060221338	0.040148834	0.135927745
asymmetricdimethylarginine.pca	-0.066840796	0.044562841	0.135935532
pcaac362.pcaec443	-0.04864162	0.032431409	0.135959247
glycine.pcaec404	0.051638939	0.034434787	0.136014961
pcaac421.lysopcac204	0.074175241	0.049466483	0.136043557
pcaac344.c142	0.028753949	0.019177215	0.136075734
citruline.alphaaminoadipicacid	0.002989324	0.00199386	0.136105709
lysopcac160.smohc221	-0.061083851	0.040742743	0.136107768
pcaac421.citruline	-0.011424985	0.007621032	0.136138457
pcaec363.pcaac381	0.05173302	0.034511617	0.136173629
kynurenine.pcaac341	0.014846984	0.009904801	0.136182611
pcaac422.citruline	-0.015647055	0.010440122	0.136241181
lysopcac140.alphaaminoadipicac	-0.004395019	0.002932737	0.136276409
pcaac344.pcaac403	-0.021288237	0.014207948	0.136347405
pcaec321.kynurenine	-0.016041799	0.010706586	0.136353021
serotonin.smc160	0.144146699	0.09622055	0.136411385
pcaec401.pcaec362	0.028791031	0.01921969	0.136434691
c142.c14	-0.081576284	0.054464951	0.136492037
pcaac361.c101	-0.025984232	0.017348747	0.136496549
smohc241.alphaaminoadipicacid	-0.004036159	0.002694896	0.136510412
pcaec364.pcaac405	-0.069643906	0.046509929	0.136589984

pcaec362.pcaec361	-0.017184291	0.011479619	0.136709412
pcaec364.pcaac362	0.044930215	0.030018403	0.13675713
pcaac384.smc161	-0.074088749	0.049500116	0.136761494
pcaec404.pcaec401	0.13424621	0.089721605	0.136887939
c3.lysopcac170	-0.044932993	0.030031331	0.136900699
pcaec425.alphaaminoadipicacid	-0.004144266	0.002770019	0.136923934
c141oh.c5	-0.013280191	0.00887665	0.136932552
c5.alphaaminoadipicacid	-0.015006364	0.010032633	0.137017481
pcaec361.pcaac281	-0.004682043	0.003130481	0.13705
c18.pcaec385	-0.095907928	0.064138918	0.137132036
pcaec301.tyrosine	0.069300546	0.046353397	0.137201856
pcaec444.pcaac361	0.056462746	0.037767922	0.137216375
c8.pcaac362	-0.0105283	0.007044822	0.137351661
smc261.pcaec381	-0.069456324	0.0464815	0.137402451
methioninesulfoxide.alphaamino	-0.001603098	0.001072893	0.13742786
c51dc.c12	-0.064612577	0.043248214	0.137476796
pcaac321.citruline	0.010933755	0.007318814	0.137495001
c10.pcaac320	-0.084296057	0.056445786	0.137632387
asparticacid.pcaec404	-0.05249969	0.035155994	0.1376489
putrescine.pcaac341	-0.017286461	0.011576183	0.137663858
pcaac401.smohc222	0.042695013	0.02859393	0.137697484
c8.alphaaminoadipicacid	-0.00163743	0.001096633	0.137699224
pcaec402.alphaaminoadipicacid	-0.002323149	0.001556028	0.137736659
pcaac425.pcaac362	0.031833131	0.021323803	0.137777159
pcaec443.pcaac405	-0.086305258	0.05782104	0.137834105
asparticacid.c18	0.077203146	0.051727538	0.137868437
pcaec321.c141oh	-0.17039126	0.11418188	0.137925162
pcaec406.glycine	0.084143258	0.056403106	0.138045567
smc181.prolinr	0.03028357	0.020300211	0.138054365
pcaec423.alphaaminoadipicacid	-0.005891224	0.003949658	0.138108762
pcaec364.kynurenine	-0.024810418	0.016640109	0.138260169
leucine.pcaec443	0.078033704	0.052350576	0.138365684
pcaec343.c16	0.102300574	0.068636118	0.138397571
arginine.pcaec360	0.013803544	0.009261817	0.138425433
pcaec401.asparagine	-0.018269254	0.012260625	0.138503132
smohc222.pcaac362	-0.010411914	0.006987533	0.138504459
pcaac362.c5	0.020801553	0.013960238	0.138507854
pcaac364.c181	-0.010161909	0.006820831	0.138566408
c16.glutamine	0.03692881	0.024788103	0.138580802
pcaec343.pcaec404	-0.014554602	0.009770287	0.138606974
lysopcac203.lysopcac280	0.01301244	0.00873531	0.13861831
c101.pcaec300	0.019160586	0.012863141	0.138635127
lysopcac280.c51dc	0.187164834	0.125653714	0.138646701

c162.c12	0.119756214	0.080403628	0.138670605
alphaaminoadipicacid.pcaac381	0.04182366	0.028082212	0.138699174
pcaec406.pcaec421	0.003277083	0.002200702	0.138757334
pcaec341.lysopcac161	0.102293757	0.068698569	0.138779697
smc260.pcaec385	-0.056452436	0.03791567	0.138813506
c141oh.smohc241	-0.085097319	0.057160929	0.138856225
pcaec443.c12	0.110430268	0.074191285	0.138929639
c10.alphaaminoadipicacid	-0.002637181	0.001771765	0.138930989
phenylalanine.kynurenine	-0.008244087	0.005538769	0.138934989
spermidine.alphaaminoadipicaci	0.033495106	0.022504611	0.138952555
pcaac343.pcaec423	-0.010994235	0.007386956	0.138961869
pcaac401.alphaaminoadipicacid	-0.003087743	0.002074938	0.13901914
pcaac420.c181	-0.02396695	0.016107843	0.139073914
pcaec401.arginine	0.09211956	0.061915666	0.139095649
c4.smc161	0.168767371	0.113447134	0.139146521
pcaec384.asymmetricdimethylar	0.042183966	0.028356552	0.139147403
lysopcac180.lysopcac280	0.011524805	0.007747166	0.139150323
c141.prolinr	-0.051323779	0.034504974	0.139198926
pcaec362.c181	-0.025614531	0.017220856	0.139203663
c16.smc181	0.057201408	0.038457715	0.13921128
smc160.pcaac366	-0.03806074	0.025590354	0.139231441
pcaec406.transhydroxyproline	0.113866703	0.076565768	0.139266502
alphaaminoadipicacid.pcaec423	0.022417852	0.015076027	0.139316187
c141.pcaec443	-0.168640087	0.113415221	0.139332116
glycine.creatinine	-0.023655505	0.015910939	0.139380067
pcaec381.alphaaminoadipicacid	-0.001966598	0.00132278	0.139387767
pcaac365.leucine	-0.015773289	0.010609679	0.139394877
pcaac402.alphaaminoadipicacid	-0.003582229	0.002409809	0.139439377
lysopcac180.pcaac420	-0.105788033	0.071168557	0.139459608
methioninesulfoxide.pcaec365	0.03283755	0.022092625	0.139482178
ornithine.pcaac366	0.024864208	0.016729197	0.139503754
isoleucine.prolinr	-0.076355535	0.05138504	0.13959029
glutamine.pcaec383	-0.07891147	0.053117338	0.139680707
glutamicacid.alphaaminoadipicac	-0.001620995	0.001091452	0.139795378
pcaac386.c141oh	-0.132599349	0.08928578	0.13981164
arginine.pcaac361	0.070188644	0.047262444	0.139819257
pcaac406.arginine	0.067913389	0.045735106	0.139859933
c162.pcaac300	0.075187976	0.050640762	0.139912022
pcaec384.pcaac344	0.122004054	0.082178785	0.13994245
pcaac403.alphaaminoadipicacid	-0.004247573	0.002861075	0.13994517
pcaac344.pcaac361	-0.103396345	0.069652958	0.139986735
pcaec423.lysopcac170	0.021405143	0.014420044	0.139999477
pcaac385.lysopcac280	0.008925789	0.006013232	0.140011171

asparagine.c8	-0.089798157	0.060503929	0.140060767
asparticacid.alphaaminoadipicaci	-0.000896867	0.000604307	0.140073014
pcaec380.glycine	0.073869013	0.049774199	0.140084153
pcaac364.pcaac385	-0.057717357	0.038891453	0.140089363
h1.c14	0.133071356	0.089671596	0.140109815
lysopcac170.asparticacid	0.030000402	0.020223777	0.140259148
c3.c101	0.029158133	0.019660472	0.140348887
pcaec363.pcaec365	0.044621814	0.030094899	0.140449905
pcaac424.alphaaminoadipicacid	-0.002203192	0.001485943	0.140453727
phenylalanine.alphaaminoadipica	-0.002514012	0.001695734	0.140490416
serotonin.c102	0.083476451	0.056313089	0.140540294
pcaec424.alphaaminoadipicacid	-0.004170481	0.002814246	0.140658662
spermidine.c182	-0.292223109	0.197260851	0.140795165
leucine.c18	0.071614797	0.048347471	0.140835684
glycine.pcaec405	0.077637525	0.052415	0.140847403
pcaac425.smohc221	-0.08347314	0.056359399	0.140879801
lysopcac180.asparticacid	0.024393419	0.016470234	0.140886601
prolinr.c101	0.017106548	0.011550839	0.140908439
pcaac384.lysopcac161	-0.110882778	0.074878138	0.140944556
c51dc.pcaec404	-0.020487114	0.013835779	0.140973516
lysopcac280.alphaaminoadipicac	-0.003052236	0.002061448	0.141002131
c0.pcaec443	-0.118775202	0.080223629	0.141022267
c12.lysopcac180	-0.075531123	0.051035354	0.141175458
histidine.pcaec421	-0.004902979	0.003313227	0.141217194
smc161.pcaac384	0.099096444	0.066967951	0.141233379
pcaac365.pcaac366	0.201231451	0.136018413	0.141317805
pcaac366.asymmetricdimethylarg	0.046152063	0.031195951	0.14132259
pcaac381.alphaaminoadipicacid	-0.002892612	0.00195547	0.141371642
pcaec361.lysopcac161	0.09489389	0.064154142	0.141394707
lysine.c14	0.066130723	0.044709779	0.141406348
c51.pcaac362	-0.014680139	0.009925892	0.141442814
pcaec361.lysopcac280	-0.024865011	0.016813028	0.141459101
pcaec322.pcaec340	0.095896137	0.064851149	0.141513006
glycine.glutamine	0.037345292	0.025259919	0.141585344
c14.pcaec405	0.065656088	0.044415667	0.14164455
histidine.leucine	-0.023736778	0.016058422	0.141662676
smc180.pcaac360	0.104722628	0.070848879	0.141672997
asparagine.alphaaminoadipicacid	0.003089323	0.00209023	0.141707946
pcaec444.pcaac281	-0.003631618	0.002457154	0.141709216
isoleucine.pcaac320	-0.199184304	0.134772077	0.141720636
kynurenine.c51	0.097970679	0.066297605	0.141772423
pcaac406.alphaaminoadipicacid	0.005221078	0.003533337	0.141793492
pcaec444.pcaec362	0.026744252	0.018099169	0.141796498

pcaec361.lysopcac182	-0.029255545	0.019800124	0.141825214
c2.lysopcac280	0.023965602	0.016220197	0.141832549
pcaac384.pcaec446	-0.068513826	0.046376911	0.141883362
glutamine.pcaac402	-0.153403933	0.103842066	0.141895405
asparticacid.pcaac365	-0.115018463	0.077863511	0.141922354
pcaac424.c101	0.031834779	0.021554648	0.141988519
pcaec300.arginine	-0.075460397	0.05109702	0.142022577
smohc221.pcaac361	0.068693767	0.046516615	0.142035586
pcaac426.lysopcac170	0.027982625	0.018950349	0.142070178
pcaec342.pcaec365	0.039463134	0.026726758	0.142093828
acetylornithoine.pcaec405	-0.076153337	0.051589307	0.142199547
pcaac365.pcaec300	0.061494834	0.041659616	0.142204904
smohc241.citruline	-0.013475859	0.00913284	0.142362368
pcaac386.lysopcac182	-0.018589815	0.012599865	0.142400125
pcaac320.pcaec444	0.167384448	0.113462636	0.142442941
pcaac421.glutamicacid	0.112621714	0.076343436	0.142453709
pcaec404.c182	-0.057348103	0.038875584	0.142461279
c101.c141oh	0.167853787	0.113788097	0.14246841
pcaec444.pcaec443	-0.04714506	0.03195999	0.142472737
c181.pcaac281	0.015676708	0.010627495	0.142477859
putrescine.pcaec382	0.029614786	0.020076612	0.142483128
c141oh.pcaec300	0.031922047	0.021642232	0.142510024
pcaac342.lysopcac182	-0.026477998	0.017952735	0.142541178
pcaac362.smohc161	0.04234628	0.028714635	0.142579994
serine.c14	0.109492229	0.074247419	0.142589143
pcaac420.tyrosine	-0.092445669	0.062691852	0.142613293
lysopcac203.pcaec340	-0.070440156	0.04777496	0.142663923
c8.pcaac322	0.061312386	0.041590893	0.142727802
pcaac344.pcaac363	0.036538317	0.024787614	0.142760811
pcaac384.pcaec444	0.126888063	0.086085695	0.142782747
smc241.pcaac320	0.076411842	0.051846995	0.142830846
pcaec321.pcaec340	0.138583462	0.094035705	0.142847703
c102.arginine	0.077757859	0.052766692	0.142878919
pcaac403.pcaec404	0.060046955	0.040749545	0.142893712
smc161.pcaac281	0.002834094	0.001923317	0.14289815
c4.c51	0.038704198	0.026267886	0.142925929
pcaec445.pcaac344	0.086899122	0.058985998	0.142986652
c142.pcaec403	0.050521296	0.034307478	0.143151789
asymmetricdimethylarginine.c18	-0.042122334	0.028611828	0.143260409
c141oh.putrescine	-0.041999704	0.028529779	0.143277788
pcaec405.smohc222	-0.016368861	0.01111942	0.143288302
c102.glutamine	0.052815307	0.03587778	0.143290196
pcaec403.alphaaminoadipicacid	-0.002792222	0.001897074	0.143352981

pcaac323.lysopcac281	0.033839178	0.022994213	0.14341179
smohc222.tyrosine	-0.115227308	0.078299856	0.143418006
c4.pcaec385	-0.152757434	0.103826167	0.143508477
lysopcac182.glutamine	0.028835343	0.019600267	0.143538115
pcaec382.alphaaminoadipicacid	-0.002532769	0.001721646	0.143548563
pcaac405.smc240	-0.04556701	0.030975547	0.143567013
pcaec362.lysopcac180	0.135421252	0.092059801	0.143580509
pcaec364.prolinr	0.047922753	0.032581923	0.14362786
pcaec424.lysopcac180	-0.088716334	0.06032149	0.143658527
pcaec403.smc240	-0.091381824	0.062141321	0.143706284
pcaac405.pcaac426	0.094954874	0.064574628	0.14372824
prolinr.pcaac363	-0.048289308	0.032845261	0.14379881
pcaac406.pcaec342	0.16751971	0.113951871	0.143829759
pcaac383.pcaec382	0.009353485	0.006362749	0.143844182
smc161.smohc221	0.024853625	0.016907404	0.14385874
c3.smc260	0.055728467	0.037911295	0.143862699
pcaac320.kynurenine	-0.023750654	0.016160451	0.143941798
asymmetricdimethylarginine.smc	-0.013313487	0.009059385	0.143968342
pcaec341.kynurenine	-0.012596112	0.008572579	0.144030701
c5.pcaec383	0.167043585	0.113712744	0.144126329
kynurenine.creatinine	-0.014915698	0.010153741	0.144129203
pcaec406.smc202	0.07472908	0.050875747	0.144164661
c14.tyrosine	0.08604527	0.058585551	0.144203477
pcaec343.lysopcac180	0.118251339	0.080513906	0.144204676
c4.alphaaminoadipicacid	0.005635238	0.003837233	0.144242396
smc202.pcaec404	-0.010878984	0.007408543	0.144277692
lysopcac203.pcaac323	-0.179032047	0.121946964	0.144365458
pcaac343.c12	0.067002321	0.045642247	0.144399346
pcaec424.pcaec443	-0.081901922	0.055794584	0.144418407
pcaac360.pcaec361	-0.02208708	0.015046969	0.144430111
glutamine.alphaaminoadipicacid	0.001594004	0.001085957	0.14444143
pcaac342.lysopcac203	0.029341568	0.0199936	0.144519039
pcaac381.smc202	0.072880016	0.049661617	0.144523374
pcaec425.pcaec300	0.017411333	0.011864962	0.144543488
c51.lysine	-0.152573299	0.103986597	0.144602677
pcaac365.putrescine	-0.085412976	0.05821714	0.144628541
pcaac343.pcaac322	0.197362611	0.13454558	0.1446997
phenylalanine.pcaec420	0.068847161	0.046937355	0.144725425
pcaac384.pcaec363	-0.047549944	0.032422394	0.144782785
isoleucine.c182	-0.090544163	0.061738628	0.144784114
isoleucine.pcaec443	0.074537446	0.050834278	0.144862745
pcaec422.c18	0.067084158	0.045755068	0.144896798
pcaec405.citruline	-0.013082849	0.00892516	0.144983541

pcaac361.pcaec340	0.115013641	0.078467008	0.145005641
pcaac422.lysopcac204	0.090383233	0.061675922	0.145088363
serotonin.alphaaminoadipicacid	0.016467786	0.011237454	0.145092901
smohc221.smohc222	-0.02340516	0.015972474	0.145118508
smohc222.c51	0.05324447	0.036345769	0.14522766
pcaac406.pcaac341	0.031534491	0.021526367	0.145232782
lysopcac140.smohc141	-0.048524157	0.033126383	0.145261359
smc241.lysopcac203	-0.018424921	0.01257915	0.145288619
c14.c2	0.108254716	0.073927521	0.145393124
pcaec301.valine	0.110148815	0.075229963	0.145440669
c4.kynurenine	-0.022105666	0.015098403	0.14545575
pcaac385.pcaec404	-0.014214512	0.009708668	0.14545603
leucine.creatinine	-0.018591291	0.012698874	0.145481744
histidine.pcaec340	-0.057849907	0.039518741	0.145522927
pcaac363.c16	0.066444757	0.045404014	0.145645394
lysopcac161.pcaac281	0.004516889	0.003086554	0.145646333
pcaec446.lysopcac180	-0.073739295	0.050390989	0.145664134
pcaec380.asymmetricdimethylarg	0.051432859	0.035153221	0.145729084
lysopcac281.lysopcac280	0.022247871	0.01520849	0.145796449
smohc222.smc240	0.120978589	0.082707024	0.145829814
pcaec424.pcaec405	0.191080891	0.130633228	0.145832136
histidine.lysopcac160	0.058772633	0.040181269	0.145843278
lysopcac280.c162	-0.119897709	0.081993749	0.145955038
asparagine.pcaec445	-0.16746775	0.114532979	0.145982226
pcaec340.asparagine	0.010256014	0.007014453	0.145996788
pcaec405.pcaac403	-0.031340234	0.021435753	0.146016441
pcaac425.alphaaminoadipicacid	-0.004590064	0.003139542	0.146026611
citruline.pcaac341	-0.009952589	0.006807518	0.146031403
pcaac383.lysopcac280	0.009984759	0.006830172	0.14606948
lysopcac140.pcaec403	-0.128742019	0.08809493	0.146195287
smc261.pcaec322	-0.138891926	0.095041536	0.146200673
kynurenine.pcaac320	-0.084562575	0.057868312	0.1462248
pcaec361.pcaec443	-0.081923451	0.056069028	0.146272889
pcaec443.alphaaminoadipicacid	-0.002814112	0.001926068	0.146287182
pcaac422.pcaec362	0.018743216	0.012833431	0.146442457
smc202.pcaec381	-0.030762879	0.021063809	0.146452837
pcaec360.smc202	0.071347821	0.04885481	0.146468229
pcaac341.c8	-0.065925034	0.045143994	0.14648945
pcaec361.alphaaminoadipicacid	-0.002811847	0.001925593	0.146510762
pcaec401.lysopcac170	0.015753637	0.010789411	0.146551489
pcaec402.pcaec404	0.03736944	0.025594791	0.14656822
pcaec444.c182	-0.06589762	0.045135904	0.146584287
smc181.c14	-0.040732375	0.027902316	0.146628668

pcaec383.pcaec360	-0.012545655	0.008594253	0.146641832
pcaac360.histidine	-0.104082529	0.071301835	0.1466493
acetylornithoine.glutamine	-0.031440742	0.021540532	0.146686922
pcaac384.spermidine	-0.101639832	0.069635536	0.146690015
lysopcac281.c12	0.086551313	0.059345485	0.147010465
pcaec321.asparagine	0.00945679	0.006484705	0.14704045
pcaec383.alphaaminoadipicacid	-0.003238414	0.002222145	0.14731169
c2.alphaaminoadipicacid	0.002434639	0.001670758	0.147347679
pcaac360.pcaec402	0.099743568	0.068449821	0.147355571
pcaec444.valine	0.092565099	0.063527121	0.147378211
asparticacid.pcaec382	-0.021107434	0.014487624	0.147424282
pcaec404.alanine	-0.030450459	0.020902418	0.147462174
lysopcac140.c12	-0.1199855	0.082364525	0.147470262
pcaac363.prolinr	0.033429921	0.022950667	0.14751515
c181.c51	0.089682919	0.061593568	0.147668441
asparagine.smohc161	-0.021462713	0.014741068	0.14768574
lysopcac281.pcaec443	-0.075148325	0.051621151	0.147745031
pcaec380.pcaec423	-0.022345091	0.015352958	0.147839044
c14.pcaec402	-0.061482318	0.042246603	0.147868335
c2.pcaec322	0.097744961	0.067174139	0.14792961
pcaac385.arginine	0.050551183	0.034740756	0.147929807
h1.c5	-0.031323886	0.021530834	0.148001378
pcaec421.pcaac422	-0.296655342	0.203938493	0.148058645
c141oh.pcaec406	-0.014756968	0.010146513	0.148125934
pcaac365.kynurenine	-0.017960423	0.01234931	0.148131953
pcaac380.pcaac361	0.125649293	0.086465746	0.148463743
c8.lysine	0.10664539	0.073389005	0.148468259
pcaec382.pcaec421	-0.008773386	0.006038433	0.148531379
pcaec401.asymmetricdimethylar;	0.050990534	0.035104347	0.148637497
pcaac406.pcaec443	-0.052330309	0.036032539	0.148702667
lysopcac182.asymmetricdimethy	0.032970322	0.022707251	0.148795304
pcaec321.pcaec361	-0.012883953	0.008874703	0.148854103
pcaec360.pcaec421	0.002798211	0.001927777	0.148920644
pcaac342.asymmetricdimethylar;	0.039918624	0.027502639	0.14894149
pcaac362.lysopcac182	-0.019624428	0.013522406	0.148995496
pcaec425.pcaac381	0.036390281	0.025077289	0.149030993
pcaac240.alphaaminoadipicacid	-0.004781566	0.00329512	0.149036572
pcaec341.smohc241	0.14807681	0.102062813	0.149110247
pcaac404.putrescine	-0.086567363	0.059676572	0.149174547
pcaec443.lysopcac161	0.109648052	0.075592592	0.149201157
pcaec343.prolinr	0.029885212	0.020603522	0.149207471
pcaac384.lysopcac182	-0.051410099	0.035448341	0.149265426
pcaec404.pcaac405	-0.089216283	0.061524677	0.14931911

lysopcac140.pcaec301	-0.085076579	0.058675262	0.149356109
ornithine.smohc141	-0.041332293	0.028506414	0.149363605
pcaec403.pcaec424	-0.035714768	0.024638508	0.149469127
prolinr.ornithine	-0.060301668	0.041607799	0.149542466
creatinine.c5	0.026454793	0.018254572	0.149562817
lysopcac140.c101	-0.017873102	0.012337032	0.149696296
lysopcac180.pcaec380	-0.062462708	0.043119876	0.149739049
pcaac385.smc202	0.049837885	0.034409743	0.149799666
pcaec380.pcaac385	-0.082093064	0.056689178	0.149866575
pcaac384.serine	-0.071190544	0.049161919	0.149878513
alphaaminoadipicacid.lysopcac2(	0.042788257	0.029549324	0.14989382
arginine.pcaec424	-0.020713746	0.014309559	0.150028538
pcaec341.pcaac402	-0.075223401	0.051976357	0.150107942
isoleucine.pcaec362	-0.015362621	0.010617777	0.150215497
h1.alphaaminoadipicacid	-0.005848072	0.004042106	0.150240352
c181.pcaec404	-0.022630382	0.015641994	0.150245244
lysine.pcaac361	0.075657263	0.052298642	0.150282118
pcaac300.pcaac426	0.113027436	0.07814467	0.150352675
c10.pcaac362	-0.011853287	0.00819521	0.150358099
pcaec343.c142	0.051411488	0.035556115	0.15048194
acetylornithoine.c3	0.063570566	0.043979736	0.150614761
pcaec363.pcaec340	0.125086512	0.086539926	0.150623623
pcaec444.lysopcac181	-0.092814441	0.06421752	0.150653421
pcaec342.pcaec404	-0.01502873	0.010399007	0.1506829
pcaac421.lysopcac161	0.128757303	0.089095584	0.150696652
pcaec404.pcaac365	-0.08766081	0.060662157	0.150722834
histidine.pcaac403	-0.010247391	0.007091521	0.1507356
lysopcac170.lysine	0.113186265	0.078342732	0.150809263
c101.alphaaminoadipicacid	0.016309316	0.01129298	0.150965927
lysopcac182.serotonin	-0.161911816	0.112128117	0.151024926
pcaac384.arginine	0.04622516	0.032013567	0.151043291
citruline.lysopcac280	-0.016947085	0.011738556	0.151103108
asparagine.pcaac366	-0.039056706	0.027054375	0.151123848
c181.putrescine	-0.059444369	0.041180772	0.151162952
smc261.serotonin	-0.095039658	0.065842871	0.151181765
lysopcac280.lysopcac170	-0.066618627	0.046162229	0.151263232
lysopcac181.c3	-0.115026736	0.079708443	0.151276727
pcaec382.ornithine	-0.058729296	0.040712013	0.15142838
acetylornithoine.smc181	0.047917355	0.033219395	0.151457452
c162.asparagine	-0.011393684	0.007899279	0.151480294
serine.c51dc	0.179389976	0.124398864	0.151569065
lysopcac161.citruline	0.012933297	0.00897081	0.151666511
lysopcac180.pcaec342	0.141939104	0.098482266	0.151791455

creatinine.lysopcac204	-0.037496724	0.026017042	0.151799525
pcaec403.pcaac405	-0.085564469	0.059369572	0.151805009
c4.pcaec404	-0.025457051	0.017666065	0.151862356
c142.smc181	-0.03169397	0.02199933	0.151956974
c182.c101	-0.015546347	0.010791154	0.151963251
c3.pcaac300	-0.08060457	0.055954341	0.151995634
glycine.leucine	-0.034129209	0.02369426	0.152035721
pcaec401.c182	-0.041224734	0.028620994	0.152045017
pcaec300.valine	0.09256579	0.064267609	0.152058851
methioninesulfoxide.pcaac366	0.041529735	0.028834485	0.152069531
methionine.asymmetricdimethyla	0.027564572	0.019145388	0.152219272
pcaec422.lysopcac161	0.117041311	0.081307328	0.152291973
pcaac322.alanine	-0.064403703	0.044745866	0.152340289
methioninesulfoxide.prolinr	-0.09298267	0.064614216	0.152419004
pcaac240.pcaac405	-0.08670217	0.060260582	0.152491449
pcaac342.smohc221	0.040887925	0.028420148	0.152517609
c8.glutamine	0.037529199	0.026088134	0.152557427
pcaec362.pcaec385	-0.100026224	0.069536727	0.152582412
pcaac341.serotonin	-0.175730217	0.122176673	0.152621305
arginine.alphaaminoadipicacid	-0.002302943	0.00160113	0.152622594
pcaac320.lysopcac203	0.020184908	0.014035138	0.152666393
pcaac321.pcaec362	-0.047374203	0.032940779	0.152668293
pcaac421.pcaec342	0.167650056	0.116574884	0.152677045
pcaac404.pcaec363	-0.072700645	0.050559393	0.152735481
pcaec405.alphaaminoadipicacid	-0.005469823	0.003804097	0.15274927
arginine.serine	0.085924656	0.059759119	0.152756865
pcaec386.pcaec365	-0.052985361	0.0368511	0.152764534
smohc221.alphaaminoadipicacid	-0.006711135	0.004668939	0.15288434
pcaac424.alanine	-0.030747561	0.021391334	0.152888984
lysopcac161.c51	-0.122298594	0.085087761	0.152906284
pcaac386.pcaec405	0.114385945	0.079593449	0.152961682
pcaac384.pcaec362	-0.019544636	0.013600687	0.152988531
pcaac403.lysopcac280	-0.019186309	0.013354963	0.153099274
pcaec362.prolinr	0.037121788	0.025843046	0.153158979
pcaec443.c18	0.085017905	0.059188563	0.153170797
pcaac425.pcaac381	0.141257148	0.098350892	0.153208804
pcaac281.lysopcac280	0.010733611	0.00747353	0.153220107
pcaec361.citruline	-0.03397878	0.023659123	0.153230403
pcaac366.c142	0.052354128	0.036461779	0.153320643
pcaac425.kynurenine	-0.026325719	0.018337487	0.153388895
pcaec401.pcaac385	-0.070571896	0.049164636	0.153446654
pcaec341.pcaac344	0.101428176	0.070678175	0.153545973
pcaec343.creatinine	-0.029111555	0.020294403	0.153719115

histidine.smohc161	-0.016532172	0.011525332	0.153731023
pcaac344.pcaac420	-0.128482083	0.089584721	0.153794833
spermidine.asymmetricdimethyla	0.15510352	0.108149985	0.153807466
pcaac384.smc160	-0.173760141	0.121159053	0.153808314
c8.smohc222	0.003875892	0.002702672	0.153823391
smohc241.lysopcac182	-0.035579904	0.024810079	0.15382494
pcaac405.pcaac320	-0.175232147	0.122213892	0.153903408
lysopcac161.c12	0.114858296	0.080120477	0.153973378
lysopcac204.pcaec421	0.013276074	0.009262884	0.154063158
pcaec322.pcaec386	0.051407245	0.035869788	0.154089447
lysopcac182.smc181	-0.034966182	0.024398338	0.154096554
pcaac383.smohc141	-0.034864868	0.02432821	0.154106061
pcaac421.pcaac405	-0.07497582	0.052320157	0.154130244
pcaec445.lysopcac180	-0.054921638	0.038339699	0.15427845
c162.pcaac362	-0.049282512	0.034403432	0.154281942
smohc141.pcaec361	-0.022034577	0.01538317	0.154312253
pcaec343.glycine	0.072832275	0.05084867	0.15432601
pcaac363.pcaec361	-0.018518667	0.012929337	0.154336219
pcaac323.kynurenine	-0.006418356	0.004481449	0.154362649
pcaac424.pcaac366	0.051513766	0.035974248	0.154432171
pcaec404.alphaaminoadipicacid	-0.006410475	0.004478431	0.154589769
pcaec382.lysopcac160	0.128293711	0.089631725	0.154609296
glycine.pcaec300	-0.032855225	0.022955135	0.154627226
pcaec385.putrescine	-0.092604441	0.06471383	0.154711957
smc240.citruline	-0.016163029	0.011295913	0.154743351
c18.pcaac281	-0.002989276	0.002089202	0.15475825
pcaac320.c2	-0.072450556	0.050641807	0.154808298
pcaec405.pcaac385	-0.059617441	0.041672233	0.154813934
c0.alphaaminoadipicacid	0.016113818	0.011264095	0.154836962
c142.pcaec362	0.026811437	0.018742466	0.154845234
pcaec406.alphaaminoadipicacid	0.008729034	0.006102518	0.154879426
pcaac363.pcaac281	0.00845439	0.005910866	0.154903911
pcaac404.pcaec365	0.040813767	0.028535734	0.154916672
c162.pcaec361	-0.01985675	0.01388414	0.154943499
pcaac322.spermidine	-0.103201177	0.072160176	0.154945501
pcaac300.kynurenine	-0.016879269	0.01180311	0.154973696
c51dc.kynurenine	0.012730581	0.008903261	0.15502845
glutamicacid.asparagine	0.023891156	0.016708567	0.155029556
smc160.pcaec362	0.018454638	0.012908234	0.155085478
pcaac362.pcaac406	-0.076658402	0.053645308	0.15528434
pcaac341.pcaec445	0.047271621	0.033082809	0.155312579
pcaec405.alanine	-0.024429674	0.017098699	0.155353669
pcaac341.asparagine	0.013278155	0.009294437	0.155391396

c16.lysopcac170	-0.078805517	0.055168813	0.155440189
pcaac421.leucine	-0.031840998	0.022291623	0.155457195
pcaac421.pcaac361	0.103108172	0.072192713	0.155499943
lysopcac281.pcaec382	0.051550201	0.036095515	0.155521303
lysopcac204.lysopcac170	-0.03918707	0.027440854	0.155551479
lysopcac204.lysopcac280	0.012810457	0.008971249	0.155583101
pcaec322.pcaac381	0.052931889	0.037069521	0.155593771
smc241.pcaec383	-0.132936335	0.093100269	0.155600996
pcaec421.leucine	-0.123756738	0.086673554	0.15561093
pcaec404.pcaac385	-0.059031283	0.041355577	0.155737673
isoleucine.lysopcac180	0.086237323	0.06042623	0.15581176
pcaac383.smc181	-0.049240763	0.034510597	0.15590391
pcaac420.c142	0.086269616	0.060468068	0.155942235
pcaac365.pcaec380	-0.092022259	0.06451483	0.15603534
pcaac364.smc161	-0.064293801	0.045080998	0.156089976
putrescine.serotonin	0.138328491	0.097009353	0.156163342
pcaec386.isoleucine	0.069515933	0.048761455	0.156248822
pcaac406.pcaec382	0.012530139	0.008789544	0.156265984
glutamicacid.pcaec443	0.153349678	0.107596846	0.156366549
c51dc.pcaac406	0.098217771	0.068937585	0.15650793
pcaec423.lysine	0.142714236	0.100181941	0.156561113
pcaec362.asparticacid	0.038998802	0.027379064	0.156603918
c181.c14	-0.06131331	0.043048745	0.156640287
spermidine.kynurenine	-0.063105837	0.044312403	0.156687697
pcaac401.c181	-0.022862784	0.016060827	0.15686114
kynurenine.alphaaminoadipicaci	0.006442134	0.004526723	0.156970498
pcaec321.smohc161	0.048100209	0.033801239	0.157000366
leucine.pcaec362	-0.018366819	0.012907512	0.157022118
smc240.lysopcac160	0.051733712	0.036357396	0.157032099
c181.pcaec406	-0.0126742	0.008907947	0.157068094
c5.pcaec424	-0.035353623	0.024849254	0.157089279
pcaec360.pcaec445	0.084574789	0.059492336	0.157412493
pcaec342.transhydroxyproline	0.049392042	0.034744593	0.157422119
pcaec445.pcaac406	-0.050255721	0.035352226	0.157423082
pcaac322.leucine	-0.010782625	0.007585407	0.157445005
prolinr.pcaec301	0.102319235	0.071983031	0.157462558
pcaec321.pcaac341	0.024406656	0.017171124	0.15747925
lysopcac203.c141	0.039218165	0.027598038	0.157574673
pcaec321.pcaac403	-0.011564108	0.008138414	0.157609524
pcaec386.lysopcac182	-0.017471325	0.012295966	0.157618226
pcaec341.c16	0.06089649	0.042859849	0.157638711
pcaec322.pcaec404	-0.009781428	0.006885733	0.157723748
pcaac300.c8	-0.069863675	0.049183607	0.157743727

pcaac422.asparagine	-0.015843137	0.011155416	0.157815578
smc261.pcaec444	-0.166941127	0.117546177	0.157816133
kynurenine.c101	-0.022128336	0.015582914	0.157868144
pcaec341.c51	0.073075137	0.051465676	0.157913878
pcaec425.citruline	-0.01345518	0.009477213	0.157954838
pcaec425.pcaec423	0.025221253	0.017768533	0.158043953
pcaec300.c16	0.069822245	0.049191828	0.158057502
pcaac323.transhydroxyproline	0.043559348	0.030691471	0.158092842
pcaac365.asymmetricdimethylar[illegible]	0.035800616	0.025228858	0.158160126
smc181.smohc221	0.025796651	0.018179941	0.158181072
pcaac281.lysopcac182	-0.031237585	0.022017986	0.158248609
h1.pcaec404	0.046121763	0.032513959	0.158309301
pcaac420.lysopcac180	-0.076190308	0.053718798	0.158368857
pcaec445.pcaec322	0.107444311	0.075759755	0.158396041
lysopcac160.lysopcac170	0.036518595	0.025751188	0.158422753
pcaec363.pcaec443	-0.049527665	0.034926077	0.158440586
pcaac322.pcaec423	-0.007542452	0.005319958	0.158529764
pcaec382.pcaec365	0.030005519	0.021166659	0.158582589
pcaac364.creatinine	0.015213395	0.010733122	0.158629016
pcaec444.pcaec340	0.051511568	0.03634382	0.15865363
glycine.citruline	-0.0186918	0.013189606	0.15870588
pcaec403.arginine	-0.178825271	0.126201397	0.158757852
pcaac406.lysopcac182	-0.015501481	0.010941994	0.158841668
pcaac404.c181	-0.011109805	0.007842572	0.158869136
pcaac366.pcaec300	0.027484146	0.019404287	0.158929457
pcaac281.pcaac384	0.131122871	0.092590194	0.158997187
pcaec380.serotonin	-0.169169975	0.119457189	0.158999532
alphaaminoadipicacid.c10	0.043226598	0.030527657	0.159050419
pcaec343.pcaac363	-0.020461474	0.014451214	0.159074236
pcaac426.alphaaminoadipicacid	0.025774337	0.01821006	0.159223369
pcaac364.pcaec444	0.129234804	0.091322888	0.159296082
pcaec343.lysopcac280	-0.013012696	0.009195924	0.159322728
c0.pcaac344	-0.107948264	0.076295875	0.159377372
methionine.pcaec383	-0.09878453	0.069820846	0.159387642
pcaac342.asparticacid	0.050166723	0.035458125	0.159391325
asparagine.pcaac405	-0.186052097	0.131585325	0.159651623
pcaec363.pcaac360	0.065078202	0.046029809	0.159680959
alphaaminoadipicacid.pcaec360	-0.009898411	0.007002162	0.159741133
pcaac321.c8	-0.065550983	0.046373207	0.159761402
alphaaminoadipicacid.lysopcac1[illegible]	0.03457878	0.024469405	0.159881514
c51dc.pcaac405	0.124313866	0.087980137	0.159930522
asparticacid.pcaec300	0.023489206	0.016625948	0.159981157
lysopcac170.pcaec362	0.040129273	0.028409545	0.160062248

pcaac342.pcaec382	0.018915989	0.013393098	0.16010907
c51.pcaac341	0.004538943	0.003214086	0.160157527
c12.c181	-0.033341211	0.023611507	0.16019537
pcaec361.pcaac405	-0.060201362	0.042636297	0.160224823
smohc221.valine	-0.055227778	0.039114409	0.160230592
pcaac420.serotonin	-0.188259727	0.133345035	0.160269031
lysopcac140.pcaac366	0.037588224	0.026624342	0.160276354
pcaac424.pcaac361	0.07173004	0.050811887	0.160311871
lysopcac180.c141oh	-0.12807505	0.090729929	0.160332695
c12.c2	0.077890612	0.055191146	0.160426622
alphaaminoadipicacid.c2	0.088838156	0.062949162	0.160432472
c5.pcaec403	-0.182114206	0.129053839	0.160467544
pcaec423.pcaac426	-0.133792642	0.094821006	0.160510787
citruline.creatinine	-0.014102347	0.009994897	0.160524735
ornithine.pcaac422	-0.037719605	0.026735361	0.16055533
pcaec301.alphaaminoadipicacid	-0.007227025	0.005123073	0.160605097
lysopcac281.alphaaminoadipicac	-0.004330917	0.003070152	0.160613735
pcaec364.smohc222	-0.009155477	0.006490807	0.160649849
pcaac366.pcaac320	0.128051502	0.090788055	0.160675006
prolinr.pcaac341	0.013142852	0.009319748	0.160742461
pcaec385.kynurenine	-0.013127808	0.009310084	0.160787282
c16.c51	0.081536595	0.057824835	0.1607877
phenylalanine.smohc241	0.106716272	0.075703911	0.16090812
pcaac402.alanine	-0.040533194	0.028760346	0.160999638
pcaec382.lysopcac280	-0.015576842	0.011055908	0.16112576
c102.ornithine	-0.031596378	0.022432141	0.161239175
lysopcac182.smohc222	0.006030989	0.00428179	0.161242399
pcaec381.pcaec423	-0.026348526	0.018709682	0.16131258
alanine.taurine	-0.070890894	0.050347469	0.16138647
pcaac300.smohc241	0.074898168	0.053194241	0.161392438
pcaec405.lysopcac280	-0.029415553	0.020891889	0.1613994
prolinr.lysopcac280	-0.013657688	0.009700904	0.16143228
pcaac342.putrescine	-0.039930524	0.028365576	0.161481724
pcaac426.pcaec300	-0.033647942	0.023902863	0.16148635
pcaec420.pcaac402	0.062440242	0.044365133	0.161568574
lysopcac180.lysopcac204	0.046599918	0.033114182	0.161618106
smohc241.pcaac365	0.097876114	0.069563809	0.161692653
pcaac386.c102	0.059871043	0.042552451	0.161693729
smc261.pcaec300	-0.019647893	0.01396723	0.161776727
pcaec403.c101	0.032554535	0.023144311	0.161813616
pcaac300.asparagine	0.009540038	0.006782394	0.161813749
pcaac420.pcaec342	0.174690253	0.124195284	0.161817125
pcaac401.c142	0.051371576	0.036525121	0.161848145

c182.c181	-0.022413808	0.015939077	0.161923675
c10.c3	0.077159007	0.054872705	0.161945
pcaac422.pcaec382	-0.02445168	0.017389672	0.161957427
lysopcac160.pcaac323	-0.157804348	0.112260164	0.162076426
glutamicacid.pcaec382	-0.02525232	0.017964471	0.162082598
pcaac323.leucine	-0.014478276	0.01030018	0.162096845
pcaac323.pcaec402	-0.044703768	0.031804541	0.162113183
pcaec422.c101	-0.06205246	0.044147707	0.162117159
pcaac366.pcaec342	0.154455978	0.109909669	0.162195898
pcaac364.pcaac380	0.079536562	0.056607874	0.162271597
pcaec380.pcaec340	-0.098046745	0.069787344	0.162303725
pcaec422.lysopcac170	0.018492032	0.013163501	0.162345276
leucine.smc202	0.084823229	0.060383411	0.162360721
glycine.pcaec402	-0.080407907	0.057243429	0.162383807
pcaec384.lysopcac204	-0.054069412	0.03850436	0.162509996
pcaec360.c5	0.017223559	0.012265607	0.162517575
c16.creatinine	0.019092274	0.013600708	0.162649882
kynurenine.pcaec301	-0.101306769	0.072171481	0.162672268
lysopcac180.pcaac362	0.049512547	0.035274674	0.162692059
pcaec404.c101	0.032724352	0.023314888	0.162706022
pcaac405.smc160	-0.14104749	0.10049386	0.162717453
lysopcac161.pcaec300	0.042786655	0.03048519	0.162723611
smc202.pcaec421	0.002533579	0.001805261	0.162747584
smc240.pcaac363	-0.014565437	0.010379021	0.162773887
prolinr.c141	-0.081789969	0.058288228	0.162819957
pcaac383.kynurenine	-0.012916939	0.009205831	0.162841666
lysopcac181.pcaec340	0.068580784	0.048897324	0.163014031
pcaac323.prolinr	0.012469751	0.008890828	0.163015772
pcaec383.lysopcac170	-0.05103354	0.036389813	0.163053929
pcaac322.pcaac366	0.083135479	0.059290549	0.163126015
pcaac406.pcaec361	-0.01660604	0.01184467	0.163181633
smc161.smc181	-0.034509917	0.024620562	0.163275128
lysopcac204.alphaaminoadipicac	0.006201813	0.004424604	0.163276811
lysopcac140.asparticacid	-0.074977962	0.053495614	0.163304479
c181.pcaec362	0.031627716	0.022565899	0.163304745
lysopcac204.c101	-0.034053859	0.02430482	0.163440706
smohc161.c51	0.049114534	0.03505462	0.163449531
lysopcac181.c16	0.098969443	0.070640859	0.163468407
pcaec403.pcaec401	0.116906455	0.083457001	0.163535266
c18.pcaac322	-0.092736574	0.066224471	0.163673144
pcaec422.alphaaminoadipicacid	-0.0216947	0.015492943	0.163685488
creatinine.smohc222	0.005913229	0.004223544	0.163754899
pcaac420.pcaac405	-0.081529338	0.058239401	0.163803705

isoleucine.pcaec340	-0.054940185	0.039247919	0.163826385
c162.c18	-0.0814951	0.058220342	0.163842503
pcaec402.pcaec406	0.028337009	0.020245172	0.163865841
lysopcac182.putrescine	-0.090943146	0.064980487	0.163909705
lysopcac281.pcaec362	0.021943916	0.01568548	0.164074247
c51dc.phenylalanine	0.095832323	0.068505718	0.164104239
smc202.pcaec406	0.025195393	0.018011406	0.164115508
pcaac420.pcaec361	-0.034354636	0.024561208	0.164152037
acetylornithoine.pcaac401	-0.029436903	0.021045427	0.164153227
pcaec321.pcaec443	-0.037444281	0.026772624	0.164191644
pcaec365.smohc141	-0.098853318	0.070691029	0.164256841
pcaec384.prolinr	0.047042157	0.033640918	0.164264127
pcaac341.smohc241	0.080275562	0.057412607	0.164305894
pcaac343.pcaec424	-0.03252448	0.023261597	0.164310951
alphaaminoadipicacid.pcaec301	0.079980083	0.057206102	0.164341192
c182.pcaec403	0.050641887	0.036223716	0.16436301
pcaac385.pcaac322	-0.066864019	0.0478297	0.164384291
methioninesulfoxide.pcaec443	0.173341	0.124026909	0.164489887
pcaec404.lysopcac161	0.072803058	0.052091969	0.164496191
creatinine.smc181	0.050755736	0.036317325	0.1645035
pcaec425.pcaac406	-0.056665213	0.040555248	0.164601747
c4.spermidine	-0.100927726	0.072237073	0.164620203
pcaac366.c182	-0.047906212	0.034291599	0.164664846
pcaac343.pcaac385	0.082856228	0.059319704	0.164740069
lysine.pcaec424	-0.014399939	0.010310605	0.164788056
arginine.c12	-0.079105037	0.056649628	0.164855088
pcaec384.pcaac322	-0.092280802	0.066086734	0.164864749
pcaac281.pcaac402	-0.070898225	0.050788034	0.164983667
c51.pcaac420	0.109495434	0.078439272	0.164994775
pcaec405.pcaec300	0.022176644	0.015888266	0.165036458
lysopcac180.smohc221	-0.07107037	0.050925539	0.165100581
pcaac240.pcaac426	0.12356144	0.088560757	0.165208146
pcaec362.smohc222	-0.014263782	0.010224192	0.165242883
pcaac342.pcaac426	0.085836616	0.061536391	0.165305851
alanine.serotonin	0.119137712	0.085411143	0.165311501
pcaac365.c182	-0.04165796	0.029868681	0.165362507
c16.glycine	0.072622361	0.052073317	0.165388453
pcaac366.c181	-0.026767984	0.019194909	0.165413127
pcaec420.pcaac366	-0.035308506	0.025320826	0.165440336
c181.lysine	0.095862195	0.068758119	0.165515851
pcaac362.pcaac320	-0.087920623	0.063073089	0.165590069
pcaac240.pcaac380	0.093648285	0.067195382	0.165673516
pcaec422.glutamicacid	0.090814418	0.06516329	0.165681834

lysopcac182.pcaec380	-0.061934881	0.044444976	0.165719735
pcaec341.pcaec385	-0.083694619	0.060072335	0.165806448
pcaec343.pcaec362	0.039715055	0.028509284	0.165858869
histidine.alphaaminoadipicacid	0.037737863	0.027090824	0.165872207
c101.pcaec360	-0.009902316	0.007109144	0.165906686
smohc241.smohc161	0.026285302	0.018872907	0.165950534
pcaec380.pcaac320	0.140790061	0.101094358	0.165978698
pcaac383.pcaac422	-0.045399606	0.032608617	0.166100102
pcaac281.pcaec421	0.002093621	0.001503819	0.166116727
pcaec385.pcaac364	-0.09220749	0.066233507	0.166130366
alanine.pcaac402	-0.081997008	0.058905964	0.166178519
pcaec424.pcaac406	-0.059418476	0.042689128	0.166211952
pcaac381.asparticacid	-0.055292564	0.039735711	0.166326736
asparagine.asymmetricdimethyla	0.022445782	0.016133516	0.166404269
pcaac323.taurine	-0.036110203	0.025956863	0.166431401
pcaec361.c181	-0.020489543	0.014730123	0.166481625
pcaec381.alanine	-0.026253898	0.018879017	0.166589703
pcaac323.smc181	-0.028210312	0.020286098	0.166594582
pcaec380.alphaaminoadipicacid	-0.004917172	0.003536187	0.166622843
pcaec422.creatinine	-0.037849222	0.027220052	0.166634682
pcaac240.smohc222	-0.012814816	0.009216595	0.166660022
c51.smc161	0.151390197	0.108882525	0.166662261
c3.lysopcac161	-0.163407593	0.117546336	0.166736381
pcaac404.pcaac361	0.125326318	0.090163257	0.166785308
pcaec341.c2	-0.080621476	0.058020123	0.166921682
pcaec386.tyrosine	-0.092216201	0.066365165	0.166926582
c4.c162	0.172049964	0.123856108	0.167052618
pcaec423.c101	-0.054398513	0.039162248	0.167069831
pcaec424.serotonin	-0.155270592	0.111789181	0.167098934
glycine.c12	0.068771121	0.049513652	0.167106979
c51.leucine	0.061588799	0.044342838	0.167109789
pcaac381.pcaec322	-0.111052679	0.07997492	0.167209793
c142.smohc221	-0.109211188	0.078661039	0.167275592
pcaac300.c2	-0.088824655	0.063987074	0.167339845
c8.c141oh	0.202700949	0.146049705	0.167423538
pcaac406.kynurenine	-0.009360679	0.006745443	0.167480046
pcaac322.smc160	0.096444439	0.069507252	0.167528468
pcaac360.alphaaminoadipicacid	-0.008790745	0.006337894	0.167690104
pcaec322.prolinr	0.021288874	0.015349098	0.167700687
c4.pcaec342	-0.075422563	0.054381286	0.167718192
lysopcac182.lysopcac170	-0.041226113	0.029739521	0.167925615
pcaec421.pcaac385	-0.056164186	0.040529997	0.168076853
pcaac281.pcaec380	-0.060577645	0.043718058	0.168107375

pcaec445.c12	0.059146838	0.042687717	0.168129665
pcaec364.pcaec444	0.147985177	0.106823456	0.168204586
c101.pcaac320	-0.075863892	0.054764472	0.168219542
c162.arginine	0.051937555	0.037498413	0.168285311
pcaac383.pcaec365	0.043563896	0.031453257	0.168292638
pcaec425.tyrosine	-0.076565705	0.05529001	0.168364136
lysopcac203.valine	-0.085421576	0.061688962	0.168390902
phenylalanine.pcaac402	-0.054154882	0.039118206	0.16848963
pcaac385.lysopcac161	-0.106242663	0.076755726	0.168558288
asparticacid.pcaac344	0.102742797	0.074236202	0.168609425
smohc161.c162	0.156805167	0.113321004	0.168692866
pcaec422.lysopcac203	0.082432328	0.059582689	0.168763468
pcaec404.arginine	-0.151436495	0.109462432	0.168775164
pcaac406.pcaac401	0.024242978	0.017524119	0.168790438
c5.kynurenine	-0.018431592	0.013323519	0.168796155
c182.c14	-0.037255374	0.026933037	0.168835414
pcaec420.c2	0.067628176	0.048891276	0.16884253
pcaac380.serotonin	-0.174453166	0.126122409	0.168851982
pcaec444.pcaac426	-0.115642945	0.083605745	0.168855466
smc202.c162	0.189212295	0.136795653	0.168861278
citruline.pcaec322	0.083826795	0.060612053	0.1689131
lysopcac170.c142	-0.052895745	0.038250034	0.168947152
pcaec342.c142	0.058141483	0.042043908	0.168952891
c141oh.alphaaminoadipicacid	0.004996198	0.0036148	0.169174822
c0.pcaac320	-0.076941846	0.05567768	0.169246827
c141oh.c2	0.12418385	0.089864941	0.169253308
pcaec362.c101	-0.027896443	0.020187303	0.169257512
pcaac320.asparagine	0.008615946	0.00623624	0.169345623
pcaac323.pcaac381	0.046597785	0.033729637	0.169371516
smc181.smohc241	0.134894441	0.097649677	0.169401134
pcaac281.c101	-0.014700278	0.010641982	0.169420849
glycine.pcaac364	0.08656888	0.062678032	0.169476043
pcaac401.pcaac381	0.137669572	0.099677534	0.169481671
lysopcac281.lysopcac182	-0.022195611	0.016071606	0.169513699
pcaac321.asparticacid	-0.058402814	0.042293269	0.169557924
lysopcac140.kynurenine	-0.019882347	0.014402243	0.169679907
pcaac321.c142	0.043957829	0.031846476	0.169741053
pcaec401.pcaac366	-0.049934	0.036181834	0.169808479
prolinr.pcaec423	-0.016672744	0.012081321	0.169821298
pcaec445.alphaaminoadipicacid	0.007104659	0.005149453	0.169929174
serine.asparagine	0.016703283	0.012107233	0.169953821
c10.c141oh	0.211476018	0.153304723	0.170003943
c51dc.c14	-0.041353315	0.029979255	0.170019654

smohc141.pcaac361	0.087230537	0.063240707	0.170036912
pcaec380.citruline	-0.025506893	0.018492379	0.1700438
pcaec300.pcaec405	0.09688639	0.070252063	0.170103465
pcaec365.glycine	0.048050021	0.034846364	0.170169479
pcaac386.smohc241	0.092845719	0.067338229	0.170204573
h1.c182	-0.051059283	0.037036391	0.17025747
pcaec406.prolinr	0.039272673	0.028487807	0.170271754
smc260.pcaec382	0.025425427	0.01844419	0.170294004
pcaec363.prolinr	0.028486295	0.020672338	0.170452619
pcaec301.pcaec423	-0.00794944	0.005769291	0.170484168
pcaec363.pcaec362	0.032429365	0.023539916	0.170562933
acetylornithoine.kynurenine	-0.037910257	0.027519892	0.170585946
pcaac404.pcaac380	0.069010131	0.050102041	0.170637342
leucine.prolinr	-0.072598035	0.052708607	0.17065114
pcaac406.histidine	-0.068336634	0.049627357	0.170759594
pcaac404.valine	-0.071980029	0.052276947	0.170789563
pcaac300.c181	-0.012605696	0.009157541	0.170900916
creatinine.pcaec340	0.093384517	0.067840532	0.170903206
smc241.pcaec382	0.015281161	0.011101227	0.170903444
c16.pcaec421	0.004475588	0.003251366	0.17090463
pcaac364.smc240	-0.041301419	0.030007038	0.170946053
c162.pcaec362	0.016392981	0.011911114	0.170981562
citruline.pcaac402	0.050897902	0.036985923	0.171022801
pcaec422.pcaec343	0.125601446	0.091279427	0.171063596
lysine.kynurenine	-0.008308598	0.006038275	0.171070327
c2.alanine	0.079263721	0.057606533	0.171082214
pcaec424.arginine	-0.096028487	0.069793729	0.171100843
c162.pcaac405	-0.072468001	0.052696702	0.171317053
c0.pcaec424	-0.014886419	0.010825522	0.171338042
pcaec380.pcaec404	-0.039650115	0.028834821	0.171351966
pcaac403.pcaec406	0.034222795	0.024888445	0.171361251
pcaec405.pcaec404	-0.04951143	0.036026022	0.171585132
lysopcac161.pcaec360	-0.005446042	0.003963448	0.171664766
pcaec384.asparagine	0.019735932	0.014365879	0.171745598
pcaac403.pcaec402	-0.18973192	0.138111611	0.171760502
pcaec403.c182	-0.077929153	0.056728661	0.171773037
serine.pcaac361	0.094282539	0.068633431	0.171774867
smc241.prolinr	-0.058991609	0.042945812	0.171800484
pcaac344.c8	-0.050652497	0.036878609	0.171842726
pcaac404.c14	0.042612842	0.031030127	0.171910746
c14.pcaac406	0.078486179	0.05716459	0.171999835
pcaec424.pcaec404	-0.04513271	0.032873061	0.172014374
alanine.pcaec340	0.094031367	0.068501045	0.172088632

pcaec402.pcaac341	0.009834251	0.0071648	0.172126265
pcaac426.putrescine	-0.042459216	0.03093921	0.172199286
pcaec385.pcaec364	0.047307403	0.034472829	0.172209652
pcaec446.pcaac381	0.034132054	0.024872847	0.172224623
pcaac426.smc181	-0.023881128	0.01740542	0.172289976
smc240.valine	-0.041125723	0.029980661	0.172386127
smc160.c162	0.129737505	0.094589016	0.172432744
pcaec340.pcaec300	-0.049540623	0.036123145	0.172480768
h1.pcaac341	-0.027278643	0.01989348	0.172543509
smc261.pcaac365	0.051714975	0.037716974	0.172575431
pcaac384.pcaac420	-0.090915481	0.066316564	0.172637936
pcaec383.lysopcac161	0.094433453	0.068883145	0.172640799
pcaec383.c101	0.033424751	0.024382502	0.172663386
serotonin.glutamine	0.020723789	0.015124886	0.172872431
pcaac386.lysopcac170	0.017584923	0.012834229	0.172878711
lysopcac181.asparticacid	0.028851951	0.021058038	0.172891868
pcaec380.c182	-0.0525763	0.038383931	0.173006541
pcaac365.c181	-0.023184547	0.016932322	0.17316228
pcaac281.pcaac420	-0.101342301	0.074023035	0.173219528
pcaac425.pcaec422	-0.049098799	0.035866122	0.173256318
serine.c182	-0.056534834	0.041299752	0.173273792
pcaac426.creatinine	-0.030421442	0.022225068	0.173305238
pcaec386.pcaac362	-0.040843473	0.029840121	0.173319599
c18.pcaec301	-0.03921951	0.028654612	0.173333807
pcaac380.asymmetricdimethylar;	0.038217329	0.027927428	0.173410747
pcaac406.pcaac362	-0.074888795	0.05472767	0.173429937
pcaac300.smohc161	-0.037885467	0.027691135	0.173506644
serine.histidine	0.098819211	0.072233686	0.173536444
putrescine.pcaec361	0.016701952	0.012209791	0.173578367
arginine.pcaec300	0.029543362	0.021598741	0.173605433
pcaac366.pcaac421	-0.133013019	0.097248014	0.173623207
c141oh.pcaec401	-0.085054842	0.062192768	0.173676586
pcaec363.pcaac405	-0.062128326	0.045430323	0.173691669
phenylalanine.c101	0.019605036	0.014335932	0.17369373
pcaac405.creatinine	-0.062319552	0.045570845	0.173698156
lysopcac204.pcaec404	0.019906602	0.014556981	0.173709386
pcaec361.pcaec401	0.189581532	0.138644767	0.173742136
pcaac380.c181	-0.019454161	0.01423058	0.17384316
pcaac300.lysopcac280	0.012757789	0.009333326	0.173893304
pcaec360.pcaac385	-0.074773564	0.054727509	0.17408684
lysopcac180.pcaec421	-0.012366409	0.009051492	0.174105502
pcaec361.alanine	-0.022207483	0.016258265	0.174202329
lysopcac170.pcaac420	-0.138611612	0.101488099	0.174242365

pcaac320.c8	-0.075286068	0.055130221	0.174301094
pcaac322.pcaac401	0.042038307	0.030784979	0.174319342
pcaec421.alphaaminoadipicacid	-0.008909912	0.006525027	0.17433441
smc181.lysopcac181	0.071291459	0.052210918	0.174349087
pcaec420.c182	-0.036249333	0.026551289	0.174409952
lysopcac281.smc181	-0.014834352	0.010868446	0.174521587
glutamine.lysopcac280	0.008602656	0.006304192	0.17461815
pcaec300.smc240	-0.052330608	0.038360474	0.174747666
asparticacid.asymmetricdimethyl	-0.032341869	0.02370925	0.174771841
c12.pcaec404	0.031522707	0.023109655	0.174788849
pcaec381.pcaec342	0.127126707	0.09320849	0.1748369
lysopcac204.pcaec406	-0.113786415	0.083427981	0.17483951
pcaec343.pcaec340	-0.108220124	0.079359611	0.174908734
pcaac381.smohc161	0.055244179	0.040513241	0.174927436
c0.c12	-0.048883966	0.035853865	0.174985727
methioninesulfoxide.pcaac281	-0.006235488	0.00457401	0.175042029
pcaac366.pcaec380	-0.171061204	0.125524481	0.175190339
lysopcac180.pcaac402	0.100223356	0.0735491	0.175221441
pcaec422.c5	0.030867665	0.02265429	0.175259203
pcaac322.pcaac281	-0.006325442	0.004643165	0.175334728
methionine.smohc221	-0.041265555	0.030291787	0.175348576
prolinr.c14	0.052448506	0.038502312	0.175364837
asymmetricdimethylarginine.c10	0.011454518	0.008409725	0.175415476
c182.lysopcac180	-0.056125236	0.041210103	0.175455519
pcaac380.c142	0.08722786	0.064048335	0.175462532
smc261.pcaec404	0.025659057	0.018844255	0.175547133
pcaec321.pcaac381	0.03247251	0.02384889	0.175560984
pcaec443.alanine	-0.02602019	0.019114226	0.175653764
pcaac385.smohc221	0.024735872	0.018174911	0.175751381
h1.spermidine	0.091965238	0.067577331	0.175783279
c5.lysine	-0.220808434	0.162267457	0.175821259
c101.c142	0.109467538	0.080459528	0.175896717
pcaec422.pcaac365	-0.117314057	0.086229078	0.17590814
pcaac362.smc181	-0.040647671	0.029878238	0.175923585
pcaec380.pcaec300	0.025553987	0.018786007	0.175979419
pcaec362.pcaac362	-0.021877278	0.016084377	0.176014161
smohc161.c14	-0.056240094	0.041351848	0.17605167
pcaec362.c51	0.113504077	0.083457957	0.176058985
asymmetricdimethylarginine.pca	0.006015685	0.004423675	0.176100375
c4.pcaac362	-0.020967338	0.015418717	0.176107082
pcaec383.pcaec361	0.020021614	0.014724343	0.176138629
pcaac240.pcaec382	0.026707334	0.019642471	0.176167039
c181.alphaaminoadipicacid	0.005002098	0.003678966	0.176174974

c0.lysopcac204	0.060247283	0.044311981	0.176184967
pcaec421.pcaac403	-0.062951896	0.046304529	0.176215581
pcaac381.pcaec380	-0.134494353	0.098929376	0.176222068
pcaec364.pcaac361	0.162611299	0.119625096	0.176271877
pcaac365.c162	-0.113878697	0.083793931	0.176369305
pcaec444.pcaac341	0.006342702	0.00466729	0.176389554
h1.pcaec421	-0.015965417	0.011750315	0.176467393
c101.pcaec403	0.031558913	0.023226951	0.176468328
c12.creatinine	0.049478351	0.036418212	0.176501209
creatinine.pcaec423	0.013474543	0.009919201	0.176559812
leucine.alphaaminoadipicacid	-0.006096708	0.004488118	0.176565927
pcaac406.pcaac420	-0.074131239	0.054577964	0.17661271
pcaec381.asparagine	0.022736677	0.016740337	0.176633613
lysopcac182.lysopcac280	0.01482439	0.010919163	0.176807171
smohc141.phenylalanine	0.078158263	0.057573001	0.176838312
c141.pcaec404	0.03057503	0.022525336	0.176898148
pcaec300.lysopcac280	0.013531019	0.009969818	0.176949972
glutamine.lysopcac204	-0.02877242	0.021200262	0.176957997
c51.pcaac405	-0.058179144	0.04286837	0.176962761
pcaac361.pcaec300	0.029320429	0.02160433	0.176963594
alanine.pcaac403	-0.015316858	0.011286195	0.176970927
pcaac363.pcaec444	0.131208667	0.096681018	0.176971722
pcaac300.leucine	-0.011298274	0.008327699	0.177104636
pcaec420.putrescine	-0.04783565	0.035261135	0.177136074
c0.lysopcac203	0.024218755	0.017854469	0.177186129
pcaac240.pcaec404	-0.13625557	0.100450264	0.177187798
methionine.pcaec423	-0.017294606	0.012754182	0.177331625
c102.c182	-0.1463795	0.107968801	0.17740701
smc180.pcaec362	0.055782024	0.04114589	0.177421062
c16.alphaaminoadipicacid	0.014241688	0.010505808	0.177456552
pcaec425.asparticacid	0.048605239	0.035859101	0.177504455
c101.glutamine	-0.01797959	0.013266755	0.177572501
smc240.pcaac380	0.120300409	0.088784606	0.177657403
pcaac342.lysopcac280	0.021106282	0.015577198	0.177664372
pcaac321.smohc161	-0.031962801	0.023593459	0.177733003
pcaec446.pcaec322	0.113680922	0.083917829	0.177752697
pcaec343.threonine	-0.089337169	0.065966407	0.177875694
citruline.pcaec403	-0.053876019	0.039783974	0.177897546
c142.lysine	0.091360523	0.067464645	0.177902505
kynurenine.prolinr	0.026350095	0.019459576	0.177935773
pcaac421.asparagine	-0.015418148	0.011389017	0.178037832
smc180.pcaac366	0.093137085	0.06881163	0.178122355
pcaac381.pcaac420	-0.183619302	0.1356636	0.178128113

c142.phenylalanine	0.048932963	0.036153455	0.178131386
c16.pcaec383	0.117875001	0.087110931	0.178233282
pcaac366.pcaec423	-0.009876314	0.007298855	0.178242108
pcaec360.pcaac380	0.051252635	0.037877738	0.178250056
pcaec425.pcaac405	-0.091760555	0.067815326	0.178254008
methioninesulfoxide.smohc241	0.117005369	0.086474199	0.178262873
lysopcac180.smc240	-0.087041125	0.064335631	0.17830886
pcaac365.glutamicacid	0.071435368	0.052802857	0.178325804
pcaac322.kynurenine	-0.018368978	0.013580796	0.178421229
methioninesulfoxide.kynurenine	-0.02992805	0.022132315	0.17852869
methionine.c5	-0.034899564	0.025816464	0.178656184
pcaec361.c51	0.101113711	0.074799037	0.178665331
lysopcac204.c141oh	-0.19947687	0.147564899	0.178669866
pcaec340.pcaac426	0.110340389	0.081628188	0.178684897
lysopcac160.lysopcac182	0.063094252	0.046677526	0.178697201
smc181.pcaac405	-0.116711017	0.086345156	0.178705245
ornithine.lysopcac280	-0.03494879	0.025856814	0.178721889
pcaec445.pcaac341	0.006756255	0.004998816	0.178740022
pcaac323.smohc222	-0.012240487	0.009058354	0.178828916
methioninesulfoxide.pcaac426	0.215776877	0.15968534	0.178838434
pcaac360.pcaac341	0.013265618	0.009817589	0.178855536
pcaec403.smohc222	-0.021037775	0.015569772	0.178860604
glutamine.alanine	0.056517791	0.041828851	0.178868858
pcaec420.pcaac365	0.058505886	0.043302283	0.178889215
c3.lysopcac203	0.047093793	0.034856667	0.178900101
smohc221.pcaac320	0.181370422	0.13425588	0.178944503
pcaec361.pcaac422	-0.041828683	0.030964076	0.178961482
c18.pcaac366	0.07767815	0.057505696	0.178989382
c102.asymmetricdimethylarginin	-0.015733242	0.011648637	0.179034001
c142.lysopcac170	-0.023459937	0.017371351	0.179083614
pcaec365.tyrosine	-0.086668836	0.064176095	0.179087148
pcaec445.c14	-0.063015259	0.046680736	0.179267808
smohc221.asparticacid	0.042876182	0.031767131	0.179337367
pcaac320.pcaac380	0.123985929	0.091879146	0.17941961
pcaac406.putrescine	-0.0395235	0.029289829	0.179436415
pcaec404.lysopcac280	-0.01256086	0.009309704	0.179491203
pcaec443.pcaec362	0.013799751	0.010228107	0.179498661
pcaac240.pcaac402	-0.104664186	0.077586511	0.179562484
pcaac343.pcaec361	-0.020746686	0.015381718	0.179630163
lysopcac180.pcaac366	0.057994237	0.042998676	0.179644225
c101.transhydroxyproline	0.039340896	0.029172054	0.179696497
pcaac381.creatinine	-0.02512897	0.018642907	0.179911769
pcaec422.smohc222	-0.011916725	0.008842468	0.179989094

pcaec421.lysopcac203	0.158650854	0.117729202	0.180014197
pcaec321.pcaac406	-0.056565534	0.041987954	0.180144741
pcaec382.pcaac381	0.069441001	0.051547704	0.180165195
methionine.pcaac422	-0.038976889	0.028940213	0.180266048
phenylalanine.lysopcac181	0.060339375	0.044802145	0.180269475
asparticacid.lysopcac181	0.131871772	0.09792048	0.180293091
smc202.alphaaminoadipicacid	0.017743822	0.013176105	0.180311206
lysopcac203.pcaac426	-0.106345574	0.078971713	0.180323435
lysopcac203.smc181	-0.015921405	0.011823235	0.180326208
c16.pcaec406	-0.011926189	0.008856633	0.18033822
lysopcac180.pcaac405	0.122264129	0.090801497	0.180365112
pcaac424.pcaac281	-0.007437	0.005523565	0.18039276
pcaec300.c51	0.117863483	0.087539136	0.180394052
pcaec301.pcaec365	0.057307506	0.042563804	0.180399978
pcaac386.smc181	0.033474707	0.024866881	0.180475345
histidine.pcaec382	-0.038502207	0.028604336	0.18051694
pcaec365.smohc221	0.036176732	0.026877221	0.180525693
c14.pcaec365	0.033994355	0.02525658	0.180538369
alanine.pcaac422	-0.019168345	0.014247705	0.180730398
pcaec342.tyrosine	-0.069713415	0.051821768	0.180765883
smc261.smc160	-0.126807591	0.094277172	0.180831151
pcaec424.c101	0.035123798	0.026119779	0.180937637
pcaec445.c181	-0.020368625	0.015149038	0.180992841
ornithine.smc202	0.060546101	0.045033966	0.18102345
c141oh.c101	-0.008255617	0.006140698	0.18103761
pcaec301.pcaec421	-0.004918386	0.003658743	0.181078616
pcaac322.pcaac363	0.041289567	0.030716716	0.181103739
smc260.serine	-0.019446143	0.014466683	0.18110478
pcaec381.pcaec382	-0.046252276	0.034410843	0.181131434
pcaac424.lysopcac161	0.088094158	0.065543071	0.181148868
h1.pcaec405	-0.071769209	0.053399259	0.18116623
pcaac406.pcaec420	-0.100506557	0.07483608	0.18148533
serotonin.pcaac424	-0.046201992	0.034408454	0.181573058
acetylornithoine.serine	0.078284331	0.058304327	0.181594522
c141.c51	0.042141464	0.031389564	0.181644198
citruline.c8	-0.067373862	0.050189261	0.181687851
pcaac386.pcaac323	0.110430613	0.082275055	0.181747147
pcaec382.lysopcac161	0.068256301	0.050854365	0.181753932
c8.pcaec340	0.062226582	0.046362824	0.18176238
pcaec386.prolinr	0.039564361	0.029480395	0.181797566
c2.pcaac361	-0.070931355	0.052856434	0.181828003
c0.asparticacid	0.035233459	0.026258098	0.181876368
pcaac386.c16	0.065787867	0.049035596	0.181934003

pcaec401.phenylalanine	0.077293181	0.057617406	0.181980862
c51dc.pcaac361	-0.09930065	0.074027965	0.182012054
pcaec406.smohc221	0.035399951	0.026396121	0.182105768
pcaac281.c181	-0.020566904	0.015339443	0.182209009
pcaec365.pcaec402	0.050937884	0.037994949	0.182253297
c18.c101	-0.004967096	0.003705572	0.182321068
pcaac342.pcaac322	0.119275055	0.089017412	0.182493778
histidine.pcaac426	-0.035105599	0.026203896	0.182557964
lysopcac181.pcaec444	-0.183747991	0.137169893	0.182604727
lysopcac204.pcaec362	-0.029277718	0.021856985	0.182621567
pcaec321.pcaec421	0.003306901	0.002469512	0.182758566
pcaac424.pcaec382	-0.067686033	0.050546732	0.18276271
pcaac281.c14	-0.044825827	0.033476788	0.182784174
pcaac380.prolinr	0.043259019	0.032306698	0.18278462
c3.pcaac344	-0.071900567	0.053704257	0.182845178
pcaec423.pcaac341	0.005479774	0.004093546	0.182905871
pcaec360.methionine	-0.062966534	0.047044318	0.182966346
creatinine.pcaac323	0.122919874	0.09184711	0.183012414
pcaec383.smc240	-0.069073864	0.051616513	0.183043989
pcaec322.pcaac403	-0.011127213	0.008315229	0.183056928
spermidine.c181	0.108447915	0.08104474	0.183072753
lysopcac280.pcaec382	0.012450665	0.009304576	0.183073195
c18.lysine	0.076612889	0.057258289	0.183105712
glutamine.asparticacid	0.063302378	0.047340045	0.183378368
c18.alphaaminoadipicacid	0.00626282	0.004683658	0.183384988
pcaec362.c102	0.055516999	0.041521297	0.183414747
smc160.citruline	-0.020085352	0.015022026	0.183418886
pcaec445.glutamine	0.040711706	0.030450217	0.183440963
smc181.kynurenine	-0.005862018	0.004385636	0.183555605
pcaec362.c14	-0.054433061	0.040733911	0.183663793
smohc222.c142	0.051033199	0.038191739	0.183687148
smc241.serotonin	-0.101663653	0.076097032	0.183772762
pcaac365.pcaec301	-0.126743063	0.094883026	0.183835272
asparagine.smc202	0.074877286	0.056061448	0.183885314
pcaac386.putrescine	-0.043838955	0.032827556	0.183949669
pcaac365.pcaec445	0.074778717	0.056000239	0.183983291
pcaec343.pcaec424	0.01430191	0.010711344	0.184021472
pcaac422.pcaac361	0.077139241	0.057773776	0.184026951
lysopcac181.asymmetricdimethy	0.032471492	0.024321428	0.184058496
pcaac342.serotonin	-0.163901778	0.122769725	0.184079362
pcaec446.pcaec443	-0.046279115	0.034670501	0.184147062
lysopcac181.lysopcac170	0.074295646	0.055660116	0.184152748
pcaec340.lysopcac161	0.096231727	0.072095218	0.184160212

pcaec381.pcaec404	0.034579966	0.025909212	0.184201833
lysopcac140.pcaac385	0.026298209	0.019704913	0.184220601
asparagine.pcaec383	-0.034108731	0.025557283	0.184221367
c51.pcaec406	-0.01598236	0.011975538	0.184226389
pcaac422.histidine	-0.123362746	0.092443314	0.184263894
putrescine.c181	-0.020826321	0.015606544	0.184266596
pcaec363.tyrosine	-0.07384466	0.055341362	0.18430332
pcaac363.smc181	-0.023825652	0.017856291	0.184319074
serine.pcaac405	-0.078271777	0.058665341	0.184349153
c4.pcaec403	-0.124101451	0.093015321	0.184350383
pcaac383.smc240	-0.05531721	0.041461642	0.184359015
pcaac405.pcaec382	0.006395845	0.004794236	0.184394376
pcaec361.pcaac366	0.048542598	0.036390524	0.184438403
pcaac406.c51	0.093270363	0.069930054	0.184493536
pcaac240.pcaac322	0.098261825	0.073676889	0.184519911
pcaac384.kynurenine	-0.008714329	0.006534253	0.18453551
pcaec386.pcaac380	0.231277084	0.173469148	0.184663729
pcaec382.pcaac405	-0.078084594	0.058570501	0.184687779
pcaec405.lysopcac180	-0.101975646	0.076491921	0.184693322
pcaec321.c18	0.03670646	0.027536022	0.18473309
lysopcac280.c101	-0.023166062	0.017378649	0.184738213
pcaec300.alphaaminoadipicacid	-0.007291795	0.005471152	0.184819161
pcaec364.pcaac381	0.058192537	0.043667459	0.184865663
pcaec406.lysopcac182	-0.025066031	0.018811352	0.184909696
pcaac363.pcaec423	-0.007821698	0.005870318	0.184935959
asymmetricdimethylarginine.pca	-0.077519921	0.058180633	0.184940524
pcaac321.c2	-0.138778478	0.104175943	0.185021133
c0.smohc222	-0.015113716	0.011345607	0.185032335
pcaec343.pcaac406	-0.062677678	0.0470526	0.185046665
pcaac321.smohc141	-0.039676473	0.029789959	0.185113132
acetylornithoine.asparagine	-0.014170458	0.0106408	0.185166977
pcaac420.histidine	-0.09587058	0.071999131	0.185218762
pcaac342.pcaec404	-0.021280249	0.015988462	0.185407909
lysopcac180.c181	0.055962045	0.042046131	0.185410414
pcaac363.lysopcac204	-0.023189403	0.017424688	0.185453626
c141.pcaac341	-0.014308102	0.010752139	0.18549128
pcaec362.smc202	0.096908888	0.072835967	0.185561127
methioninesulfoxide.pcaec420	0.124055319	0.093239786	0.185564744
pcaec343.smohc222	-0.007010833	0.005269548	0.185582736
smc161.pcaac360	0.062142348	0.046712432	0.185624047
pcaac366.pcaec382	0.022511168	0.016922841	0.185654664
asymmetricdimethylarginine.lyso	-0.03281483	0.024671077	0.185697641
c16.pcaac426	0.096182623	0.07233188	0.18581357

pcaac426.pcaec446	-0.108814589	0.08183648	0.185840425
pcaec443.pcaec342	0.129471974	0.097373758	0.185846813
pcaec444.smohc241	0.119590419	0.089943219	0.185852733
pcaac240.pcaac385	-0.057576895	0.043307277	0.185893598
prolinr.pcaec424	-0.025865636	0.019456873	0.18593108
leucine.pcaec423	0.02475775	0.018625429	0.185976623
pcaac405.lysopcac280	0.006373658	0.004795429	0.186020536
pcaec386.pcaec342	0.24377087	0.183428525	0.186067211
lysopcac161.pcaec445	0.16932867	0.12741512	0.186072652
pcaac380.c51	0.079840896	0.060079626	0.186084084
pcaec385.pcaec404	-0.013031683	0.009808046	0.186164826
pcaec343.pcaac381	0.054992824	0.041390949	0.186182319
kynurenine.pcaac403	0.025423567	0.019136343	0.186205576
c182.methionine	-0.049579598	0.037321494	0.186239383
c12.pcaac300	0.088140252	0.066352039	0.186263526
c141oh.lysopcac280	0.018467614	0.013903955	0.186311466
phenylalanine.pcaec382	-0.052505971	0.039531182	0.186315027
lysopcac182.transhydroxyprolin	-0.048227498	0.036310398	0.186320234
asparagine.kynurenine	-0.014788035	0.011134358	0.186338839
pcaec340.c51	0.052600021	0.039604777	0.186345797
pcaec321.glutamicacid	0.07645315	0.057565598	0.186351833
creatinine.tyrosine	0.094943964	0.071492454	0.186377227
c142.smc160	-0.089697079	0.067548563	0.186422536
alanine.pcaec422	-0.0325813	0.02453771	0.186450557
c18.c2	0.099001219	0.074560257	0.186451798
pcaac422.creatinine	-0.016618533	0.012515881	0.186453694
pcaac363.pcaec342	0.182266698	0.137272708	0.186461898
acetylornithoine.pcaec443	-0.053730636	0.04047193	0.186517382
histidine.pcaec363	0.018098991	0.013633873	0.186550587
serotonin.pcaec362	0.049421595	0.037245768	0.186747448
pcaac405.pcaec365	0.049661405	0.037427541	0.186759661
pcaac365.valine	0.096556217	0.072786113	0.186856451
pcaac383.smohc222	-0.008815186	0.006645176	0.186863241
isoleucine.c101	0.010586661	0.007981242	0.186900007
pcaac344.kynurenine	-0.012495739	0.009420611	0.186905695
lysopcac280.alanine	-0.024477036	0.018455547	0.186957106
c141.lysine	0.066442095	0.050102617	0.187006524
c10.pcaac403	0.005524029	0.004165595	0.187010574
pcaec406.asparagine	-0.022455184	0.01693541	0.187069064
lysopcac280.pcaec423	-0.005890165	0.00444241	0.187081245
pcaac406.smohc241	0.068882076	0.05195215	0.187087429
citruline.pcaec406	0.054341916	0.040988332	0.187115679
phenylalanine.pcaac422	-0.025246324	0.019046439	0.187206808

c141.c3	0.089832947	0.067778887	0.187250356
c181.kynurenine	-0.013626617	0.010281551	0.187262267
c51dc.pcaac422	0.025612269	0.019331264	0.187405161
pcaec403.lysopcac280	-0.011614132	0.00876883	0.187549226
prolinr.asparagine	0.03185029	0.024049665	0.187590342
c51dc.c51	0.053037061	0.040047678	0.18759263
pcaec342.pcaac380	0.160756806	0.121404068	0.187659247
pcaac385.pcaec382	0.009193995	0.006944169	0.187711891
c18.pcaac361	-0.077845487	0.05880263	0.187759667
c8.c162	-0.127262518	0.096145835	0.187826985
pcaac365.pcaec443	-0.068836312	0.052019063	0.187943117
smc161.pcaec443	-0.074234031	0.056098386	0.187945535
smc241.smc161	0.15891675	0.120107851	0.188000492
pcaac360.kynurenine	-0.015152623	0.011453818	0.188061841
pcaec360.pcaec423	-0.006597134	0.00498757	0.188133825
pcaec420.pcaec361	0.024169982	0.01827389	0.188155055
pcaac405.pcaec301	0.091990551	0.069551209	0.18816191
pcaec386.smc180	0.035615267	0.026930723	0.188212784
pcaac386.pcaec342	0.138828972	0.10503275	0.188448145
c141.alphaaminoadipicacid	0.014823609	0.011216056	0.188490169
pcaec363.lysopcac204	-0.030501552	0.023085322	0.188619418
pcaac424.serotonin	-0.146926674	0.111221054	0.18869261
asparagine.smc180	0.116177872	0.087949802	0.188717986
lysopcac280.c141oh	-0.131093501	0.099242695	0.188723996
pcaec341.smohc221	0.063210628	0.047857655	0.188768426
c141oh.c182	-0.062636488	0.047427424	0.188809729
pcaec321.lysopcac181	0.061112765	0.046275633	0.188828245
pcaac380.c16	0.072342746	0.054783576	0.188863615
pcaec445.pcaec422	0.062052875	0.046994166	0.188890571
pcaac384.pcaec421	0.001358446	0.001029212	0.189073117
c101.phenylalanine	0.068808242	0.052132354	0.189077628
c3.pcaec423	0.025179582	0.019083705	0.189226754
c10.pcaac406	0.06785501	0.051431513	0.189260364
pcaac381.methioninesulfoxide	0.094733841	0.071815545	0.189327119
pcaac321.pcaec423	-0.014673634	0.011124901	0.18937282
c142.pcaec424	-0.015274665	0.011582434	0.18944339
pcaac404.asparticacid	0.046206544	0.035041614	0.189496552
methioninesulfoxide.smohc222	-0.017268824	0.013096257	0.189500403
pcaec386.lysopcac181	0.032010041	0.024283927	0.189650599
pcaec365.c51dc	-0.164025645	0.124444858	0.189683541
lysopcac280.pcaac341	-0.011883933	0.009016359	0.189689406
pcaac406.transhydroxyproline	0.043906971	0.033317328	0.189755973
c14.pcaec443	-0.096821397	0.073476766	0.189798535

pcaac281.smc181	-0.060439047	0.045867897	0.189811241
acetylornithoine.putrescine	-0.036889923	0.027996672	0.18981878
serotonin.pcaac365	-0.044098519	0.033467985	0.189825762
pcaec380.pcaac341	0.010338835	0.00784672	0.189836909
pcaec341.pcaac322	-0.110798561	0.084098457	0.189874766
asymmetricdimethylarginine.seri	0.018343013	0.013922945	0.18988138
pcaac343.pcaec360	-0.009292916	0.007054181	0.189916003
pcaac361.pcaec423	-0.005905683	0.00448357	0.189975987
pcaac300.lysopcac170	0.02664002	0.020227666	0.190034233
pcaac383.pcaec363	-0.053947817	0.040962991	0.190040877
pcaac404.smc240	-0.034382704	0.026107184	0.190043113
smc260.asparagine	-0.029130877	0.02211955	0.190045902
pcaac425.alanine	-0.021590254	0.016394705	0.190069329
pcaac384.creatinine	0.012698252	0.009642926	0.190088656
pcaac281.alanine	-0.037824448	0.028723747	0.190092294
pcaec386.pcaec421	0.001700783	0.001291584	0.190097813
smc161.pcaac362	-0.031367549	0.02382454	0.19016879
pcaec402.pcaec361	0.026388928	0.020047909	0.190273644
lysine.pcaec362	-0.015877976	0.012063266	0.190296371
pcaac365.pcaac383	0.014828696	0.011269372	0.190425188
pcaac342.pcaec445	0.077869199	0.059192509	0.190530994
pcaac366.pcaec364	-0.015898293	0.012085272	0.190535954
pcaac323.pcaec424	-0.040631323	0.030886895	0.190543424
lysopcac180.smc181	-0.017504788	0.013312087	0.190722025
asparagine.c182	-0.098902623	0.075219591	0.19075639
pcaac383.pcaac381	0.061554504	0.046817342	0.190780506
citruline.c51	0.046435749	0.035318343	0.190781467
smohc161.smc181	0.051376273	0.039097371	0.191022176
c51dc.smohc141	-0.035854649	0.02728703	0.191048399
c4.pcaec365	-0.057611368	0.043854376	0.191143814
pcaec420.creatinine	-0.023058911	0.017553023	0.191152327
smc240.lysopcac182	-0.074711425	0.056874259	0.19116818
lysopcac203.c5	0.030001293	0.022839041	0.19117748
pcaac323.c5	0.029241004	0.022261148	0.191195136
serine.pcaac281	0.005123635	0.003901338	0.191276507
glutamicacid.c5	-0.02416873	0.018404939	0.191322386
c162.pcaec406	-0.039417071	0.030018	0.191339511
serotonin.smohc241	-0.048615307	0.037027724	0.191396997
pcaec300.pcaac420	-0.128187217	0.097639633	0.191424876
c182.pcaec406	-0.011196956	0.008529229	0.19145371
smohc141.prolinr	0.048250733	0.036754896	0.191455222
pcaec386.smc181	0.038708757	0.029493037	0.191555807
pcaac240.alanine	-0.035627151	0.027146449	0.191577824

histidine.pcaac402	-0.072453692	0.055208553	0.19159197
pcaec445.acetylornithoine	-0.102470615	0.078087605	0.191629486
c14.asparticacid	0.050471278	0.03846444	0.191662365
pcaac281.pcaac365	-0.061420942	0.046815766	0.191723915
pcaec382.asparagine	0.023927245	0.018238707	0.191750042
c51dc.prolinr	0.037600534	0.0286619	0.191759805
smohc161.pcaec342	-0.168060156	0.128108586	0.191762361
pcaec381.pcaec321	0.062358392	0.047538869	0.191803496
pcaac384.c142	-0.054857483	0.04182273	0.19182644
pcaec446.kynurenine	0.014036155	0.010703176	0.191915916
pcaac402.pcaac405	-0.093624787	0.071404451	0.191987044
lysopcac280.asparagine	0.013402926	0.010222167	0.191996115
asparagine.pcaec402	-0.04634517	0.035352785	0.192073204
pcaec301.lysopcac170	0.014645459	0.011172144	0.192087822
glutamine.pcaac403	-0.010021652	0.007645818	0.192139843
alanine.c8	-0.053248719	0.040627869	0.192170574
smc180.smohc222	-0.008622069	0.006579611	0.192245693
c8.smohc141	-0.084428142	0.064430634	0.192262433
pcaec341.pcaac364	-0.062398104	0.04762317	0.192304901
smc240.lysopcac180	0.066911043	0.051080784	0.192419735
pcaac362.pcaec423	-0.00545722	0.004166118	0.19242017
lysopcac204.valine	-0.101997594	0.077870303	0.19244231
pcaac386.pcaac420	-0.084849212	0.064788042	0.19250857
asymmetricdimethylarginine.c14	-0.052912556	0.040413231	0.192628475
c0.pcaac421	-0.066588993	0.050859997	0.192637689
c182.pcaec424	-0.01578943	0.012062142	0.192723311
c181.alanine	0.043148324	0.032969179	0.192811104
pcaac360.pcaac380	0.182558367	0.139510043	0.192871734
pcaec403.pcaec365	0.035480422	0.027116325	0.192910774
leucine.lysopcac161	-0.129060474	0.098640257	0.192930002
ornithine.pcaec424	-0.006883748	0.005261523	0.19295601
creatinine.citruline	0.025362255	0.019389413	0.193048013
pcaec300.citruline	0.05218032	0.039894879	0.193082263
lysopcac182.pcaec424	0.017540346	0.013411633	0.193115989
smohc141.alanine	-0.05237412	0.040047667	0.193133274
c3.c51	0.038376473	0.029344519	0.193134755
putrescine.lysopcac181	0.043700925	0.033427554	0.193289668
glutamicacid.c101	0.021623934	0.016541361	0.193312656
pcaac362.c142	0.069596076	0.053239167	0.193322825
pcaec444.pcaac403	-0.07040755	0.053869917	0.193404955
c14.pcaec383	0.092119627	0.070482812	0.193409009
pcaac364.smc202	0.057131244	0.043714264	0.193427801
c4.smohc161	-0.026442175	0.020235289	0.193491754

pcaec446.alphaaminoadipicacid	0.008880178	0.006795761	0.193496016
pcaac300.pcaac402	-0.051446363	0.039376048	0.193558246
pcaec386.smc202	0.053110867	0.040652504	0.193585225
lysopcac181.pcaac402	0.07561074	0.057876838	0.193602966
histidine.pcaec444	-0.1679096	0.128548718	0.193675276
c162.pcaec443	-0.088162323	0.067500141	0.193705287
pcaac344.tyrosine	0.085788713	0.065683619	0.193710659
pcaac405.pcaac281	0.003567705	0.002731841	0.193750931
pcaec380.alanine	-0.02039746	0.015619054	0.193763396
pcaec363.pcaec342	0.300541618	0.230175668	0.193841075
ornithine.glutamicacid	-0.06907167	0.052902596	0.193863756
pcaec446.pcaec385	-0.123103334	0.094288147	0.193874223
pcaac342.pcaec423	-0.007292247	0.005587719	0.194063853
smc202.pcaac300	0.062129294	0.047623059	0.194214577
lysopcac204.smc240	-0.057691163	0.04423079	0.194311069
c3.pcaac341	0.006589517	0.005052982	0.194391489
pcaec382.pcaac341	0.016270546	0.012478801	0.194469646
alanine.asparticacid	-0.064854966	0.049741154	0.194471567
histidine.transhydroxyproline	-0.058096632	0.044564126	0.194534691
pcaec402.lysopcac161	0.064481466	0.049466558	0.194577935
pcaac320.smohc241	0.079168178	0.060733977	0.194582307
smc202.pcaac426	-0.079666983	0.061123963	0.194635484
pcaec402.smc202	0.151483886	0.116231345	0.194659675
smc240.pcaec340	-0.044582348	0.034211704	0.194715796
lysopcac180.lysopcac181	-0.147164648	0.112948353	0.194781815
pcaac420.transhydroxyproline	0.082942427	0.063671501	0.194875738
pcaec300.c182	-0.039999719	0.030710859	0.194943863
pcaac281.pcaec341	0.182892408	0.140421221	0.19494595
pcaac366.alanine	-0.021429219	0.016456774	0.195049477
pcaac342.smohc161	0.031691209	0.02433772	0.195052245
smohc241.putrescine	-0.052988223	0.040695029	0.195073625
pcaac366.pcaac281	-0.005321909	0.004087925	0.195148672
lysopcac181.lysopcac161	-0.221693352	0.170305256	0.195189497
pcaec406.lysopcac203	-0.022941255	0.01762354	0.195190106
pcaac386.pcaec361	-0.017972593	0.013812859	0.195391347
c141oh.pcaec301	-0.052391307	0.040265751	0.195395176
pcaec421.pcaec382	0.036457992	0.028020664	0.195404493
glutamine.pcaec380	-0.074520023	0.057279849	0.195448664
pcaac426.pcaec342	0.138725002	0.106731673	0.195867734
c162.c51	0.105638841	0.081287215	0.195929169
pcaec385.c8	-0.088843748	0.06837137	0.195979103
c101.acetylornithoine	-0.099456591	0.076541111	0.195993206
glycine.pcaac360	-0.071044985	0.054676512	0.195999539

pcaac421.pcaec361	-0.02754414	0.021198135	0.196000614
pcaac424.lysopcac280	-0.017866102	0.013751243	0.196045351
pcaec343.pcaec301	-0.069940487	0.05383423	0.196063475
pcaec363.alphaaminoadipicacid	0.029308905	0.022567678	0.196224643
ornithine.pcaec300	0.028583161	0.022015045	0.196349589
pcaec403.lysopcac161	0.07609062	0.058614574	0.196416316
pcaec446.pcaec360	-0.014835388	0.011429408	0.196467862
smc160.prolinr	0.049080517	0.037812656	0.19647117
serine.tyrosine	-0.076343366	0.058818774	0.19648818
putrescine.kynurenine	0.013332406	0.010272999	0.19653329
glycine.c51	0.083803834	0.064573929	0.196538025
pcaec445.pcaec402	-0.039154231	0.030170874	0.196554274
pcaec405.c18	0.066869342	0.051533965	0.196612988
pcaec301.c51dc	0.147346153	0.113567302	0.196662158
pcaac403.alanine	-0.02177176	0.016783379	0.196735249
glycine.pcaac403	-0.007263632	0.005599797	0.196768683
pcaac383.h1	0.004763872	0.003672963	0.196807599
pcaec421.creatinine	-0.022327991	0.017217877	0.196882923
pcaec340.smohc221	-0.101872	0.078601632	0.197135856
pcaac406.pcaec423	-0.004177703	0.003223697	0.197176726
methionine.pcaac362	-0.035979441	0.027763814	0.197185045
pcaec424.c141	0.035389548	0.027309912	0.197206138
asparagine.valine	0.070011582	0.05402869	0.197215634
pcaec340.lysopcac204	-0.069136081	0.053357891	0.197255984
lysopcac203.lysopcac182	0.043789855	0.033797645	0.197275503
lysopcac182.asparticacid	0.027471369	0.021204356	0.197308166
putrescine.smc180	0.058712689	0.045321682	0.197338179
pcaac341.pcaec343	0.143893398	0.111089298	0.197396942
pcaec402.pcaac403	-0.015404741	0.011894495	0.197458763
pcaec382.lysopcac182	-0.0821153	0.063404556	0.197463635
pcaac386.lysine	0.125286098	0.096747075	0.197503209
pcaec340.smc240	-0.040388317	0.031188505	0.19750715
pcaec444.pcaec360	-0.018597419	0.014362413	0.197544024
pcaac386.pcaec420	-0.079198237	0.061163264	0.197544425
pcaec405.glutamicacid	0.098073815	0.075740998	0.197547309
pcaac320.asparticacid	0.081502367	0.062960389	0.197669628
pcaec322.pcaac240	-0.023041689	0.017799827	0.197674043
pcaac386.transhydroxyproline	0.034704367	0.026811156	0.197704762
c8.pcaac323	-0.180917786	0.139779187	0.197735364
pcaec403.c5	-0.045313252	0.035015724	0.197814
pcaac300.pcaac420	-0.1342782	0.103768587	0.197837103
c141.pcaec423	-0.011324445	0.008752118	0.1978739
smohc222.pcaec401	0.106764195	0.082516113	0.19789123

pcaac402.smohc221	-0.112542444	0.086982379	0.197893088
methioninesulfoxide.alanine	-0.047220388	0.036496371	0.197898412
lysopcac281.pcaec300	-0.087562217	0.067687676	0.197973065
leucine.c101	0.011105062	0.008584967	0.197998472
pcaec405.pcaac381	0.052042545	0.040251594	0.198210933
c141oh.pcaec423	-0.014522546	0.01123281	0.198232608
asparagine.pcaec424	0.016030324	0.012399386	0.198245153
lysopcac204.pcaec360	-0.00829869	0.006419502	0.198280012
kynurenine.pcaac362	-0.022052808	0.017061098	0.198332653
smohc141.pcaac403	-0.011828102	0.009151131	0.198349722
pcaac383.pcaac425	-0.137595998	0.106456358	0.198355991
pcaac402.ornithine	-0.079317547	0.061372846	0.198398299
c8.pcaac403	0.004187025	0.003239985	0.198429691
serine.lysopcac203	0.014091257	0.010906938	0.198548387
lysine.pcaac403	-0.012628259	0.009775708	0.198601454
pcaac402.lysopcac161	0.104580711	0.080981305	0.198733421
pcaec446.pcaec424	0.020768239	0.016082771	0.198762211
pcaec363.lysopcac161	0.09029819	0.069937331	0.198832932
pcaac420.c16	0.047978209	0.037160148	0.198836422
pcaac360.glutamicacid	0.09092092	0.07042241	0.198850458
pcaec321.c51	0.077512949	0.060041547	0.198881861
pcaac361.asparticacid	0.032285088	0.025013333	0.198976413
pcaac344.pcaec362	-0.050553429	0.039167319	0.19898014
lysopcac281.pcaec423	-0.006301639	0.004882902	0.199032811
pcaac421.lysopcac180	-0.094891606	0.073535818	0.199080798
lysopcac160.c2	0.123580285	0.095778319	0.19912901
pcaac405.pcaec446	-0.063028909	0.048855513	0.199186352
pcaec342.c5	0.025935112	0.020103523	0.199196913
glutamine.pcaec424	0.026579383	0.020603457	0.199208384
histidine.pcaac360	-0.036516261	0.028311249	0.199288103
pcaec384.pcaec421	0.001390461	0.001078034	0.199288996
pcaec422.pcaac361	0.082677396	0.064109222	0.199350836
pcaec444.c142	0.059153367	0.045870635	0.199373017
pcaec383.c141	0.054033444	0.041903521	0.199406534
lysopcac280.pcaec446	0.123766036	0.095982203	0.19940794
pcaac386.pcaec423	-0.004809397	0.003729869	0.199421975
pcaac422.pcaec402	-0.080945615	0.06278282	0.19946787
pcaec424.pcaac385	-0.060140898	0.046650079	0.199503862
pcaac381.lysine	0.125142728	0.097079426	0.199544095
pcaec403.pcaec380	0.110279826	0.085559693	0.199597144
lysine.ornithine	0.080758407	0.062660581	0.199631642
pcaac323.pcaac344	0.069513138	0.053946586	0.199724669
smc160.pcaac322	-0.074087562	0.057508964	0.199820564

pcaec365.pcaec443	-0.067624614	0.052502274	0.199906076
pcaec401.lysopcac160	0.045947913	0.035681784	0.200016697
c51.asparticacid	0.025201738	0.019571177	0.200022538
c16.citruline	0.055726429	0.043284392	0.200108686
pcaac360.pcaec421	0.003130941	0.002432068	0.200140289
pcaec360.c141oh	-0.151544112	0.117719223	0.200148033
pcaac406.c18	-0.051301174	0.039853743	0.200182523
pcaac383.putrescine	-0.032984455	0.025625141	0.200198128
lysopcac181.c51	-0.08267488	0.064233198	0.200228002
lysopcac140.lysine	-0.135631022	0.105397812	0.200317181
histidine.glycine	0.048489831	0.037682272	0.200331443
pcaac365.pcaac420	-0.09501699	0.073840588	0.20033914
pcaec424.c182	-0.054099014	0.042042968	0.200349588
pcaec342.prolinr	0.027287613	0.021212339	0.200472182
smc240.c141	0.069436562	0.053984356	0.200530594
pcaec383.pcaec380	0.10634679	0.082701488	0.200643138
pcaec401.pcaac322	-0.100913362	0.078483276	0.200683852
lysopcac203.pcaec361	0.007785829	0.006055353	0.200690224
pcaac425.pcaec361	0.021109584	0.016417838	0.200691933
pcaec321.pcaac240	-0.03638929	0.028309408	0.200816559
c2.lysopcac182	-0.113780605	0.088520132	0.200833678
pcaac385.pcaec361	-0.013936285	0.01084349	0.20088344
pcaac425.pcaec340	0.076241212	0.059332264	0.200964923
lysopcac280.pcaac380	0.044501165	0.034632644	0.200978469
pcaac341.c141oh	-0.139742795	0.108759936	0.201004505
pcaac362.kynurenine	-0.009758319	0.007594857	0.201009739
pcaec421.alanine	-0.024212769	0.018850042	0.201136847
pcaac381.taurine	0.074769225	0.058210884	0.201150719
pcaec445.pcaac322	-0.087805535	0.068365441	0.201185174
pcaec425.pcaac403	-0.007699179	0.005995395	0.201245677
pcaec385.c51dc	-0.160803627	0.125225091	0.201268499
acetylornithoine.pcaac341	0.017799766	0.013863868	0.201345472
methioninesulfoxide.pcaec406	0.090229828	0.070290495	0.201424218
citruline.glutamine	0.034511378	0.026885172	0.201428403
c14.smohc222	0.018529792	0.014435833	0.20144983
c3.smc181	0.02938576	0.022901599	0.201612427
pcaac381.pcaec342	0.145456893	0.113374186	0.201665053
smc240.pcaec404	0.022493544	0.017534789	0.201729848
lysine.c51	0.055803758	0.043505583	0.201769982
putrescine.smohc221	-0.039030281	0.030431812	0.201816007
pcaec384.c141oh	-0.151721497	0.118331318	0.20194655
pcaac383.pcaec406	0.01240502	0.009675182	0.201955936
asparticacid.c51	-0.136131526	0.106208775	0.202100505

c14.pcaec421	0.005490496	0.004283905	0.202127914
pcaec383.pcaac405	-0.080595143	0.06288464	0.20213564
serotonin.pcaac321	-0.038465265	0.030015939	0.202184681
c2.pcaec402	-0.058998853	0.046041461	0.202207747
smc241.pcaec423	-0.03266201	0.025488757	0.202207967
pcaac381.smohc141	0.043102866	0.033637234	0.202216633
pcaac363.putrescine	-0.035304477	0.027556038	0.202291881
smc181.alphaaminoadipicacid	0.011998071	0.009365325	0.202317111
pcaec401.c8	-0.061528454	0.048030947	0.202351964
c18.pcaec362	0.064800072	0.050592477	0.20241943
pcaec343.putrescine	-0.045025135	0.035154058	0.202429745
pcaec381.pcaac405	-0.078844699	0.061560814	0.202441534
lysopcac170.lysopcac181	-0.131161768	0.102440476	0.202578412
pcaac386.serotonin	-0.150394014	0.117474584	0.202629089
ornithine.lysopcac182	-0.019402605	0.015156444	0.202653816
prolinr.pcaac422	-0.012977097	0.010137357	0.202664052
c2.phenylalanine	0.081154188	0.06339567	0.202665566
pcaac323.pcaec385	-0.067803314	0.052974095	0.202731679
smc240.ornithine	-0.044921256	0.035102035	0.202801726
asparticacid.pcaec420	0.062121025	0.048548191	0.202857589
citruline.pcaac383	0.02396204	0.018727896	0.202889395
c142.c3	0.066317551	0.051841841	0.202979063
pcaec382.lysopcac170	-0.066245195	0.051791293	0.203031213
creatinine.lysopcac181	-0.053150833	0.041560907	0.203106255
pcaac342.asparagine	0.009838234	0.00769294	0.203106523
prolinr.lysopcac161	-0.107130467	0.08377412	0.203128938
pcaec322.c101	-0.013759043	0.010760351	0.203171708
lysopcac281.alanine	-0.020155782	0.015763691	0.203192422
pcaec385.pcaec382	0.031952491	0.024990842	0.203210942
smohc221.glycine	0.080546888	0.063002804	0.203247086
prolinr.lysopcac170	-0.02891306	0.02261704	0.203278727
lysopcac161.glycine	-0.049095187	0.038406474	0.20330336
pcaec401.pcaac361	0.102011207	0.079805259	0.203322093
pcaac381.pcaec381	0.063220714	0.049465538	0.203383866
pcaac344.pcaac341	-0.012694257	0.009934584	0.203486324
pcaac401.leucine	-0.065336872	0.051139475	0.2035438
asparticacid.pcaec402	-0.168550073	0.131934603	0.203576755
pcaec425.pcaec362	0.016202607	0.01268285	0.203579005
pcaec384.pcaac381	0.05318706	0.041635095	0.203601389
methionine.pcaec363	0.03410882	0.026705488	0.20368444
lysopcac204.pcaac366	-0.029557429	0.023144232	0.20372822
isoleucine.pcaec404	0.03049539	0.023881971	0.203790179
glutamine.pcaac281	0.001974885	0.001546859	0.203865537

arginine.creatinine	-0.019286921	0.01510728	0.203880641
pcaac361.pcaac364	-0.079502774	0.062290286	0.203999362
pcaac404.smohc222	-0.007767603	0.006087482	0.20411594
pcaec444.c141	0.023918554	0.018745569	0.204129487
pcaec360.c101	-0.018002181	0.014109779	0.204161992
pcaec423.c142	0.061291423	0.048047996	0.204245342
pcaac365.acetylornithoine	-0.138804812	0.108821978	0.204283169
c162.putrescine	-0.0219063	0.017175154	0.204303381
glutamine.smohc161	-0.011436086	0.008966695	0.204327583
pcaec403.pcaec404	0.067491405	0.0529246	0.204383673
pcaec321.pcaec404	-0.013560611	0.010634727	0.204423001
pcaec322.pcaac341	0.010463162	0.008206847	0.204491704
pcaac401.lysopcac182	-0.111357089	0.08735674	0.204559217
pcaec406.lysine	0.13115374	0.102891095	0.204578452
c182.alphaaminoadipicacid	0.005923124	0.004646953	0.204599512
pcaac281.pcaac406	-0.048258364	0.037861323	0.204605482
lysopcac170.pcaec340	-0.072666872	0.057013612	0.204625063
c14.pcaec401	-0.050924276	0.039958959	0.204673994
smc202.c51dc	-0.175546748	0.137753537	0.204695412
pcaac281.pcaac403	-0.0128328	0.010073133	0.204833451
lysopcac203.asparticacid	0.035215744	0.027643732	0.204850721
putrescine.c18	-0.033194782	0.026057892	0.204860722
acetylornithoine.glycine	0.048580233	0.038140613	0.204921489
glutamine.pcaac341	-0.004493424	0.003527894	0.204931917
ornithine.pcaac281	0.005615103	0.004410688	0.205150175
pcaac343.pcaac420	-0.152277607	0.119626863	0.205195853
pcaec364.glycine	0.056221273	0.044178631	0.20531918
lysopcac140.pcaec404	0.030150713	0.023692951	0.205329543
pcaec322.pcaec443	-0.06457837	0.050747166	0.205332808
pcaec363.pcaac341	0.028715221	0.022565695	0.20534508
pcaac323.lysopcac280	0.013585564	0.010676339	0.205353505
kynurenine.pcaac402	0.093745719	0.07367498	0.20537831
pcaac403.pcaec342	0.127525132	0.100229531	0.205410815
smc241.c16	0.05370386	0.042210764	0.205429278
pcaac384.smohc141	-0.054213449	0.042612231	0.20543916
pcaec343.arginine	-0.067310723	0.052930104	0.205637599
methionine.c101	0.013513576	0.010626844	0.205653739
serotonin.pcaac383	-0.006172864	0.004854802	0.205706471
pcaac361.kynurenine	-0.00761719	0.005991192	0.205741343
lysopcac204.creatinine	0.020492853	0.016120446	0.205799654
pcaec364.pcaac364	-0.08986297	0.070702461	0.205881788
lysopcac160.smc202	0.053511059	0.042106435	0.205934897
smohc221.putrescine	-0.040703064	0.032028485	0.205939438

smc202.pcaec362	0.051697737	0.040680473	0.205944808
c10.methioninesulfoxide	0.021092342	0.016598127	0.205965371
acetylornithoine.asymmetricdime	0.034999412	0.027542988	0.205982019
smohc222.pcaac403	-0.007985902	0.006287139	0.206167666
histidine.smc240	-0.095651467	0.075309294	0.206196578
pcaac323.pcaac366	-0.056274485	0.044309658	0.206227664
smohc141.smc260	0.058053902	0.045712008	0.206240136
smohc241.smc240	0.046472829	0.036595238	0.206267742
pcaec445.asymmetricdimethylarg	0.028218604	0.022224431	0.206340082
phenylalanine.pcaac403	-0.013193939	0.010391901	0.206366492
pcaac406.pcaec365	-0.032719973	0.025771993	0.206381594
pcaec446.c141	0.033776786	0.026606545	0.206418044
methionine.c3	-0.120567995	0.094982914	0.206463014
c2.citruline	0.031283321	0.024646025	0.206484398
h1.pcaac405	-0.110917553	0.087385453	0.206489413
methionine.smohc141	-0.065679126	0.051747281	0.206511551
asymmetricdimethylarginine.glyc	-0.07839354	0.061768936	0.206542383
c8.c3	0.095355346	0.075135154	0.206550997
pcaec446.smc240	-0.114745652	0.090414574	0.206555225
pcaac425.smc240	-0.036749864	0.028959072	0.206582957
pcaac426.pcaec364	-0.025478662	0.020084652	0.206747965
lysopcac281.lysopcac181	0.039179418	0.030885243	0.206753531
pcaec420.pcaec423	-0.019088863	0.015052955	0.20690806
smc241.pcaec300	-0.030792875	0.024283862	0.206934884
pcaec384.transhydroxyproline	0.040643498	0.032059304	0.207034159
pcaec365.smc181	0.035498992	0.028002701	0.207055878
pcaec322.asparagine	0.014632933	0.01154465	0.207123965
c51dc.c141	0.036041601	0.028435019	0.207124029
pcaac361.pcaac405	0.11338968	0.089461925	0.207139898
lysopcac170.putrescine	-0.068186042	0.053800254	0.207164998
pcaac320.pcaec421	0.002639668	0.0020832	0.207261664
pcaac381.lysopcac161	0.084521678	0.066705648	0.207274868
asparagine.c142	0.045682288	0.036054135	0.207288109
pcaac361.putrescine	-0.033213935	0.026219507	0.207388952
pcaec322.pcaac384	0.127206091	0.100425917	0.207424021
pcaec300.pcaec423	-0.010671684	0.008426697	0.207514008
serine.pcaec423	-0.010102266	0.007977086	0.207515121
smohc161.pcaac322	-0.083288552	0.06577496	0.207566938
pcaac321.leucine	-0.012151959	0.00959769	0.207613903
smc261.pcaec343	-0.081838063	0.064642094	0.207655165
pcaac386.glutamicacid	0.049483008	0.039096823	0.20778556
pcaac405.kynurenine	-0.011575523	0.009146311	0.207806318
pcaec402.c142	0.053015114	0.041893476	0.207849375

prolinr.pcaac360	-0.027691376	0.021883331	0.207872483
phenylalanine.lysopcac280	-0.031923786	0.02522885	0.207887225
c10.pcaac380	-0.069337348	0.05480346	0.207947275
pcaac281.pcaec443	-0.067699228	0.053511023	0.207966832
pcaec382.pcaec342	0.108154659	0.085508951	0.208078022
pcaac404.pcaac360	0.041731528	0.032997983	0.208137121
pcaec361.asparagine	0.013014789	0.010292343	0.208193342
pcaec421.pcaac361	0.077453305	0.0612577	0.208238767
pcaac281.c141oh	-0.137760674	0.10899367	0.208400488
c14.smohc141	-0.034943217	0.027648847	0.208440195
glutamicacid.c51	-0.08434226	0.066742123	0.20848253
lysopcac140.smohc221	-0.056304098	0.044560525	0.208540351
pcaec322.tyrosine	-0.070043006	0.055434588	0.208546354
pcaec343.pcaac341	0.006967392	0.005514437	0.208561917
c142.c2	0.076275795	0.060372553	0.208584636
c162.valine	-0.078810965	0.062383681	0.208617504
h1.pcaec401	0.098196408	0.077731956	0.208637971
pcaec424.pcaac361	0.059316448	0.046956397	0.208654266
c16.smohc161	-0.046221573	0.036608166	0.208876863
pcaec360.pcaec363	-0.043198884	0.034216581	0.208909002
lysopcac182.pcaac385	0.040097184	0.031764827	0.208980435
pcaac323.lysopcac181	0.049460924	0.039183906	0.208993857
methionine.pcaac403	-0.006282182	0.004976902	0.208997083
pcaac425.asymmetricdimethylar[illegible]	0.036078656	0.0285841	0.209023743
pcaac341.pcaec423	-0.006969921	0.005522293	0.209041872
spermidine.lysopcac203	0.140358958	0.111212871	0.209066219
pcaac362.pcaec404	-0.017341896	0.013741448	0.20908813
c2.arginine	0.086525657	0.068565309	0.20911273
pcaec443.pcaec402	-0.135149398	0.107097889	0.209120302
c162.pcaac323	-0.092045059	0.07294157	0.209128463
pcaac426.pcaec423	-0.007296306	0.005782414	0.209161334
pcaac322.pcaac341	-0.008424274	0.006676623	0.209180376
pcaac422.serotonin	-0.140644417	0.111482205	0.209241513
smohc161.smohc222	-0.010372488	0.008222788	0.209296857
serine.pcaac403	-0.008559253	0.006785957	0.209337647
lysopcac204.lysopcac180	-0.127762197	0.101316309	0.209443752
pcaac360.c101	-0.032209584	0.025549866	0.20957572
pcaac362.smohc221	0.029789866	0.02363051	0.209576814
pcaac323.smc160	-0.125779351	0.099775611	0.209587752
pcaec342.asparticacid	0.060786955	0.048221387	0.209602627
glycine.pcaec443	-0.078441131	0.062234721	0.2096648
pcaac344.pcaec423	-0.010764552	0.008540721	0.209674899
pcaac384.pcaec382	0.006930781	0.005499638	0.209730533

leucine.pcaac341	0.007607767	0.006037093	0.209750078
h1.c4	0.093461472	0.074189238	0.209893717
c14.pcaec423	-0.016151436	0.012821759	0.209923111
alanine.pcaac341	-0.00579485	0.00460056	0.209956614
isoleucine.smohc141	-0.021682582	0.017214374	0.209968801
c51.putrescine	-0.023881568	0.018961919	0.210009724
pcaac380.c102	0.046005306	0.036540081	0.21015808
c18.smc181	0.03784016	0.030055906	0.210174039
c51dc.lysopcac203	0.021081489	0.016745273	0.210188744
smc202.pcaac384	0.057409589	0.045606281	0.210240172
pcaec424.pcaec383	-0.113692511	0.090319011	0.21024758
c8.pcaec342	0.093808087	0.074526016	0.210268786
pcaac404.c12	0.068728287	0.054605022	0.210299528
phenylalanine.pcaac281	0.003219181	0.002558206	0.210396684
pcaec423.leucine	-0.020973899	0.016671956	0.210519163
pcaec446.pcaac406	-0.05075071	0.040348087	0.210595863
putrescine.pcaec423	-0.006570569	0.005224409	0.210651592
c182.pcaac405	0.081355284	0.064709192	0.210804139
h1.pcaec342	0.159519773	0.126907159	0.210899624
asparagine.pcaec404	0.028308498	0.022522651	0.210932112
leucine.smohc241	0.063445585	0.050486939	0.211010424
citruline.pcaac343	-0.034659494	0.02758116	0.211023674
pcaec422.pcaec401	0.123930295	0.098634397	0.211087007
c141.glycine	-0.043642699	0.03473519	0.211094501
pcaec444.phenylalanine	0.069931603	0.055680221	0.21127156
pcaac361.pcaac420	-0.078599	0.062589041	0.211327813
lysopcac204.pcaec443	0.111249294	0.088589739	0.211332915
isoleucine.smohc241	0.071086616	0.056615293	0.211395392
pcaec365.h1	0.002319343	0.001847197	0.211397809
pcaec382.pcaec360	-0.010831032	0.008626489	0.21141453
pcaec343.leucine	-0.016297266	0.012981688	0.211469084
asparticacid.c51dc	0.180873912	0.144088129	0.211506598
lysopcac180.lysopcac182	0.080698239	0.064287038	0.211514007
pcaac322.c18	0.060116052	0.047891656	0.211524515
ornithine.pcaec422	-0.038821904	0.030930091	0.211561074
smohc221.smohc141	-0.082963212	0.066106381	0.211616984
isoleucine.c16	-0.074321904	0.059227473	0.211667758
pcaac425.pcaec423	-0.006593185	0.005254216	0.211674357
asparagine.pcaec363	0.014308297	0.011403276	0.211704694
smc240.prolinr	-0.041638955	0.033185073	0.21170602
serotonin.pcaec423	-0.028382536	0.022620583	0.211716113
smohc241.prolinr	-0.024843104	0.019800871	0.211743136
pcaec362.pcaec360	-0.009266316	0.007387675	0.211870974

pcaac240.c18	0.074479373	0.059403461	0.212054174
lysopcac181.pcaec404	0.040187426	0.032053083	0.212058231
lysopcac160.alanine	-0.085473859	0.068192878	0.212190186
pcaec360.pcaac426	0.096515581	0.077009918	0.212235736
smohc241.pcaec362	0.048231029	0.038485302	0.212255774
serotonin.prolinr	-0.0352515	0.028129909	0.212279173
pcaac405.lysopcac161	-0.11296125	0.090143814	0.212295616
smohc241.c51	0.054954688	0.043860907	0.212365148
pcaac362.serotonin	-0.116585225	0.093057391	0.212401257
pcaec341.lysopcac181	0.046085779	0.03678632	0.212413868
asparagine.c101	-0.032099689	0.025627865	0.212510387
lysopcac203.pcaac300	-0.062115773	0.049601847	0.212598713
smc161.valine	-0.105837328	0.084517201	0.212609483
pcaac342.pcaac360	0.032831446	0.026218808	0.21262696
pcaec444.kynurenine	0.006743869	0.005386075	0.212669178
asparagine.pcaac403	-0.0028298	0.002260123	0.212683026
lysopcac160.pcaec362	0.025969738	0.020745522	0.212767154
pcaac385.smohc161	-0.023784974	0.019004171	0.212860829
methionine.transhydroxyproline	-0.034905751	0.027892313	0.212904094
c0.pcaac403	0.008879068	0.007096523	0.212999099
smc241.pcaec444	-0.14441254	0.115439794	0.213074961
smc161.pcaac322	-0.078254534	0.062561313	0.213122853
pcaac281.pcaec342	-0.205636647	0.164406307	0.213145501
c3.c14	0.022560945	0.018044247	0.213316855
serine.pcaec424	-0.008857959	0.007085391	0.213367885
c102.pcaec424	-0.017667882	0.01413417	0.213426219
pcaac420.serine	-0.062251358	0.049804119	0.213458299
citruline.c51dc	-0.158717782	0.127012354	0.213567272
pcaec361.pcaec423	-0.007739882	0.006194069	0.213589602
citruline.c101	0.016118708	0.012901071	0.21364609
asparticacid.pcaec364	-0.010342513	0.008279804	0.213749293
c2.glutamicacid	-0.091996585	0.073655428	0.213790257
c102.lysopcac180	-0.09603849	0.076894628	0.213808739
pcaec384.pcaac422	-0.057996517	0.046438676	0.213837303
c3.spermidine	-0.085725957	0.068659649	0.213954059
pcaac344.pcaec340	0.071154419	0.05699212	0.213978917
pcaac403.pcaac385	-0.051412735	0.041180875	0.21399143
lysopcac280.pcaec340	-0.061385891	0.049176688	0.214060499
citruline.smc181	0.022525208	0.018045735	0.214076276
leucine.c16	-0.068443356	0.054840955	0.214147572
lysopcac182.smohc221	-0.047229049	0.037844256	0.214165463
pcaec424.asparticacid	0.042732212	0.034247809	0.214256374
pcaec446.glutamicacid	0.095756827	0.076762485	0.214363259

citruline.tyrosine	-0.065190643	0.052262931	0.214393919
spermidine.alanine	-0.113672718	0.091135871	0.214419741
pcaac364.asparagine	0.015353941	0.012312682	0.214524363
pcaec383.pcaec404	0.112507585	0.090232759	0.21457647
pcaec380.pcaec405	-0.129689142	0.104022612	0.214620241
pcaac424.lysopcac204	0.135225945	0.108477882	0.214680115
pcaac365.pcaec423	-0.006198373	0.004972903	0.214733814
pcaac405.lysopcac170	0.011985215	0.009616006	0.214751203
pcaec360.pcaac425	0.131253466	0.105310857	0.214765355
smc241.c12	0.058408652	0.046864839	0.214773313
acetylornithoine.pcaac422	0.081848816	0.065704418	0.214996159
pcaec420.acetylornithoine	-0.108685354	0.087249198	0.215004817
pcaac380.isoleucine	0.048092612	0.0386127	0.21506929
alanine.c14	0.053766243	0.043180448	0.215201266
pcaec360.asymmetricdimethylarg	0.097702396	0.07847713	0.215264754
pcaac424.pcaec424	-0.060163387	0.048329691	0.21531081
methionine.lysopcac280	0.013677608	0.010989535	0.215402644
lysopcac180.pcaac381	-0.043424924	0.034892484	0.21542752
pcaec405.pcaec402	-0.203120429	0.16321825	0.215450943
kynurenine.methioninesulfoxide	0.050976709	0.040962805	0.215453865
citruline.prolinr	0.022611925	0.018170668	0.215470098
pcaec363.smohc161	0.018295415	0.014704309	0.215542613
pcaec383.pcaac385	-0.040463389	0.032523528	0.215577335
arginine.pcaac362	-0.103852402	0.08348181	0.215619287
ornithine.pcaec403	-0.049310657	0.039639171	0.215628177
pcaec361.pcaac365	-0.079831721	0.064178209	0.215657948
pcaac300.spermidine	-0.088846363	0.071431585	0.21569845
glutamicacid.lysopcac204	0.077021983	0.061928496	0.215724939
pcaac341.pcaec421	0.004297057	0.003455251	0.215759218
pcaac425.pcaac380	0.092809486	0.074627872	0.215759476
pcaec420.pcaac341	0.007893865	0.006347504	0.215764276
smc161.pcaec404	-0.011112316	0.008936135	0.215797795
pcaac405.lysine	0.115685496	0.093035743	0.215824885
pcaac240.pcaac344	-0.101687117	0.0817883	0.215882
smc180.pcaec382	0.012364343	0.009945455	0.215911849
creatinine.spermidine	0.086876033	0.069880669	0.215915454
lysine.c8	-0.06439698	0.051799516	0.21591875
pcaac383.asparticacid	0.031196751	0.025094596	0.215929935
smohc222.valine	-0.125125437	0.100658828	0.215967172
pcaac404.pcaec444	0.117564481	0.094576631	0.215968696
c51dc.alphaaminoadipicacid	0.011846156	0.009529994	0.215976568
pcaac424.pcaec380	0.113198324	0.091074135	0.216018476
pcaac341.pcaac426	0.088148412	0.070925317	0.216051843

pcaac421.pcaec340	0.073584712	0.059214204	0.216106002
isoleucine.pcaac341	0.009751139	0.0078475	0.216145668
pcaec362.pcaac406	-0.055037465	0.044295424	0.216171357
pcaac360.glycine	0.065524016	0.052744297	0.216249767
pcaac402.pcaec342	0.122949405	0.098971898	0.216260778
smc202.pcaec383	-0.09972749	0.080282337	0.216281438
pcaac403.pcaac341	0.008013942	0.006451448	0.216287629
pcaec402.pcaac360	-0.104965975	0.084504457	0.216308743
lysine.alphaaminoadipicacid	-0.00731396	0.005888788	0.216353206
pcaac404.creatinine	-0.034146276	0.027492962	0.2163583
pcaac424.pcaec342	0.124001468	0.09985481	0.216425597
smc202.c141oh	-0.104234166	0.083940661	0.216446765
c5.pcaac341	0.011417305	0.009195218	0.216484785
smc202.smc180	-0.043440382	0.034998098	0.216645109
lysopcac181.c12	0.083942549	0.067631429	0.216661528
pcaac281.asparagine	0.008450305	0.006808605	0.216681925
pcaec424.smc240	-0.051946674	0.041861968	0.216762005
pcaec364.pcaec424	-0.029479261	0.023763946	0.216909421
pcaac426.serine	-0.047195513	0.038051473	0.216981844
pcaac362.pcaec342	0.155877263	0.125678024	0.216987956
pcaac381.c182	-0.07788805	0.062806251	0.217046345
pcaec380.pcaec364	-0.01381941	0.011143851	0.21706082
methioninesulfoxide.c51dc	0.233193801	0.188054219	0.217082178
c51dc.pcaac363	-0.048969663	0.039496097	0.217146385
pcaec424.pcaec300	0.018956815	0.015289689	0.217152925
alphaaminoadipicacid.smc180	-0.032297811	0.026050647	0.217165699
serine.pcaac323	-0.132549276	0.1069409	0.217293174
pcaac383.smohc241	0.075813501	0.061171247	0.217329297
smc160.pcaac406	-0.053736249	0.043359713	0.217348543
isoleucine.smc202	0.076438873	0.061704586	0.217542446
smc260.pcaec361	-0.008003721	0.006460933	0.217542647
isoleucine.pcaac385	0.100537174	0.081168301	0.217602086
c182.pcaac422	0.039989602	0.032289533	0.217659919
pcaac343.alanine	-0.02484346	0.020061893	0.217707487
c14.pcaec363	-0.026308648	0.021246195	0.217731508
leucine.valine	-0.113677579	0.091841299	0.217921765
pcaac385.pcaec321	-0.051898725	0.041934211	0.217972965
kynurenine.smohc141	-0.048973891	0.039572911	0.217995731
pcaec361.smohc241	0.110168757	0.089048463	0.218137579
pcaec341.c5	0.011676767	0.009438275	0.218139718
c182.pcaec380	-0.06014698	0.048621467	0.218186336
lysopcac170.pcaec444	-0.111902009	0.09046866	0.218234872
methionine.pcaac361	0.051944483	0.041998112	0.218266566

pcaac320.pcaac403	-0.034759794	0.028107374	0.218322157
lysopcac203.pcaac320	-0.068496046	0.055389405	0.218341268
pcaec301.smc240	-0.037662837	0.030458721	0.218380731
pcaac406.pcaec363	-0.039095642	0.031618662	0.21839814
acetylornithoine.c141oh	0.121320807	0.098125765	0.218432537
pcaec420.smc160	-0.082084852	0.066395556	0.218462297
pcaec420.pcaec424	0.007092373	0.00573696	0.218477308
phenylalanine.pcaec404	-0.034838181	0.028185049	0.21855439
lysopcac280.citruline	-0.031311808	0.025332968	0.218569786
smc161.arginine	0.077981708	0.063102624	0.218650732
putrescine.c2	0.061224588	0.049547789	0.218696854
c51.smc160	0.103209746	0.083541344	0.218783707
citruline.pcaac344	-0.07637697	0.06182294	0.218790544
creatinine.pcaac281	-0.004455285	0.003606603	0.218828049
lysopcac170.c12	0.069438433	0.056216976	0.218875288
pcaac361.smohc141	-0.02512115	0.020349857	0.219143575
c16.pcaec401	-0.06370842	0.05161124	0.219170599
pcaec421.lysopcac161	0.122916668	0.099599622	0.219275558
pcaec343.pcaec363	-0.087850136	0.07119384	0.219331573
methioninesulfoxide.pcaac300	-0.096762401	0.078417656	0.219339215
c0.pcaac362	-0.042363534	0.034332249	0.219342227
pcaec423.pcaac405	-0.072183568	0.058502381	0.219368743
glutamine.pcaec322	0.054343548	0.044049238	0.219427024
c0.pcaac420	0.051674639	0.041887421	0.219443661
pcaac405.h1	0.002220951	0.001801285	0.219694196
pcaac322.lysopcac204	-0.044704587	0.036264982	0.219791247
pcaec445.pcaec424	0.032706746	0.026534507	0.219831695
pcaac384.c51dc	-0.141931115	0.11515788	0.21987643
pcaec364.pcaec443	-0.071413485	0.057945558	0.219901646
smohc161.phenylalanine	0.063053397	0.051165948	0.219936092
c0.pcaac322	-0.072391036	0.058744535	0.21994681
c162.pcaec301	0.102000393	0.082803582	0.220120679
alphaaminoadipicacid.lysopcac2[illegible]	-0.011325802	0.009197079	0.220261901
pcaac320.smc161	-0.063954536	0.051934495	0.220265716
h1.phenylalanine	-0.08243376	0.066948354	0.22031879
c101.pcaac406	0.035481555	0.028818638	0.22035683
leucine.pcaac281	-0.004260034	0.003460427	0.220405265
pcaec380.pcaac405	-0.069685788	0.056608931	0.220430746
methioninesulfoxide.pcaac322	0.060026907	0.048766145	0.220464257
pcaec340.smohc141	-0.059777178	0.048564079	0.220471967
pcaac404.pcaec382	0.007962475	0.006469305	0.220503491
pcaec443.pcaec404	0.059320619	0.048209311	0.220625747
citruline.c3	-0.040141042	0.032626466	0.22068509

pcaac425.pcaac281	0.006111047	0.004967424	0.22072129
smc161.pcaec322	0.123093843	0.100058128	0.220721728
pcaec300.pcaac341	-0.008521859	0.00692755	0.220752697
lysopcac203.pcaac321	-0.051692859	0.042022706	0.220761283
alanine.pcaec404	-0.036526691	0.029695682	0.220792473
pcaec422.pcaec340	-0.078417701	0.063755847	0.220816736
pcaac406.pcaec402	0.046464122	0.037779565	0.220851935
smohc161.pcaec361	-0.02251215	0.018304882	0.220863294
c51.pcaac361	-0.108403651	0.088146597	0.220875653
c12.pcaec424	-0.01035675	0.008421792	0.220896141
c141oh.c51	0.047749008	0.038830648	0.220927077
pcaac420.pcaec423	-0.011804082	0.009600019	0.220958404
smohc222.kynurenine	-0.005261148	0.004279774	0.221064528
pcaec364.smohc221	0.045323203	0.036869566	0.221072114
c102.c2	0.113255479	0.092141981	0.221125929
ornithine.pcaec404	-0.054534567	0.044371834	0.221165261
pcaec301.pcaec405	-0.069159459	0.056290652	0.22132305
pcaac362.c16	0.055538292	0.045206219	0.221345237
isoleucine.pcaac344	-0.090659757	0.073799023	0.221377389
pcaac362.alphaaminoadipicacid	0.010164513	0.008274413	0.221392762
pcaac405.smohc141	-0.017125382	0.013941224	0.22140338
pcaec443.pcaec406	0.036108469	0.029396224	0.221426484
pcaec322.pcaac366	0.071544829	0.058260946	0.22155007
h1.asymmetricdimethylarginine	0.035653043	0.029035136	0.221579681
pcaac380.pcaec423	-0.005014106	0.00408377	0.221622524
arginine.alanine	-0.062069914	0.050558692	0.221672187
citruline.ornithine	-0.058445476	0.047607997	0.22168735
smc180.c16	0.06835346	0.055684057	0.221731108
pcaec384.c51	0.035535578	0.028966363	0.222006548
pcaac361.pcaac406	-0.057029217	0.046489666	0.222036645
lysopcac160.valine	-0.051326657	0.041842931	0.222057923
pcaec406.pcaec424	0.008808884	0.007181289	0.22206046
lysopcac204.pcaec424	0.007766125	0.006331512	0.222083292
pcaec446.c142	0.051132255	0.041686976	0.222085797
pcaec340.pcaac341	-0.023778375	0.019386091	0.222088586
pcaec360.prolinr	-0.04902374	0.039968885	0.222096537
serotonin.pcaec360	-0.014795649	0.012063308	0.222114318
lysopcac181.lysopcac280	0.01756913	0.014325389	0.222139483
lysopcac170.pcaec424	0.006366344	0.005191532	0.222191676
histidine.asymmetricdimethylarg	0.020590215	0.016792398	0.222240784
pcaac321.spermidine	-0.087342593	0.071245211	0.222323135
glutamine.kynurenine	-0.019243878	0.015698124	0.222350198
pcaec385.c142	-0.064419462	0.052554553	0.222390541

glutamicacid.pcaec422	-0.097346621	0.07942161	0.222416547
smc260.smohc221	-0.05341063	0.043588338	0.222548731
c141.pcaec424	-0.016477503	0.013448413	0.222587953
serine.lysopcac182	-0.032543056	0.026570255	0.222755066
methionine.pcaec424	-0.016785969	0.013707202	0.222823962
pcaac321.pcaec340	0.10918882	0.08917536	0.222892083
pcaac385.pcaec423	-0.005717899	0.004669858	0.222892677
histidine.pcaec383	-0.068119724	0.055637086	0.222918362
pcaac364.pcaac320	0.121778494	0.099471137	0.222955432
lysine.smohc141	-0.055388152	0.045243794	0.222972019
c8.pcaac425	-0.105181736	0.085928211	0.223028506
pcaac365.pcaec405	-0.06629773	0.054162791	0.22303586
pcaec360.lysopcac180	-0.056514815	0.046180802	0.223138436
pcaec360.pcaec405	0.119851911	0.097937227	0.223142331
pcaec300.c181	-0.018271522	0.014932802	0.223209877
pcaac323.pcaac281	0.002367256	0.001934838	0.223244551
c4.pcaec382	-0.022427238	0.018333351	0.223315339
acetylornithoine.asparticacid	0.018475143	0.015105011	0.223386548
c51dc.methioninesulfoxide	0.046865137	0.038316862	0.223393781
pcaac366.pcaac240	-0.032336629	0.026438512	0.223396134
pcaec342.pcaac363	-0.032724125	0.02675545	0.22339821
smc260.lysopcac181	0.031191727	0.025502856	0.223403692
pcaac344.c2	-0.095220466	0.077859506	0.223437565
pcaac341.smc161	-0.05546953	0.045357834	0.223455037
pcaac322.lysine	-0.08631005	0.07058133	0.223487533
arginine.pcaec385	0.049985077	0.040877568	0.223504727
pcaec404.smc240	-0.047556225	0.03889142	0.223506572
pcaec385.c141oh	-0.160961469	0.131653852	0.223575723
pcaac365.pcaac402	-0.104701237	0.085642305	0.223602119
pcaec406.lysopcac160	0.102081727	0.083501873	0.2236145
alphaaminoadipicacid.pcaac363	0.03286506	0.026888206	0.223698473
pcaec360.pcaac320	0.068083506	0.055703103	0.223709172
pcaac320.pcaac381	0.061863735	0.050615907	0.223723386
prolinr.smohc141	-0.024899697	0.020378385	0.223855771
pcaec420.pcaec406	0.023384452	0.019138748	0.223867032
methioninesulfoxide.pcaec405	-0.163043003	0.133442137	0.22387181
creatinine.c101	-0.016660607	0.013636937	0.22390912
smc261.c181	0.027077937	0.022163902	0.223914051
c102.spermidine	-0.092041938	0.075344498	0.22395133
c141oh.pcaec403	0.085358409	0.069875354	0.223963977
pcaec403.c181	0.042232304	0.034572792	0.223976683
prolinr.alphaaminoadipicacid	0.017791559	0.014568331	0.224089235
pcaec445.phenylalanine	0.073614988	0.060279285	0.224095671

pcaec406.pcaec423	-0.00710607	0.005820915	0.224265412
c2.pcaac403	0.007051389	0.005776557	0.224299979
ornithine.pcaec361	-0.021570034	0.017670689	0.224308733
c51.smohc141	-0.012795889	0.010484416	0.22438424
pcaec401.pcaec423	-0.050649979	0.041504892	0.22443331
pcaec341.smohc141	-0.049346026	0.040444363	0.224524379
pcaac363.transhydroxyproline	0.024190515	0.019827963	0.224553209
acetylornithoine.pcaac322	0.086111381	0.070598608	0.224662128
pcaec425.pcaac422	-0.035063776	0.02874821	0.224679689
c10.pcaec342	0.084532539	0.069314289	0.224729306
pcaec381.kynurenine	0.016520192	0.013546116	0.224730192
lysopcac281.pcaac405	0.106078155	0.086984212	0.224745884
pcaec361.c141oh	-0.113817793	0.093332887	0.224756564
c182.smohc221	0.021400051	0.017550221	0.224802481
pcaac364.pcaac403	-0.005992341	0.004915371	0.224900194
smc181.pcaec404	-0.011959925	0.009811856	0.224967011
lysopcac281.pcaac422	-0.018119341	0.014866084	0.225000418
pcaac300.smohc141	-0.04277079	0.035096468	0.22506622
putrescine.asymmetricdimethylar	0.018112255	0.014862592	0.225072475
pcaec405.serotonin	-0.147447431	0.121032204	0.225222977
pcaac321.pcaec421	0.013327785	0.010940942	0.225258021
alphaaminoadipicacid.c101	0.008845948	0.007262104	0.225280717
smohc141.pcaec443	-0.098461244	0.080834028	0.225292204
pcaec401.pcaac426	-0.114722178	0.09419814	0.225362362
pcaac404.pcaec406	0.014883646	0.01222116	0.225371034
serotonin.pcaec443	-0.087728462	0.07204452	0.225431943
pcaac323.pcaec421	0.002159879	0.001773885	0.225469722
c2.putrescine	-0.050853583	0.041766993	0.225486355
pcaec365.spermidine	-0.082916403	0.068130219	0.225685792
creatinine.pcaec424	0.006037607	0.004960989	0.225690116
putrescine.pcaec422	-0.038892515	0.031960346	0.225734804
smc160.pcaac362	-0.018916579	0.015546055	0.225768972
pcaac425.lysopcac204	0.070955223	0.058312596	0.225769408
pcaac323.pcaec301	0.085880868	0.070582897	0.225796027
pcaec446.pcaac341	0.005560664	0.004571298	0.225912747
pcaac380.lysopcac181	0.033416762	0.027473079	0.225944748
pcaac320.pcaec363	-0.039812426	0.032739696	0.226064913
pcaec322.kynurenine	-0.008933999	0.007346973	0.22607187
pcaec361.pcaac402	-0.090814368	0.074699842	0.226180927
pcaac362.alanine	-0.048885874	0.04021169	0.226184994
c18.phenylalanine	0.065585933	0.053950212	0.226199361
methioninesulfoxide.pcaac383	0.021395877	0.017600609	0.226215475
smohc241.pcaac341	0.007479382	0.006153299	0.226262989

smc160.c51	0.069945615	0.057556864	0.226363312
smohc241.pcaac406	-0.078036812	0.064219899	0.226398903
pcaac300.asparticacid	-0.051229862	0.042159671	0.226403086
pcaac320.isoleucine	0.040501942	0.033337135	0.226486457
lysopcac182.pcaec423	-0.054127642	0.044552493	0.226486973
c0.pcaec405	-0.061471324	0.050599929	0.226512969
pcaac361.pcaac422	-0.023716056	0.019522185	0.226522129
pcaec383.pcaac341	0.018600123	0.015311736	0.226546243
pcaec361.lysopcac204	-0.058234306	0.047939275	0.226550423
c182.c5	-0.012001473	0.009879795	0.226551295
lysopcac281.pcaac406	-0.049897626	0.041077764	0.22656569
lysopcac203.c12	0.10699043	0.088082861	0.226586503
pcaac341.pcaac363	-0.016711223	0.013759308	0.226631051
pcaac240.pcaac383	0.008910703	0.007336999	0.226650359
smohc221.pcaac344	-0.083096059	0.068422148	0.226660757
lysopcac161.c3	-0.081271975	0.066928357	0.226717217
kynurenine.pcaec363	-0.035829906	0.029510643	0.226784935
lysopcac180.c18	-0.059870398	0.049318398	0.22685283
pcaec386.pcaac383	0.008952712	0.007375532	0.226897423
pcaec362.pcaec423	-0.01659771	0.013674589	0.226926555
methioninesulfoxide.smohc221	-0.071635289	0.05901951	0.226929192
c14.c51	0.040595074	0.033447513	0.226952498
smc161.pcaac403	-0.005293011	0.00436182	0.227031693
pcaac383.valine	-0.088929437	0.073284814	0.227035463
pcaec343.lysopcac204	-0.031785559	0.026204073	0.227216918
glutamicacid.smohc141	-0.023966944	0.019758484	0.227219048
lysopcac180.pcaec424	0.005069504	0.004179563	0.227245116
pcaec443.pcaac363	-0.023716299	0.019553701	0.227262814
smohc141.taurine	0.075118998	0.06194528	0.227344112
c182.alanine	0.023080645	0.019033919	0.227367362
citruline.pcaec424	-0.004560227	0.003760818	0.227383952
asparagine.lysopcac140	0.028736232	0.023699746	0.227403137
pcaec423.lysopcac161	0.114137366	0.094136538	0.227420704
c18.c51	0.052937538	0.043663253	0.227444089
smc261.pcaec424	0.009604708	0.007923221	0.22751358
glutamicacid.c10	0.071110916	0.058663427	0.227527995
pcaec444.pcaec383	-0.052497485	0.043309602	0.227543476
isoleucine.pcaac281	-0.005027541	0.004147806	0.227561714
pcaec420.pcaac383	0.009302724	0.007676963	0.227685764
pcaec386.h1	0.00366053	0.003021046	0.227722012
asparticacid.kynurenine	-0.014143493	0.011672685	0.227722794
pcaec422.pcaac383	0.013551496	0.011184845	0.2277533
pcaac365.pcaac421	-0.065276995	0.053888976	0.22785665

pcaac386.smc180	0.034340582	0.028349661	0.227857121
c51.pcaec363	-0.020884409	0.017243667	0.227928725
pcaac425.pcaec364	-0.013147414	0.010855864	0.227946417
pcaec446.smc180	0.024787896	0.020473491	0.22808297
pcaac404.smohc141	-0.017683935	0.014607151	0.228119576
c102.pcaec421	-0.007446939	0.006151486	0.228136217
glutamicacid.pcaac360	0.087773008	0.072508254	0.228162048
lysopcac280.pcaec383	-0.126217389	0.104280503	0.228223265
pcaac281.pcaac341	0.024844475	0.020527959	0.228257399
smc240.creatinine	-0.010676508	0.008822731	0.228319075
pcaac342.pcaac403	-0.00433508	0.003582721	0.228363805
acetylornithoine.pcaec364	0.044109091	0.036462697	0.228475548
pcaac425.pcaac341	0.00821306	0.00678948	0.228487071
pcaac406.lysopcac161	-0.108783282	0.089929069	0.228493947
asparticacid.lysopcac204	0.060998508	0.050440633	0.228625446
pcaec422.c182	-0.032452209	0.026835324	0.228626792
smohc161.taurine	0.075389953	0.062350455	0.228694554
pcaec422.asparticacid	0.040333866	0.033365684	0.22880546
pcaac240.lysopcac161	0.102733494	0.084987652	0.228819943
pcaec424.lysopcac161	0.072526041	0.060006497	0.22888448
pcaec401.smohc222	0.009636237	0.007974708	0.228994311
pcaac404.c5	0.014290745	0.011826663	0.228994356
pcaec341.c101	-0.007373648	0.006103417	0.229083154
pcaec381.pcaec406	0.024477367	0.020268474	0.229259882
pcaac405.transhydroxyproline	0.048254554	0.039958425	0.229274504
pcaac402.pcaac383	0.011482428	0.0095086	0.229288164
pcaac380.pcaec406	-0.024349185	0.020165774	0.229338945
pcaac240.c51dc	0.16831585	0.139412915	0.229389634
pcaec380.c181	-0.033715963	0.027927553	0.229410237
pcaac386.pcaec424	0.004486064	0.0037159	0.22941152
asparticacid.pcaac320	0.058111191	0.048135253	0.229416826
pcaac343.pcaec383	-0.097039585	0.080382084	0.229424132
c0.c2	0.097016342	0.080367636	0.229451831
pcaac384.pcaec343	-0.096025209	0.079552589	0.229486779
pcaec381.pcaac384	0.078612313	0.065131013	0.229516865
creatinine.histidine	-0.067041875	0.055551306	0.22957112
serine.pcaec444	-0.162314555	0.134499072	0.229585748
prolinr.pcaac366	0.035583986	0.029486848	0.229598478
c18.histidine	-0.066522239	0.055124646	0.229604023
pcaac426.pcaac383	0.01095282	0.009076336	0.229610057
pcaac362.pcaec365	0.030241813	0.02506166	0.229628625
pcaec422.pcaac322	-0.098841783	0.081911073	0.229628654
pcaac381.pcaac363	-0.034719065	0.028773671	0.229655556

c10.pcaec443	-0.083202097	0.068960698	0.229698364
pcaac403.lysopcac161	0.095823617	0.079421986	0.229699231
lysopcac204.asymmetricdimethy	0.028902558	0.023960435	0.229795525
pcaec421.c141	0.083592865	0.069300249	0.229803164
lysopcac281.lysopcac204	-0.022661655	0.01879125	0.229908243
pcaac424.pcaac341	0.011363206	0.009422588	0.229913883
histidine.pcaac300	-0.070935031	0.058846774	0.230119418
pcaac383.pcaec360	-0.007086753	0.005879146	0.230124828
smohc141.tyrosine	-0.087298388	0.072425573	0.230144841
lysopcac170.pcaec443	0.078549693	0.065171482	0.230174075
pcaac362.c102	0.057180785	0.047442725	0.230180919
pcaac361.c16	0.047191236	0.039161214	0.230261284
pcaec403.pcaac385	-0.038357122	0.031831375	0.230276866
lysopcac182.pcaec360	-0.013347919	0.011078069	0.230320777
c0.smohc221	0.037178893	0.030857179	0.230330651
prolinr.lysopcac203	0.025055393	0.020795334	0.230335884
pcaec385.pcaac381	0.04503221	0.037376982	0.23035329
pcaec321.pcaec382	0.025883445	0.021483545	0.230356375
pcaac363.alanine	-0.029330181	0.024348133	0.230427964
pcaec424.pcaec401	0.08493637	0.070518848	0.230492679
smc241.pcaac361	0.085978106	0.071387994	0.230520225
pcaac300.pcaac383	0.007566324	0.006282911	0.2305615
lysine.leucine	-0.033894289	0.028146723	0.230588573
pcaac381.lysopcac181	0.043361062	0.036013215	0.230653368
pcaec383.pcaec443	-0.182139295	0.151283698	0.230681669
pcaac422.pcaac383	0.015072176	0.012520668	0.230749022
pcaec321.pcaec424	0.009929662	0.008250003	0.230821754
smc240.pcaec406	0.011795154	0.009800151	0.230831925
pcaec300.pcaac365	-0.073189033	0.060811234	0.230841173
c181.pcaac385	0.051272497	0.042610038	0.230936926
pcaec403.pcaec405	-0.224256649	0.186388859	0.230987321
c142.pcaac425	-0.079675155	0.066226176	0.231021661
pcaec365.pcaec383	0.082497024	0.068574278	0.231038971
c182.lysopcac161	-0.115606367	0.096097226	0.231045522
pcaac365.pcaec362	-0.070071076	0.058247118	0.23105253
smohc241.pcaac344	-0.084136974	0.069948595	0.231112841
pcaac361.pcaac383	0.009062072	0.007535279	0.231198018
glutamicacid.pcaac322	0.052754153	0.043869909	0.231238897
pcaac341.putrescine	-0.042677299	0.035490085	0.231238931
c16.pcaac422	0.040543152	0.033719106	0.231290687
pcaec343.alphaaminoadipicacid	-0.013688597	0.011385227	0.231316575
pcaac385.asymmetricdimethylarg	0.025844347	0.021495723	0.231320562
kynurenine.pcaec361	-0.009106145	0.007574562	0.231359519

pcaac401.pcaac383	0.012223987	0.010168023	0.231360285
ornithine.pcaec423	-0.005388834	0.004482674	0.231380253
pcaac386.pcaec444	0.195191694	0.162378619	0.231407145
smc260.smc241	-0.138404428	0.115154016	0.23147298
smc260.pcaac344	-0.077403659	0.064401953	0.231482004
pcaac363.pcaac403	-0.008313057	0.006917209	0.231516837
c12.pcaec363	-0.022733068	0.018916461	0.231529344
smc160.pcaac384	0.070216938	0.05843145	0.231553865
smc161.pcaec423	-0.011051479	0.009196718	0.231562088
pcaac341.pcaac383	0.007575001	0.00630472	0.231637616
lysopcac181.pcaec424	0.009127163	0.007596888	0.231655585
ornithine.c51dc	0.136894685	0.11395244	0.231695254
c5.smohc141	-0.024405442	0.020320028	0.231802774
lysopcac170.pcaec360	-0.007764181	0.006464971	0.23183836
methionine.pcaac383	0.006903973	0.005748705	0.231838388
pcaac422.smc241	-0.073016395	0.060810083	0.231928511
pcaec300.transhydroxyproline	0.041735929	0.034758972	0.23193026
lysopcac140.smc181	-0.044685007	0.037216822	0.231952309
pcaec424.smohc241	0.086053794	0.071677986	0.231993273
c5.c18	-0.135162452	0.112592073	0.232031753
pcaec421.c181	-0.021808844	0.018168415	0.232066666
pcaac421.pcaac383	0.007288996	0.006072333	0.232070364
phenylalanine.pcaac365	-0.067760266	0.056461097	0.232162722
c181.pcaac300	0.037165283	0.030970438	0.232200898
pcaec406.serotonin	-0.167630918	0.139695341	0.232220033
pcaec405.c101	0.027243191	0.022703202	0.232221476
pcaec424.pcaec380	0.09402415	0.078356036	0.232225643
methioninesulfoxide.pcaac341	0.013023824	0.01085403	0.232246444
pcaec365.pcaac383	0.006444874	0.005371466	0.232274172
serotonin.pcaac341	0.005156882	0.004298164	0.232292759
ornithine.c51	0.049986503	0.041666121	0.232329689
pcaec365.lysopcac180	0.095504064	0.079609846	0.232345324
smohc141.c141oh	-0.118304643	0.098628253	0.23240375
lysopcac161.pcaac344	0.073067239	0.060919375	0.232439259
pcaec386.pcaec402	0.063204289	0.052700988	0.232481476
pcaec403.pcaac363	-0.035271011	0.029412893	0.232532447
c16.c3	0.106726391	0.089002726	0.232544901
arginine.serotonin	0.143129693	0.119363832	0.232557237
pcaec363.c182	-0.064268547	0.05359896	0.232573151
c8.methioninesulfoxide	0.019208015	0.016022787	0.232677693
pcaac424.creatinine	-0.023787598	0.019845834	0.232745304
creatinine.pcaec363	0.023413333	0.019536203	0.232807525
smohc221.smohc161	0.046861682	0.039101831	0.232809993

lysine.smc202	0.065517237	0.054673617	0.232856023
c181.pcaec380	-0.03976371	0.033183511	0.232870154
pcaec445.c142	0.036989722	0.030869028	0.232877067
smohc241.pcaac383	0.008828933	0.007368158	0.232886505
pcaec360.pcaac381	0.0531492	0.044357066	0.232902946
leucine.c51dc	-0.204128889	0.170412904	0.233044158
lysopcac280.serotonin	-0.153821382	0.12842971	0.233098521
ornithine.pcaec342	-0.084993348	0.070971089	0.233149631
pcaac425.creatinine	-0.026906611	0.02246815	0.233162243
pcaec343.tyrosine	-0.07205127	0.060171234	0.233203951
pcaec403.smohc221	-0.09354411	0.078120737	0.233206739
pcaac426.alanine	-0.025931036	0.021657056	0.233238499
lysopcac281.transhydroxyprolin	0.042696139	0.035659353	0.233244176
smc260.pcaac386	0.037233443	0.031097732	0.2332556
c16.pcaec340	0.0894802	0.07473731	0.233271657
pcaac380.pcaec362	0.019545539	0.016325649	0.233284815
c4.pcaac422	0.049370327	0.041257981	0.233519487
pcaac401.c51	0.142735274	0.119291408	0.233557911
c10.c141	0.084083362	0.070273118	0.233559312
phenylalanine.smohc141	-0.01912157	0.015981715	0.233581455
leucine.c2	0.053962732	0.045104839	0.233612912
pcaac381.pcaec424	0.006039573	0.005048597	0.233650656
pcaac420.smohc241	0.076203659	0.063703291	0.233673785
pcaac320.lysopcac180	-0.062658427	0.052385031	0.233718414
pcaac426.pcaec382	0.010836799	0.009060129	0.233724445
putrescine.pcaac320	-0.023558615	0.019697374	0.233751476
c51dc.pcaec362	-0.033755009	0.028227238	0.233828147
pcaac426.pcaec406	0.014698826	0.012294013	0.233914788
smohc221.creatinine	-0.008968883	0.007502611	0.233982278
kynurenine.valine	0.106865834	0.089396133	0.23398851
pcaac380.pcaec424	0.00807897	0.006758379	0.233995687
alphaaminoadipicacid.asparagine	0.012168907	0.010180333	0.234021236
smohc221.alanine	-0.046797503	0.039154294	0.234070861
pcaec446.pcaac366	0.093441877	0.078194576	0.234154814
lysopcac161.lysopcac160	-0.03150766	0.026367705	0.234177436
c4.pcaac322	-0.113209513	0.094752024	0.234230362
pcaac320.c142	0.032025256	0.026808336	0.234307172
pcaac384.smc240	-0.041208787	0.034496442	0.234314906
pcaac360.pcaec423	-0.00551486	0.00461659	0.23431747
kynurenine.spermidine	0.098331298	0.082316155	0.234324372
pcaec341.pcaec423	-0.007478487	0.006260583	0.234332689
pcaec386.c51	0.041592156	0.03482179	0.234374044
pcaec384.pcaec406	0.016220476	0.013581843	0.234433461

leucine.kynurenine	-0.006853763	0.005738872	0.234436004
ornithine.lysopcac181	0.035629368	0.029834664	0.234452698
smohc241.pcaec424	0.009597551	0.008036928	0.234470528
pcaac322.pcaac362	0.043828923	0.036712492	0.234602788
c162.pcaac383	-0.008094007	0.006779885	0.234608826
c5.lysopcac182	-0.117039779	0.09804011	0.234621257
c142.pcaec423	0.018447841	0.015454041	0.234649349
smohc241.pcaac385	-0.030903829	0.02589012	0.234676443
pcaec404.pcaec423	0.027310577	0.022880345	0.234687074
acetylornithoine.smohc141	0.030260837	0.025354817	0.234738372
leucine.pcaec383	0.09252406	0.077525861	0.234751634
pcaac320.putrescine	-0.039229675	0.03287282	0.234784154
lysopcac161.pcaec422	0.085390745	0.0715584	0.234813712
c51dc.c16	-0.060112706	0.050379016	0.234849721
alanine.pcaac385	0.023927382	0.020057389	0.234952445
c102.c141oh	0.169229573	0.141862237	0.234964601
pcaec401.glutamicacid	0.07530938	0.063132613	0.234979777
lysopcac204.smohc141	0.051169291	0.042900164	0.235027877
smc241.c14	-0.064744488	0.054283392	0.235043563
pcaec362.pcaac385	-0.056743469	0.047575218	0.235044332
c102.pcaac383	-0.006180323	0.005181773	0.235046851
pcaac426.lysine	0.150517992	0.126209392	0.235085473
pcaec402.smc161	0.128186882	0.107495673	0.235132808
c4.pcaec424	-0.017139074	0.014373118	0.235150225
pcaec403.lysopcac203	0.016726186	0.014027552	0.235173098
asparagine.taurine	-0.043861897	0.036785622	0.23517924
pcaac403.pcaac383	0.008792799	0.007374319	0.235183629
pcaec443.c182	-0.059034551	0.04952085	0.235276984
pcaac383.c5	0.012050812	0.010110161	0.235341262
c18.kynurenine	0.005804806	0.004870503	0.235388828
smc161.transhydroxyproline	0.045693851	0.038340606	0.235405034
pcaac425.lysine	0.124255635	0.104264863	0.235427219
pcaac386.pcaec382	0.012542348	0.010525218	0.235459806
pcaec402.prolinr	-0.047216397	0.039627217	0.235511805
smc240.pcaac421	-0.037233723	0.03125099	0.235540328
c2.tyrosine	0.081614921	0.06850293	0.235553486
smohc241.c18	0.035562873	0.029852755	0.235604979
c12.c18	-0.059469818	0.049926749	0.235657663
pcaac321.c182	-0.032586631	0.027360152	0.235703109
pcaec421.lysopcac182	-0.130494438	0.109584679	0.235787665
pcaec424.smohc222	-0.015006577	0.012602577	0.23580901
smc160.c141oh	-0.112885726	0.094818823	0.235892501
c101.pcaac385	0.019017584	0.015976373	0.235964745

pcaac425.c181	-0.012450337	0.01045957	0.235975357
lysopcac160.pcaec424	0.005760293	0.004842022	0.236243131
smc241.pcaec424	0.004317132	0.003628953	0.236247182
pcaec341.lysopcac204	-0.028814387	0.024221288	0.236249194
lysopcac204.pcaec423	0.019074977	0.016034543	0.236254272
pcaac323.pcaec443	-0.063571731	0.053441555	0.236278396
pcaec340.ornithine	0.050647752	0.042579723	0.236308031
pcaec445.smc180	0.019759884	0.016613824	0.236353729
pcaac421.histidine	-0.114522616	0.096289994	0.236358756
alphaaminoadipicacid.pcaac420	0.079097394	0.066515191	0.23643259
smc260.pcaec444	-0.160356723	0.134854173	0.236452507
smc260.c162	-0.113996238	0.095867259	0.236455266
pcaac380.pcaac383	0.011905942	0.010013609	0.236505824
pcaec363.spermidine	-0.083662066	0.070381265	0.236614932
methioninesulfoxide.c142	0.040939683	0.034440832	0.236615666
pcaac406.pcaec424	0.003798565	0.003195622	0.236622843
lysopcac160.c141	0.032730831	0.027537716	0.236660467
pcaac362.pcaac360	0.033985179	0.028598731	0.236753164
glycine.pcaac386	0.034041668	0.028648586	0.236790927
pcaac404.pcaac403	-0.008220455	0.006919186	0.236862651
ornithine.alphaaminoadipicacid	0.009701434	0.008166695	0.236917751
pcaec424.c3	-0.041746476	0.035144747	0.236950148
c102.pcaac364	0.076525785	0.064432499	0.237010933
putrescine.pcaac422	-0.02282243	0.019216071	0.237016891
glycine.prolinr	-0.057888899	0.048760107	0.237195904
smohc221.pcaec406	0.008245753	0.006945682	0.237212273
c101.methionine	0.080049199	0.067431532	0.237235315
c3.pcaac365	-0.082078716	0.069141411	0.237237085
c5.pcaac380	-0.149750439	0.126170532	0.237325346
serine.pcaac322	0.052497964	0.044234613	0.237357519
smohc222.pcaac281	0.002604455	0.002194522	0.237361127
glycine.lysopcac280	0.01262593	0.010639086	0.237380305
pcaec446.serine	-0.019236387	0.016209766	0.237393497
pcaac364.lysopcac182	-0.054075299	0.045574712	0.237470603
arginine.transhydroxyproline	-0.058536836	0.049336214	0.237483041
asparticacid.pcaac406	0.085130302	0.071750394	0.237486845
c10.pcaac422	0.025482206	0.021477603	0.237496162
asymmetricdimethylarginine.pca	0.01752159	0.01477312	0.23765728
pcaac383.pcaac281	0.005025736	0.004237458	0.237664912
pcaac385.asparagine	-0.0096188	0.008110824	0.237706048
smc160.pcaec424	0.006391038	0.00538968	0.237757096
smc161.glutamicacid	0.055052956	0.046432692	0.237812629
glycine.asymmetricdimethylargi	0.019741608	0.016652244	0.23786319

pcaac426.lysopcac280	0.008201045	0.006917775	0.237870688
pcaec445.smc181	0.02632212	0.022207594	0.237960361
lysopcac160.lysine	0.108609889	0.091635526	0.237975271
c182.smohc141	0.039943523	0.033702179	0.237993675
leucine.pcaec404	0.038684732	0.032648946	0.238120506
asparagine.pcaec300	-0.021465586	0.018117532	0.238149222
pcaec381.pcaec422	-0.108057456	0.091208827	0.238177008
pcaec360.pcaec342	0.158602692	0.133882688	0.238211139
pcaac365.pcaec342	0.161625081	0.136456076	0.238286686
smc161.pcaec343	-0.101946428	0.086073923	0.238303314
pcaec405.pcaec362	0.01682684	0.014210607	0.238422142
pcaac360.smohc221	-0.080661693	0.068122883	0.23843894
alanine.pcaac281	0.003352056	0.002831733	0.238563098
c3.smohc141	-0.024657538	0.020831586	0.238597
pcaac421.pcaec443	-0.060327427	0.050971913	0.238643788
pcaec300.smohc141	-0.049828824	0.042102577	0.238656602
pcaec363.serotonin	-0.12533145	0.105903243	0.238679357
pcaec443.serotonin	-0.113013896	0.09551189	0.238761549
pcaac405.pcaec321	-0.04174861	0.035291281	0.238868943
alphaaminoadipicacid.c182	0.039325781	0.033243755	0.238876808
serotonin.leucine	-0.038535551	0.032578865	0.238921637
pcaec342.pcaec340	0.175399432	0.148303278	0.238973483
smc160.pcaec361	-0.01621046	0.013708637	0.239055686
c102.lysopcac181	-0.1087305	0.091962517	0.239120802
c3.pcaac383	-0.00699717	0.005919131	0.239202625
pcaac383.pcaec446	-0.053715316	0.04545625	0.239374813
pcaec362.pcaec404	-0.01496531	0.01266478	0.239392553
pcaac363.asparagine	0.061874647	0.05236449	0.239405676
smc260.pcaac360	-0.033161891	0.028065116	0.239409348
c182.valine	-0.061162311	0.051762271	0.239411375
pcaec321.smc160	0.115176928	0.097492473	0.239493358
pcaac384.pcaec321	-0.060533536	0.051243284	0.239531378
smohc141.pcaac320	-0.110780754	0.093798702	0.239629992
pcaac384.isoleucine	0.030032204	0.025430789	0.239673187
pcaac320.pcaec446	-0.05686669	0.048166235	0.239793902
asparagine.pcaec322	0.053797449	0.045569946	0.239828459
pcaac385.pcaec421	0.001877051	0.001590114	0.239866579
pcaec384.smc180	-0.018347613	0.015545228	0.239936868
c18.pcaec424	0.006290624	0.005329855	0.2399413
glutamicacid.pcaec364	-0.025517125	0.021620295	0.239950181
pcaac322.pcaec340	0.073000594	0.061852742	0.239953081
pcaac361.smohc161	-0.01863859	0.015792429	0.239956649
c141.smohc241	-0.111279646	0.094292065	0.239981996

lysopcac182.c18	-0.049028052	0.041543703	0.239983235
pcaac381.c162	-0.089473795	0.07581843	0.240003018
lysopcac160.smc261	0.057156835	0.04843402	0.240006689
lysopcac281.c141oh	-0.111600368	0.09457384	0.240031521
pcaac401.pcaec380	-0.115169258	0.097615384	0.240113698
pcaac404.h1	0.0019081	0.001617322	0.24012844
pcaac381.glutamicacid	0.06960415	0.058999948	0.24015118
c2.pcaec301	-0.080736123	0.068443643	0.240203669
pcaac364.smc160	0.111476843	0.094517123	0.24026895
pcaec362.glycine	0.049383363	0.041878846	0.240363978
pcaec380.pcaec402	0.101857211	0.086383435	0.240390531
pcaec445.pcaec385	-0.111508418	0.094580053	0.240447853
pcaac342.lysopcac204	-0.030276106	0.025680996	0.240469343
lysopcac161.smohc141	-0.065860225	0.055867238	0.240493198
c0.c3	-0.032644682	0.027691752	0.24049752
c141.smc202	-0.054018668	0.04582748	0.240545043
pcaac323.asparagine	0.015351142	0.013024321	0.240579826
pcaec424.pcaec421	0.006258852	0.005310636	0.240620226
c10.pcaac365	0.041248338	0.034999344	0.240621921
phenylalanine.pcaec406	0.005936526	0.005037404	0.240644455
kynurenine.c51dc	0.150388881	0.127627421	0.240702533
pcaac362.pcaac323	-0.089464585	0.075929883	0.240738434
pcaec342.asymmetricdimethylarg	0.065832313	0.05587404	0.240748547
pcaac360.pcaec364	-0.011077639	0.009402136	0.240757558
pcaac422.alanine	-0.023244618	0.019733098	0.240858369
pcaac362.valine	-0.044491781	0.037772073	0.24087794
smc240.pcaec424	0.004033895	0.003424931	0.240916943
pcaac426.smohc241	0.059740429	0.050731074	0.241001557
pcaec385.pcaac344	0.080184472	0.068098457	0.241046047
c51.pcaac383	-0.004287709	0.003641455	0.241048923
smc260.pcaec424	0.007660363	0.006505831	0.241052966
pcaec445.c141	0.035218587	0.029911547	0.241067548
smohc221.smohc241	0.134774401	0.114492491	0.241178201
pcaac404.lysopcac160	-0.029272957	0.024871377	0.241246812
glycine.pcaec383	-0.063279793	0.053771388	0.24130391
c141.pcaac380	-0.065090532	0.055312227	0.241322371
smohc241.pcaec385	-0.062545233	0.053150132	0.241329717
smc260.pcaec423	-0.00475359	0.00403981	0.241361035
c182.pcaac383	-0.005621407	0.004777349	0.241364017
pcaec401.c4	-0.048277943	0.041030091	0.241376716
pcaec402.pcaac405	-0.058292078	0.049549203	0.241455865
acetylornithoine.spermidine	0.079971686	0.067979187	0.241469427
pcaac405.pcaec423	-0.004679726	0.003978216	0.241499874

smc260.c102	0.045583261	0.038752887	0.241533002
pcaac402.pcaec423	0.038441445	0.032682237	0.241547448
pcaec446.asymmetricdimethylar;	0.031599726	0.026867235	0.241577205
pcaec443.creatinine	-0.014889373	0.012660674	0.241621163
pcaec421.smohc241	0.075122941	0.063881436	0.24164452
pcaec360.spermidine	-0.101051474	0.085939515	0.241696457
spermidine.c51dc	0.236691819	0.201338549	0.241797238
pcaec420.pcaac322	-0.066454229	0.056537504	0.241873081
pcaac425.lysopcac180	-0.087814972	0.074745612	0.242092201
pcaec446.transhydroxyproline	0.054583744	0.046461586	0.242106849
smohc222.pcaec363	0.054680578	0.046553657	0.242203941
pcaec322.pcaac426	0.098942468	0.084246149	0.242254231
pcaec446.pcaac383	0.009177871	0.007815241	0.242289965
pcaac420.asparticacid	0.047970541	0.040850151	0.242310056
pcaac322.pcaac360	0.049249626	0.041947019	0.242395415
histidine.pcaec445	-0.083671368	0.071265618	0.242400835
pcaec364.pcaec421	0.001227728	0.001045776	0.24243693
smc260.c141oh	-0.120715861	0.102831666	0.242464878
lysopcac161.smc261	0.079937091	0.068106119	0.242546097
pcaec381.lysopcac161	0.069724823	0.059410991	0.242590977
putrescine.pcaec444	0.115167825	0.098135329	0.242606989
pcaec422.putrescine	-0.056189128	0.047881878	0.242633617
pcaec300.asparagine	0.006753487	0.005755698	0.242688745
pcaec363.putrescine	-0.034533624	0.029431622	0.242690979
citruline.c141	0.064066998	0.054602218	0.242695157
pcaec340.pcaec385	-0.083990917	0.071583006	0.242696992
pcaac322.pcaac420	-0.099936563	0.085181202	0.242742049
pcaac361.pcaac320	-0.087936957	0.074960382	0.24278631
smc180.smohc141	-0.028890323	0.024627251	0.242789753
pcaac403.smohc241	0.08677935	0.073977288	0.242809689
putrescine.glutamicacid	-0.093556938	0.07977729	0.242940594
pcaec444.creatinine	-0.0079137	0.006748343	0.242955994
c51.pcaac365	-0.051313232	0.043757347	0.242960382
pcaec383.smohc241	0.109182895	0.093121434	0.243039663
lysopcac161.c142	-0.042607533	0.036344411	0.243100336
pcaec422.serine	-0.03551223	0.030294542	0.243138354
pcaac321.alanine	-0.044399193	0.037877295	0.24315717
glycine.spermidine	-0.083337366	0.071108557	0.243241623
pcaec365.lysopcac181	0.05219899	0.044548894	0.243341755
pcaec386.pcaec423	-0.004249351	0.003626787	0.243368397
pcaec364.pcaac383	0.005982483	0.005106633	0.243426308
smc180.pcaac341	-0.036502338	0.031159739	0.24344791
pcaac383.pcaec423	-0.006602657	0.005636528	0.24346931

isoleucine.smc180	-0.050820358	0.043384945	0.243478377
pcaec406.pcaac383	0.009747833	0.008323065	0.243558157
pcaec361.pcaec422	-0.048533614	0.041440472	0.243565571
histidine.pcaec322	0.065275806	0.05573776	0.24358199
smc240.pcaac361	0.064540805	0.055112159	0.243599027
pcaac402.glutamicacid	0.13578434	0.115959407	0.243645755
lysine.smc240	-0.056993916	0.048676462	0.243682704
pcaac362.pcaac365	-0.06543198	0.055883207	0.243683517
c3.pcaac361	-0.10175968	0.08691116	0.243692755
glycine.pcaec423	-0.031954984	0.027297178	0.243778458
pcaec360.pcaec424	0.003892914	0.003326051	0.243859415
pcaac300.pcaec423	-0.009165061	0.007830921	0.243884675
pcaec386.pcaec383	0.098411641	0.0840887	0.243899465
pcaec362.pcaac300	-0.115261429	0.098502063	0.243975269
pcaec322.pcaac383	0.005221739	0.004463333	0.244064822
pcaac360.pcaac365	0.109703023	0.093777213	0.244102112
ornithine.pcaac403	-0.006575716	0.005621526	0.244137203
pcaec422.asymmetricdimethylar[illegible]	0.027658123	0.023645881	0.24416054
pcaec421.lysopcac170	-0.064994294	0.055583069	0.244305879
pcaec340.pcaec301	-0.06638797	0.056775342	0.244309211
lysopcac280.pcaec380	-0.053638151	0.045874164	0.244335145
pcaac386.pcaec383	0.093284114	0.079781905	0.244337673
smohc161.pcaac383	0.00591912	0.005062407	0.244341152
pcaec406.h1	0.003969678	0.003395537	0.244398737
smc241.pcaec343	-0.101547004	0.086886148	0.244539484
pcaac381.pcaec423	-0.006864592	0.00587384	0.244565381
pcaec361.pcaec402	-0.152599389	0.130577147	0.244572938
pcaac404.isoleucine	0.039179015	0.033528413	0.244621693
smohc141.lysopcac181	0.061201491	0.052376408	0.24463707
pcaec424.alanine	-0.015555697	0.013312739	0.244641639
smc260.pcaac365	0.082869241	0.070922262	0.244653835
smc261.putrescine	-0.058926626	0.050434036	0.244678618
pcaec321.pcaec444	0.194604847	0.166558836	0.244680648
pcaac424.pcaec364	-0.008592347	0.007354328	0.24469919
pcaac422.c51	0.169711739	0.145281807	0.24477289
pcaac386.glycine	0.032214008	0.027586214	0.244932885
pcaac361.pcaec380	-0.077739164	0.066573503	0.244948248
pcaec424.pcaac422	-0.024685663	0.021140739	0.244963198
pcaec444.putrescine	-0.052240489	0.044742281	0.245001605
c18.pcaac363	-0.013867103	0.01187734	0.245025999
pcaac380.smohc221	0.034703803	0.029724272	0.24502676
pcaac300.pcaec403	-0.101391305	0.086862883	0.245134217
glycine.pcaec301	-0.063945129	0.05479722	0.245261323

c2.c141oh	0.168177254	0.144129363	0.245298255
c51dc.pcaec445	-0.060105051	0.051510574	0.245298484
smohc222.smohc241	0.200392129	0.17173887	0.245301209
pcaac383.creatinine	-0.010358129	0.008877605	0.245329941
c10.pcaac281	-0.005172922	0.004433858	0.245363818
pcaac343.pcaac381	0.026699077	0.022886854	0.245411422
pcaac364.pcaec406	0.018825913	0.016140042	0.245474994
pcaac406.pcaac403	0.041979263	0.035993837	0.245523369
pcaac422.smc181	-0.010653049	0.009135603	0.245599034
lysopcac182.pcaec445	-0.05487136	0.047055696	0.245602485
pcaac365.pcaec382	0.019120923	0.016397804	0.245613702
pcaac425.c18	0.057872492	0.049633772	0.245644264
smohc241.smc161	0.115130917	0.098759216	0.245731434
c18.pcaec443	-0.070913561	0.060834341	0.245768047
pcaac384.lysopcac203	-0.014072594	0.012072416	0.24576861
pcaac386.pcaac362	-0.034840103	0.02988894	0.245780566
c8.pcaac422	0.024219317	0.02077753	0.245781594
smc260.lysopcac280	-0.016704085	0.014330884	0.245801336
pcaac403.glutamicacid	0.115835946	0.099390099	0.245854884
pcaec406.isoleucine	0.04432318	0.038033934	0.245898769
asparagine.ornithine	0.044140673	0.037880725	0.24594097
pcaec402.pcaec443	-0.100701382	0.086420422	0.245942723
c3.pcaac362	-0.025347168	0.021753	0.245952197
pcaec406.pcaac322	-0.074378269	0.063834153	0.245970941
pcaec446.pcaac281	0.002534534	0.002175511	0.246031853
histidine.pcaec360	-0.009854636	0.008458792	0.24603669
pcaec406.putrescine	-0.057524757	0.049377375	0.246042547
lysopcac161.asparticacid	0.036925405	0.031699326	0.246098339
glutamine.pcaec362	-0.01442932	0.012389084	0.246172528
smc161.pcaac405	-0.06731168	0.057795045	0.246179956
c5.glutamine	-0.045629187	0.039178504	0.246185296
smc181.pcaac362	0.056881334	0.048844621	0.246230594
pcaac401.pcaac361	0.09044506	0.077667041	0.246235941
h1.pcaec424	0.007880739	0.00676922	0.246365554
pcaac404.kynurenine	-0.022710896	0.019512349	0.246477709
pcaec321.valine	0.105610127	0.09074019	0.246498111
c2.pcaac385	0.047159422	0.040520814	0.246514959
serine.pcaec364	-0.016664591	0.014320149	0.246561718
pcaac422.pcaac403	0.033492932	0.028785476	0.246634653
pcaec405.pcaec380	0.105387414	0.09058747	0.246698674
pcaac343.pcaac383	-0.005298383	0.004554525	0.24672074
smohc141.pcaec421	0.001650221	0.00141869	0.246770064
glutamicacid.pcaec342	0.139532794	0.119956908	0.246774018

pcaec403.pcaec364	-0.00799575	0.006874082	0.246781059
pcaac321.lysopcac180	-0.062284046	0.053556563	0.24686812
pcaac366.methionine	-0.051755604	0.044505114	0.246886159
smohc221.pcaac300	-0.072367993	0.062230545	0.246891069
lysopcac161.methionine	-0.054358088	0.046744214	0.246898237
pcaac380.pcaec382	0.01955748	0.016818382	0.246906507
c182.pcaec360	-0.008999572	0.007739282	0.246914577
pcaec423.pcaac383	0.015160917	0.013038588	0.246943063
c102.pcaac322	-0.094118594	0.080953485	0.24700265
pcaec446.pcaac386	0.050073455	0.04307299	0.247043088
pcaec405.pcaec364	-0.011233068	0.009663308	0.247075555
pcaec421.pcaac363	-0.022710974	0.019546872	0.247307398
pcaac365.pcaac323	0.107329353	0.092376811	0.247310593
smc161.lysopcac181	0.052329082	0.045043437	0.247358201
pcaec386.lysopcac203	-0.020821968	0.017923541	0.24737299
pcaac342.pcaac406	-0.05918297	0.050957943	0.24749548
asparagine.glycine	0.03988601	0.034344064	0.247512764
pcaac424.smohc221	-0.134856568	0.116125444	0.247538977
lysine.c141oh	-0.142840167	0.123004269	0.247554739
pcaac426.pcaac402	-0.098141203	0.084536346	0.247687199
asparagine.smohc141	-0.02516592	0.021678775	0.24771953
pcaec425.pcaec383	-0.103971195	0.089565199	0.247724216
pcaac240.pcaec406	0.009965374	0.008587112	0.247862101
pcaac386.lysopcac203	-0.019116859	0.016473352	0.247875039
pcaac425.putrescine	-0.042132412	0.036307403	0.247889565
lysopcac170.pcaac300	-0.063499725	0.054723552	0.247915111
pcaec340.kynurenine	-0.009152328	0.007887679	0.247931603
glutamicacid.c16	-0.087830921	0.075701681	0.247975414
c5.pcaac403	0.008101443	0.006983236	0.248014705
pcaec343.glutamicacid	0.06163055	0.053131819	0.248084385
lysopcac160.prolinr	-0.049983397	0.043093093	0.248109597
pcaac421.c142	0.059581689	0.051371037	0.248135206
pcaac342.pcaec342	0.137534871	0.118595422	0.248188934
pcaac384.asparticacid	0.035168558	0.030327934	0.248224884
c10.c14	-0.086914138	0.074958424	0.248270138
pcaec402.pcaec364	-0.008170261	0.007047146	0.248321393
asparagine.pcaac381	0.028800448	0.024841457	0.248321957
creatinine.pcaac322	-0.053720361	0.046343886	0.248404013
c0.serine	-0.041241597	0.03558766	0.24852373
pcaac363.pcaac422	-0.021371654	0.018449343	0.248717604
pcaac380.smc202	0.049705314	0.042912678	0.248760995
pcaac361.pcaac363	-0.01589881	0.013726869	0.248787109
histidine.lysopcac180	0.054778005	0.04729572	0.248796599

pcaec405.smc240	-0.042764489	0.036924508	0.248813725
pcaac381.pcaec364	-0.007674517	0.006626803	0.248837185
pcaac384.smc241	0.078354193	0.067669402	0.248920719
citruline.smc180	0.036091178	0.031170512	0.248934587
pcaec386.pcaec405	0.085690258	0.074023068	0.249035171
pcaac425.pcaec424	0.018921456	0.016347644	0.249105658
pcaec321.pcaec401	0.073893231	0.063843975	0.249121536
pcaec425.pcaec406	0.008755668	0.007564943	0.249122728
citruline.lysine	0.091545156	0.079099749	0.249148082
pcaec360.pcaac300	0.060375033	0.052168673	0.249161826
smc160.pcaac403	-0.005152451	0.004452507	0.249203442
arginine.pcaec301	0.060893686	0.05262158	0.24920436
c4.pcaec422	0.04620982	0.039932621	0.249206436
kynurenine.pcaec424	0.010695517	0.009244829	0.249318789
pcaec360.pcaec404	0.038130985	0.032963756	0.249385549
pcaac403.pcaac363	-0.022474569	0.019431331	0.249442521
pcaec301.smc261	0.045597064	0.039424011	0.249456349
smc181.pcaac403	-0.006056198	0.005237124	0.249531077
pcaec445.pcaac383	0.009440954	0.008164724	0.249566648
pcaac406.smohc141	-0.018856097	0.016308027	0.249592684
pcaec404.pcaec364	-0.008139561	0.007039832	0.24960538
pcaac361.smc181	-0.036873879	0.031893654	0.249631593
pcaec421.pcaac383	0.012219178	0.010568888	0.249633611
smohc161.pcaac402	-0.081436483	0.070448968	0.249707725
pcaac341.lysopcac182	-0.020471867	0.01771044	0.249725397
smohc241.pcaac320	0.078802167	0.068179765	0.249774688
pcaec443.pcaec405	0.154970578	0.13409462	0.249823256
pcaac420.smc202	0.074256176	0.064265796	0.249915724
c141.lysopcac180	-0.069970082	0.06056055	0.24994841
lysopcac170.prolinr	-0.062749885	0.054312805	0.249961287
kynurenine.pcaec382	-0.011175753	0.009673145	0.249963019
histidine.pcaac240	-0.017602339	0.015237338	0.250014917
pcaec424.pcaac420	0.121896747	0.105520155	0.250019902
lysopcac280.lysopcac181	0.032553687	0.028183011	0.250067541
arginine.pcaec380	0.064427495	0.055781364	0.25010084
smc261.pcaac380	-0.076133713	0.065917448	0.250106804
pcaec421.pcaac402	-0.255315754	0.22106474	0.25012687
c12.smc160	-0.11321109	0.098033035	0.250172029
pcaac360.lysopcac181	0.03584854	0.031047363	0.250247765
c18.pcaec403	0.048251251	0.041792027	0.250282072
pcaec386.smohc161	0.056154208	0.048643859	0.250348135
smc161.c162	0.111325524	0.096443183	0.250381865
smc180.pcaac383	0.005291921	0.004584789	0.250413571

pcaec425.pcaac383	0.006461476	0.00559845	0.250446234
smc161.pcaec401	0.072487733	0.06281263	0.250496581
prolinr.pcaac281	0.001413107	0.001224524	0.250507317
pcaec444.pcaac406	-0.04384545	0.037994445	0.250510823
pcaac401.alanine	-0.018781856	0.016276705	0.250546013
smohc222.smohc221	0.072613648	0.062953315	0.250733146
putrescine.pcaac425	-0.095975933	0.083207624	0.250733616
pcaac425.h1	0.011850819	0.010274672	0.250754059
pcaac406.pcaec445	0.095546048	0.082838604	0.250754529
pcaec340.taurine	0.074908891	0.064951189	0.250791123
alphaaminoadipicacid.leucine	0.017961085	0.015573825	0.250800865
smohc241.pcaec406	0.007370055	0.00639079	0.250823792
pcaec341.lysopcac182	-0.021869355	0.018964002	0.250834851
pcaec360.kynurenine	-0.015287315	0.013256704	0.250846094
pcaac425.lysopcac181	0.038978687	0.033812875	0.251009561
putrescine.valine	-0.087475029	0.075900278	0.251122922
pcaec342.pcaac240	-0.018418191	0.015981423	0.251132903
creatinine.pcaec301	-0.08105849	0.070335768	0.25114297
lysopcac280.serine	-0.034342103	0.029799656	0.251150188
pcaec405.pcaac365	-0.069137837	0.060001508	0.251217534
smc160.smohc221	0.051895363	0.045040348	0.251246642
pcaec343.pcaac426	0.095560911	0.082954007	0.25133779
smc202.leucine	-0.030831062	0.026764446	0.251351572
lysopcac160.pcaec342	0.133935966	0.116271942	0.251360193
pcaec444.spermidine	-0.078671641	0.068297097	0.251367061
pcaec404.pcaac383	0.007812577	0.006782387	0.251371673
pcaac404.lysopcac280	0.008336707	0.007239978	0.251539444
smc202.pcaec402	-0.06798341	0.059040331	0.251542837
pcaac405.pcaac344	-0.073694463	0.064000172	0.251543317
pcaac361.lysopcac181	0.034465166	0.029932633	0.251563149
smohc221.c182	0.040252873	0.034960378	0.251579121
pcaac363.kynurenine	-0.012080057	0.010492561	0.251615292
lysine.glycine	0.056930684	0.0494518	0.251640471
asparagine.pcaac406	-0.02430473	0.021117855	0.251774443
serotonin.pcaec424	-0.005173951	0.004495631	0.251784556
c51.pcaac322	0.094230597	0.081881691	0.251813405
pcaac342.leucine	-0.007201714	0.00625886	0.251883528
pcaac323.c182	-0.061209707	0.053202114	0.251936987
citruline.c16	-0.05078522	0.044144803	0.251973558
pcaec322.pcaac320	0.175830522	0.152870767	0.252069125
smc181.pcaac384	0.080831414	0.070283958	0.252118961
pcaec405.c182	-0.039615142	0.034449778	0.25217241
pcaac402.pcaec364	-0.006478603	0.005634287	0.25220764

pcaac422.asparticacid	0.044699825	0.03887521	0.252217753
pcaac380.pcaac341	0.007325651	0.006371378	0.252239754
pcaec384.h1	0.002187025	0.001902293	0.25227939
pcaac403.lysopcac182	-0.108264817	0.094172082	0.252291551
pcaac343.pcaec406	0.007974307	0.006937318	0.252360921
pcaac421.pcaac341	0.004988634	0.004340554	0.252431505
phenylalanine.pcaec340	-0.057124339	0.049730109	0.252686615
asymmetricdimethylarginine.pca	-0.066989086	0.058339076	0.25285761
smohc241.pcaac360	-0.035997701	0.031352238	0.252899242
c10.smohc141	-0.046800737	0.040774766	0.25305694
pcaac385.pcaec444	0.109871508	0.095742794	0.253146361
pcaec365.pcaac360	0.05744664	0.050063026	0.253180565
lysopcac281.pcaac363	0.011276353	0.00983032	0.253339936
pcaec385.c51	0.036457163	0.031786958	0.253412936
c142.pcaec406	-0.0064306	0.005607438	0.253463904
smc241.pcaac403	-0.007905424	0.006895727	0.253618312
glutamine.pcaec364	0.015247059	0.013299904	0.253626495
pcaec322.isoleucine	0.034931774	0.030474796	0.253689331
pcaac362.pcaec424	0.004155975	0.003625804	0.253701583
pcaac384.pcaec406	0.019577681	0.017081445	0.253736322
acetylornithoine.smohc161	0.023755751	0.020729557	0.253799326
pcaac240.pcaac422	-0.037873953	0.033053058	0.253853455
c182.c51	0.073297616	0.063969551	0.25386699
smc240.pcaac300	-0.070204969	0.061277076	0.253917845
pcaec363.pcaac364	-0.05553192	0.048473002	0.253947274
smc261.pcaec406	0.008887369	0.007757737	0.253952302
pcaac341.smohc141	-0.025370026	0.022148012	0.254008883
pcaec424.pcaac383	0.006636556	0.005793721	0.254009917
pcaac323.c141	-0.033102304	0.028899945	0.254036041
pcaac343.smohc161	-0.02894517	0.025276917	0.254154877
arginine.pcaac383	-0.00773155	0.006752462	0.254206662
lysopcac170.tyrosine	-0.088345155	0.077159184	0.254216797
leucine.smohc141	-0.015837641	0.013833774	0.254266099
pcaac380.asparticacid	-0.058809659	0.051370006	0.254277814
isoleucine.pcaec380	0.048699112	0.042538591	0.254279013
c51dc.threonine	-0.079618403	0.069549208	0.254297153
pcaec362.pcaac240	-0.013081924	0.011427555	0.254300376
pcaac366.pcaac341	0.012708583	0.011103833	0.254402805
c18.pcaec380	-0.021215856	0.018541523	0.254521519
glycine.pcaac322	-0.071200462	0.062232536	0.254575773
c10.pcaac386	0.028059499	0.024528284	0.254633011
pcaec361.pcaec406	0.009779755	0.008550152	0.254696764
pcaec386.pcaec424	0.012506233	0.010934364	0.25471983

prolinr.glycine	-0.021989066	0.019225729	0.254729645
c141.pcaec362	0.023197029	0.020283194	0.254760075
pcaac362.pcaac421	-0.041507132	0.036299369	0.254838867
pcaac323.pcaac365	-0.061445604	0.053738948	0.254862824
lysine.pcaac380	0.108395813	0.09481407	0.25493026
spermidine.asparticacid	-0.14441387	0.126331419	0.254976197
pcaac344.alanine	-0.045946949	0.040196679	0.255009983
ornithine.smohc241	0.09029391	0.079003823	0.255071129
pcaac360.pcaec424	0.016701954	0.014614372	0.255096407
ornithine.spermidine	-0.082804792	0.072469125	0.255188627
pcaac342.pcaac380	0.090321388	0.079049726	0.255201935
pcaec301.pcaec383	-0.092977902	0.081378274	0.255222593
lysopcac161.smc202	0.06545926	0.057300482	0.255286252
pcaec443.pcaec361	0.026962819	0.023606422	0.255370877
smohc141.pcaac383	0.007216672	0.006318442	0.255379707
pcaac362.pcaac422	-0.017642322	0.01544646	0.255379969
pcaec384.pcaac383	0.005407537	0.004734729	0.255404308
pcaec342.pcaac344	0.136491425	0.119516504	0.255433646
pcaac281.phenylalanine	0.054168512	0.047432121	0.255437049
c0.pcaac422	-0.042289277	0.037031883	0.255458727
pcaac320.pcaec423	-0.007972699	0.006982064	0.25549456
pcaac366.c141oh	-0.094997122	0.083205669	0.255564308
pcaec300.smohc241	0.087360603	0.076524712	0.255611821
c51dc.lysopcac181	-0.096351413	0.0844054	0.255640206
pcaac386.pcaac341	0.013170667	0.011537943	0.255649455
pcaec443.pcaec423	0.029806506	0.026114148	0.255697574
pcaec404.pcaac381	0.046309549	0.04057974	0.255778081
pcaec425.pcaec403	0.052908801	0.046363359	0.255787092
pcaac363.pcaec406	0.00556117	0.004873201	0.255788401
pcaec341.pcaec301	-0.05550439	0.048644469	0.255851546
putrescine.smohc141	0.010915635	0.009566565	0.255852543
kynurenine.pcaec380	-0.05802521	0.050856325	0.255875642
pcaec423.pcaec362	0.015170851	0.013296681	0.255881138
pcaec383.c182	-0.056233849	0.049301352	0.2560203
pcaac402.pcaac403	0.018844025	0.01652215	0.256055026
pcaec342.pcaac364	-0.061864295	0.054242052	0.256058469
c14.pcaec406	-0.007042523	0.006175913	0.256142229
methioninesulfoxide.pcaac365	-0.101396978	0.088933071	0.256213459
c14.pcaec403	0.031314061	0.02746859	0.25627742
pcaec363.pcaac403	-0.004807438	0.004217113	0.256282463
methioninesulfoxide.pcaec364	-0.00595963	0.005229196	0.256406766
pcaac420.glutamicacid	0.084509152	0.074177136	0.256570981
pcaec403.pcaac383	0.011586091	0.01017035	0.256606539

asparticacid.transhydroxyproline	0.059861316	0.05256099	0.256735532
pcaec365.pcaec424	0.016626931	0.014599972	0.256760248
pcaac403.c182	-0.073567632	0.064599257	0.256761113
lysopcac180.c182	-0.041694644	0.036612538	0.256770734
c51dc.lysine	-0.103451004	0.090845641	0.256792333
pcaec381.pcaec364	-0.007290692	0.006402577	0.256810484
pcaec365.pcaec444	0.120692696	0.106008816	0.256892209
pcaec383.pcaec364	-0.007154394	0.006284155	0.256906449
pcaac320.lysopcac182	-0.025092379	0.022046838	0.257048645
phenylalanine.pcaec362	-0.013403297	0.011778286	0.257120591
pcaec362.pcaac341	0.016822416	0.014782952	0.257123147
pcaac406.c102	0.035571938	0.031263271	0.25718211
lysopcac160.smc160	-0.081522045	0.071650636	0.257201847
glutamicacid.lysopcac181	0.096791109	0.085079446	0.257250045
smc241.pcaec362	0.018158879	0.015962353	0.257270406
kynurenine.smohc161	0.028205043	0.024795128	0.257305016
pcaac381.ornithine	0.040732184	0.035808631	0.257316379
pcaec406.lysopcac181	0.0435973	0.038330057	0.257348927
pcaac281.c142	0.061936026	0.054462107	0.257426618
pcaec402.alanine	-0.014736709	0.01295917	0.257454508
lysopcac161.smc160	-0.055401554	0.048721895	0.257482372
c2.pcaec405	0.074309912	0.065363933	0.257579659
pcaac364.pcaac361	-0.048462519	0.042638886	0.25769794
phenylalanine.pcaec365	0.033988362	0.029904839	0.257710348
kynurenine.lysopcac182	-0.05708396	0.050233491	0.257784545
pcaec322.pcaac281	0.002256402	0.001985858	0.25784183
pcaec421.pcaec364	-0.007506085	0.006606379	0.257861719
pcaec364.pcaac341	0.010873834	0.009570998	0.257888457
c51.c10	0.009346246	0.008227192	0.257932001
ornithine.c142	-0.087674217	0.077189473	0.258010265
pcaac341.pcaac364	-0.094683914	0.083363076	0.25802264
phenylalanine.tyrosine	-0.097893114	0.086192117	0.258042146
pcaec424.c14	-0.074706896	0.065783957	0.258090036
creatinine.glutamicacid	0.064523753	0.056824292	0.258150169
glutamicacid.pcaac383	0.00990951	0.008727146	0.258156371
c5.pcaac366	0.068896385	0.060679533	0.258184399
pcaec321.pcaac383	0.005725894	0.005043442	0.258225752
pcaac420.pcaac406	-0.059121588	0.052075508	0.258229828
smc261.histidine	-0.062293677	0.054873987	0.258268149
smc241.pcaac362	0.058634544	0.051655774	0.258314817
pcaec422.pcaec342	0.131003157	0.115417418	0.258341307
pcaec362.pcaac403	-0.004172956	0.003676498	0.258342256
pcaec422.pcaec424	0.007782481	0.006856995	0.258369888

methionine.pcaec322	0.046928432	0.041347783	0.258370328
smohc221.c51	0.046275987	0.040774632	0.258390161
pcaec386.pcaac360	0.076923604	0.067781085	0.258406014
pcaac403.pcaec364	-0.006680219	0.005886468	0.258422561
pcaec340.pcaac365	-0.080424565	0.070874151	0.258460853
pcaec342.pcaec424	-0.034610676	0.030510057	0.258606853
pcaec363.pcaac380	0.120273458	0.106036887	0.258666048
pcaec402.pcaec423	-0.032936868	0.029038742	0.258675139
pcaec301.lysopcac204	-0.048358303	0.042637641	0.258704183
smohc241.c142	0.056956526	0.050227139	0.25878368
pcaec362.smohc141	0.045702116	0.040302873	0.258788868
pcaac342.pcaec363	-0.040000704	0.035277123	0.258817064
pcaac300.pcaec340	0.111156431	0.098048324	0.258904433
arginine.c3	-0.062238516	0.054909092	0.258991162
asymmetricdimethylarginine.smc	-0.11382187	0.100442155	0.259106082
glutamine.lysopcac180	0.079156167	0.069868613	0.259222963
pcaac343.pcaac341	-0.015383762	0.013581531	0.259319937
smc160.lysopcac280	-0.011020381	0.009729847	0.259345304
pcaac405.pcaec362	-0.023314402	0.020584948	0.259362862
pcaec300.pcaec424	0.007653857	0.006757818	0.259363593
c181.c8	-0.081098039	0.071607594	0.259388226
pcaac323.c181	-0.022311852	0.019704859	0.259485382
pcaac424.pcaac402	-0.196280056	0.173360338	0.259524538
c0.asymmetricdimethylarginine	-0.007319336	0.006465702	0.259601429
pcaac385.pcaac361	-0.080861177	0.071439266	0.259659236
lysopcac203.prolinr	-0.029322601	0.02591045	0.259741742
pcaac406.lysopcac181	0.024925496	0.022026765	0.259779347
pcaec321.lysopcac160	-0.015526764	0.01372141	0.259791238
pcaec364.pcaac360	0.04542212	0.040141075	0.259795395
asymmetricdimethylarginine.pca	-0.014123602	0.012485462	0.259945615
pcaac366.transhydroxyproline	0.049696175	0.043936257	0.25999034
pcaac366.asparagine	0.015761285	0.013934564	0.259992152
pcaec446.acetylornithoine	-0.068197309	0.060294835	0.260004299
pcaec383.pcaac365	-0.07387895	0.065324237	0.260048814
smc161.c101	-0.012670379	0.011203371	0.260054797
pcaac384.c16	0.042004021	0.037141219	0.260061546
pcaac420.lysopcac182	-0.031727316	0.028057894	0.260123194
pcaac360.lysopcac160	0.11069061	0.09789435	0.260150641
citruline.pcaac365	0.049213716	0.043529176	0.260202459
pcaec425.pcaac384	0.080804449	0.071488153	0.260316634
c181.methionine	-0.032117122	0.028414238	0.260317289
smc241.pcaac323	0.089267679	0.078977019	0.26032504
pcaac380.lysine	0.139568835	0.123480684	0.260329493

pcaac426.c8	-0.055204558	0.048844445	0.260361906
pcaac322.c8	-0.04680503	0.041415458	0.26039435
c3.pcaec361	-0.009718039	0.008599167	0.260402891
methioninesulfoxide.lysopcac18(	0.105656345	0.093512587	0.260508573
pcaac323.c102	0.03277042	0.029005799	0.260539537
pcaac401.pcaec364	-0.007262525	0.006428227	0.260540395
glutamicacid.arginine	-0.13580748	0.120206518	0.260541174
pcaac420.pcaac383	0.0090428	0.008004369	0.260562854
pcaac281.pcaac320	-0.062962657	0.055734978	0.260585387
smc180.putrescine	-0.031596299	0.027977176	0.260719642
pcaec342.pcaec421	0.004050784	0.003587259	0.260780889
pcaec301.h1	-0.004790195	0.004242237	0.260800614
pcaec386.pcaec406	-0.036308039	0.032156278	0.260823801
smc161.pcaac366	-0.043163439	0.038229469	0.260844839
pcaac323.smohc161	-0.026567791	0.023534441	0.260917193
c4.smohc141	-0.020230893	0.017921683	0.260933905
c4.pcaec361	-0.013138612	0.01163896	0.260934941
pcaac366.pcaec404	-0.02571375	0.022778869	0.260937188
spermidine.pcaac240	-0.052186476	0.046233223	0.260969381
citruline.smohc141	0.011303266	0.010014566	0.261004361
alanine.lysopcac204	-0.024368254	0.02159162	0.261039992
pcaec385.pcaec424	0.011102941	0.009838749	0.261084839
c51.pcaac422	0.046133888	0.040882271	0.261099172
pcaac323.pcaec406	0.008382142	0.007428736	0.261148334
pcaec321.pcaec423	-0.008793306	0.007793573	0.261175076
methioninesulfoxide.pcaec424	-0.011555129	0.010241435	0.261176822
glutamicacid.pcaec385	0.113432504	0.100569201	0.26133147
pcaac381.pcaec404	0.02579316	0.022868728	0.261342371
pcaac360.smc202	0.0531984	0.047170552	0.261380558
lysopcac280.pcaec424	0.003357218	0.002977656	0.261514571
c51dc.asymmetricdimethylargini	0.028947581	0.025675048	0.261518951
pcaac281.pcaac421	-0.09596133	0.085126038	0.261592385
pcaec300.pcaec383	-0.065084538	0.057748763	0.261700293
citruline.pcaec425	0.016543966	0.014679637	0.261712144
c14.lysopcac280	0.01415733	0.012562335	0.261726707
asparagine.putrescine	-0.045900041	0.040731791	0.261761051
pcaec362.serotonin	-0.150175178	0.133289113	0.261844243
c4.pcaec362	-0.017357316	0.015406887	0.261883408
pcaac344.pcaec406	0.016014193	0.014214838	0.261888342
histidine.alanine	-0.047483024	0.042149131	0.261902999
lysine.pcaec404	-0.04768053	0.042326471	0.261925664
acetylornithoine.pcaac425	0.072428053	0.064300621	0.261966531
smohc161.pcaac403	-0.006777079	0.006016753	0.261978859

pcaec421.pcaec406	0.084971741	0.075449511	0.262046965
lysopcac280.pcaac426	0.087154702	0.077390246	0.262061715
pcaac386.kynurenine	-0.009776262	0.008682011	0.262118674
c4.pcaac383	-0.002894349	0.002570424	0.262125622
c101.smc240	0.045590259	0.040488355	0.262130253
pcaac361.pcaac421	-0.051431832	0.045676219	0.262130336
pcaac405.pcaec406	0.007675755	0.00681758	0.262186117
pcaec301.pcaac426	0.089116371	0.079156589	0.262208445
c181.phenylalanine	0.05841826	0.051892701	0.262239156
c0.lysopcac280	-0.002871445	0.002551023	0.262300536
c10.c16	0.052721897	0.046839899	0.262312718
pcaec404.pcaec380	0.086348903	0.076728102	0.26239224
pcaac342.pcaec424	0.006403086	0.00568982	0.262404894
smc240.pcaec405	0.067494072	0.059979606	0.262436376
c141oh.pcaec404	-0.03444422	0.030610073	0.262447346
pcaec424.pcaac341	0.005606384	0.004983671	0.26257701
pcaac300.c16	0.057130116	0.050796349	0.262687319
putrescine.pcaec322	0.051926817	0.046176964	0.262759822
pcaec341.pcaac403	-0.020227518	0.017989443	0.262805163
pcaec341.pcaac383	0.005065725	0.004506318	0.262920141
pcaec424.pcaec406	0.006126037	0.005450065	0.262965918
lysopcac281.pcaac323	-0.168336229	0.14979466	0.263071699
pcaac320.pcaec424	0.005207088	0.004633971	0.263115116
lysopcac203.pcaec365	0.025238277	0.022462554	0.263160001
c18.pcaac403	0.005565687	0.00495372	0.263174467
pcaec343.pcaac383	0.005909841	0.005261343	0.263292827
smc241.pcaac363	-0.009353364	0.008327753	0.263335685
pcaec401.smc202	0.049624659	0.044196858	0.263482184
c101.pcaec365	-0.020275516	0.018058157	0.263490579
pcaec402.smohc241	0.081186276	0.072309688	0.263504085
pcaec386.pcaac403	-0.005447919	0.004853472	0.263622293
lysopcac204.histidine	-0.082010594	0.073064986	0.263641417
pcaac363.smohc141	-0.047259895	0.04210823	0.263679598
c101.pcaec423	-0.016618925	0.014807637	0.263689023
lysopcac161.c10	0.013552776	0.012075715	0.263690934
pcaec422.pcaac341	0.006205406	0.00552991	0.263760187
methionine.taurine	-0.037721809	0.033622332	0.263855911
pcaac404.pcaac381	0.04416026	0.039364557	0.263897981
pcaec422.alanine	-0.025113923	0.022391215	0.263995799
pcaac323.alanine	-0.020153732	0.017968983	0.26400118
asparagine.pcaec380	-0.085117648	0.075893764	0.26402139
lysopcac170.citruline	0.038293349	0.034146013	0.264054415
glycine.pcaec363	0.016100688	0.014357122	0.264061316

pcaec340.lysopcac203	0.01378148	0.012291453	0.264153736
pcaac403.pcaec401	0.076189532	0.067953754	0.26416553
c8.pcaac386	0.026276691	0.023444378	0.264329641
c0.pcaec423	0.044466983	0.039684201	0.264452202
lysopcac203.c3	-0.05779888	0.051589799	0.264522767
lysine.pcaec444	0.196101048	0.175068957	0.264615637
pcaac425.prolinr	-0.053916061	0.048135167	0.264632167
glutamicacid.pcaec322	-0.086860369	0.077553604	0.264671581
pcaac384.pcaac380	0.064022923	0.057165264	0.26468954
pcaec402.pcaec322	-0.092385233	0.082496501	0.264729244
smc161.putrescine	-0.037659004	0.033629687	0.264752276
pcaec405.pcaac361	0.05230074	0.046705226	0.264756264
pcaec385.pcaec301	0.099954654	0.089264243	0.264774732
asymmetricdimethylarginine.pca	-0.004681955	0.004182071	0.264872931
histidine.pcaac361	0.049720988	0.044416503	0.264917223
acetylornithoine.smc202	-0.066752767	0.059639244	0.264981017
pcaec420.smohc221	0.139391889	0.124559126	0.265063371
smc241.valine	-0.099139042	0.088593411	0.265083806
pcaac281.pcaec363	-0.038136787	0.034081393	0.265101913
creatinine.pcaac321	-0.034076943	0.030456333	0.265149834
smc260.pcaac406	-0.038304117	0.034235909	0.265171104
isoleucine.histidine	0.107331888	0.095936948	0.265193812
c12.pcaec406	-0.006846263	0.006119447	0.265195363
pcaec444.pcaec424	0.011679165	0.01044081	0.265265306
serine.smc161	0.087535246	0.078281963	0.265436588
pcaac341.pcaac422	-0.035721486	0.031949591	0.265499076
smc181.pcaec360	0.010881413	0.009733118	0.265532922
arginine.pcaec423	0.021226444	0.018987585	0.265561192
c16.pcaec420	-0.041011398	0.03668743	0.265583368
smc260.c16	0.06464064	0.057825909	0.265587855
c16.asparagine	0.014339612	0.01282829	0.265603791
asymmetricdimethylarginine.alpl	0.011776693	0.01053679	0.265662528
asparagine.c16	-0.068126738	0.060964263	0.265742248
alanine.pcaec406	0.004151276	0.003714846	0.265743854
glycine.h1	0.004040556	0.003615792	0.265747233
pcaac342.alanine	-0.018238254	0.016321155	0.265753066
pcaac342.prolinr	0.033413231	0.02990366	0.265795003
asparagine.transhydroxyproline	-0.029029102	0.025985438	0.265894242
pcaac422.pcaec405	-0.13049405	0.116815124	0.265907301
pcaac240.smohc161	-0.062389215	0.055849636	0.265910005
asparagine.leucine	-0.00846665	0.007579577	0.265934686
pcaac300.pcaec401	0.09322148	0.083455723	0.265942113
pcaac381.pcaec422	-0.041914306	0.03752399	0.265949351

c101.pcaac380	-0.050425307	0.045145151	0.265966694
pcaec402.pcaec342	0.100241869	0.089758448	0.266036408
pcaec444.pcaec404	0.024121706	0.021602286	0.266108207
pcaac321.pcaec343	0.120920511	0.108294761	0.266125497
pcaec322.c102	0.035607728	0.031890066	0.266129534
lysopcac140.lysopcac170	0.024525542	0.021970248	0.266244806
pcaac320.pcaac383	0.006086518	0.005452723	0.266275644
pcaac424.pcaac385	-0.026339206	0.023597703	0.26630039
pcaac320.pcaec406	0.010134013	0.009079772	0.266329356
c51dc.c141oh	-0.126104778	0.112987117	0.266333636
c142.pcaac403	0.00597656	0.005355071	0.266351731
lysopcac182.smohc141	0.012217351	0.010949297	0.266456171
pcaac365.pcaec446	-0.066308069	0.059430292	0.266491581
pcaec362.pcaec424	0.010113147	0.009064171	0.266491901
pcaec402.pcaac421	-0.057062373	0.051150191	0.266552905
serine.pcaec385	-0.053729423	0.048165084	0.266577813
c101.arginine	0.035236071	0.031588804	0.266605821
pcaac403.creatinine	-0.014681297	0.013162246	0.266627633
alphaaminoadipicacid.c141	-0.027213501	0.024398462	0.266641266
alphaaminoadipicacid.c51dc	-0.163161096	0.146285284	0.266647834
glutamicacid.pcaac403	0.009525878	0.008542301	0.26674191
pcaac403.c2	0.142422482	0.127728543	0.266785286
lysopcac181.lysopcac180	-0.140347685	0.125875901	0.266815906
pcaac360.c181	-0.027984041	0.025099411	0.266833193
pcaec380.pcaac421	-0.150232275	0.134752286	0.266854891
pcaec360.pcaac360	-0.055929969	0.050167062	0.266856196
lysopcac161.smc241	-0.084425246	0.075727498	0.26686407
glycine.pcaac384	0.081422207	0.073044599	0.266934205
pcaec421.pcaac281	-0.004765006	0.004274826	0.266944929
phenylalanine.transhydroxyproli	-0.04537948	0.040713688	0.266973337
lysopcac281.pcaec406	0.004339892	0.003893763	0.266983917
methioninesulfoxide.creatinine	-0.01875528	0.016827678	0.266994921
isoleucine.c51dc	-0.182373445	0.16363705	0.267016216
pcaec422.pcaac300	-0.067067013	0.060177973	0.267025591
glycine.pcaec386	0.015328653	0.013754866	0.267051665
asymmetricdimethylarginine.pca	0.005802042	0.005206567	0.267071706
c51dc.pcaac420	-0.090068809	0.080834279	0.267127071
pcaac321.asymmetricdimethylar	0.042292717	0.037957088	0.267133821
smohc161.pcaec424	0.012642446	0.011346576	0.267140827
pcaec406.pcaac240	-0.022769278	0.020437277	0.267184775
lysopcac204.pcaec380	-0.081839567	0.073464334	0.267228035
c181.smc202	-0.056818259	0.051029221	0.267466866
smc180.glutamine	-0.017601056	0.015807928	0.26747258

h1.smohc141	-0.034703022	0.031171719	0.267535344
pcaac383.c14	0.042046397	0.037768218	0.267539999
c142.arginine	0.034410371	0.030910652	0.267563156
lysopcac181.valine	-0.101894172	0.091540056	0.267610329
pcaec446.pcaec383	-0.070169384	0.063049689	0.267690893
pcaec362.phenylalanine	0.048472049	0.043556103	0.26771542
pcaac403.lysopcac160	0.065344894	0.058720113	0.267734706
c101.c3	0.051878805	0.046628041	0.267824621
arginine.c181	0.020717715	0.01862431	0.267913605
pcaec301.pcaac420	-0.083571011	0.075128857	0.267927644
c181.pcaac403	0.00527267	0.004740257	0.267949761
pcaac424.pcaac363	-0.017155958	0.015424167	0.267966722
pcaac380.lysopcac161	0.119575902	0.107515095	0.268009529
pcaac426.smohc141	-0.014081801	0.012662984	0.268066667
lysopcac280.pcaac361	0.08892068	0.07999412	0.268261315
lysopcac140.c16	-0.11278767	0.10148212	0.268340983
c18.c8	-0.067796697	0.061001899	0.268348567
c181.pcaec360	-0.009603047	0.00864078	0.26835851
c162.pcaac385	0.043103068	0.038784109	0.268360428
pcaac402.pcaac341	0.007823733	0.007040132	0.268383652
pcaec322.pcaac406	-0.046094049	0.041478062	0.268391108
lysopcac160.lysopcac280	0.0165054	0.014852739	0.268398706
pcaac361.h1	0.002507971	0.002256951	0.268419722
pcaec340.pcaec424	0.00524149	0.004717637	0.268496637
isoleucine.glutamine	0.030067379	0.02706376	0.26852164
smohc221.pcaac406	-0.049202948	0.044296755	0.268618452
pcaac383.pcaac403	-0.005868836	0.005283884	0.26864091
smohc141.pcaec360	-0.011162138	0.010049878	0.268654243
c142.lysopcac181	-0.06196256	0.055788373	0.268655269
pcaac343.smc181	-0.010640295	0.009581187	0.268711528
pcaac361.pcaec362	-0.046415043	0.041800275	0.268771625
pcaec363.h1	0.006272303	0.005649609	0.26884951
smc241.smc240	0.09005077	0.081120168	0.26890445
pcaec444.pcaec322	0.065091778	0.058637289	0.268911402
smc161.pcaec424	0.009153165	0.008246894	0.268989721
pcaac384.smc202	0.058147392	0.052400818	0.269087092
pcaec382.pcaec364	-0.005682251	0.005120689	0.269087265
lysopcac204.lysopcac182	0.042096512	0.037939404	0.269127216
pcaac344.methionine	-0.032890624	0.029644618	0.269159432
ornithine.lysopcac180	0.053681377	0.048387236	0.269196115
pcaac300.tyrosine	0.058408445	0.052648983	0.269204004
pcaac403.c14	0.078670798	0.07091485	0.269214197
smohc222.c51dc	-0.149745929	0.134987134	0.269229255

pcaac405.c181	-0.014901651	0.013434417	0.269280926
pcaac383.smc161	-0.057252848	0.05162439	0.269361503
lysopcac182.pcaac384	0.052087074	0.046968158	0.269378775
c141.pcaec445	0.055791665	0.050309723	0.26938869
pcaac323.pcaac406	-0.035280374	0.031814317	0.269396168
pcaac343.pcaac406	-0.050389775	0.045450121	0.269509467
pcaec322.c51	0.068986159	0.062223628	0.269510274
leucine.pcaec402	0.046232668	0.041704868	0.269559053
pcaac365.pcaac344	-0.10968846	0.098949641	0.269576053
pcaec380.asparticacid	-0.049771421	0.044901111	0.269602134
pcaac361.pcaec424	0.003539928	0.003193938	0.269662473
asparagine.smc240	-0.116951383	0.1055452	0.269773496
smc181.lysopcac204	0.047462042	0.042851776	0.269981311
smohc222.pcaac322	-0.062281702	0.056233108	0.269991429
c3.pcaec342	-0.084225004	0.076050471	0.270023547
pcaac385.pcaec425	0.010352657	0.009348359	0.270048449
glutamine.pcaac360	-0.043019768	0.038847329	0.270058935
pcaac426.leucine	-0.015196031	0.013723244	0.27009574
asparticacid.pcaec321	0.025293632	0.022845476	0.270164245
pcaac342.c2	-0.090022423	0.081309377	0.27016527
c181.c5	-0.00791341	0.007147528	0.270167715
pcaec405.pcaac383	0.007852744	0.007093023	0.270187179
pcaac405.asparticacid	0.038967718	0.03519973	0.270214048
lysopcac182.pcaec384	0.051252082	0.046298977	0.270242082
pcaec322.pcaec423	-0.011070815	0.01000186	0.270287398
lysopcac181.c101	-0.010388494	0.009385437	0.270288248
pcaec365.pcaac300	-0.072372965	0.065385157	0.270289223
pcaac383.phenylalanine	-0.068132521	0.0615605	0.270338498
alphaaminoadipicacid.pcaac405	-0.065510008	0.059192501	0.270350971
c5.pcaec385	0.105446066	0.095279257	0.270360791
smc202.c101	0.027643459	0.024979781	0.270391828
pcaac363.pcaec322	0.058590862	0.052945413	0.270394264
pcaec360.pcaac422	-0.073568693	0.066480377	0.270396429
leucine.smc180	-0.013913761	0.01257425	0.270437212
pcaec385.pcaac366	-0.03762242	0.034007474	0.270536152
pcaac364.pcaac402	-0.066280366	0.059919043	0.270593617
smc161.asparagine	0.017720719	0.016021039	0.270625869
pcaac401.asymmetricdimethylar	0.04491877	0.040611474	0.270638568
pcaac344.pcaac321	-0.065158832	0.058920762	0.270720028
c3.pcaac403	0.009453497	0.008550602	0.270839908
histidine.c8	-0.069099287	0.062502508	0.27086152
pcaac381.pcaec405	0.119558263	0.108145311	0.270866153
pcaac405.pcaac360	0.038000914	0.034375745	0.270899076

smc180.c101	-0.048858682	0.044222557	0.271167157
histidine.taurine	-0.032324175	0.029263585	0.271274801
smc160.pcaac281	0.003020871	0.002734896	0.271284202
pcaec420.pcaec364	-0.017854206	0.016167397	0.271384097
pcaac385.pcaec301	0.084744013	0.076751236	0.27146847
pcaac362.pcaac363	-0.024704324	0.022376893	0.271523923
pcaec404.c5	-0.041333474	0.037447735	0.271630318
pcaac405.lysopcac181	0.029712202	0.0269197	0.271643209
pcaec401.lysopcac182	-0.068676694	0.062225193	0.271666834
pcaec300.pcaac366	0.034156301	0.03094787	0.271670188
c4.c101	0.050681053	0.045922219	0.271689214
pcaec421.pcaac380	0.064591423	0.058530638	0.271723455
pcaac323.c3	0.050852667	0.046095141	0.27186963
pcaac320.pcaec425	0.021909683	0.01985998	0.271871069
pcaac425.c101	0.013932838	0.012633016	0.272008167
c12.pcaec403	0.026073857	0.023642192	0.272024641
smc180.pcaec424	0.006842997	0.006204889	0.272030387
smc160.pcaec380	-0.037961609	0.034424974	0.272075799
spermidine.pcaac300	-0.255415494	0.231631774	0.272099852
pcaac362.pcaec446	-0.047490647	0.043083998	0.272272426
creatinine.pcaec403	0.033844227	0.030708642	0.272347292
pcaec382.pcaec444	-0.260667294	0.236533968	0.272381322
histidine.smohc221	-0.068146569	0.061839802	0.272400146
methionine.smc160	0.103007809	0.093478027	0.272416951
smc240.pcaec423	-0.021141839	0.019186809	0.272439605
pcaec342.pcaec382	-0.019682724	0.017863347	0.272458976
lysopcac281.pcaac426	0.091361165	0.082920963	0.272486557
smohc141.pcaec380	-0.043082659	0.039111779	0.272599086
c101.c2	0.062683945	0.056917812	0.272694515
pcaec365.pcaac403	-0.003597122	0.003266373	0.272715079
c12.c3	0.053772027	0.048844321	0.272876792
c5.c181	-0.035951636	0.032661619	0.272944279
glutamicacid.pcaec340	-0.079237589	0.07198646	0.272944839
pcaec363.smohc222	-0.005668071	0.005150459	0.273044995
c4.smohc222	0.012573723	0.011425512	0.273046232
arginine.pcaac403	-0.00795809	0.007231389	0.273047501
smc261.pcaac360	-0.048615456	0.044181824	0.273109455
pcaac341.pcaac360	0.053861581	0.048951321	0.273127095
lysine.pcaec364	-0.018340354	0.01666966	0.273164164
glutamine.c101	-0.01330163	0.012091085	0.273209698
lysopcac170.pcaec380	-0.040060458	0.036418685	0.273262384
pcaac385.pcaec424	0.00566301	0.005149284	0.27336263
pcaec446.prolinr	0.025181303	0.022902412	0.273476469

kynurenine.lysopcac203	-0.031146313	0.028328915	0.273498766
c18.pcaac341	0.009231373	0.008396981	0.27353565
pcaac300.pcaec424	0.004625438	0.004207998	0.273607969
pcaac384.pcaec424	0.004894712	0.004453134	0.273625614
c0.smohc141	-0.02615621	0.023797533	0.273645939
pcaac360.leucine	-0.017882658	0.016270986	0.273673079
pcaac401.glutamicacid	0.105948161	0.096400481	0.273677454
pcaac363.pcaac420	-0.079081028	0.07197613	0.273820887
c4.pcaac321	-0.017804221	0.016206051	0.273862743
pcaac343.pcaec382	0.019273866	0.017544068	0.273870688
serotonin.pcaac360	-0.029127032	0.026516157	0.273928864
pcaec422.serotonin	-0.123383872	0.112331902	0.273962263
smc241.asparagine	-0.010066132	0.009165305	0.274005828
c12.smohc141	-0.050702731	0.046167631	0.274029937
pcaac385.pcaec322	0.077153446	0.070269316	0.274144514
pcaac323.c18	-0.021756297	0.019816553	0.274180519
glycine.pcaec360	-0.009574954	0.00872134	0.27418426
pcaec382.pcaac403	-0.018254412	0.01662742	0.274195804
pcaec361.smohc221	-0.093364591	0.085044181	0.274201727
asparagine.smohc241	0.086549971	0.078839206	0.274215926
c10.pcaec363	-0.020385227	0.018572484	0.274302981
smohc241.pcaec343	-0.071892779	0.065502112	0.274320123
pcaac344.pcaec444	0.102189875	0.093129267	0.274439257
pcaec383.pcaec401	0.133675697	0.121854173	0.274559879
c102.pcaec362	0.028025277	0.025550473	0.274627085
c102.creatinine	-0.026004746	0.023715022	0.274761199
pcaec422.pcaac403	-0.014572838	0.013290671	0.274796257
pcaec444.pcaac383	0.00450659	0.004110642	0.274860943
pcaac364.smohc141	-0.07036011	0.064179981	0.27487361
smc241.lysopcac182	-0.057524109	0.052472951	0.274887387
c0.pcaec360	0.006974426	0.006362237	0.27490475
pcaac401.pcaec423	0.097352882	0.088817621	0.274958621
lysopcac280.pcaec403	-0.021085605	0.019237655	0.274975958
pcaac401.c18	0.051540763	0.047029922	0.275039049
asymmetricdimethylarginine.pca	-0.010140013	0.009252812	0.275052052
pcaec423.pcaac406	-0.053518225	0.048848218	0.275175088
ornithine.lysopcac170	-0.031218719	0.02850211	0.275301556
pcaac343.transhydroxyproline	0.023362244	0.021330658	0.275332112
smc241.smohc141	-0.026440801	0.02414156	0.275333247
pcaac385.spermidine	-0.079293757	0.07240468	0.275373871
pcaac365.c142	0.02888582	0.02637879	0.275420676
pcaec380.lysopcac161	0.072898047	0.06658642	0.27553031
pcaac364.leucine	-0.006233918	0.005695093	0.275607292

pcaec342.smohc222	-0.007403479	0.006765797	0.275765174
pcaac385.pcaec406	0.010401748	0.0095066	0.27580451
pcaac341.kynurenine	-0.020789975	0.019002601	0.275848771
pcaec321.pcaec363	-0.045558877	0.041644666	0.275878809
c2.pcaac281	0.017556512	0.016049996	0.2759342
lysopcac180.c51	-0.07770837	0.071043529	0.275956265
lysopcac140.putrescine	-0.042801347	0.039132597	0.275983247
pcaac342.pcaec406	0.005503203	0.005031699	0.276002959
c5.c51	0.05897823	0.053926377	0.276014376
pcaec404.pcaac322	-0.102260312	0.093503346	0.276025972
pcaec385.asparagine	-0.039128328	0.035777684	0.276026952
smc260.pcaac425	0.103408336	0.094556774	0.276044752
pcaec363.pcaec322	0.091053801	0.08326285	0.276062471
pcaac404.smc180	-0.014261789	0.013045514	0.276209999
pcaec422.c141oh	-0.108328059	0.099099559	0.276257968
smc260.putrescine	-0.019665687	0.017991881	0.276298373
glutamicacid.pcaec380	0.069632188	0.06370634	0.276304053
lysopcac181.pcaec423	-0.048505302	0.044379281	0.27632435
c2.pcaec404	-0.034262546	0.031349253	0.276342537
pcaac281.valine	-0.048536776	0.044415117	0.276400148
pcaac321.pcaec445	0.032868714	0.030079368	0.276428888
pcaec340.pcaec421	0.001656034	0.001515685	0.276488146
pcaec425.pcaec363	0.041843341	0.038299606	0.276519139
asymmetricdimethylarginine.vali	-0.06735995	0.061657935	0.276540351
c18.pcaac385	-0.047570719	0.04354751	0.276580428
pcaac362.pcaac403	-0.004118735	0.003770831	0.276634955
lysopcac181.pcaac420	-0.1104465	0.101122683	0.276660828
pcaec402.pcaac422	-0.076866166	0.070389825	0.27674677
ornithine.serine	-0.028152654	0.025781352	0.276759628
pcaac240.pcaac361	-0.027608571	0.02528327	0.276762908
pcaac386.pcaec402	0.040760643	0.03733603	0.276870605
pcaec402.pcaec321	0.040374017	0.036985297	0.276914695
smc181.pcaac344	0.075571157	0.06925432	0.277094928
serotonin.arginine	0.032350396	0.029646597	0.277099829
pcaec362.smc180	-0.010043887	0.009204943	0.277126298
pcaec361.lysopcac160	0.081791661	0.074964593	0.277157008
pcaac405.pcaac380	0.051634401	0.047325197	0.277163753
pcaac366.pcaac322	-0.098240552	0.090054181	0.277229627
pcaac322.transhydroxyproline	0.024879307	0.022807102	0.277250274
pcaac364.glycine	0.025896215	0.023742572	0.277315959
pcaac341.pcaec424	0.00425288	0.003899973	0.277411836
pcaac300.glutamicacid	0.050111264	0.045953611	0.277418196
lysopcac280.kynurenine	0.041400987	0.037967806	0.277440799

pcaec300.pcaac422	-0.015250475	0.013985891	0.277442953
pcaec401.smc160	-0.050323892	0.046151633	0.277449656
c18.lysopcac182	-0.022720151	0.020840591	0.277544366
methioninesulfoxide.pcaec322	-0.080237998	0.073611082	0.277615195
pcaac384.putrescine	-0.036678177	0.033650368	0.277636086
asparagine.pcaec364	0.024400694	0.022386671	0.277641879
ornithine.pcaec322	0.082594177	0.075781991	0.277674095
smc202.tyrosine	-0.093494892	0.085789802	0.277708466
putrescine.h1	0.002955776	0.002712741	0.277806634
methionine.pcaac426	-0.040050959	0.036759053	0.277822621
pcaac365.c16	0.043312621	0.039754373	0.277843593
pcaec382.pcaac385	-0.033570342	0.03081563	0.277893007
pcaec425.lysopcac203	0.02963564	0.027205688	0.277926185
c14.lysopcac180	-0.053516312	0.049134635	0.277988053
pcaec404.pcaec342	0.097015637	0.089073003	0.277991137
pcaec361.c101	-0.042555148	0.039071584	0.277996154
pcaac341.alanine	-0.028610122	0.026269766	0.278026554
c142.pcaac300	0.051378058	0.047186288	0.278138532
glutamicacid.pcaec425	-0.027387664	0.025154169	0.278157116
pcaac404.c141	-0.066521519	0.061099797	0.278182036
lysopcac204.smohc161	0.033499673	0.030772032	0.278223902
pcaec380.smc202	0.082877115	0.07613586	0.278266908
pcaac424.pcaac365	-0.0766487	0.070428755	0.278366719
pcaec422.pcaac344	-0.055938289	0.051416918	0.278533825
c3.ornithine	-0.032655478	0.030017208	0.278552986
c142.pcaac422	0.023263525	0.021385331	0.27858188
pcaac363.leucine	-0.010219315	0.009394423	0.278590542
pcaec445.serine	-0.010262639	0.009434826	0.278619755
lysopcac180.pcaec364	-0.017835548	0.016397917	0.278649935
pcaac281.smohc221	0.037463707	0.034445531	0.27867188
lysopcac182.lysopcac181	-0.077682595	0.07142837	0.278699305
prolinr.pcaec406	0.00284187	0.002613199	0.278722672
kynurenine.asymmetricdimethyla	0.028563152	0.026266013	0.278744499
lysopcac182.h1	-0.003596094	0.003306986	0.278759073
smohc241.pcaec382	0.022961132	0.021115852	0.278774577
pcaac300.c101	-0.009228684	0.008487959	0.278827689
pcaac341.pcaac380	0.065731594	0.060459551	0.278857721
c141.pcaac381	-0.013117696	0.012065735	0.278863736
pcaac366.creatinine	-0.013134384	0.012082369	0.278914616
smohc141.pcaac406	-0.042145574	0.038771145	0.278930356
smc260.smohc161	-0.016327608	0.015020405	0.278933004
pcaec423.pcaec382	-0.02271632	0.020898096	0.278943678
serotonin.pcaac381	0.039006495	0.035892467	0.279051297

pcaec406.c102	0.040088204	0.036888693	0.279062641
glycine.pcaac320	-0.015530347	0.014290964	0.279066706
pcaec364.pcaec363	-0.061935616	0.056994362	0.279078913
smc202.pcaec361	-0.008450242	0.00777668	0.279115973
pcaec382.lysopcac203	0.018874091	0.017369831	0.279120921
lysopcac161.smc240	-0.037050186	0.034105572	0.279237098
pcaac405.valine	-0.053839456	0.049563534	0.27926648
pcaac366.pcaac385	-0.057291893	0.052742144	0.279269796
arginine.pcaec404	-0.043193758	0.039770621	0.279354421
pcaec422.transhydroxyproline	0.046425294	0.042746616	0.279360709
methionine.pcaec364	0.012408333	0.011425158	0.279362576
pcaec425.pcaac341	0.004933657	0.004542877	0.279377228
pcaac420.asparagine	-0.01509284	0.013897735	0.27938936
lysopcac182.lysopcac204	-0.020887242	0.01923479	0.279426047
smc160.c141	0.029626329	0.027283143	0.279437309
lysopcac161.pcaac380	0.058284931	0.053675612	0.27944188
pcaac366.smohc221	0.067991994	0.06262108	0.279488251
pcaec386.pcaac323	0.125982347	0.116037544	0.279516973
alphaaminoadipicacid.pcaec382	-0.009481695	0.008733704	0.279543042
pcaec384.ornithine	0.043835521	0.040381428	0.279590466
smc260.c51	0.043567192	0.040144644	0.279714536
kynurenine.pcaac380	-0.056627113	0.05217973	0.279724864
lysopcac182.pcaec383	-0.069006645	0.063587757	0.279730582
smc260.pcaac422	-0.016847391	0.015524494	0.27973303
pcaac300.pcaac341	-0.012729274	0.011730931	0.27978159
pcaec420.pcaac363	-0.020070132	0.01849719	0.279810997
pcaec446.pcaac403	-0.004279784	0.003944819	0.279865857
pcaac420.pcaec406	0.018659806	0.017199885	0.279880346
asymmetricdimethylarginine.pca	0.03014507	0.027791066	0.279958067
lysopcac160.smc181	-0.010442296	0.009626866	0.279958087
pcaac344.c51	0.085537565	0.078859416	0.279966627
pcaac403.pcaec340	-0.061825919	0.057010006	0.280059052
creatinine.c51dc	-0.149244269	0.137622593	0.28007181
pcaec425.alanine	-0.015546002	0.014336293	0.280100586
pcaec301.pcaac323	0.076971603	0.07098431	0.280115655
pcaec402.pcaac402	-0.114418552	0.105536304	0.28019681
pcaac406.pcaac381	-0.019683965	0.018157629	0.280242161
pcaec402.glutamicacid	0.090517501	0.083498906	0.280244001
pcaec444.c102	-0.023824347	0.021977451	0.280252928
pcaac365.pcaec424	0.005787973	0.005339296	0.280254264
pcaac421.c181	-0.014113059	0.013020643	0.280313516
pcaec361.pcaec343	0.115619645	0.106678166	0.280349512
c8.pcaac365	0.036551003	0.033724474	0.28035169

serotonin.pcaec383	-0.093386726	0.086179044	0.280429586
pcaac240.pcaac363	-0.017611143	0.016251923	0.280430351
lysopcac280.taurine	0.1094262	0.100984784	0.280449545
c3.pcaec383	0.079402358	0.073282571	0.280485582
pcaec321.arginine	-0.059243696	0.054696033	0.280647016
pcaac383.pcaec405	0.075407475	0.069621039	0.280660718
pcaac321.kynurenine	-0.019163053	0.017694287	0.28070734
smc261.c141oh	-0.138349083	0.12775624	0.280748657
pcaec364.pcaec404	-0.019152202	0.017686492	0.280767576
phenylalanine.pcaac321	0.029662011	0.027396132	0.280839999
c16.smohc141	-0.047475018	0.043848537	0.280841449
glutamicacid.smc240	-0.058025678	0.053593549	0.280844015
asparagine.pcaac422	-0.006304843	0.005823387	0.280854064
smc160.smohc241	0.11628162	0.107404631	0.280865675
c101.pcaec404	-0.026311135	0.024302963	0.280874178
alanine.smohc221	0.059729662	0.055182764	0.280977576
pcaac422.pcaac341	0.006560659	0.006062195	0.281053732
pcaec421.pcaec380	0.139635964	0.129028861	0.281061614
pcaac405.pcaac406	-0.063823436	0.058978463	0.281087742
pcaac321.pcaec360	-0.007723769	0.007139053	0.281195927
pcaec301.glycine	0.037712868	0.034861792	0.281249764
pcaec405.tyrosine	-0.089837317	0.083052447	0.281288939
asparticacid.pcaec422	-0.057025114	0.052723351	0.281334373
pcaac240.smc161	-0.117282043	0.1084395	0.281355486
pcaec362.pcaac365	-0.057694942	0.053345612	0.281360969
lysopcac204.pcaac361	-0.049177493	0.04547139	0.281372984
histidine.valine	0.081338954	0.075209644	0.281376408
ornithine.lysopcac161	-0.111049602	0.102686972	0.281402211
smohc222.alanine	-0.02696807	0.024938409	0.281424861
smc260.lysopcac203	-0.032660164	0.030203331	0.28144428
pcaec401.pcaac341	0.007310358	0.006760679	0.281461057
pcaec365.pcaac385	-0.021986816	0.020335262	0.281500477
spermidine.creatinine	-0.055377614	0.051221789	0.281536982
methionine.c16	-0.031676141	0.02929901	0.28153718
pcaec382.pcaac383	0.021302581	0.019708956	0.281659318
pcaec402.c141oh	-0.101321637	0.093745845	0.28167966
methionine.c18	0.060502169	0.055979342	0.281687402
pcaac362.pcaec445	0.105124847	0.097267347	0.281692749
pcaac380.pcaac365	0.071162177	0.065861955	0.281829227
pcaac380.smc160	-0.069850308	0.06465507	0.281883144
pcaac341.smohc161	-0.019721798	0.018261593	0.282057367
pcaac320.c182	-0.03768267	0.034892799	0.28205956
pcaec382.pcaac365	-0.069617196	0.064464938	0.282073788

c141.pcaec363	-0.026687811	0.024716006	0.282138126
smohc161.pcaec421	0.001504509	0.001393372	0.282145858
pcaec446.c2	-0.040949075	0.037928303	0.282197683
pcaec364.pcaec360	-0.004848727	0.004491541	0.282250994
smc261.pcaec383	-0.08893121	0.082390699	0.28231318
glutamicacid.smc180	-0.018017955	0.01669424	0.282354184
ornithine.pcaec445	-0.046022654	0.042649964	0.28244894
pcaec425.c101	0.026750886	0.024793092	0.282499177
pcaec301.pcaec424	0.009475937	0.008782857	0.282522492
smc160.serine	-0.022880942	0.021207498	0.282524566
acetylornithoine.pcaec321	0.035979531	0.033352962	0.282594508
pcaac361.pcaac344	-0.086641345	0.0803172	0.282599435
lysopcac140.pcaec383	0.105707011	0.098006015	0.282671796
lysopcac160.c141oh	-0.136733037	0.12679494	0.282759558
acetylornithoine.pcaac383	-0.003168419	0.002938268	0.282781954
glycine.pcaac385	0.03627605	0.033643197	0.282813435
pcaec365.c51	0.028076426	0.026043125	0.282895108
pcaac281.pcaac364	-0.061768754	0.057296159	0.282901069
lysopcac180.smc261	0.061337499	0.056907267	0.282994878
h1.valine	0.082694025	0.076742729	0.283128925
pcaac364.pcaec424	0.00613608	0.005694884	0.283162876
pcaac426.kynurenine	-0.013564936	0.012591498	0.283235519
phenylalanine.pcaec423	-0.009288633	0.008622548	0.283262199
pcaac405.c8	-0.047994719	0.044556785	0.283302555
pcaec403.pcaac341	0.010033069	0.009314436	0.283305168
phenylalanine.pcaec321	0.045177584	0.041942549	0.283315154
pcaec423.serine	-0.028376842	0.026346755	0.283349667
lysopcac140.pcaec424	0.004764772	0.004423963	0.28335656
c141oh.lysine	0.069616323	0.064637399	0.283360393
pcaec381.c12	0.09026808	0.083822224	0.283417995
pcaac384.h1	0.0013345	0.001239593	0.283567888
pcaec420.pcaac323	0.128215463	0.119098631	0.283574083
pcaec385.pcaec445	0.074879641	0.069555767	0.283577419
c3.pcaac402	-0.073131366	0.06794686	0.283683754
pcaac322.pcaac365	-0.05115157	0.047529457	0.283725902
pcaec444.pcaec402	-0.047175059	0.043837987	0.283763737
pcaac402.creatinine	-0.019222478	0.017865836	0.283847449
pcaac281.pcaec423	-0.007436658	0.006912599	0.283902195
pcaac383.pcaec322	0.07278629	0.06765765	0.283906352
smohc241.pcaec423	-0.008478339	0.007881647	0.283949312
pcaac360.pcaac403	-0.014009642	0.013024339	0.283974078
c101.c102	-0.048203923	0.044815448	0.283992516
lysopcac181.c2	0.063335292	0.058885532	0.284011804

pcaec402.pcaac383	0.006770517	0.006295124	0.284033468
pcaac322.pcaec406	0.009147873	0.00850601	0.284059208
smohc161.lysopcac180	0.090436057	0.084094803	0.284083254
pcaac426.tyrosine	-0.080603262	0.074958779	0.284130009
pcaac401.transhydroxyproline	0.06197995	0.057640816	0.284139958
pcaac380.pcaec380	-0.072652458	0.067578053	0.284224394
pcaac240.pcaec364	-0.008992091	0.008364171	0.284231973
pcaec365.pcaec343	-0.130887144	0.12174735	0.284232302
pcaac361.smc161	-0.058065441	0.054017556	0.284292682
pcaac402.pcaec443	-0.202937132	0.188810203	0.284344289
pcaac406.c141	-0.028885696	0.026883947	0.284505794
glutamine.pcaac240	-0.022239361	0.020700181	0.284551789
pcaac321.pcaac360	0.050796097	0.047280708	0.284553759
pcaec380.c14	-0.080755336	0.075175715	0.284611973
pcaac363.pcaec446	-0.051058885	0.047533449	0.284635889
smc161.pcaec363	-0.052027045	0.048435441	0.284642625
c51dc.smohc161	-0.018419942	0.017149522	0.284675373
pcaec424.c181	-0.013520454	0.012589855	0.284747944
pcaac386.c8	-0.058707744	0.054674392	0.284813222
c12.pcaac406	0.05752609	0.053574553	0.284818884
smc240.lysopcac280	-0.008024243	0.007473062	0.284819777
pcaec343.histidine	-0.070115055	0.065302798	0.284848504
smc161.glutamine	0.027542705	0.025656139	0.284919405
c141.c2	0.070276313	0.06546681	0.284949785
methionine.smohc161	-0.035363292	0.032947908	0.285019292
pcaac343.c8	-0.04643011	0.043263341	0.285069207
pcaec342.pcaec423	-0.01046043	0.009747121	0.285076372
c4.c3	0.15886929	0.14803879	0.285086031
pcaac281.pcaac361	0.060120325	0.056022885	0.285095502
pcaec342.pcaec362	0.024489584	0.022821501	0.285116095
pcaec385.pcaac281	0.001801435	0.001679269	0.285269481
smc161.isoleucine	0.024326616	0.022677884	0.285290677
pcaac404.serine	-0.041654636	0.038843798	0.285442563
smc181.c101	-0.038344371	0.035759433	0.285476436
pcaac321.pcaec301	-0.106622325	0.099452808	0.285564685
pcaec343.c181	-0.009737119	0.009082442	0.285568301
methioninesulfoxide.smc161	0.107751049	0.100534615	0.285703008
lysopcac181.smohc221	-0.073489119	0.068587954	0.2858474
c51dc.pcaac341	-0.009234225	0.00861842	0.285850047
pcaac363.lysopcac182	-0.021002266	0.019606825	0.285975891
pcaac383.pcaec420	-0.049847053	0.046536429	0.285989646
lysopcac160.lysopcac180	-0.092200522	0.08607872	0.285999427
c102.smc180	0.029501479	0.027542701	0.285999775

smohc222.serine	-0.023868847	0.022289579	0.28611876
pcaec361.lysopcac181	0.041452496	0.03871875	0.286229479
pcaec423.smc160	-0.059712321	0.055776973	0.286251969
pcaec363.transhydroxyproline	0.024952219	0.023308305	0.286263609
pcaac342.transhydroxyproline	0.024264881	0.022671431	0.286373279
alanine.h1	-0.005197026	0.004857431	0.286540122
lysopcac170.lysopcac180	0.147798459	0.138146618	0.286560655
c182.pcaac385	0.021979148	0.020547245	0.286640667
pcaac343.c51	-0.142703447	0.133406644	0.286640928
c4.pcaec364	0.034963975	0.032686572	0.286647089
pcaac426.pcaac420	-0.102132383	0.095486668	0.286681022
smc241.pcaec401	0.068506104	0.064057555	0.286749331
pcaec420.pcaec360	-0.005610091	0.0052459	0.286759303
asparticacid.h1	-0.006871439	0.006425466	0.286766859
smc180.c51	0.042091384	0.039362672	0.286804879
pcaec384.pcaec404	-0.0180468	0.016878754	0.286858843
c4.h1	0.001765097	0.001650872	0.286864206
lysopcac182.smc241	-0.061253908	0.057291217	0.28687438
pcaec421.pcaec423	-0.191737281	0.179334003	0.286876402
smc180.pcaec385	-0.060659145	0.056738998	0.286908783
pcaec423.phenylalanine	0.056964918	0.053292945	0.28699373
asymmetricdimethylarginine.pca	0.040729373	0.038108286	0.287048371
pcaac425.isoleucine	0.036107916	0.03378576	0.287070007
pcaec385.histidine	-0.059943818	0.056103975	0.28720039
pcaac364.pcaec421	0.001498805	0.001403023	0.28727815
methioninesulfoxide.pcaec404	0.034493308	0.032301387	0.287462441
pcaec363.pcaac344	0.096437189	0.090315347	0.287496358
c141.c5	-0.011010512	0.010311689	0.287502176
pcaec362.putrescine	-0.053311282	0.049928913	0.287514007
smohc221.smc240	0.064328781	0.060264764	0.287652354
pcaac344.pcaec301	-0.097589782	0.091427422	0.287667809
pcaac406.asparticacid	0.049499302	0.046376574	0.287698165
pcaac405.pcaec424	0.005108658	0.004786463	0.287707262
pcaec423.asymmetricdimethylar	0.021313451	0.019970185	0.287729912
acetylornithoine.prolinr	-0.049210584	0.046110391	0.287743153
acetylornithoine.pcaac426	0.02433746	0.022804941	0.28775799
smc160.pcaec402	-0.051585398	0.048337885	0.287765896
pcaac343.phenylalanine	0.073045399	0.068450095	0.287788373
pcaec322.glutamicacid	0.077955858	0.073058465	0.28783325
pcaec401.pcaec382	0.019102151	0.017902216	0.287836266
pcaec361.c182	-0.042743586	0.040058768	0.287838603
smc241.pcaac425	0.118603213	0.111153822	0.287840058
pcaac385.putrescine	-0.046441715	0.043528428	0.287880732

c0.smc260	0.035515296	0.03329457	0.287983795
pcaac425.smc202	0.05176709	0.048532093	0.288002911
histidine.glutamine	0.048539107	0.04551434	0.288092679
glutamicacid.pcaac366	0.027027317	0.025343864	0.288107522
smohc161.serine	-0.03083325	0.028912943	0.288110958
pcaec381.pcaac341	0.013148376	0.012329641	0.288116831
pcaac424.pcaac383	0.007831355	0.007343811	0.28812374
pcaec340.pcaec423	-0.00721993	0.006771047	0.288166041
c102.smohc141	-0.031136313	0.029202614	0.288201097
methionine.leucine	-0.012024886	0.011278618	0.288223652
pcaec403.smc261	-0.095049626	0.089159708	0.288271512
pcaec343.h1	0.004251981	0.0039891	0.288343833
pcaac380.putrescine	-0.038885385	0.036485881	0.288404418
ornithine.pcaac300	-0.042101671	0.039512281	0.288508709
methionine.pcaac385	0.034228081	0.032125997	0.288554368
pcaec361.pcaec364	-0.007995685	0.007505755	0.288625819
acetylornithoine.c14	-0.01070089	0.010045417	0.28863616
pcaec425.smohc222	-0.007268884	0.006824739	0.288713838
asparticacid.smc181	-0.007760814	0.007287324	0.288760832
smc180.pcaac405	-0.069961845	0.065701494	0.288819654
glutamicacid.pcaac281	-0.002283267	0.002144269	0.288829259
putrescine.c162	-0.093062421	0.087413514	0.28891944
alphaaminoadipicacid.pcaac403	-0.011516597	0.01081789	0.288935073
pcaec403.lysopcac204	0.102557824	0.096343649	0.288974796
pcaec425.serine	-0.026656053	0.025041423	0.288984601
smohc222.smc160	0.243377896	0.228638504	0.288990214
lysopcac280.pcaac420	-0.105740361	0.099353409	0.289071765
pcaec363.c142	0.057697317	0.054224974	0.289184284
pcaec422.pcaac406	-0.049603271	0.046619428	0.289198534
pcaac323.pcaec322	0.093458072	0.087850705	0.289277946
c18.pcaec423	-0.006756904	0.006355097	0.289550055
pcaec361.arginine	-0.088114463	0.082876975	0.289563559
pcaac385.smohc141	-0.017318374	0.016289417	0.28957653
lysopcac140.pcaac420	-0.084124951	0.079143059	0.289675612
pcaac386.pcaec446	-0.072028622	0.067780006	0.289795601
smc181.c2	-0.069630078	0.065523694	0.289801131
pcaac406.c182	-0.034678759	0.032639028	0.289880959
pcaac426.smc161	-0.079623816	0.074947203	0.289923844
pcaac240.c12	0.104564894	0.098440226	0.290005981
alanine.c51	-0.055895618	0.052628412	0.290067705
c14.serine	0.021829563	0.020556722	0.290141053
asparticacid.pcaac381	0.030398531	0.028634476	0.290282461
pcaec424.pcaac381	0.024841071	0.023399921	0.290290651

lysopcac281.smc180	-0.007986956	0.007524116	0.290324001
serine.h1	0.002964379	0.002792774	0.290354729
pcaec361.taurine	0.089866409	0.084681721	0.290454624
pcaac401.pcaec362	0.010492792	0.009888382	0.29050096
pcaec424.c142	0.052469421	0.049447414	0.290504431
pcaac343.pcaac240	-0.023049156	0.021724927	0.290577435
c141.c162	-0.121732745	0.114742211	0.290591254
c16.pcaac403	0.008044289	0.007582452	0.290598095
histidine.pcaac422	-0.0224201	0.021138743	0.290730449
pcaac321.pcaec403	-0.0758243	0.071495946	0.290765174
c18.pcaac383	0.005151349	0.004857392	0.290775381
spermidine.c102	0.148300157	0.139878027	0.290914394
smc160.pcaac421	-0.035259332	0.033261901	0.290986408
pcaec383.pcaec423	0.034420393	0.032471108	0.2909956
c141.lysopcac160	0.038721789	0.036531456	0.291029097
glutamicacid.c162	-0.111461631	0.105158485	0.29103724
h1.smohc222	-0.018899297	0.017830971	0.291048775
smc161.kynurenine	-0.0054574	0.005149179	0.291074082
smohc141.serine	-0.017439453	0.016454716	0.291079944
pcaac323.pcaac403	-0.006032885	0.005692506	0.291103041
asparagine.pcaac323	0.10646161	0.100471183	0.291180589
pcaac404.pcaac363	-0.012732163	0.012017482	0.291250042
pcaec424.creatinine	-0.011487417	0.010842758	0.291256817
pcaec443.pcaac366	0.084323372	0.079633694	0.291512985
prolinr.pcaac361	0.042425527	0.040079636	0.291676341
c141.lysopcac280	0.01352099	0.012774371	0.29171452
lysopcac281.pcaec322	-0.093318778	0.088185638	0.291822737
pcaec406.pcaac341	0.007696779	0.007273998	0.291861878
c18.pcaec420	-0.051220463	0.048407195	0.291864343
isoleucine.c51	0.037015588	0.034984658	0.29189375
pcaac402.pcaec340	-0.058137144	0.054949666	0.291914083
smohc241.pcaac380	0.041723769	0.039442322	0.291988867
pcaec401.histidine	-0.099043378	0.093632779	0.292014904
pcaec363.pcaac240	-0.015867176	0.015001987	0.292066567
pcaac364.pcaec301	0.104158178	0.098492004	0.292131245
arginine.asparticacid	-0.032086357	0.030344167	0.292183483
prolinr.pcaec383	-0.043803304	0.041425246	0.292187276
pcaec300.lysopcac160	-0.02227805	0.021069705	0.292212783
smc161.pcaac240	-0.011763149	0.011125788	0.292241454
pcaac322.smc240	-0.055594612	0.052583789	0.292254732
pcaec381.lysopcac203	0.013475341	0.012746742	0.29229927
smc260.pcaec360	0.010899547	0.010310824	0.29232752
pcaec422.pcaec364	-0.009702354	0.0091798	0.292406262

pcaac425.smohc141	-0.0203448	0.019250708	0.292447409
smc180.pcaac402	-0.081878501	0.077475691	0.292449959
smc241.pcaec404	0.018798945	0.017792185	0.292560886
leucine.smc181	-0.016335919	0.015462659	0.292610502
pcaec384.pcaac380	0.081768738	0.077408197	0.292675837
phenylalanine.glutamine	0.024761694	0.023441357	0.29267888
smohc222.pcaec340	-0.082820668	0.078405797	0.292686676
pcaac384.pcaec404	-0.019856986	0.018798502	0.292687203
pcaec401.methionine	-0.054158174	0.051272638	0.292700185
serotonin.c51dc	-0.127979503	0.121218502	0.292929133
pcaec340.pcaac380	0.062790339	0.059482747	0.29300628
smc240.asparagine	-0.009238521	0.00875284	0.293059811
smohc141.smohc221	0.041968372	0.039764712	0.293092098
pcaec443.pcaac341	0.007195104	0.006820102	0.293289198
kynurenine.glutamicacid	0.073840082	0.0700004	0.293349615
pcaec402.pcaac381	0.03668424	0.034778489	0.293374889
phenylalanine.pcaec422	-0.047281447	0.044826422	0.29338832
pcaac386.smohc141	-0.022703321	0.021530162	0.293515192
pcaac323.pcaac361	-0.047564597	0.045121212	0.293669103
pcaec362.lysopcac204	-0.033400733	0.031687589	0.293709247
kynurenine.smc161	0.06908589	0.065550479	0.293768269
pcaec423.pcaec424	0.02771997	0.026304339	0.293821558
pcaac421.transhydroxyproline	0.045503071	0.043180221	0.293832077
phenylalanine.c102	0.029348036	0.027852341	0.29387471
serine.kynurenine	-0.007135723	0.006772209	0.29388543
pcaec446.putrescine	-0.0553565	0.052539437	0.293912501
pcaec445.pcaac381	0.021451746	0.020360886	0.293931585
smc260.c51dc	0.124282106	0.117970748	0.293966684
pcaec404.lysopcac180	-0.076589377	0.072705632	0.294004091
putrescine.pcaac402	0.022576611	0.021433212	0.294036191
glutamicacid.smohc161	-0.020510829	0.019473164	0.294063649
glycine.pcaec445	-0.061348281	0.058249817	0.294106677
pcaec365.pcaec404	-0.012742566	0.012099722	0.294135855
pcaec423.pcaac402	0.101514837	0.096399331	0.294164618
c51.pcaec424	-0.009642014	0.009160667	0.294402496
pcaec342.smc160	0.149700443	0.142233203	0.294423053
smc241.c101	-0.014349317	0.013634098	0.294442213
citruline.pcaec362	0.020141811	0.019139844	0.294491671
lysopcac280.pcaec364	-0.008824098	0.008385412	0.29450744
smohc222.glutamicacid	0.040868028	0.038836599	0.294511204
pcaec444.serine	-0.009894929	0.009403638	0.294539671
asparagine.lysopcac280	0.010933395	0.010391578	0.294587565
c182.c12	0.062038536	0.058969997	0.294635281

pcaac426.pcaec424	0.006916978	0.006575516	0.294683882
pcaac383.c51	0.043517826	0.041385903	0.29487412
serine.smc181	-0.015128878	0.014389173	0.294922708
c8.pcaec406	-0.00566743	0.005391933	0.295065809
acetylornithoine.pcaac300	-0.0984201	0.093637361	0.29507358
pcaec384.pcaac403	-0.004055259	0.003858631	0.295128241
pcaec444.c51	0.069165118	0.065812305	0.295134072
c181.pcaec322	0.073490042	0.069961821	0.295369548
pcaec425.pcaec380	0.091078296	0.086725396	0.295478954
pcaec423.creatinine	-0.015303915	0.014574417	0.295542361
smohc161.smc260	0.050556855	0.048148004	0.295552988
glycine.pcaec420	0.049119929	0.04678103	0.295568287
smohc222.pcaac240	-0.010116305	0.009637851	0.295730255
c0.pcaac384	-0.08416783	0.080195543	0.295781002
pcaac321.lysine	-0.084267267	0.08029386	0.295802403
pcaac363.pcaec360	-0.00481245	0.004586081	0.295860107
smc202.pcaec380	0.065171515	0.062115533	0.295934182
pcaec322.leucine	-0.013285723	0.01266284	0.295938074
c5.pcaec340	-0.104065373	0.099187827	0.295944962
smohc221.pcaac341	0.010853129	0.010347769	0.296099624
pcaec361.pcaac380	0.074594551	0.071124182	0.296120029
pcaac240.serine	-0.030789071	0.029358655	0.296152583
methionine.pcaec406	0.008520193	0.008125035	0.296192745
smohc222.lysopcac181	0.051284301	0.048907102	0.296205654
c141oh.arginine	0.040232067	0.038371675	0.296262075
c12.c14	0.066062358	0.06302395	0.29638739
pcaac406.pcaec321	-0.032392211	0.030903368	0.296402542
pcaac425.c2	0.097425475	0.092955622	0.296444532
pcaec444.pcaec406	0.002797181	0.002668873	0.29644908
pcaac422.smohc241	0.09811678	0.093616276	0.296450007
pcaac421.c16	0.028235244	0.026944824	0.296533893
methioninesulfoxide.pcaac403	0.019912281	0.019006315	0.296637043
pcaec322.alanine	-0.013755933	0.013132317	0.296719431
pcaec381.pcaec340	-0.056113229	0.053570409	0.296728758
lysopcac281.pcaac420	-0.127995634	0.122237955	0.296896283
smohc141.pcaac323	-0.128669382	0.12288976	0.296929036
c16.pcaec301	-0.057902811	0.055306125	0.296965709
c8.c16	0.059585266	0.056913481	0.29696868
pcaec403.pcaec443	0.233032558	0.222599485	0.29700334
pcaec384.serine	-0.04479707	0.042792078	0.297010229
pcaac420.pcaac341	0.005332021	0.005093438	0.297016275
c12.c10	0.050924494	0.048649643	0.297053621
pcaec444.ornithine	-0.039738117	0.037963097	0.297055194

c51dc.pcaec401	-0.093583613	0.08940585	0.297068235
pcaac281.prolinr	0.03198929	0.030563259	0.297100285
pcaac360.pcaac383	0.007469326	0.007136948	0.297140246
pcaac424.pcaec365	0.031907049	0.030499903	0.297340442
smohc161.pcaac406	-0.050329823	0.048114303	0.297381404
pcaac384.pcaac403	-0.004179645	0.003996045	0.297428137
pcaac342.pcaac365	-0.055798714	0.05334768	0.297428551
pcaac421.pcaec424	0.060137456	0.057515591	0.297593855
smc241.pcaec406	0.00889175	0.008504214	0.297600935
c4.pcaac240	-0.025918986	0.024795119	0.297713155
smc161.alanine	-0.024161604	0.023115193	0.297739264
c102.h1	-0.00687063	0.006573394	0.297762929
smohc222.c102	0.028955342	0.027716065	0.297995347
c10.pcaec402	-0.093695027	0.089692989	0.298038655
citruline.pcaac364	0.045282078	0.043364961	0.298227753
pcaec386.smohc221	0.026796805	0.025666105	0.298299053
pcaac281.pcaec424	0.007455039	0.007140486	0.298300063
asparagine.pcaac344	0.037590343	0.036005757	0.298319789
serine.pcaac321	0.027335618	0.026183856	0.298329813
smc160.pcaec423	-0.020587356	0.01972074	0.2983497
smc240.smohc222	0.00888988	0.008515695	0.298351433
prolinr.pcaec300	-0.071526238	0.068522787	0.298401769
pcaec342.smohc141	0.008057834	0.007720491	0.298464877
glutamine.c14	0.060830584	0.058284761	0.298472024
pcaec362.pcaac383	0.013090753	0.012544922	0.298549942
pcaac366.lysopcac170	0.010688239	0.010242833	0.298561663
smc202.pcaec424	-0.055522649	0.053212288	0.298592503
c181.c2	-0.061096322	0.058554341	0.298595035
pcaac365.pcaac322	-0.08379137	0.080312078	0.298636634
asymmetricdimethylarginine.pca	-0.002992578	0.002868523	0.298671241
lysopcac281.pcaac384	0.040424778	0.038751687	0.298704671
pcaac240.pcaec424	0.006601954	0.006329078	0.298732388
pcaec424.serine	-0.019638123	0.018827493	0.298759603
smc202.lysopcac181	0.075308674	0.072202466	0.298775669
c18.pcaac422	0.044498451	0.04266699	0.298820095
c141.pcaac403	0.005159136	0.004947414	0.298880072
pcaec444.smc240	-0.032496038	0.031162918	0.298887256
lysopcac180.lysopcac161	-0.071276148	0.0683598	0.298941394
pcaac426.asparticacid	0.037836353	0.036291706	0.298987455
alphaaminoadipicacid.pcaec364	-0.027520845	0.026398966	0.299017404
c101.pcaec406	-0.003565179	0.003419907	0.299026012
pcaec444.pcaec382	-0.012960491	0.012434802	0.29911966
c142.pcaec380	-0.063589642	0.061012076	0.299132983

asparticacid.pcaec362	-0.018814592	0.018055686	0.299232466
pcaec343.pcaac420	0.08865499	0.08508862	0.299286895
pcaec422.pcaac363	-0.032493385	0.031190044	0.299345341
pcaec380.pcaac240	-0.022233476	0.021342562	0.299365472
ornithine.tyrosine	-0.051692967	0.049627693	0.299424701
pcaac320.c51	0.055961627	0.053737928	0.299533273
pcaac384.c141	-0.031998033	0.030728873	0.299569599
c181.pcaec423	-0.008736761	0.008390335	0.299575688
smohc221.c51dc	-0.132966555	0.127748606	0.299780641
glutamine.smc180	0.009521108	0.009148238	0.299820767
pcaec421.pcaec342	0.120015706	0.115320815	0.299842569
lysopcac281.kynurenine	-0.008607143	0.008270725	0.299859124
c181.lysopcac203	-0.019177942	0.018428368	0.299859482
pcaec423.alanine	-0.015414616	0.014812486	0.299870981
smc202.lysopcac280	0.009510457	0.009139752	0.299912848
pcaac240.smc180	-0.005063926	0.00486759	0.300016534
pcaec361.smc180	-0.004499907	0.004325622	0.300037013
pcaec401.pcaec364	-0.01161068	0.011162773	0.300113941
pcaac344.pcaec405	-0.047306716	0.045482109	0.300117666
smc160.pcaec425	0.026242895	0.025231581	0.300134199
smc180.pcaec406	0.007134175	0.006859288	0.300137005
pcaec300.pcaec301	-0.05857011	0.05631689	0.300167359
pcaac383.pcaac360	0.03146985	0.03026493	0.300258668
pcaec405.ornithine	-0.059328026	0.057057273	0.300265449
pcaec445.c3	-0.020913589	0.020114513	0.300298528
pcaac322.c10	-0.009233226	0.008881318	0.300346249
pcaec401.c5	0.025148505	0.024195239	0.300450179
lysine.pcaec402	0.037277786	0.035866785	0.300477434
h1.c141	-0.020065009	0.019308346	0.300547692
pcaec443.pcaec364	-0.009126144	0.008782145	0.300556112
pcaec360.pcaac402	-0.069999313	0.067360975	0.300557532
pcaac365.smc161	-0.061730079	0.059420106	0.300692785
alanine.pcaec383	-0.046092476	0.044367988	0.300696235
pcaec445.pcaac403	-0.006017562	0.005792462	0.300699463
pcaac363.pcaac425	-0.120545093	0.116040736	0.300719779
lysopcac281.pcaac344	-0.063944598	0.061559927	0.300756696
kynurenine.pcaac385	0.032347193	0.031145362	0.300826045
pcaec321.putrescine	-0.04145338	0.039920216	0.300910453
pcaac320.spermidine	-0.071920007	0.06926645	0.300955112
pcaec386.pcaac365	0.065499994	0.063086779	0.300981595
pcaac342.smc160	0.086461596	0.083278746	0.300996926
pcaec300.pcaec406	0.003745133	0.003608998	0.301228122
pcaec360.histidine	-0.05995682	0.057780745	0.301255944

asparticacid.smc180	-0.005261299	0.005071797	0.301393752
pcaec362.alanine	-0.015366377	0.014815897	0.301490911
citruline.pcaac300	-0.069275075	0.066796991	0.301516878
arginine.pcaec364	-0.009999296	0.009642116	0.301542376
serine.serotonin	-0.084418756	0.081404634	0.301550461
smc161.c51	0.053794644	0.051876487	0.301574117
pcaac381.pcaac383	0.0064473	0.006217627	0.301591038
pcaac406.pcaac365	0.068978439	0.066527409	0.301635905
pcaec443.pcaac385	-0.019978759	0.019269453	0.301651022
pcaac406.pcaec401	-0.060440233	0.05829776	0.301678593
smohc221.pcaec403	-0.028474235	0.027467564	0.301725536
asparticacid.smc202	0.072380433	0.069833235	0.301806377
pcaac401.pcaec405	0.139933205	0.135017401	0.301837398
pcaac366.c18	0.058069649	0.056034028	0.301874737
c3.pcaec382	0.016637574	0.016055323	0.301904022
smohc222.pcaec406	0.006721787	0.006486625	0.301909537
pcaac422.pcaec364	-0.00948829	0.009157168	0.301952991
pcaec340.pcaac383	0.005372696	0.005185205	0.301953433
ornithine.c141	-0.026898041	0.025960478	0.301973866
phenylalanine.pcaac425	0.080296384	0.077508329	0.302040785
pcaec343.transhydroxyproline	0.038643723	0.037312752	0.302180437
pcaec341.pcaec401	0.069515613	0.067132186	0.302258175
pcaac380.pcaec363	-0.04345808	0.041970631	0.302287585
lysopcac204.smc181	-0.023529497	0.022725731	0.302321143
pcaec321.pcaec406	0.004332902	0.004185686	0.302412713
pcaec425.pcaec301	0.069810897	0.067472735	0.302653691
pcaac323.lysopcac204	-0.040465974	0.039123288	0.302809219
pcaac365.histidine	-0.082041114	0.079339815	0.302935963
pcaec381.pcaac385	-0.028200846	0.027273162	0.302951186
creatinine.pcaac366	-0.051913212	0.050205694	0.30295309
pcaec446.pcaac426	-0.089300525	0.086371207	0.302997339
lysopcac160.c12	0.088021607	0.08513572	0.303005706
pcaec342.pcaac403	-0.005061276	0.004895442	0.303016131
pcaec445.pcaec403	0.045598259	0.04410581	0.303033456
smc160.c182	0.03114447	0.030128806	0.303092715
pcaec420.smc202	-0.044903045	0.043439846	0.303105483
citruline.lysopcac182	0.025990773	0.02514452	0.303118404
c51dc.c5	0.019098165	0.018480341	0.303222865
smc161.smc241	-0.191873608	0.185695402	0.303297812
arginine.smc202	-0.0993353	0.096149429	0.303361244
pcaac380.pcaec446	-0.134811456	0.130500583	0.303408436
c101.pcaac426	-0.05225267	0.050582015	0.303410631
smc181.c51	0.041691853	0.040361805	0.303445844

lysopcac182.c141	0.026210439	0.025375404	0.303467237
pcaec445.kynurenine	0.020266247	0.01962146	0.303488648
pcaec401.pcaec406	-0.057314915	0.055496506	0.303533021
pcaac366.smohc141	-0.026839845	0.025990666	0.303576733
smohc221.c18	0.039532297	0.038284112	0.303609041
smc241.pcaac360	-0.050082987	0.048502326	0.303615492
asparagine.serine	-0.005478022	0.005305485	0.303647592
pcaec365.pcaec363	-0.039701564	0.038453163	0.303673287
lysopcac203.smc180	-0.008586282	0.008317081	0.303719096
pcaac420.c101	0.033080715	0.03204562	0.303750188
lysopcac182.phenylalanine	0.073629351	0.071347006	0.303895475
smohc222.pcaac383	0.005569783	0.005397776	0.303952946
pcaac421.pcaec406	-0.024778014	0.024014753	0.303991798
pcaac366.pcaec421	-0.005428746	0.005261851	0.304022174
lysopcac280.lysopcac180	0.060232249	0.058382735	0.304040309
asparticacid.histidine	0.0725617	0.070352825	0.304172005
prolinr.c18	-0.071879457	0.069693823	0.304189091
smc160.glutamicacid	0.039101183	0.037914017	0.304211723
pcaac422.pcaac402	0.101820724	0.098765021	0.304385957
c10.c162	-0.114762682	0.111326043	0.304418237
pcaec425.c2	-0.058385684	0.056643007	0.30446687
pcaec446.histidine	-0.05148448	0.049948182	0.30447067
pcaec404.methionine	-0.056262379	0.054606588	0.304674329
pcaac341.prolinr	-0.065568946	0.063648413	0.304743568
pcaec380.phenylalanine	0.057498317	0.055814532	0.304746653
pcaac402.pcaac385	-0.029425008	0.02856446	0.304765802
pcaac364.putrescine	-0.045667743	0.044339539	0.304845898
arginine.lysopcac182	0.035364264	0.034338181	0.304880333
lysopcac180.serotonin	-0.092405145	0.089735656	0.304942701
leucine.putrescine	-0.035036596	0.034025162	0.304953142
pcaac360.h1	0.00252215	0.002449716	0.30502691
histidine.asparticacid	0.043408192	0.042165956	0.305077332
acetylornithoine.c101	-0.014941512	0.014514139	0.305084491
pcaec401.alanine	-0.015363696	0.014924811	0.305102713
pcaac365.pcaec444	0.133050632	0.129255215	0.305122679
pcaec365.pcaac341	0.00760513	0.007388271	0.305128285
histidine.pcaec404	0.030101447	0.029244682	0.305154178
lysopcac170.pcaec364	-0.020495516	0.019914036	0.305199544
pcaac323.c101	-0.021776265	0.021158602	0.305203032
pcaac320.glutamicacid	0.048964496	0.047577129	0.305217869
pcaac422.pcaec424	0.015868996	0.015421037	0.305270222
pcaac381.pcaec443	0.125993118	0.122448281	0.305316519
pcaec385.pcaac403	-0.003176833	0.003087544	0.305330906

alanine.pcaac360	-0.043340136	0.042134151	0.305469728
pcaec340.c182	-0.030551377	0.029701588	0.305475173
c141.acetylornithoine	-0.049810046	0.048425009	0.30547949
asparticacid.pcaac321	0.023510699	0.022862062	0.305587202
pcaac401.c5	0.02986021	0.029042125	0.305682245
pcaec424.pcaac402	-0.0728528	0.070857928	0.305689638
pcaac343.pcaac364	-0.0892073	0.086767965	0.305708294
pcaec425.smc240	-0.034962635	0.034007726	0.305724288
c16.asymmetricdimethylarginine	0.037040126	0.036034638	0.305806698
lysopcac181.serine	-0.042074306	0.040932181	0.305806922
pcaac300.pcaec406	0.01469128	0.014295726	0.305916396
c14.c3	0.033498772	0.032602041	0.305993287
pcaec422.pcaac421	-0.070634495	0.068770468	0.306181047
pcaac343.pcaec340	0.057218835	0.055709724	0.306188652
pcaac362.pcaec406	0.004205297	0.004094609	0.30621499
c2.h1	0.010390315	0.010116904	0.306218535
c2.pcaac321	-0.028220044	0.027480308	0.306268444
pcaec445.smc160	-0.110324823	0.107437161	0.306287718
c51.glutamine	-0.018494183	0.018010355	0.306294187
asparticacid.pcaac361	0.069022248	0.067219102	0.306312483
pcaac300.smohc221	-0.041628758	0.040541648	0.306317297
c18.c102	-0.031498386	0.030677281	0.306340166
pcaec423.pcaec406	0.007861958	0.007657162	0.306349715
pcaac406.smc180	0.01927835	0.018779304	0.306430118
alanine.serine	-0.013467597	0.013119865	0.30646297
pcaec404.pcaac363	-0.021258366	0.020712548	0.306534412
pcaec423.pcaac421	-0.076317616	0.074359862	0.306545669
pcaec343.pcaac403	-0.004479654	0.004365045	0.306579567
pcaac420.asymmetricdimethylar	0.024303966	0.023682987	0.306596205
lysopcac182.valine	-0.0983478	0.095847047	0.306656958
pcaec404.pcaac341	0.00663246	0.006463829	0.3066582
pcaec361.pcaac383	0.005792806	0.005646064	0.306704377
citruline.pcaac366	-0.087348296	0.085137859	0.306717098
lysopcac281.pcaec364	-0.013113845	0.012782934	0.306752823
lysopcac160.pcaec380	-0.058663833	0.057188118	0.306791522
pcaec321.smc261	0.06814432	0.066455642	0.306976629
c8.pcaac380	-0.059307092	0.05785171	0.307095765
pcaec405.pcaec423	0.054638475	0.053301667	0.307131996
kynurenine.pcaec385	0.050205896	0.048981512	0.307171098
lysopcac281.c2	-0.092469207	0.090221923	0.307212689
pcaac344.pcaac364	-0.103259555	0.100754593	0.307234504
alanine.pcaac361	0.031652856	0.030886247	0.307254066
smc181.asymmetricdimethylargi	-0.0172492	0.016838616	0.307459584

smc241.pcaac281	0.001550563	0.00151384	0.307518573
c18.pcaec322	0.074145889	0.072394994	0.307552797
pcaac380.pcaec383	0.096635835	0.094364277	0.307606002
pcaec385.pcaec343	-0.113143524	0.110484721	0.307609459
pcaac281.pcaac383	0.007292889	0.007121533	0.307610996
pcaac363.pcaec301	0.089642169	0.087562097	0.307755125
ornithine.creatinine	-0.011636129	0.011367195	0.307800597
pcaec343.smc260	0.039517266	0.03860411	0.307802683
smc202.pcaac341	0.006841218	0.006683353	0.30781853
c182.c2	-0.065133836	0.063633165	0.307836209
glutamine.serine	-0.012576458	0.012287458	0.307865995
smc202.glutamicacid	0.061005903	0.059611171	0.307923784
putrescine.pcaec406	0.005281896	0.005161368	0.307945023
pcaec383.smc261	-0.094878546	0.092717493	0.307965803
lysopcac280.creatinine	-0.008494	0.008301299	0.30801031
smc161.pcaec425	0.014397131	0.014074103	0.308133475
c181.pcaec365	0.027512753	0.026899554	0.308207023
c12.taurine	-0.026369346	0.025782843	0.30822967
arginine.smohc141	0.028128634	0.027503092	0.308231274
pcaec382.smohc241	0.091092862	0.08908245	0.308314407
pcaec445.pcaec406	0.002744855	0.002684363	0.308329963
pcaac402.pcaac363	-0.023110216	0.02260923	0.308507448
c51.smc260	0.022429866	0.021948549	0.308615497
serine.pcaac300	-0.081313282	0.079570589	0.308628767
c4.smc240	0.054031815	0.05287384	0.308629004
pcaac425.pcaac385	-0.031903838	0.031221759	0.308654678
pcaac364.arginine	0.045200296	0.044238953	0.308709202
arginine.smc180	-0.005725683	0.005604953	0.30879928
pcaec403.pcaec340	-0.061036646	0.059750669	0.308807466
pcaec322.lysopcac203	0.021960864	0.021499373	0.308834386
pcaac404.spermidine	-0.072105161	0.070593897	0.308861508
pcaac341.pcaec406	0.009614569	0.009414125	0.30891628
pcaec380.histidine	-0.092054292	0.09014431	0.308965239
lysopcac182.c51dc	0.14338077	0.140408598	0.30897469
lysopcac182.c101	-0.009707404	0.009506653	0.308998832
pcaec382.smc180	-0.002989546	0.002928512	0.309128958
c12.c141	0.104927072	0.10279246	0.309164364
pcaec445.smc241	-0.098869617	0.096865132	0.309198678
c101.pcaec386	0.009781844	0.009583609	0.309202808
pcaec423.smohc222	0.006767983	0.006630996	0.309215214
pcaac380.pcaac281	0.001762117	0.001726679	0.309278988
lysopcac203.c142	-0.059836749	0.05863375	0.309281975
lysopcac203.creatinine	-0.008637748	0.008464384	0.309298806

c51dc.creatinine	-0.012421243	0.012175987	0.309458942
pcaac403.pcaec444	-0.153204603	0.150202296	0.30953177
pcaac361.pcaec382	0.007013394	0.006877065	0.309609649
putrescine.lysopcac204	-0.02692294	0.026399873	0.309614558
pcaac405.pcaec361	-0.010759692	0.010550777	0.309620414
lysopcac182.pcaac383	0.015600998	0.015298381	0.309629813
pcaec343.pcaec360	-0.005744162	0.005633249	0.309673274
pcaec364.c51	0.027475155	0.026949254	0.309755785
c5.asymmetricdimethylarginine	0.032293663	0.031678249	0.309797123
lysopcac182.pcaec342	0.080144996	0.078618291	0.309800828
smohc241.pcaec443	-0.056699135	0.055622426	0.309830027
pcaec381.pcaec362	0.01305823	0.01281381	0.309963731
lysopcac161.c101	-0.010504286	0.010307983	0.309978378
pcaac322.pcaac426	0.0979773	0.096157154	0.31003273
leucine.c51	0.02994343	0.029389033	0.310063393
creatinine.glycine	0.041949377	0.041177253	0.310116766
pcaac405.serine	-0.023903589	0.023466619	0.310178417
pcaac343.pcaac363	0.012277319	0.012052956	0.310181372
pcaec445.c2	-0.036945609	0.036272435	0.310207801
pcaec423.lysopcac180	-0.077912965	0.076496991	0.310230826
c16.pcaec403	0.033686815	0.033078208	0.310283448
smohc141.pcaec445	-0.03162885	0.031060858	0.310336741
c8.pcaec363	-0.018690613	0.018355124	0.310340876
pcaac300.creatinine	-0.008995036	0.008833975	0.310362487
pcaec444.pcaac363	-0.015901806	0.015618679	0.310411971
pcaec362.pcaac405	-0.056366386	0.05538417	0.310598022
pcaac366.pcaac383	0.019185518	0.018856224	0.310726491
lysopcac182.creatinine	-0.006996844	0.006876905	0.310737153
pcaec406.lysopcac170	0.028942047	0.028447156	0.31075802
pcaec403.creatinine	-0.015151584	0.014893017	0.310774716
pcaac386.pcaac366	-0.048075382	0.047255836	0.310783661
pcaec385.h1	0.002683034	0.002637398	0.310802253
glutamine.lysopcac182	-0.03951677	0.038853479	0.310912147
lysopcac280.smc180	-0.005172172	0.0050862	0.310992085
smc261.smc180	0.01081352	0.01063586	0.311086437
pcaac386.h1	0.003245203	0.003192045	0.311110409
pcaec444.pcaec445	-0.113660768	0.111823269	0.311215306
smc181.pcaec406	0.021547076	0.021202423	0.311299163
pcaec301.smohc141	0.006273124	0.006173099	0.311323828
pcaac366.pcaec340	-0.055031616	0.054155818	0.3113388
c101.pcaac422	0.013507224	0.013292762	0.311356874
pcaac381.pcaec362	0.024337348	0.023952324	0.31138491
lysopcac204.c12	0.078967535	0.07772464	0.311424579

c4.pcaac403	0.01382162	0.013606705	0.3115177
pcaec322.smc260	0.039107049	0.038501807	0.311553257
pcaec406.pcaec443	-0.064828259	0.063834784	0.311627594
pcaec322.pcaec406	0.007855753	0.007737155	0.311739073
c16.asparticacid	0.030846667	0.030381515	0.311747627
pcaec385.smc180	0.022220682	0.021887471	0.311788725
lysopcac160.pcaec401	-0.0954986	0.0940679	0.31179566
c51.pcaec380	-0.041396488	0.040778991	0.31182733
pcaac404.pcaec362	-0.018136149	0.017865729	0.311830315
pcaac363.pcaac405	0.088985675	0.087659457	0.311833656
c142.alphaaminoadipicacid	0.012804716	0.012616899	0.311949092
lysopcac280.pcaec406	0.008967243	0.00883828	0.312089123
c14.putrescine	-0.049131119	0.048428881	0.312132369
c162.pcaac422	0.074483059	0.073418966	0.312135678
lysopcac204.pcaac383	0.006704227	0.006608484	0.312138343
pcaac385.c141	-0.033800208	0.033318372	0.312150828
h1.asparticacid	-0.013919411	0.013722443	0.312202081
lysopcac160.lysopcac181	-0.077224838	0.07613293	0.312207582
pcaec404.pcaec443	-0.160629651	0.158368748	0.31223892
smc202.pcaec382	0.0086237	0.00850281	0.312266784
alphaaminoadipicacid.smohc141	-0.011748557	0.011584093	0.312276363
pcaec422.histidine	-0.098087192	0.096716997	0.312290795
pcaec341.alanine	-0.016424774	0.016196108	0.312313834
pcaec341.lysopcac180	-0.056183905	0.055408328	0.312371406
pcaac405.smohc221	0.026155	0.025795288	0.312396416
lysopcac182.serine	-0.013955092	0.013764969	0.312459575
pcaec360.glycine	0.045107349	0.044505413	0.31259609
pcaac362.leucine	-0.025417723	0.025079414	0.312612971
smc202.pcaac420	-0.072602811	0.07164355	0.312660621
c141oh.asparticacid	0.043547089	0.042972624	0.312670707
acetylornithoine.pcaac364	0.062202314	0.061383223	0.312682256
pcaec340.pcaac364	-0.055569755	0.054845329	0.312746654
serotonin.valine	0.066915069	0.06605473	0.312834155
smc261.pcaac425	0.103236309	0.10191204	0.312848628
isoleucine.kynurenine	-0.008216129	0.008111138	0.312872517
lysopcac182.pcaac403	-0.003061386	0.003022341	0.312884562
pcaec386.glutamicacid	0.046965314	0.046367576	0.312897621
lysine.pcaac300	-0.040925235	0.040405142	0.31290682
pcaec321.prolinr	0.022535113	0.022248886	0.312910252
pcaec402.c101	0.040276903	0.039766678	0.312926567
pcaec341.pcaec424	0.007050487	0.006961486	0.31294831
pcaac341.methionine	-0.040712005	0.040198096	0.312948486
pcaec364.pcaac403	-0.004204863	0.004151993	0.312972643

lysopcac161.smc180	-0.019064965	0.018825353	0.312975272
lysopcac203.lysopcac181	-0.077943232	0.077005534	0.313237714
pcaec381.smc180	-0.003823376	0.00377742	0.313243016
pcaec340.pcaac420	-0.110982245	0.109650216	0.313251545
pcaac402.c2	0.141803715	0.14011309	0.313290529
asparticacid.c182	-0.049933236	0.049349707	0.313405721
c10.lysopcac160	0.020172867	0.019938555	0.313440333
c12.tyrosine	0.044912124	0.044393609	0.313474532
pcaec385.pcaec444	0.098107853	0.096980886	0.31350286
glycine.pcaac281	0.003219835	0.003182869	0.313505921
glycine.methionine	0.02908069	0.028752067	0.313593871
smc160.pcaec363	-0.0595075	0.058836881	0.313608942
pcaac281.c18	0.029287545	0.028960485	0.313658833
pcaac361.prolinr	0.061834271	0.06114385	0.313659563
c142.lysopcac280	-0.012407373	0.012269156	0.313672133
c182.putrescine	-0.030291309	0.029954703	0.313685586
pcaec321.pcaac425	0.115403728	0.114121921	0.313688095
smohc222.pcaec342	-0.140954416	0.139389291	0.313689749
pcaec383.smc180	-0.003654623	0.003614549	0.313757254
serine.pcaec382	-0.013219578	0.013076021	0.313808848
pcaec301.pcaec406	0.01603419	0.015865239	0.313966013
smohc221.c2	0.060713455	0.060079205	0.314010028
lysopcac140.pcaac383	-0.013205884	0.013069494	0.31406782
pcaac300.pcaec425	0.018106353	0.017921637	0.314129344
pcaec382.pcaec361	0.018867522	0.018675305	0.314136138
pcaec342.pcaac405	-0.05168163	0.051157323	0.31415698
pcaec301.pcaac383	-0.006600599	0.006534415	0.314214448
smc180.alanine	-0.03055987	0.030257305	0.314275925
methioninesulfoxide.smc240	-0.054067476	0.053542974	0.314373235
c16.pcaec425	0.021598689	0.021395126	0.314507646
h1.kynurenine	-0.014129645	0.01399685	0.314520522
c51dc.pcaec406	-0.005037516	0.004990617	0.314563487
pcaac365.c51	0.068184962	0.067554473	0.314594272
ornithine.smc180	0.020103696	0.01991781	0.314594465
pcaec363.creatinine	-0.006134684	0.006079044	0.314680341
pcaec425.smc241	-0.065524433	0.064931396	0.314689711
pcaac422.c5	-0.032548302	0.032260011	0.314783742
pcaac323.methionine	-0.048867719	0.048435483	0.31478973
lysopcac170.alanine	-0.038570855	0.038230144	0.314795397
methionine.serine	-0.027296439	0.027057163	0.31482826
glutamicacid.pcaac426	0.143094873	0.141860843	0.314897303
pcaac281.pcaac426	0.047221062	0.04682247	0.314986241
pcaec342.pcaec301	-0.05045415	0.050035577	0.315056671

pcaac361.lysopcac161	0.107719644	0.106838611	0.315113633
pcaac344.pcaac365	-0.052923983	0.052500889	0.315203351
pcaac385.c51	0.043746258	0.04339769	0.315216186
pcaec340.pcaec404	0.03095748	0.030712903	0.315249011
pcaac406.c3	0.03422058	0.033950436	0.315252031
lysopcac203.pcaec322	0.080971629	0.080332699	0.315253694
creatinine.serine	-0.026322721	0.026119651	0.315339303
pcaec383.pcaac383	0.010689989	0.010609437	0.315426453
lysopcac140.pcaac281	-0.001130888	0.001122394	0.315438356
pcaac420.h1	0.010855261	0.01077406	0.315453123
pcaac365.h1	0.002178174	0.002161931	0.31546432
pcaac363.smc161	-0.061497247	0.061044975	0.315514329
smc240.alanine	-0.062542164	0.062086506	0.315547709
methionine.pcaac363	-0.014549876	0.014445011	0.315585747
pcaac360.c5	0.028869007	0.028663364	0.315626525
pcaac320.pcaac341	0.021574235	0.021429472	0.315827195
pcaec341.serotonin	-0.100056091	0.099417797	0.315987674
pcaec420.valine	0.040778988	0.04052176	0.316022369
pcaec401.c141oh	-0.107908203	0.107236375	0.316062127
citruline.pcaec444	0.121118122	0.120378918	0.316121682
pcaec384.pcaac341	0.008041708	0.007992988	0.316143387
pcaac386.pcaac365	0.078735128	0.07826156	0.3161646
pcaec300.smc181	0.043958866	0.043695062	0.316171167
smohc141.pcaac384	0.049128372	0.048843567	0.316270107
alphaaminoadipicacid.pcaec445	-0.038225674	0.03801017	0.316347451
smc202.serotonin	-0.100875263	0.1003292	0.31645626
asparticacid.pcaec421	0.004287916	0.004264755	0.316461952
pcaac321.pcaac322	0.111458895	0.11087174	0.316526717
phenylalanine.pcaec385	0.04575973	0.045520549	0.316546599
pcaec402.smc180	-0.004151258	0.004129613	0.316552885
pcaac321.creatinine	-0.011684288	0.011623847	0.316572799
c10.pcaec406	-0.005620801	0.005592021	0.316598244
lysopcac203.pcaec424	0.006880359	0.006849611	0.316913881
pcaac425.smc181	-0.011011871	0.01096484	0.317009734
pcaec342.arginine	-0.050412113	0.050204942	0.317087871
pcaec425.phenylalanine	0.056057727	0.05582815	0.31709474
pcaac385.lysopcac181	0.029803184	0.029684051	0.317142204
alanine.pcaec385	0.049644618	0.04944632	0.317143641
pcaec402.pcaac406	-0.076495533	0.076191331	0.317152181
c14.pcaac405	0.054627357	0.054412712	0.317175155
lysopcac161.pcaac383	-0.013647345	0.01359393	0.317182567
pcaec445.pcaac425	0.084423161	0.084103177	0.317242446
pcaec424.pcaac365	-0.061428103	0.061195387	0.317243326

glycine.asparagine	-0.009903113	0.00986657	0.317290968
pcaac406.c14	-0.063781378	0.063555052	0.317359456
pcaac420.lysopcac161	0.096878514	0.09653848	0.317378117
lysopcac160.pcaec364	-0.011523272	0.011484352	0.317442153
alphaaminoadipicacid.pcaec446	-0.056644409	0.056457383	0.31747882
pcaac361.taurine	0.060151978	0.059959247	0.317526074
asparagine.pcaec301	-0.085887703	0.085623395	0.317587359
smc261.pcaac341	0.014354894	0.014311866	0.317626031
c18.pcaec445	-0.076079265	0.075853346	0.317639519
smc241.pcaac341	0.010006997	0.009977395	0.317645064
lysopcac204.ornithine	0.036801149	0.036692902	0.317653116
c5.c51dc	0.258216556	0.257459899	0.317658477
pcaec403.smohc241	0.077069483	0.076851544	0.317708039
pcaec341.pcaac240	-0.018561647	0.018509969	0.317729175
pcaac425.pcaec404	0.020753626	0.020696986	0.317755748
pcaec406.pcaec362	0.02379037	0.023726359	0.317774369
pcaac360.pcaec406	-0.018563849	0.018519555	0.317921611
glutamine.pcaac422	-0.013493592	0.013461711	0.317932881
c51.pcaec340	-0.040357043	0.040265737	0.317981343
pcaec445.leucine	-0.005660705	0.005648741	0.31805331
glutamicacid.serine	0.037674502	0.037596764	0.31807752
smohc241.lysopcac181	0.039397532	0.039318609	0.318106606
histidine.smohc241	0.107903838	0.107691786	0.318124981
pcaac424.phenylalanine	0.079293153	0.079142316	0.318155382
c162.pcaac320	-0.063551575	0.063441718	0.318239255
pcaec361.pcaec424	0.007826552	0.007814217	0.318312976
c101.pcaec424	-0.015126615	0.01510513	0.318388124
smohc141.pcaec301	-0.062876145	0.062787838	0.318395809
glycine.smc180	0.029563537	0.02952303	0.318412364
serotonin.smohc141	0.0071999	0.007190886	0.318469442
pcaac364.pcaac426	0.067075047	0.067021979	0.31869185
pcaec444.alanine	-0.013180605	0.01317211	0.31876261
pcaac420.c2	-0.044447346	0.044419034	0.318766255
c141oh.c14	-0.05347039	0.053436972	0.318772042
pcaec363.smohc141	0.012536327	0.01252911	0.318795825
pcaec341.pcaac406	-0.042049299	0.042025858	0.318804615
c14.alanine	0.029078671	0.029063563	0.3188229
c16.pcaec424	0.015704603	0.015698504	0.318886198
pcaec361.pcaac341	0.010905437	0.010901711	0.318908691
pcaec403.pcaec321	0.049009685	0.048993029	0.318909597
smohc222.pcaec301	-0.075403501	0.075382451	0.318938863
pcaac240.c8	-0.07210462	0.072087133	0.318956536
smc260.pcaec406	0.004151566	0.004151004	0.319008226

pcaac361.serine	-0.018338211	0.0183362	0.319020617
smc180.lysopcac280	0.010599949	0.01059923	0.319040819
pcaec365.histidine	-0.072187963	0.072199204	0.319148575
c141oh.pcaac422	0.035364235	0.035370056	0.319152848
pcaac323.pcaec444	0.09143969	0.091468789	0.319226913
pcaac364.smohc221	0.059971065	0.060002635	0.319327249
pcaec403.asymmetricdimethylar[illegible]	-0.021500993	0.02151645	0.31942
smc202.alanine	-0.011204959	0.011213498	0.3194408
asymmetricdimethylarginine.pca[illegible]	0.05783974	0.057894183	0.319527144
pcaec421.c5	0.029191205	0.02921968	0.319543614
pcaec342.smohc161	0.009229055	0.009238689	0.319576597
pcaac361.pcaec446	-0.034381776	0.034418086	0.319582443
pcaac366.c51	0.140936233	0.141116933	0.319691319
acetylornithoine.pcaac403	-0.004049019	0.00405453	0.319729337
alanine.pcaec322	0.046870565	0.046937014	0.319756603
pcaec341.pcaac380	0.080554846	0.080682327	0.319835955
pcaac300.pcaec421	0.002611053	0.002615208	0.319840134
phenylalanine.lysopcac180	0.05576457	0.055854144	0.319847383
c101.smohc141	0.027378363	0.02742724	0.319933524
pcaac365.ornithine	0.040882648	0.040955724	0.319934598
methioninesulfoxide.c14	0.101971184	0.102165916	0.319993421
pcaac380.c162	-0.064389269	0.064516212	0.32002317
pcaec386.pcaec404	-0.011370752	0.011393569	0.320040083
pcaec423.pcaec383	-0.095099986	0.095300003	0.320086559
pcaac385.serine	-0.030712555	0.030785415	0.320216018
pcaac360.c162	-0.059598027	0.059745981	0.320269037
pcaac403.pcaec424	0.012873332	0.012906713	0.320322191
pcaec424.lysopcac181	-0.079570614	0.079787322	0.320384907
smohc141.pcaac360	0.058460939	0.058621124	0.320392869
smohc141.pcaac341	0.011914076	0.011947796	0.320436244
smc181.pcaac323	-0.117224801	0.117571329	0.320496757
histidine.pcaac321	0.038382903	0.038498593	0.320524645
c5.creatinine	-0.017906739	0.017960926	0.320530407
lysopcac160.smc180	-0.006469642	0.006489366	0.320541299
smc180.pcaac323	-0.127588664	0.127983661	0.320563947
c12.c5	-0.010665597	0.010698765	0.320570675
prolinr.pcaec422	-0.039945753	0.04007274	0.320603896
c10.pcaac322	0.053975308	0.054148278	0.320616213
pcaec386.c141oh	-0.114684806	0.115054949	0.320627207
smc261.c101	0.011565658	0.011603127	0.320633065
pcaec420.c18	-0.025429015	0.025513647	0.320675599
pcaec403.smc180	-0.004028479	0.004041996	0.320688678
smc260.asparticacid	0.050112297	0.050282281	0.320706307

smc240.smohc141	-0.048829062	0.04900471	0.320804869
pcaec301.smc260	-0.038378141	0.038521839	0.320875527
pcaac323.putrescine	-0.057371014	0.057587347	0.320888248
pcaac323.c14	-0.017245315	0.017310605	0.320895533
pcaec404.kynurenine	0.010154531	0.010195478	0.321013911
pcaec386.putrescine	-0.054965953	0.055188034	0.321017729
c10.pcaac363	-0.01129024	0.011336316	0.321037261
lysopcac281.pcaec404	0.08469384	0.085045323	0.321070409
pcaec406.pcaec385	-0.093454622	0.093854796	0.321133774
pcaec381.c51	-0.098871489	0.099295272	0.321135786
pcaec405.methionine	-0.049967604	0.050188899	0.321204231
phenylalanine.valine	0.098863461	0.099312256	0.321257402
pcaac384.pcaac361	-0.050889606	0.051129838	0.321344327
phenylalanine.pcaac383	-0.012260729	0.012320005	0.321398999
pcaec401.pcaec322	0.092857683	0.093314819	0.321441409
asparticacid.smc161	0.07144476	0.071801575	0.321475619
lysopcac160.pcaac365	-0.07302264	0.073398254	0.321547353
pcaac402.smohc161	0.026847003	0.026985821	0.321560263
pcaec424.kynurenine	0.005389645	0.005417918	0.321596263
lysopcac281.glutamicacid	0.072065077	0.072447827	0.321627633
asparagine.pcaec406	0.001484001	0.001492079	0.321690836
c8.lysopcac182	-0.099508635	0.100058893	0.321732454
pcaec300.alanine	-0.017670156	0.017768512	0.321749949
pcaac323.c142	0.023000658	0.023129245	0.321761634
pcaac341.isoleucine	0.027253455	0.027406306	0.321770216
pcaec362.pcaec406	0.006445028	0.006481221	0.321773704
c3.c10	0.016703527	0.016798937	0.321819812
pcaac404.pcaec360	-0.004810012	0.004837689	0.321840057
pcaec420.leucine	-0.007488809	0.007532706	0.321891586
c5.c12	-0.04798526	0.048275251	0.32197867
c4.pcaec384	0.08641891	0.086951146	0.322033994
pcaac320.leucine	-0.005201321	0.00523363	0.322059399
c141oh.lysopcac161	-0.088474618	0.089033239	0.322108326
pcaac322.pcaec301	-0.075037183	0.07552004	0.322166292
pcaec404.smohc241	0.080813448	0.081337045	0.322187456
pcaac401.pcaac341	0.005554888	0.005591318	0.322225375
pcaac361.lysopcac182	-0.013928602	0.014020528	0.322245301
pcaec340.alanine	-0.019952406	0.020086809	0.322310597
lysopcac204.leucine	0.033143934	0.033371931	0.322378993
smc161.smohc241	0.077543162	0.078084475	0.322427747
spermidine.c162	0.053158536	0.053531008	0.322440198
alanine.pcaec421	-0.002478867	0.002496662	0.322522399
c142.c51	0.039319087	0.039611986	0.322652014

smc241.c141oh	-0.096209324	0.096936877	0.322706046
c162.pcaec363	-0.01056598	0.01064617	0.322719094
pcaec404.smc180	-0.004093801	0.004124924	0.322725273
lysopcac160.pcaec422	0.081475961	0.08209841	0.322743118
pcaec343.pcaac385	-0.026978761	0.027185103	0.322747253
c16.c2	0.108225366	0.109067934	0.322812806
pcaec380.methionine	-0.047855129	0.048227786	0.322813698
c162.smohc141	0.016461894	0.016591875	0.32286567
pcaac426.prolinr	-0.048146497	0.048528396	0.322882992
pcaac385.c142	-0.031479142	0.031730035	0.32290122
pcaec364.lysopcac160	-0.010678275	0.010765319	0.322987975
pcaac341.lysopcac181	0.032584134	0.032852245	0.323024678
pcaac424.lysopcac182	-0.116585851	0.117546091	0.323028538
prolinr.tyrosine	-0.088290566	0.08902053	0.323043559
c141.pcaec380	-0.053949676	0.054395871	0.323044908
lysopcac204.asparticacid	0.025455584	0.025673299	0.323179834
citruline.pcaec343	0.08825561	0.089028027	0.323275091
isoleucine.lysopcac182	0.03416682	0.034466238	0.323280529
methioninesulfoxide.smc180	-0.00305972	0.003086876	0.323334128
pcaac363.pcaac380	0.059270143	0.059799832	0.323363422
smc241.pcaec321	0.089224459	0.090031964	0.323417606
pcaec443.pcaac281	-0.00375316	0.003787811	0.32350471
pcaac343.glycine	-0.111415139	0.112443865	0.32350504
pcaac300.c51	0.062391961	0.062971483	0.323531384
pcaac360.pcaac364	-0.068017233	0.068649906	0.323537717
pcaec404.smc261	-0.078262423	0.07900226	0.323610134
pcaec421.pcaac341	0.005832114	0.005887321	0.323616205
pcaac402.smc180	-0.003274678	0.003306211	0.323694213
lysine.lysopcac161	-0.092626253	0.093528487	0.32374734
lysopcac161.pcaac341	0.007132161	0.007201843	0.323761413
pcaac300.methionine	-0.041079819	0.041485243	0.323808732
pcaac342.pcaec446	-0.055583758	0.056139848	0.323873358
methionine.pcaac405	0.065066969	0.065730035	0.323962124
serotonin.spermidine	-0.072329379	0.073072027	0.323998907
pcaac323.pcaac402	-0.042248432	0.0426884	0.324068688
pcaac360.lysine	0.119169486	0.120418644	0.324101318
pcaac403.pcaac361	0.053375425	0.053936087	0.324111778
pcaac321.smohc241	0.041541947	0.041981619	0.324149791
pcaac363.pcaac240	-0.019220424	0.019423904	0.324151158
c12.lysine	0.065110735	0.065808482	0.32421301
lysopcac140.ornithine	0.032054948	0.032399954	0.324235261
smc240.phenylalanine	-0.050755514	0.051308047	0.324294023
pcaec406.c181	-0.016456383	0.016636712	0.324328281

pcaac421.pcaec364	-0.010935943	0.011057254	0.324392562
pcaec380.creatinine	-0.011769549	0.011903614	0.324534672
smc181.pcaac240	-0.011293887	0.011424846	0.32463222
histidine.pcaac323	0.120039729	0.121452467	0.324714848
c162.lysopcac180	-0.03980539	0.040278327	0.324768359
pcaac401.pcaac323	-0.118659018	0.120094112	0.324869856
c141oh.pcaac403	0.004370258	0.004423918	0.324957494
pcaac344.c14	0.061146091	0.061897669	0.324963799
histidine.pcaec406	0.005780588	0.005852093	0.325001152
ornithine.asparticacid	0.024386033	0.02469161	0.325077767
pcaec361.pcaac363	-0.017490253	0.017711055	0.325122276
c51.c12	-0.072862349	0.073782658	0.325125361
pcaec444.asparagine	-0.01424522	0.014427548	0.325205545
pcaec340.h1	0.002105254	0.002132314	0.325231476
pcaac341.lysopcac180	-0.04483515	0.045414444	0.325263278
pcaac300.pcaec404	0.01740117	0.017626196	0.325268563
smohc221.pcaac383	0.005697741	0.005771773	0.325297839
glycine.pcaec322	0.037296515	0.03778801	0.325385828
pcaec300.glutamicacid	0.05013768	0.050799714	0.325398324
pcaac404.pcaec380	0.046132752	0.046743386	0.32541362
glutamine.pcaac320	-0.024735946	0.025066066	0.32546563
c2.pcaac383	-0.009013997	0.009134997	0.325502593
pcaec322.serotonin	-0.092120003	0.093360775	0.325524292
pcaac281.pcaec406	0.003627638	0.003676989	0.325588429
c3.pcaec340	-0.030007772	0.030418574	0.325629295
smc202.isoleucine	0.024561834	0.024898699	0.325641251
pcaec321.pcaac422	-0.015847822	0.01606577	0.325659089
acetylornithoine.pcaec424	0.021871416	0.022174157	0.325701602
pcaac384.c51	0.027118258	0.027497026	0.325761208
smc202.pcaac240	-0.009790569	0.009927331	0.325761892
pcaec444.c5	0.019149757	0.01942046	0.325841483
pcaec425.spermidine	-0.067053561	0.068011778	0.325914785
serine.pcaec383	-0.083305139	0.084496968	0.325922612
pcaac386.pcaec422	-0.024731587	0.025085446	0.325923188
pcaac381.pcaec385	-0.050765058	0.051493516	0.325942958
pcaac364.serotonin	-0.106926016	0.108464386	0.325960848
lysine.pcaec340	-0.042665647	0.043282548	0.325994931
smohc221.pcaac385	-0.026014934	0.026394385	0.326055235
pcaac424.kynurenine	0.014773274	0.014989599	0.326082381
smc161.serine	-0.032556611	0.033034204	0.326095026
pcaec421.smc180	-0.003801401	0.003857176	0.326096276
spermidine.c101	-0.006276166	0.006368677	0.326128507
pcaac363.pcaec424	0.00705231	0.007156903	0.326171683

pcaec405.pcaec340	0.050467003	0.051216465	0.326180973
pcaec385.pcaac422	-0.070095605	0.071140503	0.326207678
pcaac344.smohc161	-0.01706119	0.017315971	0.326220311
pcaac322.c101	-0.008224696	0.008350415	0.326387588
pcaec384.alanine	-0.027356	0.027777274	0.326441768
creatinine.pcaac341	0.022120808	0.022467439	0.32657004
pcaac361.pcaac323	-0.098882978	0.100432594	0.326570654
pcaac403.smc180	-0.003476343	0.003531303	0.326636307
serotonin.pcaec406	-0.023051712	0.023416404	0.326641544
pcaac360.alanine	-0.016024468	0.016285717	0.326870485
pcaec360.pcaec380	-0.068398392	0.069516608	0.326892037
pcaec342.lysopcac180	0.097356656	0.098954602	0.326922744
pcaec444.pcaac386	0.034720592	0.035295354	0.32698942
pcaac383.pcaac322	0.078590684	0.079894813	0.3270084
pcaec401.c101	-0.021923891	0.022288336	0.327022274
pcaac366.alphaaminoadipicacid	-0.014080819	0.014315402	0.327039612
pcaec402.pcaec362	0.018677619	0.018989771	0.327064658
asparticacid.c142	0.041760317	0.042459609	0.3270802
citruline.pcaac360	-0.040517295	0.041204558	0.327183001
pcaec343.smohc141	0.009640478	0.009807102	0.327335415
serotonin.pcaac403	-0.003665046	0.003729333	0.327457029
pcaac380.smohc161	0.021955463	0.022341192	0.32747038
c12.c51	0.023925691	0.024349505	0.3275391
pcaec424.pcaec364	-0.009465624	0.009634084	0.32757855
pcaec424.pcaec362	0.010272619	0.010455864	0.327598009
arginine.pcaac405	-0.05543809	0.056428609	0.327611739
pcaec445.pcaac422	-0.010290992	0.010475221	0.327628242
pcaec406.methioninesulfoxide	0.030314661	0.03086034	0.327674885
pcaec300.pcaac383	0.00402728	0.004099991	0.327700462
c14.pcaec424	-0.015568892	0.015850965	0.327730396
pcaec423.pcaec364	-0.009936995	0.010117368	0.327746463
methioninesulfoxide.asparticacid	0.090644145	0.092294244	0.327771305
c141oh.pcaec363	-0.014681343	0.01494935	0.327795336
pcaac361.pcaec421	0.003456908	0.003520693	0.327888371
lysopcac140.pcaec402	0.077492332	0.078933056	0.327954808
pcaac383.prolinr	0.052864242	0.053849398	0.327975514
c8.pcaac363	-0.010994627	0.011199923	0.327992898
smohc222.pcaec445	-0.028705987	0.029248935	0.328107278
pcaec401.pcaec446	-0.060414937	0.06156432	0.328159656
pcaec382.c51	-0.099975898	0.101886735	0.328201358
pcaac366.pcaec422	-0.040588608	0.041364634	0.328204355
lysopcac161.alanine	-0.053551569	0.054585271	0.328291187
pcaac321.lysopcac181	0.05601146	0.057093253	0.328296328

smohc141.asparagine	0.008404285	0.00856821	0.328386711
pcaec301.c181	0.014051227	0.014325941	0.328408439
c181.lysopcac181	0.048522554	0.049475109	0.328446381
pcaec406.c101	-0.069687488	0.071055841	0.328448457
c3.smc160	0.091078541	0.092880275	0.328517776
pcaec341.pcaac341	-0.028594583	0.02916114	0.328532544
pcaac405.asparagine	0.014713055	0.01500591	0.328575537
pcaac365.pcaec343	0.093675872	0.095547968	0.328613536
pcaac321.smc161	-0.038553327	0.039325209	0.328630708
pcaec386.asymmetricdimethylar	0.04131321	0.042140398	0.328631271
pcaec321.pcaac364	-0.055288954	0.056398684	0.328654471
pcaac381.alanine	-0.020211952	0.020620842	0.328729432
c18.pcaec361	-0.017284064	0.017634418	0.328748429
pcaec406.pcaac361	0.095688509	0.097655742	0.328884647
smc241.pcaac386	0.020542166	0.020966237	0.328924891
pcaec403.pcaec342	0.08898221	0.090822365	0.328941942
pcaac385.pcaec420	-0.030386213	0.031015623	0.328957818
c14.pcaec301	-0.053293149	0.05440594	0.329036624
pcaac365.pcaec406	0.012774321	0.0130434	0.329123243
methioninesulfoxide.c101	0.027693035	0.028276673	0.329128544
pcaac386.lysopcac181	0.024021666	0.02453267	0.329221694
pcaac362.pcaac344	-0.053078185	0.054212786	0.329270494
pcaec382.kynurenine	0.010608724	0.010837254	0.329348661
smc180.kynurenine	-0.004777033	0.004881235	0.329476753
smc261.pcaac323	0.071467922	0.073027114	0.329478372
pcaec443.asparticacid	0.059851847	0.061161073	0.329505621
pcaac363.pcaec420	-0.039758909	0.04062969	0.329518391
pcaec341.phenylalanine	0.049980901	0.051086801	0.32962446
pcaec382.pcaac363	-0.033295724	0.034032994	0.329632299
c141oh.phenylalanine	0.052191496	0.053347377	0.329634117
lysopcac170.ornithine	0.02917263	0.029819754	0.329650934
pcaac385.h1	0.00119916	0.001225964	0.329730628
pcaac404.pcaec420	-0.041529626	0.042459163	0.329745173
putrescine.pcaec402	0.077730685	0.079474681	0.329770586
pcaec342.kynurenine	-0.007969312	0.008148158	0.329773165
pcaec405.pcaac341	0.005915472	0.006048712	0.329811845
pcaec405.pcaac344	0.061254906	0.062645177	0.329893163
pcaec301.pcaac403	0.005168579	0.005286639	0.329961696
pcaec340.pcaac281	-0.004232739	0.004329531	0.3299738
pcaec424.pcaec363	0.04195885	0.042922134	0.330016303
smc180.pcaac403	-0.012901002	0.013200779	0.330147664
c0.pcaac300	-0.062966139	0.064429463	0.33014916
pcaac320.c14	-0.050382946	0.051562677	0.33023178

pcaec382.pcaec362	0.016996711	0.017400832	0.330401809
pcaac366.pcaec343	0.085152148	0.087179556	0.330417252
pcaec401.pcaec424	0.009789237	0.010023424	0.330470745
smc261.lysopcac180	0.068162735	0.069797797	0.330501208
lysopcac280.pcaac383	-0.022634083	0.023180827	0.330580355
pcaac404.smc181	-0.018178775	0.018621316	0.330668835
phenylalanine.smc180	-0.016173683	0.016568914	0.330712507
asymmetricdimethylarginine.lyso	-0.011898491	0.012189485	0.330721771
pcaac422.putrescine	-0.044904278	0.046006836	0.330767461
pcaec443.pcaac383	0.009086524	0.00931041	0.330807828
leucine.pcaec380	0.045322871	0.046448747	0.330902768
lysopcac203.smohc161	0.029000594	0.029723789	0.330947905
lysopcac180.pcaec443	0.080225176	0.08222855	0.330964196
pcaec322.pcaac360	0.066137293	0.0677924	0.33098932
smohc161.alanine	-0.032296326	0.03310585	0.331008215
citruline.pcaac426	-0.050905858	0.052184136	0.331029427
c8.lysopcac170	-0.020480688	0.020995472	0.331040937
pcaac365.c141oh	-0.082535235	0.084618829	0.331092537
c12.c162	-0.137870307	0.141354911	0.331106454
pcaec301.lysopcac181	0.03479697	0.035676462	0.331106668
pcaac406.pcaec340	0.081196231	0.083268469	0.331222457
spermidine.glycine	0.165352541	0.169601233	0.33130393
pcaac404.c141oh	-0.137275398	0.140827277	0.33138819
pcaac281.pcaec425	0.008945285	0.009178279	0.331469176
pcaac424.c2	0.118399324	0.121483566	0.331470561
pcaec443.pcaac422	-0.095227793	0.097732343	0.331588482
pcaac420.pcaac403	-0.006925839	0.007108463	0.331620328
pcaec424.pcaac363	-0.015948861	0.016369566	0.331625009
c3.pcaac420	0.046833038	0.048082894	0.331770091
pcaec342.alanine	-0.019811107	0.020340597	0.331788589
lysopcac140.pcaac321	-0.028040572	0.028793408	0.331845461
pcaac383.pcaac420	-0.069160756	0.07102591	0.331901902
pcaac383.pcaac341	-0.008677044	0.008911271	0.331913872
asymmetricdimethylarginine.smc	-0.024769534	0.025442775	0.332001271
smohc221.pcaac403	-0.00777238	0.007984275	0.332039907
pcaac281.creatinine	-0.005440485	0.005589129	0.332067708
pcaec364.putrescine	-0.100066461	0.102813036	0.33212665
serine.smc180	-0.009912645	0.010185164	0.33214751
pcaec404.pcaac361	0.054205669	0.055701464	0.332195725
pcaac365.smohc222	-0.006600422	0.006783759	0.332280902
c51dc.pcaac300	-0.047653129	0.048978147	0.332294495
ornithine.pcaac381	0.018033061	0.018539405	0.332422547
ornithine.pcaac384	0.06033753	0.062033194	0.332433934

pcaec445.c182	0.050353754	0.051769443	0.3324395
pcaec402.pcaec401	0.048918275	0.050294767	0.332450631
pcaec321.pcaec385	-0.051135165	0.052574207	0.332452186
kynurenine.serine	-0.041337554	0.042502886	0.332475019
c2.smc261	0.042856182	0.044073115	0.332571113
c4.pcaec343	-0.081093409	0.083426351	0.332745762
acetylornithoine.pcaec425	0.010114635	0.010405923	0.33275987
pcaac361.pcaec406	0.005154981	0.005303862	0.33279839
pcaec300.pcaec380	-0.056201466	0.057829492	0.332838994
asymmetricdimethylarginine.argi	-0.090180802	0.092808905	0.332920894
pcaec362.pcaac363	-0.019976019	0.020558513	0.33292889
pcaac420.pcaec424	0.032848784	0.033808607	0.332956863
pcaac361.pcaec444	0.104205357	0.107259512	0.332998791
creatinine.asymmetricdimethylar	-0.019232568	0.019800578	0.333103962
smc260.pcaac402	-0.08797906	0.090590131	0.333171627
pcaac381.smc181	-0.005729135	0.005901068	0.333326871
c8.pcaec444	-0.107606489	0.110841122	0.333350096
pcaec422.leucine	-0.03963332	0.040827522	0.33338351
pcaec383.pcaac381	0.041962834	0.043229163	0.333405075
pcaac381.smc180	-0.00389615	0.004013963	0.333433546
pcaac405.c12	0.067406462	0.06945877	0.333531029
smohc221.pcaac380	0.060405587	0.062254077	0.333603282
alanine.smc261	0.021731849	0.022397588	0.333618677
pcaac383.pcaec424	0.004836239	0.004984848	0.333662635
pcaac343.pcaec445	0.072909116	0.075152118	0.333679493
pcaac365.smohc161	-0.018697251	0.019272956	0.333691871
glutamine.c10	-0.008250343	0.008505052	0.333730045
smc181.alanine	-0.02117752	0.021832839	0.33376347
serine.pcaac360	-0.040403896	0.04165551	0.333779124
c14.smc160	-0.076852953	0.079249917	0.333877896
pcaac344.lysopcac204	-0.019498847	0.020107906	0.333899707
pcaac380.c5	0.01959434	0.020206985	0.333914076
pcaac342.smc161	-0.082695008	0.085285716	0.333943064
asparticacid.lysopcac160	-0.024265658	0.025028587	0.333995483
pcaac300.pcaec444	0.093124114	0.096086999	0.334170975
pcaac344.c141	-0.043376727	0.044769499	0.334307379
ornithine.pcaec380	-0.043040704	0.044422996	0.334310739
pcaec301.pcaac363	0.009838826	0.010155503	0.334343679
pcaec301.pcaac341	-0.012044968	0.012433914	0.334392519
pcaec361.c141	0.036729878	0.037916708	0.33440242
kynurenine.leucine	0.033622125	0.03471719	0.334522506
smc181.smohc141	-0.027649331	0.028550169	0.334527662
isoleucine.putrescine	-0.036913172	0.038122234	0.334608547

pcaec443.smohc241	0.064618473	0.066740808	0.33465046
pcaec300.pcaac406	-0.037526245	0.038764788	0.334725365
pcaec360.pcaec361	-0.010322032	0.010662759	0.334727716
pcaac344.pcaec380	-0.063074123	0.065161918	0.3347701
pcaac342.pcaac422	-0.018273997	0.018879186	0.334777933
pcaac386.pcaec406	-0.023893588	0.024687347	0.334826003
phenylalanine.pcaac381	0.020799672	0.021491467	0.33484434
pcaac403.pcaec365	0.023796092	0.024589748	0.334887436
methionine.smc202	0.062106147	0.06418019	0.334907377
pcaac386.ornithine	0.019366959	0.020013798	0.334909247
pcaac424.smc180	-0.004552794	0.004704874	0.334911298
pcaec446.pcaec406	0.004505392	0.004656903	0.335016293
glutamicacid.pcaac385	-0.028441403	0.029410327	0.335220608
pcaec405.c14	-0.061159975	0.063246021	0.335239546
pcaec421.pcaec404	0.058549254	0.060548473	0.335257201
pcaec362.taurine	0.079684333	0.082424858	0.335371913
pcaec444.pcaac422	-0.010069041	0.010417844	0.335487803
pcaec365.leucine	-0.009309471	0.009634277	0.33560359
pcaec364.pcaac385	-0.018927901	0.019589054	0.335622272
c51dc.asparticacid	0.019099927	0.019767267	0.335626612
smc160.pcaec343	-0.079806332	0.082604981	0.335686446
pcaec443.c3	-0.053963544	0.055856504	0.335691335
smohc141.pcaec406	0.004570357	0.004730822	0.335705928
smc161.pcaac420	-0.074174376	0.076799724	0.335838244
creatinine.valine	0.067453078	0.069842347	0.335850771
pcaec380.pcaec362	0.017917402	0.018553368	0.335884776
pcaac380.pcaec365	-0.026780728	0.02773686	0.335981461
kynurenine.pcaac322	-0.060595109	0.062763229	0.336017829
pcaec300.lysopcac161	0.061737522	0.063961612	0.336131501
c16.phenylalanine	0.050514501	0.052337952	0.336165264
c182.pcaec420	-0.017718032	0.018359459	0.336213792
pcaac403.pcaec361	0.010890202	0.011285005	0.336237503
pcaec300.pcaec401	0.066295972	0.068724001	0.336409921
pcaac323.pcaac385	0.030680406	0.031811975	0.336529954
pcaec362.tyrosine	-0.063359367	0.06570514	0.336595345
asymmetricdimethylarginine.pca	-0.057620101	0.059762042	0.336665098
pcaac362.pcaac341	0.021154909	0.021942082	0.336682015
smc160.isoleucine	0.02213794	0.022964642	0.336743893
lysopcac161.lysine	0.105778346	0.109735615	0.336775325
smohc221.pcaec300	0.103316252	0.107182379	0.336779674
alphaaminoadipicacid.pcaac281	-0.002944409	0.003054652	0.336789424
alanine.pcaec380	0.048418037	0.050231638	0.336796762
pcaac322.pcaac320	-0.053235345	0.055234598	0.336842188

lysine.pcaec423	-0.009543032	0.009903337	0.336935404
alphaaminoadipicacid.c141oh	-0.113913388	0.118218467	0.33695243
c51.creatinine	-0.008483977	0.008805074	0.336977927
pcaac380.pcaec364	-0.01884533	0.01955919	0.336992958
pcaac385.c16	0.035380718	0.036725325	0.337050504
arginine.spermidine	0.069292218	0.071927127	0.337060753
pcaec343.pcaac240	-0.013918001	0.014455392	0.337332146
smohc222.pcaac320	0.099353686	0.103198205	0.337371096
c4.pcaec406	-0.011353245	0.011792971	0.337387789
pcaec363.pcaec301	-0.050047296	0.051985836	0.337389115
pcaec406.c142	-0.066518315	0.069095929	0.337396667
methionine.pcaac366	-0.039392495	0.040919882	0.33740739
creatinine.smc161	-0.082847239	0.086060226	0.337411328
pcaec341.lysopcac160	-0.027814698	0.028894197	0.337424463
pcaac426.lysopcac182	-0.017658411	0.018346047	0.337484972
lysopcac204.h1	0.002765362	0.002873125	0.337497914
alphaaminoadipicacid.lysine	-0.096876354	0.100652705	0.337503597
lysopcac281.pcaec383	-0.058889824	0.06118664	0.337513218
pcaac281.transhydroxyproline	0.035120961	0.036491653	0.3375252
pcaec385.pcaec360	-0.004465519	0.004640446	0.337592434
pcaec420.pcaac406	-0.071243734	0.074037158	0.337609364
pcaec402.lysopcac280	-0.016672204	0.017328758	0.337688473
pcaec406.pcaac420	-0.105783835	0.109953731	0.33770648
smohc221.c14	-0.032212281	0.033483952	0.337733724
pcaac402.pcaec404	0.052147991	0.054209362	0.337757527
smc202.c18	-0.027235642	0.028318284	0.3378602
pcaac380.pcaec404	-0.011903611	0.012377245	0.337877872
lysopcac181.pcaec422	0.046803925	0.048666258	0.337878338
pcaec406.tyrosine	-0.068597537	0.071329325	0.33789376
pcaec404.pcaec300	-0.046838677	0.048704816	0.337902289
smc181.pcaac281	0.001824113	0.001897254	0.33802031
acetylornithoine.lysine	0.103145022	0.10729873	0.338100758
smohc222.lysopcac204	-0.03319121	0.034530871	0.338143025
pcaec322.lysopcac160	-0.017823297	0.018543042	0.338152443
pcaac424.smohc241	0.063135456	0.06569324	0.338212782
smc260.pcaec383	-0.050455076	0.052506703	0.338282091
pcaac406.prolinr	0.028840301	0.030015398	0.338320252
creatinine.pcaac240	0.017251187	0.017957866	0.338421608
pcaec401.smc180	-0.006384933	0.006647697	0.338509367
pcaec402.pcaec384	-0.06635151	0.069121535	0.338783893
pcaac281.smc240	-0.045822251	0.04774173	0.338849475
smc261.ornithine	0.031539703	0.032861825	0.338863162
pcaac385.pcaac281	0.002049775	0.002135944	0.338918007

ornithine.pcaec301	-0.103335159	0.107702022	0.339020192
smohc221.c16	0.034498688	0.035958712	0.3390488
pcaec300.pcaac320	0.104312402	0.108731506	0.339068648
smc240.pcaac406	-0.071666929	0.07470674	0.33909251
pcaac406.glutamicacid	0.03710957	0.038684361	0.339101949
pcaec424.smc180	-0.005627902	0.005867886	0.339196817
lysopcac281.pcaec403	-0.01248228	0.013015177	0.339220175
methionine.pcaec421	0.00462659	0.004824272	0.3392364
pcaac385.smc241	0.072282482	0.075374963	0.339262171
h1.methioninesulfoxide	0.045983851	0.047953546	0.339285817
lysopcac280.pcaec422	-0.027536498	0.028717167	0.339305219
prolinr.pcaac380	-0.055374554	0.057749277	0.339309003
pcaac365.lysopcac181	0.031379412	0.032726166	0.339324529
pcaac323.lysopcac180	-0.042268485	0.044087408	0.339377258
acetylornithoine.smohc241	-0.075751504	0.079014086	0.339394293
pcaec361.phenylalanine	0.055200788	0.057606056	0.339626602
asparticacid.pcaec425	-0.006838723	0.007137615	0.339687804
pcaac362.pcaec301	0.079474128	0.082961781	0.339770053
pcaec445.smohc141	-0.021212743	0.022147552	0.339854934
smc240.isoleucine	0.020808883	0.021727487	0.339890231
pcaac402.kynurenine	0.018655934	0.019480367	0.339911739
smc180.smc240	0.029760651	0.031075976	0.339914198
acetylornithoine.valine	-0.010069399	0.010514493	0.339916885
asparagine.c14	0.061780893	0.064518667	0.339968329
smc241.kynurenine	-0.004751894	0.004962993	0.340018966
pcaec423.pcaec300	0.037737074	0.039414185	0.340027215
smc241.serine	-0.027311672	0.028526084	0.340037789
pcaec445.pcaec443	-0.042691869	0.044592489	0.340062912
glutamine.c8	-0.043923964	0.045888723	0.340160324
lysopcac181.pcaac322	-0.098478354	0.102901708	0.340246047
pcaac426.lysopcac204	-0.028931869	0.03023298	0.340271153
pcaac422.pcaac240	-0.023474884	0.024531451	0.340288154
smc180.serine	-0.022559205	0.023576048	0.340318521
pcaec425.c182	-0.033582393	0.035096834	0.340328602
lysopcac140.glutamicacid	0.037757108	0.039461681	0.340351391
c5.tyrosine	0.11756271	0.122871317	0.340355895
asparagine.tyrosine	-0.041243327	0.0431149	0.340458685
pcaec402.transhydroxyproline	0.033683256	0.035214401	0.340494758
pcaec445.lysopcac160	-0.007896978	0.008256492	0.340526238
lysopcac170.phenylalanine	0.054898535	0.057397926	0.340527133
c142.pcaec300	0.035976212	0.037614272	0.340529102
pcaec301.taurine	-0.027770574	0.029036594	0.340555242
smohc241.c181	-0.014742569	0.01541474	0.340557708

pcaec343.lysopcac161	0.10161939	0.106253856	0.340563332
c3.lysopcac180	0.050559109	0.052885116	0.340747198
methioninesulfoxide.pcaec444	-0.114966442	0.120261074	0.340769298
asymmetricdimethylarginine.pca	-0.034531446	0.036124607	0.340807397
pcaac426.pcaac323	0.077475882	0.081053649	0.340826984
pcaec341.pcaec406	0.005172173	0.005411364	0.340857645
smohc141.c51dc	-0.121673668	0.127301505	0.340861213
phenylalanine.lysopcac160	-0.071690615	0.075006711	0.340862188
c12.pcaac405	0.069645784	0.072870686	0.34088458
alphaaminoadipicacid.smc181	-0.033570258	0.035126157	0.340904456
c101.pcaec425	0.009274413	0.009704376	0.34091025
creatinine.pcaec383	0.072119095	0.075475566	0.340993265
pcaac342.smohc141	0.019025887	0.019912523	0.341021246
c8.pcaac405	-0.074641584	0.07812106	0.341027803
c162.c5	-0.006507917	0.006811891	0.341070362
c8.pcaec380	0.080768114	0.084543325	0.341085533
pcaac406.c2	0.070744123	0.074059759	0.341143759
c182.pcaac363	0.009484666	0.009929297	0.341148741
pcaec340.pcaec342	-0.156430861	0.163791133	0.341227992
serine.lysopcac181	0.033053173	0.034612222	0.341281583
pcaac364.serine	-0.043325936	0.045377354	0.341364563
alanine.pcaec361	0.009631831	0.010089894	0.341460462
smc202.kynurenine	-0.004399148	0.00460878	0.341504426
kynurenine.smc180	0.013846792	0.014506805	0.341510124
pcaec422.pcaec406	0.00861592	0.009028942	0.34163489
pcaac344.glycine	0.034959798	0.03663639	0.341644373
smohc161.pcaec363	-0.04818699	0.050501537	0.34167876
c102.taurine	-0.018689715	0.019589199	0.341722208
alanine.asymmetricdimethylargir	0.015071638	0.01579753	0.341738533
pcaac365.smohc141	-0.022942796	0.024048062	0.341744058
pcaec340.glutamicacid	0.042494133	0.044543266	0.341765491
leucine.pcaac300	-0.041730799	0.043748903	0.341829067
pcaac361.asymmetricdimethylarg	0.024505931	0.025691551	0.341838662
pcaac360.pcaec322	-0.128167151	0.134385138	0.341900018
lysopcac181.smc202	-0.048996388	0.051394879	0.34210084
pcaec404.pcaac384	0.048489053	0.050869201	0.342162249
smohc221.pcaec360	-0.007718166	0.008097516	0.342191623
pcaec340.pcaec380	-0.049137118	0.051553946	0.342207685
smc161.pcaec406	0.009551984	0.010022275	0.342230363
pcaac344.c18	0.048153512	0.050524418	0.34223109
glutamicacid.creatinine	-0.01289832	0.013535773	0.342315898
kynurenine.acetylornithoine	-0.062345831	0.06543012	0.342338481
smc202.pcaec360	-0.004751494	0.0049866	0.342342962

pcaec382.pcaac406	-0.100851968	0.105853094	0.342392626
pcaec425.pcaac385	-0.046408909	0.048719065	0.342479481
smohc141.pcaec382	-0.018331215	0.019255708	0.342779286
pcaec300.pcaac364	-0.057971185	0.060911212	0.342908692
pcaec386.glycine	0.05112043	0.053724934	0.343015394
pcaac281.serine	-0.012228786	0.012852028	0.343023077
pcaec385.asymmetricdimethylarg	0.03816384	0.040111964	0.343060226
pcaac380.methioninesulfoxide	0.020604114	0.021656218	0.343067759
pcaac365.pcaac300	-0.070913683	0.074537505	0.34308563
c10.c101	0.022136876	0.023268846	0.3431008
smc261.pcaac381	-0.045313706	0.047635013	0.343143108
glutamicacid.lysopcac161	0.070281467	0.073885018	0.343164001
smohc241.pcaac422	-0.022657866	0.023822942	0.343231375
pcaec425.pcaac365	-0.051056934	0.053690249	0.343302584
pcaac364.pcaec322	0.069150319	0.072722073	0.343337336
kynurenine.pcaac300	-0.043546252	0.045819602	0.343590369
pcaec361.pcaac406	-0.039774618	0.041859298	0.343684947
prolinr.smohc241	0.035555405	0.037422368	0.343728928
citruline.pcaac240	-0.026368415	0.027754448	0.343754338
pcaec342.lysopcac161	0.098241407	0.103405482	0.343754789
prolinr.lysine	-0.070940402	0.074674815	0.343789694
pcaac323.pcaac422	-0.025662441	0.027016338	0.343842864
pcaec364.pcaec383	0.047837003	0.050365127	0.343884339
serotonin.c5	-0.017929222	0.018880604	0.343982302
c5.pcaec406	-0.011013575	0.011598388	0.343998793
smc160.pcaec421	0.002620753	0.002759998	0.344013619
c0.pcaec446	0.088339639	0.093033454	0.344014522
pcaec386.c102	0.027937425	0.029426961	0.344098148
lysopcac161.pcaec404	-0.038101748	0.040133305	0.344099234
pcaec380.smohc141	-0.023093491	0.024326915	0.344140683
lysopcac140.pcaec443	-0.079830419	0.084097266	0.344158422
pcaac362.glycine	-0.036218393	0.038157718	0.34420244
pcaec360.pcaac420	-0.140111999	0.147621484	0.344225715
glutamine.pcaec406	0.003289574	0.003466968	0.344376326
pcaec446.lysopcac160	-0.008443891	0.008899323	0.344380839
serine.pcaac341	0.00330691	0.003485275	0.344381286
smc240.pcaec421	-0.002156436	0.002272943	0.344422409
pcaec322.pcaec363	-0.04001879	0.042192732	0.344557311
pcaec420.pcaac403	-0.009489318	0.010005145	0.344573559
pcaec420.pcaec363	0.026751113	0.028205605	0.344579322
pcaac401.c182	-0.04029244	0.042488181	0.344635787
c2.pcaec424	-0.017644018	0.018606038	0.344648893
c142.pcaac385	0.008168944	0.008615738	0.344726558

pcaac322.c102	0.046283633	0.048818514	0.344760411
pcaac362.lysopcac203	-0.022897792	0.02415355	0.344793908
glutamicacid.pcaec321	0.022928206	0.024186012	0.344801465
pcaec421.pcaec403	-0.090283591	0.095245436	0.34484703
pcaec403.pcaec444	-0.147282443	0.15537782	0.344850012
pcaac281.putrescine	-0.024222242	0.025555214	0.344880052
serine.pcaec365	0.032493027	0.034284811	0.344931452
c51dc.lysopcac170	0.009587842	0.010117071	0.344956183
pcaac401.smc180	-0.003566843	0.003765013	0.345120667
creatinine.pcaec361	0.011701145	0.012351483	0.345129811
lysopcac140.pcaac364	-0.064199086	0.067768407	0.345138307
glutamine.pcaac385	0.010359631	0.010936452	0.34517564
pcaec300.pcaec425	0.008255827	0.008716771	0.345245273
lysopcac181.alphaaminoadipicac	0.011992859	0.012667906	0.345452349
lysopcac280.pcaec363	-0.023061433	0.024360717	0.345476373
pcaec343.c51	0.060921419	0.064362252	0.345539948
c142.pcaec361	-0.012597397	0.013308934	0.345541322
pcaac406.pcaac422	-0.042634775	0.045047598	0.34559137
c142.pcaec363	-0.011609634	0.012267465	0.345623082
pcaec446.pcaac425	0.085844872	0.090709223	0.345623944
pcaec343.serine	-0.016671338	0.017616605	0.345640199
pcaec445.pcaac300	0.045170182	0.047732207	0.345648951
lysopcac281.pcaac322	-0.035289704	0.037296232	0.345712332
c181.pcaec420	-0.01773679	0.018747245	0.345762716
smohc221.serine	-0.022727199	0.02402338	0.345791227
pcaec386.pcaec425	0.006381397	0.006745369	0.345793212
c162.kynurenine	-0.006047687	0.006392876	0.345812034
c101.pcaec343	-0.071801253	0.075901287	0.345823241
putrescine.lysopcac170	-0.015135526	0.016000897	0.345856114
h1.tyrosine	0.08041125	0.08501738	0.345904916
methioninesulfoxide.pcaac344	-0.083075773	0.08785576	0.346021084
pcaec362.kynurenine	-0.007130999	0.007541683	0.346045491
c141oh.serine	0.028442055	0.030084315	0.346113286
pcaec386.pcaec444	0.120132913	0.127079302	0.346150556
pcaac281.pcaec404	-0.015540914	0.016441358	0.346204031
pcaac343.alphaaminoadipicacid	-0.011230092	0.011882278	0.346265194
pcaac422.lysine	0.088437834	0.09358827	0.346339292
serotonin.smc181	0.027337431	0.028929582	0.346340558
pcaac402.lysopcac170	-0.043034208	0.045549036	0.346430107
pcaac383.pcaac421	-0.047014457	0.049762451	0.346435605
pcaec383.acetylornithoine	0.075328317	0.079734466	0.346454957
pcaec321.c142	0.049051816	0.051927911	0.346519083
pcaac383.pcaac365	-0.035401232	0.037483128	0.346598424

pcaac364.lysopcac204	-0.014871411	0.01574644	0.346612567
pcaec322.pcaac380	0.07258292	0.076860837	0.34665734
c182.phenylalanine	0.036655305	0.038816086	0.346662016
pcaac385.serotonin	-0.122254533	0.129473285	0.346706613
smohc222.creatinine	-0.006256713	0.006626554	0.346735689
smohc241.smc260	0.080361373	0.085118993	0.346777372
smohc241.asymmetricdimethyla	0.024833028	0.026306504	0.346837495
smc181.smohc222	-0.007678091	0.00813421	0.346869236
c51.prolinr	0.034118095	0.036148414	0.346916085
pcaac281.pcaac422	-0.013358752	0.014155023	0.346960592
pcaac323.pcaac380	0.041643041	0.044128705	0.346998295
c51.smohc221	0.053010184	0.056189523	0.347128093
c141oh.pcaac381	-0.011069213	0.011733631	0.347149863
pcaac405.pcaac341	-0.006226954	0.006600723	0.347150042
c51dc.histidine	0.084331543	0.089399925	0.347184616
pcaec360.lysopcac181	0.031323628	0.033211203	0.34725705
alphaaminoadipicacid.pcaac341	0.004889628	0.005184867	0.347311513
c18.pcaec363	-0.017531431	0.018591568	0.347352314
pcaec420.pcaac386	0.017847997	0.018928007	0.347370853
pcaac401.pcaec406	0.056104021	0.05950633	0.347430374
pcaec446.pcaac422	-0.017043083	0.018077135	0.347443931
pcaec420.pcaec385	-0.052548253	0.055739216	0.347467317
pcaac385.c181	-0.01067123	0.011319937	0.347497175
pcaac363.pcaec425	0.005612905	0.00595513	0.347579087
c181.pcaac381	-0.008764366	0.009300273	0.34765835
pcaac383.lysopcac182	-0.017418923	0.018485282	0.347691076
kynurenine.pcaac323	0.143736115	0.152549256	0.34773468
c182.smc160	-0.066207749	0.070271728	0.347765243
lysopcac161.pcaec424	0.012194567	0.012943127	0.347766371
lysopcac180.smc180	-0.00909616	0.009655192	0.34779955
pcaac384.pcaec322	0.074776145	0.079377973	0.347837302
c181.pcaec443	0.0775608	0.082352036	0.347942558
pcaac361.pcaec322	0.066988376	0.071126923	0.347945338
citruline.pcaac386	0.024599119	0.026120248	0.34797096
pcaec341.c102	0.025955268	0.027560511	0.347975405
lysine.lysopcac181	0.039316307	0.04175051	0.348005633
smc260.glutamine	-0.028542696	0.030321908	0.348196452
c181.pcaec425	0.006955089	0.007389134	0.348228875
pcaac405.pcaec421	0.001098093	0.001166733	0.348274825
ornithine.pcaec402	-0.032659834	0.034703418	0.348303497
smc202.smohc141	-0.03273324	0.034791746	0.348446144
pcaec406.pcaac385	-0.058391749	0.062064209	0.348448937
pcaac281.lysopcac181	0.036979861	0.039306358	0.348457556

lysopcac281.pcaac380	0.02556705	0.027176774	0.348479397
pcaac344.smc161	-0.050077864	0.05323838	0.348547723
pcaac361.smc241	-0.053492095	0.056875093	0.348606876
pcaac421.c182	-0.036635389	0.038962756	0.348735595
c51dc.alanine	0.013749231	0.014623419	0.348759567
asparagine.smohc221	-0.060755588	0.064644164	0.348950455
leucine.phenylalanine	0.103726999	0.110367792	0.348958697
leucine.c162	0.100494562	0.106928641	0.348959739
pcaec445.c16	0.053295163	0.056709607	0.348978935
lysopcac204.pcaec342	0.07523583	0.080056877	0.348984569
asparticacid.ornithine	-0.038833728	0.041322946	0.348993708
pcaec380.pcaac361	0.041279379	0.043933893	0.349087013
smc240.pcaec364	-0.021764474	0.023165405	0.349114862
pcaac386.pcaec425	0.00432167	0.004599969	0.349127627
isoleucine.c141oh	-0.147307198	0.156802286	0.349155464
pcaac383.pcaac426	0.052225192	0.055591656	0.349156724
asymmetricdimethylarginine.leuc	0.007101696	0.007559501	0.349158406
pcaec421.serotonin	-0.12193196	0.129797791	0.349179048
pcaac405.pcaac420	-0.053139318	0.05658674	0.349343808
pcaac341.pcaac405	-0.048406316	0.05154838	0.349359616
lysopcac181.isoleucine	0.054259051	0.057785488	0.349396783
alanine.pcaac321	-0.039180608	0.041727466	0.349401482
pcaec381.pcaec401	0.063956791	0.068119235	0.349437164
pcaec406.pcaac386	0.027728551	0.029534265	0.349454733
pcaec384.c14	-0.043322343	0.046144827	0.349468114
c10.pcaec362	0.02166865	0.023081281	0.349486886
c101.pcaec362	-0.011195569	0.011925655	0.349495749
pcaac240.lysopcac180	-0.069777349	0.074342028	0.349588509
pcaac365.pcaec422	-0.031155723	0.033194355	0.349595662
smc261.pcaac361	0.062673544	0.066780753	0.349640631
asparagine.lysopcac170	-0.018032406	0.019219267	0.349769026
pcaac363.pcaac360	0.025265027	0.026928091	0.349771952
c101.pcaec363	-0.014175804	0.015109271	0.349783023
smohc241.serine	-0.020449822	0.021796484	0.349784234
smc260.pcaec364	0.017434091	0.018583776	0.349826006
pcaac406.pcaec425	0.005086543	0.005423125	0.349928066
pcaec444.pcaac381	0.017414809	0.0185674	0.349934068
pcaec443.c181	0.028101462	0.029965268	0.349996955
smc260.pcaac384	0.100775997	0.107475385	0.350066253
smohc141.pcaec425	0.016215605	0.017293697	0.35006934
c8.c101	0.02527081	0.026951804	0.350084807
pcaec421.methionine	-0.048910547	0.052165499	0.350098251
pcaec365.c141oh	-0.101856507	0.108638727	0.35011489

pcaac425.pcaac322	0.077830561	0.083029883	0.350212622
kynurenine.smc260	0.023661106	0.025243383	0.35024387
pcaec342.putrescine	-0.031723172	0.033845665	0.350259268
pcaec342.pcaac383	0.016521741	0.017628254	0.350289173
pcaec405.smc180	-0.006490241	0.006925014	0.350296132
glutamicacid.pcaec401	0.078694617	0.0839694	0.350314094
pcaec443.glutamicacid	0.082948866	0.088510195	0.350321632
lysopcac170.serotonin	-0.112173096	0.119698245	0.350339583
smohc222.smc161	0.131225526	0.140123248	0.350663347
pcaec300.c101	-0.009531476	0.01017832	0.350689967
lysopcac170.pcaec423	0.018390137	0.019641793	0.350778664
pcaac300.alanine	-0.02633832	0.028131399	0.350786519
alphaaminoadipicacid.pcaac383	-0.012034447	0.012854492	0.350814749
pcaac401.methionine	-0.060026589	0.06412064	0.350842817
pcaac426.transhydroxyproline	0.027596181	0.029491272	0.351053257
c101.methioninesulfoxide	0.026570511	0.028397678	0.351095726
pcaac344.pcaec425	0.009905034	0.010587131	0.351139302
asymmetricdimethylarginine.pca	0.006760996	0.007229143	0.351309317
pcaec404.lysopcac181	-0.068950085	0.073731999	0.351359153
glycine.smohc141	0.019976174	0.021361814	0.351364266
pcaec406.pcaac320	0.11159797	0.119369005	0.351485265
pcaec386.pcaac366	-0.042605335	0.045573392	0.351498642
pcaac281.lysopcac160	-0.014038158	0.015016434	0.351508934
pcaac342.c18	0.035025319	0.037468442	0.351538616
leucine.c12	-0.036342012	0.038879051	0.351564202
spermidine.c14	0.19529407	0.208942794	0.351599201
pcaec420.c141oh	-0.101498863	0.108606108	0.351659701
lysopcac182.glycine	-0.022934847	0.024544649	0.351734769
pcaec425.pcaec404	-0.021925922	0.023466013	0.351757377
pcaac362.serine	-0.013365189	0.014304617	0.351779133
pcaec381.pcaec421	-0.010547557	0.011290204	0.351833096
smohc141.pcaec424	0.013995222	0.014981246	0.351853217
lysopcac281.glycine	-0.054314822	0.058144594	0.351878476
c18.c16	-0.062024637	0.06640335	0.35191691
pcaec364.smc161	-0.051344229	0.05497076	0.351932775
smohc222.glutamine	-0.013987578	0.014976015	0.351947865
smc261.smc241	-0.118735337	0.127131531	0.351969447
c18.pcaac381	-0.011064192	0.011847295	0.351998418
pcaac343.pcaac321	-0.034870952	0.037345168	0.352077031
alanine.pcaec424	0.018305295	0.019604897	0.352096075
lysopcac160.pcaec406	0.016660236	0.017851281	0.352317566
pcaac422.c2	0.071920931	0.077063102	0.352320821
pcaec342.c51	0.060174213	0.064477373	0.352327157

smc160.pcaac363	-0.006801537	0.00728834	0.352354384
pcaac343.lysopcac204	-0.02104059	0.02254708	0.352366371
pcaec420.pcaec340	-0.044557863	0.047753987	0.352424816
pcaac344.pcaac426	0.090612775	0.09711417	0.352433541
lysopcac181.pcaac422	-0.044837145	0.048063479	0.352526462
pcaac403.smc181	-0.004842707	0.005191815	0.352585878
pcaac364.c51	0.034889749	0.037406755	0.352609286
lysopcac203.pcaec341	0.052824311	0.056636365	0.352619604
lysopcac182.c102	-0.038845582	0.041651245	0.352647051
arginine.pcaec342	0.088682198	0.095092436	0.352672646
lysopcac203.pcaac403	-0.007589327	0.008138832	0.352727097
pcaec300.pcaac425	0.101661842	0.109025142	0.35273803
pcaec341.c182	-0.039682516	0.042559477	0.352769413
pcaec364.h1	0.002363793	0.002535182	0.352772378
asymmetricdimethylarginine.lysi	0.088284524	0.094697718	0.352833341
c51.pcaec403	-0.04510233	0.048380449	0.352851004
lysopcac161.kynurenine	-0.008876637	0.009521905	0.352855942
asparticacid.pcaac364	-0.069446265	0.074501694	0.352902211
smc202.c14	-0.01859344	0.019947729	0.352920393
pcaac401.smohc241	0.066199106	0.071023457	0.352938048
smc261.c51	0.044517959	0.047772544	0.353041314
pcaec422.smc180	-0.005664951	0.006079609	0.353081519
methionine.c10	-0.006302171	0.006763727	0.353099617
c141.lysopcac181	0.040102024	0.043042131	0.353134508
pcaec341.smc180	-0.019896878	0.021358676	0.353202927
smc241.pcaec445	-0.059951318	0.064360008	0.353233831
pcaac406.c10	-0.003268028	0.003509141	0.353341761
smohc141.lysopcac182	-0.02747433	0.029502657	0.353362729
smc260.pcaac323	0.074837245	0.080363101	0.353368132
pcaec443.smc180	-0.005834901	0.00626731	0.353488299
c5.pcaec421	0.001831587	0.001967413	0.353510869
smohc221.pcaac240	-0.009570136	0.010279903	0.353513987
pcaac360.c10	-0.003277467	0.003520569	0.353518028
pcaac366.pcaec424	0.007913423	0.008500551	0.353526984
pcaac363.pcaac364	-0.094938327	0.101982425	0.353528191
pcaac386.c162	-0.054510409	0.058555547	0.353533546
pcaec425.c51	0.091231351	0.098003848	0.353545052
spermidine.c10	-0.025465109	0.027357376	0.353578064
methionine.c2	0.063364216	0.068085734	0.353669942
pcaac300.pcaec380	-0.053257248	0.057232252	0.353725252
lysine.pcaec363	0.031674432	0.034043329	0.353792721
smc181.pcaec423	-0.012809186	0.013769143	0.353861442
methionine.smc180	0.007497801	0.008059808	0.353867432

h1.pcaec364	0.01949299	0.020954657	0.353879908
pcaac341.c2	-0.072968895	0.07845097	0.353944471
lysine.pcaac240	-0.013994683	0.01504697	0.3539726
histidine.tyrosine	-0.070343546	0.075638025	0.354005648
serine.pcaac365	-0.04635191	0.049846647	0.354063525
pcaec342.valine	-0.046368796	0.04986644	0.35407925
smc260.creatinine	0.010061203	0.01082098	0.354116936
pcaec341.spermidine	-0.065314335	0.070254093	0.354168262
pcaec363.c3	-0.043548706	0.046847264	0.354218918
pcaac425.pcaac321	0.016632772	0.017893704	0.354248339
pcaac426.pcaec361	0.019091702	0.020539164	0.354251108
pcaac386.acetylornithoine	-0.03097003	0.033318805	0.354261798
pcaec425.lysopcac160	-0.027951977	0.03007192	0.354262722
smc161.glycine	-0.054769354	0.05892351	0.354265329
pcaec360.c14	0.080013083	0.086088249	0.354300543
c5.c101	0.023341569	0.025116897	0.35435916
methioninesulfoxide.pcaec423	0.03917821	0.042166	0.354449612
acetylornithoine.pcaac363	0.009839208	0.010590499	0.354492113
pcaac406.pcaec422	-0.020628202	0.022206213	0.354554875
c18.pcaac426	0.053356031	0.057456758	0.354714467
pcaec364.smc202	0.051570737	0.055544481	0.354802798
lysopcac160.pcaac380	0.101882742	0.109737568	0.354821715
alphaaminoadipicacid.putrescine	-0.0248788	0.026797113	0.354826002
c10.pcaac405	-0.059885986	0.064516597	0.354922823
lysopcac181.smc180	-0.014298976	0.015405251	0.354942261
pcaec342.smohc241	-0.036463957	0.039289496	0.354996168
pcaec406.smohc141	-0.018882892	0.020346417	0.355003648
smc261.lysine	0.077396024	0.08340787	0.355079795
phenylalanine.c51dc	0.122533833	0.132065763	0.355130431
smc261.pcaec423	0.033265325	0.035853258	0.355133326
lysopcac181.smc261	0.049333138	0.053176287	0.355180178
pcaec301.pcaec404	0.015917849	0.017158113	0.355186634
asparagine.c0	0.005660143	0.006103704	0.355386468
c3.c8	-0.033957327	0.036625233	0.355475725
pcaac401.pcaac385	-0.039173351	0.042253434	0.35550265
pcaec363.pcaec406	0.005107113	0.005509086	0.355538804
asymmetricdimethylarginine.tyrc	-0.049802442	0.053723166	0.355546465
lysopcac161.ornithine	0.053301016	0.057514468	0.355690741
pcaec321.pcaec425	0.028698791	0.030967538	0.355692349
smc180.pcaac384	0.067211166	0.072530967	0.355735369
pcaac364.pcaec404	-0.025787386	0.027828537	0.355736493
pcaac360.c51	0.050347784	0.054334318	0.355748424
lysopcac281.pcaec424	0.01286375	0.013882352	0.355750183

pcaac321.tyrosine	0.076801255	0.082883309	0.355753842
lysopcac204.glycine	0.045534582	0.049143573	0.355783217
methioninesulfoxide.spermidine	0.062087457	0.067018995	0.355859035
pcaec421.c8	-0.056789075	0.061299941	0.355860389
pcaac341.pcaac320	0.160449271	0.173196699	0.355867678
c14.glutamicacid	-0.044377061	0.047908341	0.355923693
histidine.lysopcac170	-0.018304804	0.019763488	0.355974396
pcaac361.pcaec404	-0.032338768	0.034916805	0.355988213
pcaec381.pcaac363	-0.019866634	0.021454657	0.356083431
pcaec425.methionine	-0.034309957	0.037055129	0.356117521
glycine.lysopcac180	0.05341553	0.05769039	0.356126111
pcaac344.pcaec421	0.003206554	0.003463447	0.356163623
alphaaminoadipicacid.pcaac402	-0.05991613	0.064738546	0.356328432
isoleucine.c12	-0.036719395	0.039675357	0.356335213
pcaec446.smc181	0.026444667	0.028578302	0.35641579
pcaac240.methionine	-0.050357611	0.054424102	0.35644653
pcaec385.pcaec443	-0.04540434	0.04908094	0.356545163
smc161.leucine	-0.009833796	0.010632011	0.356632072
pcaac343.c16	0.035708555	0.03860741	0.356636664
smc261.pcaac384	0.127322173	0.137677906	0.356704902
c14.pcaac403	0.005044367	0.00545605	0.356827984
pcaec382.smc181	-0.003979808	0.004304615	0.356828494
pcaec340.pcaec360	-0.008936944	0.009667015	0.356862893
pcaac420.lysopcac160	-0.022813742	0.024677542	0.356865146
pcaec342.c102	0.027456796	0.029700241	0.356870384
c18.isoleucine	0.016612749	0.017970511	0.356880093
pcaac426.smc180	-0.012277049	0.013281087	0.35690296
lysopcac160.smohc241	0.087521945	0.094686034	0.356935338
pcaac404.pcaac383	0.005363232	0.005802602	0.356965362
smohc161.lysopcac181	0.051935771	0.056190953	0.356969384
smc181.pcaec403	0.03608881	0.039048535	0.357005109
lysopcac181.pcaec425	0.006442225	0.006971487	0.357068392
lysopcac180.serine	-0.050113496	0.054249206	0.357233037
pcaac426.c18	0.049133806	0.053190211	0.357246962
phenylalanine.serine	-0.016215977	0.0175552	0.357259505
isoleucine.pcaec301	0.045241143	0.048981167	0.357295813
c102.lysopcac160	0.016973969	0.018377697	0.357309171
c14.lysopcac203	-0.014856521	0.016093265	0.357551279
methionine.tyrosine	-0.063783872	0.069111464	0.35767507
putrescine.lysopcac160	0.018846683	0.020424198	0.357753303
smc202.pcaac403	-0.008666492	0.009392199	0.357768607
smohc221.smc180	-0.014538128	0.015756448	0.357797162
pcaec385.pcaac383	0.005202304	0.005639062	0.357864756

smohc141.smohc161	0.042735229	0.046325251	0.357887527
c16.pcaec380	-0.043023737	0.046642804	0.357936939
c3.phenylalanine	-0.029668184	0.032168847	0.358011985
pcaac322.pcaec424	0.007052093	0.007646825	0.358032525
pcaec342.phenylalanine	0.044984735	0.048778896	0.358036553
pcaac425.valine	-0.048185581	0.052250946	0.358047872
prolinr.serine	-0.008489959	0.009206704	0.358071611
acetylornithoine.pcaec423	-0.044274308	0.048013002	0.358081006
smc260.c14	0.081303944	0.088194511	0.358216568
lysine.serotonin	0.113756066	0.123412024	0.35827501
pcaac401.lysopcac180	-0.067050252	0.072743484	0.358286897
pcaac342.pcaec301	0.070235858	0.076200861	0.358294952
c10.valine	0.079022216	0.085736778	0.358313678
lysopcac140.smc180	-0.024493683	0.026583123	0.35846147
c51.pcaac384	-0.076996882	0.083578707	0.358539357
pcaec364.pcaac380	0.065613729	0.071226284	0.358564797
isoleucine.pcaac300	-0.038307905	0.04159663	0.358701797
pcaec404.creatinine	-0.012543094	0.013620105	0.35870844
pcaac381.lysopcac160	0.038681226	0.042008252	0.358773097
lysopcac182.pcaac341	0.004200408	0.004562277	0.358834713
c51.smc261	0.029272364	0.031798418	0.358898127
pcaec360.leucine	-0.007772994	0.008444942	0.358964946
putrescine.pcaec341	0.044643466	0.048511138	0.359047975
c162.pcaec322	0.066721839	0.07251059	0.359102986
kynurenine.lysine	-0.108002497	0.11737681	0.35911961
pcaac360.pcaec363	-0.03845224	0.041792247	0.359147822
lysopcac180.arginine	0.052039463	0.056563885	0.359183496
leucine.pcaec364	-0.015310105	0.01664234	0.359216352
pcaac380.pcaac364	-0.049523082	0.05383469	0.359236578
pcaac401.kynurenine	0.013074879	0.014213399	0.359242888
pcaec386.pcaec363	-0.040169707	0.043679764	0.359376739
pcaac240.creatinine	0.006676397	0.00726106	0.359460833
c12.h1	-0.002773214	0.003016795	0.359576118
pcaec341.pcaac300	0.077978563	0.084835206	0.359618578
pcaec386.spermidine	-0.064114394	0.069764465	0.359704414
prolinr.pcaec364	0.017923791	0.019504297	0.35972835
asparticacid.pcaac323	0.119976057	0.130555738	0.359729403
pcaec342.creatinine	-0.00595451	0.006479635	0.359732877
pcaec341.pcaac422	-0.018620169	0.020263756	0.359767955
pcaac281.smohc141	0.033888733	0.036891616	0.35991795
smc261.kynurenine	-0.003930414	0.00427886	0.359937187
c181.pcaec363	-0.018557216	0.020202725	0.359945281
pcaac386.c3	0.027721938	0.030182921	0.359989992

pcaac365.pcaec425	0.004213954	0.004588324	0.3600193
serine.lysopcac160	-0.014978671	0.016310382	0.360048623
glutamicacid.pcaac320	0.113834869	0.123958189	0.360058613
pcaec361.pcaac240	-0.014628005	0.015929472	0.360076674
c162.pcaec425	0.005658962	0.006162969	0.360117397
alphaaminoadipicacid.pcaac320	0.039099646	0.042583027	0.360128952
smc240.lysopcac170	-0.013521034	0.014726095	0.360144382
c102.pcaec406	-0.005858498	0.006382047	0.360250262
pcaac342.pcaec425	0.006458404	0.007035743	0.360262457
pcaec383.pcaec421	-0.01408891	0.01535133	0.360354876
prolinr.smc181	-0.009728744	0.010601864	0.360417608
pcaec402.c181	-0.021180633	0.023082398	0.360435803
pcaec446.pcaac380	-0.087307312	0.095180012	0.360604662
putrescine.c141oh	0.112252702	0.122399659	0.360701975
c141.arginine	0.039904838	0.043512758	0.360710422
serine.smohc141	-0.073288364	0.079914691	0.360711009
pcaac386.creatinine	0.023356995	0.025473865	0.360806126
spermidine.lysopcac180	0.12518007	0.136538679	0.360853124
pcaec381.c51dc	0.131993059	0.143985922	0.360906515
pcaec300.phenylalanine	0.031359271	0.034216363	0.361015605
smc240.pcaec342	0.099023574	0.108055833	0.361061602
pcaac406.pcaec301	0.073102128	0.079773733	0.361083953
pcaec380.c141oh	-0.08607079	0.09392884	0.361098586
c18.serotonin	-0.092216476	0.100646014	0.361148067
pcaac385.asparticacid	0.034425136	0.037573113	0.3611629
smc181.c102	0.025162254	0.027471231	0.36130296
pcaec301.c14	0.040101477	0.043782487	0.361315622
c162.c14	-0.033922818	0.037038324	0.361336942
glutamicacid.smc181	-0.022080857	0.024109849	0.361358065
lysopcac160.pcaec361	0.005885939	0.006427093	0.361380426
pcaac383.pcaec386	0.010715496	0.011702316	0.361447287
pcaac420.pcaac380	-0.113471375	0.123931453	0.361486643
pcaac385.leucine	-0.007942359	0.008674728	0.361498906
alanine.c10	-0.005070802	0.005538603	0.361517934
pcaac402.pcaac361	0.069513567	0.075933598	0.361562895
pcaac402.pcaac421	-0.065554814	0.071614391	0.361597401
pcaac424.pcaac403	0.019616065	0.021430266	0.361619386
pcaac300.c10	-0.003129855	0.003419656	0.361666186
pcaac366.pcaac405	-0.062098167	0.06784993	0.361679959
pcaac424.glutamicacid	0.088158903	0.096337793	0.361745966
serotonin.pcaac380	-0.06407201	0.070018519	0.361761462
pcaac404.pcaac344	-0.077644475	0.084853587	0.361778073
glycine.serine	-0.027644249	0.030212743	0.361806423

lysopcac204.phenylalanine	0.055955518	0.061162149	0.361866429
pcaec423.smc161	0.096244643	0.105200408	0.361867575
pcaec340.pcaac361	-0.042515656	0.046472488	0.361874389
pcaac426.pcaec425	0.005431471	0.005937829	0.361944045
c182.pcaec363	-0.012666289	0.013851218	0.362085435
pcaac401.serotonin	-0.116903284	0.127845946	0.362109346
pcaec342.spermidine	-0.065241946	0.071356264	0.362158873
smc202.pcaec425	0.004392305	0.004804059	0.362170581
alphaaminoadipicacid.pcaac344	-0.047183227	0.051612516	0.362227407
pcaac405.smohc241	0.042612515	0.046614297	0.362243482
c142.c51dc	-0.119348252	0.130562532	0.362266101
asparticacid.c16	0.047214161	0.051663536	0.362386599
smc181.serine	-0.054372956	0.059500701	0.362416663
pcaec385.pcaec406	0.034604127	0.037869951	0.362447184
pcaec386.c10	-0.002845752	0.003114714	0.362506989
pcaec341.pcaec425	0.004057319	0.004440929	0.362521924
c12.pcaac386	0.017514194	0.019172681	0.362585857
pcaac402.smc181	-0.004443541	0.00486457	0.362610677
pcaec365.pcaec421	0.000888069	0.000972305	0.362655505
pcaac425.smc180	-0.006908321	0.007563824	0.362669796
c4.c2	0.062368193	0.068290252	0.362699173
smc180.pcaec421	0.001628351	0.001782999	0.362707425
pcaac322.methionine	-0.025417881	0.027832855	0.36272435
methioninesulfoxide.smc181	-0.004152185	0.004547064	0.362764001
pcaec423.c16	0.025799388	0.028254478	0.362789886
pcaac321.h1	0.002105562	0.002305956	0.362795552
pcaac343.lysopcac180	-0.067558277	0.073988938	0.362801275
methioninesulfoxide.pcaac363	-0.021399791	0.023438006	0.362826513
isoleucine.phenylalanine	0.079560422	0.087143618	0.362856668
acetylornithoine.pcaac420	0.085434598	0.093586184	0.362900128
c12.c0	-0.001306641	0.001431388	0.362925326
pcaac362.pcaec425	0.006386883	0.006997234	0.362965637
pcaac384.smohc221	0.032107771	0.035176155	0.362966493
pcaec406.pcaec425	0.004003184	0.004386566	0.363055596
smc160.pcaec341	0.089658761	0.098261428	0.36313409
c141.pcaec425	0.0039269	0.004303871	0.363155047
pcaec423.smc180	-0.006255872	0.006856454	0.363157657
smohc161.pcaec406	0.00787867	0.008636585	0.36324292
glutamine.pcaac343	-0.020490897	0.022463666	0.363276566
pcaec381.pcaac365	-0.06027564	0.066081732	0.363298492
pcaec420.pcaec425	0.002524604	0.00276784	0.363307438
smc180.pcaac426	0.034085514	0.0373776	0.363410832
pcaac401.pcaac420	-0.16095536	0.176557741	0.363564758

glycine.pcaec425	0.005426233	0.005952366	0.363575589
pcaec343.pcaec364	-0.016475944	0.018076335	0.363651501
pcaec424.c102	-0.022693295	0.024903	0.363755058
pcaac403.c181	-0.016646267	0.018268555	0.363791657
pcaac405.pcaac403	-0.006186034	0.006789108	0.363806101
smohc141.pcaec363	-0.075115827	0.082451769	0.36388107
serine.pcaac422	-0.06832723	0.075007827	0.363929797
c181.serotonin	-0.088057491	0.09666794	0.363933522
c142.pcaac381	-0.015003747	0.016471686	0.363957916
pcaec361.glutamicacid	0.053655444	0.058906986	0.363974114
c14.c10	-0.005079836	0.005577043	0.363975501
pcaec301.c0	0.00082131	0.000901713	0.363983007
acetylornithoine.pcaec404	0.025171829	0.027636273	0.363986982
c10.pcaec382	-0.013387058	0.014700073	0.36406369
putrescine.serine	-0.016141611	0.017727289	0.364130952
pcaac323.lysopcac203	0.029711315	0.032633208	0.364177713
pcaac322.pcaec425	0.007273061	0.007988481	0.364187692
c16.serine	0.026962184	0.029619893	0.364277383
pcaac360.pcaec362	0.027895265	0.030649064	0.364341592
c12.pcaac323	-0.069720071	0.076610224	0.364388044
lysine.pcaec443	0.053824039	0.05914506	0.36440267
pcaec360.alanine	-0.012911244	0.014189654	0.364470399
smc202.lysopcac180	-0.041946561	0.046102066	0.364492773
pcaec443.pcaec403	0.07236863	0.079540709	0.364509376
smc161.pcaac406	-0.02224797	0.024453499	0.364522036
smc181.isoleucine	0.020182944	0.022184861	0.364545803
lysopcac182.arginine	-0.062874791	0.069140518	0.364748255
asymmetricdimethylarginine.smc	-0.073526558	0.080858237	0.364774628
c10.pcaac425	-0.078918363	0.086800565	0.364845588
lysopcac203.pcaec406	0.021996869	0.024196565	0.364898834
pcaec420.lysopcac280	0.007445823	0.008191287	0.364950094
pcaec420.smohc141	-0.028404454	0.031249462	0.364968457
pcaac320.smohc141	-0.03537248	0.038918904	0.365011398
lysopcac182.pcaec406	0.003560749	0.003918173	0.36506323
pcaec381.pcaac406	-0.075548189	0.083137807	0.365098813
methioninesulfoxide.transhydro:	-0.058170442	0.064016476	0.365115199
c16.c141oh	-0.066295903	0.072961588	0.365135228
pcaac341.pcaac385	-0.0305151	0.033586938	0.365188099
pcaec424.transhydroxyproline	0.047626515	0.05242156	0.365194166
pcaac365.lysopcac182	-0.013050498	0.014364906	0.365210197
putrescine.pcaac321	-0.014209604	0.015641952	0.365246832
pcaac384.tyrosine	-0.064610474	0.071125682	0.365262833
pcaec404.pcaec403	0.079369197	0.087373967	0.365270046

serine.c16	0.033053427	0.036390358	0.365313825
h1.pcaac406	0.10531927	0.115977265	0.365418593
c162.glutamicacid	-0.054101642	0.059577746	0.365428054
lysopcac181.c51dc	0.130794471	0.144039418	0.365448257
pcaac240.glutamicacid	0.065481445	0.072113949	0.365458224
pcaec405.leucine	-0.051127869	0.056307278	0.365464626
pcaac426.isoleucine	0.037495226	0.041300187	0.365540789
pcaac322.pcaec384	0.056680354	0.06243482	0.365560899
pcaec424.pcaec322	0.049259187	0.054263397	0.365588876
lysopcac182.pcaec425	0.007345072	0.008091803	0.365621312
c101.lysopcac280	0.02376475	0.026184261	0.365684997
pcaec386.pcaac385	-0.027388481	0.030187969	0.36585985
c141.pcaac386	0.015810098	0.017427088	0.365886584
c2.acetylornithoine	-0.040092512	0.044194767	0.365905672
pcaec402.smc241	-0.075185939	0.082885575	0.365943903
pcaac322.pcaac422	-0.026868202	0.029621002	0.365964615
c3.pcaec341	-0.045193244	0.049823931	0.365968278
pcaec402.pcaac365	-0.058666658	0.064684623	0.36601805
pcaac425.c141	-0.042285025	0.046628223	0.366075899
pcaac344.pcaac240	-0.03416698	0.03768777	0.366220679
c12.pcaac380	-0.044843817	0.049469893	0.366269706
smohc141.pcaac365	-0.053270013	0.058770979	0.366315605
smc261.serine	-0.023634551	0.026077086	0.366350344
serine.pcaac240	-0.01177539	0.012992526	0.366357626
pcaac422.c14	-0.046946261	0.051805427	0.366419173
asparticacid.pcaec406	-0.007632725	0.0084238	0.36647879
prolinr.pcaac381	0.019688748	0.021732052	0.366538418
c181.smc261	0.073835785	0.081503549	0.366568128
smohc222.smc181	-0.057158431	0.063096527	0.366585211
lysopcac281.pcaec425	0.003289333	0.003631229	0.366607897
lysopcac161.pcaac360	0.032577912	0.035966714	0.366642826
pcaec342.lysopcac181	0.035112631	0.038767146	0.366668056
lysopcac180.spermidine	0.065860744	0.072727629	0.366747643
asymmetricdimethylarginine.pca	-0.003580304	0.003954201	0.366820355
pcaac402.serine	0.115038524	0.127067006	0.366876025
c8.smohc241	-0.086667498	0.095740455	0.366930732
c0.smc202	-0.041051572	0.045352173	0.366962724
pcaec342.leucine	-0.008878709	0.009809344	0.366986736
smohc241.pcaac403	-0.019685006	0.021748515	0.36699104
c141oh.glutamicacid	-0.047098362	0.052037459	0.367008886
pcaac240.smohc221	0.12647376	0.139744577	0.367035574
smc160.lysopcac182	-0.041710714	0.046101397	0.367180693
pcaec380.pcaec343	0.071837091	0.079404313	0.367212478

c16.lysopcac181	-0.08801235	0.097290985	0.367249464
pcaac380.tyrosine	-0.056287192	0.062225472	0.367282064
pcaac323.pcaec360	-0.006009577	0.006643617	0.367284339
pcaac386.pcaac381	-0.026117213	0.028872718	0.367284497
pcaec444.leucine	-0.006140774	0.006790235	0.367395437
pcaac385.pcaac380	0.046575305	0.051502983	0.367411912
c4.pcaec340	-0.045176273	0.049964635	0.367495103
pcaac365.arginine	0.046839742	0.051810097	0.367547446
pcaec301.pcaac361	-0.057046419	0.063110191	0.367625754
c142.lysopcac180	-0.034458644	0.038122091	0.367633917
c101.pcaac425	-0.079055175	0.087460138	0.367635368
c3.pcaac384	-0.036583602	0.040473712	0.367642765
smohc221.pcaac402	-0.042307054	0.046816722	0.367754541
pcaec445.pcaac424	0.015071862	0.016679626	0.367788853
pcaec386.c101	-0.012689467	0.014044116	0.367823635
pcaec382.c141	0.043670809	0.048337417	0.367868888
c182.lysine	0.072453265	0.080217423	0.36799925
pcaec421.pcaec360	-0.006103736	0.006759049	0.368086199
pcaec361.serotonin	-0.114497813	0.126811677	0.368165628
histidine.smohc222	0.006440212	0.007133477	0.368208573
pcaac323.pcaac300	0.040606743	0.044980045	0.368231238
lysopcac281.lysine	-0.067042499	0.074284746	0.36837167
pcaac381.c141	0.051386535	0.056938298	0.368377938
alanine.lysopcac181	0.027464778	0.030437527	0.368463818
pcaec384.serotonin	-0.109124723	0.120955996	0.36854186
smc241.lysopcac181	0.056868432	0.063042071	0.368602288
pcaec342.pcaec406	0.005678681	0.006295194	0.368604957
lysopcac280.pcaac365	-0.067423593	0.074752595	0.368662934
pcaac380.pcaac363	-0.020667602	0.022915445	0.368689139
pcaac425.pcaec405	-0.064991177	0.072072189	0.368771692
c142.pcaec425	0.014560341	0.016148881	0.368835004
c3.c2	0.046352183	0.051413192	0.368871794
pcaac386.c10	-0.002820348	0.003128489	0.368902053
pcaec384.lysopcac170	-0.010008589	0.011103315	0.368954742
methioninesulfoxide.pcaec401	0.043436661	0.048204429	0.369120364
pcaac344.asymmetricdimethylar[illegible]	0.014511864	0.016106635	0.369176533
pcaac341.c16	0.037291186	0.041390819	0.369194336
asparticacid.lysopcac170	-0.02879269	0.031973724	0.369428624
pcaec342.pcaac385	-0.021706379	0.024105579	0.36944972
c5.pcaec423	0.030389125	0.033749703	0.369473438
pcaec301.c142	0.02931616	0.032558698	0.369482431
pcaec365.putrescine	-0.072449949	0.080464921	0.369491838
c51.h1	0.003038463	0.00337495	0.369541132

histidine.pcaec362	-0.013903042	0.015444373	0.369592876
c51dc.pcaac381	-0.024982161	0.027755723	0.369661131
pcaec364.smc181	0.02377105	0.026411828	0.369691394
pcaec322.lysopcac281	0.044689595	0.04967444	0.369885297
pcaec364.c182	-0.030541475	0.033955732	0.369991365
pcaac341.leucine	-0.006431553	0.007151312	0.370042816
pcaac363.asparticacid	0.026866798	0.029874589	0.37006052
putrescine.pcaec425	0.00169031	0.001879728	0.370107216
pcaec422.lysopcac181	-0.075777029	0.084273786	0.370136223
c5.pcaac386	-0.052805984	0.058728389	0.370147237
putrescine.pcaac385	0.031051988	0.03454958	0.370354233
c4.pcaec421	0.000860334	0.000957256	0.370362353
pcaec340.pcaec425	0.003801224	0.004229635	0.370382873
pcaec405.pcaac342	0.058414402	0.064998037	0.370383833
pcaac300.pcaac380	0.045849758	0.051018036	0.370390845
asparticacid.c10	0.01376746	0.015323786	0.370528792
lysopcac280.pcaec425	0.005775089	0.006428483	0.370570045
alanine.smc180	-0.046639538	0.051918709	0.370591833
pcaec365.lysopcac160	-0.016478542	0.018344003	0.370598072
leucine.histidine	0.086913828	0.096756317	0.370614682
serotonin.pcaec342	-0.081894074	0.091171709	0.370633544
pcaec340.spermidine	-0.065213476	0.07261636	0.370731865
pcaec420.pcaac360	-0.035324149	0.039339585	0.370798805
isoleucine.lysopcac160	0.017229472	0.019190074	0.370850006
asparagine.smc160	0.07569664	0.084322277	0.370917048
c102.pcaac281	0.002218872	0.002471929	0.370958805
pcaec381.putrescine	-0.072134394	0.08036531	0.370983513
pcaac403.pcaec362	0.011035922	0.012296482	0.37103403
pcaac402.pcaac323	-0.118679966	0.132245377	0.371067916
pcaac381.pcaec406	-0.017108214	0.019064657	0.371091238
c3.c141oh	-0.115737431	0.128975731	0.371102045
pcaac322.pcaac383	-0.006807669	0.007586352	0.371102482
prolinr.c0	-0.001076975	0.00120024	0.371133481
c181.pcaac344	0.033944577	0.037834432	0.371192834
spermidine.c142	-0.146814196	0.163647425	0.371219521
arginine.lysopcac170	0.018010763	0.020077338	0.371255643
smc160.alanine	-0.01810317	0.020180471	0.371258556
pcaac343.creatinine	-0.00601842	0.006709354	0.371282194
pcaac363.pcaac321	-0.022961911	0.025598394	0.371289332
methioninesulfoxide.pcaac321	0.018927387	0.021101777	0.37131532
pcaec446.pcaec402	-0.029150155	0.032503881	0.371387783
pcaac386.pcaac403	0.021114091	0.023543272	0.371387879
kynurenine.pcaec340	-0.033653042	0.037526133	0.371404413

smc180.smc260	0.039532323	0.044085286	0.37143938
ornithine.c12	0.054914699	0.061246606	0.37149658
pcaec380.pcaac281	-0.003425802	0.003821844	0.371625346
c8.valine	0.072627535	0.081027153	0.37164576
lysopcac204.pcaec383	0.098136631	0.10950964	0.371746703
pcaac343.pcaec322	0.05564312	0.062100224	0.371813112
pcaec425.pcaac426	-0.081879062	0.091397931	0.371902935
c142.prolinr	0.04733063	0.052834452	0.37191547
c14.pcaec420	-0.022321053	0.02492043	0.371987847
citruline.pcaac361	0.042103875	0.047011038	0.372028397
smc161.lysopcac160	-0.029167464	0.032569178	0.372061713
pcaac385.lysopcac182	-0.012469824	0.013924695	0.372080627
pcaec364.pcaec420	-0.01850636	0.020665543	0.372081151
pcaec360.pcaec425	0.002635142	0.002942711	0.372100655
pcaac366.pcaac402	-0.054360683	0.060716579	0.37218715
pcaec406.pcaec404	-0.009745999	0.010885515	0.372187434
lysopcac204.c0	0.000636874	0.000711381	0.372216094
spermidine.methionine	0.009436423	0.010541509	0.372267283
pcaac424.pcaac321	0.014731021	0.016457456	0.372305139
pcaec423.pcaec403	0.044296723	0.049493969	0.372360926
c14.pcaac281	-0.001033134	0.001154415	0.37238811
pcaac386.asparagine	-0.011855573	0.013247539	0.372396024
pcaec425.smohc141	-0.017280191	0.019309155	0.372398293
c2.transhydroxyproline	0.038517855	0.043043528	0.37243236
pcaec343.pcaec406	0.004492869	0.005021488	0.372501474
pcaac300.c12	0.047317894	0.052886537	0.372513543
lysopcac182.c0	0.000683663	0.000764304	0.372628567
pcaec322.c142	0.051377881	0.057446393	0.372696675
c182.serine	0.02529115	0.028279889	0.372721433
smohc161.pcaac405	-0.061649124	0.068939387	0.372755869
methioninesulfoxide.lysopcac20	0.007360405	0.008231023	0.37276862
pcaec341.pcaac363	-0.010037069	0.01122468	0.372785009
pcaac405.pcaec404	-0.011142884	0.012462321	0.372822626
glutamine.pcaec445	-0.060685491	0.0678729	0.372833818
c51dc.pcaec340	-0.040623889	0.045447761	0.372964921
pcaac402.pcaec424	0.021323583	0.023856393	0.372979845
acetylornithoine.c12	-0.05486089	0.061377718	0.372983458
pcaac402.pcaec444	-0.145886655	0.163227566	0.373016437
c101.serine	0.010715469	0.011989423	0.373026491
smc180.c51dc	-0.112619379	0.126034592	0.373124743
pcaec365.pcaec420	-0.020037283	0.022426857	0.373182859
pcaac363.c141oh	-0.110479782	0.123660451	0.373203075
pcaac343.pcaec402	-0.024995983	0.027978291	0.373206314

c8.pcaec386	0.005920888	0.006627572	0.373224546
pcaec405.smohc241	0.06865265	0.076861891	0.373319107
c181.c102	0.042945255	0.048083369	0.373347572
serine.smc261	0.030784121	0.034471423	0.37340545
pcaec385.pcaec420	-0.027903902	0.031250197	0.373466194
pcaac380.c10	-0.00258387	0.002893804	0.373477917
pcaec363.pcaac383	0.006963419	0.007800768	0.373605575
arginine.pcaec383	-0.086140581	0.096503875	0.373629828
c181.lysopcac281	0.010468842	0.011728747	0.373647429
pcaec386.pcaac364	-0.054384885	0.060937113	0.373703006
smohc222.ornithine	0.031437281	0.035228348	0.37375089
pcaac404.smohc241	0.048707349	0.054590043	0.373829478
c18.pcaac344	-0.053195549	0.059624607	0.373863802
pcaac281.pcaec382	0.01008391	0.011303721	0.373910166
histidine.putrescine	-0.025180546	0.028236082	0.374071208
pcaac323.smc180	-0.01280971	0.014366306	0.374144134
pcaac425.pcaec425	0.023829152	0.026728874	0.374216851
pcaec360.lysopcac281	0.01834097	0.020573507	0.374232142
pcaec445.pcaac386	0.022153078	0.024851207	0.374262182
pcaac406.h1	0.002609531	0.002927438	0.37427516
pcaec343.pcaac300	-0.090733065	0.101793945	0.374309295
pcaac384.glycine	0.028109272	0.031542338	0.374405727
alanine.tyrosine	0.067147237	0.075365855	0.374517811
smc241.pcaac383	0.003833972	0.004303293	0.374523747
kynurenine.c8	0.042182599	0.04734882	0.374549987
citruline.c0	0.000305759	0.00034322	0.374568701
pcaac426.pcaac380	0.050157673	0.056304004	0.374578581
pcaac343.pcaac422	-0.023003317	0.025824643	0.374624617
lysopcac281.putrescine	-0.054306271	0.060967763	0.374631642
c181.pcaac422	0.028835593	0.032376627	0.374689156
pcaec425.lysopcac181	-0.067346324	0.075621072	0.374717898
pcaac426.methionine	-0.035950453	0.040370365	0.374750113
pcaac403.pcaac365	-0.088543631	0.099436301	0.374782262
c5.arginine	-0.072066503	0.080935221	0.374800326
putrescine.pcaec421	0.005178468	0.00581611	0.3748301
c3.c162	0.101606636	0.11412484	0.374859544
pcaec360.c10	-0.002772718	0.003114519	0.374889253
pcaac380.pcaec425	0.00266695	0.002995865	0.374913553
pcaec340.c2	-0.060800072	0.068314288	0.375023297
pcaac403.pcaac240	-0.023777749	0.02671643	0.375023558
pcaac281.pcaac380	0.045233356	0.05082547	0.375039902
methioninesulfoxide.pcaac240	-0.024474611	0.027502101	0.375070036
pcaac341.pcaec425	0.00345688	0.003884802	0.375107784

pcaac300.pcaec446	-0.044153102	0.049620116	0.375120997
creatinine.pcaec406	0.007110047	0.007990435	0.375122538
asymmetricdimethylarginine.pca	0.012537997	0.014091458	0.375155247
lysopcac170.pcaec383	0.136541769	0.153495635	0.375267857
c141oh.taurine	-0.032467515	0.036501366	0.375300271
glycine.pcaec424	-0.007423593	0.008346903	0.375356353
pcaac384.ornithine	0.025062255	0.028181519	0.375392568
smc241.pcaac406	-0.061941094	0.069657383	0.375440831
smc160.pcaac402	-0.087312467	0.098200364	0.37549406
lysopcac170.pcaec406	-0.039874355	0.044853005	0.375560968
smc240.pcaac422	-0.031242132	0.03515055	0.375663562
pcaec360.tyrosine	-0.060740676	0.0683511	0.375745141
prolinr.pcaec425	-0.016527253	0.018598422	0.375755578
c4.pcaec386	0.012875583	0.014489621	0.375771728
pcaac401.pcaec382	-0.057860894	0.0651163	0.375787547
glycine.pcaec341	0.040312559	0.045368452	0.375797433
glutamicacid.spermidine	0.0614208	0.069126964	0.375817624
serine.taurine	-0.032071845	0.036098202	0.375850081
pcaec382.c142	0.028001655	0.03152155	0.375918303
c5.c3	0.042056329	0.047344428	0.375933255
creatinine.kynurenine	-0.022093638	0.024872222	0.375944025
pcaec444.arginine	-0.035298226	0.039739	0.375962346
pcaec404.pcaec382	-0.061627082	0.069390193	0.376030772
pcaac422.pcaac344	-0.04740089	0.053380541	0.376107475
c51.pcaec402	-0.020150151	0.022692391	0.37611348
methioninesulfoxide.lysopcac16	0.160384219	0.180631674	0.376146704
c12.pcaec443	-0.073225202	0.082471394	0.376158194
asymmetricdimethylarginine.pca	-0.015656841	0.017636349	0.376225905
pcaec404.pcaac406	-0.045574821	0.051336908	0.376226117
pcaac404.pcaec301	0.064172398	0.072291079	0.37626086
kynurenine.pcaec406	0.011740407	0.013226181	0.376277147
pcaec383.pcaac420	0.124921449	0.140743568	0.376321248
smohc141.c141	0.025088	0.028266899	0.376343805
pcaac422.pcaec343	0.066744097	0.075216532	0.376440583
lysopcac203.c51	-0.075971087	0.085636531	0.376561421
pcaac360.smohc241	0.065751213	0.074117723	0.376569727
pcaac404.pcaac385	-0.020797787	0.023445786	0.376601899
putrescine.c102	-0.032302039	0.03641673	0.376627476
lysopcac161.serotonin	-0.119819873	0.135087923	0.37664573
pcaec401.lysopcac180	-0.069615436	0.078493698	0.376691283
c10.serotonin	-0.083759193	0.09446793	0.376825683
pcaac321.c10	-0.007542559	0.008507769	0.376875074
pcaec385.c0	0.000369852	0.000417183	0.376877056

lysopcac182.pcaac300	-0.03889765	0.0438806	0.376932263
pcaac240.pcaec422	0.095024606	0.107208236	0.376979237
pcaac384.valine	-0.058452436	0.065951651	0.377013173
isoleucine.leucine	-0.043837453	0.049462773	0.377024237
c181.lysopcac170	-0.020063805	0.022643485	0.377130299
c51dc.h1	0.005727887	0.00646451	0.377142534
pcaac341.pcaac406	-0.043283431	0.048850831	0.37715255
pcaec386.pcaec382	0.016474414	0.018594692	0.377184026
pcaac420.pcaec425	0.004309986	0.004864943	0.377209115
smohc241.pcaac381	-0.020385881	0.023010792	0.377209295
pcaec381.smc181	-0.004918757	0.005553124	0.377296871
smc180.c12	0.06734445	0.076044792	0.377390668
smohc161.pcaec445	-0.026193115	0.029579236	0.377425864
pcaac343.pcaac365	-0.049565083	0.055973413	0.377432604
pcaac300.alphaaminoadipicacid	-0.007602759	0.008585753	0.377433924
c16.pcaec362	-0.015543541	0.017556989	0.377535668
alanine.smohc141	-0.040375792	0.045610849	0.377587204
ornithine.leucine	0.017739139	0.020041749	0.377648515
pcaec421.kynurenine	0.010953076	0.012375468	0.377673093
creatinine.c10	0.003072118	0.003471723	0.377762465
putrescine.c10	-0.002774299	0.003135637	0.377834071
pcaac405.lysopcac160	-0.02586822	0.02923835	0.377849182
pcaec386.pcaac341	0.008169877	0.00923455	0.377864382
pcaac402.pcaec365	0.021140475	0.023897941	0.377914242
asparagine.pcaac300	-0.095916476	0.108431223	0.377931112
pcaec384.c51dc	-0.106796457	0.120735375	0.377949237
pcaec423.lysopcac280	-0.010486536	0.011857252	0.3780306
pcaec384.lysopcac161	0.076423688	0.086416987	0.378051554
pcaec382.transhydroxyproline	0.071837437	0.081232641	0.378060984
c14.pcaec425	0.00966286	0.010926712	0.37806546
c51.lysopcac180	0.043935173	0.04968399	0.378087689
pcaac425.pcaac383	0.005342227	0.00604152	0.378109315
pcaec363.c51	0.059894175	0.067737244	0.378130181
lysine.smc180	-0.009386947	0.010616772	0.378157792
pcaac421.pcaec322	0.082095799	0.092863033	0.378216676
pcaec406.pcaac426	-0.098289393	0.11118315	0.378228046
c141.pcaac405	-0.06183637	0.069949871	0.378239665
lysopcac140.smohc241	0.058994684	0.066775612	0.378526579
c14.pcaec380	-0.043564653	0.049318953	0.37860824
smc181.pcaac420	-0.067322787	0.076223762	0.378661553
pcaec363.leucine	-0.010195431	0.011543466	0.378664111
smc240.pcaec360	-0.011583064	0.013116095	0.378719351
glycine.pcaec385	-0.043399123	0.049149902	0.378785707

serotonin.lysopcac182	-0.054196813	0.061379791	0.378796593
prolinr.pcaac383	0.023087025	0.026151216	0.378875686
pcaec422.pcaac422	-0.030282341	0.034305065	0.378924831
pcaec403.pcaac323	-0.076618284	0.086807888	0.378988273
c18.alanine	0.012688406	0.01437617	0.378998528
pcaac404.c51	0.030671991	0.034752407	0.379005938
asymmetricdimethylarginine.c0	-0.002460802	0.002788973	0.379142595
pcaac240.smc181	-0.00630547	0.007147888	0.379243816
pcaac403.c142	0.033992844	0.038534706	0.379248507
c181.c0	0.001165284	0.001321073	0.379281756
pcaac321.pcaec322	0.063374152	0.071858291	0.379358131
pcaec361.pcaec342	0.12501998	0.141758074	0.379362135
pcaac362.lysopcac181	0.022596236	0.025622416	0.379379206
c10.pcaec425	-0.031096304	0.035267925	0.379474577
pcaec321.alanine	-0.014121895	0.016016959	0.379492053
pcaec342.pcaec322	0.099272919	0.112598678	0.379508902
c10.prolinr	-0.047511081	0.053908684	0.379685422
pcaec342.pcaec425	0.002708407	0.003073259	0.379708835
pcaac402.c101	0.045362493	0.051473463	0.379710204
c18.glutamine	0.021175301	0.024032546	0.3798018
c182.lysopcac203	-0.016427912	0.018645197	0.379817557
pcaac323.pcaec425	0.003641375	0.004133457	0.379886964
c4.c0	-0.001009237	0.001145663	0.379904155
isoleucine.c4	0.034568412	0.039243959	0.37993658
creatinine.lysopcac160	0.010946091	0.012426859	0.379946365
c10.pcaac341	0.007472774	0.008483871	0.379957163
prolinr.glutamicacid	-0.0628929	0.07140548	0.379976562
c0.pcaac402	-0.048303473	0.054844849	0.38000671
pcaac341.c162	-0.068881825	0.078213393	0.380027496
pcaac320.smc180	-0.008314535	0.009441768	0.380070019
c51dc.leucine	-0.023797183	0.027028234	0.380153996
lysopcac280.smohc161	-0.011294013	0.012828411	0.380189501
pcaec405.pcaac384	0.047930307	0.054457866	0.380327152
c0.h1	0.005503958	0.006253644	0.380335454
ornithine.asymmetricdimethylarg	0.01590026	0.018072388	0.380503164
pcaac366.tryptophan	-0.038185576	0.043402542	0.380508078
pcaec402.asparagine	0.009382202	0.010665405	0.380570167
methionine.smc181	0.01035125	0.011767737	0.3806003
pcaac421.lysopcac160	-0.017436307	0.019825447	0.38067503
pcaac405.pcaac361	0.144784358	0.164628156	0.380690336
c0.asparagine	0.013402424	0.015239792	0.380704773
pcaec421.pcaec362	0.010868202	0.012358157	0.380705145
pcaac365.c10	-0.003737192	0.004249807	0.380735562

pcaec322.pcaec425	0.003324208	0.003780328	0.380754631
pcaac360.valine	-0.047829616	0.05440017	0.380822548
lysopcac182.pcaac422	-0.013377444	0.015216258	0.38085686
pcaec380.smc180	-0.007325686	0.008333984	0.380932994
pcaec365.smohc161	0.031933137	0.03632942	0.380946677
pcaec444.pcaac424	0.015720084	0.017885765	0.380985808
lysopcac170.glutamicacid	0.035445073	0.040335702	0.381074463
pcaec401.glycine	0.033104395	0.037677088	0.38113781
pcaac425.pcaac363	-0.013171974	0.014991449	0.381139003
c181.serine	0.025275153	0.028769106	0.381182538
pcaec360.c51dc	0.133833676	0.152352472	0.381238867
c2.pcaec425	0.004014987	0.004570667	0.381251224
pcaac405.c51	0.027857704	0.031719742	0.381348454
lysopcac203.c102	-0.031580325	0.035960324	0.381373247
pcaec401.asparticacid	0.03523733	0.040130447	0.38144322
serotonin.serine	0.037901915	0.043173443	0.381535616
lysopcac280.pcaec322	-0.050623045	0.057680252	0.381670474
lysine.h1	0.003540919	0.004034593	0.38167579
pcaec363.pcaec425	0.002742273	0.003124846	0.381713137
smohc241.pcaec403	-0.014856794	0.016930919	0.381754122
methioninesulfoxide.pcaec445	-0.107060111	0.1220314	0.381850851
c142.glycine	-0.022545642	0.025698588	0.381853851
smc181.transhydroxyproline	0.025446455	0.029007807	0.381898617
smc181.asparagine	0.016976423	0.019353175	0.381918771
alphaaminoadipicacid.pcaec425	-0.002758818	0.003145156	0.381933062
smc240.c162	0.082381998	0.093922246	0.381951671
asparagine.pcaec385	0.058612348	0.066827756	0.381986218
lysopcac140.pcaac363	0.012996878	0.014818907	0.381996455
smohc221.kynurenine	-0.003999908	0.00456078	0.382009615
lysopcac204.smc161	-0.081828748	0.093304044	0.382015513
pcaec386.leucine	-0.007603383	0.008670053	0.382037709
pcaac344.valine	0.058369166	0.066558105	0.382040543
c141oh.smohc161	0.007480931	0.008530649	0.382050367
lysopcac170.c141	0.017366068	0.019804636	0.382092896
pcaec401.pcaac381	-0.063235951	0.072121853	0.382133843
smc261.pcaac363	-0.007373261	0.008409404	0.382136904
pcaac404.pcaec421	0.00114423	0.001305108	0.382166975
lysopcac140.pcaac344	0.06479128	0.073917099	0.382271027
pcaec445.c0	0.000319865	0.000364962	0.382328898
pcaec343.spermidine	-0.063332891	0.072262469	0.382331271
c10.tyrosine	0.068179921	0.077825554	0.382530415
smc202.pcaac425	-0.070223206	0.080161769	0.382553264
asymmetricdimethylarginine.h1	-0.001076659	0.001229079	0.382569853

pcaac406.smc181	0.019905341	0.022730719	0.382724702
pcaec421.smc161	-0.106706128	0.121857231	0.382744803
pcaac403.c101	0.06425214	0.073379904	0.382774958
pcaac364.pcaec360	-0.00431942	0.004933659	0.382834043
methionine.pcaac402	-0.032853018	0.037534456	0.382955561
pcaec360.lysopcac203	0.026086902	0.02981	0.383048002
c141oh.c10	-0.005591074	0.006389916	0.383114018
pcaec383.pcaec444	-0.171903872	0.196472539	0.383131813
pcaac240.phenylalanine	0.069899252	0.079892434	0.383150302
c101.c0	-0.00095412	0.001090791	0.383265748
pcaac360.pcaec425	0.003660125	0.004184857	0.383315948
pcaec404.pcaec362	0.008709042	0.009958322	0.383349796
pcaec421.pcaec361	0.010449164	0.011948291	0.383359076
pcaac420.pcaac281	0.001918156	0.002193565	0.383405316
pcaec360.asparagine	0.011590984	0.01325587	0.383428611
pcaac300.lysopcac181	0.035265876	0.040333224	0.38345083
pcaec321.pcaac380	0.095955205	0.109767782	0.38355796
glycine.c141oh	0.084855862	0.097075631	0.383582006
pcaec383.creatinine	-0.009699565	0.011099198	0.383703177
arginine.pcaec365	-0.020702578	0.023691168	0.383727991
pcaac421.putrescine	-0.036144768	0.041371461	0.383829953
pcaac364.phenylalanine	0.049075538	0.056175301	0.383857164
pcaac300.lysopcac204	-0.015822294	0.018113365	0.383911053
serine.pcaec421	0.002500065	0.002862297	0.3839479
c18.lysopcac180	-0.066595768	0.0762507	0.383984769
pcaec341.h1	0.001375198	0.001574643	0.384006444
phenylalanine.smc161	0.068241628	0.078150058	0.384075098
pcaac343.smc180	-0.005776476	0.006616006	0.384133024
pcaac344.pcaac322	-0.102713863	0.11764966	0.384164283
pcaac421.alanine	-0.011027135	0.012633328	0.384266345
pcaac321.pcaec425	0.007575052	0.008678587	0.38427542
lysopcac203.smc161	-0.097823387	0.112091493	0.384348071
smohc141.smc161	0.10381476	0.118966575	0.38438726
c102.pcaec342	0.065710421	0.075336395	0.384610748
asymmetricdimethylarginine.pca	-0.073771177	0.084580818	0.384626671
alphaaminoadipicacid.serine	0.017165398	0.019682197	0.384664352
smc181.spermidine	0.062082777	0.07118842	0.384684576
c3.arginine	-0.045863592	0.052590406	0.38468483
pcaac343.spermidine	-0.061246936	0.070230313	0.384686778
pcaec386.c141	-0.033110249	0.037967958	0.384702662
pcaac344.smc160	0.056328983	0.064605281	0.38479142
asparticacid.pcaec360	-0.005464326	0.006267296	0.384799592
smc181.ornithine	0.072468317	0.083130494	0.384874452

smohc161.pcaac362	-0.024162729	0.027722176	0.384949922
pcaec360.pcaac403	-0.017208754	0.019744129	0.384957787
acetylornithoine.pcaec446	-0.017168428	0.019699193	0.384989839
pcaec421.valine	-0.029124078	0.033417762	0.384997775
pcaac381.pcaac321	0.025760666	0.029559672	0.385016693
pcaec446.c0	0.000324239	0.000372099	0.385071727
pcaac341.pcaac300	0.125400784	0.143919404	0.385100266
pcaac361.valine	-0.049577046	0.056911909	0.385213111
pcaac342.pcaec322	0.054099699	0.06210704	0.385238731
pcaac422.c3	-0.062875683	0.072199045	0.38535085
spermidine.c2	0.268009973	0.307777503	0.385391419
pcaec363.c51dc	-0.1244784	0.142963396	0.385440453
pcaac383.lysopcac181	0.02668066	0.030643311	0.385449465
glutamine.pcaac321	-0.012658135	0.014539075	0.385479953
lysopcac161.pcaec386	0.00907279	0.010421008	0.385481919
pcaac426.glutamicacid	0.060908121	0.069963658	0.385512957
pcaac341.spermidine	-0.063682217	0.073151272	0.385519942
smohc222.pcaec343	-0.0784364	0.090108793	0.385569922
lysopcac281.pcaec421	0.003278935	0.003766907	0.385572744
pcaac341.pcaec301	-0.068984336	0.079259357	0.38562506
serotonin.c0	0.000783066	0.000899842	0.385699033
pcaac386.c141	-0.035966312	0.041332634	0.385731008
pcaec444.lysopcac160	-0.010822909	0.012439448	0.385796392
pcaac321.pcaec404	0.03001778	0.034509768	0.385912417
pcaac344.pcaec401	0.09206856	0.105849563	0.38592796
pcaac364.h1	0.001084696	0.001247109	0.385948624
smc160.pcaac361	0.06026977	0.069303704	0.386014353
pcaac300.lysine	-0.072662518	0.083561887	0.386058943
lysopcac170.smohc141	-0.022815606	0.026240154	0.386098834
pcaac281.pcaec385	-0.053640777	0.061697475	0.386140236
pcaec342.pcaac381	0.035051554	0.040320355	0.38618904
methionine.pcaac420	0.048329188	0.055594426	0.386194144
pcaec385.pcaac360	0.042612867	0.049026936	0.386272944
isoleucine.pcaac425	-0.09012838	0.103713515	0.386359959
pcaac421.pcaac422	0.077402326	0.089082185	0.386428728
c181.taurine	-0.060641166	0.069791812	0.386428822
asymmetricdimethylarginine.pca	-0.089384547	0.102877975	0.386453941
c101.creatinine	0.005743555	0.00661138	0.386509972
pcaec384.threonine	-0.039576473	0.045557039	0.386517675
lysopcac280.lysopcac161	0.099113841	0.1141067	0.386581392
lysopcac204.smc180	-0.012007678	0.013825232	0.386621234
serine.pcaec422	-0.03006188	0.034616025	0.386673221
c4.ornithine	0.044225393	0.050934416	0.386758901

pcaac380.c141oh	-0.101583018	0.116993618	0.386760459
pcaec321.pcaec362	0.026066372	0.030035957	0.387000131
pcaec322.histidine	-0.065264251	0.075206867	0.387023303
pcaec340.c5	0.008177207	0.009424243	0.387088005
pcaac426.pcaac403	-0.02042387	0.02355181	0.387354832
pcaec322.smohc141	0.015218691	0.017550182	0.3873745
isoleucine.tyrosine	0.072026771	0.083064789	0.387394903
pcaec385.lysopcac180	-0.049268293	0.056824466	0.387443758
pcaec385.smohc141	-0.050991396	0.058820974	0.387517296
asymmetricdimethylarginine.alar	0.024173496	0.027889612	0.38759108
alphaaminoadipicacid.pcaec362	-0.013804852	0.015928438	0.387632955
pcaac365.isoleucine	0.031653339	0.036523288	0.387642664
serine.smohc222	-0.007052679	0.00813937	0.387736724
pcaec383.pcaec362	0.010121082	0.011681685	0.387782393
pcaac240.lysopcac280	-0.041243052	0.047608511	0.387842605
pcaac281.pcaec402	-0.048315747	0.05577637	0.38787284
lysine.pcaec406	-0.00989275	0.011421798	0.387933731
smohc221.pcaec424	0.020546543	0.023722563	0.387939635
smohc222.pcaac406	-0.029168822	0.033677845	0.387942422
pcaec300.c10	-0.002006565	0.002317078	0.388009877
pcaac426.c51	0.05768178	0.06661061	0.388028891
smohc161.histidine	-0.053753799	0.062081395	0.388080694
c3.pcaac323	0.068609626	0.079240845	0.388093491
pcaec361.c162	-0.083837242	0.096828927	0.388097951
pcaac343.methionine	-0.029952277	0.034594709	0.388110691
pcaac386.pcaac320	0.036355848	0.041991089	0.388113995
isoleucine.serotonin	0.094182865	0.108802078	0.388203808
lysopcac182.pcaac406	-0.028479328	0.032901997	0.388233473
pcaec422.pcaac426	0.081605459	0.094281992	0.388252013
lysopcac182.pcaac322	0.084821583	0.098022615	0.388372266
lysopcac204.taurine	0.054963026	0.063519704	0.388391786
creatinine.prolinr	0.036467774	0.042146113	0.388403163
pcaec381.pcaac383	0.013408798	0.015497002	0.388413572
pcaac361.pcaec425	0.002564669	0.00296414	0.388423989
c10.pcaec341	-0.062562938	0.072309884	0.388438306
pcaec401.smc161	-0.076439025	0.088349894	0.3884496
putrescine.histidine	-0.05664675	0.065477673	0.388479389
pcaac404.pcaec424	0.007450445	0.008612079	0.388487596
pcaec444.smohc141	-0.03691868	0.042678615	0.388529403
c51.c5	-0.005816789	0.006725232	0.388594585
pcaac320.alanine	-0.011335914	0.013107436	0.388635132
phenylalanine.c14	0.037812416	0.043725407	0.388676767
lysopcac280.pcaac406	-0.042486141	0.049143904	0.388810668

c4.methioninesulfoxide	0.018771002	0.021713769	0.388838333
c142.pcaac281	0.001359854	0.001573099	0.388855901
pcaec420.smohc161	0.014248331	0.016488154	0.389012896
pcaac321.pcaac381	0.021481891	0.024860304	0.389041066
smc260.alanine	-0.018765011	0.021719315	0.38911011
pcaec385.serine	-0.076122822	0.08810885	0.389118104
pcaac404.pcaac321	0.034700456	0.040167552	0.389156791
pcaec363.arginine	-0.043501112	0.05035586	0.389167116
pcaac322.creatinine	-0.009038313	0.010464699	0.389264811
lysopcac170.pcaac403	-0.008744871	0.01012498	0.389266352
pcaac424.pcaac381	0.04538392	0.05254952	0.389294622
pcaec362.pcaec425	0.003421889	0.003962276	0.389307679
pcaec423.pcaec425	0.003648927	0.004225339	0.389326861
smohc222.leucine	-0.012738713	0.014751556	0.389344187
c182.pcaec425	0.006545507	0.007581388	0.389445602
alphaaminoadipicacid.pcaac323	0.113018593	0.130922357	0.389509312
pcaac405.c10	-0.004638936	0.005373941	0.389520862
pcaac363.creatinine	-0.00526134	0.006095338	0.389550091
histidine.pcaac343	-0.016739821	0.019395037	0.389591937
pcaac360.isoleucine	0.032678098	0.037862641	0.389607533
pcaac425.pcaec301	0.057461797	0.066581141	0.389627017
pcaac320.pcaac420	-0.081674965	0.094645923	0.389671571
smohc141.pcaec423	-0.009753037	0.011302147	0.389680425
c142.c5	-0.00562863	0.006522703	0.389684555
c51.ornithine	-0.021227324	0.024600234	0.38970536
smohc141.pcaec362	0.014718464	0.01705765	0.389719211
serine.pcaec425	-0.002444228	0.00283275	0.389729978
pcaac322.pcaac380	0.037093179	0.042989834	0.389735547
asymmetricdimethylarginine.pca	0.007080225	0.008205824	0.389739251
pcaac361.pcaec342	0.109719757	0.127163851	0.389743291
pcaac404.pcaec446	-0.037089979	0.04299046	0.389783204
smc180.pcaac281	0.002289856	0.002654414	0.389832049
pcaac342.pcaac383	-0.007689148	0.008914041	0.389871302
pcaac363.pcaac406	-0.035912377	0.041636021	0.389902474
pcaac401.smohc161	0.020837332	0.024161024	0.389954891
smohc161.pcaac323	-0.090756751	0.105247086	0.390017941
pcaac380.pcaac403	-0.008299885	0.009625078	0.39001908
pcaec421.asparagine	0.019637	0.022774534	0.390064992
smc202.arginine	0.025064598	0.029074308	0.390145842
pcaec360.c12	0.045907198	0.053254082	0.39017138
pcaac362.transhydroxyproline	0.021473053	0.024911755	0.390213195
pcaec384.pcaec343	-0.065347537	0.075825284	0.390294054
pcaec383.pcaec363	0.027606859	0.032035778	0.390330455

alphaaminoadipicacid.lysopcac18	-0.050269373	0.058347815	0.390442395
pcaac421.lysine	0.08307558	0.096429973	0.390461472
pcaac344.serine	-0.040222019	0.046688499	0.390469501
pcaec384.smohc141	-0.022203647	0.02577398	0.390481617
pcaec381.creatinine	-0.012633226	0.01466705	0.390559236
pcaec365.pcaac420	0.057052852	0.066241383	0.390584843
ornithine.c0	-0.001311344	0.001522551	0.390588327
pcaec424.pcaec403	0.052956909	0.061492985	0.39064024
pcaac362.pcaac426	0.046123903	0.053560884	0.390660636
pcaec401.pcaec425	0.003364576	0.003907571	0.390720364
prolinr.pcaec343	-0.086125266	0.100027856	0.390735385
smc260.c10	-0.001906016	0.002213762	0.390750537
pcaec341.c8	-0.038346192	0.044539487	0.390770827
asparagine.lysopcac180	0.04765005	0.055349152	0.390797649
pcaac383.serine	-0.026939164	0.031292929	0.390813411
lysopcac140.isoleucine	-0.025199243	0.029275187	0.390867916
methionine.c51dc	-0.11043669	0.128322761	0.390952908
c18.threonine	-0.032365931	0.037615466	0.39104867
pcaac421.pcaac240	-0.020018204	0.023265469	0.391057772
pcaec364.smc241	0.102173768	0.118748307	0.391059198
c182.lysopcac182	-0.017959341	0.020872708	0.391059614
pcaec383.smc181	-0.004555091	0.005294662	0.391117039
pcaac381.smohc222	-0.008802939	0.010233788	0.391190489
pcaec362.serine	-0.018903809	0.021976616	0.391193513
smc260.c0	0.000606598	0.000705267	0.391238255
pcaec360.smc181	0.03105277	0.036107391	0.391285181
pcaec425.pcaec364	-0.011388358	0.013243487	0.39133466
[truncated: 394,934 more chars]
